# NF-κB Transcription Factors: Their Distribution, Family Expansion, Structural Conservation, and Evolution in Animals

**DOI:** 10.3390/ijms25189793

**Published:** 2024-09-10

**Authors:** Siphesihle Msweli, Suresh B. Pakala, Khajamohiddin Syed

**Affiliations:** 1 Department of Biochemistry and Microbiology, Faculty of Science, Agriculture and Engineering, University of Zululand, KwaDlangezwa 3886, South Africa; siphesihlemsweli2001@gmail.com (S.M.); pakalasb@uohyd.ac.in (S.B.P.); 2 Department of Biochemistry, School of Life Sciences, University of Hyderabad, Hyderabad 500-046, India

**Keywords:** NF-κB, evolution, RHD, c-Rel, p50/p105, RelA/p65, p52/p100, RelB

## Abstract

The Nuclear Factor Kappa B (NF-κB) transcription factor family consists of five members: RelA (p65), RelB, c-Rel, p50 (p105/NF-κB1), and p52 (p100/NF-κB2). This family is considered a master regulator of classical biochemical pathways such as inflammation, immunity, cell proliferation, and cell death. The proteins in this family have a conserved Rel homology domain (RHD) with the following subdomains: DNA binding domain (RHD-DBD) and dimerization domain (RHD-DD). Despite the importance of the NF-κB family in biology, there is a lack of information with respect to their distribution patterns, evolution, and structural conservation concerning domains and subdomains in animals. This study aims to address this critical gap regarding NF-κB proteins. A comprehensive analysis of NF-κB family proteins revealed their distinct distribution in animals, with differences in protein sizes, conserved domains, and subdomains (RHD-DBD and RHD-DD). For the first time, NF-κB proteins with multiple RHD-DBDs and RHD-DDs have been identified, and in some cases, this is due to subdomain duplication. The presence of RelA/p65 exclusively in vertebrates shows that innate immunity originated in fishes, followed by amphibians, reptiles, aves, and mammals. Phylogenetic analysis showed that NF-κB family proteins grouped according to animal groups, signifying structural conservation after speciation. The evolutionary analysis of RHDs suggests that NF-κB family members p50/p105 and c-Rel may have been the first to emerge in arthropod ancestors, followed by RelB, RelA, and p52/p100.

## 1. Introduction

NF-κB is a family of transcription factors first discovered four decades ago as a regulator of immunoglobin kappa light-chain expression in mature B cells and plasma cells [1]. It is expressed in almost all cell types and tissues and is crucial in maintaining cellular homeostasis. NF-κB is inducible in most cells and is also found as a constitutively active protein in mature B cells, macrophages, neurons, vascular smooth muscle cells, and tumor cells [2]. In resting cells, NF-κB dimer is sequestered in the cytoplasm by inhibitors called IκBs, which mask its nuclear localization signal. However, when stimulated with various signals such as inflammatory cytokines, growth factors, and infectious microbes, IκBs are phosphorylated and ubiquitinated, allowing NF-κB dimers to localize to the nucleus to regulate the transcription of target genes [3]. NF-κB regulates many biological processes, including immune and inflammatory responses, developmental processes, cell proliferation, and apoptosis [4] (Table 1). Dysregulation of NF-κB can lead to cancer, autoimmune diseases, and chronic inflammatory disorders [5,6,7,8]. NF-κB activation affects cancer and inflammatory diseases by regulating the transcription of target genes involved in cell proliferation, angiogenesis, inflammation, tumor promotion, and metastasis [9] (Table 1). Additionally, NF-κB plays a role in cancer progression by interacting with other transcription factors such as STAT3 (signal transducer and activator of transcription 3), p53 (tumor protein p53), ATF3 (activating transcription factor 3), and SMAD3/4 (Suppressor of Mothers against Decapentaplegic 3/4), which then bind to the promoter regions of their target genes, regulating their expression patterns [10,11]. Furthermore, GSK-3β has been found to modulate NF-κB activity in glioma, prostate, and colon cancer to trigger cell proliferation [12,13,14,15,16].

**Figure 1:**
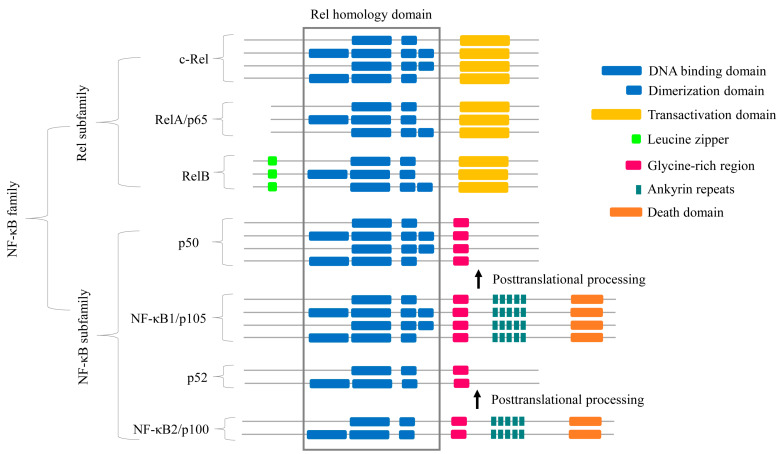
Schematic diagram illustrating the classification and characteristic domains of NF-κB proteins, along with additional DNA binding and dimerization domains identified in this study.

There are five NF-κB family proteins: RelA (p65), RelB, c-Rel, p50 (p105/NF-κB1), and p52 (p100/NF-κB2) [25] (Figure 1). The p105 and p100 undergo post-translational modification to generate p50 and p52, respectively [2] (Figure 1). These family members regulate the transcription of target genes involved in different physiological and pathological processes by recruiting onto their promoter sequences, i.e., κB consensus DNA sequence, as various homo or hetero dimers [26] (Table 1).

The NF-κB signaling pathway can be activated through two major pathways: canonical and noncanonical. The canonical pathway is a key regulator of the inflammatory response, triggered by Toll-like receptors (TLRs), tumor necrosis factor α (TNF-α), interleukin 1 (IL-1), and various pathogens. This pathway results in the activation of RelA, which governs the expression of proinflammatory and cell survival genes. Canonical NF-κB signaling provides a rapid response to acute inflammatory cues. It mediates the innate immune response under conditions such as inflammatory bowel disease (IBD), rheumatoid arthritis (RA), and chronic obstructive pulmonary disease (COPD) [2,3,27]. On the other hand, the noncanonical NF-κB pathway is activated by lymphotoxin β (LTβ), CD40 ligand (CD40L), B-cell activating factor (BAFF), and receptor activator of nuclear factor kappa-B ligand (RANKL), resulting in the activation of RelB/p52 complexes. Noncanonical signaling responses are typically slow and sustained, which is characteristic of immune dysregulation. Activation of the noncanonical pathway is associated with the adaptive immune response, which regulates genes essential for lymph-organogenesis and B-cell activation. Dysregulation of this pathway results in lymphoid malignancies and other immune disorders [28,29]. These pathways are characterized by their requirement for IκB kinase (IKK) subunits. IKKβ regulates the activation of the canonical pathway through the phosphorylation of IkBs, requiring an IKKγ subunit, while IKKα is necessary for the activation of the noncanonical pathway [30]. Despite their different signaling mechanisms, both pathways are crucial for controlling immune and inflammatory responses [3], contributing to innate and acquired immunity [31] (Table 1).

The NF-κB proteins can be divided into two subclasses: NF-κB proteins (p52/p100 and p50/p105) and Rel proteins (p65/RelA, c-Rel, and RelB) [25] (Figure 1). They all have a conserved N-terminal region, known as the Rel homology domain (RHD), which contains the subdomains DNA binding domain (RHD-DBD) and dimerization domain (RHD-DD). This RHD is responsible for the homodimerization and heterodimerization of NF-κB proteins [25] (Figure 1). Additionally, members of the NF-κB subclass (p50 and p52) have a C-terminal inhibitory domain made up mainly of Ankyrin (ANK) repeats [32,33], while the C-terminus of RelA, RelB, and c-Rel contains a transcription activation domain (TAD) [34,35] (Figure 1). The TAD of NF-κB plays a crucial role in recruiting co-activators such as p300 and CBP, involved in histone deacetylation and chromatin remodeling, to facilitate gene transcription [36,37]. Phosphorylation of TAD at Ser276 and Ser536 enhances the recruitment of elongation factors like Brd4 and P-TEFb, which are involved in mRNA processing and transcriptional elongation [37,38]. These modifications induce conformational changes in the TAD, allowing it to interact more effectively with other proteins involved in the transcription of target genes. By facilitating the interaction of NF-κB with specific cofactors, the TAD influences the transcription of genes involved in inflammation, immune responses, and cell survival [37,38]. Further, its interaction with SMAD3/4 proteins also regulates signaling pathways affecting bone development and differentiation [39]. The proteins p50 and p52 lack TAD and hence act as transcriptional repressors as homodimers but activate transcription when heterodimerized with transactivating Rel subunit [25,34,35]. Finally, the N terminus of RelB contains a leucine zipper (LZ) motif, which is critical for transcriptional regulation [40]. Whether the LZ gives RelB any extra functional specificity, such as the ability to heterodimerize with NF-κB2 or other potential partners, remains unknown.

It has been suggested that NF-κB proteins were formed through the fusion of ANK repeat protein and RHD protein, followed by gene splitting and duplication events, which eventually led to the present-day NF-κB/Rel proteins [41]. The evolution of NF-κB proteins appears to have occurred after the split between mammals and fungi [42]. In a study aiming to identify the origin of NF-κB/Rel transcription factors in animals, researchers analyzed the presence of NF-κB/Rel protein domains in a handful of metazoan species and non-metazoans [42]. Other than these two studies, a comprehensive analysis of NF-κB proteins, their distribution patterns, and how these families have evolved, particularly in terms of the RHD and its subdomains RHD-DBD and RHD-DD, has not been previously reported. Therefore, our aim was to address this research gap, which also provides insights into the origins of innate immunity in different taxa.

## 2. Results and Discussion

### 2.1. p50/p105 Proteins Are Present in Most of the Animals

The analysis of genome data and annotation of NF-κB family proteins revealed that there are a total of 5860 NF-κB proteins present in 2819 animals (refer to Table S1). Among the NF-κB family, c-Rel has the highest number of members with 2463, followed by p50/p105 with 1401, p52/p100 with 1088, RelA/p65 with 522, and RelB with the least number of members at 386 (refer to Table S1). Overall, the Rel subfamily has more proteins compared to the NF-κB subfamily. On average, there are approximately two proteins for each NF-κB family member in every species (refer to Table S1). A total of 1716 duplicates were identified, with the majority being c-Rel proteins at 618 duplicates, followed by p50/p105 proteins at 462 duplicates, p52/p100 at 344 duplicates, RelA/p65 at 150 duplicates, and RelB at 142 duplicates. Detailed information regarding the number of species and NF-κB proteins is presented in Table S1. The NF-κB protein sequences, identification numbers, and respective domains are presented in Supplementary Dataset S1.

**Figure 2:**
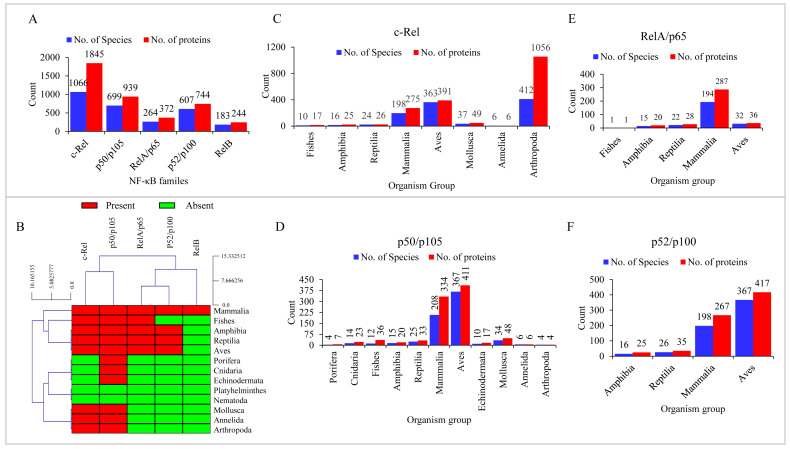
Genome-wide data mining, identification, and classification of animal NF-κB family proteins. (**A**) Comparative analysis of five NF-κB family members in animals. (**B**) Heat-map representation of NF-κB family members conservation in animals. The animal groups are depicted vertically, while NF-κB families are represented horizontally. Comparative analysis of c-Rel (**C**), p50/p105 (**D**), RelA/p65 (**E**), and p52/p100 (**F**) NF-κB family members in different animal groups. Detailed information on the number of species and NF-κB proteins in animals is presented in Table S1. The complete NF-κB protein sequences without duplicates identified and annotated in this study are presented in Supplementary Dataset S1.

It is well-known that the ancestral vertebrate genome underwent two rounds of whole genome duplication, and consequently, vertebrates have more genes than invertebrates [43]. In concurring with this fact, we observed multiple identical copies of NF-κB family genes/proteins within the same species in many animals (Supplementary Dataset S1). For further analysis, we selected one of the duplicates as a representative, as the duplicates did not affect the outcome. After removing duplicates, the total number of NF-κB family proteins was 4144 in 2819 animal species (Figure 2A and Table S1). Comparative analysis of NF-κB proteins showed that c-Rel was the dominant family with 1845 members, followed by p50/p105 with 939 members, p52/p100 with 744 members, RelA/p65 with 372 members, and RelB with 244 members (Figure 2A). The average number of proteins in each NF-κB family confirmed that the c-Rel family is more prevalent in animals, with an average of two c-Rel proteins present in each species compared to a single protein for the other four NF-κB families (Figure 2A and Table S1). Even after removing the duplicates, there was still a high number of NF-κB family proteins, indicating diversification of these protein family members.

Genome-wide analysis revealed the presence of c-Rel proteins in five different classes of the phylum Chordata: Fishes, Amphibia, Reptilia, Mammalia, and Aves, as well as in three other phyla: Mollusca, Annelida, and Arthropoda (Figure 2B). Arthropoda has the highest number of c-Rel proteins, with 1056, while Annelida has the least, with just six. Among the different animal groups, Arthropoda has the highest average c-Rel proteins per species (three proteins), followed by Fishes and Amphibia with two proteins, and Reptilia, Aves, Mammalia, Mollusca, and Annelida each having a single c-Rel protein (Figure 2C). This suggests that chordates such as Fishes and Amphibia have more c-Rel proteins in their genomes than Aves, Reptilia, and Mammalia.

The function of c-Rel includes regulating immune responses through innate and acquired immunity [44]. It promotes innate immune response by producing cytokines like IL-12 and IL-23 and activating macrophages and dendritic cells against microbial infections [44]. Additionally, c-Rel promotes T cell activation and proliferation, allowing T cells to differentiate into Th1 (T helper 1) cells, which combat intracellular pathogens. c-Rel also plays a role in coordinating the adaptive immune response by controlling the expression of different cytokines and chemokines [44]. Studies have shown that c-Rel and RelA/p65 differ functionally, particularly in their affinity for binding DNA. This difference may have enabled c-Rel’s neofunctionalization and allowed it to assume specialized roles in immune regulation [45]. Furthermore, because c-Rel can bind to non-consensus DNA motifs, it can more effectively activate the transcription of immune-related genes, which is crucial for controlling adaptive immunity in vertebrates [45].

Although most research on c-Rel has focused on vertebrates, insights from model organisms such as *Caenorhabditis elegans* indicate that immune signaling pathways have ancient origins. Despite lacking TLRs, *C. elegans* has evolved other immune pathways, demonstrating a distinct evolutionary strategy for pathogen defense. This suggests that the evolutionary mechanisms of the immune response are diverse throughout the tree of life despite the critical role that c-Rel and its counterparts play in vertebrates [45]. Research comparing various species, such as bony fish and cartilaginous fish, suggests that c-Rel has remained essential throughout evolution, adapting to meet the unique immunological demands of various organisms [44,46,47]. The identification of c-Rel in both vertebrate and invertebrate species (Figure 2C) strongly supports its critical role in these organisms, as discussed above.

The p50/p105 proteins were present in all five classes of Chordata and six other phyla of animals (Figure 2B). However, the phyla Platyhelminthes and Nematoda did not have p50/p105 proteins (Figure 2B). Among the different groups, Aves had the highest number of p50/p105 proteins, with 411, followed by Mammalia, with 334 (Figure 2D). Arthropoda had the lowest number of p50/p105 proteins, with only four. On average, fishes had three p50/p105 proteins in each species, while Porifera, Cnidaria, Mammalia, and Echinodermata had an average of two proteins (Figure 2D). The remaining animal groups had an average of one p50/p105 protein in each species (Figure 2D).

Genome-wide analysis revealed the presence of RelA/p65 proteins in five classes of Chordata: Fishes, Amphibia, Reptilia, Aves, and Mammalia (Figure 2B). On average, each of the five Chordata classes had one RelA/p65 protein (Figure 2E). RelA/p65 proteins were more abundant in Mammalia, with 287 proteins, while Fishes had the lowest number of proteins (one protein) (Figure 2E). Notably, RelA/p65 proteins are only present in Chordata classes and are not found in other animal species (Figure 2B). RelA/p65 is an important protein that is activated when pathogens are recognized by cellular pattern recognition receptors (PRRs) like TLRs and nucleotide-binding oligomerization domain-like receptors (NOD-like receptors). This activation leads to the expression of several downstream cytokines and effector molecules that are involved in innate immune response [48,49,50]. It is interesting to note that NF-κB signaling pathways integrate information from various PRRs to tailor inflammatory responses based on the identity and virulence of the pathogen [48,49,50]. The regulation of RelA/p65s is dynamic, and it controls the transcription of pro-inflammatory cytokines and other genes involved in cellular processes, including cell survival, apoptosis, and pathogen clearance in response to infections to offer innate immunity [3,11,48]. Furthermore, the expression levels of RelA/p65 maintain the balance between pro- and anti-inflammatory responses, thereby preventing chronic inflammation and autoimmune diseases [3]. These findings suggest that innate immunity might have originated in vertebrates, initially in Fishes, followed by Amphibians, Reptiles, Aves, and Mammals.

The p52/p100 proteins were found in only four classes of Chordata: Amphibia, Reptilia, Mammalia, and Aves, and were not present in other animal species (see Figure 2B). Among these classes, p52/p100 proteins were not found in Fishes (see Figure 2B). On average, only one p52/p100 protein was found per species in all animal groups, except for Amphibia, which had an average of two proteins (see Figure 2F). Aves had the highest number of p52/p100 proteins, with 417 proteins, while Amphibia had the least, with 25 members (see Figure 2F).

In the study, it was found that RelB proteins were only present in mammals among all the animal groups analyzed (Figure 2B). A total of 244 RelB proteins were identified in 183 species, indicating an average of one protein in each species (Figure 2A and Table S1). RelB is an important transcription factor in the NF-κB family, playing a crucial role in regulating adaptive immunity by modulating the development and activation of dendritic cells (DCs) and T-cell responses. It is essential for the differentiation and function of dendritic cells, which are antigen-presenting cells that link innate and adaptive immunity [3,51,52]. Without RelB, the ability of DCs’ to present antigens to T cells is compromised, affecting the adaptive immune response. Additionally, RelB is necessary for the development of the thymus, where T cells mature. The absence of RelB leads to defects in T-cell activation, cytokine production, and overall immune regulation [3,51,52]. This analysis determined that RelB is exclusively present in mammals, suggesting that RelB-modulated adaptive immunity is limited to mammals.

### 2.2. Some NF-κB Proteins Have Multiple RHD Subdomains

All NF-κB protein families have members with multiple RHD-DBDs and RHD-DDs (see Figure 1 and Table 2). There are 31 c-Rel proteins, five p50/p105 proteins, four RelA/p65 proteins, three p52/p100 proteins, and four RelB proteins with more than one RHD-DBD and/or RHD-DD (Table 2). The highest number of c-Rel proteins with multiple domains were found in Arthropoda (24 proteins), followed by Mammalia (three proteins) and Aves (two proteins), and a single protein was found in Reptilia and Mollusca. Two p50/p105 proteins with multiple RHD subdomains were found in Mammalia, and a single protein was found in Cnidaria, Reptilia, and Mollusca. Two RelA/p65 proteins with multiple RHD subdomains were identified in each Mammalia and Aves. Two and one p52/p100 proteins with multiple RHD subdomains were identified in Mammalia and Aves, respectively, and four RelB proteins with multiple RHD subdomains were identified in Mammalia (Table 2).

The percentage identity among these multiple RHD subdomains reveals that most of these domains share less percentage identity, except for some that are duplicated (two RHD-DBD and one RHD-DD have 100% identity, and four RHD-DBD and three RHD-DD have more than 85% identity) (Table 2). This result indicates that these NF-κB proteins can recognize/bind more than one DNA sequence (in case of multiple RHD-DBD) or may have binding affinity to more than one NF-κB protein (in case of multiple RHD-DD). However, experimental evidence is needed to unravel the role of these multiple RHD subdomains in these proteins.

### 2.3. NF-κB Proteins Conserved Domains and Subdomains Differ in Their Size

**Figure 3:**
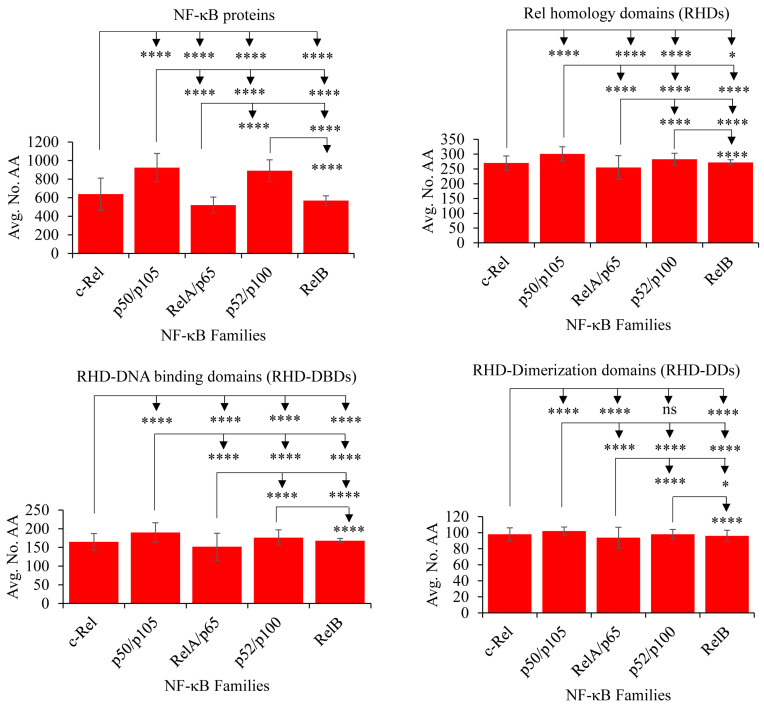
Statistical analysis of NF-κB family members protein sizes and the sizes of Rel homology domains (RHDs) and its subdomains, DNA binding domains (RHD-DBDs), and Dimerization domains (RHD-DDs). Statistical analysis was carried out as mentioned in the methods Section 3.8, and the corresponding *p*-values comparing the different NF-κB family members are presented in the figure. Detailed information on protein and domain sizes, as well as the results of Welch’s *t*-test, can be found in Tables S2 and S3. The statistical representation uses the following symbols: ns for *p* > 0.05; * for *p* ≤ 0.05; **** for *p* ≤ 0.0001.

The NF-κB family members share many conserved domains in their structures [53]. This raises the question of whether there are any differences in the proteins and domain sizes. To address this, a comprehensive analysis of the five different NF-κB family members was conducted to identify variations in complete protein lengths and the RHD and its subdomains, RHD-DBD and RHD-DD (refer to Figure 3 and Tables S2 and S3). The analysis revealed significant differences in protein sizes, RHD domain sizes, and sizes of its subdomains (RHD-DBD and RHD-DD) among all NF-κB family members. Notably, there was no significant difference in the RHD-DD size between the c-Rel and p52/p100 members (refer to Figure 3 and Table S2). Among the five NF-κB families, p50/p105 proteins displayed the largest protein lengths and RHD sizes (RHDs, RHD-DBDs, and RHD-DDs). In contrast, the RelA/p65 proteins exhibited smaller sizes in proteins, RHDs, RHD-DBDs, and RHD-DDs (refer to Figure 3 and Tables S2 and S3). The amino acid sequences of NF-κB family proteins, RHDs, RHD-DBDs, and RHD-DDs are presented in Supplementary Dataset S1.

### 2.4. RelB and c-Rel Have the Most and Least Conserved Amino Acids in the RHDs

An interesting pattern of amino acid conservation was observed for NF-κB family members in relation to RHDs (Table 3). The conservation of amino acids was not dependent on sample size (number of proteins) or the presence of the NF-κB family proteins in the different animal groups. This indicates that amino acid conservation is characteristic of each NF-κB family behavior.

Upon analyzing the amino acid conservation in the NF-κB RHDs, it was found that the RelB family had the most conserved amino acids, followed by p52/p100, RelA/p65, p50/p105, and c-Rel (Table 3). These findings suggest that the RelB family proteins underwent fewer amino acid changes, while c-Rel family proteins underwent more changes. Interestingly, despite p52/p100 and RelA/p65 being identified in vertebrates, p52/p100 ranked second regarding amino acid conservation. This suggests that the observed changes are real and not dependent on population size, as 741 p52/p100 proteins were analyzed compared to only 368 RelA/p65 proteins (Table 3).

However, a different pattern was observed for RHD-DBD and RHD-DD (Table 3). For RHD-DBD, the RelB family had the highest conserved amino acids, followed by p52/p100, RelA/p65, c-Rel, and p50/p105 (Table 3). On the other hand, for RHD-DD, the RelB family had the highest conserved amino acids, followed by RelA/p65, p52/p100, p50/p105, and c-Rel (Table 3). These differences in RHD-DBD and RHD-DD suggest that these proteins have specific regions for binding to DNA, which leads to the regulation of different genes.

In conclusion, these results indicate that c-Rel proteins mutate or evolve faster than other NF-κB family members.

### 2.5. p50/p105 and c-Rel Might Have Been the Earliest NF-κB Family Members to Emerge

**Figure 4:**
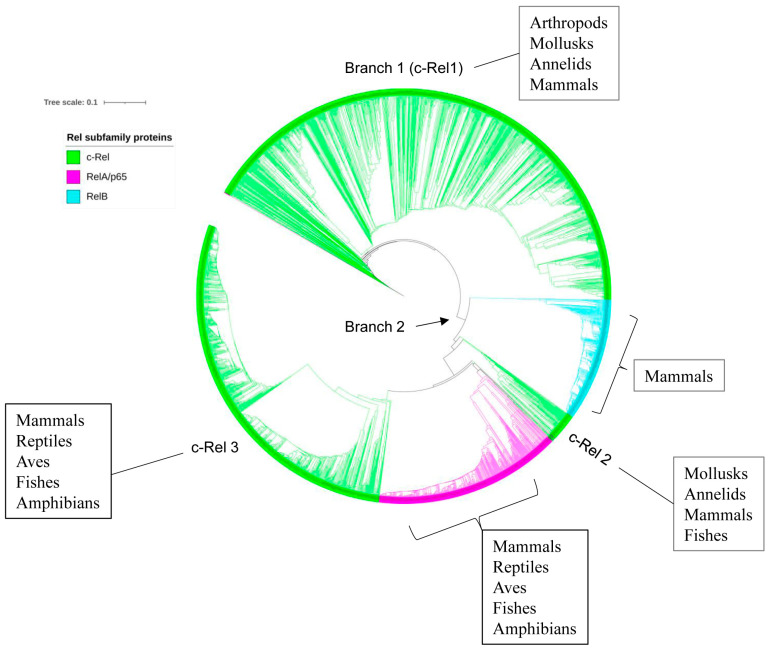
Evolutionary analysis of Rel proteins (c-Rel, RelA/p65, and RelB). The phylogenetic tree was constructed using 2461 Rel protein sequences (Supplementary Dataset S1). A high-resolution phylogenetic tree with individual node information is provided in Supplementary Figure S1.

The evolutionary analysis of Rel subfamily proteins showed that proteins from the same taxonomic groups clustered together on the phylogenetic tree except for c-Rel (Figure 4). This indicates the conservation of primary amino acid sequences after speciation. The phylogenetic tree analysis revealed that c-Rel proteins were grouped in three places (c-Rel 1–3) (Figure 4), and it is evident that the c-Rel proteins evolved and then diverged into two branches (Figure 4). Branch 1 contains various c-Rel members, mainly from arthropods, two from mollusks, one from annelids, and a single vertebrate member from a mammal, while branch 2 further evolved into RelB and RelA/p65 proteins and two more groups of c-Rel (c-Rel 2 and 3). The second c-Rel group (c-Rel 2) includes members of mollusks, annelids, mammals, and fishes, and the third group (c-Rel 3) includes only vertebrate members of fishes, amphibians, reptiles, mammals, and aves. One interesting observation was one RelA/p65 protein from the Aves at the base of branch 1, indicating its evolutionary linkage with c-Rel proteins (Figure 4).

**Figure 5:**
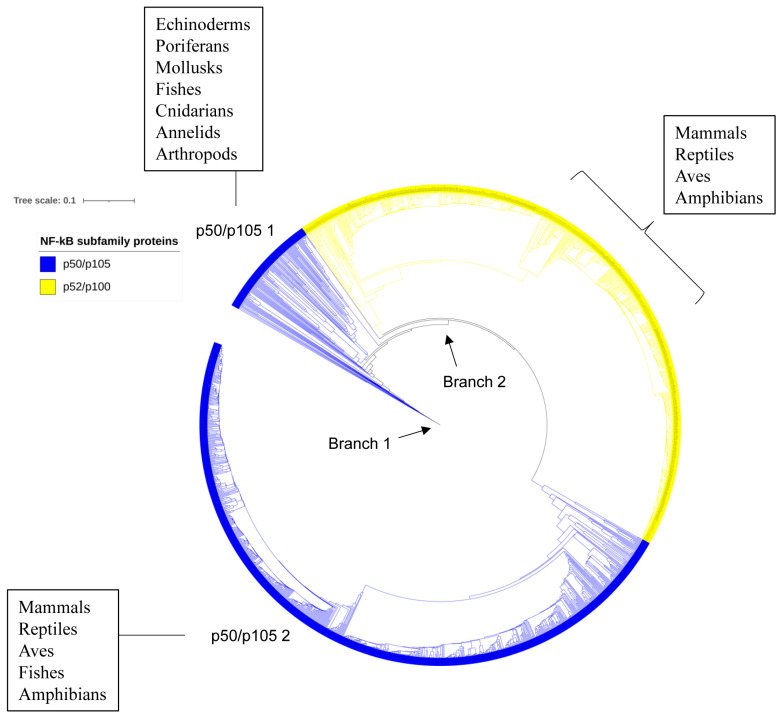
Evolutionary analysis of p50/p105 and p52/p100 proteins. The phylogenetic tree was constructed with 1683 protein sequences (Supplementary Dataset S1). A high-resolution phylogenetic tree is provided in Supplementary Figure S2, where one can see individual node information.

As observed for Rel proteins, the p50/p105 and p52/p100 proteins from the same taxonomic groups clustered on the phylogenetic tree with a few exceptions, indicating the conservation of primary amino acid sequences after speciation (Figure 5). The tree has two major branches (Figure 5). The first branch mainly consists of p50/p105 invertebrate members (echinoderms, poriferans, cnidarians, mollusks, annelids, and arthropods) and fishes (p50/p105 1), while the second branch includes p52/p100 and p50/p105 members from vertebrates (p50/p105 2) (Figure 5). This indicates that p50/p105s appeared first and then evolved into p52/p100. The first branch consists of various p50/p105 invertebrate members and fishes, while the second branch evolved into different vertebrate taxonomic groups from p52/p100 and p50/p105 (Figure 5).

**Figure 6:**
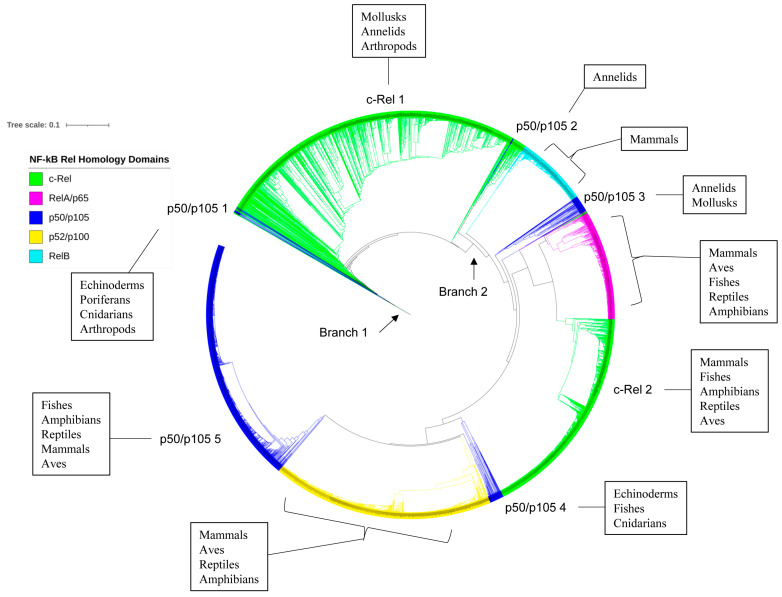
Phylogenetic analysis of Rel Homology Domain (RHD) of different NF-κB family members. 4097 NF-κB family members RHDs (Supplementary Dataset S1) were used to construct the tree. A high-resolution phylogenetic tree is provided in Supplementary Figure S3, where one can see information for each tree node.

All NF-κB family members have a common RHD, indicating that they originated from a single RHD [41]. Then, the RHD can combine with different domains, leading to the diversification of NF-κB family members [41]. Thus, the RHD may retain evolutionary traces of NF-κB family members. To understand this better, we analyzed the phylogenetic relationship of RHDs from different NF-κB members (see Figure 6). Analysis revealed that RHDs from the same NF-κB family members tend to gather together on the tree, suggesting their evolutionary conservation after diversification (Figure 6). Notably, the RHDs of p50/p105 are clustered into five groups on the tree, named p50/p105 1–5 (Figure 6). Members of the first group (p50/p105 1) are from echinoderms, poriferans, cnidarians, and arthropods. The second group (p50/p105 2) consisted of members of annelids, and the third group (p50/p105 3) included members from annelids and mollusks. The fourth group (p50/p105 4) included echinoderms, cnidarians and fishes members. The fifth group (p50/p105 5) included only vertebrate members (fishes, amphibians, reptiles, mammals, and aves) (Figure 6). RHDs of c-Rel were clustered into two large groups (c-Rel 1–2). Members of the first c-Rel group (c-Rel 1) were from arthropods, mollusks, and annelids (invertebrates), while members of the second group (c-Rel 2) were mostly from vertebrates (mammals, amphibians, reptiles, aves and fishes) (Figure 6). Based on the evolutionary analysis of their RHDs, we propose a possible evolutionary scenario for NF-κB family members, suggesting that p50/p105 and c-Rel may have been the earliest NF-κB family members to emerge in an arthropod ancestor, followed by the emergence of RelB, RelA, and p52/p100 at a later stage.

## 3. Materials and Methods

### 3.1. Reference NF-κB Family Members Used in This Study

The NF-κB proteins, which belong to five NF-κB family members (see Table 4), were used as reference proteins for genome data mining of NF-κB proteins in animals. Initially, many protein sequences were collected from the Dr. Thomas Gilmore website (https://www.bu.edu/nf-kb/the-gilmore-lab/, last accessed on 26 May 2024) for each of the NF-κB family members. Multiple sequence alignments were then carried out using Clustal Omega [54] analysis to determine the percentage identity among the collected sequences. A representative for each of the NF-κB family members was selected for each of the five families, whereas for NF-κB family members, c-Rel and p50/p105, two reference sequences were selected as representatives for vertebrates and invertebrates (Table 4). NF-κB family members from *Homo sapiens* showed more than 50% identity to the analyzed sequences and were therefore chosen as reference proteins for data mining for homolog proteins. However, for c-Rel and p50/p105, *H. sapiens* members have less than 50% identity to the invertebrates. As a result, one of the invertebrate NF-κB members was chosen as an additional reference for data mining. The rationale behind this decision is that these invertebrate NF-κB family members, which are closely related to their group, can potentially aid in the discovery of homolog NF-κB members.

### 3.2. Selection of Suitable Method for Analyzing NF-κB Family Characteristic Domains

**Figure 7:**
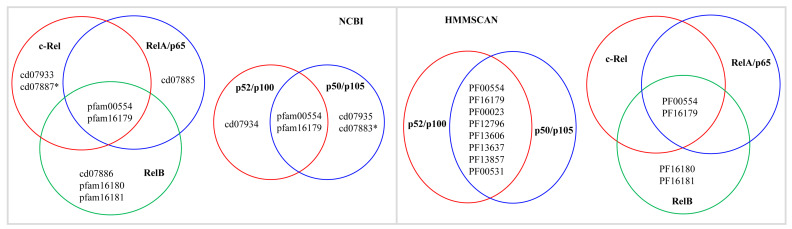
Analysis of the type and pattern of domains in protein families of NF-κB family members using NCBI Batch Web CD-search [55] and HMMSCAN [58]. An asterisk denotes the domain in invertebrate NF-κB family members for families c-Rel and p50/p105. For a detailed analysis of domains, including their names, please refer to Table 4.

Although the characteristic domains of the NF-κB family were well-defined [53], for easy identification and screening of homologous proteins on a large scale, we conducted conserved domain analysis of NF-κB reference protein sequences (Table 4) using the National Center for Biotechnology Information (NCBI) Batch Web CD-Search Tool [55] and the Hidden Markov Model Scan (HMMSCAN) [56,57] at the HMMER website [58]. This analysis helped us identify the type and pattern of domains or domain protein families (Figure 7). The Batch Web CD-Search analysis clearly distinguished between NF-κB families as the characteristic domains for each of the NF-κB families are unique, as shown in Figure 7. However, the HMMSCAN was unable to do this. As a result, we used the NCBI Batch Web CD-Search analysis to identify NF-κB family members in this study.

### 3.3. Genome Data Mining and Annotation of NF-κB Proteins in Animals

A protein BLAST (Basic Local Alignment Search Tool) [59] was performed at NCBI using the reference protein sequences from Table 4 against each of the taxonomic identification numbers (taxon ID) from Table S4 for each animal group. The reason for using NCBI taxon ID is to ensure that the resulting hit proteins are only from the specific animal group, making it easier to sort and analyze NF-κB family statistics for that group. Information on species used for genome data mining of homolog NF-κB proteins in this study, including their respective NCBI taxonomy identification numbers (Taxa ID), number of species, and the protein hits, are shown in Table S4. Protein BLAST was performed with a maximum option of 5000 hits. The hit sequences were then checked for the presence of NF-κB family characteristics domains (Figure 7) using the NCBI Batch Web CD-Search Tool [55] as mentioned in Section 3.2. Hit sequences with NF-κB family characteristics domains (Figure 7) were selected for further analysis and removed those without these domains. The proteins that had NF-κB family characteristics domains were subjected to duplicate analysis using Clustal Omega [54]. Sequences that shared 100% identity were considered duplicates, and only one of each duplicate sequence was included in the study for further analysis.

In our study, we also analyzed the NF-κB/Rel proteins found in non-metazoans from the literature [42] in order to classify them into their respective NF-κB protein families using the method detailed in Section 3.2. However, we were unable to identify the characteristic domains of the NF-κB family, as discussed in Section 3.2. Therefore, we could not assign these proteins to their respective NF-κB families. As a result, these proteins were not included in the study.

### 3.4. Generation of NF-κB Profile Heat-Map

The heat map profile illustrating the presence and absence of NF-κB protein families across different animal groups was created using a method described previously [60]. A tab-delimited file was imported into Mev (Multi-experiment viewer) [61], and the data was clustered using a hierarchical clustering algorithm with an Euclidean distance metric. NF-κB family members were represented on the vertical axis, while animal groups were displayed on the horizontal axis.

### 3.5. Phylogenetic Analysis of NF-κB Proteins

Phylogenetic analysis of the NF-κB family members and their RHDs was conducted using a method described in a published article [60]. First, the protein sequences were aligned with the MAFFT v6.864 program [62], which is available on the T-REX web server [63]. The alignments were then automatically subjected to interpret the best tree using the maximum likelihood method available on the T-rex web server [63]. Finally, the best-inferred tree was visualized, colored, and generated by the Interactive Tree of Life (iTOL) [64].

### 3.6. Identification of RHD, RHD-DBD and RHD-DD in NF-κB Proteins

The NF-κB RHDs, RHD-DBDs, and RHD-DDs were identified using the MOTIF search tool (https://www.genome.jp/tools/motif/, last accessed on 26 August 2024). Detailed information on NF-κB protein sizes and the sizes of RHDs, RHD-DBDs, and RHD-DDs can be found in Table S2, and the respective sequences are provided in Supplementary Dataset S1.

### 3.7. Analysis of Amino Acid Conservation

The number of amino acids conserved in the RHDs, RHD-DBDs, and RHD-DDs was analyzed following the method previously described [65]. The number of amino acids conserved was analyzed using PROMALS3D (Profile Multiple Alignment with predicted Local Structures and 3D constraints) [66]. The NF-κB protein sequences that had more than one RHD-DBD and RHD-DD (Table S2) were not included for analysis of amino acid conservation. PROMALS3D is an alignment tool that aligns different protein sequences using homologous crystal structures based on secondary structure prediction. The output alignment assigns numbers as conservation indices ranging from 4 to 9, where 9 is the most consistently conserved amino acid among the protein sequences analyzed [66]. The ranking was based on the number of conserved amino acids, with the NF-κB family member with the highest number of conserved amino acids given first rank, indicating that this family member is subjected to fewer mutations or high conservation during evolution.

### 3.8. Statistical Analysis of the NF-κB Proteins

Statistical analysis was performed on the sizes (number of amino acids) of NF-κB family proteins and sizes of RHDs, RHD-DBDs, and RHD-DDs using Welch’s *t*-test (https://www.statskingdom.com/150MeanT2uneq.html, last accessed on 15 July 2024) following a method described elsewhere [67]. This analysis aimed to determine if the differences between NF-κB family proteins are statistically significant. The analysis used the average number of amino acids, standard deviation, and sample size (number of NF-κB proteins) as input data (refer to Table S3). The results of the statistical analysis are presented in Table S3.

## 4. Conclusions

This study analyzes the five NF-κB family members involved in different biological processes. It shows that these proteins have unique distribution patterns across different animal groups, with significant differences in sizes, domains, subdomains, and structural conservation. The presence of RelA/p65 only in vertebrates suggests that innate immunity might have evolved primarily in fishes, followed by amphibians, reptiles, birds, and mammals. NF-κB family proteins group according to animal groups, indicating structural conservation after speciation. The study suggests that p50/p105 and c-Rel might be the earliest NF-κB family members in arthropod ancestors, with RelB, RelA/p65, and p52/p100 emerging later. Additionally, the study identified NF-κB family proteins with multiple RHD-DBDs and RHD-DDs in various species, but further research is needed to understand their functional significance.

## Figures and Tables

**Table 1:** Human NF-κB family members and their associated regulatory functions.

NF-κB Family Member	Gene Targets	Cellular Functions	Cancer-Related Functions	Reference
RelA/p65	IL-6, TNF-α, Bcl-2, IL-1β, COX-2	An important player in the canonical NF-κB pathway, it regulates gene expression related to inflammation, immune response, and cell survival.	Promotes the proliferation and survival of cancer cells by inducing anti-apoptotic genes; its overexpression is linked to aggressive cancer types.	[17,18,19,20]
RelB	IL-10, Bcl-2, CD40, MMP9, VEGF	A crucial player in the non-canonical NF-κB pathway, it regulates the development of lymphoid organs and the movement of lymphocytes.	Promotes immune evasion.	[17,19,21]
c-Rel	IL-2, IL-4, CDOL, Bcl-XL	It regulates T-cell activation and its differentiation, and influences B cell development and its function.	Associated with lymphoid malignancies, it promotes cancer cell survival and contributes to tumor formation.	[17,22,23]
p105/p50 (NF-κB1)	IL-1, TNF-α, GM-CSF, MMP9	Acts as a transcriptional repressor or activator depending on its dimerization partners. It is involved in regulating genes related to inflammation and immune responses.	It dimerizes with RelA or c-Rel to promote oncogenesis. It also promotes tumor growth and metastasis in a signal-dependent manner.	[17,21,24]
p100/p52 (NF-κB2)	IL-6, IL-10, lymphotoxin-α,	Involved in the regulation of genes associated with immune responses and lymphocyte development.	Associated with the development of certain lymphomas.	[17,21,24]

**Table 2:** NF-κB proteins with multiple Rel homology domain (RHD) subdomains, including the percentage identity between the subdomains.

NF-κB Family Member	Organism Group	Species Names	Protein ID	Protein Size	RHD-DBD Position (% Identity)	RHD-DD Position (% Identity)
c-Rel	Reptilia	*Naja naja*	KAG8123041.1 *	447	3–46	55–83 and 79–130 (17.24%)
	Mammalia	*Microtus ochrogaster*	KAH0516101.1	566	83–135 and 138–192 (21.62%)	192–261
	Mammalia	*Orycteropus afer afer*	XP_042636495.1	558	21–62 and 76–154 (19.51%)	163–258
	Mammalia	*Tupaia chinensis*	ELW63349.1	458	1–77 and 86–133 (26.92%)	133–202
	Aves	*Columbina picui*	NWQ80171.1	289	7–50 and 77–159 (22.50%)	168–263
	Aves	*Lamprotornis superbus*	KAI1240354.1	1426	115–259 and 453–500 (25.00%)	651–746
	Mollusca	*Mya arenaria*	WAR19259.1	412	89–193 and 196–233 (23.68%)	241–341
	Arthropoda	*Dermatophagoides pteronyssinus*	XP_027194418.1	794	27–150 and 152–244 (100.00%)	252–355
	Arthropoda	*Drosophila kikkawai*	KAH8308414.1	1024	51–221	229–308 and 328–352 (24.00%)
	Arthropoda	*Drosophila sulfurigaster*	KAH8391813.1	1056	63–233	241–321 and 331–362 (17.86%)
	Arthropoda	*Leptinotarsa decemlineata*	XP_023017353.1	534	122–292	3–68 and 299–397 (45.45%)
	Arthropoda	*Drosophila pandora*	KAH8323486.1	994	53–223	231–317 and 320–350 (23.33%)
	Arthropoda	*Drosophila bipectinata*	KAH8278094.1	1000	55–225	233–313 and 325–350 (20.00%)
	Arthropoda	*Drosophila pseudoananassae*	KAH8324519.1	998	53–223	231–313 and 323–348 (22.22%)
	Arthropoda	*Pseudolycoriella hygida*	KAJ6649769.1	1011	74–244	252–348 and 447–469 (88.89%)
	Arthropoda	*Gonioctena quinquepunctata*	KAG5873706.1	818	76–201 and 207–288 (43.24%)	296–394
	Arthropoda	*Aphidius gifuensis*	KAF7994522.1	694	75–245	253–352 and 431–533 (41.00%)
	Arthropoda	*Phthorimaea operculella*	KAI5633865.1	804	67–193 and 198–274 (94.29%)	281–389
	Arthropoda	*Spodoptera exigua*	CAH0702166.1	1373	17–140 and 467–625 (35.40%)	633–731
	Arthropoda	*Temnothorax longispinosus*	TGZ37724.1 *	1121	133–255 and 254–280 (29.17%)	288–388
	Arthropoda	*Timema shepardi*	CAD7261384.1	722	2–89 and 122–165 (25.00%)	173–273
	Arthropoda	*Apis florea*	XP_031771860.1	460	12–91 and 95–134 (17.50%)	142–242
	Arthropoda	*Apis dorsata*	XP_006618954.1 *	466	10–91 and 88–134 (22.86%)	142–242
	Arthropoda	*Diaphorina citri*	KAI5744658.1	480	48–146 and 151–188 (15.79%)	197–293
	Arthropoda	*Diaphorina citri*	KAI5710357.1	512	48–146 and 151–188 (15.79%)	197–293
	Arthropoda	*Drosophila birchii*	KAH8245463.1	1015	80–250	257–336 and 354–380 (25.93%)
	Arthropoda	*Rhagoletis zephyria*	XP_017461252.1	307	21–144 and 147–218 (85.71%)	256–307
	Arthropoda	*Choristoneura fumiferana*	KAI8422849.1	639	327–382 and 432–593 (22.64%)	600–624
	Arthropoda	*Timema bartmani*	CAD7444423.1	1022	68–123 and 126–170 (30.56%)	178–278
	Arthropoda	*Aphidius gifuensis*	XP_044005943.1	340	20–93 and 104–164 (34.38%)	170–268
	Arthropoda	*Copidosoma floridanum*	XP_023248498.1	1289	25–199 and 348–522 (29.76%)	209–308 and 533–629 (32.61%)
p50/p105	Cnidaria	*Orbicella faveolata*	XP_020612557.1	1121	45–260	269–295 and 307–410 (100.00%)
	Reptilia	*Thamnophis sirtalis*	XP_013917902.1	589	32–230 and 265–424 (98.11%)	239–268 and 433–534 (93.33%)
	Mammalia	*Microtus ochrogaster*	KAH0500796.1	1000	11–49 and 64–226 (20.51%)	235–336
	Mammalia	*Heterocephalus glaber*	EHB13915.1	1164	125–166 and 198–340 (26.19%)	349–450
	Mollusca	*Plakobranchus ocellatus*	GFN89059.1	1056	67–254	263–290 and 324–377 (14.29%)
RelA/p65	Mammalia	*Galemys pyrenaicus*	KAG8522598.1	756	204–369	378–404 and 424–494 (20.00%)
	Mammalia	*Sousa chinensis*	TEA11684.1	591	17–62 and 102–226 (18.60%)	235–331
	Aves	*Melopsittacus undulatus*	XP_033927754.1	405	13–54 and 72–221 (96.15%)	230–326
	Aves	*Onychostruthus taczanowskii*	XP_041269398.1	443	1–32 and 40–115 (12.90%)	124–220
p52/p100	Mammalia	*Galemys pyrenaicus*	KAG8523592.1	955	70–114 and 116–274 (100.00%)	283–381
	Aves	*Pygoscelis adeliae*	KFW65979.1	657	30–122 and 172–220 (17.39%)	229–279
	Aves	*Pygoscelis adeliae*	XP_009320748.1	568	9–101 and 116–163 (17.78%)	172–224
RelB	Mammalia	*Pteropus alecto*	XP_024902475.1	637	154–322	331–357 and 401–456 (13.04%)
	Mammalia	*Tupaia chinensis*	ELW71096.1 *	547	72–240	249–322 and 318–369 (82.76%)
	Mammalia	*Propithecus coquereli*	XP_012508693.1	559	112–202 and 232–275 (26.32%)	284–380
	Mammalia	*Neotoma lepida*	OBS57525.1	455	44–132 and 138–166 (21.43%)	175–276

Note: proteins with overlapping subdomains are indicated with an asterisk. The percentage identity between the domains is indicated in brackets.

**Table 3:** Comparative analysis of amino acid conservation in NF-κB RHDs and subdomains. Each NF-κB family member was ranked based on the number of conserved amino acids (indicated by the number 9). The multiple sequence alignment of domains is provided in Supplementary Dataset S2.

NF-κB Family	Number of NF-κB Protein Sequences	PROMALS3D Conservation Index					Ranking
		5	6	7	8	9	
Rel homology domain (RHD)							
c-Rel	1814	18	10	9	1	2	V
p50/p105	934	28	12	25	11	7	IV
RelA/p65	368	30	0	82	0	92	III
p52/p100	741	15	33	69	20	93	II
RelB	240	23	92	0	0	126	I
Rel homology domain-DNA binding domain (RHD-DBD)							
c-Rel	1814	8	10	5	0	2	IV
p50/p105	934	16	8	12	6	2	IV
RelA/p65	368	19	0	47	0	51	III
p52/p100	741	10	20	37	8	59	II
RelB	240	0	59	0	0	72	I
Rel homology domain-dimerization domain (RHD-DD)							
c-Rel	1814	3	3	5	1	2	V
p50/p105	934	14	11	7	6	7	IV
RelA/p65	368	10	5	22	0	40	II
p52/p100	741	26	20	21	0	32	III
RelB	240	0	16	0	0	72	I

**Table 4:** Information on the reference proteins utilized for genome data mining of homologous NF-κB family members. The table also presents protein characteristics, such as protein size and domains, as per the NCBI Batch Web CD-Search database [55].

NF-κB Family	NCBI Protein ID	Organism	Protein Size	NCBI Batch Web CD-Search Domain	
				Accession	Short name
c-Rel	CAA52954.1	*Homo sapiens*	619	c07933	RHD-n_c-Rel
				pfam00554	RHD_DNA_bind
				pfam16179	RHD_dimer
c-Rel	AAK72690.1	*Crassostrea gigas*	615	cd07887	RHD-n_Dorsal_Dif
				pfam00554	RHD_DNA_bind
				pfam16179	RHD_dimer
RelA/p65	AAA36408.1	*Homo sapiens*	551	cd07885	RHD-n_RelA
				pfam00554	RHD_DNA_bind
				pfam16179	RHD_dimer
RelB	NP_006500.2	*Homo sapiens*	579	cd07886	RHD-n_RelB
				pfam16181	RelB_transactiv
				pfam00554	RHD_DNA_bind
				pfam16179	RHD_dimer
				pfam16180	RelB_leu_zip
p50/p105	AAA36361.1	*Homo sapiens*	969	cd07935	RHD-n_NFkB1
				pfam00554	RHD_DNA_bind
				pfam16179	RHD_dimer
p50/p105	NP_999819.1	*Strongylocentrotus purpuratus*	1125	cd07883	RHD-n_NFkB
				pfam00554	RHD_DNA_bind
				pfam16179	RHD_dimer
p52/p100	NP_002493.3	*Homo sapiens*	899	cd07934	RHD-n_NFkB2
				pfam00554	RHD_DNA_bind
				pfam16179	RHD_dimer

## Data Availability

The data generated in the study is provided in the manuscript and Supplementary Materials.

## Supplementary Materials

The following supporting information can be downloaded at https://www.mdpi.com/article/10.3390/ijms25189793/s1; refs in Table S4 are cited as [68,69,70,71].
