# Supplementary figures and images for: NF-κB Transcription Factors: Their Distribution, Family Expansion, Structural Conservation, and Evolution in Animals
Source: Int J Mol Sci. 2024 Sep 10;25(18):9793. doi: 10.3390/ijms25189793 (PMC11432056; doi:10.3390/ijms25189793)

Tree scale: 0.1

### Rel subfamily proteins

- c-Rel
- RelA/p65
- RelB

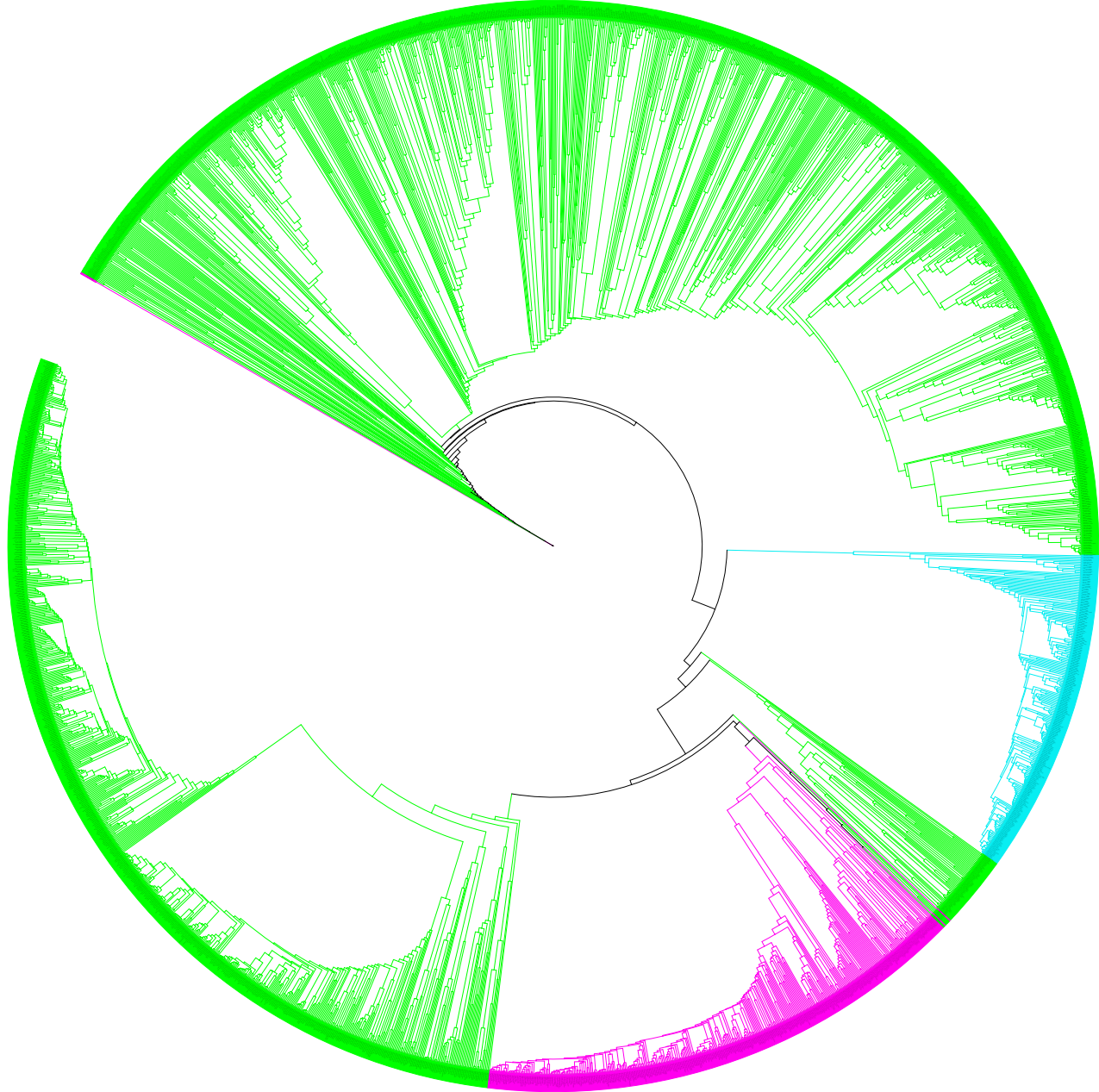

Supplement: Supplementary file 1 [file ijms-25-09793-s001.zip › Figure S1.pdf]

Tree scale: 0.1

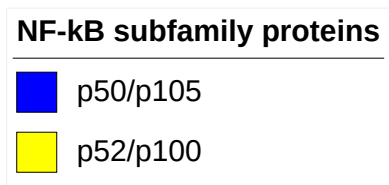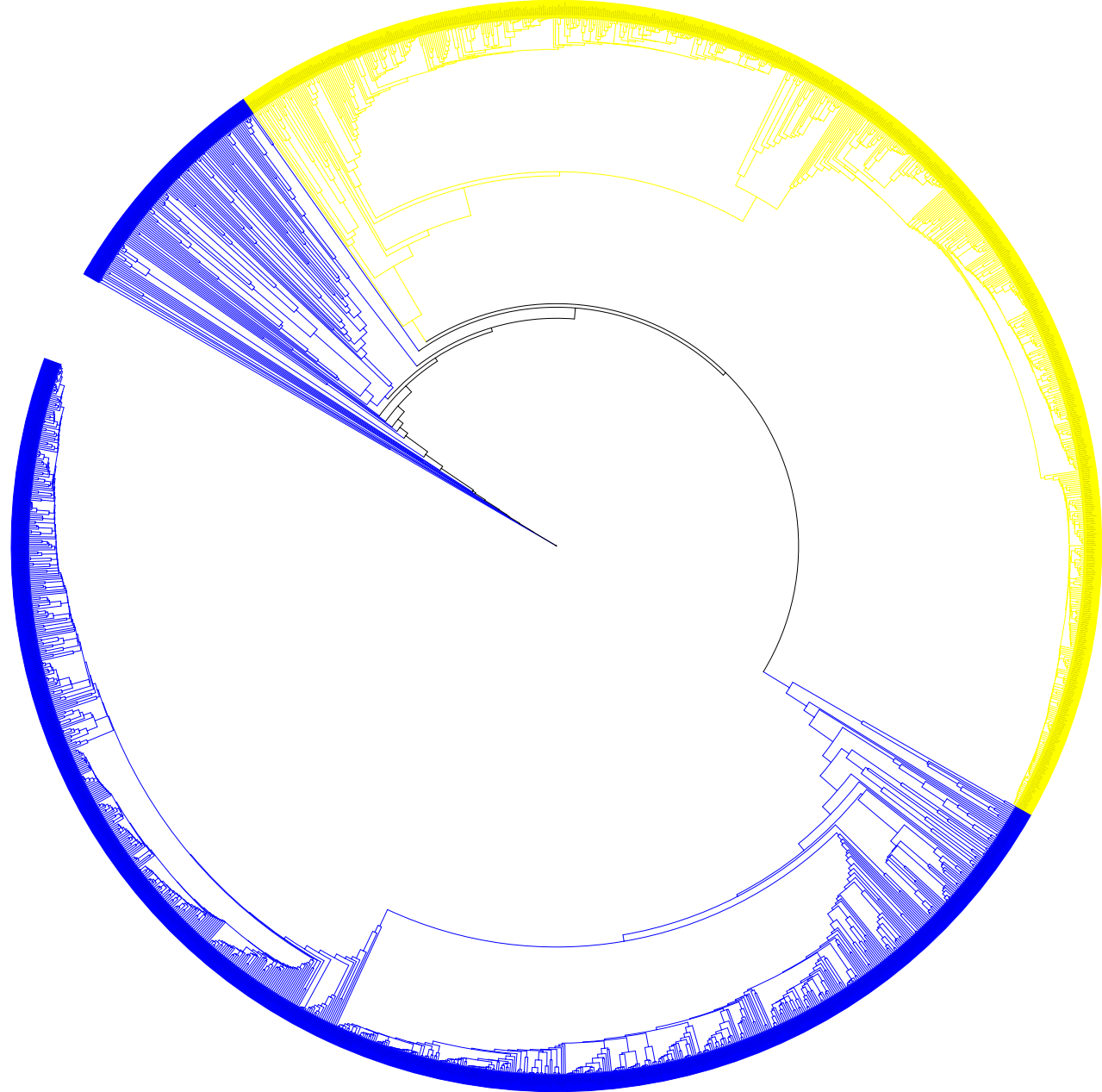

Supplement: Supplementary file 1 [file ijms-25-09793-s001.zip › Figure S2.pdf]

Tree scale: 0.1

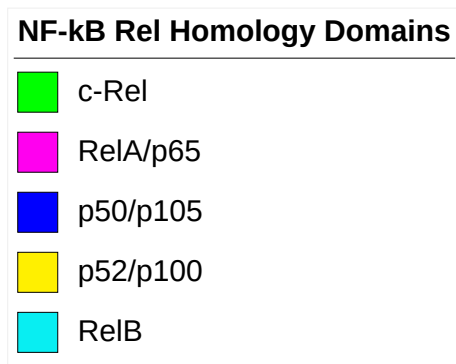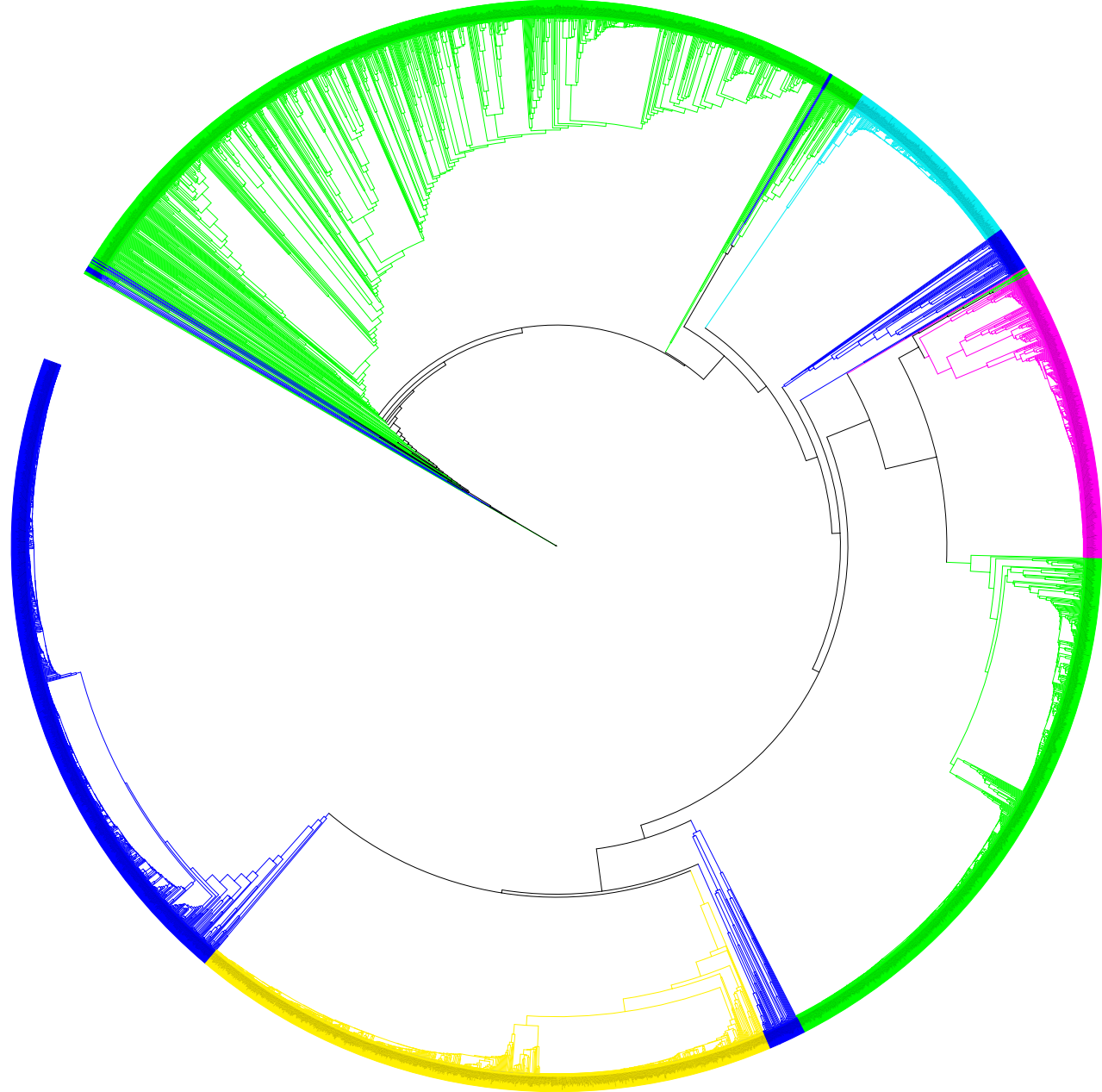

Supplement: Supplementary file 1 [file ijms-25-09793-s001.zip › Figure S3.pdf]
