# Supplementary material for: NF-κB Transcription Factors: Their Distribution, Family Expansion, Structural Conservation, and Evolution in Animals
Source: Int J Mol Sci. 2024 Sep 10;25(18):9793. doi: 10.3390/ijms25189793 (PMC11432056; doi:10.3390/ijms25189793)
Supplement: Supplementary file 1 [file Supplementary Dataset 2.pdf › Supplementary Dataset 2.pdf]

Supplementary Dataset 2: The multiple sequence alignments of Rel homology domains (RHD), Rel homology domain-DNA binding domains (RHD-DBD), and Rel homology domain-Dimerization domains (RHD-DD) in each NF-κB family member.

RHD

c-Rel

```
Conservation:             6 579       5777        7               5 6                      5
_XP_001652840.2_    1  -VVITEQPQ-SKAL-RFRYEC------E---------GRSAGSIPGVHSTP---EQK---------TFPG   40
_AAW67214.1_        1  -VVITEQPQ-SKAL-RFRYEC------E---------GRSAGSIPGVHSTP---EQK---------TFPG   40
_XP_021704027.1_    1  -VVITEQPQ-SKAL-RFRYEC------E---------GRSAGSIPGVHSTP---EQK---------TFPG   40
_XP_019540833.2_    1  -VVITEQPQ-SKAL-RFRYEC------E---------GRSAGSIPGVHSTP---EQK---------TFPG   40
_XP_029720231.1_    1  -VVITEQPQ-SKAL-RFRYEC------E---------GRSAGSIPGVHSTP---EQK---------TFPG   40
_AAW67215.1_        1  -VVITEQPQ-SKAL-RFRYEC------E---------GRSAGSIPGVHSTP---EQK---------TFPG   40
_XP_021704025.1_    1  -VVITEQPQ-SKAL-RFRYEC------E---------GRSAGSIPGVHSTP---EQK---------TFPG   40
_XP_019540831.2_    1  -VVITEQPQ-SKAL-RFRYEC------E---------GRSAGSIPGVHSTP---EQK---------TFPG   40
_KXJ81105.1_        1  -VVITEQPQ-SKAL-RFRYEC------E---------GRSAGSIPGVHSTP---EQK---------TFPG   40
_KXJ76497.1_        1  -VVITEQPQ-SKAL-RFRYEC------E---------GRSAGSIPGVHSTP---EQK---------TFPG   40
_XP_023240428.1_    1  -IVILEQPA-SRSI-RFRYEC------E---------GRSAGSIPGVNSTS---TNK---------TYPT   40
_CAD7241875.1_      1  -VRIVEQPA-PKEH-RFRYKC------E---------GRSAGSIPGINSTE---ENK---------TYPT   40
_TRY80291.1_        1  -VKVLEEPA-DNKL-RFRYEC------E---------GRSAGALHGGKSSG---DNK---------TYPT   40
_ALF84899.1_        1  -VKITEQPA-SNKL-RFRYEC------E---------GRSAGALQGARSSS---ENK---------TFPS   40
_KAF4528208.1_      1  --VMLEQPA-PKAL-RFRYEC------E---------GRSAGSIPGVNSTP---ENK---------TYPT   39
_XP_054260955.1_    1  -IKIVEQPA-SKAL-RFRYEC------E---------GRSAGSIPGVNSST---ENK---------TYPT   40
_XP_054264401.1_    1  -IKIVEQPA-SKAL-RFRYEC------E---------GRSAGSIPGVNSST---ENK---------TYPT   40
_XP_054260954.1_    1  -IKIVEQPA-SKAL-RFRYEC------E---------GRSAGSIPGVNSST---ENK---------TYPT   40
_XP_038114042.1_    1  -VVITEQPQ-SKAL-RFRYEC------E---------GRSAGSIPGVHSTP---EQK---------TFPS   40
_XP_039437558.1_    1  -VVITEQPQ-SKAL-RFRYEC------E---------GRSAGSIPGVHSTP---EQK---------TFPS   40
_XP_001844078.2_    1  -VVITEQPQ-SKAL-RFRYEC------E---------GRSAGSIPGVHSTP---EQK---------TFPS   40
_EDS35747.1_        1  -VVITEQPQ-SKAL-RFRYEC------E---------GRSAGSIPGVHSTP---EQK---------TFPS   40
_XP_053696925.1_    1  -VVITEQPQ-SKAL-RFRYEC------E---------GRSAGSIPGVNSTP---EQK---------TFPS   40
_XP_001869619.2_    1  -VVITEQPQ-SKGL-RFRYEC------E---------GRSAGSIPGVRSTT---EHK---------THPT   40
_XP_039434954.1_    1  -VVITEQPQ-SKGL-RFRYEC------E---------GRSAGSIPGVRSTT---EHK---------THPT   40
_EDS29879.1_        1  -VVITEQPQ-SKGL-RFRYEC------E---------GRSAGSIPGVRSTT---EHK---------THPT   40
_XP_019550742.2_    1  -IVITEQPQ-SKAL-RFRYEC------E---------GRSAGSIPGVSSTA---EQK---------TFPT   40
_KXJ74587.1_        1  -IVITEQPQ-SKAL-RFRYEC------E---------GRSAGSIPGVSSTA---EQK---------TFPT   40
_XP_035908773.1_    1  -VEITEQPH-PKAL-RFRYEC------E---------GRSAGSIPGVSTTA---EHK---------TFPS   40
```

```
_AJC98655.1_        1  -VEITEQPH-PKAL-RFRYEC------E---------GRSAGSIPGVNTTA---EHK---------TFPS   40
_AJC98650.1_        1  -VEITEQPH-PKAL-RFRYEC------E---------GRSAGSIPGVNTTA---EHK---------TFPS   40
_AJC98644.1_        1  -VEITEQPH-PKAL-RFRYEC------E---------GRSAGSIPGVNTTA---EHK---------TFPS   40
_AJC98657.1_        1  -VEITEQPH-PKAL-RFRYEC------E---------GRSAGSIPGVNTTA---EHK---------TFPS   40
_AJC98648.1_        1  -VEITEQPH-PKAL-RFRYEC------E---------GRSAGSIPGVNTTA---EHK---------TFPS   40
_AJC98647.1_        1  -VEITEQPH-PKAL-RFRYEC------E---------GRSAGSIPGVNTTA---EHK---------TFPS   40
_AJC98651.1_        1  -VEITEQPH-PKAL-RFRYEC------E---------GRSAGSIPGVNTTA---EHK---------TFPS   40
_AJC98643.1_        1  -VEITEQPH-PKAL-RFRYEC------E---------GRSAGSIPGVNTTA---EHK---------TFPS   40
_AJC98656.1_        1  -VEITEQPH-PKAL-RFRYEC------E---------GRSAGSIPGVNTTA---EHK---------TFPS   40
_AJC98641.1_        1  -VEITEQPH-PKAL-RFRYEC------E---------GRSAGSIPGVNTTA---EHK---------TFPS   40
_AJC98640.1_        1  -VEITEQPH-PKAL-RFRYEC------E---------GRSAGSIPGVNTTA---EHK---------TFPS   40
_AJC98653.1_        1  -VEITEQPH-PKAL-RFRYEC------E---------GRSAGSIPGVNTTA---EHK---------TFPS   40
_AJC98654.1_        1  -VEITEQPH-PKAL-RFRYEC------E---------GRSAGSIPGVNTTA---EHK---------TFPS   40
_AJC98652.1_        1  -VEITEQPH-PKAL-RFRYEC------E---------GRSAGSIPGVNTTA---EHK---------TFPS   40
_AJC98646.1_        1  -VEITEQPH-PKAL-RFRYEC------E---------GRSAGSIPGVNTTA---EHK---------TFPS   40
_AJC98658.1_        1  -VEITEQPH-PKAL-RFRYEC------E---------GRSAGSIPGVNTTA---EHK---------TFPS   40
_AJC98639.1_        1  -VEITEQPH-PKAL-RFRYEC------E---------GRSAGSIPGVNTTA---EHK---------TFPS   40
_XP_035908772.1_    1  -VEITEQPH-PKAL-RFRYEC------E---------GRSAGSIPGVSTTA---EHK---------TFPS   40
_XP_050076803.1_    1  -VEITEQPH-PKAL-RFRYEC------E---------GRSAGSIPGVSTTA---EHK---------TFPS   40
_XP_049282441.1_    1  -VEITEQPH-PKAL-RFRYEC------E---------GRSAGSIPGVSTTS---EHK---------TFPS   40
_XP_040170080.1_    1  -VEITEQPH-PKAL-RFRYEC------E---------GRSAGSIPGVNTTA---EQK---------TFPS   40
_XP_050076802.1_    1  -VEITEQPH-PKAL-RFRYEC------E---------GRSAGSIPGVSTTA---EHK---------TFPS   40
_XP_041780138.1_    1  -VEITEQPH-PKAL-RFRYEC------E---------GRSAGSIPGVNTTA---EHK---------TFPS   40
_XP_040239229.2_    1  -VEITEQPH-PKAL-RFRYEC------E---------GRSAGSIPGVNTTA---EHK---------TFPS   40
_XP_040239222.2_    1  -VEITEQPH-PKAL-RFRYEC------E---------GRSAGSIPGVNTTA---EHK---------TFPS   40
_XP_035908780.1_    1  -VEITEQPH-PKAL-RFRYEC------E---------GRSAGSIPGVSTTA---EHK---------TFPS   40
_XP_040239221.2_    1  -VEITEQPH-PKAL-RFRYEC------E---------GRSAGSIPGVNTTA---EHK---------TFPS   40
_XP_052897786.1_    1  -VEITEQPH-PKAL-RFRYEC------E---------GRSAGSIPGVSTTT---EHK---------TFPS   40
_XP_052895493.1_    1  -VEITEQPH-PKAL-RFRYEC------E---------GRSAGSIPGVSTTT---KHK---------TFPS   40
_XP_053664395.1_    1  -VEITEQPH-PKAL-RFRYEC------E---------GRSAGSIPGVSTTT---DHK---------TFPS   40
_XP_050076801.1_    1  -VEITEQPH-PKAL-RFRYEC------E---------GRSAGSIPGVSTTA---EHK---------TFPS   40
_XP_310177.3_       1  -VEITEQPH-PKAL-RFRYEC------E---------GRSAGSIPGVNTTA---EQK---------TFPS   40
_XP_001652339.1_    1  -VVITEQPQ-SKAL-RFRYEC------E---------GRSAGSIPGVSSTA---DHK---------TFPT   40
_XP_053686256.1_    1  -VAITEQPQ-SKAL-RFRYEC------E---------GRSAGSIPGVHSTP---EQK---------TFPA   40
_XP_053675540.1_    1  -VEITEQPH-PKAL-RFRYEC------E---------GRSAGSIPGINTTS---DHK---------TFPS   40
_ETN66814.1_        1  -VEITEQPH-PKAI-RFRYLC------E---------GRSAGSIPGVNTTA---DNK---------TFPT   40
_XP_049534501.1_    1  -VEITEQPH-PKAI-RFRYLC------E---------GRSAGSIPGVNTTA---DNK---------TFPT   40
_XP_050084431.1_    1  -VEITEQPH-PKAI-RFRYLC------E---------GRSAGSIPGVNTTA---DNK---------TFPT   40
_XP_035782874.1_    1  -VEITEQPH-PKAI-RFRYLC------E---------GRSAGSIPGVNTTA---DNK---------TFPT   40
```

```
_XP_035782869.1_      1  -VEITEQPH-PKAI-RFRYLC------E---------GRSAGSIPGVNTTA---DNK---------TFPT   40
_KFB39849.1_          1  -VEITEQPH-PKAL-RFRYEC------E---------GRSAGSIPGIHSTN---DHK---------TYPS   40
_XP_052871375.1_      1  -MEITEQPH-PKAL-RFRYEC------E---------GRSAGSIPGVNTTA---DHK---------TFPS   40
_AAR13743.1_          1  ------------------YEC------E---------GRSAGSIPGVNTTA---EQK---------TFPS   25
_AAR13744.1_          1  ------------------YEC------E---------GRSAGSIPGVNTTA---EHK---------TFPS   25
_CAA65157.1_          1  ---------------------------------------SAGSIPGVNTTA---EHK---------TFPS   19
_XP_022170156.1_      1  -IKIVEQPA-SKAL-RFRYEC------E---------GRSAGSIPGVNSST---ENK---------TYPT   40
_XP_025205168.1_      1  -IKIVEQPA-SKAL-RFRYEC------E---------GRSAGSIPGVNSTT---ENK---------TYPT   40
_XP_026817162.1_      1  -IKIVEQPA-SKAL-RFRYEC------E---------GRSAGSIPGVNSTT---ENK---------TYPT   40
_XP_027846205.1_      1  -IKIVEQPA-SKAL-RFRYEC------E---------GRSAGSIPGVNSTT---ENK---------TYPT   40
_XP_015370635.1_      1  -IKIVEQPA-SKAL-RFRYEC------E---------GRSAGSIPGVNSSS---ENK---------TYPT   40
_CAH1723797.1_        1  -IKIVEQPA-SKAL-RFRYEC------E---------GRSAGSIPGVNSTT---ENK---------TYPT   40
_KAF0769940.1_        1  -IKIVEQPA-SKAL-RFRYEC------E---------GRSAGSIPGVNSTT---ENK---------TYPT   40
_CAB4065202.1_        1  -LQIIEQPA-YNKL-RFRYEC------E---------GRSAGALVGENTKD---KKR---------TYPT   40
_XP_050440903.1_      1  -IKIVEQPA-SKAL-RFRYEC------E---------GRSAGSIPGVNSTT---ENK---------TYPT   40
_XP_050533799.1_      1  -IKIVEQPA-SKAL-RFRYEC------E---------GRSAGSIPGVNSTT---ENK---------TYPT   40
_XP_025410632.1_      1  -IEIIEQPA-SKAL-RFRYEC------E---------GRSAGSIPGVNSTT---ENK---------TYPT   40
_XP_008180746.1_      1  -IKIVEQPA-SKAL-RFRYEC------E---------GRSAGSIPGVNSST---ENK---------TYPT   40
_XP_026465450.1_      1  -VRIIEQPA-PKAL-RFRYEC------E---------GRSAGSIPGISSTP---EYR---------TFPS   40
_VVC36026.1_          1  -IKIIEQPA-SKAL-RFRYEC------E---------GRSAGSIPGVNSTP---ENK---------TYPT   40
_XP_016661522.1_      1  -IKIVEQPA-SKAL-RFRYKC------E---------GRLAGSIPGVNSST---ENK---------TYPT   40
_XP_001947429.3_      1  -IKIVEQPA-SKAL-RFRYKC------E---------GRLAGSIPGVNSST---ENK---------TYPT   40
_XP_026466007.1_      1  -VRIIEQPA-PKAL-RFRYEC------E---------GRSAGSIPGINSTP---EYR---------TFPS   40
_KRT81254.1_          1  ---IVEQPA-EKRI-RFRYAC------E---------GRLTGSIPGVNSTP---ERK---------TYPT   38
_XP_033224832.1_      1  -IKILEQPS--TGI-RFRYEI------E---------GRSAGSIPGRRSTP---DQK---------TYPT   39
_XP_025407590.1_      1  -IEIIEQPDSV----RYRYVC------E---------GRTAGNIFGVNSLY---DNK---------TYPT   38
_XP_045476729.1_      1  -VKIIEQPA-PDRA-RFRYQS------E---------TFS--TLMGVSSTK---KQK---------TYPT   38
_XP_034174253.1_      1  -VEIIEQPA-SKAL-RFRYEC------E---------GRSAGSIPGVNSTP---ENK---------TFPS   40
_XP_029047866.1_      1  -VEIIEQPA-SKAL-RFRYEC------E---------GRSAGSIPGVNSTP---ENK---------TFPS   40
_XP_015430648.1_      1  -VEIIEQPA-SKAL-RFRYEC------E---------GRSAGSIPGVNSTP---ENK---------TFPS   40
_XP_017798081.1_      1  -VEIVEQPA-SKAL-RFRYEC------E---------GRSAGSIPGVNSTP---ENK---------TFPS   40
_XP_017756471.1_      1  -VEIIEQPA-SKAL-RFRYEC------E---------GRSAGSIPGVNSTP---ENK---------TFPS   40
_XP_043248447.1_      1  -VEIIEQPA-SKAL-RFRYEC------E---------GRSAGSIPGVNSTP---ENK---------TFPS   40
_XP_029047855.1_      1  -VEIIEQPA-SKAL-RFRYEC------E---------GRSAGSIPGVNSTP---ENK---------TFPS   40
_XP_034174252.1_      1  -VEIIEQPA-SKAL-RFRYEC------E---------GRSAGSIPGVNSTP---ENK---------TFPS   40
_XP_031829337.1_      1  -VEIIEQPA-SKAL-RFRYEC------E---------GRSAGSIPGVNSTP---ENK---------TFPS   40
_KZC09125.1_          1  -VEIIEQPA-SKAL-RFRYEC------E---------GRSAGSIPGVNSTP---ENK---------TFPS   40
_XP_033327213.1_      1  -VEITEQPA-SKAL-RFRYEC------E---------GRSAGSIPGVNSTP---ENK---------IFPS   40
_XP_033327212.1_      1  -VEITEQPA-SKAL-RFRYEC------E---------GRSAGSIPGVNSTP---ENK---------IFPS   40
```

```
_XP_017756445.1_    1  -VEIIEQPA-SKAL-RFRYEC------E---------GRSAGSIPGVNSTP---ENK---------TFPS  40
_OAD62393.1_        1  -VEIIEQPA-SKAL-RFRYEC------E---------GRSAGSIPGVNSTP---ENK---------TFPS  40
_XP_043248413.1_    1  -VEIIEQPA-SKAL-RFRYEC------E---------GRSAGSIPGVNSTP---ENK---------TFPS  40
_XP_033327208.1_    1  -VEITEQPA-SKAL-RFRYEC------E---------GRSAGSIPGVNSTP---ENK---------IFPS  40
_XP_034950477.1_    1  -VEIVEQPA-SKAL-RFRYEC------E---------GRSAGSIPGVNSTP---ENK---------TFPS  40
_XP_034950475.1_    1  -VEIVEQPA-SKAL-RFRYEC------E---------GRSAGSIPGVNSTP---ENK---------TFPS  40
_XP_012343274.1_    1  -VEIIEQPA-SKAL-RFRYEC------E---------GRSAGSIPGVNSTS---ENK---------TFPT  40
_XP_016909449.1_    1  -VEIIEQPA-SKAL-RFRYEC------E---------GRSAGSIPGVNSTS---ENK---------TFPT  40
_XP_012343273.1_    1  -VEIIEQPA-SKAL-RFRYEC------E---------GRSAGSIPGVNSTS---ENK---------TFPT  40
_XP_006619742.1_    1  -VEIIEQPA-SKAL-RFRYEC------E---------GRSAGSIPGVNSTS---ENK---------TFPT  40
_XP_006567065.1_    1  -VEIIEQPA-SKAL-RFRYEC------E---------GRSAGSIPGVNSTS---ENK---------TFPT  40
_XP_006619741.1_    1  -VEIIEQPA-SKAL-RFRYEC------E---------GRSAGSIPGVNSTS---ENK---------TFPT  40
_NP_001011577.1_    1  -VEIIEQPA-SKAL-RFRYEC------E---------GRSAGSIPGVNSTS---ENK---------TFPT  40
_XP_016909448.1_    1  -VEIIEQPA-SKAL-RFRYEC------E---------GRSAGSIPGVNSTS---ENK---------TFPT  40
_XP_043802365.1_    1  -VEIIEQPA-SKAL-RFRYEC------E---------GRSAGSIPGVNSTS---ENK---------TFPT  40
_NP_001164477.1_    1  -VEIIEQPA-SKAL-RFRYEC------E---------GRSAGSIPGVNSTS---ENK---------TFPT  40
_XP_016909443.1_    1  -VEIIEQPA-SKAL-RFRYEC------E---------GRSAGSIPGVNSTS---ENK---------TFPT  40
_XP_043802361.1_    1  -VEIIEQPA-SKAL-RFRYEC------E---------GRSAGSIPGVNSTS---ENK---------TFPT  40
_XP_012343269.1_    1  -VEIIEQPA-SKAL-RFRYEC------E---------GRSAGSIPGVNSTS---ENK---------TFPT  40
_XP_006619738.1_    1  -VEIIEQPA-SKAL-RFRYEC------E---------GRSAGSIPGVNSTS---ENK---------TFPT  40
_PBC29878.1_        1  -VEIIEQPA-SKAL-RFRYEC------E---------GRSAGSIPGVNSTS---ENK---------TFPT  40
_XP_046663984.1_    1  -IKIVEQPA-SKAL-RFRYEC------E---------GRSAGSIPGVNSTP---ENK---------TYPT  40
_XP_046663985.1_    1  -IKIVEQPA-SKAL-RFRYEC------E---------GRSAGSIPGVNSTP---ENK---------TYPT  40
_XP_043274771.1_    1  -VEIIEQPA-SKAL-RFRYEC------E---------GRSAGSIPGINSTV---ENK---------TFPS  40
_XP_043274769.1_    1  -VEIIEQPA-SKAL-RFRYEC------E---------GRSAGSIPGINSTV---ENK---------TFPS  40
_XP_043289571.1_    1  -VEIVEQPA-SKAL-RFRYEC------E---------GRSAGSIPGANSSS---EYK---------TYPT  40
_XP_032665873.1_    1  -VKIIEQPA-SKAL-RFRYEC------E---------GRSAGSIPGANSTP---ENK---------TFPS  40
_XP_032665875.1_    1  -VKIIEQPA-SKAL-RFRYEC------E---------GRSAGSIPGANSTP---ENK---------TFPS  40
_XP_032665874.1_    1  -VKIIEQPA-SKAL-RFRYEC------E---------GRSAGSIPGANSTP---ENK---------TFPS  40
_XP_032665869.1_    1  -VKIIEQPA-SKAL-RFRYEC------E---------GRSAGSIPGANSTP---ENK---------TFPS  40
_XP_014470289.1_    1  -VKIIEQPA-SKAL-RFRYEC------E---------GRSAGSIPGANSTP---ENK---------TFPS  40
_XP_011149046.1_    1  -VKIMEQPA-SKAL-RFRYEC------E---------GRSAGSIPGANSTP---ENK---------TFPS  40
_XP_017877914.1_    1  --EIVEQPA-SKAL-RFRYEC------E---------GRSAGSIPGVNSTP---ESK---------TFPS  39
_XP_017877911.1_    1  --EIVEQPA-SKAL-RFRYEC------E---------GRSAGSIPGVNSTP---ESK---------TFPS  39
_XP_033174774.1_    1  -VEIIEQPA-SKAL-RFRYEC------E---------GRSAGSIPGVNSTP---ENK---------TFPS  40
_XP_043581013.1_    1  -VEIIEQPA-SKAL-RFRYEC------E---------GRSAGSIPGINSTP---ENK---------TFPS  40
_XP_048261507.1_    1  -VEIIEQPA-SKAL-RFRYEC------E---------GRSAGSIPGVNSTP---ENK---------TFPS  40
_XP_050573946.1_    1  -VEIIEQPA-SKAL-RFRYEC------E---------GRSAGSIPGVNSTP---ENK---------TFPS  40
_XP_050478075.1_    1  -VEIIEQPA-SKAL-RFRYEC------E---------GRSAGSIPGVNSTP---ENK---------TFPS  40
```

```
_XP_033204249.1_    1  -VEIIEQPA-SKAL-RFRYEC------E---------GRSAGSIPGVNSTP---ENK---------TFPS   40
_XP_033358451.1_    1  -VEIIEQPA-SKAL-RFRYEC------E---------GRSAGSIPGVNSTP---ENK---------TFPS   40
_XP_033358450.1_    1  -VEIIEQPA-SKAL-RFRYEC------E---------GRSAGSIPGVNSTP---ENK---------TFPS   40
_XP_033204244.1_    1  -VEIIEQPA-SKAL-RFRYEC------E---------GRSAGSIPGVNSTP---ENK---------TFPS   40
_XP_033312956.1_    1  -VEIIEQPA-SKAL-RFRYEC------E---------GRSAGSIPGVNSTP---ENK---------TFPS   40
_XP_012236639.1_    1  -VEIIEQPA-SKAL-RFRYEC------E---------GRSAGSIPGVNSTP---ENK---------TFPS   40
_XP_050573940.1_    1  -VEIIEQPA-SKAL-RFRYEC------E---------GRSAGSIPGVNSTP---ENK---------TFPS   40
_XP_012174118.1_    1  -VEIIEQPA-SKAL-RFRYEC------E---------GRSAGSIPGVNSTP---ENK---------TFPS   40
_XP_043581010.1_    1  -VEIIEQPA-SKAL-RFRYEC------E---------GRSAGSIPGINSTP---ENK---------TFPS   40
_EZA51108.1_        1  -VEILEQPA-SKAL-RFRYEC------E---------GRSAGSIPGVNSTA---ENK---------SFPS   40
_XP_011344085.1_    1  -VEILEQPA-SKAL-RFRYEC------E---------GRSAGSIPGVNSTA---ENK---------SFPS   40
_XP_043519475.1_    1  -VEIIEQPA-SKAL-RFRYEC------E---------GRSAGSIPGINSTP---ENK---------TFPS   40
_CAD1481114.1_      1  -VEIIEQPA-SKAL-RFRYEC------E---------GRSAGSIPGINSTP---ENK---------TFPS   40
_XP_043519473.1_    1  -VEIIEQPA-SKAL-RFRYEC------E---------GRSAGSIPGINSTP---ENK---------TFPS   40
_KOX75360.1_        1  -VEIIEQPA-SKAL-RFRYEC------E---------GRSAGSIPGINSTP---ENK---------TFPS   40
_KAF3423741.1_      1  -VEIIEQPA-SKAL-RFRYEC------E---------GRSAGSIPGINSTP---ENK---------TFPS   40
_KOC69050.1_        1  -VEIVEQPA-SKAL-RFRYEC------E---------GRSAGSIPGVNSTP---ENK---------TFPS   40
_XP_053980095.1_    1  -IEIIEQPA-SKAL-RFRYEC------E---------GRSAGSIPGANSTP---ENK---------TFPS   40
_XP_054002893.1_    1  -IEIIEQPA-SKAL-RFRYEC------E---------GRSAGSIPGANSTP---ENK---------TFPS   40
_XP_053980091.1_    1  -IEIIEQPA-SKAL-RFRYEC------E---------GRSAGSIPGANSTP---ENK---------TFPS   40
_XP_028049144.1_    1  -VEILEQPA-SKAL-RFRYEC------E---------GRSAGSIPGVNSIP---ENK---------TFPS   40
_XP_012541458.1_    1  -VEILEQPA-SKAL-RFRYEC------E---------GRSAGSIPGVNSIP---ENK---------TFPS   40
_XP_003708412.1_    1  -IEIIEQPA-SKAL-RFRYEC------E---------GRSAGSIPGVNSTP---ENK---------TFPS   40
_XP_012152807.1_    1  -IEIIEQPA-SKAL-RFRYEC------E---------GRSAGSIPGVNSTP---ENK---------TFPS   40
_KAH0945556.1_      1  -VEILEQPA-SKAL-RFRYEC------E---------GRSAGSIPGINSTP---ENK---------TFPS   40
_XP_024871832.1_    1  -VEILEQPA-SKAL-RFRYEC------E---------GRSAGSIPGINSAP---ENK---------TFPS   40
_XP_011307641.1_    1  --EIVEQPA-GKAL-RFRYEC------E---------GRSAGSIPGVNSMP---ENK---------TFPT   39
_XP_015118260.1_    1  --EIVEQPA-GKAL-RFRYEC------E---------GRSAGSIPGVNSMP---ENK---------TFPT   39
_XP_011307640.1_    1  --EIVEQPA-GKAL-RFRYEC------E---------GRSAGSIPGVNSMP---ENK---------TFPT   39
_XP_015118252.1_    1  --EIVEQPA-GKAL-RFRYEC------E---------GRSAGSIPGVNSMP---ENK---------TFPT   39
_EFN68841.1_        1  -VEILEQPA-SKAL-RFRYEC------E---------GRSAGSIPGVNSTP---ENK---------TFPS   40
_XP_011647340.1_    1  -VEILEQPA-SKAL-RFRYEC------E---------GRSAGSIPGVNSTP---ENK---------TFPS   40
_XP_029166122.1_    1  -VEILEQPA-SKAL-RFRYEC------E---------GRSAGSIPGINSTP---ENK---------TFPS   40
_KMQ96453.1_        1  -VEILEQPA-SKAL-RFRYEC------E---------GRSAGSIPGINSTP---ENK---------TFPS   40
_XP_050464492.1_    1  -VEILEQPA-SKAL-RFRYEC------E---------GRSAGSIPGINSTP---ENK---------TFPS   40
_XP_029673315.1_    1  -VEILEQPA-SKAL-RFRYEC------E---------GRSAGSIPGINSAP---ENK---------TFPS   40
_XP_025265518.1_    1  -VEILEQPA-SKAL-RFRYEC------E---------GRSAGSIPGVNSTP---ENK---------TFPS   40
_XP_011255523.1_    1  -VEILEQPA-SKAL-RFRYEC------E---------GRSAGSIPGVNSTP---ENK---------TFPS   40
_XP_011698133.1_    1  -VEILEQPA-SKAL-RFRYEC------E---------GRSAGSIPGVNSTP---ENK---------TFPS   40
```

```
_XP_011698131.1_     1  -VEILEQPA-SKAL-RFRYEC------E---------GRSAGSIPGVNSTP---ENK---------TFPS   40
_XP_018357420.1_     1  -VEILEQPA-SKAL-RFRYEC------E---------GRSAGSIPGVNSTP---ENK---------TFPS   40
_KYM76140.1_         1  -VEILEQPA-SKAL-RFRYEC------E---------GRSAGSIPGVNSTP---ENK---------TFPS   40
_XP_011647339.1_     1  -VEILEQPA-SKAL-RFRYEC------E---------GRSAGSIPGVNSTP---ENK---------TFPS   40
_KAG5315434.1_       1  -VEILEQPA-SKAL-RFRYEC------E---------GRSAGSIPGVNSTP---ENK---------TFPS   40
_XP_011055463.1_     1  -VEILEQPA-SKAL-RFRYEC------E---------GRSAGSIPGVNSTP---ENK---------TFPS   40
_XP_018342550.1_     1  -VEILEQPA-SKAL-RFRYEC------E---------GRSAGSIPGVNSTP---ENK---------TFPS   40
_XP_018403565.1_     1  -VEILEQPA-SKAL-RFRYEC------E---------GRSAGSIPGVNSTP---ENK---------TFPS   40
_KAG5322912.1_       1  -VEILEQPA-SKAL-RFRYEC------E---------GRSAGSIPGVNSTL---ENK---------TFPS   40
_XP_018357417.1_     1  -VEILEQPA-SKAL-RFRYEC------E---------GRSAGSIPGVNSTP---ENK---------TFPS   40
_XP_018314560.1_     1  -VEILEQPA-SKAL-RFRYEC------E---------GRSAGSIPGVNSTP---ENK---------TFPS   40
_XP_018057217.1_     1  -VEILEQPA-SKAL-RFRYEC------E---------GRSAGSIPGVNSTP---ENK---------TFPS   40
_KAG5346558.1_       1  -VEILEQPA-SKAL-RFRYEC------E---------GRSAGSIPGVNSTL---ENK---------TFPS   40
_XP_047348408.1_     1  -VEIVEQPA-SKAL-RFRYEC------E---------GRSAGSIPGANSSP---ENK---------TFPT   40
_XP_047348412.1_     1  -VEIVEQPA-SKAL-RFRYEC------E---------GRSAGSIPGANSSP---ENK---------TFPT   40
_XP_046820611.1_     1  -VEIVEQPA-SKAL-RFRYEC------E---------GRSAGSIPGANSSP---ENK---------TFPT   40
_XP_050848254.1_     1  -VEIIEQPA-SKAL-RFRYEC------E---------GRSAGSIPGANSSP---ENK---------TFPT   40
_XP_046820612.1_     1  -VEIVEQPA-SKAL-RFRYEC------E---------GRSAGSIPGANSSP---ENK---------TFPT   40
_XP_050848252.1_     1  -VEIIEQPA-SKAL-RFRYEC------E---------GRSAGSIPGANSSP---ENK---------TFPT   40
_XP_043667564.1_     1  -VEIIEQPA-SKAL-RFRYEC------E---------GRSAGSIPGANSSP---ENK---------TFPT   40
_XP_035725337.1_     1  -VEIVEQPA-SKAL-RFRYEC------E---------GRSAGSIPGANSSP---ENK---------TFPT   40
_XP_043667562.1_     1  -VEIIEQPA-SKAL-RFRYEC------E---------GRSAGSIPGANSSP---ENK---------TFPT   40
_XP_046820605.1_     1  -VEIVEQPA-SKAL-RFRYEC------E---------GRSAGSIPGANSSP---ENK---------TFPT   40
_XP_050848249.1_     1  -VEIIEQPA-SKAL-RFRYEC------E---------GRSAGSIPGANSSP---ENK---------TFPT   40
_XP_043667560.1_     1  -VEIIEQPA-SKAL-RFRYEC------E---------GRSAGSIPGANSSP---ENK---------TFPT   40
_XP_035725333.1_     1  -VEIVEQPA-SKAL-RFRYEC------E---------GRSAGSIPGANSSP---ENK---------TFPT   40
_KAF7405659.1_       1  -VEIIEQPA-SKAL-RFRYEC------E---------GRSAGSIPGANSSP---ENK---------TFPT   40
_KAF7402294.1_       1  -VEIIEQPA-SKAL-RFRYEC------E---------GRSAGSIPGANSSP---ENK---------TFPT   40
_XP_015172825.1_     1  -VEIVEQPA-SKSL-RFRYEC------E---------GRSAGSIPGVNSTP---ENK---------TFPT   40
_XP_015172824.1_     1  -VEIVEQPA-SKSL-RFRYEC------E---------GRSAGSIPGVNSTP---ENK---------TFPT   40
_XP_015172823.1_     1  -VEIVEQPA-SKSL-RFRYEC------E---------GRSAGSIPGVNSTP---ENK---------TFPT   40
_XP_014609306.1_     1  -VEIVEQPA-SKSL-RFRYEC------E---------GRSAGSIPGINSTP---ENK---------TFPT   40
_XP_043491219.1_     1  -VEIVEQPA-SKSL-RFRYEC------E---------GRSAGSIPGINSTP---ENK---------TFPT   40
_XP_014609305.1_     1  -VEIVEQPA-SKSL-RFRYEC------E---------GRSAGSIPGINSTP---ENK---------TFPT   40
_XP_043491218.1_     1  -VEIVEQPA-SKSL-RFRYEC------E---------GRSAGSIPGINSTP---ENK---------TFPT   40
_KAI4498556.1_       1  -VEIVEQPA-SKSL-RFRYEC------E---------GRSAGSIPGVNSTP---ENK---------TFPT   40
_XP_015172818.1_     1  -VEIVEQPA-SKSL-RFRYEC------E---------GRSAGSIPGVNSTP---ENK---------TFPT   40
_XP_043491211.1_     1  -VEIVEQPA-SKSL-RFRYEC------E---------GRSAGSIPGINSTP---ENK---------TFPT   40
_KAI4495142.1_       1  -VEIVEQPA-SKSL-RFRYEC------E---------GRSAGSIPGINSTP---ENK---------TFPT   40
```

```
_XP_014609300.1_     1  -VEIVEQPA-SKSL-RFRYEC------E---------GRSAGSIPGINSTP---ENK---------TFPT   40
_ADK91833.1_         1  -VEIIEQPA-SKAL-RFRYEC------E---------GRSAGSIPGVNSTP---ENK---------TFPS   40
_KAG7213837.1_       1  -VEIVEQPA-SKAL-RFRYEC------E---------GRSAGSIPGVNSTP---ENK---------TFPS   40
_XP_014226628.1_     1  -VEIIEQPA-SKAL-RFRYKC------E---------GRSAGSIPGVHSTP---DVK---------TFPT   40
_XP_020287946.1_     1  -VEILEQPA-SKAL-RFRYEC------E---------GRSAGSIPGVNSTP---ENK---------TFPS   40
_XP_012232007.1_     1  -VEILEQPA-SKAL-RFRYEC------E---------GRSAGSIPGVNSTP---ENK---------TFPS   40
_XP_011874538.1_     1  -VEILEQPA-SKAL-RFRYEC------E---------GRSAGSIPGVNSTP---ENK---------TFPS   40
_XP_011874541.1_     1  -VEILEQPA-SKAL-RFRYEC------E---------GRSAGSIPGVNSTP---ENK---------TFPS   40
_XP_011173619.1_     1  -VEILEQPA-SKAL-RFRYEC------E---------GRSAGSIPGINSMP---ENK---------TFPS   40
_XP_034950793.1_     1  -VEILEQPA-NKGI-RFRYEC------E---------GRSTGCIPGENSEP---EKK---------TFPS   40
_XP_034950792.1_     1  -----KQPA-NKGI-RFRYEC------E---------GRSTGCIPGENSEP---EKK---------TFPS   36
_XP_012340880.2_     1  -VEVIEQP--TKTL-RFRYEC------E---------GRSAGNIPGANSTL---ENK---------TCPS   39
_XP_031771859.1_     1  -VEVIEQP--TKTL-RFRYEC------E---------GRSAGNIPGANSTL---ENK---------TCPS   39
_XP_006618953.1_     1  -VEIIEQP--TKTL-RFRYEC------E---------GRLAGNIPGVSSTL---ENK---------TCPS   39
_XP_031367904.1_     1  -VEIIEQP--TKTL-RFRYEC------E---------GRLAGNIPGVSSTL---ENK---------TCPS   39
_XP_043795948.1_     1  -VEIIEQP--TKTL-RFRYEC------E---------GRLAGNIPGVSSTL---ENK---------TCPS   39
_XP_395180.5_        1  -VEIIEQP--TKNL-RFRYEC------E---------GRSAGNIPGASSTI---ENK---------TCPS   39
_KAG6799500.1_       1  -VEIIEQP--TKNL-RFRYEC------E---------GRSAGNIPGASSTI---ENK---------TCPS   39
_XP_016768607.2_     1  -VEIIEQP--TKNL-RFRYEC------E---------GRSAGNIPGASSTI---ENK---------TCPS   39
_PBC31989.1_         1  -VEIIEQP--TKTL-RFRYEC------E---------GRLAGNIPGASSTL---ENK---------TCPS   39
_XP_016914450.1_     1  -VEIIEQP--TKTL-RFRYEC------E---------GRLAGNIPGASSTL---ENK---------TCPS   39
_KAG5344958.1_       1  -VEILEQPA-SKAL-RFRYEC------E---------GRSAGSIPGVNSTP---ENK---------TFPS   40
_KAJ3630188.1_       1  -IKIIEQP--SKSV-RFRYEC---------------EGRPGTLAGASSTP---EHK---------IPFA   38
_QHN75199.1_         1  -IKIIEQP--SKSV-RFRYEC---------------EGRPGTLAGASSTP---EHK---------IPFA   38
_XP_051171660.1_     1  -IEILEQPA--SGI-RFRYEI------E---------GRSAGSIPGENSRP---DMK---------TYPT   39
_XP_016909447.1_        ----------------------------------------------------------------------
_XP_031773030.1_        ----------------------------------------------------------------------
_KAH0956427.1_       1  --IILEQPT-SEAI-RFRYRR------EI-------QNRSIGNIPGARSTT---NNK---------TYPT   41
_XP_012283784.1_     1  -VEILEQPA-SKAL-RFRYEC------E---------GRSAGSIPGVNSTP---ENK---------TFPS   40
_XP_023224928.1_     1  -VVILEQPA-PRTL-RFRYEC------E---------GRLAGCITGINSTP---ENK---------TFPT   40
_XP_008556434.1_     1  -VEIVEQPA-SKAL-RFRYEC------E---------GRSAGSIPGVNSSP---ENK---------TFPS   40
_XP_008556433.1_     1  -VEIVEQPA-SKAL-RFRYEC------E---------GRSAGSIPGVNSSP---ENK---------TFPS   40
_XP_014299728.1_     1  -VEIVEQPA-SKAL-RFRYEC------E---------GRSAGSIPGVNSSP---ENK---------TFPS   40
_CAD6234449.1_       1  -VEIIEQPA-SKAL-RFRYEC------E---------GRSAGSIPGVNSIP---ENK---------TFPS   40
_KAG8037552.1_       1  -VEIVEQPA-SKAL-RFRYEC------E---------GRSAGSIPGVNSIP---ENK---------TFPS   40
_XP_044576138.1_     1  -VEIVEQPA-SKAL-RFRYEC------E---------GRSAGSIPGVNSIP---ENK---------TFPS   40
_XP_044576125.1_     1  -VEIVEQPA-SKAL-RFRYEC------E---------GRSAGSIPGVNSIP---ENK---------TFPS   40
_XP_026465448.1_     1  -VVITEQPA-SKAL-RFRYEC------E---------GRSAGSIPGENSTP---ENK---------TFPS   40
_XP_026465446.1_     1  -VVITEQPA-SKAL-RFRYEC------E---------GRSAGSIPGENSTP---ENK---------TFPS   40
```

```
_XP_031785072.1_     1  -VEIVEQPA-SKAL-RFRYEC------E---------GRSAGSIPGVSSTP---ENK---------TFPT   40
_XP_015607185.1_     1  -VEIVQQPA-SKAL-RFRYEC------E---------GRSAGSIPGVNSTP---ENK---------TFPS   40
_XP_011496611.1_     1  -VEIVEQPA-SKAL-RFRYEC------E---------GRSAGSIPGVNSTP---ENK---------TFPS   40
_XP_018903707.1_     1  -IQIVEQPA-SQEV-RFRYEC------E---------GRSAGSIPGVNSTP---ENK---------TYPT   40
_CAB3365562.1_       1  -VEIIEQPA-PKAL-RFRYEC------E---------GRSAGSIPGENTTN---ENK---------TFPT   40
_XP_011496447.1_        ----------------------------------------------------------------------
_KAG8230996.1_       1  -C-ILEQPA-SKAL-RFRYEC------E---------GRSAGSIPGVNSTP---ENK---------TFPA   39
_XP_040567422.1_     1  -LQIIEQPA-YNKL-RFRYEC------E---------GRSAGALVGENTKD---KKR---------TYPT   40
_XP_014250205.1_     1  -INIIEQPA-SKAL-RFRYEC------E---------GRSAGSIPGVNSTT---ENK---------TFPT   40
_XP_014250206.1_     1  -INIIEQPA-SKAL-RFRYEC------E---------GRSAGSIPGVNSTT---ENK---------TFPT   40
_XP_047119528.1_     1  -VRIVEQPA-PKAL-RFRYEC------E---------GRSAGSIPGVNSTS---ENK---------TFPT   40
_XP_047001845.1_     1  -VRIVEQPA-PKAL-RFRYEC------E---------GRSAGSIPGVNSTS---ENK---------TFPT   40
_XP_049939433.1_     1  -VRIVEQPA-PKAL-RFRYEC------E---------GRSAGSIPGVNSTS---ENK---------TFPT   40
_XP_049764197.1_     1  -VRIVEQPA-PKAL-RFRYEC------E---------GRSAGSIPGVNSTS---ENK---------TFPT   40
_XP_049833656.1_     1  -VRIVEQPA-PKAL-RFRYEC------E---------GRSAGSIPGVNSTS---ENK---------TFPT   40
_XP_049790026.1_     1  -VRIVEQPA-PKAL-RFRYEC------E---------GRSAGSIPGVNSTS---ENK---------TFPT   40
_XP_049833651.1_     1  -VRIVEQPA-PKAL-RFRYEC------E---------GRSAGSIPGVNSTS---ENK---------TFPT   40
_XP_047001842.1_     1  -VRIVEQPA-PKAL-RFRYEC------E---------GRSAGSIPGVNSTS---ENK---------TFPT   40
_XP_049790025.1_     1  -VRIVEQPA-PKAL-RFRYEC------E---------GRSAGSIPGVNSTS---ENK---------TFPT   40
_XP_049833654.1_     1  -VRIVEQPA-PKAL-RFRYEC------E---------GRSAGSIPGVNSTS---ENK---------TFPT   40
_XP_049833659.1_     1  -VRIVEQPA-PKAL-RFRYEC------E---------GRSAGSIPGVNSTS---ENK---------TFPT   40
_XP_021925503.1_     1  -VKITEQPA-SKAL-RFRYEC------E---------GRSAGSIPGVNSTP---ENK---------TFPT   40
_KDR23857.1_         1  -VKITEQPA-SKAL-RFRYEC------E---------GRSAGSIPGVNSTP---ENK---------TFPT   40
_ABU96700.1_         1  -IKIIEQPA-SKAL-RFRYEC------E---------GRSAGSIPGVNSTP---ENK---------TFPT   40
_ABU96699.1_         1  -IKIIEQPA-SKAL-RFRYEC------E---------GRSAGSIPGVNSTP---ENK---------TFPT   40
_XP_014275495.1_     1  -VRIVEQPA-SKAL-RFRYEC------E---------GRSAGSLPGVNSTP---EVK---------TFPT   40
_BBE08137.1_         1  -VRIVEQPA-SKAL-RFRYEC------E---------GRSAGSLPGVHSTP---EVK---------TFPT   40
_XP_014275494.1_     1  -VRIVEQPA-SKAL-RFRYEC------E---------GRSAGSLPGVNSTP---EVK---------TFPT   40
_XP_014275493.1_     1  -VRIVEQPA-SKAL-RFRYEC------E---------GRSAGSLPGVNSTP---EVK---------TFPT   40
_BBE08136.1_         1  -VRIVEQPA-SKAL-RFRYEC------E---------GRSAGSLPGVHSTP---EVK---------TFPT   40
_KAF6207519.1_       1  -VKILEQPA-SKAL-RFRYEC------E---------GRSAGSIPGVNSTP---ENK---------TFPT   40
_CAB0015508.1_       1  -VKILEQPA-SKAL-RFRYEC------E---------GRSAGSIPGVNSTP---ENK---------TFPT   40
_CAB0015504.1_       1  -VKILEQPA-SKAL-RFRYEC------E---------GRSAGSIPGVNSTP---ENK---------TFPT   40
_PNF18474.1_         1  -VKIIEQPA-SKAL-RFRYKC------E---------GRSAGSIPGVNSTP---ENK---------TFPT   40
_CEG62431.1_         1  -VKILEQPA-SKAL-RFRYEC------E---------GRSAGSIPGVNSTP---ENK---------TFPT   40
_PSN36690.1_         1  -VKILEQPA-SKAL-RFRYEC------E---------GRSAGSIPGVNSTP---ENK---------TFPT   40
_XP_046387412.1_     1  -INIIEQPA-SKAL-RFRYEC------E---------GRSAGSIPGVNSTP---ENK---------TFPA   40
_XP_046387414.1_     1  -INIIEQPA-SKAL-RFRYEC------E---------GRSAGSIPGVNSTP---ENK---------TFPA   40
_XP_031634874.1_     1  -VKIIEQPA-PKAL-RFRYEC------E---------GRSAGSIPGANSTP---DNK---------AYPG   40
```

```
_GFG29240.1_        1  -VKITEQPA-SKAL-RFRYEC------E---------GRSAGSIPGVNSTP---ENK---------TYPT   40
_BBG28464.1_        1  -VQIIEQPA-SKAL-RFRYEC------E---------GRSAGSIPGANSTP---ENK---------TFPT   40
_WCL52334.1_        1  -VQIIEQPA-SKAL-RFRYEC------E---------GRSAGSIPGANSTP---ENK---------TFPT   40
_GLH00135.1_        1  -VQIIEQPA-SKAL-RFRYEC------E---------GRSAGSIPGANSTP---ENK---------TFPT   40
_CAD7205239.1_      1  -VKILEQPA-SKAL-RFRYEC------E---------GRSAGSIPGVNSTP---ENK---------TFPS   40
_CAD7431383.1_      1  -VKILEQPA-SKAL-RFRYEC------E---------GRSAGSIPGVNSTP---ENK---------TFPS   40
_CAD7410836.1_      1  -VKILEQPA-SKAL-RFRYEC------E---------GRSAGSIPGVNSTP---ENK---------TFPS   40
_CAD7415345.1_      1  -VKILEQPA-SKAL-RFRYEC------E---------GRSAGSIPGVNSTP---ENK---------TFPS   40
_CAD7606635.1_      1  -VKILEQPA-SKAL-RFRYEC------E---------GRSAGSIPGVNSTP---ENK---------TFPS   40
_CAD7462957.1_      1  -VKILEQPA-SKAL-RFRYEC------E---------GRSAGSIPGVNSTP---ENK---------TFPS   40
_XP_012265400.2_    1  -VKILEQPA-SKAL-RFRYEC------E---------GRSAGSIPGVNSTP---ENK---------TFPS   40
_XP_046473587.1_    1  -VNIIEQPA-SKAL-RFRYEC------E---------GRSAGSIPGVNSTP---ENK---------TFPK   40
_XP_046611732.1_    1  -VNIIEQPA-SKAL-RFRYEC------E---------GRSAGSIPGVNSTP---ENK---------TFPK   40
_XP_015519750.2_    1  -VNIIEQPA-SKAL-RFRYEC------E---------GRSAGSIPGVNSTP---ENK---------TFPK   40
_XP_046611731.1_    1  -VNIIEQPA-SKAL-RFRYEC------E---------GRSAGSIPGVNSTP---ENK---------TFPK   40
_XP_046417903.1_    1  -VNIIEQPA-SKAL-RFRYEC------E---------GRSAGSIPGVNSTP---ENK---------TFPK   40
_GLG94364.1_        1  -VEITEQPA-AKAL-RFRYEC------E---------GRSAGSIPGASSTP---DNK---------TYPA   40
_GLG94363.1_        1  -VEITEQPA-AKAL-RFRYEC------E---------GRSAGSIPGASSTP---DNK---------TYPA   40
_BBG28463.1_        1  -VEITEQPA-AKAL-RFRYEC------E---------GRSAGSIPGASSTP---DNK---------TYPA   40
_WCL52335.1_        1  -VEITEQPA-AKAL-RFRYEC------E---------GRSAGSIPGASSTP---DNK---------TYPA   40
_GLG94365.1_        1  -VEITEQPA-AKAL-RFRYEC------E---------GRSAGSIPGASSTP---DNK---------TYPA   40
_XP_002431518.1_    1  -VKIIEQPA-SKAL-RFRYEC------E---------GRSAGSIPGVNSTP---ENK---------TYPG   40
_KAE8746525.1_      1  -IVILEQPA-SKAL-RFRYEC------E---------GRSAGSIPGVNSTP---ENK---------TYPT   40
_XP_026277770.1_    1  -IVILEQPA-SKAL-RFRYEC------E---------GRSAGSIPGVNSTP---ENK---------TYPT   40
_KAJ1519446.1_      1  -IVILEQPA-SKAL-RFRYEC------E---------GRSAGSIPGVNSTS---ENK---------TYPT   40
_ALC78349.1_        1  -VKITEQPA-PKAL-RFRYEC------E---------GRSAGSIPGVHSTP---ENK---------TFPS   40
_XP_046738772.1_    1  -VKIIEQPA-SKAL-RFRYEC------E---------GRSAGSIPGVNSTP---ENK---------TFPR   40
_XP_046417904.1_    1  -VNIIEQPA-SKAL-RFRYEC------E---------GRSAGSIPGVNSTP---ENK---------TFPK   40
_CRK94170.1_        1  -VAIIEQPA-PKAL-RFRYEC------E---------GRSAGSIPGANSTP---DNK---------TYPT   40
_CRK94167.1_        1  -VAIIEQPA-PKAL-RFRYEC------E---------GRSAGSIPGANSTP---DNK---------TYPT   40
_KAG5676160.1_      1  -VAILEQPA-SKAL-RFRYEC------E---------GRSAGSIPGANSTA---DNK---------TYPT   40
_XP_034245464.1_    1  -IVILEQPA-SKAM-RFRYEC------E---------GRSAGSIPGVNSTP---ENK---------TFPT   40
_XP_034245457.1_    1  -IVILEQPA-SKAM-RFRYEC------E---------GRSAGSIPGVNSTP---ENK---------TFPT   40
_KAG8287980.1_      1  -IKIVEQPA-SKAL-RFRYEC------E---------GRSAGSIPGVNSTP---ENK---------TYPT   40
_XP_044759045.1_    1  -VKITEQPA-PDRS-RFRYES------E---------GFS--TLVGISSTS---IRR---------TYPT   38
_XP_017305229.1_    1  -IEILEQPK--SKV-RFRYEC------E---------GRSAGSIMGANSTL---ECK---------SYPK   39
_XP_026675878.1_    1  -IEILEQPK--SKV-RFRYEC------E---------GRSAGSIMGANSTL---ECK---------SYPK   39
_KAE8574083.1_      1  -VRIVEQPA-SKAL-RFRYEC------E---------GRSAGSLPGVNSTP---EVK---------TFPT   40
_XP_037926497.1_    1  -VRIIEQPA-SKAL-RFRYEC------E---------GRSAGSIPGVNSTS---ENK---------TYPT   40
```

```
_CAD7079567.1_        1   -VRIIEQPA-SKAL-RFRYEC------E---------GRSAGSIPGVNSTS---ENK---------TYPT   40
_XP_037882485.1_      1   -VKIVEQPA-SKAL-RFRYEC------E---------GRSAGSIPGVNSTS---ENK---------TYPT   40
_XP_037037597.1_      1   -VKITEQPA-SKAL-RFRYEC------E---------GRSAGSIPGVLSSP---ENK---------TFPS   40
_XP_037037592.1_      1   -VKITEQPA-SKAL-RFRYEC------E---------GRSAGSIPGVLSSP---ENK---------TFPS   40
_KAG4075389.1_        1   -VKITEQPA-SKAL-RFRYEC------E---------GRSAGSIPGVLSSP---ENK---------TFPS   40
_XP_034101357.1_      1   -VKITEQPA-GKAL-RFRYEC------E---------GRSAGSIPGVNSTA---ENK---------TYPT   40
_XP_026844963.1_      1   -VKITEQPA-GKAL-RFRYEC------E---------GRSAGSIPGVNSTP---ENK---------TYPT   40
_EDW28798.1_          1   -VKITEQPA-GKAL-RFRYEC------E---------GRSAGSIPGVNSTP---ENK---------TYPT   40
_XP_034125966.1_      1   -VKITEQPA-GKAL-RFRYEC------E---------GRSAGSIPGVNSTP---ENK---------TYPT   40
_XP_034666519.1_      1   -VKITEQPA-GKAL-RFRYEC------E---------GRSAGSIPGVNSTP---ENK---------TYPT   40
_XP_001356570.3_      1   -VKITEQPA-GKAL-RFRYEC------E---------GRSAGSIPGVNSTP---ENK---------TYPT   40
_XP_023164577.2_      1   -VKITEQPA-GKAL-RFRYEC------E---------GRSAGSIPGVNSTP---ENK---------TYPT   40
_XP_017153206.1_      1   -VKITEQPA-GKAL-RFRYEC------E---------GRSAGSIPGVNSTP---ENK---------TYPT   40
_XP_030564932.1_      1   -VKITEQPA-GKAL-RFRYEC------E---------GRSAGSIPGVNSTP---ENK---------TYPT   40
_XP_002059145.1_      1   -VKITEQPA-GKAL-RFRYEC------E---------GRSAGSIPGVNSTP---ENK---------TYPT   40
_EDW04266.1_          1   -VKITEQPA-GKAL-RFRYEC------E---------GRSAGSIPGVNSTP---ENK---------TYPT   40
_XP_017025862.1_      1   -VKITEQPA-GKAL-RFRYEC------E---------GRSAGSIPGVNSTP---ENK---------TYPT   40
_XP_017859907.1_      1   -VKITEQPA-GKAL-RFRYEC------E---------GRSAGSIPGVNSTP---ENK---------TYPT   40
_XP_032584327.1_      1   -VKITEQPA-GKAL-RFRYEC------E---------GRSAGSIPGVNSTP---ENK---------TYPT   40
_XP_016987288.2_      1   -VKITEQPA-GKAL-RFRYEC------E---------GRSAGSIPGVNSTQ---DNK---------TYPT   40
_XP_017954173.1_      1   -VKITEQPA-GKAL-RFRYEC------E---------GRSAGSIPGVNSTP---ENK---------TYPT   40
_XP_017854504.1_      1   -VKITEQPA-GKAL-RFRYEC------E---------GRSAGSIPGVNSTP---ENK---------TYPT   40
_XP_034125965.1_      1   -VKITEQPA-GKAL-RFRYEC------E---------GRSAGSIPGVNSTP---ENK---------TYPT   40
_XP_034666515.1_      1   -VKITEQPA-GKAL-RFRYEC------E---------GRSAGSIPGVNSTP---ENK---------TYPT   40
_XP_043641917.1_      1   -VKITEQPA-GKAL-RFRYEC------E---------GRSAGSIPGVNSTP---ENK---------TYPT   40
_XP_026844960.1_      1   -VKITEQPA-GKAL-RFRYEC------E---------GRSAGSIPGVNSTP---ENK---------TYPT   40
_XP_015035988.2_      1   -VKITEQPA-GKAL-RFRYEC------E---------GRSAGSIPGVNSTP---ENK---------TYPT   40
_XP_041450744.1_      1   -VKITEQPA-GKAL-RFRYEC------E---------GRSAGSIPGVNSTP---ENK---------TYPT   40
_XP_015052361.1_      1   -VKITEQPA-GKAL-RFRYEC------E---------GRSAGSIPGVNSTP---ENK---------TYPT   40
_XP_039480515.1_      1   -VKITEQPA-GKAL-RFRYEC------E---------GRSAGSIPGVNSTP---ENK---------TYPT   40
_XP_033248563.1_      1   -VKITEQPA-GKAL-RFRYEC------E---------GRSAGSIPGVNSTP---ENK---------TYPT   40
_KAH8412150.1_        1   -VKITEQPA-GKAL-RFRYEC------E---------GRSAGSIPGVNSTP---ENK---------TYPT   40
_XP_043864974.1_      1   -VKITEQPA-GKAL-RFRYEC------E---------GRSAGSIPGVNSTP---ENK---------TYPT   40
_XP_020809119.1_      1   -VKITEQPA-GKAL-RFRYEC------E---------GRSAGSIPGVNSTP---ENK---------TYPT   40
_KAH8298353.1_        1   -VKITEQPA-GKAL-RFRYEC------E---------GRSAGSIPGVNSTP---ENK---------TYPT   40
_XP_030564927.1_      1   -VKITEQPA-GKAL-RFRYEC------E---------GRSAGSIPGVNSTP---ENK---------TYPT   40
_XP_041631592.1_      1   -VKITEQPA-GKAL-RFRYEC------E---------GRSAGSIPGVNSTP---ENK---------TYPT   40
_XP_017859909.1_      1   -VKITEQPA-GKAL-RFRYEC------E---------GRSAGSIPGVNSTP---ENK---------TYPT   40
_XP_032591602.1_      1   -VKITEQPA-GKAL-RFRYEC------E---------GRSAGSIPGVNSTP---ENK---------TYPT   40
```

```
_XP_023164572.2_     1  -VKITEQPA-GKAL-RFRYEC------E---------GRSAGSIPGVNSTP---ENK---------TYPT   40
_TDG46699.1_         1  -VKITEQPA-GKAL-RFRYEC------E---------GRSAGSIPGVNSTP---ENK---------TYPT   40
_KAH8411895.1_       1  -VKITEQPA-GKAL-RFRYEC------E---------GRSAGSIPGVNSTP---ENK---------TYPT   40
_KAH8377496.1_       1  -VKITEQPA-GKAL-RFRYEC------E---------GRSAGSIPGVNSTA---ENK---------TYPT   40
_XP_017854500.2_     1  -VKITEQPA-GKAL-RFRYEC------E---------GRSAGSIPGVNSTP---ENK---------TYPT   40
_XP_016987284.2_     1  -VKITEQPA-GKAL-RFRYEC------E---------GRSAGSIPGVNSTQ---DNK---------TYPT   40
_KAH8293209.1_       1  -VKITEQPA-GKAL-RFRYEC------E---------GRSAGSIPGVNSTA---ENK---------TYPT   40
_EEC00225.1_         1  -VVITEQPA-SRAL-RFRYQC------E---------GRYPGTLVGVNSTA---ENK---------TYPT   40
_XP_029825505.2_     1  -VVITEQPA-SRAL-RFRYQC------E---------GRYPGTLVGVNSTA---ENK---------TYPT   40
_KAG0424790.1_       1  -VVITEQPA-SRAL-RFRYQC------E---------GRYPGTLVGVNSTA---ENK---------TYPT   40
_XP_037272127.1_     1  -VVILEQPA-SRAL-RFRYQC------E---------GRYPGTLVGVSSTA---ENK---------TYPT   40
_XP_054934017.1_     1  -VVILEQPA-SRAL-RFRYQC------E---------GRYPGTLVGVSSTA---ENK---------TYPT   40
_XP_050048524.1_     1  -VVILEQPA-SRAL-RFRYQC------E---------GRYPGTLVGVSSTA---ENK---------TYPT   40
_XP_037272126.1_     1  -VVILEQPA-SRAL-RFRYQC------E---------GRYPGTLVGVSSTA---ENK---------TYPT   40
_XP_037524244.1_     1  -VVILEQPA-SRAL-RFRYQC------E---------GRYPGTLVGVSSTA---ENK---------TYPT   40
_XP_037524245.1_     1  -VVILEQPA-SRAL-RFRYQC------E---------GRYPGTLVGVSSTA---ENK---------TYPT   40
_XP_049512094.1_     1  -VVILEQPA-SRAL-RFRYQC------E---------GRYPGTLVGVNSTA---ENK---------TYPT   40
_XP_037524243.1_     1  -VVILEQPA-SRAL-RFRYQC------E---------GRYPGTLVGVSSTA---ENK---------TYPT   40
_XP_037580160.1_     1  -VVILEQPA-SRAL-RFRYQC------E---------GRYPGTLVGVNSTA---ENK---------TYPT   40
_XP_037272128.1_     1  -VVILEQPA-SRAL-RFRYQC------E---------GRYPGTLVGVSSTA---ENK---------TYPT   40
_KAH9380259.1_       1  -VVILEQPA-SRAL-RFRYQC------E---------GRYPGTLVGVSSTA---ENK---------TYPT   40
_KAI1291715.1_       1  ---------------------------------------------DQMSTS---SIK---------TFPT   13
_XP_035209023.1_     1  -VEILIQPAFSNYF-RYTCET------E---------NKCRGRIVGVNSTA---QMK---------QYPT   41
_XP_023332679.1_     1  -MEIVEQPK-SNSL-RFRYEC------E---------GKGAGALQGKFSTQ---ETK---------TFPK   40
_XP_046801942.1_     1  -VKITEQPA-GKAL-RFRYEC------E---------GRSAGSIPGVNSTP---ENK---------TYPT   40
_XP_037811120.1_     1  -VKITEQPA-GKAL-RFRYEC------E---------GRSAGSIPGVNSTP---ENK---------TYPT   40
_AFI98401.1_         1  -VKITEQPA-GKAL-RFRYEC------G---------GRSAGSIPGVNSTP---ENK---------TYPT   40
_AFJ54621.1_         1  -VKITEQPA-GKAL-RFRYEC------G---------GRSAGSIPGVNSTP---ENK---------TYPT   40
_KAI8126534.1_       1  -VKITEQPA-GKAL-RFRYEC------E---------GRSAGSIPGVNSTP---ENK---------TYPT   40
_XP_013118266.1_     1  -VKITEQPA-GKAL-RFRYEC------E---------GRSAGSIPGVNSTP---ENK---------TYPT   40
_XP_023293634.2_     1  -VKITEQPA-GKAL-RFRYEC------E---------GRSAGSIPGVNSTP---ENK---------TYPT   40
_XP_037811062.1_     1  -VKITEQPA-GKAL-RFRYEC------E---------GRSAGSIPGVNSTP---ENK---------TYPT   40
_KNC34132.1_         1  -VKITEQPA-GKAL-RFRYEC------E---------GRSAGSIPGVNSTP---ENK---------TYPT   40
_TMW48951.1_         1  -VKITEQPA-SKAL-RFRYEC------E---------GRSAGSIPGVNSTP---ECK---------TYPT   40
_XP_037957363.1_     1  -VKITEQPA-GKAL-RFRYEC------E---------GRSAGSIPGISSTP---ENK---------TFPT   40
_XP_005175014.1_     1  -VKITEQPA-GKAL-RFRYEC------E---------GRSAGSIPGVNSTP---ENK---------TYPT   40
_XP_037957360.1_     1  -VKITEQPA-GKAL-RFRYEC------E---------GRSAGSIPGISSTP---ENK---------TFPT   40
_XP_030383330.1_     1  -VKITEQPA-GKAL-RFRYEC------E---------GRSAGSIPGVNSTP---ENK---------TYPT   40
_XP_030383328.1_     1  -VKITEQPA-GKAL-RFRYEC------E---------GRSAGSIPGVNSTP---ENK---------TYPT   40
```

```
_CAH1707987.1_       1  --SILEQPA-SKAL-RFRYEC------E---------GRSAGSIPGANSTS---DNK---------TYPT   39
_TMW54796.1_         1  -VKITEQPA-SKAL-RFRYEC------E---------GRSAGSILGANSTP---ECK---------TYPT   40
_XP_026836077.1_     1  -VKITEQPA-GKAL-RFRYEC------E---------GRSAGSIPGVNSTP---ENK---------TYPT   40
_XP_017116803.1_     1  -VKITEQPA-GKAL-RFRYEC------E---------GRSAGSIPGVNSTQ---ENK---------TYPT   40
_XP_039480519.1_     1  -VKITEQPA-GKAL-RFRYEC------E---------GRSAGSIPGVNSTP---ENK---------TYPT   40
_XP_043641920.1_     1  -VKITEQPA-GKAL-RFRYEC------E---------GRSAGSIPGVNSTP---ENK---------TYPT   40
_XP_023036748.1_     1  -VKITEQPA-GKAL-RFRYEC------E---------GRSAGSIPGVNSTP---ENK---------TYPT   40
_XP_002090466.1_     1  -VKITEQPA-GKAL-RFRYEC------E---------GRSAGSIPGVNSTP---ENK---------TYPT   40
_XP_034471824.1_     1  -VKITEQPA-GKAL-RFRYEC------E---------GRSAGSIPGVNSTA---ENK---------TYPT   40
_AAT94434.1_         1  -VKITEQPA-GKAL-RFRYEC------E---------GRSAGSIPGVNSTP---ENK---------TYPT   40
_XP_036670985.1_     1  -VKITEQPA-GKAL-RFRYEC------E---------GRSAGSIPGVNSTP---ENK---------TYPT   40
_AAA28479.1_         1  -VKITEQPA-GKAL-RFRYEC------E---------GRSAGSIPGVNSTP---ENK---------TYPT   40
_XP_037708206.1_     1  -VKITEQPA-GKAL-RFRYEC------E---------GRSAGSIPGVNSTP---ENK---------TYPT   40
_XP_002038915.1_     1  -VKITEQPA-GKAL-RFRYEC------E---------GRSAGSIPGVNSTP---ENK---------TYPT   40
_XP_016993636.1_     1  -VKITEQPA-GKAL-RFRYEC------E---------GRSAGSIPGVNSTP---ENK---------TYPT   40
_XP_052852517.1_     1  -VKITEQPA-GKAL-RFRYEC------E---------GRSAGSIPGVNSTQ---ENK---------TYPT   40
_XP_016963139.1_     1  -VKITEQPA-GKAL-RFRYEC------E---------GRSAGSIPGVNSTP---ENK---------TYPT   40
_XP_033156151.1_     1  -VKITEQPA-GKAL-RFRYEC------E---------GRSAGSIPGVNSTP---ENK---------TYPT   40
_XP_001974446.1_     1  -VKITEQPA-GKAL-RFRYEC------E---------GRSAGSIPGVNSTP---ENK---------TYPT   40
_XP_014761886.1_     1  -VKITEQPA-GKAL-RFRYEC------E---------GRSAGSIPGVNSTP---ENK---------TYPT   40
_XP_017107216.2_     1  -VKITEQPA-GKAL-RFRYEC------E---------GRSAGSIPGVNSTP---ENK---------TYPT   40
_XP_017044881.1_     1  -VKITEQPA-GKAL-RFRYEC------E---------GRSAGSIPGVNSTS---ENK---------TYPT   40
_XP_016944798.1_     1  -VKITEQPA-GKAL-RFRYEC------E---------GRSAGSIPGVNSTP---ENK---------TYPT   40
_XP_017116800.1_     1  -VKITEQPA-GKAL-RFRYEC------E---------GRSAGSIPGVNSTQ---ENK---------TYPT   40
_XP_016993633.2_     1  -VKITEQPA-GKAL-RFRYEC------E---------GRSAGSIPGVNSTP---ENK---------TYPT   40
_XP_037708198.1_     1  -VKITEQPA-GKAL-RFRYEC------E---------GRSAGSIPGVNSTP---ENK---------TYPT   40
_XP_032583259.1_     1  -VKITEQPA-GKAL-RFRYEC------E---------GRSAGSIPGVNSTP---ENK---------TYPT   40
_AAC35296.1_         1  -VKITEQPA-GKAL-RFRYEC------E---------GRSAGSIPGVNSTP---ENK---------TYPT   40
_NP_001286014.1_     1  -VKITEQPA-GKAL-RFRYEC------E---------GRSAGSIPGVNSTP---ENK---------TYPT   40
_XP_016963136.1_     1  -VKITEQPA-GKAL-RFRYEC------E---------GRSAGSIPGVNSTP---ENK---------TYPT   40
_XP_017063904.2_     1  -VKITEQPA-GKAL-RFRYEC------E---------GRSAGSIPGVNSTP---ESK---------TYPT   40
_XP_033156135.1_     1  -VKITEQPA-GKAL-RFRYEC------E---------GRSAGSIPGVNSTP---ENK---------TYPT   40
_XP_052852512.1_     1  -VKITEQPA-GKAL-RFRYEC------E---------GRSAGSIPGVNSTQ---ENK---------TYPT   40
_XP_032307758.1_     1  -VKITEQPA-GKAL-RFRYEC------E---------GRSAGSIPGVNSTP---ENK---------TYPT   40
_XP_017107215.2_     1  -VKITEQPA-GKAL-RFRYEC------E---------GRSAGSIPGVNSTP---ENK---------TYPT   40
_AWT86616.1_         1  --VITEQPA-SKAL-RFRYEC------E---------GRSAGSIPGISSTP---ENK---------TFPT   39
_QUF98534.1_         1  --VITEQPA-SKAL-RFRYEC------E---------GRSAGSIPGVSSTP---ENK---------TYPT   39
_UNA06287.1_         1  --VITEQPA-SKAL-RFRYEC------E---------GRSAGSIPGISSTP---ENK---------TFPT   39
_AAQ65068.1_         1  -VKITEQPX-GKAL-RFRYEC------E---------GRSAGSIPGVNSTP---ENK---------TYPT   40
```

```
_AAQ64916.1_        1  -VKITEQPA-GKAL-RFRYEC------E---------GRSAGSIPGVNSTP---ENK---------TYPT   40
_AAQ64921.1_        1  -VKITEQPA-GKAL-RFRYEC------E---------GRSAGSIPGVNSTP---ENK---------TYPT   40
_AAQ64918.1_        1  -VKITEQPA-GKAL-RFRYEC------X---------GRSAGSIPGVNSTP---ENK---------TYPT   40
_KAH8245459.1_      1  -VKITEQPA-GKAL-RFRYEC------E---------GRSAGSIPGVNSTP---ENK---------TYPT   40
_KAH8283155.1_      1  -VKITEQPA-GKAL-RFRYEC------E---------GRSAGSIPGVNSTP---ENK---------TYPT   40
_KAH8246384.1_      1  -VKITEQPA-GKAL-RFRYEC------E---------GRSAGSIPGVNSTP---ENK---------TYPT   40
_KAH8366418.1_      1  -VKITEQPA-GKAL-RFRYEC------E---------GRSAGSIPGVNSTP---ENK---------TYPT   40
_XP_022195378.2_    1  --VITEQPA-SKAL-RFRYEC------E---------GRSAGSIPGVNSTP---ENK---------THPT   39
_XP_039284931.1_    1  --VITEQPA-SKAL-RFRYEC------E---------GRSAGSIPGVNSTP---ENK---------THPT   39
_ALC40091.1_        1  -VKITEQPA-GKAL-RFRYEC------E---------GRSAGSIPGVNSTP---ENK---------TYPT   40
_XP_036342419.1_    1  -VRVVEQPA-SKAL-RFRYEC------E---------GRSAGSIPGAHSTP---EKK---------TYPT   40
_XP_022903025.1_    1  --VIVEEPA-QKQL-RFRYEC------E---------GRSAGSIPGANSTP---DNK---------TYPT   39
_XP_022903017.1_    1  --VIVEEPA-QKQL-RFRYEC------E---------GRSAGSIPGANSTP---DNK---------TYPT   39
_XP_036218946.1_    1  -VKIVEQPA-SKAV-RFRYEC------E---------GRSAGSIPGENSTL---EMR---------TYPT   40
_XP_018790401.1_    1  -VKIVEQPA-SKAV-RFRYEC------E---------GRSAGSIPGVNSTS---ALR---------TYPT   40
_KAI9579887.1_      1  -VEIVEQP--NKAL-RFRYEC------E---------GRYASCIPGISSTP---GNK---------THPE   39
_XP_037892537.1_    1  -VEIVEQP--NKAL-RFRYEC------E---------GRYASCIPGISSTP---GNK---------THPE   39
_XP_018903705.1_    1  -IEILEQPDNQ----RYRYVS------E---------GRTAGSIRGVSSGS---GAKCKQSRDTSVTYPT   47
_CAH0395667.1_      1  -IEILEQPDNQ----RYRYVS------E---------GRTAGSIRGVSSGS---GAKCKQSRDTSVTYPT   47
_XP_050330165.1_    1  -IKIVEQPA-SKAV-RFRYEC------E---------GRSAGSIPGVNSTP---ALR---------TYPT   40
_XP_039970878.1_    1  -IKIVEQPA-SKAV-RFRYEC------E---------GRTAGSIPGVNSTP---ALR---------TYPT   40
_XP_049305234.1_    1  -VKIVEQPA-SKAV-RFRYEC------E---------GRSAGSIPGVSSTS---ALR---------TYPT   40
_GLH04793.1_        1  -VRIVEQPA-PRAL-RFRYAC------E---------GRSAGSLPGVHSTP---GAR---------TVPE   40
_XP_015835035.1_    1  -IRIIEQPQ--KSL-RFRYQC----------------EKRTGNIPGASSTS---TLK---------TVCA   38
_XP_015835036.1_    1  -IRIIEQPQ--KSL-RFRYQC----------------EKRTGNIPGASSTS---TLK---------TVCA   38
_XP_039283832.1_    1  --VITEEPA-SKEL-RFRYDS------ERRPVSVNRPYSSAGSIPGVRSTP---QNK---------TYPS   48
_XP_031788427.1_    1  -VRIIEQPA--KAI-RFRYER------E---------GRAAGKLDGEMTTD---HQK---------TYPT   39
_KAJ6646894.1_      1  -VEILEQPA-ANGV-RFRYKN----------------EKQSSVIYGASSTH---DKK---------TFPS   39
_XP_026466084.1_       ----------------------------------------------------------------------
_XP_044002069.1_    1  -IEVIEQPV--RTL-TFKYQC----------------EGP----MKSNTHK---LEK---------KFIS   34
_XP_046611735.1_    1  -IKIIEQPA-SKAV-QFRYRN------E---------DRGISIIPGVNSTR---AKK---------TYPS   40
_XP_046417906.1_    1  -IKIIEQPA-SKAV-QFRYRN------E---------DRGISIIPGVNSTR---AKK---------TYPS   40
_XP_046590309.1_    1  -IKIIEQPA-SKAV-QFRYRN------E---------DRGISIIPGVNSTR---AKK---------TYPS   40
_XP_046473589.1_    1  -IKIIEQPA-SKAV-QFRYRN------E---------DRGISIIPGVNSTR---AKK---------TYPS   40
_XP_019892116.1_       ----------------------------------------------------------------------
_XP_005183027.3_       ----------------------------------------------------------------------
_XP_011496598.1_       ----------------------------------------------------------------------
_AAW67216.1_           ----------------------------------------------------------------------
_AAW67217.1_           ----------------------------------------------------------------------
```

```
_XP_017489788.1_        ----------------------------------------------------------------------
_BAI67894.1_            ----------------------------------------------------------------------
_XP_054741511.1_     1  -VKIVEQPA-SKAL-RFRYEC------E---------GRSAGSIPGVNSTP---ELK---------TFPT   40
_XP_053961684.1_     1  -VKIVEQPA-SKAL-RFRYEC------E---------GRSAGSIPGVNSTP---ELK---------TFPT   40
_XP_036341313.1_     1  -VKIVEQPA-SKAL-RFRYEC------E---------GRSAGSIPGVNSTP---EMK---------TFPT   40
_XP_036341343.1_     1  -VKIVEQPA-SKAL-RFRYEC------E---------GRSAGSIPGVNSTP---EMK---------TFPT   40
_XP_017471383.1_     1  -VKIVEQPA-SKAL-RFRYEC------E---------GRSAGSIPGVNSTP---EMK---------TFPT   40
_XP_054741498.1_     1  -VKIVEQPA-SKAL-RFRYEC------E---------GRSAGSIPGVNSTP---ELK---------TFPT   40
_XP_053961677.1_     1  -VKIVEQPA-SKAL-RFRYEC------E---------GRSAGSIPGVNSTP---ELK---------TFPT   40
_XP_050320008.1_     1  -VKIVEQPA-SKAL-RFRYEC------E---------GRSAGSIPGVNSTP---EMK---------TFPT   40
_XP_028894490.2_     1  -VKIVEQPA-SKAL-RFRYEC------E---------GRSAGSIPGVNSTP---EMK---------TFPT   40
_XP_050320015.1_     1  -VKIVEQPA-SKAL-RFRYEC------E---------GRSAGSIPGVNSTP---EMK---------TFPT   40
_XP_018787554.1_     1  -VKIVEQPA-SKAL-RFRYEC------E---------GRSAGSIPGVNSTP---EMK---------TFPT   40
_XP_029407438.2_     1  -VKIVEQPA-SKAL-RFRYEC------E---------GRSAGSIPGVNSTP---EMK---------TFPT   40
_XP_036225551.1_     1  -VKIVEQPA-SKAL-RFRYEC------E---------GRSAGSIPGVNSTP---EMK---------TFPT   40
_XP_011178374.2_     1  -VKIVEQPA-SKAL-RFRYEC------E---------GRSAGSIPGVNSTP---EMK---------TFPT   40
_XP_039952593.1_     1  -VKIVEQPA-SKAL-RFRYEC------E---------GRSAGSIPGVNSTP---EMK---------TFPT   40
_XP_018787542.1_     1  -VKIVEQPA-SKAL-RFRYEC------E---------GRSAGSIPGVNSTP---EMK---------TFPT   40
_XP_011207866.2_     1  -VKIVEQPA-SKAL-RFRYEC------E---------GRSAGSIPGVNSTP---EMK---------TFPT   40
_XP_014086506.1_     1  -VKIVEQPA-SKAL-RFRYEC------E---------GRSAGSIPGVNSTP---EMK---------TFPT   40
_XP_004526638.1_     1  -VKIVEQPA-SKAL-RFRYEC------E---------GRSAGSIPGVNSTP---EMK---------TFPT   40
_XP_017471402.1_     1  -VKIVEQPA-SKAL-RFRYEC------E---------GRSAGSIPGVNSTP---EMK---------TFPT   40
_XP_012157903.1_     1  -VKIVEQPA-SKAL-RFRYEC------E---------GRSAGSIPGVNSTP---EMK---------TFPT   40
_XP_011185661.2_     1  -VKIVEQPA-SKAV-RFRYEC------E---------GRSAGSIPGVNSTL---ETR---------TYPT   40
_XP_050322757.1_     1  -VKIVEQPA-SKAV-RFRYEC------E---------GRTAGSIPGVNSTP---ALR---------TYPT   40
_XP_039962922.1_     1  -VKIVEQPA-SKAV-RFRYEC------E---------GRTAGSIPGVNSTP---ALR---------TYPT   40
_XP_050322739.1_     1  -VKIVEQPA-SKAV-RFRYEC------E---------GRTAGSIPGVNSTP---ALR---------TYPT   40
_XP_050322749.1_     1  -VKIVEQPA-SKAV-RFRYEC------E---------GRTAGSIPGVNSTP---ALR---------TYPT   40
_XP_050322724.1_     1  -VKIVEQPA-SKAV-RFRYEC------E---------GRTAGSIPGVNSTP---ALR---------TYPT   40
_XP_050322682.1_     1  -VKIVEQPA-SKAV-RFRYEC------E---------GRTAGSIPGVNSTP---ALR---------TYPT   40
_CAG9823112.1_       1  -VKIVEQPA-SKAL-RFRYEC------E---------GRSAGSIPGARSTP---ETK---------TYPT   40
_KAG5873705.1_       1  -IKIIEQPA-PKAL-RFRYEC------E---------GRSAGSIPGTNSTP---ENK---------TFPA   40
_KAH8030364.1_       1  -VVILEQPA-SRAL-RFRYQC------E---------GRYPGTLVG------------------------   28
_XP_023013891.1_     1  -IKIVEQPA-PKAL-RFRYEC------E---------GRSAGSIPGTNSTP---ENK---------TYPT   40
_XP_028130780.2_     1  -IRIVEQPA-SKAL-RFRYEC------E---------GRSAGSIPGSRSTP---ENK---------SYPT   40
_KAG5873704.1_       1  -IKIIEQPA-PKAL-RFRYEC------E---------GRSAGSIPGTNSTP---ENK---------TFPA   40
_CAH1187690.1_       1  -VTIVEQPA-SKAL-RFRYEC------E---------GRSAGSIPGARSTP---ENK---------TYPT   40
_CAH1098641.1_       1  -IRIVEQPA-SKAL-RFRYEC------E---------GRSAGSIPGSRSTP---ENK---------SYPS   40
_XP_023028116.1_     1  -IRILEQPA-PKIL-RFRYLC------E---------SRSAGSFPGKNSTP---DNP---------TYPT   40
```

```
_XP_031630227.1_      1  -VRIKEEPA-EKSF-RFRYES------E---------GRWSGSIHGENSTH---DKT---------TYPS   40
_KAG5861047.1_           ----------------------------------------------------------------------
_KAF2880990.1_        1  -VKITEQPA-SKAL-RFRYEC------E---------GRSAGSIPGVSSTP---ENK---------TFPA   40
_XP_018326789.1_      1  -VKIIEQPA-PKAL-RFRYEC------E---------GRCAGSIPGVSSTP---ENK---------TYPA   40
_XP_018326788.1_      1  -VKIIEQPA-PKAL-RFRYEC------E---------GRCAGSIPGVSSTP---ENK---------TYPA   40
_XP_031339184.1_      1  -VRIIEQPA-SKAL-RFRYEC------E---------GRSAGSIPGVSSTP---ENK---------TFPS   40
_XP_045476069.1_      1  -VKIIEQPA-PKAL-RFRYLC------E---------GRSAGSIPGISSTP---ENK---------TFPA   40
_XP_044734149.1_      1  -VEIVEQPA-SKAM-RFRYEC------E---------GRSAGSIPGQNSTS---ENK---------TFPT   40
_XP_044734150.1_      1  -VEIVEQPA-SKAM-RFRYEC------E---------GRSAGSIPGQNSTS---ENK---------TFPT   40
_XP_044734148.1_      1  -VEIVEQPA-SKAM-RFRYEC------E---------GRSAGSIPGQNSTS---ENK---------TFPT   40
_XP_044760528.1_      1  -VKIIEQPA-PKAL-RFRYLC------E---------GRSAGSIPGISSTP---ENK---------TFPA   40
_XP_044747918.1_      1  -VEIVEQPA-PKAL-RFRYEC------E---------GRSAGSIPGISSTA---ENK---------TYPT   40
_XP_044747917.1_      1  -VEIVEQPA-PKAL-RFRYEC------E---------GRSAGSIPGISSTA---ENK---------TYPT   40
_XP_045481017.1_      1  -VDIIEQPA-SKAL-RFRYEC------E---------GRSAGSIPGKSSTS---ENK---------TYPT   40
_XP_045481018.1_      1  -VDIIEQPA-SKAL-RFRYEC------E---------GRSAGSIPGKSSTS---ENK---------TYPT   40
_KAF5302274.1_        1  -VKIIEQPA-SKAL-RFRYEC------E---------GRSAGSIPGISSTP---ENK---------TFPA   40
_KAF5308883.1_        1  -VKIIEQPA-SKAL-RFRYEC------E---------GRSAGSIPGVSSTP---ENK---------TFPA   40
_KRT79621.1_          1  --VIVEEPA-QKQL-RFRYPC------E---------GRSAGSIPGANSSP---ENK---------TFPT   39
_BAD20728.1_          1  --VIVEQPA-QKQL-RFRYPC------E---------GRSAGSIPGVGSTP---ENK---------TYPT   39
_KAF2880988.1_        1  -IKITEQPA--LKV-QFRYES------D---------GRCPPPISGVSSTE---KSK---------TFPS   39
_CAH1403258.1_        1  -VRIVEQPA-SKAL-RFRYEC------E---------GRSAGSLPGVHSTP-------------------   33
_CAD6992094.1_        1  -VKIIEQPA-SRMV-RFRYQC------E---------GRSAAAVPGMSSTP---RNK---------TYPE   40
_XP_004537184.1_      1  -VKIIEQPA-SRMV-RFRYQC------E---------GRSAAAVPGMSSTP---RNK---------TYPE   40
_XP_018572644.1_      1  -VRIIEQPA-SKAL-RFRYEC------E---------GRSAGSIPGASSTP---ENK---------TFPT   40
_RZB54393.1__         1  -VKIVEQPA-SKAL-RFRYEC------E---------GRSAGSIPGASSTP---ENK---------TFPS   40
_XP_044255725.1_      1  -VKIIEQPA-SKAL-RFRYEC------E---------GRSAGSIPGASSTP---ENK---------TFPS   40
_EFA02850.1_          1  -VKIIEQPA-SKAL-RFRYEC------E---------GRSAGSIPGASSTP---ENK---------TFPS   40
_NP_001034507.1_      1  -VKIIEQPA-SKAL-RFRYEC------E---------GRSAGSIPGASSTP---ENK---------TFPS   40
_CAH1370311.1_        1  -VKIVEQPA-SKAL-RFRYEC------E---------GRSAGSIPGASSTP---ENK---------TFPS   40
_KAJ3630692.1_        1  -VKIVEQPA-SKAL-RFRYEC------E---------GRSAGSIPGASSTP---ENK---------TFPS   40
_XP_044255724.1_      1  -VKIIEQPA-SKAL-RFRYEC------E---------GRSAGSIPGASSTP---ENK---------TFPS   40
_KAH0811528.1_        1  -VKIVEQPA-SKAL-RFRYEC------E---------GRSAGSIPGASSTP---ENK---------TFPS   40
_CAH7716406.1_        1  -IEIVEQPA-PKAL-RFRYEC------E---------GRSAGSIPGVNSTS---DKK---------TFPT   40
_CAH7716405.1_        1  -IEIVEQPA-PKAL-RFRYEC------E---------GRSAGSIPGVNSTS---DKK---------TFPT   40
_CAH7716404.1_        1  -IEIVEQPA-PKAL-RFRYEC------E---------GRSAGSIPGVNSTS---DKK---------TFPT   40
_XP_028130776.2_      1  -IRIVEQPA-SKAL-RFRYEC------E---------GRSAGSIPGSRSTP---ENK---------SYPT   40
_CAH1985684.1_        1  -IEIVEQPA-PKAL-RFRYEC------E---------GRSAGSIPGIYSSA---EKR---------TFPT   40
_XP_017781152.1_      1  -VRIIEQPA-SKAL-RFRYEC------E---------GRSAGSIPGVNSTA---ENK---------TFPT   40
_XP_017781151.1_      1  -VRIIEQPA-SKAL-RFRYEC------E---------GRSAGSIPGVNSTA---ENK---------TFPT   40
```

```
_XP_048522053.1_     1  -VRIIEQPA-SKAL-RFRYEC------E---------GRSAGSIPGASSTP---ESK---------TFPE   40
_XP_050303070.1_     1  -IRIIEQPA-PKAL-RFRYEC------E---------GRSAGSIPGASSTP---ENK---------KFPE   40
_CAG9762924.1_       1  -VRIIEQPA-SKAL-RFRYEC------E---------GRSAGSIPGASSTP---ENK---------TFPE   40
_CAH0560654.1_       1  -VKIVEQPA-SKAL-RFRYLC------E---------GRSAGSIPGASSTQ---ENK---------TYPA   40
_CAH0560655.1_       1  -VKIVEQPA-SKAL-RFRYLC------E---------GRSAGSIPGASSTQ---ENK---------TYPA   40
_XP_018572643.1_     1  -VRIIEQPA-SKAL-RFRYEC------E---------GRSAGSIPGASSTP---ENK---------TFPT   40
_XP_030763656.1_     1  -VRIIEQPA-SKAL-RFRYEC------E---------GRSAGSIPGASSTP---ENK---------TYPE   40
_XP_030763654.1_     1  -VRIIEQPA-SKAL-RFRYEC------E---------GRSAGSIPGASSTP---ENK---------TYPE   40
_XP_018577945.1_     1  -VRIIEQPA-SKAL-RFRYEC------E---------GRCAGSIPGASSTP---QKK---------TFPT   40
_KAF7283276.1_       1  -VRIIEQPA-SKAL-RFRYEC------E---------GRSAGSIPGVSSTP---ENK---------TYPE   40
_KAF7283275.1_       1  -VRIIEQPA-SKAL-RFRYEC------E---------GRSAGSIPGVSSTP---ENK---------TYPE   40
_XP_030763617.1_     1  -VRIIEQPA-SKAL-RFRYEC------E---------SRFAGSIPGASSTP---EYK---------TYPE   40
_XP_030763619.1_     1  -VRILEQPA-SRAY-RFRYQC------ERS-----PVGSIHGSIQGVSSTP---QIK---------TYPE   44
_VEN60240.1_            ----------------------------------------------------------------------
_KOB78236.1_         1  ----------------FRYRR------E---------CRTNGSIPGISSTA---ANK---------IHPK   27
_ERL85515.1_         1  -GKLI-----------------------------------------------------------------    4
_ENN78766.1_            ----------------------------------------------------------------------
_KAH1019371.1_          ----------------------------------------------------------------------
_KAI9565420.1_       1  -VKILEQPA-SKAL-RFRYEC------E---------GRSAGSLPGANSTT---ENK---------TYPT   40
_XP_045024905.1_     1  -VKILEQPA-SKAL-RFRYEC------E---------GRSAGSLPGANSTT---ENK---------TYPT   40
_XP_046447094.1_     1  -VKILEQPA-SKAL-RFRYEC------E---------GRSAGSLPGANSTT---ENK---------TYPT   40
_KZS08518.1_         1  -VKILEQPA-SKAL-RFRYEC------E---------GRSAGSLPGANSTT---ENK---------TYPT   40
_XP_046636307.1_     1  -VKILEQPA-SKAL-RFRYEC------E---------GRSAGSLPGANSTT---ENK---------TYPT   40
_EFX79013.1_         1  -VKILEQPA-SKAL-RFRYEC------E---------GRSAGSLPGANSTT---ENK---------TYPT   40
_XP_032776606.2_     1  -VKILEQPA-SKAL-RFRYEC------E---------GRSAGSLPGANSTT---ENK---------TYPT   40
_XP_046447093.1_     1  -VKILEQPA-SKAL-RFRYEC------E---------GRSAGSLPGANSTT---ENK---------TYPT   40
_XP_046636306.1_     1  -VKILEQPA-SKAL-RFRYEC------E---------GRSAGSLPGANSTT---ENK---------TYPT   40
_CAH0099799.1_       1  -VKIIEQPA-SKAL-RFRYEC------E---------GRSAGSLPGANSTT---ENK---------TYPT   40
_KAJ1531033.1_       1  -IKILEEPA--QQV-RFRYES------E---------KLFPGT-PGQSSNS---TNK---------TFPT   38
_ACJ36225.1_         1  -VRIIEQPQ-AKAL-RFRYIC------E---------GRSAGSIPGVRSTT---ENK---------TYPA   40
_ACZ98167.1_         1  -VRIIEQPQ-AKAL-RFRYIC------E---------GRSAGSIPGVRSTT---ENK---------TYPA   40
_AME17867.1_         1  -VRIIEQPQ-AKAL-RFRYIC------E---------GRSAGSIPGVRSTT---ENK---------TYPA   40
_XP_042868409.1_     1  -VRIIEQPQ-AKAL-RFRYIC------E---------GRSAGSIPGVRSTT---ENK---------TYPA   40
_XP_045102711.1_     1  -VRLIEQPQ-PKAL-RFRYIC------E---------GRSAGSIPGVNSTA---ENK---------TYPA   40
_XP_047487558.1_     1  -VRIIEQPQ-AKAL-RFRYIC------E---------GRSAGSIPGVRSTT---ENK---------TYPA   40
_QBJ05651.1_         1  -VRLIEQPQ-PKAL-RFRYIC------E---------GRSAGSIPGVNSTA---ENK---------TYPA   40
_AZK36046.1_         1  -VRLIEQPQ-PKAL-RFRYIC------E---------GRSAGSIPGVNSTA---ENK---------TYPA   40
_XP_045581920.1_     1  -VRILEQPQ-AKAL-RFRYIC------E---------GRSAGSIPGVRSTA---ENK---------TYPA   40
_XP_050704598.1_     1  -VRILEQPQ-PKAL-RFRYIC------E---------GRSAGSIPGVSSTA---ENK---------TYPA   40
```

```
_XP_027237995.1_     1  -VRIIEQPQ-AKAL-RFRYIC------E---------GRSAGSIPGVRSTT---ENK---------TYPA  40
_AXB88326.1_         1  -VRLIEQPQ-PKAL-RFRYIC------E---------GRSAGSIPGVNSTA---ENK---------TYPA  40
_XP_045102712.1_     1  -VRLIEQPQ-PKAL-RFRYIC------E---------GRSAGSIPGVNSTA---ENK---------TYPA  40
_MPC17461.1_         1  -VRLIEQPQ-PKAL-RFRYIC------E---------GRSAGSIPGVNSTA---ENK---------TYPA  40
_XP_027237994.1_     1  -VRIIEQPQ-AKAL-RFRYIC------E---------GRSAGSIPGVRSTT---ENK---------TYPA  40
_XP_027237996.1_     1  -VRIIEQPQ-AKAL-RFRYIC------E---------GRSAGSIPGVRSTT---ENK---------TYPA  40
_XP_045102715.1_     1  -VRLIEQPQ-PKAL-RFRYIC------E---------GRSAGSIPGVNSTA---ENK---------TYPA  40
_XP_053627990.1_     1  -VRILEQPQ-AKAL-RFRYIC------E---------GRSAGSIPGVRSTA---ENK---------TYPA  40
_XP_053627989.1_     1  -VRILEQPQ-AKAL-RFRYIC------E---------GRSAGSIPGVRSTA---ENK---------TYPA  40
_XP_053627983.1_     1  -VRILEQPQ-AKAL-RFRYIC------E---------GRSAGSIPGVRSTA---ENK---------TYPA  40
_KAB7495058.1_       1  -VQIIEQPQ-AKAL-RFRYIC------E---------GRSAGSIPGVRSTP---ENK---------TYPT  40
_KAF2353654.1_       1  -VRIIEQPQ-AKAL-RFRYIC------E---------GRSAGSIPGVSSTA---ENK---------TFPS  40
_ANH22235.1_         1  -VKILEQPQ-AKAL-RFRYMC------E---------GRSAGSIPGVRSTA---DNK---------TFPG  40
_CAD7275741.1_       1  --VLLEQPA-SKAL-RFRYIC------E---------GRSAGSLPGERTHG---TYK---------THPT  39
_XP_042243403.1_     1  -VRILEQPQ-PKAL-RFRYIC------E---------GRSAGSIPGVRSTT---ENK---------TYPA  40
_QTC09942.1_         1  -VRIIEQPQ-AKAL-RFRYIC------E---------GRSAGSIPGVRSTT---ENK---------TYPA  40
_XP_018023738.1_     1  -VRIIEQPQ-AKAL-RFRYIC------E---------GRSAGSIPGVRSTA---ENK---------TYPG  40
_XP_019877552.1_     1  -IEIIEQPA-QKAL-RFRYEC------E---------GRSAGSIPGASSTP---ENK---------TFPA  40
_XP_049825875.1_     1  -IEIVEQPA-TKAL-RFRYEC------E---------GRSAGSIPGASSTP---KKK---------TYPT  40
_CAD7649074.1_       1  -VRIVEQPA-KCAL-RFRYEC------E---------GRSAGSIPGSGATA---DNK---------TYPT  40
_CAD7630012.1_       1  -VRIVEQPA-KCAL-RFRYEC------E---------GRSAGSIPASTATA---DNK---------TYPT  40
_XP_054165343.1_     1  -VRIVEQPA-KCAL-RFRYEC------E---------GRSAGSIPGSAATA---DNK---------TYPS  40
_XP_044254219.1_     1  -IRIIEQPQ--KSL-RFRYQC----------------EKRTGTIPGASSTS---TIR---------TLCA  38
_XP_053201827.1_     1  -LKILEQPA-RCAL-RFRYEC------E---------GRSAGSIPGSNSTP---ENK---------TYPS  40
_XP_015786990.1_     1  -VKILEQPA-RCAL-RFRYEC------E---------GRSAGSIPGANSTP---ENK---------TYPT  40
_KAI1295507.1_       1  -VKLIEQPA-RCAL-RFRYEC------E---------GRSAGSIPGSNSTP---ENK---------SYPT  40
_RWS07527.1_         1  -VKIIEQPA-RCAL-RFRYEC------E---------GRSAGSIPGQSTTQ---DNK---------TYPS  40
_RWS29859.1_         1  -VKIVEQPA-KCAL-RFRYEC------E---------GRSAGSIPGQNSSQ---DNK---------TYPT  40
_XP_053201825.1_     1  -LKILEQPA-RCAL-RFRYEC------E---------GRSAGSIPGSNSTP---ENK---------TYPS  40
_XP_054713467.1_     1  -IKIVEQPV--RLL-KYRYKS------DF--------SKRSPGSIYGENSCA---QRT---------TYPT  41
_XP_046915158.1_     1  -VRIIEQPA-KCAI-RFRYEC------E---------GRSAGSIPGVNATP---ENK---------TFPT  40
_KAJ6217218.1_       1  -VRIVEQPA-KCAI-RFRYEC------E---------GRSAGSIPGVNSTT---ENK---------SFPT  40
_KAI2801352.1_       1  -VRIVEQPA-KCAI-RFRYEC------E---------GRSAGSIPGVNSTT---ENK---------SFPT  40
_KAH9406651.1_       1  -VRIVEQPA-KCAI-RFRYEC------E---------GRSAGSIPGVNSTA---ENK---------SFPT  40
_XP_017483538.1_     1  -VRIVEQPA-KCAI-RFRYEC------E---------GRSAGSIPGVNSTA---ENK---------SFPT  40
_KPM10359.1_         1  -VRIIEQPA-KCAI-RFRYEC------E---------GRSAGSIPGINSTP---ENK---------TFPT  40
_UXI17582.1_         1  -VRIIEQPA-KCAI-RFRYEC------E---------GRSAGSIPGINSTP---ENK---------TFPT  40
_KAF7488016.1_       1  -VRIIEQPA-KCAI-RFRYEC------E---------GRSAGSIPGINSTP---ENK---------TFPT  40
_KAH9423377.1_       1  -VRIVEQPA-KCAI-RFRYEC------E---------GRSAGSIPGVNATP---ENK---------TFPT  40
```

```
_OTF73464.1_        1  -VRIIEQPA-KCAI-RFRYEC------E---------GRSAGSIPGVNATP---ENK---------TFPT   40
_XP_037072056.1_    1  -VVITEQPA-SKAL-RFRYEC------E---------GRSAGSIPGANSTA---DSK---------TFPS   40
_XP_037083492.1_    1  -VEITEQPA-SKAL-RFRYEC------E---------GRSAGSIPGANSTS---DNK---------TFPS   40
_XP_037083508.1_    1  -VEITEQPA-SKAL-RFRYEC------E---------GRSAGSIPGANSTS---DNK---------TFPS   40
_XP_043215018.1_    1  -VEITEQPA-SKAL-RFRYEC------E---------GRSAGSIPGANSTA---DNK---------TFPS   40
_KAF0314765.1_      1  -VEITEQPA-SKAL-RFRYEC------E---------GRSAGSIPGANSTA---DNK---------TFPS   40
_XP_043215020.1_    1  -VEITEQPA-SKAL-RFRYEC------E---------GRSAGSIPGANSTA---DNK---------TFPS   40
_XP_043189654.1_    1  -VVITEQPA-SKAL-RFRYEC------E---------GRSAGSIPGAHSTA---DSK---------TFPS   40
_KAF0293344.1_      1  -------------ALRFR-------------------------------------K---------TFPS   10
_XP_011496609.1_    1  -VNIVEQPA-SKGT-RFRYEC------E---------GRFAGTIFGEKTTS---FNK---------SFPT   40
_AGS12619.1_        1  -VKILEQPA-SKGL-RFRYEC------E---------GRSAGSIPGANCTN---EKK---------TFPT   40
_TRY78048.1_        1  -VKILEQPA-SKGL-RFRYEC------E---------GRSAGSIPGANCTN---EKK---------TFPT   40
_XP_040576307.1_    1  -VSVTEEPA-SKGL-RFRYEC------E---------GRSAGSIPGVSSTN---DKK---------TYPT   40
_CAB4056363.1_      1  -VSVTEEPA-SKGL-RFRYEC------E---------GRSAGSIPGVSSTN---DKK---------TYPT   40
_ALF84898.1_        1  -VKITEQPA-SKGL-RFRYEC------E---------GRSAGSIPGISSTA---DVK---------TFPS   40
_XP_023349538.1_    1  -VEVVEQPA-GKGL-RFRYEC------E---------GRSAGSIPG---TV---QYS---------TVQY   37
_XP_004522582.1_    1  -VRIVEQPA-SKAL-RFRYEC------E---------SRFTGTLLGQSSTP---NLK---------TYPT   40
_CAD7013622.1_      1  -VRIAEQPA-SKAL-RFRYEC------E---------SRFTGTLLGQSSTP---NLK---------TYPT   40
_XP_042902217.1_    1  -VRILEQPA-PRSL-RFRYEC------EI-------EGRSAGSIVGINNTS---EKR---------TFPT   42
_XP_042902218.1_    1  -VRILEQPA-PRSL-RFRYEC------EI-------EGRSAGSIVGINNTS---EKR---------TFPT   42
_XP_035215470.1_    1  -VRIIEQPA-SRSL-RFRYEC------EI-------EGRSAGSIVGANNTA---ENR---------TYPT   42
_GBN14612.1_        1  -VRIIEQPA-PRSL-RFRYEC------EI-------EGRSAGSIVGINNTA---ENR---------TYPT   42
_KAF8795965.1_      1  -VRIIEQPA-PRSL-RFRYEC------EI-------EGRSAGSIVGINNTA---ENR---------TYPT   42
_KAF8795964.1_      1  -VRIIEQPA-PRSL-RFRYEC------EI-------EGRSAGSIVGINNTA---ENR---------TYPT   42
_GFY66827.1_        1  -VRIIEQPA-PRSL-RFRYEC------EI-------EGRSAGSIVGINNTP---ENR---------TYPT   42
_GFW66965.1_        1  -VRIIEQPA-PRSL-RFRYEC------EI-------EGRSAGSIVGVNNTP---ENR---------TYPT   42
_GFQ85823.1_        1  -VRIIEQPS-PRSL-RFRYEC------EI-------EGRSAGSIVGVNNTP---ENR---------TYPT   42
_GFT35953.1_        1  -VRIIEQPA-PRSL-RFRYEC------EI-------EGRSAGSIVGVNNTP---ENR---------TYPT   42
_XP_054714028.1_    1  -VRILEQPA-SRSL-RFRYEC------EI-------EGRSAGSIVGASNTP---ENR---------TYPT   42
_KAG8194583.1_      1  -VRIIEQPA-PRSL-RFRYEC------EI-------EGRSAGSIVGVNNTA---ENR---------TYPT   42
_GIY09899.1_        1  -VRIIEQPA-PRSL-RFRYEC------EI-------EGRSAGSIVGISNTA---ENR---------TYPT   42
_GIY93593.1_        1  -VRIVEQPA-PRSL-RFRYEC------EI-------EGRSAGSIVGISNTA---ENR---------TYPT   42
_XP_042902221.1_    1  -VRIVEQPS-PSNF-RFRYER------EYE-----DRCGCIGNLVGINSTY---EKR---------TFPK   44
_XP_046962867.1_    1  -VRIVEQPA-SKAL-RFRYEC------E---------GRSAGSIPGVNSTP---ERK---------TYPT   40
_XP_047530540.1_    1  -VRIVEQPA-SKAL-RFRYEC------E---------GRSAGSIPGVNSTP---ERK---------TYPT   40
_XP_026486466.1_    1  -VRIVEQPA-SKAL-RFRYEC------E---------GRSAGSIPGVNSTP---ERK---------TYPT   40
_XP_047530539.1_    1  -VRIVEQPA-SKAL-RFRYEC------E---------GRSAGSIPGVNSTP---ERK---------TYPT   40
_XP_047530538.1_    1  -VRIVEQPA-SKAL-RFRYEC------E---------GRSAGSIPGVNSTP---ERK---------TYPT   40
_XP_047530537.1_    1  -VRIVEQPA-SKAL-RFRYEC------E---------GRSAGSIPGVNSTP---ERK---------TYPT   40
```

```
_XP_026486464.1_     1  -VRIVEQPA-SKAL-RFRYEC------E---------GRSAGSIPGVNSTP---ERK---------TYPT   40
_XP_047530541.1_     1  -VRIVEQPA-SKAL-RFRYEC------E---------GRSAGSIPGVNSTP---ERK---------TYPT   40
_XP_050347286.1_     1  -VRIVEQPA-SKAL-RFRYEC------E---------GRSAGSIPGVNSTP---ERK---------TYPT   40
_XP_050347283.1_     1  -VRIVEQPA-SKAL-RFRYEC------E---------GRSAGSIPGVNSTP---ERK---------TYPT   40
_XP_032515351.1_     1  -VRIVEQPA-GKAL-RFRYEC------E---------GRSAGSIPGVNSTL---ERK---------TYPT   40
_OWR46016.1_         1  -VRIVEQPA-GKAL-RFRYEC------E---------GRSAGSIPGVNSTL---ERK---------TYPT   40
_XP_032515346.1_     1  -VRIVEQPA-GKAL-RFRYEC------E---------GRSAGSIPGVNSTL---ERK---------TYPT   40
_CAG9579094.1_       1  -VRIVEQPA-GKAL-RFRYEC------E---------GRSAGSIPGVNSTL---ERK---------TYPT   40
_CAG9579091.1_       1  -VRIVEQPA-GKAL-RFRYEC------E---------GRSAGSIPGVNSTL---ERK---------TYPT   40
_XP_050668638.1_     1  -VRIVEQPA-SKAL-RFRYEC------E---------GRSAGSIPGVNSTP---ERK---------TYPT   40
_XP_050668632.1_     1  -VRIVEQPA-SKAL-RFRYEC------E---------GRSAGSIPGVNSTP---ERK---------TYPT   40
_XP_045495329.1_     1  -VRIVEQPA-SKAL-RFRYEC------E---------GRSAGSIPGVNSTP---ERK---------TYPT   40
_XP_038214802.1_     1  -VRIVEQPA-SKAL-RFRYEC------E---------GRSAGSIPGVNSTP---ERK---------TYPT   40
_CAH0714714.1_       1  -VRIVEQPA-GKAL-RFRYEC------E---------GRSAGSIPGVNSTP---ERK---------TYPT   40
_CAH0714713.1_       1  -VRIVEQPA-GKAL-RFRYEC------E---------GRSAGSIPGVNSTP---ERK---------TYPT   40
_CAH4029256.1_       1  -VRIVEQPA-SKAL-RFRYEC------E---------GRSAGSIPGVNSTS---ERK---------TYPT   40
_XP_047513081.1_     1  -VRIVEQPA-SKAL-RFRYEC------E---------GRSAGSIPGVNSTS---ERK---------TYPT   40
_XP_045529569.1_     1  -VRIVEQPA-SKAL-RFRYEC------E---------GRSAGSIPGVNSTS---ERK---------TYPT   40
_XP_045487500.1_     1  -VRIVEQPA-SKAL-RFRYEC------E---------GRSAGSIPGVNSTS---ERK---------TYPT   40
_XP_047513077.1_     1  -VRIVEQPA-SKAL-RFRYEC------E---------GRSAGSIPGVNSTS---ERK---------TYPT   40
_CAH4029257.1_       1  -VRIVEQPA-SKAL-RFRYEC------E---------GRSAGSIPGVNSTS---ERK---------TYPT   40
_XP_045487498.1_     1  -VRIVEQPA-SKAL-RFRYEC------E---------GRSAGSIPGVNSTS---ERK---------TYPT   40
_CAF4952989.1_       1  -VRIVEQPA-SKAL-RFRYEC------E---------GRSAGSIPGVNSTS---DRK---------TYPT   40
_XP_045447470.1_     1  -VRIVEQPA-SKAL-RFRYEC------E---------GRSAGSIPGVNSVP---ERK---------TYPT   40
_CAH2108688.1_          ----------------------------------------------------------------------
_CAH0714724.1_       1  -VRIVEQPA--QSV-RFRYES------E---------GRIEVIFGRSSVKC---KRK---------TYPT   39
_XP_034827218.1_     1  -VRIVEQPA-GKAL-RFRYEC------E---------GRSAGSIPGANSTP---ERR---------SFPT   40
_XP_045768234.1_     1  -VRIVEQPA-GKAL-RFRYEC------E---------GRSAGSIPGANSTP---ERR---------SFPT   40
_XP_034827214.1_     1  -VRIVEQPA-GKAL-RFRYEC------E---------GRSAGSIPGANSTP---ERR---------SFPT   40
_XP_045768230.1_     1  -VRIVEQPA-GKAL-RFRYEC------E---------GRSAGSIPGANSTP---ERR---------SFPT   40
_XP_039751073.1_     1  -VRIVEQPA-GKAL-RFRYEC------E---------GRSAGSIPGANSTP---ERR---------SYPT   40
_XP_023942270.1_     1  -VRIIEQPA-GKAL-RFRYEC------E---------GRSAGSIPGANSTP---ERR---------SYPT   40
_XP_023942268.1_     1  -VRIIEQPA-GKAL-RFRYEC------E---------GRSAGSIPGANSTP---ERR---------SYPT   40
_XP_039751072.1_     1  -VRIVEQPA-GKAL-RFRYEC------E---------GRSAGSIPGANSTP---ERR---------SYPT   40
_XP_023942266.1_     1  -VRIIEQPA-GKAL-RFRYEC------E---------GRSAGSIPGANSTP---ERR---------SYPT   40
_CAH2240541.1_       1  ----------------FRYEC------E---------GRSAGSIPGANSTP---ERR---------SYPT   27
_XP_041985045.1_     1  -VAIVEQPA-GKAL-RFRYEC------E---------GRSAGSIPGCNSTP---ERK---------TYPT   40
_XP_041985024.1_     1  -VAIVEQPA-GKAL-RFRYEC------E---------GRSAGSIPGCNSTP---ERK---------TYPT   40
_XP_049868286.1_     1  -VRIVEQPA-SKAL-RFRYEC------E---------GRSAGSIPGVNSTQ---DRK---------TFPT   40
```

```
_XP_026314794.1_     1  -VRIVEQPA-SRAL-RFRYEC------E---------GRSAGSIPGVNSTQ---DRR---------TYPT   40
_XP_047994271.1_     1  -VRIIEQPA-GKSL-RFRYEC------E---------GRSAGSIPGVHSTP---EKK---------TYPT   40
_KAJ2950301.1_       1  -VRIVEQPA-SKAL-RFRYEC------E---------GRSAGSIPGVNSTA---DHK---------TYPT   40
_XP_031784347.2_     1  -VKIIEEPT-DREC-RFRYDS------E---------GRFA-ELLGVNANE---DHR---------SYPT   39
_KAG7305875.1_       1  -VRILEQPA-SKAL-RFRYEC------E---------GRSAGSIPGASSTP---EKK---------TFPA   40
_WDD23886.1_         1  -VRILEQPA-SKAL-RFRYEC------E---------GRSAGSIPGASSTP---EKK---------TFPA   40
_XP_048480424.1_     1  -VRILEQPA-SKAL-RFRYEC------E---------GRSAGSIPGASSTP---EKK---------TFPA   40
_XP_048480423.1_     1  -VRILEQPA-SKAL-RFRYEC------E---------GRSAGSIPGASSTP---EKK---------TFPA   40
_XP_048480422.1_     1  -VRILEQPA-SKAL-RFRYEC------E---------GRSAGSIPGASSTP---EKK---------TFPA   40
_XP_048480421.1_     1  -VRILEQPA-SKAL-RFRYEC------E---------GRSAGSIPGASSTP---EKK---------TFPA   40
_KAG7305876.1_       1  -VRILEQPA-SKAL-RFRYEC------E---------GRSAGSIPGASSTP---EKK---------TFPA   40
_CAG9129242.1_       1  -VRILEQPA-SKAL-RFRYEC------E---------GRSAGSIPGASSTP---EKK---------TFPA   40
_XP_048480420.1_     1  -VRILEQPA-SKAL-RFRYEC------E---------GRSAGSIPGASSTP---EKK---------TFPA   40
_GBP53004.1_            ----------------------------------------------------------------------
_XP_045534737.1_     1  -VRIVEQPA-SKAL-RFRYEC------E---------GRSAGSIPGVNSTS---ERK---------TYPT   40
_XP_013180060.1_     1  -VRIVEQPA-SKAL-RFRYEC------E---------GRSAGSIPGVNSTS---ERK---------TYPT   40
_XP_013180043.1_     1  -VRIVEQPA-SKAL-RFRYEC------E---------GRSAGSIPGVNSTS---ERK---------TYPT   40
_XP_013180068.1_     1  -VRIVEQPA-SKAL-RFRYEC------E---------GRSAGSIPGVNSTS---ERK---------TYPT   40
_XP_045534736.1_     1  -VRIVEQPA-SKAL-RFRYEC------E---------GRSAGSIPGVNSTS---ERK---------TYPT   40
_KPJ06840.1_         1  -VRIVEQPA-SKAL-RFRYEC------E---------GRSAGSIPGVNSTS---ERK---------TYPT   40
_KPJ05518.1_         1  -VRIVEQPA-SKAL-RFRYEC------E---------GRSAGSIPGVNSTS---ERK---------TYPT   40
_CAG4934519.1_       1  -VRIVEQPA-SKAL-RFRYEC------E---------GRSAGSIPGVNTTS---ERK---------TYPT   40
_XP_013149112.1_     1  -VRIVEQPA-SKAL-RFRYEC------E---------GRSAGSIPGVNSTS---ERK---------TYPT   40
_CAH2077165.1_       1  -VRIVEQPA-SKAL-RFRYEC------E---------GRSAGSIPGVNSTS---ERK---------TYPT   40
_CAH2077167.1_       1  -VRIVEQPA-SKAL-RFRYEC------E---------GRSAGSIPGVNSTS---ERK---------TYPT   40
_XP_037051415.1_     1  -VEIVEQPK--SVE-RFRYES------E--------GRTNRSKIPM--------TPR---------TYAS   35
_XP_037051414.1_     1  -VEIIEQPL--SVD-RFRYPS------EG-------RTSRYTKIPM--------TPR---------TYAS   36
_XP_022815079.1_     1  -VEIVEQPA-SKAL-RFRYEC------E---------GRSAGSIPGVNSTS---DNK---------TYPT   40
_CAB3516522.1_       1  -VEIVEQPA-SKAL-RFRYEC------E---------GRSAGSIPGVNSTS---DNK---------TYPT   40
_XP_050562120.1_     1  -VEIVEQPA-SKAL-RFRYEC------E---------GRSAGSIPGVNSTS---DNK---------TYPT   40
_XP_050562121.1_     1  -VEIVEQPA-SKAL-RFRYEC------E---------GRSAGSIPGVNSTS---DNK---------TYPT   40
_QHD56218.1_         1  -VEIVEQPA-SKAL-RFRYEC------E---------GRSAGSIPGVNSTS---DNK---------TYPT   40
_AIA24466.1_         1  -VEIVEQPC-CKAL-RFRYEC------E---------GRSAGSIPGVNSTS---DNK---------TYPT   40
_XP_022815076.1_     1  -VEIVEQPA-SKAL-RFRYEC------E---------GRSAGSIPGVNSTS---DNK---------TYPT   40
_QHD56217.1_         1  -VEIVEQPA-SKAL-RFRYEC------E---------GRSAGSIPGVNSTS---DNK---------TYPT   40
_XP_050562118.1_     1  -VEIVEQPA-SKAL-RFRYEC------E---------GRSAGSIPGVNSTS---DNK---------TYPT   40
_AIA24467.1_         1  -VEIVEQPC-CKAL-RFRYEC------E---------GRSAGSIPGVNSTS---DNK---------TYPT   40
_XP_047019808.1_     1  -VEIVEQPA-SKAL-RFRYEC------E---------GRSAGSIPGVNSTS---DNK---------TYPT   40
_AEO51736.1_         1  -VEIVEQPA-SKAL-RFRYEC------E---------GRSAGSIPGVNSTS---DNK---------TYPT   40
```

```
_XP_049696680.1_     1  -VEIVEQPA-SKAL-RFRYEC------E---------GRSAGSIPGVNSTS---DNK---------TYPT   40
_XP_049696682.1_     1  -VEIVEQPA-SKAL-RFRYEC------E---------GRSAGSIPGVNSTS---DNK---------TYPT   40
_XP_049696676.1_     1  -VEIVEQPA-SKAL-RFRYEC------E---------GRSAGSIPGVNSTS---DNK---------TYPT   40
_XP_026744931.1_     1  -VEIVEQPA-SKAL-RFRYEC------E---------GRSAGSIPGVNSTT---DNK---------TYPT   40
_XP_026732895.1_     1  -VEIVEQPA-SKAL-RFRYEC------E---------GRSAGSIPGVNSTT---DNK---------TYPT   40
_CAH0581650.1_       1  -VEIVEQPA-SKAL-RFRYEC------E---------GRSAGSIPGVNSTT---DNK---------TYPT   40
_KAH9640014.1_       1  -VEIVEQPA-SKAL-RFRYEC------E---------GRSAGSIPGVNSTS---DNK---------TYPT   40
_PZC78315.1_            ----------------------------------------------------------------------
_KAF9795796.1_          ----------------------------------------------------------------------
_AEC52926.1_         1  -VRIVQQPA-SKAL-RFRYEC------E---------GRSAGSIPGASSTP---ENR---------TYPT   40
_KAH7940301.1_       1  -VVILEQPA-SRAL-RFRYQC------E---------GRYPGTLVGVSSTA---ENK---------TYPT   40
_ADK39025.1_         1  --VVVQQPA-SKAL-RFRYEC------E---------GRSAGSIPGASSTP---ENR---------TFPA   39
_XP_037298148.1_     1  --VVVQQPA-SKAL-RFRYEC------E---------GRSAGSIPGASSTP---ENR---------TFPA   39
_RVE50742.1_         1  --YIVEQPA-PKSL-RFRYEC------E---------GRSAGSIPGVHSTP---ENK---------TFPT   39
_CAH0692665.1_       1  --YIVEQPA-PKSL-RFRYEC------E---------GRSAGSIPGVHSTP---ENK---------TFPT   39
_XP_028166351.1_     1  ---IIEQPA-PKSL-RFRYEC------E---------GRSAGSIPGVNSTQ---ENK---------TFPT   38
_XP_028166291.1_     1  ---IIEQPA-PKSL-RFRYEC------E---------GRSAGSIPGVNSTQ---ENK---------TFPT   38
_CAB3235314.1_       1  -VRIIEQPA-SKAL-RFRYEC------E---------GRSAGSIPGVNSTS---ENK---------TYPT   40
_KAJ0175867.1_       1  -VRIVEQPA-SKAL-RFRYEC------E---------GRSAGSIPGVNSTP---ENR---------TYPT   40
_XP_028031027.1_     1  -VVIVEQPA-SKAL-RFRYKC------E---------GRSAGSLPGVSSTP---ENK---------TYPT   40
_NP_001166296.1_     1  -VVIVEQPA-SKAL-RFRYKC------E---------GRSAGSLPGVSSTP---ENK---------TYPT   40
_XP_037870194.1_     1  -VVIVEQPA-SKAL-RFRYKC------E---------GRSAGSLPGVSSTP---ENK---------TYPT   40
_XP_037870193.1_     1  -VVIVEQPA-SKAL-RFRYKC------E---------GRSAGSLPGVSSTP---ENK---------TYPT   40
_XP_028031025.1_     1  -VVIVEQPA-SKAL-RFRYKC------E---------GRSAGSLPGVSSTP---ENK---------TYPT   40
_XP_013187225.1_     1  --RITEQPA-GKAL-RFRYEC------E---------GRSAGSIPGVNSTP---ENR---------TYPT   39
_XP_052755011.1_     1  --KIVEQPA-GKAL-RFRYEC------E---------GRSAGSIPGVNSTP---ENR---------TYPT   39
_XP_053613182.1_     1  --RITEQPA-GKAL-RFRYEC------E---------GRSAGSIPGVKSTP---ENR---------TYPT   39
_XP_053613180.1_     1  --RITEQPA-GKAL-RFRYEC------E---------GRSAGSIPGVKSTP---ENR---------TYPT   39
_CAG9785863.1_       1  --YIVEQPA-AKAL-RFRYEC------E---------GRSAGSIPGVHSTP---ENK---------TYPT   39
_KAG6461275.1_       1  --VVVQQPA-SKAL-RFRYEC------E---------GRSAGSIPGASSTP---ENR---------TFPA   39
_KAG6461281.1_       1  --VVVQQPA-SKAL-RFRYEC------E---------GRSAGSIPGASSTP---ENR---------TFPA   39
_XP_037302137.1_     1  --IITEQPA-SSKL-RFRYSS------E---------GMS-GSILGALSRP---ENP---------TFPT   38
_KAG6461269.1_       1  --IITEQPA-SSKL-RFRYSS------E---------GMS-GSILGALSRP---ENP---------TFPT   38
_KAG4069896.1_       1  -VEIVEQPA-SVE--RFRYQS----------------EGR----INRSNIA---TPR---------TYAS   34
_XP_044003989.1_     1  -IEIVEQPA--SAT-RFRYTG----------------ENRTETLLGRSSTK---TTK---------TYFK   38
_XP_045768240.1_     1  -VRIVEQPA--KTV-RFRYPC------E---------GRLAGSVQGVNSRP--PMRR---------TYPT   40
_KAG9509099.1_       1  -VRIVEQPA-KRAR-RFRYAC------E---------SRSAGSWPGVNSCD---EQK---------TYPA   40
_XP_013118267.1_     1  -IRIVEQPH-PKGL-RFRYIC------E---------GRSAGSLPGINSTP---ENK---------TYPT   40
_XP_005183026.2_     1  -IRIVEQPH-PKGL-RFRYIC------E---------GRSAGSIPGINSTP---DNK---------TYPT   40
```

```
_XP_050303432.1_     1  --SMIEQPA-SDGLGKFRYQS------ET------GSGRRAGCLLGVNTTN---DNK---------SFPK   43
_TMW48950.1_         1  -IKIVEQPA-LKGL-RFRYIC------E---------GRSAGSIPGVHSTP---ENK---------TFPT   40
_XP_037882085.1_     1  -IRIVEQPA-NKAL-RFRYEC------E---------GRSAGSIPGANSTT---ENK---------TFPT   40
_KAI8126541.1_       1  RLKIVEQPA-PKGL-RFRYIC------E---------GRSAGSIPGVHSTP---ENK---------TFPT   41
_KAI8126540.1_       1  -LKIVEQPA-PKGL-RFRYIC------E---------GRSAGSIPGVHSTP---ENK---------TFPT   40
_XP_037811156.1_     1  -LKIVEQPA-LKGL-RFRYIC------E---------GRSAGSIPGVHSTP---ENK---------TFPT   40
_XP_046812595.1_     1  -LKIVEQPA-PKGL-RFRYIC------E---------GRSAGSIPGVHSTP---ENK---------TFPT   40
_XP_046812596.1_     1  -LKIVEQPA-PKGL-RFRYIC------E---------GRSAGSIPGVHSTP---ENK---------TFPT   40
_XP_046812591.1_     1  -LKIVEQPA-PKGL-RFRYIC------E---------GRSAGSIPGVHSTP---ENK---------TFPT   40
_XP_046812590.1_     1  -LKIVEQPA-PKGL-RFRYIC------E---------GRSAGSIPGVHSTP---ENK---------TFPT   40
_KNC34143.1_         1  -LKIVEQPA-PKGL-RFRYIC------E---------GRSAGSIPGVHSTP---ENK---------TFPT   40
_XP_046812589.1_     1  -LKIVEQPA-PKGL-RFRYIC------E---------GRSAGSIPGVHSTP---ENK---------TFPT   40
_KAI8126539.1_       1  -LKIVEQPA-PKGL-RFRYIC------E---------GRSAGSIPGVHSTP---ENK---------TFPT   40
_XP_037958917.1_     1  -INIVEQPA-TKAL-RFRYKC------E---------GRSAGSIPGVNSTP---ENK---------TYPT   40
_XP_037929595.1_     1  -INIVEQPA-TKPL-KFRYKS------EC-------EGRSAGSIPGLNSTP---ENK---------TYPT   42
_XP_037957513.1_     1  -INIVEQPA-TKAL-RFRYK--------C-------EGRSAGSIPGLNSTP---ENK---------TYPT   40
_XP_037958115.1_     1  -INIVEQPA-TKPL-KFRYKS------EC-------EGRSAGSIPGLNSTP---ENK---------TYPT   42
_XP_037958114.1_     1  -INIVEQPA-TKPL-KFRYKS------EC-------EGRSAGSIPGLNSTP---ENK---------TYPT   42
_XP_037957511.1_     1  -INIVEQPA-TKAL-RFRYK--------C-------EGRSAGSIPGLNSTP---ENK---------TYPT   40
_XP_037958918.1_     1  -INIVEQPA-TKAL-RFRYKC------E---------GRSAGSIPGVNSTP---ENK---------TYPT   40
_RXG71823.1_         1  -VQITEQPR-PKAL-RFRYIC------E---------GRSAGSIPGVHSTP---TRK---------TFPT   40
_KAB7494622.1_          ----------------------------------------------------------------------
_KAI9584339.1_       1  -VRIVEQPA-SKPM-RFRYEC------E---------GRSGGSILGANSTP---ENK---------TYPS   40
_XP_037884983.1_     1  -VRIVEQPF-NKAM-RFRYEC------E---------GRSGSAILGANSTL---EHK---------TYPS   40
_XP_054744821.1_     1  -LRVVEQPT-RKPM-RFRYKC------E---------GRTAGTIPGENSFQ---ETK---------TFPT   40
_XP_053961676.1_     1  -LRVVEQPT-RKPM-RFRYKC------E---------GRTAGTIPGENSFQ---ETK---------TFPT   40
_XP_053961674.1_     1  -LRVVEQPT-RKPM-RFRYKC------E---------GRTAGTIPGENSFQ---ETK---------TFPT   40
_XP_004526634.2_     1  -LRVVEQPT-RKPM-RFRYKC------E---------GRTAGTIPGENSFQ---DTK---------TFPT   40
_CAD6998451.1_       1  -LRVVEQPT-RKPM-RFRYKC------E---------GRTAGTIPGENSFQ---DTK---------TFPT   40
_XP_012157894.1_     1  -LRVVEQPT-RKPM-RFRYKC------E---------GRTAGTIPGENSFQ---DTK---------TFPT   40
_XP_028894493.1_     1  -LRVVEQPT-RKPM-RFRYKC------E---------GRTAGTIPGENSFQ---ETK---------TFPT   40
_XP_036327693.1_     1  -LHVVEQPT-RKPM-RFRYKC------E---------GRTAGTIPGENSFQ---ETK---------TFPT   40
_XP_017471333.1_     1  -LHVVEQPT-RKPM-RFRYKC------E---------GRTAGTIPGENSFQ---ETK---------TFPT   40
_XP_039952608.1_     1  -LRVVEQPS-RKPM-RFRYNS------E---------GRTAGNIPGENSFQ---ETK---------TFPT   40
_XP_050320023.1_     1  -LRVVEQPS-RKPM-RFRYNS------E---------GRTAGNIPGENSFQ---ETK---------TFPT   40
_XP_036225572.1_     1  -LRVVEQPS-RKPM-RFRYNS------E---------GRTAGNIPGENSFQ---ETK---------TFPT   40
_XP_039952607.1_     1  -LRVVEQPS-RKPM-RFRYNS------E---------GRTAGNIPGENSFQ---ETK---------TFPT   40
_XP_036225564.1_     1  -LRVVEQPS-RKPM-RFRYNS------E---------GRTAGNIPGENSFQ---ETK---------TFPT   40
_XP_036225569.1_     1  -LRVVEQPS-RKPM-RFRYNS------E---------GRTAGNIPGENSFQ---ETK---------TFPT   40
```

```
_XP_050320021.1_     1  -LRVVEQPS-RKPM-RFRYNS------E---------GRTAGNIPGENSFQ---ETK---------TFPT   40
_XP_039952606.1_     1  -LRVVEQPS-RKPM-RFRYNS------E---------GRTAGNIPGENSFQ---ETK---------TFPT   40
_XP_011207860.1_     1  -LRVVEQPS-RKPM-RFRYNS------E---------GRTAGNIPGENSFQ---ETK---------TFPT   40
_XP_011207859.1_     1  -LRVVEQPS-RKPM-RFRYNS------E---------GRTAGNIPGENSFQ---ETK---------TFPT   40
_XP_029407466.1_     1  -LRVVEQPS-RKPM-RFRYNS------E---------GRTAGNIPGENSFQ---ETK---------TFPT   40
_XP_019847003.2_     1  -LRVVEQPS-RKPM-RFRYNS------E---------GRTAGNIPGENSFQ---ETK---------TFPT   40
_XP_014086488.2_     1  -LRVVEQPS-RKPM-RFRYNS------E---------GRTAGNIPGENSFQ---ETK---------TFPT   40
_XP_049317393.1_     1  -LRVVEQPS-RKPM-RFRYNS------E---------GRTAGNIPGENSFQ---ETK---------TFPT   40
_XP_039952604.1_     1  -LRVVEQPS-RKPM-RFRYNS------E---------GRTAGNIPGENSFQ---ETK---------TFPT   40
_XP_018787557.1_     1  -LRVVEQPS-RKPM-RFRYNS------E---------GRTAGNIPGENSFQ---ETK---------TFPT   40
_OQR68950.1_         1  -VRILVQPQ-SRAL-RFRYKC------E---------GRYPGALTGVGTTN---EVK---------MYPQ   40
_XP_003741763.1_     1  -VTILVQPQ-SRAL-RFRYKC------E---------GRYPGALTGLGSTA---ENK---------TYPT   40
_XP_022699297.1_     1  -VRITVQPQ-SRAL-RFRYKC------E---------GRYPGALTGVGTTN---DVK---------TYPQ   40
_XP_022665229.1_     1  -VRITVQPQ-SRAL-RFRYKC------E---------GRYPGALTGVGTTN---DVK---------TYPQ   40
_XP_022665225.1_     1  -VRITVQPQ-SRAL-RFRYKC------E---------GRYPGALTGVGTTN---DVK---------TYPQ   40
_XP_022665224.1_     1  -VRITVQPQ-SRAL-RFRYKC------E---------GRYPGALTGVGTTN---DVK---------TYPQ   40
_XP_033219687.1_     1  -VEVVEQPS-SNTI-RFRYEC------E---------KRSTGCIMGVNSSP---KEK---------TYPS   40
_XP_033227990.1_     1  -VEVVEQPS-TNTV-RFRYEC------E---------KRATRCIMGVNSSP---KEK---------TYPS   40
_XP_043466013.1_     1  -VTIIEHPS--HSL-RFRYEC------E---------RRCAGRILGENSVA---ERR---------TFPT   39
_XP_051172671.1_     1  -VTIIEHPT--HSL-RFRYEC------E---------RRCAGRILGENSVA---DRR---------TFPT   39
_CAD7643228.1_       1  --RITEQPA-KCGV-RFRYEC------E---------GRISGAIPGQSSTT---VRP---------TFPT   39
_XP_054157157.1_     1  -VQIVEQPA-KCGV-RFRYEC------E---------GRISGAIPGSRSTP---SSP---------TFPA   40
_CAD7645633.1_       1  --RIVEQPA-KCGV-RFRYEC------E---------GRISGAIPGQNSTT---LRP---------TFPT   39
_CAD7632611.1_          ----------------------------------------------------------------------
_KAJ6223158.1_       1  ---IVEQPA-KCGV-RFRYEC------E---------GRISSAIPGSSATN---SCY---------SFPT   38
_KAI2804035.1_       1  ---IVEQPA-KCGV-RFRYEC------E---------GRISSAIPGSSATN---SCY---------SFPT   38
_KAH9398209.1_       1  ---IVEQPA-KCGV-RFRYEC------E---------GRISSAIPGASSTA---SRF---------TYPT   38
_GFY77286.1_         1  -VEILEQPV-SIGA-RFRYKC------E---------GRSAGCIRGINSSD---KQK---------TYPT   40
_GFQ73388.1_         1  -VEILEQPV-SIGA-RFRYKC------E---------GRSAGCIRGINSSD---KQK---------TYPT   40
_GFT25575.1_         1  -VEILEQPV-SIGA-RFRYKC------E---------GRSAGCIRGINSSD---KQK---------TYPT   40
_KAF8763655.1_       1  -VEILEQPV-SIGA-RFRYKC------E---------GRSAGCIRGINSSD---KQK---------TYPT   40
_GFX76677.1_         1  -VEILEQPV-SIGA-RFRYKC------E---------GRSAGCIRGINSSD---KQK---------TYPT   40
_XP_035207052.1_     1  -VEIIEQPV-SIGA-RFRYKC------E---------GRSAGCIRGVNSTD---KQK---------TYPT   40
_KFM74898.1_         1  -VEIIEQPV-SIGA-RFRYKC------E---------GRSAGCIRGVNSTD---KQK---------TYPT   40
_XP_035207059.1_     1  -VEIIEQPV-SIGA-RFRYKC------E---------GRSAGCIRGVNSTD---KQK---------TYPT   40
_XP_035207066.1_     1  -VEIIEQPV-SIGA-RFRYKC------E---------GRSAGCIRGVNSTD---KQK---------TYPT   40
_BBD75287.1_         1  -VEVLEQPV-SIGA-RFRYKC------E---------GRSAGCIRGVNSTD---KQK---------TYPT   40
_XP_042900952.1_     1  -VEVLEQPV-SIGA-RFRYKC------E---------GRSAGCIRGVNSTD---KQK---------TYPT   40
_KAG8201816.1_       1  -VEILEQPV--SSV-RFRYKC------E---------GRSAGCIKGVNSTD---KQK---------TYPT   39
```

```
_GIX87435.1_        1  -VEILEQPV-SVGA-RFRYKC------E---------GRSAGCIRGINSSD---KQK---------TYPT  40
_GBL81029.1_        1  -VEILEQPI-SVGA-RFRYKC------E---------GRSAGCIRGINSSD---KQK---------TYPT  40
_GIY01087.1_        1  -VEILEQPV-SVGA-RFRYKC------E---------GRSAGCIRGINSSD---KQK---------TYPT  40
_XP_054715436.1_    1  -VQIVEQPA-SIGA-RFRYKC------E---------GRSAGCIRGVSSTE---KDK---------TFPA  40
_XP_044576112.1_    1  -IEILEQPC--SKS-RFRYPC------E---------ARNNGSIMGATNNS---ERK---------TWPK  39
_XP_008556435.3_    1  --EIIEQPC--SRT-RFRYEC------E---------ARFIGSLVGCNSTE---HHK---------SWPT  38
_CAD6234446.1_      1  -IEILEQPC--SKS-RFRYPC------E---------ARNNGSIVGATNNS---ERK---------TWPK  39
_XP_044284598.1_    1  -IEICEQPR-QRGM-RFRYKC------E---------GRSAGSIPGERSTD---INR---------TFPS  40
_XP_033794511.1_    1  -VEMFEQPK-QRGM-RFRYKC------E---------GRSAGSILGERSTV---NNK---------SYPS  40
_XP_033794512.1_    1  -VEMFEQPK-QRGM-RFRYKC------E---------GRSAGSILGERSTV---NNK---------SYPS  40
_XP_029449578.1_    1  -VEIFEQPK-QRGM-RFRYKC------E---------GRSAGSILGERSTV---NNK---------SYPS  40
_XP_030052048.1_    1  -VEMFEQPK-QRGM-RFRYKC------E---------GRSAGSILGERSTV---NNK---------SYPS  40
_XP_007423639.1_    1  -IEICEQPR-QRGM-RFRYKC------E---------GRSAGSIPGEHSTD---TNR---------TFPS  40
_XP_039203483.1_    1  -IEIHEQPR-QRGM-RFRYKC------E---------GRSAGSIPGEHSTD---INR---------TFPS  40
_XP_034283206.1_    1  -IEIHEQPR-QRGM-RFRYKC------E---------GRSAGSIPGEHSTD---INR---------TFPS  40
_XP_033001023.1_    1  -IEICEQPR-QRGM-RFRYKC------E---------GRSAGSIPGEHSSD---MNR---------TFPA  40
_XP_053239675.1_    1  -IEICEQPR-QRGM-RFRYKC------E---------GRSAGSIPGEHSSD---MNR---------TFPA  40
_XP_034966804.1_    1  -IEICEQPR-QRGM-RFRYKC------E---------GRSAGSIPGEHSSD---MNR---------TFPA  40
_XP_028580982.1_    1  -------------M-RFRYKC------E---------GRSAGSIPGEHSSD---MNR---------TFPA  29
_XP_042302822.1_    1  -IEICEQPR-QRGM-RFRYKC------E---------GRSAGSIPGEHSTD---INR---------TFPS  40
_XP_015280979.1_    1  -------------M-RFRYQC------E---------GRSAGSIPGEHSTD---INR---------TFPA  29
_XP_048363544.1_    1  -IEIREQPR-QRGM-RFRYQC------E---------GRSAGSILGEHSTD---INR---------TFPT  40
_KAF7252806.1_      1  -SGI---------------------------------NAKAGQLAASQEN--------------------  16
_XP_034958398.1_       ----------------------------------------------------------------------
_CAD5116312.1_      1  -LEIIEEPR-QKGQ-RFRYEC------E---------GRYAGHIPGEKSTE---ENK---------SYPT  40
_KAJ1519398.1_      1  -VRILEEPF-PRA--KFRYEA----------------ELLGPTIKGARATA---YTK---------TFPK  38
_XP_053103001.1_    1  -IEICEQPR-QRGM-RFRYKC------E---------GRSAGSIPGEHSTD---INR---------TFPS  40
_XP_032070730.1_    1  -IEIHEQPR-QRGM-RFRYKC------E---------GRSAGSIPGEHSTD---INR---------TFPS  40
_XP_013922116.1_    1  -IEIHEQPR-QRGM-RFRYKC------E---------GRSAGSIPGEHSTD---INR---------TFPS  40
_XP_015667999.1_    1  -IEIHEQPR-QRGM-RFRYKC------E---------GRSAGSIPGEHSTD---INR---------TFPS  40
_XP_026551846.1_    1  -IEIYEQPR-QRGM-RFRYKC------E---------GRSAGSIPGEHSTD---INR---------TFPS  40
_XP_026532518.1_    1  -IEIHEQPR-QRGM-RFRYKC------E---------GRSAGSIPGEHSTD---INR---------TFPS  40
_KAJ7341301.1_      1  -IEIHEQPR-QRGM-RFRYKC------E---------GRSAGSIPGEHSTD---INR---------TFPS  40
_KAJ6667038.1_      1  -------------M-RFRYQC------E---------GRSAGSIPGEHSTD---LNR---------TFPS  29
_XP_003227818.2_    1  -------------M-RFRYKC------E---------GRSAGSIPGEHTTD---INR---------TFPS  29
_XP_020633036.1_    1  -------------M-RFRYKC------E---------GRSAGSIPGEHSTD---INR---------TFPS  29
_XP_054842059.1_    1  -IEIYEQPR-QRGM-RFRYQC------E---------GRSAGSIPGEHSTD---INR---------TFPS  40
_XP_005238641.2_    1  -------------M-RFRYKC------E---------GRSAGSIPGERSTE---NNK---------TFPS  29
_XP_005445671.2_    1  -------------M-RFRYKC------E---------GRSAGSIPGERSTE---NNK---------TFPS  29
```

```
_XP_054050029.1_     1  -------------M-RFRYKC------E---------GRSAGSIPGEHSTD---NNK---------TFPS   29
_XP_013810845.1_     1  -------------M-RFRYKC------E---------GRSAGSIPGEHSTE---NNK---------TFPS   29
_XP_026702002.1_     1  -------------M-RFRYKC------E---------GRSAGSIPGEHSTE---NNK---------TFPS   29
_XP_025892242.1_     1  -------------M-RFRYKC------E---------GRSAGSIPGEHSTD---NNK---------TFPS   29
_XP_005507389.1_     1  -------------M-RFRYKC------E---------GRSAGSIPGEHSTE---NNK---------TFPS   29
_XP_032911247.1_     1  -------------M-RFRYKC------E---------GRSAGSIPGEHSTE---NNK---------TFPS   29
_XP_031959657.1_     1  -------------M-RFRYKC------E---------GRSAGSIPGEHSTE---NNK---------TFPS   29
_XP_041905291.1_     1  -------------M-RFRYKC------E---------GRSAGSIPGEHSTE---NNK---------TFPS   29
_XP_051648836.1_     1  -------------M-RFRYKC------E---------GRSAGSIPGEHSTE---NNK---------TFPS   29
_XP_054239488.1_     1  -------------M-RFRYKC------E---------GRSAGSIPGEHSTE---NNK---------TFPS   29
_XP_039914164.1_     1  -------------M-RFRYKC------E---------GRSAGSIPGEHSTE---NNK---------TFPS   29
_XP_005422689.1_     1  -------------M-RFRYKC------E---------GRSAGSIPGEHSTE---NNK---------TFPS   29
_KAF2987757.1_       1  -------------M-RFRYKC------E---------GRSAGSIPGERSTE---NNK---------SFPS   29
_XP_038665087.1_     1  -VEIVEQPK-QRGM-RFRYKC------E---------GRSAGSIPGEHSTD---NNR---------TYPS   40
_GCB59945.1_         1  -VEIVEQPK-QRGM-RFRYKC------E---------GRSAGSIPGEHSTD---NNR---------TYPS   40
_XP_041065616.1_     1  -VEIVEQPK-QRGM-RFRYKC------E---------GRSAGSIPGEHSTD---NNR---------TYPS   40
_XP_007890564.1_     1  -VEIAEQPK-QRGM-RFRYKC------E---------GRSAGSIPGEHSTD---NSR---------TYPS   40
_XP_051868609.1_     1  -VEIVEQPK-QRGM-RFRYKC------E---------GRSAGSIPGEHSMD---NNR---------TYPS   40
_XP_032881051.1_     1  --EMVEQPK-PRGM-RFRYKC------E---------GRSAGSIPGANTKD---NNR---------TYPA   39
_XP_048456938.1_        ----------------------------------------------------------------------
_KAJ1155552.1_       1  -VEIFEQPK-QRGM-RFRYRV------E---------GRSAGSIPGERSTD---NNR---------TYPA   40
_KAH0622877.1_          ----------------------------------------------------------------------
_CAI5653329.1_       1  -IQIFEQPK-QRGM-RFRYKC------E---------GRSAGSIPGEKSSD---NNR---------TYPS   40
_CAI5653328.1_       1  -IQIFEQPK-QRGM-RFRYKC------E---------GRSAGSIPGEKSSD---NNR---------TYPS   40
_NXS54806.1_         1  -IEIFEQPR-QRGM-RFRYKC------E---------GRSAGSIPGEHSTD---NNK---------TFPS   40
_XP_009504395.1_     1  ----FEQPR-QRGM-RFRYKC------E---------GRSAGSIPGEHSTE---NNK---------TFPS   37
_NXJ41381.1_         1  -IEIFEQPR-QRGM-RFRYKC------E---------GRSAGSIPGEHSTE---NNK---------TFPS   40
_NXT24719.1_         1  -IEIFEQPR-QRGM-RFRYKC------E---------GRSAGSIPGEHSTE---NNK---------TFPS   40
_NXK23502.1_         1  -IEIFEQPR-QRGM-RFRYKC------E---------GRSAGSIPGEHSTE---NNK---------TFPS   40
_NXK03070.1_         1  -IEIFEQPR-QRGM-RFRYKC------E---------GRSAGSIPGERSTE---NNK---------TFPS   40
_XP_037260944.1_     1  -IEIFEQPR-QRGM-RFRYKC------E---------GRSAGSIPGERSTE---NNK---------TFPS   40
_XP_040467369.1_     1  -IEIFEQPR-QRGM-RFRYKC------E---------GRSAGSIPGERSTE---NNK---------TFPS   40
_NXY71491.1_         1  -IEIFEQPR-QRGM-RFRYKC------E---------GRSAGSIPGEHSTD---NNK---------TFPS   40
_NXN54417.1_         1  -IEIFEQPR-QRGM-RFRYKC------E---------GRSAGSIPGEHSTD---NNK---------TFPS   40
_NXX19490.1_         1  -IEIFEQPR-QRGM-RFRYKC------E---------GRSAGSIPGEHSTE---NNK---------TFPS   40
_NXL47199.1_         1  -IEIFEQPR-QRGM-RFRYKC------E---------GRSAGSIPGEHSTE---NNK---------TFPS   40
_XP_050749583.1_     1  -IEIFEQPR-QRGM-RFRYKC------E---------GRSAGSIPGEHSTE---NNK---------TFPS   40
_NXD76242.1_         1  -IEIFEQPR-QRGM-RFRYKC------E---------GRSAGSIPGEHSTE---NNK---------TFPS   40
_KFZ56827.1_         1  -IEIFEQPR-QRGM-RFRYKC------E---------GRSAGSIPGEHSTE---NNK---------TFPS   40
```

NXH67691.1	1	-IEIFEQPR-QRGM-RFRYKC------E---------GRSAGSIPGEHSTE---NNK---------TFPS	40
NXS45828.1	1	-IEMFEQPR-QRGM-RFRYKC------E---------GRSAGSIPGEHSTE---NNK---------TFPS	40
KFP52034.1	1	-IEIFEQPR-QRGM-RFRYKC------E---------GRSAGSIPGEHSTE---NNK---------TFPS	40
NWH44485.1	1	-IEMFEQPR-QRGM-RFRYKC------E---------GRSAGSIPGEHSTE---NNK---------TFPS	40
NWW85416.1	1	-VEIFEQPR-QRGM-RFRYKC------E---------GRSAGSIPGEHSTE---NNK---------TFPS	40
NWU66320.1	1	-IEIFEQPR-QRGM-RFRYKC------E---------GRSAGSIPGEHSTD---NNK---------TFPS	40
_XP_025919906.1_	1	-IEIFEQPR-QRGM-RFRYKC------E---------GRSAGSIPGEHSTE---NNK---------TFPS	40
KAF1483947.1	1	-IEIFEQPR-QRGM-RFRYKC------E---------GRSAGSIPGEHSTE---NNK---------TFPS	40
NXQ87688.1	1	-IEIFEQPR-QRGM-RFRYKC------E---------GRSAGSIPGEHSTE---NNK---------TFPS	40
NXX07562.1	1	-IEIFEQPR-QRGM-RFRYKC------E---------GRSAGSIPGEHSTD---NNK---------TFPS	40
_XP_009280788.2_	1	-IEIFEQPR-QRGM-RFRYKC------E---------GRSAGSIPGEHSTE---NNK---------TFPS	40
_XP_014821857.1_	1	-IEIFEQPR-QRGM-RFRYKC------E---------GRSAGSIPGEHSTE---NNK---------TFPS	40
NWU51443.1	1	-IEIFEQPR-QRGM-RFRYKC------E---------GRSAGSIPGEHSTD---NNK---------TFPS	40
NXV35521.1	1	-IEIFEQPR-QRGM-RFRYKC------E---------GRSAGSIPGEHSTD---NNK---------TFPS	40
KFV80780.1	1	-IEIFEQPR-QRGM-RFRYKC------E---------GRSAGSIPGEHSTE---NNK---------TFPS	40
_XP_032039522.1_	1	-IEIFEQPR-QRGM-RFRYKC------E---------GRSAGSIPGEHSTE---NNK---------TFPS	40
NWT43027.1	1	-IEIFEQPR-QRGM-RFRYKC------E---------GRSAGSIPGEHSTD---NNK---------TFPS	40
KAF1651581.1	1	-IEIFEQPR-QRGM-RFRYKC------E---------GRSAGSIPGEHSTE---NNK---------TFPS	40
NXU26374.1	1	-IEIFEQPR-QRGM-RFRYKC------E---------GRSAGSIPGEHSTE---NNK---------TFPS	40
NWX46229.1	1	-IEIFEQPR-QRGM-RFRYKC------E---------GRSAGSIPGEHSTE---NNK---------TFPS	40
PKU40340.1	1	-IEIFEQPR-QRGM-RFRYKC------E---------GRSAGSIPGEHSTE---NNK---------TFPS	40
_XP_027309078.1_	1	-IEIFEQPR-QRGM-RFRYKC------E---------GRSAGSIPGEHSTE---NNK---------TFPS	40
KAF1573683.1	1	-IEIFEQPR-QRGM-RFRYKC------E---------GRSAGSIPGEHSTE---NNK---------TFPS	40
KAF1578928.1	1	-IEIFEQPR-QRGM-RFRYKC------E---------GRSAGSIPGEHSTE---NNK---------TFPS	40
NXN47104.1	1	-IEIFEQPR-QRGM-RFRYKC------E---------GRSAGSIPGEHSTD---NNK---------TFPS	40
NXV20393.1	1	-IEIFEQPR-QRGM-RFRYKC------E---------GRSAGSIPGEHSTD---NNK---------TFPS	40
KAF1551267.1	1	-IEIFEQPR-QRGM-RFRYKC------E---------GRSAGSIPGEHSTE---NNK---------TFPS	40
KAF1406210.1	1	-IEIFEQPR-QRGM-RFRYKC------E---------GRSAGSIPGEHSTE---NNK---------TFPS	40
NXV43201.1	1	-IEIFEQPR-QRGM-RFRYKC------E---------GRSAGSIPGEHSTD---NNK---------TFPS	40
NWQ98669.1	1	-IEIFEQPR-QRGM-RFRYKC------E---------GRSAGSIPGEHSTE---NNK---------TFPS	40
_XP_052639968.1_	1	-IEIFEQPR-QRGM-RFRYKC------E---------GRSAGSIPGEHSTE---NNK---------TFPS	40
_XP_010573143.1_	1	-IEIFEQPR-QRGM-RFRYKC------E---------GRSAGSIPGEHSTE---NNK---------TFPS	40
KAF1479604.1	1	-IEIFEQPR-QRGM-RFRYKC------E---------GRSAGSIPGEHSTE---NNK---------TFPS	40
NXG89109.1	1	-IEIFEQPR-QRGM-RFRYKC------E---------GRSAGSIPGEHSTD---NNK---------TFPS	40
KFQ89757.1	1	-IEIFEQPR-QRGM-RFRYKC------E---------GRSAGSIPGEHSTE---NNK---------TFPS	40
NWZ21787.1	1	-IEIFEQPR-QRGM-RFRYKC------E---------GRSAGSIPGEHSTE---NNK---------TFPS	40
NXI74048.1	1	-IEIFEQPR-QRGM-RFRYKC------E---------GRSAGSIPGEHSTE---NNK---------TFPS	40
NWX76247.1	1	-IEIFEQPR-QRGM-RFRYKC------E---------GRSAGSIPGEHSTD---NNK---------TFPS	40
NXF29788.1	1	-IEIFEQPR-QRGM-RFRYKC------E---------GRSAGSIPGEHSTE---NNK---------TFPS	40

```
_KAF1444142.1_      1  -IEIFEQPR-QRGM-RFRYKC------E---------GRSAGSIPGEHSTE---NNK---------TFPS  40
_KFV20215.1_        1  -IEIFEQPR-QRGM-RFRYKC------E---------GRSAGSIPGEHSTD---NNK---------TFPS  40
_NXI44657.1_        1  -IEIFEQPR-QRGM-RFRYKC------E---------GRSAGSIPGEHSTE---NNK---------TFPS  40
_NXJ92321.1_        1  -IEIFEQPR-QRGM-RFRYKC------E---------GRSAGSIPGEHSTD---NNK---------TFPS  40
_NWZ49902.1_        1  -IEIFEQPR-QRGM-RFRYKC------E---------GRSAGSIPGEHSTE---NNK---------TFPS  40
_NWY59444.1_        1  -IEIFEQPR-QRGM-RFRYKC------E---------GRSAGSIPGEHSTD---NNK---------TFPS  40
_KFW64019.1_        1  -IEIFEQPR-QRGM-RFRYKC------E---------GRSAGSIPGEHSTE---NNK---------TFPS  40
_NXJ55634.1_        1  -IEIFEQPR-QRGM-RFRYKC------E---------GRSAGSIPGEHSTE---NNK---------TFPS  40
_KAF1513372.1_      1  -IEIFEQPR-QRGM-RFRYKC------E---------GRSAGSIPGEHSTE---NNK---------TFPS  40
_NXW14598.1_        1  -IEIFEQPR-QRGM-RFRYKC------E---------GRSAGSIPGEHSTE---NNK---------TFPS  40
_NXC74247.1_        1  -IEMFEQPR-QRGM-RFRYKC------E---------GRSAGSIPGEHSTD---NNK---------TFPS  40
_NXT57254.1_        1  -IEIFEQPR-QRGM-RFRYKC------E---------GRSAGSIPGEHSTD---NNK---------TFPS  40
_XP_047916679.1_    1  -IEIFEQPR-QRGM-RFRYKC------E---------GRSAGSIPGEHSTE---NNK---------TFPS  40
_XP_029879190.1_    1  -IEIFEQPR-QRGM-RFRYKC------E---------GRSAGSIPGEHSTE---NNK---------TFPS  40
_NXH09241.1_        1  -IEIFEQPR-QRGM-RFRYKC------E---------GRSAGSIPGEHSTD---NNK---------TFPS  40
_XP_040407374.1_    1  -IEIFEQPR-QRGM-RFRYKC------E---------GRSAGSIPGEHSTE---NNK---------TFPS  40
_KFW76037.1_        1  -IEMFEQPR-QRGM-RFRYKC------E---------GRSAGSIPGEHSTE---NNK---------TFPS  40
_KFP65945.1_        1  -IEIFEQPR-QRGM-RFRYKC------E---------GRSAGSIPGEHSTE---NNK---------TFPS  40
_NXX55682.1_        1  -IEMFEQPR-QRGM-RFRYKC------E---------GRSAGSIPGEHSTE---NNK---------TFPS  40
_XP_035178333.1_    1  -IEIFEQPR-QRGM-RFRYKC------E---------GRSAGSIPGEHSTE---NNK---------TFPS  40
_NXE47317.1_        1  -IEIFEQPR-QRGM-RFRYKC------E---------GRSAGSIPGEHSTE---NNK---------TFPS  40
_XP_035408877.1_    1  -IEIFEQPR-QRGM-RFRYKC------E---------GRSAGSIPGEHSTE---NNK---------TFPS  40
_KGL94141.1_        1  -IEIFEQPR-QRGM-RFRYKC------E---------GRSAGSIPGEHSTE---NNK---------TFPS  40
_NXN68403.1_        1  -IEIFEQPR-QRGM-RFRYKC------E---------GRSAGSIPGEHSTE---NNK---------TFPS  40
_NXW28331.1_        1  -IEIFEQPR-QRGM-RFRYKC------E---------GRSAGSIPGEHSTD---NNK---------TFPS  40
_XP_049657509.1_    1  -IEIFEQPR-QRGM-RFRYKC------E---------GRSAGSIPGEHSTE---NNK---------TFPS  40
_XP_049657511.1_    1  -IEIFEQPR-QRGM-RFRYKC------E---------GRSAGSIPGEHSTE---NNK---------TFPS  40
_NXG30098.1_        1  -IEIFEQPR-QRGM-RFRYKC------E---------GRSAGSIPGEHSTE---NNK---------TFPS  40
_NXV86645.1_        1  -IEIFEQPR-QRGM-RFRYKC------E---------GRSAGSIPGEHSTE---NNK---------TFPS  40
_NXX83200.1_        1  -IEIFEQPR-QRGM-RFRYKC------E---------GRSAGSIPGEHSTE---NNK---------TFPS  40
_NXI59898.1_        1  -IEIFEQPR-QRGM-RFRYKC------E---------GRSAGSIPGEHSTE---NNK---------TFPS  40
_NWU89521.1_        1  -IEIFEQPR-QRGM-RFRYKC------E---------GRSAGSIPGEHSTE---NNK---------TFPS  40
_NXK45490.1_        1  -IEIFEQPR-QRGM-RFRYKC------E---------GRSAGSIPGEHSTE---NNK---------TFPS  40
_NWR55599.1_        1  -IEIFEQPR-QRGM-RFRYKC------E---------GRSAGSIPGEHSTE---NNK---------TFPS  40
_NXL37824.1_        1  -IEIFEQPR-QRGM-RFRYKC------E---------GRSAGSIPGEHSTE---NNK---------TFPS  40
_NWI33553.1_        1  -IEMFEQPR-QRGM-RFRYKC------E---------GRSAGSIPGEHSTE---NNK---------TFPS  40
_KFQ74517.1_        1  -IEIFEQPR-QRGM-RFRYKC------E---------GRSAGSIPGEHSTE---NNK---------TFPS  40
_NXV82662.1_        1  -IEIFEQPR-QRGM-RFRYKC------E---------GRSAGSIPGEHSTE---NNK---------TFPS  40
_NWJ10087.1_        1  -IEIFEQPR-QRGM-RFRYKC------E---------GRSAGSIPGEHSTD---NNK---------TFPS  40
```

```
_NXN24483.1_         1  -IEIFEQPR-QRGM-RFRYKC------E---------GRSAGSIPGEHSTE---NSK---------TFPS   40
_NWR80208.1_         1  -VEIFEQPR-QRGM-RFRYKC------E---------GRSAGSIPGEHSTE---NNK---------TFPS   40
_NXT37538.1_         1  -IEIFEQPR-QRGM-RFRYKC------E---------GRSAGSIPGEHSTE---NNK---------TFPS   40
_XP_032844994.2_     1  -IEIFEQPR-QRGM-RFRYKC------E---------GRSAGSIPGEHSTE---NNK---------TFPS   40
_NXW47649.1_         1  -IEIFEQPR-QRGM-RFRYKC------E---------GRSAGSIPGEHSTE---NNK---------TFPS   40
_NXA40398.1_         1  -IEIFEQPR-QRGM-RFRYKC------E---------GRSAGSIPGEHSTD---NNK---------TFPS   40
_NXW64893.1_         1  -IEIFEQPR-QRGM-RFRYKC------E---------GRSAGSIPGEHSTE---NNK---------TFPS   40
_NXX89165.1_         1  -VEIFEQPR-QRGM-RFRYKC------E---------GRSAGSIPGEHSTE---NNK---------TFPS   40
_NXQ96911.1_         1  -IEIFEQPR-QRGM-RFRYKC------E---------GRSAGSIPGEHSTE---NNK---------TFPS   40
_KFP18273.1_         1  -IEMFEQPR-QRGM-RFRYKC------E---------GRSAGSIPGEHSTE---NNK---------TFPS   40
_NXC40882.1_         1  -IEIFEQPR-QRGM-RFRYKC------E---------GRSAGSIPGEHSTE---NNK---------TFPS   40
_NXE10305.1_         1  -IEIFEQPR-QRGM-RFRYKC------E---------GRSAGSIPGEHSTE---NNK---------TFPS   40
_KFP88087.1_         1  -IEIFEQPR-QRGM-RFRYKC------E---------GRSAGSIPGEHSTE---NNK---------TFPS   40
_NWW47871.1_         1  -IEIFEQPR-QRGM-RFRYKC------E---------GRSAGSIPGEHSTE---NNK---------TFPS   40
_NXJ61595.1_         1  -IEIFEQPR-QRGM-RFRYKC------E---------GRSAGSIPGEHSTE---NNK---------TFPS   40
_NXD13814.1_         1  -IEIFEQPR-QRGM-RFRYKC------E---------GRSAGSIPGEHSTD---NNK---------TFPS   40
_KFR16961.1_         1  -VEIFEQPR-QRGM-RFRYKC------E---------GRSAGSIPGEHSTE---NNK---------TFPS   40
_NWX24312.1_         1  -IEIFEQPR-QRGM-RFRYKC------E---------GRSAGSIPGEHSTE---NNK---------TFPS   40
_NXA28267.1_         1  -IEIFEQPR-QRGM-RFRYKC------E---------GRSAGSIPGEHSTE---NNK---------TFPS   40
_NXL66270.1_         1  -IEIFEQPR-QRGM-RFRYKC------E---------GRSAGSIPGEHSTE---NNK---------TFPS   40
_NXA49976.1_         1  -IEIFEQPR-QRGM-RFRYKC------E---------GRSAGSIPGEHSTD---NNK---------TFPS   40
_NXT77624.1_         1  -IEIFEQPR-QRGM-RFRYKC------E---------GRSAGSIPGEHSTE---NNK---------TFPS   40
_KFQ92336.1_         1  -IEMFEQPR-QRGM-RFRYKC------E---------GRSAGSIPGEHSTE---NNK---------TFPS   40
_NXE25013.1_         1  -IEIFEQPR-QRGM-RFRYKC------E---------GRSAGSIPGEHSTE---NNK---------TFPS   40
_NXE92240.1_         1  -IEIFEQPR-QRGM-RFRYKC------E---------GRSAGSIPGEHSTE---NNK---------TFPS   40
_NWH80943.1_         1  -IEIFEQPR-QRGM-RFRYKC------E---------GRSAGSIPGEHSTE---NNK---------TFPS   40
_NWI19058.1_         1  -IEIFEQPR-QRGM-RFRYKC------E---------GRSAGSIPGEHSTD---NNK---------TFPS   40
_NXG80610.1_         1  -IEIFEQPR-QRGM-RFRYKC------E---------GRSAGSIPGEHSTE---NNK---------TFPS   40
_NWR01486.1_         1  -IEIFEQPR-QRGM-RFRYKC------E---------GRSAGSIPGEHSTE---NNK---------TFPS   40
_NXK81337.1_         1  -IEIFEQPR-QRGM-RFRYKC------E---------GRSAGSIPGEHSTE---NNK---------TFPS   40
_NXS90563.1_         1  -IEIFEQPR-QRGM-RFRYKC------E---------GRSAGSIPGEHSTE---NNK---------TFPS   40
_NXC17430.1_         1  -IEIFEQPR-QRGM-RFRYKC------E---------GRSAGSIPGEHSTE---NNK---------TFPS   40
_NXE77245.1_         1  -IEMFEQPR-QRGM-RFRYKC------E---------GRSAGSIPGEHSTE---NNK---------TFPS   40
_KFQ29788.1_         1  -IEIFEQPR-QRGM-RFRYKC------E---------GRSAGSIPGEHSTE---NNK---------TFPS   40
_KFO89327.1_         1  -IEIFEQPR-QRGM-RFRYKC------E---------GRSAGSIPGEHSTE---NNK---------TFPS   40
_NXD64381.1_         1  -IEIFEQPR-QRGM-RFRYKC------E---------GRSAGSIPGEHSTE---SNK---------TFPS   40
_NXY45081.1_         1  -IEIFEQPR-QRGM-RFRYKC------E---------GRSAGSIPGEHSTE---NNK---------TFPS   40
_KGL72871.1_         1  -IEIFEQPR-QRGM-RFRYKC------E---------GRSAGSIPGEHSTD---NNK---------TFPS   40
_XP_053919504.1_     1  -IEIFEQPR-QRGM-RFRYKC------E---------GRSAGSIPGEHSTE---NNK---------TFPS   40
```

```
_NXL85459.1_        1  -IEIFEQPR-QRGM-RFRYKC------E---------GRSAGSIPGEHSTE---NNK---------TFPS   40
_NXO02254.1_        1  -IEIFEQPR-QRGM-RFRYKC------E---------GRSAGSIPGEHSTD---NNK---------TFPS   40
_NXJ76034.1_        1  -IEIFEQPR-QRGM-RFRYKC------E---------GRSAGSIPGEHSTE---NNK---------TFPS   40
_NXM93491.1_        1  -IEIFEQPR-QRGM-RFRYKC------E---------GRSAGSIPGEHSTE---NNK---------TFPS   40
_XP_030345800.1_    1  -IEIFEQPR-QRGM-RFRYKC------E---------GRSAGSIPGEHSTE---NNK---------TFPS   40
_XP_030302544.1_    1  -IEIFEQPR-QRGM-RFRYKC------E---------GRSAGSIPGEHSTE---NNK---------TFPS   40
_XP_030345801.1_    1  -IEIFEQPR-QRGM-RFRYKC------E---------GRSAGSIPGEHSTE---NNK---------TFPS   40
_KQK77724.1_        1  -IEIFEQPR-QRGM-RFRYKC------E---------GRSAGSIPGEHSTE---NNK---------TFPS   40
_NXP09913.1_        1  -IEIFEQPR-QRGM-RFRYKC------E---------GRSAGSIPGEHSTD---NNK---------TFPS   40
_NXR33018.1_        1  -IEIFEQPR-QRGM-RFRYKC------E---------GRSAGSIPGEHSTE---NNK---------TFPS   40
_NXM76881.1_        1  -IEIFEQPR-QRGM-RFRYKC------E---------GRSAGSIPGEHSTE---NNK---------TFPS   40
_NXS27423.1_        1  -IEIFEQPR-QRGM-RFRYKC------E---------GRSAGSIPGEHSTE---NNK---------TFPS   40
_NXM55821.1_        1  -IEIFEQPR-QRGM-RFRYKC------E---------GRSAGSIPGEHSTE---NNK---------TFPS   40
_XP_054678500.1_    1  -IEIFEQPR-QRGM-RFRYKC------E---------GRSAGSIPGEHSTE---NNK---------TFPS   40
_XP_015712248.1_    1  -IEIFEQPR-QRGM-RFRYKC------E---------GRSAGSIPGEHSTD---NNK---------TFPS   40
_NWX92058.1_        1  -IEIFEQPR-QRGM-RFRYKC------E---------GRSAGSIPGEHSTD---NNK---------TFPS   40
_XP_048793646.1_    1  -IEIFEQPR-QRGM-RFRYKC------E---------GRSAGSIPGEHSTD---NNK---------TFPS   40
_NXF04247.1_        1  -IEIFEQPR-QRGM-RFRYKC------E---------GRSAGSIPGEHSTE---NNK---------TFPS   40
_XP_042683331.1_    1  -IEIFEQPR-QRGM-RFRYKC------E---------GRSAGSIPGEHSTD---NNK---------TFPS   40
_NWV22147.1_        1  -IEIFEQPR-QRGM-RFRYKC------E---------GRSAGSIPGEHSTE---NNK---------TFPS   40
_NXA04361.1_        1  -IEIFEQPR-QRGM-RFRYKC------E---------GRSAGSIPGEHSTE---NNK---------TFPS   40
_XP_010116198.1_    1  -IEIFEQPR-QRGM-RFRYKCXXXXXXX---------XXXXGSIPGEHSTE---NNK---------TFPS   46
_NXG59741.1_        1  -IEIFEQPR-QRGM-RFRYKC------E---------GRSAGSIPGEHSTE---NNK---------TFPS   40
_XP_042735326.1_    1  -IEIFEQPR-QRGM-RFRYKC------E---------GRSAGSIPGEHSTD---NNK---------TFPS   40
_NXM29886.1_        1  -IEIFEQPR-QRGM-RFRYKC------E---------GRSAGSIPGEHSTE---NNK---------TFPS   40
_NP_001161198.2_    1  -IEIFEQPR-QRGM-RFRYKC------E---------GRSAGSIPGEHSTD---NNK---------TFPS   40
_XP_031451029.1_    1  -IEIFEQPR-QRGM-RFRYKC------E---------GRSAGSIPGEHSTD---NNK---------TFPS   40
_NXR49262.1_        1  -IEIFEQPR-QRGM-RFRYKC------E---------GRSAGSIPGEHSTE---NNK---------TFPS   40
_XP_052555416.1_    1  -IEIFEQPR-QRGM-RFRYKC------E---------GRSAGSIPGEHSTD---NNK---------TFPS   40
_NWZ67085.1_        1  -IEIFEQPR-QRGM-RFRYKC------E---------GRSAGSIPGEHSTE---NNK---------TFPS   40
_P16236.2_          1  -IEIFEQPR-QRGM-RFRYKC------E---------GRSAGSIPGEHSTD---NNK---------TFPS   40
_XP_050566010.1_    1  -IEIFEQPR-QRGM-RFRYKC------E---------GRSAGSIPGEHSTE---NNK---------TFPS   40
_NWS67030.1_        1  -IEIFEQPR-QRGM-RFRYKC------E---------GRSAGSIPGEHSTD---NNK---------TFPS   40
_XP_005143850.3_    1  -IEIFEQPR-QRGM-RFRYKC------E---------GRSAGSIPGEHSTE---NNK---------TFPS   40
_XP_050566011.1_    1  -IEIFEQPR-QRGM-RFRYKC------E---------GRSAGSIPGEHSTE---NNK---------TFPS   40
_XP_050566009.1_    1  -IEIFEQPR-QRGM-RFRYKC------E---------GRSAGSIPGEHSTE---NNK---------TFPS   40
_NWR30963.1_        1  -IEIFEQPR-QRGM-RFRYKC------E---------GRSAGSIPGEHSTE---NNK---------TFPS   40
_NWS17896.1_        1  -IEIFEQPR-QRGM-RFRYKC------E---------GRSAGSIPGEHSTE---NNK---------TFPS   40
_XP_021248574.1_    1  -IEIFEQPR-QRGM-RFRYKC------E---------GRSAGSIPGEHSTD---NNK---------TFPS   40
```

```
_AAA48721.1_        1  -IEIFEQPR-QRGM-RFRYKC------E---------GRSAGSIPGEHSTD---NNK---------TFPS   40
_NWI54208.1_        1  -IEIFEQPR-QRGM-RFRYKC------E---------GRSAGSIPGEHSTE---NNK---------TFPS   40
_XP_027509532.1_    1  -IEIFEQPR-QRGM-RFRYKC------E---------GRSAGSIPGEHSTE---NNK---------TFPS   40
_NXP71979.1_        1  -IEIFEQPR-QRGM-RFRYKC------E---------GRSAGSIPGEHSTE---NNK---------TFPS   40
_NXX25352.1_        1  -IEIFEQPR-QRGM-RFRYKC------E---------GRSAGSIPGEHSTE---NNK---------TFPS   40
_NXI18618.1_        1  -IEIFEQPR-QRGM-RFRYKC------E---------GRSAGSIPGEHSTE---NNK---------TFPS   40
_NXS09623.1_        1  -IEIFEQPR-QRGM-RFRYKC------E---------GRSAGSIPGEHSTD---NNK---------TFPS   40
_NXU77980.1_        1  -IEIFEQPR-QRGM-RFRYKC------E---------GRSAGSIPGEHSTE---NNK---------TFPS   40
_XP_027509533.1_    1  -IEIFEQPR-QRGM-RFRYKC------E---------GRSAGSIPGEHSTE---NNK---------TFPS   40
_NWV71036.1_        1  -IEIFEQPR-QRGM-RFRYKC------E---------GRSAGSIPGEHSTE---NNK---------TFPS   40
_NXF89482.1_        1  -IEIFEQPR-QRGM-RFRYKC------E---------GRSAGSIPGEHSTE---NNK---------TFPS   40
_NWY39470.1_        1  -IEIFEQPR-QRGM-RFRYKC------E---------GRSAGSIPGEHSTE---NNK---------TFPS   40
_NXT59960.1_        1  -IEIFEQPR-QRGM-RFRYKC------E---------GRSAGSIPGEHSTE---NNK---------TFPS   40
_NWU74340.1_        1  -IEIFEQPR-QRGM-RFRYKC------E---------GRSAGSIPGEHSTE---NNK---------TFPS   40
_NXB84481.1_        1  -IEIFEQPR-QRGM-RFRYKC------E---------GRSAGSIPGEHSTE---NNK---------TFPS   40
_NWV73885.1_        1  -IEIFEQPR-QRGM-RFRYKC------E---------GRSAGSIPGEHSTE---NNK---------TFPS   40
_OPJ58894.1_        1  -IEIFEQPR-QRGM-RFRYKC------E---------GRSAGSIPGEHSTE---NNK---------TFPS   40
_XP_048152104.1_    1  -IEIFEQPR-QRGM-RFRYKC------E---------GRSAGSIPGEHSTE---NNK---------TFPS   40
_XP_054036037.1_    1  -IEIFEQPR-QRGM-RFRYKC------E---------GRSAGSIPGEHSTE---NNK---------TFPS   40
_NXR18149.1_        1  -IEIFEQPR-QRGM-RFRYKC------E---------GRSAGSIPGEHSTE---NNK---------TFPS   40
_NXP97533.1_        1  -IEIFEQPR-QRGM-RFRYKC------E---------GRSAGSIPGEHSTE---NNK---------TFPS   40
_NXJ05495.1_        1  -IEIFEQPR-QRGM-RFRYKC------E---------GRSAGSIPGEHSTD---NNK---------TFPS   40
_XP_032538932.1_    1  -IEIFEQPR-QRGM-RFRYKC------E---------GRSAGSIPGEHSTE---NNK---------TFPS   40
_NWI96387.1_        1  -IEIFEQPR-QRGM-RFRYKC------E---------GRSAGSIPGEHSTE---NNK---------TFPS   40
_NXR65995.1_        1  -IEIFEQPR-QRGM-RFRYKC------E---------GRSAGSIPGEHSTE---NNK---------TFPS   40
_NXK42398.1_        1  -IEIFEQPR-QRGM-RFRYKC------E---------GRSAGSIPGEHSTE---NNK---------TFPS   40
_XP_032538931.1_    1  -IEIFEQPR-QRGM-RFRYKC------E---------GRSAGSIPGEHSTE---NNK---------TFPS   40
_NXY33181.1_        1  -IEIFEQPR-QRGM-RFRYKC------E---------GRSAGSIPGEHSTE---NNK---------TFPS   40
_XP_050171009.1_    1  -IEIFEQPR-QRGM-RFRYKC------E---------GRSAGSIPGEHSTE---NNK---------TFPS   40
_NXO50939.1_        1  -IEIFEQPR-QRGM-RFRYKC------E---------GRSAGSIPGEHSTE---NSK---------TFPS   40
_NWZ32937.1_        1  -IEIFEQPR-QRGM-RFRYKC------E---------GRSAGSIPGEHSTE---NNK---------TFPS   40
_NXP01828.1_        1  -IEIFEQPR-QRGM-RFRYKC------E---------GRSAGSIPGEHSTE---NNK---------TFPS   40
_NXL00458.1_        1  -IEMFEQPR-QRGM-RFRYKC------E---------GRSAGSIPGEHSTE---NNK---------TFPS   40
_XP_010401310.4_    1  -IEIFEQPR-QRGM-RFRYKC------E---------GRSAGSIPGEHSTE---NNK---------TFPS   40
_NXH62149.1_        1  -IEIFEQPR-QRGM-RFRYKC------E---------GRSAGSIPGEHSTE---NNK---------TFPS   40
_NXG43824.1_        1  -IEIFEQPR-QRGM-RFRYKC------E---------GRSAGSIPGEHSTE---NNK---------TFPS   40
_XP_017677337.1_    1  -IEIFEQPR-QRGM-RFRYKC------E---------GRSAGSIPGEHSTE---NNK---------TFPS   40
_NXU09156.1_        1  -IEIFEQPR-QRGM-RFRYKC------E---------GRSAGSIPGEHSTE---NNK---------TFPS   40
_NXB38645.1_        1  -IEIFEQPR-QRGM-RFRYKC------E---------GRSAGSIPGEHSTE---NNK---------TFPS   40
```

_XP_027594504.1_	1	-IEIFEQPR-QRGM-RFRYKC------E---------GRSAGSIPGEHSTE---NNK---------TFPS	40
NXM46269.1	1	-IEIFEQPR-QRGM-RFRYKC------E---------GRSAGSIPGEHSTE---NNK---------TFPS	40
NWS79257.1	1	-IEIFEQPR-QRGM-RFRYKC------E---------GRSAGSIPGEHSTE---NNK---------TFPS	40
NXB04839.1	1	-IEIFEQPR-QRGM-RFRYKC------E---------GRSAGSIPGEHSTE---NNK---------TFPS	40
NXC62865.1	1	-IEIFEQPR-QRGM-RFRYKC------E---------GRSAGSIPGEHSTE---NNK---------TFPS	40
NXN18277.1	1	-IEIFEQPR-QRGM-RFRYKC------E---------GRSAGSIPGEHSTE---NNK---------TFPS	40
_XP_027760039.1_	1	-IEIFEQPR-QRGM-RFRYKC------E---------GRSAGSIPGEHSTE---NNK---------TFPS	40
NXI90311.1	1	-IEIFEQPR-QRGM-RFRYKC------E---------GRSAGSIPGEHSTE---NNK---------TFPS	40
NWY17003.1	1	-IEIFEQPR-QRGM-RFRYKC------E---------GRSAGSIPGEHSTE---NNK---------TFPS	40
NXH97099.1	1	-IEIFEQPR-QRGM-RFRYKC------E---------GRSAGSIPGEHSTE---NNK---------TFPS	40
NWS97308.1	1	-IEIFEQPR-QRGM-RFRYKC------E---------GRSAGSIPGEHSTE---NNK---------TFPS	40
NWU45160.1	1	-IEIFEQPR-QRGM-RFRYKC------E---------GRSAGSIPGEHSTD---NNK---------TFPS	40
NXQ43004.1	1	-IEIFEQPR-QRGM-RFRYKC------E---------GRSAGSIPGEHSTE---NNK---------TFPS	40
_XP_027549142.1_	1	-IEIFEQPR-QRGM-RFRYKC------E---------GRSAGSIPGEHSTE---NNK---------TFPS	40
_XP_017933082.2_	1	-IEIFEQPR-QRGM-RFRYKC------E---------GRSAGSIPGEHSTE---NNK---------TFPS	40
NWV96326.1	1	-IEIFEQPR-QRGM-RFRYKC------E---------GRSAGSIPGEHSTE---NNK---------TFPS	40
NXP57783.1	1	-IEIFEQPR-QRGM-RFRYKC------E---------GRSAGSIPGEHSTE---NNK---------TFPS	40
NXY57955.1	1	-IEIFEQPR-QRGM-RFRYKC------E---------GRSAGSIPGEHSTE---NNK---------TFPS	40
AAA99433.1	1	-IEIFEQPR-QRGM-RFRYKC------E---------GRSAGSIPGEHSTD---NNK---------TFPS	40
NXY21218.1	1	-IEIFEQPR-QRGM-RFRYKC------E---------GRSAGSIPGEHSTE---NNK---------TFPS	40
NXX50824.1	1	-IEIFEQPR-QRGM-RFRYKC------E---------GRSAGSIPGEHSTE---NNK---------TFPS	40
_XP_030124623.3_	1	-IEIFEQPR-QRGM-RFRYKC------E---------GRSAGSIPGEHSTE---NNK---------TFPS	40
NXA92235.1	1	-IEIFEQPR-QRGM-RFRYKC------E---------GRSAGSIPGEHSTE---NNK---------TFPS	40
NWI05522.1	1	-IEIFEQPR-QRGM-RFRYKC------E---------GRSAGSIPGEHSTE---NNK---------TFPS	40
NWX29009.1	1	-IEIFEQPR-QRGM-RFRYKC------E---------GRSAGSIPGEHSTE---NNK---------TFPS	40
_XP_027549144.1_	1	-IEIFEQPR-QRGM-RFRYKC------E---------GRSAGSIPGEHSTE---NNK---------TFPS	40
NWW35420.1	1	-IEIFEQPR-QRGM-RFRYKC------E---------GRSAGSIPGEHSTE---NNK---------TFPS	40
NWV07969.1	1	-IEIFEQPR-QRGM-RFRYKC------E---------GRSAGSIPGEHSTE---NNK---------TFPS	40
NXH42214.1	1	-IEIFEQPR-QRGM-RFRYKC------E---------GRSAGSIPGEHSTE---NNK---------TFPS	40
NXQ18773.1	1	-IEIFEQPR-QRGM-RFRYKC------E---------GRSAGSIPGEHSTE---NNK---------TFPS	40
NWX10672.1	1	-IEIFEQPR-QRGM-RFRYKC------E---------GRSAGSIPGEHSTE---NNK---------TFPS	40
NXU50687.1	1	-IEIFEQPR-QRGM-RFRYKC------E---------GRSAGSIPGEHSTE---NNK---------TFPS	40
KFU84784.1	1	-IEIFEQPR-QRGM-RFRYKC------E---------GRSAGSIPGEHSTE---NNK---------TFPS	40
NXQ52438.1	1	-IEIFEQPR-QRGM-RFRYKC------E---------GRSAGSIPGEHSTE---NNK---------TFPS	40
NWW24536.1	1	-IEIFEQPR-QRGM-RFRYKC------E---------GRSAGSIPGEHSTE---NNK---------TFPS	40
NXF73449.1	1	-IEIFEQPR-QRGM-RFRYKC------E---------GRSAGSIPGEHSTE---NNK---------TFPS	40
_XP_050827906.1_	1	-IEIFEQPR-QRGM-RFRYKC------E---------GRSAGSIPGEHSTE---NNK---------TFPS	40
NWX65205.1	1	-IEIFEQPR-QRGM-RFRYKC------E---------GRSAGSIPGEHSTE---NNK---------TFPS	40
KFW81749.1	1	-IEIFEQPR-QRGM-RFRYKC------E---------GRSAGSIPGEHSTE---NNK---------TFPS	40

```
_XP_014750593.1_    1  -IEIFEQPR-QRGM-RFRYKC------E---------GRSAGSIPGEHSTE---NNK---------TFPS   40
_NXJ17066.1_        1  -IEIFEQPR-QRGM-RFRYKC------E---------GRSAGSIPGEHSTE---NNK---------TFPS   40
_NWW67019.1_        1  -IEIFEQPR-QRGM-RFRYKC------E---------GRSAGSIPGEHSTE---NNK---------TFPS   40
_NXH03360.1_        1  -IEIFEQPR-QRGM-RFRYKC------E---------GRSAGSIPGEHSTE---NNK---------TFPS   40
_XP_037987525.1_    1  -IEIFEQPR-QRGM-RFRYKC------E---------GRSAGSIPGEHSTE---NNK---------TFPS   40
_NXL74522.1_        1  -IEIFEQPR-QRGM-RFRYKC------E---------GRSAGSIPGEHSTE---NNK---------TFPS   40
_NWQ60824.1_        1  -IEIFEQPR-QRGM-RFRYKC------E---------GRSAGSIPGEHSTE---NNK---------TFPS   40
_NXR70971.1_        1  -IEIFEQPR-QRGM-RFRYKC------E---------GRSAGSIPGERSTE---NNK---------TFPS   40
_XP_051468947.1_    1  -IEIFEQPR-QRGM-RFRYKC------E---------GRSAGSIPGEHSTE---NNK---------TFPS   40
_NXB50199.1_        1  -IEIFEQPR-QRGM-RFRYKC------E---------GRSAGSIPGEHSTE---NNK---------TFPS   40
_NXP18380.1_        1  -IEIFEQPR-QRGM-RFRYKC------E---------GRSAGSIPGEHSTE---NNK---------TFPS   40
_NXI84319.1_        1  -IEIFEQPR-QRGM-RFRYKC------E---------GRSAGSIPGEHSTE---NNK---------TFPS   40
_NWH33757.1_        1  -IEIFEQPR-QRGM-RFRYKC------E---------GRSAGSIPGEHSTE---NNK---------TFPS   40
_NWR99781.1_        1  -IEIFEQPR-QRGM-RFRYKC------E---------GRSAGSIPGEHSTE---NNK---------TFPS   40
_NXW94404.1_        1  -IEIFEQPR-QRGM-RFRYKC------E---------GRSAGSIPGEHSTE---NNK---------TFPS   40
_CAA27220.1_        1  -IEIFEQPR-QRGM-RFRYKC------E---------GRSAGSIPGEHSTD---NNK---------TFPS   40
_XP_015477679.1_    1  -IEIFEQPR-QRGM-RFRYKC------E---------GRSAGSIPGEHSTE---NNK---------TFPS   40
_NWS32597.1_        1  -IEIFEQPR-QRGM-RFRYKC------E---------GRSAGSIPGEHSTE---NNK---------TFPS   40
_NXG22126.1_        1  -IEIFEQPR-QRGM-RFRYKC------E---------GRSAGSIPGEHSTE---NNK---------TFPS   40
_NWY97127.1_        1  -IEIFEQPR-QRGM-RFRYKC------E---------GRSAGSIPGEHSTE---NNK---------TFPS   40
_NXW80148.1_        1  -IEIFEQPR-QRGM-RFRYKC------E---------GRSAGSIPGEHSTE---NNK---------TFPS   40
_NXC27367.1_        1  -IEIFEQPR-QRGM-RFRYKC------E---------GRSAGSIPGEHSTE---NNK---------TFPS   40
_NWH83726.1_        1  -IEIFEQPR-QRGM-RFRYKC------E---------GRSAGSIPGEHSTE---NNK---------TFPS   40
_NXA73512.1_        1  -IEIFEQPR-QRGM-RFRYKC------E---------GRSAGSIPGEHSTE---NNK---------TFPS   40
_NWZ83230.1_        1  -IEIFEQPR-QRGM-RFRYKC------E---------GRSAGSIPGEHSTE---NNK---------TFPS   40
_XP_005525572.2_    1  -IEIFEQPR-QRGM-RFRYKC------E---------GRSAGSIPGEHSTE---NNK---------TFPS   40
_NXD20033.1_        1  -IEIFEQPR-QRGM-RFRYKC------E---------GRSAGSIPGEHSTE---NNK---------TFPS   40
_NXE37177.1_        1  -IEIFEQPR-QRGM-RFRYKC------E---------GRSAGSIPGEHSTE---NNK---------TFPS   40
_NWV44369.1_        1  -IEIFEQPR-QRGM-RFRYKC------E---------GRSAGSIPGEHSTE---NNK---------TFPS   40
_NXC81400.1_        1  -IEIFEQPR-QRGM-RFRYKC------E---------GRSAGSIPGEHSTE---NNK---------TFPS   40
_NXA67345.1_        1  -IEIFEQPR-QRGM-RFRYKC------E---------GRSAGSIPGEHSTD---NNK---------TFPS   40
_NXB55155.1_        1  -IEIFEQPR-QRGM-RFRYKC------E---------GRSAGSIPGEHSTE---NNK---------TFPS   40
_NXN81229.1_        1  -IEIFEQPR-QRGM-RFRYKC------E---------GRSAGSIPGEHSTE---NNK---------TFPS   40
_NXM15601.1_        1  -IEIFEQPR-QRGM-RFRYKC------E---------GRSAGSIPGEHSTE---NNK---------TFPS   40
_XP_054484850.1_    1  -IEIFEQPR-QRGM-RFRYKC------E---------GRSAGSIPGEHSTE---NNK---------TFPS   40
_NWU02685.1_        1  -IEIFEQPR-QRGM-RFRYKC------E---------GRSAGSIPGEHSTE---NNK---------TFPS   40
_XP_041273165.1_    1  -IEIFEQPR-QRGM-RFRYKC------E---------GRSAGSIPGEHSTE---NNK---------TFPS   40
_NWW80397.1_        1  -IEIFEQPR-QRGM-RFRYKC------E---------GRSAGSIPGEHSTE---NNK---------TFPS   40
_XP_036236540.1_    1  -IEIFEQPR-QRGM-RFRYKC------E---------GRSAGSIPGEHSTE---NNK---------TFPS   40
```

```
_NWV50957.1_        1  -IEIFEQPR-QRGM-RFRYKC------E---------GRSAGSIPGEHSTE---NNK---------TFPS   40
_NXT97287.1_        1  -IEIFEQPR-QRGM-RFRYKC------E---------GRSAGSIPGEHSTE---NNK---------TFPS   40
_XP_023778876.1_    1  -IEIFEQPR-QRGM-RFRYKC------E---------GRSAGSIPGEHSTE---NNK---------TFPS   40
_NXD42381.1_        1  -IEIFEQPR-QRGM-RFRYKC------E---------GRSAGSIPGEHSTE---NNK---------TFPS   40
_NXK69295.1_        1  -IEIFEQPR-QRGM-RFRYKC------E---------GRSAGSIPGEHSTE---NNK---------TFPS   40
_XP_049657510.1_    1  -IEIFEQPR-QRGM-RFRYKC------E---------GRSAGSIPGEHSTE---NNK---------TFPS   40
_NXV62539.1_        1  -IEIFEQPR-QRGM-RFRYKC------E---------GRSAGSIPGEHSTE---NNK---------TFPS   40
_NXB80231.1_        1  -IEIFEQPR-QRGM-RFRYKC------E---------GRSAGSIPGEHSTE---NNK---------TFPS   40
_NWU15026.1_        1  -IEIFEQPR-QRGM-RFRYKC------E---------GRSAGSIPGEHSTE---NNK---------TFPS   40
_NXL26104.1_        1  -IEIFEQPR-QRGM-RFRYKC------E---------GRSAGSIPGEHSTE---NNK---------TFPS   40
_NWW05001.1_        1  -IEIFEQPR-QRGM-RFRYKC------E---------GRSAGSIPGEHSTD---NNK---------TFPS   40
_RLW07261.1_        1  -IEIFEQPR-QRGM-RFRYKC------E---------GRSAGSIPGEHSTE---NNK---------TFPS   40
_NXR96625.1_        1  -IEIFEQPR-QRGM-RFRYKC------E---------GRSAGSIPGEHSTE---NNK---------TFPS   40
_XP_021383204.1_    1  -IEIFEQPR-QRGM-RFRYKC------E---------GRSAGSIPGEHSTE---NNK---------TFPS   40
_NXQ25764.1_        1  -IEIFEQPR-QRGM-RFRYKC------E---------GRSAGSIPGEHSTE---NNK---------TFPS   40
_NXO08412.1_        1  -IEIFEQPR-QRGM-RFRYKC------E---------GRSAGSIPGEHSTE---NNK---------TFPS   40
_NXI32280.1_        1  -IEIFEQPR-QRGM-RFRYKC------E---------GRSAGSIPGEHSTE---NNK---------TFPS   40
_NXG00288.1_        1  -IEIFEQPR-QRGM-RFRYKC------E---------GRSAGSIPGEHSTE---NNK---------TFPS   40
_XP_041329879.1_    1  -IEIFEQPR-QRGM-RFRYKC------E---------GRSAGSIPGEHSTE---NNK---------TFPS   40
_NXV03759.1_        1  -IEIFEQPR-QRGM-RFRYKC------E---------GRSAGSIPGEHSTE---NNK---------TFPS   40
_NWT16975.1_        1  -IEIFEQPR-QRGM-RFRYKC------E---------GRSAGSIPGEHSTE---NNK---------TFPS   40
_NWT67953.1_        1  -IEIFEQPR-QRGM-RFRYKC------E---------GRSAGSIPGEHSTE---NNK---------TFPS   40
_XP_005042765.2_    1  -IEIFEQPR-QRGM-RFRYKC------E---------GRSAGSIPGEHSTE---NNK---------TFPS   40
_XP_017600715.1_    1  -IEIFEQPR-QRGM-RFRYKC------E---------GRSAGSIPGEHSTE---NNK---------TFPS   40
_NXD93279.1_        1  -IEIFEQPR-QRGM-RFRYKC------E---------GRSAGSIPGEHSTE---NNK---------TFPS   40
_NXY02260.1_        1  -IEIFEQPR-QRGM-RFRYKC------E---------GRSAGSIPGEHSTE---NSK---------TFPS   40
_NXO84677.1_        1  -IEIFEQPR-QRGM-RFRYKC------E---------GRSAGSIPGEHSTE---NNK---------TFPS   40
_NXO64371.1_        1  -IEIFEQPR-QRGM-RFRYKC------E---------GRSAGSIPGEHSTE---NNK---------TFPS   40
_NXO42022.1_        1  -IEIFEQPR-QRGM-RFRYKC------E---------GRSAGSIPGEHSTE---NNK---------TFPS   40
_NWY65013.1_        1  -IEIFEQPR-QRGM-RFRYKC------E---------GRSAGSIPGEHSTE---NNK---------TFPS   40
_NXP90563.1_        1  -IEIFEQPR-QRGM-RFRYKC------E---------GRSAGSIPGEHSTE---NNK---------TFPS   40
_NWI73723.1_        1  -IEIFEQPR-QRGM-RFRYKC------E---------GRSAGSIPGEHSTE---NNK---------TFPS   40
_NXH30511.1_        1  -IEIFEQPR-QRGM-RFRYKC------E---------GRSAGSIPGEHSTE---NNK---------TFPS   40
_NWT77800.1_        1  -IEIFEQPR-QRGM-RFRYKC------E---------GRSAGSIPGEHSTE---NNK---------TFPS   40
_NWT34209.1_        1  -IEIFEQPR-QRGM-RFRYKC------E---------GRSAGSIPGEHSTE---NNK---------TFPS   40
_NWY29369.1_        1  -IEIFEQPR-QRGM-RFRYKC------E---------GRSAGSIPGEHSTE---NNK---------TFPS   40
_NXQ73821.1_        1  -IEIFEQPR-QRGM-RFRYKC------E---------GRSAGSIPGEHSTE---NNK---------TFPS   40
_XP_039572786.1_    1  -IEIFEQPR-QRGM-RFRYKC------E---------GRSAGSIPGEHSTE---NNK---------TFPS   40
_XP_030802035.1_    1  -IEIFEQPR-QRGM-RFRYKC------E---------GRSAGSIPGEHSTE---NNK---------TFPS   40
```

```
_NXE68211.1_        1  -IEIFEQPR-QRGM-RFRYKC------E---------GRSAGSIPGEHSTE---NNK---------TFPS   40
_NXS80652.1_        1  -IEIFEQPR-QRGM-RFRYKC------E---------GRSAGSIPGEHSTE---NNK---------TFPS   40
_NXO32431.1_        1  -IEIFEQPR-QRGM-RFRYKC------E---------GRSAGSIPGEHSTE---NNK---------TFPS   40
_NWR17317.1_        1  -IEIFEQPR-QRGM-RFRYKC------E---------GRSAGSIPGEHSTE---NNK---------TFPS   40
_NWZ89732.1_        1  -IEIFEQPR-QRGM-RFRYKC------E---------GRSAGSIPGEHSTE---NNK---------TFPS   40
_XP_032039523.1_    1  -IEIFEQPR-QRGM-RFRYKC------E---------GRSAGSIPGEHSTE---NNK---------TFPS   40
_KFO04362.1_        1  -IEIFEQPR-QRGM-RFRYKC------E---------GRSAGSIPGEHSTE---NNK---------TFPS   40
_XP_054146213.1_    1  -IEIFEQPR-QRGM-RFRYKC------E---------GRSAGSIPGERSTE---NNK---------SFPS   40
_XP_005486916.2_    1  -IEIFEQPR-QRGM-RFRYKC------E---------GRSAGSIPGERSTE---NNK---------SFPS   40
_KAJ7422946.1_      1  -IEIFEQPR-QRGM-RFRYKC------E---------GRSAGSIPGEHSTE---NNK---------TFPS   40
_NXC99388.1_        1  -IEIFEQPR-QRGM-RFRYKC------E---------GRSAGSIPGEHSTE---NNK---------TFPS   40
_RMC07975.1_        1  -IEIFEQPR-QRGM-RFRYKC------E---------GRSAGSIPGEHSTE---NNK---------TFPS   40
_NXT95159.1_           ----------------------------------------------------------------------
_XP_009957589.1_       ----------------------------------------------------------------------
_TRZ13602.1_           ----------------------------------------------------------------------
_NWR45911.1_           ----------------------------------------------------------------------
_KAF4799649.1_         ----------------------------------------------------------------------
_KAJ7420138.1_      1  -IEIFEQPR-QRGM-RFRYKC------E---------GRSAGSIPGEHSTE---NNK---------TFPS   40
_XP_054678501.1_    1  -IEIFEQPR-QRGM-RFRYKC------E---------GRSAGSIPGEHSTE---NNK---------TFPS   40
_NXP48685.1_        1  -IEIFEQPR-QRGM-RFRYKC------E---------GRSAGSIPGEHSTE---NNK---------TFPS   40
_XP_010704701.1_       ----------------------------------------------------------------------
_KFV10805.1_           ----------------------------------------------------------------------
_XP_009572567.1_       ----------------------------------------------------------------------
_NXF64277.1_           ----------------------------------------------------------------------
_KFV54465.1_           ----------------------------------------------------------------------
_KFV90717.1_           ----------------------------------------------------------------------
_KFQ03674.1_           ----------------------------------------------------------------------
_NXW01028.1_           ----------------------------------------------------------------------
_KFP32908.1_           ----------------------------------------------------------------------
_KFV93389.1_           ----------------------------------------------------------------------
_NWI63728.1_           ----------------------------------------------------------------------
_KAI6072063.1_         ----------------------------------------------------------------------
_KFQ58901.1_           ----------------------------------------------------------------------
_NXP33753.1_           ----------------------------------------------------------------------
_XP_009476353.1_       ----------------------------------------------------------------------
_KFQ55903.1_           ----------------------------------------------------------------------
_XP_042683349.1_       ----------------------------------------------------------------------
_NXR08991.1_           ----------------------------------------------------------------------
_XP_008947250.1_       ----------------------------------------------------------------------
```

```
_KFP72464.1_             ----------------------------------------------------------------------
_KFV60523.1_             ----------------------------------------------------------------------
_NXT13165.1_             ----------------------------------------------------------------------
_NWT50506.1_             ----------------------------------------------------------------------
_OXB76139.1_             ----------------------------------------------------------------------
_XP_050827907.1_         ----------------------------------------------------------------------
_XP_030302545.1_         ----------------------------------------------------------------------
_XP_054036038.1_         ----------------------------------------------------------------------
_XP_012788337.1_      1  -IEIIEQPR-QRGL-RFRYEC------E---------GRSAGSIPGEHSTE---NNR---------TYPS   40
_XP_008070154.1_      1  -------------M-RFRYKC------E---------GRSAGSIPGEHSTD---NNR---------TYPS   29
_XP_027477445.1_      1  -------------M-RFRYKC------E---------GRSAGSIPGEHSTE---NNR---------TYPS   29
_XP_025848473.1_      1  -------------M-RFRYKC------E---------GRSAGSIPGEHSTD---NNR---------TYPS   29
_XP_049640716.1_      1  -IEIIEQPR-QRGL-RFRYEC------E---------GRSAGSIPGEHSTE---NNR---------TYPS   40
_XP_023084089.1_      1  -IEIIEQPR-QRGM-RFRYKC------E---------GRSAGSIPGEHSTD---NNR---------TYPS   40
_EHH22143.1_          1  -IEIIEQPR-QRGM-RFRYKC------E---------GRSAGSIPGEHSTD---NNR---------TYPS   40
_XP_038504711.1_      1  -IEIIEQPR-QRGM-RFRYKC------E---------GRSAGSIPGEHSTD---NNR---------TYPS   40
_XP_038504703.1_      1  -IEIIEQPR-QRGM-RFRYKC------E---------GRSAGSIPGEHSTD---NNR---------TYPS   40
_XP_038504718.1_      1  -IEIIEQPR-QRGM-RFRYKC------E---------GRSAGSIPGEHSTD---NNR---------TYPS   40
_XP_048971856.1_      1  -IEIIEQPR-QRGM-RFRYKC------E---------GRSAGSIPGEHSTD---NNR---------TYPS   40
_KAB1266826.1_        1  -IEIIEQPR-QRGM-RFRYKC------E---------GRSAGSIPGEHSTD---NNR---------TYPS   40
_XP_048391424.1_      1  -VEIVEQPK-QRGM-RFRYKC------E---------GRSAGSIPGEHSTD---NNR---------TYPS   40
_XP_048391425.1_      1  -VEIVEQPK-QRGM-RFRYKC------E---------GRSAGSIPGEHSTD---NNR---------TYPS   40
_GCC28026.1_          1  -VEIVEQPK-QRGM-RFRYKC------E---------GRSAGSIPGENSTD---NNR---------TYPS   40
_XP_043551991.1_      1  -VEIVEQPK-QRGM-RFRYKC------E---------GRSAGSIPGKNSTD---NNR---------TYPS   40
_XP_043551989.1_      1  -VEIVEQPK-QRGM-RFRYKC------E---------GRSAGSIPGKNSTD---NNR---------TYPS   40
_XP_054534389.1_         ----------------------------------------------------------------------
_XP_011531312.1_         ----------------------------------------------------------------------
_XP_021780541.1_         ----------------------------------------------------------------------
_XP_037862912.1_         ----------------------------------------------------------------------
_XP_015413428.1_      1  -------------M-RFRYKC------E---------GRSAGSIPGEHSTD---NNR---------TYPS   29
_XP_005884642.1_      1  -------------M-RFRYKC------E---------GRSAGSIPGEHSTD---NNR---------TYPS   29
_KAF6105903.1_        1  -------------M-RFRYKC------E---------GRSAGSIPGEHSTD---NNR---------TYPS   29
_XP_015413426.1_      1  -------------M-RFRYKC------E---------GRSAGSIPGEHSTD---NNR---------TYPS   29
_XP_014387539.1_      1  -------------M-RFRYKC------E---------GRSAGSIPGEHSTD---NNR---------TYPS   29
_XP_014444723.1_      1  -------------M-RFRYKC------E---------GRSAGSIPGEHSTE---NNR---------TYPS   29
_XP_023448058.1_      1  -------------M-RFRYKC------E---------GRSAGSIPGEHSTD---NNR---------TYPS   29
_EGW11604.1_          1  -------------M-RFRYKC------E---------GRSAGSIPGEHSTD---NNR---------TYPS   29
_XP_015985129.1_      1  -------------M-RFRYKC------E---------GRSAGSIPGEHSTE---NNR---------TYPS   29
_XP_012878014.1_      1  -------------M-RFRYKC------E---------GRSAGSIPGEHSTD---NNQ---------TYPS   29
```

```
_KAG5211506.1_      1  -----------------------------------------REIEGEE---------------------T   8
_MXQ82539.1_        1  -----------------------------------------F----CP---------------------V   4
_XP_032729748.1_    1  -IEIIEQPR-QRGM-RFRYKC------E---------GRSAGSIPGEHSTD---NNR---------TYPS  40
_XP_032208113.1_    1  -IEIIEQPR-QRGM-RFRYKC------E---------GRSAGSIPGEHSTD---NNR---------TYPS  40
_XP_048208817.1_    1  -IEIIEQPR-QRGM-RFRYKC------E---------GRSAGSIPGEHSTD---SNQ---------TYPS  40
_XP_038200333.1_    1  -VEIIEQPR-QRGM-RFRYKC------E---------GRSAGSIPGEHSTD---NNR---------TYPS  40
_P15307.2_          1  -VEIIEQPR-QRGM-RFRYKC------E---------GRSAGSIPGERSTD---NNR---------TYPS  40
_XP_041524635.1_    1  -------------M-RFRYKC------E---------GRSAGSIPGEHSTD---NNR---------TYPS  29
_CAA33843.1_        1  -VEIIEQPR-QRGM-RFRYKC------E---------GRSAGSIPGERSTD---NNR---------TYPS  40
_XP_028732210.2_    1  -------------M-RFRYKC------E---------GRSAGSIPGEHSTD---NNR---------TYPS  29
_XP_025848474.1_    1  -------------M-RFRYKC------E---------GRSAGSIPGEHSTD---NNR---------TYPS  29
_EDL97996.1_        1  -------------M-RFRYKC------E---------GRSAGSIPGERSTD---NNR---------TYPS  29
_KAI4577886.1_      1  -------------M-RFRYKC------E---------GRSAGSIPGEHSTD---NNR---------TYPS  29
_KAG8512309.1_      1  -IEIFEQPR-QRGM-RFRYKC------E---------GRSAGSIPGERSTD---NNR---------TYPS  40
_XP_047637971.1_    1  -IEITEQPR-QRGM-RFRYKC------E---------GRSAGSIPGEHSTD---SSR---------TYPS  40
_XP_019491454.1_    1  -IEIIEQPR-QRGM-RFRYKC------E---------GRSAGSIPGEHSTD---NNR---------TYPS  40
_XP_043842482.1_    1  -VEIIEQPR-QRGM-RFRYKC------E---------GRSAGSIPGEHSSD---NNR---------TYPS  40
_XP_027721170.1_    1  -VEIIEQPR-QRGM-RFRYKC------E---------GRSAGSIPGEHSTD---NNR---------TYPS  40
_XP_036604632.1_    1  -VEIIEQPR-QRGM-RFRYKC------E---------GRSAGSIPGEHSTD---NNR---------TYPS  40
_XP_020841164.1_    1  -VEIIEQPR-QRGM-RFRYKC------E---------GRSAGSIPGEHSTD---NNR---------TYPS  40
_XP_051831295.1_    1  -VEIIEQPR-QRGM-RFRYKC------E---------GRSAGSIPGEHSTD---NNR---------TYPS  40
_XP_031806378.1_    1  -VEIIEQPR-QRGM-RFRYKC------E---------GRSAGSIPGEHSTD---NNR---------TYPS  40
_XP_051831294.1_    1  -VEIIEQPR-QRGM-RFRYKC------E---------GRSAGSIPGEHSTD---NNR---------TYPS  40
_XP_003339776.1_    1  -VQIIEQPR-QRGM-RFRYKC------E---------GRSAGSIPGEHSTD---NNR---------TYPS  40
_XP_044515413.1_    1  -VQIIEQPR-QRGM-RFRYKC------E---------GRSAGSIPGEHSTD---NNR---------TYPS  40
_XP_028928361.1_    1  -VEIIEQPR-QRGM-RFRYKC------E---------GRSAGSIPGEHSTD---SNR---------TYPS  40
_XP_038607403.1_    1  -VEIIEQPR-QRGM-RFRYKC------E---------GRSAGSIPGEHSTD---NNR---------TYPS  40
_XP_040849334.1_       ----------------------------------------------------------------------
_TKC46270.1_           ----------------------------------------------------------------------
_OWK10634.1_           ----------------------------------------------------------------------
_XP_036190342.1_       ----------------------------------------------------------------------
_KAB0395596.1_         ----------------------------------------------------------------------
_KAF6430170.1_         ----------------------------------------------------------------------
_XP_038948811.1_       ----------------------------------------------------------------------
_KAB1266828.1_      1  -IEIIEQPR-QRGM-RFRYKC------E---------GRSAGSIPGEHSTD---NNR---------TYPS  40
_NP_002899.1_       1  -IEIIEQPR-QRGM-RFRYKC------E---------GRSAGSIPGEHSTD---NNR---------TYPS  40
_XP_001159537.3_    1  -IEIIEQPR-QRGM-RFRYKC------E---------GRSAGSIPGEHSTD---NNR---------TYPS  40
_PNI66333.1_        1  -IEIIEQPR-QRGM-RFRYKC------E---------GRSAGSIPGEHSTD---NNR---------TYPS  40
_EHH55592.1_        1  -IEIIEQPR-QRGM-RFRYKC------E---------GRSAGSIPGEHSTD---NNR---------TYPS  40
```

_XP_003262470.1_	1	-IEIIEQPR-QRGM-RFRYKC------E---------GRSAGSIPGEHSTD---NNR---------TYPS	40
BAG64854.1	1	-IEIIEQPR-QRGM-RFRYKC------E---------GRSAGSIPGEHSTD---NNR---------TYPS	40
_XP_032034471.1_	1	-IEIIEQPR-QRGM-RFRYKC------E---------GRSAGSIPGEHSTD---NNR---------TYPS	40
_XP_037862911.1_	1	-IEIIEQPR-QRGM-RFRYKC------E---------GRSAGSIPGEHSTD---NNR---------TYPS	40
_XP_003830911.1_	1	-IEIIEQPR-QRGM-RFRYKC------E---------GRSAGSIPGEHSTD---NNR---------TYPS	40
_XP_004029331.1_	1	-VEIIEQPR-QRGM-RFRYKC------E---------GRSAGSIPGEHSTD---NNR---------TYPS	40
_XP_002812051.1_	1	-IEIIEQPR-QRGM-RFRYKC------E---------GRSAGSIPGEHSTD---NNR---------TYPS	40
_XP_011711623.1_	1	-IEIIEQPR-QRGM-RFRYKC------E---------GRSAGSIPGEHSTD---NNR---------TYPS	40
_XP_033095023.1_	1	-IEIIEQPR-QRGM-RFRYKC------E---------GRSAGSIPGEHSTD---NNR---------TYPS	40
_XP_002799280.1_	1	-IEIIEQPR-QRGM-RFRYKC------E---------GRSAGSIPGEHSTD---NNR---------TYPS	40
_XP_011853172.1_	1	-IEIIEQPR-QRGM-RFRYKC------E---------GRSAGSIPGEHSTD---NNR---------TYPS	40
_XP_050609443.1_	1	-IEIIEQPR-QRGM-RFRYKC------E---------GRSAGSIPGEHSTD---NNR---------TYPS	40
_XP_003908735.1_	1	-IEIIEQPR-QRGM-RFRYKC------E---------GRSAGSIPGEHSTD---NNR---------TYPS	40
_XP_025209693.1_	1	-IEIIEQPR-QRGM-RFRYKC------E---------GRSAGSIPGEHSTD---NNR---------TYPS	40
_XP_023084087.1_	1	-IEIIEQPR-QRGM-RFRYKC------E---------GRSAGSIPGEHSTD---NNR---------TYPS	40
_XP_021780539.1_	1	-IEIIEQPR-QRGM-RFRYKC------E---------GRSAGSIPGEHSTD---NNR---------TYPS	40
_XP_017704568.1_	1	-IEIIEQPR-QRGM-RFRYKC------E---------GRSAGSIPGEHSTD---NNR---------TYPS	40
_XP_010375811.1_	1	-IEIIEQPR-QRGM-RFRYKC------E---------GRSAGSIPGEHSTD---NNR---------TYPS	40
_XP_030776399.1_	1	-IEIIEQPR-QRGM-RFRYKC------E---------GRSAGSIPGEHSTD---NNR---------TYPS	40
_XP_011797211.1_	1	-IEIIEQPR-QRGM-RFRYKC------E---------GRSAGSIPGEHSTD---NNR---------TYPS	40
_XP_012332682.1_	1	-IEIIEQPR-QRGM-RFRYKC------E---------GRSAGSIPGEHSTD---NNR---------TYPS	40
_XP_010331875.2_	1	-IEIIEQPR-QRGM-RFRYKC------E---------GRSAGSIPGEHSTD---NNR---------TYPS	40
_XP_012332683.1_	1	-IEIIEQPR-QRGM-RFRYKC------E---------GRSAGSIPGEHSTD---NNR---------TYPS	40
_XP_017400475.1_	1	-IEIIEQPR-QRGM-RFRYKC------E---------GRSAGSIPGEHSTD---NNR---------TYPS	40
_XP_008568357.1_	1	-IEIIEQPR-QRGM-RFRYKC------E---------GRSAGSIPGEHSTD---NNR---------TYPS	40
_XP_012620284.1_	1	-IEITEQPR-QRGM-RFRYKC------E---------GRSAGSIPGEHSTD---NSR---------TYPS	40
_XP_002709741.2_	1	-IEIIEQPR-QRGM-RFRYKC------E---------GRSAGSIPGEHSTD---NNR---------TYPS	40
_XP_022452943.1_	1	-IEIIEQPR-QRGM-RFRYKC------E---------GRSAGSIPGEHSTD---NNR---------TYPS	40
_XP_045405625.1_	1	-IEITEQPR-QRGM-RFRYKC------E---------GRSAGSIPGEHSTD---NSR---------TYPS	40
_XP_004280707.1_	1	-IEIIEQPR-QRGM-RFRYKC------E---------GRSAGSIPGEHSTD---NNR---------TYPS	40
_XP_030733169.1_	1	-IEIIEQPR-QRGM-RFRYKC------E---------GRSAGSIPGEHSTD---NNR---------TYPS	40
TEA23022.1	1	-IEIIEQPR-QRGM-RFRYKC------E---------GRSAGSIPGEHSTD---NNR---------TYPS	40
_XP_012494629.1_	1	-IEITEQPR-QRGM-RFRYKC------E---------GRSAGSIPGEHSTD---NSR---------TYPS	40
_XP_010950191.1_	1	-IEIIEQPR-QRGM-RFRYKC------E---------GRSAGSIPGEHSTD---NNR---------TYPS	40
_XP_024608785.1_	1	-IEIIEQPR-QRGM-RFRYKC------E---------GRSAGSIPGEHSTD---NNR---------TYPS	40
_XP_006201637.1_	1	-IEIIEQPR-QRGM-RFRYKC------E---------GRSAGSIPGEHSTD---NNR---------TYPS	40
_XP_019285354.1_	1	-IEITEQPR-QRGM-RFRYKC------E---------GRSAGSIPGEHSTD---NNR---------TYPS	40
_XP_032509224.1_	1	-IEIIEQPR-QRGM-RFRYKC------E---------GRSAGSIPGEHSTD---NNR---------TYPS	40
_XP_047379355.1_	1	-IKIIEQPR-QRGM-RFRYKC------E---------GRSAGSIPGERSTD---NNR---------TYPS	40

```
_XP_032981134.1_    1  -IEIIEQPR-QRGM-RFRYKC------E---------GRSAGSIPGEHSTD---NNR---------TYPS   40
_XP_005600018.1_    1  -IEIMEQPR-QRGM-RFRYKC------E---------GRSAGSIPGEHSTD---NNR---------TYPS   40
_XP_046517051.1_    1  -IEIMEQPR-QRGM-RFRYKC------E---------GRSAGSIPGEHSTD---NNR---------TYPS   40
_XP_007464205.1_    1  -IAIIEQPR-QRGM-RFRYKC------E---------GRSAGSIPGEHSTD---NNR---------TYPS   40
_XP_019572903.1_    1  -IEIIEQPR-QRGM-RFRYKC------E---------GRSAGSIPGEHSTD---NNR---------TYPS   40
_XP_007189755.1_    1  -IEIIEQPR-QRGM-RFRYKC------E---------GRSAGSIPGEHSTD---NNR---------TYPS   40
_XP_004397771.1_    1  -IEIIEQPR-QRGM-RFRYKC------E---------GRSAGSIPGEHSTE---NNR---------TYPS   40
_XP_004435713.1_    1  -IEIIEQPR-QRGM-RFRYKC------E---------GRSAGSIPGEHSTD---NNR---------TYPS   40
_XP_008698124.1_    1  -IEIIEQPR-QRGM-RFRYKC------E---------GRSAGSIPGEHSTD---NNR---------TYPS   40
_XP_023989255.1_    1  -IEIIEQPR-QRGM-RFRYKC------E---------GRSAGSIPGEHSTD---NNR---------TYPS   40
_XP_011233012.2_    1  -IEIIEQPR-QRGM-RFRYKC------E---------GRSAGSIPGEHSTD---NNR---------TYPS   40
_XP_025724444.1_    1  -IEIIEQPR-QRGM-RFRYKC------E---------GRSAGSIPGEHSTE---NNR---------TYPS   40
_XP_005626186.1_    1  -IEIIEQPR-QRGM-RFRYKC------E---------GRSAGSIPGEHSTD---NNR---------TYPS   40
_XP_045643991.1_    1  -IEIIEQPR-QRGM-RFRYKC------E---------GRSAGSIPGEHSTD---NNR---------TYPS   40
_XP_034857383.1_    1  -IEIIEQPR-QRGM-RFRYKC------E---------GRSAGSIPGEHSTE---NNR---------TYPS   40
_XP_027945501.1_    1  -IEIIEQPR-QRGM-RFRYKC------E---------GRSAGSIPGEHSTE---NNR---------TYPS   40
_XP_026341094.1_    1  -IEIIEQPR-QRGM-RFRYKC------E---------GRSAGSIPGEHSTD---NNR---------TYPS   40
_EFB29923.1_        1  -IEIIEQPR-QRGM-RFRYKC------E---------GRSAGSIPGEHSTD---NNR---------TYPS   40
_XP_045740897.1_    1  -IEIIEQPR-QRGM-RFRYKC------E---------GRSAGSIPGEHSTE---NNR---------TYPS   40
_CAD7677454.1_      1  -IEIIEQPR-QRGM-RFRYKC------E---------GRSAGSIPGEHSTD---NNR---------TYPS   40
_XP_035577553.1_    1  -IEIIEQPR-QRGM-RFRYKC------E---------GRSAGSIPGEHSTD---NNR---------TYPS   40
_XP_006733255.1_    1  -IEIIEQPR-QRGM-RFRYKC------E---------GRSAGSIPGEHSTE---NNR---------TYPS   40
_XP_032266820.1_    1  -IEIIEQPR-QRGM-RFRYKC------E---------GRSAGSIPGEHSTE---NNR---------TYPS   40
_XP_041611730.1_    1  -IEIIEQPR-QRGM-RFRYKC------E---------GRSAGSIPGEHSTD---NNR---------TYPS   40
_XP_048971854.1_    1  -IEIIEQPR-QRGM-RFRYKC------E---------GRSAGSIPGEHSTD---NNR---------TYPS   40
_XP_021554840.1_    1  -IEIIEQPR-QRGM-RFRYKC------E---------GRSAGSIPGEHSTE---NNR---------TYPS   40
_VCW78562.1_        1  -IEIIEQPR-QRGM-RFRYKC------E---------GRSAGSIPGEHSTD---NNR---------TYPS   40
_XP_034857381.1_    1  -IEIIEQPR-QRGM-RFRYKC------E---------GRSAGSIPGEHSTE---NNR---------TYPS   40
_XP_008524231.1_    1  ----MEQPR-QRGM-RFRYKC------E---------GRSAGSIPGEHSTD---NNR---------TYPS   37
_XP_045740895.1_    1  -IEIIEQPR-QRGM-RFRYKC------E---------GRSAGSIPGEHSTE---NNR---------TYPS   40
_XP_045834746.1_    1  -IEIIEQPR-QRGM-RFRYKC------E---------GRSAGSIPGEHSTD---NNR---------TYPS   40
_XP_035932906.1_    1  -IEIIEQPR-QRGM-RFRYKC------E---------GRSAGSIPGEHSTE---NNR---------TYPS   40
_XP_030883859.1_    1  -IEIIEQPR-QRGM-RFRYKC------E---------GRSAGSIPGEHSTE---NNR---------TYPS   40
_XP_032266818.1_    1  -IEIIEQPR-QRGM-RFRYKC------E---------GRSAGSIPGEHSTE---NNR---------TYPS   40
_XP_024408528.2_    1  -IEIIEQPR-QRGM-RFRYKC------E---------GRSAGSIPGEHSTD---NNR---------TYPS   40
_XP_004580347.1_    1  -IEIIEQPR-QRGM-RFRYKC------E---------GRSAGSIPGEHSTD---NNR---------TYPS   40
_XP_030166525.1_    1  -IEIIEQPR-QRGM-RFRYKC------E---------GRSAGSIPGEHSTD---NNR---------TYPS   40
_XP_035932904.1_    1  -IEIIEQPR-QRGM-RFRYKC------E---------GRSAGSIPGEHSTE---NNR---------TYPS   40
_XP_020040690.1_    1  -IEIIEQPR-QRGM-RFRYKC------E---------GRSAGSIPGEHSTD---NNR---------TYPS   40
```

```
_XP_053445604.1_    1  -IEIIEQPR-QRGM-RFRYKC------E---------GRSAGSIPGEHSTD---NSR---------TYPS   40
_XP_049507482.1_    1  -IEITEQPR-QRGM-RFRYKC------E---------GRSAGSIPGEHSTD---NNR---------TYPS   40
_XP_015335127.1_    1  -IKIIEQPR-QRGM-RFRYKC------E---------GRSAGSIPGEHSTE---NNR---------TFPS   40
_XP_027790245.1_    1  -IKIIEQPR-QRGM-RFRYKC------E---------GRSAGSIPGEHSTE---NNR---------TFPS   40
_XP_003984077.1_    1  -IEIIEQPR-QRGM-RFRYKC------E---------GRSAGSIPGEHSTD---NNR---------TYPS   40
_XP_039096259.1_    1  -IEIIEQPR-QRGM-RFRYKC------E---------GRSAGSIPGEHSTD---NNR---------TYPS   40
_XP_007075434.1_    1  -IEITEQPR-QRGM-RFRYKC------E---------GRSAGSIPGEHSTD---NNR---------TYPS   40
_XP_022360130.1_    1  -IEIIEQPR-QRGM-RFRYKC------E---------GRSAGSIPGEHSTD---NNR---------TYPS   40
_XP_047707378.1_    1  -IEIIEQPR-QRGM-RFRYKC------E---------GRSAGSIPGEHSTD---NNR---------TYPS   40
_XP_026243031.1_    1  -IKIIEQPR-QRGM-RFRYKC------E---------GRSAGSIPGEHSTE---NNR---------TFPS   40
_XP_037383340.1_    1  -IEIFEQPR-QRGM-RFRYKC------E---------GRSAGSIPGERSTE---NNR---------TYPS   40
_XP_005322095.1_    1  -IKIIEQPR-QRGM-RFRYKC------E---------GRSAGSIPGEHSTE---NNR---------TFPS   40
_XP_005662584.1_    1  -IEITEQPR-QRGM-RFRYKC------E---------GRSAGSIPGEHSTD---SSR---------TYPS   40
_XP_040080101.1_    1  -IEIIEQPR-QRGM-RFRYKC------E---------GRSAGSIPGEHSTD---NNR---------TYPS   40
_XP_017503999.1_    1  -IEIAEQPR-QRGM-RFRYKC------E---------GRSAGSIPGEHSTD---NNR---------TYPS   40
_XP_044119954.1_    1  -IEIIEQPR-QRGM-RFRYKC------E---------GRSAGSIPGEHSTD---NNR---------TYPS   40
_XP_032208110.1_    1  -IEIIEQPR-QRGM-RFRYKC------E---------GRSAGSIPGEHSTD---NNR---------TYPS   40
_XP_012586936.1_    1  -IEIFEQPR-QRGM-RFRYKC------E---------GRSAGSIPGERSTD---NNR---------TYPS   40
_XP_032729745.1_    1  -IEIIEQPR-QRGM-RFRYKC------E---------GRSAGSIPGEHSTD---NNR---------TYPS   40
_XP_052504199.1_    1  -IEIIEQPR-QRGM-RFRYKC------E---------GRSAGSIPGEHSTD---NNR---------TYPS   40
_XP_054431389.1_    1  -IEIIEQPR-QRGM-RFRYKC------E---------GRSAGSIPGEHSTD---NNR---------TYPS   40
_XP_023576632.1_    1  -IEIVEQPR-QRGM-RFRYKC------E---------GRSAGSIPGEHSTE---DNR---------TYPS   40
_XP_004742070.1_    1  -IEIIEQPR-QRGM-RFRYKC------E---------GRSAGSIPGEHSTD---NNR---------TYPS   40
_XP_037663267.1_    1  -IEITEQPR-QRGM-RFRYKC------E---------GRSAGSIPGEHSTD---NNR---------TYPS   40
_XP_004005978.1_    1  -IEIIEQPR-QRGM-RFRYKC------E---------GRSAGSIPGEHSTD---NNR---------TYPS   40
_XP_036781908.1_    1  -IEIAEQPR-QRGM-RFRYKC------E---------GRSAGSIPGEHSTD---NNR---------TYPS   40
_XP_040849333.1_    1  -IEIIEQPR-QRGM-RFRYKC------E---------GRSAGSIPGEHSTD---NNR---------TYPS   40
_XP_005686691.1_    1  -IEIIEQPR-QRGM-RFRYKC------E---------GRSAGSIPGEHSTD---NNR---------TYPS   40
_XP_023367900.1_    1  -IEIIEQPR-QRGM-RFRYKC------E---------GRSAGSIPGEHSTD---NNR---------TYPS   40
_KAF0879576.1_      1  -IEIIEQPR-QRGM-RFRYKC------E---------GRSAGSIPGEHSTD---NNR---------TYPS   40
_XP_029793284.1_    1  -IEIIEQPR-QRGM-RFRYKC------E---------GRSAGSIPGEHSTD---NNR---------TYPS   40
_NP_001179899.1_    1  -IEIIEQPR-QRGM-RFRYKC------E---------GRSAGSIPGEHSTD---NNR---------TYPS   40
_XP_020764718.1_    1  -IEIIEQPR-QRGM-RFRYKC------E---------GRSAGSIPGEHSTD---NNR---------TYPS   40
_XP_054431388.1_    1  -IEIIEQPR-QRGM-RFRYKC------E---------GRSAGSIPGEHSTD---NNR---------TYPS   40
_XP_037663265.1_    1  -IEITEQPR-QRGM-RFRYKC------E---------GRSAGSIPGEHSTD---NNR---------TYPS   40
_XP_019825763.1_    1  -IEIIEQPR-QRGM-RFRYKC------E---------GRSAGSIPGEHSTD---NNR---------TYPS   40
_XP_005889096.1_    1  -IEIIEQPR-QRGM-RFRYKC------E---------GRSAGSIPGEHSTD---NNR---------TYPS   40
_XP_043324571.1_    1  -IEIIEQPR-QRGM-RFRYKC------E---------GRSAGSIPGEHSTD---NNR---------TYPS   40
_KAJ1078272.1_      1  -IEIIEQPR-QRGM-RFRYKC------E---------GRSAGSIPGEHSTD---NNR---------TYPS   40
```

```
_XP_036190339.1_    1  -IEIIEQPR-QRGM-RFRYKC------E---------GRSAGSIPGEHSTD---NNR---------TYPS   40
_XP_013005522.1_    1  -VEIVEQPR-QRGM-RFRYKC------E---------GRSAGSIPGEHSTE---DNR---------TYPS   40
_XP_006073936.1_    1  -IEIIEQPR-QRGM-RFRYKC------E---------GRSAGSIPGEHSTD---NNR---------TYPS   40
_XP_016069529.1_    1  -IEIIEQPR-QRGM-RFRYKC------E---------GRSAGSIPGEHSTD---NNR---------TYPS   40
_KAB0354136.1_      1  -IEIIEQPR-QRGM-RFRYKC------E---------GRSAGSIPGEHSTD---NNR---------TYPS   40
_XP_029424871.1_    1  -IEITEQPR-QRGM-RFRYKC------E---------GRSAGSIPGEHSTD---NNR---------TYPS   40
_XP_036118792.1_    1  -IEIIEQPR-QRGM-RFRYKC------E---------GRSAGSIPGEHSTE---NNR---------TYPS   40
_XP_037019725.2_    1  -V-ISEQPR-QRGM-RFRYKC------E---------GRSAGSIPGEHSTD---NNR---------TYPS   39
_XP_047602572.1_    1  -IEIIEQPR-QRGM-RFRYKC------E---------GRSAGSIPGEHSTD---NNR---------TYPS   40
_XP_014586639.1_    1  -IEIMEQPR-QRGM-RFRYKC------E---------GRSAGSIPGEHSTD---NNR---------TYPS   40
_XP_046517052.1_    1  -IEIMEQPR-QRGM-RFRYKC------E---------GRSAGSIPGEHSTD---NNR---------TYPS   40
_XP_006909635.2_    1  -IEITEQPR-QRGM-RFRYKC------E---------GRSAGSIPGEHSTD---NNR---------TYPS   40
_XP_011362222.1_    1  -IEITEQPR-QRGM-RFRYKC------E---------GRSAGSIPGEHSTD---NNR---------TYPS   40
_XP_045684409.1_    1  -IEIIEQPR-QRGM-RFRYKC------E---------GRSAGSIPGEHSTD---NNR---------TYPS   40
_XP_048208816.1_    1  -IEIIEQPR-QRGM-RFRYKC------E---------GRSAGSIPGEHSTD---SNQ---------TYPS   40
_XP_052590629.1_    1  -VEIIEQPR-QRGM-RFRYKC------E---------GRSAGSIPGEHSTD---NNR---------TYPS   40
_XP_032353499.1_    1  -IEIIEQPR-QRGM-RFRYKC------E---------GRSAGSIPGEHSTD---NNR---------TYPS   40
_XP_038200327.1_    1  -VEIIEQPR-QRGM-RFRYKC------E---------GRSAGSIPGEHSTD---NNR---------TYPS   40
_XP_051056550.1_    1  -VEIIEQPR-QRGM-RFRYKC------E---------GRSAGSIPGEHSAD---NNR---------TYPS   40
_XP_012805850.2_    1  -IEIIEQPR-QRGM-RFRYKC------E---------GRSAGSIPGEHSTE---NNR---------TYPS   40
_XP_016050906.1_    1  -IEIIEQPR-QRGM-RFRYKC------E---------GRSAGSIPGEHSTE---NNR---------TYPS   40
_XP_030166526.1_    1  -IEIIEQPR-QRGM-RFRYKC------E---------GRSAGSIPGEHSTD---NNR---------TYPS   40
_XP_036903878.1_    1  -V-ISEQPR-QRGM-RFRYKC------E---------GRSAGSIPGEHSTD---NNR---------TYPS   39
_CAH7004032.1_      1  -VEIIEQPR-QRGM-RFRYKC------E---------GRSAGSIPGEHSAD---NNR---------TYPS   40
_XP_051056549.1_    1  -VEIIEQPR-QRGM-RFRYKC------E---------GRSAGSIPGEHSAD---NNR---------TYPS   40
_XP_042547166.1_    1  -IEIIEQPR-QRGM-RFRYKC------E---------GRSAGSIPGEHSTD---NNQ---------TYPS   40
_XP_054584456.1_    1  -IEIIEQPR-QRGM-RFRYKC------E---------GRSAGSIPGEHSTD---NNR---------TYPS   40
_XP_049996768.1_    1  -VEIIEQPR-QRGM-RFRYKC------E---------GRSAGSIPGEHSTD---NNR---------TYPS   40
_XP_027243805.1_    1  -VEIIEQPR-QRGM-RFRYKC------E---------GRSAGSIPGEHSTD---NNR---------TYPS   40
_XP_047707379.1_    1  -IEIIEQPR-QRGM-RFRYKC------E---------GRSAGSIPGEHSTD---NNR---------TYPS   40
_XP_026645370.1_    1  -VEIIEQPR-QRGM-RFRYKC------E---------GRSAGSIPGEHSTD---NNR---------TYPS   40
_XP_014302519.1_    1  -IEIIEQPR-QRGM-RFRYKC------E---------GRSAGSIPGEHSTD---NNR---------TYPS   40
_XP_049726303.1_    1  -IEIIEQPR-QRGM-RFRYKC------E---------GRSAGSIPGEHSTD---SNR---------TYPS   40
_XP_049726302.1_    1  -IEIIEQPR-QRGM-RFRYKC------E---------GRSAGSIPGEHSTD---SNR---------TYPS   40
_XP_012969026.1_    1  -VEIIEQPR-QRGM-RFRYKC------E---------GRSAGSIPGEHSTD---NNR---------TYPS   40
_XP_008524232.1_    1  ----MEQPR-QRGM-RFRYKC------E---------GRSAGSIPGEHSTD---NNR---------TYPS   37
_XP_051020904.1_    1  -VEIIEQPR-QRGM-RFRYKC------E---------GRSAGSIPGEHSTD---NNR---------TYPS   40
_XP_023590712.1_    1  -IEIIEQPR-QRGM-RFRYKC------E---------GRSAGSIPGEHSTD---NNR---------TYPS   40
_XP_027243803.1_    1  -VEIIEQPR-QRGM-RFRYKC------E---------GRSAGSIPGEHSTD---NNR---------TYPS   40
```

_XP_040589151.1_	1	-VEIIEQPR-QRGM-RFRYKC------E---------GRSAGSIPGEHSTD---NNR---------TYPS	40
_XP_023412959.1_	1	-IEIIEQPR-QRGM-RFRYKC------E---------GRSAGSIPGEHSTD---SNR---------TYPS	40
_XP_036057111.1_	1	-VEIIEQPR-QRGM-RFRYKC------E---------GRSAGSIPGEHSTD---NNR---------TYPS	40
_XP_027822602.1_	1	-IEIIEQPR-QRGM-RFRYKC------E---------GRSAGSIPGEHSTD---NNR---------TYPS	40
_XP_031322949.1_	1	-IEIIEQPR-QRGM-RFRYKC------E---------GRSAGSIPGEHSTD---NNR---------TYPS	40
_XP_028634966.1_	1	-VEIIEQPR-QRGM-RFRYKC------E---------GRSAGSIPGERSTD---NNR---------TYPS	40
_XP_031531529.1_	1	-IEIIEQPR-QRGM-RFRYKC------E---------GRSAGSIPGEHSTD---NNR---------TYPS	40
_XP_006073937.1_	1	-IEIIEQPR-QRGM-RFRYKC------E---------GRSAGSIPGEHSTD---NNR---------TYPS	40
_XP_034364887.1_	1	-VEIIEQPR-QRGM-RFRYKC------E---------GRSAGSIPGERSTD---NNR---------TYPS	40
_XP_021482246.1_	1	-VEITEQPR-QRGM-RFRYKC------E---------GRSAGSIPGERSTD---NSR---------TYPS	40
KAF4016326.1	1	-IEIIEQPR-QRGM-RFRYKC------E---------GRSAGSIPGEHSTD---NNR---------TYPS	40
_XP_023602319.1_	1	-IEIIEQPR-QRGM-RFRYKC------E---------GRSAGSIPGEHSTD---NNR---------TYPS	40
_XP_052054164.1_	1	-VEIIEQPR-QRGM-RFRYKC------E---------GRSAGSIPGERSTD---NNR---------TYPS	40
_XP_036291310.1_	1	-IQIIEQPR-QRGM-RFRYKC------E---------GRSAGSIPGEHSTD---NNR---------TYPS	40
_XP_013370168.1_	1	-VEIVEQPR-QRGM-RFRYKC------E---------GRSAGSIPGEHSTE---DNR---------TYPS	40
_XP_023590722.1_	1	-IEIIEQPR-QRGM-RFRYKC------E---------GRSAGSIPGEHSTD---NNR---------TYPS	40
_XP_048291328.1_	1	-VEIIEQPR-QRGM-RFRYKC------E---------GRSAGSIPGEHSTD---NNR---------TYPS	40
_XP_032772910.1_	1	-VEIIEQPR-QRGM-RFRYKC------E---------GRSAGSIPGERSTD---NNR---------TYPS	40
_XP_008768670.2_	1	-VEIIEQPR-QRGM-RFRYKC------E---------GRSAGSIPGERSTD---NNR---------TYPS	40
_XP_014302521.1_	1	-IEIIEQPR-QRGM-RFRYKC------E---------GRSAGSIPGEHSTD---NNR---------TYPS	40
_XP_031194882.1_	1	-VEIIEQPR-QRGM-RFRYKC------E---------GRSAGSIPGERSTD---NNR---------TYPS	40
_NP_033070.2_	1	-VEIIEQPR-QRGM-RFRYKC------E---------GRSAGSIPGERSTD---NNR---------TYPS	40
_XP_036291312.1_	1	-IQIIEQPR-QRGM-RFRYKC------E---------GRSAGSIPGEHSTD---NNR---------TYPS	40
_XP_021033157.1_	1	-VEIIEQPR-QRGM-RFRYKC------E---------GRSAGSIPGERSTD---NNR---------TYPS	40
_XP_015849136.1_	1	-VEIIEQPR-QRGM-RFRYKC------E---------GRSAGSIPGEHSTD---NNR---------TYPS	40
_XP_045150027.1_	1	-IEIIEQPR-QRGM-RFRYKC------E---------GRSAGSIPGEHSTD---NNR---------TYPS	40
_XP_021066684.1_	1	-VEIIEQPR-QRGM-RFRYKC------E---------GRSAGSIPGERSTD---NNR---------TYPS	40
_XP_006881088.1_	1	-VKIIEQPR-QRGM-RFRYKC------E---------GRSAGSIPGEHSTD---SNR---------TYPS	40
_XP_008524234.1_	1	----MEQPR-QRGM-RFRYKC------E---------GRSAGSIPGEHSTD---NNR---------TYPS	37
_XP_021482260.1_	1	-VEITEQPR-QRGM-RFRYKC------E---------GRSAGSIPGERSTD---NSR---------TYPS	40
_XP_007125500.1_	1	-IEIIEQPR-QRGM-RFRYKC------E---------GRSAGSIPGEHSTD---NNR---------TYPS	40
_XP_028352731.1_	1	-IEIIEQPR-QRGM-RFRYKC------E---------GRSAGSIPGEHSTD---NNR---------TYPS	40
_XP_028352733.1_	1	-IEIIEQPR-QRGM-RFRYKC------E---------GRSAGSIPGEHSTD---NNR---------TYPS	40
_XP_028352734.1_	1	-IEIIEQPR-QRGM-RFRYKC------E---------GRSAGSIPGEHSTD---NNR---------TYPS	40
_XP_028352736.1_	1	-IEIIEQPR-QRGM-RFRYKC------E---------GRSAGSIPGEHSTD---NNR---------TYPS	40
_XP_028352735.1_	1	-IEIIEQPR-QRGM-RFRYKC------E---------GRSAGSIPGEHSTD---NNR---------TYPS	40
ERE88482.1	1	-VEIIEQPR-QRGM-RFRYKC------E---------GRSAGSIPGEHSTD---NNR---------TYPS	40
KAF3826750.1		--	
OBS70420.1	1	-------------M-RFRYKC------E---------GRSAGSIPGEHSTD---NNR---------TYPS	29

```
_XP_006878094.1_     1  -------------------------------------------------------------------FPF   3
_XP_008978905.3_        ----------------------------------------------------------------------
_XP_032147941.1_        ----------------------------------------------------------------------
_XP_008524233.1_        ----------------------------------------------------------------------
_XP_044628438.1_        ----------------------------------------------------------------------
_XP_010608101.1_        ----------------------------------------------------------------------
_KAF5918007.1_          ----------------------------------------------------------------------
_XP_021101942.1_        ----------------------------------------------------------------------
_ABM89308.1_            ----------------------------------------------------------------------
_MBW00678.1_            ----------------------------------------------------------------------
_NP_001096177.1_     1  -IEIFEQPR-QRGM-RYRYKC------E---------GRCAGSILGETSTE---NNR---------TYPS  40
_OCT77375.1_         1  -IEIFEQPR-QRGM-RYRYKC------E---------GRSAGSILGERSTE---NNR---------TYPS  40
_AAI70187.1_         1  -IEIFEQPR-QRGM-RYRYKC------E---------GRSAGSILGERSTE---NNR---------TYPS  40
_AAI69362.1_         1  -IEIFEQPR-QRGM-RYRYKC------E---------GRCAGSIHGEHSTE---NNR---------TYPS  40
_XP_041418586.1_     1  -IEIFEQPR-QRGM-RYRYKC------E---------GRCAGSIHGEHSTE---NNR---------TYPS  40
_XP_041418585.1_     1  -IEIFEQPR-QRGM-RYRYKC------E---------GRCAGSIHGEHSTE---NNR---------TYPS  40
_XP_041418587.1_     1  -IEIFEQPR-QRGM-RYRYKC------E---------GRCAGSIHGEHSTE---NNR---------TYPS  40
_XP_053316221.1_     1  -IEIFEQPR-QRGM-RFRYKC------E---------GRLAGSILGEHSTD---NNR---------IYPS  40
_XP_053568018.1_     1  -IEIFEQPR-QRGM-RFRYKC------E---------GRSAGSILGERSTE---NNR---------SYPS  40
_KAG8443975.1_       1  -IEIFEQPR-QRGM-RFRYKC------E---------GRSAGSILGERSTD---NNR---------TYPA  40
_XP_044145067.1_     1  -IEIFEQPR-QRGM-RFRYKC------E---------GRSAGSILGERSSE---NNR---------SYPS  40
_KAG9484268.1_       1  -IEIFEQPR-QRGM-RFRYKC------E---------GRSAGSILGERSSE---NNR---------TYPS  40
_XP_040285660.1_     1  -MEIFEQPR-QRGM-RFRYKC------E---------GRSAGSILGERSSE---NNR---------SYPS  40
_XP_040285659.1_     1  -MEIFEQPR-QRGM-RFRYKC------E---------GRSAGSILGERSSE---NNR---------SYPS  40
_KAG8583825.1_       1  -IEIFEQPR-QRGM-RFRYKC------E---------GRSAGSILGERSSE---NNR---------SYPS  40
_XP_018411072.1_     1  -IEIFEQPR-QRGM-RFRYKC------E---------GRSAGSILGERSTE---NNR---------SYPS  40
_XP_040207073.1_     1  -IEIFEQPR-QRGM-RFRYKC------E---------GRSAGSILGERSTE---NNR---------SYPS  40
_NP_001081750.1_     1  -IEIFEQPR-QRGM-RYRYKC------E---------GRSAGSILGEQPLR--TTGH---------THPS  41
_PIO39092.1_            ----------------------------------------------------------------------
_PIO39091.1_            ----------------------------------------------------------------------
_XP_041035877.1_     1  -IRILEQPK-QRGM-RFRYKC------E---------GRSAGSIPGEKSTD---STR---------TYPA  40
_XP_042202691.1_     1  -VRIMEQPK-QRGM-RFRYKC------E---------GRSAGSILGEKSTD---TTR---------TYPT  40
_XP_048475509.1_     1  -------------M-RFRYKC------E---------GRSAGSIPGEKSTD---STR---------TYPA  29
_XP_048380947.1_     1  -------------M-RFRYKC------E---------GRSAGSIPGEKSTD---STR---------TYPA  29
_XP_051901152.1_     1  --------K-QRGM-RFRYKC------E---------GRSAGSIPGEKSTD---STR---------TYPA  33
_BAD60879.1_         1  ----------------FRYEC------E---------GRSAGSLPGEHSTN---EQK---------SFPT  27
_XP_050405370.1_     1  -VEIIEQPR-QRGL-RFRYQC------E---------GRSAGSIPGQSSTA---EKK---------TFPT  40
_XP_009063713.1_     1  -VVIIEEPR-QRGL-RFRYQC------E---------GRSAGSIPGQSSSA---EKK---------TYPT  40
_CAB0044847.1_       1  --VIDEQPC-GKVR-YDRRKQ------G---------GQAATPLLGAHSKPGGGEKK---------SYPS  42
```

```
_XP_048259911.1_     1  -MEITEQPQ-FRGL-RFRYEC------E---------GRSAGSIPGEHSTA---ERK---------TFPT   40
_ADI72431.1_         1  -MEITEQPQ-FRGL-RFRYEC------E---------GRSAGSIPGEHSTA---ERK---------TFPT   40
_CAG5133522.1_       1  -VEITEQPK-SRGL-RFRYEC------E---------GRSAGSVPGENSSN---EHR---------TYPT   40
_KAG5688355.1_          ----------------------------------------------------------------------
_AMT84567.1_         1  -VRIVEQPK-ARGQ-RFRYKC------E---------GRSAGSIPGEKSIN---DQR---------TFPT   40
_XP_052276378.1_     1  -VEIVEQPK-ARGL-RFRYEC------E---------GRSAGSIPGESSTP---ERK---------TFPA   40
_XP_052276377.1_     1  -VEIVEQPK-ARGL-RFRYEC------E---------GRSAGSIPGESSTP---ERK---------TFPA   40
_AAK72690.1_         1  -VEIVEQPK-QRGL-RFRYEC------E---------GRSAGSIPGEGSTS---EKK---------TFPT   40
_XP_011414731.2_     1  -VEIVEQPK-QRGL-RFRYEC------E---------GRSAGSIPGEGSTS---EKK---------TFPT   40
_XP_052699807.1_     1  -VEIVEQPK-QRGL-RFRYEC------E---------GRSAGSIPGEGSTS---EKK---------TFPT   40
_NP_001292261.1_     1  -VEIVEQPK-QRGL-RFRYEC------E---------GRSAGSIPGEGSTS---EKK---------TFPT   40
_XP_048738054.1_     1  -VEITEQPK-QRGL-RFRYEC------E---------GRSAGSIPGESSTS---EKK---------TFPT   40
_XP_052699808.1_     1  -VEIVEQPK-QRGL-RFRYEC------E---------GRSAGSIPGEGSTS---EKK---------TFPT   40
_XP_022332457.1_     1  -VEITEQPK-PRGL-RFRYEC------E---------GRSAGSIPGENSTA---EKK---------TFPT   40
_ABL63469.1_         1  --EIMEQPK-QRGL-RFRYEC------E---------GRSAGSIPGESSTN---EKK---------TFPT   39
_XP_033733822.1_     1  -VEIVEQPK-QRGL-RFRYEC------E---------GRSAGSIPGDKSSA---ERK---------TFPT   40
_XP_021347355.1_     1  -VEIVEQPK-QRGL-RFRYEC------E---------GRSAGSIPGDKSTT---ERK---------TFPT   40
_UOA58607.1_         1  -VEIVEQPK-QRGL-RFRYEC------E---------GRSAGSIPGDKSTT---ERK---------TFPT   40
_XP_021347356.1_     1  -VEIVEQPK-QRGL-RFRYEC------E---------GRSAGSIPGDKSTT---ERK---------TFPT   40
_CAG2186872.1_       1  --EIVEQPK-QRGL-RFRYEC------E---------GRSAGSIPGEKSTQ---DRK---------SFPT   39
_XP_052058915.1_     1  --EIVEQPK-QRGL-RFRYEC------E---------GRSAGSIPGEKSTQ---DRK---------SFPT   39
_KAJ8309232.1_       1  -VEITEQPR-QRGL-RFRYEC------E---------GRSAGSIPGEKSTP---ERK---------TFPT   40
_UZH84475.1_         1  -VELVEQPK-SRGL-RFRYEC------E---------GRSAGSIPGERSTT---EKR---------TYPT   40
_ALA99939.1_         1  -VEIVEQPR-ARGL-RFRYEC------E---------GRSAGSIPGENSTP---ERK---------TFPS   40
_XP_052767764.1_     1  -VEIVEQPK-ARGL-RFRYEC------E---------GRSAGSIPGENSTP---ERK---------IFPS   40
_ADD25211.1_         1  -VEIVEQPK-QRGL-RFRYEC------E---------GRSAGSIPGDKSTT---ERK---------TFPT   40
_XP_052224245.1_     1  -VEIVEQPK-ARGM-RFRNEC------E---------GRSAGSIPGESSTP---ERK---------TFPA   40
_KAJ8314234.1_       1  -VEILEQPQ-QRGL-RFRYEC------E---------SRFGGSIHGEKSVP---TKK---------TFPT   40
_VDH95719.1_            ----------------------------------------------------------------------
_XP_041352913.1_     1  -VEIVEQPK-SRGL-RFRYEC------E---------GRSAGSIPGERTSA---EKK---------TFPT   40
_XP_046555158.1_     1  -MEIVEQPQ-SRGL-RFRYEC------E---------GRSAGSIPGEHSSS---ERK---------TFPT   40
_AAW33559.1_         1  -MEIVEQPQ-SRGL-RFRYEC------E---------GRSAGSIPGEHSSS---ERK---------TFPT   40
_ABY87435.1_         1  -----------------FRYEC------E---------GRSAGSIPGEHSSS---ERK---------TFPT   27
_XP_009028834.1_     1  -VSIDEQPK-QRGL-RFRYKC------E---------GRSAGSIPGEHSTS---DRK---------TYPS   40
_XP_025106109.1_     1  -VEIVEQPK-QRGL-RFRYQC------E---------GRSAGSIPGEHSTN---EKK---------TFPT   40
_NP_001298191.1_     1  -VEILEQPK-SRGL-RFRYEC------E---------GRSAGSVPGENSTN---DHR---------TYPT   40
_KAH9519545.1_       1  -VEIVEQPK-SRGL-RFRYEC------E---------GRSAGSVPGENSTN---DHR---------TYPT   40
_RUS74561.1_         1  -VQILEQPK-SRGL-RFRYEC------E---------GRSAGSVPGENSTN---DHR---------TYPT   40
_GFO11973.1_         1  -VEILEQPK-SRGL-RFRYEC------E---------GRSAGSVPGENSTN---DHR---------TYPT   40
```

```
_CAG5132534.1_      1  -VEILEQPK-SRGL-RFRYEC------E---------GRSAGSVPGENSSN---EHR---------TYPT  40
_GFR69641.1_        1  -VEIIEQPK-SRGL-RFRYEC------E---------GRSAGSVPGENSTN---DHR---------TYPT  40
_XP_035824873.1_    1  -VEILEQPK-SRGL-RFRYEC------E---------GRSAGSVPGENSTN---DHR---------TYPT  40
_AAY27981.1_        1  -VVITEQPK-SRGF-RFRYQC------E---------GRSAGSILGESSTA---ENK---------TYPT  40
_XP_052827753.1_    1  --IIVDQPK-THGF-RFRYQC------E---------GRSAGSIPGEKSTN---EKK---------SYPT  39
_XP_029642523.1_    1  --IIVDQPK-THGF-RFRYQC------E---------GRSAGSIPGEKSTN---EKK---------SYPT  39
_CAE1288013.1_         ----------------------------------------------------------------------
_ELU04547.1_        1  -LEITEQPK-ARGQ-RFRYKC------E---------GRSAGSIPGEHSTN---EHK---------TYPS  40
_KAI0227899.1_      1  -LVIVEQPK-PRGL-RFRYEC------E---------GRSAGAIPGENSTT---ENR---------TYPT  40
_XP_044002103.1_    1  --VIPEQPA--KTV-RFRYKL------EC------ESRRSASSIPGRNSTP---ENK---------TLPT  41
_XP_044002102.1_    1  ---IVEQPA-SKAI-RFRYKC------E---------GRSAGSIPGVNSTP---ENK---------TYPT  38
_XP_044002099.1_    1  ---IVEQPA-SKAI-RFRYKC------E---------GRSAGSIPGVNSTP---ENK---------TYPT  38
_XP_037026629.1_    1  -VEIIEQPQ-PKSM-RFRYSS------E---------GNSAGSIFGVNSTE---DAK---------TYPS  40
_CAD7632570.1_      1  -VRIVEQPA-RSAV-RFRYEC------E---------GRSAGPIPGVSSTP---SCA---------TYPT  40
_CAD7650738.1_      1  -VRIVEQPA-RSAV-RFRYEC------E---------GRSAGPIPGVSSTP---SCA---------TYPT  40
_XP_054163335.1_    1  -VRIIEQPA-RGGT-RFRYEC------E---------GRSAGSIPGVNSTP---DIT---------TYPA  40
_CAD7629985.1_      1  -VRIIEQPA-RGGT-RFRYEC------E---------GRSAGSIPGVNSTP---DIT---------TYPA  40
_CAD7652069.1_      1  -VRIIEQPA-RGGT-RFRYEC------E---------GRSAGSIPGVNSTP---DIT---------TYPA  40
_XP_017954168.1_    1  -IRIIEEPT-NKII-RFRYKC------E---------GRTAGSIAGMNSTP---EAK---------TFPT  40
_XP_017858583.1_    1  -IRIIEEPT-NKII-RFRYKC------E---------GRTAGSIAGMNSTP---EAK---------TFPT  40
_XP_002001588.1_    1  -IRIIEEPT-NKII-RFRYKC------E---------GRTAGSIAGMNSTP---EAK---------TFPT  40
_XP_017954163.1_    1  -IRIIEEPT-NKII-RFRYKC------E---------GRTAGSIAGMNSTP---EAK---------TFPT  40
_XP_023164563.2_    1  -IRIVEEPT-NKII-RFRYKC------E---------GRTAGSIAGMNSTP---DAK---------TFPT  40
_TDG46702.1_        1  -IRIIEEPT-NKII-RFRYKC------E---------GRTAGSIAGMNSTP---EAK---------TFPT  40
_XP_030564898.1_    1  -IRIVEEPT-NKII-RFRYKC------E---------GRTAGSIAGMNSTP---EAK---------TFPT  40
_XP_015024496.1_    1  -IRIVEEPT-NKII-RFRYKC------E---------GRTAGSIAGMNSTP---EAK---------TFPT  40
_XP_034475238.1_    1  -IQIIEEPT-NKII-RFRYEC------E---------GRTAGSIAGMHSTP---TKK---------TYPT  40
_XP_034475235.1_    1  -IQIIEEPT-NKII-RFRYEC------E---------GRTAGSIAGMHSTP---TKK---------TYPT  40
_XP_017854415.1_    1  -IHIVEEPT-DNVI-RFRYKC------E---------GRTAGSIPGKNSKP---EMK---------TFPT  40
_XP_017854416.1_    1  -IHIVEEPT-DNVI-RFRYKC------E---------GRTAGSIPGKNSKP---EMK---------TFPT  40
_ALC40089.1_        1  -IHIVEEPT-DNVI-RFRYKC------E---------GRTAGSIPGKNSKP---EMK---------TFPT  40
_XP_030382983.1_    1  -IRIIEEPA-ERVT-RFRYKC------E---------DRFATPIAGKNSDA--QKCK---------TYPT  41
_XP_030382981.1_    1  -IRIIEEPA-ERVT-RFRYKC------E---------DRFATPIAGKNSDA--QKCK---------TYPT  41
_XP_001989397.1_    1  -IRIVEEPT-NKII-RFRYKC------E---------GRTAGSIAGMNSTA---ESK---------TFPT  40
_XP_002067135.2_    1  -IRIVEEPT-NNII-RFRYKC------E---------GRTAGSIAGMNSSP--ESGK---------TFPT  41
_XP_034475239.1_    1  -IQIIEEPT-NKII-RFRYEC------E---------GRTAGSIVGMSSTP---TKK---------TYPT  40
_KAH8411901.1_      1  -VRIVEEPT-NHAI-RFRYKC------E---------GRTAGSIAGMHSSP---TNK---------TFPT  40
_XP_016963143.1_    1  -LRIVEEPT-NHII-RFRYKC------E---------GRTAGSIPGMNSSS--ETGK---------TFPT  41
_XP_037712361.1_    1  -LRIVEEPT-NNII-RFRYKC------E---------GRTAGSIPGMNSSP--ETGK---------TFPT  41
```

```
_XP_016993627.2_     1  -LRIVEEPT-NHII-RFRYKC------E---------GRTAGSIPGMNSSS--ETGK---------TFPT   41
_XP_016963141.1_     1  -LRIVEEPT-NHII-RFRYKC------E---------GRTAGSIPGMNSSS--ETGK---------TFPT   41
_XP_016993624.2_     1  -LRIVEEPT-NHII-RFRYKC------E---------GRTAGSIPGMNSSS--ETGK---------TFPT   41
_XP_037712358.1_     1  -LRIVEEPT-NNII-RFRYKC------E---------GRTAGSIPGMNSSP--ETGK---------TFPT   41
_XP_001974449.1_     1  -LRIVEQPT-SNII-RFRYKC------E---------GRTAGSIPGMSSSS--DTGK---------TFPT   41
_AAM48404.1_         1  -LRIVEEPT-SNII-RFRYKC------E---------GRTAGSIPGMNSSS--ETGK---------TFPT   41
_NP_001162999.1_     1  -LRIVEEPT-SNII-RFRYKC------E---------GRTAGSIPGMNSSS--ETGK---------TFPT   41
_XP_026835878.1_     1  -LRIVEQPT-SNII-RFRYKC------E---------GRTAGSIPGMSSSS--DTGK---------TFPT   41
_AAA28465.1_         1  -LRIVEEPT-SNII-RFRYKC------E---------GRTAGSIPGMNSSS--ETGK---------TFPT   41
_XP_002038912.1_     1  -LRIVEEPT-NNII-RFRYKC------E---------GRTAGSIPGMNSSS--ETGK---------TFPT   41
_XP_033169558.1_     1  -LRIVEEPT-SNII-RFRYKC------E---------GRTAGSIPGMNSSS--ETGK---------TFPT   41
_EDX05310.1_         1  -LRIVEEPT-SNII-RFRYKC------E---------GRTAGSIPGMNSSS--ETGK---------TFPT   41
_NP_001162998.1_     1  -LRIVEEPT-SNII-RFRYKC------E---------GRTAGSIPGMNSSS--ETGK---------TFPT   41
_XP_032579766.1_     1  -LRIVEEPT-NNII-RFRYKC------E---------GRTAGSIPGMNSSS--ETGK---------TFPT   41
_XP_033169541.1_     1  -LRIVEEPT-SNII-RFRYKC------E---------GRTAGSIPGMNSSS--ETGK---------TFPT   41
_XP_034669502.1_     1  -LRIIEEPT-NNII-RFRYKC------E---------GRTAGSIPGMSSSP--ETGK---------TFPT   41
_XP_034125957.1_     1  -LRIIEEPT-NNII-RFRYKC------E---------GRTAGSIPGMSSSP--ETGK---------TFPT   41
_XP_034669501.1_     1  -LRIIEEPT-NNII-RFRYKC------E---------GRTAGSIPGMSSSP--ETGK---------TFPT   41
_XP_034125956.1_     1  -LRIIEEPT-NNII-RFRYKC------E---------GRTAGSIPGMSSSP--ETGK---------TFPT   41
_XP_034125955.1_     1  -LRIIEEPT-NNII-RFRYKC------E---------GRTAGSIPGMSSSP--ETGK---------TFPT   41
_XP_017152033.1_     1  -LRIIEEPT-NNII-RFRYKC------E---------GRTAGSIPGMSTST--ETGK---------TFPT   41
_XP_017152031.1_     1  -LRIIEEPT-NNII-RFRYKC------E---------GRTAGSIPGMSTST--ETGK---------TFPT   41
_XP_002014799.1_     1  -LRIIEEPT-NNII-RFRYKC------E---------GRTAGSIPGMSTST--ETGK---------TFPT   41
_XP_026844902.1_     1  -LRIIEEPT-NNII-RFRYKC------E---------GRTAGSIPGMSTST--ETGK---------TFPT   41
_XP_015035983.2_     1  -LRIIEEPT-NNII-RFRYKC------E---------GRTAGSIPGMSTST--ETGK---------TFPT   41
_XP_017063896.1_     1  -LRIVEEPT-NHII-RFRYKC------E---------GRTAGSIPGMTSSS--ETGK---------TFPT   41
_XP_016935332.1_     1  -LRIVEEPT-NHII-RFRYKC------E---------GRTAGSIPGMNSSA--ETGK---------TFPT   41
_XP_017063894.2_     1  -LRIVEEPT-NHII-RFRYKC------E---------GRTAGSIPGMTSSS--ETGK---------TFPT   41
_XP_036668637.1_     1  -LRIVEEPT-NHII-RFRYKC------E---------GRTAGSIPGMNSSA--ETGK---------TFPT   41
_XP_016935310.1_     1  -LRIVEEPT-NHII-RFRYKC------E---------GRTAGSIPGMNSSA--ETGK---------TFPT   41
_KAH8298318.1_       1  -LRIVEEPT-NNII-RFRYKC------E---------GRTAGSIPGMNSSS--ETGK---------TFPT   41
_KAH8412187.1_       1  -LRIVEEPT-NNII-RFRYKC------E---------GRTAGSIPGMSTSS--ETGK---------TFPT   41
_KAH8278042.1_       1  -LRIVEQPT-NNII-RFRYKC------E---------GRTAGSIPGENSSD---KGK---------TFPT   40
_KAH8366423.1_       1  -LRIVEEPT-SNII-RFRYKC------E---------GRTAGSIPGMNSSP--ETGK---------TFPT   41
_XP_017044860.1_     1  -LRIVEEPT-NNII-RFRYKC------E---------GRTAGSIPGMNSTT---EHK---------TFPT   40
_KAH8324526.1_       1  -LRIVEEPT-SNII-RFRYKC------E---------GRTAGSIPGSNSD----KGK---------SFPT   39
_XP_017116817.1_     1  -LRIVEEPT-NNII-RFRYKC------E---------GRTAGSIPGMNSSP--ENGK---------TFPT   41
_XP_041566118.1_     1  -LRIVEEPT-NNII-RFRYKC------E---------GRTAGSIPGMNSSP--ENGK---------TFPT   41
_XP_052852584.1_     1  -LRIVEEPT-NNII-RFRYKC------E---------GRTAGSIPGMNSSP--ENGK---------TFPT   41
```

```
_XP_052852582.1_     1  -LRIVEEPT-NNII-RFRYKC------E---------GRTAGSIPGMNSSP--ENGK---------TFPT   41
_XP_017107132.2_     1  -LRIVEQPT-NNII-RFRYKC------E---------GRTAGSIPGMNSSD---KGK---------TFPT   40
_XP_043069650.1_     1  -LRIVEQPT-NNII-RFRYKC------E---------GRTAGSIPGMNSSD---KGK---------TFPT   40
_KAH8283158.1_       1  -LRIVEEPT-NNII-RFRYKC------E---------GRTAGSIPGMNSNS--ENGK---------TFPT   41
_XP_017025640.1_     1  -LRIVEEPT-NNII-RFRYKC------E---------GRTAGSIPGMNSNS--ENGK---------TFPT   41
_XP_020803071.1_     1  -LRIVEEPT-NNII-RFRYKC------E---------GRTAGSIPGMNSNS--ENGK---------TFPT   41
_XP_017025638.1_     1  -LRIVEEPT-NNII-RFRYKC------E---------GRTAGSIPGMNSNS--ENGK---------TFPT   41
_KAH8308067.1_       1  -LRIVEEPT-NNII-RFRYKC------E---------GRTAGSIPGMNSNS--ENGK---------TFPT   41
_KAH8362606.1_       1  -LRIVEEPT-NNII-RFRYKC------E---------GRTAGSIPGMNSNS--ENGK---------TFPT   41
_XP_020803068.1_     1  -LRIVEEPT-NNII-RFRYKC------E---------GRTAGSIPGMNSNS--ENGK---------TFPT   41
_XP_015052364.1_     1  -LRIVEQPT-GNVI-RFRYEC------E---------GRTAGSIPGKNSKS---NCK---------TFPT   40
_XP_002090469.1_     1  -LRIVEQPT-GNVI-RFRYEC------E---------GRTAGSIPGKNSKS---NCK---------TFPT   40
_XP_039480354.1_     1  -LRIVEQPT-GNII-RFRYKC------E---------GRTAGSIPGKNSKL---NCK---------TFPT   40
_XP_039480352.1_     1  -LRIVEQPT-GNII-RFRYKC------E---------GRTAGSIPGKNSKL---NCK---------TFPT   40
_XP_041450795.1_     1  -LRIIEEPT-NNII-RFRYKC------E---------GRTAGSIPGMSSSA--ERGK---------TFPT   41
_XP_044315461.1_     1  -LRIVEEPT-NNII-RFRYKC------E---------GRTAGSIPGMNSSP--ENGK---------TFPT   41
_XP_044315463.1_     1  -LRIVEEPT-NNII-RFRYKC------E---------GRTAGSIPGMNSSP--ENGK---------TFPT   41
_XP_001961710.2_     1  -LRIVEEPT-NNII-RFRYKC------E---------GRTAGSIPGMNSNT--DKGK---------TFPT   41
_XP_014761890.1_     1  -LRIVEEPT-NNII-RFRYKC------E---------GRTAGSIPGMNSNT--DKGK---------TFPT   41
_KAH8323309.1_       1  -LRIVEEPT-NNII-RFRYKC------E---------GRTAGSIPGMNSNT--DKGK---------TFPT   41
_XP_043646206.1_     1  -LRIVVQPS-SNTI-RFRYKC------E---------GRTAGSIPGRNNNQ---NGK---------TYPT   40
_XP_043646204.1_     1  -LRIVVQPS-SNTI-RFRYKC------E---------GRTAGSIPGRNNNQ---NGK---------TYPT   40
_AAQ65066.1_         1  -LRIVEEPT-SNII-RFRYKC------E---------GRTAGSIPGMNSCS--ETGK---------TFPT   41
_AAQ64900.1_         1  -LRIVEEPT-SNII-RFRYKC------E---------GRTAGSIPGMNSSS--ETGK---------TFPT   41
_AAQ64905.1_         1  -LRIVEEPT-SNII-RFRYKC------E---------GRTAGSIPGMNSSS--ETGK---------TFPT   41
_KAH8378209.1_       1  -IRIVEEPT-NHII-RFRYKC------E---------GRTAGSIPGINSKP---NGK---------TYPT   40
_KAH8293207.1_       1  -VRIVVEPT-RNII-RFRYKC------E---------GRTAGSIPGINSTA---GVK---------TYPT   40
_KAH8391978.1_       1  --RIVVEPT-NHII-RFRYKC------E---------GRTAGSIPGNNSKP---EAK---------TYPT   39
_XP_034101352.1_     1  --RIVVEPT-NHII-RFRYKC------E---------GRTAGSIPGNNSKP---EAK---------TYPT   39
_KAJ3658269.1_       1  -IVIIEQPS--KSL-RFRYVC----------------EGRPGTIPGASSTP---ENK---------TSFA   38
_KAJ3658270.1_       1  -IEIIEQPY--KAH-KFRYIQ----------------EGRGMPIPGASSTP---GNR---------TSFA   38
_KAJ3658271.1_       1  -ITIVEQPF--RYH-RFRYTQ----------------EGRPIPIPGASSTP---ENK---------TSVA   38
_XP_015117085.1_     1  -IEVLEQPA-SKEF-RFRYEC------E---------RRSHGSLPGVHSTV---WKK---------TYPK   40
_XP_011307643.1_     1  -VEVIEQPA-SKEF-RFRYEC------E---------RRSHGSIPGVHSTS---SNK---------TYPK   40
_XP_011307741.1_     1  -VEIVEQPA-SKAF-RFRYES------E---------RRPHGSIPGINSTP---THK---------TYPT   40
_XP_015119445.1_     1  -IEILEQPA-SKEF-RFRYEC------E---------RRLHGSIPGVHSTL---LRK---------TYPK   40
_XP_052128594.1_     1  ---ILEQPA--SLV-RFRYES------E---------GLYPTQ--GATSTA---MKK---------TFPT   35
_XP_052128597.1_     1  ---ILEQPA--SLV-RFRYES------E---------GLYPTQ--GATSTA---MKK---------TFPT   35
_KAE8741315.1_       1  ---ILEQPA--SLV-RFRYES------E---------GLYPTQ--GATSTA---MKK---------TFPT   35
```

```
_XP_028982160.1_      1   -IEIVEQPA-PRAV-RFRYES------E---------GRPSGNLIGVNSTK---ECT---------TFPK   40
_XP_011307645.1_      1   -IEIIEQPA-PRAV-RFRYQS------E---------GRPSGNLIGLNSTK---ERT---------TYPK   40
_XP_054725384.1_      1   -VRIVEHPA-DKWF-KFRYSS------D---------SQTSSMLLGENWSP---SRE---------TYPA   40
_XP_053965385.1_      1   -VRIVEHPA-AKGF-KFRYSS------D---------SQTSSMLLGENWSP---SRE---------TYPA   40
_XP_013149158.1_      1   -VRVVVQPV--DSH-RFRYKS------E---------GRWAGSIPGVNKEG---NKE---------GFPT   39
_XP_045534655.1_      1   -VRVVVQPV--DSH-RFRYKS------E---------GRWAGSIPGVNKKG---NRE---------GFPT   39
_KPJ06852.1_          1   -VRVVVQPV--DSH-RFRYKS------E---------GRWAGSIPGVNKKG---NRE---------GFPT   39
_XP_013176418.1_      1   -VRVIVQPV--DSH-RFRYKS------E---------GRWAGSIPGVNKKG---NKE---------GYPT   39
_OXU28425.1_          1   -IKIIVQPSNS----RFGYKD----------------EKRASFIKG---------SEG---------NYPA   32
_XP_008216913.1_      1   -IKIVVQPSNS----RFGYKD------E---------QRASF-IQ---------GSE---------AYPA   31
_XP_008216914.1_      1   -IKIVVQPSNS----RFGYKD------E---------QRASF-IQ---------GSE---------AYPA   31
_XP_040572301.1_      1   -LRIVKQPA-HNKQ-RFRYPC------E---------GRNPEVLYGEDSTK---KTR---------TYPT   40
_CAB4068408.1_        1   -------------------------------------------------K---KTR---------TYPT    8
_KAF7993284.1_            ---------------------------------------------------------------------
_KAF7987489.1_        1   --EIIEQPK--KLV-RFRYKC----------------EGSAAGIQGINSTH---EKK---------TYPT   37
_KAF7994520.1_        1   -IEIIEEPK--KLI-RFRYAC------E---------GCTAGSIQGINSSS---EKR---------TYPT   39
_XP_044019368.1_      1   -ISIMEQPT--SSM-KFRYRC----------------ERRNNVIYGMNSTT---ATK---------TYPT   38
_XP_044019332.1_          ---------------------------------------------------------------------
_XP_044003986.1_          ---------------------------------------------------------------------
_XP_037042732.1_      1   -IEIVEEPA-PKLK-RFRYES----------------EGRSAPLEGVRTTQ---KKK---------SFPS   39
_CAD7223243.1_        1   -LEIIEQPA-GKAN-RFRYQC------E--------GGGRAGPIPGARSTT---RNK---------TFPT   41
_XP_044732233.1_      1   --EITVQPN--DSM-RFRYVS----------------EGTAGRIYGKNHTN---EIP---------IYPT   37
_XP_012231885.1_      1   -VQILEQPT--SKF-RFRYES----------------EGPAGSIPGVNYTS---RNK---------IYPS   38
_XP_022814699.1_      1   -LSIAVQPE--KYK-RFRYED------E---------LTSTTVLQGAKATTEGDDDEI-------TTYPT   44
_CAB3516523.1_        1   -LSIAVQPE--KYK-RFRYED------E---------LTSTTVLQGAKTTTEGDDDEI-------TTYPT   44
_XP_035458245.2_      1   -LTIPVQPE--KFK-RFRYED------E---------LTSSTVLQGATAADGDDEGH--------TTFPT   43
_KAF9795795.1_        1   ---------------RFRYED------E---------LTSSTVLQGATAADGDDEGN--------ATFPT   32
_XP_014233257.1_      1   ----HEQPA--HRA-LFRWAG------D---------ACGCESQIGSRDP----RRA---------DCPS   35
_XP_034324400.1_      1   -----------KMT-RFRYDT----------------ELN-------NRNI---GKE---------ELVS   23
_XP_052699907.1_      1   -----------KMT-RFRYDT----------------ELN-------NRNI---GKE---------ELVS   23
Consensus_aa:             .lcIhEQP...p.h.RFRYpt......E.........sp.tGsl.G.poo....pp+.........T@Po
Consensus_ss:               eeeee          eeeeee------                                         ee


Conservation:                        5                  5567                79   6
_XP_001652840.2_     41   IE---------IRGYK----GRAV----VVVSCVTKDP--------PYRPHPHNLV--GKEGCK------   77
_AAW67214.1_         41   IE---------IRGYK----GRAV----VVVSCVTKDP--------PYRPHPHNLV--GKEGCK------   77
_XP_021704027.1_     41   IE---------IRGYK----GRAV----VVVSCVTKDP--------PYRPHPHNLV--GKEGCK------   77
_XP_019540833.2_     41   IE---------IRGYK----GRAV----VVVSCVTKDP--------PYRPHPHNLV--GKEGCK------   77
```

_XP_029720231.1_	41	IE---------IRGYK----GRAV----VVVSCVTKDP--------PYRPHPHNLV--GKEGCK------	77
AAW67215.1	41	IE---------IRGYK----GRAV----VVVSCVTKDP--------PYRPHPHNLV--GKEGCK------	77
_XP_021704025.1_	41	IE---------IRGYK----GRAV----VVVSCVTKDP--------PYRPHPHNLV--GKEGCK------	77
_XP_019540831.2_	41	IE---------IRGYK----GRAV----VVVSCVTKDP--------PYRPHPHNLV--GKEGCK------	77
KXJ81105.1	41	IE---------IRGYK----GRAV----VVVSCVTKDP--------PYRPHPHNLV--GKEGCK------	77
KXJ76497.1	41	IE---------IRGYK----GRAV----VVVSCVTKDP--------PYRPHPHNLV--GKEGCK------	77
_XP_023240428.1_	41	IQ---------IQNYS----GSAV----VVVSCVTKDP--------PYRAHPNNLV--GKEGCR------	77
CAD7241875.1	41	IE---------VVGHK----GRAI----AVVSCVTKDGP-------PYRPHPHSLV--GREGCK------	78
TRY80291.1	41	IQ---------IVGYQ----GPAV----CVVSLVEHEK--------PYRAHPHNLV--GKDGLC------	77
ALF84899.1	41	IE---------IVGYQ----GPAV----VVVSCVEESP--------PHRTHPHNLV--GKHCK-------	76
KAF4528208.1	40	IK---------VMNYQ----GRAV----VVVSCVTKDV--------PYRPHPHNLV--GKDGFK------	76
_XP_054260955.1_	41	IQ---------IKNYK----GKVV----VVVSCVTKDA--------PYKPHPHNLV--GREGCK------	77
_XP_054264401.1_	41	IQ---------IKNYK----GKVV----VVVSCVTKDA--------PYKPHPHNLV--GREGCK------	77
_XP_054260954.1_	41	IQ---------IKNYK----GKVV----VVVSCVTKDA--------PYKPHPHNLV--GREGCK------	77
_XP_038114042.1_	41	IE---------VRGYK----GRAV----VVVSCVTKDP--------PYRPHPHNLV--GKEGCK------	77
_XP_039437558.1_	41	IE---------VRGYK----GRAV----VVVSCVTKDP--------PYRPHPHNLV--GKEGCK------	77
_XP_001844078.2_	41	IE---------VRGYK----GRAV----VVVSCVTKDP--------PYRPHPHNLV--GKEGCK------	77
EDS35747.1	41	IE---------VRGYK----GRAV----VVVSCVTKDP--------PYRPHPHNLV--GKEGCK------	77
_XP_053696925.1_	41	IE---------VRGYK----GRAV----VVVSCVTKDP--------PYRPHPHNLV--GKEGCK------	77
_XP_001869619.2_	41	IE---------LRGYK----GRAV----VVVSCVTKDP--------PYRAHPHNLV--GKDGCK------	77
_XP_039434954.1_	41	IE---------LRGYK----GRAV----VVVSCVTKDP--------PYRAHPHNLV--GKDGCK------	77
EDS29879.1	41	IE---------LRGYK----GRAV----VVVSCVTKDP--------PYRAHPHNLV--GKDGCK------	77
_XP_019550742.2_	41	IE---------IRGYT----GRVV----VVVSCVTKDP--------PYRPHPHNLV--GKEGCK------	77
KXJ74587.1	41	IE---------IRGYT----GRVV----VVVSCVTKDP--------PYRPHPHNLV--GKEGCK------	77
_XP_035908773.1_	41	IQ---------VHGYR----GRAV----VVVSCVTKEGP-------DHKPHPHNLV--GKEGCK------	78
AJC98655.1	41	IQ---------VHGYR----GRAV----VVVSCVTKEGP-------EHKPHPHNLV--GKEGCK------	78
AJC98650.1	41	IQ---------VHGYR----GRAV----VVVSCVTKEGP-------EHKPHPHNLV--GKEGCK------	78
AJC98644.1	41	IQ---------VHGYR----GRAV----VVVSCVTKEGP-------EHKPHPHNLV--GKEGCK------	78
AJC98657.1	41	IQ---------VHGYR----GRAV----VVVSCVTKEGP-------EHKPHPHNLV--GKEGCK------	78
AJC98648.1	41	IQ---------VHGYR----GRAV----VVVSCVTKEGP-------EHKPHPHNLV--GKEGCK------	78
AJC98647.1	41	IQ---------VHGYR----GRAV----VVVSCVTKEGP-------EHKPHPHNLV--GKEGCK------	78
AJC98651.1	41	IQ---------VHGYR----GRAV----VVVSCVTKEGP-------EHKPHPHNLV--GKEGCK------	78
AJC98643.1	41	IQ---------VHGYR----GRAV----VVVSCVTKEGP-------EHKPHPHNLV--GKEGCK------	78
AJC98656.1	41	IQ---------VHGYR----GRAV----VVVSCVTKEGP-------EHKPHPHNLV--GKEGCK------	78
AJC98641.1	41	IQ---------VHGYR----GRAV----VVVSCVTKEGP-------EHKPHPHNLV--GKEGCK------	78
AJC98640.1	41	IQ---------VHGYR----GRAV----VVVSCVTKEGP-------EHKPHPHNLV--GKEGCK------	78
AJC98653.1	41	IQ---------VHGYR----GRAV----VVVSCVTKEGP-------EHKPHPHNLV--GKEGCK------	78
AJC98654.1	41	IQ---------VHGYR----GRAV----VVVSCVTKEGP-------EHKPHPHNLV--GKEGCK------	78
AJC98652.1	41	IQ---------VHGYR----GRAV----VVVSCVTKEGP-------EHKPHPHNLV--GKEGCK------	78

AJC98646.1	41	IQ---------VHGYR----GRAV----VVVSCVTKEGP-------EHKPHPHNLV--GKEGCK------	78
AJC98658.1	41	IQ---------VHGYR----GRAV----VVVSCVTKEGP-------EHKPHPHNLV--GKEGCK------	78
AJC98639.1	41	IQ---------VHGYR----GRAV----VVVSCVTKEGP-------EHKPHPHNLV--GKEGCK------	78
_XP_035908772.1_	41	IQ---------VHGYR----GRAV----VVVSCVTKEGP-------DHKPHPHNLV--GKEGCK------	78
_XP_050076803.1_	41	IQ---------VHGYR----GRAV----VVVSCVTKEGP-------DHKPHPHNLV--GKEGCK------	78
_XP_049282441.1_	41	IQ---------VHGYR----GRAV----VVVSCVTKEGP-------DHKPHPHNLV--GKEGCK------	78
_XP_040170080.1_	41	IQ---------VHGYR----GRAV----VVVSCVTKEGP-------EHKPHPHNLV--GKEGCK------	78
_XP_050076802.1_	41	IQ---------VHGYR----GRAV----VVVSCVTKEGP-------DHKPHPHNLV--GKEGCK------	78
_XP_041780138.1_	41	IQ---------VHGYR----GRAV----VVVSCVTKEGP-------EHKPHPHNLV--GKEGCK------	78
_XP_040239229.2_	41	IQ---------VHGYR----GRAV----VVVSCVTKEGP-------EHKPHPHNLV--GKEGCK------	78
_XP_040239222.2_	41	IQ---------VHGYR----GRAV----VVVSCVTKEGP-------EHKPHPHNLV--GKEGCK------	78
_XP_035908780.1_	41	IQ---------VHGYR----GRAV----VVVSCVTKEGP-------DHKPHPHNLV--GKEGCK------	78
_XP_040239221.2_	41	IQ---------VHGYR----GRAV----VVVSCVTKEGP-------EHKPHPHNLV--GKEGCK------	78
_XP_052897786.1_	41	IQ---------VHGYR----GRAV----VVVSCVTKEGP-------EHKPHPHNLV--GKEGCK------	78
_XP_052895493.1_	41	IQ---------VHGYR----GRAV----VVVSCVTKEGP-------EHKPHPHNLV--GKEGCK------	78
_XP_053664395.1_	41	IQ---------VHGYR----GRAV----VVVSCVTKEGP-------DHKPHPHNLV--GKEGCK------	78
_XP_050076801.1_	41	IQ---------VHGYR----GRAV----VVVSCVTKEGP-------DHKPHPHNLV--GKEGCK------	78
_XP_310177.3_	41	IQ---------VHGYR----GRAV----VVVSCVTKEGP-------EHKPHPHNLV--GKEGCK------	78
_XP_001652339.1_	41	IE---------VRGYT----GRVV----VVVSCVTKDP--------PYRPHPHNLV--GKDGCK------	77
_XP_053686256.1_	41	IE---------VRGYK----GPAM----VVVSCVTKDP--------PYRPHPHNLV--GKEGCK------	77
_XP_053675540.1_	41	IQ---------VHGYR----GRAV----VVVSCVTKEGP-------DYKPHPHNLV--GKEGCK------	78
ETN66814.1	41	IR---------VQGYR----GRAV----VVVSCVTIDGP-------NHKPHPHNLV--GKDCK-------	77
_XP_049534501.1_	41	IR---------VQGYR----GRAV----VVVSCVTIDGP-------NHKPHPHNLV--GKDCK-------	77
_XP_050084431.1_	41	IR---------VQGYR----GRAV----VVVSCVTVDGP-------AHKPHPHNLV--GKDCK-------	77
_XP_035782874.1_	41	IR---------VQGYR----GRAV----VVVSCVTVDGP-------AHKPHPHNLV--GKDCK-------	77
_XP_035782869.1_	41	IR---------VQGYR----GRAV----VVVSCVTVDGP-------AHKPHPHNLV--GKDCK-------	77
KFB39849.1	41	IQ---------IHGYR----GRAV----VVVSCVTKGEPNESPEEAKYKPHPHNLV--GKEGCK------	85
_XP_052871375.1_	41	IQ---------VHGYR----GRAV----VVVSCVAKEGP-------NHKPHPHNLV--GKEHCR------	78
AAR13743.1	26	IQ---------VHGYR----GRAV----VVVSCVTKEGP-------EHKPHPHNLV--GKEGCK------	63
AAR13744.1	26	IQ---------VHGYR----GRAV----VVVSCVTKEGP-------EHKPHPHNLV--GKEGCK------	63
CAA65157.1	20	IQ---------VHGYR----GRAV----VVVSCVTKEGP-------EHKPHPHNLV--GKEGCK------	57
_XP_022170156.1_	41	IQ---------IVGYK----GRAV----VVVSCVTKDR--------PYRPHPHNLV--GRDNCK------	77
_XP_025205168.1_	41	IQ---------IVGYK----GRAV----VVVSCVTKDR--------PFRPHPHNLV--GRDNCK------	77
_XP_026817162.1_	41	IQ---------IVGYK----GRAV----VVVSCVTKDR--------PFRPHPHNLV--GRDNCK------	77
_XP_027846205.1_	41	IQ---------IVGYK----GRAV----VVVSCVTKDR--------PFRPHPHNLV--GRDNCK------	77
_XP_015370635.1_	41	IQ---------IVGYK----GRAV----VVVSCVTKDR--------PYRPHPHNLV--GRDNCK------	77
CAH1723797.1	41	IQ---------IVGYK----GRAV----VVVSCVTKDR--------PFRPHPHNLV--GRDNCK------	77
KAF0769940.1	41	IQ---------IVGYK----GRAV----VVVSCVTKDR--------PFRPHPHNLV--GRDNCK------	77
CAB4065202.1	41	IQ---------IVGYH----GPAV----VVVSCVEDKA--------PYRAHPHNLV--ASKILQ------	77

```
_XP_050440903.1_    41  IQ---------IVGYK----GRAV----VVVSCVTKDR--------PFRPHPHNLV--GRDNCK------   77
_XP_050533799.1_    41  IQ---------IVGYK----GRAV----VVVSCVTKDR--------PYRPHPHNLV--GRDNCK------   77
_XP_025410632.1_    41  IQ---------IVGYK----GRAV----VVVSCVTKDK--------PYRPHPHNLV--GRDNCK------   77
_XP_008180746.1_    41  IQ---------IVGYK----GRAV----VVVSCVTKDR--------PYRPHPHNLV--GRDNCK------   77
_XP_026465450.1_    41  IQ---------VVGYK----GRAL----VVVSCVSKDK--------PYRPHPHNLV--GKDWCT------   77
_VVC36026.1_        41  IQ---------IVGYK----GRAV----VVVSCVTKDS--------PFRSHPHNLV--GRDNCK------   77
_XP_016661522.1_    41  IQ---------IVGYK----GRAV----VVVSCVTKDR--------PYRPHPHNLV--GRDNCK------   77
_XP_001947429.3_    41  IQ---------IVGYK----GRAV----VVVSCVTKDR--------PYRPHPHNLV--GRDNCK------   77
_XP_026466007.1_    41  IQ---------VVGYK----GRAL----VVVSCVSKDK--------PYRPHPHNLV--GKDWCT------   77
_KRT81254.1_        39  IK---------IEGYK----GKVT----VVVSCVTKEK--------PYRAHPHNLV--GSDCK-------   74
_XP_033224832.1_    40  IT---------IVGYT----GKMS----VVVSCVTAEDNP----AIKPRPHPHNLV--GKQNCE------   80
_XP_025407590.1_    39  IK---------INGYQ----GKVL----VIVSCVTKDK--------PYRPHPFKLV--GGKHCK------   75
_XP_045476729.1_    39  IQ---------IFGYK----KKAV----VLVSCVTKDK--------PYRPHPFIMD--GKEGCK------   75
_XP_034174253.1_    41  IR---------IVGYK----GRAV----VVVSCVTKDQ--------PYRPHPHNLV--GKEACK------   77
_XP_029047866.1_    41  IR---------IVGYK----GRAV----VVVSCVTKDQ--------PYRPHPHNLV--GKEACK------   77
_XP_015430648.1_    41  IR---------IMGYK----GRAV----VVVSCVTKDQ--------PHRPHPHNLV--GKEACK------   77
_XP_017798081.1_    41  IR---------IVGYK----GRAV----VVVSCVTKDQ--------PHRPHPHNLV--GKEACK------   77
_XP_017756471.1_    41  IR---------IVGYK----GRAV----VVVSCVTKDQ--------PHRPHPHNLV--GKEACK------   77
_XP_043248447.1_    41  IR---------IVGYK----GRAV----VVVSCVTKDQ--------PHRPHPHNLV--GKEACK------   77
_XP_029047855.1_    41  IR---------IVGYK----GRAV----VVVSCVTKDQ--------PYRPHPHNLV--GKEACK------   77
_XP_034174252.1_    41  IR---------IVGYK----GRAV----VVVSCVTKDQ--------PYRPHPHNLV--GKEACK------   77
_XP_031829337.1_    41  IR---------IVGYK----GRAV----VIVSCVTKDQ--------PHRPHPHNLV--GKEACK------   77
_KZC09125.1_        41  IR---------IMGYK----GRAV----VVVSCVTKDQ--------PHRPHPHNLV--GKEACK------   77
_XP_033327213.1_    41  IR---------IVGYK----GRAL----VVVSCVTKDP--------PHRPHPHNLV--GKEACK------   77
_XP_033327212.1_    41  IR---------IVGYK----GRAL----VVVSCVTKDP--------PHRPHPHNLV--GKEACK------   77
_XP_017756445.1_    41  IR---------IVGYK----GRAV----VVVSCVTKDQ--------PHRPHPHNLV--GKEACK------   77
_OAD62393.1_        41  IR---------IVGYK----GRAV----VVVSCVTKDQ--------PHRPHPHNLV--GKEACK------   77
_XP_043248413.1_    41  IR---------IVGYK----GRAV----VVVSCVTKDQ--------PHRPHPHNLV--GKEACK------   77
_XP_033327208.1_    41  IR---------IVGYK----GRAL----VVVSCVTKDP--------PHRPHPHNLV--GKEACK------   77
_XP_034950477.1_    41  IR---------IVGYK----GRAV----VVVSCVTKDS--------PYRPHPHNLV--GKEACR------   77
_XP_034950475.1_    41  IR---------IVGYK----GRAV----VVVSCVTKDS--------PYRPHPHNLV--GKEACR------   77
_XP_012343274.1_    41  IK---------IVGYK----GRAL----VVVSCVTKDQ--------PYRPHPHNLV--GKEACK------   77
_XP_016909449.1_    41  IK---------IVGYK----GRAL----VVVSCVTKDQ--------PYRPHPHNLV--GKEACK------   77
_XP_012343273.1_    41  IK---------IVGYK----GRAL----VVVSCVTKDQ--------PYRPHPHNLV--GKEACK------   77
_XP_006619742.1_    41  IK---------IVGYK----GRAL----VVVSCVTKDQ--------PYRPHPHNLV--GKEACK------   77
_XP_006567065.1_    41  IK---------IVGYK----GRAL----VVVSCVTKDQ--------PYRPHPHNLV--GKEACK------   77
_XP_006619741.1_    41  IK---------IVGYK----GRAL----VVVSCVTKDQ--------PYRPHPHNLV--GKEACK------   77
_NP_001011577.1_    41  IK---------IVGYK----GRAL----VVVSCVTKDQ--------PYRPHPHNLV--GKEACK------   77
_XP_016909448.1_    41  IK---------IVGYK----GRAL----VVVSCVTKDQ--------PYRPHPHNLV--GKEACK------   77
```

_XP_043802365.1_	41	IK---------IVGYK----GRAL----VVVSCVTKDQ--------PYRPHPHNLV--GKEACK------	77
_NP_001164477.1_	41	IK---------IVGYK----GRAL----VVVSCVTKDQ--------PYRPHPHNLV--GKEACK------	77
_XP_016909443.1_	41	IK---------IVGYK----GRAL----VVVSCVTKDQ--------PYRPHPHNLV--GKEACK------	77
_XP_043802361.1_	41	IK---------IVGYK----GRAL----VVVSCVTKDQ--------PYRPHPHNLV--GKEACK------	77
_XP_012343269.1_	41	IK---------IVGYK----GRAL----VVVSCVTKDQ--------PYRPHPHNLV--GKEACK------	77
_XP_006619738.1_	41	IK---------IVGYK----GRAL----VVVSCVTKDQ--------PYRPHPHNLV--GKEACK------	77
PBC29878.1	41	IK---------IVGYK----GRAL----VVVSCVTKDQ--------PYRPHPHNLV--GKEACK------	77
_XP_046663984.1_	41	IQ---------IVGYK----GRAV----VVVSCVTKEQ--------PHRPHPHNLV--GRDGCK------	77
_XP_046663985.1_	41	IQ---------IVGYK----GRAV----VVVSCVTKEQ--------PHRPHPHNLV--GRDGCK------	77
_XP_043274771.1_	41	IR---------IVGYK----GRAV----VVVSCVTKDQ--------PYRPHPHNLV--GKEGCK------	77
_XP_043274769.1_	41	IR---------IVGYK----GRAV----VVVSCVTKDQ--------PYRPHPHNLV--GKEGCK------	77
_XP_043289571.1_	41	IK---------ICNYQ----GRGI----VVVSCVTKDQ--------PYRPHPHSLV--GKGTCK------	77
_XP_032665873.1_	41	IR---------IIGYK----GRAM----VVVSCVTKDA--------PYRPHPHNLV--GKEVCK------	77
_XP_032665875.1_	41	IR---------IIGYK----GRAM----VVVSCVTKDA--------PYRPHPHNLV--GKEVCK------	77
_XP_032665874.1_	41	IR---------IIGYK----GRAM----VVVSCVTKDA--------PYRPHPHNLV--GKEVCK------	77
_XP_032665869.1_	41	IR---------IIGYK----GRAM----VVVSCVTKDA--------PYRPHPHNLV--GKEVCK------	77
_XP_014470289.1_	41	IR---------IIGYK----GRAM----VVVSCVTKDA--------PYRPHPHNLV--GKEVCK------	77
_XP_011149046.1_	41	IR---------IIGYK----GRAM----VVVSCVTKDA--------PYRPHPHNLV--GKEVCK------	77
_XP_017877914.1_	40	IR---------IVGYK----GRAV----VVVSCVTKDQ--------PYKPHPHNLV--GKEACN------	76
_XP_017877911.1_	40	IR---------IVGYK----GRAV----VVVSCVTKDQ--------PYKPHPHNLV--GKEACN------	76
_XP_033174774.1_	41	IR---------IVGYK----GRAV----VVVSCVTKDQ--------PHRPHPHNLV--GKEACK------	77
_XP_043581013.1_	41	IR---------IVGYK----GRAV----VVVSCVTKDQ--------PHRPHPHNLV--GKEACK------	77
_XP_048261507.1_	41	IR---------IVGYK----GRAV----VVVSCVTKDQ--------PHRPHPHNLV--GKEACK------	77
_XP_050573946.1_	41	IR---------IVGYK----GRAV----VVVSCVTKDQ--------PHRPHPHNLV--GKEACK------	77
_XP_050478075.1_	41	IR---------IVGYK----GRAV----VVVSCVTKDQ--------PHRPHPHNLV--GKEACK------	77
_XP_033204249.1_	41	IR---------IVGYK----GRAV----VVVSCVTKDQ--------PHRPHPHNLV--GKEACK------	77
_XP_033358451.1_	41	IR---------IVGYK----GRAV----VVVSCVTKDQ--------PHRPHPHNLV--GKEACK------	77
_XP_033358450.1_	41	IR---------IVGYK----GRAV----VVVSCVTKDQ--------PHRPHPHNLV--GKEACK------	77
_XP_033204244.1_	41	IR---------IVGYK----GRAV----VVVSCVTKDQ--------PHRPHPHNLV--GKEACK------	77
_XP_033312956.1_	41	IR---------IVGYK----GRAV----VVVSCVTKDQ--------PHRPHPHNLV--GKEACK------	77
_XP_012236639.1_	41	IR---------IVGYK----GRAV----VVVSCVTKDQ--------PHRPHPHNLV--GKEACK------	77
_XP_050573940.1_	41	IR---------IVGYK----GRAV----VVVSCVTKDQ--------PHRPHPHNLV--GKEACK------	77
_XP_012174118.1_	41	IR---------IVGYK----GRAV----VVVSCVTKDQ--------PHRPHPHNLV--GKEACK------	77
_XP_043581010.1_	41	IR---------IVGYK----GRAV----VVVSCVTKDQ--------PHRPHPHNLV--GKEACK------	77
EZA51108.1	41	IR---------IIGYK----GRAM----VVVSCVTKDL--------PYRPHPHNLV--GKEVCK------	77
_XP_011344085.1_	41	IR---------IIGYK----GRAM----VVVSCVTKDL--------PYRPHPHNLV--GKEVCK------	77
_XP_043519475.1_	41	IR---------IVGYK----GRAV----VVVSCVTKDR--------PHRPHPHNLV--GKESCK------	77
CAD1481114.1	41	IR---------IVGYK----GRAV----VVVSCVTKDR--------PHRPHPHNLV--GKESCK------	77
_XP_043519473.1_	41	IR---------IVGYK----GRAV----VVVSCVTKDR--------PHRPHPHNLV--GKESCK------	77

KOX75360.1	41	IR---------IVGYK----GRAV----VVVSCVTKDR--------PHRPHPHNLV--GKESCK------	77
KAF3423741.1	41	IR---------IVGYK----GRAV----VVVSCVTKDR--------PHRPHPHNLV--GKESCK------	77
KOC69050.1	41	IR---------VGRYK----GRAV----VVVSCVTKDQ--------PHRPHPHNLV--GKEACK------	77
_XP_053980095.1_	41	IR---------IVGYK----GRAI----VVVSCVTKEE--------PYRPHPHNLV--GKEACK------	77
_XP_054002893.1_	41	IR---------IVGYK----GRAI----VVVSCVTKEE--------PYRPHPHNLV--GKEACK------	77
_XP_053980091.1_	41	IR---------IVGYK----GRAI----VVVSCVTKEE--------PYRPHPHNLV--GKEACK------	77
_XP_028049144.1_	41	IR---------IMGYK----GRAM----VVVSCVTKDS--------PYRPHPHNLV--GKEACK------	77
_XP_012541458.1_	41	IR---------IMGYK----GRAM----VVVSCVTKDS--------PYRPHPHNLV--GKEACK------	77
_XP_003708412.1_	41	IR---------IVNHN----GPTV----VIVSCVTKDE--------PYRPHPHNLV--GKEACK------	77
_XP_012152807.1_	41	IR---------IVNHN----GPTV----VIVSCVTKDE--------PYRPHPHNLV--GKEACK------	77
KAH0945556.1	41	IK---------IVGYK----GRAM----VVVSCVTKDL--------PYRPHPHNLV--GKEVCK------	77
_XP_024871832.1_	41	IR---------IVGYT----GRAM----VVVSCVTKDA--------PYRPHPHNLV--GKEACK------	77
_XP_011307641.1_	40	IR---------IAGYK----GRAV----VVVSCVTMDP--------PYRPHPHNLV--GKEGCK------	76
_XP_015118260.1_	40	IR---------IVGYK----GRAV----VVVSCVTMDP--------PYRPHPHNLV--GKEGCK------	76
_XP_011307640.1_	40	IR---------IAGYK----GRAV----VVVSCVTMDP--------PYRPHPHNLV--GKEGCK------	76
_XP_015118252.1_	40	IR---------IVGYK----GRAV----VVVSCVTMDP--------PYRPHPHNLV--GKEGCK------	76
EFN68841.1	41	IR---------IMGYN----GRAM----VIVSCVTKDAPD----NKGYRPHPHNLV--GKEVCK------	81
_XP_011647340.1_	41	IR---------IVGYK----GRAM----VVVSCVTKDSP--------YRPHPHNLV--GKEVCK------	77
_XP_029166122.1_	41	IR---------IVGYN----GRAM----VVVSCVTKDAPD----NKGYRPHPHNLV--GKEVCK------	81
KMQ96453.1	41	IR---------IVGYN----GRAM----VVVSCITKDAPD----NKGYRPHPHNLV--GKEVCK------	81
_XP_050464492.1_	41	IR---------IVGYN----GRAM----VVVSCVTKDAPD----NKGYRPHPHNLV--GKEVCK------	81
_XP_029673315.1_	41	IR---------IVGYN----GRAM----VVVSCVTKDAPD----NKGYRPHPHNLV--GKEVCK------	81
_XP_025265518.1_	41	IR---------IMGYN----GRAM----VIVSCVTKDAPD----NKGYRPHPHNLV--GKEVCK------	81
_XP_011255523.1_	41	IR---------IMGYN----GRAM----VIVSCVTKDAPD----NKGYRPHPHNLV--GKEVCK------	81
_XP_011698133.1_	41	IR---------IMGYQ----GRAM----VVVSCVTKDL--------PYRPHPHNLV--GKEVCK------	77
_XP_011698131.1_	41	IR---------IMGYQ----GRAM----VVVSCVTKDL--------PYRPHPHNLV--GKEVCK------	77
_XP_018357420.1_	41	IR---------IVGYK----GRAM----VVVSCVTKDA--------PYRPHPHNLV--GKEVCK------	77
KYM76140.1	41	IR---------IVGYK----GRAM----VVVSCVTKDA--------PYRPHPHNLV--GKEVCK------	77
_XP_011647339.1_	41	IR---------IVGYK----GRAM----VVVSCVTKDS--------PYRPHPHNLV--GKEVCK------	77
KAG5315434.1	41	IR---------IVGYK----GRAM----VVVSCVTKDA--------PYRPHPHNLV--GKEVCK------	77
_XP_011055463.1_	41	IR---------IVGYK----GRAM----VVVSCVTKDA--------PYRPHPHNLV--GKEVCK------	77
_XP_018342550.1_	41	IR---------IVGYK----GRAM----VVVSCVTKDA--------PYRPHPHNLV--GKEVCK------	77
_XP_018403565.1_	41	IR---------IVGYK----GRAM----VVVSCVTKDA--------PYRPHPHNLV--GKEVCK------	77
KAG5322912.1	41	IR---------IVGYK----GRAM----VVVSCVTKDA--------PYRPHPHNLV--GKEVCK------	77
_XP_018357417.1_	41	IR---------IVGYK----GRAM----VVVSCVTKDA--------PYRPHPHNLV--GKEVCK------	77
_XP_018314560.1_	41	IR---------IVGYK----GRAM----VVVSCVTKDA--------PYRPHPHNLV--GKEVCK------	77
_XP_018057217.1_	41	IR---------IVGYK----GRAM----VVVSCVTKDA--------PYRPHPHNLV--GKEVCK------	77
KAG5346558.1	41	IR---------IVGYK----GRAM----VVVSCVTKDA--------PYRPHPHNLV--GKEVCK------	77
_XP_047348408.1_	41	IR---------IVGYK----GRAV----VVVSCVTKDL--------PYRPHPHNLV--GKEICK------	77

```
_XP_047348412.1_    41  IR---------IVGYK----GRAV----VVVSCVTKDL--------PYRPHPHNLV--GKEICK------   77
_XP_046820611.1_    41  IR---------IVGYK----GRAV----VVVSCVTKDL--------PYRPHPHNLV--GKEICK------   77
_XP_050848254.1_    41  IR---------IVGYK----GRAV----VVVSCVTKDL--------PYRPHPHNLV--GKEICK------   77
_XP_046820612.1_    41  IR---------IVGYK----GRAV----VVVSCVTKDL--------PYRPHPHNLV--GKEICK------   77
_XP_050848252.1_    41  IR---------IVGYK----GRAV----VVVSCVTKDL--------PYRPHPHNLV--GKEICK------   77
_XP_043667564.1_    41  IR---------IVGYK----GRAV----VVVSCVTKDL--------PYRPHPHNLV--GKEICK------   77
_XP_035725337.1_    41  IR---------IVGYK----GRAV----VVVSCVTKDL--------PYRPHPHNLV--GKEICK------   77
_XP_043667562.1_    41  IR---------IVGYK----GRAV----VVVSCVTKDL--------PYRPHPHNLV--GKEICK------   77
_XP_046820605.1_    41  IR---------IVGYK----GRAV----VVVSCVTKDL--------PYRPHPHNLV--GKEICK------   77
_XP_050848249.1_    41  IR---------IVGYK----GRAV----VVVSCVTKDL--------PYRPHPHNLV--GKEICK------   77
_XP_043667560.1_    41  IR---------IVGYK----GRAV----VVVSCVTKDL--------PYRPHPHNLV--GKEICK------   77
_XP_035725333.1_    41  IR---------IVGYK----GRAV----VVVSCVTKDL--------PYRPHPHNLV--GKEICK------   77
_KAF7405659.1_      41  IR---------IVGYK----GRAV----VVVSCVTKDL--------PYRPHPHNLV--GKEICK------   77
_KAF7402294.1_      41  IR---------IVGYK----GRAV----VVVSCVTKDL--------PYRPHPHNLV--GKEICK------   77
_XP_015172825.1_    41  IR---------IVGYK----GRAV----VVVSCVTKDL--------PYRPHPHNLV--GKEACK------   77
_XP_015172824.1_    41  IR---------IVGYK----GRAV----VVVSCVTKDL--------PYRPHPHNLV--GKEACK------   77
_XP_015172823.1_    41  IR---------IVGYK----GRAV----VVVSCVTKDL--------PYRPHPHNLV--GKEACK------   77
_XP_014609306.1_    41  IR---------IVGYK----GRAV----VVVSCVTKDL--------PYRPHPHNLV--GKEACK------   77
_XP_043491219.1_    41  IR---------IVGYK----GRAV----VVVSCVTKDL--------PYRPHPHNLV--GKEACK------   77
_XP_014609305.1_    41  IR---------IVGYK----GRAV----VVVSCVTKDL--------PYRPHPHNLV--GKEACK------   77
_XP_043491218.1_    41  IR---------IVGYK----GRAV----VVVSCVTKDL--------PYRPHPHNLV--GKEACK------   77
_KAI4498556.1_      41  IR---------IVGYK----GRAV----VVVSCVTKDL--------PYRPHPHNLV--GKEACK------   77
_XP_015172818.1_    41  IR---------IVGYK----GRAV----VVVSCVTKDL--------PYRPHPHNLV--GKEACK------   77
_XP_043491211.1_    41  IR---------IVGYK----GRAV----VVVSCVTKDL--------PYRPHPHNLV--GKEACK------   77
_KAI4495142.1_      41  IR---------IVGYK----GRAV----VVVSCVTKDL--------PYRPHPHNLV--GKEACK------   77
_XP_014609300.1_    41  IR---------IVGYK----GRAV----VVVSCVTKDL--------PYRPHPHNLV--GKEACK------   77
_ADK91833.1_        41  IR---------IVGYK----GRAV----VVVSCVTKDQ--------PHRPHPHNLV--GKEACK------   77
_KAG7213837.1_      41  VR---------IVGYK----GRAV----VVVSCVTKDL--------PHRPHPHNLV--GKEGCK------   77
_XP_014226628.1_    41  IR---------IAGYK----GKAI----CVVSCVTKDP--------PYRPHPHSLV--GKENCR------   77
_XP_020287946.1_    41  IR---------IVGYK----GRAM----VVVSCVTKDS--------PYRPHPHNLV--GKEVCK------   77
_XP_012232007.1_    41  IR---------IVGYK----GRAM----VVVSCVTKDA--------PYRPHPHNLV--GKEVCK------   77
_XP_011874538.1_    41  IR---------IMGYQ----GRAM----VVVSCVTKDA--------PYRPHPHNLV--GKEVCK------   77
_XP_011874541.1_    41  IR---------IMGYQ----GRAM----VVVSCVTKDA--------PYRPHPHNLV--GKEVCK------   77
_XP_011173619.1_    41  IR---------IMGYK----GRAM----VVVSCVTKDA--------PYRPHPHNLV--GKEVCK------   77
_XP_034950793.1_    41  IR---------IVGYE----GRAV----IVISCVTKDE--------PYMPHPHSIV--SKEMCS------   77
_XP_034950792.1_    37  IR---------IVGYE----GRAV----IVISCVTKDE--------PYMPHPHSIV--SKEMCS------   73
_XP_012340880.2_    40  IR---------VIGYK----GRAI----VVASCVTKDK--------PYRPHPHNLI--GREGCK------   76
_XP_031771859.1_    40  IR---------VIGYK----GRAI----VVASCVTKDK--------PYRPHPHNLI--GREGCK------   76
_XP_006618953.1_    40  IR---------VIGYK----GRAI----AVASCVTNDK--------PYRPHPHNLI--GREGCE------   76
```

```
_XP_031367904.1_   40  IR---------VIGYK----GRAI----AVASCVTNDK--------PYRPHPHNLI--GREGCE------   76
_XP_043795948.1_   40  IR---------VIGYK----GRAI----AVASCVTNDK--------PYRPHPHNLI--GREGCE------   76
_XP_395180.5_      40  IR---------VIGFK----GRAL----AVASCVTKDK--------PYRPHPHNLI--GREGCE------   76
_KAG6799500.1_     40  IR---------VIGFK----GRAL----AVASCVTKDK--------PYRPHPHNLI--GREGCE------   76
_XP_016768607.2_   40  IR---------VIGFK----GRAL----AVASCVTKDK--------PYRPHPHNLI--GREGCE------   76
_PBC31989.1_       40  IRVSSEEMKNKVVGYK----GQAI----AVASCVTKDK--------PYRPHPHNLI--GREGCE------   85
_XP_016914450.1_   40  IR---------VVGYK----GQAI----AVASCVTKDK--------PYRPHPHNLI--GREGCE------   76
_KAG5344958.1_     41  IR---------IVGYK----GRAM----VVVSCVTKDA--------PYRPHPHNLV--GKEVCK------   77
_KAJ3630188.1_     39  IR---------VMGYQ----GRAA----VVVSCVTKDE--------PYKPHPYTLV--GQNCK-------   74
_QHN75199.1_       39  IR---------VMGYQ----GRAA----VVVSCVTKDE--------PYKPHPYTLV--GQNCK-------   74
_XP_051171660.1_   40  IR---------VVGYT----GSIT----IVVSCVTVEDNP----NNRPRAHPHKLV--GRAESC------   80
_XP_016909447.1_    1  -----------IVGYK----GRAL----VVVSCVTKDQ--------PYRPHPHNLV--GKEACK------   35
_XP_031773030.1_    1  -----------IVGYK----GRAL----VVVSCVTKDQ--------PYRPHPHNLV--GKEACK------   35
_KAH0956427.1_     42  IK---------IMDYD----GPAV----VIVSCVTSKE--------PYMPHPYNLV--GKCCD-------   77
_XP_012283784.1_   41  IR---------IIGYK----GRAV----VVVSCVTKDL--------PHRPHPHNLV--GKEGCK------   77
_XP_023224928.1_   41  IK---------IKNYK----GAAV----VIVSCVTKDP--------PYYPHPHNLV--GKEGCK------   77
_XP_008556434.1_   41  IR---------IVGYK----GRAV----VVVSCVTKDP--------PYRPHPHNLV--GKEACR------   77
_XP_008556433.1_   41  IR---------IVGYK----GRAV----VVVSCVTKDP--------PYRPHPHNLV--GKEACR------   77
_XP_014299728.1_   41  IR---------IVGYK----GRAV----VVVSCVTKDP--------PYRPHPHNLV--GKEACR------   77
_CAD6234449.1_     41  IR---------IVGYK----GRAV----VVVSCVTKDP--------PYRPHPHNLV--GKEACR------   77
_KAG8037552.1_     41  IR---------IVGYK----GRAV----VVVSCITKDP--------PYRPHPHNLV--GKEACR------   77
_XP_044576138.1_   41  IK---------IVGYK----GRAV----VVVSCVTKDP--------PYRPHPHNLV--GKEACR------   77
_XP_044576125.1_   41  IK---------IVGYK----GRAV----VVVSCVTKDP--------PYRPHPHNLV--GKEACR------   77
_XP_026465448.1_   41  IQ---------IVGYK----GRAF----VVVSCVTKDK--------PYRAHPHNLV--GKEGCK------   77
_XP_026465446.1_   41  IQ---------IVGYK----GRAF----VVVSCVTKDK--------PYRAHPHNLV--GKEGCK------   77
_XP_031785072.1_   41  IR---------IVGYK----GRAI----VVVSCVTKDL--------PYRPHPHNLV--GKEVCR------   77
_XP_015607185.1_   41  IR---------VVGYK----GRAI----AVVSCVTKDV--------PYRPHPHNLV--GKDVCK------   77
_XP_011496611.1_   41  IR---------IVGYK----GRAI----VVVSCVTKDP--------PYRPHPHKLV--GKGVCR------   77
_XP_018903707.1_   41  VK---------INGYR----GRVV----VVVSCVTKDY--------PHKAHPHKLV--GKEGCK------   77
_CAB3365562.1_     41  IK---------VLNYS----GPCM----VVVSCVTKDE--------PFLAHPHNLV--GKDCK-------   76
_XP_011496447.1_       ----------------------------------------------------------------------
_KAG8230996.1_     40  IQ---------VVGYK----GRAV----VVVSCVTKDP--------PYRPHPHNLV--GKEGCK------   76
_XP_040567422.1_   41  IQ---------IVGYH----GPAV----VVVSCVEDKA--------PYRAHPHNLV--GKDGIC------   77
_XP_014250205.1_   41  VQ---------IVGYK----GRAV----VVVSCVTKDQ--------PYKPHPHNLV--GKESCK------   77
_XP_014250206.1_   41  VQ---------IVGYK----GRAV----VVVSCVTKDQ--------PYKPHPHNLV--GKESCK------   77
_XP_047119528.1_   41  IK---------VEGYK----GRAV----VVVSCVTKDP--------PYRPHPHNLV--GKEGCK------   77
_XP_047001845.1_   41  IK---------VEGYK----GRAV----VVVSCVTKDP--------PYRPHPHNLV--GKEGCK------   77
_XP_049939433.1_   41  IK---------VEGYK----GRAV----VVVSCVTKDP--------PYRPHPHNLV--GKEGCK------   77
_XP_049764197.1_   41  IK---------VEGYK----GRAV----VVVSCVTKDP--------PYRPHPHNLV--GKEGCK------   77
```

```
_XP_049833656.1_   41  IK---------VEGYK----GRAV----VVVSCVTKDP--------PYRPHPHNLV--GKEGCK------   77
_XP_049790026.1_   41  IK---------VEGYK----GRAV----VVVSCVTKDP--------PYRPHPHNLV--GKEGCK------   77
_XP_049833651.1_   41  IK---------VEGYK----GRAV----VVVSCVTKDP--------PYRPHPHNLV--GKEGCK------   77
_XP_047001842.1_   41  IK---------VEGYK----GRAV----VVVSCVTKDP--------PYRPHPHNLV--GKEGCK------   77
_XP_049790025.1_   41  IK---------VEGYK----GRAV----VVVSCVTKDP--------PYRPHPHNLV--GKEGCK------   77
_XP_049833654.1_   41  IK---------VEGYK----GRAV----VVVSCVTKDP--------PYRPHPHNLV--GKEGCK------   77
_XP_049833659.1_   41  IK---------VEGYK----GRAV----VVVSCVTKDP--------PYRPHPHNLV--GKEGCK------   77
_XP_021925503.1_   41  IQ---------IVGYK----GPAV----VVVSCVTKDT--------PYRAHPHNLV--GKEGCK------   77
_KDR23857.1_       41  IQ---------IVGYK----GPAV----VVVSCVTKDT--------PYRAHPHNLV--GKEGCK------   77
_ABU96700.1_       41  IQ---------IVGYR----GRAV----VVVSCVTKDS--------PYRPHPHNLV--GKEGCK------   77
_ABU96699.1_       41  IQ---------IVGYR----GRAV----VVVSCVTKDS--------PYRPHPHNLV--GKEGCK------   77
_XP_014275495.1_   41  IQ---------IVGYQ----GRAV----VVVSCVTKDP--------PYRPHPHNLV--GKEGCK------   77
_BBE08137.1_       41  IQ---------IVGYQ----GRAV----VVVSCVTKDP--------PYRPHPHNLV--GKEGCK------   77
_XP_014275494.1_   41  IQ---------IVGYQ----GRAV----VVVSCVTKDP--------PYRPHPHNLV--GKEGCK------   77
_XP_014275493.1_   41  IQ---------IVGYQ----GRAV----VVVSCVTKDP--------PYRPHPHNLV--GKEGCK------   77
_BBE08136.1_       41  IQ---------IVGYQ----GRAV----VVVSCVTKDP--------PYRPHPHNLV--GKEGCK------   77
_KAF6207519.1_     41  IQ---------IVGYK----GKAV----VVVSCVTKDP--------PYRPHPHNLV--GKDNCK------   77
_CAB0015508.1_     41  IQ---------VVGYK----GRAV----VVVSCVTKDP--------PYRPHPHNLV--GKESCK------   77
_CAB0015504.1_     41  IQ---------VVGYK----GRAV----VVVSCVTKDP--------PYRPHPHNLV--GKESCK------   77
_PNF18474.1_       41  IE---------IVGYK----GAAV----VVVSCVTKDR--------PFRAHPHSLV--GKEGCK------   77
_CEG62431.1_       41  IQ---------IVGYK----GPAV----VVVSCVTKDP--------PHRPHPHNLV--GKEGCK------   77
_PSN36690.1_       41  IQ---------IVGYK----GPAV----VVVSCVTKDP--------PHRPHPHNLV--GKEGCK------   77
_XP_046387412.1_   41  IQ---------VVGYK----GRAI----VVVSCVTKDP--------PYRPHPHNLV--GKEGCK------   77
_XP_046387414.1_   41  IQ---------VVGYK----GRAI----VVVSCVTKDP--------PYRPHPHNLV--GKEGCK------   77
_XP_031634874.1_   41  IQ---------VVNYR----GRAV----VVVSCVTKEK--------PYRAHPHKLV--GKDICS------   77
_GFG29240.1_       41  IQ---------VCGYI----GPAV----VVVSCVTKDA--------PYRAHPHNLV--GKEGCK------   77
_BBG28464.1_       41  IQ---------VVGYQ----GRAV----VVVSCVTKDA--------PYRPHPHNLV--GKEGCK------   77
_WCL52334.1_       41  IQ---------VVGYQ----GRAV----VVVSCVTKDA--------PYRPHPHNLV--GKEGCK------   77
_GLH00135.1_       41  IQ---------VVGYQ----GRAV----VVVSCVTKDA--------PYRPHPHNLV--GKEGCK------   77
_CAD7205239.1_     41  IQ---------VVGYK----GRAV----VVVSCVTKDP--------PYRSHPHNLV--GKEGCK------   77
_CAD7431383.1_     41  IQ---------VVGYK----GRAV----VVVSCVTKDP--------PYRSHPHNLV--GKEGCK------   77
_CAD7410836.1_     41  IQ---------VVGYK----GRAV----VVVSCVTKDP--------PYRSHPHNLV--GKEGCK------   77
_CAD7415345.1_     41  IQ---------VVGYK----GRAV----VVVSCVTKDP--------PYRSHPHNLV--GKEGCK------   77
_CAD7606635.1_     41  IQ---------VVGYK----GRAV----VVVSCVTKDP--------PYRSHPHNLV--GKEGCK------   77
_CAD7462957.1_     41  IQ---------VVGYK----GRAV----VVVSCVTKDP--------PYRSHPHNLV--GKEGCK------   77
_XP_012265400.2_   41  IR---------IEGYK----GRAV----VVVSCVTKDP--------PYKPHPHNLV--GKEACR------   77
_XP_046473587.1_   41  IQ---------VENHT----GRAI----VVVSCVTKDP--------PYRPHPHNLV--GKESCD------   77
_XP_046611732.1_   41  IQ---------VEGHT----GRAI----VVVSCVTKDP--------PYRPHPHNLV--GKESCD------   77
_XP_015519750.2_   41  IQ---------VENHT----GRAI----VVVSCVTKDP--------PYRPHPHNLV--GKESCD------   77
```

```
_XP_046611731.1_    41  IQ---------VEGHT----GRAI----VVVSCVTKDP--------PYRPHPHNLV--GKESCD------  77
_XP_046417903.1_    41  IQ---------VEGHT----GRAI----VVVSCVTKDP--------PYRPHPHNLV--GKESCD------  77
_GLG94364.1_        41  IR---------VVGYI----GRAV----VVVSCVTKDS--------PYRPHPHSLV--GKEGCK------  77
_GLG94363.1_        41  IR---------VVGYI----GRAV----VVVSCVTKDS--------PYRPHPHSLV--GKEGCK------  77
_BBG28463.1_        41  IR---------VVGYI----GRAV----VVVSCVTKDS--------PYRPHPHSLV--GKEGCK------  77
_WCL52335.1_        41  IR---------VVGYV----GRAV----VVVSCVTKES--------PYRPHPHSLV--GKEGCK------  77
_GLG94365.1_        41  IR---------VVGYI----GRAV----VVVSCVTKDS--------PYRPHPHSLV--GKEGCK------  77
_XP_002431518.1_    41  IQ---------IVGHN----GRAV----VVVSCVTKDA--------PYRPHPHNLV--GKEGCS------  77
_KAE8746525.1_      41  IQ---------VVGYK----GRAA----VIVSCVTKDP--------PYRPHPHNLV--GKEGCK------  77
_XP_026277770.1_    41  IQ---------VVGYK----GRAA----VIVSCVTKDP--------PYRPHPHNLV--GKEGCK------  77
_KAJ1519446.1_      41  IQ---------ILGGT----GKAA----VIVSCVTKDA--------PHRPHPHNLV--GKEGCK------  77
_ALC78349.1_        41  IQ---------VVGFT----GRAV----VVVSCVTKDR--------PYKPHPHNLV--GKDGCK------  77
_XP_046738772.1_    41  IQ---------VEGYT----GRAI----VVVSCVTKDP--------PYRPHPHNLV--GKESCD------  77
_XP_046417904.1_    41  IQ---------VEGHT----GRAI----VVVSCVTKDP--------PYRPHPHNLV--GKESCD------  77
_CRK94170.1_        41  IQ---------VKNYV----GRAV----VVVSCVTKDA--------PYKPHPHSLV--GREGCR------  77
_CRK94167.1_        41  IQ---------VKNYV----GRAV----VVVSCVTKDA--------PYKPHPHSLV--GREGCR------  77
_KAG5676160.1_      41  IQ---------IKNYV----GKAV----VVVSCVTKDF--------PYKPHPHNLV--GKEGCK------  77
_XP_034245464.1_    41  IQ---------VVGYK----GRAA----VIVSCVTKDP--------PYRAHPHNLV--GKEGCK------  77
_XP_034245457.1_    41  IQ---------VVGYK----GRAA----VIVSCVTKDP--------PYRAHPHNLV--GKEGCK------  77
_KAG8287980.1_      41  IQ---------IVGYK----GRAV----VVVSCVTKEQ--------PHRPHPHNLV--GRDGCK------  77
_XP_044759045.1_    39  IQ---------IIGYK----GKAV----VIVSCVTKDA--------PYRPHPFNID--GKQGCK------  75
_XP_017305229.1_    40  IM---------IRNYV----GDAH----LVVSCVSKDS--------PYRSHPHKLV--SKDNCT------  76
_XP_026675878.1_    40  IM---------IRNYV----GDAH----LVVSCVSKDS--------PYRSHPHKLV--SKDNCT------  76
_KAE8574083.1_      41  IQ---------IVGYQ----GRAV----VVVSCVTKDP--------PYRPHPHNLV--GKEGCK------  77
_XP_037926497.1_    41  IE---------IADYV----GRAV----VVVSCVTKDP--------PYRPHPHNLV--GREGCK------  77
_CAD7079567.1_      41  IE---------IADYV----GRAV----VVVSCVTKDP--------PYRPHPHNLV--GREGCK------  77
_XP_037882485.1_    41  IE---------IVGYK----GRAV----VVVSCVTKDE--------PYRPHPHNLV--GKEGCK------  77
_XP_037037597.1_    41  IQ---------VVGYR----GRAV----VVVSCVTKDP--------PYRPHPHNLV--GKEGCK------  77
_XP_037037592.1_    41  IQ---------VVGYR----GRAV----VVVSCVTKDP--------PYRPHPHNLV--GKEGCK------  77
_KAG4075389.1_      41  IQ---------VVGYR----GRAV----VVVSCVTKDP--------PYRPHPHNLV--GKEGCK------  77
_XP_034101357.1_    41  IE---------ILGYK----GRAV----VVVSCVTKDM--------PYRPHPHNLV--GKEGCK------  77
_XP_026844963.1_    41  IE---------IVGYK----GRAV----VVVSCVTKDT--------PYRPHPHNLV--GKEGCK------  77
_EDW28798.1_        41  IE---------IVGYK----GRAV----VVVSCVTKDT--------PYRPHPHNLV--GKEGCK------  77
_XP_034125966.1_    41  IE---------IVGYK----GRAV----VVVSCVTKDT--------PYRPHPHNLV--GKEGCK------  77
_XP_034666519.1_    41  IE---------IVGYK----GRAV----VVVSCVTKDT--------PYRPHPHNLV--GKEGCK------  77
_XP_001356570.3_    41  IE---------IVGYK----GRAV----VVVSCVTKDT--------PYRPHPHNLV--GKEGCK------  77
_XP_023164577.2_    41  IE---------IVGYK----GRAV----VVVSCVTKDT--------PYRPHPHNLV--GKEGCK------  77
_XP_017153206.1_    41  IE---------IVGYK----GRAV----VVVSCVTKDT--------PYRPHPHNLV--GKEGCK------  77
_XP_030564932.1_    41  IE---------IVGYK----GRAV----VVVSCVTKDT--------PYRPHPHNLV--GKEGCK------  77
```

```
_XP_002059145.1_    41  IE---------IVGYK----GRAV----VVVSCVTKDT--------PYRPHPHNLV--GKEGCK------   77
_EDW04266.1_        41  IE---------IVGYK----GRAV----VVVSCVTKDT--------PYRPHPHNLV--GKEGCK------   77
_XP_017025862.1_    41  IE---------IVGYK----GRAV----VVVSCVTKDT--------PYRPHPHNLV--GKEGCK------   77
_XP_017859907.1_    41  IE---------IVGYK----GRAV----VVVSCVTKDT--------PYRPHPHNLV--GKEGCK------   77
_XP_032584327.1_    41  IE---------IVGYK----GRAV----VVVSCVTKDT--------PYRPHPHNLV--GKEGCK------   77
_XP_016987288.2_    41  IE---------IVGYK----GRAV----VVVSCVTKDL--------PHRPHPHNLV--GKEGCK------   77
_XP_017954173.1_    41  IE---------IVGYK----GRAV----VVVSCVTKDT--------PYRPHPHNLV--GKEGCK------   77
_XP_017854504.1_    41  IE---------IVGYK----GRAV----VVVSCVTKDT--------PYRPHPHNLV--GKEGCK------   77
_XP_034125965.1_    41  IE---------IVGYK----GRAV----VVVSCVTKDT--------PYRPHPHNLV--GKEGCK------   77
_XP_034666515.1_    41  IE---------IVGYK----GRAV----VVVSCVTKDT--------PYRPHPHNLV--GKEGCK------   77
_XP_043641917.1_    41  IE---------IVGYK----GRAV----VVVSCVTKDT--------PYRPHPHNLV--GKEGCK------   77
_XP_026844960.1_    41  IE---------IVGYK----GRAV----VVVSCVTKDT--------PYRPHPHNLV--GKEGCK------   77
_XP_015035988.2_    41  IE---------IVGYK----GRAV----VVVSCVTKDT--------PYRPHPHNLV--GKEGCK------   77
_XP_041450744.1_    41  IE---------IVGYK----GRAV----VVVSCVTKDT--------PYRPHPHNLV--GKEGCK------   77
_XP_015052361.1_    41  IE---------IVGYK----GRAV----VVVSCVTKDT--------PYRPHPHNLV--GKEGCK------   77
_XP_039480515.1_    41  IE---------IVGYK----GRAV----VVVSCVTKDT--------PYRPHPHNLV--GKEGCK------   77
_XP_033248563.1_    41  IE---------IVGYK----GRAV----VVVSCVTKDT--------PYRPHPHNLV--GKEGCK------   77
_KAH8412150.1_      41  IE---------IVGYK----GRAV----VVVSCVTKDT--------PYRPHPHNLV--GKEGCK------   77
_XP_043864974.1_    41  IE---------IVGYK----GRAV----VVVSCVTKDT--------PYRPHPHNLV--GKEGCK------   77
_XP_020809119.1_    41  IE---------IVGYK----GRAV----VVVSCVTKDT--------PYRPHPHNLV--GKEGCK------   77
_KAH8298353.1_      41  IE---------IVGYK----GRAV----VVVSCVTKDT--------PYRPHPHNLV--GKEGCK------   77
_XP_030564927.1_    41  IE---------IVGYK----GRAV----VVVSCVTKDT--------PYRPHPHNLV--GKEGCK------   77
_XP_041631592.1_    41  IE---------IVGYK----GRAV----VVVSCVTKDT--------PYRPHPHNLV--GKEGCK------   77
_XP_017859909.1_    41  IE---------IVGYK----GRAV----VVVSCVTKDT--------PYRPHPHNLV--GKEGCK------   77
_XP_032591602.1_    41  IE---------IVGYK----GRAV----VVVSCVTKDT--------PYRPHPHNLV--GKEGCK------   77
_XP_023164572.2_    41  IE---------IVGYK----GRAV----VVVSCVTKDT--------PYRPHPHNLV--GKEGCK------   77
_TDG46699.1_        41  IE---------IVGYK----GRAV----VVVSCVTKDT--------PYRPHPHNLV--GKEGCK------   77
_KAH8411895.1_      41  IE---------IVGYK----GRAV----VVVSCVTKDT--------PYRPHPHNLV--GKEGCK------   77
_KAH8377496.1_      41  IE---------ILGYK----GRAV----VVVSCVTKDM--------PYRPHPHNLV--GKEGCK------   77
_XP_017854500.2_    41  IE---------IVGYK----GRAV----VVVSCVTKDT--------PYRPHPHNLV--GKEGCK------   77
_XP_016987284.2_    41  IE---------IVGYK----GRAV----VVVSCVTKDL--------PHRPHPHNLV--GKEGCK------   77
_KAH8293209.1_      41  IE---------IMGYK----GRAV----VVVSCVTKDM--------PYRPHPHNLV--GKEGCK------   77
_EEC00225.1_        41  IK---------VMHHQ----GKAA----VVVSCVTKDQ--------PYRVHPHNLV--GKEGCK------   77
_XP_029825505.2_    41  IK---------VMHHQ----GKAA----VVVSCVTKDQ--------PYRVHPHNLV--GKEGCK------   77
_KAG0424790.1_      41  IK---------VMHHQ----GKAA----VVVSCVTKDQ--------PYRVHPHNLV--GKEGCK------   77
_XP_037272127.1_    41  IK---------VMGVQ----KPAV----VVVSCVTKDQ--------PYRVHPHNLV--GKEGCK------   77
_XP_054934017.1_    41  IK---------VMGIQ----KPAV----VVVSCVTKDQ--------PYRVHPHNLV--GKEGCK------   77
_XP_050048524.1_    41  IK---------VMGIQ----KPAV----VVVSCVTKDQ--------PYRVHPHNLV--GKEGCK------   77
_XP_037272126.1_    41  IK---------VMGVQ----KPAV----VVVSCVTKDQ--------PYRVHPHNLV--GKEGCK------   77
```

```
_XP_037524244.1_    41  IK---------VMGVQ----KPAV----VVVSCVTKDQ--------PYRVHPHNLV--GKEGCK------   77
_XP_037524245.1_    41  IK---------VMGVQ----KPAV----VVVSCVTKDQ--------PYRVHPHNLV--GKEGCK------   77
_XP_049512094.1_    41  IK---------VMGIQ----KPAV----VVVSCVTKDQ--------PYRVHPHNLV--GKEGCK------   77
_XP_037524243.1_    41  IK---------VMGVQ----KPAV----VVVSCVTKDQ--------PYRVHPHNLV--GKEGCK------   77
_XP_037580160.1_    41  IK---------VMGIQ----KPAV----VVVSCVTKDQ--------PYRVHPHNLV--GKEGCK------   77
_XP_037272128.1_    41  IK---------VMGVQ----KPAV----VVVSCVTKDQ--------PYRVHPHNLV--GKEGCK------   77
_KAH9380259.1_      41  IK---------VMNVQ----KPAV----VVVSCVTKDQ--------PYRVHPHNLV--GKEGCK------   77
_KAI1291715.1_      14  IR---------IANYS----GPAL----VIVSCVTKDE--------PFKPHPHHIV--GRSCD-------   49
_XP_035209023.1_    42  IQ---------VYNYT----GEFT----LIASCVTCDGP-------PHRPHPHKLC--GSHCS-------   78
_XP_023332679.1_    41  IK---------VHGFQ----GFAT----VVVSCVTHNQD-------PPKAHPHNLV--SPASVPNG----   80
_XP_046801942.1_    41  IE---------IVGYK----GRAV----VVVSCVTKDQ--------PYRPHPHNLV--GKEGCK------   77
_XP_037811120.1_    41  IE---------IVGYK----GRAV----VVVSCVTKDQ--------PYRPHPHNLV--GKEGCK------   77
_AFI98401.1_        41  IE---------IVGYK----GRAV----VVVSCVTKDQ--------PYRPHPHNLV--GKEGCK------   77
_AFJ54621.1_        41  IE---------IVGYK----GRAV----VVVSCVTKDQ--------PYRPHPHNLV--GKEGCK------   77
_KAI8126534.1_      41  IE---------IVGYK----GRAV----VVVSCVTKDQ--------PYRPHPHNLV--GKEGCK------   77
_XP_013118266.1_    41  IE---------IVGYK----GRAV----VVVSCVTKDQ--------PYRPHPHNLV--GKEGCK------   77
_XP_023293634.2_    41  IE---------IVGYK----GRAV----VVVSCVTKDQ--------PYRPHPHNLV--GKEGCK------   77
_XP_037811062.1_    41  IE---------IVGYK----GRAV----VVVSCVTKDQ--------PYRPHPHNLV--GKEGCK------   77
_KNC34132.1_        41  IE---------IVGYK----GRAV----VVVSCVTKDQ--------PYRPHPHNLV--GKEGCK------   77
_TMW48951.1_        41  IE---------IVGYK----GRAV----VVVSCVTKDP--------PYRPHPHNLV--GKEGCK------   77
_XP_037957363.1_    41  IE---------IVGYK----GRAV----VVVSCVTKDM--------PYRPHPHNLV--GKEGCK------   77
_XP_005175014.1_    41  IE---------IVGYK----GRAV----VVVSCVTKDQ--------PYRPHPHNLV--GKEGCK------   77
_XP_037957360.1_    41  IE---------IVGYK----GRAV----VVVSCVTKDM--------PYRPHPHNLV--GKEGCK------   77
_XP_030383330.1_    41  IE---------IVGYK----GRVV----VVVSCVTKDA--------PYRPHPHNLV--GKEGCK------   77
_XP_030383328.1_    41  IE---------IVGYK----GRVV----VVVSCVTKDA--------PYRPHPHNLV--GKEGCK------   77
_CAH1707987.1_      40  IQ---------IANYV----GRAV----VVVSCVTKDF--------PYKPHPHNLV--GKEGCK------   76
_TMW54796.1_        41  IE---------IVGYK----GRAI----VVVSCVTKDP--------PYRPHPHNLV--GKEGCK------   77
_XP_026836077.1_    41  IE---------IVGYK----GRAV----VVVSCVTKDT--------PYRPHPHNLV--GKEGCK------   77
_XP_017116803.1_    41  IE---------IVGYK----GRAV----VVVSCVTKDT--------PYRPHPHNLV--GKEGCK------   77
_XP_039480519.1_    41  IE---------IVGYK----GRAV----VVVSCVTKDT--------PYRPHPHNLV--GKEGCK------   77
_XP_043641920.1_    41  IE---------IVGYK----GRAV----VVVSCVTKDT--------PYRPHPHNLV--GKEGCK------   77
_XP_023036748.1_    41  IE---------IVGYK----GRAV----VVVSCVTKDT--------PYRPHPHNLV--GKEGCK------   77
_XP_002090466.1_    41  IE---------IVGYK----GRAV----VVVSCVTKDT--------PYRPHPHNLV--GKEGCK------   77
_XP_034471824.1_    41  IE---------IVGYK----GRAV----VVVSCVTKDT--------PYRPHPHNLV--GKEGCK------   77
_AAT94434.1_        41  IE---------IVGYK----GRAV----VVVSCVTKDT--------PYRPHPHNLV--GKEGCK------   77
_XP_036670985.1_    41  IE---------IVGYK----GRAV----VVVSCVTKDT--------PYRPHPHNLV--GKEGCK------   77
_AAA28479.1_        41  IE---------IVGYK----GRAV----VVVSCVTKDT--------PYRPHPHNLV--GKEGCK------   77
_XP_037708206.1_    41  IE---------IVGYK----GRAV----VVVSCVTKDT--------PYRPHPHNLV--GKEGCK------   77
_XP_002038915.1_    41  IE---------IVGYK----GRAV----VVVSCVTKDT--------PYRPHPHNLV--GKEGCK------   77
```

```
_XP_016993636.1_   41  IE---------IVGYK----GRAV----VVVSCVTKDT--------PYRPHPHNLV--GKEGCK------   77
_XP_052852517.1_   41  IE---------IVGYK----GRAV----VVVSCVTKDT--------PYRPHPHNLV--GKEGCK------   77
_XP_016963139.1_   41  IE---------IVGYK----GRAV----VVVSCVTKDT--------PYRPHPHNLV--GKEGCK------   77
_XP_033156151.1_   41  IE---------IVGYK----GRAV----VVVSCVTKDT--------PYRPHPHNLV--GKEGCK------   77
_XP_001974446.1_   41  IE---------IVGYK----GRAV----VVVSCVTKDT--------PYRPHPHNLV--GKEGCK------   77
_XP_014761886.1_   41  IE---------IVGYK----GRAV----VVVSCVTKDV--------PHRPHPHNLV--GKEGCK------   77
_XP_017107216.2_   41  IE---------IVGYK----GRAV----VVVSCVTKDV--------PHRPHPHNLV--GKEGCK------   77
_XP_017044881.1_   41  IE---------IVGYK----GRAV----VVVSCVTKDT--------PHRPHPHNLV--GKEGCK------   77
_XP_016944798.1_   41  IE---------IVGYK----GRAV----VVVSCVTKDT--------PYRPHPHNLV--GKEGCK------   77
_XP_017116800.1_   41  IE---------IVGYK----GRAV----VVVSCVTKDT--------PYRPHPHNLV--GKEGCK------   77
_XP_016993633.2_   41  IE---------IVGYK----GRAV----VVVSCVTKDT--------PYRPHPHNLV--GKEGCK------   77
_XP_037708198.1_   41  IE---------IVGYK----GRAV----VVVSCVTKDT--------PYRPHPHNLV--GKEGCK------   77
_XP_032583259.1_   41  IE---------IVGYK----GRAV----VVVSCVTKDT--------PYRPHPHNLV--GKEGCK------   77
_AAC35296.1_       41  IE---------IVGYK----GRAV----VVVSCVTKDT--------PYRPHPHNLV--GKEGCK------   77
_NP_001286014.1_   41  IE---------IVGYK----GRAV----VVVSCVTKDT--------PYRPHPHNLV--GKEGCK------   77
_XP_016963136.1_   41  IE---------IVGYK----GRAV----VVVSCVTKDT--------PYRPHPHNLV--GKEGCK------   77
_XP_017063904.2_   41  IE---------IVGYK----GRAV----VVVSCVTKDM--------PYRPHPHNLV--GKEGCK------   77
_XP_033156135.1_   41  IE---------IVGYK----GRAV----VVVSCVTKDT--------PYRPHPHNLV--GKEGCK------   77
_XP_052852512.1_   41  IE---------IVGYK----GRAV----VVVSCVTKDT--------PYRPHPHNLV--GKEGCK------   77
_XP_032307758.1_   41  IE---------IVGYK----GRAV----VVVSCVTKDV--------PHRPHPHNLV--GKEGCK------   77
_XP_017107215.2_   41  IE---------IVGYK----GRAV----VVVSCVTKDV--------PHRPHPHNLV--GKEGCK------   77
_AWT86616.1_       40  IQ---------IEGYK----GRAV----VVVSCVTKDS--------PYKPHPHSLV--GKEGCK------   76
_QUF98534.1_       40  IQ---------IENYK----GRAV----VVVSCVTKDS--------PYKPHPHSLV--GKEGCK------   76
_UNA06287.1_       40  IQ---------IEGYK----GRAV----VVVSCVTKDS--------PYKPHPHSLV--GKEGCK------   76
_AAQ65068.1_       41  IE---------IVGYK----GRAV----VVVSCVTKDT--------PYRPHPHNLV--GKEGCK------   77
_AAQ64916.1_       41  IE---------IVGYK----GRAV----VVVSCVTKDT--------PYRPHPHNLV--GKEGCK------   77
_AAQ64921.1_       41  IE---------IVGYK----GRAV----VVVSCVTKDT--------PYRPHPHNLV--GKEGCK------   77
_AAQ64918.1_       41  IE---------IVGYK----GRAV----VVVSCVTKDT--------PYRPHPHNLX--GKEGXK------   77
_KAH8245459.1_     41  IE---------IVGYK----GRAV----VVVSCVTKDT--------PYRPHPHNLV--GKEGCK------   77
_KAH8283155.1_     41  IE---------IVGYK----GRAV----VVVSCVTKDT--------PYRPHPHNLV--GKEGCK------   77
_KAH8246384.1_     41  IE---------IVGYK----GRAV----VVVSCVTKDT--------PYRPHPHNLV--GKEGCK------   77
_KAH8366418.1_     41  IE---------IVGYK----GRAV----VVVSCVTKDT--------PYRPHPHNLV--GKEGCK------   77
_XP_022195378.2_   40  IQ---------IKNYV----GRAV----VVVSCVTKDS--------PYRPHPHSLV--GKEGCK------   76
_XP_039284931.1_   40  IQ---------IKNYV----GRAV----VVVSCVTKDS--------PYRPHPHSLV--GKEGCK------   76
_ALC40091.1_       41  IE---------IVGYK----GRAV----VVVSCVTKDT--------PYRPHPHNLV--GKEGCK------   77
_XP_036342419.1_   41  IE---------IVGYQ----GRAV----VVVSCVTKDA--------PYRPHPHNLA--GKEGCK------   77
_XP_022903025.1_   40  IK---------VEGYK----GKAV----VVVSCVTKDE--------PFRPHPHKLV--GREGCK------   76
_XP_022903017.1_   40  IK---------VEGYK----GKAV----VVVSCVTKDE--------PFRPHPHKLV--GREGCK------   76
_XP_036218946.1_   41  IE---------IVGYK----GRSV----CVVSCVSKDP--------PYRPHPHTLV--GKEGCK------   77
```

```
_XP_018790401.1_   41  IE---------IVGYK----GRSV----CVVSCVSADP--------PYRPHPHTLV--GKEGCK------   77
_KAI9579887.1_     40  IR---------IIGYK----GPAV----VIVSCVTKDE--------PYRPHPHRLV--GLKCS-------   75
_XP_037892537.1_   40  IR---------IIGYK----GPAV----VIVSCVTKDE--------PYRPHPHRLV--GLKCS-------   75
_XP_018903705.1_   48  IQ---------INGYR----GRVV----VVVSCVTKDY--------PHKAHPHKLV--GKEGCK------   84
_CAH0395667.1_     48  IQ---------INGYR----GRVV----VVVSCVTKDY--------PHKAHPHKLV--GKEGCK------   84
_XP_050330165.1_   41  IE---------IVGYK----GRSV----CVVSCVSADP--------PYRPHPHTLV--GKKGCK------   77
_XP_039970878.1_   41  IE---------IVGYK----GRSV----CVVSCVSADP--------PYRPHPHTLV--GKKGCK------   77
_XP_049305234.1_   41  IE---------IVGYK----GRSV----CVVSCVSADP--------PYRPHPHTLV--GKEGCK------   77
_GLH04793.1_       41  IE---------VLGLPD--NIRAV----ALVSCVTRDA--------PYRPHPHAVV--GGRGELGGI---   82
_XP_015835035.1_   39  IQ---------IVGYQ----GRAA----VVVSCVTKDE--------PHKPHPHKLV--GEHCK-------   74
_XP_015835036.1_   39  IQ---------IVGYQ----GRAA----VVVSCVTKDE--------PHKPHPHKLV--GEHCK-------   74
_XP_039283832.1_   49  IE---------VMNLK----GRAI----VVVCCVTVDL--------PHKPHPHRLV--GLNCHPK-----   86
_XP_031788427.1_   40  IK---------IENYK----GPAQ----VVVSCVSKDP--------PYYPHPHNLVSTGKQVS-------   77
_KAJ6646894.1_     40  FR---------LLNYT----GHAV----AIISCVSKDT--------PYRPHPTAIV--GKPHCN------   76
_XP_026466084.1_    1  ---------------------------------------------------PHNLV--GKDWCT------   11
_XP_044002069.1_   35  LRI--------HGGYE----GPAL----LFASCVTKNQDD----YKKFRPHPHKII--GKNCNS------   76
_XP_046611735.1_   41  IQ---------IEGYE----GSVA----VVVSCVTKDS--------PYKPHPHQLV--GNDFCK------   77
_XP_046417906.1_   41  IQ---------IEGYE----GSVA----VVVSCVTKDS--------PYKPHPHQLV--GNDFCK------   77
_XP_046590309.1_   41  IQ---------IEGYE----GSVA----VVVSCVAKDS--------PYKPHPHQLV--GNDFCK------   77
_XP_046473589.1_   41  IQ---------IEGYE----GSVA----VVVSCVAKDS--------PYKPHPHQLV--GNDFCK------   77
_XP_019892116.1_       ----------------------------------------------------------------------
_XP_005183027.3_       ----------------------------------------------------------------------
_XP_011496598.1_    1  -----------------------------------------------------------KELS-------    4
_AAW67216.1_           ----------------------------------------------------------------------
_AAW67217.1_           ----------------------------------------------------------------------
_XP_017489788.1_       ----------------------------------------------------------------------
_BAI67894.1_           ----------------------------------------------------------------------
_XP_054741511.1_   41  IE---------IVGYK----GRAV----VVVSCVTKDA--------PYRPHPHNLV--GKEGCK------   77
_XP_053961684.1_   41  IE---------IVGYK----GRAV----VVVSCVTKDA--------PYRPHPHNLV--GKEGCK------   77
_XP_036341313.1_   41  IE---------IVGYK----GRAV----VVVSCVTKDA--------PYRPHPHNLV--GKEGCK------   77
_XP_036341343.1_   41  IE---------IVGYK----GRAV----VVVSCVTKDA--------PYRPHPHNLV--GKEGCK------   77
_XP_017471383.1_   41  IE---------IVGYK----GRAV----VVVSCVTKDA--------PYRPHPHNLV--GKEGCK------   77
_XP_054741498.1_   41  IE---------IVGYK----GRAV----VVVSCVTKDA--------PYRPHPHNLV--GKEGCK------   77
_XP_053961677.1_   41  IE---------IVGYK----GRAV----VVVSCVTKDA--------PYRPHPHNLV--GKEGCK------   77
_XP_050320008.1_   41  IE---------IVGYK----GRAV----VVVSCVTKDA--------PYRPHPHNLV--GKEGCK------   77
_XP_028894490.2_   41  IE---------IVGYK----GRAV----VVVSCVTKDA--------PYRPHPHNLV--GKEGCK------   77
_XP_050320015.1_   41  IE---------IVGYK----GRAV----VVVSCVTKDA--------PYRPHPHNLV--GKEGCK------   77
_XP_018787554.1_   41  IE---------IVGYK----GRAV----VVVSCVTKDA--------PYRPHPHNLV--GKEGCK------   77
_XP_029407438.2_   41  IE---------IVGYK----GRAV----VVVSCVTKDA--------PYRPHPHNLV--GKEGCK------   77
```

```
_XP_036225551.1_    41  IE---------IVGYK----GRAV----VVVSCVTKDA--------PYRPHPHNLV--GKEGCK------  77
_XP_011178374.2_    41  IE---------IVGYK----GRAV----VVVSCVTKDA--------PYRPHPHNLV--GKEGCK------  77
_XP_039952593.1_    41  IE---------IVGYK----GRAV----VVVSCVTKDA--------PYRPHPHNLV--GKEGCK------  77
_XP_018787542.1_    41  IE---------IVGYK----GRAV----VVVSCVTKDA--------PYRPHPHNLV--GKEGCK------  77
_XP_011207866.2_    41  IE---------IVGYK----GRAV----VVVSCVTKDA--------PYRPHPHNLV--GKEGCK------  77
_XP_014086506.1_    41  IE---------IVGYK----GRAV----VVVSCVTKDA--------PYRPHPHNLV--GKEGCK------  77
_XP_004526638.1_    41  IE---------IVGYK----GRAV----VVVSCVTKDA--------PYRPHPHNLV--GKEGCK------  77
_XP_017471402.1_    41  IE---------IVGYK----GRAV----VVVSCVTKDA--------PYRPHPHNLV--GKEGCK------  77
_XP_012157903.1_    41  IE---------IVGYK----GRAV----VVVSCVTKDA--------PYRPHPHNLV--GKEGCK------  77
_XP_011185661.2_    41  IE---------IVGYK----GRSV----CVVSCVTKDP--------PFKPHPHNLV--GKEGCK------  77
_XP_050322757.1_    41  IE---------IVGYK----GPSV----CVVSCVSTDH--------PYRPHPHTLV--GKEGCK------  77
_XP_039962922.1_    41  IE---------IVGYK----GPSV----CVVSCVSTDH--------PYRPHPHTLV--GKEGCK------  77
_XP_050322739.1_    41  IE---------IVGYK----GPSV----CVVSCVSTDH--------PYRPHPHTLV--GKEGCK------  77
_XP_050322749.1_    41  IE---------IVGYK----GPSV----CVVSCVSTDH--------PYRPHPHTLV--GKEGCK------  77
_XP_050322724.1_    41  IE---------IVGYK----GPSV----CVVSCVSTDH--------PYRPHPHTLV--GKEGCK------  77
_XP_050322682.1_    41  IE---------IVGYK----GPSV----CVVSCVSTDH--------PYRPHPHTLV--GKEGCK------  77
_CAG9823112.1_      41  VQ---------IVGYQ----GRAV----VVVSCVTKDA--------PHKPHPYKLV--SKSNNKD-----  78
_KAG5873705.1_      41  IQ---------IVGYQ----GKAV----VVVSCVTKDE--------PHKPHPHNLV--GREGCK------  77
_KAH8030364.1_      29  -----------VMGVQ----KPAV----VVVSCVTKDQ--------PYRVHPHNLV--GKEGCK------  63
_XP_023013891.1_    41  IQ---------IVGYQ----GKAV----VVVSCVTKDN--------PHKPHPHNLV--GREGCR------  77
_XP_028130780.2_    41  IR---------VEGYQ----GRAV----VVVSCVTRDQ--------PYRPHPYNLV--SKANNKE-----  78
_KAG5873704.1_      41  IQ---------IVGYQ----GKAV----VVVSCVTKDE--------PHKPHPYKLV--SKGNIKE-----  78
_CAH1187690.1_      41  IR---------VEGYQ----SRAV----VVVSCVTRDE--------PYKPHPYKLV--SKSNKEC-----  78
_CAH1098641.1_      41  IR---------VEGYQ----GRAV----VVVSCVTRDA--------PYKPHPYKLV--SKAKNTE-----  78
_XP_023028116.1_    41  IQ---------IVGYK----GRAI----VQVSCVTKDP--------PYRAHPFNLV--SKANNTD-----  78
_XP_031630227.1_    41  IE---------ISNCT----GFVA----ICISCVTNEI--------RPKQHPHKLA--TKSLSN------  77
_KAG5861047.1_          ----------------------------------------------------------------------
_KAF2880990.1_      41  IQ---------IAGYQ----GRAV----VVVSCVTKDA--------PHRPHPHNLV--GREGCK------  77
_XP_018326789.1_    41  IQ---------VIGYK----GRAV----VVVSCVTKDP--------PYRPHPHNLV--GRDGCR------  77
_XP_018326788.1_    41  IQ---------VIGYK----GRAV----VVVSCVTKDP--------PYRPHPHNLV--GRDGCR------  77
_XP_031339184.1_    41  IQ---------IVGYQ----GRAV----VVVSCVTKDS--------PHRPHPHNLV--GREGCK------  77
_XP_045476069.1_    41  IQ---------VVGYQ----GRAV----VVVSCVTKDQ--------PYRPHPHNLV--GREGCK------  77
_XP_044734149.1_    41  IR---------VVGYE----GKAV----VVVSCVTKDK--------PYKPHPHNLV--GKEGCK------  77
_XP_044734150.1_    41  IR---------VVGYE----GKAV----VVVSCVTKDK--------PYKPHPHNLV--GKEGCK------  77
_XP_044734148.1_    41  IR---------VVGYE----GKAV----VVVSCVTKDK--------PYKPHPHNLV--GKEGCK------  77
_XP_044760528.1_    41  IQ---------VVGYQ----GRAV----VVVSCVTKDQ--------PYRPHPHNLV--GREGCK------  77
_XP_044747918.1_    41  IQ---------VVGYH----GRAV----VVVSCVTRDE--------PYRPHPHNLV--GREGCK------  77
_XP_044747917.1_    41  IQ---------VVGYH----GRAV----VVVSCVTRDE--------PYRPHPHNLV--GREGCK------  77
_XP_045481017.1_    41  IQ---------VVGYN----GRAV----VVVSCVTRDE--------PFRPHPHNLV--GREGCK------  77
```

```
_XP_045481018.1_    41  IQ---------VVGYN----GRAV----VVVSCVTRDE--------PFRPHPHNLV--GREGCK------   77
_KAF5302274.1_      41  IQ---------IVGYQ----GRAV----VVVSCVTKDL--------PHRPHPHNLV--GREGCK------   77
_KAF5308883.1_      41  IQ---------IVGYQ----GRAV----VVVSCVTKDL--------PHRPHPHNLV--GREGCK------   77
_KRT79621.1_        40  IK---------ITGYK----GKAI----VVVSCVTKDE--------PYRPHPHHLV--GREGCK------   76
_BAD20728.1_        40  IK---------ITGYK----GKAI----VVVSCVTKDE--------PYRPHPHHLV--GREGCK------   76
_KAF2880988.1_      40  IQ---------ITGYQ----GRAI----VVVSCVTKDA--------PHRPHPYNIV--GKEGCK------   76
_CAH1403258.1_      34  EI----------VGYQ----GRAV----VVVSCVTKDE--------PYRPHPHNLV--GKEGCK------   69
_CAD6992094.1_      41  IE---------IVGYK----GASV----VVVSCVSKDA--------PYRPHPYNLI--GREGCR------   77
_XP_004537184.1_    41  IE---------IVGYK----GASV----VVVSCVSKDA--------PYRPHPYNLI--GREGCR------   77
_XP_018572644.1_    41  IQ---------IVGHQ----GRAV----VVVSCVTKDA--------PHRPHPHNLV--GREGCK------   77
_RZB54393.1__       41  IQ---------VMGYQ----GRAV----VVVSCVTKDE--------PYRPHPHNLV--GREGCK------   77
_XP_044255725.1_    41  IQ---------VVGYQ----GRAV----VVVSCVTKDE--------PYRPHPHNLV--GREGCK------   77
_EFA02850.1_        41  IQ---------VMGYQ----GRAV----VVVSCVTKDE--------PFRPHPHNLV--GREGCK------   77
_NP_001034507.1_    41  IQ---------VMGYQ----GRAV----VVVSCVTKDE--------PFRPHPHNLV--GREGCK------   77
_CAH1370311.1_      41  IQ---------VVGYK----GRAV----VVVSCVTKDE--------PYRPHPHNLV--GREGCK------   77
_KAJ3630692.1_      41  IQ---------VVGYK----GRAV----VVVSCVTKDE--------PYRPHPHNLV--GREGCK------   77
_XP_044255724.1_    41  IQ---------VVGYQ----GRAV----VVVSCVTKDE--------PYRPHPHNLV--GREGCK------   77
_KAH0811528.1_      41  IQ---------VVGYK----GRAV----VVVSCVTKDE--------PYRPHPHNLV--GREGCK------   77
_CAH7716406.1_      41  IR---------VVGYS----GNAW----VLVSCVTKDS--------PYRPHPHNLV--GREGCT------   77
_CAH7716405.1_      41  IR---------VVGYS----GNAW----VLVSCVTKDS--------PYRPHPHNLV--GREGCT------   77
_CAH7716404.1_      41  IR---------VVGYS----GNAW----VLVSCVTKDS--------PYRPHPHNLV--GREGCT------   77
_XP_028130776.2_    41  IR---------VEGYQ----GRAV----VVVSCVTRDQ--------PYRPHPHNLV--GREGCK------   77
_CAH1985684.1_      41  IR---------VVGYT----GSAW----VLVSCVTKDS--------PYRPHPHNLV--GREGCT------   77
_XP_017781152.1_    41  IQ---------IDGYK----GKAV----VVVSCVTKDH--------PHRPHPHNLV--GRDGCK------   77
_XP_017781151.1_    41  IQ---------IDGYK----GKAV----VVVSCVTKDH--------PHRPHPHNLV--GRDGCK------   77
_XP_048522053.1_    41  IQ---------IVGYQ----GRAV----VVVSCVTKDE--------PYMVHPHNLV--GKDCK-------   76
_XP_050303070.1_    41  IQ---------VVGCDPGILRNAV----VVVSCVTKDP--------PYLAHPHNLV--GRDNCK------   81
_CAG9762924.1_      41  IE---------VVGYK----GKAV----VVVSCVTKDI--------PYMVHPHNLV--GKEGCK------   77
_CAH0560654.1_      41  IK---------VVGKPA---KKAV----VVVSCVTKDA--------PHKPHPHNLV--GREGCK------   78
_CAH0560655.1_      41  IK---------VVGKPA---KKAV----VVVSCVTKDA--------PHKPHPHNLV--GREGCK------   78
_XP_018572643.1_    41  IQ---------IVGHQ----GRAV----VVVSCVTKDA--------PHRPHPHNIV--GRENCR------   77
_XP_030763656.1_    41  IQ---------VEGYR----GKAV----VVVSCVMKDP--------PYAVHPHKLV--GREGCR------   77
_XP_030763654.1_    41  IQ---------VEGYR----GKAV----VVVSCVMKDP--------PYAVHPHKLV--GREGCR------   77
_XP_018577945.1_    41  IQ---------IVGHR----GRAM----VVVSCVTKDT--------PHRPHPHNLV--GKEGCK------   77
_KAF7283276.1_      41  IQ---------VEGYQ----GPAV----VVVSCVMKDY--------PYAVHPHNLV--GREGCK------   77
_KAF7283275.1_      41  IQ---------VEGYQ----GPAV----VVVSCVMKDY--------PYAVHPHNLV--GREGCK------   77
_XP_030763617.1_    41  IE---------VVGYK----GKAV----VVVFCVMKDP--------PYTVHPHNLV--GKEGCK------   77
_XP_030763619.1_    45  IQ---------VEGYR----GNAL----VVVSLVMKDP--------PYAAHPHKIV--GKGCR-------   80
_VEN60240.1_            ----------------------------------------------------------------------
```

```
_KOB78236.1_        28  IK---------VNNFV----GPAI----IRVSCVTKDA--------PHRPHPHKLV--GKSNKS------  64
_ERL85515.1_         5  --------------------------------C--------------YRVHPHNLV--GKDCK-------  19
_ENN78766.1_         1  -----------------------------------------------YRVHPHNLV--GKDCK-------  14
_KAH1019371.1_       1  -----------------------------------------------YRVHPHNLV--GKDCK-------  14
_KAI9565420.1_      41  IQ---------VLGYK----GKAV----VVVSCVTKDF--------PYKPHPHSLV--GKEGCK------  77
_XP_045024905.1_    41  IQ---------VLGYK----GKAV----VVVSCVTKDF--------PYKPHPHSLV--GKEGCK------  77
_XP_046447094.1_    41  IQ---------VLGYR----GKAV----VVVSCVTKDF--------PYKPHPHSLV--GKEGCK------  77
_KZS08518.1_        41  IQ---------VLGYK----GKAV----VVVSCVTKDF--------PYKPHPHSLV--GKEGCK------  77
_XP_046636307.1_    41  IQ---------VLGYR----GKAV----VVVSCVTKDF--------PYKPHPHSLV--GKEGCK------  77
_EFX79013.1_        41  IQ---------VLGYR----GKAV----VVVSCVTKDF--------PYKPHPHSLV--GKEGCK------  77
_XP_032776606.2_    41  IQ---------VLGYK----GKAV----VVVSCVTKDF--------PYKPHPHSLV--GKEGCK------  77
_XP_046447093.1_    41  IQ---------VLGYR----GKAV----VVVSCVTKDF--------PYKPHPHSLV--GKEGCK------  77
_XP_046636306.1_    41  IQ---------VLGYR----GKAV----VVVSCVTKDF--------PYKPHPHSLV--GKEGCK------  77
_CAH0099799.1_      41  IQ---------VLGYR----GKAV----VVVSCVTKDF--------PYKPHPHSLV--GKEGCK------  77
_KAJ1531033.1_      39  IQ---------IQHYKG---PSIA----VVVSCVSKDL--------PVRAHPHSLV--GENCK-------  75
_ACJ36225.1_        41  IQ---------VVGYK----GPAV----VVVSCVTVDP--------PYRPHPHNLV--GKEGCK------  77
_ACZ98167.1_        41  IQ---------VVGYK----GPAV----VVVSCVTVDP--------PYRPHPHNLV--GKEGCK------  77
_AME17867.1_        41  IQ---------VVGYK----GPAV----VVVSCVTVDP--------PYRPHPHNLV--GKEGCK------  77
_XP_042868409.1_    41  IQ---------VVGYK----GPAV----VVVSCVTVDP--------PYRPHPHNLV--GKEGCK------  77
_XP_045102711.1_    41  IQ---------VMGYK----GPAV----VVVSCVTVDP--------PYRPHPHNLV--GKEGCK------  77
_XP_047487558.1_    41  IQ---------VVGYK----GPAV----VVVSCVTVDP--------PYRPHPHNLV--GKEGCK------  77
_QBJ05651.1_        41  IQ---------VMGYK----GPAV----VVVSCVTVDP--------PYRPHPHNLV--GKEGCK------  77
_AZK36046.1_        41  IQ---------VMGYK----GPAV----VVVSCVTVDP--------PYRPHPHNLV--GKEGCK------  77
_XP_045581920.1_    41  IQ---------VMGYK----GPAV----VVVSCVTVDP--------PYRAHPHNLV--GKEGCK------  77
_XP_050704598.1_    41  IQ---------VMGYK----GPAV----VVVSCVTVDP--------PYRPHPHNLV--GKEGCK------  77
_XP_027237995.1_    41  IQ---------VVGYK----GPAV----VVVSCVTVDP--------PYRPHPHNLV--GKEGCK------  77
_AXB88326.1_        41  IQ---------VMGYK----GPAV----VVVSCVTVDP--------PYRPHPHNLV--GKEGCK------  77
_XP_045102712.1_    41  IQ---------VMGYK----GPAV----VVVSCVTVDP--------PYRPHPHNLV--GKEGCK------  77
_MPC17461.1_        41  IQ---------VMGYK----GPAV----VVVSCVTVDP--------PYRPHPHNLV--GKEGCK------  77
_XP_027237994.1_    41  IQ---------VVGYK----GPAV----VVVSCVTVDP--------PYRPHPHNLV--GKEGCK------  77
_XP_027237996.1_    41  IQ---------VVGYK----GPAV----VVVSCVTVDP--------PYRPHPHNLV--GKEGCK------  77
_XP_045102715.1_    41  IQ---------VMGYK----GPAV----VVVSCVTVDP--------PYRPHPHNLV--GKEGCK------  77
_XP_053627990.1_    41  IQ---------VMGYK----GPAV----VVVSCVTVDP--------PYRPHPHNLV--GKEGCK------  77
_XP_053627989.1_    41  IQ---------VMGYK----GPAV----VVVSCVTVDP--------PYRPHPHNLV--GKEGCK------  77
_XP_053627983.1_    41  IQ---------VMGYK----GPAV----VVVSCVTVDP--------PYRPHPHNLV--GKEGCK------  77
_KAB7495058.1_      41  IK---------IMNYK----GPAV----VVVSCVTVDP--------PYRPHPHNLV--GKEGCS------  77
_KAF2353654.1_      41  IQ---------VMNYT----GPAV----VVVSCVTVDP--------PHRAHPHNLV--GKEGCK------  77
_ANH22235.1_        41  IQ---------VVGYK----GPAV----VVVSCVTVDP--------PYRPHPHNLV--GKEGCK------  77
_CAD7275741.1_      40  IQ---------VMGYH----GPAV----VVVSCVTKDP--------PFRPHPHNLV--GREGCD------  76
```

```
_XP_042243403.1_    41  IQ---------VVGYK----GPAV----VVVSCVTVNP--------PYRAHPHNLV--GKEGCK------   77
_QTC09942.1_        41  IQ---------VVGYK----GPAV----VVVSCVTVDP--------PYRPHPHNLV--GKEGCK------   77
_XP_018023738.1_    41  IQ-------------------VRT----IGIQVRTIGI--------QVRAI-------GIQVRTI-----   67
_XP_019877552.1_    41  IK---------VVGGPP---RKAV----VVVSCVTKDH--------PYRPHPHKLV--GREGCK------   78
_XP_049825875.1_    41  IK---------LVGGPP---RKAV----VVVSCVTKDQ--------PHRPHPHKLV--GRDGCE------   78
_CAD7649074.1_      41  IQ---------VCGYR----GQAV----VVVSCVTKEQ--------PYRPHPHNLV--GKEGCK------   77
_CAD7630012.1_      41  IQ---------VCNYK----GQAV----VVVSCVTKDT--------PYRPHPHNLV--GKEGCK------   77
_XP_054165343.1_    41  IQ---------VCNYRG---GAAV----VVVSCVTKEQ--------PYRPHPHNLV--GKEGCK------   78
_XP_044254219.1_    39  IQ---------IVGYQ----GRIA----VVVSCVTKEE--------PYKPHPHKLV--GEHCK-------   74
_XP_053201827.1_    41  IQ---------IESFK----GPAV----VVVSCVTKDF--------PYRPHPHNLV--GKDGCK------   77
_XP_015786990.1_    41  IQ---------IESFK----GPAV----VVVSCVTKDF--------PYRPHPHNLV--GKDGCK------   77
_KAI1295507.1_      41  IQ---------IMNYR----GPAV----VVVSCVTKDL--------PYRPHPHNLV--GKEGCK------   77
_RWS07527.1_        41  IQ---------VVNYK----GPAV----VVVSCVTKDF--------PHRPHPHNLV--GKEGCK------   77
_RWS29859.1_        41  IQ---------VVNYK----GPAV----VVVSCVTKDY--------PYRPHPHNLV--GKEGCK------   77
_XP_053201825.1_    41  IQ---------IESFK----GPAV----VVVSCVTKDF--------PYRPHPHNLV--GKDGCK------   77
_XP_054713467.1_    42  IQ---------VINCD----IPFV----VIVSCVTKDVYRVE-NRKGYLAHPHNLI--GINCK-------   84
_XP_046915158.1_    41  IQ---------VCNYH----GKAV----VVVSCVTKEP--------PYRPHPHNLV--GKEGCK------   77
_KAJ6217218.1_      41  IQ---------VCNYN----GKAV----VVVSCVTKDP--------PYRPHPHNLV--GKEGCK------   77
_KAI2801352.1_      41  IQ---------VCNYN----GKAV----VVVSCVTKDP--------PYRPHPHNLV--GKEGCK------   77
_KAH9406651.1_      41  IQ---------VCNYR----GKAV----VVVSCVTKDP--------PYRPHPHNLV--GKEGCK------   77
_XP_017483538.1_    41  IQ---------VCNYR----GKAV----VVVSCVTKDP--------PYRPHPHNLV--GKEGCK------   77
_KPM10359.1_        41  IQ---------VCNYR----GKAV----VVVSCVTKEP--------PYRPHPHNLV--GKEGCK------   77
_UXI17582.1_        41  IQ---------VCNYR----GKAV----VVVSCVTKEP--------PYRPHPHNLV--GKEGCK------   77
_KAF7488016.1_      41  IQ---------VCNYR----GKAV----VVVSCVTKEP--------PYRPHPHNLV--GKEGCK------   77
_KAH9423377.1_      41  IK---------VFNYQ----GKAV----VVVSCVTKES--------PYRPHPHNLV--GKEGCK------   77
_OTF73464.1_        41  IK---------VYNYH----GKAV----VVVSCVTKEP--------PYRPHPHNLV--GKEGCK------   77
_XP_037072056.1_    41  VK---------VVNYR----GPAV----VVVSCVTKDP--------PHWPHPHNLV--GKDGCK------   77
_XP_037083492.1_    41  VK---------VVNYQ----GPAV----VVVSCVTKDP--------PYRPHPHNLV--GKDGCK------   77
_XP_037083508.1_    41  VK---------VVNYQ----GPAV----VVVSCVTKDP--------PYRPHPHNLV--GKDGCK------   77
_XP_043215018.1_    41  VR---------VVGYQ----GPAV----VVVSCVTKDP--------PYRPHPHNLV--GKDGCK------   77
_KAF0314765.1_      41  VR---------VVGYQ----GPAV----VVVSCVTKDP--------PYRPHPHNLV--GKDGCK------   77
_XP_043215020.1_    41  VR---------VVGYQ----GPAV----VVVSCVTKDP--------PYRPHPHNLV--GKDGCK------   77
_XP_043189654.1_    41  VK---------VVGYR----GPAV----VVVSCVTKDP--------PHWPHPHNLV--GKDGCK------   77
_KAF0293344.1_      11  VK---------VVGYR----GPAV----VVVSCVTKDP--------PHWPHPHNLV--GKDGCK------   47
_XP_011496609.1_    41  IE---------IVNYK----GPAL----IVISCVSKDL--------PYWPHPYNII--GKADEI------   77
_AGS12619.1_        41  IE---------IVGYT----GRAV----VVVSCVTADP--------PYRPHPHNLV--GKEGCK------   77
_TRY78048.1_        41  IE---------IVGYT----GRAV----VVVSCVTADP--------PYRPHPHNLV--GKEGCK------   77
_XP_040576307.1_    41  IE---------VVGYK----GRAV----VVVSCVTSET--------PYRPHPHNLV--GKEGCK------   77
_CAB4056363.1_      41  IE---------VVGYK----GRAV----VVVSCVTSET--------PYRPHPHNLV--GKEGCK------   77
```

```
_ALF84898.1_       41  IQ---------VVGYK----GRAV----VVVSTVTIDS--------PYRPHPHNLV--GKEGCS------   77
_XP_023349538.1_   38  ST---------VVGYK----GRAV----VVVSCVSAEV--------PYRPHPHNLV--GKEGCK------   74
_XP_004522582.1_   41  IE---------IVGFK----GSAI----VVVSCVTMDA--------PYRPHPHNLV--GKENCT------   77
_CAD7013622.1_     41  IE---------IVGFK----GSAI----VVVSCVTTDA--------PYRPHPHNLV--GKENCT------   77
_XP_042902217.1_   43  IQ---------VMNYQ----GQYA----VVVSCVSKDGP-------PYRPHPHNLV--GKENCK------   80
_XP_042902218.1_   43  IQ---------VMNYQ----GQYA----VVVSCVSKDGP-------PYRPHPHNLV--GKENCK------   80
_XP_035215470.1_   43  IQ---------VMNYQ----GPYA----VVVSCVTKDGP-------PYRPHPHNLV--GREGCK------   80
_GBN14612.1_       43  IQ---------VMNHQ----GPYA----VVVSCVTKDA--------PYRPHPHNLV--GREGCK------   79
_KAF8795965.1_     43  IQ---------VMNHQ----GPYA----VVVSCVTKDGP-------PYRPHPHNLV--GREGCK------   80
_KAF8795964.1_     43  IQ---------VMNHQ----GPYA----VVVSCVTKDGP-------PYRPHPHNLV--GREGCK------   80
_GFY66827.1_       43  IQ---------VMNHQ----GPYA----VVVSCVTKDGP-------PYRPHPHNLV--GREGCK------   80
_GFW66965.1_       43  IQ---------VMNHQ----GPYA----VVVSCVTKDGP-------PYRPHPHNLV--GREGCK------   80
_GFQ85823.1_       43  IQ---------VMNHQ----GPYA----VVVSCVTKDGP-------PYRPHPHNLV--GREGCK------   80
_GFT35953.1_       43  IQ---------VMNHQ----GPYA----VVVSCVTKDGP-------PYRPHPHNLV--GREGCK------   80
_XP_054714028.1_   43  IQ---------VMNYT----GQYA----VVVSCVAKDGP-------PYRPHPHNLV--GREGCK------   80
_KAG8194583.1_     43  IQ---------VLNHQ----GPYA----VVVSCVTKDA--------PYRPHPHNLV--GREGCK------   79
_GIY09899.1_       43  IQ---------VMNHQ----GPYA----VVVSCVTKDGP-------SYRPHPHNLV--GREGCK------   80
_GIY93593.1_       43  IQ---------VMNHQ----GPYA----VVVSCVTKDGP-------SYRPHPHNLV--GREGCK------   80
_XP_042902221.1_   45  IQ---------VMNCK----GSFA----VVVSCVTKDGP-------PYRPHPNKIV--ENLKKEAYHKLE   88
_XP_046962867.1_   41  IE---------ICGHK----GPAI----VVVSCVTREE--------PYKPHPHNLV--GRERCE------   77
_XP_047530540.1_   41  IE---------ICGHK----GPAI----VVVSCVTREE--------PYKPHPHNLV--GRERCE------   77
_XP_026486466.1_   41  IE---------ICGHK----GPAI----VVVSCVTREE--------PYKPHPHNLV--GRERCE------   77
_XP_047530539.1_   41  IE---------ICGHK----GPAI----VVVSCVTREE--------PYKPHPHNLV--GRERCE------   77
_XP_047530538.1_   41  IE---------ICGHK----GPAI----VVVSCVTREE--------PYKPHPHNLV--GRERCE------   77
_XP_047530537.1_   41  IE---------ICGHK----GPAI----VVVSCVTREE--------PYKPHPHNLV--GRERCE------   77
_XP_026486464.1_   41  IE---------ICGHK----GPAI----VVVSCVTREE--------PYKPHPHNLV--GRERCE------   77
_XP_047530541.1_   41  IE---------ICGHK----GPAI----VVVSCVTREE--------PYKPHPHNLV--GRERCE------   77
_XP_050347286.1_   41  IE---------ICGHK----GSVV----VVVSCVTREE--------PYKPHPHNLV--GRERCN------   77
_XP_050347283.1_   41  IE---------ICGHK----GSVV----VVVSCVTREE--------PYKPHPHNLV--GRERCN------   77
_XP_032515351.1_   41  IE---------IVGYK----GDAV----VVVSCVTREQ--------PYRPHPHNLV--GRERSC------   77
_OWR46016.1_       41  IE---------IVGYK----GDAV----VVVSCVTREQ--------PYRPHPHNLV--GRERSC------   77
_XP_032515346.1_   41  IE---------IVGYK----GDAV----VVVSCVTREQ--------PYRPHPHNLV--GRERSC------   77
_CAG9579094.1_     41  IE---------IVGYK----GDAV----VVVSCVTREQ--------PYRPHPHNLV--GRERSC------   77
_CAG9579091.1_     41  IE---------IVGYK----GDAV----VVVSCVTREQ--------PYRPHPHNLV--GRERSC------   77
_XP_050668638.1_   41  IE---------ISGHR----GPAV----IVVSCVTREE--------PFKPHPHNLV--GRDRCD------   77
_XP_050668632.1_   41  IE---------ISGHR----GPAV----IVVSCVTREE--------PFKPHPHNLV--GRDRCD------   77
_XP_045495329.1_   41  IE---------ICGHR----GPAV----IVVSCVTREE--------PFKPHPHNLV--GRERCD------   77
_XP_038214802.1_   41  IE---------ICGHR----GPAV----VVVSCVTREE--------PFKPHPHNLV--GRERCD------   77
_CAH0714714.1_     41  IE---------ICGHK----GQVV----IVVSCVTRDE--------PFKPHPHNLV--GRERCE------   77
```

```
_CAH0714713.1_      41  IE---------ICGHK----GQVV----IVVSCVTRDE--------PFKPHPHNLV--GRERCE------   77
_CAH4029256.1_      41  IE---------ICGHR----GPAI----VVVSCVTRDE--------PFKPHPHNLV--GRDRCD------   77
_XP_047513081.1_    41  IE---------ICGHR----GPAI----VVVSCVTRDE--------PFKPHPHNLV--GRDRCD------   77
_XP_045529569.1_    41  IE---------ICGHR----GPAI----VVVSCVTRDE--------PFKPHPHNLV--GRDRCD------   77
_XP_045487500.1_    41  IE---------ICGHR----GPAI----VVVSCVTRDE--------PFKPHPHNLV--GRDRCD------   77
_XP_047513077.1_    41  IE---------ICGHR----GPAI----VVVSCVTRDE--------PFKPHPHNLV--GRDRCD------   77
_CAH4029257.1_      41  IE---------ICGHR----GPAI----VVVSCVTRDE--------PFKPHPHNLV--GRDRCD------   77
_XP_045487498.1_    41  IE---------ICGHR----GPAI----VVVSCVTRDE--------PFKPHPHNLV--GRDRCD------   77
_CAF4952989.1_      41  IE---------ICGHR----GPAI----VVVSCVTRDE--------PFKPHPHNLV--GRDRCD------   77
_XP_045447470.1_    41  IE---------ICGHR----GPAI----VVVSCVTREE--------PYKPHPHNLV--GRERCD------   77
_CAH2108688.1_       1  ----------------------------------------------SVKPHPHNLV--GRERCD------   16
_CAH0714724.1_      40  LE---------IYGYE----GEAT----ITISLVTAQK--------PYLQHPHKLY--GNEENT------   76
_XP_034827218.1_    41  IE---------ICGHK----GPAI----IVVSCVTREA--------PYKPHPHNLV--GREKCD------   77
_XP_045768234.1_    41  IE---------ICGHK----GPAI----IVVSCVTREA--------PYKPHPHNLV--GREKCD------   77
_XP_034827214.1_    41  IE---------ICGHK----GPAI----IVVSCVTREA--------PYKPHPHNLV--GREKCD------   77
_XP_045768230.1_    41  IE---------ICGHK----GPAI----IVVSCVTREA--------PYKPHPHNLV--GREKCD------   77
_XP_039751073.1_    41  IE---------VCGHK----GPAI----IVVSCVTREA--------PYKPHPHNLV--GREKCD------   77
_XP_023942270.1_    41  IE---------VVGHK----GPAI----IVVSCVTREA--------PYKPHPHNLV--GREKCD------   77
_XP_023942268.1_    41  IE---------VVGHK----GPAI----IVVSCVTREA--------PYKPHPHNLV--GREKCD------   77
_XP_039751072.1_    41  IE---------VCGHK----GPAI----IVVSCVTREA--------PYKPHPHNLV--GREKCD------   77
_XP_023942266.1_    41  IE---------VVGHK----GPAI----IVVSCVTREA--------PYKPHPHNLV--GREKCD------   77
_CAH2240541.1_      28  IE---------VCGHK----GPAI----IVVSCVTREA--------PYKPHPHNLV--GREKCD------   64
_XP_041985045.1_    41  IE---------IRGHR----GPAV----IVVSCVTKDE--------PYKPHPHNLV--GRERCE------   77
_XP_041985024.1_    41  IE---------IRGHR----GPAV----IVVSCVTKDE--------PYKPHPHNLV--GRERCE------   77
_XP_049868286.1_    41  IE---------IVGHT----GKAI----VVVSCVTREE--------PYKPHPHNLV--GRDRCS------   77
_XP_026314794.1_    41  IE---------ICGHT----GKAI----VVVSCVTKES--------PYKPHPHNLV--GRDRCQ------   77
_XP_047994271.1_    41  IE---------VVGYT----GRFV----VLVSCVTRDK--------PYRPHPHNLV--GREPLC------   77
_KAJ2950301.1_      41  IE---------IVGYT----GKAI----IFVSCVTRDA--------PHKPHPHNLV--GRDRCD------   77
_XP_031784347.2_    40  IQ---------IMNYT----GSFY----VVVSCVTKDE--------PYMPHPHNLI--GKSRGI------   76
_KAG7305875.1_      41  IE---------ICGHK----GRAI----VVVSCVTKDA--------PHRPHPHNLV--GRELCK------   77
_WDD23886.1_        41  IE---------ICGHK----GRAI----VVVSCVTKDA--------PHRPHPHNLV--GRELCK------   77
_XP_048480424.1_    41  IE---------ICGHK----GRAI----VVVSCVTKDA--------PHRPHPHNLV--GRELCK------   77
_XP_048480423.1_    41  IE---------ICGHK----GRAI----VVVSCVTKDA--------PHRPHPHNLV--GRELCK------   77
_XP_048480422.1_    41  IE---------ICGHK----GRAI----VVVSCVTKDA--------PHRPHPHNLV--GRELCK------   77
_XP_048480421.1_    41  IE---------ICGHK----GRAI----VVVSCVTKDA--------PHRPHPHNLV--GRELCK------   77
_KAG7305876.1_      41  IE---------ICGHK----GRAI----VVVSCVTKDA--------PHRPHPHNLV--GRELCK------   77
_CAG9129242.1_      41  IE---------ICGHK----GRAI----VVVSCVTKDA--------PHRPHPHNLV--GRELCK------   77
_XP_048480420.1_    41  IE---------ICGHK----GRAI----VVVSCVTKDA--------PHRPHPHNLV--GRELCK------   77
_GBP53004.1_            ----------------------------------------------------------------------
```

```
_XP_045534737.1_    41  IE---------ISGCS----GNAI----VVVSCVTKDH--------PYKPHPHNLV--GRERCE------   77
_XP_013180060.1_    41  IE---------ISGCN----GNAI----VVVSCVTKDH--------PYKPHPHNLV--GRERCE------   77
_XP_013180043.1_    41  IE---------ISGCN----GNAI----VVVSCVTKDH--------PYKPHPHNLV--GRERCE------   77
_XP_013180068.1_    41  IE---------ISGCN----GNAI----VVVSCVTKDH--------PYKPHPHNLV--GRERCE------   77
_XP_045534736.1_    41  IE---------ISGCS----GNAI----VVVSCVTKDH--------PYKPHPHNLV--GRERCE------   77
_KPJ06840.1_        41  IE---------ISGCS----GNAI----VVVSCVTKDH--------PYKPHPHNLV--GRERCE------   77
_KPJ05518.1_        41  IE---------ISGCN----GNAI----VVVSCVTKDH--------PYKPHPHNLV--GRERCE------   77
_CAG4934519.1_      41  IE---------ISGCT----GSAI----VVVSCVTKDH--------PYKPHPHNLV--GRERCD------   77
_XP_013149112.1_    41  IE---------ISGCT----GNAI----VVVSCVTKDH--------PYKPHPHNLV--GRERCE------   77
_CAH2077165.1_      41  IE---------IRGCT----GSAI----VVVSCVTKEH--------PYKPHPHNLV--GRERCD------   77
_CAH2077167.1_      41  IE---------IRGCT----GSAI----VVVSCVTKEH--------PYKPHPHNLV--GRERCD------   77
_XP_037051415.1_    36  IR---------VLNYT----GPVT----VVVSCVTTEK--------PYRQHPNVLV--GEDCK-------   71
_XP_037051414.1_    37  IR---------VLNYT----GQVT----VVVSCVTTEK--------PYRQHPSILV--GEDCK-------   72
_XP_022815079.1_    41  IK---------ICGYT----GQVV----IVVSCVTKDE--------PYRAHPHNLV--GRERCE------   77
_CAB3516522.1_      41  IK---------ICGYT----GQVV----IVVSCVTKDE--------PYRAHPHNLV--GRERCE------   77
_XP_050562120.1_    41  IK---------ICGYT----GQVV----IVVSCVTKDE--------PYRAHPHNLV--GRERCE------   77
_XP_050562121.1_    41  IK---------ICGYT----GQVV----IVVSCVTKDE--------PYRAHPHNLV--GRERCE------   77
_QHD56218.1_        41  IK---------ICGYT----GQVV----IVVSCVTKDE--------PYRAHPHNLV--GRERCE------   77
_AIA24466.1_        41  IK---------ICGYT----GQVV----IVVSCVTKDE--------PYRAHPHNLV--GRERCE------   77
_XP_022815076.1_    41  IK---------ICGYT----GQVV----IVVSCVTKDE--------PYRAHPHNLV--GRERCE------   77
_QHD56217.1_        41  IK---------ICGYT----GQVV----IVVSCVTKDE--------PYRAHPHNLV--GRERCE------   77
_XP_050562118.1_    41  IK---------ICGYT----GQVV----IVVSCVTKDE--------PYRAHPHNLV--GRERCE------   77
_AIA24467.1_        41  IK---------ICGYT----GQVV----IVVSCVTKDE--------PYRAHPHNLV--GRERCE------   77
_XP_047019808.1_    41  IR---------ICGFT----GQVV----IVVSCVTKDE--------PYRAHPHNLV--GRERCE------   77
_AEO51736.1_        41  IR---------ICDFT----GQVV----IVVSCVTKDE--------PYRAHPHNLV--GRERCE------   77
_XP_049696680.1_    41  IR---------ICGFT----GQVV----IVVSCVTKDE--------PYRAHPHNLV--GRERCE------   77
_XP_049696682.1_    41  IR---------ICGFT----GQVV----IVVSCVTKDE--------PYRAHPHNLV--GRERCE------   77
_XP_049696676.1_    41  IR---------ICGFT----GQVV----IVVSCVTKDE--------PYRAHPHNLV--GRERCE------   77
_XP_026744931.1_    41  IK---------ICGYT----GQVV----IVVSCVTKDE--------PYRAHPHNLV--GRERCE------   77
_XP_026732895.1_    41  IK---------ICGYT----GQVV----IVVSCVTKDE--------PYRAHPHNLV--GRERCE------   77
_CAH0581650.1_      41  IK---------ICGYT----GQVV----IVVSCVTKDE--------PYRAHPHNLV--GRERCE------   77
_KAH9640014.1_      41  IK---------ICGYT----GQVV----IVVSCVTKDE--------PYRAHPHNLV--GRERCE------   77
_PZC78315.1_            ----------------------------------------------------------------------
_KAF9795796.1_          ----------------------------------------------------------------------
_AEC52926.1_        41  IK---------IYGYT----GLVT----IVVSCVTKDE--------PFRPHPHNLV--GRDRCD------   77
_KAH7940301.1_      41  IK---------VMGVQ----KPAV----VVVSCVTKDQ--------PYRVHPHNLV--GKEGCK------   77
_ADK39025.1_        40  IK---------IIGYT----GTVS----IVVSCVTKDE--------PCRPHPHNLV--GRDHCD------   76
_XP_037298148.1_    40  IK---------IIGYT----GTVS----IVVSCVTKDE--------PYRPHPHNLV--GRDHCD------   76
_RVE50742.1_        40  IK---------VCGYK----GCMV----IVVSCVTKDE--------PYKPHPHNLV--GRDCN-------   75
```

```
_CAH0692665.1_        40  IK---------VCGYK----GCMV----IVVSCVTKDE--------PYKPHPHNLV--GRDCN-------   75
_XP_028166351.1_      39  IK---------VCGYK----GCMV----IVVSCVTKEE--------PYKPHPHNLV--GRDCQ-------   74
_XP_028166291.1_      39  IK---------VCGYK----GCMV----IVVSCVTKEE--------PYKPHPHNLV--GRDCQ-------   74
_CAB3235314.1_        41  IK---------IYNYI----GQVV----IVVSCVTKDE--------PYRPHPHNLV--GRERCD------   77
_KAJ0175867.1_        41  IK---------ILGYK----GPVT----IVVSCVTKDGD-------PYKPHPHNLV--GRDRCD------   78
_XP_028031027.1_      41  IK---------ICSYK----GPAT----IVVSCVNKDP--------PFKPHPHNLV--GRERCD------   77
_NP_001166296.1_      41  IK---------ICSYK----GPAT----IVVSCVNKDP--------PFKPHPHNLV--GRERCD------   77
_XP_037870194.1_      41  IK---------ICSYK----GPAT----IVVSCVNKDP--------PFKPHPHNLV--GRERCD------   77
_XP_037870193.1_      41  IK---------ICSYK----GPAT----IVVSCVNKDP--------PFKPHPHNLV--GRERCD------   77
_XP_028031025.1_      41  IK---------ICSYK----GPAT----IVVSCVNKDP--------PFKPHPHNLV--GRERCD------   77
_XP_013187225.1_      40  IM---------ICGYK----GRVV----VVVSCVTKDE--------PFKPHPHNLV--GRECQ-------   75
_XP_052755011.1_      40  IK---------ICGYR----GRVV----IVVSCVTKEE--------PYKPHPHNLV--GRDCL-------   75
_XP_053613182.1_      40  IM---------ICGYK----GRVV----VVVSCVTKDE--------PFKPHPHNLV--GRECQ-------   75
_XP_053613180.1_      40  IM---------ICGYK----GRVV----VVVSCVTKDE--------PFKPHPHNLV--GRECQ-------   75
_CAG9785863.1_        40  IK---------VSGYK----GCMI----IVVSCVTKDE--------PYKPHPHNLV--GRDCQ-------   75
_KAG6461275.1_        40  IK---------IIGYT----GTVS----IVVSCVTKDE--------PYRPHPHNLV--GRDHCD------   76
_KAG6461281.1_        40  IK---------IIGYT----GTVS----IVVSCVTKDE--------PYRPHPHNLV--GRDHCD------   76
_XP_037302137.1_      39  IK---------ILGYT----GFVR----IIVSCVTKDE--------PYRPHPHKLV--GREHCH------   75
_KAG6461269.1_        39  IK---------ILGYT----GFVR----IIVSCVTKDE--------PYRPHPHKLV--GREHCH------   75
_KAG4069896.1_        35  IR---------VLNYT----GPVT----VVVSCVSTEK--------PYWQHPNILV--GEDCR-------   70
_XP_044003989.1_      39  IK---------IHNLQ----GPAR----VSVSCVSKND--------PYHIHSHGLR--FKDNNVKNI---   78
_XP_045768240.1_      41  IE---------ICNCK----GLAI----IVVSCVTTED--------PYKPHPHNLV--GRENCE------   77
_KAG9509099.1_        41  IQ---------IMNYV----GRAV----VVVSCVTKEY--------PFRPHPHKLV--GKEGCK------   77
_XP_013118267.1_      41  IE---------IVGYT----GNVK----IIVSCVTADE--------PYRPHPHNLV--GKEGCE------   77
_XP_005183026.2_      41  IE---------IVGYT----GNVK----IIVSCVTADE--------PYRPHPHNLV--GKEGCD------   77
_XP_050303432.1_      44  IR---------IQVQDP---NYAEKELLLIVSCVTTEQ--------PYLVHPNKLT--GNGCE-------   84
_TMW48950.1_          41  IE---------IEGYE----GKVK----VVVSCVTKEE--------PYRPHPHNLV--GKEGCE------   77
_XP_037882085.1_      41  IE---------IAGYK----GEVT----IVVSCVTKDP--------PYRPHPHNLV--GKEGCE------   77
_KAI8126541.1_        42  IE---------VEGYE----GKVK----IVVSCVTKDE--------PYRPHPHNLV--GKEGCE------   78
_KAI8126540.1_        41  IE---------VEGYE----GKVK----IVVSCVTKDE--------PYRPHPHNLV--GKEGCE------   77
_XP_037811156.1_      41  IE---------VEGYE----GKVK----IVVSCVTKDE--------PYRPHPHNLV--GKEGCE------   77
_XP_046812595.1_      41  IE---------VEGYE----GKVK----IVVSCVTKDE--------PYRPHPHNLV--GKEGCE------   77
_XP_046812596.1_      41  IE---------VEGYE----GKVK----IVVSCVTKDE--------PYRPHPHNLV--GKEGCE------   77
_XP_046812591.1_      41  IE---------VEGYE----GKVK----IVVSCVTKDE--------PYRPHPHNLV--GKEGCE------   77
_XP_046812590.1_      41  IE---------VEGYE----GKVK----IVVSCVTKDE--------PYRPHPHNLV--GKEGCE------   77
_KNC34143.1_          41  IE---------VEGYE----GKVK----IVVSCVTKDE--------PYRPHPHNLV--GKEGCE------   77
_XP_046812589.1_      41  IE---------VEGYE----GKVK----IVVSCVTKDE--------PYRPHPHNLV--GKEGCE------   77
_KAI8126539.1_        41  IE---------VEGYE----GKVK----IVVSCVTKDE--------PYRPHPHNLV--GKEGCE------   77
_XP_037958917.1_      41  IE---------IVGYE----GDVV----IVASCVTKDP--------PYRQHPHYLV--GKDGCK------   77
```

```
_XP_037929595.1_    43  IE---------IVGYK----GDVV----IVVSCVTKDP--------PYRQHPHYLV--GKDGCK------   79
_XP_037957513.1_    41  IE---------IVGYK----GDVV----IVVSCVTKDP--------PYRQHPHYLV--GKDGCK------   77
_XP_037958115.1_    43  IE---------IVGYK----GDVV----IVVSCVTKDP--------PYRQHPHYLV--GKDGCK------   79
_XP_037958114.1_    43  IE---------IVGYK----GDVV----IVVSCVTKDP--------PYRQHPHYLV--GKDGCK------   79
_XP_037957511.1_    41  IE---------IVGYK----GDVV----IVVSCVTKDP--------PYRQHPHYLV--GKDGCK------   77
_XP_037958918.1_    41  IE---------IVGYE----GDVV----IVASCVTKDP--------PYRQHPHYLV--GKDGCK------   77
_RXG71823.1_        41  IR---------VVNSK----GPAL----VVVSCVTVTE--------PYKPHPHNLV--GKEGCE------   77
_KAB7494622.1_       1  ------------VNSK----GPAM----VIVSCVTVTE--------PYKPHPHNLV--GKEGCE------   34
_KAI9584339.1_      41  IE---------IVGYA----GEAV----VIISCVTKEP--------PYCPHPHKLI--GIDCQ-------   76
_XP_037884983.1_    41  IE---------IIGYA----GDAV----VIISCVSKDP--------PYCPHPHKLV--GTDCQ-------   76
_XP_054744821.1_    41  VE---------IVGYK----GEAL----ILVSCVTKDK--------PYRQHPHQLV--GKNCK-------   76
_XP_053961676.1_    41  VE---------IVGYN----GEAL----ILVSCVTKDK--------PYRQHPHQLV--GKNCK-------   76
_XP_053961674.1_    41  VE---------IVGYN----GEAL----ILVSCVTKDK--------PYRQHPHQLV--GKNCK-------   76
_XP_004526634.2_    41  VE---------IVGYN----GEAL----ILVSCVTKEK--------PYRQHPHQLV--GKNCK-------   76
_CAD6998451.1_      41  VE---------IVGYN----GEAL----ILVSCVTKEK--------PYRQHPHQLV--GKNCK-------   76
_XP_012157894.1_    41  VE---------IVGYN----GEAL----ILVSCVTKEK--------PYRQHPHQLV--GKNCK-------   76
_XP_028894493.1_    41  VE---------VVGYK----GEAL----ILVSCVTKDR--------PFRQHPHQLV--GKNCK-------   76
_XP_036327693.1_    41  VE---------IVGYC----GEAL----ILVSCVTKDK--------PYRQHPHQLV--GKNCK-------   76
_XP_017471333.1_    41  VE---------IVGYC----GEAL----ILVSCVTKDK--------PYRQHPHQLV--GKNCK-------   76
_XP_039952608.1_    41  VE---------VVGYE----GKAR----ILVSCVTKDK--------PFRQHPHQLV--GQDCK-------   76
_XP_050320023.1_    41  VE---------VVGYE----GKAR----ILVSCVTKDK--------PFRQHPHQLV--GQDCK-------   76
_XP_036225572.1_    41  VE---------VVGYE----GKAR----ILVSCVTKDK--------PFRQHPHQLV--GQECK-------   76
_XP_039952607.1_    41  VE---------VVGYE----GKAR----ILVSCVTKDK--------PFRQHPHQLV--GQDCK-------   76
_XP_036225564.1_    41  VE---------VVGYE----GKAR----ILVSCVTKDK--------PFRQHPHQLV--GQECK-------   76
_XP_036225569.1_    41  VE---------VVGYE----GKAR----ILVSCVTKDK--------PFRQHPHQLV--GQECK-------   76
_XP_050320021.1_    41  VE---------VVGYE----GKAR----ILVSCVTKDK--------PFRQHPHQLV--GQDCK-------   76
_XP_039952606.1_    41  VE---------VVGYE----GKAR----ILVSCVTKDK--------PFRQHPHQLV--GQDCK-------   76
_XP_011207860.1_    41  VE---------VVGYE----GKAR----ILVSCVTKDK--------PFRQHPHQLV--GQDCK-------   76
_XP_011207859.1_    41  VE---------VVGYE----GKAR----ILVSCVTKDK--------PFRQHPHQLV--GQDCK-------   76
_XP_029407466.1_    41  VE---------VVGYE----GKAR----ILVSCVTKDK--------PFRQHPHQLV--GQDCK-------   76
_XP_019847003.2_    41  VE---------VVGYE----GKAR----ILVSCVTKDK--------PFRQHPHQLV--GQDCK-------   76
_XP_014086488.2_    41  VE---------VVGYE----GKAR----ILVSCVTKDK--------PFRQHPHQLV--GQECK-------   76
_XP_049317393.1_    41  VE---------VVGYE----GKAR----ILVSCVTKDK--------PFRQHPHQLV--GQDCK-------   76
_XP_039952604.1_    41  VE---------VVGYE----GKAR----ILVSCVTKDK--------PFRQHPHQLV--GQDCK-------   76
_XP_018787557.1_    41  VE---------VVGYE----GKAR----ILVSCVTKDK--------PFRQHPHQLV--GQDCK-------   76
_OQR68950.1_        41  IK---------VENYV----GKFV----VVISCVTKDA--------PYYPHPHNIV--SKEACK------   77
_XP_003741763.1_    41  IR---------VENYV----GKFV----VAVSCVTKDH--------PYHPHPHNIV--SKEDCR------   77
_XP_022699297.1_    41  IR---------VENYV----GKFV----VVISCVTKEP--------PYYPHPHNIV--SKEGCR------   77
_XP_022665229.1_    41  IR---------VENYV----GKFV----VVISCVTKEP--------PYYPHPHNIV--SKEGCR------   77
```

```
_XP_022665225.1_    41  IR---------VENYV----GKFV----VVISCVTKEP--------PYYPHPHNIV--SKEGCR------   77
_XP_022665224.1_    41  IR---------VENYV----GKFV----VVISCVTKEP--------PYYPHPHNIV--SKEGCR------   77
_XP_033219687.1_    41  IK---------VHGYQ----GTAA----ILISCVTKDD--------PPKPHPHSLV--GRDSCK------   77
_XP_033227990.1_    41  IK---------VHGYQ----GTAA----ILISCVTKDD--------PPKPHPHSLV--GRDSFK------   77
_XP_043466013.1_    40  IQ---------IHGYK----GPVV----IVVSCVTRDN--------PPRPHPHSIM--GKNSSA------   76
_XP_051172671.1_    40  IQ---------IHGYK----GPVV----IVVSCVTRDN--------PPRPHPHSIM--GKNSAA------   76
_CAD7643228.1_      40  IQ---------LFNHS----GRAK----VVVSCVTKNH--------PFRAHPHNLV--GKKSCK------   76
_XP_054157157.1_    41  IR---------LIGHT----GSAK----VVVSCVTKNP--------PFRPHPHNLV--GKKSCR------   77
_CAD7645633.1_      40  IK---------LFNYS----GRAK----VVVSCVTKNP--------PFRPHPHNLV--GKKSCK------   76
_CAD7632611.1_       1  -------------------------------------HH-------PFRAHPHNLV--GKKSCK------   18
_KAJ6223158.1_      39  IA---------VANHS----GPVK----VIVSCVTKNA--------PYRPHPHNLI--GKKYCD------   75
_KAI2804035.1_      39  IA---------VANHS----GPVK----VIVSCVTKNA--------PYRPHPHNLI--GKKYCD------   75
_KAH9398209.1_      39  VA---------VANYS----GPIK-------------IL-------PYRPHPHNLV--GKQFCS------   67
_GFY77286.1_        41  IK---------VHGYQ----GAAG----VVVSCVTKDGP-------PYYPHPHNLV--GKTCT-------   77
_GFQ73388.1_        41  IK---------VHGYQ----GAAG----VVVSCVTKDGP-------PYYPHPHNLV--GKTCT-------   77
_GFT25575.1_        41  IK---------VHGYQ----GAAG----VVVSCVTKDGP-------PYYPHPHNLV--GKTCT-------   77
_KAF8763655.1_      41  IK---------VHGYQ----GAAG----VVVSCVTKDGP-------PYYPHPHNLV--GKTCA-------   77
_GFX76677.1_        41  IK---------VHGYQ----GAAG----VVVSCVTKDGP-------PYYPHPHNLV--GKTCT-------   77
_XP_035207052.1_    41  IK---------VHGFQ----GAAG----VVVSCVTKDGP-------PYYPHPHNLV--GKTCT-------   77
_KFM74898.1_        41  IK---------VHGFQ----GAAG----VVVSCVTKDGP-------PYYPHPHNLV--GKTCT-------   77
_XP_035207059.1_    41  IK---------VHGFQ----GAAG----VVVSCVTKDGP-------PYYPHPHNLV--GKTCT-------   77
_XP_035207066.1_    41  IK---------VHGFQ----GAAG----VVVSCVTKDGP-------PYYPHPHNLV--GKTCT-------   77
_BBD75287.1_        41  IK---------VHGYQ----GAAS----VVVSCVTKDGP-------PYFPHPHNLV--GKTCA-------   77
_XP_042900952.1_    41  IK---------VHGYQ----GAAS----VVVSCVTKDGP-------PYFPHPHNLV--GKTCA-------   77
_KAG8201816.1_      40  IK---------VHGYQ----GAAD----VVVSCVTKDGP-------PYYPHPHNLI--GKTCT-------   76
_GIX87435.1_        41  IK---------VHGYQ----GAAG----VVVSCITKDGP-------PYYPHPHNLV--GKTCT-------   77
_GBL81029.1_        41  IK---------VHGYQ----GAAG----VVVSCVTKDGP-------PYYPHPHNLV--GKTCT-------   77
_GIY01087.1_        41  IK---------VHGYQ----GAAG----VVVSCITKDGP-------PYYPHPHNLV--GKTCT-------   77
_XP_054715436.1_    41  IK---------INGYQ----GIAG----VVVSCVTKDGP-------PYYPHPHNLV--GKFCA-------   77
_XP_044576112.1_    40  IK---------IHGFK----EKAV----VIVSCVTKDE--------PHRPHPHEII--GKEKCN------   76
_XP_008556435.3_    39  IK---------IHGLD----GSAV----IIVSCVTKDS--------PHRPHPHAIV--SKDKSD------   75
_CAD6234446.1_      40  IK---------IHGFK----GKAV----VIVSCVTKDE--------PHRPHPHEII--GKEKCN------   76
_XP_044284598.1_    41  IQ---------IVNYF----EKVK----IRITLVTKNE--------PYRPHPHRLV--GKDCE-------   76
_XP_033794511.1_    41  IQ---------IMNYS----GKGK----VRVTLVTKSE--------PFKPHPHDLV--GKDCR-------   76
_XP_033794512.1_    41  IQ---------IMNYS----GKGK----VRVTLVTKSE--------PFKPHPHDLV--GKDCR-------   76
_XP_029449578.1_    41  IQ---------IINYS----GKGK----VRVTLVTKSE--------PFKPHPHDLV--GKDCR-------   76
_XP_030052048.1_    41  IQ---------IMNYS----GKGK----VRVTLVTKSE--------PFKPHPHDLV--GKDCM-------   76
_XP_007423639.1_    41  IQ---------ILNYF----GKVK----IRITLVTKNE--------PYRPHPHQLV--GKDCE-------   76
_XP_039203483.1_    41  IQ---------ILNYF----GKVK----IRITLVTKNE--------PYRPHPHQLV--GKDCE-------   76
```

```
_XP_034283206.1_    41  IQ---------ILNYF----GKVK----IRITLVTKNE--------PYRPHPHQLV--GKDCE-------   76
_XP_033001023.1_    41  LQ---------VFNYF----GKIK----IRITLVTKNE--------PYRPHPHRLV--GKDCE-------   76
_XP_053239675.1_    41  LQ---------VFNYF----GKIK----IRITLVTKNE--------PYRPHPHRLV--GKDCE-------   76
_XP_034966804.1_    41  LQ---------VFNYF----GKIK----IRITLVTKNE--------PYRPHPHRLV--GKDCE-------   76
_XP_028580982.1_    30  IQ---------VFNYF----GKIK----IRITLVTKNE--------PYRPHPHRLV--GKDCE-------   65
_XP_042302822.1_    41  IQ---------ILNYV----GKVK----IRVTLVTKDE--------PYRPHPHRLV--GKDCE-------   76
_XP_015280979.1_    30  IQ---------IFNYF----GKIK----IRVTLVTKNE--------PYRPHPHRLV--GKDCE-------   65
_XP_048363544.1_    41  IQ---------IFNYF----GKIK----IRVTLVTKNE--------PYRPHPHRLV--GKDCE-------   76
_KAF7252806.1_      17  -----------IVNYF----EKVK----IRITLVTKNE--------PYRPHPHRLV--GKDCE-------   50
_XP_034958398.1_        ----------------------------------------------------------------------
_CAD5116312.1_      41  IR---------LCNMGN---ATEA---IIVASCVTRDK--------PYRAHPHSLV--GKECN-------   78
_KAJ1519398.1_      39  IQ---------VSEHR----GAVV----VVISCVTRYA--------PHRPHPHSLV--GAGCE-------   74
_XP_053103001.1_    41  IQ---------ILNYV----GKVK----IRITLVTKNE--------PYRPHPHRLV--GKDCE-------   76
_XP_032070730.1_    41  IQ---------ILNYF----GKVK----IRITLVTKNE--------PYRPHPHQLV--GKDCE-------   76
_XP_013922116.1_    41  IQ---------ILNYF----GKVK----IRITLVTKNE--------PYRPHPHQLV--GKDCE-------   76
_XP_015667999.1_    41  IQ---------ILNYF----GKVK----IRITLVTKNE--------PYRPHPHQLV--GKDCE-------   76
_XP_026551846.1_    41  IQ---------ILNYF----GKVK----IRITLVTKNE--------PYRPHPHQLV--GKDCE-------   76
_XP_026532518.1_    41  IQ---------ILNYF----GKVK----IRITLVTKNE--------PYRPHPHQLV--GKDCE-------   76
_KAJ7341301.1_      41  IQ---------ILNYV----GKVK----IRITLVTKNE--------PYRPHPHRLV--GKDCE-------   76
_KAJ6667038.1_      30  IE---------ILNYI----GKIK----IRITLVTKNE--------PYRPHPHRLV--GKDCE-------   65
_XP_003227818.2_    30  IQ---------ILNYV----GKIK----IRITLVTKTE--------PYRPHPHRLV--GKDCE-------   65
_XP_020633036.1_    30  VQ---------ILNYV----GKVK----IRITLVTKNE--------PYRPHPHRLV--GKDCE-------   65
_XP_054842059.1_    41  IQ---------IFNYF----GKLK----VRVTLVTKNE--------PYRPHPHRLV--GKDCE-------   76
_XP_005238641.2_    30  IQ---------ILNYF----GKVK----IRTTLVTKNE--------PYKPHPHDLV--GKDCR-------   65
_XP_005445671.2_    30  IQ---------ILNYF----GKVK----IRTTLVTKNE--------PYKPHPHDLV--GKDCR-------   65
_XP_054050029.1_    30  IQ---------ILNYF----GKVK----IRTTLVTKNE--------PYKPHPHDLV--GKDCR-------   65
_XP_013810845.1_    30  IQ---------ILNYF----GKVK----IRTTLVTKNE--------PYKPHPHDLV--GKDCR-------   65
_XP_026702002.1_    30  IQ---------ILNYF----GKVK----IRTTLVTKNE--------PYKPHPHDLV--GKDCR-------   65
_XP_025892242.1_    30  IQ---------ILNYF----GKVK----IRTTLVTKNE--------PYKPHPHDLV--GKDCR-------   65
_XP_005507389.1_    30  IQ---------ILNYF----GKVK----IRTTLVTKNE--------PYKPHPHDLV--GKDCR-------   65
_XP_032911247.1_    30  IQ---------ILNYF----GKVK----IRTTLVTKNE--------PYKPHPHDLV--GKDCR-------   65
_XP_031959657.1_    30  IQ---------ILNYF----GKVK----IRTTLVTKNE--------PYKPHPHDLV--GKDCR-------   65
_XP_041905291.1_    30  IQ---------ILNYF----GKVK----IRTTLVTKNE--------PYKPHPHDLV--GKDCR-------   65
_XP_051648836.1_    30  IQ---------ILNYF----GKVK----IRTTLVTKNE--------PYKPHPHDLV--GKDCR-------   65
_XP_054239488.1_    30  IQ---------ILNYF----GKIK----IRTTLVTKNE--------PYKPHPHDLV--GKDCR-------   65
_XP_039914164.1_    30  IQ---------ILNYF----GKVK----IRTTLVTKNE--------PYKPHPHDLV--GKDCR-------   65
_XP_005422689.1_    30  IQ---------ILNYF----GKIK----IRTTLVTKNE--------PYKPHPHDLV--GKDCR-------   65
_KAF2987757.1_      30  IQ---------ILNYF----GKFK----IRTTLVTKNE--------PYKPHPHDLV--GKDCR-------   65
_XP_038665087.1_    41  IQ---------VVNYF----GRGR----VLVTLVTKVE--------PFKPHPHDLV--GKDCR-------   76
```

```
_GCB59945.1_        41  IQ---------VVNYF----GRGR----VLVTLVTKVE--------PFKPHPHDLV--GKDCR-------   76
_XP_041065616.1_    41  IQ---------VVNYF----GRGR----VLVTLVTKVE--------PFKPHPHDLV--GKDCR-------   76
_XP_007890564.1_    41  IQ---------IMNYV----GRGR----VLITLVTKSE--------PFKPHPHDLV--GKDCR-------   76
_XP_051868609.1_    41  IR---------VVNYL----GRVR----VLVSLVTKIE--------PFKPHPHDLV--GKDCR-------   76
_XP_032881051.1_    40  IQ---------VVNYF----GRVR----VLVTLVTKSE--------PFKPHPHDLV--GKDCK-------   75
_XP_048456938.1_        ----------------------------------------------------------------------
_KAJ1155552.1_      41  IQ---------IMNYA----GKGK----VRVSLVTKNE--------PCKPHPHDLV--GKDCK-------   76
_KAH0622877.1_          ----------------------------------------------------------------------
_CAI5653329.1_      41  LQ---------ILNYC----GKGK----VRVYLVTKNE--------PYRPHPHDLV--GKDCK-------   76
_CAI5653328.1_      41  LQ---------ILNYC----GKGK----VRVYLVTKNE--------PYRPHPHDLV--GKDCK-------   76
_NXS54806.1_        41  IQASAA--FIPILNYF----GKVK----IRCTLVTKNE--------PYKPHPHDLV--GKDCR-------   83
_XP_009504395.1_    38  IQ---------ILNYF----GKVK----IRTTLVTKNE--------PYKPHPHDLV--GKDCR-------   73
_NXJ41381.1_        41  IQ---------ILNYF----GKVK----IRTTLVTKNE--------PYKPHPHDLV--GKDCR-------   76
_NXT24719.1_        41  IQ---------ILNYF----GKVK----IRTTLVTKNE--------PYKPHPHDLV--GKDCR-------   76
_NXK23502.1_        41  IQ---------ILNYF----GKVK----IRTTLVTKNE--------PYKPHPHDLV--GKDCR-------   76
_NXK03070.1_        41  IQ---------ILNYF----GKVK----IRTTLVTKNE--------PYKPHPHDLV--GKDCR-------   76
_XP_037260944.1_    41  IQ---------ILNYF----GKVK----IRTTLVTKNE--------PYKPHPHDLV--GKDCR-------   76
_XP_040467369.1_    41  IQ---------ILNYF----GKVK----IRTTLVTKNE--------PYKPHPHDLV--GKDCR-------   76
_NXY71491.1_        41  IQ---------ILNYF----GKVK----IRTTLVTKNE--------PYKPHPHDLV--GKDCR-------   76
_NXN54417.1_        41  IQ---------ILNYF----GKVK----IRTTLVTKNE--------PYKPHPHDLV--GKDCR-------   76
_NXX19490.1_        41  IQ---------ILNYF----GKVK----IRTTLVTKNE--------PYKPHPHDLV--GKDCR-------   76
_NXL47199.1_        41  IQ---------ILNYF----GKVK----IRTTLVTKNE--------PYKPHPHDLV--GKDCR-------   76
_XP_050749583.1_    41  IQ---------ILNYF----GKVK----IRTTLVTKNE--------PYKPHPHDLV--GKDCR-------   76
_NXD76242.1_        41  IQ---------ILNYY----GKVK----IRTTLVTKNE--------PYKPHPHDLV--GKDCR-------   76
_KFZ56827.1_        41  IQ---------ILNYF----GKVK----IRTTLVTKNE--------PYKPHPHDLV--GKDCR-------   76
_NXH67691.1_        41  IQ---------ILNYF----GKVK----IRTTLVTKNE--------PYKPHPHDLV--GKDCR-------   76
_NXS45828.1_        41  IQ---------ILNYF----GKVK----IRTTLVTKNE--------PYKPHPHDLV--GKDCR-------   76
_KFP52034.1_        41  IQ---------ILNYF----GKVK----IRTTLVTKNE--------PYKPHPHDLV--GKDCR-------   76
_NWH44485.1_        41  IQ---------ILNYF----GKVK----IRTTLVTKNE--------PYKPHPHDLV--GKDCR-------   76
_NWW85416.1_        41  IQ---------ILNYF----GKVK----IRTTLVTKNE--------PYKPHPHDLV--GKDCR-------   76
_NWU66320.1_        41  IQ---------ILNYF----GKVK----IRTTLVTKNE--------PYKPHPHDLV--GKDCR-------   76
_XP_025919906.1_    41  IQ---------ILNYF----GKVK----IRTTLVTKNE--------PYKPHPHDLV--GKDCR-------   76
_KAF1483947.1_      41  IQ---------ILNYF----GKVK----IRTTLVTKNE--------PYKPHPHDLV--GKDCR-------   76
_NXQ87688.1_        41  IQ---------ILNYF----GKVK----IRTTLVTKNE--------PYKPHPHDLV--GKDCR-------   76
_NXX07562.1_        41  IQ---------ILNYF----GKVK----IRTTLVTKNE--------PYKPHPHDLV--GKDCR-------   76
_XP_009280788.2_    41  IQ---------ILNYF----GKVK----IRTTLVTKNE--------PYKPHPHDLV--GKDCR-------   76
_XP_014821857.1_    41  IQ---------ILNYF----GKVK----IRTTLVTKNE--------PYKPHPHDLV--GKDCR-------   76
_NWU51443.1_        41  IQ---------ILNYF----GKVK----IRTTLVTKNE--------PYKPHPHDLV--GKDCR-------   76
_NXV35521.1_        41  IQ---------ILNYF----GKVK----IRTTLVTKNE--------PYKPHPHDLV--GKDCR-------   76
```

```
_KFV80780.1_       41  IQ---------ILNYF----GKVK----IRTSLVTKNE--------PYKPHPHDLV--GKDCR-------   76
_XP_032039522.1_   41  IQ---------ILNYF----GKVK----IRTTLVTKNE--------PYKPHPHDLV--GKDCR-------   76
_NWT43027.1_       41  IQ---------ILNYF----GKVK----IRTTLVTKNE--------PYKPHPHDLV--GKDCR-------   76
_KAF1651581.1_     41  IQ---------ILNYF----GKVK----IRTTLVTKNE--------PYKPHPHDLV--GKDCR-------   76
_NXU26374.1_       41  IQ---------ILNYF----GKVK----IRTTLVTKNE--------PYKPHPHDLV--GKDCR-------   76
_NWX46229.1_       41  IQ---------ILNYF----GKVK----IRTTLVTKNE--------PYKPHPHDLV--GKDCR-------   76
_PKU40340.1_       41  IQ---------ILNYF----GKVK----IRTTLVTKNE--------PYKPHPHDLV--GKDCR-------   76
_XP_027309078.1_   41  IQ---------ILNYF----GKVK----IRTTLVTKNE--------PYKPHPHDLV--GKDCR-------   76
_KAF1573683.1_     41  IQ---------ILNYF----GKVK----IRTTLVTKNE--------PYKPHPHDLV--GKDCR-------   76
_KAF1578928.1_     41  IQ---------ILNYF----GKVK----IRTTLVTKNE--------PYKPHPHDLV--GKDCR-------   76
_NXN47104.1_       41  IQ---------ILNYF----GKVK----IRTTLVTKNE--------PYKPHPHDLV--GKDCR-------   76
_NXV20393.1_       41  IQ---------ILNYF----GKVK----IRTTLVTKNE--------PYKPHPHDLV--GKDCR-------   76
_KAF1551267.1_     41  IQ---------ILNYF----GKVK----IRTTLVTKNE--------PYKPHPHDLV--GKDCR-------   76
_KAF1406210.1_     41  IQ---------ILNYF----GKVK----IRTTLVTKNE--------PYKPHPHDLV--GKDCR-------   76
_NXV43201.1_       41  IQ---------ILNYF----GKVK----IRTTLVTKNE--------PYKPHPHDLV--GKDCR-------   76
_NWQ98669.1_       41  IQ---------ILNYF----GKVK----IRTTLVTKNE--------PYKPHPHDLV--GKDCR-------   76
_XP_052639968.1_   41  IQ---------ILNYF----GKVK----IRTTLVTKNE--------PYKPHPHDLV--GKDCR-------   76
_XP_010573143.1_   41  IQ---------ILNYF----GKVK----IRTTLVTKNE--------PYKPHPHDLV--GKDCR-------   76
_KAF1479604.1_     41  IQ---------ILNYF----GKVK----IRTTLVTKNE--------PYKPHPHDLV--GKDCR-------   76
_NXG89109.1_       41  IQ---------ILNYF----GKVK----IRTTLVTKNE--------PYKPHPHDLV--GKDCR-------   76
_KFQ89757.1_       41  IQ---------ILNYF----GKVK----IRTTLVTKNE--------PYKPHPHDLV--GKDCR-------   76
_NWZ21787.1_       41  IQ---------ILNYF----GKVK----IRTTLVTKNE--------PYKPHPHDLV--GKDCR-------   76
_NXI74048.1_       41  IQ---------ILNYF----GKVK----IRTTLVTKNE--------PYKPHPHDLV--GKDCR-------   76
_NWX76247.1_       41  IQ---------ILNYF----GKVK----IRTTLVTKNE--------PYKPHPHDLV--GKDCR-------   76
_NXF29788.1_       41  IQ---------ILNYF----GKVK----IRTTLVTKNE--------PYKPHPHDLV--GKDCR-------   76
_KAF1444142.1_     41  IQ---------ILNYF----GKVK----IRTTLVTKNE--------PYKPHPHDLV--GKDCR-------   76
_KFV20215.1_       41  IQ---------ILNYF----GKVK----IRTTLVTKNE--------PYKPHPHYLV--GKDCR-------   76
_NXI44657.1_       41  IQ---------ILNYF----GKIK----IRTTLVTKNE--------PYKPHPHDLV--GKDCR-------   76
_NXJ92321.1_       41  IQ---------ILNYF----GKVK----IRTTLVTKNE--------PYKPHPHDLV--GKDCR-------   76
_NWZ49902.1_       41  IQ---------ILNYF----GKVK----IRTTLVTKNE--------PYKPHPHDLV--GKDCR-------   76
_NWY59444.1_       41  IQ---------ILNYF----GKVK----IRTTLVTKNE--------PYKPHPHDLV--GKDCR-------   76
_KFW64019.1_       41  IQ---------ILNYF----GKVK----IRTTLVTKNE--------PYKPHPHDLV--GKDCR-------   76
_NXJ55634.1_       41  IQ---------ILNYF----GKVK----IRTTLVTKNE--------PYKPHPHDLV--GKDCR-------   76
_KAF1513372.1_     41  IQ---------ILNYF----GKVK----IRTTLVTKNE--------PYKPHPHDLV--GKDCR-------   76
_NXW14598.1_       41  IQ---------ILNYF----GKVK----IRTTLVTKNE--------PYKPHPHDLV--GKDCR-------   76
_NXC74247.1_       41  IQ---------ILNYF----GKVK----IRTTLVTKNE--------PYKPHPHDLV--GKDCR-------   76
_NXT57254.1_       41  IQ---------ILNYF----GKVK----IRTTLVTKNE--------PYKPHPHDLV--GKDCR-------   76
_XP_047916679.1_   41  IQ---------ILNYF----GKVK----IRTTLVTKNE--------PYKPHPHDLV--GKDCR-------   76
_XP_029879190.1_   41  IQ---------ILNYF----GKVK----IRTTLVTKNE--------PYKPHPHDLV--GKDCR-------   76
```

```
_NXH09241.1_        41  IQ---------ILNYF----GKIK----IRTTLVTKNE--------PYKPHPHDLV--GKDCR-------   76
_XP_040407374.1_    41  IQ---------ILNYF----GKVK----IRTTLVTKNE--------PYKPHPHDLV--GKDCR-------   76
_KFW76037.1_        41  IQ---------ILNYF----GKVK----IRTTLVTKNE--------PYKPHPHDLV--GKDCR-------   76
_KFP65945.1_        41  IQ---------ILNYF----GKVK----IRTTLVTKNE--------PYKPHPHDLV--GKDCR-------   76
_NXX55682.1_        41  IQ---------ILNYF----GKVK----IRTTLVTKNE--------PYKPHPHDLV--GKDCR-------   76
_XP_035178333.1_    41  IQ---------ILNYF----GKVK----IRTTLVTKNE--------PYKPHPHDLV--GKDCR-------   76
_NXE47317.1_        41  IQ---------ILNYF----GKVK----IRTTLVTKNE--------PYKPHPHDLV--GKDCR-------   76
_XP_035408877.1_    41  IQ---------ILNYF----GKVK----IRTTLVTKNE--------PYKPHPHDLV--GKDCR-------   76
_KGL94141.1_        41  IQ---------ILNYF----GKVK----IRTTLVTKNE--------PYKPHPHDLV--GKDCR-------   76
_NXN68403.1_        41  IQ---------ILNYF----GKVK----IRTTLVTKNE--------PYKPHPHDLV--GKDCR-------   76
_NXW28331.1_        41  IQ---------ILNYF----GKVK----IRTTLVTKNE--------PYKPHPHDLV--GKDCR-------   76
_XP_049657509.1_    41  IQ---------ILNYF----GKVK----IRTTLVTKNE--------PYKPHPHDLV--GKDCR-------   76
_XP_049657511.1_    41  IQ---------ILNYF----GKVK----IRTTLVTKNE--------PYKPHPHDLV--GKDCR-------   76
_NXG30098.1_        41  IQ---------ILNYF----GKVK----IRTTLVTKNE--------PYKPHPHDLV--GKDCR-------   76
_NXV86645.1_        41  IQ---------ILNYF----GKVK----IRTTLVTKNE--------PYKPHPHDLV--GKDCR-------   76
_NXX83200.1_        41  IQ---------ILNYF----GKVK----IRTTLVTKNE--------PYKPHPHDLV--GKDCR-------   76
_NXI59898.1_        41  IQ---------ILNYY----GKVK----IRTTLVTKNE--------PYKPHPHDLV--GKDCR-------   76
_NWU89521.1_        41  IQ---------ILNYF----GKVK----IRTTLVTKNE--------PYKPHPHDLV--GKDCR-------   76
_NXK45490.1_        41  IQ---------ILNYF----GKVK----IRTTLVTKNE--------PYKPHPHDLV--GKDCR-------   76
_NWR55599.1_        41  IQ---------ILNYY----GKVK----IRTTLVTKNE--------PYKPHPHDLV--GKDCR-------   76
_NXL37824.1_        41  IQ---------ILNYF----GKVK----IRTTLVTKNE--------PYKPHPHDLV--GKDCR-------   76
_NWI33553.1_        41  IQ---------ILNYF----GKVK----IRTTLVTKNE--------PYKPHPHDLV--GKDCR-------   76
_KFQ74517.1_        41  IQ---------ILNYF----GKVK----IRTTLVTKNE--------PYKPHPHDLV--GKDCR-------   76
_NXV82662.1_        41  IQ---------ILNYF----GKVK----IRTTLVTKSE--------PYKPHPHDLV--GKDCR-------   76
_NWJ10087.1_        41  IQ---------ILNYF----GKVK----IRTTLVTKNE--------PYKPHPHDLV--GKDCR-------   76
_NXN24483.1_        41  IQ---------ILNYF----GKVK----IRTTLVTKNE--------PYKPHPHDLV--GKDCR-------   76
_NWR80208.1_        41  IQ---------ILNYY----GKVK----IRTTLVTKNE--------PYKPHPHDLV--GKDCR-------   76
_NXT37538.1_        41  IQ---------ILNYF----GKVK----IRTTLVTKNE--------PYKPHPHDLV--GKDCR-------   76
_XP_032844994.2_    41  IQ---------ILNYF----GKVK----IRTTLVTKNE--------PYKPHPHDLV--GKDCR-------   76
_NXW47649.1_        41  IQ---------ILNYF----GKVK----IRTTLVTKNE--------PYKPHPHDLV--GKDCR-------   76
_NXA40398.1_        41  IQ---------ILNYF----GKVK----IRTTLVTKNE--------PYKPHPHDLV--GKDCK-------   76
_NXW64893.1_        41  IQ---------ILNYF----GKVK----IRCTLVTKNE--------PYKPHPHDLV--GKDCR-------   76
_NXX89165.1_        41  IQ---------ILNYY----GKVK----IRTTLVTKNE--------PYKPHPHDLV--GKDCR-------   76
_NXQ96911.1_        41  IQ---------IVNYF----GKVK----IRTTLVTKNE--------PYKPHPHDLV--GKDCR-------   76
_KFP18273.1_        41  IQ---------ILNYF----GKVK----IRTTLVTKNE--------PYKPHPHDLV--GKDCR-------   76
_NXC40882.1_        41  IQ---------ILNYF----GKVK----IRTTLVTKNE--------PYKPHPHDLV--GKDCR-------   76
_NXE10305.1_        41  IQ---------ILNYF----GKVK----IRTTLVTKNE--------PYKPHPHDLV--GKDCR-------   76
_KFP88087.1_        41  IQ---------ILNYF----GKVK----IRTTLVTKNE--------PYKPHPHDLV--GKDCR-------   76
_NWW47871.1_        41  IQ---------ILNYF----GKVK----IRTTLVTKNE--------PYKPHPHDLV--GKDCR-------   76
```

```
_NXJ61595.1_       41  IQ---------ILNYF----GKVK----IRTTLVTKNE--------PYKPHPHDLV--GKDCR-------   76
_NXD13814.1_       41  IQ---------ILNYF----GKVK----IRTTLVTKNE--------PYKPHPHDLV--GKDCR-------   76
_KFR16961.1_       41  IQ---------ILNYF----GKVK----IRTTLVTKNE--------PHKPHPHDLV--GKDCR-------   76
_NWX24312.1_       41  IQ---------ILNYF----GKIK----IRTTLVTKNE--------PYKPHPHDLV--GKDCR-------   76
_NXA28267.1_       41  IQ---------ILNYF----GKVK----IRTTLVTKNE--------PYKPHPHDLV--GKDCR-------   76
_NXL66270.1_       41  IQ---------ILNYF----GKVK----IRTTLVTKNE--------PYKPHPHDLV--GKDCR-------   76
_NXA49976.1_       41  IQ---------ILNYF----GKVK----IRTTLVTKNE--------PYKPHPHDLV--GKDCR-------   76
_NXT77624.1_       41  IQ---------ILNYF----GKVK----IRTTLVTKSE--------PYKPHPHDLV--GKDCR-------   76
_KFQ92336.1_       41  IQ---------ILNYF----GKVK----IRTTLVTKNE--------PYKPHPHDLV--GKDCR-------   76
_NXE25013.1_       41  IQ---------ILNYF----GKVK----IRTTLVTKNE--------PYKPHPHDLV--GKDCR-------   76
_NXE92240.1_       41  IQ---------ILNYF----GKVK----IRTTLVTKNE--------PYKPHPHDLV--GKDCR-------   76
_NWH80943.1_       41  IQ---------ILNYY----GKVK----IRTTLVTKNE--------PYKPHPHDLV--GKDCR-------   76
_NWI19058.1_       41  IQ---------ILNYF----GKVK----IRTTLVTKNE--------PYKPHPHDLV--GKDCR-------   76
_NXG80610.1_       41  IQ---------ILNYF----GKVK----IRTTLVTKSE--------PYKPHPHDLV--GKDCR-------   76
_NWR01486.1_       41  IQ---------ILNYF----GKVK----IRTTLVTKNE--------PYKPHPHDLV--GKDCR-------   76
_NXK81337.1_       41  IQ---------ILNYF----GKVK----IRTTLVTKNE--------PYKPHPHDLV--GKDCR-------   76
_NXS90563.1_       41  IQ---------ILNYF----GKVK----IRTTLVTKNE--------PYKPHPHDLV--GKDCR-------   76
_NXC17430.1_       41  IQ---------ILNYF----GKVK----IRTTLVTKNE--------PYKPHPHDLV--GKDCR-------   76
_NXE77245.1_       41  IQ---------ILNYF----GKVK----IRTTLVTKNE--------PYKPHPHDLV--GKDCR-------   76
_KFQ29788.1_       41  IQ---------ILNYF----GKVK----IRTTLVTKNEP-------PKKPHPHDLV--GKDCR-------   77
_KFO89327.1_       41  IQ---------ILNYY----GKVK----IRTTLVTKNE--------PYKPHPHDLV--GKDCR-------   76
_NXD64381.1_       41  IQ---------ILNYF----GKVK----IRTTLVTKNE--------PYKPHPHDLV--GKDCR-------   76
_NXY45081.1_       41  IQ---------ILNYY----GKVK----IRTTLVTKNE--------PYKPHPHDLV--GKDCR-------   76
_KGL72871.1_       41  IQ---------ILNYF----GKVK----IRTTLVTKNE--------PYKPHPHDLV--GKDCR-------   76
_XP_053919504.1_   41  IQ---------ILNYY----GKVK----IRTTLVTKNE--------PYKPHPHDLV--GKDCR-------   76
_NXL85459.1_       41  IQ---------ILNYF----GKVK----IRTTLVTKNE--------PYKPHPHDLV--GKDCR-------   76
_NXO02254.1_       41  IQ---------ILNYF----GKVK----IRATLVTKNE--------PYKPHPHVLV--GKDCR-------   76
_NXJ76034.1_       41  IQ---------ILNYF----GKVK----IRTTLVTKNE--------PYKPHPHDLV--GKDCR-------   76
_NXM93491.1_       41  IQ---------ILNYF----GKIK----IRTTLVTKNE--------PYKPHPHDLV--GKDCR-------   76
_XP_030345800.1_   41  IQ---------ILNYF----GKVK----IRTTLVTKNE--------PYKPHPHDLV--GKDCR-------   76
_XP_030302544.1_   41  IQ---------ILNYF----GKVK----IRTTLVTKNE--------PYKPHPHDLV--GKDCR-------   76
_XP_030345801.1_   41  IQ---------ILNYF----GKVK----IRTTLVTKNE--------PYKPHPHDLV--GKDCR-------   76
_KQK77724.1_       41  IQ---------ILNYF----GKVK----IRTTLVTKNE--------PYKPHPHDLV--GKDCR-------   76
_NXP09913.1_       41  IQ---------ILNYF----GKVK----IRTTLVTKNE--------PYKPHPHDLV--GKDCR-------   76
_NXR33018.1_       41  IQ---------ILNYY----GKVK----IRTTLVTKNE--------PYKPHPHDLV--GKDCR-------   76
_NXM76881.1_       41  IQ---------ILNYF----GKVK----IRTTLVTKNE--------PYKPHPHDLV--GKDCR-------   76
_NXS27423.1_       41  IQ---------ILNYF----GKVK----IRTTLVTKNE--------PYKPHPHDLV--GKDCR-------   76
_NXM55821.1_       41  IQ---------ILNYF----GKVK----IRTTLVTKNE--------PYKPHPHDLV--GKDCR-------   76
_XP_054678500.1_   41  IQ---------ILNYF----GKVK----IRTTLVTKSE--------PYKPHPHDLV--GKDCR-------   76
```

```
_XP_015712248.1_   41  IQ---------ILNYF----GKVK----IRTTLVTKNE--------PYKPHPHDLV--GKDCR-------   76
_NWX92058.1_       41  IQ---------ILNYF----GKVK----IRTTLVTKNE--------PYKPHPHDLV--GKDCR-------   76
_XP_048793646.1_   41  IQ---------ILNYF----GKVK----IRTTLVTKNE--------PYKPHPHDLV--GKDCR-------   76
_NXF04247.1_       41  IQ---------ILNYF----GKVK----IRTTLVTKNE--------PYKPHPHDLV--GKDCR-------   76
_XP_042683331.1_   41  IQ---------ILNYF----GKVK----IRTTLVTKNE--------PYKPHPHDLV--GKDCR-------   76
_NWV22147.1_       41  IQ---------ILNYF----GKVK----IRTTLVTKNE--------PYKPHPHDLV--GKDCR-------   76
_NXA04361.1_       41  IQ---------ILNYF----GKVK----IRTTLVTKNE--------PYKPHPHDLV--GKDCR-------   76
_XP_010116198.1_   47  IQ---------ILNYF----GKVK----IRTTLVTKNE--------PYKPHPHDLV--GKDCR-------   82
_NXG59741.1_       41  IQ---------ILNYF----GKVK----IRTTLVTKNE--------PYKPHPHDLV--GKDCR-------   76
_XP_042735326.1_   41  IQ---------ILNYF----GKVK----IRTTLVTKNE--------PYKPHPHDLV--GKDCR-------   76
_NXM29886.1_       41  IQ---------ILNYF----GKVK----IRTTLVTKNE--------PYKPHPHDLV--GKDCR-------   76
_NP_001161198.2_   41  IQ---------ILNYF----GKVK----IRTTLVTKNE--------PYKPHPHDLV--GKDCR-------   76
_XP_031451029.1_   41  IQ---------ILNYF----GKVK----IRTTLVTKNE--------PYKPHPHDLV--GKDCR-------   76
_NXR49262.1_       41  IQ---------ILNYF----GKVK----IRTTLVTKNE--------PYKPHPHDLV--GKDCR-------   76
_XP_052555416.1_   41  IQ---------ILNYF----GKVK----IRTTLVTKNE--------PYKPHPHDLV--GKDCR-------   76
_NWZ67085.1_       41  IQ---------ILNYF----GKVK----IRTTLVTKNE--------PYKPHPHDLV--GKDCR-------   76
_P16236.2_         41  IQ---------ILNYF----GKVK----IRTTLVTKNE--------PYKPHPHDLV--GKDCR-------   76
_XP_050566010.1_   41  IQ---------ILNYF----GKVK----IRTTLVTKNE--------PYKPHPHDLV--GKDCR-------   76
_NWS67030.1_       41  IQ---------ILNYY----GKVK----IRTTLVTKNE--------PYKPHPHDLV--GKDCR-------   76
_XP_005143850.3_   41  IQ---------ILNYF----GKVK----IRTTLVTKNE--------PYKPHPHDLV--GKDCR-------   76
_XP_050566011.1_   41  IQ---------ILNYF----GKVK----IRTTLVTKNE--------PYKPHPHDLV--GKDCR-------   76
_XP_050566009.1_   41  IQ---------ILNYF----GKVK----IRTTLVTKNE--------PYKPHPHDLV--GKDCR-------   76
_NWR30963.1_       41  IQ---------ILNYF----GKVK----IRTTLVTKNE--------PYKPHPHDLV--GKDCK-------   76
_NWS17896.1_       41  IQ---------ILNYF----GKVK----IRTTLVTKNE--------PYKPHPHDLV--GKDCR-------   76
_XP_021248574.1_   41  IQ---------ILNYF----GKVK----IRTTLVTKNE--------PYKPHPHDLV--GKDCR-------   76
_AAA48721.1_       41  IQ---------ILNYF----GKVK----IRTTLVTKNE--------PYKPLPHDLV--GKDCR-------   76
_NWI54208.1_       41  IQ---------ILNYF----GKVK----IRTTLVTKNE--------PYKPHPHDLV--GKDCK-------   76
_XP_027509532.1_   41  IQ---------ILNYF----GKVK----IRTTLVTKNE--------PYKPHPHDLV--GKDCR-------   76
_NXP71979.1_       41  IQ---------ILNYF----GKIK----IRTTLVTKNE--------PYKPHPHDLV--GKDCR-------   76
_NXX25352.1_       41  IQ---------ILNYF----GKVK----IRTTLVTKNE--------PYKPHPHDLV--GKDCR-------   76
_NXI18618.1_       41  IQ---------ILNYF----GKVK----IRTTLVTKNE--------PYKPHPHDLV--GKDCR-------   76
_NXS09623.1_       41  IQ---------ILNYF----GKLK----IRTTLVTKNE--------PYKPHPHHLV--GKDCR-------   76
_NXU77980.1_       41  IQ---------IVNYF----GKVK----IRTTLVTKNE--------PYKPHPHDLV--GKDCR-------   76
_XP_027509533.1_   41  IQ---------ILNYF----GKVK----IRTTLVTKNE--------PYKPHPHDLV--GKDCR-------   76
_NWV71036.1_       41  IQ---------ILNYF----GKVK----IRTTLVTKNE--------PYKPHPHDLV--GKDCK-------   76
_NXF89482.1_       41  IQ---------ILNYF----GKIK----IRTTLVTKNE--------PYKPHPHDLV--GKDCR-------   76
_NWY39470.1_       41  IQ---------ILNYF----GKVK----IRTTLVTKNE--------PYKPHPHDLV--GKDCR-------   76
_NXT59960.1_       41  IQ---------ILNYF----GKVK----IRTTLVTKNE--------PYKPHPHDLV--GKDCK-------   76
_NWU74340.1_       41  IQ---------ILNYF----GKVK----IRTTLVTKNE--------PYKPHPHDLV--GKDCR-------   76
```

```
_NXB84481.1_       41  IQ---------ILNYF----GKIK----IRTTLVTKNE--------PYKPHPHDLV--GKDCK-------   76
_NWV73885.1_       41  IQ---------ILNYF----GKVK----IRTTLVTKNE--------PYKPHPHDLV--GKDCR-------   76
_OPJ58894.1_       41  IQ---------ILNYF----GKVK----IRTTLVTKNE--------PYKPHPHDLV--GKDCR-------   76
_XP_048152104.1_   41  IQ---------ILNYF----GKVK----IRTTLVTKNE--------PYKPHPHDLV--GKDCR-------   76
_XP_054036037.1_   41  IQ---------ILNYF----GKIK----IRTTLVTKNE--------PYKPHPHDLV--GKDCR-------   76
_NXR18149.1_       41  IQ---------ILNYF----GKVK----IRTTLVTKNE--------PYKPHPHDLV--GKDCR-------   76
_NXP97533.1_       41  IQ---------ILNYF----GKIK----IRTTLVTKNE--------PYKPHPHDLV--GKDCK-------   76
_NXJ05495.1_       41  IQ---------ILNYF----GKVK----IRTTLVTKNE--------PYKPHPHDLV--GKDCK-------   76
_XP_032538932.1_   41  IQ---------ILNYF----GKVK----IRTTLVTKNE--------PYKPHPHDLV--GKDCR-------   76
_NWI96387.1_       41  IQ---------ILNYF----GKVK----IRTTLVTKNE--------PYKPHPHDLV--GKDCR-------   76
_NXR65995.1_       41  IQ---------ILNYF----GKVK----IRTTLVTKNE--------PYKPHPHDLV--GKDCR-------   76
_NXK42398.1_       41  IQ---------ILNYF----GKVK----IRTTLVTKNE--------PYKPHPHDLV--GKDCR-------   76
_XP_032538931.1_   41  IQ---------ILNYF----GKVK----IRTTLVTKNE--------PYKPHPHDLV--GKDCR-------   76
_NXY33181.1_       41  IQ---------ILNYF----GKVK----IRTTLVTKNE--------PYKPHPHDLV--GKDCR-------   76
_XP_050171009.1_   41  IQ---------ILNYF----GKVK----IRTTLVTKNE--------PYKPHPHDLV--GKDCR-------   76
_NXO50939.1_       41  IQ---------ILNYF----GKVK----IRTTLVTKSE--------PYKPHPHDLV--GKDCR-------   76
_NWZ32937.1_       41  IQ---------ILNYF----GKVK----IRTTLVTKNE--------PYKPHPHDLV--GKDCR-------   76
_NXP01828.1_       41  IQ---------ILNYF----GKVK----IRTTLVTKNE--------PYKPHPHDLV--GKDCR-------   76
_NXL00458.1_       41  IQ---------ILNYF----GKVK----IRTTLVTKNE--------PYKPHPHDLV--GKDCR-------   76
_XP_010401310.4_   41  IQ---------ILNYF----GKVK----IRTTLVTKNE--------PYKPHPHDLV--GKDCR-------   76
_NXH62149.1_       41  IQ---------ILNYF----GKVK----IRTTLVTKNE--------PYKPHPHDLV--GKDCR-------   76
_NXG43824.1_       41  IQ---------ILNYF----GKIK----IRTTLVTKNE--------PYKPHPHDLV--GKDCR-------   76
_XP_017677337.1_   41  IQ---------ILNYF----GKVK----IRTTLVTKNE--------PYKPHPHDLV--GKDCR-------   76
_NXU09156.1_       41  IQ---------ILNYF----GKVK----IRTTLVTKNE--------PYKPHPHDLV--GKDCR-------   76
_NXB38645.1_       41  IQ---------ILNYF----GKVK----IRTTLVTKNE--------PYKPHPHDLV--GKDCR-------   76
_XP_027594504.1_   41  IQ---------ILNYF----GKVK----IRTTLVTKNE--------PYKPHPHDLV--GKDCR-------   76
_NXM46269.1_       41  IQ---------ILNYF----GKVK----IRTTLVTKNE--------PYKPHPHDLV--GKDCR-------   76
_NWS79257.1_       41  IQ---------ILNYF----GKVK----IRTTLVTKNE--------PYKPHPHDLV--GKDCR-------   76
_NXB04839.1_       41  IQ---------ILNYF----GKVK----IRTTLVTKNE--------PYKPHPHDLV--GKDCR-------   76
_NXC62865.1_       41  IQ---------ILNYF----GKVK----IRTTLVTKNE--------PYKPHPHDLV--GKDCR-------   76
_NXN18277.1_       41  IQ---------ILNYF----GKIK----IRTTLVTKNE--------PYKPHPHDLV--GKDCR-------   76
_XP_027760039.1_   41  IQ---------ILNYF----GKVK----IRTTLVTKNE--------PYKPHPHDLV--GKDCR-------   76
_NXI90311.1_       41  IQ---------ILNYG----GKVK----IRTTLVTKSE--------PYKPHPHDLV--GKDCR-------   76
_NWY17003.1_       41  IQ---------ILNYF----GKVK----IRTTLVTKNE--------PYKPHPHDLV--GKDCR-------   76
_NXH97099.1_       41  IQ---------ILNYF----GKVK----IRTTLVTKNE--------PYKPHPHDLV--GKDCR-------   76
_NWS97308.1_       41  IQ---------ILNYF----GKVK----IRTTLVTKNE--------PYKPHPHDLV--GKDCR-------   76
_NWU45160.1_       41  IQ---------ILNYF----GKVK----IRTTLVTKNE--------PYKPHPHDLV--GKDCR-------   76
_NXQ43004.1_       41  IQ---------ILNYF----GKVK----IRTTLVTKNE--------PYKPHPHDLV--GKDCR-------   76
_XP_027549142.1_   41  IQ---------ILNYF----GKVK----IRTTLVTKNE--------PYKPHPHDLV--GKDCR-------   76
```

```
_XP_017933082.2_    41  IQ---------ILNYF----GKVK----IRTTLVTKNE--------PYKPHPHDLV--GKDCR-------   76
_NWV96326.1_        41  IQ---------ILNYF----GKVK----IRTTLVTKNE--------PYKPHPHDLV--GKDCR-------   76
_NXP57783.1_        41  IQ---------ILNYF----GKVK----IRTTLVTKNE--------PYKPHPHDLV--GKDCR-------   76
_NXY57955.1_        41  IQ---------ILNYF----GKVK----IRTTLVTKNE--------PYKPHPHDLV--GKDCR-------   76
_AAA99433.1_        41  IQ---------ILNYF----GKVK----IRTTLVTKNE--------PYKPHPHDLV--GKDCR-------   76
_NXY21218.1_        41  IQ---------ILNYF----GKVK----IRTTLVTKNE--------PYKPHPHDLV--GKDCR-------   76
_NXX50824.1_        41  IQ---------ILNYF----GKIK----IRTTLVTKNE--------PYKPHPHDLV--GKDCR-------   76
_XP_030124623.3_    41  IQ---------ILNYF----GKIK----IRTTLVTKNE--------PYKPHPHDLV--GKDCK-------   76
_NXA92235.1_        41  IQ---------ILNYF----GKVK----IRTTLVTKNE--------PYKPHPHDLV--GKDCR-------   76
_NWI05522.1_        41  IQ---------ILNYF----GKVK----IRTTLVTKNE--------PYKPHPHDLV--GKDCR-------   76
_NWX29009.1_        41  IQ---------ILNYF----GKVK----IRTTLVTKNE--------PYKPHPHDLV--GKDCR-------   76
_XP_027549144.1_    41  IQ---------ILNYF----GKVK----IRTTLVTKNE--------PYKPHPHDLV--GKDCR-------   76
_NWW35420.1_        41  IQ---------ILNYF----GKVK----IRTTLVTKNE--------PYKPHPHDLV--GKDCR-------   76
_NWV07969.1_        41  IQ---------ILNYF----GKVK----IRTTLVTKNE--------PYKPHPHDLV--GKDCR-------   76
_NXH42214.1_        41  IQ---------ILNYF----GKVK----IRTTLVTKNE--------PYKPHPHDLV--GKDCR-------   76
_NXQ18773.1_        41  IQ---------IVNYF----GKIK----IRTTLVTKNE--------PYKPHPHDLV--GKDCR-------   76
_NWX10672.1_        41  IQ---------ILNYF----GKVK----IRTTLVTKNE--------PYKPHPHDLV--GKDCR-------   76
_NXU50687.1_        41  IQ---------ILNYF----GKVK----IRTTLVTKNE--------PYKPHPHDLV--GKDCR-------   76
_KFU84784.1_        41  IQ---------ILNYF----GKVK----IRTTLVTKNE--------PYKPHPHDLV--GKDCR-------   76
_NXQ52438.1_        41  IQ---------ILNYF----GKVK----IRTTLVTKNE--------PYKPHPHDLV--GKDCR-------   76
_NWW24536.1_        41  IQ---------ILNYF----GKVK----IRTTLVTKNE--------PYKPHPHDLV--GKDCR-------   76
_NXF73449.1_        41  IQ---------ILNYF----GKVK----IRTTLVTKNE--------PYKPHPHDLV--GKDCR-------   76
_XP_050827906.1_    41  IQ---------ILNYF----GKIK----IRTTLVTKNE--------PYKPHPHDLV--GKDCR-------   76
_NWX65205.1_        41  IQ---------ILNYF----GKVK----IRTTLVTKNE--------PYKPHPHDLV--GKDCR-------   76
_KFW81749.1_        41  IQ---------ILNYF----GKVK----IRTTLVTKNE--------PYKPHPHDLV--GKDCR-------   76
_XP_014750593.1_    41  IQ---------ILNYF----GKVK----IRTTLVTKNE--------PYKPHPHDLV--GKDCR-------   76
_NXJ17066.1_        41  IQ---------ILNYF----GKVK----IRTTLVTKNE--------PYKPHPHDLV--GKDCR-------   76
_NWW67019.1_        41  IQ---------ILNYF----GKVK----IRTTLVTKNE--------PYKPHPHDLV--GKDCR-------   76
_NXH03360.1_        41  IQ---------ILNYF----GKIK----IRTTLVTKNE--------PYKPHPHDLV--GKDCR-------   76
_XP_037987525.1_    41  IQ---------ILNYF----GKIK----IRTTLVTKNE--------PYKPHPHDLV--GKDCR-------   76
_NXL74522.1_        41  IQ---------ILNYF----GKLK----IRTTLVTKNE--------PYKPHPHDLV--GKDCR-------   76
_NWQ60824.1_        41  IQ---------ILNYF----GKVK----IRTTLVTKNE--------PYKPHPHDLV--GKDCR-------   76
_NXR70971.1_        41  IQ---------IQNYF----GKVK----IRTTLVTKNE--------PYKPHPHDLV--GKDCK-------   76
_XP_051468947.1_    41  IQ---------ILNYF----GKVK----IRTTLVTKNE--------PYKPHPHDLV--GKDCR-------   76
_NXB50199.1_        41  IQ---------ILNYF----GKVK----IRTTLVTKNE--------PYKPHPHDLV--GKDCR-------   76
_NXP18380.1_        41  IQ---------ILNYF----GKVK----IRTTLVTKNE--------PYKPHPHDLV--GKDCR-------   76
_NXI84319.1_        41  IQ---------ILNYF----GKVK----IRTTLVTKNE--------PYKPHPHDLV--GKDCR-------   76
_NWH33757.1_        41  IQ---------ILNYF----GKVK----IRTTLVTKNE--------PYKPHPHDLV--GKDCR-------   76
_NWR99781.1_        41  IQ---------ILNYF----GKIK----IRTTLVTKNE--------PYKPHPHDLV--GKDCR-------   76
```

NXW94404.1	41	IQ---------ILNYF----GKVK----IRTTLVTKNE--------PYKPHPHDLV--GKDCR-------	76
CAA27220.1	41	IQ---------ILNYF----GKVK----IRTTLVTKNE--------PYKPHPHDLV--GKDCR-------	76
_XP_015477679.1_	41	IQ---------ILNYF----GKVK----IRTTLVTKSE--------PYKPHPHDLV--GKDCR-------	76
NWS32597.1	41	IQ---------ILNYF----GKVK----IRTTLVTKNE--------PYKPHPHDLV--GKDCK-------	76
NXG22126.1	41	IQ---------ILNYF----GKVK----IRTTLVTKNE--------PYKPHPHDLV--GKDCR-------	76
NWY97127.1	41	IQ---------ILNYF----GKIK----IRTTLVTKNE--------PYKPHPHDLV--GKDCR-------	76
NXW80148.1	41	IQ---------ILNYF----GKVK----IRTTLVTKNE--------PYKPHPHDLV--GKDCR-------	76
NXC27367.1	41	IQ---------ILNYF----GKVK----IRTTLVTKNE--------PYKPHPHDLV--GKDCR-------	76
NWH83726.1	41	IQ---------ILNYF----GKVK----IRTTLVTKNE--------PYKPHPHDLV--GKDCR-------	76
NXA73512.1	41	IQ---------ILNYF----GKVK----IRTTLVTKNE--------PYKPHPHDLV--GKDCR-------	76
NWZ83230.1	41	IQ---------ILNYF----GKVK----IRTTLVTKSE--------PYKPHPHDLV--GKDCR-------	76
_XP_005525572.2_	41	IQ---------ILNYF----GKVK----IRTTLVTKSE--------PYKPHPHDLV--GKDCR-------	76
NXD20033.1	41	IQ---------ILNYF----GKVK----IRTTLVTKNE--------PYKPHPHDLV--GKDCR-------	76
NXE37177.1	41	IQ---------ILNYF----GKVK----IRTTLVTKNE--------PYKPHPHDLV--GKDCR-------	76
NWV44369.1	41	IQ---------ILNYF----GKVK----IRTTLVTKNE--------PYKPHPHDLV--GKDCR-------	76
NXC81400.1	41	IQ---------ILNYF----GKVK----IRTTLVTKNE--------PYKPHPHDLV--GKDCR-------	76
NXA67345.1	41	IQ---------ILNYF----GKVK----IRTTLVTKNE--------PYKPHPHDLV--GKDCR-------	76
NXB55155.1	41	IQ---------ILNYV----GKVK----IRTTLVTKNE--------PYKPHPHDLV--GKDCR-------	76
NXN81229.1	41	IQ---------ILNYF----GKIK----IRTTLVTKNE--------PYKPHPHDLV--GKDCR-------	76
NXM15601.1	41	IQ---------ILNYF----GKIK----IRTTLVTKNE--------PYKPHPHDLV--GKDCR-------	76
_XP_054484850.1_	41	IQ---------ILNYF----GKIK----IRTTLVTKNE--------PYKPHPHDLV--GKDCR-------	76
NWU02685.1	41	IQ---------ILNYF----GKIK----IRTTLVTKNE--------PYKPHPHDLV--GKDCR-------	76
_XP_041273165.1_	41	IQ---------ILNYF----GKIK----IRTTLVTKNE--------PYKPHPHDLV--GKDCR-------	76
NWW80397.1	41	IQ---------ILNYF----GKVK----IRTTLVTKNE--------PYKPHPHDLV--GKDCR-------	76
_XP_036236540.1_	41	IQ---------ILNYF----GKIK----IRTTLVTKNE--------PYKPHPHDLV--GKDCR-------	76
NWV50957.1	41	IQ---------ILNYF----GKVK----IRTTLVTKNE--------PYKPHPHDLV--GKDCR-------	76
NXT97287.1	41	IQ---------ILNYF----GKVK----IRTTLVTKNE--------PYKPHPHDLV--GKDCR-------	76
_XP_023778876.1_	41	IQ---------ILNYF----GKVK----IRTTLVTKSE--------PYKPHPHDLV--GKDCR-------	76
NXD42381.1	41	IQ---------ILNYF----GKVK----IRTTLVTKNE--------PYKPHPHDLV--GKDCK-------	76
NXK69295.1	41	IQ---------ILNYF----GKVK----IRTTLVTKNE--------PYKPHPHDLV--GKDCR-------	76
_XP_049657510.1_	41	IQ---------ILNYF----GKVK----IRTTLVTKNE--------PYKPHPHDLV--GKDCR-------	76
NXV62539.1	41	IQ---------ILNYF----GKIK----IRTTLVTKNE--------PYKPHPHDLV--GKDCR-------	76
NXB80231.1	41	IQ---------ILNYF----GKVK----IRTTLVTKNE--------PYKPHPHDLV--GKDCR-------	76
NWU15026.1	41	IQ---------ILNYF----GKVK----IRTTLVTKNE--------PYKPHPHDLV--GKDCR-------	76
NXL26104.1	41	IQ---------ILNYF----GKIK----IRTTLVTKNE--------PYKPHPHDLV--GKDCR-------	76
NWW05001.1	41	IQ---------ILNYF----GKVK----IRTTLVTKNE--------PYKPHPHDLV--GKDCK-------	76
RLW07261.1	41	IQ---------ILNYF----GKIK----IRTTLVTKNE--------PYKPHPHDLV--GKDCK-------	76
NXR96625.1	41	IQ---------ILNYF----GKIK----IRTTLVTKNE--------PYKPHPHDLV--GKDCR-------	76
_XP_021383204.1_	41	IQ---------ILNYF----GKIK----IRTTLVTKNE--------PYKPHPHDLV--GKDCK-------	76

```
_NXQ25764.1_        41  IQ---------ILNYF----GKVK----IRTTLVTKNE--------PYKPHPHDLV--GKDCR-------   76
_NXO08412.1_        41  IQ---------ILNYF----GKVK----IRTTLVTKNE--------PYKPHPHDLV--GKDCK-------   76
_NXI32280.1_        41  IQ---------ILNYY----GKVK----IRTTLVTKNE--------PYKPHPHDLV--GKDCR-------   76
_NXG00288.1_        41  IQ---------ILNYF----GKVK----IRTTLVTKNE--------PYKPHPHDLV--GKDCR-------   76
_XP_041329879.1_    41  IQ---------ILNYF----GKIK----IRTTLVTKNE--------PYKPHPHDLV--GKDCR-------   76
_NXV03759.1_        41  IQ---------ILNYF----GKVK----IRTTLVTKNE--------PYKPHPHDLV--GKDCR-------   76
_NWT16975.1_        41  IQ---------ILNYF----GKVK----IRTTLVTKNE--------PYKPHPHDLV--GKDCR-------   76
_NWT67953.1_        41  IQ---------ILNYF----GKIK----IRTTLVTKNE--------PYKPHPHDLV--GKDCR-------   76
_XP_005042765.2_    41  IQ---------ILNYF----GKVK----IRTTLVTKNE--------PYKPHPHDLV--GKDCR-------   76
_XP_017600715.1_    41  IQ---------ILNYF----GKVK----IRTTLVTKNE--------PYKPHPHDLV--GKDCR-------   76
_NXD93279.1_        41  IQ---------ILNYF----GKVK----IRTTLVTKNE--------PYKPHPHDLV--GKDCK-------   76
_NXY02260.1_        41  IQ---------ILNYF----GKVK----IRTTLVTKNE--------PYKPHPHDLV--GKDCR-------   76
_NXO84677.1_        41  IQ---------ILNYF----GKVK----IRTTLVTKNE--------PYKPHPHDLV--GKDCR-------   76
_NXO64371.1_        41  IQ---------ILNYF----GKVK----IRTTLVTKNE--------PYKPHPHDLV--GKDCR-------   76
_NXO42022.1_        41  IQ---------ILNYF----GKVK----IRTTLVTKNE--------PYKPHPHDLV--GKDCR-------   76
_NWY65013.1_        41  IQ---------ILNYF----GKVK----IRTTLVTKNE--------PYKPHPHDLV--GKDCR-------   76
_NXP90563.1_        41  IQ---------ILNYF----GKIK----IRTTLVTKNE--------PYKPHPHDLV--GKDCK-------   76
_NWI73723.1_        41  IQ---------ILNYF----GKVK----IRTTLVTKNE--------PYKPHPHDLV--GKDCR-------   76
_NXH30511.1_        41  IQ---------ILNYF----GKVK----IRTTLVTKNE--------PYKPHPHDLV--GKDCR-------   76
_NWT77800.1_        41  IQ---------ILNYF----GKVK----IRTTLVTKNE--------PYKPHPHDLV--GKDCR-------   76
_NWT34209.1_        41  IQ---------ILNYF----GKIK----IRTTLVTKNE--------PYKPHPHDLV--GKDCR-------   76
_NWY29369.1_        41  IQ---------ILNYF----GKIK----IRTTLVTKNE--------PYKPHPHDLV--GKDCR-------   76
_NXQ73821.1_        41  IQ---------ILNYF----GKIK----IRTTLVTKNE--------PYKPHPHDLV--GKDCR-------   76
_XP_039572786.1_    41  IQ---------ILNYF----GKIK----IRTTLVTKNE--------PYKPHPHDLV--GKDCR-------   76
_XP_030802035.1_    41  IQ---------ILNYF----GKIK----IRTTLVTKNE--------PYKPHPHDLV--GKDCR-------   76
_NXE68211.1_        41  IQ---------ILNYF----GKIK----IRTTLVTKNE--------PYKPHPHDLV--GKDCR-------   76
_NXS80652.1_        41  IQ---------ILNYF----GKVK----IRTTLVTKNE--------PYKPHPHDLV--GKDCR-------   76
_NXO32431.1_        41  IQ---------ILNYF----GKVK----IRTTLVTKNE--------PYKPHPHDLV--GKDCR-------   76
_NWR17317.1_        41  IQ---------ILNYF----GKIK----IRTTLVTKNE--------PYKPHPHDLV--GKDCR-------   76
_NWZ89732.1_        41  IQ---------ILNYF----GKIK----IRTTLVTKNE--------PYKPHPHDLV--GKDCR-------   76
_XP_032039523.1_    41  IQ---------ILNYF----GKVK----IRTTLVTKNE--------PYKPHPHDLV--GKDCR-------   76
_KFO04362.1_        41  IQ---------ILNYF----GKVK----IRTTLVTKSE--------PYKPHPHDLV--GKDCR-------   76
_XP_054146213.1_    41  IQ---------ILNYF----GKFK----IRTTLVTKNE--------PYKPHPHDLV--GKDCR-------   76
_XP_005486916.2_    41  IQ---------ILNYF----GKFK----IRTTLVTKNE--------PYKPHPHDLV--GKDCR-------   76
_KAJ7422946.1_      41  IQ---------ILNYF----GKVK----IRTTLVTKNE--------PYKPHPHDLV--GKDCR-------   76
_NXC99388.1_        41  IQ---------ILNYF----GKVK----IRTTLVTKNE--------PYKPHPHDLV--GKDCR-------   76
_RMC07975.1_        41  IQ---------ILNYF----GKVK----IRTTLVTKNE--------PYKPHPHDLV--GKDCR-------   76
_NXT95159.1_         1  -----------ILNYF----GKVK----IRTTLVTKNE--------PYKPHPHDLV--GKDCR-------   34
_XP_009957589.1_     1  -----------ILNYF----GKVK----IRTTLVTKNE--------PYKPHPHDLV--GKDCR-------   34
```

```
_TRZ13602.1_         1  -----------ILNYY----GKVK----IRTTLVTKNE--------PYKPHPHDLV--GKDCR-------   34
_NWR45911.1_         1  ----------------------------IRTTLVTKNE--------PYKPHPHDLV--GKDCR-------   25
_KAF4799649.1_       1  -----------ILNYF----GKVK----IRTTLVTKNE--------PYKPHPHDLV--GKDCR-------   34
_KAJ7420138.1_      41  IQ---------ILNYF----GKVK----IRTTLVTKNE--------PYKPHPHDLV--GKDCR-------   76
_XP_054678501.1_    41  IQ---------ILNYF----GKVK----IRTTLVTKSE--------PYKPHPHDLV--GKDCR-------   76
_NXP48685.1_        41  IQ---------ILNYF----GKIK----IRTTLVTKSE--------PYKPHPHDLV--GKDCR-------   76
_XP_010704701.1_        ----------------------------------------------------------------------
_KFV10805.1_            ----------------------------------------------------------------------
_XP_009572567.1_        ----------------------------------------------------------------------
_NXF64277.1_            ----------------------------------------------------------------------
_KFV54465.1_            ----------------------------------------------------------------------
_KFV90717.1_            ----------------------------------------------------------------------
_KFQ03674.1_            ----------------------------------------------------------------------
_NXW01028.1_            ----------------------------------------------------------------------
_KFP32908.1_            ----------------------------------------------------------------------
_KFV93389.1_            ----------------------------------------------------------------------
_NWI63728.1_            ----------------------------------------------------------------------
_KAI6072063.1_          ----------------------------------------------------------------------
_KFQ58901.1_            ----------------------------------------------------------------------
_NXP33753.1_            ----------------------------------------------------------------------
_XP_009476353.1_        ----------------------------------------------------------------------
_KFQ55903.1_            ----------------------------------------------------------------------
_XP_042683349.1_        ----------------------------------------------------------------------
_NXR08991.1_            ----------------------------------------------------------------------
_XP_008947250.1_        ----------------------------------------------------------------------
_KFP72464.1_            ----------------------------------------------------------------------
_KFV60523.1_            ----------------------------------------------------------------------
_NXT13165.1_            ----------------------------------------------------------------------
_NWT50506.1_            ----------------------------------------------------------------------
_OXB76139.1_            ----------------------------------------------------------------------
_XP_050827907.1_        ----------------------------------------------------------------------
_XP_030302545.1_        ----------------------------------------------------------------------
_XP_054036038.1_        ----------------------------------------------------------------------
_XP_012788337.1_    41  IQ---------IANYY----GKGK----VRITLVTKND--------PYKPHPHDLV--GKDCR-------   76
_XP_008070154.1_    30  IQ---------IMNYY----GKGK----VRITLVTKND--------PYKPHPHDLV--GKDCR-------   65
_XP_027477445.1_    30  IQ---------IMNYY----GKGK----VRITLVTKND--------PYKPHPHDLV--GKDCR-------   65
_XP_025848473.1_    30  IQ---------IMNYY----GKGK----VRITLVTKND--------PYKPHPHDLV--GKDCR-------   65
_XP_049640716.1_    41  IQ---------IANYY----GKGK----IRITLVTKNE--------PYKPHPHDLV--GKDCK-------   76
_XP_023084089.1_    41  IQ---------IMNYY----GKGK----VRITLVTKND--------PYKPHPHDLV--GKDCR-------   76
```

EHH22143.1	41	IQ---------IMNYY----GKGK----VRITLVTKND--------PYKPHPHDLV--GKDCR-------	76
_XP_038504711.1_	41	IQ---------IMNYY----GKGK----VRITLVTKND--------PYKPHPHDLV--GKDCR-------	76
_XP_038504703.1_	41	IQ---------IMNYY----GKGK----VRITLVTKND--------PYKPHPHDLV--GKDCR-------	76
_XP_038504718.1_	41	IQ---------IMNYY----GKGK----VRITLVTKND--------PYKPHPHDLV--GKDCR-------	76
_XP_048971856.1_	41	IQ---------IMNYY----GKGK----VRITLVTKND--------PYKPHPHDLV--GKDCR-------	76
KAB1266826.1	41	IQ---------IMNYY----GKGK----VRITLVTKND--------PYKPHPHDLV--GKDCR-------	76
_XP_048391424.1_	41	IQ---------IANYF----GRGR----VLVTLVTKME--------PFKPHPHDLV--GKDCK-------	76
_XP_048391425.1_	41	IQ---------IANYF----GRGR----VLVTLVTKME--------PFKPHPHDLV--GKDCK-------	76
GCC28026.1	41	IQ---------VANYF----GRGR----VLVTLVTKME--------PFKPHPHDLV--GKDCK-------	76
_XP_043551991.1_	41	IQ---------VANYF----GRGR----VLVTLVTKME--------PFKPHPHDLV--GKDCK-------	76
_XP_043551989.1_	41	IQ---------VANYF----GRGR----VLVTLVTKME--------PFKPHPHDLV--GKDCK-------	76
_XP_054534389.1_		--	
_XP_011531312.1_		--	
_XP_021780541.1_		--	
_XP_037862912.1_	1	---V--GKDCR-------	6
_XP_015413428.1_	30	IQ---------IKNCY----GKGK----VRITLVTKND--------PFKPHPHDLV--GKDCR-------	65
_XP_005884642.1_	30	IQ---------IKNCY----GKGK----VRITLVTKND--------PFKPHPHDLV--GKDCR-------	65
KAF6105903.1	30	IQ---------IMNYC----GKGK----VRITLVTKNE--------PFKPHPHDLV--GKDCR-------	65
_XP_015413426.1_	30	IQ---------IKNCY----GKGK----VRITLVTKND--------PFKPHPHDLV--GKDCR-------	65
_XP_014387539.1_	30	IQ---------IKNCY----GKGK----VRITLVTKND--------PFKPHPHDLV--GKDCR-------	65
_XP_014444723.1_	30	IQ---------IANYY----GKGK----VRITLVTKND--------PYKPHPHDLV--GKDCR-------	65
_XP_023448058.1_	30	IQ---------IMNYY----GKGK----VRITLVTKND--------PYKPHPHDLV--GKDCR-------	65
EGW11604.1	30	IQ---------VMNYY----GKGK----VRITLVTKND--------PYKPHPHDLV--GKDCR-------	65
_XP_015985129.1_	30	IQ---------IMNFY----GKGK----VRITLVTKNE--------PYKPHPHDLV--GKDCR-------	65
_XP_012878014.1_	30	IQ---------IMNYY----GKGK----VRITLVTKND--------PYKPHPHDLV--GKDCR-------	65
KAG5211506.1	9	VQ---------ILNYY----GKGK----VRITLVTKND--------PYKPHPHDLV--GKDCR-------	44
MXQ82539.1	5	LK---------ILNYY----GKGK----VRITLVTKND--------PYKPHPHDLV--GKDCR-------	40
_XP_032729748.1_	41	IQ---------IMNYY----GKGK----VRITLVTKND--------PYKPHPHDLV--GKDCR-------	76
_XP_032208113.1_	41	IQ---------IMNYY----GKGK----VRITLVTKND--------PYKPHPHDLV--GKDCR-------	76
_XP_048208817.1_	41	IQ---------IVNYY----GKGK----VRITLVTKND--------PYKPHPHDLV--GKDCR-------	76
_XP_038200333.1_	41	IQ---------IMNYY----GKGK----VRITLVTKND--------PYKPHPHDLV--GKDCR-------	76
_P15307.2_	41	VQ---------IMNYY----GKGK----IRITLVTKND--------PYKPHPHDLV--GKDCR-------	76
_XP_041524635.1_	30	IQ---------IMNYY----GKGK----VRITLVTKND--------PYKPHPHDLV--GKDCR-------	65
CAA33843.1	41	VQ---------IMNYY----GKGK----IRITLVTKND--------PYKPHPHDLV--GKDCR-------	76
_XP_028732210.2_	30	IQ---------IMNYY----GKGK----VRITLVTKND--------PYKPHPHDLV--GKDCR-------	65
_XP_025848474.1_	30	IQ---------IMNYY----GKGK----VRITLVTKND--------PYKPHPHDLV--GKDCR-------	65
EDL97996.1	30	IQ---------IMNYY----GKGK----VRITLVTKND--------PYKPHPHDLV--GKDCR-------	65
KAI4577886.1	30	IQ---------ILNYY----GKGK----VRITLVTKND--------PYKPHPHDLV--GKDCR-------	65
KAG8512309.1	41	IQ---------IRNYY----GKGK----VRITLVTKND--------PYKPHPHDLV--GKDCR-------	76

```
_XP_047637971.1_    41  IQ---------IKNYF----GKGK----VRITLVTKND--------PYKPHPHDLV--GKDCR-------   76
_XP_019491454.1_    41  IQ---------IMNYY----GKGK----VRITLVTKND--------PYKPHPHDLV--GKDCR-------   76
_XP_043842482.1_    41  IQ---------IMNCYG--KGKGK----VRITLVTKNE--------PYKPHPHDLV--GKDCR-------   78
_XP_027721170.1_    41  IQ---------IMNCYG--KGRGK----VRITLVTKNE--------PYKPHPHDLV--GKDCR-------   78
_XP_036604632.1_    41  IQ---------IMNCYG--KGKGK----VRITLVTKNE--------PYKPHPHDLV--GKDCR-------   78
_XP_020841164.1_    41  IQ---------IMNCYG--KGRGK----VRITLVTKNE--------PYKPHPHDLV--GKDCR-------   78
_XP_051831295.1_    41  IQ---------IMNCYG--KGKGK----VRITLVTKNE--------PYKPHPHDLV--GKDCK-------   78
_XP_031806378.1_    41  IQ---------IMNCYG--KGKGK----VRITLVTKNE--------PYKPHPHDLV--GKDCK-------   78
_XP_051831294.1_    41  IQ---------IMNCYG--KGKGK----VRITLVTKNE--------PYKPHPHDLV--GKDCK-------   78
_XP_003339776.1_    41  IQ---------IMNYFG--KGKGK----VRITLVTKNE--------PYKPHPHDLV--GKDCR-------   78
_XP_044515413.1_    41  IQ---------IMNCYG--KGKGK----VRITLVTKNE--------PYKPHPHDLV--GKDCR-------   78
_XP_028928361.1_    41  IQ---------IMNSYG--RGKGK----VRITLVTKNE--------PYKPHPHDLV--GKDCR-------   78
_XP_038607403.1_    41  IQ---------IMNCYG--RGKGK----VRITLVTKNE--------PYKPHPHDLV--GKDCR-------   78
_XP_040849334.1_        ----------------------------------------------------------------------
_TKC46270.1_            ----------------------------------------------------------------------
_OWK10634.1_            ----------------------------------------------------------------------
_XP_036190342.1_        ----------------------------------------------------------------------
_KAB0395596.1_          ----------------------------------------------------------------------
_KAF6430170.1_          ----------------------------------------------------------------------
_XP_038948811.1_        ----------------------------------------------------------------------
_KAB1266828.1_      41  IQ---------IMNYY----GKGK----VRITLVTKND--------PYKPHPHDLV--GKDCR-------   76
_NP_002899.1_       41  IQ---------IMNYY----GKGK----VRITLVTKND--------PYKPHPHDLV--GKDCR-------   76
_XP_001159537.3_    41  IQ---------IMNYY----GKGK----VRITLVTKND--------PYKPHPHDLV--GKDCR-------   76
_PNI66333.1_        41  IQ---------IMNYY----GKGK----VRITLVTKND--------PYKPHPHDLV--GKDCR-------   76
_EHH55592.1_        41  IQ---------IMNYY----GKGK----VRITLVTKND--------PYKPHPHDLV--GKDCR-------   76
_XP_003262470.1_    41  IQ---------IMNYY----GKGK----VRITLVTKND--------PYKPHPHDLV--GKDCR-------   76
_BAG64854.1_        41  IQ---------IMNYY----GKGK----VRITLVTKND--------PYKPPPHDLV--GKDCR-------   76
_XP_032034471.1_    41  IQ---------IMNYY----GKGK----VRITLVTKND--------PYKPHPHDLV--GKDCR-------   76
_XP_037862911.1_    41  IQ---------IMNYY----GKGK----VRITLVTKND--------PYKPHPHDLV--GKDCR-------   76
_XP_003830911.1_    41  IQ---------IMNYY----GKGK----VRITLVTKND--------PYKPHPHDLV--GKDCR-------   76
_XP_004029331.1_    41  IQ---------IMNYY----GKGK----VRITLVTKND--------PYKPHPHDLV--GKDCR-------   76
_XP_002812051.1_    41  IQ---------IMNYY----GKGK----VRITLVTKND--------PYKPHPHDLV--GKDCR-------   76
_XP_011711623.1_    41  IQ---------IMNYY----GKGK----VRITLVTKND--------PYKPHPHDLV--GKDCR-------   76
_XP_033095023.1_    41  IQ---------IMNYY----GKGK----VRITLVTKND--------PYKPHPHDLV--GKDCR-------   76
_XP_002799280.1_    41  IQ---------IMNYY----GKGK----VRITLVTKND--------PYKPHPHDLV--GKDCR-------   76
_XP_011853172.1_    41  IQ---------IMNYY----GKGK----VRITLVTKND--------PYKPHPHDLV--GKDCR-------   76
_XP_050609443.1_    41  IQ---------IMNYY----GKGK----VRITLVTKND--------PYKPHPHDLV--GKDCR-------   76
_XP_003908735.1_    41  IQ---------IMNYY----GKGK----VRITLVTKND--------PYKPHPHDLV--GKDCR-------   76
_XP_025209693.1_    41  IQ---------IMNYY----GKGK----VRITLVTKND--------PYKPHPHDLV--GKDCR-------   76
```

```
_XP_023084087.1_    41  IQ---------IMNYY----GKGK----VRITLVTKND--------PYKPHPHDLV--GKDCR-------   76
_XP_021780539.1_    41  IQ---------IMNYY----GKGK----VRITLVTKND--------PYKPHPHDLV--GKDCR-------   76
_XP_017704568.1_    41  IQ---------IMNYY----GKGK----VRITLVTKND--------PYKPHPHDLV--GKDCR-------   76
_XP_010375811.1_    41  IQ---------IMNYY----GKGK----VRITLVTKND--------PYKPHPHDLV--GKDCR-------   76
_XP_030776399.1_    41  IQ---------IMNYY----GKGK----VRITLVTKND--------PYKPHPHDLV--GKDCR-------   76
_XP_011797211.1_    41  IQ---------IMNYY----GKGK----VRITLVTKND--------PYKPHPHDLV--GKDCR-------   76
_XP_012332682.1_    41  IQ---------IMNYY----GKGK----VRITLVTKND--------PYKPHPHDLV--GKDCR-------   76
_XP_010331875.2_    41  IQ---------IMNYY----GKGK----VRITLVTKND--------PYKPHPHDLV--GKDCR-------   76
_XP_012332683.1_    41  IQ---------IMNYY----GKGK----VRITLVTKND--------PYKPHPHDLV--GKDCR-------   76
_XP_017400475.1_    41  IQ---------IMNYY----GKGK----VRITLVTKND--------PYKPHPHDLV--GKDCR-------   76
_XP_008568357.1_    41  IQ---------IMNYY----GKGK----VRITLVTKND--------PYKPHPHDLV--GKDCR-------   76
_XP_012620284.1_    41  IQ---------IMNYY----GKGK----VRITLVTKND--------PYKPHPHDLV--GKDCR-------   76
_XP_002709741.2_    41  IQ---------IINYY----GKGK----VRITLVTKND--------PYKPHPHDLV--GKDCR-------   76
_XP_022452943.1_    41  IQ---------IMNYY----GKGK----VRITLVTKNI--------PHKPHPHDLV--GKDCR-------   76
_XP_045405625.1_    41  IQ---------IMNYY----GKGK----VRITLVTKND--------PYKPHPHDLV--GKDCR-------   76
_XP_004280707.1_    41  IQ---------IMNYY----GKGK----VRITLVTKNI--------PHKPHPHDLV--GKDCR-------   76
_XP_030733169.1_    41  IQ---------IMNYY----GKGK----VRITLVTKNI--------PHKPHPHDLV--GKDCR-------   76
_TEA23022.1_        41  IQ---------IMNYY----GKGK----VRITLVTKNI--------PHKPHPHDLV--GKDCR-------   76
_XP_012494629.1_    41  IQ---------IMNYY----GKGK----VRITLVTKND--------PYKPHPHDLV--GKDCR-------   76
_XP_010950191.1_    41  IQ---------IMNYY----GKGK----VRITLVTKND--------PYKPHPHDLV--GKDCR-------   76
_XP_024608785.1_    41  IQ---------IMNYY----GKGK----VRITLVTKNI--------PHKPHPHDLV--GKDCR-------   76
_XP_006201637.1_    41  IQ---------IMNYY----GKGK----VRITLVTKND--------PYKPHPHDLV--GKDCR-------   76
_XP_019285354.1_    41  IQ---------IMNYY----GKGK----VRITLVTKND--------PYKPHPHDLV--GKDCR-------   76
_XP_032509224.1_    41  IQ---------IMNYY----GKGK----VRITLVTKNI--------PHKPHPHDLV--GKDCR-------   76
_XP_047379355.1_    41  IQ---------IMNYY----GKGK----VRITLVTKND--------PYKPHPHDLV--GKDCR-------   76
_XP_032981134.1_    41  IQ---------IMNYY----GKGK----VRITLVTKND--------PYKPHPHDLV--GKDCR-------   76
_XP_005600018.1_    41  IQ---------IMNYY----GKGK----VRITLVTKSD--------PYKPHPHDLV--GKDCR-------   76
_XP_046517051.1_    41  IQ---------IMNYY----GKGK----VRITLVTKSD--------PYKPHPHDLV--GKDCR-------   76
_XP_007464205.1_    41  IQ---------IMNYY----GKGK----VRITLVTKND--------PHKPHPHDLV--GKDCR-------   76
_XP_019572903.1_    41  IQ---------IMNYY----GKGK----VRITLVTKND--------PYKPHPHDLV--GKDCR-------   76
_XP_007189755.1_    41  IQ---------IMNYY----GKGK----VRITLVTKND--------PYKPHPHDLV--GKDCR-------   76
_XP_004397771.1_    41  IQ---------IMNYY----GKGK----VRITLVTKND--------PYKPHPHDLV--GKDCR-------   76
_XP_004435713.1_    41  IQ---------IMHYY----GKGK----VRITLVTKND--------PYKPHPHDLV--GKDCR-------   76
_XP_008698124.1_    41  IQ---------IMNYY----GKGK----VRITLVTKND--------PYKPHPHDLV--GKDCR-------   76
_XP_023989255.1_    41  IQ---------IMNYY----GKGK----VRITLVTKNK--------PYKPHPHDLV--GKDCR-------   76
_XP_011233012.2_    41  IQ---------IMNYY----GKGK----VRITLVTKND--------PYKPHPHDLV--GKDCR-------   76
_XP_025724444.1_    41  IQ---------IMNYY----GKGK----VRITLVTKND--------PYKPHPHDLV--GKDCR-------   76
_XP_005626186.1_    41  IQ---------IMNYY----GKGK----VRITLVTKND--------PYKPHPHDLV--GKDCR-------   76
_XP_045643991.1_    41  IQ---------IMNYY----GKGK----VRITLVTKND--------PYKPHPHDLV--GKDCR-------   76
```

```
_XP_034857383.1_    41  IQ---------IMNYY----GKGK----VRITLVTKND--------PYKPHPHDLV--GKDCR-------   76
_XP_027945501.1_    41  IQ---------IMNYY----GKGK----VRITLVTKND--------PYKPHPHDLV--GKDCR-------   76
_XP_026341094.1_    41  IQ---------IMNYY----GKGK----VRITLVTKND--------PYKPHPHDLV--GKDCR-------   76
_EFB29923.1_        41  IQ---------IMNYY----GKGK----VRITLVTKND--------PYKPHPHDLV--GKDCR-------   76
_XP_045740897.1_    41  IQ---------IMNYY----GKGK----VRITLVTKND--------PYKPHPHDLV--GKDCR-------   76
_CAD7677454.1_      41  IQ---------IMNYY----GKGK----VRITLVTKND--------PYKPHPHDLV--GKDCR-------   76
_XP_035577553.1_    41  IQ---------IMNYY----GKGK----VRITLVTKND--------PYKPHPHDLV--GKDCR-------   76
_XP_006733255.1_    41  IQ---------IMNYY----GKGK----VRITLVTKND--------PYKPHPHDLV--GKDCR-------   76
_XP_032266820.1_    41  IQ---------IMNYY----GKGK----VRITLVTKND--------PYKPHPHDLV--GKDCR-------   76
_XP_041611730.1_    41  IQ---------IMNYY----GKGK----VRITLVTKND--------PYKPHPHDLV--GKDCR-------   76
_XP_048971854.1_    41  IQ---------IMNYY----GKGK----VRITLVTKND--------PYKPHPHDLV--GKDCR-------   76
_XP_021554840.1_    41  IQ---------IMNYY----GKGK----VRITLVTKND--------PYKPHPHDLV--GKDCR-------   76
_VCW78562.1_        41  IQ---------IMNYY----GKGK----VRITLVTKND--------PYKPHPHDLV--GKDCR-------   76
_XP_034857381.1_    41  IQ---------IMNYY----GKGK----VRITLVTKND--------PYKPHPHDLV--GKDCR-------   76
_XP_008524231.1_    38  IQ---------IMNYY----GKGK----VRITLVTKSD--------PYKPHPHDLV--GKDCR-------   73
_XP_045740895.1_    41  IQ---------IMNYY----GKGK----VRITLVTKND--------PYKPHPHDLV--GKDCR-------   76
_XP_045834746.1_    41  IQ---------IMNYY----GKGK----VRITLVTKND--------PYKPHPHDLV--GKDCR-------   76
_XP_035932906.1_    41  IQ---------IMNYY----GKGK----VRITLVTKND--------PYKPHPHDLV--GKDCR-------   76
_XP_030883859.1_    41  IQ---------IMNYY----GKGK----VRITLVTKND--------PYKPHPHDLV--GKDCR-------   76
_XP_032266818.1_    41  IQ---------IMNYY----GKGK----VRITLVTKND--------PYKPHPHDLV--GKDCR-------   76
_XP_024408528.2_    41  IQ---------IMNYC----GKGK----VRITLVTKNE--------PFKPHPHDLV--GKDCR-------   76
_XP_004580347.1_    41  IQ---------IMNYY----GKGK----VRITLVTKND--------PYKPHPHDLV--GKDCR-------   76
_XP_030166525.1_    41  IQ---------IMNYY----GKGK----VRITLVTKND--------PYKPHPHDLV--GKDCR-------   76
_XP_035932904.1_    41  IQ---------IMNYY----GKGK----VRITLVTKND--------PYKPHPHDLV--GKDCR-------   76
_XP_020040690.1_    41  IQ---------IMNYY----GKGK----VRITLVTKND--------PYKPHPHDLV--GKDCR-------   76
_XP_053445604.1_    41  IQ---------IMNYY----GKGK----VRITLVTKND--------PYKPHPHDLV--GKDCR-------   76
_XP_049507482.1_    41  IQ---------IMNYY----GKGK----VRITLVTKND--------PYKPHPHDLV--GKDCR-------   76
_XP_015335127.1_    41  IQ---------IMNYY----GKGK----VRITLVTKND--------PYKPHPHDLV--GKDCR-------   76
_XP_027790245.1_    41  IQ---------IMNYC----GKGK----VRITLVTKND--------PYKPHPHDLV--GKDCR-------   76
_XP_003984077.1_    41  IQ---------IMNYY----GKGK----VRITLVTKND--------PYKPHPHDLV--GKDCR-------   76
_XP_039096259.1_    41  IQ---------IMNYY----GKGK----VRITLVTKND--------PYKPHPHDLV--GKDCR-------   76
_XP_007075434.1_    41  IQ---------IMNYY----GKGK----VRITLVTKND--------PYKPHPHDLV--GKDCR-------   76
_XP_022360130.1_    41  IQ---------IMNYY----GKGK----VRITLVTKND--------PYKPHPHDLV--GKDCR-------   76
_XP_047707378.1_    41  IQ---------IMNYY----GKGK----VRITLVTKND--------PYKPHPHDLV--GKDCR-------   76
_XP_026243031.1_    41  IQ---------IMNYY----GKGK----VRITLVTKND--------PYKPHPHDLV--GKDCR-------   76
_XP_037383340.1_    41  IQ---------IKNYC----GKGK----VRITLVTKND--------PYKPHPHDLV--GKDCR-------   76
_XP_005322095.1_    41  IQ---------IMNYY----GKGK----VRITLVTKND--------PYKPHPHDLV--GKDCR-------   76
_XP_005662584.1_    41  IQ---------IKNYF----GKGK----VRITLVTKND--------PYKPHPHDLV--GKDCR-------   76
_XP_040080101.1_    41  IQ---------ILNYY----GKGK----VRITLVTKND--------PYKPHPHDLV--GKDCR-------   76
```

```
_XP_017503999.1_    41  IQ---------IKNYY----GKGK----VRITLVTKND--------PYKPHPHDLV--GKDCR-------   76
_XP_044119954.1_    41  IQ---------IMNYY----GKGK----VRITLVTKND--------PYKPHPHDLV--GKDCR-------   76
_XP_032208110.1_    41  IQ---------IMNYY----GKGK----VRITLVTKND--------PYKPHPHDLV--GKDCR-------   76
_XP_012586936.1_    41  IQ---------IRNYY----GKGK----VRITLVTKND--------PYKPHPHDLV--GKDCR-------   76
_XP_032729745.1_    41  IQ---------IMNYY----GKGK----VRITLVTKND--------PYKPHPHDLV--GKDCR-------   76
_XP_052504199.1_    41  IQ---------ILNYY----GKGK----VRITLVTKND--------PYKPHPHDLV--GKDCR-------   76
_XP_054431389.1_    41  IQ---------IMNYY----GKGK----VRITLVTKNE--------PFKPHPHDLV--GKDCR-------   76
_XP_023576632.1_    41  IQ---------IMNYY----GKGK----VRITLVTKND--------PYKPHPHDLV--GKDCR-------   76
_XP_004742070.1_    41  IQ---------IMNYY----GKGK----VRITLVTKND--------PYKPHPHDLV--GKDCR-------   76
_XP_037663267.1_    41  IQ---------IMNYC----GKGK----VRITLVTKND--------PYKPHPHDLV--GKDCR-------   76
_XP_004005978.1_    41  IQ---------ILNYY----GKGK----VRITLVTKND--------PYKPHPHDLV--GKDCR-------   76
_XP_036781908.1_    41  IQ---------IKNYY----GKGK----VRITLVTKND--------PYKPHPHDLV--GKDCR-------   76
_XP_040849333.1_    41  IQ---------IMNYY----GKGK----VRITLVTKND--------PYKPHPHDLV--GKDCR-------   76
_XP_005686691.1_    41  IQ---------ILNYY----GKGK----VRITLVTKND--------PYKPHPHDLV--GKDCR-------   76
_XP_023367900.1_    41  IQ---------IMNYY----GKGK----VRITLVTKND--------PYKPHPHDLV--GKDCR-------   76
_KAF0879576.1_      41  IQ---------IMNYY----GKGK----VRITLVTKND--------PYKPHPHDLV--GKDCR-------   76
_XP_029793284.1_    41  IQ---------IMNYY----GKGK----VRITLVTKND--------PYKPHPHDLV--GKDCR-------   76
_NP_001179899.1_    41  IQ---------ILNYY----GKGK----VRITLVTKND--------PYKPHPHDLV--GKDCR-------   76
_XP_020764718.1_    41  IQ---------ISNYY----GKLK----VRITLVTKND--------PYKPHPHDLV--GKDCR-------   76
_XP_054431388.1_    41  IQ---------IMNYY----GKGK----VRITLVTKNE--------PFKPHPHDLV--GKDCR-------   76
_XP_037663265.1_    41  IQ---------IMNYC----GKGK----VRITLVTKND--------PYKPHPHDLV--GKDCR-------   76
_XP_019825763.1_    41  IQ---------ILNYY----GKGK----VRITLVTKND--------PYKPHPHDLV--GKDCR-------   76
_XP_005889096.1_    41  IQ---------ILNYY----GKGK----VRITLVTKND--------PYKPHPHDLV--GKDCR-------   76
_XP_043324571.1_    41  IQ---------ISNYY----GKLK----VRITLVTKND--------PYKPHPHDLV--GKDCR-------   76
_KAJ1078272.1_      41  IQ---------ILNYY----GKGK----VRITLVTKND--------PYKPHPHDLV--GKDCR-------   76
_XP_036190339.1_    41  IQ---------IKNCY----GKGK----VRITLVTKND--------PFKPHPHDLV--GKDCR-------   76
_XP_013005522.1_    41  IQ---------IMNYY----GKGK----VRITLVTKND--------PYKPHPHDLV--GKDCR-------   76
_XP_006073936.1_    41  IQ---------ILNYY----GKGK----VRITLVTKND--------PYKPHPHDLV--GKDCR-------   76
_XP_016069529.1_    41  IQ---------IMNYY----GKGK----VRITLVTKND--------PFKPHPHDLV--GKDCR-------   76
_KAB0354136.1_      41  IQ---------ILNYY----GKLK----VRITLVTKND--------PYKPHPHDLV--GKDCR-------   76
_XP_029424871.1_    41  IQ---------IMNYY----GKGK----VRITLVTKND--------PYKPHPHDLV--GKDCR-------   76
_XP_036118792.1_    41  IQ---------IMNYY----GKGK----VRITLVTKNE--------PFKPHPHDLV--GKDCR-------   76
_XP_037019725.2_    40  IQ---------IMNYC----GKGK----VRITLVTKNE--------PYKPHPHDLV--GKDCR-------   75
_XP_047602572.1_    41  IQ---------IMNYY----GKGK----VRITLVTKND--------PYKPHPHDLV--GKDCR-------   76
_XP_014586639.1_    41  IQ---------IMNYY----GKGK----VRITLVTKSD--------PYKPHPHDLV--GKDCR-------   76
_XP_046517052.1_    41  IQ---------IMNYY----GKGK----VRITLVTKSD--------PYKPHPHDLV--GKDCR-------   76
_XP_006909635.2_    41  IQ---------IMNYY----GKGK----VRITLVTKNE--------PYKPHPHDLV--GKDCR-------   76
_XP_011362222.1_    41  IQ---------IMNYY----GKGK----VRITLVTKNE--------PYKPHPHDLV--GKDCR-------   76
_XP_045684409.1_    41  IQ---------IMNYC----GKGK----VRITLVTKNE--------PFKPHPHDLV--GKDCR-------   76
```

```
_XP_048208816.1_    41  IQ---------IVNYY----GKGK----VRITLVTKND--------PYKPHPHDLV--GKDCR-------   76
_XP_052590629.1_    41  IQ---------IMNYY----GKGK----VRITLVTKND--------PYKPHPHDLV--GKDCR-------   76
_XP_032353499.1_    41  IQ---------IMNYY----GKGK----VRITLVTKND--------PYKPHPHDLV--GKDCR-------   76
_XP_038200327.1_    41  IQ---------IMNYY----GKGK----VRITLVTKND--------PYKPHPHDLV--GKDCR-------   76
_XP_051056550.1_    41  IQ---------VMNYY----GKGK----VRITLVTKND--------PYKPHPHDLV--GKDCR-------   76
_XP_012805850.2_    41  IQ---------IMNYY----GKGK----VRITLVTKND--------PYKPHPHDLV--GKDCR-------   76
_XP_016050906.1_    41  IQ---------IKNYY----GKGK----VRITLVTKND--------PYKPHPHDLV--GKDCR-------   76
_XP_030166526.1_    41  IQ---------IMNYY----GKGK----VRITLVTKND--------PYKPHPHDLV--GKDCR-------   76
_XP_036903878.1_    40  IQ---------IMNYC----GKGK----VRITLVTKNE--------PYKPHPHDLV--GKDCR-------   75
_CAH7004032.1_      41  IQ---------VMNYY----GKGK----VRITLVTKND--------PYKPHPHDLV--GKDCR-------   76
_XP_051056549.1_    41  IQ---------VMNYY----GKGK----VRITLVTKND--------PYKPHPHDLV--GKDCR-------   76
_XP_042547166.1_    41  IQ---------IMNYY----GKGK----VRITLVTKND--------PYKPHPHDLV--GKDCR-------   76
_XP_054584456.1_    41  IQ---------IMNCY----GKGK----VRITLVTKND--------PFKPHPHDLV--GKDCR-------   76
_XP_049996768.1_    41  IQ---------IMNYY----GKGK----VRITLVTKND--------PYKPHPHDLV--GKDCR-------   76
_XP_027243805.1_    41  IQ---------VMNYY----GKGK----VRITLVTKND--------PYKPHPHDLV--GKDCR-------   76
_XP_047707379.1_    41  IQ---------IMNYY----GKGK----VRITLVTKND--------PYKPHPHDLV--GKDCR-------   76
_XP_026645370.1_    41  IQ---------IMNYY----GKGK----VRITLVTKND--------PYKPHPHDLV--GKDCR-------   76
_XP_014302519.1_    41  IQ---------IKNCY----GKGK----VRITLVTKND--------PFKPHPHDLV--GKDCR-------   76
_XP_049726303.1_    41  IQ---------IMNYY----GKGK----VRITLVTKND--------PYKPHPHDLV--GKDCR-------   76
_XP_049726302.1_    41  IQ---------IMNYY----GKGK----VRITLVTKND--------PYKPHPHDLV--GKDCR-------   76
_XP_012969026.1_    41  IQ---------VMNYY----GKGK----VRITLVTKND--------PYKPHPHDLV--GKDCR-------   76
_XP_008524232.1_    38  IQ---------IMNYY----GKGK----VRITLVTKSD--------PYKPHPHDLV--GKDCR-------   73
_XP_051020904.1_    41  IQ---------IMNYY----GKGK----VRITLVTKND--------PYKPHPHDLV--GKDCR-------   76
_XP_023590712.1_    41  IQ---------IMNYY----GKGK----VRITLVTKND--------PYKPHPHDLV--GKDCR-------   76
_XP_027243803.1_    41  IQ---------VMNYY----GKGK----VRITLVTKND--------PYKPHPHDLV--GKDCR-------   76
_XP_040589151.1_    41  IQ---------VMNYY----GKGK----VRITLVTKND--------PYKPHPHDLV--GKDCR-------   76
_XP_023412959.1_    41  IQ---------IMNYY----GKGK----VRITLVTKND--------PYKPHPHDLV--GKDCR-------   76
_XP_036057111.1_    41  IQ---------IMNYY----GKGK----VRITLVTKND--------PYKPHPHDLV--GKDCR-------   76
_XP_027822602.1_    41  IQ---------ILNYY----GKGK----VRITLVTKND--------PYKPHPHDLV--GKDCR-------   76
_XP_031322949.1_    41  IQ---------IMNYY----GKGK----VRITLVTKND--------PYKPHPHDLV--GKDCR-------   76
_XP_028634966.1_    41  IQ---------IMNYY----GKGK----VRITLVTKND--------PYKPHPHDLV--GKDCR-------   76
_XP_031531529.1_    41  IQ---------IMNYY----GKGK----VRITLVTKND--------PYKPHPHDLV--GKDCR-------   76
_XP_006073937.1_    41  IQ---------ILNYY----GKGK----VRITLVTKND--------PYKPHPHDLV--GKDCR-------   76
_XP_034364887.1_    41  IQ---------IMNYY----GKGK----VRITLVTKND--------PYKPHPHDLV--GKDCR-------   76
_XP_021482246.1_    41  IQ---------VMNYY----GKGK----VRITLVTKND--------PYKPHPHDLV--GKDCR-------   76
_KAF4016326.1_      41  IQ---------ISNYY----GKLK----VRITLVTKND--------PYKPHPHDLV--GKDCR-------   76
_XP_023602319.1_    41  IQ---------IKNCY----GKGK----VRITLVTKND--------PFKPHPHDLV--GKDCR-------   76
_XP_052054164.1_    41  IQ---------IMNYY----GKGK----VRITLVTKND--------PYKPHPHDLV--GKDCR-------   76
_XP_036291310.1_    41  IQ---------IMNCY----GKGK----VRITLVTKND--------PYKPHPHDLV--GKDCR-------   76
```

```
_XP_013370168.1_    41  IQ---------IMNYY----GKGK----VRITLVTKND--------PYKPHPHDLV--GKDCR-------   76
_XP_023590722.1_    41  IQ---------IMNYY----GKGK----VRITLVTKND--------PYKPHPHDLV--GKDCR-------   76
_XP_048291328.1_    41  IQ---------IMNYY----GKVK----VRITLVTKND--------PYKPHPHDLV--GKDCR-------   76
_XP_032772910.1_    41  IQ---------IMNYY----GKGK----VRITLVTKND--------PYKPHPHDLV--GKDCR-------   76
_XP_008768670.2_    41  IQ---------IMNYY----GKGK----VRITLVTKND--------PYKPHPHDLV--GKDCR-------   76
_XP_014302521.1_    41  IQ---------IKNCY----GKGK----VRITLVTKND--------PFKPHPHDLV--GKDCR-------   76
_XP_031194882.1_    41  IQ---------IMNYY----GKGK----VRITLVTKND--------PYKPHPHDLV--GKDCR-------   76
_NP_033070.2_       41  VQ---------IMNYY----GKGK----IRITLVTKND--------PYKPHPHDLV--GKDCR-------   76
_XP_036291312.1_    41  IQ---------IMNCY----GKGK----VRITLVTKND--------PYKPHPHDLV--GKDCR-------   76
_XP_021033157.1_    41  VQ---------IMNYY----GKGK----IRITLVTKND--------PYKPHPHDLV--GKDCR-------   76
_XP_015849136.1_    41  IQ---------IMNYY----GKGK----VRITLVTKND--------PYKPHPHDLV--GKDCR-------   76
_XP_045150027.1_    41  IQ---------IMNYY----GKGK----VRITLITKND--------PYKPHPHDLV--GKDCR-------   76
_XP_021066684.1_    41  IQ---------IMNYY----GKGK----IRITLVTKND--------PYKPHPHDLV--GKDCR-------   76
_XP_006881088.1_    41  IQ---------IMNYY----GKGK----VRITLITKND--------PYKPHPHDLV--GKDCR-------   76
_XP_008524234.1_    38  IQ---------IMNYY----GKGK----VRITLVTKSD--------PYKPHPHDLV--GKDCR-------   73
_XP_021482260.1_    41  IQ---------VMNYY----GKGK----VRITLVTKND--------PYKPHPHDLV--GKDCR-------   76
_XP_007125500.1_    41  IQ---------IMNYY----GKGK----VRITLVTKNK--------PYKPHPHDLV--GKDCR-------   76
_XP_028352731.1_    41  IQ---------IMNYY----GKGK----VRITLVTKNK--------PYKPHPHDLV--GKDCR-------   76
_XP_028352733.1_    41  IQ---------IMNYY----GKGK----VRITLVTKNK--------PYKPHPHDLV--GKDCR-------   76
_XP_028352734.1_    41  IQ---------IMNYY----GKGK----VRITLVTKNK--------PYKPHPHDLV--GKDCR-------   76
_XP_028352736.1_    41  IQ---------IMNYY----GKGK----VRITLVTKNK--------PYKPHPHDLV--GKDCR-------   76
_XP_028352735.1_    41  IQ---------IMNYY----GKGK----VRITLVTKNK--------PYKPHPHDLV--GKDCR-------   76
_ERE88482.1_        41  IQ---------VMNYY----GKGK----VRITLVTKND--------PYKPHPHDLV--GKDCR-------   76
_KAF3826750.1_          ----------------------------------------------------------------------
_OBS70420.1_        30  IQ---------IMNYY----GKGK----VRITLVTKND--------PYKPHPHDLV--GKDCR-------   65
_XP_006878094.1_     4  FQ---------IMNYY----GKGK----VRITLITKND--------PYKPHPHDLV--GKDCR-------   39
_XP_008978905.3_     1  ------------MNYY----GKGK----VRITLVTKND--------PYKPHPHDLV--GKDCR-------   33
_XP_032147941.1_     1  ------------MNYY----GKGK----VRITLVTKND--------PYKPHPHDLV--GKDCR-------   33
_XP_008524233.1_     1  -E---------IMNYY----GKGK----VRITLVTKSD--------PYKPHPHDLV--GKDCR-------   35
_XP_044628438.1_     1  ------------MNYY----GKGK----VRITLVTKSD--------PYKPHPHDLV--GKDCR-------   33
_XP_010608101.1_     1  ------------MNYY----GKGK----VRITLVTKND--------PYKPHPHDLV--GKDCR-------   33
_KAF5918007.1_       1  -R---------IMHYY----GKGK----VRITLVTKND--------PYKPHPHDLV--GKDCR-------   35
_XP_021101942.1_     1  ------------MNYY----GKGK----VRITLVTKND--------PYKPHPHDLV--GKDCR-------   33
_ABM89308.1_            ----------------------------------------------------------------------
_MBW00678.1_            ----------------------------------------------------------------------
_NP_001096177.1_    41  IK---------IMNYT----GKGI----LRITLVTKNE--------PYKPHPHDLV--GKDCR-------   76
_OCT77375.1_        41  IK---------IMNYT----GKGI----VRITLVTKNE--------PHKPHPHDLV--GKDCR-------   76
_AAI70187.1_        41  IK---------IMNYT----GKGI----VRITLVTKNE--------PHKPHPHDLV--GKDCR-------   76
_AAI69362.1_        41  IK---------IMNYT----GKGI----VRITLVTKNE--------PHKPHPHDLV--GKDCR-------   76
```

```
_XP_041418586.1_    41  IK---------IMNYT----GKGI----VRITLVTKNE--------PHKPHPHDLV--GKDCR-------   76
_XP_041418585.1_    41  IK---------IMNYT----GKGI----VRITLVTKNE--------PHKPHPHDLV--GKDCR-------   76
_XP_041418587.1_    41  IK---------IMNYT----GKGI----VRITLVTKNE--------PHKPHPHDLV--GKDCR-------   76
_XP_053316221.1_    41  IQ---------IMNYV----GKGK----VRVTLVTKNE--------PYRPHPHDLV--GKDSR-------   76
_XP_053568018.1_    41  IQ---------IMNYI----GKGK----VRVTLVTKNE--------PYKPHPHDLV--GRDCR-------   76
_KAG8443975.1_      41  IK---------IMNYV----GKGK----VRITLVTKNE--------PYKPHPHDLV--GKDCR-------   76
_XP_044145067.1_    41  IQL-------QNMNYN----GKGK----VRVTLVTKNE--------PYRPHPHDLV--GKDCR-------   78
_KAG9484268.1_      41  IQL-------QNMNYN----GKGK----VRVTLVTKNE--------PYRPHPHDLV--GKDCR-------   78
_XP_040285660.1_    41  IQL-------QNMNYN----GKGK----VRVTLVTKNE--------PYRPHPHDLV--GKDCR-------   78
_XP_040285659.1_    41  IQL-------QNMNYN----GKGK----VRVTLVTKNE--------PYRPHPHDLV--GKDCR-------   78
_KAG8583825.1_      41  IQL-------HNMSYN----DKGK----VRVTLVTKNE--------PYRPHPHDLV--GKDCR-------   78
_XP_018411072.1_    41  IQL-------QNLNYN----VKGK----VKVTLVTKNE--------PYRPHPHDLV--GKDCR-------   78
_XP_040207073.1_    41  IQL-------QNLGYG----VKGK----VRVTLVTKNE--------PYRPHPHDLV--GKDCR-------   78
_NP_001081750.1_    42  RL-----------LII----LKRD----SRITLVTKKE--------PHKPHPHDLV--GKDCR-------   75
_PIO39092.1_            ----------------------------------------------------------------------
_PIO39091.1_            ----------------------------------------------------------------------
_XP_041035877.1_    41  IE---------IVNYL----GPAR----IRICLVTKKE--------PYKPHPHDLV--GKDCK-------   76
_XP_042202691.1_    41  IE---------IMNYL----GRAR----IRISLVTKKE--------PFKPHPHDLV--GKDCK-------   76
_XP_048475509.1_    30  IE---------IVNYV----GPAR----IRICLVTKKE--------PYKPHPHDLV--GKDCK-------   65
_XP_048380947.1_    30  IE---------IVNYV----GPAR----IRICLVTKKE--------PFKPHPHDLV--GKDCK-------   65
_XP_051901152.1_    34  IE---------IVNYL----GPAR----VRICLVTKKE--------PYKPHPHDLV--GKDCK-------   69
_BAD60879.1_        28  IR---------INNFNG---PAAI----VVVSCVTKDK--------PYCPHPHNLV--GQDCR-------   64
_XP_050405370.1_    41  IK---------IQNHR----GPAI----VVVSCVTKDS--------PYKPHPHCLV--GKDCK-------   76
_XP_009063713.1_    41  IK---------IQNHR----GPAI----VVVSCVTKDS--------PHKPHPHALV--GKDCK-------   76
_CAB0044847.1_      43  LR---------VLNCR----GPGL----VLVSLVTEQA--------PYYPHPNRLA--RLRGGAADG---   82
_XP_048259911.1_    41  IK---------IRNYK----GPAI----VVVSCVTRDAA------PLCKPHPHSLV--GKDCK-------   78
_ADI72431.1_        41  IK---------IRNYK----GPAI----VVVSCVTRDAA------PNCKPHPHSLV--GKDCK-------   78
_CAG5133522.1_      41  IK---------IHGCN----GPAI----IVVSCVTKDNP------PHCKPHPHAIV--GRDCK-------   78
_KAG5688355.1_          ----------------------------------------------------------------------
_AMT84567.1_        41  IE---------ICNYNG---PSSI----VVVSCVTKDD--------KPRPHPHNLV--GQDCS-------   77
_XP_052276378.1_    41  IK---------IHNYS----GPAV----IVVSCVTKDE--------PYKPHPHNLV--GKDCK-------   76
_XP_052276377.1_    41  IK---------IHNYS----GPAV----IVVSCVTKDE--------PYKPHPHNLV--GKDCK-------   76
_AAK72690.1_        41  IK---------IHNYT----GTAV----IVVSCVTKDQ--------PYEPHPHNLV--GRDCK-------   76
_XP_011414731.2_    41  IK---------IHNYT----GTAV----IVVSCVTKDQ--------PYEPHPHNLV--GRDCK-------   76
_XP_052699807.1_    41  IK---------IHNYT----GTAV----IVVSCVTKDQ--------PYEPHPHNLV--GRDCK-------   76
_NP_001292261.1_    41  IK---------IHNYT----GTAV----IVVSCVTKDQ--------PYEPHPHNLV--GRDCK-------   76
_XP_048738054.1_    41  IK---------IHNYT----GTAV----IVVSCVTKDQ--------PYEPHPHNLV--GRDCK-------   76
_XP_052699808.1_    41  IK---------IHNYT----GTAV----IVVSCVTKDQ--------PYEPHPHNLV--GRDCK-------   76
_XP_022332457.1_    41  IK---------INNYT----GTAV----IVVSCVTKDT--------PYEPHPHNLV--GRDCK-------   76
```

```
_ABL63469.1_        40  IK---------IHNYT----GTAV----IVVSCVTKDT--------PYDPHPHNLV--GRDCK-------   75
_XP_033733822.1_    41  IK---------IMNYT----GTAV----VVVSCVTKDA--------PYEPHPHNLV--GRDCK-------   76
_XP_021347355.1_    41  IK---------IVNYS----GTAV----VVVSCVTKDA--------PYEPHPHNLV--GRDCK-------   76
_UOA58607.1_        41  IK---------IVNYT----GTAV----VVVSCVTKDT--------PYEPHPHNLV--GRDCK-------   76
_XP_021347356.1_    41  IK---------IVNYS----GTAV----VVVSCVTKDA--------PYEPHPHNLV--GRDCK-------   76
_CAG2186872.1_      40  IK---------IHQYQ----GVAV----IVVSCVTKDN--------PYEPHPHNLV--GKDCK-------   75
_XP_052058915.1_    40  IK---------IHQYQ----GVAV----IVVSCVTKDH--------PYEPHPHNLV--GKDCK-------   75
_KAJ8309232.1_      41  IK---------IHNHI----GTAV----IVVSCVTKDQ--------PYEPHPHNLV--GRDCK-------   76
_UZH84475.1_        41  IK---------IHNYN----GPAV----IVVSCVTKDE--------PYRPHPHNLV--GKDCK-------   76
_ALA99939.1_        41  IK---------IHNYN----GPAV----IVVSCVTKEE--------PYKPHPHNLV--GKDCK-------   76
_XP_052767764.1_    41  IK---------IHNYN----GPAV----IVVSCVTKDE--------PYKPHPHNLV--GKDCK-------   76
_ADD25211.1_        41  IK---------IVNYT----GTAV----VVVSCVTKDT--------PYEPHPHNLV--GRDCK-------   76
_XP_052224245.1_    41  MK---------VHNYS----GPAE----IVVSCVTKDD--------PYKPHPHNLV--GKDCK-------   76
_KAJ8314234.1_      41  IK---------IHNYV----GTAV----IVVSCVTKDT--------PYKPHPHNLV--GKNCR-------   76
_VDH95719.1_         1  -----------IHQYQ----GVAV----IVVSCVTKDN--------PYEPHPHNLV--GKDCK-------   34
_XP_041352913.1_    41  IK---------IHNYS----GNAI----VVVSCVTKEGA------PHCKPHPHSLV--GKDCK-------   78
_XP_046555158.1_    41  IK---------IRNYK----GPAI----VVVSCVTRDAL------PHCKPHPHSLV--GKDCK-------   78
_AAW33559.1_        41  IK---------IRNYK----GPAI----VVVSCVTKDPH------PHCKPHPHSLV--GKDCK-------   78
_ABY87435.1_        28  IK---------IRNYK----GLAI----VVVSCVTKDPH------PHCKPHPHSLV--GKDCK-------   65
_XP_009028834.1_    41  IK---------IHNFEG---PAAI----VVVSCVTKDK--------PYYPHPHNLV--GQDCK-------   77
_XP_025106109.1_    41  IK---------IHNYK----GQAI----IVVSCVTKDPE------PHCKPHPHSLV--GRDCK-------   78
_NP_001298191.1_    41  IK---------IHNYN----GPAI----IVVSCVTKENP------PHCKPHPHAIV--GRDCK-------   78
_KAH9519545.1_      41  IK---------IHNYN----GPAI----IVVSCVTKENP------PHCKPHPHAIV--GRDCK-------   78
_RUS74561.1_        41  IK---------VHNYS----GPAI----IVVSCVTKENP------PHCKPHPHAIV--GRDCK-------   78
_GFO11973.1_        41  IK---------VHNYT----GPAI----IVVSCVTKENP------PLCKAHPHAIV--GRDCK-------   78
_CAG5132534.1_      41  IK---------IHNYT----GPAI----IVVSCVTKELP------PHCKPHPHAVV--GRDCK-------   78
_GFR69641.1_        41  IK---------VHNYS----GPAI----IVVSCVTKENP------PHCKPHPHAIV--GRDCK-------   78
_XP_035824873.1_    41  IK---------IHNYS----GAAI----IVVSCVTKENP------PHCKPHPHAIV--GRDCK-------   78
_AAY27981.1_        41  IQ---------VCNYQ----GPAI----VIVSCVTKDD--------IPKPHPHSLV--GKDCK-------   76
_XP_052827753.1_    40  IK---------ICNYK----GPAV----VIVSCVTKDD--------PPKPHPHSLV--GKDCK-------   75
_XP_029642523.1_    40  IK---------ICNYK----GPAV----VIVSCVTKDD--------PPKPHPHSLV--GKDCK-------   75
_CAE1288013.1_       1  -N---------VCNYQ----GPAI----VIVSCVTKDE--------KPKPHPHSLV--GKDCK-------   35
_ELU04547.1_        41  IK---------IHNYDG---PAAI----VVVSCVTKDN--------TPRPHPHSLV--GQDCN-------   77
_KAI0227899.1_      41  IR---------IENMDA---ASAV----IVVSCVSKDD--------IARPHPHSLT--GQNCK-------   77
_XP_044002103.1_    42  IR---------VIDYV----GPAF----VFASLVTKDSYN-----DKYRAHPHNLV--GNKCK-------   80
_XP_044002102.1_    39  IR---------VIGYV----GRAV----VVVSLVTKDSYS-----GKYRAHPHNLV--GKGCIE------   78
_XP_044002099.1_    39  IR---------VIGYV----GRAV----VVVSLVTKDSYS-----GKYRAHPHNLV--GKGCIE------   78
_XP_037026629.1_    41  IR---------ILNYE----GAAR----VCVSCVTIDE--------IPKPHPNCLV--GKPCS-------   76
_CAD7632570.1_      41  IQ---------IVGYGG---SRAM----VVVSCVTVSQ--------PYRQHPHHLV--GRVGCH------   78
```

```
_CAD7650738.1_      41  IQ---------IVNYRG---SRAM----VVVSCVTVSP--------PYRQHPHHLV--GRVGCH------   78
_XP_054163335.1_    41  IQ---------IMNYT----GSAL----AIVSCVTKDF--------PYRQHPHNLV--GERCS-------   76
_CAD7629985.1_      41  IQVTIS--HIYVVNYT----GSAL----AIVSCVTKDF--------PYRQHPHNLV--GERCT-------   83
_CAD7652069.1_      41  IQ---------VVNYS----GSAL----AIVSCVTKDF--------PYRQHPHNLV--GERCT-------   76
_XP_017954168.1_    41  IE---------IVDYS----GPVV----VVVSCVTRDK--------PYRQHPHWLV--SKEEADN-----   78
_XP_017858583.1_    41  IE---------IVDYS----GPVV----VVVSCVTRDK--------PYRQHPHWLV--SKEEVDN-----   78
_XP_002001588.1_    41  IE---------IVDYS----GPVV----VVVSCVTRDK--------PYRQHPHWLV--SKEEADN-----   78
_XP_017954163.1_    41  IE---------IVDYS----GPVV----VVVSCVTRDK--------PYRQHPHWLV--SKEEADN-----   78
_XP_023164563.2_    41  IE---------IVDYS----GPVV----VVVSCVTRDK--------PYRQHPHWLV--SKEEVDN-----   78
_TDG46702.1_        41  IE---------IVDYS----GPVV----VVVSCVTRDK--------PYRQHPHWLV--SKEEADN-----   78
_XP_030564898.1_    41  IE---------IVDYS----GPVV----VVVSCVTRDK--------PYRQHPHWLV--SKEEVDN-----   78
_XP_015024496.1_    41  IE---------IVDYS----GPVV----VVVSCVTRDK--------PYRQHPHWLV--SKEEVDN-----   78
_XP_034475238.1_    41  IQ---------ICNYNG---PVVI----LVISCVTVEQ--------PYLQHPHHLI--SKDEGSS-----   79
_XP_034475235.1_    41  IQ---------ICNYNG---PVVI----LVISCVTVEQ--------PYLQHPHHLI--SKDEGSS-----   79
_XP_017854415.1_    41  IE---------IVNFD----GPVV----VVVSLVTHEK--------PYRQHPHWLV--SKEEVEN-----   78
_XP_017854416.1_    41  IE---------IVNFD----GPVV----VVVSLVTHEK--------PYRQHPHWLV--SKEEVEN-----   78
_ALC40089.1_        41  IE---------IVNFD----GPVV----VVVSLVTHEK--------PYRQHPHWLV--SKEEVEN-----   78
_XP_030382983.1_    42  IE---------IVGYH----GKKV----VVVSCVTREK--------PYRQHPHWLV--SKDDSNL-----   79
_XP_030382981.1_    42  IE---------IVGYH----GKKV----VVVSCVTREK--------PYRQHPHWLV--SKDDSNL-----   79
_XP_001989397.1_    41  IE---------IVDYS----GPVV----VVVSCVTRDK--------PYRQHPHWLV--SKEEADN-----   78
_XP_002067135.2_    42  IE---------IVDYK----GPVV----VVVSCVTKDE--------PYRQHPHWLV--SKDEAEN-----   79
_XP_034475239.1_    41  IK---------ICNYN----GPVV----VVITCVTVDQ--------PYLQHPHKLI--ERGETNN-----   78
_KAH8411901.1_      41  IE---------IVGFD----GPVI----VIVSCVTSDP--------PYRQHPHWLV--SKDEAEN-----   78
_XP_016963143.1_    42  IE---------VCNYD----GPVV----IVVSCVTSDE--------PYRQHPHWLV--SKEEADA-----   79
_XP_037712361.1_    42  IE---------VCNYD----GPVV----IVVSCVTIDE--------PYRQHPHWLV--SKEEADA-----   79
_XP_016993627.2_    42  IE---------VCNYD----GPVV----IVVSCVTSDE--------PFRQHPHWLV--SKEEADA-----   79
_XP_016963141.1_    42  IE---------VCNYD----GPVV----IVVSCVTSDE--------PYRQHPHWLV--SKEEADA-----   79
_XP_016993624.2_    42  IE---------VCNYD----GPVV----IVVSCVTSDE--------PFRQHPHWLV--SKEEADA-----   79
_XP_037712358.1_    42  IE---------VCNYD----GPVV----IVVSCVTIDE--------PYRQHPHWLV--SKEEADA-----   79
_XP_001974449.1_    42  IE---------VCNYD----GPVI----IVVSCVTSDE--------PYRQHPHWLV--SKEEADA-----   79
_AAM48404.1_        42  IE---------VCNYD----GPVI----IVVSCVTSDE--------PFRQHPHWLV--SKEEADA-----   79
_NP_001162999.1_    42  IE---------VCNYD----GPVI----IVVSCVTSDE--------PFRQHPHWLV--SKEEADA-----   79
_XP_026835878.1_    42  IE---------VCNYD----GPVI----IVVSCVTSDE--------PYRQHPHWLV--SKEEADA-----   79
_AAA28465.1_        42  IE---------VCNYD----GPVI----IVVSCVTSDE--------PFRQHPHWLV--SKEEADA-----   79
_XP_002038912.1_    42  IE---------VCNYD----GPVI----IVVSCVTSDE--------PFRQHPHWLV--SKEEADA-----   79
_XP_033169558.1_    42  IE---------VCNFD----GPVI----IVVSCVTSDE--------PFRQHPHWLV--SKEEADA-----   79
_EDX05310.1_        42  IE---------VCNYD----GPVI----IVVSCVTSDE--------PFRQHPHWLV--SKEEADA-----   79
_NP_001162998.1_    42  IE---------VCNYD----GPVI----IVVSCVTSDE--------PFRQHPHWLV--SKEEADA-----   79
_XP_032579766.1_    42  IE---------VCNYD----GPVI----IVVSCVTSDE--------PFRQHPHWLV--SKEEADA-----   79
```

```
_XP_033169541.1_    42  IE---------VCNFD----GPVI----IVVSCVTSDE--------PFRQHPHWLV--SKEEADA-----   79
_XP_034669502.1_    42  IE---------VCDYT----GPVI----IIVSCVTSDE--------PYRQHPHWLV--SKEEADS-----   79
_XP_034125957.1_    42  IE---------VCDYT----GPVI----IIVSCVTSDA--------PYRQHPHWLV--SKEEADS-----   79
_XP_034669501.1_    42  IE---------VCDYT----GPVI----IIVSCVTSDE--------PYRQHPHWLV--SKEEADS-----   79
_XP_034125956.1_    42  IE---------VCDYT----GPVI----IIVSCVTSDA--------PYRQHPHWLV--SKEEADS-----   79
_XP_034125955.1_    42  IE---------VCDYT----GPVI----IIVSCVTSDA--------PYRQHPHWLV--SKEEADS-----   79
_XP_017152033.1_    42  IE---------VCDYN----GPVT----VVVSCVTSDE--------PYRQHPHWLV--SKEEADS-----   79
_XP_017152031.1_    42  IE---------VCDYN----GPVT----VVVSCVTSDE--------PYRQHPHWLV--SKEEADS-----   79
_XP_002014799.1_    42  IE---------VCDYN----GPVT----VVVSCVTSDE--------PYRQHPHWLV--SKEEADS-----   79
_XP_026844902.1_    42  IE---------VCDYN----GPVT----VVVSCVTSDE--------PYRQHPHWLV--SKEEADS-----   79
_XP_015035983.2_    42  IE---------VCDYN----GPVT----VVVSCVTSDE--------PYRQHPHWLV--SKEEADS-----   79
_XP_017063896.1_    42  IE---------VCNYD----GPVI----IVVSCVTSDE--------PYRQHPHWLV--SKEEADA-----   79
_XP_016935332.1_    42  IE---------VCNYD----GPVI----IVVSCVTSDE--------PYRQHPHWLV--SKEEADA-----   79
_XP_017063894.2_    42  IE---------VCNYD----GPVI----IVVSCVTSDE--------PYRQHPHWLV--SKEEADA-----   79
_XP_036668637.1_    42  IE---------VCNYD----GPVI----IVVSCVTSDE--------PYRQHPHWLV--SKEEADA-----   79
_XP_016935310.1_    42  IE---------VCNYD----GPVI----IVVSCVTSDE--------PYRQHPHWLV--SKEEADA-----   79
_KAH8298318.1_      42  IE---------VCNYD----GPVI----VVVSCVTSDE--------PYRQHPHWLV--SKEEADA-----   79
_KAH8412187.1_      42  IE---------VVNCE----GPVI----VVVSCVTSEE--------PYRQHPHWLV--SKEEADA-----   79
_KAH8278042.1_      41  VE---------VCNYD----GPVI----IVVSCVTSDE--------PYRQHPHWLV--SKEEADA-----   78
_KAH8366423.1_      42  IE---------VCNYD----GPVV----IVVSCVTSDE--------PFRQHPHWLV--SKEEADA-----   79
_XP_017044860.1_    41  VE---------VCNFD----GQVT----MVVSCVTSEE--------PHRQHPHWLV--SKEEADA-----   78
_KAH8324526.1_      40  VE---------VCNYD----GPVI----ITVSCVTSDE--------PYRQHPHWLV--SKEEADA-----   77
_XP_017116817.1_    42  VE---------VCNYD----GPVI----IVVSCVTSDE--------PYRQHPHWLV--SKEEADA-----   79
_XP_041566118.1_    42  VE---------VCNYD----GPVI----IVVSCVTSDE--------PYRQHPHWLV--SKEEADA-----   79
_XP_052852584.1_    42  VE---------VCNYD----GPVI----IVVSCVTSEE--------PYRQHPHRLV--SKEEADA-----   79
_XP_052852582.1_    42  VE---------VCNYD----GPVI----IVVSCVTSEE--------PYRQHPHRLV--SKEEADA-----   79
_XP_017107132.2_    41  VE---------VCNYD----GPVT----IVVSCVTSDE--------PYRAHPHWLV--SKEEADA-----   78
_XP_043069650.1_    41  VE---------VCNYD----GPVT----IVVSCVTSDE--------PYRAHPHWLV--SKEEADA-----   78
_KAH8283158.1_      42  IE---------VCNYD----GPVV----IVVSCVTSDE--------PYRQHPHWLV--SKEEADA-----   79
_XP_017025640.1_    42  IE---------VCNYD----GP-V----IVVSCVTSDE--------PYRQHPHWLV--SKEEADA-----   78
_XP_020803071.1_    42  IE---------VCNYD----GPVV----IVVSCVTSDE--------PYRQHPHWLV--SKEEADA-----   79
_XP_017025638.1_    42  IE---------VCNYD----GPVV----IVVSCVTSDE--------PYRQHPHWLV--SKEEADA-----   79
_KAH8308067.1_      42  IE---------VCNYD----GPVV----IVVSCVTSDE--------PYRQHPHWLV--SKEEADA-----   79
_KAH8362606.1_      42  IE---------VCNYD----GPVV----IVVSCVTSDE--------PYRQHPHWLV--SKEEADA-----   79
_XP_020803068.1_    42  IE---------VCNYD----GPVV----IVVSCVTSDE--------PYRQHPHWLV--SKEEADA-----   79
_XP_015052364.1_    41  VE---------VLNYH----GKVT----IIVSCVTSNM--------PYRQHPHCLV--SKEEADA-----   78
_XP_002090469.1_    41  VE---------VLNYH----GKVT----IIVSCVTSNM--------PYRQHPHCLV--SKEEADA-----   78
_XP_039480354.1_    41  VE---------VLNYH----GKVI----IIVSCVTSEM--------PYRQHPHWLV--SKEEADA-----   78
_XP_039480352.1_    41  VE---------VLNYH----GKVI----IIVSCVTSEM--------PYRQHPHWLV--SKEEADA-----   78
```

```
_XP_041450795.1_    42  IE---------VCDYS----GPVT----VVVSCVTSDE--------PYRQHPHWLV--SKEEADS-----   79
_XP_044315461.1_    42  VE---------VCNYD----GPVT----IVVSCVTSEK--------PYRQHPHWLV--SKEEADA-----   79
_XP_044315463.1_    42  VE---------VCNYD----GPVT----IVVSCVTSEK--------PYRQHPHWLV--SKEEADA-----   79
_XP_001961710.2_    42  VE---------VCNYD----GPVV----VVVSCVTSDE--------PYRQHPHWLV--SKEEADA-----   79
_XP_014761890.1_    42  VE---------VCNYD----GPVV----VVVSCVTSDE--------PYRQHPHWLV--SKEEADA-----   79
_KAH8323309.1_      42  VE---------VCNYD----GPVV----VVVSCVTSDE--------PYRQHPHWLV--SKEEADA-----   79
_XP_043646206.1_    41  VE---------VLNYH----GKVI----IIVSCVTSDE--------PYRQHPHWLV--SKEEADA-----   78
_XP_043646204.1_    41  VE---------VLNYH----GKVI----IIVSCVTSDE--------PYRQHPHWLV--SKEEADA-----   78
_AAQ65066.1_        42  IE---------VCNYD----GPVI----IVVSCVTSDE--------PFRQHPHWLV--SKEEADA-----   79
_AAQ64900.1_        42  IE---------VCNYD----GPVI----IVVSCVTSDE--------PFRQHPHWLV--SKEEADA-----   79
_AAQ64905.1_        42  IE---------VCNYD----GPVI----IVVSCVTSDE--------PFRQHPHWLV--SKEEADA-----   79
_KAH8378209.1_      41  IE---------IVNYN----GPAH----VVVSCVTADP--------PYRQHPHELV--SKDEADK-----   78
_KAH8293207.1_      41  IE---------ILNYN----GPAH----VIVSCVTADE--------PHRQHPHELV--SKDEADN-----   78
_KAH8391978.1_      40  IE---------IVNYN----GPAH----VFVSCVTADP--------PYRQHPHELV--SKDEADK-----   77
_XP_034101352.1_    40  IE---------IVNYN----GPAH----VFVSCVTADP--------PYRQHPHELV--SKDEADK-----   77
_KAJ3658269.1_      39  VQ---------IHGYQ----GRLV----VIVSCVTKDP--------PYRQHPHQLV--GHNCK-------   74
_KAJ3658270.1_      39  IQ---------IHGYQ----GRLL----VNVSCVTKDP--------PYRQHPYQLV--GPNCN-------   74
_KAJ3658271.1_      39  IQ---------VHGYE----GRLR----VIVSCVTRDP--------PYRQHPYQLV--GRKCQ-------   74
_XP_015117085.1_    41  IK---------IVGLN----TDAH----LIISCVSKEA--------PYKVHPHRIV--SKGKGT------   77
_XP_011307643.1_    41  IK---------IVGLQ----TDAH----VIVSCVSKEA--------PYKVHPHRLI--SKGKEI------   77
_XP_011307741.1_    41  IR---------IVGPW----LNGR----VIVSCVTKDE---------YRAHPNRVV--SKEAGS------   76
_XP_015119445.1_    41  IK---------IIGLD----TDAY----VIVSCVTKEA--------PYKVHPHRLV--SKDKGS------   77
_XP_052128594.1_    36  IQ---------IANFD----RLAT----VIVSCVTKDQ--------PYRPHPHCLA--GDDCK-------   71
_XP_052128597.1_    36  IQ---------IANFD----RLAT----VIVSCVTKDQ--------PYRPHPHCLA--GDDCK-------   71
_KAE8741315.1_      36  IQ---------IANFD----RLAT----VIVSCVTKDQ--------PYRPHPHCLA--GDDSG-------   71
_XP_028982160.1_    41  IR---------VTGLR----AIAY----IVVSCVTKDE--------PYRIHPHSLV--SGEAMNNCQ---   80
_XP_011307645.1_    41  IR---------VTGLR----TIAY----VVVSCVTKDK--------PYRIHPHSLI--NGEPENNCQ---   80
_XP_054725384.1_    41  IE---------ILNYK----GRAV----VIISCVTEDA--------PYKPHPHTIL--NKDGYI------   77
_XP_053965385.1_    41  IE---------ILNYK----GRAV----VIISCVTEDA--------PYKPHPHTIL--NKEGYI------   77
_XP_013149158.1_    40  IE---------ISGCPE---GKAV----IVVSCVTKDS--------PYKTHPHGVV--ARELYMSDE---   80
_XP_045534655.1_    40  IE---------ISGCPE---GKAV----IVVSCVTKDS--------PYKTHPHGVV--ARELFMSEE---   80
_KPJ06852.1_        40  IE---------ISGCPE---GKAV----IVVSCVTKDS--------PYKTHPHGVV--ARELFMSEE---   80
_XP_013176418.1_    40  IE---------ISGCPD---GKAA----ILVSCVTKDS--------PYKTHPHGVV--AREMYLSEE---   80
_OXU28425.1_        33  IR---------IHNNR----GPVT----VIISCVTVDP--------PYKPHPCKVF--NGNAVD------   69
_XP_008216913.1_    32  IR---------IHNYR----GPVT----VIISCVTVDP--------PYKPHPCKLF--NGNTMD------   68
_XP_008216914.1_    32  IR---------IHNYR----GPVT----VIISCVTVDP--------PYKPHPCKLF--NGNTMD------   68
_XP_040572301.1_    41  IE---------IVGYK----GPMT----VVASCVEDHA--------PYRVHPNKLI--DRNNAS------   77
_CAB4068408.1_       9  IE---------IVGYK----GPMT----VVASCVEDHA--------PYRVHPNKLI--DRNNAS------   45
_KAF7993284.1_       1  -----------IVGYQ----GNVG----ILVTCVMKDQKN-----GKYSSHPHHVI--NVANVNE-----   39
```

```
_KAF7987489.1_       38  IK---------INNCNN---YKKG---VVLISCVNKEKIN-----GKYSVHPHKIV--SKDSKE------  79
_KAF7994520.1_       40  IK---------INNCDN---YKKG---TLIVTCVTAKKIN-----GKYLIHPHKIN--GKNSKK------  81
_XP_044019368.1_     39  II---------INNCNG---YQNG---VLYISCVSEKETG-----GKYHMHPHKIL--KKDSAE------  80
_XP_044019332.1_         ----------------------------------------------------------------------
_XP_044003986.1_         ----------------------------------------------------------------------
_XP_037042732.1_     40  IR---------IVNYT----GMAT----VFVSCVTVEK--------PYRQHPHKLL--GKNCE-------  75
_CAD7223243.1_       42  VK---------IVNYK----GPAL----VIVSLVCKDE--------PYRQHPHKLV--GRDGCR------  78
_XP_044732233.1_     38  IK---------IYNYT----GMVY----IRLSCVTKGPP------DEHRPHPHNLK--KLPVRNNAYADR  82
_XP_012231885.1_     39  IR---------IVGCK----GCAM----VLVSCVTKDA--------PYRPHPHNLI--NKKVCQ------  75
_XP_022814699.1      45  LM---------VNNVEK--HDKAL----VVISTVTANK--------PFRAHPNGLM--GRNCY-------  82
_CAB3516523.1_       45  LM---------VNNVEK--HDKAL----IVISTVTANE--------PFRAHPNGLM--GRNCF-------  82
_XP_035458245.2_     44  LM---------ISNVPV--QDKAL----ITVSTVTANE--------PFRAHPNGLM--GRDCH-------  81
_KAF9795795.1_       33  LM---------INNVPV--QDKAL----ITVSTVTANE--------PFRAHPNGLM--GRDCH-------  70
_XP_014233257.1_     36  II---------VHNYE----GPVL----LTISLVTSQE--------PHYPHPYLLQ--GGRGYKM-----  73
_XP_034324400.1_     24  IR---------FLNIY----TEVF----VRIHVIDPSN---------KKAHPYCLI--GDKCK-------  58
_XP_052699907.1_     24  IR---------FLNIY----TEVF----VRIHVIDPSN---------KKAHPYCLI--GDKCK-------  58
Consensus_aa:            Ip.........lhsh.....G.h.....l.lohVTps.........P@+sHPHpLl..tcps........
Consensus_ss:            ee         ee          ee     eeeeeee                   eeee  eee


Conservation:                6                                  5              6
_XP_001652840.2_     78  ----KGVCTVEIN--------------SSTMSYTFSNLGIQCVKKKDIEEALRLREEIRV-DPFKT----  124
_AAW67214.1_         78  ----KGVCTVEIN--------------SSTMSYTFSNLGIQCVKKKDIEEALRLREEIRV-DPFKT----  124
_XP_021704027.1_     78  ----KGVCTVEIN--------------SSTMSYTFSNLGIQCVKKKDIEEALRLREEIRV-DPFKT----  124
_XP_019540833.2_     78  ----KGVCTVEIN--------------SSTMSYTFSNLGIQCVKKKDIEEALRLREEIRV-DPFKT----  124
_XP_029720231.1_     78  ----KGVCTVEIN--------------SSTMSYTFSNLGIQCVKKKDIEEALRLREEIRV-DPFKT----  124
_AAW67215.1_         78  ----KGVCTVEIN--------------SSTMSYTFSNLGIQCVKKKDIEEALRLREEIRV-DPFKT----  124
_XP_021704025.1_     78  ----KGVCTVEIN--------------SSTMSYTFSNLGIQCVKKKDIEEALRLREEIRV-DPFKT----  124
_XP_019540831.2_     78  ----KGVCTVEIN--------------SSTMSYTFSNLGIQCVKKKDIEEALRLREEIRV-DPFKT----  124
_KXJ81105.1_         78  ----KGVCTVEIN--------------SSTMSYTFSNLGIQCVKKKDIEEALRLREEIRV-DPFKT----  124
_KXJ76497.1_         78  ----KGVCTVEIN--------------SSTMSYTFSNLGIQCVKKKDIEEALRLREEIRV-DPFKT----  124
_XP_023240428.1_     78  ----QGVCTVIIN--------------NSDMTYSFTSLGIQCVKKKNIEESLKLRENIKV-DPFLT----  124
_CAD7241875.1_       79  ----KGICTMPVD--------------PNTMRLVFQSLGVQCAKKRDIDQELKLREEIRV-DPFQT----  125
_TRY80291.1_         78  ---KQGVCSVEIT--------------KPDMTYSFQNLGIQCVKRKDAQQSLERRQKIRV-DPFKQ----  125
_ALF84899.1_         77  ----KGVCSVEVN--------------QVDMTCVFQNLGIQCVKRKDAPDSLTQRQEIRV-DPYKQ----  123
_KAF4528208.1_       77  ----RGVCTLEIN--------------PDNMTCVFSNLGIQCVKKKDIEEALKVREEIRV-DPYRT----  123
_XP_054260955.1_     78  ----RGVCTLEIH--------------NDTMTQSFDHLGIQCVKKKDIEDALRIREEIRV-DPFKT----  124
_XP_054264401.1_     78  ----RGVCTLEIH--------------NDTMTQSFDHLGIQCVKKKDIEDALRIREEIRV-DPFKT----  124
_XP_054260954.1_     78  ----RGVCTLEIH--------------NDTMTQSFDHLGIQCVKKKDIEDALRIREEIRV-DPFKT----  124
```

_XP_038114042.1_	78	----KGVCTVEIN--------------SASMSYTFSNLGIQCVKKKDIEEALRLREEIRV-DPFKT----	124
_XP_039437558.1_	78	----KGVCTVEIN--------------SASMSYTFSNLGIQCVKKKDIEEALRLREEIRV-DPFKT----	124
_XP_001844078.2_	78	----KGVCTVEIN--------------SASMSYTFSNLGIQCVKKKDIEEALRLREEIRV-DPFKT----	124
EDS35747.1	78	----KGVCTVEIN--------------SASMSYTFSNLGIQCVKKKDIEEALRLREEIRV-DPFKT----	124
_XP_053696925.1_	78	----KGVCTVEIN--------------SSSMTYTFSNLGIQCVKKKDIEDALRLREDIRV-DPFKT----	124
_XP_001869619.2_	78	----EGVCTVVLN--------------SATMSYTFNNLGIQCVKKKDIEGALRTREKLRV-DPYKT----	124
_XP_039434954.1_	78	----EGVCTVVLN--------------SATMSYTFNNLGIQCVKKKDIEGALKTREKLRV-DPYKT----	124
EDS29879.1	78	----EGVCTVVLN--------------SATMSYTFNNLGIQCVKKKDIEGALRTREKLRV-DPYKT----	124
_XP_019550742.2_	78	----KGVCTVEIN--------------NSSMSYTFSNLGIQCVKKKDIKDALQLREEIRV-DPFKT----	124
KXJ74587.1	78	----KGVCTVEIN--------------NSSMSYTFSNLGIQCVKKKDIKDALQLREEIRV-DPFKT----	124
_XP_035908773.1_	79	----KGVCTVEIN--------------STTMSYTFNNLGIQCVKKKDVEEALRLRQEIRV-DPYRT----	125
AJC98655.1	79	----KGVCTVEIN--------------STTMSYTFNNLGIQCVKKKDVEEALRLRQEIRV-DPFRT----	125
AJC98650.1	79	----KGVCTVEIN--------------STTMSYTFNNLGIQCVKKKDVEEALRLRQEIRV-DPFRT----	125
AJC98644.1	79	----KGVCTVEIN--------------STTMSYTFNNLGIQCVKKKDVEEALRLRQEIRV-DPFRT----	125
AJC98657.1	79	----KGVCTVEIN--------------STTMSYTFNNLGIQCVKKKDVEEALRLRQEIRV-DPFRT----	125
AJC98648.1	79	----KGVCTVEIN--------------STTMSYTFNNLGIQCVKKKDVEEALRLRQEIRV-DPFRT----	125
AJC98647.1	79	----KGVCTVEIN--------------STTMSYTFNNLGIQCVKKKDVEEALRLRQEIRV-DPFRT----	125
AJC98651.1	79	----KGVCTVEIN--------------STTMSYTFNNLGIQCVKKKDVEEALRLRQEIRV-DPFRT----	125
AJC98643.1	79	----KGVCTVEIN--------------STTMSYTFNNLGIQCVKKKDVEEALRLRQEIRV-DPFRT----	125
AJC98656.1	79	----KGVCTVEIN--------------STTMSYTFNNLGIQCVKKKDVEEALRLRQEIRV-DPFRT----	125
AJC98641.1	79	----KGVCTVEIN--------------STTMSYTFNNLGIQCVKKKDVEEALRLRQEIRV-DPFRT----	125
AJC98640.1	79	----KGVCTVEIN--------------STTMSYTFNNLGIQCVKKKDVEEALRLRQEIRV-DPFRT----	125
AJC98653.1	79	----KGVCTVEIN--------------STTMSYTFNNLGIQCVKKKDVEEALRLRQEIRV-DPFRT----	125
AJC98654.1	79	----KGVCTVEIN--------------STTMSYTFNNLGIQCVKKKDVEEALRLRQEIRV-DPFRT----	125
AJC98652.1	79	----KGVCTVEIN--------------STTMSYTFNNLGIQCVKKKDVEEALRLRQEIRV-DPFRT----	125
AJC98646.1	79	----KGVCTVEIN--------------STTMSYTFNNLGIQCVKKKDVEEALRLRQEIRV-DPFRT----	125
AJC98658.1	79	----KGVCTVEIN--------------STTMSYTFNNLGIQCVKKKDVEEALRLRQEIRV-DPFRT----	125
AJC98639.1	79	----KGVCTVEIN--------------STTMSYTFNNLGIQCVKKKDVEEALRLRQEIRV-DPFRT----	125
_XP_035908772.1_	79	----KGVCTVEIN--------------STTMSYTFNNLGIQCVKKKDVEEALRLRQEIRV-DPYRT----	125
_XP_050076803.1_	79	----KGVCTVEIN--------------STTMSYTFNNLGIQCVKKKDVEEALRLRQEIRV-DPYRT----	125
_XP_049282441.1_	79	----KGVCTVEIN--------------STTMSYTFNNLGIQCVKKKDVEEALRLRQEIRV-DPYRT----	125
_XP_040170080.1_	79	----KGVCTVEIN--------------STTMSYTFNNLGIQCVKKKDVEEALRLRQEIRV-DPFRT----	125
_XP_050076802.1_	79	----KGVCTVEIN--------------STTMSYTFNNLGIQCVKKKDVEEALRLRQEIRV-DPYRT----	125
_XP_041780138.1_	79	----KGVCTVEIN--------------STTMSYTFNNLGIQCVKKKDVEEALRLRQEIRV-DPFRT----	125
_XP_040239229.2_	79	----KGVCTVEIN--------------STTMSYTFNNLGIQCVKKKDVEEALRLRQEIRV-DPFRT----	125
_XP_040239222.2_	79	----KGVCTVEIN--------------STTMSYTFNNLGIQCVKKKDVEEALRLRQEIRV-DPFRT----	125
_XP_035908780.1_	79	----KGVCTVEIN--------------STTMSYTFNNLGIQCVKKKDVEEALRLRQEIRV-DPYRT----	125
_XP_040239221.2_	79	----KGVCTVEIN--------------STTMSYTFNNLGIQCVKKKDVEEALRLRQEIRV-DPFRT----	125
_XP_052897786.1_	79	----KGVCTVEIN--------------STTMSYTFNNLGIQCVKKKDVEEALRLRQEIRV-DPYRT----	125

```
_XP_052895493.1_    79  ----KGVCTVEIN--------------STTMSYTFNNLGIQCVKKKDVEEALRLRQEIRV-DPYRT----  125
_XP_053664395.1_    79  ----KGVCTVEIN--------------STTMSYTFNNLGIQCVKKKDVEEALRLRQEIRV-DPYRT----  125
_XP_050076801.1_    79  ----KGVCTVEIN--------------STTMSYTFNNLGIQCVKKKDVEEALRLRQEIRV-DPYRT----  125
_XP_310177.3_       79  ----KGVCTVEIN--------------STTMSYTFNNLGIQCVKKKDVEEALRLRQEIRV-DPFRT----  125
_XP_001652339.1_    78  ----KGVCTVEIN--------------KSVMSYTFCSLGIQCVKKKDIKDALRLREEIRV-DPFKT----  124
_XP_053686256.1_    78  ----EGVCTVKLD--------------CNSMSYVFSNLGIQCVKKKGIEESLGLRERIQV-DPFHT----  124
_XP_053675540.1_    79  ----KGVCTVEIN--------------STTMSYTFNNLGIQCVKKKDVEEALRLRQEIRV-DPYRT----  125
_ETN66814.1_        78  ----KGVCTVEMN--------------STTMSYSFNNLGIQCVKKKEVAEALKQRLEIRV-DPFRT----  124
_XP_049534501.1_    78  ----KGVCTVEMN--------------STTMSYSFNNLGIQCVKKKEVAEALKQRLEIRV-DPFRT----  124
_XP_050084431.1_    78  ----KGVCTVEMN--------------STTMSYSFNNLGIQCVKKKEVAEALKQRLEIRV-DPFRT----  124
_XP_035782874.1_    78  ----KGVCTVEMN--------------STTMSYSFNNLGIQCVKKKEVADALKQRLDIRV-DPFRT----  124
_XP_035782869.1_    78  ----KGVCTVEMN--------------STTMSYSFNNLGIQCVKKKEVADALKQRLDIRV-DPFRT----  124
_KFB39849.1_        86  ----KGVCTVEIN--------------CNTMAYQFNNLGIQCVKKKDVEEALRLRQEIRV-DPFRT----  132
_XP_052871375.1_    79  ----KGVCTVEIN--------------STTMSYNFNNLGIQCVRKKDVDDALKLRQEIRV-DPFRT----  125
_AAR13743.1_        64  ----KGVCTVEIN--------------STTMSYTFNNLGIQCVKKKDVEEALRLRQEIRV-DPFRT----  110
_AAR13744.1_        64  ----KGVCTVEIN--------------STTMSYTFNNLGIQCVKKKDVEEALRLRQEIRV-DPFRT----  110
_CAA65157.1_        58  ----KGVCTVEIN--------------STTMSYTFNNLGIQCVKKKDVEEALRLRQEIRV-DPFRT----  104
_XP_022170156.1_    78  ----RGICTIEIN--------------NESMTASFQNLGIQCVKRKDIDEALKVREEIRV-DPFRT----  124
_XP_025205168.1_    78  ----RGICTIEIN--------------NESMTASFQNLGIQCVKRKDIDEALKVREEIRV-DPFRT----  124
_XP_026817162.1_    78  ----RGICTIEIN--------------NESMTASFQNLGIQCVKRKDIDEALKVREEIRV-DPFRT----  124
_XP_027846205.1_    78  ----RGICTIEIN--------------NESMTASFQNLGIQCVKRKDIDEALKVREEIRV-DPFRT----  124
_XP_015370635.1_    78  ----RGICTIEIN--------------NESMTASFQNLGIQCVKRKDIDEALKVREEIRV-DPFRT----  124
_CAH1723797.1_      78  ----RGICTIEIN--------------NESMTASFQNLGIQCVKRKDIDEALKVREEIRV-DPFRT----  124
_KAF0769940.1_      78  ----RGICTIEIN--------------NESMTASFQNLGIQCVKRKDIDEALKVREEIRV-DPFRT----  124
_CAB4065202.1_          ----------------------------------------------------------------------
_XP_050440903.1_    78  ----RGICTIEIN--------------NESMTASFQNLGIQCVKRKDIDEHLKIREDIRV-DPFRT----  124
_XP_050533799.1_    78  ----RGICTIEIN--------------NESMTASFQNLGIQCVKRKDIDDALKVREDIRV-DPFRT----  124
_XP_025410632.1_    78  ----KGICTIEIN--------------NESMTASFQNLGIQCVKRKDIDDALKVREKIRV-DPFRT----  124
_XP_008180746.1_    78  ----KGICTIEIN--------------NESMAASFQNLGIQCVKRKDIDEALRVREEIRV-DPFRT----  124
_XP_026465450.1_    78  ----KGVCTAKIE-------------EANDMTITFNNLGVQCVKKKDIKQSLKLREDINV-DPFRT----  125
_VVC36026.1_        78  ----KGICTIEIN--------------NDSMTASFQNLGIQCVKRKDIDEALKVREEIRV-DPFRT----  124
_XP_016661522.1_    78  ----NGICSIEIN--------------NESMTESFQNLGIQCVKRKDIDKALRVREERKL-DPFKT----  124
_XP_001947429.3_    78  ----NGICSIEIN--------------NESMTESFQNLGIQCVKRKDIDKALRVREERKL-DPFKT----  124
_XP_026466007.1_    78  ----KGVCTAKIE-------------EANDMTITFNNLGVQCVKKKDIKQSLKLREDINV-DPFRT----  125
_KRT81254.1_        75  ----SGVCYVKVP--------------EDTMTVSFQNLGIRCVTKKDMANALKIRKSINV-DPFKR----  121
_XP_033224832.1_    81  ----HGFCKVKLK--------------GDNMTVPFQNLGIQCVKRKDVEKSLSIRKTFGV-DPFNT----  127
_XP_025407590.1_    76  ----RGIFKAEIN--------------NESMTLSLQNVGIQCVKRSNVDEALKERESQRI-DPFKT----  122
_XP_045476729.1_    76  ----SGVYTRTIP--------------EGFEKIEFSNIGIKCVRKKDVGDSLKERRKINV-DPFN-----  121
_XP_034174253.1_    78  ----RGVCTVEVS--------------SENMTVTFANLGIQCVKKKDIEEALRIREEIRV-DPFRT----  124
```

```
_XP_029047866.1_   78  ----RGVCTVEVS--------------SENMTVTFANLGIQCVKKKDIEEALRIREEIRV-DPFRT----  124
_XP_015430648.1_   78  ----RGVCTVEVS--------------SENMTVTFANLGIQCVKKKDIEEALRVREEIRV-DPFRT----  124
_XP_017798081.1_   78  ----KGVCTIEVS--------------SENMTVTFANLGIQCVKKKDIEDALRIREDIRV-DPFRT----  124
_XP_017756471.1_   78  ----RGVCTVEVS--------------SENMTVTFANLGIQCVKKKDIEEALRIREEIRV-DPFRT----  124
_XP_043248447.1_   78  ----RGVCTVEVS--------------SENMTVTFANLGIQCVKKKDIEEALRIREEIRV-DPFRT----  124
_XP_029047855.1_   78  ----RGVCTVEVS--------------SENMTVTFANLGIQCVKKKDIEEALRIREEIRV-DPFRT----  124
_XP_034174252.1_   78  ----RGVCTVEVS--------------SENMTVTFANLGIQCVKKKDIEEALRIREEIRV-DPFRT----  124
_XP_031829337.1_   78  ----RGVCTVEVS--------------SENMTVTFANLGIQCVKKKDIEEALRIREEIRV-DPFRT----  124
_KZC09125.1_       78  ----RGVCTVEVS--------------SENMTVTFANLGIQCVKKKDIEEALRVREEIRV-DPFRT----  124
_XP_033327213.1_   78  ----RGVCTVEVS--------------SENMTVTFANLGIQCVKKKDIEDALRIREEIRV-DPFRT----  124
_XP_033327212.1_   78  ----RGVCTVEVS--------------SENMTVTFANLGIQCVKKKDIEDALRIREEIRV-DPFRT----  124
_XP_017756445.1_   78  ----RGVCTVEVS--------------SENMTVTFANLGIQCVKKKDIEEALRIREEIRV-DPFRT----  124
_OAD62393.1_       78  ----RGVCTVEVS--------------SENMTVTFANLGIQCVKKKDIEEALRIREEIRV-DPFRT----  124
_XP_043248413.1_   78  ----RGVCTVEVS--------------SENMTVTFANLGIQCVKKKDIEEALRIREEIRV-DPFRT----  124
_XP_033327208.1_   78  ----RGVCTVEVS--------------SENMTVTFANLGIQCVKKKDIEDALRIREEIRV-DPFRT----  124
_XP_034950477.1_   78  ----KGVCTVEIS--------------SENMTVTFANLGIQCVKKKDIEEALRIREDIRV-DPFRT----  124
_XP_034950475.1_   78  ----KGVCTVEIS--------------SENMTVTFANLGIQCVKKKDIEEALRIREDIRV-DPFRT----  124
_XP_012343274.1_   78  ----QGVCTVEVS--------------SENMTVTFANLGIQCVKKKDIEEALKIREEIRV-DPFRT----  124
_XP_016909449.1_   78  ----QGVCTVEVS--------------SENMTVTFANLGIQCVKKKDIEEALKIREEIRV-DPFRT----  124
_XP_012343273.1_   78  ----QGVCTVEVS--------------SENMTVTFANLGIQCVKKKDIEEALKIREEIRV-DPFRT----  124
_XP_006619742.1_   78  ----QGVCTVEIS--------------SENMTVTFANLGIQCVKKKDIEEALKIREEIRV-DPFRT----  124
_XP_006567065.1_   78  ----QGVCTVEVS--------------SENMTVTFANLGIQCVKKKDIEEALKIREEIRV-DPFRT----  124
_XP_006619741.1_   78  ----QGVCTVEIS--------------SENMTVTFANLGIQCVKKKDIEEALKIREEIRV-DPFRT----  124
_NP_001011577.1_   78  ----QGVCTVEVS--------------SENMTVTFANLGIQCVKKKDIEEALKIREEIRV-DPFRT----  124
_XP_016909448.1_   78  ----QGVCTVEVS--------------SENMTVTFANLGIQCVKKKDIEEALKIREEIRV-DPFRT----  124
_XP_043802365.1_   78  ----QGVCTVEVS--------------SENMTVTFANLGIQCVKKKDIEEALKIREEIRV-DPFRT----  124
_NP_001164477.1_   78  ----QGVCTVEVS--------------SENMTVTFANLGIQCVKKKDIEEALKIREEIRV-DPFRT----  124
_XP_016909443.1_   78  ----QGVCTVEVS--------------SENMTVTFANLGIQCVKKKDIEEALKIREEIRV-DPFRT----  124
_XP_043802361.1_   78  ----QGVCTVEVS--------------SENMTVTFANLGIQCVKKKDIEEALKIREEIRV-DPFRT----  124
_XP_012343269.1_   78  ----QGVCTVEVS--------------SENMTVTFANLGIQCVKKKDIEEALKIREEIRV-DPFRT----  124
_XP_006619738.1_   78  ----QGVCTVEIS--------------SENMTVTFANLGIQCVKKKDIEEALKIREEIRV-DPFRT----  124
_PBC29878.1_       78  ----QGVCTVEVS--------------SENMTVTFANLGIQCVKKKDIEEALKIREEIRV-DPFRT----  124
_XP_046663984.1_   78  ----KGVCTVEVH--------------CESMTQSFDHLGIQCVKKKDIEEALKAREEIRV-DPFRT----  124
_XP_046663985.1_   78  ----KGVCTVEVH--------------CESMTQSFDHLGIQCVKKKDIEEALKAREEIRV-DPFRT----  124
_XP_043274771.1_   78  ----KGVCTVEVA--------------TENMTVTFANLGIQCVKKKDIEDALRVREEMRV-DPFRT----  124
_XP_043274769.1_   78  ----KGVCTVEVA--------------TENMTVTFANLGIQCVKKKDIEDALRVREEMRV-DPFRT----  124
_XP_043289571.1_   78  ----TGVCRMEIT--------------PGHPIVSFVNLGIQCVKKKDIAEALSVREEIRV-DPFRT----  124
_XP_032665873.1_   78  ----QGVCTVEVP--------------AGNMIVTFSNLGIQCVKKKDIEEALKVREELRV-DPFRT----  124
_XP_032665875.1_   78  ----QGVCTVEVP--------------AGNMIVTFSNLGIQCVKKKDIEEALKVREELRV-DPFRT----  124
```

```
_XP_032665874.1_   78  ----QGVCTVEVP---------------AGNMIVTFSNLGIQCVKKKDIEEALKVREELRV-DPFRT----  124
_XP_032665869.1_   78  ----QGVCTVEVP---------------AGNMIVTFSNLGIQCVKKKDIEEALKVREELRV-DPFRT----  124
_XP_014470289.1_   78  ----QGVCTVEVP---------------AGNMIVTFSNLGIQCVKKKDIEEALKVREELRV-DPFRT----  124
_XP_011149046.1_   78  ----QGVCTVEVP---------------AGNMIVTFSNLGIQCVKKKDIEEALKVREDLRV-DPFRT----  124
_XP_017877914.1_   77  ----KGVCTVEVS---------------PENMTVIFANLGIQCVKKKDIEEALRIREEIRV-DPFRT----  123
_XP_017877911.1_   77  ----KGVCTVEVS---------------PENMTVIFANLGIQCVKKKDIEEALRIREEIRV-DPFRT----  123
_XP_033174774.1_   78  ----RGVCTVEVS---------------SENMTVTFANLGIQCVKKKDIEEALKIREEIRV-DPFRT----  124
_XP_043581013.1_   78  ----RGVCTVEVS---------------SENMTVTFANLGIQCVKKKDIEEALKIREEIRV-DPFRT----  124
_XP_048261507.1_   78  ----RGVCTVEVS---------------SENMTVTFANLGIQCVKKKDIEEALKIREEIRV-DPFRT----  124
_XP_050573946.1_   78  ----RGVCTVEVS---------------SENMTVTFANLGIQCVKKKDIEEALKIREEIRV-DPFRT----  124
_XP_050478075.1_   78  ----RGVCTVEVS---------------SENMTVTFANLGIQCVKKKDIEEALKIREEIRV-DPFRT----  124
_XP_033204249.1_   78  ----RGVCTVEVS---------------SENMTVTFANLGIQCVKKKDIEEALKIREEIRV-DPFRT----  124
_XP_033358451.1_   78  ----RGVCTVEVS---------------SENMTVTFANLGIQCVKKKDIEEALKIREEIRV-DPFRT----  124
_XP_033358450.1_   78  ----RGVCTVEVS---------------SENMTVTFANLGIQCVKKKDIEEALKIREEIRV-DPFRT----  124
_XP_033204244.1_   78  ----RGVCTVEVS---------------SENMTVTFANLGIQCVKKKDIEEALKIREEIRV-DPFRT----  124
_XP_033312956.1_   78  ----RGVCTVEVS---------------SENMTVTFANLGIQCVKKKDIEEALKIREEIRV-DPFRT----  124
_XP_012236639.1_   78  ----RGVCTVEVS---------------SENMTVTFANLGIQCVKKKDIEEALKIREEIRV-DPFRT----  124
_XP_050573940.1_   78  ----RGVCTVEVS---------------SENMTVTFANLGIQCVKKKDIEEALKIREEIRV-DPFRT----  124
_XP_012174118.1_   78  ----RGVCTVEVS---------------SENMTVTFANLGIQCVKKKDIEEALKIREEIRV-DPFRT----  124
_XP_043581010.1_   78  ----RGVCTVEVS---------------SENMTVTFANLGIQCVKKKDIEEALKIREEIRV-DPFRT----  124
_EZA51108.1_       78  ----QGVCTVEVP---------------VGNMVVSFSNLGIQCVKKKDIEDALRVRQELRV-DPFRT----  124
_XP_011344085.1_   78  ----QGVCTVEVP---------------VGNMVVSFSNLGIQCVKKKDIEDALRVRQELRV-DPFRT----  124
_XP_043519475.1_   78  ----RGVCTVEVS---------------PENMTVTFANLGIQCVKKKDIEEALKVREEIRV-DPFRT----  124
_CAD1481114.1_     78  ----RGVCTVEVS---------------PENMTVTFANLGIQCVKKKDIEEALKIREEIRV-DPFRT----  124
_XP_043519473.1_   78  ----RGVCTVEVS---------------PENMTVTFANLGIQCVKKKDIEEALKVREEIRV-DPFRT----  124
_KOX75360.1_       78  ----RGVCTVEVS---------------PENMTVTFANLGIQCVKKKDIEEALKVREEIRV-DPFRT----  124
_KAF3423741.1_     78  ----RGVCTVEVS---------------PENMTVTFANLGIQCVKKKDIEEALKVREEIRV-DPFRT----  124
_KOC69050.1_       78  ----KGVCTIEVS---------------SENMTVTFANLGIQCVKKKDIEDALRIREDIRV-DPFRT----  124
_XP_053980095.1_   78  ----RGVCTVEVS---------------SENMTVTFANLGIQCVKKKDIEEALRIREEIRV-DPFRT----  124
_XP_054002893.1_   78  ----RGVCTVEVS---------------SENMTVTFANLGIQCVKKKDIEEALRIREEIRV-DPFRT----  124
_XP_053980091.1_   78  ----RGVCTVEVS---------------SENMTVTFANLGIQCVKKKDIEEALRIREEIRV-DPFRT----  124
_XP_028049144.1_   78  ----QGVCTVEVP---------------SGNMVVTFSNLGIQCVKKKDIEEALRVRQELRV-DPYRT----  124
_XP_012541458.1_   78  ----QGVCTVEVP---------------SGNMVVTFSNLGIQCVKKKDIEEALRVRQELRV-DPYRT----  124
_XP_003708412.1_   78  ----QGVCTVEVS---------------SKTMTVTFANLGIQCVKRKDIEEALRIREAIRV-DPFRT----  124
_XP_012152807.1_   78  ----QGVCTVEVS---------------SKTMTVTFANLGIQCVKRKDIEEALRIREAIRV-DPFRT----  124
_KAH0945556.1_     78  ----QGVCTVEVP---------------AGNMVATFSNLGIQCVKKKDIEEALRVRQELRV-DPFRT----  124
_XP_024871832.1_   78  ----QGVCTIEVP---------------AGNMVISFSNLGIQCVKKKDIEEALRVRQDLRV-DPFRT----  124
_XP_011307641.1_   77  ----KGVCTIEVP---------------VETMTVSFANLGIQCVKKRDIEEALNVREKIQV-DPFRT----  123
_XP_015118260.1_   77  ----KGVCTIEVP---------------VETMTVSFANLGIQCVKKRDIEEALNVREKIQV-DPFRT----  123
```

_XP_011307640.1_	77	----KGVCTIEVP--------------VETMTVSFANLGIQCVKKRDIEEALNVREKIQV-DPFRT----	123
_XP_015118252.1_	77	----KGVCTIEVP--------------VETMTVSFANLGIQCVKKRDIEEALNVREKIQV-DPFRT----	123
EFN68841.1	82	----QGVCKVEVP--------------AGNMVVTFSNLGIQCVKKKDIEEALRVRQELRV-DPFRT----	128
_XP_011647340.1_	78	----QGVCTVEVP--------------SGNMVVTFSNLGIQCVKKKDIEEALRMRQELRV-DPYRT----	124
_XP_029166122.1_	82	----KGVCTVEVP--------------AGNMVVTFSNLGIQCVKKKDIEEALRVRQELRV-DPFRT----	128
KMQ96453.1	82	----KGVCTVEVP--------------AGNMIVTFSNLGIQCVKKKDIEEALRVRQELRV-DPFRT----	128
_XP_050464492.1_	82	----QGVCTVEVP--------------AGNMVVTFSNLGIQCVKKKDIEEALRVRQELRV-DPFRT----	128
_XP_029673315.1_	82	----QGVCTIEVP--------------AGNMVVTFSNLGIQCVKKKDIEEALRVRQELRV-DPFRT----	128
_XP_025265518.1_	82	----QGVCKVEVP--------------AGNMVVTFSNLGIQCVKKKDIEEALRVRQELRV-DPFRT----	128
_XP_011255523.1_	82	----QGVCKVEVP--------------AGNMVVTFSNLGIQCVKKKDIEEALRVRQELRV-DPFRT----	128
_XP_011698133.1_	78	----QGVCTVEIP--------------SGNMVVTFSNLGIQCVKKKDIEEALRVRQELRV-DPFRT----	124
_XP_011698131.1_	78	----QGVCTVEIP--------------SGNMVVTFSNLGIQCVKKKDIEEALRVRQELRV-DPFRT----	124
_XP_018357420.1_	78	----QGVCTVEIP--------------SGNMVVTFSNLGIQCVKKKDIEEALRMRQELRV-DPFRT----	124
KYM76140.1	78	----QGVCTVEIP--------------SGNMVVTFSNLGIQCVKKKDIEEALRMRQELRV-DPFRT----	124
_XP_011647339.1_	78	----QGVCTVEVP--------------SGNMVVTFSNLGIQCVKKKDIEEALRMRQELRV-DPYRT----	124
KAG5315434.1	78	----QGVCTVEIP--------------SGNMVVTFSNLGIQCVKKKDIEEALRMRQELRV-DPFRT----	124
_XP_011055463.1_	78	----QGVCTVEIP--------------SGNMVVTFSNLGIQCVKKKDIEEALRMRQELRV-DPFRT----	124
_XP_018342550.1_	78	----QGVCTVEIP--------------SGNMVVTFSNLGIQCVKKKDIEEALRMRQELRV-DPFRT----	124
_XP_018403565.1_	78	----QGVCTVEIP--------------SGNMVVTFSNLGIQCVKKKDIEEALKMRQELRV-DPFRT----	124
KAG5322912.1	78	----QGVCTVEIP--------------SGNMVVTFSNLGIQCVKKKDIEEALKTRQELRV-DPFRT----	124
_XP_018357417.1_	78	----QGVCTVEIP--------------SGNMVVTFSNLGIQCVKKKDIEEALRMRQELRV-DPFRT----	124
_XP_018314560.1_	78	----QGVCTVEIP--------------SGNMVVTFSNLGIQCVKKKDIEEALRMRQELRV-DPFRT----	124
_XP_018057217.1_	78	----QGVCTVEIP--------------SGNMVVTFSNLGIQCVKKKDIEEALRMRQELRV-DPFRT----	124
KAG5346558.1	78	----QGVCTVEIP--------------SGNMVVTFSNLGIQCVKKKDIEEALKTRQELRV-DPFRT----	124
_XP_047348408.1_	78	----RGVCTLEIS--------------SENMTVTFANLGIQCVKKKDIEEALKIREEIRV-DPFRT----	124
_XP_047348412.1_	78	----RGVCTLEIS--------------SENMTVTFANLGIQCVKKKDIEEALKIREEIRV-DPFRT----	124
_XP_046820611.1_	78	----RGVCTLEIS--------------SENMTVTFANLGIQCVKKKDIEEALKIREEIRV-DPFRT----	124
_XP_050848254.1_	78	----RGVCTLEIS--------------SENMTVTFANLGIQCVKKKDIEEALKIREEIRV-DPFRT----	124
_XP_046820612.1_	78	----RGVCTLEIS--------------SENMTVTFANLGIQCVKKKDIEEALKIREEIRV-DPFRT----	124
_XP_050848252.1_	78	----RGVCTLEIS--------------SENMTVTFANLGIQCVKKKDIEEALKIREEIRV-DPFRT----	124
_XP_043667564.1_	78	----RGVCTLEIS--------------SENMTVTFANLGIQCVKKKDIEEALKIREEIRV-DPFRT----	124
_XP_035725337.1_	78	----RGVCTLEIS--------------SENMTVTFANLGIQCVKKKDIEEALKIREEIRV-DPFRT----	124
_XP_043667562.1_	78	----RGVCTLEIS--------------SENMTVTFANLGIQCVKKKDIEEALKIREEIRV-DPFRT----	124
_XP_046820605.1_	78	----RGVCTLEIS--------------SENMTVTFANLGIQCVKKKDIEEALKIREEIRV-DPFRT----	124
_XP_050848249.1_	78	----RGVCTLEIS--------------SENMTVTFANLGIQCVKKKDIEEALKIREEIRV-DPFRT----	124
_XP_043667560.1_	78	----RGVCTLEIS--------------SENMTVTFANLGIQCVKKKDIEEALKIREEIRV-DPFRT----	124
_XP_035725333.1_	78	----RGVCTLEIS--------------SENMTVTFANLGIQCVKKKDIEEALKIREEIRV-DPFRT----	124
KAF7405659.1	78	----RGVCTLEIS--------------SENMTVTFANLGIQCVKKKDIEEALKIREEIRV-DPFRT----	124
KAF7402294.1	78	----RGVCTLEIS--------------SENMTVTFANLGIQCVKKKDIEEALKIREEIRV-DPFRT----	124

```
_XP_015172825.1_    78  ----QGICTLEIT--------------SDNMTVTFANLGIQCVKKKDIEEALKIREEIRV-DPFRT----  124
_XP_015172824.1_    78  ----QGICTLEIT--------------SDNMTVTFANLGIQCVKKKDIEEALKIREEIRV-DPFRT----  124
_XP_015172823.1_    78  ----QGICTLEIT--------------SDNMTVTFANLGIQCVKKKDIEEALKIREEIRV-DPFRT----  124
_XP_014609306.1_    78  ----QGICTLEIT--------------SDNMTVTFANLGIQCVKKKDIEEALKIREEIRV-DPFRT----  124
_XP_043491219.1_    78  ----QGICTLEIT--------------SDNMTVTFANLGIQCVKKKDIEEALKIREEIRV-DPFRT----  124
_XP_014609305.1_    78  ----QGICTLEIT--------------SDNMTVTFANLGIQCVKKKDIEEALKIREEIRV-DPFRT----  124
_XP_043491218.1_    78  ----QGICTLEIT--------------SDNMTVTFANLGIQCVKKKDIEEALKIREEIRV-DPFRT----  124
_KAI4498556.1_      78  ----QGICTLEIT--------------SDNMTVTFANLGIQCVKKKDIEEALRIREEIRV-DPFRT----  124
_XP_015172818.1_    78  ----QGICTLEIT--------------SDNMTVTFANLGIQCVKKKDIEEALKIREEIRV-DPFRT----  124
_XP_043491211.1_    78  ----QGICTLEIT--------------SDNMTVTFANLGIQCVKKKDIEEALKIREEIRV-DPFRT----  124
_KAI4495142.1_      78  ----QGICTLEIT--------------SDNMTVTFANLGIQCVKKKDIEEALKIREEIRV-DPFRT----  124
_XP_014609300.1_    78  ----QGICTLEIT--------------SDNMTVTFANLGIQCVKKKDIEEALKIREEIRV-DPFRT----  124
_ADK91833.1_        78  ----RGVCTVEVS--------------SENMTVTFANLGIQCVKKKDIEEALKIREEIRV-DPFRT----  124
_KAG7213837.1_      78  ----QGICTVGIS--------------SENMMVTFANLGIQCVKKKDIEDALRVREENRV-DPFRT----  124
_XP_014226628.1_    78  ----KGVCTVQFP--------------QDTMTITFDKLGVQCVKKKEIEEALRVREEIRV-DPFKT----  124
_XP_020287946.1_    78  ----QGICTVEVP--------------AGNMVVTFSNLGIQCVKKKDIEEALRVRQDLRV-DPFRT----  124
_XP_012232007.1_    78  ----QGVCTVEVP--------------AGNMVVTFSNLGIQCVKKKDIEEALKVRQELRV-DPFRT----  124
_XP_011874538.1_    78  ----QGVCTVEVP--------------SGNMVVTFSNLGIQCVKKKDIEDALRVRQDLRV-DPFRT----  124
_XP_011874541.1_    78  ----QGVCTVEVP--------------SGNMVVTFSNLGIQCVKKKDIEDALRVRQDLRV-DPFRT----  124
_XP_011173619.1_    78  ----QGVCTVEVP--------------SGNMVVTFSNLGIQCVKKKDIEEALRVRQELRV-DPFRT----  124
_XP_034950793.1_    78  ----QGVCTVERY--------------TDQEPIVFPTLGIQCVKKKDIAEALQQRKKARV-DPFKT----  124
_XP_034950792.1_    74  ----QGVCTVERY--------------TDQEPIVFPTLGIQCVKKKDIAEALQQRKKARV-DPFKT----  120
_XP_012340880.2_    77  ----KGVCTNEIP--------------SEDMTVTFSNLGIMCVKRKDIRRELKIREEIRV-DPFQT----  123
_XP_031771859.1_    77  ----KGVCTNEIP--------------SEDMTVTFSNLGIMCVKRKDIRRELKIREEIRV-DPFQT----  123
_XP_006618953.1_    77  ----KGVCTIEIP--------------LEDMTVTFANLGIMCVKRKDVKRELKIREEIRV-DPFRT----  123
_XP_031367904.1_    77  ----KGVCTIEIP--------------LEDMTVTFANLGIMCVKRKDVKRELKIREEIRV-DPFRT----  123
_XP_043795948.1_    77  ----KGVCTIEIP--------------LEDMTVTFANLGIMCVKKKDVKRELKIREEIRV-DPFRT----  123
_XP_395180.5_       77  ----KGICTIEIS--------------SEDMTVTFANLGIMCVKRNDIKRELKIREEIRV-DPFRT----  123
_KAG6799500.1_      77  ----KGICTIEIS--------------SEDMTVTFANLGIMCVKRNDIKRELKIREEIRV-DPFRT----  123
_XP_016768607.2_    77  ----KGICTIEIS--------------SEDMTVTFANLGIMCVKRNDIKRELKIREEIRV-DPFRT----  123
_PBC31989.1_        86  ----KGVCTIEIT--------------SEDMTVTFANLGIMCVKRNDIKRELKIREEIRV-DPFRT----  132
_XP_016914450.1_    77  ----KGVCTIEIA--------------SEDMTVTFANLGIMCVKRNDIKRELKIREEIRV-DPFRT----  123
_KAG5344958.1_      78  ----QGVCTVEIP--------------SGNMVVTFSNLGIQCVKKKDIEEALKMRQELRV-DPFRT----  124
_KAJ3630188.1_      75  ----KGVCTFTTN-------------ITADTTISFTKQAVQCVKKRDLKKSLKEREQDNI-DPFRT----  122
_QHN75199.1_        75  ----KGVCTFTTN-------------ITADTTISFTKQAVQCVKKRDLKKSLKEREQDNI-DPFRT----  122
_XP_051171660.1_    81  ---KNGVCRVRLR--------------GDNMTVSFPNLGIQCVRRKDVEASLTEREKERV-DPFAV----  128
_XP_016909447.1_    36  ----QGVCTVEVS--------------SENMTVTFANLGIQCVKKKDIEEALKIREEIRV-DPFRT----   82
_XP_031773030.1_    36  ----QGVCTVEVS--------------SENMTVTFANLGIQCVKKKDIEEALKIREEIRV-DPFRT----   82
_KAH0956427.1_      78  ----QGICTIYTKDV----------RNGEKNVVVFSNMNIECITRKEIEKSLDMREKLRV-DPFRT----  128
```

```
_XP_012283784.1_    78   ----RGVCTVEIA--------------SENMTVTFANLGIQCVKKKDIEDALRVREEIRV-DPFRT----   124
_XP_023224928.1_    78   ----KGICSLEIK--------------SADMVQSFTSLGIQCVKKKNIEASLNQREQLRV-DPFRT----   124
_XP_008556434.1_    78   ----RGVCTLEIS--------------SENMTVTFANLGIQCVKKKDIEEALRVREEIRV-DPFRT----   124
_XP_008556433.1_    78   ----RGVCTLEIS--------------SENMTVTFANLGIQCVKKKDIEEALRVREEIRV-DPFRT----   124
_XP_014299728.1_    78   ----RGVCTLEIS--------------SENMTVTFANLGIQCVKKKDIEEALRVREEIRV-DPFRT----   124
_CAD6234449.1_      78   ----RGVCTLEIS--------------SENMTVTFANLGIQCVKKKDIEEALRIREEIRV-DPFRT----   124
_KAG8037552.1_      78   ----RGVCTLEIS--------------SENMTVTFANLGIQCVKKKDIEEALRVREEIRV-DPFRT----   124
_XP_044576138.1_    78   ----RGVCTLEIS--------------SENMTVTFANLGIQCVKKKDIEEALRIREEIRV-DPFRT----   124
_XP_044576125.1_    78   ----RGVCTLEIS--------------SENMTVTFANLGIQCVKKKDIEEALRIREEIRV-DPFRT----   124
_XP_026465448.1_    78   ----KGVCTMQVN--------------TDTMSVTFSNLGIQCVKKKDIEEALKARELIKV-DPFGN----   124
_XP_026465446.1_    78   ----KGVCTMQVN--------------TDTMSVTFSNLGIQCVKKKDIEEALKARELIKV-DPFGN----   124
_XP_031785072.1_    78   ----RGVCTVEVP--------------SENMTVSFDKLGIQCVKRKDIEEALRLREENRV-DPFRT----   124
_XP_015607185.1_    78   ----RGVCTVEIA--------------SENMTVTFANLGIQCVKKKDIEEALRIREEIRV-DPFRT----   124
_XP_011496611.1_    78   ----QGVGTVEVS--------------SDNMTVSFDKLGIQCAKRIDIEEALKLREEIRV-DPFRT----   124
_XP_018903707.1_    78   ----RGICTVEVN--------------NESMTASFPNLGIQCIKRKDMVAALEEREKIKV-DPFRM----   124
_CAB3365562.1_      77   ----QGVCTTHVD--------------TETMTCQFPNLGIQCVKKKEVERSLEVRQKIRV-DPFRR----   123
_XP_011496447.1_         ----------------------------------------------------------------------
_KAG8230996.1_      77   ----KGVCTVEMN--------------NENMVCTFSNLGIQCVKKKDIEEALKVREEIRV-DPYRT----   123
_XP_040567422.1_    78   ---KKGVCSMDIN--------------NDEMMCSFQNLGIQCVKRKDAPDSLTQRQMIRV-DPFKQ----   125
_XP_014250205.1_    78   ----KGVCTVEVN--------------NDNMIVSFANLGIQCVKKKDIEEALKIREEIRV-DPFRT----   124
_XP_014250206.1_    78   ----KGVCTVEVN--------------NDNMIVSFANLGIQCVKKKDIEEALKIREEIRV-DPFRT----   124
_XP_047119528.1_    78   ----RGVCTVEIS--------------PDNMTASFSNLGIQCVKKKDIDEALKTREEIRV-DPFRT----   124
_XP_047001845.1_    78   ----RGVCTVEIS--------------PDNMTASFSNLGIQCVKKKDIDEALKTREEIRV-DPFRT----   124
_XP_049939433.1_    78   ----RGVCTVEIS--------------PDNMTASFSNLGIQCVKKKDIDEALKTREEIRV-DPFRT----   124
_XP_049764197.1_    78   ----RGVCTVEIS--------------PDNMTASFSNLGIQCVKKKDIDEALKTREEIRV-DPFRT----   124
_XP_049833656.1_    78   ----RGVCTVEIS--------------PDNMTASFSNLGIQCVKKKDIDEALKTREEIRV-DPFRT----   124
_XP_049790026.1_    78   ----RGVCTVEIS--------------PDNMTASFSNLGIQCVKKKDIDEALKTREEIRV-DPFRT----   124
_XP_049833651.1_    78   ----RGVCTVEIS--------------PDNMTASFSNLGIQCVKKKDIDEALKTREEIRV-DPFRT----   124
_XP_047001842.1_    78   ----RGVCTVEIS--------------PDNMTASFSNLGIQCVKKKDIDEALKTREEIRV-DPFRT----   124
_XP_049790025.1_    78   ----RGVCTVEIS--------------PDNMTASFSNLGIQCVKKKDIDEALKTREEIRV-DPFRT----   124
_XP_049833654.1_    78   ----RGVCTVEIS--------------PDNMTASFSNLGIQCVKKKDIDEALKTREEIRV-DPFRT----   124
_XP_049833659.1_    78   ----RGVCTVEIS--------------PDNMTASFSNLGIQCVKKKDIDEALKTREEIRV-DPFRT----   124
_XP_021925503.1_    78   ----KGICTTRIN--------------NEAMAVSFSNLGIQCVKKKDIEEALKLREEIRV-DPFRS----   124
_KDR23857.1_        78   ----KGICTTRIN--------------NEAMAVSFSNLGIQCVKKKDIEEALKLREEIRV-DPFRS----   124
_ABU96700.1_        78   ----KGVCTVEIN--------------NETMTAAFANLGIQCVKKKDIEEALRVREEIRV-DPFRT----   124
_ABU96699.1_        78   ----KGVCTVEIN--------------NETMTAAFANLGIQCVKKKDIEEALRVREEIRV-DPFRT----   124
_XP_014275495.1_    78   ----RGVCTVEVG--------------GETMTATFANLGIQCVKKKDIEEALKVREEMRV-DPFRT----   124
_BBE08137.1_        78   ----KGVCTVEVS--------------GETMTATFANLGIQCVKKKDIEEALKVREEMRV-DPFKT----   124
_XP_014275494.1_    78   ----RGVCTVEVG--------------GETMTATFANLGIQCVKKKDIEEALKVREEMRV-DPFRT----   124
```

```
_XP_014275493.1_   78  ----RGVCTVEVG--------------GETMTATFANLGIQCVKKKDIEEALKVREEMRV-DPFRT----  124
_BBE08136.1_       78  ----KGVCTVEVS--------------GETMTATFANLGIQCVKKKDIEEALKVREEMRV-DPFKT----  124
_KAF6207519.1_     78  ----KGVCTVEVN--------------NENMIATFANLGIQCVKKKDIEEALKIREEIRV-DPFRT----  124
_CAB0015508.1_     78  ----KGVCTVEIN--------------NDNMIATFANLGIQCVKKKDIEEALKIREEIRV-DPFRT----  124
_CAB0015504.1_     78  ----KGVCTVEIN--------------NDNMIATFANLGIQCVKKKDIEEALKIREEIRV-DPFRT----  124
_PNF18474.1_       78  ----RGICTVPIK--------------EETMTASFCNLGIQCVKKKDIEEALKIREEIRV-DPFRT----  124
_CEG62431.1_       78  ----KGICTMRIN--------------SDTMTVTFSNLGIQCVKKKDIEDALKVREEIRV-DPFHT----  124
_PSN36690.1_       78  ----KGICTMRIN--------------SDTMTVTFSNLGIQCVKKKDIEDALKVREEIRV-DPFHT----  124
_XP_046387412.1_   78  ----KGVCTVEMN--------------SENMVCTFSNLGIQCVKKKDIEEALKVREDIRV-DPYRT----  124
_XP_046387414.1_   78  ----KGVCTVEMN--------------SENMVCTFSNLGIQCVKKKDIEEALKVREDIRV-DPYRT----  124
_XP_031634874.1_   78  ----KGVCTLEIN--------------NDNMTATFANLGIQCVKKKDIDDSLKEREKIRV-DPFRK----  124
_GFG29240.1_       78  ----KGICTMRIS--------------VDTMTASFSNLGIQCVKKKDIEEALKIREEIRV-DPFRS----  124
_BBG28464.1_       78  ----KGVCTVEIS--------------GEAMTATFSNLGIQCVKKKDIDEALRIREGIRV-DPFHT----  124
_WCL52334.1_       78  ----KGVCTVEIS--------------GEAMTATFSNLGIQCVKKKDIDEALRIREGIRV-DPFHT----  124
_GLH00135.1_       78  ----KGVCTVEIS--------------GEAMTATFSNLGIQCVKKKDIDEALRIREGIRV-DPFHT----  124
_CAD7205239.1_     78  ----KGVCTVEIN--------------TDSMAVSFSNLGIQCVKKKDIEEALRVRKEIRV-DPFRN----  124
_CAD7431383.1_     78  ----KGVCTVEIN--------------TDSMAVSFSNLGIQCVKKKDIEEALRVRKEIRV-DPFRN----  124
_CAD7410836.1_     78  ----KGVCTVEIN--------------TDSMAVSFSNLGIQCVKKKDIEEALRVRKEIRV-DPFRN----  124
_CAD7415345.1_     78  ----KGVCTVEIN--------------TDSMAVSFSNLGIQCVKKKDIEEALRVRKEIRV-DPFRN----  124
_CAD7606635.1_     78  ----KGVCTVEMN--------------SDSMAVSFSNLGIQCVKKKDIEEALRVREEIRV-DPFRT----  124
_CAD7462957.1_     78  ----KGVCTVEMN--------------SDSMAVSFSNLGIQCVKKKDIEEALRVREEIRV-DPFRT----  124
_XP_012265400.2_   78  ----RGVCTVEIA--------------TESMTVTFANLGIQCVKKKDIEDALRVREEIRV-DPYRT----  124
_XP_046473587.1_   78  ----KGICTLQIP--------------PESMTVTFSNLGIQCVKKKDIEEALKVREEIRV-DPFRT----  124
_XP_046611732.1_   78  ----KGICTLEIP--------------PESMTVTFSNLGIQCVKKKDIEEALKVREEIRV-DPFRT----  124
_XP_015519750.2_   78  ----KGICTLQIP--------------PESMTVTFSNLGIQCVKKKDIEEALKVREEIRV-DPFRT----  124
_XP_046611731.1_   78  ----KGICTLEIP--------------PESMTVTFSNLGIQCVKKKDIEEALKVREEIRV-DPFRT----  124
_XP_046417903.1_   78  ----KGICTLEIP--------------PESMTVTFSNLGIQCVKKKDIEEALKVREEIRV-DPFRT----  124
_GLG94364.1_       78  ----RGVCTLEL---------------SDDMTATFSNLGIQCVKKRDIDEALRVREEIRV-DPFRT----  123
_GLG94363.1_       78  ----RGVCTLEL---------------SDDMTATFSNLGIQCVKKRDIDEALRVREEIRV-DPFRT----  123
_BBG28463.1_       78  ----RGVCTLEL---------------SDDMTATFSNLGIQCVKKRDIDEALRVREEIRV-DPFRT----  123
_WCL52335.1_       78  ----RGVCTLEL---------------SDDMTATFSNLGIQCVKKRDIDEALRVREEIRV-DPFRT----  123
_GLG94365.1_       78  ----RGVCTLEL---------------SDDMTATFSNLGIQCVKKRDIDEALRVREEIRV-DPFRT----  123
_XP_002431518.1_   78  ----RGVCTLKMN--------------SDT--ITFSNLGIQCVKKKDIEEALKVREEIRV-DPFRT----  122
_KAE8746525.1_     78  ----RGVFSQHVS--------------SNNMIVSFSNLGIQCVKKKDIEDALRVREEIRV-DPFRT----  124
_XP_026277770.1_   78  ----RGVFSQHVS--------------SNNMIVSFSNLGIQCVKKKDIEDALRVREEIRV-DPFRT----  124
_KAJ1519446.1_     78  ----RGVFSQHVC--------------SKNMIVSFSNLGIQCVKKKDIDEALRVREEIRV-DPFRT----  124
_ALC78349.1_       78  ----QGVCTVNIN--------------EETMSVTFSNLGIQCVKKKDIEEALRVRQEIKV-DPYKT----  124
_XP_046738772.1_   78  ----KGICTLEIP--------------PESMTVTFSNLGIQCVKKKDIEEALKVREEIRV-DPFRT----  124
_XP_046417904.1_   78  ----KGICTLEIP--------------PESMTVTFSNLGIQCVKKKDIEEALKVREEIRV-DPFRT----  124
```

```
_CRK94170.1_       78  ----RGVCTLEIN--------------TDDMKLAFSNLGIQCVKKRDIEEALKTREEIRV-DPYRT----  124
_CRK94167.1_       78  ----RGVCTLEIN--------------TDDMKLAFSNLGIQCVKKRDIEEALKTREEIRV-DPYRT----  124
_KAG5676160.1_     78  ----KGVCTLEIN--------------SDDMKMVFSNLGIQCVKRRDIEEALRIREEIRV-DPYRT----  124
_XP_034245464.1_   78  ----KGVFSQQVH-------------SNNNMIVSFSNLGIQCVKKRDIEEALRVREEIRV-DPFGK----  125
_XP_034245457.1_   78  ----KGVFSQQVH-------------SNNNMIVSFSNLGIQCVKKRDIEEALRVREEIRV-DPFGK----  125
_KAG8287980.1_     78  ----KGVCTVEVH--------------CESMTQSFDHLGIQCVKKKDIEEALKAREEIRV-DPFRT----  124
_XP_044759045.1_   76  ----KGVCTVKVP--------------EGTNIVEFSNIGIKCVRKKDAAESLKERKALKI-DPFRT----  122
_XP_017305229.1_   77  ----NGIFCATLL--------------EGDMTYSFTNLGIQCMKKKDIQASLQQREKQRV-DPFGL----  123
_XP_026675878.1_   77  ----NGIFCATLL--------------EGDMTYSFTNLGIQCMKKKDIQASLQQREKQRV-DPFGL----  123
_KAE8574083.1_     78  ----RGVCTVEVG--------------GETMTATFANLGIQCVKKKDIEEALKVREEMRV-DPFR-----  123
_XP_037926497.1_   78  ----KGVCTVEIN--------------SETMRAVFSNLGIQCVKKKDIESALRAREEIRV-DPFKT----  124
_CAD7079567.1_     78  ----KGVCTVEIN--------------SETMRAVFSNLGIQCVKKKDIESALRAREEIRV-DPFKT----  124
_XP_037882485.1_   78  ----KGVCTIEIN--------------SETMRTVFSNLGIQCVKKKDIEAALKAREEIRV-DPFKT----  124
_XP_037037597.1_   78  ----KGVCTVEIN--------------SDTMSVTFSNLGIQCVKKRDIDEALRVREEIRV-DPYRT----  124
_XP_037037592.1_   78  ----KGVCTVEIN--------------SDTMSVTFSNLGIQCVKKRDIDEALRVREEIRV-DPYRT----  124
_KAG4075389.1_     78  ----KGVCTVEIN--------------SDTMSVTFSNLGIQCVKKRDIDEALRVREEIRV-DPYRT----  124
_XP_034101357.1_   78  ----KGVCTLEIS--------------SETMRAVFSNLGIQCVKKKDIEAALKAREEIRV-DPFKT----  124
_XP_026844963.1_   78  ----KGVCTLEIN--------------SETMRAVFSNLGIQCVKKKDIEAALKAREEIRV-DPFKT----  124
_EDW28798.1_       78  ----KGVCTLEIN--------------SETMRAVFSNLGIQCVKKKDIEAALKAREEIRV-DPFKT----  124
_XP_034125966.1_   78  ----KGVCTLEIN--------------SETMRAVFSNLGIQCVKKKDIEAALKAREEIRV-DPFKT----  124
_XP_034666519.1_   78  ----KGVCTLEIN--------------SETMRAVFSNLGIQCVKKKDIEAALKAREEIRV-DPFKT----  124
_XP_001356570.3_   78  ----KGVCTLEIN--------------SETMRAVFSNLGIQCVKKKDIEAALKAREEIRV-DPFKT----  124
_XP_023164577.2_   78  ----KGVCTLEIS--------------SETMRAVFSNLGIQCVKKKDIEAALKAREEIRV-DPFKT----  124
_XP_017153206.1_   78  ----KGVCTLEIN--------------SETMRAVFSNLGIQCVKKKDIEAALKAREEIRV-DPFKT----  124
_XP_030564932.1_   78  ----KGVCTLEIS--------------SETMRAVFSNLGIQCVKKKDIEAALKAREEIRV-DPFKT----  124
_XP_002059145.1_   78  ----KGVCTLEIS--------------SETMRAVFSNLGIQCVKKKDIEAALKAREEIRV-DPFKT----  124
_EDW04266.1_       78  ----KGVCTLEIS--------------SETMRAVFSNLGIQCVKKKDIEAALKAREEIRV-DPFKT----  124
_XP_017025862.1_   78  ----KGVCTLEIN--------------SETMRAVFSNLGIQCVKKKDIEAALKAREEIRV-DPFKT----  124
_XP_017859907.1_   78  ----KGVCTLEIS--------------SETMRAVFSNLGIQCVKKKDIEAALKAREEIRV-DPFKT----  124
_XP_032584327.1_   78  ----KGVCTLEIS--------------SETMRAVFSNLGIQCVKKKDIEAALKAREEIRV-DPFKT----  124
_XP_016987288.2_   78  ----KGVCTLEIN--------------SETMRAVFSNLGIQCVKKKDIEAALKAREEIRV-DPFKT----  124
_XP_017954173.1_   78  ----KGVCTLEIS--------------SETMRAVFSNLGIQCVKKKDIEAALKAREEIRV-DPFKT----  124
_XP_017854504.1_   78  ----KGVCTLEIS--------------SETMRAVFSNLGIQCVKKKDIEAALKAREEIRV-DPFKT----  124
_XP_034125965.1_   78  ----KGVCTLEIN--------------SETMRAVFSNLGIQCVKKKDIEAALKAREEIRV-DPFKT----  124
_XP_034666515.1_   78  ----KGVCTLEIN--------------SETMRAVFSNLGIQCVKKKDIEAALKAREEIRV-DPFKT----  124
_XP_043641917.1_   78  ----KGVCTLEIN--------------SETMRAVFSNLGIQCVKKKDIEAALKAREEIRV-DPFKT----  124
_XP_026844960.1_   78  ----KGVCTLEIN--------------SETMRAVFSNLGIQCVKKKDIEAALKAREEIRV-DPFKT----  124
_XP_015035988.2_   78  ----KGVCTLEIN--------------SETMRAVFSNLGIQCVKKKDIEAALKAREEIRV-DPFKT----  124
_XP_041450744.1_   78  ----KGVCTLEIN--------------SETMRAVFSNLGIQCVKKKDIEAALKAREEIRV-DPFKT----  124
```

```
_XP_015052361.1_    78  ----KGVCTLEIN--------------SETMRAVFSNLGIQCVKKKDIEAALKAREEIRV-DPFKT----  124
_XP_039480515.1_    78  ----KGVCTLEIN--------------SETMRAVFSNLGIQCVKKKDIEAALKAREEIRV-DPFKT----  124
_XP_033248563.1_    78  ----KGVCTLEIN--------------SETMRAVFSNLGIQCVKKKDIEAALKAREEIRV-DPFKT----  124
_KAH8412150.1_      78  ----KGVCTLEIN--------------SETMRAVFSNLGIQCVKKKDIEAALKAREEIRV-DPFKT----  124
_XP_043864974.1_    78  ----KGVCTLEIS--------------SETMRAVFSNLGIQCVKKKDIEAALKAREEIRV-DPFKT----  124
_XP_020809119.1_    78  ----KGVCTLEIN--------------SETMRAVFSNLGIQCVKKKDIEAALKAREEIRV-DPFKT----  124
_KAH8298353.1_      78  ----KGVCTLEIN--------------SETMRAVFSNLGIQCVKKKDIEAALKAREEIRV-DPFKT----  124
_XP_030564927.1_    78  ----KGVCTLEIS--------------SETMRAVFSNLGIQCVKKKDIEAALKAREEIRV-DPFKT----  124
_XP_041631592.1_    78  ----KGVCTLEIN--------------SETMRAVFSNLGIQCVKKKDIEAALKAREEIRV-DPFKT----  124
_XP_017859909.1_    78  ----KGVCTLEIS--------------SETMRAVFSNLGIQCVKKKDIEAALKAREEIRV-DPFKT----  124
_XP_032591602.1_    78  ----KGVCTLEIS--------------SETMRAVFSNLGIQCVKKKDIEAALKAREEIRV-DPFKT----  124
_XP_023164572.2_    78  ----KGVCTLEIS--------------SETMRAVFSNLGIQCVKKKDIEAALKAREEIRV-DPFKT----  124
_TDG46699.1_        78  ----KGVCTLEIS--------------SETMRAVFSNLGIQCVKKKDIEAALKAREEIRV-DPFKT----  124
_KAH8411895.1_      78  ----KGVCTLEIS--------------SETMRAVFSNLGIQCVKKKDIEAALKAREEIRV-DPFKT----  124
_KAH8377496.1_      78  ----KGVCTLEIS--------------SETMRAVFSNLGIQCVKKKDIEAALKAREEIRV-DPFKT----  124
_XP_017854500.2_    78  ----KGVCTLEIS--------------SETMRAVFSNLGIQCVKKKDIEAALKAREEIRV-DPFKT----  124
_XP_016987284.2_    78  ----KGVCTLEIN--------------SETMRAVFSNLGIQCVKKKDIEAALKAREEIRV-DPFKT----  124
_KAH8293209.1_      78  ----KGVCTLEIS--------------SETMRAVFSNLGIQCVKKKDIEAALKAREEIRV-DPFKT----  124
_EEC00225.1_        78  ----KGICTQYLK---------------PDMTCSFTSLGIQCVKRRDVEQNLADRESIRV-DPFQN----  123
_XP_029825505.2_    78  ----KGICTQYLK---------------PDMTCSFTSLGIQCVKRRDVEQNLADRESIRV-DPFQN----  123
_KAG0424790.1_      78  ----KGICTQYLK---------------PDMTCSFTSLGIQCVKRRDVEQNLADRESIRV-DPFQN----  123
_XP_037272127.1_    78  ----NGICTQHLK---------------PDMTCTFTSLGIQCVKRRDVEQNLLQRENIRV-DPFRN----  123
_XP_054934017.1_    78  ----NGICTQHLK---------------PDMTCTFTSLGIQCVKRRDVEQNLVQRENIRV-DPFRN----  123
_XP_050048524.1_    78  ----NGICTQHLK---------------PDMTCTFTSLGIQCVKRRDVEQNLVQRENIRV-DPFRN----  123
_XP_037272126.1_    78  ----NGICTQHLK---------------PDMTCTFTSLGIQCVKRRDVEQNLLQRENIRV-DPFRN----  123
_XP_037524244.1_    78  ----NGICTQHLK---------------PDMTCTFTSLGIQCVKRRDVEQNLLQRENIRV-DPFRN----  123
_XP_037524245.1_    78  ----NGICTQHLK---------------PDMTCTFTSLGIQCVKRRDVEQNLLQRENIRV-DPFRN----  123
_XP_049512094.1_    78  ----NGICTQHLK---------------PDMTCTFTSLGIQCVKRRDVEQNLVQRENIRV-DPFRN----  123
_XP_037524243.1_    78  ----NGICTQHLK---------------PDMTCTFTSLGIQCVKRRDVEQNLLQRENIRV-DPFRN----  123
_XP_037580160.1_    78  ----NGICTQHLK---------------PDMTCTFTSLGIQCVKRRDVEQNLVQRENIRV-DPFRN----  123
_XP_037272128.1_    78  ----NGICTQHLK---------------PDMTCTFTSLGIQCVKRRDVEQNLLQRENIRV-DPFRN----  123
_KAH9380259.1_      78  ----NGICTQHLK---------------PDMTCTFTSLGIQCVKRRDVEQNLVQRETIRV-DPFRN----  123
_KAI1291715.1_      50  ----NGICTMFMN--------------SSTMVQSFTNMGIQCVKRKKFGDSLKLRESLEI-DPFKT----   96
_XP_035209023.1_    79  ----DGIFSRKVA--------------ASSY--SFTDLTIQCMRRNQIDESLRLRRNYNI-DPYQT----  123
_XP_023332679.1_    81  --CKFGICTQVIN--------------PETMTAEFPHLGIQCVRRKDIKRSLDLRREKKV-DPYRQ----  129
_XP_046801942.1_    78  ----KGVCTLEIN--------------SETMRAVFSNLGIQCVKKKDIEAALKAREEIRV-DPFKT----  124
_XP_037811120.1_    78  ----KGVCTLEIN--------------SETMRAVFSNLGIQCVKKKDIEAALKAREEIRV-DPFKT----  124
_AFI98401.1_        78  ----KGVCTLEIN--------------SETMRAVFSNLGIQCVKKKDIEGALKAREEIRV-DPFKT----  124
_AFJ54621.1_        78  ----KGVCTLEIN--------------SETMRAVFSNLGIQCVKKKDIEGALKAREEIRV-DPFKT----  124
```

```
_KAI8126534.1_      78  ----KGVCTLEIN--------------SETMRAVFSNLGIQCVKKKDIEAALKAREEIRV-DPFKT----  124
_XP_013118266.1_    78  ----KGVCTLEIN--------------SETMRAVFSNLGIQCVKKKDIEAALKAREEIRV-DPFKT----  124
_XP_023293634.2_    78  ----KGVCTLEIN--------------SETMRAVFSNLGIQCVKKKDIEAALKAREEIRV-DPFKT----  124
_XP_037811062.1_    78  ----KGVCTLEIN--------------SETMRAVFSNLGIQCVKKKDIEAALKAREEIRV-DPFKT----  124
_KNC34132.1_        78  ----KGVCTLEIN--------------SETMRAVFSNLGIQCVKKKDIEAALKAREEIRV-DPFKT----  124
_TMW48951.1_        78  ----KGVCTLEIN--------------SETMRAVFSNLGIQCVKKKDIEAALKAREEIRV-DPFKT----  124
_XP_037957363.1_    78  ----KGVCTLEIN--------------SETMRAVFSNLGIQCVKKKDIEAALKAREEIRV-DPFKT----  124
_XP_005175014.1_    78  ----KGVCTLEIN--------------SETMRAVFSNLGIQCVKKKDIEAALKAREEIRV-DPFKT----  124
_XP_037957360.1_    78  ----KGVCTLEIN--------------SETMRAVFSNLGIQCVKKKDIEAALKAREEIRV-DPFKT----  124
_XP_030383330.1_    78  ----KGVCTLEIN--------------SETMRAVFSNLGIQCVKKKDIEAALKAREDIRV-DPFKT----  124
_XP_030383328.1_    78  ----KGVCTLEIN--------------SETMRAVFSNLGIQCVKKKDIEAALKAREDIRV-DPFKT----  124
_CAH1707987.1_      77  ----KGVCTLEIN--------------TDDMKMVFSNLGIQCVKRRDIEEALRVREEIRV-DPFRT----  123
_TMW54796.1_        78  ----KGVCTLEIN--------------SETMKAVFSNLGIQCVKKKDIEAALRAREEIRV-DPFKT----  124
_XP_026836077.1_    78  ----KGVCTLEIN--------------SETMRAVFSNLGIQCVKKKDIEAALKAREEIRV-DPFKT----  124
_XP_017116803.1_    78  ----KGVCTLEIN--------------SETMRAVFSNLGIQCVKKKDIESALKAREEIRV-DPFKT----  124
_XP_039480519.1_    78  ----KGVCTLEIN--------------SETMRAVFSNLGIQCVKKKDIEAALKAREEIRV-DPFKT----  124
_XP_043641920.1_    78  ----KGVCTLEIN--------------SETMRAVFSNLGIQCVKKKDIEAALKAREEIRV-DPFKT----  124
_XP_023036748.1_    78  ----KGVCTLEIN--------------SETMRAVFSNLGIQCVKKKDIEAALKAREEIRV-DPFKT----  124
_XP_002090466.1_    78  ----KGVCTLEIN--------------SETMRAVFSNLGIQCVKKKDIEAALKAREEIRV-DPFKT----  124
_XP_034471824.1_    78  ----KGVCTLEIS--------------SETMRAVFSNLGIQCVKKKDIEAALKAREEIRV-DPFKT----  124
_AAT94434.1_        78  ----KGVCTLEIN--------------SETMRAVFSNLGIQCVKKKDIEAALKAREEIRV-DPFKT----  124
_XP_036670985.1_    78  ----KGVCTLEIN--------------SETMRAVFSNLGIQCVKKKDIEAALKAREEIRV-DPFKT----  124
_AAA28479.1_        78  ----KGVCTLEIN--------------SETMRAVFSNLGIQCVKKKDIEAALKAREEIRV-DPFKT----  124
_XP_037708206.1_    78  ----KGVCTLEIN--------------SETMRAVFSNLGIQCVKKKDIEAALKAREEIRV-DPFKS----  124
_XP_002038915.1_    78  ----KGVCTLEIN--------------SETMRAVFSNLGIQCVKKKDIEAALKAREEIRV-DPFKT----  124
_XP_016993636.1_    78  ----KGVCTLEIN--------------SETMRAVFSNLGIQCVKKKDIEAALKAREEIRV-DPFKT----  124
_XP_052852517.1_    78  ----KGVCTLEIN--------------SETMRAVFSNLGIQCVKKKDIELALKAREEIRV-DPFKT----  124
_XP_016963139.1_    78  ----KGVCTLEIN--------------SETMRAVFSNLGIQCVKKKDIEAALKAREEIRV-DPFKT----  124
_XP_033156151.1_    78  ----KGVCTLEIN--------------SETMRAVFSNLGIQCVKKKDIDAALKAREEIRV-DPFKT----  124
_XP_001974446.1_    78  ----KGVCTLEIN--------------SETMRAVFSNLGIQCVKKKDIEAALKAREEIRV-DPFKT----  124
_XP_014761886.1_    78  ----KGVCTLEIN--------------SETMRAVFSNLGIQCVKKKDIEAALKAREEIRV-DPFKT----  124
_XP_017107216.2_    78  ----KGVCTLEIN--------------SETMRAVFSNLGIQCVKKKDIEAALKAREEIRV-DPFKT----  124
_XP_017044881.1_    78  ----KGVCTLEIN--------------SETMRAVFSNLGIQCVKKKDIEAALKAREQIRV-DPFKT----  124
_XP_016944798.1_    78  ----KGVCTLEIN--------------SETMRAVFSNLGIQCVKKKDIEAALKAREEIRV-DPFKT----  124
_XP_017116800.1_    78  ----KGVCTLEIN--------------SETMRAVFSNLGIQCVKKKDIESALKAREEIRV-DPFKT----  124
_XP_016993633.2_    78  ----KGVCTLEIN--------------SETMRAVFSNLGIQCVKKKDIEAALKAREEIRV-DPFKT----  124
_XP_037708198.1_    78  ----KGVCTLEIN--------------SETMRAVFSNLGIQCVKKKDIEAALKAREEIRV-DPFKS----  124
_XP_032583259.1_    78  ----KGVCTLEIN--------------SETMRAVFSNLGIQCVKKKDIEAALKAREEIRV-DPFKT----  124
_AAC35296.1_        78  ----KGVCTLEIN--------------SETMRAVFSNLGIQCVKKKDIEAALKAREEIRV-DPFKT----  124
```

```
_NP_001286014.1_    78  ----KGVCTLEIN--------------SETMRAVFSNLGIQCVKKKDIEAALKAREEIRV-DPFKT----  124
_XP_016963136.1_    78  ----KGVCTLEIN--------------SETMRAVFSNLGIQCVKKKDIEAALKAREEIRV-DPFKT----  124
_XP_017063904.2_    78  ----KGVCTLEIN--------------SETMRAVFSNLGIQCVKKKDIEAALKAREEIRV-DPFKT----  124
_XP_033156135.1_    78  ----KGVCTLEIN--------------SETMRAVFSNLGIQCVKKKDIDAALKAREEIRV-DPFKT----  124
_XP_052852512.1_    78  ----KGVCTLEIN--------------SETMRAVFSNLGIQCVKKKDIELALKAREEIRV-DPFKT----  124
_XP_032307758.1_    78  ----KGVCTLEIN--------------SETMRAVFSNLGIQCVKKKDIEAALKAREEIRV-DPFKT----  124
_XP_017107215.2_    78  ----KGVCTLEIN--------------SETMRAVFSNLGIQCVKKKDIEAALKAREEIRV-DPFKT----  124
_AWT86616.1_        77  ----HGVCTMEVN--------------TETMSLSFANLGIQCIKKKDIDNALNQREKLRI-DPFKT----  123
_QUF98534.1_        77  ----HGVCTMEVN--------------TETMALSFANLGIQCIKKKDIDAALNQREKLRI-DPFKT----  123
_UNA06287.1_        77  ----HGVCTMEVN--------------TETMSLSFANLGIQCIKKKDIDNALNQREKLRI-DPFKT----  123
_AAQ65068.1_        78  ----KGVCTLEIN--------------SETMRAVFSNLGIQCVKKKDIEAALKAREEIRV-DPFKT----  124
_AAQ64916.1_        78  ----KGVCTLEIN--------------SETMRAVFSNLGIQCVKKKDIEAALKAREEIRV-DPFKT----  124
_AAQ64921.1_        78  ----KGVCTLEIN--------------SETMRAVFSNLGIQCVKKKDIEAALKAREEIRV-DPFKT----  124
_AAQ64918.1_        78  ----KGVCTLEIN--------------SETMRAVFSNLGIQCVKKKDIEAALKAREEIRV-DPFKT----  124
_KAH8245459.1_      78  ----KGVCTLEIN--------------SETMRAVFSNLGIQCVKKKDIEAALKAREEIRV-DPFKSEYLE  128
_KAH8283155.1_      78  ----KGVCTLEIN--------------SETMRAVFSNLGIQCVKKKDIEAALKAREEIRV-DPFKSEYLR  128
_KAH8246384.1_      78  ----KGVCTLEIN--------------SETMRAVFSNLGIQCVKKKDIEAALKAREEIRV-DPFKSEYWE  128
_KAH8366418.1_      78  ----KGVCTLEIN--------------SETMRAVFSNLGIQCVKKKDIEAALKAREEIRV-DPFKSKWNY  128
_XP_022195378.2_    77  ----HGVCTMEVN--------------TETMALAFANLGIQCIKKKDIEAALSQREKLRI-DPFKT----  123
_XP_039284931.1_    77  ----HGVCTMEVN--------------TETMALAFANLGIQCIKKKDIEAALSQREKLRI-DPFKT----  123
_ALC40091.1_        78  ----KGVCTLEIS--------------SETMRAVFSNLGIQCVKKKDIEAALKAREEIRV-DPFKSKPNA  128
_XP_036342419.1_    78  ----SGVCTLEIK--------------SDSMRLELNSLGIQCVKKKDIEAALKAREDIRV-DPFKT----  124
_XP_022903025.1_    77  ----QGVCTVPIP--------------EDTMTVSFQNLGVQCVKKKEIEDALKAREDIRV-DPFMT----  123
_XP_022903017.1_    77  ----QGVCTVPIP--------------EDTMTVSFQNLGVQCVKKKEIEDALKAREDIRV-DPFMT----  123
_XP_036218946.1_    78  ----SGVYTFSIN--------------ENTMRAELCNLGIQCVKKKDIEASLKVREEIRV-DPFKT----  124
_XP_018790401.1_    78  ----SGVYTFSIN--------------EVTMRAELHNLGIQCVKKKDIEASLKMREEIRV-DPFKT----  124
_KAI9579887.1_      76  ----SGIFRDDIN--------------EDTMSMTFNHLGIQCVRKKDIEEALSEREGRKV-DPFQT----  122
_XP_037892537.1_    76  ----SGIFRDDIN--------------EDTMSMTFNHLGIQCVRKKDIEEALSEREGRKV-DPFQT----  122
_XP_018903705.1_    85  ----RGICTVEVN--------------NESMTASFPNLGIQCIKRKDMVAALEEREKIKV-DPFRM----  131
_CAH0395667.1_      85  ----RGICTVEVN--------------NESMTASFPNLGIQCIKRKDMVAALEEREKIKV-DPFRM----  131
_XP_050330165.1_    78  ----SGVYTFSVN--------------EVTMRAELCNLGIQCVKKKDIEASLKVREEVRV-DPFKT----  124
_XP_039970878.1_    78  ----SGVYTFSVN--------------EVTMRAELCNLGIQCVKKKDIEASLKVREEVRV-DPFKT----  124
_XP_049305234.1_    78  ----HGVYTFSVN--------------EVTMRAELCNLGIQCVKKKDIEGSLKTRQEIRV-DPFKT----  124
_GLH04793.1_        83  -ACHGGVCVAALN----------------GSRLPLPHLGVQCVKRRDVEIALREREKLRI-DPFQT----  130
_XP_015835035.1_    75  ----KGVYTFEAEI------------TPNNPDIVFRKLGVQCVKRNELKESLNVREEKNI-DPFKT----  123
_XP_015835036.1_    75  ----KGVYTFEAEI------------TPNNPDIVFRKLGVQCVKRNELKESLNVREEKNI-DPFKT----  123
_XP_039283832.1_    87  ---YGGACVKEVD--------------AETKTATLDHVGILCIKRKDMPTAFVERQARDI-DPFRT----  134
_XP_031788427.1_    78  ----NGIRSFITD--------------TQEMIVRFQNLRIQCVPRKNMESSLEVRKSLGV-DPFKT----  124
_KAJ6646894.1_      77  ----KGICRIDFLT-----------RKSDPTKVSLKNIGLQCCKKADAEKILTEREILRV-DPYRT----  126
```

```
_XP_026466084.1_    12  ----KGVCTAKIE-------------EATTMTITFNNLGVQCVKKKDIKQSLNLREDINV-DPFRT----   59
_XP_044002069.1_    77  ----QGIYVLPVV-------------LKNDNVINIPQLNILCIKKIDYAKTLQERAKINV-DPFQA----  124
_XP_046611735.1_    78  ----YGCYRAKIP--------------PKSRKVIFGHLGIQRVKRSEIEEALEMKEKKGV-DPYKT----  124
_XP_046417906.1_    78  ----YGCYRVKIP--------------PESRKVIFGHLGIQRVKRSEIEEALEMKEKKGV-DPYKT----  124
_XP_046590309.1_    78  ----YGCYRAKIP--------------PESRKVIFGHLGIQRVKRSEIEEALEMKEKKGV-DPYKT----  124
_XP_046473589.1_    78  ----YGCYRAKIP--------------PESRKVIFGHLGIQRVKRSEIEEALEMKEKKGV-DPYKT----  124
_XP_019892116.1_     1  ------------------------------MRAVFSNLGIQCVKKKDIEAALKAREEIRV-DPFKT----   35
_XP_005183027.3_     1  ------------------------------MRAVFSNLGIQCVKKKDIEAALKAREEIRV-DPFKT----   35
_XP_011496598.1_     5  ----EGICYFKTV--------------PNQSVVVFPHLGIQCIKKKDIDRSLTERERLQV-DPFKT----   51
_AAW67216.1_         1  ------------------------------------------VKKKDIEEALRLREEIRV-DPFKT----   23
_AAW67217.1_         1  ------------------------------------------VKKKDIEEALRLREEIRV-DPFKT----   23
_XP_017489788.1_     1  -------------------------------------LGIQCVKKKDIEAALKAREDIRV-DPFKT----   28
_BAI67894.1_         1  --------------------------------------GIQCVKKKDIEAALKAREEIRV-DPFKT----   27
_XP_054741511.1_    78  ----KGVCTLEIN--------------SDTMRAVFSNLGIQCVKKKDIEGALKAREDIRV-DPFKT----  124
_XP_053961684.1_    78  ----KGVCTLEIN--------------SDTMRAVFSNLGIQCVKKKDIEGALKAREDIRV-DPFKT----  124
_XP_036341313.1_    78  ----KGVCTLEIN--------------SETMRAVFSNLGIQCVKKKDIESALKAREDIRV-DPFKT----  124
_XP_036341343.1_    78  ----KGVCTLEIN--------------SETMRAVFSNLGIQCVKKKDIESALKAREDIRV-DPFKT----  124
_XP_017471383.1_    78  ----KGVCTLEIN--------------SETMRAVFSNLGIQCVKKKDIESALKAREDIRV-DPFKT----  124
_XP_054741498.1_    78  ----KGVCTLEIN--------------SDTMRAVFSNLGIQCVKKKDIEGALKAREDIRV-DPFKT----  124
_XP_053961677.1_    78  ----KGVCTLEIN--------------SDTMRAVFS-LGIQCVKKKDIEGALKAREDIRV-DPFKT----  123
_XP_050320008.1_    78  ----KGVCTLEIN--------------SDTMRAVFGNLGIQCVKKKDIESALKAREDIRV-DPFKT----  124
_XP_028894490.2_    78  ----KGVCTLEIN--------------SDTMRAVFSNLGIQCVKKKDIESALKAREDIRV-DPFKT----  124
_XP_050320015.1_    78  ----KGVCTLEIN--------------SDTMRAVFGNLGIQCVKKKDIESALKAREDIRV-DPFKT----  124
_XP_018787554.1_    78  ----KGVCTLEIN--------------SDTMRAVFGNLGIQCVKKKDIESALKAREDIRV-DPFKT----  124
_XP_029407438.2_    78  ----KGVCTLEIN--------------SDTMRAVFGNLGIQCVKKKDIESALKAREDIRV-DPFKT----  124
_XP_036225551.1_    78  ----KGVCTLEIN--------------SDTMRAVFGNLGIQCVKKKDIESALKAREDIRV-DPFKT----  124
_XP_011178374.2_    78  ----KGVCTLEIN--------------SDTMRAVFSNLGIQCVKKKDIESALKAREDIRV-DPFKT----  124
_XP_039952593.1_    78  ----KGVCTLEIN--------------SDTMRAVFGNLGIQCVKKKDIESALKAREDIRV-DPFKT----  124
_XP_018787542.1_    78  ----KGVCTLEIN--------------SDTMRAVFGNLGIQCVKKKDIESALKAREDIRV-DPFKT----  124
_XP_011207866.2_    78  ----KGVCTLEIN--------------SDTMRAVFGNLGIQCVKKKDIESALKAREDIRV-DPFKT----  124
_XP_014086506.1_    78  ----KGVCTLEIN--------------SDTMRAVFGNLGIQCVKKKDIESALKAREDIRV-DPFKT----  124
_XP_004526638.1_    78  ----KGVCTLEIN--------------SETMRAVFSNLGIQCVKKKDIESALKAREDIRV-DPFKT----  124
_XP_017471402.1_    78  ----KGVCTLEIN--------------SETMRAVFSNLGIQCVKKKDIESALKAREDIRV-DPFKT----  124
_XP_012157903.1_    78  ----KGVCTLEIN--------------SETMRAVFSNLGIQCVKKKDIESALKAREDIRV-DPFKT----  124
_XP_011185661.2_    78  ----HGVYTFSVN--------------EETMRGELCNLGIQCVKKKDIEASLKARKDIRV-DPFKT----  124
_XP_050322757.1_    78  ----SGVYTCSVN--------------EVTMRAELYNLGIQCVKKKDIEASLKMREEVRV-DPFKT----  124
_XP_039962922.1_    78  ----SGVYTCSVN--------------EVTMRAELYNLGIQCVKKKDIEASLKMREEVRV-DPFKT----  124
_XP_050322739.1_    78  ----SGVYTCSVN--------------EVTMRAELYNLGIQCVKKKDIEASLKMREEVRV-DPFKT----  124
_XP_050322749.1_    78  ----SGVYTCSVN--------------EVTMRAELYNLGIQCVKKKDIEASLKMREEVRV-DPFKT----  124
```

```
_XP_050322724.1_    78  ----SGVYTCSVN--------------EVTMRAELYNLGIQCVKKKDIEASLKMREEVRV-DPFKT----  124
_XP_050322682.1_    78  ----SGVYTCSVN--------------EVTMRAELYNLGIQCVKKKDIEASLKMREEVRV-DPFKT----  124
_CAG9823112.1_      79  --CRDGIFQTEVNV------------KGPFHLVDLKNVGIQCVKRRRMANSLKLREELKI-DPFNT----  129
_KAG5873705.1_      78  ----RGVCTLEIP-------------I-DTMTVQFSNLGIQCVKKKDIELALRTREEIRV-DPFRT----  124
_KAH8030364.1_      64  ----NGICTQHLK---------------PDMTCTFTSLGIQCVKRRD-------SKCLRS-SCIAD----  102
_XP_023013891.1_    78  ----RGVCTLEIP--------------VDTMTVQFSNLGIQCVKKKEIENALRTREEIRV-DPFRT----  124
_XP_028130780.2_    79  --CGKGIISLNNVS-----------IDGPHTLISFKNIGVQCVKRTSMAASLELRESLGV-DPFLT----  130
_KAG5873704.1_      79  --CTNGICRLEVDT------------RENFGVVQFRNMGVQCVKRRKMAVSLEIRMQQRV-DPFYT----  129
_CAH1187690.1_      79  ---RDGIVSKEVNL------------LKSGVDVSIKKVAVQRVKLSAMLASLQLRESQRIADPFAT----  129
_CAH1098641.1_      79  --CKNGILERILVI------------KGPETQISLKGIGVQRIKLNAMAKSLEEREFLEI-DPFST----  129
_XP_023028116.1_    79  --YKNGICKVKVDT------------NENYGVIQFKNMGVQCVKRRGMSASLEMRQAMRV-DPFKT----  129
_XP_031630227.1_    78  ----TGEIWRSVK-------------IEASKVVEFKDLCVIFVKKQDIKKSLELRRRQNI-DPTKG----  125
_KAG5861047.1_          ----------------------------------------------------------------------
_KAF2880990.1_      78  ----KGVCTLEVS--------------PETMSVTFSNLGIQCVKKKDIEAALKVREEIRV-DPFRT----  124
_XP_018326789.1_    78  ----KGVCTLEIS--------------PETMSVTFPNLGIQCVKKKDIESALRQREEIRV-DPFRT----  124
_XP_018326788.1_    78  ----KGVCTLEIS--------------PETMSVTFPNLGIQCVKKKDIESALRQREEIRV-DPFRT----  124
_XP_031339184.1_    78  ----KGVCTLEVS--------------PETMSVTFSNLGIQCVKKKDIESALRVREEIRV-DPFRT----  124
_XP_045476069.1_    78  ----RGVCTLEIP--------------TETMSIQFCNLGIQCVKKKEIETALRLREEIRV-DPFRT----  124
_XP_044734149.1_    78  ----KGVCTIEIP--------------TDTMTVTFSNLGIRCVKKRDIESALQTREEIRV-DPFRT----  124
_XP_044734150.1_    78  ----KGVCTIEIP--------------TDTMTVTFSNLGIRCVKKRDIESALQTREEIRV-DPFRT----  124
_XP_044734148.1_    78  ----KGVCTIEIP--------------TDTMTVTFSNLGIRCVKKRDIESALQTREEIRV-DPFRT----  124
_XP_044760528.1_    78  ----RGVCTLEIP--------------TETMSIQFCNLGIQCVKKKEIESALRLREEIRV-DPFRT----  124
_XP_044747918.1_    78  ----KGVCTLEIP--------------PDTMSVSFSNLGIQCVKKKDIESSLKTREEIRV-DPFRT----  124
_XP_044747917.1_    78  ----KGVCTLEIP--------------PDTMSVSFSNLGIQCVKKKDIESSLKTREEIRV-DPFRT----  124
_XP_045481017.1_    78  ----KGVCTLEIP--------------PDTMTVSFSNLGIQCVKKKDIESSLRTREEIRV-DPFRT----  124
_XP_045481018.1_    78  ----KGVCTLEIP--------------PDTMTVSFSNLGIQCVKKKDIESSLRTREEIRV-DPFRT----  124
_KAF5302274.1_      78  ----KGVCTLEVS--------------PETMSVTFSNLGIQCVKKKDIEAALKVREEIRV-DPFRT----  124
_KAF5308883.1_      78  ----KGVCTLEVS--------------PETMSVTFSNLGIQCVKKKDIEAALKVREEIRV-DPFRT----  124
_KRT79621.1_        77  ----KGVCTVQVP--------------EDTMTVSFQNLGIQCVKKKDIEEALKTREEIRV-DPFRT----  123
_BAD20728.1_        77  ----KGVCTVQVP--------------EDTMTVSFQNLGIQCVKKKDIEEALKTREEIRV-DPFRT----  123
_KAF2880988.1_      77  ----KGVCKLEVS--------------PETMSVTFSNLGIQCAKKKDVEASLKVREEIKV-DPFKT----  123
_CAH1403258.1_      70  ----KGVCTVEVG--------------GETMTATFANLGIQCVKKKDIEEALKVREEMRV-DPFKT----  116
_CAD6992094.1_      78  ----RGVYTVANK--------------TGTTRIKFCSLGVQCVKRTGVQASLKIRENIRV-DPFKT----  124
_XP_004537184.1_    78  ----RGVYTVANK--------------TGTTRIKFCSLGVQCVKRTGVQASLKIRENIRV-DPFKT----  124
_XP_018572644.1_    78  ----RGVCTLEIP--------------TDTMTVHFSNLGIQCVKKKDIEHALRVREEIRV-DPFRT----  124
_RZB54393.1__       78  ----RGVCTLEIP--------------ADTMIVTFSNLGIQCVKKKDIESALRVREEIKV-DPFMT----  124
_XP_044255725.1_    78  ----KGVCTMEIN--------------SDTMCVTFSNLGIQCVKKKDIESALRLREEIKV-DPFMT----  124
_EFA02850.1_        78  ----KGVCTMEIN--------------SDTMCVTFSNLGIQCVKKKDIESALRLREEIKV-DPFMT----  124
_NP_001034507.1_    78  ----KGVCTMEIN--------------SDTMCVTFSNLGIQCVKKKDIESALRLREEIKV-DPFMT----  124
```

```
_CAH1370311.1_      78  ----KGVCTMEIS---------------PDTMCVTFSNLGIQCVKKKDIENALRVREEIKV-DPFMT----  124
_KAJ3630692.1_      78  ----KGVCTMEIS---------------PDTMCVTFSNLGIQCVKKKDIENALRVREEIKV-DPFMT----  124
_XP_044255724.1_    78  ----KGVCTMEIN---------------SDTMCVTFSNLGIQCVKKKDIESALRLREEIKV-DPFMT----  124
_KAH0811528.1_      78  ----KGVCTMEIS---------------PDTMCVTFSNLGIQCVKKKDIENALRVREEIKV-DPFMT----  124
_CAH7716406.1_      78  ----SGICRLQIP---------------PDTMTISFPNLGIQCVKKKDIAQALDQRQRLQV-DPFKT----  124
_CAH7716405.1_      78  ----SGICRLQIP---------------PDTMTISFPNLGIQCVKKKDIAQALDQRQRLQV-DPFKT----  124
_CAH7716404.1_      78  ----SGICRLQIP---------------PDTMTISFPNLGIQCVKKKDIAQALDQRQRLQV-DPFKT----  124
_XP_028130776.2_    78  ----RGVCTLEIP---------------TDTMTVHFSNLGIQCVKKKDIEHALRVREEIRV-DPFRT----  124
_CAH1985684.1_      78  ----NGICRLQIP---------------PDTMTISFANLGIQCVKKKDIAQALDQRQRLQV-DPFKT----  124
_XP_017781152.1_    78  ----RGVCTMEIS---------------PDSMSISFSNLGIQCVKKKDIEAHLRVREEIRV-DPFKT----  124
_XP_017781151.1_    78  ----RGVCTMEIS---------------PDSMSISFSNLGIQCVKKKDIEAHLRVREEIRV-DPFKT----  124
_XP_048522053.1_    77  ----NGVCTVVIA---------------PETMRAQFQNLGIQCVKKRDIESSLRQRENIKV-DPFRQ----  123
_XP_050303070.1_    82  ----EGVCTVPIP---------------LDTMRVEFQNLGIQCVKKRDVEKSLTIREKLKV-DPFKR----  128
_CAG9762924.1_      78  ----NGVCTVIIP---------------ENTMRAQFQNLGIQCVKKRDIESSLRQREQIRV-DPFRH----  124
_CAH0560654.1_      79  ----KGVCTMEIP---------------AETMSVTFTNLGIQCVKKKDIESALREREELRV-DPFRT----  125
_CAH0560655.1_      79  ----KGVCTMEIP---------------AETMSVTFTNLGIQCVKKKDIESALREREELRV-DPFRT----  125
_XP_018572643.1_    78  ----NGLYKIECD---------------ASSF-IQLSNLGVQCVTRRKMEESLKIRQLLKV-DPFNT----  123
_XP_030763656.1_    78  ----KGVCTLHIP---------------QDTMRVQFSNLGIQCVKKRDIESSLRTREEIKV-DPFRQ----  124
_XP_030763654.1_    78  ----KGVCTLHIP---------------QDTMRVQFSNLGIQCVKKRDIESSLRTREEIKV-DPFRQ----  124
_XP_018577945.1_    78  ----KGVCTLNVP---------------TDTMTVHFSNLGIQCVKKKDIEQSLKIREEIRV-DPFRT----  124
_KAF7283276.1_      78  ----KGVCTLQIP---------------EDTMRVQFSNLGIQCVKKRDIEASLRQREEIRV-DPFRQ----  124
_KAF7283275.1_      78  ----KGVCTLQIP---------------EDTMRVQFSNLGIQCVKKRDIEASLRQREEIRV-DPFRQ----  124
_XP_030763617.1_    78  ----NGFCTLPIP---------------ADTMRVRFSKLAIQCVKKKDIKSSLKLKEKYNV-DYFRQ----  124
_XP_030763619.1_    81  ----HGVCTLTIP---------------VDTMRVEFPHLGIQCVKKKEVESSLTQRESIRV-DPFRQ----  127
_VEN60240.1_         1  ------------------------------MTISFPNLGIQCVKKKDIAQALDQRQRLQV-DPFKT----   35
_KOB78236.1_        65  ---KGGECNFKVDI-------------TAENPMVEIKNIGIQCMKRDECQDSLQDRARKGI-DPFRT----  114
_ERL85515.1_        20  ----NGVCTVVIA---------------PETMRAQFQNLGIQCVKKRDIESSLRQRENIKV-DPFRQ----   66
_ENN78766.1_        15  ----NGVCTVVIA---------------PETMRAQFQNLGIQCVKKRDIESSLRQRENIKV-DPFRQ----   61
_KAH1019371.1_      15  ----NGVCTVVIA---------------PETMRAQFQNLGIQCVKKRDIESSLRQRENIKV-DPFRQ----   61
_KAI9565420.1_      78  ----KGVCTLEIN---------------NENMICTFSNLGVQCVKKKGIEEALKLREEIRV-DPFHT----  124
_XP_045024905.1_    78  ----KGVCTLEIN---------------NENMICTFSNLGVQCVKKKGIEEALKLREEIRV-DPFHT----  124
_XP_046447094.1_    78  ----KGVCTLEIN---------------NDNMICTFSNLGVQCVKKKGIEEALKLREEIRV-DPFHT----  124
_KZS08518.1_        78  ----KGVCTLEIN---------------NENMICTFSNLGVQCVKKKGIEEALKLREEIRV-DPFHT----  124
_XP_046636307.1_    78  ----KGVCTLEIN---------------NDNMICTFSNLGVQCVKKKGIEEALKLREEIRV-DPFHT----  124
_EFX79013.1_        78  ----KGVCTLEIN---------------NDNMICTFSNLGVQCVKKKGIEEALKLREEIRV-DPFHT----  124
_XP_032776606.2_    78  ----KGVCTLEIN---------------NENMICTFSNLGVQCVKKKGIEEALKLREEIRV-DPFHT----  124
_XP_046447093.1_    78  ----KGVCTLEIN---------------NDNMICTFSNLGVQCVKKKGIEEALKLREEIRV-DPFHT----  124
_XP_046636306.1_    78  ----KGVCTLEIN---------------NDNMICTFSNLGVQCVKKKGIEEALKLREEIRV-DPFHT----  124
_CAH0099799.1_      78  ----KGVCTLEIN---------------NENMICTFSNLGVQCVKKKGIEEALKLP---------------  114
```

```
_KAJ1531033.1_      76  ----DGIFRQRITS-----------DDMDGMTISFPKLNIQCTKKKNVKEKLMIRQKRRV-DPFEA----  125
_ACJ36225.1_        78  ----KGVCTMTIN--------------NDSMQCVFSNLGIQCVKKRDVEDALKLREEIRV-DPFQT----  124
_ACZ98167.1_        78  ----KGVCTMTIN--------------NDSMQCVFSNLGIQCVKKRDVEDALKLREEIRV-DPFQT----  124
_AME17867.1_        78  ----KGVCTMTIN--------------NDSMQCVFSNLGIQCVKKRDVEDALKLREEIRV-DPFQT----  124
_XP_042868409.1_    78  ----KGVCTMTIN--------------NDSMQCVFSNLGIQCVKKRDVEDALKLREEIRV-DPFQT----  124
_XP_045102711.1_    78  ----KGVCTMTIN--------------NDSMQCVFSNLGIQCVKKRDVEDALKLREEIRV-DPFQT----  124
_XP_047487558.1_    78  ----KGVCTMTIN--------------NDSMQCVFSNLGIQCVKKRDVEDALKLREEIRV-DPFQT----  124
_QBJ05651.1_        78  ----KGVCTMTIN--------------NDSMQCVFSNLGIQCVKKRDVEDALKLREEIRV-DPFQT----  124
_AZK36046.1_        78  ----KGVCTMTIN--------------NDSMQCVFSNLGIQCVKKRDVEDALKLREEIRV-DPFQT----  124
_XP_045581920.1_    78  ----KGVCTMTIN--------------NDSMQCVFSNLGIQCVKKRDVEDALKLREEIRV-DPFQT----  124
_XP_050704598.1_    78  ----KGVCTMTIN--------------NDSMQCVFSNLGIQCVKKRDVDDALKLREEIRV-DPFQT----  124
_XP_027237995.1_    78  ----KGVCTMTIN--------------NDSMQCVFSNLGIQCVKKRDVEDALKLREEIRV-DPFQT----  124
_AXB88326.1_        78  ----KGVCTMTIN--------------NDSMQCVFSNRGIQCVKKRDVEDALKLREEIRV-DPFQT----  124
_XP_045102712.1_    78  ----KGVCTMTIN--------------NDSMQCVFSNLGIQCVKKRDVEDALKLREEIRV-DPFQT----  124
_MPC17461.1_        78  ----KGVCTMTIN--------------NDSMQCVFSNLGIQCVKKRDVEDALKLREEIRV-DPFQT----  124
_XP_027237994.1_    78  ----KGVCTMTIN--------------NDSMQCVFSNLGIQCVKKRDVEDALKLREEIRV-DPFQT----  124
_XP_027237996.1_    78  ----KGVCTMTIN--------------NDSMQCVFSNLGIQCVKKRDVEDALKLREEIRV-DPFQT----  124
_XP_045102715.1_    78  ----KGVCTMTIN--------------NDSMQCVFSNLGIQCVKKRDVEDALKLREEIRV-DPFQT----  124
_XP_053627990.1_    78  ----KGVCTMTIN--------------NDSMQCVFSNLGIQCVKKRDVEDALRLREEIRV-DPFQT----  124
_XP_053627989.1_    78  ----KGVCTMTIN--------------NDSMQCVFSNLGIQCVKKRDVEDALRLREEIRV-DPFQT----  124
_XP_053627983.1_    78  ----KGVCTMTIN--------------NDSMQCVFSNLGIQCVKKRDVEDALRLREEIRV-DPFQT----  124
_KAB7495058.1_      78  ----KGVCTITID--------------NDSMECMFSNLGIQCVKKKDVEQALKLREEIRV-DPFNT----  124
_KAF2353654.1_      78  ----KGVCTMTIN--------------NESMQCVFSNLGIQCVKRKDVEEALRLREEIRV-DPFQT----  124
_ANH22235.1_        78  ----KGICTMTIS--------------SDTMQCMFSNLGIQCIKKRDVEDALKLREEIRV-DPFQT----  124
_CAD7275741.1_      77  ----KGVCTMYID--------------PNTMKCQFQNLGIQCVRKRDIAEALDQRQQIKV-DPFQT----  123
_XP_042243403.1_    78  ----KGVCTMTIN--------------PDSMQCVFSNLGIQCVKKRDVEDALKLREEIRV-DPFQT----  124
_QTC09942.1_        78  ----KGVCTMTIN--------------NDSMQCVFSNLGIQCVKKRDVEDALKLREEIRV-DPFQT----  124
_XP_018023738.1_    68  ---GIQVRTIGIQ--------------VRTIGIQVRTIGIQ-VRSIGIQGACGS----------------  103
_XP_019877552.1_    79  ----RGVCTLEIP--------------PETMSVSFSNLGIQCVKKKDIEEALRVREEIRV-DPFKT----  125
_XP_049825875.1_    79  ----KGVCTVKIP--------------PDTMTVSFPNLGIQCVKRKDVEESLRIREEIRV-DPFKT----  125
_CAD7649074.1_      78  ----KGVCTLVIN--------------NHDMICSFTSLGIQCVKRKDIEDSLKLRESIKV-DPYRT----  124
_CAD7630012.1_      78  ----KGVCTMVIN--------------NHDMIQSFTSLGIQCVKRKDIEDSLKLRESIKV-DPYRT----  124
_XP_054165343.1_    79  ----KGVCTMVIN--------------NQDMICSFTSLGIQCVKRKDIEDSLKLRESIKV-DPFRT----  125
_XP_044254219.1_    75  ----RGVCSFEAEV------------TPNNPDITFRKLGVQCVKKNELKESLSVREESNI-DPFST----  123
_XP_053201827.1_    78  ----KGVCTMVIN--------------NPDMICSFTSLGIQCVKRKDIEDSLKLRESIKV-DPFRT----  124
_XP_015786990.1_    78  ----KGVCTMVIN--------------NSDMICSFTSLGIQCVKRKDIEESLKLRESIKV-DPFRT----  124
_KAI1295507.1_      78  ----KGVCTMVIN--------------NPEMICSFTSLGIQCVKRKDVEESLKLRESIKV-DPFRT----  124
_RWS07527.1_        78  ----KGVCTMVIT--------------NPDMICSFTSLGIQCVKRKDIEESLKLRESIKV-DPFRT----  124
_RWS29859.1_        78  ----KGVCTMVIN--------------NPDMLCSFTSLGIQCVKRKDIEESLKLREAIKV-DPFRT----  124
```

```
_XP_053201825.1_    78   ----KGVCTMVIN--------------NPDMICSFTSLGIQCVKRKDIEDSLKLRESIKV-DPFRT----   124
_XP_054713467.1_    85   ----NGFCSLQ----------------SESDIVSFSNIVIERVKEDEIAASLETRRKFRI-DPYGM----   129
_XP_046915158.1_    78   ----KGVCTLVID-------------NNAEMTCTFTSLGIQCVKRNKIEESLKLREMIKV-DPFRT----   125
_KAJ6217218.1_      78   ----KGVCTLVID-------------NNPDMTCTFTSLGIQCVKRKDIEESLKLRELIKV-DPYRT----   125
_KAI2801352.1_      78   ----KGVCTLVID-------------NNPDMTCTFTSLGIQCVKRKDIEESLKLRELIKV-DPYRT----   125
_KAH9406651.1_      78   ----KGVCTIVID-------------NNAEMTCTFTSLGIQCVKRKDIEESLKLREVIKV-DPFRT----   125
_XP_017483538.1_    78   ----KGVCTIVID-------------NNAEMTCTFTSLGIQCVKRKDIEESLKLREVIKV-DPFRT----   125
_KPM10359.1_        78   ----KGVCTLVID-------------NNVDMICTFTSLGIQCVKRNKIEESLKLREMIKV-DPFRT----   125
_UXI17582.1_        78   ----KGVCTLVID-------------NNVDMICTFTSLGIQCVKRNKIEESLKLREMIKV-DPFRT----   125
_KAF7488016.1_      78   ----KGVCTLVID-------------NNVDMICTFTSLGIQCVKRNKIEESLKLREMIKV-DPFRT----   125
_KAH9423377.1_      78   ----KGVCTLVID-------------NNPDMICTFTSLGIQCVKRNKIEESLKLREMIKV-DPFRT----   125
_OTF73464.1_        78   ----KGVCTLVID-------------NNPEMTCTFTSLGIQCVKRNKIEESLKLREMIKV-DPFRT----   125
_XP_037072056.1_    78   ----KGVCTVNLN--------------VETMSVTFQNLGIQCVKKKDVEDSLRQREAIRV-DPFQT----   124
_XP_037083492.1_    78   ----KGVCTVNLN--------------VETMSVTFQNLGIQCVKKKDVEESLRLRESIRV-DPFQT----   124
_XP_037083508.1_    78   ----KGVCTVNLN--------------VETMSVTFQNLGIQCVKKKDVEESLRLRESIRV-DPFQT----   124
_XP_043215018.1_    78   ----KGVCTVNLN--------------VETMSVTFQNLGIQCVKKKDVEESLRLRESIRV-DPFQT----   124
_KAF0314765.1_      78   ----KGVCTVNLN--------------VETMSVTFQNLGIQCVKKKDVEESLRLRESIRV-DPFQT----   124
_XP_043215020.1_    78   ----KGVCTVNLN--------------VETMSVTFQNLGIQCVKKKDVEESLRLRESIRV-DPFQT----   124
_XP_043189654.1_    78   ----KGVCTVNLN--------------VETMSVTFQNLGIQCVKKKDVEDSLRQREAILV-DPFRT----   124
_KAF0293344.1_      48   ----KGVCTVNLN--------------VETMSVTFQNLGIQCVKKKDVEDSLRQREAILV-DPIQKVEGG    98
_XP_011496609.1_    78   ---TNGIYSFKTI--------------SEEMTITFNNLGIQCVKRKDIQKSLTVRRSLKI-DPFNT----   125
_AGS12619.1_        78   ----KGVCTLEIS--------------PETMQISFQSLGIQCVKKKEIEEALALRQQIRV-DPFQT----   124
_TRY78048.1_        78   ----KGVCTLEIS--------------PETMQISFQSLGIQCVKKKEIEEALTLRQQIRV-DPFQT----   124
_XP_040576307.1_    78   ----KGVCTLEIN--------------PESMACSFPSLGIQCVKKKEIEESLNLRQQIRV-DPFQT----   124
_CAB4056363.1_      78   ----KGVCTLEIN--------------PESMACSFPSLGIQCVKKKEIEESLNLRQQIRV-DPFQT----   124
_ALF84898.1_        78   ----KGVCTIEMD--------------SETMTCSFPSLGIQCVKKKEIEESLKLRQQIRV-DPFQT----   124
_XP_023349538.1_    75   ----KGVCTLELG---------------ADMTCSFPSLGIQCVKKKDIEESLNLRQQIRV-DPFQT----   120
_XP_004522582.1_    78   ----KGVCTVEIN--------------TDTMCAVFKNLGIQCVKKRDIEQSLRIRENIRV-DPFRT----   124
_CAD7013622.1_      78   ----KGVCTVEIN--------------TDTMCAVFKNLGIQCVKKRDIEQSLRIRENIRV-DPFRT----   124
_XP_042902217.1_    81   ----RGVCTQLVN--------------NPDMTCSFPSLGIQCVKKRDIEKSLKIREKHSI-DPFGT----   127
_XP_042902218.1_    81   ----RGVCTQLVN--------------NPDMTCSFPSLGIQCVKKRDIEKSLKIREKHSI-DPFGT----   127
_XP_035215470.1_    81   ----RGVCTLLVN--------------NADMTCSFTSLGIQCVKKRDIEKSLKVREAHKL-DPFGT----   127
_GBN14612.1_        80   ----RGVCTLLVN--------------NADMTCSFTSLGIQCVKKRDIEKSLKVREAHKL-DPFGT----   126
_KAF8795965.1_      81   ----RGVCTLLVN--------------NADMTCSFTSLGIQCVKKRDIEKSLKVREAHKL-DPFGT----   127
_KAF8795964.1_      81   ----RGVCTLLVN--------------NADMTCSFTSLGIQCVKKRDIEKSLKVREAHKL-DPFGT----   127
_GFY66827.1_        81   ----RGVCTLLVN--------------NADMTCSFTSLGIQCVKKRDIEKSLKVREAHKL-DPFAT----   127
_GFW66965.1_        81   ----RGVCTLLVN--------------NADMTCSFTSLGIQCVKKRDIEKSLKVREAHKL-DPFGT----   127
_GFQ85823.1_        81   ----RGVCTLLVN--------------NADMTCSFTSLGIQCVKKRDIEKSLKVREAHKL-DPFGT----   127
_GFT35953.1_        81   ----RGVCTLLVN--------------NADMTCSFTSLGIQCVKKRDIEKSLKVREAHKL-DPFGT----   127
```

```
_XP_054714028.1_   81  ----RGVCTLLVN--------------NPDMTCSFTSLGIQCVKKRDIDKSLKVREAHKL-DPFGT----  127
_KAG8194583.1_     80  ----RGVCTLLVN--------------NADMICSFTSLGIQCVKKRDIEKSLKVREAHNL-DPFNT----  126
_GIY09899.1_       81  ----RGVCTLLVN--------------NVDMTCSFTSLGIQCVKKRDIEKSLKIREAHKL-DPFGT----  127
_GIY93593.1_       81  ----RGVCTLLVN--------------NVDMTCSFTSLGIQCVKKRDIEKSLKIREAHKL-DPFGT----  127
_XP_042902221.1_   89  DEERRGIYCKIIK--------------NSTMTCSFPNLGILSVKKQDIPKSLKLREKYLV-DPFGT----  139
_XP_046962867.1_   78  ----NGVCTVKTTI------------SEENPQVSFSNLGIQCVKRKDIADALKTRERLRV-DPFRT----  126
_XP_047530540.1_   78  ----NGVCTVKTTI------------NEENPQVSFSNLGIQCVKRKDIADALKTRERLRV-DPFRT----  126
_XP_026486466.1_   78  ----NGVCTVKTTI------------NEENPQVSFSNLGIQCVKRKDIADALKTRERLRV-DPFRT----  126
_XP_047530539.1_   78  ----NGVCTVKTTI------------NEENPQVSFSNLGIQCVKRKDIADALKTRERLRV-DPFRT----  126
_XP_047530538.1_   78  ----NGVCTVKTTI------------NEENPQVSFSNLGIQCVKRKDIADALKTRERLRV-DPFRT----  126
_XP_047530537.1_   78  ----NGVCTVKTTI------------NEENPQVSFSNLGIQCVKRKDIADALKTRERLRV-DPFRT----  126
_XP_026486464.1_   78  ----NGVCTVKTTI------------NEENPQVSFSNLGIQCVKRKDIADALKTRERLRV-DPFRT----  126
_XP_047530541.1_   78  ----NGVCTVKTTI------------NEENPQVSFSNLGIQCVKRKDIADALKTRERLRV-DPFRT----  126
_XP_050347286.1_   78  ----NGVCTFKTSI------------TEENPQVSFSNLGIQCVKRKDIADALKVREGLRV-DPFRT----  126
_XP_050347283.1_   78  ----NGVCTFKTSI------------TEENPQVSFSNLGIQCVKRKDIADALKVREGLRV-DPFRT----  126
_XP_032515351.1_   78  ---ENGVCTVKRSI------------SEESPQVSFSNLGIQCVKRKDIAEALKTRERLRV-DPFKT----  127
_OWR46016.1_       78  ---ENGVCTVKRSI------------SEESPQVSFSNLGIQCVKRKDIAEALKTRERLRV-DPFKT----  127
_XP_032515346.1_   78  ---ENGVCTVKRSI------------SEESPQVSFSNLGIQCVKRKDIAEALKTRERLRV-DPFKT----  127
_CAG9579094.1_     78  ---ENGVCTVKRSI------------SEESPQVSFSNLGIQCVKRKDIAEALKTRERLRV-DPFKT----  127
_CAG9579091.1_     78  ---ENGVCTVKRSI------------SEESPQVSFSNLGIQCVKRKDIAEALKTRERLRV-DPFKT----  127
_XP_050668638.1_   78  ----KGVCTVKTT-------------LMDDNKVSFSNLGIQCVKRKDIAEALKTREQLKV-DPFKT----  125
_XP_050668632.1_   78  ----KGVCTVKTT-------------LMDDNKVSFSNLGIQCVKRKDIAEALKTREQLKV-DPFKT----  125
_XP_045495329.1_   78  ----KGVCTVRTT-------------VGDDSRVSFSNLGIQCVKRKDIADALKMREQLRV-DPFKT----  125
_XP_038214802.1_   78  ----KGVCTVRTS-------------VGDDSRVSFSNLGIQCVKRKDIADALKTREQLRV-DPFKT----  125
_CAH0714714.1_     78  ----NGVCTLKTTI------------TDENPQVSFSNLGIQCVKRKDITDALKTREKLRV-DPFKT----  126
_CAH0714713.1_     78  ----NGVCTLKTTI------------TDENPQVSFSNLGIQCVKRKDITDALKTREKLRV-DPFKT----  126
_CAH4029256.1_     78  ----KGVCTVRTN-------------INDDNRISFSNLGIQCVKRKDVADALAVREKLRV-DPFKT----  125
_XP_047513081.1_   78  ----KGVCTVRTN-------------ISDDNRISFSNLGIQCVKRKDVADALAVREKLRV-DPFKT----  125
_XP_045529569.1_   78  ----KGVCTVRTN-------------INDDNRISFSNLGIQCVKRKDVADALAVREKLRV-DPFKT----  125
_XP_045487500.1_   78  ----KGVCTVRTN-------------ITDDNRISFSNLGIQCVKRKDVADALAVREKLRV-DPFKT----  125
_XP_047513077.1_   78  ----KGVCTVRTN-------------ISDDNRISFSNLGIQCVKRKDVADALAVREKLRV-DPFKT----  125
_CAH4029257.1_     78  ----KGVCTVRTN-------------INDDNRISFSNLGIQCVKRKDVADALAVREKLRV-DPFKT----  125
_XP_045487498.1_   78  ----KGVCTVRTN-------------ITDDNRISFSNLGIQCVKRKDVADALAVREKLRV-DPFKT----  125
_CAF4952989.1_     78  ----KGVCTVRTN-------------ISDDNRISFSNLGIQCVKRKDVADALAVREKLRV-DPFKT----  125
_XP_045447470.1_   78  ----NGVCTVKTNV------------TDENPQVSFSNLGIQCVKRKDIAEALKTRERLRV-DPFRT----  126
_CAH2108688.1_     17  ----RGVCTVRTNV------------TDENPQISFSNLGIQCVKRKDIAEALRTREELRV-DPFRT----   65
_CAH0714724.1_     77  ----NGIYEFNVQL------------KKENARIVVPNLGIQHTLQRDVVKELMARKKTGV-DPFQT----  125
_XP_034827218.1_   78  ----EGVCTVKTTI------------TEDNPQVSFSNLGIQCVKRKDIANALKTRERLRV-DPFKT----  126
_XP_045768234.1_   78  ----EGVCTVKTTI------------TEDNPQVSFSNLGIQCVKRKDIANALKTRERLRV-DPFKT----  126
```

```
_XP_034827214.1_   78  ----EGVCTVKTTI------------TEDNPQVSFSNLGIQCVKRKDIANALKTRERLRV-DPFKT----  126
_XP_045768230.1_   78  ----EGVCTVKTTI------------TEDNPQVSFSNLGIQCVKRKDIANALKTRERLRV-DPFKT----  126
_XP_039751073.1_   78  ----EGVCTVKTTI------------TEDSPQVSFSNLGIQCVKRKDIANALKTRERLRV-DPFRT----  126
_XP_023942270.1_   78  ----EGVCTVKTTI------------TEENPQVSFSNLGIQCVKRKDIASALKTRERLRV-DPFRT----  126
_XP_023942268.1_   78  ----EGVCTVKTTI------------TEENPQVSFSNLGIQCVKRKDIASALKTRERLRV-DPFRT----  126
_XP_039751072.1_   78  ----EGVCTVKTTI------------TEDSPQVSFSNLGIQCVKRKDIANALKTRERLRV-DPFRT----  126
_XP_023942266.1_   78  ----EGVCTVKTTI------------TEENPQVSFSNLGIQCVKRKDIASALKTRERLRV-DPFRT----  126
_CAH2240541.1_     65  ----EGVCTVKTTI------------TEDSPQVSFSNLGIQCVKRKDIANALKTRERLRV-DPFRT----  113
_XP_041985045.1_   78  ----NGVCTLKINV------------TEENPQVSFSNLGIQCVKRKDIGEALNNRERLKV-DPFKT----  126
_XP_041985024.1_   78  ----NGVCTLKINV------------TEENPQVSFSNLGIQCVKRKDIGEALNNRERLKV-DPFKT----  126
_XP_049868286.1_   78  ----RGVCTQKIDV------------TPDNALVTFSNLGIQCVKRRDIADALRVREELRV-DPFRT----  126
_XP_026314794.1_   78  ----RGVCTQRVDV------------TADNRLVTFSNLGIQCVKRRDIAEALRVREELRV-DPFRT----  126
_XP_047994271.1_   78  ---NRGVCT-VEC-------------NADTSVVQFSNLGIQCVKRKDIAEALRVREEIKV-DPFKT----  125
_KAJ2950301.1_     78  ----KGVCTQKVDV------------TPENNRFAFSNLGIQCVKRRDIADALKVREELRV-DPFRT----  126
_XP_031784347.2_   77  ---SQGICH-FQI-------------PAGQSIVSFDNIGIQRVKKKDMAKSLEERQRLRV-DPFKT----  124
_KAG7305875.1_     78  ----KGVCTMNAP--------------ADSAVVTFTNLGIQCVKRRDIEDALAVREEMRV-DPFKT----  124
_WDD23886.1_       78  ----KGVCTMNAP--------------ADSAVVTFTNLGIQCVKRRDIEDALAVREEMRV-DPFKT----  124
_XP_048480424.1_   78  ----KGVCTMNAP--------------ADSAVVTFTNLGIQCVKRRDIEDALAVREEMRV-DPFKT----  124
_XP_048480423.1_   78  ----KGVCTMNAP--------------ADSAVVTFTNLGIQCVKRRDIEDALAVREEMRV-DPFKT----  124
_XP_048480422.1_   78  ----KGVCTMNAP--------------ADSAVVTFTNLGIQCVKRRDIEDALAVREEMRV-DPFKT----  124
_XP_048480421.1_   78  ----KGVCTMNAP--------------ADSAVVTFTNLGIQCVKRRDIEDALAVREEMRV-DPFKT----  124
_KAG7305876.1_     78  ----KGVCTMNAP--------------ADSAVVTFTNLGIQCVKRRDIEDALAVREEMRV-DPFKT----  124
_CAG9129242.1_     78  ----KGVCTMNAP--------------ADSAVVTFTNLGIQCVKRRDIEDALAVREEMRV-DPFKT----  124
_XP_048480420.1_   78  ----KGVCTMNAP--------------ADSAVVTFTNLGIQCVKRRDIEDALAVREEMRV-DPFKT----  124
_GBP53004.1_           ----------------------------------------------------------------------
_XP_045534737.1_   78  ----RGVCTVKAEL------------SGDSTLVSFSNLGIQCVKRKDVDAALRTREELRV-DPFKT----  126
_XP_013180060.1_   78  ----RGVCTVKAEL------------SGDSTLVSFSNLGIQCVKRKDVDAALRTREELRV-DPFKT----  126
_XP_013180043.1_   78  ----RGVCTVKAEL------------SGDSTLVSFSNLGIQCVKRKDVDAALRTREELRV-DPFKT----  126
_XP_013180068.1_   78  ----RGVCTVKAEL------------SGDSTLVSFSNLGIQCVKRKDVDAALRTREELRV-DPFKT----  126
_XP_045534736.1_   78  ----RGVCTVKAEL------------SGDSTLVSFSNLGIQCVKRKDVDAALRTREELRV-DPFKT----  126
_KPJ06840.1_       78  ----RGVCTVKAEL------------SGDSTLVSFSNLGIQCVKRKDVDAALRTREELRV-DPFKT----  126
_KPJ05518.1_       78  ----RGVCTVKAEL------------SGDSTLVSFSNLGIQCVKRKDVDAALRTREELRV-DPFKT----  126
_CAG4934519.1_     78  ----KGVCTVKAEL------------TGDSTLVSFSNLGIQCVKRKDVDAALRTREELRV-DPFKT----  126
_XP_013149112.1_   78  ----RGVCTVKAEL------------SGDSTLVSFSNLGIQCVKRKDVDTALRTREELRV-DPFKT----  126
_CAH2077165.1_     78  ----RGVCTVRADLGNGGSGGNGGSGGSGGTLVAFSNLGIQCVKRKDIDAALRTREELRV-DPFKT----  138
_CAH2077167.1_     78  ----RGVCTVRADLGNGGSGGNGGSGGSGGTLVAFSNLGIQCVKRKDIDAALRTREELRV-DPFKT----  138
_XP_037051415.1_   72  ----RGVCTVSFKT-----------DESDPTVYSFNNMAIQCIKKGDIEQVLTERQKCNV-DPFKT----  121
_XP_037051414.1_   73  ----RGVCTVVFKT-----------DESDPTVYLFNNMAIQCIKKGDMEQVLTEREKCNV-DPFKT----  122
_XP_022815079.1_   78  ----RGVCTIPLRV------------TEETCEYQFKNLGIQCVKRRDIAEALTIREKLRV-DPFRK----  126
```

```
_CAB3516522.1_      78  ----RGVCTIPLRV------------TEETCEYQFKNLGIQCVKRRDIAEALTIREKLRV-DPFRK----  126
_XP_050562120.1_    78  ----RGVCTIPLRV------------TEETCEYQFKNLGIQCVKRRDIAEALTTREKLRV-DPFRK----  126
_XP_050562121.1_    78  ----RGVCTIPLRV------------TEETCEYQFKNLGIQCVKRRDIAEALTTREKLRV-DPFRK----  126
_QHD56218.1_        78  ----RGVCTIPLRV------------TEETCEYQFKNLGIQCVKRRDIAEALTIREKLRV-DPFRK----  126
_AIA24466.1_        78  ----RGVCTIPLRV------------TEETCEYQFKNLGIQCVKRRDVAEALTIREKLRV-DPFRK----  126
_XP_022815076.1_    78  ----RGVCTIPLRV------------TEETCEYQFKNLGIQCVKRRDIAEALTIREKLRV-DPFRK----  126
_QHD56217.1_        78  ----RGVCTIPLRV------------TEETCEYQFKNLGIQCVKRRDIAEALTIREKLRV-DPFRK----  126
_XP_050562118.1_    78  ----RGVCTIPLRV------------TEETCEYQFKNLGIQCVKRRDIAEALTTREKLRV-DPFRK----  126
_AIA24467.1_        78  ----RGVCTIPLRV------------TEETCEYQFKNLGIQCVKRRDVAEALTIREKLRV-DPFRK----  126
_XP_047019808.1_    78  ----RGVCTIPTLI------------TKETCEYQFKNLGIQCVKRRDIAEALTVREKLRV-DPFRK----  126
_AEO51736.1_        78  ----RGVCTIPTLI------------TKETCEYQFKNLGIQCVKRRDIAEALTVREKLRA-DPFRK----  126
_XP_049696680.1_    78  ----RGVCTIPTLI------------TKETCEYQFKNLGIQCVKRRDIAEALTVREKLRV-DPFRK----  126
_XP_049696682.1_    78  ----RGVCTIPTLI------------TKETCEYQFKNLGIQCVKRRDIAEALTVREKLRV-DPFRK----  126
_XP_049696676.1_    78  ----RGVCTIPTLI------------TKETCEYQFKNLGIQCVKRRDIAEALTVREKLRV-DPFRK----  126
_XP_026744931.1_    78  ----RGVCTIPLRI------------TEDSCEHQFKNLGIQCVKRRDIADALTVREKLRV-DPFRK----  126
_XP_026732895.1_    78  ----RGVCTIPLRI------------TEDSCEHQFKNLGIQCVKRRDIADALTVREKLRV-DPFRK----  126
_CAH0581650.1_      78  ----RGVCTIPLRI------------TEETCEHQFKNLGIQCVKRRDIADALTVREKLRV-DPFRK----  126
_KAH9640014.1_      78  ----RGVCTIPLRV------------TEETCEYQFKNLGIQCVKRRDIAEALTIREKLRV-DPFRSKLFL  130
_PZC78315.1_            ----------------------------------------------------------------------
_KAF9795796.1_          ----------------------------------------------------------------------
_AEC52926.1_        78  ----RGVCTIRTEI------------TEENNEYQFRNLGIQCVKRRDIAEALRIREELRV-DPFRT----  126
_KAH7940301.1_      78  ----NGICTQHL---------------KPDMTCTFTSLGIQCVKRRDVEQNLLQRENIRV-DPFRN----  123
_ADK39025.1_        77  ----RGVFSVRIEI------------TDENNEYQFRNLGIQCVKRRDIGEALRIREDLRV-DPFKT----  125
_XP_037298148.1_    77  ----RGVFSVRTEI------------TDENNEYQFRNLGIQCVKRRDIGEALRIREDLRV-DPFKT----  125
_RVE50742.1_        76  ----RGVCTVKVHTE----------GDADAFHVQFKNLGIQCVKKRDITEALRVRQEMRV-NPFKT----  126
_CAH0692665.1_      76  ----RGVCTVKVHTE----------GDADAFHVQFKNLGIQCVKKRDITEALRVRQEMRV-NPFKT----  126
_XP_028166351.1_    75  ----MGVCTVKVRTE----------GDADACQVQFKNLGIQCVKRRDIADALRTRQELRV-NPFKT----  125
_XP_028166291.1_    75  ----MGVCTVKVRTE----------GDADACQVQFKNLGIQCVKRRDIADALRTRQELRV-NPFKT----  125
_CAB3235314.1_      78  ----RGVCTVPYRI------------TEESNEYQFKNLGIQCVKRRDIADALRVRQTLRV-DPFRT----  126
_KAJ0175867.1_      79  ----RGVCTVRTEI------------TEENNEYQFRNLGIQCVKRRDIGEALKAREELRV-DPFRT----  127
_XP_028031027.1_    78  ----RGVCSVRTDV------------TEDNNEYQFRNLGIQCVKRKDIVAELRLREELRV-DPFRT----  126
_NP_001166296.1_    78  ----RGVCSVRTDV------------TEDNNEYQFRNLGIQCVKRKDIVAELRLREELRV-DPFRT----  126
_XP_037870194.1_    78  ----RGVCSVRTDV------------TEDNNEYQFRNLGIQCVKRKDIVAELRLREELRV-DPFRT----  126
_XP_037870193.1_    78  ----RGVCSVRTDV------------TEDNNEYQFRNLGIQCVKRKDIVAELRLREELRV-DPFRT----  126
_XP_028031025.1_    78  ----RGVCSVRTDV------------TEDNNEYQFRNLGIQCVKRKDIVAELRLREELRV-DPFRT----  126
_XP_013187225.1_    76  ----RGVCTVRETI------------TDENCRVAFKNLGIQCVKRRDIAEALRVREELRV-DPFKT----  124
_XP_052755011.1_    76  ----RGVCTVRETI------------DSDSCQVQFKNLGIQCIKRRDIAEALRVREELRV-DPFRT----  124
_XP_053613182.1_    76  ----RGVCTVRETI------------TEENCQVAFKNLGIQCVKRRDIAEALRVREELRV-DPFKT----  124
_XP_053613180.1_    76  ----RGVCTVRETI------------TEENCQVAFKNLGIQCVKRRDIAEALRVREELRV-DPFKT----  124
```

```
_CAG9785863.1_      76  ----KGVCTVKVKTE----------GDADSCQVQFKNLGIQCVKRRDIADALRVRQELRV-NPFK-----  125
_KAG6461275.1_      77  ----RGVFSVRTEI------------TDENNEYQFRNLGIQCVKRRDIGEALRIREDLRV-DPFKT----  125
_KAG6461281.1_      77  ----RGVFSVRTEI------------TDENNEYQFRNLGIQCVKRRDIGEALRIREDLRV-DPFKT----  125
_XP_037302137.1_    76  ----DGVFIVCTEI------------TDENNEYQFRNLGIQCVKRCEVDEALCIREAMEI-DPFST----  124
_KAG6461269.1_      76  ----DGVFIVCTEI------------TDENNEYQFRNLGIQCVKRCEVDEALCIREAMEI-DPFST----  124
_KAG4069896.1_      71  ----RGVCTISFKT-----------DESDPTVNTFTNMAIQCIKKVDIEKVLTEREKCNV-DPFKT----  120
_XP_044003989.1_    79  -ECVNGIVYFNLT--------------PDNPVMEFKKIGIQSCKKNEVDQFIRAKMLMGH-DPCNA----  128
_XP_045768240.1_    78  ----NGVCIVKITI------------AEDNRIVSFNNMGIQCVKRKGIAKSLNTRRWLRV-DPFRT----  126
_KAG9509099.1_      78  ----NGICTMVLNGNN--------DGENKDMICTFTNLGIQCVKRKEIEESLKLRESKQV-DPFRT----  130
_XP_013118267.1_    78  ----HGVCTMRLK--------------GAPMRAVFSNLGIQCVKKKDIKAALEERERINI-DPFKT----  124
_XP_005183026.2_    78  ----RGVCTMRLK--------------GPPMRAVFSNLGIQCVKKKEIKSSLEERERINV-DPFKT----  124
_XP_050303432.1_    85  ----NGVFRTNVS-------------IGTSAIIELRYMGIVRVRKGDVKSWLEERERINV-DPYKN----  132
_TMW48950.1_        78  ----HGVCLATIN--------------GPPMRAVFSNLGIQCIRKKDIKDALEERERKRI-DPFKT----  124
_XP_037882085.1_    78  ----YGICTMRVK--------------GDPPRAVFSNLGVQCVRKKDIKAALELRENRNV-DPFKT----  124
_KAI8126541.1_      79  ----YGVCLATIN--------------GPPMRAVFSNLGIQCVKKKDIKAALEERERKRI-DPFKT----  125
_KAI8126540.1_      78  ----YGVCLATIN--------------GPPMRAVFSNLGIQCVKKKDIKAALEERERKRI-DPFKT----  124
_XP_037811156.1_    78  ----YGVCLATIN--------------GPPMRAVFSNLGIQCVKKKDIKAALEERERKRI-DPFKT----  124
_XP_046812595.1_    78  ----YGVCLATIN--------------GPPMRAVFSNLGIQCVKKKDIKAALEERERKRI-DPFKT----  124
_XP_046812596.1_    78  ----YGVCLATIN--------------GPPMRAVFSNLGIQCVKKKDIKAALEERERKRI-DPFKT----  124
_XP_046812591.1_    78  ----YGVCLATIN--------------GPPMRAVFSNLGIQCVKKKDIKAALEERERKRI-DPFKT----  124
_XP_046812590.1_    78  ----YGVCLATIN--------------GPPMRAVFSNLGIQCVKKKDIKAALEERERKRI-DPFKT----  124
_KNC34143.1_        78  ----YGVCLATIN--------------GPPMRAVFSNLGIQCVKKKDIKAALEERERKRI-DPFKT----  124
_XP_046812589.1_    78  ----YGVCLATIN--------------GPPMRAVFSNLGIQCVKKKDIKAALEERERKRI-DPFKT----  124
_KAI8126539.1_      78  ----YGVCLATIN--------------GPPMRAVFSNLGIQCVKKKDIKAALEERERKRI-DPFKT----  124
_XP_037958917.1_    78  ----DGICTLVLT--------------GETRRAVFSNLGVQCVKKSEIKSALEVRERLQV-DPFRT----  124
_XP_037929595.1_    80  ----DGICTLVLS--------------GEPLQAVFSNLGIQCVKKNDIKAALEVRERLQV-DPFKT----  126
_XP_037957513.1_    78  ----DGICTLVLS--------------GEPLRAVFSNLGIQCVKKNDIKAALEVRERLQV-DPFKT----  124
_XP_037958115.1_    80  ----DGICTLVLS--------------GEPLQAVFSNLGIQCVKKNDIKAALEVRERLQV-DPFKT----  126
_XP_037958114.1_    80  ----DGICTLVLS--------------GEPLQAVFSNLGIQCVKKNDIKAALEVRERLQV-DPFKT----  126
_XP_037957511.1_    78  ----DGICTLVLS--------------GEPLRAVFSNLGIQCVKKNDIKAALEVRERLQV-DPFKT----  124
_XP_037958918.1_    78  ----DGICTLVLT--------------GETRRAVFSNLGVQCVKKSEIKSALEVRERLQV-DPFRT----  124
_RXG71823.1_        78  ----DGICSVLID--------------NAMMECSFSNLGIQCVKKRDIESSLAKRAAKMI-DPYH-----  123
_KAB7494622.1_      35  ----DGICSVLID--------------NAMMECSFSNLGIHNFMYISAGRTSIILVFCIF-SGF------   79
_KAI9584339.1_      77  ----HGVCKKVFS--------------TEQMPLAFKNLSVQYVKKKNIESALKEREAMRI-DPFRT----  123
_XP_037884983.1_    77  ----HGVCKKVFS--------------TEQMPLAFQNLSVRHVKKKNIESVLTEREALRI-DPFRT----  123
_XP_054744821.1_    77  ----SGVCTKNVG-------------PGLPLRAEFSNIGIQCVRKKEISESLEERKKKKV-DPFKT----  124
_XP_053961676.1_    77  ----SGVCTKNVG-------------PGLPLRAEFSNIGIQCVRKKEISESLEERKKKKV-DPFKT----  124
_XP_053961674.1_    77  ----SGVCTKNVG-------------PGLPLRAEFSNIGIQCVRKKEISESLEERKKKKV-DPFKT----  124
_XP_004526634.2_    77  ----SGVCTKNVG-------------PGSPLRAEFSNIGIQCVRKKEIAESLEERKKKKV-DPFKT----  124
```

```
_CAD6998451.1_      77  ----SGVCTKNVG-------------PGSPLRAEFSNIGIQCVRKKEIAESLEERKKKKV-DPFKT----  124
_XP_012157894.1_    77  ----SGVCTKNVG-------------PGSPLRAEFSNIGIQCVRKKEIAESLEERKKKKV-DPFKT----  124
_XP_028894493.1_    77  ----SGVCTRNVG-------------PGAALRVEFSNIGIQCVRKKEISESLEERKKKKI-DPFKT----  124
_XP_036327693.1_    77  ----SGVCTRNVG-------------PGLPLRAEFSNVGIQCVRKKEIAESLEERKKKKV-DPFKT----  124
_XP_017471333.1_    77  ----SGVCTRNVG-------------PGLPLRAEFSNVGIQCVRKKEIAESLEERKKKKV-DPFKT----  124
_XP_039952608.1_    77  ----HGYCIRYVG-------------PDTPLRVELSNIGIRCVKKKEISESLEMRKSRKI-DPFKT----  124
_XP_050320023.1_    77  ----HGYCIRYVG-------------PDTPLRVELSNIGIRCVKKKEISESLEMRKSRKI-DPFKT----  124
_XP_036225572.1_    77  ----HGYCIRYVG-------------PDTPLRVELSNIGIRCVKKKEISESLEKRKNRKI-DPFKT----  124
_XP_039952607.1_    77  ----HGYCIRYVG-------------PDTPLRVELSNIGIRCVKKKEISESLEMRKSRKI-DPFKT----  124
_XP_036225564.1_    77  ----HGYCIRYVG-------------PDTPLRVELSNIGIRCVKKKEISESLEKRKNRKI-DPFKT----  124
_XP_036225569.1_    77  ----HGYCIRYVG-------------PDTPLRVELSNIGIRCVKKKEISESLEKRKNRKI-DPFKT----  124
_XP_050320021.1_    77  ----HGYCIRYVG-------------PDTPLRVELSNIGIRCVKKKEISESLEMRKSRKI-DPFKT----  124
_XP_039952606.1_    77  ----HGYCIRYVG-------------PDTPLRVELSNIGIRCVKKKEISESLEMRKSRKI-DPFKT----  124
_XP_011207860.1_    77  ----HGYCIRYVG-------------PDTPLRVELSNIGIRCVKKKEISESLEMRKSRKI-DPFKT----  124
_XP_011207859.1_    77  ----HGYCIRYVG-------------PDTPLRVELSNIGIRCVKKKEISESLEMRKSRKI-DPFKT----  124
_XP_029407466.1_    77  ----HGYCIRYVG-------------PDTPLRVELSNIGIRCVKKKEISESLEMRKSRKI-DPFKT----  124
_XP_019847003.2_    77  ----HGYCIRYVG-------------PDTPLRVELSNIGIRCVKKKEISESLEMRKSRKI-DPFKT----  124
_XP_014086488.2_    77  ----HGYCIRYVG-------------PDTPLRVELSNIGIRCVKKKEISESLEKRKNRKI-DPFKT----  124
_XP_049317393.1_    77  ----HGYCIRYVG-------------PDTPLRVELSNIGIRCVKKKEISESLEMRKSRKI-DPFKT----  124
_XP_039952604.1_    77  ----HGYCIRYVG-------------PDTPLRVELSNIGIRCVKKKEISESLEMRKSRKI-DPFKT----  124
_XP_018787557.1_    77  ----HGYCIRYVG-------------PDTPLRVELSNIGIRCVKKKEITESLEMRKSRKI-DPFKT----  124
_OQR68950.1_        78  ----DGVYSNKFD--------------KSDMTATFTSLGIQCVKKTEVMGSLDERFKLRI-DPFKT----  124
_XP_003741763.1_    78  ----KGVYRNKYE--------------RSNMTAQFSTLGIQCVKKTEVKESLEQRKSLKI-DPFNT----  124
_XP_022699297.1_    78  ----DGVYSNKFD--------------KTDMTATFTSLGIQCVKKTEVLGSLDERAKLRI-DPFKT----  124
_XP_022665229.1_    78  ----DGVYSNKFD--------------KTDMTATFTSLGIQCVKKTEVLGSLDERAKLRI-DPFKT----  124
_XP_022665225.1_    78  ----DGVYSNKFD--------------KTDMTATFTSLGIQCVKKTEVLGSLDERAKLRI-DPFKT----  124
_XP_022665224.1_    78  ----DGVYSNKFD--------------KTDMTATFTSLGIQCVKKTEVLGSLDERAKLRI-DPFKT----  124
_XP_033219687.1_    78  ----NGVCSMKMN--------------LDGNAVSFSHLAIQCVKKKQVRVALETRKREKV-DPFAT----  124
_XP_033227990.1_    78  ----NGICSIKMN--------------LDGNAVSFSHLAIQCVKKKQVRDALEIRKREQV-DPFAT----  124
_XP_043466013.1_    77  ----NGIYSLKMN--------------IVDGIVTFTHLGIQCMKKKQVAAALEIRKRENV-DPYGT----  123
_XP_051172671.1_    77  ----NGIYSLKMN--------------LVDGIVTFNHLGIQCMKKKQVASALEIRKRENV-DPYGT----  123
_CAD7643228.1_      77  ----DGVCSLVFA----------------DGVCVFNNLGILCAKRKDIRERLRVREALRV-DPFRT----  121
_XP_054157157.1_    78  ----DGVCSLVFD----------------GPVCVFNNLGILCAKRKDIRDRLRLRESLRV-DPFRT----  122
_CAD7645633.1_      77  ----DGVCQLVFC----------------DGICVFNNLGILCAKRKDIRERLRVREHLRC-DPFRT----  121
_CAD7632611.1_      19  ----DGVCSLVFA----------------DGVCVFNNLGILCAKRKDIRERLRVREALRV-DPFRT----   63
_KAJ6223158.1_      76  ----RGISVTIFS----------------EPICQFTNLGILCAKRKDVKERLRMREQLRI-DPFGT----  120
_KAI2804035.1_      76  ----RGISVTIFS----------------EPICQFTNLGILCAKRKDVKERLRMREQLRI-DPFGT----  120
_KAH9398209.1_      68  ----QGVCVALFG----------------EPYAVFNNLGILCAKRKDVKERLRVREQLQI-DPFGT----  112
_GFY77286.1_        78  ----NGVCYKSIN--------------TPDMVCSFANLGIQCVKKKEIKESLSQREEIKV-DPFGT----  124
```

```
_GFQ73388.1_       78  ----NGVCYKSIN--------------TPDMVCSFANLGIQCVKKKEIKESLSQREEIKV-DPFGT----  124
_GFT25575.1_       78  ----NGVCYKSIN--------------TPDMVCSFANLGIQCVKKKDIKESLTQREEIKV-DPFGT----  124
_KAF8763655.1_     78  ----NGVCYKSIN--------------TPDMVCNFANLGIQCVKKKDIKESLSHREAIQV-DPFGT----  124
_GFX76677.1_       78  ----NGVCYKSIN--------------TPDMVCSFANLGIQCVKKKEIKESLSQREEIKV-DPFGT----  124
_XP_035207052.1_   78  ----NGVCYKPIT--------------NPDMICNFANLGIQCVKKKDIKESLSHREALQV-DPFGT----  124
_KFM74898.1_       78  ----NGVCYKPIT--------------NPDMICNFANLGIQCVKKKDIKESLSHREALQV-DPFGT----  124
_XP_035207059.1_   78  ----NGVCYKPIT--------------NPDMICNFANLGIQCVKKKDIKESLSHREALQV-DPFGT----  124
_XP_035207066.1_   78  ----NGVCYKPIT--------------NPDMICNFANLGIQCVKKKDIKESLSHREALQV-DPFGT----  124
_BBD75287.1_       78  ----NGVCYKTIN--------------SPDMVCSFANLGIQCVKKKDIKESLSHREAIQV-DPFGT----  124
_XP_042900952.1_   78  ----NGVCYKTIN--------------SPDMVCSFANLGIQCVKKKDIKESLSHREAIQV-DPFGT----  124
_KAG8201816.1_     77  ----NGVCYKSMN--------------TPDMICSFSNLGIQCVKKKDIKDSLLHRQSFKV-DPFGT----  123
_GIX87435.1_       78  ----NGVCYKSIN--------------TPDMVCNFANLGIQCVKKKDIKESLSHREAIQV-DPFGT----  124
_GBL81029.1_       78  ----NGVCYKSIN--------------TPDMVCNFANLGIQCVKKKDIKESLSHREAIQV-DPFGT----  124
_GIY01087.1_       78  ----NGVCYKSIN--------------TPDMVCNFANLGIQCVKKKDIKESLSHREAIQV-DPFGT----  124
_XP_054715436.1_   78  ----NGVCYKSIE--------------DAQAVCNFPNLGIQCVKRKDVKDSLLSRKELQI-DPFGT----  124
_XP_044576112.1_   77  ----LGYYHYKT----------------YDAVVSFQNLGIQCVKKKNILEALEKRKKNKI-DPFNT----  121
_XP_008556435.3_   76  ----RGIYTHQT----------------DAQVVYFPNLGVQCVKKKNVKEALEKREELRI-DPFDT----  120
_CAD6234446.1_     77  ----LGYYHHKT----------------YDAEVSFQNLGIQCVKKKAILAALEKRKRNKI-DPFNT----  121
_XP_044284598.1_   77  ----NGYHEAEFG--------------PERRILTFQNLGIQCVKKRELKESIISRIGKKI-NPFGV----  123
_XP_033794511.1_   77  ----DGFYESEFG--------------PERRIVSFQNLGIQCVRKKEVKDAIHFRMARKI-NPYRV----  123
_XP_033794512.1_   77  ----DGFYESEFG--------------PERRIVSFQNLGIQCVRKKEVKDAIHFRMARKI-NPYRV----  123
_XP_029449578.1_   77  ----DGYYESEFG--------------PERRIVSFQNLGIQCVRRKEVKDAIHFRMARKI-NAYGV----  123
_XP_030052048.1_   77  ----EGFYESEFG--------------PERRIVSFQNLGIQCVKKKEVKNAIHFRMSRKI-NPYRV----  123
_XP_007423639.1_   77  ----NGYYEAEFG--------------PERRILIFQNLGIQCVKKRELKESIISRIAKKI-NPFNV----  123
_XP_039203483.1_   77  ----NGYYEAEFG--------------PERRVLSFQNLGIQCVKKRELKESIISRIAKKI-NPFNV----  123
_XP_034283206.1_   77  ----NGYYEAEFG--------------PERRVLSFQNLGIQCVKKRELKESIISRIAKKI-NPFNV----  123
_XP_033001023.1_   77  ----NGFYEAEFG--------------PERRVLIFQNLGIQCVKKRELKESIISRIAKQI-NPFSV----  123
_XP_053239675.1_   77  ----NGFYEAEFG--------------PERRVLIFQNLGIQCVKKRELKESIVSRITKQI-NPFSV----  123
_XP_034966804.1_   77  ----NGFYEAEFG--------------PERRVLIFQNLGIQCVKKRELKESIISRIAKQI-NPFSV----  123
_XP_028580982.1_   66  ----NGFYEAEFG--------------PERRVLIFQNLGIQCVKKRELKESIVSRITKQI-NPFSV----  112
_XP_042302822.1_   77  ----NGYYEAEFG--------------PERRVLIFQNLGIQCVTKRKVKESIITRITKKI-NPFNV----  123
_XP_015280979.1_   66  ----NGYYEAEFG--------------QDRRILSFQNLGIQCVKKRELKESIISRITNNI-NPFNV----  112
_XP_048363544.1_   77  ----KSYYEAEFG--------------PDRRVLSYQSTGCICSWRKEIRLCMHTQISVRM-TDFQL----  123
_KAF7252806.1_     51  ----NGYHEAEFG--------------PERRILTFQNLGIQCVKKRELKESIISRIGKKI-NPFGV----   97
_XP_034958398.1_    1  ----------------------------------------------------------------TV----    2
_CAD5116312.1_     79  ----KGVCTVKLK--------------NGETTYKFQNLGIRCAKKRAVKDKLQERQQIRV-DPYGN----  125
_KAJ1519398.1_     75  ----DGVYRRRLV-------------CGDDGVLEFPGLRVIRTKRRQLADKLRLRSTYNV-DPFMT----  122
_XP_053103001.1_   77  ----NGYYEAEFG--------------PERRILIFQNLGIQCVKKRELKESIISRITKKI-NPFGV----  123
_XP_032070730.1_   77  ----NGYYEAEFG--------------PERRVLSFQNLGIQCVKKRELKESIISRIAKKI-NPFNV----  123
```

```
_XP_013922116.1_    77  ----NGYYEAEFG--------------PERRVLSFQNLGIQCVKKRELKESIISRIAKKI-NPFNV----  123
_XP_015667999.1_    77  ----NGYYEAEFG--------------PERRVLSFQNLGIQCVKKRELKESIISRIAKKI-NPFNV----  123
_XP_026551846.1_    77  ----NGYYEAEFG--------------PERRVLSFQNLGIQCVKKRELKESIISRIAKKI-NPFNV----  123
_XP_026532518.1_    77  ----NGYYEAEFG--------------PERRVLSFQNLGIQCVKKRELKESIISRIAKKI-NPFNV----  123
_KAJ7341301.1_      77  ----NGYYEAEFG--------------PDRRILCFQNLGIQCVKKRELKESIVSRISKKI-NPFNV----  123
_KAJ6667038.1_      66  ----NGYYEAEFG--------------PERKVLIFQNLGIQCVKKRELKESIISRIAKKI-NPFGV----  112
_XP_003227818.2_    66  ----NGYYEAEFG--------------PERRVLIFQNLGIQCVKKRDLKESIILRMSKKI-NPFGV----  112
_XP_020633036.1_    66  ----NGYYEAEFG--------------PDRRILCFQNLGIQCVKKRELKESIISRISKKI-NPFNV----  112
_XP_054842059.1_    77  ----HGYYEAEFG--------------PERRVLSFQNLGIQCVKKRELKESITSRITNNI-NPFSV----  123
_XP_005238641.2_    66  ----DGYYEAEFG--------------PERRVLSFQNLGIQCVKKKDLKESISSRISKKI-NPFNV----  112
_XP_005445671.2_    66  ----DGYYEAEFG--------------PERRVLSFQNLGIQCVKKKDLKESISSRISKKI-NPFNV----  112
_XP_054050029.1_    66  ----DGYYEAEFG--------------PERRVLSFQNLGIQCVKKKDLKESISLRISKKI-NPFNV----  112
_XP_013810845.1_    66  ----DGYYEAEFG--------------PERRVLSFQNLGIQCVKKRDLKESISLRISKKI-NPFNV----  112
_XP_026702002.1_    66  ----DGYYEAEFG--------------PERRVLSFQNLGIQCVKKKDLKESISLRISKKI-NPFNV----  112
_XP_025892242.1_    66  ----DGYYEAEFG--------------PERRVLSFQNLGIQCVKKKELKESISLRISRKI-NPFNV----  112
_XP_005507389.1_    66  ----DGYYEAEFG--------------PERRVLSFQNLGIQCVKKKDLKESIALRISKKI-NPFNV----  112
_XP_032911247.1_    66  ----DGYYEAEFG--------------PERRVLSFQNLGIQCVKKKDLKESISLRISRKI-NPFNV----  112
_XP_031959657.1_    66  ----DGYYEAEFG--------------PERRVLSFQNLGIQCVKKKDLKESISLRISKKI-NPFNV----  112
_XP_041905291.1_    66  ----DGYYEAEFG--------------PERRVLSFQNLGIQCVKKKDLKESISLRISKKI-NPFNV----  112
_XP_051648836.1_    66  ----DGYYEAEFG--------------PERRVLSFQNLGIQCVKKKDLKESISLRISKKI-NPFNV----  112
_XP_054239488.1_    66  ----DGYYEAEFG--------------PERRVLSFQNLGIQCVKKKDLKESISLRISKKI-NPFNV----  112
_XP_039914164.1_    66  ----DGYYEAEFG--------------PERRVLSFQNLGIQCVKKKDLKESISLRISKKI-NPFNV----  112
_XP_005422689.1_    66  ----DGYYEAEFG--------------PERRVLSFQNLGIQCVKKKDLKESISLRISKKI-NPFNV----  112
_KAF2987757.1_      66  ----DGYYEAEFG--------------PDRRVLSFQNLGIQCVKKKDLKESISLRISKKI-NPFNV----  112
_XP_038665087.1_    77  ----DGFYEADFG--------------PDRRILSFQNLGIQCVKKKEVKGAILLRIQRSL-NPFAV----  123
_GCB59945.1_        77  ----DGFYEADFG--------------PDRRILCFQNLGIQCVKKKEVKGAILLRIQRRL-NPFVV----  123
_XP_041065616.1_    77  ----DGYYEADFG--------------PDRRILCFQNLGIQCVKKKEVKEA----IQRRL-TRFTV----  119
_XP_007890564.1_    77  ----EGFYEADFG--------------PDRRVLCFQNLGIQCVRRREVKEAILFRIQRCL-NPFNV----  123
_XP_051868609.1_    77  ----DGFYEADFG--------------PDRRILCFQNLGIQCVRRKEVKDAILFRIQRQL-NPFNV----  123
_XP_032881051.1_    76  ----DGSYEAEFG--------------AERRILCFQNLGIQCVKRKEVKDAILFRIQRQL-NPFNV----  122
_XP_048456938.1_     1  ---------------------------------SFQNLGIQCAKKKEVKEAILQRIQRCL-NPFNV----   32
_KAJ1155552.1_      77  ----EGYYEADFG--------------PERRVLAFQNLGIQCVRRKEVRDAIHLRISRKI-NPFNV----  123
_KAH0622877.1_          ----------------------------------------------------------------------
_CAI5653329.1_      77  ----DGFYEAEFG--------------PDRRVIAFQNLGIQCVRRREVKEAIIQRITRGI-NPFNV----  123
_CAI5653328.1_      77  ----DGFYEAEFG--------------PDRRVIAFQNLGIQCVRRREVKEAIIQRITRGI-NPFNV----  123
_NXS54806.1_        84  ----DGYYEAEFG--------------PERRVLSFQNLGIQCVKKKDLKESISLRISKKI-NPFNV----  130
_XP_009504395.1_    74  ----DGYYEAEFG--------------PERRVLSFQNLGIQCVKKKDLKESISSRILKKI-NPFNV----  120
_NXJ41381.1_        77  ----DGYYEAEFG--------------PERRVLSFQNLGIQCVKKKDLKESISSRILKKI-NPFNV----  123
_NXT24719.1_        77  ----DGYYEAEFG--------------PERRVLSFQNLGIQCVKKKDLKESISLRISKKI-NPFNV----  123
```

NXK23502.1	77	----DGYYEAEFG--------------PERRVLSFQNLGIQCVKKKDLKESISLRISKKI-NPFNV----	123
NXK03070.1	77	----DGYYEAEFG--------------PERRVLSFQNLGIQCVKKKDLKESISSRISKKI-NPFNV----	123
_XP_037260944.1_	77	----DGYYEAEFG--------------PERRVLSFQNLGIQCVKKKDLKESISSRISKKI-NPFNV----	123
_XP_040467369.1_	77	----DGYYEAEFG--------------PERRVLSFQNLGIQCVKKKDLKESISSRISKKI-NPFNV----	123
NXY71491.1	77	----DGYYEAEFG--------------PERRVLSFQNLGIQCVKKKDLKESISLRISKKI-NPFNV----	123
NXN54417.1	77	----DGYYEAEFG--------------PERRVLSFQNLGIQCVKKKDLKESISLRISKKI-NPFNV----	123
NXX19490.1	77	----DGYYEAEFG--------------PERRVLSFQNLGIQCVKKKDLKESISLRISKKI-NPFNV----	123
NXL47199.1	77	----DGYYEAEFG--------------PERRVLSFQNLGIQCVKKKDLKESISLRISKKI-NPFNV----	123
_XP_050749583.1_	77	----DGYYEAEFG--------------PERRVLSFQNLGIQCVKKKDLKESISLRISKKI-NPFNV----	123
NXD76242.1	77	----DGYYEAEFG--------------PERRVLSFQNLGIQCVKKKDLKESISLRISKKI-NPFNV----	123
KFZ56827.1	77	----DGYYEAEFG--------------PERRVLSFQNLGIQCVKKKDLKESISLRISKKI-NPFNV----	123
NXH67691.1	77	----DGYYEAEFG--------------PERRVLSFQNLGIQCVKKKDLKESISLRISKKI-NPFNV----	123
NXS45828.1	77	----DGYYEAEFG--------------PERRVLSFQNLGIQCVKKKDLKESISSRILKKI-NPFNV----	123
KFP52034.1	77	----DGYYEAEFG--------------PERRVLSFQNLGIQCVKKKDLKESISLRISKKI-NPFNV----	123
NWH44485.1	77	----DGYYEAEFG--------------PERRVLSFQNLGIQCVKKKDLKESISSRILKKI-NPFNV----	123
NWW85416.1	77	----DGYYEAEFG--------------PERRVLSFQNLGIQCVKKKDLKESISLRISKKI-NPFNV----	123
NWU66320.1	77	----DGYYEAEFG--------------PERRVLSFQNLGIQCVKKKDLKESISLRISKKI-NPFNV----	123
_XP_025919906.1_	77	----DGYYEAEFG--------------PERRVLSFQNLGIQCVKKRDLKESISLRISKKI-NPFNV----	123
KAF1483947.1	77	----DGYYEAEFG--------------PERRVLSFQNLGIQCVKKKDLKESISLRISKKI-NPFNV----	123
NXQ87688.1	77	----DGYYEAEFG--------------PERRVLSFQNLGIQCVKKKDLKESISLRISKKI-NPFKV----	123
NXX07562.1	77	----DGYYEAEFG--------------PERRVLSFQNLGIQCVKKKDLKESISLRISKKI-NPFNV----	123
_XP_009280788.2_	77	----DGYYEAEFG--------------PERRVLSFQNLGIQCVKKKDLKESISLRISKKI-NPFNV----	123
_XP_014821857.1_	77	----DGYYEAEFG--------------PERRVLSFQNLGIQCVKKKDLKESISLRISKKI-NPFNV----	123
NWU51443.1	77	----DGYYEAEFG--------------PERRVLSFQNLGIQCVKKKDLKESISLRISKKI-NPFNV----	123
NXV35521.1	77	----DGYYEAEFG--------------PERRVLSFQNLGIQCVKKKDLKESISLRISKKI-NPFNV----	123
KFV80780.1	77	----DGYYEAEFG--------------PERRVLSFQNLGIQCVKKKDLKESIALRISKKI-NPFNV----	123
_XP_032039522.1_	77	----DGYYEAEFG--------------PERRVLSFQNLGIQCVKKRDLKESISLRISKKI-NPFNV----	123
NWT43027.1	77	----DGYYEAEFG--------------PERRVLSFQNLGIQCVKKKDLKESISLRISKKI-NPFNV----	123
KAF1651581.1	77	----DGYYEAEFG--------------PERRVLSFQNLGIQCVKKKDLKESISLRISKKI-NPFNV----	123
NXU26374.1	77	----DGYYEAEFG--------------PERRVLSFQNLGIQCVKKKDLKESISLRISKKI-NPFNV----	123
NWX46229.1	77	----DGYYEAEFG--------------PERRVLSFQNLGIQCVKKKDLKESISLRISKKI-NPFNV----	123
PKU40340.1	77	----DGYYEAEFG--------------PERRVLSFQNLGIQCVKKKDLKESISLRISKKI-NPFNV----	123
_XP_027309078.1_	77	----DGYYEAEFG--------------PERRVLSFQNLGIQCVKKRDLKESISLRISKKI-NPFNV----	123
KAF1573683.1	77	----DGYYEAEFG--------------PERRVLSFQNLGIQCVKKKDLKESISLRISKKI-NPFNV----	123
KAF1578928.1	77	----DGYYEAEFG--------------PERRVLSFQNLGIQCVKKKDLKESISLRISKKI-NPFNV----	123
NXN47104.1	77	----DGYYEAEFG--------------PERRVLSFQNLGIQCVKKKDLKESISLRISRKI-NPFNV----	123
NXV20393.1	77	----DGYYEAEFG--------------PERRVLSFQNLGIQCVKKKDLKESISLRISKKI-NPFNV----	123
KAF1551267.1	77	----DGYYEAEFG--------------PERRVLSFQNLGIQCVKKKDLKESISLRISKKI-NPFNV----	123
KAF1406210.1	77	----DGYYEAEFG--------------PERRVLSFQNLGIQCVKKKDLKESISLRISKKI-NPFNV----	123

```
_NXV43201.1_       77  ----DGYYEAEFG--------------PERRVLSFQNLGIQCVKKKDLKESISLRISKKI-NPFNV----  123
_NWQ98669.1_       77  ----DGYYEAEFG--------------PERRVLSFQNLGIQCVKKKDLKESISLRISKKI-NPFNV----  123
_XP_052639968.1_   77  ----DGYYEAEFG--------------PERRVLSFQNLGIQCVKKKDLKESISLRISKKI-NPFNV----  123
_XP_010573143.1_   77  ----DGYYEAEFG--------------PERRVLSFQNLGIQCVKKKDLKESISLRISKKI-NPFNV----  123
_KAF1479604.1_     77  ----DGYYEAEFG--------------PERRVLSFQNLGIQCVKKKDLKESISLRISKKI-NPFNV----  123
_NXG89109.1_       77  ----DGYYEAEFG--------------PERRVLSFQNLGIQCVKKKDLKESISLRISKKI-NPFNV----  123
_KFQ89757.1_       77  ----DGYYEAEFG--------------PERRVLSFQNLGIQCVKKKDLKESISLRISKKI-NPFNV----  123
_NWZ21787.1_       77  ----DGYYEAEFG--------------PERRVLSFQNLGIQCVKKRDLKESISLRISKKI-NPFNV----  123
_NXI74048.1_       77  ----DGYYEAEFG--------------PERRVLSFQNLGIQCVKKRDLKESISLRISKKI-NPFNV----  123
_NWX76247.1_       77  ----DGYYEAEFG--------------PERRVLSFQNLGIQCVKKKDLKESISLRISKKI-NPFNV----  123
_NXF29788.1_       77  ----DGYYEAEFG--------------PERRVLSFQNLGIQCVKKKDLKESISLRISKKI-NPFNV----  123
_KAF1444142.1_     77  ----DGYYEAEFG--------------PERRVLSFQNLGIQCVKKKDLKESISLRISKKI-NPFNV----  123
_KFV20215.1_       77  ----DGYYEAEFG--------------PERRVLSFQNLGIQCVKKKELKESISLRISNKI-NPFNV----  123
_NXI44657.1_       77  ----DGYYEAEFG--------------PERRVLSFQNLGIQCVKKKDLKESIALRISKKI-NPFNV----  123
_NXJ92321.1_       77  ----DGYYEAEFG--------------PERRVLSFQNLGIQCVKKKDLKESISLRISRKI-NPFNV----  123
_NWZ49902.1_       77  ----DGYYEAEFG--------------PERRVLSFQNLGIQCVKKKDLKESISLRISKKI-NPFNV----  123
_NWY59444.1_       77  ----DGYYEAEFG--------------PERRVLSFQNLGIQCVKKKDLKESISLRISKKI-NPFNV----  123
_KFW64019.1_       77  ----DGYYEAEFG--------------PERRVLSFQNLGIQCVKKKDLKESISLRISKKI-NPFNV----  123
_NXJ55634.1_       77  ----DGYYEAEFG--------------PERRVLSFQNLGIQCVKKKDLKESISLRISKKI-NPFNV----  123
_KAF1513372.1_     77  ----DGYYEAEFG--------------PERRVLSFQNLGIQCVKKKDLKESISLRISKKI-NPFNV----  123
_NXW14598.1_       77  ----DGYYEAEFG--------------PERRVLSFQNLGIQCVKKKDLKESISLRISKKI-NPFNV----  123
_NXC74247.1_       77  ----DGYYEAEFG--------------PERRVLSFQNLGIQCVKKKDLKESISSRILKKI-NPFNV----  123
_NXT57254.1_       77  ----DGYYEAEFG--------------PERRVLSFQNLGIQCVKKKDLKESISLRISKKI-NPFNV----  123
_XP_047916679.1_   77  ----DGYYEAEFG--------------PERRVLSFQNLGIQCVKKRDLKESISLRISKKI-NPFNV----  123
_XP_029879190.1_   77  ----DGYYEAEFG--------------PERRVLSFQNLGIQCVKKKDLKESISLRISKKI-NPFNV----  123
_NXH09241.1_       77  ----DGYYEAEFG--------------PERRVLSFQNLGIQCVKKKDLKESISLRISKKI-NPFNV----  123
_XP_040407374.1_   77  ----DGYYEAEFG--------------PERRVLSFQNLGIQCVKKRDLKESISLRISKKI-NPFNV----  123
_KFW76037.1_       77  ----DGYYEAEFG--------------PERRVLSFQNLGIQCVKKKDLKESISSRILKKI-NPFNV----  123
_KFP65945.1_       77  ----DGYYEAEFG--------------PERRVLSFQNLGIQCVKKKDLKESISLRISKKI-NPFNV----  123
_NXX55682.1_       77  ----DGYYEAEFG--------------PERRVLSFQNLGIQCVKKKDLKESISSRILKKI-NPFNV----  123
_XP_035178333.1_   77  ----DGYYEAEFG--------------PERRVLSFQNLGIQCVKKRDLKESISLRISKKI-NPFNV----  123
_NXE47317.1_       77  ----DGYYEAEFG--------------PERRVLSFQNLGIQCVKKRDLKESISLRISKKI-NPFNV----  123
_XP_035408877.1_   77  ----DGYYEAEFG--------------PERRVLSFQNLGIQCVKKRDLKESISLRISKKI-NPFNV----  123
_KGL94141.1_       77  ----DGYYEAEFG--------------PERRVLSFQNLGIQCVKKKDLKESISLRISKKI-NPFNV----  123
_NXN68403.1_       77  ----DGYYEAEFG--------------PERRVLSFQNLGIQCVKKKDLKESISLRISKKI-NPFNV----  123
_NXW28331.1_       77  ----DGYYEAEFG--------------PERRVLSFQNLGIQCVKKKDLKESISLRISKKI-NPFNV----  123
_XP_049657509.1_   77  ----DGYYEAEFG--------------PERRVLSFQNLGIQCVKKKDLKESISLRISKKI-NPFNV----  123
_XP_049657511.1_   77  ----DGYYEAEFG--------------PERRVLSFQNLGIQCVKKKDLKESISLRISKKI-NPFNV----  123
_NXG30098.1_       77  ----DGYYEAEFG--------------PERRVLSFQNLGIQCVKKRDLKESISLRISKKI-NPFNV----  123
```

```
_NXV86645.1_       77  ----DGYYEAEFG--------------PERRVLSFQNLGIQCVKKKDLKESISLRISKKI-NPFNV----  123
_NXX83200.1_       77  ----DGYYEAEFG--------------PERRVLSFQNLGIQCVKKKDLKESISLRISKKI-NPFNV----  123
_NXI59898.1_       77  ----DGYYEAEFG--------------PERRVLSFQNLGIQCVKKKDLKESISLRISKKI-NPFNV----  123
_NWU89521.1_       77  ----DGYYEAEFG--------------PERRVLSFQNLGIQCVKKKELKESIALRISKKI-NPFNV----  123
_NXK45490.1_       77  ----DGYYEAEFG--------------PERRVLSFQNLGIQCVKKRDLKESISLRISKKI-NPFNV----  123
_NWR55599.1_       77  ----DGYYEAEFG--------------PERRVLSFQNLGIQCVKKKELKESISLRISKKI-NPFNV----  123
_NXL37824.1_       77  ----DGYYEAEFG--------------PERRVLSFQNLGIQCVKKKDLKESISLRISKKI-NPFNV----  123
_NWI33553.1_       77  ----DGYYEAEFG--------------PERRVLSFQNLGIQCVKKKDLKESISSRILKKI-NPFNV----  123
_KFQ74517.1_       77  ----DGYYEAEFG--------------PERRVLSFQNLGIQCVKKKDLKESISLRISKKI-NPFNV----  123
_NXV82662.1_       77  ----DGYYEAEFG--------------PERRVLSFQNLGIQCVKKKDLKESISLRISKKI-NPFNV----  123
_NWJ10087.1_       77  ----DGYYEAEFG--------------PERRVLSFQNLGIQCVKKKELKESISLRISKKI-NPFNV----  123
_NXN24483.1_       77  ----DGYYEAEFG--------------PERRVLSFQNLGIQCVKKKDLKESISLRISKKI-NPFNV----  123
_NWR80208.1_       77  ----DGYYEAEFG--------------PERRVLSFQNLGIQCVKKKDLKESISLRISKKI-NPFNV----  123
_NXT37538.1_       77  ----DGYYEAEFG--------------PERRVLSFQNLGIQCVKKKDLKESISLRISKKI-NPFNV----  123
_XP_032844994.2_   77  ----DGYYEAEFG--------------PERRVLSFQNLGIQCVKKKDLKESISLRISKKI-NPFNV----  123
_NXW47649.1_       77  ----DGYYEAEFG--------------PERRVLSFQNLGIQCVKKKDLKESISLRISRKI-NPFNV----  123
_NXA40398.1_       77  ----DGYYEAEFG--------------PERRVLSFQNLGIQCVKKKELKESISLRISKKI-NPFNV----  123
_NXW64893.1_       77  ----DGYYEAEFG--------------PERRVLSFQNLGIQCVKKKDLKESISLRISKKI-NPFNV----  123
_NXX89165.1_       77  ----DGYYEAEFG--------------PERRVLSFQNLGIQCVKKKDLKESISLRISKKI-NPFNV----  123
_NXQ96911.1_       77  ----DGYYEAEFG--------------PERRVLSFQNLGIQCVKKKDLKESISLRISKKI-NPFNV----  123
_KFP18273.1_       77  ----DGYYEAEFG--------------PERRVLSFQNLGIQCVKKKDLKESISSRILKKI-NPFNV----  123
_NXC40882.1_       77  ----DGYYEAEFG--------------PERRVLSFQNLGIQCVKKRDLKESISLRISKKI-NPFNV----  123
_NXE10305.1_       77  ----DGYYEAEFG--------------PERRVLSFQNLGIQCVKKKDLKESISLRISKKI-NPFNV----  123
_KFP88087.1_       77  ----DGYYEAEFG--------------PERRVLSFQNLGIQCVKKKDLKESISLRISKKI-NPFNV----  123
_NWW47871.1_       77  ----DGYYEAEFG--------------PERRVLSFQNLGIQCVKKKDLKESISLRISKKI-NPFNV----  123
_NXJ61595.1_       77  ----DGYYEAEFG--------------PERRVLSFQNLGIQCVKKKDLKESISLRISKKI-NPFNV----  123
_NXD13814.1_       77  ----DGYYEAEFG--------------PERRVLSFQNLGIQCVKKKELKESISLRISKKI-NPFNV----  123
_KFR16961.1_       77  ----DGYYEAEFG--------------PERRVLSFQNLGIQCVKKKDLKESISLRISKKI-NPFNV----  123
_NWX24312.1_       77  ----DGYYEAEFG--------------PERRVLSFQNLGIQCVKKKDLKESISLRISKKI-NPFNV----  123
_NXA28267.1_       77  ----DGYYEAEFG--------------PERRVLSFQNLGIQCVKKKDLKESISLRISKKI-NPFNV----  123
_NXL66270.1_       77  ----DGYYEAEFG--------------PERRVLSFQNLGIQCVKKKDLKESISLRISRKI-NPFNV----  123
_NXA49976.1_       77  ----DGYYEAEFG--------------PERRVLSFQNLGIQCVKKKELKESISLRISKKI-NPFNV----  123
_NXT77624.1_       77  ----DGYYEAEFG--------------PERRVLSFQNLGIQCVKKKDLKESISLRISKKI-NPFNV----  123
_KFQ92336.1_       77  ----DGYYEAEFG--------------PERRVLSFQNLGIQCVKKKDLKESISSRILKKI-NPFNV----  123
_NXE25013.1_       77  ----DGYYEAEFG--------------PERRVLSFQNLGIQCVKKKDLKESISLRISKKI-NPFNV----  123
_NXE92240.1_       77  ----DGYYEAEFG--------------PERRVLSFQNLGIQCVKKKDLKESISLRILKKI-NPFNV----  123
_NWH80943.1_       77  ----DGYYEAEFG--------------PERRVLSFQNLGIQCVKKKDLKESISLRISRKI-NPFNV----  123
_NWI19058.1_       77  ----DGYYEAEFG--------------PERRVLSFQNLGIQCVKKKELKESISLRISKKI-NPFNV----  123
_NXG80610.1_       77  ----DGYYEAEFG--------------PERRVLSFQNLGIQCVKKKDLKESISLRISKKI-NPFNV----  123
```

NWR01486.1	77	----DGYYEAEFG--------------PERRVLSFQNLGIQCVKKKDLKESISLRISKKI-NPFNV----	123
NXK81337.1	77	----DGYYEAEFG--------------PERRVLSFQNLGIQCVKKKDLKESISLRISRKI-NPFNV----	123
NXS90563.1	77	----DGYYEAEFG--------------PERRVLSFQNLGIQCVKKKDLKESISLRISKKI-NPFNV----	123
NXC17430.1	77	----DGYYEAEFG--------------PERRVLSFQNLGIQCVKKKDLKESISLRISKKI-NPFNV----	123
NXE77245.1	77	----DGYYEAEFG--------------PERRVLSFQNLGIQCVKKKDLKESISSRILKKI-NPFNV----	123
KFQ29788.1	78	----DGYYEAEFG--------------PERRVLSFQNLGIQCVKKKDLKESISLRISKKI-NPFNV----	124
KFO89327.1	77	----DGYYEAEFG--------------PERRVLSFQNLGIQCVKKKELKESISLRISKKI-NPFNV----	123
NXD64381.1	77	----DGYYEAEFG--------------PERRVLSFQNLGIQCVKKKDLKESISLRISKKI-NPFNV----	123
NXY45081.1	77	----DGYYEAEFG--------------PERRVLSFQNLGIQCVKKKDLKESISLRISKKI-NPFNV----	123
KGL72871.1	77	----DGYYEAEFG--------------PERRVLSFQNLGIQCVKKKELKESISLRISKKI-NPFNV----	123
_XP_053919504.1_	77	----DGYYEAEFG--------------PERRVLSFQNLGIQCVKKKDLKESISLRISKKI-NPFNV----	123
NXL85459.1	77	----DGYYEAEFG--------------PERRVLSFQNLGIQCVKKRDLKESISLRISKKI-NPFNV----	123
NXO02254.1	77	----DGYYEAEFG--------------PERRVLSFQNLGIQCVKKKELKESIALRISKKI-NPFNV----	123
NXJ76034.1	77	----DGYYEAEFG--------------PERRVLSFQNLGIQCVKKKDLKESISLRISKKI-NPFNV----	123
NXM93491.1	77	----DGYYEAEFG--------------PERRVLSFQNLGIQCVKKKDLKESISLRISKKI-NPFNV----	123
_XP_030345800.1_	77	----DGYYEAEFG--------------PERRVLSFQNLGIQCVKKKDLKESISLRISKKI-NPFNV----	123
_XP_030302544.1_	77	----DGYYEAEFG--------------PERRVLSFQNLGIQCVKKKDLKESISLRISKKI-NPFNV----	123
_XP_030345801.1_	77	----DGYYEAEFG--------------PERRVLSFQNLGIQCVKKKDLKESISLRISKKI-NPFNV----	123
KQK77724.1	77	----DGYYEAEFG--------------PERRVLSFQNLGIQCVKKKDLKESISLRISRKI-NPFNV----	123
NXP09913.1	77	----DGYYEAEFG--------------PERRVLSFQNLGIQCVKKKDLKESISLRISKKI-NPFNV----	123
NXR33018.1	77	----DGYYEAEFG--------------PERRVLSFQNLGIQCVKKKDLKESISLRISKKI-NPFNV----	123
NXM76881.1	77	----DGYYEAEFG--------------PERRVLSFQNLGIQCVKKKDLKESIALRISKKI-NPFNV----	123
NXS27423.1	77	----DGYYEAEFG--------------PERRVLSFQNLGIQCVKKKDLKESISLRISKKI-NPFNV----	123
NXM55821.1	77	----DGYYEAEFG--------------PERRVLSFQNLGIQCVKKKDLKESIALRISKKI-NPFNV----	123
_XP_054678500.1_	77	----DGYYEAEFG--------------PERRVLSFQNLGIQCVKKKDLKESISLRISKKI-NPFNV----	123
_XP_015712248.1_	77	----DGYYEAEFG--------------PERRVLSFQNLGIQCVKKKDLKESISLRISKKI-NPFNV----	123
NWX92058.1	77	----DGYYEAEFG--------------PERRVLSFQNLGIQCVKKKELKESISLRISRKI-NPFNV----	123
_XP_048793646.1_	77	----DGYYEAEFG--------------PERRVLSFQNLGIQCVKKKDLKESISLRISKKI-NPFNV----	123
NXF04247.1	77	----DGYYEAEFG--------------PERRVLSFQNLGIQCVKKKDLKESIALRISKKI-NPFNV----	123
_XP_042683331.1_	77	----DGYYEAEFG--------------PERRVLSFQNLGIQCVKKKDLKESISLRISKKI-NPFNV----	123
NWV22147.1	77	----DGYYEAEFG--------------PERRVLSFQNLGIQCVKKKDLKESISLRISKKI-NPFNV----	123
NXA04361.1	77	----DGYYEAEFG--------------PERRVLSFQNLGIQCVKKKDLKESISLRISKKI-NPFNV----	123
_XP_010116198.1_	83	----DGYYEAEFG--------------PERRVLSFQNLGIQCVKKKDLKESISLRISKKI-NPFNV----	129
NXG59741.1	77	----DGYYEAEFG--------------PERRVLSFQNLGIQCVKKKDLKESISLRISKKI-NPFNV----	123
_XP_042735326.1_	77	----DGYYEAEFG--------------PERRVLSFQNLGIQCVKKKDLKESISLRISKKI-NPFNV----	123
NXM29886.1	77	----DGYYEAEFG--------------PERRVLSFQNLGIQCVKKKDLKESISLRISKKI-NPFNV----	123
_NP_001161198.2_	77	----DGYYEAEFG--------------PERRVLSFQNLGIQCVKKKDLKESISLRISKKI-NPFNV----	123
_XP_031451029.1_	77	----DGYYEAEFG--------------PERRVLSFQNLGIQCVKKKDLKESISLRISKKI-NPFNV----	123
NXR49262.1	77	----DGYYEAEFG--------------PERRVLSFQNLGIQCVKKKDLKESISLRISKKI-NPFNV----	123

```
_XP_052555416.1_   77  ----DGYYEAEFG--------------PERRVLSFQNLGIQCVKKKDLKESISLRISKKI-NPFNV----  123
_NWZ67085.1_       77  ----DGYYEAEFG--------------PERRVLSFQNLGIQCVKKKDLKESISLRISKKI-NPFNV----  123
_P16236.2_         77  ----DGYYEAEFG--------------PERRVLSFQNLGIQCVKKKDLKESISLRISKKI-NPFNV----  123
_XP_050566010.1_   77  ----DGYYEAEFG--------------PERRVLSFQNLGIQCVKKRDLKESISLRISKKI-NPFNV----  123
_NWS67030.1_       77  ----DGYYEAEFG--------------PERRVLSFQNLGIQCVKKKDLKESISLRISKKI-NPFNV----  123
_XP_005143850.3_   77  ----DGYYEAEFG--------------PERRVLSFQNLGIQCVKKKDLKESISLRISKKI-NPFNV----  123
_XP_050566011.1_   77  ----DGYYEAEFG--------------PERRVLSFQNLGIQCVKKRDLKESISLRISKKI-NPFNV----  123
_XP_050566009.1_   77  ----DGYYEAEFG--------------PERRVLSFQNLGIQCVKKRDLKESISLRISKKI-NPFNV----  123
_NWR30963.1_       77  ----DGYYEAEFG--------------PERRVLSFQNLGIQCVKKKDLKESISLRISKKI-NPFNV----  123
_NWS17896.1_       77  ----DGYYEAEFG--------------PERRVLSFQNLGIQCVKKKDLKESISLRISKKI-NPFNV----  123
_XP_021248574.1_   77  ----DGYYEAEFG--------------PERRVLSFQNLGIQCVKKKDLKESISLRISKKI-NPFNV----  123
_AAA48721.1_       77  ----DGYYEAEFG--------------PERRVLSFQNLGIQCVKKKDLKESISLRISKKI-NPFNV----  123
_NWI54208.1_       77  ----DGYYEAEFG--------------PERRVLSFQNLGIQCVKKKDLKESIALRISKKI-NPFNV----  123
_XP_027509532.1_   77  ----DGYYEAEFG--------------PERRVLSFQNLGIQCVKKKDLKESISLRISKKI-NPFNV----  123
_NXP71979.1_       77  ----DGYYEAEFG--------------PERRVLSFQNLGIQCVKKKDLKESISLRISKKI-NPFNV----  123
_NXX25352.1_       77  ----DGYYEAEFG--------------PERRVLSFQNLGIQCVKKKDLKESISLRISKKI-NPFNV----  123
_NXI18618.1_       77  ----DGYYEAEFG--------------PERRVLSFQNLGIQCVKKKDLKESISLRISKKI-NPFNV----  123
_NXS09623.1_       77  ----DGYYEAEFG--------------PERRVLSFQNLGIQCVKKKDLKESISLRISKKV-NPFNV----  123
_NXU77980.1_       77  ----DGYYEAEFG--------------PERRVLSFQNLGIQCVKKKDLKESISLRISKKI-NPFNV----  123
_XP_027509533.1_   77  ----DGYYEAEFG--------------PERRVLSFQNLGIQCVKKKDLKESISLRISKKI-NPFNV----  123
_NWV71036.1_       77  ----DGYYEAEFG--------------PERRVLSFQNLGIQCVKKKDLKESISLRISKKI-NPFSV----  123
_NXF89482.1_       77  ----DGYYEAEFG--------------PERRVLSFQNLGIQCVKKKDLKESISLRISKKI-NPFNV----  123
_NWY39470.1_       77  ----DGYYEAEFG--------------PERRVLSFQNLGIQCVKKKELKESISLRISKKI-NPFNV----  123
_NXT59960.1_       77  ----DGYYEAEFG--------------PERRVLSFQNLGIQCVKKKDLKESISLRISKKI-NPFNV----  123
_NWU74340.1_       77  ----DGYYEAEFG--------------PERRVLSFQNLGIQCVKKKDLKESISLRISKKI-NPFNV----  123
_NXB84481.1_       77  ----DGYYEAEFG--------------PERRVLSFQNLGIQCVKKKELKESISLRILKKI-NPFNV----  123
_NWV73885.1_       77  ----DGYYEAEFG--------------PERRVLSFQNLGIQCVKKKDLKESISLRISKKI-NPFNV----  123
_OPJ58894.1_       77  ----DGYYEAEFG--------------PERRVLSFQNLGIQCVKKKDLKESIALRISKKI-NPFNV----  123
_XP_048152104.1_   77  ----DGYYEAEFG--------------PERRVLSFQNLGIQCVKKKDLKESISLRISKKI-NPFNV----  123
_XP_054036037.1_   77  ----DGYYEAEFG--------------PERRVLSFQNLGIQCVKKKDLKDSISLRISKKI-NPFNV----  123
_NXR18149.1_       77  ----DGYYEAEFG--------------PERRVLSFQNLGIQCVKKKDLKESISLRISKKI-NPFNV----  123
_NXP97533.1_       77  ----DGYYEAEFG--------------PERRVLSFQNLGIQCVKKKELKESISLRILKKI-NPFNV----  123
_NXJ05495.1_       77  ----DGYYEAEFG--------------PERRVLSFQNLGIQCVKKKDLKESISLRISKKI-NPFNV----  123
_XP_032538932.1_   77  ----DGYYEAEFG--------------PERRVLSFQNLGIQCVKKKDLKESISLRISKKI-NPFNV----  123
_NWI96387.1_       77  ----DGYYEAEFG--------------PERRVLSFQNLGIQCVKKKDLKESISLRISKKI-NPFNV----  123
_NXR65995.1_       77  ----DGYYEAEFG--------------PERRVLSFQNLGIQCVKKKDLKESISLRISKKI-NPFNV----  123
_NXK42398.1_       77  ----DGYYEAEFG--------------PERRVLSFQNLGIQCVKKKDLKESISLRISKKI-NPFNV----  123
_XP_032538931.1_   77  ----DGYYEAEFG--------------PERRVLSFQNLGIQCVKKKDLKESISLRISKKI-NPFNV----  123
_NXY33181.1_       77  ----DGYYEAEFG--------------PERRVLSFQNLGIQCVKKKDLKESISLRISKKI-NPFNV----  123
```

```
_XP_050171009.1_    77  ----DGYYEAEFG---------------PERRVLSFQNLGIQCVKKKDLKESISLRISKKI-NPFNV----  123
_NXO50939.1_        77  ----DGYYEAEFG---------------PERRVLSFQNLGIQCVKKKDLKESISLRISKKI-NPFNV----  123
_NWZ32937.1_        77  ----DGYYEAEFG---------------PERRVLSFQNLGIQCVKKKDLKESISLRISKKI-NPFNV----  123
_NXP01828.1_        77  ----DGYYEAEFG---------------PERRVLSFQNLGIQCVKKKDLKESISLRISKKI-NPFNV----  123
_NXL00458.1_        77  ----DGYYEAEFG---------------PERRVFSFQNLGIQCVKKKDLKESISSRILKKI-NPFNV----  123
_XP_010401310.4_    77  ----DGYYEAEFG---------------PERRVLSFQNLGIQCVKKKDLKESISLRISKKI-NPFNV----  123
_NXH62149.1_        77  ----DGYYEAEFG---------------PERRVLSFQNLGIQCVKKKDLKESISLRISKKI-NPFNV----  123
_NXG43824.1_        77  ----DGYYEAEFG---------------PERRVLSFQNLGIQCVKKKDLKESISLRISKKI-NPFNV----  123
_XP_017677337.1_    77  ----DGYYEAEFG---------------PERRVLSFQNLGIQCVKKKDLKESISLRISKKI-NPFNV----  123
_NXU09156.1_        77  ----DGYYEAEFG---------------PERRVLSFQNLGIQCVKKKDLKESISLRILKKI-NPFNV----  123
_NXB38645.1_        77  ----DGYYEAEFG---------------PERRVLSFQNLGIQCVKKKDLKESISLRISKKI-NPFNV----  123
_XP_027594504.1_    77  ----DGYYEAEFG---------------PERRVLSFQNLGIQCVKKKDLKESISLRISKKI-NPFNV----  123
_NXM46269.1_        77  ----DGYYEAEFG---------------PERRVLSFQNLGIQCVKKKDLKESISLRISKKI-NPFNV----  123
_NWS79257.1_        77  ----DGYYEAEFG---------------PERRVLSFQNLGIQCVKKKDLKESISLRISKKI-NPFNV----  123
_NXB04839.1_        77  ----DGYYEAEFG---------------PERRVLSFQNLGIQCVKKKDLKESISLRISKKI-NPFNV----  123
_NXC62865.1_        77  ----DGYYEAEFG---------------PERRVLSFQNLGIQCVKKKDLKESISLRIAKKI-NPFNV----  123
_NXN18277.1_        77  ----DGYYEAEFG---------------PERRVLSFQNLGIQCVKKKDLKESISLRISKKI-NPFNV----  123
_XP_027760039.1_    77  ----DGYYEAEFG---------------PERRVLSFQNLGIQCVKKKDLKESISLRISKKI-NPFNV----  123
_NXI90311.1_        77  ----DGYYEAEFG---------------PERRVLSFQNLGIQCVKKKDLKESISLRISKKI-NPFNV----  123
_NWY17003.1_        77  ----DGYYEAEFG---------------PERRVLSFQNLGIQCVKKKDLKESISLRISKKI-NPFNV----  123
_NXH97099.1_        77  ----DGYYEAEFG---------------PERRVLSFQNLGIQCVKKKDLKESISLRISKKI-NPFNV----  123
_NWS97308.1_        77  ----DGYYEAEFG---------------PERRVLSFQNLGIQCVKKKDLKESISLRISKKI-NPFNV----  123
_NWU45160.1_        77  ----DGYYEAEFG---------------PERRVLSFQNLGIQCVKKKDLKESISLRILKKI-NPFNV----  123
_NXQ43004.1_        77  ----DGYYEAEFG---------------PERRVLSFQNLGIQCVKKKDLKESISLRISRKI-NPFNV----  123
_XP_027549142.1_    77  ----DGYYEAEFG---------------PERRVLSFQNLGIQCVKKKDLKESISLRISKKI-NPFNV----  123
_XP_017933082.2_    77  ----DGYYEAEFG---------------PERRVLSFQNLGIQCVKKKDLKESISLRISKKI-NPFNV----  123
_NWV96326.1_        77  ----DGYYEAEFG---------------PERRVLSFQNLGIQCVKKKDLKESISLRISKKI-NPFNV----  123
_NXP57783.1_        77  ----DGYYEAEFG---------------PERRVLSFQNLGIQCVKKKDLKESISLRISKKI-NPFNV----  123
_NXY57955.1_        77  ----DGYYEAEFG---------------PERRVLSFQNLGIQCVKKKDLKESISLRISKKI-NPFNV----  123
_AAA99433.1_        77  ----DGYYEAEFG---------------PERRVLSFQNLGIQCVKKKDLKESISLRISKKI-NPFNV----  123
_NXY21218.1_        77  ----DGYYEAEFG---------------PERRVLSFQNLGIQCVKKKDLKESISLRILKKI-NPFNV----  123
_NXX50824.1_        77  ----DGYYEAEFG---------------PERRVLSFQNLGIQCVKKKDLKESISLRISKKI-NPFNV----  123
_XP_030124623.3_    77  ----DGYYEAEFG---------------PERRVLSFQNLGIQCVKKKDLKESISLRILKKI-NPFNV----  123
_NXA92235.1_        77  ----DGYYEAEFG---------------PERRVLSFQNLGIQCVKKKDLKESISLRISKKI-NPFNV----  123
_NWI05522.1_        77  ----DGYYEAEFG---------------PERRVLSFQNLGIQCVKKKDLKESISLRILKKI-NPFNV----  123
_NWX29009.1_        77  ----DGYYEAEFG---------------PERRVLSFQNLGIQCVKKKDLKESISLRISKKI-NPFNV----  123
_XP_027549144.1_    77  ----DGYYEAEFG---------------PERRVLSFQNLGIQCVKKKDLKESISLRISKKI-NPFNV----  123
_NWW35420.1_        77  ----DGYYEAEFG---------------PERRVLSFQNLGIQCVKKKDLKESISLRISKKI-NPFNV----  123
_NWV07969.1_        77  ----DGYYEAEFG---------------PERRVLSFQNLGIQCVKKKDLKESISLRISKKI-NPFNV----  123
```

```
_NXH42214.1_       77  ----DGYYEAEFG--------------PERRVLSFQNLGIQCVKKKDLKESISLRISKKI-NPFNV----  123
_NXQ18773.1_       77  ----DGYYEAEFG--------------PERRVLSFQNLGIQCVKKKDLKESISLRISKKI-NPFNV----  123
_NWX10672.1_       77  ----DGYYEAEFG--------------PERRVLSFQNLGIQCVKKKDLKESIALRISKKI-NPFNV----  123
_NXU50687.1_       77  ----DGYYEAEFG--------------PERRVLSFQNLGIQCVKKKDLKESISLRISKKI-NPFNV----  123
_KFU84784.1_       77  ----DGYYEAEFG--------------PERRVLSFQNLGIQCVKKKDLKESISLRISKKI-NPFNV----  123
_NXQ52438.1_       77  ----DGYYEAEFG--------------PERRVLSFQNLGIQCVKKKDLKESISLRILKKI-NPFNV----  123
_NWW24536.1_       77  ----DGYYEAEFG--------------PERRVLSFQNLGIQCVKKKDLKESISLRISKKI-NPFNV----  123
_NXF73449.1_       77  ----DGYYEAEFG--------------PERRVLSFQNLGIQCVKKKDLKESISLRISKKI-NPFNV----  123
_XP_050827906.1_   77  ----DGYYEAEFG--------------PERRVLSFQNLGIQCVKKKDLKESISLRISKKI-NPFNV----  123
_NWX65205.1_       77  ----DGYYEAEFG--------------PERRVLSFQNLGIQCVKKKDLKESISLRILKKI-NPFNV----  123
_KFW81749.1_       77  ----DGYYEAEFG--------------PERRVLSFQNLGIQCVKKKDLKESISLRISKKI-NPFNV----  123
_XP_014750593.1_   77  ----DGYYEAEFG--------------PERRVLSFQNLGIQCVKKKDLKESISLRISKKI-NPFNV----  123
_NXJ17066.1_       77  ----DGYYEAEFG--------------PERRVLSFQNLGIQCVKKKDLKESISLRISKKI-NPFNV----  123
_NWW67019.1_       77  ----DGYYEAEFG--------------PERRVLSFQNLGIQCVKKKDLKESISLRISKKI-NPFNV----  123
_NXH03360.1_       77  ----DGYYEAEFG--------------PERRVLSFQNLGIQCVKKKDLKESISLRISKKI-NPFNV----  123
_XP_037987525.1_   77  ----DGYYEAEFG--------------PERRVLSFQNLGIQCVKKKDLKESISLRISKKI-NPFNV----  123
_NXL74522.1_       77  ----DGYYEAEFG--------------PERRVLSFQNLGIQCVKKKDLKESISLRILKKI-NPFNV----  123
_NWQ60824.1_       77  ----DGYYEAEFG--------------PERRVLSFQNLGIQCVKKKDLKESISLRISKKI-NPFNV----  123
_NXR70971.1_       77  ----DGYYEAEFG--------------PERRVLSFQNLGIQCVKKKDLKESISLRISKKI-NPFNV----  123
_XP_051468947.1_   77  ----DGYYEAEFG--------------PERRVLSFQNLGIQCVKKKDLKESISLRISKKI-NPFNV----  123
_NXB50199.1_       77  ----DGYYEAEFG--------------PERRVLSFQNLGIQCVKKKDLKESISLRISKKI-NPFNV----  123
_NXP18380.1_       77  ----DGYYEAEFG--------------PERRVLSFQNLGIQCVKKKDLKESISLRISKKI-NPFNV----  123
_NXI84319.1_       77  ----DGYYEAEFG--------------PERRVLSFQNLGIQCVKKKDLKESISLRISRKI-NPFNV----  123
_NWH33757.1_       77  ----DGYYEAEFG--------------PERRVLSFQNLGIQCVKKKDLKESISLRISKKI-NPFNV----  123
_NWR99781.1_       77  ----DGYYEAEFG--------------PERRVLSFQNLGIQCVKKKDLKESISLRISKKI-NPFNV----  123
_NXW94404.1_       77  ----DGYYEAEFG--------------PERRVLSFQNLGIQCVKKKDLKESIALRISKKI-NPFNV----  123
_CAA27220.1_       77  ----DGYYEAEFG--------------PERRVLSFQNLGIQCVKKKDLKESISLRISKKI-NPFNV----  123
_XP_015477679.1_   77  ----DGYYEAEFG--------------PERRVLSFQNLGIQCVKKKDLKESISLRISKKI-NPFNV----  123
_NWS32597.1_       77  ----DGYYEAEFG--------------PERRVLSFQNLGIQCVKKKDLKESISLRISKKI-NPFNV----  123
_NXG22126.1_       77  ----DGYYEAEFG--------------PERRVLSFQNLGIQCVKKKDLKESISLRISKKI-NPFNV----  123
_NWY97127.1_       77  ----DGYYEAEFG--------------PERRVLSFQNLGIQCVKKKDLKESISLRISKKI-NPFNV----  123
_NXW80148.1_       77  ----DGYYEAEFG--------------PERRVLSFQNLGIQCVKKKDLKESISLRISKKI-NPFNV----  123
_NXC27367.1_       77  ----DGYYEAEFG--------------PERRVLSFQNLGIQCVKKKDLKESISLRISKKI-NPFNV----  123
_NWH83726.1_       77  ----DGYYEAEFG--------------PERRVLSFQNLGIQCVKKKDLKESISLRISKKI-NPFNV----  123
_NXA73512.1_       77  ----DGYYEAEFG--------------PERRVLSFQNLGIQCVKKKDLKESISLRISKKI-NPFNV----  123
_NWZ83230.1_       77  ----DGYYEAEFG--------------PERRVLSFQNLGIQCVKKKDLKESISLRISKKI-NPFNV----  123
_XP_005525572.2_   77  ----DGYYEAEFG--------------PERRVLSFQNLGIQCVKKKDLKESISLRISKKI-NPFNV----  123
_NXD20033.1_       77  ----DGYYEAEFG--------------PERRVLSFQNLGIQCVKKKDLKESISLRISRKI-NPFNV----  123
_NXE37177.1_       77  ----DGYYEAEFG--------------PERRVLSFQNLGIQCVKKKDLKESISLRISKKI-NPFNV----  123
```

```
_NWV44369.1_       77  ----DGYYEAEFG--------------PERRVLSFQNLGIQCVKKKDLKESISLRISKKI-NPFNV----  123
_NXC81400.1_       77  ----DGYYEAEFG--------------PERRVLSFQNLGIQCVKKKDLKESISLRISRKI-NPFNV----  123
_NXA67345.1_       77  ----DGYYEAEFG--------------PERRVLSFQNLGIQCVKKKDLKESISLRISKKI-NPFNV----  123
_NXB55155.1_       77  ----DGYYEAEFG--------------PERRVLSFQNLGIQCVKKKDLKESISLRISKKI-NPFNV----  123
_NXN81229.1_       77  ----DGYYEAEFG--------------PERRVLSFQNLGIQCVKKKDLKESISLRILKKI-NPFNV----  123
_NXM15601.1_       77  ----DGYYEAEFG--------------PERRVLSFQNLGIQCVKKKDLKESISLRISKKI-NPFNV----  123
_XP_054484850.1_   77  ----DGYYEAEFG--------------PERRVLSFQNLGIQCVKKKDLKESISLRISKKI-NPFNV----  123
_NWU02685.1_       77  ----DGYYEAEFG--------------PERRVLSFQNLGIQCVKKKDLKESISLRISKKI-NPFNV----  123
_XP_041273165.1_   77  ----DGYYEAEFG--------------PERRVLSFQNLGIQCVKKKDLKESISLRISKKI-NPFNV----  123
_NWW80397.1_       77  ----DGYYEAEFG--------------PERRVLSFQNLGIQCVKKKDLKESISLRISKKI-NPFNV----  123
_XP_036236540.1_   77  ----DGYYEAEFG--------------PERRVLSFQNLGIQCVKKKDLKESISLRISKKI-NPFNV----  123
_NWV50957.1_       77  ----DGYYEAEFG--------------PERRVLSFQNLGIQCVKKKDLKESISLRIAKKI-NPFNV----  123
_NXT97287.1_       77  ----DGYYEAEFG--------------PERRVLSFQNLGIQCVKKKDLKESISLRISKKI-NPFNV----  123
_XP_023778876.1_   77  ----DGYYEAEFG--------------PERRVLSFQNLGIQCVKKKDLKESISLRISKKI-NPFNV----  123
_NXD42381.1_       77  ----DGYYEAEFG--------------PERRVLSFQNLGIQCVKKKDLKESISLRISRKI-NPFNV----  123
_NXK69295.1_       77  ----DGYYEAEFG--------------PERRVLSFQNLGIQCVKKKDLKESISLRISKKI-NPFNV----  123
_XP_049657510.1_   77  ----DGYYEAEFG--------------PERRVLSFQNLGIQCVKKKDLKESISLRISKKI-NPFNV----  123
_NXV62539.1_       77  ----DGYYEAEFG--------------PERRVLSFQNLGIQCVKKKDLKESISLRISKKI-NPFNV----  123
_NXB80231.1_       77  ----DGYYEAEFG--------------PERRVLSFQNLGIQCVKKKDLKDSISLRISKKI-NPFNV----  123
_NWU15026.1_       77  ----DGYYEAEFG--------------PERRVLSFQNLGIQCVKKKDLKESISLRISRKI-NPFNV----  123
_NXL26104.1_       77  ----DGYYEAEFG--------------PERRVLSFQNLGIQCVKKKDLKESISLRISKKI-NPFNV----  123
_NWW05001.1_       77  ----DGYYEAEFG--------------PERRVLSFQNLGIQCVKKKDLKESISLRISKKI-NPFNV----  123
_RLW07261.1_       77  ----DGYYEAEFG--------------PERRVLSFQNLGIQCVKKKDLKESISLRILKKI-NPFNV----  123
_NXR96625.1_       77  ----DGYYEAEFG--------------PERRVLSFQNLGIQCVKKKDLKESISLRISKKI-NPFNV----  123
_XP_021383204.1_   77  ----DGYYEAEFG--------------PERRVLSFQNLGIQCVKKKDLKESISLRILKKI-NPFNV----  123
_NXQ25764.1_       77  ----DGYYEAEFG--------------PERRVLSFQNLGIQCVKKKDLKESISLRISRKI-NPFNV----  123
_NXO08412.1_       77  ----DGYYEAEFG--------------PERRVLSFQNLGIQCVKKKDLKESISLRISKKI-NPFNV----  123
_NXI32280.1_       77  ----DGYYEAEFG--------------PERRVLSFQNLGIQCVKKKDLKESISLRISKKI-NPFNV----  123
_NXG00288.1_       77  ----DGYYEAEFG--------------PERRVLSFQNLGIQCVKKKDLKESISLRISKKI-NPFNV----  123
_XP_041329879.1_   77  ----DGYYEAEFG--------------PERRVLSFQNLGIQCVKKKDLKESISLRISKKI-NPFNV----  123
_NXV03759.1_       77  ----DGYYEAEFG--------------PERRVLSFQNLGIQCVKKKDLKESISLRISKKI-NPFNV----  123
_NWT16975.1_       77  ----DGYYEAEFG--------------PERRVLSFQNLGIQCVKKKDLKESISLRISKKI-NPFNV----  123
_NWT67953.1_       77  ----DGYYEAEFG--------------PERRVLSFQNLGIQCVKKKDLKESISLRISKKI-NPFNV----  123
_XP_005042765.2_   77  ----DGYYEAEFG--------------PERRVLSFQNLGIQCVKKKDLKESISLRISRKI-NPFNV----  123
_XP_017600715.1_   77  ----DGYYEAEFG--------------PERRVLSFQNLGIQCVKKKDLKESISLRISKKI-NPFNV----  123
_NXD93279.1_       77  ----DGYYEAEFG--------------PERRVLSFQNLGIQCVKKKDLKESISLRISKKI-NPFNV----  123
_NXY02260.1_       77  ----DGYYEAEFG--------------PERRVLSFQNLGIQCVKKKDLKESISLRISKKI-NPFNV----  123
_NXO84677.1_       77  ----DGYYEAEFG--------------PERRVLSFQNLGIQCVKKKDLKESISLRISKKI-NPFNV----  123
_NXO64371.1_       77  ----DGYYEAEFG--------------PERRVLSFQNLGIQCVKKKDLKESISLRISRKI-NPFNV----  123
```

```
_NXO42022.1_       77  ----DGYYEAEFG--------------PERRVLSFQNLGIQCVKKKDLKESISLRISKKI-NPFNV----  123
_NWY65013.1_       77  ----DGYYEAEFG--------------PERRVLSFQNLGIQCVKKKDLKESISLRISRKI-NPFNV----  123
_NXP90563.1_       77  ----DGYYEAEFG--------------PERRVLSFQNLGIQCVKKKDLKESISLRISKKI-NPFNV----  123
_NWI73723.1_       77  ----DGYYEAEFG--------------PERRVLSFQNLGIQCVKKKDLKESISLRISKKI-NPFNV----  123
_NXH30511.1_       77  ----DGYYEAEFG--------------PERRVLSFQNLGIQCVKKKDLKESISLRISKKI-NPFNV----  123
_NWT77800.1_       77  ----DGYYEAEFG--------------PERRVLSFQNLGIQCVKKKDLKESISLRISKKI-NPFNV----  123
_NWT34209.1_       77  ----DGYYEAEFG--------------PERRVLSFQNLGIQCVKKKDLKESISLRISKKI-NPFNV----  123
_NWY29369.1_       77  ----DGYYEAEFG--------------PERRVLSFQNLGIQCVKKKDLKESISLRISKKI-NPFNV----  123
_NXQ73821.1_       77  ----DGYYEAEFG--------------PERRVLSFQNLGIQCVKKKDLKESISLRISKKI-NPFNV----  123
_XP_039572786.1_   77  ----DGYYEAEFG--------------PERRVLSFQNLGIQCVKKKDLKESISLRISKKI-NPFNV----  123
_XP_030802035.1_   77  ----DGYYEAEFG--------------PERRVLSFQNLGIQCVKKKDLKESISLRISKKI-NPFNV----  123
_NXE68211.1_       77  ----DGYYEAEFG--------------PERRVLSFQNLGIQCVKKKDLKESISLRISKKI-NPFNV----  123
_NXS80652.1_       77  ----DGYYEAEFG--------------PERRVLSFQNLGIQCVKKKDLKESISLRISKKI-NPFNV----  123
_NXO32431.1_       77  ----DGYYEAEFG--------------PERRVLSFQNLGIQCVKKKDLKESISLRISKKI-NPFNV----  123
_NWR17317.1_       77  ----DGYYEAEFG--------------PERRVLSFQNLGIQCVKKKDLKESISLRISKKI-NPFNV----  123
_NWZ89732.1_       77  ----DGYYEAEFG--------------PERRVLSFQNLGIQCVKKKDLKESISLRISKKI-NPFNV----  123
_XP_032039523.1_   77  ----DGYYEAEFG--------------PERRVLSFQNLGIQCVKKRDLKESISLRISKKI-NPFNV----  123
_KFO04362.1_       77  ----DGYYEAEFG--------------PERRVLSFQNLGIQCVKKKDLKESISLRISKKI-NPFNV----  123
_XP_054146213.1_   77  ----DGYYEAEFG--------------PDRRVLSFQNLGIQCVKKKDLKESISLRISKKI-NPFNV----  123
_XP_005486916.2_   77  ----DGYYEAEFG--------------PDRRVLSFQNLGIQCVKKKDLKESISLRISKKI-NPFNV----  123
_KAJ7422946.1_     77  ----DGYYEAEFG--------------PERRVLSFQNLGIQCVKKKDLKESISLRISKKI-NPFNV----  123
_NXC99388.1_       77  ----DGYYEAEFG--------------PERRVLSFQNLGIQCVKKKDLKESISLRILKKI-NPFNV----  123
_RMC07975.1_       77  ----DGYYEAEFG--------------PERRVLSFQNLGIQCVKKKDLKESISLRISKKI-NPFNV----  123
_NXT95159.1_       35  ----DGYYEAEFG--------------PERRVLSFQNLGIQCVKKKDLKESISSRILKKI-NPFNV----   81
_XP_009957589.1_   35  ----DGYYEAEFG--------------PERRVLSFQNLGIQCVKKKDLKESIYLRISKKI-NPFNV----   81
_TRZ13602.1_       35  ----DGYYEAEFG--------------PERRVLSFQNLGIQCVKKKDLKESISLRISKKI-NPFNV----   81
_NWR45911.1_       26  ----DGYYEAEFG--------------PERRVLSFQNLGIQCVKKKDLKESISLRISKKI-NPFNV----   72
_KAF4799649.1_     35  ----DGYYEAEFG--------------PERRVLSFQNLGIQCVKKKDLKESISLRISRKI-NPFNV----   81
_KAJ7420138.1_     77  ----DGYYEAEFG--------------PERRVLSLGH------------QRNIPRLLVGF-QPFQLDSY-  114
_XP_054678501.1_   77  ----DGYYEAEFG--------------PERRVLSFQNLGIQCVKKKDLKESISLRISKKI-NPFNV----  123
_NXP48685.1_       77  ----DGYYEAEFG--------------PERRVLSFQNLGIQCVKKKDLKESISLRISKKI-NPFNV----  123
_XP_010704701.1_    1  -----------LK--------------PYHVYFSFQNLGIQCVKKKDLKESISLRISKKI-NPFNV----   40
_KFV10805.1_        1  ---------------------------------SFQNLGIQCVKKKDLKESISLRISKKI-NPFNV----   32
_XP_009572567.1_    1  ----------------------------------------------------------------KV----    2
_NXF64277.1_        1  -----------------------------------------------------------------V----    1
_KFV54465.1_        1  -----------------------------------------------------------------V----    1
_KFV90717.1_        1  -----------------------------------------------------------------V----    1
_KFQ03674.1_        1  -----------------------------------------------------------------V----    1
_NXW01028.1_        1  -----------------------------------------------------------------V----    1
```

```
_KFP32908.1_        1  -----------------------------------------------------------------V----    1
_KFV93389.1_        1  -----------------------------------------------------------------V----    1
_NWI63728.1_        1  -----------------------------------------------------------------V----    1
_KAI6072063.1_      1  ----------------------------------------------------------------PV----    2
_KFQ58901.1_        1  -----------------------------------------------------------------V----    1
_NXP33753.1_        1  -----------------------------------------------------------------V----    1
_XP_009476353.1_       ----------------------------------------------------------------------
_KFQ55903.1_        1  -----------------------------------------------------------------V----    1
_XP_042683349.1_    1  -----------------------------------------------------------------L----    1
_NXR08991.1_        1  -----------------------------------------------------------------V----    1
_XP_008947250.1_    1  -----------------------------------------------------------------V----    1
_KFP72464.1_        1  -----------------------------------------------------------------V----    1
_KFV60523.1_        1  -----------------------------------------------------------------V----    1
_NXT13165.1_        1  -----------------------------------------------------------------V----    1
_NWT50506.1_        1  -----------------------------------------------------------------V----    1
_OXB76139.1_           ----------------------------------------------------------------------
_XP_050827907.1_    1  ----------------------------------------------------TTKLSSGQ-NAESC----   13
_XP_030302545.1_    1  ---------------------------------------------------------LGQ-NGESC----    8
_XP_054036038.1_    1  -----------------------------------------------------MKQSLGQ-NVESC----   12
_XP_012788337.1_   77  ----DGYYEAEFG--------------QERRPLFFQNLGIRCVKKKEVKEAIISRIKAGI-NPFNV----  123
_XP_008070154.1_   66  ----DGYYEAEFG--------------QERRPLFFQNLGIRCVKKKEVKEAIISRIKAGI-NPFNV----  112
_XP_027477445.1_   66  ----DGYYEAEFG--------------QERRPLFFQNLGIRCVKKKEVKEAIISRIRAGI-NPFNV----  112
_XP_025848473.1_   66  ----DGYYEAEFG--------------QERRPLFFQNLGIRCVKKKEVKEAIISRIRAGI-NPFNV----  112
_XP_049640716.1_   77  ----DGYYEAEFG--------------QERRPLFFQNLGIRCVKKKEVKEAIISRIRAGI-NPFNV----  123
_XP_023084089.1_   77  ----DGYYEAEFG--------------QERRPLFFQNLGIRCVKKKEVKEAIISRIKAGI-NPFNV----  123
_EHH22143.1_       77  ----DGYYEAEFG--------------QERRPLFFQNLGIRCVKKKEVKEAIISRIKAGI-NPFNV----  123
_XP_038504711.1_   77  ----DGYYEAEFG--------------QERRPLFFQNLGIRCVKKKEVKEAIISRIRAGI-NPFNV----  123
_XP_038504703.1_   77  ----DGYYEAEFG--------------QERRPLFFQNLGIRCVKKKEVKEAIISRIRAGI-NPFNV----  123
_XP_038504718.1_   77  ----DGYYEAEFG--------------QERRPLFFQNLGIRCVKKKEVKEAIISRIRAGI-NPFNV----  123
_XP_048971856.1_   77  ----DGYYEAEFG--------------QERRPLFFQNLGIRCVKKKEVKEAIISRIRAGI-NPFNV----  123
_KAB1266826.1_     77  ----DGYYEAEFG--------------QERRPLFFQNLGIRCVKKKEVKEAIISRIRAGI-NPFNV----  123
_XP_048391424.1_   77  ----DGFYEADFG--------------PDRRIICFQNLGIQCAKKKEVKEAILQRIQRCL-NPFNV----  123
_XP_048391425.1_   77  ----DGFYEADFG--------------PDRRIICFQNLGIQCAKKKEVKEAILQRIQRCL-NPFNV----  123
_GCC28026.1_       77  ----DGFYEADFG--------------PDRRIICFQNLGIQCAKKKEVKEAILQRIQRSL-NPFNV----  123
_XP_043551991.1_   77  ----DGFYEADFG--------------PDRRIICFQNLGIQCAKKKEVKEAILQRIQRNL-NPFNV----  123
_XP_043551989.1_   77  ----DGFYEADFG--------------PDRRIICFQNLGIQCAKKKEVKEAILQRIQRNL-NPFNV----  123
_XP_054534389.1_    1  --------------------------------LCFQNLGIRCVKKKEVKEAIITRIKAGI-NPFNV----   33
_XP_011531312.1_    1  --------------------------------LCFQNLGIRCVKKKEVKEAIITRIKAGI-NPFNV----   33
_XP_021780541.1_    1  ---------------------------------NFQNLGIRCVKKKEVKEAIISRIKAGI-NPFNV----   32
```

```
_XP_037862912.1_     7  ----DGYYEAEFG--------------QERRPLFFQNLGIRCVKKKEVKEAIISRIKAGI-NPFNV----   53
_XP_015413428.1_    66  ----DGYYEAEFG--------------QERRPLFFQNLGIRCVKKKEVKEAIISRIRAGI-NPFNV----  112
_XP_005884642.1_    66  ----DGYYEAEFG--------------QERRPLFFQNLGIRCVKKKEVKEAIISRIRAGI-NPFNV----  112
_KAF6105903.1_      66  ----DGYYEAEFG--------------QERRPLFFQNLGIRCVKKKEVKEAIVSRISAGI-NPFNI----  112
_XP_015413426.1_    66  ----DGYYEAEFG--------------QERRPLFFQNLGIRCVKKKEVKEAIISRIRAGI-NPFNV----  112
_XP_014387539.1_    66  ----DGYYEAEFG--------------QERRPLFFQNLGIRCVKKKEVKEAIISRIRAGI-NPFNV----  112
_XP_014444723.1_    66  ----DGYYEAEFV--------------QEHRPLFFQNLGIRCVKKKEVKEAVISRIRAGI-NPFKV----  112
_XP_023448058.1_    66  ----DGYYEAEFG--------------QERRPLFFQNLGIRCVKKKEVKEAIASRIRARI-NPFSV----  112
_EGW11604.1_        66  ----DGYYEAEFG--------------PERRPLFFQNLGIRCVKKKEVKEAIILRISAGI-NPFNV----  112
_XP_015985129.1_    66  ----DGYYEAEFG--------------QERRPLFFQNLGIRCVKKKEVKEAIISRIRAGI-NPFAV----  112
_XP_012878014.1_    66  ----DGYYEAEFG--------------QERRPLFFQNLGIRCVKKKEVKEVIISRINAGI-NPFNV----  112
_KAG5211506.1_      45  ----DGYYEAEFG--------------QERRPLFFQNLGIRCVKKKEVKEAIISRMRAGI-NPFNV----   91
_MXQ82539.1_        41  ----DGYYEAEFG--------------QERRPLFFQNLGIRCVKKKEVKDAVISRVRAGI-NPFNV----   87
_XP_032729748.1_    77  ----DGYYEAEFG--------------QERRPLFFQNLGIRCVKKKEVKEAITSRIRAGI-NPF------  121
_XP_032208113.1_    77  ----DGYYEAEFG--------------QERRPLFFQNLGIRCVKKKEVKEAITSRIRAGI-NPF------  121
_XP_048208817.1_    77  ----DGYYEAEFG--------------QERRPLFFQNLGIRCVKKKEVKEVIISRINAGI-NPFNV----  123
_XP_038200333.1_    77  ----DGYYEAEFG--------------PERRPLFFQNLGIRCVKKKEVKDAIILRISAGI-NPFNV----  123
_P15307.2_          77  ----DGYYEAEFG--------------PERRPLFFQNLGIRCVKKKEVKGAIILRISAGI-NPFNV----  123
_XP_041524635.1_    66  ----DGYYEAEFG--------------PERRPLFFQNLGIRCVKKKEVKDAIILRISAGI-NPFNV----  112
_CAA33843.1_        77  ----DPYYEAEFG--------------PERRPLFFQNLGIRCVKKKEVKGAIILRISAGI-NPFNV----  123
_XP_028732210.2_    66  ----DGYYEAEFG--------------PERRPLFFQNLGIRCVKKKEVKEAIILRISAGI-NPFNV----  112
_XP_025848474.1_    66  ----DGYYEAEFG--------------QERRPLFFQNLGIRCVKKKEVKEAIISRIRAGI-NPFNV----  112
_EDL97996.1_        66  ----DGYYEAEFG--------------PERRPLFFQNLGIRCVKKKEVKEAIILRISAGI-NPFNV----  112
_KAI4577886.1_      66  ----DGYYEAEFG--------------QERRPLFFQNLGIRCVKKKEVKEAIISRMRAGI-NPFNV----  112
_KAG8512309.1_      77  ----DGYYEAEFG--------------QERRPLFFQNLGIRCVKKKEVKEAIISRIRAGI-NPFNV----  123
_XP_047637971.1_    77  ----DGYYEAEFG--------------QERRPLCFQNLGIRCVKKKEVKEAIISRIRAGI-NPFNV----  123
_XP_019491454.1_    77  ----DGYYEAEFG--------------QERRPLFFQNLGIRCVKKKEVKEAIISRIKAGI-NPFKV----  123
_XP_043842482.1_    79  ----DGYYEAEFG--------------QERRALIFQNLGIRCVKKKEVKEAIISRISAEI-NPFNV----  125
_XP_027721170.1_    79  ----DGYYEAEFG--------------QERRALIFQNLGIRCVKKKEVKEAIISRISAEI-NPFNV----  125
_XP_036604632.1_    79  ----DGYYEAEFG--------------QERRALIFQNLGIRCVKKKEVKEAIISRISAEI-NPFNV----  125
_XP_020841164.1_    79  ----DGYYEAEFG--------------QERRALIFQNLGIRCVKKKEVKEAIISRISAEI-NPFNV----  125
_XP_051831295.1_    79  ----DGYYEAEFG--------------QERRALIFQNLGIRCVKKKEVKEAIISRISAEI-NPFNV----  125
_XP_031806378.1_    79  ----DGYYEAEFG--------------QERRALIFQNLGIRCVKKKEVKEAIVSRISAEI-NPFNV----  125
_XP_051831294.1_    79  ----DGYYEAEFG--------------QERRALIFQNLGIRCVKKKEVKEAIISRISAEI-NPFNV----  125
_XP_003339776.1_    79  ----DGYYEAEFG--------------QERRALIFQNLGIRCVKKKEVKEAIISRISAEI-NPFNV----  125
_XP_044515413.1_    79  ----DGYYEAEFG--------------QERRALIFQNLGIRCVKKKEVKEAIISRISAEI-NPFNV----  125
_XP_028928361.1_    79  ----DGYYEAEFG--------------QDRRALCFQNLGIRCVKKKEVKEAIISRIATEI-NPFNV----  125
_XP_038607403.1_    79  ----DGYYEAEFG--------------QDRRALCFQNLGIRCVKKKEVKEAIISRIATEI-NPFNV----  125
_XP_040849334.1_     1  ----------------------------------------------------------------F-----    1
```

```
_TKC46270.1_             ----------------------------------------------------------------------
_OWK10634.1_             ----------------------------------------------------------------------
_XP_036190342.1_         ----------------------------------------------------------------------
_KAB0395596.1_           ----------------------------------------------------------------------
_KAF6430170.1_           ----------------------------------------------------------------------
_XP_038948811.1_     1   ----------------------------------------------------------------F-----    1
_KAB1266828.1_      77   ----DGYYEAEFG--------------QERRPLFFQNLGIRCVKKKEVKEAIISRIRAGI-NPFNV----  123
_NP_002899.1_       77   ----DGYYEAEFG--------------QERRPLFFQNLGIRCVKKKEVKEAIITRIKAGI-NPFNV----  123
_XP_001159537.3_    77   ----DGYYEAEFG--------------QERRPLFFQNLGIRCVKKKEVKEAIITRIKAGI-NPFNV----  123
_PNI66333.1_        77   ----DGYYEAEFG--------------QERRPLFFQNLGIRCVKKKEVKEAIITRIKAGI-NPFNV----  123
_EHH55592.1_        77   ----DGYYEAEFG--------------QERRPLFFQNLGIRCVKKKEVKEAIISRIKAGI-NPFNV----  123
_XP_003262470.1_    77   ----DGYYEAEFG--------------QERRPLFFQNLGIRCVKKKEVKEAIITRIKAGI-NPFNV----  123
_BAG64854.1_        77   ----DGYYEAEFG--------------QERRPLFFQNLGIRCVKKKEVKEAIITRIKAGI-NPFNV----  123
_XP_032034471.1_    77   ----DGYYEAEFG--------------QERRPLFFQNLGIRCVKKKEVKEAIITRIKAGI-NPFNV----  123
_XP_037862911.1_    77   ----DGYYEAEFG--------------QERRPLFFQNLGIRCVKKKEVKEAIISRIKAGI-NPFNV----  123
_XP_003830911.1_    77   ----DGYYEAEFG--------------QERRPLFFQNLGIRCVKKKEVKEAIITRIKAGI-NPFNV----  123
_XP_004029331.1_    77   ----DGYYEAEFG--------------QERRPLFFQNLGIRCVKKKEVKEAIITRIKAGI-NPFNV----  123
_XP_002812051.1_    77   ----DGYYEAEFG--------------QERRPLFFQNLGIRCVKKKEVKEAIITRIKAGI-NPFNV----  123
_XP_011711623.1_    77   ----DGYYEAEFG--------------QERRPLFFQNLGIRCVKKKEVKEAIISRIKAGI-NPFNV----  123
_XP_033095023.1_    77   ----DGYYEAEFG--------------QERRPLFFQNLGIRCVKKKEVKEAIISRIKAGI-NPFNV----  123
_XP_002799280.1_    77   ----DGYYEAEFG--------------QERRPLFFQNLGIRCVKKKEVKEAIISRIKAGI-NPFNV----  123
_XP_011853172.1_    77   ----DGYYEAEFG--------------QERRPLFFQNLGIRCVKKKEVKEAIISRIKAGI-NPFNV----  123
_XP_050609443.1_    77   ----DGYYEAEFG--------------QERRPLFFQNLGIRCVKKKEVKEAIISRIKAGI-NPFNV----  123
_XP_003908735.1_    77   ----DGYYEAEFG--------------QERRPLFFQNLGIRCVKKKEVKEAIISRIKAGI-NPFNV----  123
_XP_025209693.1_    77   ----DGYYEAEFG--------------QERRPLFFQNLGIRCVKKKEVKEAIISRIKAGI-NPFNV----  123
_XP_023084087.1_    77   ----DGYYEAEFG--------------QERRPLFFQNLGIRCVKKKEVKEAIISRIKAGI-NPFNV----  123
_XP_021780539.1_    77   ----DGYYEAEFG--------------QERRPLFFQNLGIRCVKKKEVKEAIISRIKAGI-NPFNV----  123
_XP_017704568.1_    77   ----DGYYEAEFG--------------QERRPLFFQNLGIRCVKKKEVKEAIISRIKAGI-NPFNV----  123
_XP_010375811.1_    77   ----DGYYEAEFG--------------QERRPLFFQNLGIRCVKKKEVKEAIISRIKAGI-NPFNV----  123
_XP_030776399.1_    77   ----DGYYEAEFG--------------QERRPLFFQNLGIRCVKKKEVKEAIISRIKAGI-NPFNV----  123
_XP_011797211.1_    77   ----DGYYEAEFG--------------QERRPLFFQNLGIRCVKKKEVKEAIISRIKAGI-NPFNV----  123
_XP_012332682.1_    77   ----DGYYEAEFG--------------QERRPLFFQNLGIRCVKKKEVKEAIISRIKAGI-NPFNV----  123
_XP_010331875.2_    77   ----DGYYEAEFG--------------QERRPLFFQNLGIRCVKKKEVKEAIISRIKAGI-NPFNV----  123
_XP_012332683.1_    77   ----DGYYEAEFG--------------QERRPLFFQNLGIRCVKKKEVKEAIISRIKAGI-NPFNV----  123
_XP_017400475.1_    77   ----DGYYEAEFG--------------QERRPLFFQNLGIRCVKKKEVKEAIISRIKAGI-NPFNV----  123
_XP_008568357.1_    77   ----DGYYEAEFG--------------QERRPLFFQNLGIRCVKKKEVKEAIISRIRAGI-NPFSV----  123
_XP_012620284.1_    77   ----DGYYEAEFV--------------QERRPLFFQNLGIRCVKKKEVKEAIISRIKAGI-NPFNV----  123
_XP_002709741.2_    77   ----DGYYEAEFG--------------QERRPLFFQNLGIRCVKKKEVKEAIISRISAGI-NPFNV----  123
_XP_022452943.1_    77   ----DGYYEAEFG--------------QERRPLFFQNLGIRCVKKKEVKEAIISRIRAGI-NPFNV----  123
```

_XP_045405625.1_	77	----DGYYEAEFV---------------QERRPLFFQNLGIRCVKKKEVKEAIISRIKAGI-NPFNV----	123
_XP_004280707.1_	77	----DGYYEAEFG---------------QERRPLFFQNLGIRCVKKKEVKEAIISRIRAGI-NPFNV----	123
_XP_030733169.1_	77	----DGYYEAEFG---------------QERRPLFFQNLGIRCVKKKEVKEAIISRIRAGI-NPFNV----	123
TEA23022.1	77	----DGYYEAEFG---------------QERRPLFFQNLGIRCVKKKEVKEAIISRIRAGI-NPFNV----	123
_XP_012494629.1_	77	----DGYYEAEFV---------------QERRPLFFQNLGIRCVKKKEVKEAIISRIKAGI-NPFNV----	123
_XP_010950191.1_	77	----DGYYEAEFG---------------QERRPLFFQNLGIRCVKKKEVKEAIISRIRAGI-NPFNV----	123
_XP_024608785.1_	77	----DGYYEAEFG---------------QERRPLFFQNLGIRCVKKKEVKEAIISRIRAGI-NPFNV----	123
_XP_006201637.1_	77	----DGYYEAEFG---------------QERRPLFFQNLGIRCVKKKEVKEAIISRIRAGI-NPFSV----	123
_XP_019285354.1_	77	----DGYYEAEFG---------------QERRPLFFQNLGIRCVKKKEVKEAIISRIRAGI-NPFNV----	123
_XP_032509224.1_	77	----DGYYEAEFG---------------QERRPLFFQNLGIRCVKKKEVKEAIISRIRAGI-NPFNV----	123
_XP_047379355.1_	77	----DGYYEAEFG---------------QERRPLFFQNLGIRCVKKKEVKEAIISRISSGI-NPFNV----	123
_XP_032981134.1_	77	----DGFYEAEFG---------------QERRPLFFQNLGIRCVKKKEVKEAIISRIRAGI-NPFKV----	123
_XP_005600018.1_	77	----DGYYEAEFG---------------QERRPLFFQNLGIRCVKKKEVKEAIVARIRAGI-NPFNV----	123
_XP_046517051.1_	77	----DGYYEAEFG---------------QERRPLFFQNLGIRCVKKKEVKEAIVARIRAGI-NPFNV----	123
_XP_007464205.1_	77	----DGYYEAEFG---------------QERRPLFFQNLGIRCVKKKEVKEAIISRIRAGI-NPFNV----	123
_XP_019572903.1_	77	----DGFYEAEFG---------------QERRPLFFQNLGIRCVKKKEVKEAIISRIRAGI-NPFKV----	123
_XP_007189755.1_	77	----DGYYEAEFG---------------QERRPLFFQNLGIRCVKKKEVKEAIISRIRAGI-NPFNV----	123
_XP_004397771.1_	77	----DGYYEAEFG---------------QERRPLFFQNLGIRCVKKKEVKEAIISRIRAGI-NPFNV----	123
_XP_004435713.1_	77	----DGYYEAEFG---------------QERRPLFFQNLGIRCVKKKEVKEAIISRIRAGI-NPFNV----	123
_XP_008698124.1_	77	----DGYYEAEFG---------------QERRPLFFQNLGIRCVKKKEVKEAIISRIRAGI-NPFNV----	123
_XP_023989255.1_	77	----DGYYEAEFG---------------QERRPLFFQNLGIRCVKKKEVKEAVISRIRAGI-NPFNV----	123
_XP_011233012.2_	77	----DGYYEAEFG---------------QERRPLFFQNLGIRCVKKKEVKEAIISRIRAGI-NPFNV----	123
_XP_025724444.1_	77	----DGYYEAEFG---------------QERRPLFFQNLGIRCVKKKEVKEAIISRIRAGI-NPFNV----	123
_XP_005626186.1_	77	----DGYYEAEFG---------------QERRPLFFQNLGIRCVKKKEVKEAIISRIRAGI-NPFNV----	123
_XP_045643991.1_	77	----DGYYEAEFG---------------QERRPLFFQNLGIRCVKKKEVKEAIISRIRAGI-NPFNV----	123
_XP_034857383.1_	77	----DGYYEAEFG---------------QERRPLFFQNLGIRCVKKKEVKEAIVSRIRAGI-NPFNV----	123
_XP_027945501.1_	77	----DGYYEAEFG---------------QERRPLFFQNLGIRCVKKKEVKEAIISRIRAGI-NPFNV----	123
_XP_026341094.1_	77	----DGYYEAEFG---------------QERRPLFFQNLGIRCVKKKEVKEAIISRIRAGI-NPFNV----	123
EFB29923.1	77	----DGYYEAEFG---------------QERRPLFFQNLGIRCVKKKEVKEAIISRIRAGI-NPFNV----	123
_XP_045740897.1_	77	----DGYYEAEFG---------------QERRPLFFQNLGIRCVKKKEVKEAIVSRIRAGI-NPFNV----	123
CAD7677454.1	77	----DGYYEAEFG---------------QERRPLFFQNLGIRCVKKKEVKEAIISRIRAGI-NPFNV----	123
_XP_035577553.1_	77	----DGYYEAEFG---------------QERRPLFFQNLGIRCVKKKEVKEAIISRIRAGI-NPFNV----	123
_XP_006733255.1_	77	----DGYYEAEFG---------------QERRPLFFQNLGIRCVKKKEVKEAIISRIRAGI-NPFNV----	123
_XP_032266820.1_	77	----DGYYEAEFG---------------QERRPLFFQNLGIRCVKKKEVKEAIISRIRAGI-NPFNV----	123
_XP_041611730.1_	77	----DGYYEAEFG---------------QERRPLFFQNLGIRCVKKKEVKEAIISRIRAGI-NPFNV----	123
_XP_048971854.1_	77	----DGYYEAEFG---------------QERRPLFFQNLGIRCVKKKEVKEAIISRIRAGI-NPFNV----	123
_XP_021554840.1_	77	----DGYYEAEFG---------------QERRPLFFQNLGIRCVKKKEVKEAIISRIRAGI-NPFNV----	123
VCW78562.1	77	----DGYYEAEFG---------------QERRPLFFQNLGIRCVKKKEVKEAITSRIRAGI-NPFNV----	123
_XP_034857381.1_	77	----DGYYEAEFG---------------QERRPLFFQNLGIRCVKKKEVKEAIVSRIRAGI-NPFNV----	123

```
_XP_008524231.1_   74  ----DGYYEAEFG--------------QERRPLFFQNLGIRCVKKKEVKEAIVARIRAGI-NPFNV----  120
_XP_045740895.1_   77  ----DGYYEAEFG--------------QERRPLFFQNLGIRCVKKKEVKEAIVSRIRAGI-NPFNV----  123
_XP_045834746.1_   77  ----DGYYEAEFG--------------QERRPLFFQNLGIRCVKKKEVKEAITSRIRAGI-NPFNV----  123
_XP_035932906.1_   77  ----DGYYEAEFG--------------QERRPLFFQNLGIRCVKKKEVKEAIISRIRAGI-NPFNV----  123
_XP_030883859.1_   77  ----DGYYEAEFG--------------QERRPLFFQNLGIRCVKKKEVKEAIISRIRAGI-NPFNV----  123
_XP_032266818.1_   77  ----DGYYEAEFG--------------QERRPLFFQNLGIRCVKKKEVKEAIISRIRAGI-NPFNV----  123
_XP_024408528.2_   77  ----DGYYEAEFG--------------QERRPLFFQNLGIRCVKKKEVKEAIISRISAGI-NPFNV----  123
_XP_004580347.1_   77  ----DGYYEAEFG--------------QERRPLFFQNLGIRCVKKKEVKEAVISRISAGI-NPFNV----  123
_XP_030166525.1_   77  ----DGYYEAEFG--------------QERRPLFFQNLGIRCVKKKEVKEAIISRIRAGI-NPFNV----  123
_XP_035932904.1_   77  ----DGYYEAEFG--------------QERRPLFFQNLGIRCVKKKEVKEAIISRIRAGI-NPFNV----  123
_XP_020040690.1_   77  ----DGYYEAEFG--------------QERRPLFFQNLGIRCVKKKEVKEAIISRISAGI-NPFNV----  123
_XP_053445604.1_   77  ----DGYYEAEFG--------------QERRPLFFQNLGIRCVKKKEVKEAIISRIKAGI-NPFNV----  123
_XP_049507482.1_   77  ----DGYYEAEFG--------------QERRPLFFQNLGIRCVKKKEVKEAIISRIRAGI-NPFNV----  123
_XP_015335127.1_   77  ----DGYYEAEFG--------------QERRPLFFQNLGIRCVKKKEVKEAIISRISSGI-NPFNV----  123
_XP_027790245.1_   77  ----DGYYEAEFG--------------QERRPLFFQNLGIRCVKKKEVKEAIISRISSGI-NPFNV----  123
_XP_003984077.1_   77  ----DGYYEAEFG--------------QERRPLFFQNLGIRCVKKKEVKEAIISRIRAGI-NPFNV----  123
_XP_039096259.1_   77  ----DGYYEAEFG--------------QERRPLFFQNLGIRCVKKKEVKEAIISRIKAGI-NPFNV----  123
_XP_007075434.1_   77  ----DGYYEAEFG--------------QERRPLFFQNLGIRCVKKKEVKEAIISRIRAGI-NPFNV----  123
_XP_022360130.1_   77  ----DGYYEAEFG--------------QERRPLFFQNLGIRCVKKKEVKEAITSRIRAGI-NPFNV----  123
_XP_047707378.1_   77  ----DGYYEAEFG--------------QERRPLFFQNLGIRCVKKKEVKEAIISRIRAGI-NPFNV----  123
_XP_026243031.1_   77  ----DGYYEAEFG--------------QERRPLFFQNLGIRCVKKKEVKEAIISRISSGI-NPFNV----  123
_XP_037383340.1_   77  ----DGYYEAEFG--------------QERRPLFFQNLGIRCVKKKEVKEAIFSRVRAGI-NPFNV----  123
_XP_005322095.1_   77  ----DGYYEAEFG--------------QERRPLFFQNLGIRCVKKKEVKEAIISRISSGI-NPFNV----  123
_XP_005662584.1_   77  ----DGYYEAEFG--------------QERRPLCFQNLGIRCVKKKEVKEAIISRIRAGI-NPFNV----  123
_XP_040080101.1_   77  ----DGYYEAEFG--------------QERRPLFFQNLGIRCVKKKEVKEAIISRIRAGI-NPFNV----  123
_XP_017503999.1_   77  ----DGYYEAEFG--------------QERRPLFFQNLGIRCVKKKEVKEAIISRIRAGI-NPFNV----  123
_XP_044119954.1_   77  ----DGYYEAEFG--------------QERRPLFFQNLGIRCVKKKEVKEAITSRIRAGI-NPFNV----  123
_XP_032208110.1_   77  ----DGYYEAEFG--------------QERRPLFFQNLGIRCVKKKEVKEAITSRIRAGI-NPFNV----  123
_XP_012586936.1_   77  ----DGYYEAEFG--------------QERRPLFFQNLGIRCVKKKEVKEAVISRIRAGI-NPFNV----  123
_XP_032729745.1_   77  ----DGYYEAEFG--------------QERRPLFFQNLGIRCVKKKEVKEAITSRIRAGI-NPFNV----  123
_XP_052504199.1_   77  ----DGYYEAEFG--------------QERRPLFFQNLGIRCVKKKEVKEAIISRIRAGI-NPFNV----  123
_XP_054431389.1_   77  ----DGYYEAEFG--------------QERRPLFFQNLGIRCVKKKEVKEAIISRISAGI-NPFNV----  123
_XP_023576632.1_   77  ----DGYYEADFG--------------QERRPLFFQNLGIRCVKKKEVKEAVISRISAGI-NPFNV----  123
_XP_004742070.1_   77  ----DGYYEAEFG--------------QERRPLFFQNLGIRCVKKKEVKEAITSRIRAGI-NPFNV----  123
_XP_037663267.1_   77  ----DGYYEAEFG--------------QERRPLFFQNLGIRCVKKKEVKEAVVSRIRAGI-NPFSV----  123
_XP_004005978.1_   77  ----DGYYEAEFG--------------QERRPLFFQNLGIRCVKKKEVKEAIISRMRAGI-NPFNV----  123
_XP_036781908.1_   77  ----DGYYEAEFG--------------QERRPLFFQNLGIRCVKKKEVKEAIISRLRAGI-NPFNV----  123
_XP_040849333.1_   77  ----DGYYEAEFG--------------QERRPLFFQNLGIRCVKKKEVKEAVISRISAGI-NPFNV----  123
_XP_005686691.1_   77  ----DGYYEAEFG--------------QERRPLFFQNLGIRCVKKKEVKEAIISRIRAGI-NPFNV----  123
```

```
_XP_023367900.1_   77  ----DGYYEAEFG--------------QERRPLFFQNLGIRCVKKKEVKEAVISRIKAGI-NPFNV----  123
_KAF0879576.1_     77  ----DGYYEAEFG--------------QERRPLFFQNLGIRCVKKKEVKEAIISRIKAGI-NPFNV----  123
_XP_029793284.1_   77  ----DGYYEAEFG--------------QERRPLFFQNLGIRCVKKKEVKEAIISRIRAGI-NPFNV----  123
_NP_001179899.1_   77  ----DGYYEAEFG--------------QERRPLFFQNLGIRCVKKKEVKDAVISRVRAGI-NPFNV----  123
_XP_020764718.1_   77  ----DGYYEAEFG--------------QERRPLFFQNLGIRCVKKKEVKEAIISRIRAGI-NPFNV----  123
_XP_054431388.1_   77  ----DGYYEAEFG--------------QERRPLFFQNLGIRCVKKKEVKEAIISRISAGI-NPFNV----  123
_XP_037663265.1_   77  ----DGYYEAEFG--------------QERRPLFFQNLGIRCVKKKEVKEAVVSRIRAGI-NPFSV----  123
_XP_019825763.1_   77  ----DGYYEAEFG--------------QERRPLFFQNLGIRCVKKKEVKDAVISRVRAGI-NPFNV----  123
_XP_005889096.1_   77  ----DGYYEAEFG--------------QERRPLFFQNLGIRCVKKKEVKDAVISRVRAGI-NPFNV----  123
_XP_043324571.1_   77  ----DGYYEAEFG--------------QERRPLFFQNLGIRCVKKKEVKEAIISRIRAGI-NPFNV----  123
_KAJ1078272.1_     77  ----DGYYEAEFG--------------QERRPLFFQNLGIRCVKKKEVKEAIISRIRAGI-NPFNV----  123
_XP_036190339.1_   77  ----DGYYEAEFG--------------QERRPLFFQNLGIRCVKKKEVKEAIISRIRAGI-NPFNV----  123
_XP_013005522.1_   77  ----DGYYEADFG--------------QERRPLFFQNLGIRCVKKKEVKEAVISRINAGI-NPFNV----  123
_XP_006073936.1_   77  ----DGYYEAEFG--------------QERRPLFFQNLGIRCVKKKEVKDAVISRIRAGI-NPFNV----  123
_XP_016069529.1_   77  ----DGYYEAEFG--------------QERRPLFFQNLGIRCVKKKEVKEAIISRIRAGI-NPFKV----  123
_KAB0354136.1_     77  ----DGYYEAEFG--------------QERRPLFFQNLGIRCVKKKEVKEAIISRIRAGI-NPFNV----  123
_XP_029424871.1_   77  ----DGYYEAEFG--------------QERRQLFFQNLGIRCVKKKEVKEAIISRISAGI-NPFNV----  123
_XP_036118792.1_   77  ----DGYYEAEFG--------------QERRPLFFQNLGIRCVKKKEVKEAIISRIRAGI-NPFKV----  123
_XP_037019725.2_   76  ----DGYYEAEFG--------------QERRPLFFQNLGIRCVKKKEVKEAIISRISAGI-NPFNI----  122
_XP_047602572.1_   77  ----DGYYEAEFG--------------QERRPLFFQNLGIRCVKKKEVKEAITSRIRAGI-NPFNV----  123
_XP_014586639.1_   77  ----DGYYEAEFG--------------QERRPLFFQNLGIRCVKKKEVKEAIVARIRAGI-NPFNV----  123
_XP_046517052.1_   77  ----DGYYEAEFG--------------QERRPLFFQNLGIRCVKKKEVKEAIVARIRAGI-NPFNV----  123
_XP_006909635.2_   77  ----DGYYEAEFG--------------QERRPLFFQNLGIRCVKKKEVKEAIISRIRAGI-NPFEV----  123
_XP_011362222.1_   77  ----DGYYEAEFG--------------QERRPLFFQNLGIRCVKKKEVKEAIISRIRAGI-NPFEV----  123
_XP_045684409.1_   77  ----DGYYEAEFG--------------QERRPLFFQNLGIRCVKKKEVKEAIVSRISAGI-NPFNI----  123
_XP_048208816.1_   77  ----DGYYEAEFG--------------QERRPLFFQNLGIRCVKKKEVKEVIISRINAGI-NPFNV----  123
_XP_052590629.1_   77  ----DGYYEAEFG--------------PERRPLFFQNLGIRCVKKKEVKEAIILRISAGI-NPFNV----  123
_XP_032353499.1_   77  ----DGYYEAEFG--------------QERRPLFFQNLGIRCVKKKEVKEAIISRIRAGI-NPFNV----  123
_XP_038200327.1_   77  ----DGYYEAEFG--------------PERRPLFFQNLGIRCVKKKEVKDAIILRISAGI-NPFNV----  123
_XP_051056550.1_   77  ----DGYYEAEFG--------------PERRPLFFQNLGIRCVKKKEVKEAVILRISAGI-NPFNV----  123
_XP_012805850.2_   77  ----DGYYEAEFG--------------QERRPLFFQNLGIRCVKKKEVKEAIISRINLGI-NPFNV----  123
_XP_016050906.1_   77  ----DGFYEAEFG--------------QERRPLFFQNLGIRCVKKKEVKEAIISRIRAGI-NPFNV----  123
_XP_030166526.1_   77  ----DGYYEAEFG--------------QERRPLFFQNLGIRCVKKKEVKEAIISRIRAGI-NPFNV----  123
_XP_036903878.1_   76  ----DGYYEAEFG--------------QERRPLFFQNLGIRCVKKKEVKEAIISRISAGI-NPFNI----  122
_CAH7004032.1_     77  ----DGYYEAEFG--------------PERRPLFFQNLGIRCVKKKEVKEAVILRISAGI-NPFNV----  123
_XP_051056549.1_   77  ----DGYYEAEFG--------------PERRPLFFQNLGIRCVKKKEVKEAVILRISAGI-NPFNV----  123
_XP_042547166.1_   77  ----DGYYEAEFG--------------QERRPLFFQNLGIRCVKKKEVKEVIISRINAGI-NPFNV----  123
_XP_054584456.1_   77  ----DGYYEAEFG--------------QERRPLFFQNLGIRCVKKKEVKEAIISRIRAGI-NPFNV----  123
_XP_049996768.1_   77  ----DGYYEAEFG--------------PERRPLFFQNLGIRCVKKKEVKDAIILRISAGI-NPFNV----  123
```

```
_XP_027243805.1_   77  ----DGYYEAEFG---------------PERRPLFFQNLGIRCVKKKEVKEAIILRISAGI-NPFNV----  123
_XP_047707379.1_   77  ----DGYYEAEFG---------------QERRPLFFQNLGIRCVKKKEVKEAIISRIRAGI-NPFNV----  123
_XP_026645370.1_   77  ----DGYYEAEFG---------------PERRPLFFQNLGIRCVKKKEVKDAIILRISAGI-NPFNV----  123
_XP_014302519.1_   77  ----DGYYEAEFG---------------QERRPLFFQNLGIRCVKKKEVKEAIISRIRAGI-NPFNV----  123
_XP_049726303.1_   77  ----DGYYEAEFG---------------QERRPLFFQNLGIRCVKKKEVKEAVISRIRAGI-NPFSV----  123
_XP_049726302.1_   77  ----DGYYEAEFG---------------QERRPLFFQNLGIRCVKKKEVKEAVISRIRAGI-NPFSV----  123
_XP_012969026.1_   77  ----DGYYEAEFG---------------PERRPLFFQNLGIRCVKKKEVKEAIILRISAGI-NPFSV----  123
_XP_008524232.1_   74  ----DGYYEAEFG---------------QERRPLFFQNLGIRCVKKKEVKEAIVARIRAGI-NPFNV----  120
_XP_051020904.1_   77  ----DGYYEAEFG---------------PERRPLFFQNLGIRCVKKKEVKEAIILRISAGI-NPFNV----  123
_XP_023590712.1_   77  ----DGYYEAEFG---------------QERRPLFFQNLGIRCVKKKEVKEAVISRIRAGI-NPFSV----  123
_XP_027243803.1_   77  ----DGYYEAEFG---------------PERRPLFFQNLGIRCVKKKEVKEAIILRISAGI-NPFNV----  123
_XP_040589151.1_   77  ----DGYYEAEFG---------------PERRPLFFQNLGIRCVKKKEVKEAIILRISAGI-NPFSV----  123
_XP_023412959.1_   77  ----DGYYEAEFG---------------QERRPLFFQNLGIRCVKKKEVKEAVISRIRAGI-NPFSV----  123
_XP_036057111.1_   77  ----DGYYEAEFG---------------PERRPLFFQNLGIRCVKKKEVKEAIILRISAGI-NPFNV----  123
_XP_027822602.1_   77  ----DGYYEAEFG---------------QERRPLFFQNLGIRCVKKKEVKEAIISRMRAGI-NPFNV----  123
_XP_031322949.1_   77  ----DGYYEAEFG---------------QERRPLFFQNLGIRCVKKKEVKEAIISRIRAGI-NPFNV----  123
_XP_028634966.1_   77  ----DGYYEAEFG---------------PERRPLFFQNLGIRCVKKKEVKEAIILRISAGI-NPFNV----  123
_XP_031531529.1_   77  ----DGYYEAEFG---------------QERRPLFFQNLGIRCVKKKEVKEAIISRIRAGI-NPFSV----  123
_XP_006073937.1_   77  ----DGYYEAEFG---------------QERRPLFFQNLGIRCVKKKEVKDAVISRIRAGI-NPFNV----  123
_XP_034364887.1_   77  ----DGYYEAEFG---------------PERRPLFFQNLGIRCVKKKEVKEAIILRISAGI-NPFNV----  123
_XP_021482246.1_   77  ----DGYYEAEFG---------------PERRPLFFQNLGIRCVKKKEVKEAIILRIGAGI-NPFNV----  123
_KAF4016326.1_     77  ----DGYYEAEFG---------------QERRPLFFQNLGIRCVKKKEVKEAIISRIRAGI-NPFNV----  123
_XP_023602319.1_   77  ----DGYYEAEFG---------------QERRPLFFQNLGIRCVKKKEVKEAIISRIRAGI-NPFNV----  123
_XP_052054164.1_   77  ----DGYYEAEFG---------------PERRPLFFQNLGIRCVKKKEVKEAIILRISAGI-NPFNV----  123
_XP_036291310.1_   77  ----DGYYEAEFG---------------QERRPLFFQNLGIRCVKKKEVKEAIISRIRAGI-NPFNV----  123
_XP_013370168.1_   77  ----DGYYEADFG---------------QERRPLFFQNLGIRCVKKKEVKEAVISRISAGI-NPFNV----  123
_XP_023590722.1_   77  ----DGYYEAEFG---------------QERRPLFFQNLGIRCVKKKEVKEAVISRIRAGI-NPFSV----  123
_XP_048291328.1_   77  ----DGFYEAEFG---------------PERRPLFFQNLGIRCVKKKEVKDAVILRISAGI-NPFNV----  123
_XP_032772910.1_   77  ----DGYYEAEFG---------------PERRPLFFQNLGIRCVKKKEVKEAIILRISAGI-NPFNV----  123
_XP_008768670.2_   77  ----DGYYEAEFG---------------PERRPLFFQNLGIRCVKKKEVKEAIILRISAGI-NPFNV----  123
_XP_014302521.1_   77  ----DGYYEAEFG---------------QERRPLFFQNLGIRCVKKKEVKEAIISRIRAGI-NPFNV----  123
_XP_031194882.1_   77  ----DGYYEAEFG---------------PERRPLFFQNLGIRCVKKKEVKEAIILRISAGI-NPFNV----  123
_NP_033070.2_      77  ----DGYYEAEFG---------------PERRPLFFQNLGIRCVKKKEVKEAIILRISAGI-NPFNV----  123
_XP_036291312.1_   77  ----DGYYEAEFG---------------QERRPLFFQNLGIRCVKKKEVKEAIISRIRAGI-NPFNV----  123
_XP_021033157.1_   77  ----DGYYEAEFG---------------PERRPLFFQNLGIRCVKKKEVKEAIILRISAGI-NPFNV----  123
_XP_015849136.1_   77  ----DGYYEAEFG---------------PERRPLFFQNLGIRCVKKKEVKEAIILRISAGI-NPFNV----  123
_XP_045150027.1_   77  ----DGYYEAEFG---------------QERRPLFFQNLGIRCVKKKEVKEAVISRRRAGI-NPFNV----  123
_XP_021066684.1_   77  ----DGYYEAEFG---------------PERRPLFFQNLGIRCVKKKEVKEAIILRISAGI-NPFNV----  123
_XP_006881088.1_   77  ----DGYYEAEFG---------------QEHRPLFFQNLGIRCVKKKEVKEAVISRIRAGI-NPFNV----  123
```

```
_XP_008524234.1_    74  ----DGYYEAEFG--------------QERRPLFFQNLGIRCVKKKEVKEAIVARIRAGI-NPFNV----  120
_XP_021482260.1_    77  ----DGYYEAEFG--------------PERRPLFFQNLGIRCVKKKEVKEAIILRIGAGI-NPFNV----  123
_XP_007125500.1_    77  ----DGYYEAEFG--------------QERRPLFFQNLGIRCVKKKEVKEAVISRIRAGI-NPFNV----  123
_XP_028352731.1_    77  ----DGYYEAEFG--------------QERRPLFFQNLGIRCVKKKEVKEAVISRIRAGI-NPFNV----  123
_XP_028352733.1_    77  ----DGYYEAEFG--------------QERRPLFFQNLGIRCVKKKEVKEAVISRIRAGI-NPFNV----  123
_XP_028352734.1_    77  ----DGYYEAEFG--------------QERRPLFFQNLGIRCVKKKEVKEAVISRIRAGI-NPFNV----  123
_XP_028352736.1_    77  ----DGYYEAEFG--------------QERRPLFFQNLGIRCVKKKEVKEAVISRIRAGI-NPFNV----  123
_XP_028352735.1_    77  ----DGYYEAEFG--------------QERRPLFFQNLGIRCVKKKEVKEAVISRIRAGI-NPFNV----  123
_ERE88482.1_        77  ----DGYYEAEFG--------------PERRPLFFQNLGIRCVKKKEVKEAIILRISAGI-NPFNV----  123
_KAF3826750.1_       1  ----------------------------------------------------------------KI----    2
_OBS70420.1_        66  ----DGYYEAEFG--------------PERRPLFFQNLGIRCVKKKEVKEAIILRISAGI-NPFNV----  112
_XP_006878094.1_    40  ----DGYYEAEFG--------------QERRPLFFQNLGIRCVKKKEVKEAIISRIRAGI-NPFNV----   86
_XP_008978905.3_    34  ----DGYYEAEFG--------------QERRPLFFQNLGIRCVKKKEVKEAVISRIKAGI-NPFNV----   80
_XP_032147941.1_    34  ----DGYYEAEFG--------------QERRPLFFQNLGIRCVKKKEVKEAIISRIKAGI-NPFNV----   80
_XP_008524233.1_    36  ----DGYYEAEFG--------------QERRPLFFQNLGIRCVKKKEVKEAIVARIRAGI-NPFNV----   82
_XP_044628438.1_    34  ----DGYYEAEFG--------------QERRPLFFQNLGIRCVKKKEVKEAIVARIRAGI-NPFNV----   80
_XP_010608101.1_    34  ----DGYYEAEFG--------------QERRPLFFQNLGIRCVKKKEVKEAVISRISAGI-NPFNV----   80
_KAF5918007.1_      36  ----DGYYEAEFG--------------QERRPLFFQNLGIRCVKKKEVKEAIISRIRAGI-NPFNV----   82
_XP_021101942.1_    34  ----DGYYEAEFG--------------QERRPLFFQNLGIRCVKKKEVKEAVISRISAGI-NPFNV----   80
_ABM89308.1_            ----------------------------------------------------------------------
_MBW00678.1_         1  ----------------------------------FQNLGIRCVKKKEVKEAIISRIRAGI-NPF------   29
_NP_001096177.1_    77  ----DGYYELEFG--------------SDRTVLCFQNLGIQCVRRKEVREAIHTRIMRKM-NPFGV----  123
_OCT77375.1_        77  ----DGYYELEFG--------------SDRTVLCFQNLGIQCVRRREVREAIHARIIRKM-NPFGV----  123
_AAI70187.1_        77  ----DGYYELEFG--------------SDRTVLCFQNLGIQCVRRREVREAIHARIIRKM-NPFGV----  123
_AAI69362.1_        77  ----DGYYELEFG--------------SDRTVLCFQNLGIQCVRRKEVREAIHARILRKM-NPFGV----  123
_XP_041418586.1_    77  ----DGYYELEFG--------------SDRTVLCFQNLGIQCVRRKEVREAIHARILRKM-NPFGV----  123
_XP_041418585.1_    77  ----DGYYELEFG--------------SDRTVLCFQNLGIQCVRRKEVREAIHARILRKM-NPFGV----  123
_XP_041418587.1_    77  ----DGYYELEFG--------------SDRTVLCFQNLGIQCVRRKEVREAIHARILRKM-NPFGV----  123
_XP_053316221.1_    77  ----DGYYEAEFG--------------PDRKVLSFQNLGIQCIRRKEVREAIHNRIVSKI-NPFGV----  123
_XP_053568018.1_    77  ----DGYYEIEFG--------------SDRRVLCFQNLGIQCVRRKEVRDAIHMRMIRKI-NPFGV----  123
_KAG8443975.1_      77  ----DGYYELEFG--------------SDRTVLSFQNLGIQCVRRREVRDAINSRIIRQM-NPFGV----  123
_XP_044145067.1_    79  ----DGFYETEFG--------------ADRRVLCFQNLGIQCVRRREVKDAIHSRMMRKI-NPFGV----  125
_KAG9484268.1_      79  ----DGYYETEFG--------------ADRRVLCFQNLGIQCVRRKEVKDAIHSRMIRKI-NPFAV----  125
_XP_040285660.1_    79  ----DGYYETEFG--------------ADRRVLCFQNLGIQCVRRREVKDAIHSRMMRKI-NPFGV----  125
_XP_040285659.1_    79  ----DGYYETEFG--------------ADRRVLCFQNLGIQCVRRREVKDAIHSRMMRKI-NPFGV----  125
_KAG8583825.1_      79  ----DGYYETEFG--------------ADRRVLCFQNLGIQCVRRREVKDAIHSRMIRKI-NPFGV----  125
_XP_018411072.1_    79  ----DGYYETEFG--------------ADRRVLCFQNLGIQCVRRKEVREAILARKTRQI-NPFGV----  125
_XP_040207073.1_    79  ----DGYYEIEFG--------------ADRRVLCFQNLGIQCVRRREVREAIHTRRNRNI-NPFGV----  125
_NP_001081750.1_    76  ----DGYYELEFG--------------SDRTVLCFQNLGIQCVRRREVREAIHARIFAN--EPFGV----  121
```

```
_PIO39092.1_        1  ----------------------------------------------------------------V-----    1
_PIO39091.1_        1  ----------------------------------------------------------------V-----    1
_XP_041035877.1_   77  ----DGYYEADLP---------------ARSIHSFQNLGIQCVKKREVSLAIQSRISKNV-NPFNI----  122
_XP_042202691.1_   77  ----DGYYEADLQ---------------ERSVHSFQNLGIQCVKKREVSLAIQQRLMKDV-NPFNI----  122
_XP_048475509.1_   66  ----DGYYEADLP---------------ERSVHSFQNLGIQCVKKREVAMAIQSRINKNV-NPFNI----  111
_XP_048380947.1_   66  ----DGYYEADLP---------------ERSVHSFQNLGIQCVKKREVAMAVQSRINKNV-NPFKI----  111
_XP_051901152.1_   70  ----DGYYEADLP---------------ERSVHSFQNLGIQCVKKREVAMALQNRLSKNV-NPFNV----  115
_BAD60879.1_       65  ----DGVCTVKVR---------------GRNTVSFPNIGIQCCKRHDVEESLKLREKIRV-DPFGT----  110
_XP_050405370.1_   77  ----KGVCTVKVK---------------DTTVVSFPHLGIQCAKKKGIEESLKLRKTINV-DPFQT----  122
_XP_009063713.1_   77  ----KGVCTVKVK---------------DTSVISFPHLGIQCAKKKGIQESLDLRKSVNV-DPFQT----  122
_CAB0044847.1_     83  -KCQNGVYLSEIDSSG--------DGEGDTMKVVLKNLAIQRVDERDVAAELEIRKRAKV-DPFQT----  138
_XP_048259911.1_   79  ----KGVCTVKVK---------------DTDTITFPHLGIQCAKKKDVENSLKLRKEINV-DPFQT----  124
_ADI72431.1_       79  ----KGVCTVKVK---------------DTDTITFPHLGIQCAKKKDVENSLKLRKEINV-DPFQT----  124
_CAG5133522.1_     79  ----KGVCTVRVK--------------DTSDKIVFPQIGIQCAKKKDVEGALRLRKEINV-DPFQT----  125
_KAG5688355.1_      1  ------------------------------MHLFFPQLGIQCAKKKDVEEALRLRKEINV-DPFQT----   35
_AMT84567.1_       78  ----KGVCTIKLK---------------GTNTVSFPNIGIQCTTKKEVQSALAQREKIRV-DPFGS----  123
_XP_052276378.1_   77  ----KGVCTLRIR---------------DTATVTFPHLGIQCAKKKDVEGNLKLRQEINV-DPFQT----  122
_XP_052276377.1_   77  ----KGVCTLRIR---------------DTATVTFPHLGIQCAKKKDVEGNLKLRQEINV-DPFQT----  122
_AAK72690.1_       77  ----RGVCTLKVK---------------DTNVISFPHLGIQCAKKKDVENNLKQRKEINV-DPFQS----  122
_XP_011414731.2_   77  ----RGVCTLKVK---------------DTNVISFPHLGIQCAKKKDVENNLKQRKEINV-DPFQS----  122
_XP_052699807.1_   77  ----RGVCTLKVK---------------DTNVISFPHLGIQCAKKKDVENNLKQRKEINV-DPFQS----  122
_NP_001292261.1_   77  ----RGVCTLKVK---------------DTNVISFPHLGIQCAKKKDVENNLKQRKEINV-DPFQS----  122
_XP_048738054.1_   77  ----RGVCTLKVK---------------DTNVISFPHLGIQCAKKKDVESNLKQRKEINV-DPFQS----  122
_XP_052699808.1_   77  ----RGVCTLKVK---------------DTNVISFPHLGIQCAKKKDVENNLKQRKEINV-DPFQS----  122
_XP_022332457.1_   77  ----RGVCTLKVK---------------DTNVISFPHLGIQCAKKKDVENNLKQRKEINV-DPFQS----  122
_ABL63469.1_       76  ----RGVCTLKVK---------------DTNTITFPHLGIQCAKKKDVTDNLRQRKEINV-DPYQS----  121
_XP_033733822.1_   77  ----RGVCTLKVK---------------DTNTVSFPHLGIQCAKKKDVENNLKQRKEINV-DPYQS----  122
_XP_021347355.1_   77  ----RGVCTLKVK---------------DTNTVSFPHLGIQCAKKKDVESNLKQRKEINV-DPYQS----  122
_UOA58607.1_       77  ----RGVCTLKVK---------------DTNTISFPHLGIQCAKKKDVESNLKQRKEINV-DPYQS----  122
_XP_021347356.1_   77  ----RGVCTLKVK---------------DTNTVSFPHLGIQCAKKKDVESNLKQRKEINV-DPYQS----  122
_CAG2186872.1_     76  ----RGVCTLKVK---------------DTNVISFPHLGIQCAKKKDVMDNLKQRKEINV-DPFQS----  121
_XP_052058915.1_   76  ----RGVCTLKVK---------------DTNVISFPHLGIQCAKKKDVMDNLKQRKEINV-DPFQS----  121
_KAJ8309232.1_     77  ----RGVCTLKVK---------------DTNVISFPHLGIQCAKKKDVEGNLRQRQEINV-DPFQS----  122
_UZH84475.1_       77  ----KGVCTLRIR---------------DTNTVTFPHLGIQCAKKKDVEDNLKLRQEINV-DPYQT----  122
_ALA99939.1_       77  ----KGVCTLRIR---------------DTTTVSFPHLGIQCAKKKDVEGNLKMRQDINV-DPYQT----  122
_XP_052767764.1_   77  ----KGVCTLRIR---------------DTSTVSFPHLGIQCAKKKDVDGNLKMRQDINV-DPFQT----  122
_ADD25211.1_       77  ----RGVCTLKVK---------------DTNTISFPHLGIQCAKKKDVESNLKQRKEINV-DPYQS----  122
_XP_052224245.1_   77  ----KGVCTLRIR---------------DTATVTFPHLGIQCAKKKDVEGNLKLRQEINV-DPFXM----  122
_KAJ8314234.1_     77  ----RGVCTIKVT---------------DSNVISFPHLSIQCAKKKDIIDNLKQRQDINV-DPFL-----  121
```

```
_VDH95719.1_        35  ----RGVCTLKVK---------------DTNVISFPHLGIQCAKKKDVMDNLKQRKEINV-DPFQS----   80
_XP_041352913.1_    79  ----KGVCTVKVK---------------ETNVISFPHLGIQCAKKKDVENSLKTRKEINV-DPFQT----  124
_XP_046555158.1_    79  ----KGVCTVKVK---------------DTDTITFPHLGIQCAKKKDVENSLKQRKEINV-DPFQT----  124
_AAW33559.1_        79  ----KGVCTVKVK---------------DTDTITFPHLGIQCAKKKDVENSLKLRKDINV-DPFQT----  124
_ABY87435.1_        66  ----KSVCTVKVK---------------DTDTITFPHLGIQCAKKKDVENSLKLRKDINV-DPFQT----  111
_XP_009028834.1_    78  ----DGVCTVKVK--------------NPSSVITFPNIGIQCCKRHDVEDNLKIREKIRV-DPYST----  124
_XP_025106109.1_    79  ----KGVCTVRVK---------------DASVVSFPQLGIQCAKKKDVEGALSLRKEINV-DPYQT----  124
_NP_001298191.1_    79  ----KGVCTLRVK--------------DTSDKIVFPQIGIQCAKKKDVESSLRLRKEINV-DPYQT----  125
_KAH9519545.1_      79  ----KGVCTLRVK--------------DTSEKIVFPQIGIQCAKKKDVESSLRLRKEINV-DPYQT----  125
_RUS74561.1_        79  ----KGVCTLRVK--------------DTTDKIIFPQIGIQCAKKKDVESALRTRKEINV-DPFQT----  125
_GFO11973.1_        79  ----KGVCTLRVK--------------DTTDKIMFPQIGIQCAKKKDVEGALKLRKEINV-DPYQT----  125
_CAG5132534.1_      79  ----KGVCTLRVK--------------DTSDKIVFPQIGIQCAKKKDVEAALKLRKEINV-DPFQT----  125
_GFR69641.1_        79  ----KGVCTLRVK--------------DTTEKIIFPQIGIQCAKKKDVETALRIRKEINV-DPYQT----  125
_XP_035824873.1_    79  ----KGVCTLRVK--------------DTSEKIVFPNIGIQCAKKKDVEGALRLRKDINV-DPFQT----  125
_AAY27981.1_        77  ----KGVCTLQVS---------------STDSITFPNLGIQCVKKREVVDALNARKSINV-DPYRT----  122
_XP_052827753.1_    76  ----KGVCTVKCS---------------VSDTISFQNLGIQCVKKKEVADALKTRQEINV-DPFKT----  121
_XP_029642523.1_    76  ----KGVCTVKCS---------------VSDTISFQNLGIQCVKKKEVAEALKTRQEINV-DPFKT----  121
_CAE1288013.1_      36  ----KGVCTLKVL---------------NTDSISFANLGIQCVKKREVAEALNARKSINV-DPYQT----   81
_ELU04547.1_        78  ----KGVCTIKLR---------------GTNIVSFPNIGIQCATRKEVQDHLQKRKEIRV-DPFGT----  123
_KAI0227899.1_      78  ----KGVCTVKLT---------------NSNVISFKNLGIQCVKRKQVESSLAMRASVKV-DPFSR----  123
_XP_044002103.1_    81  ----EGVCHFKIT-------------LTDNTDVLFPNLGIQCVKKNEIKTSLETRKELRI-DPFRA----  128
_XP_044002102.1_    79  ---ERGVCHVKIS-------------PNNNMEVSFSNLGIQCVKKKEIGTSFKTRKEIRV-DPFKA----  127
_XP_044002099.1_    79  ---ERGVCHVKIS-------------PNNNMEVSFSNLGIQCVKKKEIGTSFKTRKEIRV-DPFKA----  127
_XP_037026629.1_    77  ----EGICVFNFTT-----------KAASPRRVTFPQLGIQCPKLIEVKENLEKRQAQNI-DPFKT----  126
_CAD7632570.1_      79  ----QGVCTMIVD--------------NSDHLCSFTHLGIQCAKRNEIAARLKLRKQLRI-DPTFG----  125
_CAD7650738.1_      79  ----QGVCTLIVD--------------NTDHLCSFTHLGIQCAKRNEISSRLKLRKQLRV-DPTSA----  125
_XP_054163335.1_    77  ----NGIAKIVID--------------NKDMIATFPSMGIQCAKRNDIRVRLKRRQALQI-DPFDC----  123
_CAD7629985.1_      84  ----NGISKMIVD--------------NKDMIATFPSMGIQCAKRNDIRVRLKRRQALQI-DPFDC----  130
_CAD7652069.1_      77  ----NGISKMIVD--------------NKDMIATFPSMGIQCAKRNDIRVRLKRRQALQI-DPFDC----  123
_XP_017954168.1_    79  --CKSGVYSKKLP--------------PEERRLELQKVGIQCVKKTDVRESLMLREQKNI-DPYHA----  127
_XP_017858583.1_    79  --CKSGVYSKKLP--------------PEERRLELQKVGIQCVKKTDVRESLMLREQKNI-DPFHA----  127
_XP_002001588.1_    79  --CKSGVYSKKLP--------------PEERRLELQKVGIQCVKKTDVRESLMLREQKNI-DPFHA----  127
_XP_017954163.1_    79  --CKSGVYSKKLP--------------PEERRLELQKVGIQCVKKTDVRESLMLREQKNI-DPYHA----  127
_XP_023164563.2_    79  --CKSGVYSKKLP--------------PEERRLELQKVGIQCVKKTDVRESLMAREQKNI-DPYHA----  127
_TDG46702.1_        79  --CKSGVYSKKLP--------------PEERRLELQKVGIQCVKKTDVRESLMLREQKNI-DPYHA----  127
_XP_030564898.1_    79  --CKSGVYSKRLP--------------PEERRLELQKVGIQCVKKTDVRESLLAREQKNI-DPYHA----  127
_XP_015024496.1_    79  --CKSGVYSKRLP--------------PEERRLELQKVGIQCVKKTDVRESLLAREQKNI-DPYHA----  127
_XP_034475238.1_    80  --CKDGIYRRRLG--------------PNEHRFELQKVGIQCVKKSGVHDSLKKRMEKKI-DPFSA----  128
_XP_034475235.1_    80  --CKDGIYRRRLG--------------PNEHRFELQKVGIQCVKKSGVHDSLKKRMEKKI-DPFSA----  128
```

```
_XP_017854415.1_   79  --CKIGVYRKQLP--------------PHERRLELQKVGIQCVKKSDARESLLAREQINV-DPFNA----  127
_XP_017854416.1_   79  --CKIGVYRKQLP--------------PHERRLELQKVGIQCVKKSDARESLLAREQINV-DPFNA----  127
_ALC40089.1_       79  --CKIGVYRKQLP--------------PHERRLELQKVGIQCVKKSDARESLLAREQINV-DPFNA----  127
_XP_030382983.1_   80  --CKKGVYRIMLP--------------PEQNTLELQKVGIQCVKKNEVLSSLTERQNMKV-DPFKA----  128
_XP_030382981.1_   80  --CKKGVYRIMLP--------------PEQNTLELQKVGIQCVKKNEVLSSLTERQNMKV-DPFKA----  128
_XP_001989397.1_   79  --CKSGVYNRLLP--------------PEERRLEMQKVGIQCVKKTDVRESLLAREQKNI-DPYHA----  127
_XP_002067135.2_   80  --CKSGVYSKRLP--------------PEERRLVLQKVGIQCVKKTEVRNSLLEREQRNV-DPYRA----  128
_XP_034475239.1_   79  --SRFGSFVKRLR--------------PDQRILELQ-FGIQCVKRSDVRHSLLQRQQRNI-DPFNV----  126
_KAH8411901.1_     79  --CRFGVYTKRLP--------------PEQRRLELQKVGIQCVKKPEVRESLELRKSRDI-DPFRT----  127
_XP_016963143.1_   80  --CKSGIYQKKLP--------------PEERRLVLQKVGIQCAKKLEMRESLLEREKRNV-DPFNA----  128
_XP_037712361.1_   80  --CKSGIYQKKLP--------------PEERRLVLQKVGIQCAKKLEMRDSLVEREKRNV-DPFNA----  128
_XP_016993627.2_   80  --CKSGIYQKRLP--------------PEERRLVLQKVGIQCAKKLEMRDSLVERERKNV-DPFNA----  128
_XP_016963141.1_   80  --CKSGIYQKKLP--------------PEERRLVLQKVGIQCAKKLEMRESLLEREKRNV-DPFNA----  128
_XP_016993624.2_   80  --CKSGIYQKRLP--------------PEERRLVLQKVGIQCAKKLEMRDSLVERERKNV-DPFNA----  128
_XP_037712358.1_   80  --CKSGIYQKKLP--------------PEERRLVLQKVGIQCAKKLEMRDSLVEREKRNV-DPFNA----  128
_XP_001974449.1_   80  --CKSGIYQKKLP--------------PEERRLVLQKVGIQCAKKLEMRDSLVERERRNI-DPFNA----  128
_AAM48404.1_       80  --CKSGIYQKKLP--------------PEERRLVLQKVGIQCAKKLEMRDSLVERERRNI-DPFNA----  128
_NP_001162999.1_   80  --CKSGIYQKKLP--------------PEERRLVLQKVGIQCAKKLEMRDSLVERERRNI-DPFNA----  128
_XP_026835878.1_   80  --CKSGIYQKKLP--------------PEERRLVLQKVGIQCAKKLEMRDSLVERERRNI-DPFNA----  128
_AAA28465.1_       80  --CKSGIYQKKLP--------------PEERRLVLQKVGIQCAKKLEMRDSLVERERRNI-DPFNA----  128
_XP_002038912.1_   80  --CKSGIYQKKLP--------------PEERRLVLQKVGIQCAKKLEMRDSLVEREKRNI-DPFNA----  128
_XP_033169558.1_   80  --CKSGIYQKKLP--------------PEERRLVLQKVGIQCAKKLEMRDSLVEREKRNI-DPFNA----  128
_EDX05310.1_       80  --CKSGIYQKRLP--------------PEERRLVLQKVGIQCAKKLEMRDSLVEREKRNI-DPFNA----  128
_NP_001162998.1_   80  --CKSGIYQKKLP--------------PEERRLVLQKVGIQCAKKLEMRDSLVERERRNI-DPFNA----  128
_XP_032579766.1_   80  --CKSGIYQKKLP--------------PEERRLVLQKVGIQCAKKLEMRDSLVEREKRNI-DPFNA----  128
_XP_033169541.1_   80  --CKSGIYQKKLP--------------PEERRLVLQKVGIQCAKKLEMRDSLVEREKRNI-DPFNA----  128
_XP_034669502.1_   80  --CKSGVYSKRLP--------------PEERRLVLQKVGIQCAKKLEMRDSLLERERKNV-DPFKA----  128
_XP_034125957.1_   80  --CKSGVYSKRLP--------------PEERRLVLQKVGIQCAKKLEMRDSLLERERKNV-DPFKA----  128
_XP_034669501.1_   80  --CKSGVYSKRLP--------------PEERRLVLQKVGIQCAKKLEMRDSLLERERKNV-DPFKA----  128
_XP_034125956.1_   80  --CKSGVYSKRLP--------------PEERRLVLQKVGIQCAKKLEMRDSLLERERKNV-DPFKA----  128
_XP_034125955.1_   80  --CKSGVYSKRLP--------------PEERRLVLQKVGIQCAKKLEMRDSLLERERKNV-DPFKA----  128
_XP_017152033.1_   80  --CKSGVYAKRLP--------------PEERRLVLQKVGIQCAKKLEMRDSLLERERKNV-DPFGA----  128
_XP_017152031.1_   80  --CKSGVYAKRLP--------------PEERRLVLQKVGIQCAKKLEMRDSLLERERKNV-DPFGA----  128
_XP_002014799.1_   80  --CKSGVYAKRLP--------------PEERRLVLQKVGIQCAKKLEMRESLLERERKNV-DPFGA----  128
_XP_026844902.1_   80  --CKSGVYAKRLP--------------PEERRLVLQKVGIQCAKKLEMRESLLERERKNV-DPFGA----  128
_XP_015035983.2_   80  --CKSGVYAKRLP--------------PEERRLVLQKVGIQCAKKLEMRESLLERERKNV-DPFGA----  128
_XP_017063896.1_   80  --CKSGVYSKRLP--------------PEERRLVLQKVGIQCAKKLEMRDSLVEREKRNV-DPFNA----  128
_XP_016935332.1_   80  --CKSGIYQKKLP--------------PEERRLVLQKVGIQCAKKLEMRDSLVEREKRNV-DPFNA----  128
_XP_017063894.2_   80  --CKSGVYSKRLP--------------PEERRLVLQKVGIQCAKKLEMRDSLVEREKRNV-DPFNA----  128
```

```
_XP_036668637.1_    80  --CKSGIYQKKLP--------------PEERRLVLQKVGIQCAKKLEMRDSLVEREKRNV-DPFNA----  128
_XP_016935310.1_    80  --CKSGIYQKKLP--------------PEERRLVLQKVGIQCAKKLEMRDSLVEREKRNV-DPFNA----  128
_KAH8298318.1_      80  --CKSGVYSKKLP--------------PEERRLVLQKVGIQCAKKLEMRDSLLERERKNV-DPFGA----  128
_KAH8412187.1_      80  --CKSGVYSKRLP--------------PEERRLVLQKVGIQCAKKLEMRDSLLERERKNV-DPFGA----  128
_KAH8278042.1_      79  --CKSGIYSKRLP--------------PEERRLVLQKVGIQCAKKLEMRESLVERERKNV-DPFEA----  127
_KAH8366423.1_      80  --CKSGIYQKRLP--------------PEERRLVLQKVGIQCAKKLEMRDSLVERQKKNV-DPFNA----  128
_XP_017044860.1_    79  --CKSGIYQKRLP--------------PGERKLVLQKVGIQCAKKTEMRDSLVAREKMDV-DPFNT----  127
_KAH8324526.1_      78  --CKSGIYSKRLP--------------PEERRLVLQKVGIQCAKKLEMRESLEERERKNF-DPFGA----  126
_XP_017116817.1_    80  --CKSGIYRKRLE--------------PQERRLVLQKVGIQCAKKLEMRDSLVEREQRNV-DPFCA----  128
_XP_041566118.1_    80  --CKSGIYRKRLE--------------PQERRLVLQKVGIQCAKKLEMRDSLVEREQRNV-DPFCA----  128
_XP_052852584.1_    80  --CKSGIYRKRLE--------------PQERRLVLQKVGIQCAKKLEMRDSLVEREQRNV-DPFCA----  128
_XP_052852582.1_    80  --CKSGIYRKRLE--------------PQERRLVLQKVGIQCAKKLEMRDSLVEREQRNV-DPFCA----  128
_XP_017107132.2_    79  --CKSGIYSKRLP--------------PEERRLVLQKVGIQCAKKLDMRESLVKRERMNV-DPFGA----  127
_XP_043069650.1_    79  --CKSGIYSKRLP--------------PEERRLVLQKVGIQCAKKLDMRESLVKRERMNV-DPFGA----  127
_KAH8283158.1_      80  --CKSGVYSKRLP--------------PEERRLVLQKVGIQCAKKLEMRDSLLERERKNV-DPFGA----  128
_XP_017025640.1_    79  --CKSGVYSKRLP--------------PEERRLVLQKVGIQCAKKLEMRDSLLERERKNV-DPFGA----  127
_XP_020803071.1_    80  --CKSGVYSKRLP--------------PEERRLVLQKVGIQCAKKLEMRDSLLERERKNV-DPFGA----  128
_XP_017025638.1_    80  --CKSGVYSKRLP--------------PEERRLVLQKVGIQCAKKLEMRDSLLERERKNV-DPFGA----  128
_KAH8308067.1_      80  --CKSGVYSKRLP--------------PEERRLVLQKVGIQCAKKLEMRDSLLERERKNV-DPFGA----  128
_KAH8362606.1_      80  --CKSGVYSKRLP--------------PEERRLVLQKVGIQCAKKLEMRDSLLERERKNV-DPFGA----  128
_XP_020803068.1_    80  --CKSGVYSKRLP--------------PEERRLVLQKVGIQCAKKLEMRDSLLERERKNV-DPFGA----  128
_XP_015052364.1_    79  --CKSGVYQKILQ--------------PEERRLVLQKVGIKCVKKSDMFESLAVRQRRGI-DPFNA----  127
_XP_002090469.1_    79  --CKSGVYQKILQ--------------PEERRLVLQKVGIKCVKKSDMFESLAVRQRRGI-DPFNA----  127
_XP_039480354.1_    79  --CKSGVYQKILQ--------------PEERRLVLQKVGIQCVKKPDMFESLAVRQRKEI-DPFNA----  127
_XP_039480352.1_    79  --CKSGVYQKILQ--------------PEERRLVLQKVGIQCVKKPDMFESLAVRQRKEI-DPFNA----  127
_XP_041450795.1_    80  --CKSGVYSKRLP--------------PEERRLVLQKVGIQCAKKLEMRDSLLERERKNV-DPFLA----  128
_XP_044315461.1_    80  --CKSGYYRKHLK--------------PGERRLVLQKVGIQCAKKLEMRDSLVERERKNI-DPFGA----  128
_XP_044315463.1_    80  --CKSGYYRKHLK--------------PGERRLVLQKVGIQCAKKLEMRDSLVERERKNI-DPFGA----  128
_XP_001961710.2_    80  --CRSGTYSKRLP--------------PEERRLVLQKVGIQCAKKLEMRDSLLERERINV-DPFGA----  128
_XP_014761890.1_    80  --CRSGTYSKRLP--------------PEERRLVLQKVGIQCAKKLEMRDSLLERERINV-DPFGA----  128
_KAH8323309.1_      80  --CRSGTYSKRLP--------------PEERRLVLQKVGIQCAKKLEMRDSLLERERINV-DPFGA----  128
_XP_043646206.1_    79  --CKSGVYQKKLQ--------------PEERRLVLQKVGIQCVKKSEMVESLAERQRRKI-DPFDA----  127
_XP_043646204.1_    79  --CKSGVYQKKLQ--------------PEERRLVLQKVGIQCVKKSEMVESLAERQRRKI-DPFDA----  127
_AAQ65066.1_        80  --CKSGIYQKKLP--------------PEERRLVLQKVGIQCAKKLEMRDSLVEREKRNI-DPFNA----  128
_AAQ64900.1_        80  --CKSGIYQKRLP--------------PEERRLVLQKVGIQCAKKLEMRDSLVEREKRNI-DPFNA----  128
_AAQ64905.1_        80  --CKSGIYQKRLP--------------PEERRLVLQKVGIQCAKKLEMRDSXVEREKRNX-DPFNA----  128
_KAH8378209.1_      79  --CKTGAYIKRLK--------------PGEQRLELQKVGIQCTKKTEVAEALSKRKEKNI-DPYRA----  127
_KAH8293207.1_      79  --CKNGAYIKRLR--------------PGEQRLELQRVGIQCTKKTEVAQALEKRKEKNI-DPFKV----  127
_KAH8391978.1_      78  --CKAGAYIKRLK--------------PGEQRLELQKVGIQCTKKTEVAEALKKRKEKNI-DPFRA----  126
```

```
_XP_034101352.1_    78  --CKAGAYIKRLK--------------PGEQRLELQKVGIQCTKKTEVAEALKKRKEKNI-DPFRA----  126
_KAJ3658269.1_      75  ----YGVCSFEQEV------------TATNSTITFPKLAVQCVKKADVKESLRVREEKNI-DPFER----  123
_KAJ3658270.1_      75  ----HGVCSFEQEV------------TATNSTITFAKMGARCIKRSNFQESLRIRQENNV-DPFEE----  123
_KAJ3658271.1_      75  ----FGVCYFEEEV------------TAASSTITLPKMAVRCLKRAELKESLRVREEKNV-DPFQR----  123
_XP_015117085.1_    78  ---NNGVFVTTVG--------------PDENEIELKNLGIQCVRKKDIKKSLEERKKVRV-DPFQQ----  125
_XP_011307643.1_    78  ---NHGVFTITAG--------------PDTNEIELKNLGIQCVRKRDIKKSLEERKRVRV-DPFQQ----  125
_XP_011307741.1_    77  ---YHGIFTTALD--------------PNCNEIAFKHLGIQCVKRKDIRKSLEERKQTGV-DPFG-----  123
_XP_015119445.1_    78  ---NHGVFATTVR--------------PDDNEIELKNIGIQCVRKRDVKKSLDERKEIRV-DPFQQ----  125
_XP_052128594.1_    72  ----WGVYQKQVH----------------SSVISFQKLSVQFTKRKHIEEVMLVRQQKNI-DPFQIKFNF  120
_XP_052128597.1_    72  ----WGVYQKQVH----------------SSVISFQKLSVQFTKRKHIEEVMLVRQQKNI-DPFQ-----  115
_KAE8741315.1_          ----------------------------------------------------------------------
_XP_028982160.1_    81  -RLFPGIISAPVE--------------PHKPEVTFSTVKLQCVPKEDAKRALMTRFTFPL-NPFPC----  130
_XP_011307645.1_    81  -KMYPGIINAPVD--------------PNHPEVTFTTVKLQCVPREDAKRALMSRVTFPM-NPFPC----  130
_XP_054725384.1_    78  ----HGVCILQIP-----------------HRAEFHSLGIKSTKRSDVDMALKKRESIRV-DPFET----  121
_XP_053965385.1_    78  ----HGVCILQIP-----------------HRAEFQSLGIKSTKRSDVDMALKKRESIHV-DPFKT----  121
_XP_013149158.1_    81  -EVKKGACKVVVEL------------TGESTKVPFTNIGIHSCKHNEVKEVLETRRKLGV-DPFNS----  132
_XP_045534655.1_    81  -EVKKGACKVVVEL------------TGESTKVSFTNIGIHSCKHNEVKEVLETRRRLGV-DPFGS----  132
_KPJ06852.1_        81  -EVKKGACKVVVEL------------TGESTKVSFTNIGIHSCKHNEVKEVLETRRRLGV-DPFGS----  132
_XP_013176418.1_    81  -EVRKGACKVVVEL------------TGESTKVSFNNIGIHSCKQNEVKEVLETRRSLGV-DPFGS----  132
_OXU28425.1_        70  ---NHGVCLFYVH--------------RPKKEIHFKKLFVETLNEEDVFESLTERERKKV-DPFKSKFHI  121
_XP_008216913.1_    69  ---KHGVCHIYVH--------------RTKKQIHFKNLFVESVNVEDVVESLTERERKKV-DPFKIVL--  118
_XP_008216914.1_    69  ---KHGVCHIYVH--------------RTKKQIHFKNLFVESVNVEDVVESLTERERKKV-DPFK-----  115
_XP_040572301.1_    78  ---KQSVCSRNVD--------------VNTMTCSFENIGIQCIKRHEIPDSLEERRSIKV-DPFNQ----  125
_CAB4068408.1_      46  ---KQSVCSRNVD----------------------------------IPWKRDGR---------------   63
_KAF7993284.1_      40  ---TGFTCLFKYV--------------KNNEIINFPNLVIKKCKKVNFKDALIMREKMEI-DPFKA----   87
_KAF7987489.1_      80  ----DGTGIRVIDFK----------IMNNTVILECTGLGIQNTRRRDIKKIIEQREKMEF-DPFKA----  130
_KAF7994520.1_      82  ----NNYNSRVIDFE----------INDSSIVIECSNLAIQHAKKSDVKKILESYEKINM-DPFKA----  132
_XP_044019368.1_    81  ----NCIGFRILKFTM-------NDNDNNFIFIECKNLCIEYTKKHDVEKVLESYERLKF-DPFKA----  134
_XP_044019332.1_     1  -----------------------------TVILECTGLGIQNTRRRDIKKIIEQREKMEF-DPFKA----   36
_XP_044003986.1_     1  -------------------------------------------------------------DPFKA----    5
_XP_037042732.1_    76  ----NGVCYIPQFYT----------DSRDPKVISFRELRIQSCLISEIPARLAERTRLNI-NPFDL----  126
_CAD7223243.1_      79  ----GGVCTLPVE--------------DQDMTLSFTSLGIQCSKRNEFAKVLEERRAAGI-DPFNN----  125
_XP_044732233.1_    83  AVCNEGIYELAIE--------------NSSDGITFPNLGLHCVKKGDIKGVLETRAQYGV-DPFGT----  133
_XP_012231885.1_    76  ----QGVCTVEVP--------------AGNMVVTFSNLSIQCVTRMGIKNALKERQELRV-DPFGT----  122
_XP_022814699.1     83  ----SYVYSKEVEL------------RPGNNTIVFNDIRIIRVKTADYKHALRIREVQRI-DPFQQ----  131
_CAB3516523.1_      83  ----SYVYSKEVEL------------RPGNNMIVFNDLRIIRVKTADYKHALRIREVQKV-DPFQK----  131
_XP_035458245.2_    82  ----SYVYSKDVPL------------RPGNNTVVFDDLRIIRVKSVDYKNALRIREVQKI-DPFQQ----  130
_KAF9795795.1_      71  ----SYVFSKDVPL------------RPGNNTVVFDDLRIIRVKSVDYKNALRIREVQKI-DPFQQ----  119
_XP_014233257.1_    74  ---SKGICYFRAR-------------AEPGKPIELRNLRVKKLAADSLRASLLDRRNLCY-DPFQT----  122
```

```
_XP_034324400.1_    59  ----NGLYVEKFIP------------TTENKCTLSCKATIKIPKRGEYEDELAKRKQAME-KYMGI----  107
_XP_052699907.1_    59  ----NGLYVEKFIP------------TTENKCTLSCKATIKIPKRGEYEDELAKRKQAME-KYMGI----  107
Consensus aa:           ....pGhhp..h.................p..h.h.pltIpthK+p-l.psl..Rbp..l.sPFph....
Consensus ss:                 eeeeee                   eeee   eeeeeehhhhhhhhhhhhh

Conservation:
_XP_001652840.2_   125  -----------------------------------------GYGHARQPA---------------TIDLN  138
_AAW67214.1_       125  -----------------------------------------GYGHARQPA---------------TIDLN  138
_XP_021704027.1_   125  -----------------------------------------GYGHARQPA---------------TIDLN  138
_XP_019540833.2_   125  -----------------------------------------GYGHAKQPA---------------TIDLN  138
_XP_029720231.1_   125  -----------------------------------------GYGHAKQPA---------------TIDLN  138
_AAW67215.1_       125  -----------------------------------------GYGHARQPA---------------TIDLN  138
_XP_021704025.1_   125  -----------------------------------------GYGHARQPA---------------TIDLN  138
_XP_019540831.2_   125  -----------------------------------------GYGHAKQPA---------------TIDLN  138
_KXJ81105.1_       125  -----------------------------------------GYGHAKQPA---------------TIDLN  138
_KXJ76497.1_       125  -----------------------------------------GYGHAKQPA---------------TIDLN  138
_XP_023240428.1_   125  -----------------------------------------GFSHSSIIN---------------DLDLS  138
_CAD7241875.1_     126  -----------------------------------------GFGHKSQPG---------------SIDLS  139
_TRY80291.1_       126  -----------------------------------------GFDHKNR-----------------SIDLN  137
_ALF84899.1_       124  -----------------------------------------GFKHKTA-----------------AINLN  135
_KAF4528208.1_     124  -----------------------------------------GFSHRSQPT---------------SIDLN  137
_XP_054260955.1_   125  -----------------------------------------GFSHRTQAG---------------SIDLN  138
_XP_054264401.1_   125  -----------------------------------------GFSHRTQAG---------------SIDLN  138
_XP_054260954.1_   125  -----------------------------------------GFSHRTQAG---------------SIDLN  138
_XP_038114042.1_   125  -----------------------------------------GYSHAKQPA---------------TIDLN  138
_XP_039437558.1_   125  -----------------------------------------GYSHAKQPA---------------TIDLN  138
_XP_001844078.2_   125  -----------------------------------------GYSHAKQPA---------------TIDLN  138
_EDS35747.1_       125  -----------------------------------------GYSHAKQPA---------------TIDLN  138
_XP_053696925.1_   125  -----------------------------------------GYSHAKQPA---------------TIDLN  138
_XP_001869619.2_   125  -----------------------------------------GFGHVKQLA---------------TIDLN  138
_XP_039434954.1_   125  -----------------------------------------GFGHVKQLA---------------TIDLN  138
_EDS29879.1_       125  -----------------------------------------GFGHVKQLA---------------TIDLN  138
_XP_019550742.2_   125  -----------------------------------------GYGHGKQPA---------------SIDLN  138
_KXJ74587.1_       125  -----------------------------------------GYGHGKQPA---------------SIDLN  138
_XP_035908773.1_   126  -----------------------------------------GFGHAKEPG---------------SIDLN  139
_AJC98655.1_       126  -----------------------------------------GFGHAKEPG---------------SIDLN  139
_AJC98650.1_       126  -----------------------------------------GFGHAKEPG---------------SIDLN  139
_AJC98644.1_       126  -----------------------------------------GFGHAKEPG---------------SIDLN  139
```

```
_AJC98657.1_      126 -----------------------------------------GFGHAKEPG---------------SIDLN 139
_AJC98648.1_      126 -----------------------------------------GFGHAKEPG---------------SIDLN 139
_AJC98647.1_      126 -----------------------------------------GFGHAKEPG---------------SIDLN 139
_AJC98651.1_      126 -----------------------------------------GFGHAKEPG---------------SIDLN 139
_AJC98643.1_      126 -----------------------------------------GFGHAKEPG---------------SIDLN 139
_AJC98656.1_      126 -----------------------------------------GFGHAKEPG---------------SIDLN 139
_AJC98641.1_      126 -----------------------------------------GFGHAKEPG---------------SIDLN 139
_AJC98640.1_      126 -----------------------------------------GFGHAKEPG---------------SIDLN 139
_AJC98653.1_      126 -----------------------------------------GFGHAKEPG---------------SIDLN 139
_AJC98654.1_      126 -----------------------------------------GFGHAKEPG---------------SIDLN 139
_AJC98652.1_      126 -----------------------------------------GFGHAKEPG---------------SIDLN 139
_AJC98646.1_      126 -----------------------------------------GFGHAKEPG---------------SIDLN 139
_AJC98658.1_      126 -----------------------------------------GFGHAKEPG---------------SIDLN 139
_AJC98639.1_      126 -----------------------------------------GFGHAKEPG---------------SIDLN 139
_XP_035908772.1_  126 -----------------------------------------GFGHAKEPG---------------SIDLN 139
_XP_050076803.1_  126 -----------------------------------------GFGHAKEPG---------------SIDLN 139
_XP_049282441.1_  126 -----------------------------------------GFGHAKEPG---------------SIDLN 139
_XP_040170080.1_  126 -----------------------------------------GFGHAKEPG---------------SIDLN 139
_XP_050076802.1_  126 -----------------------------------------GFGHAKEPG---------------SIDLN 139
_XP_041780138.1_  126 -----------------------------------------GFGHAKEPG---------------SIDLN 139
_XP_040239229.2_  126 -----------------------------------------GFGHAKEPG---------------SIDLN 139
_XP_040239222.2_  126 -----------------------------------------GFGHAKEPG---------------SIDLN 139
_XP_035908780.1_  126 -----------------------------------------GFGHAKEPG---------------SIDLN 139
_XP_040239221.2_  126 -----------------------------------------GFGHAKEPG---------------SIDLN 139
_XP_052897786.1_  126 -----------------------------------------GFGHAKEPG---------------SIDLN 139
_XP_052895493.1_  126 -----------------------------------------GFGHAKEPG---------------SIDLN 139
_XP_053664395.1_  126 -----------------------------------------GFGHAKEPG---------------SIDLN 139
_XP_050076801.1_  126 -----------------------------------------GFGHAKEPG---------------SIDLN 139
_XP_310177.3_     126 -----------------------------------------GFGHAKEPG---------------SIDLN 139
_XP_001652339.1_  125 -----------------------------------------GYGHGRQPA---------------SIDLN 138
_XP_053686256.1_  125 -----------------------------------------KFGHAKQPA---------------TIDLN 138
_XP_053675540.1_  126 -----------------------------------------GFAHAKEPG---------------SIDLN 139
_ETN66814.1_      125 -----------------------------------------GFNHATEAS---------------SIDLN 138
_XP_049534501.1_  125 -----------------------------------------GFNHATEAS---------------SIDLN 138
_XP_050084431.1_  125 -----------------------------------------GFNHATEAS---------------SIDLN 138
_XP_035782874.1_  125 -----------------------------------------GFNHATEAS---------------SIDLN 138
_XP_035782869.1_  125 -----------------------------------------GFNHATEAS---------------SIDLN 138
_KFB39849.1_      133 -----------------------------------------GFGHAKEPG---------------SIDLN 146
_XP_052871375.1_  126 -----------------------------------------GFAHAKEPG---------------SIDLN 139
```

```
_AAR13743.1_      111  -----------------------------------------GFGHAKEPG---------------SIDLN  124
_AAR13744.1_      111  -----------------------------------------GFGHAKEPG---------------SIDLN  124
_CAA65157.1_      105  -----------------------------------------GFGHAKEPG---------------SIDLN  118
_XP_022170156.1_  125  -----------------------------------------GFSHKENPT---------------SIDLN  138
_XP_025205168.1_  125  -----------------------------------------GFSHKDNPT---------------SIDLN  138
_XP_026817162.1_  125  -----------------------------------------GFSHKDNPT---------------SIDLN  138
_XP_027846205.1_  125  -----------------------------------------GFSHKDNPT---------------SIDLN  138
_XP_015370635.1_  125  -----------------------------------------GFLHKENPT---------------SIDLN  138
_CAH1723797.1_    125  -----------------------------------------GFSHKDNPT---------------SIDLN  138
_KAF0769940.1_    125  -----------------------------------------GFSHKDNPT---------------SIDLN  138
_CAB4065202.1_     78  -----------------------------------------GFAHKNS-----------------AVNLN   89
_XP_050440903.1_  125  -----------------------------------------GFSHKDNPS---------------SIDLN  138
_XP_050533799.1_  125  -----------------------------------------GFSHKDNPS---------------SIDLN  138
_XP_025410632.1_  125  -----------------------------------------GFSHKDNPA---------------SIDLN  138
_XP_008180746.1_  125  -----------------------------------------GFSHKENPT---------------SIDLN  138
_XP_026465450.1_  126  -----------------------------------------GFSHASEPS---------------SIDLN  139
_VVC36026.1_      125  -----------------------------------------GFSHKEIPT---------------SIDLN  138
_XP_016661522.1_  125  -----------------------------------------GFSHKENPT---------------SIDLN  138
_XP_001947429.3_  125  -----------------------------------------GFSHKENPT---------------SIDLN  138
_XP_026466007.1_  126  -----------------------------------------GFSHASEPS---------------SIDLN  139
_KRT81254.1_      122  -----------------------------------------GFGHESELT---------------SIDLN  135
_XP_033224832.1_  128  -----------------------------------------GFAHSKTRG---------------NFALN  141
_XP_025407590.1_  123  -----------------------------------------GYSHRD-------------------FDYN  132
_XP_045476729.1_  122  ------------------------------------------TGIDLNAA---------------NYDLG  134
_XP_034174253.1_  125  -----------------------------------------GFEHKRQPT---------------SIDLN  138
_XP_029047866.1_  125  -----------------------------------------GFEHKRQPT---------------SIDLN  138
_XP_015430648.1_  125  -----------------------------------------GFEHKRQPT---------------SIDLN  138
_XP_017798081.1_  125  -----------------------------------------GFEHKRQPT---------------SIDLN  138
_XP_017756471.1_  125  -----------------------------------------GFEHKRQPT---------------SIDLN  138
_XP_043248447.1_  125  -----------------------------------------GFEHKRQPT---------------SIDLN  138
_XP_029047855.1_  125  -----------------------------------------GFEHKRQPT---------------SIDLN  138
_XP_034174252.1_  125  -----------------------------------------GFEHKRQPT---------------SIDLN  138
_XP_031829337.1_  125  -----------------------------------------GFEHKRQPT---------------SIDLN  138
_KZC09125.1_      125  -----------------------------------------GFEHKRQPT---------------SIDLN  138
_XP_033327213.1_  125  -----------------------------------------GFEHKRQPT---------------SIDLN  138
_XP_033327212.1_  125  -----------------------------------------GFEHKRQPT---------------SIDLN  138
_XP_017756445.1_  125  -----------------------------------------GFEHKRQPT---------------SIDLN  138
_OAD62393.1_      125  -----------------------------------------GFEHKRQPT---------------SIDLN  138
_XP_043248413.1_  125  -----------------------------------------GFEHKRQPT---------------SIDLN  138
```

```
_XP_033327208.1_  125  -----------------------------------------GFEHKRQPT---------------SIDLN  138
_XP_034950477.1_  125  -----------------------------------------GFDHKRQPA---------------SIDLN  138
_XP_034950475.1_  125  -----------------------------------------GFDHKRQPA---------------SIDLN  138
_XP_012343274.1_  125  -----------------------------------------GFEHKRQPT---------------SIDLN  138
_XP_016909449.1_  125  -----------------------------------------GFEHKRQPT---------------SIDLN  138
_XP_012343273.1_  125  -----------------------------------------GFEHKRQPT---------------SIDLN  138
_XP_006619742.1_  125  -----------------------------------------GFEHKRQPT---------------SIDLN  138
_XP_006567065.1_  125  -----------------------------------------GFEHKRQPT---------------SIDLN  138
_XP_006619741.1_  125  -----------------------------------------GFEHKRQPT---------------SIDLN  138
_NP_001011577.1_  125  -----------------------------------------GFEHKRQPT---------------SIDLN  138
_XP_016909448.1_  125  -----------------------------------------GFEHKRQPT---------------SIDLN  138
_XP_043802365.1_  125  -----------------------------------------GFEHKRQPT---------------SIDLN  138
_NP_001164477.1_  125  -----------------------------------------GFEHKRQPT---------------SIDLN  138
_XP_016909443.1_  125  -----------------------------------------GFEHKRQPT---------------SIDLN  138
_XP_043802361.1_  125  -----------------------------------------GFEHKRQPT---------------SIDLN  138
_XP_012343269.1_  125  -----------------------------------------GFEHKRQPT---------------SIDLN  138
_XP_006619738.1_  125  -----------------------------------------GFEHKRQPT---------------SIDLN  138
_PBC29878.1_      125  -----------------------------------------GFEHKRQPT---------------SIDLN  138
_XP_046663984.1_  125  -----------------------------------------GFSHRSQPG---------------SIDLN  138
_XP_046663985.1_  125  -----------------------------------------GFSHRSQPG---------------SIDLN  138
_XP_043274771.1_  125  -----------------------------------------GFEHKKQPT---------------SIDLN  138
_XP_043274769.1_  125  -----------------------------------------GFEHKKQPT---------------SIDLN  138
_XP_043289571.1_  125  -----------------------------------------GFDHKRQPS---------------SVDLN  138
_XP_032665873.1_  125  -----------------------------------------GFEHKKQPT---------------NIDLN  138
_XP_032665875.1_  125  -----------------------------------------GFEHKKQPT---------------NIDLN  138
_XP_032665874.1_  125  -----------------------------------------GFEHKKQPT---------------NIDLN  138
_XP_032665869.1_  125  -----------------------------------------GFEHKKQPT---------------NIDLN  138
_XP_014470289.1_  125  -----------------------------------------GFEHKKQPT---------------NIDLN  138
_XP_011149046.1_  125  -----------------------------------------GFEHKKQPT---------------NIDLN  138
_XP_017877914.1_  124  -----------------------------------------GFDHKRHPT---------------SIDLN  137
_XP_017877911.1_  124  -----------------------------------------GFDHKRHPT---------------SIDLN  137
_XP_033174774.1_  125  -----------------------------------------GFEHKRQPT---------------SIDLN  138
_XP_043581013.1_  125  -----------------------------------------GFEHKRQPT---------------SIDLN  138
_XP_048261507.1_  125  -----------------------------------------GFEHKRQPT---------------SIDLN  138
_XP_050573946.1_  125  -----------------------------------------GFEHKRQPT---------------SIDLN  138
_XP_050478075.1_  125  -----------------------------------------GFEHKRQPT---------------SIDLN  138
_XP_033204249.1_  125  -----------------------------------------GFEHKRQPT---------------SIDLN  138
_XP_033358451.1_  125  -----------------------------------------GFEHKRQPT---------------SIDLN  138
_XP_033358450.1_  125  -----------------------------------------GFEHKRQPT---------------SIDLN  138
```

```
_XP_033204244.1_  125  -----------------------------------------GFEHKRQPT---------------SIDLN  138
_XP_033312956.1_  125  -----------------------------------------GFEHKRQPT---------------SIDLN  138
_XP_012236639.1_  125  -----------------------------------------GFEHKRQPT---------------SIDLN  138
_XP_050573940.1_  125  -----------------------------------------GFEHKRQPT---------------SIDLN  138
_XP_012174118.1_  125  -----------------------------------------GFEHKRQPT---------------SIDLN  138
_XP_043581010.1_  125  -----------------------------------------GFEHKRQPT---------------SIDLN  138
_EZA51108.1_      125  -----------------------------------------GFEHKKHPT---------------NIDLN  138
_XP_011344085.1_  125  -----------------------------------------GFEHKKHPT---------------NIDLN  138
_XP_043519475.1_  125  -----------------------------------------GFEHKKQPT---------------SIDLN  138
_CAD1481114.1_    125  -----------------------------------------GFEHKKQPT---------------SIDLN  138
_XP_043519473.1_  125  -----------------------------------------GFEHKKQPT---------------SIDLN  138
_KOX75360.1_      125  -----------------------------------------GFEHKKQPT---------------SIDLN  138
_KAF3423741.1_    125  -----------------------------------------GFEHKKQPT---------------SIDLN  138
_KOC69050.1_      125  -----------------------------------------GFEHKRQPT---------------SIDLN  138
_XP_053980095.1_  125  -----------------------------------------GFDHKRQPS---------------NIDLN  138
_XP_054002893.1_  125  -----------------------------------------GFDHKRQPS---------------NIDLN  138
_XP_053980091.1_  125  -----------------------------------------GFDHKRQPS---------------NIDLN  138
_XP_028049144.1_  125  -----------------------------------------GFDHKRHPS---------------NIDLN  138
_XP_012541458.1_  125  -----------------------------------------GFDHKRHPS---------------NIDLN  138
_XP_003708412.1_  125  -----------------------------------------GFEHKRQPT---------------SIDLN  138
_XP_012152807.1_  125  -----------------------------------------GFEHKRQPT---------------SIDLN  138
_KAH0945556.1_    125  -----------------------------------------GFEHKKHPT---------------NIDLN  138
_XP_024871832.1_  125  -----------------------------------------GFEHKRHPT---------------NIDLN  138
_XP_011307641.1_  124  -----------------------------------------GYDHKRQPT---------------SIDLN  137
_XP_015118260.1_  124  -----------------------------------------GYEHKRQPT---------------SIDLN  137
_XP_011307640.1_  124  -----------------------------------------GYDHKRQPT---------------SIDLN  137
_XP_015118252.1_  124  -----------------------------------------GYEHKRQPT---------------SIDLN  137
_EFN68841.1_      129  -----------------------------------------GFEHKRHPT---------------SIDLN  142
_XP_011647340.1_  125  -----------------------------------------GFEHKRHPT---------------NIDLN  138
_XP_029166122.1_  129  -----------------------------------------GFEHKRHPT---------------SIDLN  142
_KMQ96453.1_      129  -----------------------------------------GFEHKRHPT---------------SIDLN  142
_XP_050464492.1_  129  -----------------------------------------GFEHKRHPT---------------SIDLN  142
_XP_029673315.1_  129  -----------------------------------------GFEHKRHPT---------------SIDLN  142
_XP_025265518.1_  129  -----------------------------------------GFEHKRHPT---------------SIDLN  142
_XP_011255523.1_  129  -----------------------------------------GFEHKRHPT---------------SIDLN  142
_XP_011698133.1_  125  -----------------------------------------GFDHRRHPT---------------QIDLN  138
_XP_011698131.1_  125  -----------------------------------------GFDHRRHPT---------------QIDLN  138
_XP_018357420.1_  125  -----------------------------------------GFEHRRHPT---------------NIDLN  138
_KYM76140.1_      125  -----------------------------------------GFEHRRHPT---------------NIDLN  138
```

```
_XP_011647339.1_  125  -----------------------------------------GFEHKRHPT---------------NIDLN  138
_KAG5315434.1_    125  -----------------------------------------GFEHRRHPT---------------NIDLN  138
_XP_011055463.1_  125  -----------------------------------------GFEHRRHPT---------------NIDLN  138
_XP_018342550.1_  125  -----------------------------------------GFEHRRHPT---------------NIDLN  138
_XP_018403565.1_  125  -----------------------------------------GFEHKRHPT---------------NIDLN  138
_KAG5322912.1_    125  -----------------------------------------GFEHRRHPT---------------NIDLN  138
_XP_018357417.1_  125  -----------------------------------------GFEHRRHPT---------------NIDLN  138
_XP_018314560.1_  125  -----------------------------------------GFEHRRHPT---------------NIDLN  138
_XP_018057217.1_  125  -----------------------------------------GFEHRRHPT---------------NIDLN  138
_KAG5346558.1_    125  -----------------------------------------GFEHRRHPT---------------NIDLN  138
_XP_047348408.1_  125  -----------------------------------------GFEHKKQPT---------------SIDLN  138
_XP_047348412.1_  125  -----------------------------------------GFEHKKQPT---------------SIDLN  138
_XP_046820611.1_  125  -----------------------------------------GFEHKKQPT---------------SIDLN  138
_XP_050848254.1_  125  -----------------------------------------GFEHKKQPT---------------SIDLN  138
_XP_046820612.1_  125  -----------------------------------------GFEHKKQPT---------------SIDLN  138
_XP_050848252.1_  125  -----------------------------------------GFEHKKQPT---------------SIDLN  138
_XP_043667564.1_  125  -----------------------------------------GFEHKKQPT---------------SIDLN  138
_XP_035725337.1_  125  -----------------------------------------GFEHKKQPT---------------SIDLN  138
_XP_043667562.1_  125  -----------------------------------------GFEHKKQPT---------------SIDLN  138
_XP_046820605.1_  125  -----------------------------------------GFEHKKQPT---------------SIDLN  138
_XP_050848249.1_  125  -----------------------------------------GFEHKKQPT---------------SIDLN  138
_XP_043667560.1_  125  -----------------------------------------GFEHKKQPT---------------SIDLN  138
_XP_035725333.1_  125  -----------------------------------------GFEHKKQPT---------------SIDLN  138
_KAF7405659.1_    125  -----------------------------------------GFEHKKQPT---------------SIDLN  138
_KAF7402294.1_    125  -----------------------------------------GFEHKKQPT---------------SIDLN  138
_XP_015172825.1_  125  -----------------------------------------GFEHRKQPT---------------SIDLN  138
_XP_015172824.1_  125  -----------------------------------------GFEHRKQPT---------------SIDLN  138
_XP_015172823.1_  125  -----------------------------------------GFEHRKQPT---------------SIDLN  138
_XP_014609306.1_  125  -----------------------------------------GFEHRKQPT---------------SIDLN  138
_XP_043491219.1_  125  -----------------------------------------GFEHRKQPT---------------SIDLN  138
_XP_014609305.1_  125  -----------------------------------------GFEHRKQPT---------------SIDLN  138
_XP_043491218.1_  125  -----------------------------------------GFEHRKQPT---------------SIDLN  138
_KAI4498556.1_    125  -----------------------------------------GFEHRKQPT---------------SIDLN  138
_XP_015172818.1_  125  -----------------------------------------GFEHRKQPT---------------SIDLN  138
_XP_043491211.1_  125  -----------------------------------------GFEHRKQPT---------------SIDLN  138
_KAI4495142.1_    125  -----------------------------------------GFEHRKQPT---------------SIDLN  138
_XP_014609300.1_  125  -----------------------------------------GFEHRKQPT---------------SIDLN  138
_ADK91833.1_      125  -----------------------------------------GFEHKRQPT---------------SIDLN  138
_KAG7213837.1_    125  -----------------------------------------GFEHKRHPT---------------SIDLN  138
```

```
_XP_014226628.1_  125  -----------------------------------------GFDHKKNPS---------------SIDLN  138
_XP_020287946.1_  125  -----------------------------------------GFDHKRHPT---------------NIDLN  138
_XP_012232007.1_  125  -----------------------------------------GFEHRRHPT---------------NIDLN  138
_XP_011874538.1_  125  -----------------------------------------GFEHKRHPT---------------NIDLN  138
_XP_011874541.1_  125  -----------------------------------------GFEHKRHPT---------------NIDLN  138
_XP_011173619.1_  125  -----------------------------------------GFDHKRHPT---------------NIDLN  138
_XP_034950793.1_  125  -----------------------------------------GFD--INPI---------------TLDLS  136
_XP_034950792.1_  121  -----------------------------------------GFD--INPI---------------TLDLS  132
_XP_012340880.2_  124  -----------------------------------------GFEFKRD-----------------SVDLN  135
_XP_031771859.1_  124  -----------------------------------------GFEFKRD-----------------SVDLN  135
_XP_006618953.1_  124  -----------------------------------------GFEFKRDPD---------------SVDLN  137
_XP_031367904.1_  124  -----------------------------------------GFEFKRDPD---------------SVDLN  137
_XP_043795948.1_  124  -----------------------------------------GFEFKRDPD---------------SVDLN  137
_XP_395180.5_     124  -----------------------------------------GFEFKRDLN---------------SVDLN  137
_KAG6799500.1_    124  -----------------------------------------GFEFKRDLN---------------SVDLN  137
_XP_016768607.2_  124  -----------------------------------------GFEFKRDLN---------------SVDLN  137
_PBC31989.1_      133  -----------------------------------------GFQFKRDLN---------------SIDLN  146
_XP_016914450.1_  124  -----------------------------------------GFQFKRDLN---------------SIDLN  137
_KAG5344958.1_    125  -----------------------------------------GFEHRRHPT---------------NIDLN  138
_KAJ3630188.1_    123  -----------------------------------------GFKHRDDVK---------------SMDLS  136
_QHN75199.1_      123  -----------------------------------------GFKHRDDVK---------------SMDLS  136
_XP_051171660.1_  129  -----------------------------------------GFEHRRTHA---------------NIALN  142
_XP_016909447.1_   83  -----------------------------------------GFEHKRQPT---------------SIDLN   96
_XP_031773030.1_   83  -----------------------------------------GFEHKRQPT---------------SIDLN   96
_KAH0956427.1_    129  -----------------------------------------GFDHRNGKK--------------YCINFS  143
_XP_012283784.1_  125  -----------------------------------------GFEHKRQPT---------------SIDLN  138
_XP_023224928.1_  125  -----------------------------------------GFEHKQQPN---------------SIDLN  138
_XP_008556434.1_  125  -----------------------------------------GFEHKRQPA---------------SIDLN  138
_XP_008556433.1_  125  -----------------------------------------GFEHKRQPA---------------SIDLN  138
_XP_014299728.1_  125  -----------------------------------------GFEHKRQPA---------------SIDLN  138
_CAD6234449.1_    125  -----------------------------------------GFSHKRQPA---------------SIDLN  138
_KAG8037552.1_    125  -----------------------------------------GFSHKRQPA---------------SIDLN  138
_XP_044576138.1_  125  -----------------------------------------GFSHKRQPA---------------SIDLN  138
_XP_044576125.1_  125  -----------------------------------------GFSHKRQPA---------------SIDLN  138
_XP_026465448.1_  125  -----------------------------------------GFAHRSQPS---------------NIDLN  138
_XP_026465446.1_  125  -----------------------------------------GFAHRSQPS---------------NIDLN  138
_XP_031785072.1_  125  -----------------------------------------GFDHKRQPT---------------SIDLN  138
_XP_015607185.1_  125  -----------------------------------------GFDHKRQPT---------------SIDLN  138
_XP_011496611.1_  125  -----------------------------------------GFDHKRQPT---------------SIDLN  138
```

```
_XP_018903707.1_   125  -----------------------------------------GFSHKNQVN---------------TIDLN  138
_CAB3365562.1_     124  -----------------------------------------GFAHGSQPS---------------NIDLN  137
_XP_011496447.1_     1  --------------------------------------------HRNKPE---------------SIDLN   11
_KAG8230996.1_     124  -----------------------------------------GFAHRNQPS---------------SIDLN  137
_XP_040567422.1_   126  -----------------------------------------GFAHKNS-----------------AVNLN  137
_XP_014250205.1_   125  -----------------------------------------GFGHKTQAS---------------SIDLN  138
_XP_014250206.1_   125  -----------------------------------------GFGHKTQAS---------------SIDLN  138
_XP_047119528.1_   125  -----------------------------------------GFAHRSQPT---------------SIDLN  138
_XP_047001845.1_   125  -----------------------------------------GFAHRSQPT---------------SIDLN  138
_XP_049939433.1_   125  -----------------------------------------GFAHRSQPT---------------SIDLN  138
_XP_049764197.1_   125  -----------------------------------------GFAHRSQPT---------------SIDLN  138
_XP_049833656.1_   125  -----------------------------------------GFAHRSQPT---------------SIDLN  138
_XP_049790026.1_   125  -----------------------------------------GFAHRSQPT---------------SIDLN  138
_XP_049833651.1_   125  -----------------------------------------GFAHRSQPT---------------SIDLN  138
_XP_047001842.1_   125  -----------------------------------------GFAHRSQPT---------------SIDLN  138
_XP_049790025.1_   125  -----------------------------------------GFAHRSQPT---------------SIDLN  138
_XP_049833654.1_   125  -----------------------------------------GFAHRSQPT---------------SIDLN  138
_XP_049833659.1_   125  -----------------------------------------GFAHRSQPT---------------SIDLN  138
_XP_021925503.1_   125  -----------------------------------------GFSHRNQPT---------------SIDLN  138
_KDR23857.1_       125  -----------------------------------------GFSHRNQPT---------------SIDLN  138
_ABU96700.1_       125  -----------------------------------------GFSHKTQTS---------------GIDLN  138
_ABU96699.1_       125  -----------------------------------------GFSHKTQTS---------------GIDLN  138
_XP_014275495.1_   125  -----------------------------------------GFAHKTQAS---------------GIDLN  138
_BBE08137.1_       125  -----------------------------------------GFAHKTQAS---------------GIDLN  138
_XP_014275494.1_   125  -----------------------------------------GFAHKTQAS---------------GIDLN  138
_XP_014275493.1_   125  -----------------------------------------GFAHKTQAS---------------GIDLN  138
_BBE08136.1_       125  -----------------------------------------GFAHKTQAS---------------GIDLN  138
_KAF6207519.1_     125  -----------------------------------------KFTHKNQPS---------------SIDLN  138
_CAB0015508.1_     125  -----------------------------------------KFNHKTQPS---------------SIDLN  138
_CAB0015504.1_     125  -----------------------------------------KFNHKTQPS---------------SIDLN  138
_PNF18474.1_       125  -----------------------------------------GFSHRNQPT---------------SIDLN  138
_CEG62431.1_       125  -----------------------------------------GFSHRSQHT---------------SIDLN  138
_PSN36690.1_       125  -----------------------------------------GFSHRSQHT---------------SIDLN  138
_XP_046387412.1_   125  -----------------------------------------GFAHRSQPS---------------SIDLN  138
_XP_046387414.1_   125  -----------------------------------------GFAHRSQPS---------------SIDLN  138
_XP_031634874.1_   125  -----------------------------------------GFGHASQPS---------------SIDLN  138
_GFG29240.1_       125  -----------------------------------------GFSHRNQPT---------------SIDLN  138
_BBG28464.1_       125  -----------------------------------------GFAHRSQPT---------------SIDLN  138
_WCL52334.1_       125  -----------------------------------------GFAHRSQPT---------------SIDLN  138
```

```
_GLH00135.1_       125  -----------------------------------------GFAHRSQPT---------------SIDLN  138
_CAD7205239.1_     125  -----------------------------------------GFTHGDQPT---------------TIDLN  138
_CAD7431383.1_     125  -----------------------------------------GFTHGDQPT---------------TIDLN  138
_CAD7410836.1_     125  -----------------------------------------GFAHGDQPT---------------TIDLN  138
_CAD7415345.1_     125  -----------------------------------------GFTHGDQPT---------------TIDLN  138
_CAD7606635.1_     125  -----------------------------------------GFTHRAQPT---------------TIDLN  138
_CAD7462957.1_     125  -----------------------------------------GFTHRAQPT---------------TIDLN  138
_XP_012265400.2_   125  -----------------------------------------GFAHRAQPT---------------SIDLN  138
_XP_046473587.1_   125  -----------------------------------------GFSHRTQPT---------------GIDLN  138
_XP_046611732.1_   125  -----------------------------------------GFSHRTQPT---------------GIDLN  138
_XP_015519750.2_   125  -----------------------------------------GFSHRTQPT---------------GIDLN  138
_XP_046611731.1_   125  -----------------------------------------GFSHRTQPT---------------GIDLN  138
_XP_046417903.1_   125  -----------------------------------------GFAHRTQPT---------------GIDLN  138
_GLG94364.1_       124  -----------------------------------------GFAHRNQPG---------------NIDLN  137
_GLG94363.1_       124  -----------------------------------------GFAHRNQPG---------------NIDLN  137
_BBG28463.1_       124  -----------------------------------------GFAHRNQPG---------------NIDLN  137
_WCL52335.1_       124  -----------------------------------------GFAHRNQPG---------------NIDLN  137
_GLG94365.1_       124  -----------------------------------------GFAHRNQPG---------------NIDLN  137
_XP_002431518.1_   123  -----------------------------------------GFSHKVQPS---------------SIDLN  136
_KAE8746525.1_     125  -----------------------------------------GFAHRGHPT---------------GIDLN  138
_XP_026277770.1_   125  -----------------------------------------GFAHRGHPT---------------GIDLN  138
_KAJ1519446.1_     125  -----------------------------------------GFAHRSNPT---------------GIDLN  138
_ALC78349.1_       125  -----------------------------------------GFSHRDQPS---------------SIDLN  138
_XP_046738772.1_   125  -----------------------------------------GFSHRAQPT---------------GIDLN  138
_XP_046417904.1_   125  -----------------------------------------GFAHRTQPT---------------GIDLN  138
_CRK94170.1_       125  -----------------------------------------GYDHRNQPS---------------SIDLN  138
_CRK94167.1_       125  -----------------------------------------GYDHRNQPS---------------SIDLN  138
_KAG5676160.1_     125  -----------------------------------------GYTHRNQPS---------------SIDLN  138
_XP_034245464.1_   126  -----------------------------------------GFAHRSQPT---------------GIDLN  139
_XP_034245457.1_   126  -----------------------------------------GFAHRSQPT---------------GIDLN  139
_KAG8287980.1_     125  -----------------------------------------GFSHRSQPG---------------SIDLN  138
_XP_044759045.1_   123  -----------------------------------------GFS--TDQS---------------VYDFT  134
_XP_017305229.1_   124  -----------------------------------------GFNNRD------------------NLDLN  134
_XP_026675878.1_   124  -----------------------------------------GFNNRD------------------NLDLN  134
_KAE8574083.1_          ----------------------------------------------------------------------
_XP_037926497.1_   125  -----------------------------------------GFDHRFQPA---------------SIDLN  138
_CAD7079567.1_     125  -----------------------------------------GFDHRFQPA---------------SIDLN  138
_XP_037882485.1_   125  -----------------------------------------GFQHRFQPS---------------GIDLN  138
_XP_037037597.1_   125  -----------------------------------------GFNHRSQPS---------------SIDLN  138
```

```
_XP_037037592.1_   125  -----------------------------------------GFNHRSQPS---------------SIDLN  138
_KAG4075389.1_     125  -----------------------------------------GFNHRSQPS---------------SIDLN  138
_XP_034101357.1_   125  -----------------------------------------GFSHRFQPS---------------SIDLN  138
_XP_026844963.1_   125  -----------------------------------------GFSHRFQPS---------------SIDLN  138
_EDW28798.1_       125  -----------------------------------------GFSHRFQPS---------------SIDLN  138
_XP_034125966.1_   125  -----------------------------------------GFSHRFQPS---------------SIDLN  138
_XP_034666519.1_   125  -----------------------------------------GFSHRFQPS---------------SIDLN  138
_XP_001356570.3_   125  -----------------------------------------GFSHRFQPS---------------SIDLN  138
_XP_023164577.2_   125  -----------------------------------------GFSHRFQPS---------------SIDLN  138
_XP_017153206.1_   125  -----------------------------------------GFSHRFQPS---------------SIDLN  138
_XP_030564932.1_   125  -----------------------------------------GFSHRFQPS---------------SIDLN  138
_XP_002059145.1_   125  -----------------------------------------GFSHRFQPS---------------SIDLN  138
_EDW04266.1_       125  -----------------------------------------GFSHRFQPS---------------SIDLN  138
_XP_017025862.1_   125  -----------------------------------------GFSHRFQPS---------------SIDLN  138
_XP_017859907.1_   125  -----------------------------------------GFSHRFQPS---------------SIDLN  138
_XP_032584327.1_   125  -----------------------------------------GFSHRFQPS---------------SIDLN  138
_XP_016987288.2_   125  -----------------------------------------GFSHRFQPS---------------SIDLN  138
_XP_017954173.1_   125  -----------------------------------------GFSHRFQPS---------------SIDLN  138
_XP_017854504.1_   125  -----------------------------------------GFSHRFQPS---------------SIDLN  138
_XP_034125965.1_   125  -----------------------------------------GFSHRFQPS---------------SIDLN  138
_XP_034666515.1_   125  -----------------------------------------GFSHRFQPS---------------SIDLN  138
_XP_043641917.1_   125  -----------------------------------------GFSHRFQPS---------------SIDLN  138
_XP_026844960.1_   125  -----------------------------------------GFSHRFQPS---------------SIDLN  138
_XP_015035988.2_   125  -----------------------------------------GFSHRFQPS---------------SIDLN  138
_XP_041450744.1_   125  -----------------------------------------GFSHRFQPS---------------SIDLN  138
_XP_015052361.1_   125  -----------------------------------------GFSHRFQPS---------------SIDLN  138
_XP_039480515.1_   125  -----------------------------------------GFSHRFQPS---------------SIDLN  138
_XP_033248563.1_   125  -----------------------------------------GFSHRFQPS---------------SIDLN  138
_KAH8412150.1_     125  -----------------------------------------GFSHRFQPS---------------SIDLN  138
_XP_043864974.1_   125  -----------------------------------------GFSHRFQPS---------------SIDLN  138
_XP_020809119.1_   125  -----------------------------------------GFSHRFQPS---------------SIDLN  138
_KAH8298353.1_     125  -----------------------------------------GFSHRFQPS---------------SIDLN  138
_XP_030564927.1_   125  -----------------------------------------GFSHRFQPS---------------SIDLN  138
_XP_041631592.1_   125  -----------------------------------------GFSHRFQPS---------------SIDLN  138
_XP_017859909.1_   125  -----------------------------------------GFSHRFQPS---------------SIDLN  138
_XP_032591602.1_   125  -----------------------------------------GFSHRFQPS---------------SIDLN  138
_XP_023164572.2_   125  -----------------------------------------GFSHRFQPS---------------SIDLN  138
_TDG46699.1_       125  -----------------------------------------GFSHRFQPS---------------SIDLN  138
_KAH8411895.1_     125  -----------------------------------------GFSHRFQPS---------------SIDLN  138
```

```
_KAH8377496.1_    125  -----------------------------------------GFSHRFQPS---------------SIDLN  138
_XP_017854500.2_  125  -----------------------------------------GFSHRFQPS---------------SIDLN  138
_XP_016987284.2_  125  -----------------------------------------GFSHRFQPS---------------SIDLN  138
_KAH8293209.1_    125  -----------------------------------------GFSHRFQPS---------------SIDLN  138
_EEC00225.1_      124  -----------------------------------------GFDHKDQAG---------------SIDLN  137
_XP_029825505.2_  124  -----------------------------------------GFDHKDQAG---------------SIDLN  137
_KAG0424790.1_    124  -----------------------------------------GFDHKDQAG---------------SIDLN  137
_XP_037272127.1_  124  -----------------------------------------GFAHKDQAA---------------SIDLN  137
_XP_054934017.1_  124  -----------------------------------------GFAHKDQAA---------------SIDLN  137
_XP_050048524.1_  124  -----------------------------------------GFAHKDQAA---------------SIDLN  137
_XP_037272126.1_  124  -----------------------------------------GFAHKDQAA---------------SIDLN  137
_XP_037524244.1_  124  -----------------------------------------GFAHKDQAA---------------SIDLN  137
_XP_037524245.1_  124  -----------------------------------------GFAHKDQAA---------------SIDLN  137
_XP_049512094.1_  124  -----------------------------------------GFAHKDQAA---------------SIDLN  137
_XP_037524243.1_  124  -----------------------------------------GFAHKDQAA---------------SIDLN  137
_XP_037580160.1_  124  -----------------------------------------GFAHKDQAA---------------SIDLN  137
_XP_037272128.1_  124  -----------------------------------------GFAHKDQAA---------------SIDLN  137
_KAH9380259.1_    124  -----------------------------------------GFGHKDQAA---------------SIDLN  137
_KAI1291715.1_     97  -----------------------------------------GFTHKNRPS---------------KIELN  110
_XP_035209023.1_  124  -----------------------------------------GFENTT------------------DFDLN  134
_XP_023332679.1_  130  -----------------------------------------GWKHVENPQ---------------NIDLN  143
_XP_046801942.1_  125  -----------------------------------------GFAHRFQPS---------------SIDLN  138
_XP_037811120.1_  125  -----------------------------------------GFAHRFQPS---------------SIDLN  138
_AFI98401.1_      125  -----------------------------------------GFAHRFQPS---------------SIDLN  138
_AFJ54621.1_      125  -----------------------------------------GFAHRFQPS---------------SIDLN  138
_KAI8126534.1_    125  -----------------------------------------GFAHRFQPS---------------SIDLN  138
_XP_013118266.1_  125  -----------------------------------------GFAHRFQPS---------------SIDLN  138
_XP_023293634.2_  125  -----------------------------------------GFAHRFQPS---------------SIDLN  138
_XP_037811062.1_  125  -----------------------------------------GFAHRFQPS---------------SIDLN  138
_KNC34132.1_      125  -----------------------------------------GFAHRFQPS---------------SIDLN  138
_TMW48951.1_      125  -----------------------------------------GFAHRYQPS---------------SIDLN  138
_XP_037957363.1_  125  -----------------------------------------GFSHRFQPS---------------SIDLN  138
_XP_005175014.1_  125  -----------------------------------------GFSHRFQPS---------------SIDLN  138
_XP_037957360.1_  125  -----------------------------------------GFSHRFQPS---------------SIDLN  138
_XP_030383330.1_  125  -----------------------------------------GFSHRFQPS---------------SIDLN  138
_XP_030383328.1_  125  -----------------------------------------GFSHRFQPS---------------SIDLN  138
_CAH1707987.1_    124  -----------------------------------------GYAHRSQPS---------------SIDLN  137
_TMW54796.1_      125  -----------------------------------------GFEHRYQPS---------------SIDLN  138
_XP_026836077.1_  125  -----------------------------------------GFSHRFQPS---------------SIDLN  138
```

```
_XP_017116803.1_  125  -----------------------------------------GFSHRFQPS---------------SIDLN  138
_XP_039480519.1_  125  -----------------------------------------GFSHRFQPS---------------SIDLN  138
_XP_043641920.1_  125  -----------------------------------------GFSHRFQPS---------------SIDLN  138
_XP_023036748.1_  125  -----------------------------------------GFSHRFQPS---------------SIDLN  138
_XP_002090466.1_  125  -----------------------------------------GFSHRFQPS---------------SIDLN  138
_XP_034471824.1_  125  -----------------------------------------GFSHRFQPS---------------SIDLN  138
_AAT94434.1_      125  -----------------------------------------GFSHRFQPS---------------SIDLN  138
_XP_036670985.1_  125  -----------------------------------------GFSHRFQPS---------------SIDLN  138
_AAA28479.1_      125  -----------------------------------------GFSHRFQPS---------------SIDLN  138
_XP_037708206.1_  125  -----------------------------------------GFTHRFQPS---------------SIDLN  138
_XP_002038915.1_  125  -----------------------------------------GFSHRFQPS---------------SIDLN  138
_XP_016993636.1_  125  -----------------------------------------GFSHRFQPS---------------SIDLN  138
_XP_052852517.1_  125  -----------------------------------------GFSHRFQPS---------------SIDLN  138
_XP_016963139.1_  125  -----------------------------------------GFSHRFQPS---------------SIDLN  138
_XP_033156151.1_  125  -----------------------------------------GFSHRFQPS---------------SIDLN  138
_XP_001974446.1_  125  -----------------------------------------GFSHRFQPS---------------SIDLN  138
_XP_014761886.1_  125  -----------------------------------------GFSHRFQPS---------------SIDLN  138
_XP_017107216.2_  125  -----------------------------------------GFSHRFQPS---------------SIDLN  138
_XP_017044881.1_  125  -----------------------------------------GFSHRFQPS---------------SIDLN  138
_XP_016944798.1_  125  -----------------------------------------GFSHRFQPS---------------SIDLN  138
_XP_017116800.1_  125  -----------------------------------------GFSHRFQPS---------------SIDLN  138
_XP_016993633.2_  125  -----------------------------------------GFSHRFQPS---------------SIDLN  138
_XP_037708198.1_  125  -----------------------------------------GFTHRFQPS---------------SIDLN  138
_XP_032583259.1_  125  -----------------------------------------GFSHRFQPS---------------SIDLN  138
_AAC35296.1_      125  -----------------------------------------GFSHRFQPS---------------SIDLN  138
_NP_001286014.1_  125  -----------------------------------------GFSHRFQPS---------------SIDLN  138
_XP_016963136.1_  125  -----------------------------------------GFSHRFQPS---------------SIDLN  138
_XP_017063904.2_  125  -----------------------------------------GFSHRFQPS---------------SIDLN  138
_XP_033156135.1_  125  -----------------------------------------GFSHRFQPS---------------SIDLN  138
_XP_052852512.1_  125  -----------------------------------------GFSHRFQPS---------------SIDLN  138
_XP_032307758.1_  125  -----------------------------------------GFSHRFQPS---------------SIDLN  138
_XP_017107215.2_  125  -----------------------------------------GFSHRFQPS---------------SIDLN  138
_AWT86616.1_      124  -----------------------------------------GYEHKNQPG---------------SIDLN  137
_QUF98534.1_      124  -----------------------------------------GYEHKNQPG---------------SIDLN  137
_UNA06287.1_      124  -----------------------------------------GYEHKNQPG---------------SIDLN  137
_AAQ65068.1_      125  -----------------------------------------GFSHRFQPS---------------SIDLN  138
_AAQ64916.1_      125  -----------------------------------------GFSHRFQPS---------------SIDLN  138
_AAQ64921.1_      125  -----------------------------------------GFSHRFQPS---------------SIDLN  138
_AAQ64918.1_      125  -----------------------------------------GFSHRFQPS---------------SIDLN  138
```

```
_KAH8245459.1_     129  NALKFCNF--------------------ILLQTFLITLHPAGFSHRFQPS---------------SIDLN  163
_KAH8283155.1_     129  NLI---SI--------------------FFIKMYRLSLLSAGFSHRFQPS---------------SIDLN  160
_KAH8246384.1_     129  SVS-F-SF--------------------LYLKC-RLYIFPAGFSHRFQPS---------------SIDLN  160
_KAH8366418.1_     129  FKLSNFE-----------------------ENPILFIPPLAGFSHRFQPS---------------SIDLN  160
_XP_022195378.2_   124  -----------------------------------------GYDHRNQPG---------------SIDLN  137
_XP_039284931.1_   124  -----------------------------------------GYDHRNQPG---------------SIDLN  137
_ALC40091.1_       129  HICILKS-----------------------LLKYYLCQLAAGFSHRFQPS---------------SIDLN  160
_XP_036342419.1_   125  -----------------------------------------GFAHRFQPG---------------AINLN  138
_XP_022903025.1_   124  -----------------------------------------GFSHRSSPT---------------SIDLN  137
_XP_022903017.1_   124  -----------------------------------------GFSHRSSPT---------------SIDLN  137
_XP_036218946.1_   125  -----------------------------------------GFSHVTQPS---------------SIDIN  138
_XP_018790401.1_   125  -----------------------------------------GFSHATQPG---------------TIDIN  138
_KAI9579887.1_     123  -----------------------------------------GFSHH-------------------SIDLS  132
_XP_037892537.1_   123  -----------------------------------------GFSHH-------------------SIDLS  132
_XP_018903705.1_   132  -----------------------------------------GFSHKNQVN---------------TIDLN  145
_CAH0395667.1_     132  -----------------------------------------GFSHKNQVN---------------TIDLN  145
_XP_050330165.1_   125  -----------------------------------------GFSHAKQPG---------------SININ  138
_XP_039970878.1_   125  -----------------------------------------GFSHAKQPG---------------SININ  138
_XP_049305234.1_   125  -----------------------------------------GFSHAEQPG---------------SININ  138
_GLH04793.1_       131  -----------------------------------------GFDHRKNPS---------------VIDLN  144
_XP_015835035.1_   124  -----------------------------------------GFSHKDHLN---------------CIDLN  137
_XP_015835036.1_   124  -----------------------------------------GFSHKDHLN---------------CIDLN  137
_XP_039283832.1_   135  -----------------------------------------GFDHKEQPN---------------NIKMN  148
_XP_031788427.1_   125  -----------------------------------------GFDQDLD-----------------EIDIC  136
_KAJ6646894.1_     127  -----------------------------------------GYNHKTNA----------------VYDAS  139
_XP_026466084.1_    60  -----------------------------------------GFSHASEPS---------------SIDLN   73
_XP_044002069.1_   125  -----------------------------------------GFDYKNETN---------------TIDPS  138
_XP_046611735.1_   125  -----------------------------------------GFSHKVTPI---------------DIDMN  138
_XP_046417906.1_   125  -----------------------------------------GFSHKVTPI---------------DIDMN  138
_XP_046590309.1_   125  -----------------------------------------GFSHKVTPI---------------DIDMN  138
_XP_046473589.1_   125  -----------------------------------------GFSHKVTPI---------------DIDMN  138
_XP_019892116.1_    36  -----------------------------------------GFSHRFQPS---------------SIDLN   49
_XP_005183027.3_    36  -----------------------------------------GFSHRFQPS---------------SIDLN   49
_XP_011496598.1_    52  -----------------------------------------GFSHKLQPE---------------NIDLN   65
_AAW67216.1_        24  -----------------------------------------GYGHARQPA---------------TIDLN   37
_AAW67217.1_        24  -----------------------------------------GYGHARQPA---------------TIDLN   37
_XP_017489788.1_    29  -----------------------------------------GFAHRFQPG---------------AINLN   42
_BAI67894.1_        28  -----------------------------------------GFAHRYQPS---------------SIDLN   41
_XP_054741511.1_   125  -----------------------------------------GFSHRFQPT---------------SIDLN  138
```

```
_XP_053961684.1_  125  -----------------------------------------GFSHRFQPT---------------SIDLN  138
_XP_036341313.1_  125  -----------------------------------------GFSHRFQPT---------------SIDLN  138
_XP_036341343.1_  125  -----------------------------------------GFSHRFQPT---------------SIDLN  138
_XP_017471383.1_  125  -----------------------------------------GFSHRFQPT---------------SIDLN  138
_XP_054741498.1_  125  -----------------------------------------GFSHRFQPT---------------SIDLN  138
_XP_053961677.1_  124  -----------------------------------------GFSHRFQPT---------------SIDLN  137
_XP_050320008.1_  125  -----------------------------------------GFSHRFQPT---------------SIDLN  138
_XP_028894490.2_  125  -----------------------------------------GFSHRFQPT---------------SIDLN  138
_XP_050320015.1_  125  -----------------------------------------GFSHRFQPT---------------SIDLN  138
_XP_018787554.1_  125  -----------------------------------------GFSHRFQPT---------------SIDLN  138
_XP_029407438.2_  125  -----------------------------------------GFSHRFQPT---------------SIDLN  138
_XP_036225551.1_  125  -----------------------------------------GFSHRFQPT---------------SIDLN  138
_XP_011178374.2_  125  -----------------------------------------GFSHRFQPT---------------SIDLN  138
_XP_039952593.1_  125  -----------------------------------------GFSHRFQPT---------------SIDLN  138
_XP_018787542.1_  125  -----------------------------------------GFSHRFQPT---------------SIDLN  138
_XP_011207866.2_  125  -----------------------------------------GFSHRFQPT---------------SIDLN  138
_XP_014086506.1_  125  -----------------------------------------GFSHRFQPT---------------SIDLN  138
_XP_004526638.1_  125  -----------------------------------------GFSHRFQPT---------------SIDLN  138
_XP_017471402.1_  125  -----------------------------------------GFSHRFQPT---------------SIDLN  138
_XP_012157903.1_  125  -----------------------------------------GFSHRFQPT---------------SIDLN  138
_XP_011185661.2_  125  -----------------------------------------GFSHAMMPG---------------TLNIN  138
_XP_050322757.1_  125  -----------------------------------------GFSHATQPC---------------SIDIN  138
_XP_039962922.1_  125  -----------------------------------------GFSHATQPC---------------SIDIN  138
_XP_050322739.1_  125  -----------------------------------------GFSHATQPC---------------SIDIN  138
_XP_050322749.1_  125  -----------------------------------------GFSHATQPC---------------SIDIN  138
_XP_050322724.1_  125  -----------------------------------------GFSHATQPC---------------SIDIN  138
_XP_050322682.1_  125  -----------------------------------------GFSHATQPC---------------SIDIN  138
_CAG9823112.1_    130  -----------------------------------------GFSHRNQPT---------------SIDLN  143
_KAG5873705.1_    125  -----------------------------------------GFGHRNQPT---------------SIDLN  138
_KAH8030364.1_    103  -----------------------------------------GFAHKDQAA---------------SIDLN  116
_XP_023013891.1_  125  -----------------------------------------GFAHRNQPT---------------SIDLN  138
_XP_028130780.2_  131  -----------------------------------------GFSHRNQPT---------------SIDLN  144
_KAG5873704.1_    130  -----------------------------------------GFGHRNQPT---------------SIDLN  143
_CAH1187690.1_    130  -----------------------------------------GFSHRNQPT---------------AIDLN  143
_CAH1098641.1_    130  -----------------------------------------GFSHRNQPT---------------SIDLN  143
_XP_023028116.1_  130  -----------------------------------------GFAHKNDSS---------------AVDLV  143
_XP_031630227.1_  126  -----------------------------------------GWDQMMKPH---------------LIDLH  139
_KAG5861047.1_      1  -------------------------------------------GHRNQPT---------------SIDLN   12
_KAF2880990.1_    125  -----------------------------------------GFAHRNQPT---------------SIDLN  138
```

```
_XP_018326789.1_  125  -----------------------------------------GFSHRNQPT---------------SIDLN  138
_XP_018326788.1_  125  -----------------------------------------GFSHRNQPT---------------SIDLN  138
_XP_031339184.1_  125  -----------------------------------------GFSHRNQPT---------------SIDLN  138
_XP_045476069.1_  125  -----------------------------------------GFNHRNQPT---------------SIDLN  138
_XP_044734149.1_  125  -----------------------------------------GFAHRNQAS---------------SIDLN  138
_XP_044734150.1_  125  -----------------------------------------GFAHRNQAS---------------SIDLN  138
_XP_044734148.1_  125  -----------------------------------------GFAHRNQAS---------------SIDLN  138
_XP_044760528.1_  125  -----------------------------------------GFSHRNQPT---------------SIDLN  138
_XP_044747918.1_  125  -----------------------------------------GFSHRHHPT---------------SIDLN  138
_XP_044747917.1_  125  -----------------------------------------GFSHRHHPT---------------SIDLN  138
_XP_045481017.1_  125  -----------------------------------------GFSHKHHPT---------------SIDLN  138
_XP_045481018.1_  125  -----------------------------------------GFSHKHHPT---------------SIDLN  138
_KAF5302274.1_    125  -----------------------------------------GFAHRNQPT---------------SIDLN  138
_KAF5308883.1_    125  -----------------------------------------GFAHRNQPT---------------SIDLN  138
_KRT79621.1_      124  -----------------------------------------GFSHRSQPT---------------SIDLN  137
_BAD20728.1_      124  -----------------------------------------GFSHRNQPT---------------SIDLN  137
_KAF2880988.1_    124  -----------------------------------------GFAHRSQPN---------------SIDRK  137
_CAH1403258.1_    117  -----------------------------------------GFAHKTQAS---------------GIDLN  130
_CAD6992094.1_    125  -----------------------------------------GFEHATEAI---------------FVDMT  138
_XP_004537184.1_  125  -----------------------------------------GFEHATEAI---------------FVDMT  138
_XP_018572644.1_  125  -----------------------------------------GFGHRNQPT---------------SIDLN  138
_RZB54393.1__     125  -----------------------------------------GFSHRSQPT---------------SIDLN  138
_XP_044255725.1_  125  -----------------------------------------GFSHRNQPT---------------SIDLN  138
_EFA02850.1_      125  -----------------------------------------GFSHRNQPT---------------SIDLN  138
_NP_001034507.1_  125  -----------------------------------------GFSHRNQPT---------------SIDLN  138
_CAH1370311.1_    125  -----------------------------------------GFSHRNQPT---------------SIDLN  138
_KAJ3630692.1_    125  -----------------------------------------GFSHRNQPT---------------SIDLN  138
_XP_044255724.1_  125  -----------------------------------------GFSHRNQPT---------------SIDLN  138
_KAH0811528.1_    125  -----------------------------------------GFSHRNQPT---------------SIDLN  138
_CAH7716406.1_    125  -----------------------------------------GYSHRDQPT---------------SIDLN  138
_CAH7716405.1_    125  -----------------------------------------GYSHRDQPT---------------SIDLN  138
_CAH7716404.1_    125  -----------------------------------------GYSHRDQPT---------------SIDLN  138
_XP_028130776.2_  125  -----------------------------------------GFSHRNQPT---------------SIDLN  138
_CAH1985684.1_    125  -----------------------------------------GYSHKDQPT---------------SIDLN  138
_XP_017781152.1_  125  -----------------------------------------GYTHRSQPT---------------SIDLN  138
_XP_017781151.1_  125  -----------------------------------------GYTHRSQPT---------------SIDLN  138
_XP_048522053.1_  124  -----------------------------------------GYSHRSQPT---------------SIDLN  137
_XP_050303070.1_  129  -----------------------------------------GFSHKNQPT---------------SIDLN  142
_CAG9762924.1_    125  -----------------------------------------GYAHRSQPT---------------SIDLN  138
```

```
_CAH0560654.1_     126  -----------------------------------------GFSHRNQPT---------------SIDLN  139
_CAH0560655.1_     126  -----------------------------------------GFSHRNQPT---------------SIDLN  139
_XP_018572643.1_   124  -----------------------------------------GFGHRNQPT---------------SIDLN  137
_XP_030763656.1_   125  -----------------------------------------HYHHKNQPT---------------SIDLN  138
_XP_030763654.1_   125  -----------------------------------------HYHHKNQPT---------------SIDLN  138
_XP_018577945.1_   125  -----------------------------------------GFGHGSQPA---------------TIDLN  138
_KAF7283276.1_     125  -----------------------------------------HYHHKNQPT---------------SIDLN  138
_KAF7283275.1_     125  -----------------------------------------HYHHKNQPT---------------SIDLN  138
_XP_030763617.1_   125  -----------------------------------------HFAPQNELS---------------SIDLN  138
_XP_030763619.1_   128  -----------------------------------------HFHHKYNPN---------------LINLS  141
_VEN60240.1_        36  -----------------------------------------GYSHRDQPT---------------SIDLN   49
_KOB78236.1_       115  -----------------------------------------GFGSTER-------------------IDLH  125
_ERL85515.1_        67  -----------------------------------------GYSHRSQPT---------------SIDLN   80
_ENN78766.1_        62  -----------------------------------------GYSHRSQPT---------------SIDLN   75
_KAH1019371.1_      62  -----------------------------------------GYSHRSQPT---------------SIDLN   75
_KAI9565420.1_     125  -----------------------------------------GFAHKNQPQ---------------SIDLN  138
_XP_045024905.1_   125  -----------------------------------------GFAHKNQPQ---------------SIDLN  138
_XP_046447094.1_   125  -----------------------------------------GFAHKNQPQ---------------SIDLN  138
_KZS08518.1_       125  -----------------------------------------GFTHKNQPQ---------------SIDLN  138
_XP_046636307.1_   125  -----------------------------------------GFAHKNQPQ---------------SIDLN  138
_EFX79013.1_       125  -----------------------------------------GFAHKNQPQ---------------SIDLN  138
_XP_032776606.2_   125  -----------------------------------------GFAHKNQPQ---------------SIDLN  138
_XP_046447093.1_   125  -----------------------------------------GFAHKNQPQ---------------SIDLN  138
_XP_046636306.1_   125  -----------------------------------------GFAHKNQPQ---------------SIDLN  138
_CAH0099799.1_     115  -----------------------------------------GFAHKNQPQ---------------SIDLN  128
_KAJ1531033.1_     126  -----------------------------------------GFAHCEE-----------------EIDMD  137
_ACJ36225.1_       125  -----------------------------------------GFSHRNQPQ---------------SIDLN  138
_ACZ98167.1_       125  -----------------------------------------GFSHRNQPQ---------------SIDLN  138
_AME17867.1_       125  -----------------------------------------GFSHRNQPQ---------------SIDLN  138
_XP_042868409.1_   125  -----------------------------------------GFSHRNQPQ---------------SIDLN  138
_XP_045102711.1_   125  -----------------------------------------GFSHRSQPQ---------------SIDLN  138
_XP_047487558.1_   125  -----------------------------------------GFSHRNQPQ---------------SIDLN  138
_QBJ05651.1_       125  -----------------------------------------GFSHRSQPQ---------------SIDLN  138
_AZK36046.1_       125  -----------------------------------------GFSHRSQPQ---------------SIDLN  138
_XP_045581920.1_   125  -----------------------------------------GFSHRNQPQ---------------SIDLN  138
_XP_050704598.1_   125  -----------------------------------------GFSHRSQTQ---------------SIDLN  138
_XP_027237995.1_   125  -----------------------------------------GFSHRNQPQ---------------SIDLN  138
_AXB88326.1_       125  -----------------------------------------GFSHRSQPQ---------------SIDLN  138
_XP_045102712.1_   125  -----------------------------------------GFSHRSQPQ---------------SIDLN  138
```

```
_MPC17461.1_      125  -----------------------------------------GFSHRSQPQ---------------SIDLN  138
_XP_027237994.1_  125  -----------------------------------------GFSHRNQPQ---------------SIDLN  138
_XP_027237996.1_  125  -----------------------------------------GFSHRNQPQ---------------SIDLN  138
_XP_045102715.1_  125  -----------------------------------------GFSHRSQPQ---------------SIDLN  138
_XP_053627990.1_  125  -----------------------------------------GFSHRNQPQ---------------SIDLN  138
_XP_053627989.1_  125  -----------------------------------------GFSHRNQPQ---------------SIDLN  138
_XP_053627983.1_  125  -----------------------------------------GFSHRNQPQ---------------SIDLN  138
_KAB7495058.1_    125  -----------------------------------------GFTHRSQPQ---------------SIDLN  138
_KAF2353654.1_    125  -----------------------------------------GFSHRGQPQ---------------SIDLN  138
_ANH22235.1_      125  -----------------------------------------GFSHRNQPQ---------------SIDLN  138
_CAD7275741.1_    124  -----------------------------------------GFDHKHQPN---------------SIDLN  137
_XP_042243403.1_  125  -----------------------------------------GFSYRTQPQ---------------NIDLN  138
_QTC09942.1_      125  -----------------------------------------GFSHRNQPQ---------------SIDLN  138
_XP_018023738.1_  104  -----------------------------------------GFGHRNQPQ---------------SIDLN  117
_XP_019877552.1_  126  -----------------------------------------GFSHRNQPT---------------SIDLN  139
_XP_049825875.1_  126  -----------------------------------------GFGHKSQP-----------------IDLN  137
_CAD7649074.1_    125  -----------------------------------------GFSHKSQSS---------------SIDLN  138
_CAD7630012.1_    125  -----------------------------------------GFSHKSQSS---------------SIDLN  138
_XP_054165343.1_  126  -----------------------------------------GFAHKSQSS---------------SIDLN  139
_XP_044254219.1_  124  -----------------------------------------GFLHKDHLN---------------CIDLN  137
_XP_053201827.1_  125  -----------------------------------------GFAHKNQSS---------------SIDLN  138
_XP_015786990.1_  125  -----------------------------------------GFAHKNQAS---------------SIDLN  138
_KAI1295507.1_    125  -----------------------------------------GFSHKTQAS---------------NIDLN  138
_RWS07527.1_      125  -----------------------------------------GFNHKTQAS---------------NIDLN  138
_RWS29859.1_      125  -----------------------------------------GFAHKSQAS---------------NIDLN  138
_XP_053201825.1_  125  -----------------------------------------GFAHKNQSS---------------SIDLN  138
_XP_054713467.1_  130  -----------------------------------------GFNHRE------------------NIDLR  140
_XP_046915158.1_  126  -----------------------------------------GFAHRTQAS---------------SIDLN  139
_KAJ6217218.1_    126  -----------------------------------------GFNHRSQAS---------------SIDLN  139
_KAI2801352.1_    126  -----------------------------------------GFNHRSQAS---------------SIDLN  139
_KAH9406651.1_    126  -----------------------------------------GFNHRSQAS---------------SIDLN  139
_XP_017483538.1_  126  -----------------------------------------GFNHRSQAS---------------SIDLN  139
_KPM10359.1_      126  -----------------------------------------GFSHRNQTS---------------SIDLN  139
_UXI17582.1_      126  -----------------------------------------GFSHRNQTS---------------SIDLN  139
_KAF7488016.1_    126  -----------------------------------------GFSHRNQTS---------------SIDLN  139
_KAH9423377.1_    126  -----------------------------------------GFAHRTQAS---------------SIDLN  139
_OTF73464.1_      126  -----------------------------------------GFAHRTQAS---------------SIDLN  139
_XP_037072056.1_  125  -----------------------------------------GFEHRLQPT---------------SIDLN  138
_XP_037083492.1_  125  -----------------------------------------GFDHRLQPT---------------TIDLN  138
```

```
_XP_037083508.1_  125  -----------------------------------------GFDHRLQPT---------------TIDLN  138
_XP_043215018.1_  125  -----------------------------------------GFDHRLQPT---------------TIDLN  138
_KAF0314765.1_    125  -----------------------------------------GFDHRLQPT---------------TIDLN  138
_XP_043215020.1_  125  -----------------------------------------GFDHRLQPT---------------TIDLN  138
_XP_043189654.1_  125  -----------------------------------------GFDHRLQPT---------------AIDLN  138
_KAF0293344.1_     99  ERVS-----------------------------GYTRWVDAGFDHRLQPT---------------AIDLN  124
_XP_011496609.1_  126  -----------------------------------------GFSHMSNSE---------------SIDLS  139
_AGS12619.1_      125  -----------------------------------------GFSHKDNAQ---------------SLDLN  138
_TRY78048.1_      125  -----------------------------------------GFSHKDNAQ---------------SLDLN  138
_XP_040576307.1_  125  -----------------------------------------GFAHRDNPQ---------------SIDLN  138
_CAB4056363.1_    125  -----------------------------------------GFAHRDNPQ---------------SIDLN  138
_ALF84898.1_      125  -----------------------------------------GFQHKDAHQ---------------SIDLN  138
_XP_023349538.1_  121  -----------------------------------------GFTHKTNPA---------------AVDLN  134
_XP_004522582.1_  125  -----------------------------------------GFAHSTDPA---------------SLDLS  138
_CAD7013622.1_    125  -----------------------------------------GFAHSSDPA---------------SLDLS  138
_XP_042902217.1_  128  -----------------------------------------GFN---HPN---------------SIDLN  138
_XP_042902218.1_  128  -----------------------------------------GFN---HPN---------------SIDLN  138
_XP_035215470.1_  128  -----------------------------------------GFN---HPG---------------SIDLN  138
_GBN14612.1_      127  -----------------------------------------GFS---HPG---------------NIDLN  137
_KAF8795965.1_    128  -----------------------------------------GFS---HPG---------------NIDLN  138
_KAF8795964.1_    128  -----------------------------------------GFS---HPG---------------NIDLN  138
_GFY66827.1_      128  -----------------------------------------GFS---HPG---------------SIDLN  138
_GFW66965.1_      128  -----------------------------------------GFS---HPG---------------SIDLN  138
_GFQ85823.1_      128  -----------------------------------------GFN---HPG---------------SIDLN  138
_GFT35953.1_      128  -----------------------------------------GFT---HPG---------------SIDLN  138
_XP_054714028.1_  128  -----------------------------------------GFN---HPG---------------SIDLN  138
_KAG8194583.1_    127  -----------------------------------------GFN---HPN---------------SIDLN  137
_GIY09899.1_      128  -----------------------------------------GFNSS-------------------NIDLN  137
_GIY93593.1_      128  -----------------------------------------GFNSS-------------------NIDLN  137
_XP_042902221.1_  140  -----------------------------------------GFD---YTK---------------DIDLN  150
_XP_046962867.1_  127  -----------------------------------------GYNHRNQPQ---------------SIDLN  140
_XP_047530540.1_  127  -----------------------------------------GYNHRNQPQ---------------SIDLN  140
_XP_026486466.1_  127  -----------------------------------------GYNHRNQPQ---------------SIDLN  140
_XP_047530539.1_  127  -----------------------------------------GYNHRNQPQ---------------SIDLN  140
_XP_047530538.1_  127  -----------------------------------------GYNHRNQPQ---------------SIDLN  140
_XP_047530537.1_  127  -----------------------------------------GYNHRNQPQ---------------SIDLN  140
_XP_026486464.1_  127  -----------------------------------------GYNHRNQPQ---------------SIDLN  140
_XP_047530541.1_  127  -----------------------------------------GYNHRNQPQ---------------SIDLN  140
_XP_050347286.1_  127  -----------------------------------------GYSHRNQPQ---------------SIDLN  140
```

```
_XP_050347283.1_  127  -----------------------------------------GYSHRNQPQ---------------SIDLN  140
_XP_032515351.1_  128  -----------------------------------------GFGHRNKPQ---------------SIDLN  141
_OWR46016.1_      128  -----------------------------------------GFGHRNKPQ---------------SIDLN  141
_XP_032515346.1_  128  -----------------------------------------GFGHRNKPQ---------------SIDLN  141
_CAG9579094.1_    128  -----------------------------------------GFGHRNKPQ---------------SIDLN  141
_CAG9579091.1_    128  -----------------------------------------GFGHRNKPQ---------------SIDLN  141
_XP_050668638.1_  126  -----------------------------------------GYSHRNQPQ---------------SIDLN  139
_XP_050668632.1_  126  -----------------------------------------GYSHRNQPQ---------------SIDLN  139
_XP_045495329.1_  126  -----------------------------------------GFGHRNQPQ---------------SIDLN  139
_XP_038214802.1_  126  -----------------------------------------GFGHRNQPQ---------------SIDLN  139
_CAH0714714.1_    127  -----------------------------------------GYNHRNQPQ---------------SIDLN  140
_CAH0714713.1_    127  -----------------------------------------GYNHRNQPQ---------------SIDLN  140
_CAH4029256.1_    126  -----------------------------------------GFHHRNQPQ---------------TIDLN  139
_XP_047513081.1_  126  -----------------------------------------GFHHRNQPQ---------------TIDLN  139
_XP_045529569.1_  126  -----------------------------------------GFHHRNQPQ---------------TIDLN  139
_XP_045487500.1_  126  -----------------------------------------GFHHRNQPQ---------------TIDLN  139
_XP_047513077.1_  126  -----------------------------------------GFHHRNQPQ---------------TIDLN  139
_CAH4029257.1_    126  -----------------------------------------GFHHRNQPQ---------------TIDLN  139
_XP_045487498.1_  126  -----------------------------------------GFHHRNQPQ---------------TIDLN  139
_CAF4952989.1_    126  -----------------------------------------GFHHRNQPQ---------------TIDLN  139
_XP_045447470.1_  127  -----------------------------------------GYNHRNQPQ---------------SIDLN  140
_CAH2108688.1_     66  -----------------------------------------GYNHRNQPQ---------------SIDLN   79
_CAH0714724.1_    126  -----------------------------------------GFKHINDPK---------------NINLK  139
_XP_034827218.1_  127  -----------------------------------------GFNHRNQPQ---------------SIDLN  140
_XP_045768234.1_  127  -----------------------------------------GFNHRNQPQ---------------SIDLN  140
_XP_034827214.1_  127  -----------------------------------------GFNHRNQPQ---------------SIDLN  140
_XP_045768230.1_  127  -----------------------------------------GFNHRNQPQ---------------SIDLN  140
_XP_039751073.1_  127  -----------------------------------------GFNHRNQPQ---------------GIDLN  140
_XP_023942270.1_  127  -----------------------------------------GYNHRNQPQ---------------GIDLN  140
_XP_023942268.1_  127  -----------------------------------------GYNHRNQPQ---------------GIDLN  140
_XP_039751072.1_  127  -----------------------------------------GFNHRNQPQ---------------GIDLN  140
_XP_023942266.1_  127  -----------------------------------------GYNHRNQPQ---------------GIDLN  140
_CAH2240541.1_    114  -----------------------------------------GFNHRNQPQ---------------GIDLN  127
_XP_041985045.1_  127  -----------------------------------------GYAHRNQLQ---------------SIDLN  140
_XP_041985024.1_  127  -----------------------------------------GYAHRNQLQ---------------SIDLN  140
_XP_049868286.1_  127  -----------------------------------------GYAHRNQPQ---------------AIDLN  140
_XP_026314794.1_  127  -----------------------------------------GYAHRNQPQ---------------SIDLN  140
_XP_047994271.1_  126  -----------------------------------------GFSHKNQPQ---------------NIDLN  139
_KAJ2950301.1_    127  -----------------------------------------GYGHRNQPQ---------------SIDLN  140
```

```
_XP_031784347.2_  125  -----------------------------------------GYKPEKGVE---------------KMDIT  138
_KAG7305875.1_    125  -----------------------------------------GFSHKNSPQ---------------SIDLN  138
_WDD23886.1_      125  -----------------------------------------GFSHKNSPQ---------------SIDLN  138
_XP_048480424.1_  125  -----------------------------------------GFSHKNSPQ---------------SIDLN  138
_XP_048480423.1_  125  -----------------------------------------GFSHKNSPQ---------------SIDLN  138
_XP_048480422.1_  125  -----------------------------------------GFSHKNSPQ---------------SIDLN  138
_XP_048480421.1_  125  -----------------------------------------GFSHKNSPQ---------------SIDLN  138
_KAG7305876.1_    125  -----------------------------------------GFSHKNSPQ---------------SIDLN  138
_CAG9129242.1_    125  -----------------------------------------GFSHKNSPQ---------------SIDLN  138
_XP_048480420.1_  125  -----------------------------------------GFSHKNSPQ---------------SIDLN  138
_GBP53004.1_        1  -----------------------------------------GFKHKKEPS---------------SIDLN   14
_XP_045534737.1_  127  -----------------------------------------GFSHRAQPQ---------------SIDLN  140
_XP_013180060.1_  127  -----------------------------------------GFSHRAQPQ---------------SIDLN  140
_XP_013180043.1_  127  -----------------------------------------GFSHRAQPQ---------------SIDLN  140
_XP_013180068.1_  127  -----------------------------------------GFSHRAQPQ---------------SIDLN  140
_XP_045534736.1_  127  -----------------------------------------GFSHRAQPQ---------------SIDLN  140
_KPJ06840.1_      127  -----------------------------------------GFSHRAQPQ---------------SIDLN  140
_KPJ05518.1_      127  -----------------------------------------GFSHRAQPQ---------------SIDLN  140
_CAG4934519.1_    127  -----------------------------------------GFSHRSQPQ---------------SIDLN  140
_XP_013149112.1_  127  -----------------------------------------GFSHRGQPQ---------------SIDLN  140
_CAH2077165.1_    139  -----------------------------------------GFGHRAQPQ---------------SIDLN  152
_CAH2077167.1_    139  -----------------------------------------GFGHRAQPQ---------------SIDLN  152
_XP_037051415.1_  122  -----------------------------------------GFAHKKD-----------------KTFRT  133
_XP_037051414.1_  123  -----------------------------------------GFAHKKD-----------------KTFRT  134
_XP_022815079.1_  127  -----------------------------------------NFDHKNHPQ---------------SIDLN  140
_CAB3516522.1_    127  -----------------------------------------NFDHKNHPQ---------------SIDLN  140
_XP_050562120.1_  127  -----------------------------------------NFDHKNHPQ---------------SIDLN  140
_XP_050562121.1_  127  -----------------------------------------NFDHKNHPQ---------------SIDLN  140
_QHD56218.1_      127  -----------------------------------------NFDHKNHPQ---------------SIDLN  140
_AIA24466.1_      127  -----------------------------------------NFDHKNHPQ---------------SIDLN  140
_XP_022815076.1_  127  -----------------------------------------NFDHKNHPQ---------------SIDLN  140
_QHD56217.1_      127  -----------------------------------------NFDHKNHPQ---------------SIDLN  140
_XP_050562118.1_  127  -----------------------------------------NFDHKNHPQ---------------SIDLN  140
_AIA24467.1_      127  -----------------------------------------NFDHKNHPQ---------------SIDLN  140
_XP_047019808.1_  127  -----------------------------------------NFDHKNHPQ---------------SIDLN  140
_AEO51736.1_      127  -----------------------------------------NFDHKNHPQ---------------SIDLN  140
_XP_049696680.1_  127  -----------------------------------------NFDHKNHPQ---------------SIDLN  140
_XP_049696682.1_  127  -----------------------------------------NFDHKNHPQ---------------SIDLN  140
_XP_049696676.1_  127  -----------------------------------------NFDHKNHPQ---------------SIDLN  140
```

```
_XP_026744931.1_  127  -----------------------------------------NFDHKNHPQ---------------SIDLN  140
_XP_026732895.1_  127  -----------------------------------------NFDHKNHPQ---------------SIDLN  140
_CAH0581650.1_    127  -----------------------------------------NFDHKNHPQ---------------SIDLN  140
_KAH9640014.1_    131  LNLTQRFYLYLDHVSRTVFRIRIYSQAISFLYKSFYDVFSENFDHKNHPQ---------------SIDLN  185
_PZC78315.1_        1  -----------------------------------------NFDHKNHPQ---------------SIDLN   14
_KAF9795796.1_      1  -----------------------------------------NFDHKNHPQ---------------SIDLN   14
_AEC52926.1_      127  -----------------------------------------GFGHRNHPQ---------------SIDLN  140
_KAH7940301.1_    124  -----------------------------------------GFAHKDQAA---------------SIDLN  137
_ADK39025.1_      126  -----------------------------------------GFTHRNHPQ---------------GIDLN  139
_XP_037298148.1_  126  -----------------------------------------GFTHRNHPQ---------------GIDLN  139
_RVE50742.1_      127  -----------------------------------------GYGHKSQPQ---------------SIDLN  140
_CAH0692665.1_    127  -----------------------------------------GYGHKSQPQ---------------SIDLN  140
_XP_028166351.1_  126  -----------------------------------------GYAHRSQPQ---------------SIDLN  139
_XP_028166291.1_  126  -----------------------------------------GYAHRSQPQ---------------SIDLN  139
_CAB3235314.1_    127  -----------------------------------------GFGHMEQPQ---------------SIDLN  140
_KAJ0175867.1_    128  -----------------------------------------GFSHRNHPQ---------------SIDLN  141
_XP_028031027.1_  127  -----------------------------------------GFDHRSHTQ---------------SIDLN  140
_NP_001166296.1_  127  -----------------------------------------GFDHRSHTQ---------------SIDLN  140
_XP_037870194.1_  127  -----------------------------------------GFDHRSHTQ---------------SIDLN  140
_XP_037870193.1_  127  -----------------------------------------GFDHRSHTQ---------------SIDLN  140
_XP_028031025.1_  127  -----------------------------------------GFDHRSHTQ---------------SIDLN  140
_XP_013187225.1_  125  -----------------------------------------RYHHKNQPQ---------------SIDLN  138
_XP_052755011.1_  125  -----------------------------------------RYAHRNQPQ---------------SIDLN  138
_XP_053613182.1_  125  -----------------------------------------RYHHKNQPQ---------------SIDLN  138
_XP_053613180.1_  125  -----------------------------------------RYHHKNQPQ---------------SIDLN  138
_CAG9785863.1_    126  ------------------------------------------NSHRSQPQ---------------SIDLN  138
_KAG6461275.1_    126  -----------------------------------------GFTHRNHPQ---------------GIDLN  139
_KAG6461281.1_    126  -----------------------------------------GFTHRNHPQ---------------GIDLN  139
_XP_037302137.1_  125  -----------------------------------------GFSHRKKQQ---------------CIDMS  138
_KAG6461269.1_    125  -----------------------------------------GFSHRKKQQ---------------CIDMS  138
_KAG4069896.1_    121  -----------------------------------------GFAHKKDKT--------------FSPD--  133
_XP_044003989.1_  129  -----------------------------------------GFDCAYYSS--------------SSFDLS  143
_XP_045768240.1_  127  -----------------------------------------GFNHSKEPQ---------------SIDLN  140
_KAG9509099.1_    131  -----------------------------------------GFAHKSRMS---------------DIDLS  144
_XP_013118267.1_  125  -----------------------------------------KFAHKDQPS---------------TIDLN  138
_XP_005183026.2_  125  -----------------------------------------KFAHMDQPS---------------SIDLN  138
_XP_050303432.1_  133  -----------------------------------------GFCHKSNNR--------------TTISSK  147
_TMW48950.1_      125  -----------------------------------------GFAHKDQPS---------------SIDLN  138
_XP_037882085.1_  125  -----------------------------------------KFAHKDQPS---------------SIDLN  138
```

```
_KAI8126541.1_     126  -----------------------------------------SFAHKDQPS---------------SIDLN  139
_KAI8126540.1_     125  -----------------------------------------SFAHKDQPS---------------SIDLN  138
_XP_037811156.1_   125  -----------------------------------------SFAHKDQPS---------------SIDLN  138
_XP_046812595.1_   125  -----------------------------------------SFAHKDQPS---------------SIDLN  138
_XP_046812596.1_   125  -----------------------------------------SFAHKDQPS---------------SIDLN  138
_XP_046812591.1_   125  -----------------------------------------SFAHKDQPS---------------SIDLN  138
_XP_046812590.1_   125  -----------------------------------------SFAHKDQPS---------------SIDLN  138
_KNC34143.1_       125  -----------------------------------------SFAHKDQPS---------------SIDLN  138
_XP_046812589.1_   125  -----------------------------------------SFAHKDQPS---------------SIDLN  138
_KAI8126539.1_     125  -----------------------------------------SFAHKDQPS---------------SIDLN  138
_XP_037958917.1_   125  -----------------------------------------RFGHKDQPS---------------SIDLN  138
_XP_037929595.1_   127  -----------------------------------------KFGHKDQPA---------------SIDLN  140
_XP_037957513.1_   125  -----------------------------------------KFGHKDQPA---------------SIDLN  138
_XP_037958115.1_   127  -----------------------------------------KFGHKDQPA---------------SIDLN  140
_XP_037958114.1_   127  -----------------------------------------KFGHKDQPA---------------SIDLN  140
_XP_037957511.1_   125  -----------------------------------------KFGHKDQPA---------------SIDLN  138
_XP_037958918.1_   125  -----------------------------------------RFGHKDQPS---------------SIDLN  138
_RXG71823.1_       124  ------------------------------------------TRTSENPQ---------------SIDLN  136
_KAB7494622.1_      80  -------------------------------------------RTSEKPSINR--------PKFNSTLFS   98
_KAI9584339.1_     124  -----------------------------------------GFNHRSQTS---------------PIDLY  137
_XP_037884983.1_   124  -----------------------------------------GFNHRLETS---------------AIDLY  137
_XP_054744821.1_   125  -----------------------------------------GFDHINDPN---------------SIELN  138
_XP_053961676.1_   125  -----------------------------------------GFDHINDPN---------------SIELN  138
_XP_053961674.1_   125  -----------------------------------------GFDHINDPN---------------SIELN  138
_XP_004526634.2_   125  -----------------------------------------GFDHITDPN---------------SIELN  138
_CAD6998451.1_     125  -----------------------------------------GFDHITDPN---------------SIELN  138
_XP_012157894.1_   125  -----------------------------------------GFDHITDPN---------------SIELN  138
_XP_028894493.1_   125  -----------------------------------------GFDHIGDPN---------------SIELN  138
_XP_036327693.1_   125  -----------------------------------------GFDHISDPN---------------SIELN  138
_XP_017471333.1_   125  -----------------------------------------GFDHISDPN---------------SIELN  138
_XP_039952608.1_   125  -----------------------------------------GFDHITDPN---------------SIELN  138
_XP_050320023.1_   125  -----------------------------------------GFDHITDPN---------------SIELN  138
_XP_036225572.1_   125  -----------------------------------------GFDHITDPN---------------SIELN  138
_XP_039952607.1_   125  -----------------------------------------GFDHITDPN---------------SIELN  138
_XP_036225564.1_   125  -----------------------------------------GFDHITDPN---------------SIELN  138
_XP_036225569.1_   125  -----------------------------------------GFDHITDPN---------------SIELN  138
_XP_050320021.1_   125  -----------------------------------------GFDHITDPN---------------SIELN  138
_XP_039952606.1_   125  -----------------------------------------GFDHITDPN---------------SIELN  138
_XP_011207860.1_   125  -----------------------------------------GFDHISDPN---------------SIELN  138
```

```
_XP_011207859.1_  125  -----------------------------------------GFDHISDPN---------------SIELN  138
_XP_029407466.1_  125  -----------------------------------------GFDHISDPN---------------SIELN  138
_XP_019847003.2_  125  -----------------------------------------GFDHISDPN---------------SIELN  138
_XP_014086488.2_  125  -----------------------------------------GFDHITDPN---------------SIELN  138
_XP_049317393.1_  125  -----------------------------------------GFDHISDPN---------------SIELN  138
_XP_039952604.1_  125  -----------------------------------------GFDHITDPN---------------SIELN  138
_XP_018787557.1_  125  -----------------------------------------GFDHVTDPN---------------SIELN  138
_OQR68950.1_      125  -----------------------------------------NYEHRARPG---------------DIDLN  138
_XP_003741763.1_  125  -----------------------------------------RYDHMGKLT---------------DIDLN  138
_XP_022699297.1_  125  -----------------------------------------NYEHRARPG---------------DIDLN  138
_XP_022665229.1_  125  -----------------------------------------NYEHRARPG---------------DIDLN  138
_XP_022665225.1_  125  -----------------------------------------NYEHRARPG---------------DIDLN  138
_XP_022665224.1_  125  -----------------------------------------NYEHRARPG---------------DIDLN  138
_XP_033219687.1_  125  -----------------------------------------GFEHMDFPD---------------TIDLN  138
_XP_033227990.1_  125  -----------------------------------------GFEHMDFPD---------------TIDLN  138
_XP_043466013.1_  124  -----------------------------------------GFEHMEYPD---------------SIDLN  137
_XP_051172671.1_  124  -----------------------------------------GFDHMEFPD---------------SIDLN  137
_CAD7643228.1_    122  -----------------------------------------GFGHKSQAF--------------KIIDLA  136
_XP_054157157.1_  123  -----------------------------------------GFSHRGQAF--------------KVIDLA  137
_CAD7645633.1_    122  -----------------------------------------GFGHKSQAF--------------KIIDLA  136
_CAD7632611.1_     64  -----------------------------------------GFGHKSQAF--------------KIIDLA   78
_KAJ6223158.1_    121  -----------------------------------------GFNHRSHTF--------------KLIDLN  135
_KAI2804035.1_    121  -----------------------------------------GFNHRSHTF--------------KLIDLN  135
_KAH9398209.1_    113  -----------------------------------------GFSHRLHTF--------------KLIDLN  127
_GFY77286.1_      125  -----------------------------------------GFSHHLEPN---------------KINLN  138
_GFQ73388.1_      125  -----------------------------------------GFSHHLEPN---------------KINLN  138
_GFT25575.1_      125  -----------------------------------------GFSHHLEPN---------------KINLN  138
_KAF8763655.1_    125  -----------------------------------------GFSHHLEPN---------------KINLN  138
_GFX76677.1_      125  -----------------------------------------GFSHHLEPN---------------KINLN  138
_XP_035207052.1_  125  -----------------------------------------GFSHHLEPN---------------KINLN  138
_KFM74898.1_      125  -----------------------------------------GFSHHLEPN---------------KINLN  138
_XP_035207059.1_  125  -----------------------------------------GFSHHLEPN---------------KINLN  138
_XP_035207066.1_  125  -----------------------------------------GFSHHLEPN---------------KINLN  138
_BBD75287.1_      125  -----------------------------------------GFSHHLEPG---------------KINLN  138
_XP_042900952.1_  125  -----------------------------------------GFSHHLEPG---------------KINLN  138
_KAG8201816.1_    124  -----------------------------------------GFSHYTEPN---------------KINLN  137
_GIX87435.1_      125  -----------------------------------------GFSHHLEPN---------------KINLN  138
_GBL81029.1_      125  -----------------------------------------GFSHHLEPN---------------KINLN  138
_GIY01087.1_      125  -----------------------------------------GFSHHLEPN---------------KINLN  138
```

```
_XP_054715436.1_   125  -----------------------------------------GFSHVKEPS---------------KINLN  138
_XP_044576112.1_   122  -----------------------------------------GFQHMEVPD---------------TIDLS  135
_XP_008556435.3_   121  -----------------------------------------GFNHKNSIS---------------DIDLS  134
_CAD6234446.1_     122  -----------------------------------------GFKHMKLPD---------------IIDLT  135
_XP_044284598.1_   124  -----------------------------------------PEEQLLAIE---------------EYDLN  137
_XP_033794511.1_   124  -----------------------------------------PEEQLPSIE---------------DYDLN  137
_XP_033794512.1_   124  -----------------------------------------PEEQLPSIE---------------DYDLN  137
_XP_029449578.1_   124  -----------------------------------------PEEQLLTIE---------------DYDLN  137
_XP_030052048.1_   124  -----------------------------------------PEEQLSTLE---------------DYDLN  137
_XP_007423639.1_   124  -----------------------------------------SEEQLLAVE---------------EYDLN  137
_XP_039203483.1_   124  -----------------------------------------SEEQLLIVE---------------EYDLN  137
_XP_034283206.1_   124  -----------------------------------------SEEQLLTVE---------------EYDLN  137
_XP_033001023.1_   124  -----------------------------------------SEEDLMAIE---------------EYNLN  137
_XP_053239675.1_   124  -----------------------------------------SEEELMAIE---------------EYNLN  137
_XP_034966804.1_   124  -----------------------------------------SEEELMAIE---------------EYNLN  137
_XP_028580982.1_   113  -----------------------------------------SEEELMAIE---------------EYNLN  126
_XP_042302822.1_   124  -----------------------------------------SEEQILAVD---------------EYELN  137
_XP_015280979.1_   113  -----------------------------------------SQEQLLAIE---------------EYDLN  126
_XP_048363544.1_   124  -----------------------------------------REDQLLAIE---------------EYDLN  137
_KAF7252806.1_      98  -----------------------------------------PEEQLLAIE---------------EYDLN  111
_XP_034958398.1_     3  -----------------------------------------SEEELMAIE---------------EYNLN   16
_CAD5116312.1_     126  -----------------------------------------GFDHINDPS---------------NIDAH  139
_KAJ1519398.1_     123  -----------------------------------------GFGHQDHVE---------------DIDVD  136
_XP_053103001.1_   124  -----------------------------------------SEEQLLAIE---------------EYDLN  137
_XP_032070730.1_   124  -----------------------------------------SEEQLLTVE---------------EYDLN  137
_XP_013922116.1_   124  -----------------------------------------SEEQLLTVE---------------EYDLN  137
_XP_015667999.1_   124  -----------------------------------------SEEQLLTVE---------------EYDLN  137
_XP_026551846.1_   124  -----------------------------------------SEEQLLTVE---------------EYDLN  137
_XP_026532518.1_   124  -----------------------------------------SEEQLLTVE---------------EYDLN  137
_KAJ7341301.1_     124  -----------------------------------------PEQQLLAVE---------------EYDLN  137
_KAJ6667038.1_     113  -----------------------------------------PEEQLLAIE---------------EYDLN  126
_XP_003227818.2_   113  -----------------------------------------SEDQMLAID---------------EYDLS  126
_XP_020633036.1_   113  -----------------------------------------PEEQLLAVE---------------EYDLN  126
_XP_054842059.1_   124  -----------------------------------------SQEQLMAIE---------------EYDLN  137
_XP_005238641.2_   113  -----------------------------------------PEEQLHNID---------------EYDLN  126
_XP_005445671.2_   113  -----------------------------------------PEEQLHNID---------------EYDLN  126
_XP_054050029.1_   113  -----------------------------------------PEEQLHNID---------------EYDLN  126
_XP_013810845.1_   113  -----------------------------------------PEEQLHNID---------------EYDLN  126
_XP_026702002.1_   113  -----------------------------------------PEEQLHNID---------------EYDLN  126
```

```
_XP_025892242.1_  113  -----------------------------------------PEEQLHNID---------------EYDLN  126
_XP_005507389.1_  113  -----------------------------------------PEEQLHNID---------------EYDLN  126
_XP_032911247.1_  113  -----------------------------------------PEEQLHNID---------------EYDLN  126
_XP_031959657.1_  113  -----------------------------------------PEEQLHNID---------------EYDLN  126
_XP_041905291.1_  113  -----------------------------------------PEEQLHNID---------------EYDLN  126
_XP_051648836.1_  113  -----------------------------------------PEEQLHNID---------------EYDLN  126
_XP_054239488.1_  113  -----------------------------------------PEEQLHNID---------------EYDLN  126
_XP_039914164.1_  113  -----------------------------------------PEEQLHNID---------------EYDLN  126
_XP_005422689.1_  113  -----------------------------------------PEEQLHNID---------------EYDLN  126
_KAF2987757.1_    113  -----------------------------------------PEEQLHNID---------------EYDLN  126
_XP_038665087.1_  124  -----------------------------------------PQEQLLQVE---------------DYDLN  137
_GCB59945.1_      124  -----------------------------------------PQEQLLQVE---------------DYDLN  137
_XP_041065616.1_  120  -----------------------------------------PQEPLLQVE---------------DYDLN  133
_XP_007890564.1_  124  -----------------------------------------PQEQLLQIE---------------DYDLN  137
_XP_051868609.1_  124  -----------------------------------------PQEQLLQIE---------------DYDLN  137
_XP_032881051.1_  123  -----------------------------------------PQEELLQIA---------------SYDLN  136
_XP_048456938.1_   33  -----------------------------------------TQEQLLQIE---------------DIDLN   46
_KAJ1155552.1_    124  -----------------------------------------PEDKLLIIE---------------DYDLN  137
_KAH0622877.1_      1  -------------------------------------------------D---------------EYDLN    6
_CAI5653329.1_    124  -----------------------------------------PREQLLQTE---------------EYDLN  137
_CAI5653328.1_    124  -----------------------------------------PREQLLQTE---------------EYDLN  137
_NXS54806.1_      131  -----------------------------------------PEEQLHNID---------------EYDLN  144
_XP_009504395.1_  121  -----------------------------------------PEEQLHNID---------------EYDLN  134
_NXJ41381.1_      124  -----------------------------------------PEEQLHNID---------------EYDLN  137
_NXT24719.1_      124  -----------------------------------------PEEQLHNID---------------EYDLN  137
_NXK23502.1_      124  -----------------------------------------PEEQLHNID---------------EYDLN  137
_NXK03070.1_      124  -----------------------------------------PEEQLHNID---------------EYDLN  137
_XP_037260944.1_  124  -----------------------------------------PEEQLHNID---------------EYDLN  137
_XP_040467369.1_  124  -----------------------------------------PEEQLHNID---------------EYDLN  137
_NXY71491.1_      124  -----------------------------------------PEEQLHNID---------------EYDLN  137
_NXN54417.1_      124  -----------------------------------------PEEQLHNID---------------EYDLN  137
_NXX19490.1_      124  -----------------------------------------PEEQLHNID---------------EYDLN  137
_NXL47199.1_      124  -----------------------------------------PEEQLHNID---------------EYDLN  137
_XP_050749583.1_  124  -----------------------------------------PEEQLHNID---------------EYDLN  137
_NXD76242.1_      124  -----------------------------------------PEEQLHNID---------------EYDLN  137
_KFZ56827.1_      124  -----------------------------------------PEEQLHNID---------------EYDLN  137
_NXH67691.1_      124  -----------------------------------------PEEQLHNID---------------EYDLN  137
_NXS45828.1_      124  -----------------------------------------PEEQLHNID---------------EYDLN  137
_KFP52034.1_      124  -----------------------------------------PEEQLHNID---------------EYDLN  137
```

```
_NWH44485.1_      124  -----------------------------------------PEEQLHNID---------------EYDLN  137
_NWW85416.1_      124  -----------------------------------------PEEQLHNID---------------EYDLN  137
_NWU66320.1_      124  -----------------------------------------PEEQLHNID---------------EYDLN  137
_XP_025919906.1_  124  -----------------------------------------PEEQLHNID---------------EYDLN  137
_KAF1483947.1_    124  -----------------------------------------PEEQLHNID---------------EYDLN  137
_NXQ87688.1_      124  -----------------------------------------PEEQLHNID---------------EYDLN  137
_NXX07562.1_      124  -----------------------------------------PEEQLHNID---------------EYDLN  137
_XP_009280788.2_  124  -----------------------------------------PEEQLHNID---------------EYDLN  137
_XP_014821857.1_  124  -----------------------------------------PEEQLHNID---------------EYDLN  137
_NWU51443.1_      124  -----------------------------------------PEEQLHNID---------------EYDLN  137
_NXV35521.1_      124  -----------------------------------------PEEQLHNID---------------EYDLN  137
_KFV80780.1_      124  -----------------------------------------PEEQLHNID---------------EYDLN  137
_XP_032039522.1_  124  -----------------------------------------PEEQLHNID---------------EYDLN  137
_NWT43027.1_      124  -----------------------------------------PEEQLHNID---------------EYDLN  137
_KAF1651581.1_    124  -----------------------------------------PEEQLHNID---------------EYDLN  137
_NXU26374.1_      124  -----------------------------------------PEEQLHNID---------------EYDLN  137
_NWX46229.1_      124  -----------------------------------------PEEQLHNID---------------EYDLN  137
_PKU40340.1_      124  -----------------------------------------PEEQLHNID---------------EYDLN  137
_XP_027309078.1_  124  -----------------------------------------PEEQLHNID---------------EYDLN  137
_KAF1573683.1_    124  -----------------------------------------PEEQLHNID---------------EYDLN  137
_KAF1578928.1_    124  -----------------------------------------PEEQLHNID---------------EYDLN  137
_NXN47104.1_      124  -----------------------------------------PEEQLHNID---------------EYDLN  137
_NXV20393.1_      124  -----------------------------------------PEEQLHNID---------------EYDLN  137
_KAF1551267.1_    124  -----------------------------------------PEEQLHNID---------------EYDLN  137
_KAF1406210.1_    124  -----------------------------------------PEEQLHNID---------------EYDLN  137
_NXV43201.1_      124  -----------------------------------------PEEQLHNID---------------EYDLN  137
_NWQ98669.1_      124  -----------------------------------------PEEQLHNID---------------EYDLN  137
_XP_052639968.1_  124  -----------------------------------------PEEQLHNID---------------EYDLN  137
_XP_010573143.1_  124  -----------------------------------------PEEQLHNID---------------EYDLN  137
_KAF1479604.1_    124  -----------------------------------------PEEQLHNID---------------EYDLN  137
_NXG89109.1_      124  -----------------------------------------PEEQLHNID---------------EYDLN  137
_KFQ89757.1_      124  -----------------------------------------PEEQLHNID---------------EYDLN  137
_NWZ21787.1_      124  -----------------------------------------PEEQLHNID---------------EYDLN  137
_NXI74048.1_      124  -----------------------------------------PEEQLHNID---------------EYDLN  137
_NWX76247.1_      124  -----------------------------------------PEEQLHNID---------------EYDLN  137
_NXF29788.1_      124  -----------------------------------------PEEQLHNID---------------EYDLN  137
_KAF1444142.1_    124  -----------------------------------------PEEQLHNID---------------EYDLN  137
_KFV20215.1_      124  -----------------------------------------PEEQLHNID---------------EYDLN  137
_NXI44657.1_      124  -----------------------------------------PEEQLHNID---------------EYDLN  137
```

```
_NXJ92321.1_       124  -----------------------------------------PEEQLHNID---------------EYDLN  137
_NWZ49902.1_       124  -----------------------------------------PEEQLHNID---------------EYDLN  137
_NWY59444.1_       124  -----------------------------------------PEEQLHNID---------------EYDLN  137
_KFW64019.1_       124  -----------------------------------------PEEQLHNID---------------EYDLN  137
_NXJ55634.1_       124  -----------------------------------------PEEQLHNID---------------EYDLN  137
_KAF1513372.1_     124  -----------------------------------------PEEQLHNID---------------EYDLN  137
_NXW14598.1_       124  -----------------------------------------PEEQLHNID---------------EYDLN  137
_NXC74247.1_       124  -----------------------------------------PEEQLHNID---------------EYDLN  137
_NXT57254.1_       124  -----------------------------------------PEEQLHNID---------------EYDLN  137
_XP_047916679.1_   124  -----------------------------------------PEEQLHNID---------------EYDLN  137
_XP_029879190.1_   124  -----------------------------------------PEEQLHNID---------------EYDLN  137
_NXH09241.1_       124  -----------------------------------------PEEQLHNID---------------EYDLN  137
_XP_040407374.1_   124  -----------------------------------------PEEQLHNID---------------EYDLN  137
_KFW76037.1_       124  -----------------------------------------PEEQLHNID---------------EYDLN  137
_KFP65945.1_       124  -----------------------------------------PEEQLHNID---------------EYDLN  137
_NXX55682.1_       124  -----------------------------------------PEEQLHNID---------------EYDLN  137
_XP_035178333.1_   124  -----------------------------------------PEEQLHNID---------------EYDLN  137
_NXE47317.1_       124  -----------------------------------------PEEQLHNID---------------EYDLN  137
_XP_035408877.1_   124  -----------------------------------------PEEQLHNID---------------EYDLN  137
_KGL94141.1_       124  -----------------------------------------PEEQLHNID---------------EYDLN  137
_NXN68403.1_       124  -----------------------------------------PEEQLHNID---------------EYDLN  137
_NXW28331.1_       124  -----------------------------------------PEEQLHNID---------------EYDLN  137
_XP_049657509.1_   124  -----------------------------------------PEEQLHNID---------------EYDLN  137
_XP_049657511.1_   124  -----------------------------------------PEEQLHNID---------------EYDLN  137
_NXG30098.1_       124  -----------------------------------------PEEQLHNID---------------EYDLN  137
_NXV86645.1_       124  -----------------------------------------PEEQLHNID---------------EYDLN  137
_NXX83200.1_       124  -----------------------------------------PEEQLHNID---------------EYDLN  137
_NXI59898.1_       124  -----------------------------------------PEEQLHNID---------------EYDLN  137
_NWU89521.1_       124  -----------------------------------------PEEQLHNID---------------EYDLN  137
_NXK45490.1_       124  -----------------------------------------PEEQLHNID---------------EYDLN  137
_NWR55599.1_       124  -----------------------------------------PEEQLHNID---------------EYDLN  137
_NXL37824.1_       124  -----------------------------------------PEEQLHNID---------------EYDLN  137
_NWI33553.1_       124  -----------------------------------------PEEQLHNID---------------EYDLN  137
_KFQ74517.1_       124  -----------------------------------------PEEQLHNID---------------EYDLN  137
_NXV82662.1_       124  -----------------------------------------PEEQLHNID---------------EYDLN  137
_NWJ10087.1_       124  -----------------------------------------PEEQLHNID---------------EYDLN  137
_NXN24483.1_       124  -----------------------------------------PEEQLHNID---------------EYDLN  137
_NWR80208.1_       124  -----------------------------------------PEEQLHNID---------------EYDLN  137
_NXT37538.1_       124  -----------------------------------------PEEQLHNID---------------EYDLN  137
```

```
_XP_032844994.2_  124  -----------------------------------------PEEQLHNID---------------EYDLN  137
_NXW47649.1_      124  -----------------------------------------PEEQLHNID---------------EYDLN  137
_NXA40398.1_      124  -----------------------------------------PEEQLHNID---------------EYDLN  137
_NXW64893.1_      124  -----------------------------------------PEEQLHNID---------------EYDLN  137
_NXX89165.1_      124  -----------------------------------------PEEQLHNID---------------EYDLN  137
_NXQ96911.1_      124  -----------------------------------------PEEQLHNID---------------EYDLN  137
_KFP18273.1_      124  -----------------------------------------PEEQLHNID---------------EYDLN  137
_NXC40882.1_      124  -----------------------------------------PEEQLHNID---------------EYDLN  137
_NXE10305.1_      124  -----------------------------------------PEEQLHNID---------------EYDLN  137
_KFP88087.1_      124  -----------------------------------------PEEQLHNID---------------EYDLN  137
_NWW47871.1_      124  -----------------------------------------PEEQLHNID---------------EYDLN  137
_NXJ61595.1_      124  -----------------------------------------PEEQLHNID---------------EYDLN  137
_NXD13814.1_      124  -----------------------------------------PEEQLHNID---------------EYDLN  137
_KFR16961.1_      124  -----------------------------------------PEEQLHNID---------------GYDLN  137
_NWX24312.1_      124  -----------------------------------------PEEQLHNID---------------EYDLN  137
_NXA28267.1_      124  -----------------------------------------PEEQLHNID---------------EYDLN  137
_NXL66270.1_      124  -----------------------------------------PEEQLHNID---------------EYDLN  137
_NXA49976.1_      124  -----------------------------------------PEEQLHNID---------------EYDLN  137
_NXT77624.1_      124  -----------------------------------------PEEQLHNID---------------EYDLN  137
_KFQ92336.1_      124  -----------------------------------------PEEQLHNID---------------EYDLN  137
_NXE25013.1_      124  -----------------------------------------PEEQLHNID---------------EYDLN  137
_NXE92240.1_      124  -----------------------------------------PEEQLHNID---------------EYDLN  137
_NWH80943.1_      124  -----------------------------------------PEEQLHNID---------------EYDLN  137
_NWI19058.1_      124  -----------------------------------------PEEQLHNID---------------EYDLN  137
_NXG80610.1_      124  -----------------------------------------PEEQLHNID---------------EYDLN  137
_NWR01486.1_      124  -----------------------------------------PEEQLHNID---------------EYDLN  137
_NXK81337.1_      124  -----------------------------------------PEEQLHNID---------------EYDLN  137
_NXS90563.1_      124  -----------------------------------------PEEQLHNID---------------EYDLN  137
_NXC17430.1_      124  -----------------------------------------PEEQLHNID---------------EYDLN  137
_NXE77245.1_      124  -----------------------------------------PEEQLHNID---------------EYDLN  137
_KFQ29788.1_      125  -----------------------------------------PEEQLHNID---------------EYDLN  138
_KFO89327.1_      124  -----------------------------------------PEEQLHNID---------------EYDLN  137
_NXD64381.1_      124  -----------------------------------------PEEQLHNID---------------EYDLN  137
_NXY45081.1_      124  -----------------------------------------PEEQLHNID---------------EYDLN  137
_KGL72871.1_      124  -----------------------------------------PEEQLHNID---------------EYDLN  137
_XP_053919504.1_  124  -----------------------------------------PEEQLHNID---------------EYDLN  137
_NXL85459.1_      124  -----------------------------------------PEEQLHNID---------------EYDLN  137
_NXO02254.1_      124  -----------------------------------------PEEQLHNID---------------EYDLN  137
_NXJ76034.1_      124  -----------------------------------------PEEQLHNID---------------EYDLN  137
```

```
_NXM93491.1_       124  -----------------------------------------PEEQLHNID---------------EYDLN  137
_XP_030345800.1_   124  -----------------------------------------PEEQLHNID---------------EYDLN  137
_XP_030302544.1_   124  -----------------------------------------PEEQLHNID---------------EYDLN  137
_XP_030345801.1_   124  -----------------------------------------PEEQLHNID---------------EYDLN  137
_KQK77724.1_       124  -----------------------------------------PEEQLHNID---------------EYDLN  137
_NXP09913.1_       124  -----------------------------------------PEEQLHNID---------------EYDLN  137
_NXR33018.1_       124  -----------------------------------------PEEQLHNID---------------EYDLN  137
_NXM76881.1_       124  -----------------------------------------PEEQLHNID---------------EYDLN  137
_NXS27423.1_       124  -----------------------------------------PEEQLHNID---------------EYDLN  137
_NXM55821.1_       124  -----------------------------------------PEEQLHNID---------------EYDLN  137
_XP_054678500.1_   124  -----------------------------------------PEEQLHNID---------------EYDLN  137
_XP_015712248.1_   124  -----------------------------------------PEEQLHNID---------------EYDLN  137
_NWX92058.1_       124  -----------------------------------------PEEQLHNID---------------EYDLN  137
_XP_048793646.1_   124  -----------------------------------------PEEQLHNID---------------EYDLN  137
_NXF04247.1_       124  -----------------------------------------PEEQLHNID---------------EYDLN  137
_XP_042683331.1_   124  -----------------------------------------PEEQLHNID---------------EYDLN  137
_NWV22147.1_       124  -----------------------------------------PEEQLHNID---------------EYDLN  137
_NXA04361.1_       124  -----------------------------------------PEEQLHNID---------------EYDLN  137
_XP_010116198.1_   130  -----------------------------------------PEEQLHNID---------------EYDLN  143
_NXG59741.1_       124  -----------------------------------------PEEQLHNID---------------EYDLN  137
_XP_042735326.1_   124  -----------------------------------------PEEQLHNID---------------EYDLN  137
_NXM29886.1_       124  -----------------------------------------PEEQLHNID---------------EYDLN  137
_NP_001161198.2_   124  -----------------------------------------PEEQLHNID---------------EYDLN  137
_XP_031451029.1_   124  -----------------------------------------PEEQLHNID---------------EYDLN  137
_NXR49262.1_       124  -----------------------------------------PEEQLHNID---------------EYDLN  137
_XP_052555416.1_   124  -----------------------------------------PEEQLHNID---------------EYDLN  137
_NWZ67085.1_       124  -----------------------------------------PEEQLHNID---------------EYDLN  137
_P16236.2_         124  -----------------------------------------PEEQLHNID---------------EYDLN  137
_XP_050566010.1_   124  -----------------------------------------PEEQLHNID---------------EYDLN  137
_NWS67030.1_       124  -----------------------------------------PEEQLHNID---------------EYDLN  137
_XP_005143850.3_   124  -----------------------------------------PEEQLHNID---------------EYDLN  137
_XP_050566011.1_   124  -----------------------------------------PEEQLHNID---------------EYDLN  137
_XP_050566009.1_   124  -----------------------------------------PEEQLHNID---------------EYDLN  137
_NWR30963.1_       124  -----------------------------------------PEEQLHNID---------------EYDLN  137
_NWS17896.1_       124  -----------------------------------------PEEQLHNID---------------EYDLN  137
_XP_021248574.1_   124  -----------------------------------------PEEQLHNID---------------EYDLN  137
_AAA48721.1_       124  -----------------------------------------PEEQLHNID---------------EYDLN  137
_NWI54208.1_       124  -----------------------------------------PEEQLHNID---------------EYDLN  137
_XP_027509532.1_   124  -----------------------------------------PEEQLHNID---------------EYDLN  137
```

```
_NXP71979.1_       124  -----------------------------------------PEEQLHNID---------------EYDLN  137
_NXX25352.1_       124  -----------------------------------------PEEQLHNID---------------EYDLN  137
_NXI18618.1_       124  -----------------------------------------PEEQLHNID---------------EYDLN  137
_NXS09623.1_       124  -----------------------------------------PEEQLHNID---------------EYDLN  137
_NXU77980.1_       124  -----------------------------------------PEEQLHNID---------------EYDLN  137
_XP_027509533.1_   124  -----------------------------------------PEEQLHNID---------------EYDLN  137
_NWV71036.1_       124  -----------------------------------------PEEQLHNID---------------EYDLN  137
_NXF89482.1_       124  -----------------------------------------PEEQLHNID---------------EYDLN  137
_NWY39470.1_       124  -----------------------------------------PEEQLHNID---------------EYDLN  137
_NXT59960.1_       124  -----------------------------------------PEEQLHNID---------------EYDLN  137
_NWU74340.1_       124  -----------------------------------------PEEQLHNID---------------EYDLN  137
_NXB84481.1_       124  -----------------------------------------PEEQLHNID---------------EYDLN  137
_NWV73885.1_       124  -----------------------------------------PEEQLHNID---------------EYDLN  137
_OPJ58894.1_       124  -----------------------------------------PEEQLHNID---------------EYDLN  137
_XP_048152104.1_   124  -----------------------------------------PEEQLHNID---------------EYDLN  137
_XP_054036037.1_   124  -----------------------------------------PEEQLHNID---------------EYDLN  137
_NXR18149.1_       124  -----------------------------------------PEEQLHNID---------------EYDLN  137
_NXP97533.1_       124  -----------------------------------------PEEQLHNID---------------EYDLN  137
_NXJ05495.1_       124  -----------------------------------------PEEQLHNID---------------EYDLN  137
_XP_032538932.1_   124  -----------------------------------------PEEQLHNID---------------EYDLN  137
_NWI96387.1_       124  -----------------------------------------PEEQLHNID---------------EYDLN  137
_NXR65995.1_       124  -----------------------------------------PEEQLHNID---------------EYDLN  137
_NXK42398.1_       124  -----------------------------------------PEEQLHNID---------------EYDLN  137
_XP_032538931.1_   124  -----------------------------------------PEEQLHNID---------------EYDLN  137
_NXY33181.1_       124  -----------------------------------------PEEQLHNID---------------EYDLN  137
_XP_050171009.1_   124  -----------------------------------------PEEQLHNID---------------EYDLN  137
_NXO50939.1_       124  -----------------------------------------PEEQLHNID---------------EYDLN  137
_NWZ32937.1_       124  -----------------------------------------PEEQLHNID---------------EYDLN  137
_NXP01828.1_       124  -----------------------------------------PEEQLHNID---------------DYDLN  137
_NXL00458.1_       124  -----------------------------------------PEEQLHNID---------------EYDLN  137
_XP_010401310.4_   124  -----------------------------------------PEEQLHNID---------------EYDLN  137
_NXH62149.1_       124  -----------------------------------------PEEQLHNID---------------EYDLN  137
_NXG43824.1_       124  -----------------------------------------PEEQLHNID---------------EYDLN  137
_XP_017677337.1_   124  -----------------------------------------PEEQLHNID---------------EYDLN  137
_NXU09156.1_       124  -----------------------------------------PEEQLHNID---------------EYDLN  137
_NXB38645.1_       124  -----------------------------------------PEEQLHNID---------------EYDLN  137
_XP_027594504.1_   124  -----------------------------------------PEEQLHNID---------------EYDLN  137
_NXM46269.1_       124  -----------------------------------------PEEQLHNID---------------EYDLN  137
_NWS79257.1_       124  -----------------------------------------PEEQLHNID---------------EYDLN  137
```

```
_NXB04839.1_      124  -----------------------------------------PEEQLHNID---------------EYDLN  137
_NXC62865.1_      124  -----------------------------------------PEEQLHNID---------------EYDLN  137
_NXN18277.1_      124  -----------------------------------------PEEQLHNID---------------EYDLN  137
_XP_027760039.1_  124  -----------------------------------------PEEQLHNID---------------EYDLN  137
_NXI90311.1_      124  -----------------------------------------PEEQLHNID---------------EYDLN  137
_NWY17003.1_      124  -----------------------------------------PEEQLHNID---------------EYDLN  137
_NXH97099.1_      124  -----------------------------------------PEEQLHNID---------------EYDLN  137
_NWS97308.1_      124  -----------------------------------------PEEQLHNID---------------EYDLN  137
_NWU45160.1_      124  -----------------------------------------PEEQLHNVD---------------EYDLN  137
_NXQ43004.1_      124  -----------------------------------------PEEQLHNID---------------EYDLN  137
_XP_027549142.1_  124  -----------------------------------------PEEQLHNID---------------EYDLN  137
_XP_017933082.2_  124  -----------------------------------------PEEQLHNID---------------EYDLN  137
_NWV96326.1_      124  -----------------------------------------PEEQLHNID---------------EYDLN  137
_NXP57783.1_      124  -----------------------------------------PEEQLHNID---------------EYDLN  137
_NXY57955.1_      124  -----------------------------------------PEEQLHNID---------------EYDLN  137
_AAA99433.1_      124  -----------------------------------------PEEQLHNID---------------EYDLN  137
_NXY21218.1_      124  -----------------------------------------PEEQLHNID---------------EYDLN  137
_NXX50824.1_      124  -----------------------------------------PEEQLHNID---------------EYDLN  137
_XP_030124623.3_  124  -----------------------------------------PEEQLHNID---------------EYDLN  137
_NXA92235.1_      124  -----------------------------------------PEEQLHNID---------------EYDLN  137
_NWI05522.1_      124  -----------------------------------------PEEQLHNID---------------EYDLN  137
_NWX29009.1_      124  -----------------------------------------PEEQLHNID---------------EYDLN  137
_XP_027549144.1_  124  -----------------------------------------PEEQLHNID---------------EYDLN  137
_NWW35420.1_      124  -----------------------------------------PEEQLHNID---------------EYDLN  137
_NWV07969.1_      124  -----------------------------------------PEEQLHNID---------------EYDLN  137
_NXH42214.1_      124  -----------------------------------------PEEQLHNID---------------EYDLN  137
_NXQ18773.1_      124  -----------------------------------------PEEQLHNID---------------EYDLN  137
_NWX10672.1_      124  -----------------------------------------PEEQLHNID---------------EYDLN  137
_NXU50687.1_      124  -----------------------------------------PEEQLHNID---------------EYDLN  137
_KFU84784.1_      124  -----------------------------------------PEEQLHNID---------------EYDLN  137
_NXQ52438.1_      124  -----------------------------------------PEEQLHNID---------------EYDLN  137
_NWW24536.1_      124  -----------------------------------------PEEQLHNID---------------EYDLN  137
_NXF73449.1_      124  -----------------------------------------PEEQLHNID---------------EYDLN  137
_XP_050827906.1_  124  -----------------------------------------PEEQLHNID---------------EYDLN  137
_NWX65205.1_      124  -----------------------------------------PEEQLHNID---------------EYDLN  137
_KFW81749.1_      124  -----------------------------------------PEEQLHNID---------------EYDLN  137
_XP_014750593.1_  124  -----------------------------------------PEEQLHNID---------------EYDLN  137
_NXJ17066.1_      124  -----------------------------------------PEEQLHNID---------------EYDLN  137
_NWW67019.1_      124  -----------------------------------------PEEQLHNID---------------EYDLN  137
```

```
_NXH03360.1_       124  -----------------------------------------PEEQLHNID---------------EYDLN  137
_XP_037987525.1_   124  -----------------------------------------PEEQLHNID---------------EYDLN  137
_NXL74522.1_       124  -----------------------------------------PEEQLHNID---------------EYDLN  137
_NWQ60824.1_       124  -----------------------------------------PEEQLHNID---------------EYDLN  137
_NXR70971.1_       124  -----------------------------------------PEEQLHNID---------------EYDLN  137
_XP_051468947.1_   124  -----------------------------------------PEEQLHNID---------------EYDLN  137
_NXB50199.1_       124  -----------------------------------------PEEQLHNID---------------EYDLN  137
_NXP18380.1_       124  -----------------------------------------PEEQLHNID---------------EYDLN  137
_NXI84319.1_       124  -----------------------------------------PEEQLHNID---------------EYDLN  137
_NWH33757.1_       124  -----------------------------------------PEEQLHNID---------------EYDLN  137
_NWR99781.1_       124  -----------------------------------------PEEQLHNID---------------EYDLN  137
_NXW94404.1_       124  -----------------------------------------PEEQLHNID---------------EYDLN  137
_CAA27220.1_       124  -----------------------------------------PEEQLHNID---------------EYDLN  137
_XP_015477679.1_   124  -----------------------------------------PEEQLHNID---------------EYDLN  137
_NWS32597.1_       124  -----------------------------------------PEEQLHNID---------------EYDLN  137
_NXG22126.1_       124  -----------------------------------------PEEQLHNID---------------EYDLN  137
_NWY97127.1_       124  -----------------------------------------PEEQLHNID---------------EYDLN  137
_NXW80148.1_       124  -----------------------------------------PEEQLHNID---------------EYDLN  137
_NXC27367.1_       124  -----------------------------------------PEEQLHNID---------------EYDLN  137
_NWH83726.1_       124  -----------------------------------------PEEQLHNID---------------EYDLN  137
_NXA73512.1_       124  -----------------------------------------PEEQLHNID---------------EYDLN  137
_NWZ83230.1_       124  -----------------------------------------PEEQLHNID---------------EYDLN  137
_XP_005525572.2_   124  -----------------------------------------PEEQLHNID---------------EYDLN  137
_NXD20033.1_       124  -----------------------------------------PEEQLHNID---------------EYDLN  137
_NXE37177.1_       124  -----------------------------------------PEEQLHNID---------------EYDLN  137
_NWV44369.1_       124  -----------------------------------------PEEQLHNID---------------EYDLN  137
_NXC81400.1_       124  -----------------------------------------PEEQLHNID---------------EYDLN  137
_NXA67345.1_       124  -----------------------------------------PEEQLHNID---------------EYDLN  137
_NXB55155.1_       124  -----------------------------------------PEEQLHNID---------------EYDLN  137
_NXN81229.1_       124  -----------------------------------------PEEQLQNID---------------EYDLN  137
_NXM15601.1_       124  -----------------------------------------PEEQLHNID---------------EYDLN  137
_XP_054484850.1_   124  -----------------------------------------PEEQLHNID---------------EYDLN  137
_NWU02685.1_       124  -----------------------------------------PEEQLHNID---------------EYDLN  137
_XP_041273165.1_   124  -----------------------------------------PEEQLHNID---------------EYDLN  137
_NWW80397.1_       124  -----------------------------------------PEEQLHNID---------------EYDLN  137
_XP_036236540.1_   124  -----------------------------------------PEEQLHNID---------------EYDLN  137
_NWV50957.1_       124  -----------------------------------------PEEQLHNID---------------EYDLN  137
_NXT97287.1_       124  -----------------------------------------PEEQLHNID---------------EYDLN  137
_XP_023778876.1_   124  -----------------------------------------PEEQLHNID---------------EYDLN  137
```

```
_NXD42381.1_       124  -----------------------------------------PEEQLHNID---------------EYDLN  137
_NXK69295.1_       124  -----------------------------------------PEEQLHNID---------------EYDLN  137
_XP_049657510.1_   124  -----------------------------------------PEEQLHNID---------------EYDLN  137
_NXV62539.1_       124  -----------------------------------------PEEQLHNID---------------EYDLN  137
_NXB80231.1_       124  -----------------------------------------PEEQLHNID---------------EYDLN  137
_NWU15026.1_       124  -----------------------------------------PEEQLHNID---------------EYDLN  137
_NXL26104.1_       124  -----------------------------------------PEEQLHNID---------------EYDLN  137
_NWW05001.1_       124  -----------------------------------------PEEQLHNID---------------EYDLN  137
_RLW07261.1_       124  -----------------------------------------PEEQLHNID---------------EYDLN  137
_NXR96625.1_       124  -----------------------------------------PEEQLHNID---------------EYDLN  137
_XP_021383204.1_   124  -----------------------------------------PEEQLHNID---------------EYDLN  137
_NXQ25764.1_       124  -----------------------------------------PEEQLHNID---------------EYDLN  137
_NXO08412.1_       124  -----------------------------------------PEEQLHNID---------------EYDLN  137
_NXI32280.1_       124  -----------------------------------------PEEQLHNID---------------EYDLN  137
_NXG00288.1_       124  -----------------------------------------PEEQLHNID---------------EYDLN  137
_XP_041329879.1_   124  -----------------------------------------PEEQLHNID---------------EYDLN  137
_NXV03759.1_       124  -----------------------------------------PEEQLHNID---------------EYDLN  137
_NWT16975.1_       124  -----------------------------------------PEEQLHNID---------------EYDLN  137
_NWT67953.1_       124  -----------------------------------------PEEQLHNID---------------EYDLN  137
_XP_005042765.2_   124  -----------------------------------------PEEQLHNID---------------EYDLN  137
_XP_017600715.1_   124  -----------------------------------------PEEQLHNID---------------EYDLN  137
_NXD93279.1_       124  -----------------------------------------PEEQLHNID---------------EYDLN  137
_NXY02260.1_       124  -----------------------------------------PEEQLHNID---------------EYDLN  137
_NXO84677.1_       124  -----------------------------------------PEEQLHNID---------------EYDLN  137
_NXO64371.1_       124  -----------------------------------------PEEQLHNID---------------EYDLN  137
_NXO42022.1_       124  -----------------------------------------PEEQLHNID---------------EYDLN  137
_NWY65013.1_       124  -----------------------------------------PEEQLHNID---------------EYDLN  137
_NXP90563.1_       124  -----------------------------------------PEEQLHNID---------------EYDLN  137
_NWI73723.1_       124  -----------------------------------------PEEQLHNID---------------EYDLN  137
_NXH30511.1_       124  -----------------------------------------PEEQLHNID---------------EYDLN  137
_NWT77800.1_       124  -----------------------------------------PEEQLHNID---------------EYDLN  137
_NWT34209.1_       124  -----------------------------------------PEEQLHNID---------------EYDLN  137
_NWY29369.1_       124  -----------------------------------------PEEQLHNID---------------EYDLN  137
_NXQ73821.1_       124  -----------------------------------------PEEQLHNID---------------EYDLN  137
_XP_039572786.1_   124  -----------------------------------------PEEQLHNID---------------EYDLN  137
_XP_030802035.1_   124  -----------------------------------------PEEQLHNID---------------EYDLN  137
_NXE68211.1_       124  -----------------------------------------PEEQLHNID---------------EYDLN  137
_NXS80652.1_       124  -----------------------------------------PEEQLHNID---------------EYDLN  137
_NXO32431.1_       124  -----------------------------------------PEEQLHNID---------------EYDLN  137
```

```
_NWR17317.1_      124  -----------------------------------------PEEQLHNID---------------EYDLN  137
_NWZ89732.1_      124  -----------------------------------------PEEQLHNID---------------EYDLN  137
_XP_032039523.1_  124  -----------------------------------------PEEQLHNID---------------EYDLN  137
_KFO04362.1_      124  -----------------------------------------PEEQLHNID---------------EYDLN  137
_XP_054146213.1_  124  -----------------------------------------PEEQLHNID---------------EYDLN  137
_XP_005486916.2_  124  -----------------------------------------PEEQLHNID---------------EYDLN  137
_KAJ7422946.1_    124  -----------------------------------------PEEQLHNID---------------EYDLN  137
_NXC99388.1_      124  -----------------------------------------PEEQLHNID---------------DYDLN  137
_RMC07975.1_      124  -----------------------------------------PEEQLHNID---------------EYDLN  137
_NXT95159.1_       82  -----------------------------------------PEEQLHNID---------------EYDLN   95
_XP_009957589.1_   82  -----------------------------------------PEEQLHNID---------------EYDLN   95
_TRZ13602.1_       82  -----------------------------------------PEEQLHNID---------------EYDLN   95
_NWR45911.1_       73  -----------------------------------------PEEQLHNID---------------EYDLN   86
_KAF4799649.1_     82  -----------------------------------------PEEQLHNID---------------EYDLN   95
_KAJ7420138.1_    115  --------------------------------------EAMPEEQLHNID---------------EYDLN  131
_XP_054678501.1_  124  -----------------------------------------PEEQLHNID---------------EYDLN  137
_NXP48685.1_      124  -----------------------------------------PEEQLHNID---------------EYDLN  137
_XP_010704701.1_   41  -----------------------------------------PEEQLHNID---------------EYDLN   54
_KFV10805.1_       33  -----------------------------------------PEEQLHNID---------------EYDLN   46
_XP_009572567.1_    3  -----------------------------------------PEEQLHNID---------------EYDLN   16
_NXF64277.1_        2  -----------------------------------------PEEQLHNID---------------EYDLN   15
_KFV54465.1_        2  -----------------------------------------PEEQLHNID---------------EYDLN   15
_KFV90717.1_        2  -----------------------------------------PEEQLHNID---------------EYDLN   15
_KFQ03674.1_        2  -----------------------------------------PEEQLHNID---------------EYDLN   15
_NXW01028.1_        2  -----------------------------------------PEEQLHNID---------------EYDLN   15
_KFP32908.1_        2  -----------------------------------------PEEQLHNID---------------EYDLN   15
_KFV93389.1_        2  -----------------------------------------PEEQLHNID---------------EYDLN   15
_NWI63728.1_        2  -----------------------------------------PEEQLHNID---------------EYDLN   15
_KAI6072063.1_      3  -----------------------------------------PEEQLHNID---------------EYDLN   16
_KFQ58901.1_        2  -----------------------------------------PEEQLHNID---------------EYDLN   15
_NXP33753.1_        2  -----------------------------------------PEEQLHNID---------------EYDLN   15
_XP_009476353.1_    1  -----------------------------------------PEEQLHNID---------------EYDLN   14
_KFQ55903.1_        2  -----------------------------------------PEEQLHNID---------------EYDLN   15
_XP_042683349.1_    2  -----------------------------------------PEEQLHNID---------------EYDLN   15
_NXR08991.1_        2  -----------------------------------------PEEQLHNID---------------EYDLN   15
_XP_008947250.1_    2  -----------------------------------------PEEQLHNID---------------EYDLN   15
_KFP72464.1_        2  -----------------------------------------PEEQLHNID---------------EYDLN   15
_KFV60523.1_        2  -----------------------------------------PEEQLHNID---------------EYDLN   15
_NXT13165.1_        2  -----------------------------------------PEEQLHNID---------------EYDLN   15
```

```
_NWT50506.1_        2  -----------------------------------------PEEQLHNID---------------EYDLN   15
_OXB76139.1_        1  ------------------------------------------EEQLHNID---------------EYDLN   13
_XP_050827907.1_   14  ----------------------------------------LPEEQLHNID---------------EYDLN   28
_XP_030302545.1_    9  ----------------------------------------LPEEQLHNID---------------EYDLN   23
_XP_054036038.1_   13  ----------------------------------------LPEEQLHNID---------------EYDLN   27
_XP_012788337.1_  124  -----------------------------------------PEPQLLDIE---------------DCDLK  137
_XP_008070154.1_  113  -----------------------------------------PEKQLIDIE---------------DCDLN  126
_XP_027477445.1_  113  -----------------------------------------PEQQLLDIE---------------DCDLN  126
_XP_025848473.1_  113  -----------------------------------------PEQQLLDIE---------------DCDLN  126
_XP_049640716.1_  124  -----------------------------------------PEPQLLDIE---------------DCDLK  137
_XP_023084089.1_  124  -----------------------------------------PEKQLNDIE---------------DCDLN  137
_EHH22143.1_      124  -----------------------------------------PEKQLNDIE---------------DCDLN  137
_XP_038504711.1_  124  -----------------------------------------PEQQLLDIE---------------DCDLN  137
_XP_038504703.1_  124  -----------------------------------------PEQQLLDIE---------------DCDLN  137
_XP_038504718.1_  124  -----------------------------------------PEQQLLDIE---------------DCDLN  137
_XP_048971856.1_  124  -----------------------------------------PEQQLLDIE---------------DCDLN  137
_KAB1266826.1_    124  -----------------------------------------PEQQLLDIE---------------DCDLN  137
_XP_048391424.1_  124  -----------------------------------------TQEQLLQIE---------------DIDLN  137
_XP_048391425.1_  124  -----------------------------------------TQEQLLQIE---------------DIDLN  137
_GCC28026.1_      124  -----------------------------------------TQEQVLQTE---------------DIDLN  137
_XP_043551991.1_  124  -----------------------------------------TQEQVLQTE---------------DIDLN  137
_XP_043551989.1_  124  -----------------------------------------TQEQVLQTE---------------DIDLN  137
_XP_054534389.1_   34  -----------------------------------------PEKQLNDIE---------------DCDLN   47
_XP_011531312.1_   34  -----------------------------------------PEKQLNDIE---------------DCDLN   47
_XP_021780541.1_   33  -----------------------------------------PEKQLNDIE---------------DCDLN   46
_XP_037862912.1_   54  -----------------------------------------PEKQLNDIE---------------DCDLN   67
_XP_015413428.1_  113  -----------------------------------------PEPQLLDTE---------------DCDLN  126
_XP_005884642.1_  113  -----------------------------------------PEPQLLDTE---------------DCDLN  126
_KAF6105903.1_    113  -----------------------------------------PEPQLLDTE---------------DCDLN  126
_XP_015413426.1_  113  -----------------------------------------PEPQLLDTE---------------DCDLN  126
_XP_014387539.1_  113  -----------------------------------------PEPQLLDTE---------------DCDLN  126
_XP_014444723.1_  113  -----------------------------------------PEQQLLDIE---------------DCDLN  126
_XP_023448058.1_  113  -----------------------------------------PEQELCVIE---------------DCDLN  126
_EGW11604.1_      113  -----------------------------------------PEQQLLDIE---------------DCDLN  126
_XP_015985129.1_  113  -----------------------------------------PEPQLLDTE---------------DCDLN  126
_XP_012878014.1_  113  -----------------------------------------PEQQLLDIE---------------DCDLK  126
_KAG5211506.1_     92  -----------------------------------------PAQQLLDIE---------------DCDLN  105
_MXQ82539.1_       88  -----------------------------------------PEQQLLDIE---------------DCDLN  101
_XP_032729748.1_       ----------------------------------------------------------------------
```

```
_XP_032208113.1_         ----------------------------------------------------------------------
_XP_048208817.1_   124   -----------------------------------------PEQQLLDIE---------------DCDLK  137
_XP_038200333.1_   124   -----------------------------------------PEQQLLDIE---------------DCDLN  137
_P15307.2_         124   -----------------------------------------GEQQLLDIE---------------DCDLN  137
_XP_041524635.1_   113   -----------------------------------------PEQQLLDIE---------------DCDLN  126
_CAA33843.1_       124   -----------------------------------------GEQQLLDIE---------------DCDLN  137
_XP_028732210.2_   113   -----------------------------------------PEQQLLDIE---------------DCDLN  126
_XP_025848474.1_   113   -----------------------------------------PEQQLLDIE---------------DCDLN  126
_EDL97996.1_       113   -----------------------------------------PEQQLLDIE---------------DCDLN  126
_KAI4577886.1_     113   -----------------------------------------PAQQLLDIE---------------DCDLN  126
_KAG8512309.1_     124   -----------------------------------------PEPQLLDIE---------------DCDLN  137
_XP_047637971.1_   124   -----------------------------------------PEQQLLDIE---------------DCDLN  137
_XP_019491454.1_   124   -----------------------------------------PEAQLLDTE---------------DCDLN  137
_XP_043842482.1_   126   -----------------------------------------PEQQLLNIE---------------DCDLN  139
_XP_027721170.1_   126   -----------------------------------------PEQQLLNVE---------------DCDLN  139
_XP_036604632.1_   126   -----------------------------------------PEQQLLNIE---------------DCDLN  139
_XP_020841164.1_   126   -----------------------------------------PEQQLLNIE---------------DCDLN  139
_XP_051831295.1_   126   -----------------------------------------PDQELLNIE---------------DCDLN  139
_XP_031806378.1_   126   -----------------------------------------PEQELLNIE---------------DCDLN  139
_XP_051831294.1_   126   -----------------------------------------PDQELLNIE---------------DCDLN  139
_XP_003339776.1_   126   -----------------------------------------PEQQLLNIE---------------DCDLN  139
_XP_044515413.1_   126   -----------------------------------------PEQQLLNIE---------------DCDLN  139
_XP_028928361.1_   126   -----------------------------------------PEEQLLNIE---------------DCDLN  139
_XP_038607403.1_   126   -----------------------------------------PEEQLLNIE---------------DCDLN  139
_XP_040849334.1_     2   -----------------------------------------PEQQLLDIE---------------DCDLN   15
_TKC46270.1_         1   ------------------------------------------KQELLDIE---------------DCDLN   13
_OWK10634.1_         1   -----------------------------------------PEQQLLDIE---------------DCDLN   14
_XP_036190342.1_     1   ------------------------------------------EPQLLDTE---------------DCDLN   13
_KAB0395596.1_       1   ---------------------------------------------LLDIE---------------DCDLN   10
_KAF6430170.1_       1   -----------------------------------------PEPQLLDTE---------------DCDLN   14
_XP_038948811.1_     2   -----------------------------------------PEQQLLDIE---------------DCDLN   15
_KAB1266828.1_     124   -----------------------------------------PEQQLLDIE---------------DCDLN  137
_NP_002899.1_      124   -----------------------------------------PEKQLNDIE---------------DCDLN  137
_XP_001159537.3_   124   -----------------------------------------PEKQLNDIE---------------DCDLN  137
_PNI66333.1_       124   -----------------------------------------PEKQLNDIE---------------DCDLN  137
_EHH55592.1_       124   -----------------------------------------PEKQLNDIE---------------DCDLN  137
_XP_003262470.1_   124   -----------------------------------------PEKQLNDIE---------------DCDLN  137
_BAG64854.1_       124   -----------------------------------------PEKQLNDIE---------------DCDLN  137
_XP_032034471.1_   124   -----------------------------------------PEKQLNDIE---------------DCDLN  137
```

```
_XP_037862911.1_  124  -----------------------------------------PEKQLNDIE---------------DCDLN  137
_XP_003830911.1_  124  -----------------------------------------PEKQLNDIE---------------DCDLN  137
_XP_004029331.1_  124  -----------------------------------------PEKQLNDIE---------------DCDLN  137
_XP_002812051.1_  124  -----------------------------------------PEKQLNDIE---------------DCDLN  137
_XP_011711623.1_  124  -----------------------------------------PEKQLNDIE---------------DCDLN  137
_XP_033095023.1_  124  -----------------------------------------PEKQLNDIE---------------DCDLN  137
_XP_002799280.1_  124  -----------------------------------------PEKQLNDIE---------------DCDLN  137
_XP_011853172.1_  124  -----------------------------------------PEKQLNDIE---------------DCDLN  137
_XP_050609443.1_  124  -----------------------------------------PEKQLNDIE---------------DCDLN  137
_XP_003908735.1_  124  -----------------------------------------PEKQLNDIE---------------DCDLN  137
_XP_025209693.1_  124  -----------------------------------------PEKQLNDIE---------------DCDLN  137
_XP_023084087.1_  124  -----------------------------------------PEKQLNDIE---------------DCDLN  137
_XP_021780539.1_  124  -----------------------------------------PEKQLNDIE---------------DCDLN  137
_XP_017704568.1_  124  -----------------------------------------PEKQLNDIE---------------DCDLN  137
_XP_010375811.1_  124  -----------------------------------------PEKQLNDIE---------------DCDLN  137
_XP_030776399.1_  124  -----------------------------------------PEKQLNDIE---------------DCDLN  137
_XP_011797211.1_  124  -----------------------------------------PEKQLNDIE---------------DCDLN  137
_XP_012332682.1_  124  -----------------------------------------PEKQLIDIE---------------DCDLN  137
_XP_010331875.2_  124  -----------------------------------------PEKQLIDIE---------------DCDLN  137
_XP_012332683.1_  124  -----------------------------------------PEKQLIDIE---------------DCDLN  137
_XP_017400475.1_  124  -----------------------------------------PEKQLIDIE---------------DCDLN  137
_XP_008568357.1_  124  -----------------------------------------PEPQLLDIE---------------DCDLN  137
_XP_012620284.1_  124  -----------------------------------------PEKQLIDIE---------------DCDLN  137
_XP_002709741.2_  124  -----------------------------------------PEQQLLDIE---------------DCDLN  137
_XP_022452943.1_  124  -----------------------------------------SEQELLDIE---------------DCDLN  137
_XP_045405625.1_  124  -----------------------------------------PEKQLIDIE---------------DCDLN  137
_XP_004280707.1_  124  -----------------------------------------SEQELLDIE---------------DCDLN  137
_XP_030733169.1_  124  -----------------------------------------SEQELLDIE---------------DCDLN  137
_TEA23022.1_      124  -----------------------------------------SEQELLDIE---------------DCDLN  137
_XP_012494629.1_  124  -----------------------------------------PEKQLIDIE---------------DCDLN  137
_XP_010950191.1_  124  -----------------------------------------PEQQLLDIE---------------DCDLN  137
_XP_024608785.1_  124  -----------------------------------------SEQELLDIE---------------DCDLN  137
_XP_006201637.1_  124  -----------------------------------------PEQQLLDIE---------------DCDLN  137
_XP_019285354.1_  124  -----------------------------------------PEQQLLDIE---------------DCDLN  137
_XP_032509224.1_  124  -----------------------------------------SEQELLDIE---------------DCDLN  137
_XP_047379355.1_  124  -----------------------------------------PEQQLLDIE---------------DCDLN  137
_XP_032981134.1_  124  -----------------------------------------PEAQLLDTE---------------DCDLN  137
_XP_005600018.1_  124  -----------------------------------------PEAQLLDIE---------------DCDLN  137
_XP_046517051.1_  124  -----------------------------------------PEAQLLDIE---------------DCDLN  137
```

```
_XP_007464205.1_  124  -----------------------------------------AEQELLDIE---------------DCDLN  137
_XP_019572903.1_  124  -----------------------------------------PEAQLLDTE---------------DCDLN  137
_XP_007189755.1_  124  -----------------------------------------PEQQLLDIE---------------DCDLN  137
_XP_004397771.1_  124  -----------------------------------------PEQQLLDIE---------------DCDLN  137
_XP_004435713.1_  124  -----------------------------------------PESQLLDIE---------------DCDLN  137
_XP_008698124.1_  124  -----------------------------------------PEQQLLDIE---------------DCDLN  137
_XP_023989255.1_  124  -----------------------------------------PEQQLLDIE---------------DCDLN  137
_XP_011233012.2_  124  -----------------------------------------PEQQLLDIE---------------DCDLN  137
_XP_025724444.1_  124  -----------------------------------------PEQQLLDIE---------------DCDLN  137
_XP_005626186.1_  124  -----------------------------------------PEQQLLDIE---------------DCDLN  137
_XP_045643991.1_  124  -----------------------------------------PEQQLLDIE---------------DCDLN  137
_XP_034857383.1_  124  -----------------------------------------PEQQLLDIE---------------DCDLN  137
_XP_027945501.1_  124  -----------------------------------------PEQQLLDIE---------------DCDLN  137
_XP_026341094.1_  124  -----------------------------------------PEQQLLDIE---------------DCDLN  137
_EFB29923.1_      124  -----------------------------------------PEQQLLDIE---------------DCDLN  137
_XP_045740897.1_  124  -----------------------------------------PEQQLLDIE---------------DCDLN  137
_CAD7677454.1_    124  -----------------------------------------PEQQLLDIE---------------DCDLN  137
_XP_035577553.1_  124  -----------------------------------------PEQQLLDIE---------------DCDLN  137
_XP_006733255.1_  124  -----------------------------------------PEQQLLDIE---------------DCDLN  137
_XP_032266820.1_  124  -----------------------------------------PEQQLLDIE---------------DCDLN  137
_XP_041611730.1_  124  -----------------------------------------PEQQLLDIE---------------DCDLN  137
_XP_048971854.1_  124  -----------------------------------------PEQQLLDIE---------------DCDLN  137
_XP_021554840.1_  124  -----------------------------------------PEQQLLDIE---------------DCDLN  137
_VCW78562.1_      124  -----------------------------------------PEQQVFDTE---------------DCDLN  137
_XP_034857381.1_  124  -----------------------------------------PEQQLLDIE---------------DCDLN  137
_XP_008524231.1_  121  -----------------------------------------PEAQLLDIE---------------DCDLN  134
_XP_045740895.1_  124  -----------------------------------------PEQQLLDIE---------------DCDLN  137
_XP_045834746.1_  124  -----------------------------------------PEQQVFDTE---------------DCDLN  137
_XP_035932906.1_  124  -----------------------------------------PEQQLLDIE---------------DCDLN  137
_XP_030883859.1_  124  -----------------------------------------PEQQLLDIE---------------DCDLN  137
_XP_032266818.1_  124  -----------------------------------------PEQQLLDIE---------------DCDLN  137
_XP_024408528.2_  124  -----------------------------------------PEPQLLDTE---------------DCDLN  137
_XP_004580347.1_  124  -----------------------------------------PEQQLLDIE---------------DCDLN  137
_XP_030166525.1_  124  -----------------------------------------PEQQLLDIE---------------DCDLN  137
_XP_035932904.1_  124  -----------------------------------------PEQQLLDIE---------------DCDLN  137
_XP_020040690.1_  124  -----------------------------------------PEQQLLDIE---------------DCDLN  137
_XP_053445604.1_  124  -----------------------------------------PEKQLIDIE---------------DCDLN  137
_XP_049507482.1_  124  -----------------------------------------PEQQLLDIE---------------DCDLN  137
_XP_015335127.1_  124  -----------------------------------------PEQQLLDIE---------------DCDLN  137
```

```
_XP_027790245.1_  124  -----------------------------------------PEQQLLDIE---------------DCDLN  137
_XP_003984077.1_  124  -----------------------------------------PEQQLLDIE---------------DCDLN  137
_XP_039096259.1_  124  -----------------------------------------PEQQLLDIE---------------DCDLN  137
_XP_007075434.1_  124  -----------------------------------------PEQQLLDIE---------------DCDLN  137
_XP_022360130.1_  124  -----------------------------------------PEQQVFDTE---------------DCDLN  137
_XP_047707378.1_  124  -----------------------------------------PEQQLLDIE---------------DCDLN  137
_XP_026243031.1_  124  -----------------------------------------PEQQLLDIE---------------DCDLN  137
_XP_037383340.1_  124  -----------------------------------------PEPQLSDIE---------------DYDLN  137
_XP_005322095.1_  124  -----------------------------------------PEQQLLDIE---------------DCDLN  137
_XP_005662584.1_  124  -----------------------------------------PEQQLLDIE---------------DCDLN  137
_XP_040080101.1_  124  -----------------------------------------PEQQLLDIE---------------DCDLN  137
_XP_017503999.1_  124  -----------------------------------------PEQQLLDIE---------------DCDLN  137
_XP_044119954.1_  124  -----------------------------------------PEQQVFDTE---------------DCDLN  137
_XP_032208110.1_  124  -----------------------------------------PEQQVFDTE---------------DCDLN  137
_XP_012586936.1_  124  -----------------------------------------PEPQLLDIE---------------DCDLN  137
_XP_032729745.1_  124  -----------------------------------------PEQQVFDTE---------------DCDLN  137
_XP_052504199.1_  124  -----------------------------------------PEQQLLDIE---------------DCDLN  137
_XP_054431389.1_  124  -----------------------------------------PEPQLLDTE---------------DCDLN  137
_XP_023576632.1_  124  -----------------------------------------PEQQLLDIE---------------DCDLN  137
_XP_004742070.1_  124  -----------------------------------------PEQQVFDTE---------------DCDLN  137
_XP_037663267.1_  124  -----------------------------------------PEQQLLDIE---------------DCDLN  137
_XP_004005978.1_  124  -----------------------------------------PAQQLLDIE---------------DCDLN  137
_XP_036781908.1_  124  -----------------------------------------PEQQLLDIE---------------DCDLN  137
_XP_040849333.1_  124  -----------------------------------------PEQQLLDIE---------------DCDLN  137
_XP_005686691.1_  124  -----------------------------------------PEQQLLDIE---------------DCDLN  137
_XP_023367900.1_  124  -----------------------------------------PEKQLIDIE---------------DCDLN  137
_KAF0879576.1_    124  -----------------------------------------PEQQLLDIE---------------DCDLN  137
_XP_029793284.1_  124  -----------------------------------------PEQQLLDIE---------------DCDLN  137
_NP_001179899.1_  124  -----------------------------------------PEQQLLDIE---------------DCDLN  137
_XP_020764718.1_  124  -----------------------------------------PEQQLLDIE---------------DCDLN  137
_XP_054431388.1_  124  -----------------------------------------PEPQLLDTE---------------DCDLN  137
_XP_037663265.1_  124  -----------------------------------------PEQQLLDIE---------------DCDLN  137
_XP_019825763.1_  124  -----------------------------------------PEQQLLDIE---------------DCDLN  137
_XP_005889096.1_  124  -----------------------------------------PEQQLLDIE---------------DCDLN  137
_XP_043324571.1_  124  -----------------------------------------PEQQLLDIE---------------DCDLN  137
_KAJ1078272.1_    124  -----------------------------------------PEQQLLDIE---------------DCDLN  137
_XP_036190339.1_  124  -----------------------------------------PEPQLLDTE---------------DCDLN  137
_XP_013005522.1_  124  -----------------------------------------PEQQLLDIE---------------DCDLN  137
_XP_006073936.1_  124  -----------------------------------------PEQQLLDIE---------------DCDLN  137
```

```
_XP_016069529.1_  124  -----------------------------------------PEAQLLDTE---------------DCDLN  137
_KAB0354136.1_    124  -----------------------------------------PEQQLLDIE---------------DCDLN  137
_XP_029424871.1_  124  -----------------------------------------PEQQLLDIE---------------DCDLN  137
_XP_036118792.1_  124  -----------------------------------------PEPQLLDTE---------------DCDLN  137
_XP_037019725.2_  123  -----------------------------------------PEPQLLDTE---------------DCDLN  136
_XP_047602572.1_  124  -----------------------------------------PEQQVFDTE---------------DCDLN  137
_XP_014586639.1_  124  -----------------------------------------PEAQLLDIE---------------DCDLN  137
_XP_046517052.1_  124  -----------------------------------------PEAQLLDIE---------------DCDLN  137
_XP_006909635.2_  124  -----------------------------------------PEPQLLDTE---------------DCDLN  137
_XP_011362222.1_  124  -----------------------------------------PEPQLLDTE---------------DCDLN  137
_XP_045684409.1_  124  -----------------------------------------PEPQLLDTE---------------DCDLN  137
_XP_048208816.1_  124  -----------------------------------------PEQQLLDIE---------------DCDLK  137
_XP_052590629.1_  124  -----------------------------------------PEQQLLDIE---------------DCDLN  137
_XP_032353499.1_  124  -----------------------------------------PEQQLLDIE---------------DCDLN  137
_XP_038200327.1_  124  -----------------------------------------PEQQLLDIE---------------DCDLN  137
_XP_051056550.1_  124  -----------------------------------------PEQQLLDIE---------------DCDLN  137
_XP_012805850.2_  124  -----------------------------------------PEQQLLDIE---------------DCDLN  137
_XP_016050906.1_  124  -----------------------------------------PEPQLLDIE---------------DCDLN  137
_XP_030166526.1_  124  -----------------------------------------PEQQLLDIE---------------DCDLN  137
_XP_036903878.1_  123  -----------------------------------------PEPQLLDTE---------------DCDLN  136
_CAH7004032.1_    124  -----------------------------------------PEQQLLDIE---------------DCDLN  137
_XP_051056549.1_  124  -----------------------------------------PEQQLLDIE---------------DCDLN  137
_XP_042547166.1_  124  -----------------------------------------PEQQLLDIE---------------DCDLK  137
_XP_054584456.1_  124  -----------------------------------------PEPQLLDTE---------------DCDLN  137
_XP_049996768.1_  124  -----------------------------------------PEQQLLDIE---------------DCDLN  137
_XP_027243805.1_  124  -----------------------------------------PEQQLLDIE---------------DCDLN  137
_XP_047707379.1_  124  -----------------------------------------PEQQLLDIE---------------DCDLN  137
_XP_026645370.1_  124  -----------------------------------------PEQQLLDIE---------------DCDLN  137
_XP_014302519.1_  124  -----------------------------------------PEPQLLDTE---------------DCDLN  137
_XP_049726303.1_  124  -----------------------------------------PEQQLLDIE---------------DCDLN  137
_XP_049726302.1_  124  -----------------------------------------PEQQLLDIE---------------DCDLN  137
_XP_012969026.1_  124  -----------------------------------------PEQQLLDIE---------------DCDLN  137
_XP_008524232.1_  121  -----------------------------------------PEAQLLDIE---------------DCDLN  134
_XP_051020904.1_  124  -----------------------------------------PEQQLLDID---------------DCDLN  137
_XP_023590712.1_  124  -----------------------------------------PEPQLLDIE---------------DCDLN  137
_XP_027243803.1_  124  -----------------------------------------PEQQLLDIE---------------DCDLN  137
_XP_040589151.1_  124  -----------------------------------------PEQQLLDIE---------------DCDLN  137
_XP_023412959.1_  124  -----------------------------------------PEQQLLDIE---------------DCDLN  137
_XP_036057111.1_  124  -----------------------------------------PEQQLLDIE---------------DCDLN  137
```

```
_XP_027822602.1_  124  -----------------------------------------PAQQLLDIE---------------DCDLN  137
_XP_031322949.1_  124  -----------------------------------------PEQQLLDIE---------------DCDLN  137
_XP_028634966.1_  124  -----------------------------------------PEQQLLDIE---------------DCDLN  137
_XP_031531529.1_  124  -----------------------------------------PEQQLLDIE---------------DCDLN  137
_XP_006073937.1_  124  -----------------------------------------PEQQLLDIE---------------DCDLN  137
_XP_034364887.1_  124  -----------------------------------------PEQQLLDIE---------------DCDLN  137
_XP_021482246.1_  124  -----------------------------------------PEQQLLDIE---------------DCDLN  137
_KAF4016326.1_    124  -----------------------------------------PEQQLLDIE---------------DCDLN  137
_XP_023602319.1_  124  -----------------------------------------PEPQLLDTE---------------DCDLN  137
_XP_052054164.1_  124  -----------------------------------------PEQQLLDIE---------------DCDLN  137
_XP_036291310.1_  124  -----------------------------------------PEPQLLDTE---------------DCDLN  137
_XP_013370168.1_  124  -----------------------------------------PEQQLLDIE---------------DCDLN  137
_XP_023590722.1_  124  -----------------------------------------PEPQLLDIE---------------DCDLN  137
_XP_048291328.1_  124  -----------------------------------------PEQQLLDID---------------DCDLN  137
_XP_032772910.1_  124  -----------------------------------------PEQQLLDIE---------------DCDLN  137
_XP_008768670.2_  124  -----------------------------------------PEQQLLDIE---------------DCDLN  137
_XP_014302521.1_  124  -----------------------------------------PEPQLLDTE---------------DCDLN  137
_XP_031194882.1_  124  -----------------------------------------PEQQLLDIE---------------DCDLN  137
_NP_033070.2_     124  -----------------------------------------PEQQLLDIE---------------DCDLN  137
_XP_036291312.1_  124  -----------------------------------------PEPQLLDTE---------------DCDLN  137
_XP_021033157.1_  124  -----------------------------------------PEQQLLDIE---------------DCDLN  137
_XP_015849136.1_  124  -----------------------------------------PEQQLLDIE---------------DCDLN  137
_XP_045150027.1_  124  -----------------------------------------PEQQLLDIE---------------DCDLN  137
_XP_021066684.1_  124  -----------------------------------------PEQQLLDIE---------------DCDLN  137
_XP_006881088.1_  124  -----------------------------------------PEQQLLDIE---------------DCDLN  137
_XP_008524234.1_  121  -----------------------------------------PEAQLLDIE---------------DCDLN  134
_XP_021482260.1_  124  -----------------------------------------PEQQLLDIE---------------DCDLN  137
_XP_007125500.1_  124  -----------------------------------------PEQQLLDIE---------------DCDLN  137
_XP_028352731.1_  124  -----------------------------------------PEQQLLDIE---------------DCDLN  137
_XP_028352733.1_  124  -----------------------------------------PEQQLLDIE---------------DCDLN  137
_XP_028352734.1_  124  -----------------------------------------PEQQLLDIE---------------DCDLN  137
_XP_028352736.1_  124  -----------------------------------------PEQQLLDIE---------------DCDLN  137
_XP_028352735.1_  124  -----------------------------------------PEQQLLDIE---------------DCDLN  137
_ERE88482.1_      124  -----------------------------------------PEQQLLDIE---------------DCDLN  137
_KAF3826750.1_      3  -----------------------------------------PEQQLLDIE---------------DCDLN   16
_OBS70420.1_      113  -----------------------------------------PEKQLLDIE---------------DCDLN  126
_XP_006878094.1_   87  -----------------------------------------PEQQLLDIE---------------DCDLN  100
_XP_008978905.3_   81  -----------------------------------------PEKQLIDIE---------------DCDLN   94
_XP_032147941.1_   81  -----------------------------------------PEKQLIDIE---------------DCDLN   94
```

```
_XP_008524233.1_    83  -----------------------------------------PEAQLLDIE---------------DCDLN   96
_XP_044628438.1_    81  -----------------------------------------PEAQLLDIE---------------DCDLN   94
_XP_010608101.1_    81  -----------------------------------------PEQQLLDIE---------------DCDLN   94
_KAF5918007.1_      83  -----------------------------------------PESQLLDIE---------------DCDLN   96
_XP_021101942.1_    81  -----------------------------------------PEQQLLDIE---------------DCDLN   94
_ABM89308.1_         1  ------------------------------------------EKQLNDIE---------------DCDLN   13
_MBW00678.1_            ----------------------------------------------------------------------
_NP_001096177.1_   124  -----------------------------------------REDQLLTIE---------------DYDLN  137
_OCT77375.1_       124  -----------------------------------------REEQLLTIE---------------DYDLN  137
_AAI70187.1_       124  -----------------------------------------REEQLLTIE---------------DYDLN  137
_AAI69362.1_       124  -----------------------------------------REEQLLTIE---------------DYDLN  137
_XP_041418586.1_   124  -----------------------------------------REEQLLTIE---------------DYDLN  137
_XP_041418585.1_   124  -----------------------------------------REEQLLTIE---------------DYDLN  137
_XP_041418587.1_   124  -----------------------------------------REEQLLTIE---------------DYDLN  137
_XP_053316221.1_   124  -----------------------------------------REEQLLTIE---------------DYDLN  137
_XP_053568018.1_   124  -----------------------------------------REEQLLSVE---------------DYDLN  137
_KAG8443975.1_     124  -----------------------------------------REEQLLTIE---------------DYDLN  137
_XP_044145067.1_   126  -----------------------------------------REDQLLTIE---------------DYDLN  139
_KAG9484268.1_     126  -----------------------------------------RDDQLLTIE---------------DYDLN  139
_XP_040285660.1_   126  -----------------------------------------REDQLLTIE---------------DYDLN  139
_XP_040285659.1_   126  -----------------------------------------REDQLLTIE---------------DYDLN  139
_KAG8583825.1_     126  -----------------------------------------REDQLLTIE---------------DYDLN  139
_XP_018411072.1_   126  -----------------------------------------REEQLLTIE---------------DYDLN  139
_XP_040207073.1_   126  -----------------------------------------REEQLLTIE---------------DYDLN  139
_NP_001081750.1_   122  -----------------------------------------REEQLLTIE---------------DYDLN  135
_PIO39092.1_         2  -----------------------------------------REEQLLTIE---------------DYDLN   15
_PIO39091.1_         2  -----------------------------------------REEQLLTIE---------------DYDLN   15
_XP_041035877.1_   123  -----------------------------------------SREELLNET---------------EEDLN  136
_XP_042202691.1_   123  -----------------------------------------SPEVLANEN---------------EYDLN  136
_XP_048475509.1_   112  -----------------------------------------SREELINET---------------EEDLN  125
_XP_048380947.1_   112  -----------------------------------------SREELMNET---------------EEDLN  125
_XP_051901152.1_   116  -----------------------------------------PVEELQNDS---------------EEDLN  129
_BAD60879.1_       111  -----------------------------------------GYFR--NMN---------------NIDLN  122
_XP_050405370.1_   123  -----------------------------------------GFEF--NNV---------------SSEMN  134
_XP_009063713.1_   123  -----------------------------------------GFEF--VNS---------------SAEMS  134
_CAB0044847.1_     139  -----------------------------------------GYEHASETS---------------KIDLG  152
_XP_048259911.1_   125  -----------------------------------------GFNH--GIG---------------QIDLN  136
_ADI72431.1_       125  -----------------------------------------GFNH--GIG---------------QIDLN  136
_CAG5133522.1_     126  -----------------------------------------GFDH--AQS---------------NIDLN  137
```

```
_KAG5688355.1_      36  -----------------------------------------GFDH--ASS---------------NIDLN   47
_AMT84567.1_       124  -----------------------------------------GYFV--NIG---------------NIDLN  135
_XP_052276378.1_   123  -----------------------------------------GFKH--KGQ---------------NIDLN  134
_XP_052276377.1_   123  -----------------------------------------GFKH--KGQ---------------NIDLN  134
_AAK72690.1_       123  -----------------------------------------GFKHLNKIN---------------SIDLN  136
_XP_011414731.2_   123  -----------------------------------------GFKHLNKIN---------------SIDLN  136
_XP_052699807.1_   123  -----------------------------------------GFKHLNKIN---------------SIDLN  136
_NP_001292261.1_   123  -----------------------------------------GFKHLNKIN---------------SIDLN  136
_XP_048738054.1_   123  -----------------------------------------GFKHMNKIN---------------SIDLN  136
_XP_052699808.1_   123  -----------------------------------------GFKHLNKIN---------------SIDLN  136
_XP_022332457.1_   123  -----------------------------------------GFKHLNKIN---------------SIDLN  136
_ABL63469.1_       122  -----------------------------------------GFKHMNKAN---------------NIDLN  135
_XP_033733822.1_   123  -----------------------------------------GFKHMTKAG---------------NIDLN  136
_XP_021347355.1_   123  -----------------------------------------GFKHIPKAA---------------NMDLN  136
_UOA58607.1_       123  -----------------------------------------GFKHMTKAA---------------NMDLN  136
_XP_021347356.1_   123  -----------------------------------------GFKHIPKAA---------------NMDLN  136
_CAG2186872.1_     122  -----------------------------------------GFKHMQKAN---------------NIDLN  135
_XP_052058915.1_   122  -----------------------------------------GFKHMQKAN---------------NIDLN  135
_KAJ8309232.1_     123  -----------------------------------------GFKHMAKVN---------------NIDLN  136
_UZH84475.1_       123  -----------------------------------------GFKH--KST---------------NLDLN  134
_ALA99939.1_       123  -----------------------------------------GFKH--KSQ---------------NIDLN  134
_XP_052767764.1_   123  -----------------------------------------GFKH--RGQ---------------NIDLN  134
_ADD25211.1_       123  -----------------------------------------GFKHMTKAA---------------NMDLN  136
_XP_052224245.1_   123  -----------------------------------------GFKH--KGQ---------------NIDLN  134
_KAJ8314234.1_          ----------------------------------------------------------------------
_VDH95719.1_        81  -----------------------------------------GFKHMQKAN---------------NIDLN   94
_XP_041352913.1_   125  -----------------------------------------GFSH--GSG---------------NIDLN  136
_XP_046555158.1_   125  -----------------------------------------GFNH--GIG---------------QIDLN  136
_AAW33559.1_       125  -----------------------------------------GFNH--GIG---------------QIDLN  136
_ABY87435.1_       112  -----------------------------------------GFNH--GIG---------------QIDLN  123
_XP_009028834.1_   125  -----------------------------------------GYPS--ANN---------------NIDLN  136
_XP_025106109.1_   125  -----------------------------------------GFDH--ASS---------------NIDLN  136
_NP_001298191.1_   126  -----------------------------------------GFDH--AQS---------------NIDLN  137
_KAH9519545.1_     126  -----------------------------------------GFDH--AQS---------------NIDLN  137
_RUS74561.1_       126  -----------------------------------------GFEH--AQS---------------NIDLN  137
_GFO11973.1_       126  -----------------------------------------GFDH--AQS---------------NIDLN  137
_CAG5132534.1_     126  -----------------------------------------GFEH--IQS---------------NIDLN  137
_GFR69641.1_       126  -----------------------------------------GFEH--AQS---------------NIDLN  137
_XP_035824873.1_   126  -----------------------------------------GFDH--SQS---------------NIDLN  137
```

```
_AAY27981.1_      123  -----------------------------------------GFAH-AKDM---------------NIDFS  135
_XP_052827753.1_  122  -----------------------------------------GFDH-AHNL---------------NIDFS  134
_XP_029642523.1_  122  -----------------------------------------GFDH-AHNL---------------NIDFS  134
_CAE1288013.1_     82  -----------------------------------------GFSH-AKDM---------------NIDFS   94
_ELU04547.1_      124  -----------------------------------------GYFN--NVG---------------SIDLN  135
_KAI0227899.1_    124  -----------------------------------------GYSDK-------------------DHDLT  133
_XP_044002103.1_  129  -----------------------------------------GFSH--ANR---------------SYALN  140
_XP_044002102.1_  128  -----------------------------------------GFSH--KNQ---------------PIELN  139
_XP_044002099.1_  128  -----------------------------------------GFSH--KNQ---------------PIELN  139
_XP_037026629.1_  127  -----------------------------------------GFGH-KELK---------------SINLH  139
_CAD7632570.1_    126  -----------------------------------------GFDHIMKSS--------------RSLDLG  140
_CAD7650738.1_    126  -----------------------------------------GFDHIMKSS--------------RSLDLG  140
_XP_054163335.1_  124  -----------------------------------------GFDHMNCSS--------------FEINLS  138
_CAD7629985.1_    131  -----------------------------------------GFDHMNCSS--------------FEINLS  145
_CAD7652069.1_    124  -----------------------------------------GFDHMNCSS--------------FEINLS  138
_XP_017954168.1_  128  -----------------------------------------KFDHKDNIS---------------SINLY  141
_XP_017858583.1_  128  -----------------------------------------KFDHKDNIS---------------SINLY  141
_XP_002001588.1_  128  -----------------------------------------KFDHKDNIS---------------SINLY  141
_XP_017954163.1_  128  -----------------------------------------KFDHKDNIS---------------SINLY  141
_XP_023164563.2_  128  -----------------------------------------KFDHKDNIS---------------SINLY  141
_TDG46702.1_      128  -----------------------------------------KFDHKDNIS---------------SINLY  141
_XP_030564898.1_  128  -----------------------------------------KFDHKDNIS---------------SINLY  141
_XP_015024496.1_  128  -----------------------------------------KFDHKDNIS---------------SINLY  141
_XP_034475238.1_  129  -----------------------------------------GFNHLEDLT---------------RLNLY  142
_XP_034475235.1_  129  -----------------------------------------GFNHLEDLT---------------RLNLY  142
_XP_017854415.1_  128  -----------------------------------------KFDHKDNIN---------------GINLY  141
_XP_017854416.1_  128  -----------------------------------------KFDHKDNIN---------------GINLY  141
_ALC40089.1_      128  -----------------------------------------KFDHKDNIN---------------GINLY  141
_XP_030382983.1_  129  -----------------------------------------KFDHKNDIA---------------SINMY  142
_XP_030382981.1_  129  -----------------------------------------KFDHKNDIA---------------SINMY  142
_XP_001989397.1_  128  -----------------------------------------KFDHKDNIS---------------GINLY  141
_XP_002067135.2_  129  -----------------------------------------KFDHKDNIG---------------SINLY  142
_XP_034475239.1_  127  -----------------------------------------GFDHLNNLA---------------LINLY  140
_KAH8411901.1_    128  -----------------------------------------RFDHMDDVR---------------SIKLN  141
_XP_016963143.1_  129  -----------------------------------------KFDHKDQVD---------------KINRY  142
_XP_037712361.1_  129  -----------------------------------------KFDHKDQVD---------------KINRY  142
_XP_016993627.2_  129  -----------------------------------------KFDHKDQID---------------KINRY  142
_XP_016963141.1_  129  -----------------------------------------KFDHKDQVD---------------KINRY  142
_XP_016993624.2_  129  -----------------------------------------KFDHKDQID---------------KINRY  142
```

```
_XP_037712358.1_  129  -----------------------------------------KFDHKDQVD---------------KINRY  142
_XP_001974449.1_  129  -----------------------------------------KFDHKDQVD---------------KINRY  142
_AAM48404.1_      129  -----------------------------------------KFDHKDQID---------------KINRY  142
_NP_001162999.1_  129  -----------------------------------------KFDHKDQID---------------KINRY  142
_XP_026835878.1_  129  -----------------------------------------KFDHKDQVD---------------KINRY  142
_AAA28465.1_      129  -----------------------------------------KFDHKDQID---------------KINRY  142
_XP_002038912.1_  129  -----------------------------------------KFDHKDQID---------------KINRY  142
_XP_033169558.1_  129  -----------------------------------------KFDHKDQID---------------KINRY  142
_EDX05310.1_      129  -----------------------------------------KFDHKDQID---------------KINRY  142
_NP_001162998.1_  129  -----------------------------------------KFDHKDQID---------------KINRY  142
_XP_032579766.1_  129  -----------------------------------------KFDHKDQID---------------KINRY  142
_XP_033169541.1_  129  -----------------------------------------KFDHKDQID---------------KINRY  142
_XP_034669502.1_  129  -----------------------------------------KFDHKDQID---------------KINRY  142
_XP_034125957.1_  129  -----------------------------------------KFDHKDQID---------------KINRY  142
_XP_034669501.1_  129  -----------------------------------------KFDHKDQID---------------KINRY  142
_XP_034125956.1_  129  -----------------------------------------KFDHKDQID---------------KINRY  142
_XP_034125955.1_  129  -----------------------------------------KFDHKDQID---------------KINRY  142
_XP_017152033.1_  129  -----------------------------------------KFDHKDQID---------------KINRY  142
_XP_017152031.1_  129  -----------------------------------------KFDHKDQID---------------KINRY  142
_XP_002014799.1_  129  -----------------------------------------KFDHKDQID---------------KINRY  142
_XP_026844902.1_  129  -----------------------------------------KFDHKDQID---------------KINRY  142
_XP_015035983.2_  129  -----------------------------------------KFDHKDQID---------------KINRY  142
_XP_017063896.1_  129  -----------------------------------------KFDHKDQID---------------KINRY  142
_XP_016935332.1_  129  -----------------------------------------KFDHKDQVD---------------KINRY  142
_XP_017063894.2_  129  -----------------------------------------KFDHKDQID---------------KINRY  142
_XP_036668637.1_  129  -----------------------------------------KFDHKDQVD---------------KINRY  142
_XP_016935310.1_  129  -----------------------------------------KFDHKDQVD---------------KINRY  142
_KAH8298318.1_    129  -----------------------------------------KFDHKDQID---------------KINRY  142
_KAH8412187.1_    129  -----------------------------------------KFDHKDQID---------------KINRY  142
_KAH8278042.1_    128  -----------------------------------------KFDHKDQID---------------KINRY  141
_KAH8366423.1_    129  -----------------------------------------KFDHKDQID---------------KINRY  142
_XP_017044860.1_  128  -----------------------------------------KFDHKDQVD---------------KINRY  141
_KAH8324526.1_    127  -----------------------------------------KFDHKDQID---------------KINRY  140
_XP_017116817.1_  129  -----------------------------------------KFDHKDQID---------------KINRY  142
_XP_041566118.1_  129  -----------------------------------------KFDHKDQID---------------KINRY  142
_XP_052852584.1_  129  -----------------------------------------KFDHKDQID---------------KINRY  142
_XP_052852582.1_  129  -----------------------------------------KFDHKDQID---------------KINRY  142
_XP_017107132.2_  128  -----------------------------------------KFDHKDQID---------------KINRY  141
_XP_043069650.1_  128  -----------------------------------------KFDHKDQID---------------KINRY  141
```

```
_KAH8283158.1_     129  -----------------------------------------KFDHKDQID---------------KINRY  142
_XP_017025640.1_   128  -----------------------------------------KFDHKDQID---------------KINRY  141
_XP_020803071.1_   129  -----------------------------------------KFDHKDQID---------------KINRY  142
_XP_017025638.1_   129  -----------------------------------------KFDHKDQID---------------KINRY  142
_KAH8308067.1_     129  -----------------------------------------KFDHKDQID---------------KINRY  142
_KAH8362606.1_     129  -----------------------------------------KFDHKDQID---------------KINRY  142
_XP_020803068.1_   129  -----------------------------------------KFDHKDQID---------------KINRY  142
_XP_015052364.1_   128  -----------------------------------------KFDHMDHVD---------------KINRY  141
_XP_002090469.1_   128  -----------------------------------------KFDHMDHVD---------------KINRY  141
_XP_039480354.1_   128  -----------------------------------------KFDHMDHVD---------------KINRY  141
_XP_039480352.1_   128  -----------------------------------------KFDHMDHVD---------------KINRY  141
_XP_041450795.1_   129  -----------------------------------------KFDHKDQVD---------------KINRY  142
_XP_044315461.1_   129  -----------------------------------------KFDHKDQVD---------------KINRY  142
_XP_044315463.1_   129  -----------------------------------------KFDHKDQVD---------------KINRY  142
_XP_001961710.2_   129  -----------------------------------------KFDHKDQID---------------KINRY  142
_XP_014761890.1_   129  -----------------------------------------KFDHKDQID---------------KINRY  142
_KAH8323309.1_     129  -----------------------------------------KFDHKDQID---------------KINRY  142
_XP_043646206.1_   128  -----------------------------------------KFDHMKYTE---------------KINRY  141
_XP_043646204.1_   128  -----------------------------------------KFDHMKYTE---------------KINRY  141
_AAQ65066.1_       129  -----------------------------------------KFDHKDQID---------------KINRY  142
_AAQ64900.1_       129  -----------------------------------------KFDHKDQID---------------KINRY  142
_AAQ64905.1_       129  -----------------------------------------KFDHKDQID---------------KINRY  142
_KAH8378209.1_     128  -----------------------------------------GFEHVKDVS---------------SINLS  141
_KAH8293207.1_     128  -----------------------------------------GFEHGRNVN---------------TIDLT  141
_KAH8391978.1_     127  -----------------------------------------GFEHAKDVS---------------SINLS  140
_XP_034101352.1_   127  -----------------------------------------GFEHAKDVS---------------SINLS  140
_KAJ3658269.1_     124  -----------------------------------------GFDHKEDVR---------------SLDLN  137
_KAJ3658270.1_     124  -----------------------------------------GFGHKDDFG---------------PLERN  137
_KAJ3658271.1_     124  -----------------------------------------GFQHNEDVK---------------SLDLT  137
_XP_015117085.1_   126  -----------------------------------------GYDHGDHPT---------------SIDLN  139
_XP_011307643.1_   126  -----------------------------------------GYDHGDHPV---------------LIDLN  139
_XP_011307741.1_   124  -----------------------------------------KGYDDTRIT---------------ADDLN  137
_XP_015119445.1_   126  -----------------------------------------GYDHSEDPA---------------SIDLN  139
_XP_052128594.1_   121  S-----------------------------------HVAGFDHCKQLDLE---------------SIDLD  140
_XP_052128597.1_   116  --------------------------------------TGFDHCKQLDLE---------------SIDLD  132
_KAE8741315.1_      72  ----------------------------------------FDHCKQLDLE---------------SIDLD   86
_XP_028982160.1_   131  -----------------------------------------SGWEDSDIS---------------AIDFN  144
_XP_011307645.1_   131  -----------------------------------------PGWEDSDIG---------------AIDLN  144
_XP_054725384.1_   122  -----------------------------------------GFSHRLSPH---------------CIDLN  135
```

```
_XP_053965385.1_  122  -----------------------------------------GFSHRLSPH---------------CIDLN  135
_XP_013149158.1_  133  -----------------------------------------GFDYGKDLK---------------NIDLN  146
_XP_045534655.1_  133  -----------------------------------------GFDYGREPK---------------RIDLN  146
_KPJ06852.1_      133  -----------------------------------------GFDYGREPK---------------RIDLN  146
_XP_013176418.1_  133  -----------------------------------------GFDYCREPK---------------SIDLN  146
_OXU28425.1_      122  E----------------------------------QRFLQTGFAHKDSPK---------------DICLD  142
_XP_008216913.1_  119  ---------------------------------------AAGFAHKDSPE---------------DISLN  134
_XP_008216914.1_  116  ----------------------------------------TGFAHKDSPE---------------DISLN  130
_XP_040572301.1_  126  -----------------------------------------KFNH--THS---------------SVNPY  137
_CAB4068408.1_         ----------------------------------------------------------------------
_KAF7993284.1_     88  -----------------------------------------GFEHKNSPN---------------KIDLN  101
_KAF7987489.1_    131  -----------------------------------------GFDHKNSLS---------------TIDVT  144
_KAF7994520.1_    133  -----------------------------------------GFKHKNSPN---------------LIDMK  146
_XP_044019368.1_  135  -----------------------------------------GFEHKDLSS--------------KSIDPS  149
_XP_044019332.1_   37  -----------------------------------------GFDHKNSLS---------------TIDVT   50
_XP_044003986.1_    6  -----------------------------------------GFKHKNSPN---------------LIDMK   19
_XP_037042732.1_  127  -----------------------------------------SKCGVDNL----------------RRDIH  139
_CAD7223243.1_    126  -----------------------------------------GFDHADGLV---------------RVDQN  139
_XP_044732233.1_  134  -----------------------------------------GFG--HQIT---------------SADLH  145
_XP_012231885.1_  123  -----------------------------------------GFKHPN------------------NIDLS  133
_XP_022814699.1   132  -----------------------------------------GFRHE-LTK---------------DFTLT  144
_CAB3516523.1_    132  -----------------------------------------GFRHE-LTK---------------DFTLT  144
_XP_035458245.2_  131  -----------------------------------------GFKHE-LTK---------------DFRLT  143
_KAF9795795.1_    120  -----------------------------------------GFKHE-LTK---------------DFRLT  132
_XP_014233257.1_  123  -----------------------------------------NYEHLEELDQQRVVSSTSTDSGKQSIDLT  151
_XP_034324400.1_  108  -----------------------------------------YSEDMKKAG---------------YWNMN  121
_XP_052699907.1_  108  -----------------------------------------YSEDMKKAG---------------YWNIN  121
Consensus aa:          .........................................sb.p..p.p...............phsls
Consensus ss:                                                    hhhhhhh


Conservation:            56 5                                         6  6
_XP_001652840.2_  139  -AIRLCFQVFLEGQQR----------GRFT------EPLQ----PVVSDVIYD------------KKA--  173
_AAW67214.1_      139  -AIRLCFQVFLEGQQR----------GRFT------EPLQ----PVVSDVIYD------------KKA--  173
_XP_021704027.1_  139  -AIRLCFQVFLEGQQR----------GRFT------EPLQ----PVVSDVIYD------------KKA--  173
_XP_019540833.2_  139  -AIRLCFQVFLEGQQR----------GRFT------EPLQ----PVVSDVIYD------------KKA--  173
_XP_029720231.1_  139  -AIRLCFQVFLEGQQR----------GRFT------EPLQ----PVVSDVIYD------------KKA--  173
_AAW67215.1_      139  -AIRLCFQVFLEGQQR----------GRFT------EPLQ----PVVSDVIYD------------KKA--  173
_XP_021704025.1_  139  -AIRLCFQVFLEGQQR----------GRFT------EPLQ----PVVSDVIYD------------KKA--  173
```

```
_XP_019540831.2_  139  -AIRLCFQVFLEGQQR----------GRFT------EPLQ----PVVSDVIYD------------KKA--  173
_KXJ81105.1_      139  -AIRLCFQVFLEGQQR----------GRFT------EPLQ----PVVSDVIYD------------KKA--  173
_KXJ76497.1_      139  -AIRLCFQVFLEGQQR----------GRFT------EPLQ----PVVSDVIYD------------KKA--  173
_XP_023240428.1_  139  -SVRLCFQAFIKGPD-----------DKYT------VSLT----PVVSDIIYD------------KKA--  172
_CAD7241875.1_    140  -CVRLCFQVFLESEK-----------NKYP------IQLE----PVVSDPIYG------------NKS--  173
_TRY80291.1_      138  -MVRLCYQVFLKMPDR------------SL------VPLK----PVVSAIIKD------------RKA--  170
_ALF84899.1_      136  -AIRLCFQVFLKVEPN----------L---------VPLE----PVVSEVIRD------------KKA--  167
_KAF4528208.1_    138  -AVRLAFQVFLENPNE---------PGKFK------RPLP----PIVSDPIYD------------KKA--  173
_XP_054260955.1_  139  -AVRLCFQAFLEDRPN---------SGTFT------IPLV----PVVSEIIYD------------KKA--  174
_XP_054264401.1_  139  -AVRLCFQAFLEDRPN---------SGTFT------IPLV----PVVSEIIYD------------KKA--  174
_XP_054260954.1_  139  -AVRLCFQAFLEDRPN---------SGTFT------IPLV----PVVSEIIYD------------KKA--  174
_XP_038114042.1_  139  -AVRLCFQVFLEGQQR----------GRFT------EPLQ----PVVSDVIYD------------KKA--  173
_XP_039437558.1_  139  -AVRLCFQVFLEGQQR----------GRFT------EPLQ----PVVSDVIYD------------KKA--  173
_XP_001844078.2_  139  -AVRLCFQVFLEGQQR----------GRFT------EPLQ----PVVSDVIYD------------KKA--  173
_EDS35747.1_      139  -AVRLCFQVFLEGQQR----------GRFT------EPLQ----PVVSDVIYD------------KKA--  173
_XP_053696925.1_  139  -AVRLCFQVFLEGQQR----------GRFT------EPLQ----PVVSDIIYD------------KKA--  173
_XP_001869619.2_  139  -AVRLCFQVFIEGQQP----------GRFS------EPLQ----PVVSDVIYD------------KKA--  173
_XP_039434954.1_  139  -AVRLCFQVFIEGQQP----------GRFS------EPLQ----PVVSDVIYD------------KKA--  173
_EDS29879.1_      139  -AVRLCFQVFIEGQQP----------GRFS------EPLQ----PVVSDVIYD------------KKA--  173
_XP_019550742.2_  139  -AVRLCFQAFLEGQQR----------GHFT------NPLV----PVVSDIIYD------------KKA--  173
_KXJ74587.1_      139  -AVRLCFQAFLEGQQR----------GHFT------NPLV----PVVSDIIYD------------KKA--  173
_XP_035908773.1_  140  -AVRLCFQVFLEGQQR----------GRFT------EPLT----PVVSDIIYD------------KKA--  174
_AJC98655.1_      140  -AVRLCFQVFLEGQQR----------GRFT------EPLT----PVVSDIIYD------------KKA--  174
_AJC98650.1_      140  -AVRLCFQVFLEGQQR----------GRFT------EPLT----PVVSDIIYD------------KKA--  174
_AJC98644.1_      140  -AVRLCFQVFLEGQQR----------GRFT------EPLT----PVVSDIIYD------------KKA--  174
_AJC98657.1_      140  -AVRLCFQVFLEGQQR----------GRFT------EPLT----PVVSDIIYD------------KKA--  174
_AJC98648.1_      140  -AVRLCFQVFLEGQQR----------GRFT------EPLT----PVVSDIIYD------------KKA--  174
_AJC98647.1_      140  -AVRLCFQVFLEGQQR----------GRFT------EPLT----PVVSDIIYD------------KKA--  174
_AJC98651.1_      140  -AVRLCFQVFLEGQQR----------GRFT------EPLT----PVVSDIIYD------------KKA--  174
_AJC98643.1_      140  -AVRLCFQVFLEGQQR----------GRFT------EPLT----PVVSDIIYD------------KKA--  174
_AJC98656.1_      140  -AVRLCFQVFLEGQQR----------GRFT------EPLT----PVVSDIIYD------------KKA--  174
_AJC98641.1_      140  -AVRLCFQVFLEGQQR----------GRFT------EPLT----PVVSDIIYD------------KKA--  174
_AJC98640.1_      140  -AVRLCFQVFLEGQQR----------GRFT------EPLT----PVVSDIIYD------------KKA--  174
_AJC98653.1_      140  -AVRLCFQVFLEGQQR----------GRFT------EPLT----PVVSDIIYD------------KKA--  174
_AJC98654.1_      140  -AVRLCFQVFLEGQQR----------GRFT------EPLT----PVVSDIIYD------------KKA--  174
_AJC98652.1_      140  -AVRLCFQVFLEGQQR----------GRFT------EPLT----PVVSDIIYD------------KKA--  174
_AJC98646.1_      140  -AVRLCFQVFLEGQQR----------GRFT------EPLT----PVVSDIIYD------------KKA--  174
_AJC98658.1_      140  -AVRLCFQVFLEGQQR----------GRFT------EPLT----PVVSDIIYD------------KKA--  174
_AJC98639.1_      140  -AVRLCFQVFLEGQQR----------GRFT------EPLT----PVVSDIIYD------------KKA--  174
```

```
_XP_035908772.1_   140   -AVRLCFQVFLEGQQR----------GRFT------EPLT----PVVSDIIYD------------KKA--   174
_XP_050076803.1_   140   -AVRLCFQVFLEGQQR----------GRFT------EPLT----PVVSDIIYD------------KKA--   174
_XP_049282441.1_   140   -AVRLCFQVFLEGQQR----------GRFT------EPLA----PVVSDIIYD------------KKA--   174
_XP_040170080.1_   140   -AVRLCFQVFLEGQQR----------GRFT------EPLT----PVVSDIIYD------------KKA--   174
_XP_050076802.1_   140   -AVRLCFQVFLEGQQR----------GRFT------EPLT----PVVSDIIYD------------KKA--   174
_XP_041780138.1_   140   -AVRLCFQVFLEGQQR----------GRFT------EPLT----PVVSDIIYD------------KKA--   174
_XP_040239229.2_   140   -AVRLCFQVFLEGQQR----------GRFT------EPLT----PVVSDIIYD------------KKA--   174
_XP_040239222.2_   140   -AVRLCFQVFLEGQQR----------GRFT------EPLT----PVVSDIIYD------------KKA--   174
_XP_035908780.1_   140   -AVRLCFQVFLEGQQR----------GRFT------EPLT----PVVSDIIYD------------KKA--   174
_XP_040239221.2_   140   -AVRLCFQVFLEGQQR----------GRFT------EPLT----PVVSDIIYD------------KKA--   174
_XP_052897786.1_   140   -AVRLCFQVFLEGQQR----------GRFT------EPLT----PVVSDIIYD------------KKA--   174
_XP_052895493.1_   140   -AVRLCFQVFLEGQQR----------ERFM------EPLT----PVVSDIIYD------------KKA--   174
_XP_053664395.1_   140   -AVRLCFQVFLEGQQR----------GRFT------EPLT----PVVSDIIYD------------KKA--   174
_XP_050076801.1_   140   -AVRLCFQVFLEGQQR----------GRFT------EPLT----PVVSDIIYD------------KKA--   174
_XP_310177.3_      140   -AVRLCFQVFLEGQQR----------GRFT------EPLT----PVVSDIIYD------------KKA--   174
_XP_001652339.1_   139   -AVRLCFQAFLEGPQQ----------GHFT------NPLV----PVVSDIIYD------------KKA--   173
_XP_053686256.1_   139   -AVRLCFQVYLPGGPD----------KKFT------QPLQ----PVVSDIIYD------------KKA--   173
_XP_053675540.1_   140   -AVRLCFQVFLEGQQR----------GRFT------EPLT----PVVSDIIYD------------KKA--   174
_ETN66814.1_       139   -AVRLCFQVFLEGKTT----------GRFT------EPLT----PVVSDVIYD------------KKA--   173
_XP_049534501.1_   139   -AVRLCFQVFLEGKTT----------GRFT------EPLT----PVVSDVIYD------------KKA--   173
_XP_050084431.1_   139   -AVRLCFQVFLEGKTT----------GRFT------EPLT----PVVSDVIYD------------KKA--   173
_XP_035782874.1_   139   -AVRLCFQVFLEGKTT----------GRFT------EPLT----PVVSDVIYD------------KKA--   173
_XP_035782869.1_   139   -AVRLCFQVFLEGKTT----------GRFT------EPLT----PVVSDVIYD------------KKA--   173
_KFB39849.1_       147   -AVRLCFQVFLEGPTK----------GRFT------EPLT----PVVSDVIYD------------KKA--   181
_XP_052871375.1_   140   -AVRLCFQVFLEGQQR----------GRFT------EPLA----PVVSDIIYD------------KKA--   174
_AAR13743.1_       125   -AVRLCFQVFLEGQQR----------GRFT------EPLT----PVVSDIIYD------------KKA--   159
_AAR13744.1_       125   -AVRLCFQVFLEGQQR----------GRFT------EPLT----PVVSDIIYD------------KKA--   159
_CAA65157.1_       119   -AVRLCFQVFLEGQQR----------GRFT------EPLT----PVVSDIIYD------------KKA--   153
_XP_022170156.1_   139   -AVRLCFQAFLGGTQ-----------KGKY------SILE----PIVSDPIYD------------KKA--   172
_XP_025205168.1_   139   -AVRLCFQAFLGGTQ-----------KGKY------SILE----PIVSDPIYD------------KKA--   172
_XP_026817162.1_   139   -AVRLCFQAFLGGTQ-----------KGKY------SILE----PIVSDPIYD------------KKA--   172
_XP_027846205.1_   139   -AVRLCFQAFLGGTQ-----------KGKY------SILE----PIVSDPIYD------------KKA--   172
_XP_015370635.1_   139   -AVRLCFQAFLGGTQ-----------KGKY------SILE----PIVSDPIYD------------KKA--   172
_CAH1723797.1_     139   -AVRLCFQAFLGGTQ-----------KGKY------SILE----PIVSDPIYD------------KKA--   172
_KAF0769940.1_     139   -AVRLCFQAFLGGTQ-----------KGKY------SILE----PIVSDPIYD------------KKA--   172
_CAB4065202.1_      90   -AVRLCFQAFLKK-------------GESL------TPLP----AVVSNTVVD------------RKV--   121
_XP_050440903.1_   139   -AVRLCFQAFLGGSQ-----------KGKY------SILE----PVVSDPIYD------------KKA--   172
_XP_050533799.1_   139   -AVRLCFQAFLGGSQ-----------KGKY------SILE----PVVSDPIYD------------KKA--   172
_XP_025410632.1_   139   -AVKLCFQAFLEGSQ-----------KGEY------SILK----PIVSETIYD------------KKA--   172
```

```
_XP_008180746.1_   139   -AVRLCFQAFLGGTQ-----------KGKY------SILE----PIVSDPIYD------------KKA--   172
_XP_026465450.1_   140   -AVRLCFQVILEGDA-----------HGKF-----TIPLR----PVVSEPIYD------------KKS--   174
_VVC36026.1_       139   -AVRLCFQAFLGGAQ-----------KGKY------SILE----PVVSDPIYD------------KKA--   172
_XP_016661522.1_   139   -AVRLCFQAYLGGTQ-----------KRKY------CILE----PIVSDPIYD------------KKA--   172
_XP_001947429.3_   139   -AVRLCFQAYLGGTQ-----------KRKY------CILE----PIVSDPIYD------------KKA--   172
_XP_026466007.1_   140   -AVRLCFQVILEGDA-----------HGKF-----TIPLR----PVVSEPIYD------------KKS--   174
_KRT81254.1_       136   -CVRLCFQAFIPGEK-----------PDTY------KTLL----PVVSKPIYD------------KKA--   169
_XP_033224832.1_   142   -KVRLCFQGFILKKL-----------EDGTT---GCIPLD----PVVSEPILE------------RKA--   178
_XP_025407590.1_   133   -TVKLCFQAILMGSQ-----------EGQY------YQLE----PIVSDNIPN------------KK---   165
_XP_045476729.1_   135   -SVRLCFQVFIKGED-----------GKCR------VPLD----PVVSEPIYD------------RKH--   168
_XP_034174253.1_   139   -AVRLCFQVFLEGSQK----------GKFN------IQLP----PVVSDPIFD------------KKA--   173
_XP_029047866.1_   139   -AVRLCFQVFLEGSQK----------GKFN------IQLP----PVVSDPIFD------------KKA--   173
_XP_015430648.1_   139   -AVRLCFQVFLEGSQK----------GKFN------LPLP----PVVSDPIFD------------KKA--   173
_XP_017798081.1_   139   -AVRLCFQVFLDGSQK----------GKFN------VALP----PVVSDPIFD------------KKA--   173
_XP_017756471.1_   139   -AVRLCFQVFLEGSQK----------GKFN------VPLP----PVVSDPIFD------------KKA--   173
_XP_043248447.1_   139   -AVRLCFQVFLQGSQK----------AKFN------VPLP----PVVSDPIFD------------KKA--   173
_XP_029047855.1_   139   -AVRLCFQVFLEGSQK----------GKFN------IQLP----PVVSDPIFD------------KKA--   173
_XP_034174252.1_   139   -AVRLCFQVFLEGSQK----------GKFN------IQLP----PVVSDPIFD------------KKA--   173
_XP_031829337.1_   139   -AVRLCFQVFLEGSQK----------GKFN------LPLP----PVVSDPIFD------------KKA--   173
_KZC09125.1_       139   -AVRLCFQVFLEGSQK----------GKFN------LPLP----PVVSDPIFD------------KKA--   173
_XP_033327213.1_   139   -AVRLCFQVFLEGSQK----------GKFN------VALP----AVVSDPIFD------------KKA--   173
_XP_033327212.1_   139   -AVRLCFQVFLEGSQK----------GKFN------VALP----AVVSDPIFD------------KKA--   173
_XP_017756445.1_   139   -AVRLCFQVFLEGSQK----------GKFN------VPLP----PVVSDPIFD------------KKA--   173
_OAD62393.1_       139   -AVRLCFQVFLEGSQK----------GKFN------VPLP----PVVSDPIFD------------KKA--   173
_XP_043248413.1_   139   -AVRLCFQVFLQGSQK----------AKFN------VPLP----PVVSDPIFD------------KKA--   173
_XP_033327208.1_   139   -AVRLCFQVFLEGSQK----------GKFN------VALP----AVVSDPIFD------------KKA--   173
_XP_034950477.1_   139   -SVRLCFQVFLEGTEK----------GKFT------VPLS----PVVSDPIYD------------KKA--   173
_XP_034950475.1_   139   -SVRLCFQVFLEGTEK----------GKFT------VPLS----PVVSDPIYD------------KKA--   173
_XP_012343274.1_   139   -AVRLCFQVFLEGSQK----------RKFN------VPLQ----PVVSDPIFD------------KKA--   173
_XP_016909449.1_   139   -AVRLCFQVFLEGSQK----------RKFN------VPLQ----PVVSDPIFD------------KKA--   173
_XP_012343273.1_   139   -AVRLCFQVFLEGSQK----------RKFN------VPLQ----PVVSDPIFD------------KKA--   173
_XP_006619742.1_   139   -AVRLCFQVFLEGSQK----------RKFN------VPLQ----PVVSDPIFD------------KKA--   173
_XP_006567065.1_   139   -AVRLCFQVFLEGSQK----------RKFN------VPLQ----PVVSDPIFD------------KKA--   173
_XP_006619741.1_   139   -AVRLCFQVFLEGSQK----------RKFN------VPLQ----PVVSDPIFD------------KKA--   173
_NP_001011577.1_   139   -AVRLCFQVFLEGSQK----------RKFN------VPLQ----PVVSDPIFD------------KKA--   173
_XP_016909448.1_   139   -AVRLCFQVFLEGSQK----------RKFN------VPLQ----PVVSDPIFD------------KKA--   173
_XP_043802365.1_   139   -AVRLCFQVFLEGSQK----------RKFN------VPLQ----PVVSDPIFD------------KKA--   173
_NP_001164477.1_   139   -AVRLCFQVFLEGSQK----------RKFN------VPLQ----PVVSDPIFD------------KKA--   173
_XP_016909443.1_   139   -AVRLCFQVFLEGSQK----------RKFN------VPLQ----PVVSDPIFD------------KKA--   173
```

```
_XP_043802361.1_  139  -AVRLCFQVFLEGSQK----------RKFN------VPLQ----PVVSDPIFD------------KKA--  173
_XP_012343269.1_  139  -AVRLCFQVFLEGSQK----------RKFN------VPLQ----PVVSDPIFD------------KKA--  173
_XP_006619738.1_  139  -AVRLCFQVFLEGSQK----------RKFN------VPLQ----PVVSDPIFD------------KKA--  173
_PBC29878.1_      139  -AVRLCFQVFLEGSQK----------RKFN------VPLQ----PVVSDPIFD------------KKA--  173
_XP_046663984.1_  139  -AVRLCFQAFLE-EEP----------GKFK------RALM----PVISDIIYD------------KKA--  172
_XP_046663985.1_  139  -AVRLCFQAFLE-EEP----------GKFK------RALM----PVISDIIYD------------KKA--  172
_XP_043274771.1_  139  -AVRLCFQVFLEGTEK----------GKFN------VPLP----SVVSDPIYD------------KKA--  173
_XP_043274769.1_  139  -AVRLCFQVFLEGTEK----------GKFN------VPLP----SVVSDPIYD------------KKA--  173
_XP_043289571.1_  139  -AVRMCFQVFVEGRE-----------GKIN------VPLQ----PVVSQPIYD------------KKA--  172
_XP_032665873.1_  139  -AVRLCFQVFLEGSRK----------GKFT------RPLA----PIVSDPIYD------------KKA--  173
_XP_032665875.1_  139  -AVRLCFQVFLEGSRK----------GKFT------RPLA----PIVSDPIYD------------KKA--  173
_XP_032665874.1_  139  -AVRLCFQVFLEGSRK----------GKFT------RPLA----PIVSDPIYD------------KKA--  173
_XP_032665869.1_  139  -AVRLCFQVFLEGSRK----------GKFT------RPLA----PIVSDPIYD------------KKA--  173
_XP_014470289.1_  139  -AVRLCFQVFLEGSRK----------GKFT------KPLA----PIVSDPIYD------------KKA--  173
_XP_011149046.1_  139  -AVRLCFQVFLEGSRK----------GKFT------RPLT----PIVSDPIYD------------KKA--  173
_XP_017877914.1_  138  -AVRLCFQVFLEGSQK-----------KLN------VPLP----PIVSDPIYD------------KKA--  171
_XP_017877911.1_  138  -AVRLCFQVFLEGSQK-----------KLN------VPLP----PIVSDPIYD------------KKA--  171
_XP_033174774.1_  139  -AVRLCFQVFLEGGQK----------GKFN------VPLP----PVVSDPIFD------------KKA--  173
_XP_043581013.1_  139  -AVRLCFQVFLEGGQK----------GKFN------VPLP----PVVSEPIFD------------KKA--  173
_XP_048261507.1_  139  -AVRLCFQVFLEGGQK----------GKFN------VPLP----PVVSDPIFD------------KKA--  173
_XP_050573946.1_  139  -AVRLCFQVFLEGGQK----------GKFN------VPLP----PVVSDPIFD------------KKA--  173
_XP_050478075.1_  139  -AVRLCFQVFLEGGQK----------GKFN------VPLP----PVVSDPIFD------------KKA--  173
_XP_033204249.1_  139  -AVRLCFQVFLEGGQK----------GKFN------VPLP----PVVSDPIFD------------KKA--  173
_XP_033358451.1_  139  -AVRLCFQVFLEGGQK----------GKFN------VPLP----PVVSDPIFD------------KKA--  173
_XP_033358450.1_  139  -AVRLCFQVFLEGGQK----------GKFN------VPLP----PVVSDPIFD------------KKA--  173
_XP_033204244.1_  139  -AVRLCFQVFLEGGQK----------GKFN------VPLP----PVVSDPIFD------------KKA--  173
_XP_033312956.1_  139  -AVRLCFQVFLEGGQK----------GKFN------VPLP----PVVSDPIFD------------KKA--  173
_XP_012236639.1_  139  -AVRLCFQVFLEGGQK----------GKFN------VPLP----PVVSDPIFD------------KKA--  173
_XP_050573940.1_  139  -AVRLCFQVFLEGGQK----------GKFN------VPLP----PVVSDPIFD------------KKA--  173
_XP_012174118.1_  139  -AVRLCFQVFLEGGQK----------GKFN------VPLP----PVVSDPIFD------------KKA--  173
_XP_043581010.1_  139  -AVRLCFQVFLEGGQK----------GKFN------VPLP----PVVSEPIFD------------KKA--  173
_EZA51108.1_      139  -AVRLCFQVFLEGSQK----------GKFN------KPLA----PIVSDPIYD------------KKA--  173
_XP_011344085.1_  139  -AVRLCFQVFLEGSQK----------GKFN------KPLA----PIVSDPIYD------------KKA--  173
_XP_043519475.1_  139  -AVRLCFQVFLEGTQK----------GKFN------VPLS----PIVSDPIFD------------KKA--  173
_CAD1481114.1_    139  -AVRLCFQVFLEGTQK----------GKFN------VPLS----PIVSDPIFD------------KKA--  173
_XP_043519473.1_  139  -AVRLCFQVFLEGTQK----------GKFN------VPLS----PIVSDPIFD------------KKA--  173
_KOX75360.1_      139  -AVRLCFQVFLEGTQK----------GKFN------VPLS----PIVSDPIFD------------KKA--  173
_KAF3423741.1_    139  -AVRLCFQVFLEGTQK----------GKFN------VPLS----PIVSDPIFD------------KKA--  173
_KOC69050.1_      139  -AVRLCFQVFLDGSQK----------GKFN------VALP----PVVSDPIFD------------KKA--  173
```

```
_XP_053980095.1_  139  -AVRLCFQVFIEGSEK----------AKFN------LPLK----PVVSDIIYD------------KKA--  173
_XP_054002893.1_  139  -AVRLCFQVFIEGSEK----------AKFN------LPLK----PVVSDIIYD------------KKA--  173
_XP_053980091.1_  139  -AVRLCFQVFIEGSEK----------AKFN------LPLK----PVVSDIIYD------------KKA--  173
_XP_028049144.1_  139  -AVRLCFQVFLEGSQK----------GKFN------KPLA----PIVSDPIYD------------KKA--  173
_XP_012541458.1_  139  -AVRLCFQVFLEGSQK----------GKFN------KPLA----PIVSDPIYD------------KKA--  173
_XP_003708412.1_  139  -AVRLCFQGFIEGSQK----------RKFN------VPLA----PVVSDPIFD------------KKA--  173
_XP_012152807.1_  139  -AVRLCFQGFIEGSQK----------RKFN------VPLA----PVVSDPIFD------------KKA--  173
_KAH0945556.1_    139  -AVRLCFQVFLEGSQK----------GKFN------KPLA----PVVSDPIYD------------KKA--  173
_XP_024871832.1_  139  -AVRLCFQVFLEGQR-----------GKFN------KPLP----PVVSDPIYD------------KKA--  172
_XP_011307641.1_  138  -AVRLCFQVFLEGPTP----------GKFK------MPLK----PIVSDPIYD------------KKA--  172
_XP_015118260.1_  138  -AVRLCFQVFLEGPTP----------GKFK------TPLK----PIVSDPIYD------------KKA--  172
_XP_011307640.1_  138  -AVRLCFQVFLEGPTP----------GKFK------MPLK----PIVSDPIYD------------KKA--  172
_XP_015118252.1_  138  -AVRLCFQVFLEGPTP----------GKFK------TPLK----PIVSDPIYD------------KKA--  172
_EFN68841.1_      143  -AVRLCFQVFLEGSQK----------GKFN------KPLP----PVVSDPIYD------------KKA--  177
_XP_011647340.1_  139  -AVRLCFQVFLEGSQK----------GKFN------KPLP----PIVSDPIYD------------KKA--  173
_XP_029166122.1_  143  -AVRLCFQVFLEGSQK----------GKFN------KPLP----PVVSDPIYD------------KKA--  177
_KMQ96453.1_      143  -AVRLCFQVFLEGSQK----------GKFN------KPLP----PVVSDPIYD------------KKA--  177
_XP_050464492.1_  143  -AVRLCFQVFLEGSQK----------GKFN------KPLP----PVVSDPIYD------------KKA--  177
_XP_029673315.1_  143  -AVRLCFQVFLEGSQK----------GKFN------KPLP----PVVSDPIYD------------KKA--  177
_XP_025265518.1_  143  -AVRLCFQVFLEGSQK----------GKFN------KPLP----PVVSDPIYD------------KKA--  177
_XP_011255523.1_  143  -AVRLCFQVFLEGSQK----------GKFN------KPLP----PVVSDPIYD------------KKA--  177
_XP_011698133.1_  139  -VVRLCFQVFLEGSQK----------GKFN------RPLQ----PIVSDPIYD------------KKA--  173
_XP_011698131.1_  139  -VVRLCFQVFLEGSQK----------GKFN------RPLQ----PIVSDPIYD------------KKA--  173
_XP_018357420.1_  139  -AVRLCFQVFLEGSQK----------GKFN------KPLA----PIVSDPIYD------------KKA--  173
_KYM76140.1_      139  -AVRLCFQVFLEGSQK----------GKFN------KPLA----PIVSDPIYD------------KKA--  173
_XP_011647339.1_  139  -AVRLCFQVFLEGSQK----------GKFN------KPLP----PIVSDPIYD------------KKA--  173
_KAG5315434.1_    139  -AVRLCFQVFLEGSQK----------GKFN------KPLA----PIVSDPIYD------------KKA--  173
_XP_011055463.1_  139  -AVRLCFQVFLEGSQK----------GKFN------KPLA----PIVSDPIYD------------KKA--  173
_XP_018342550.1_  139  -AVRLCFQVFLEGSQK----------GKFN------KPLA----PIVSDPIYD------------KKA--  173
_XP_018403565.1_  139  -AVRLCFQVFLEGSQK----------GKFN------KPLA----PIVSDPIYD------------KKA--  173
_KAG5322912.1_    139  -AVRLCFQVFLEGSQK----------GKFN------KPLA----PIVSDPIYD------------KKA--  173
_XP_018357417.1_  139  -AVRLCFQVFLEGSQK----------GKFN------KPLA----PIVSDPIYD------------KKA--  173
_XP_018314560.1_  139  -AVRLCFQVFLEGSQK----------GKFN------KPLA----PIVSDPIYD------------KKA--  173
_XP_018057217.1_  139  -AVRLCFQVFLEGSQK----------GKFN------KPLA----PIVSDPIYD------------KKA--  173
_KAG5346558.1_    139  -AVRLCFQVFLEGSQK----------GKFN------KPLA----PIVSDPIYD------------KKA--  173
_XP_047348408.1_  139  -AVRLCFQVFLEGSQK----------GKFT------HPLT----PVVSDPIFD------------KKA--  173
_XP_047348412.1_  139  -AVRLCFQVFLEGSQK----------GKFT------HPLT----PVVSDPIFD------------KKA--  173
_XP_046820611.1_  139  -AVRLCFQVFLEGSQK----------GKFT------HPLT----PVVSDPIFD------------KKA--  173
_XP_050848254.1_  139  -AVRLCFQVFLEGSQK----------GKFT------HPLS----PVVSDPIFD------------KKA--  173
```

```
_XP_046820612.1_  139  -AVRLCFQVFLEGSQK----------GKFT------HPLT----PVVSDPIFD------------KKA--  173
_XP_050848252.1_  139  -AVRLCFQVFLEGSQK----------GKFT------HPLS----PVVSDPIFD------------KKA--  173
_XP_043667564.1_  139  -AVRLCFQVFLEGSQK----------GKFT------HPLS----PVVSDPIFD------------KKA--  173
_XP_035725337.1_  139  -AVRLCFQVFLEGSQK----------GKFT------HPLT----PVVSDPIFD------------KKA--  173
_XP_043667562.1_  139  -AVRLCFQVFLEGSQK----------GKFT------HPLS----PVVSDPIFD------------KKA--  173
_XP_046820605.1_  139  -AVRLCFQVFLEGSQK----------GKFT------HPLT----PVVSDPIFD------------KKA--  173
_XP_050848249.1_  139  -AVRLCFQVFLEGSQK----------GKFT------HPLS----PVVSDPIFD------------KKA--  173
_XP_043667560.1_  139  -AVRLCFQVFLEGSQK----------GKFT------HPLS----PVVSDPIFD------------KKA--  173
_XP_035725333.1_  139  -AVRLCFQVFLEGSQK----------GKFT------HPLT----PVVSDPIFD------------KKA--  173
_KAF7405659.1_    139  -AVRLCFQVFLEGSQK----------GKFT------HPLS----PVVSDPIFD------------KKA--  173
_KAF7402294.1_    139  -AVRLCFQVFLEGSQK----------GKFT------HPLS----PVVSDPIFD------------KKA--  173
_XP_015172825.1_  139  -AVRLCFQVFLEGTRK----------GKFT------HPLQ----PVVSDPIFD------------KKA--  173
_XP_015172824.1_  139  -AVRLCFQVFLEGTRK----------GKFT------HPLQ----PVVSDPIFD------------KKA--  173
_XP_015172823.1_  139  -AVRLCFQVFLEGTRK----------GKFT------HPLQ----PVVSDPIFD------------KKA--  173
_XP_014609306.1_  139  -AVRLCFQVFLEGTRK----------GKFT------HPLQ----PVVSDPIFD------------KKA--  173
_XP_043491219.1_  139  -AVRLCFQVFLEGTRK----------GKFT------HPLQ----PVVSDPIFD------------KKA--  173
_XP_014609305.1_  139  -AVRLCFQVFLEGTRK----------GKFT------HPLQ----PVVSDPIFD------------KKA--  173
_XP_043491218.1_  139  -AVRLCFQVFLEGTRK----------GKFT------HPLQ----PVVSDPIFD------------KKA--  173
_KAI4498556.1_    139  -AVRLCFQVFLEKERK----------GKFT------QVLQ----PVVSDPIFD------------KKA--  173
_XP_015172818.1_  139  -AVRLCFQVFLEGTRK----------GKFT------HPLQ----PVVSDPIFD------------KKA--  173
_XP_043491211.1_  139  -AVRLCFQVFLEGTRK----------GKFT------HPLQ----PVVSDPIFD------------KKA--  173
_KAI4495142.1_    139  -AVRLCFQVFLEGTRK----------GKFT------HPLQ----PVVSDPIFD------------KKA--  173
_XP_014609300.1_  139  -AVRLCFQVFLEGTRK----------GKFT------HPLQ----PVVSDPIFD------------KKA--  173
_ADK91833.1_      139  -AVRLCFQVFLEGGQK----------GKFN------VPLP----PVVSDPIFD------------KKA--  173
_KAG7213837.1_    139  -AVRLCFQVFIEGSQK----------GKCN------VPLP----PVVSDPIFD------------KKA--  173
_XP_014226628.1_  139  -VIRLCFQVFVM-NKE----------GKFN------RGLA----PVVSEPIYD------------KKA--  172
_XP_020287946.1_  139  -AVRLCFQVFTEGSQK----------GKFN------RPLQ----PVVSDPIYD------------KKA--  173
_XP_012232007.1_  139  -AVRLCFQVFLEGSQK----------GKFN------KPLQ----PIVSDPIYD------------KKA--  173
_XP_011874538.1_  139  -SVRLCFQVFLEGSQK----------GKFN------KPLP----PIVSDPIYD------------KKA--  173
_XP_011874541.1_  139  -SVRLCFQVFLEGSQK----------GKFN------KPLP----PIVSDPIYD------------KKA--  173
_XP_011173619.1_  139  -AVRLCFQVFLEGKQK----------GKFN------KPLP----PIVSDPIYD------------KKA--  173
_XP_034950793.1_  137  -AVRLCFQVFLQGRN-----------GKFQ------IPLK----PVVSNPVYD------------KKT--  170
_XP_034950792.1_  133  -AVRLCFQVFLQGRN-----------GKFQ------IPLK----PVVSNPVYD------------KKT--  166
_XP_012340880.2_  136  -SVRLCFQVFLEGSQK----------GKFN------VPLP----PVVSDPIFD------------KKI--  170
_XP_031771859.1_  136  -SVRLCFQVFLEGSQK----------GKFN------VPLP----PVVSDPIFD------------KKI--  170
_XP_006618953.1_  138  -SVRLCFQVFLEGSQK----------GKFN------VALP----PIVSDPIFD------------KKI--  172
_XP_031367904.1_  138  -SVRLCFQVFLEGSQK----------GKFN------VALP----PIVSDPIFD------------KKI--  172
_XP_043795948.1_  138  -SVRLCFQVFLEGSQK----------GKFN------VPLS----PIVSDPIFD------------KKI--  172
_XP_395180.5_     138  -AVRLCFQVFLEGSQK----------GKFN------VPLS----PIVSDPIFD------------KKI--  172
```

```
_KAG6799500.1_     138  -AVRLCFQVFLEGSQK----------GKFN------VPLS----PIVSDPIFD------------KKI--  172
_XP_016768607.2_   138  -AVRLCFQVFLEGSQK----------GKFN------VPLS----PIVSDPIFD------------KKI--  172
_PBC31989.1_       147  -SVRLCFQVFLEGTQK----------GKFN------VPLT----PIVSDPIFD------------KKI--  181
_XP_016914450.1_   138  -SVRLCFQVFLEGTQK----------GKFN------VPLT----PIVSDPIFD------------KKI--  172
_KAG5344958.1_     139  -AVRLCFQLILNDFKS----------SWMV------LKDQ----HTYHKPINL-----------------  170
_KAJ3630188.1_     137  -AVRLCFQVVIEGEVP----------GQFS------VVLN----PVVSDPIYN------------RRT--  171
_QHN75199.1_       137  -AVRLCFQVVIEGEVP----------GQFS------VVLN----PVVSDPIYN------------RRT--  171
_XP_051171660.1_   143  -VVRLCFQGFIPRKIN----------GKKD-----WEPLA----PVVSNPIVE------------KKA--  178
_XP_016909447.1_    97  -AVRLCFQVFLEGSQK----------RKFN------VPLQ----PVVSDPIFD------------KKA--  131
_XP_031773030.1_    97  -AVRLCFQVFLEGSQK----------RKFN------VPLQ----PVVSDPIFD------------KKA--  131
_KAH0956427.1_     144  -SVRLCFQVYVEGSQK----------GKFN------VPLV----PIVSNPIYD------------KKL--  178
_XP_012283784.1_   139  -AVRLCFQVFLEGTEK----------GKFT------VPLT----PVVSDPIYD------------KKA--  173
_XP_023224928.1_   139  -VVRLAFQVILMSPDK-----------TKK------YPLK----PVVSDPIYD------------KKA--  172
_XP_008556434.1_   139  -AVRLCFQVFLEGTDK----------GKFT------VPLT----PIVSDPIYD------------KKA--  173
_XP_008556433.1_   139  -AVRLCFQVFLEGTDK----------GKFT------VPLT----PIVSDPIYD------------KKA--  173
_XP_014299728.1_   139  -AVRLCFQVFLEGTDK----------GKFT------VPLT----PIVSDPIYD------------KKA--  173
_CAD6234449.1_     139  -AVRLCFQVFLEGTEP----------GKFT------VPLT----PVVSDPIYD------------KKA--  173
_KAG8037552.1_     139  -AVRLCFQVFLEGTEP----------GKFT------VPLT----PVVSDPIYD------------KKA--  173
_XP_044576138.1_   139  -AVRLCFQVFLEGTEP----------GKFT------VPLT----PVVSDPIYD------------KKA--  173
_XP_044576125.1_   139  -AVRLCFQVFLEGTEP----------GKFT------VPLT----PVVSDPIYD------------KKA--  173
_XP_026465448.1_   139  -AVRLCFQVFLEDTP-----------GKFT-------PLR----VVVSNPIYD------------KKA--  171
_XP_026465446.1_   139  -AVRLCFQVFLEDTP-----------GKFT-------PLR----VVVSNPIYD------------KKA--  171
_XP_031785072.1_   139  -AVRLCFQVFVEGQEK----------GKFT------VPLQ----PVVSEPIYD------------KKA--  173
_XP_015607185.1_   139  -AVRLCFQVFLEGAEK----------GKFN------VSLP----PVVSDPIYD------------KKA--  173
_XP_011496611.1_   139  -AVRLCFQVFLEGQEK----------GKFT------LPLQ----PTVSEPIYD------------KKA--  173
_XP_018903707.1_   139  -VVRFCFQAFFRTNQW--------------------VTIP----PVVSEPIYD------------KKA--  169
_CAB3365562.1_     138  -AVRLCFQVFTEGDKP----------NTFT------KGLP----PVASNIIYD------------KKA--  172
_XP_011496447.1_    12  -CIRLCFQVFIETTQN-------------L------IPLQ----PIVSEPIYD------------KKA--   43
_KAG8230996.1_     138  -AVRLCFQVFLEGAEK----------GKFN------QPLT----PVVSDPIYD------------KKA--  172
_XP_040567422.1_   138  -AVRLCFQAFLKN-------------GESL------TPLP----AVVSNTVVD------------RKV--  169
_XP_014250205.1_   139  -AVRLCFQAFIEGPQR----------GRFT------VPLK----PVVSEPIYD------------KKA--  173
_XP_014250206.1_   139  -AVRLCFQAFIEGPQR----------GRFT------VPLK----PVVSEPIYD------------KKA--  173
_XP_047119528.1_   139  -AVRLCFQVFLEGSER----------GKFT------FQLP----PVVSDPIYD------------KKA--  173
_XP_047001845.1_   139  -AVRLCFQVFLEGSER----------GKFT------FQLP----PVVSDPIYD------------KKA--  173
_XP_049939433.1_   139  -AVRLCFQVFLEGSER----------GKFT------FQLP----PVVSDPIYD------------KKA--  173
_XP_049764197.1_   139  -AVRLCFQVFLEGSER----------GKFT------FQLP----PVVSDPIYD------------KKA--  173
_XP_049833656.1_   139  -AVRLCFQVFLEGSER----------GKFT------FQLP----PVVSDPIYD------------KKA--  173
_XP_049790026.1_   139  -AVRLCFQVFLEGSER----------GKFT------FQLP----PVVSDPIYD------------KKA--  173
_XP_049833651.1_   139  -AVRLCFQVFLEGSER----------GKFT------FQLP----PVVSDPIYD------------KKA--  173
```

```
_XP_047001842.1_  139  -AVRLCFQVFLEGSER----------GKFT------FQLP----PVVSDPIYD------------KKA--  173
_XP_049790025.1_  139  -AVRLCFQVFLEGSER----------GKFT------FQLP----PVVSDPIYD------------KKA--  173
_XP_049833654.1_  139  -AVRLCFQVFLEGSER----------GKFT------FQLP----PVVSDPIYD------------KKA--  173
_XP_049833659.1_  139  -AVRLCFQVFLEGSER----------GKFT------FQLP----PVVSDPIYD------------KKA--  173
_XP_021925503.1_  139  -AVRLCFQVFLEDGER----------DKFT------LPLA----PVVSDPIYD------------KKA--  173
_KDR23857.1_      139  -AVRLCFQVFLEDGER----------DKFT------LPLA----PVVSDPIYD------------KKA--  173
_ABU96700.1_      139  -SVRLCFQAFLEGPQR----------GKFT------NPLS----PIVSEPIYD------------KKA--  173
_ABU96699.1_      139  -SVRLCFQAFLEGPQR----------GKFT------NPLS----PIVSEPIYD------------KKA--  173
_XP_014275495.1_  139  -SVRLCFQAFLEGPQR----------GKFT------KALT----PVVSDPIYD------------KKA--  173
_BBE08137.1_      139  -SVRLCFQAFLEGPQR----------GKFT------RALI----PVVSDPIYD------------KKA--  173
_XP_014275494.1_  139  -SVRLCFQAFLEGPQR----------GKFT------KALT----PVVSDPIYD------------KKA--  173
_XP_014275493.1_  139  -SVRLCFQAFLEGPQR----------GKFT------KALT----PVVSDPIYD------------KKA--  173
_BBE08136.1_      139  -SVRLCFQAFLEGPQR----------GKFT------RALI----PVVSDPIYD------------KKA--  173
_KAF6207519.1_    139  -AVRLCFQVFLEGPTP----------GKFT------VPLQ----PITSEPIYD------------KKA--  173
_CAB0015508.1_    139  -AVRLCFQVFLEGPAP----------GKFT------IPLP----PVTSEPIYD------------KKA--  173
_CAB0015504.1_    139  -AVRLCFQVFLEGPAP----------GKFT------IPLP----PVTSEPIYD------------KKA--  173
_PNF18474.1_      139  -AVRLCFQVFLEDGVP----------NKFT------YPLT----PVVSDPIYD------------KKA--  173
_CEG62431.1_      139  -AVRLCFQVFLEDEER----------ERFT------LPLA----PVVSEPIFD------------KKA--  173
_PSN36690.1_      139  -AVRLCFQVFLEDEER----------ERFT------LPLA----PVVSEPIFD------------KKA--  173
_XP_046387412.1_  139  -AVRLCFQVFLEGSER----------GKFN------QPLT----PVVSDPIYD------------KKA--  173
_XP_046387414.1_  139  -AVRLCFQVFLEGSER----------GKFN------QPLT----PVVSDPIYD------------KKA--  173
_XP_031634874.1_  139  -AVRLCFQVFLKDPEG----------HNFN------IPLE----PVVSEPIFD------------KKA--  173
_GFG29240.1_      139  -AVRLCFQVFIEDGER----------DKFT------YPLT----PVVSDPIYD------------KKA--  173
_BBG28464.1_      139  -AVRLCFQVFLEGSEK----------GKFT------HGLP----PVVSDPIYD------------KKA--  173
_WCL52334.1_      139  -AVRLCFQVFLEGSEK----------GKFV------HGLP----PVVSDPIYD------------KKA--  173
_GLH00135.1_      139  -AVRLCFQVFLEGSEK----------GKFT------HGLP----PVVSDPIYD------------KKA--  173
_CAD7205239.1_    139  -AVRLCFQVFLEGNEK----------GKFT------FQLP----PVVSEPIYD------------KKA--  173
_CAD7431383.1_    139  -AVRLCFQVFLEGNEK----------GKFT------FQLP----PVVSEPIYD------------KKA--  173
_CAD7410836.1_    139  -AVRLCFQVFLEGNEK----------GKFT------FQLP----PVVSEPIYD------------KKA--  173
_CAD7415345.1_    139  -AVRLCFQVFLEGNEK----------GKFT------FQLP----PVVSEPIYD------------KKA--  173
_CAD7606635.1_    139  -AVRLCFQVFLEGNEK----------GKFT------FQLP----PVVSEPIYD------------KKA--  173
_CAD7462957.1_    139  -AVRLCFQVFLEGNEK----------GKFT------FQLP----PVVSEPIYD------------KKA--  173
_XP_012265400.2_  139  -AVRLCFQVFLEGAEK----------GKFT------VPLA----PVVSDPIYD------------KKA--  173
_XP_046473587.1_  139  -AVRLCFQVFLESTKK----------GKFS------VALQ----PVVSDPIYD------------KKA--  173
_XP_046611732.1_  139  -AVRLCFQVFLESTKK----------GKFS------VALQ----PVVSDPIYD------------KKA--  173
_XP_015519750.2_  139  -AVRLCFQVFLESTKK----------GKFS------VALQ----PVVSDPIYD------------KKA--  173
_XP_046611731.1_  139  -AVRLCFQVFLESTKK----------GKFS------VALQ----PVVSDPIYD------------KKA--  173
_XP_046417903.1_  139  -AVRLCFQVFLESTKK----------GKFS------VALQ----PVVSDPIYD------------KKA--  173
_GLG94364.1_      138  -AVRLCFQVFLEGSEK----------GKFT------FQLT----PVVSDPIYD------------KKA--  172
```

```
_GLG94363.1_       138  -AVRLCFQVFLEGSEK----------GKFT------FQLT----PVVSDPIYD------------KKA--  172
_BBG28463.1_       138  -AVRLCFQVFLEGSEK----------GKFT------FQLT----PVVSDPIYD------------KKA--  172
_WCL52335.1_       138  -AVRLCFQVFLEGSEK----------GKFT------FQLT----PVVSDPIYD------------KKA--  172
_GLG94365.1_       138  -AVRLCFQVFLEGSEK----------GKFT------FQLT----PVVSDPIYD------------KKA--  172
_XP_002431518.1_   137  -SVRLCFQVFLEGPKK----------EEFT------IPLR----PIVSDPIYD------------KKA--  171
_KAE8746525.1_     139  -AVRLCFQVFLEGPEP----------NTYT------RALA----PVVSEPIYD------------KKA--  173
_XP_026277770.1_   139  -AVRLCFQVFLEGPEP----------NTYT------RALA----PVVSEPIYD------------KKA--  173
_KAJ1519446.1_     139  -AVRLCFQVFLEGPEP----------NTYT------RALA----PVVSEPIYD------------KKA--  173
_ALC78349.1_       139  -SVRLCFQVFVQQDKN----------SSY-------VPLP----PEVSDPIYD------------KKA--  172
_XP_046738772.1_   139  -AVRLCFQVFLESSKK----------GKFS------VGLQ----PVVSDPIFD------------KKA--  173
_XP_046417904.1_   139  -AVRLCFQVFLESTKK----------GKFS------VALQ----PVVSDPIYD------------KKA--  173
_CRK94170.1_       139  -AVRLCFQVFLEGPQK----------GKFT------TLVK----PVVSEPIYD------------KKS--  173
_CRK94167.1_       139  -AVRLCFQVFLEGPQK----------GKFT------TLVK----PVVSEPIYD------------KKS--  173
_KAG5676160.1_     139  -AVRLCFQVFLEGPQR----------GRFT------QPLK----PVVSEPIFD------------KKS--  173
_XP_034245464.1_   140  -AVRLCFQVFLEGPEP----------NTYT------RALA----PVVSDPIYD------------KKA--  174
_XP_034245457.1_   140  -AVRLCFQVFLEGPEP----------NTYT------RALA----PVVSDPIYD------------KKA--  174
_KAG8287980.1_     139  -AVRLCFQAFLE-EEP----------GKFK------RALM----PVISDIIYD------------KK---  171
_XP_044759045.1_   135  -AVRLCFQVFTEGEN-----------GKFT------VPIP----PVVSDPIYD------------RKN--  168
_XP_017305229.1_   135  -AVRLCFQAYL--PKD----------KDNV------IKLE----PVVSDIIYD------------AKT--  167
_XP_026675878.1_   135  -AVRLCFQAYL--PKD----------KDNV------IKLE----PVVSDIIYD------------AKT--  167
_KAE8574083.1_     124  ------------------------------------------------------------------KA--  125
_XP_037926497.1_   139  -SVRLCFQVFLESDVK----------GRFT------TPLT----PVVSDPVYD------------KKA--  173
_CAD7079567.1_     139  -SVRLCFQVFLESDVK----------GRFT------TPLT----PVVSDPVYD------------KKA--  173
_XP_037882485.1_   139  -SVRLCFQVFMEGEQK----------GRFT------QPLP----PVVSEPIYD------------KKA--  173
_XP_037037597.1_   139  -AVRLCFQVFLESEQR----------GRFT------VPLS----PVVSDPIFD------------KKA--  173
_XP_037037592.1_   139  -AVRLCFQVFLESEQR----------GRFT------VPLS----PVVSDPIFD------------KKA--  173
_KAG4075389.1_     139  -AVRLCFQVFLESEQR----------GRFT------VPLS----PVVSDPIFD------------KKA--  173
_XP_034101357.1_   139  -SVRLCFQVFMESEQK----------GRFT------SPLP----PVVSEPIYD------------KKA--  173
_XP_026844963.1_   139  -SVRLCFQVFMESEQK----------GRFT------SPLP----PVVSEPIFD------------KKA--  173
_EDW28798.1_       139  -SVRLCFQVFMESEQK----------GRFT------SPLP----PVVSEPIFD------------KKA--  173
_XP_034125966.1_   139  -SVRLCFQVFMESEQK----------GRFT------SPLP----PVVSEPIFD------------KKA--  173
_XP_034666519.1_   139  -SVRLCFQVFMESEQK----------GRFT------SPLP----PVVSEPIFD------------KKA--  173
_XP_001356570.3_   139  -SVRLCFQVFMESEQK----------GRFT------SPLP----PVVSEPIFD------------KKA--  173
_XP_023164577.2_   139  -SVRLCFQVFMESEQK----------GRFT------SPLP----PVVSEPIFD------------KKA--  173
_XP_017153206.1_   139  -SVRLCFQVFMESEQK----------GRFT------SPLP----PVVSEPIFD------------KKA--  173
_XP_030564932.1_   139  -SVRLCFQVFMESEQK----------GRFT------SPLP----PVVSEPIFD------------KKA--  173
_XP_002059145.1_   139  -SVRLCFQVFMESEQK----------GRFT------SPLP----PVVSEPIFD------------KKA--  173
_EDW04266.1_       139  -SVRLCFQVFMESEQK----------GRFT------SPLP----PVVSEPIFD------------KKA--  173
_XP_017025862.1_   139  -SVRLCFQVFMESEQK----------GRFT------SPLP----PVVSEPIFD------------KKA--  173
```

```
_XP_017859907.1_  139  -SVRLCFQVFMESEQK----------GRFT------SPLP----PVVSEPIFD------------KKA--  173
_XP_032584327.1_  139  -SVRLCFQVFMESEQK----------GRFT------SPLP----PVVSEPIFD------------KKA--  173
_XP_016987288.2_  139  -SVRLCFQVFMESEQK----------GRFT------SPLP----PVVSEPIFD------------KKA--  173
_XP_017954173.1_  139  -SVRLCFQVFMESEQK----------GRFT------SPLP----PVVSEPIFD------------KKA--  173
_XP_017854504.1_  139  -SVRLCFQVFMESEQK----------GRFT------SPLP----PVVSEPIFD------------KKA--  173
_XP_034125965.1_  139  -SVRLCFQVFMESEQK----------GRFT------SPLP----PVVSEPIFD------------KKA--  173
_XP_034666515.1_  139  -SVRLCFQVFMESEQK----------GRFT------SPLP----PVVSEPIFD------------KKA--  173
_XP_043641917.1_  139  -SVRLCFQVFMESEQK----------GRFT------SPLP----PVVSEPIFD------------KKA--  173
_XP_026844960.1_  139  -SVRLCFQVFMESEQK----------GRFT------SPLP----PVVSEPIFD------------KKA--  173
_XP_015035988.2_  139  -SVRLCFQVFMESEQK----------GRFT------SPLP----PVVSEPIFD------------KKA--  173
_XP_041450744.1_  139  -SVRLCFQVFMESEQK----------GRFT------SPLP----PVVSEPIFD------------KKA--  173
_XP_015052361.1_  139  -SVRLCFQVFMESEQK----------GRFT------SPLP----PVVSEPIFD------------KKA--  173
_XP_039480515.1_  139  -SVRLCFQVFMESEQK----------GRFT------SPLP----PVVSEPIFD------------KKA--  173
_XP_033248563.1_  139  -SVRLCFQVFMESEQK----------GRFT------SPLP----PVVSEPIFD------------KKA--  173
_KAH8412150.1_    139  -SVRLCFQVFMESEQK----------GRFT------SPLP----PVVSEPIFD------------KKA--  173
_XP_043864974.1_  139  -SVRLCFQVFMESEQK----------GRFT------SPLP----PVVSEPIFD------------KKA--  173
_XP_020809119.1_  139  -SVRLCFQVFMESEQK----------GRFT------SPLP----PVVSEPIFD------------KKA--  173
_KAH8298353.1_    139  -SVRLCFQVFMESEQK----------GRFT------SPLP----PVVSEPIFD------------KKA--  173
_XP_030564927.1_  139  -SVRLCFQVFMESEQK----------GRFT------SPLP----PVVSEPIFD------------KKA--  173
_XP_041631592.1_  139  -SVRLCFQVFMESEQK----------GRFT------SPLP----PVVSEPIFD------------KKA--  173
_XP_017859909.1_  139  -SVRLCFQVFMESEQK----------GRFT------SPLP----PVVSEPIFD------------KKA--  173
_XP_032591602.1_  139  -SVRLCFQVFMESEQK----------GRFT------SPLP----PVVSEPIFD------------KKA--  173
_XP_023164572.2_  139  -SVRLCFQVFMESEQK----------GRFT------SPLP----PVVSEPIFD------------KKA--  173
_TDG46699.1_      139  -SVRLCFQVFMESEQK----------GRFT------SPLP----PVVSEPIFD------------KKA--  173
_KAH8411895.1_    139  -SVRLCFQVFMESEQK----------GRFT------SPLP----PVVSEPIFD------------KKA--  173
_KAH8377496.1_    139  -SVRLCFQVFMESEQK----------GRFT------SPLP----PVVSEPIFD------------KKA--  173
_XP_017854500.2_  139  -SVRLCFQVFMESEQK----------GRFT------SPLP----PVVSEPIFD------------KKA--  173
_XP_016987284.2_  139  -SVRLCFQVFMESEQK----------GRFT------SPLP----PVVSEPIFD------------KKA--  173
_KAH8293209.1_    139  -SVRLCFQVFMESEQK----------GRFT------SPLP----PVVSEPIFD------------KKA--  173
_EEC00225.1_      138  -AVRLCFQVFLEGSTP----------GKYT------MPLH----PVVSEIIYD------------RKA--  172
_XP_029825505.2_  138  -AVRLCFQVFLEGSTP----------GKYT------VPLH----PVVSEIIYD------------RKA--  172
_KAG0424790.1_    138  -AVRLCFQVFLEGSTP----------GKYT------VPLH----PVVSEIIYD------------RKA--  172
_XP_037272127.1_  138  -AVRLCFQVFLEGSQP----------GKFT------VPLH----PVVSDIIYD------------RKA--  172
_XP_054934017.1_  138  -AVRLCFQVFLEGSQP----------GKFT------VPLH----PVVSDIIYD------------RKA--  172
_XP_050048524.1_  138  -AVRLCFQVFLEGSQP----------GKFT------VPLH----PVVSDIIYD------------RKA--  172
_XP_037272126.1_  138  -AVRLCFQVFLEGSQP----------GKFT------VPLH----PVVSDIIYD------------RKA--  172
_XP_037524244.1_  138  -AVRLCFQVFLEGSQP----------GKFT------VPLH----PIVSDIIYD------------RKA--  172
_XP_037524245.1_  138  -AVRLCFQVFLEGSQP----------GKFT------VPLH----PIVSDIIYD------------RKA--  172
_XP_049512094.1_  138  -AVRLCFQVFLEGSQP----------GKFT------VPLH----PVVSDIIYD------------RKA--  172
```

```
_XP_037524243.1_  138  -AVRLCFQVFLEGSQP----------GKFT------VPLH----PIVSDIIYD------------RKA--  172
_XP_037580160.1_  138  -AVRLCFQVFLEGSQP----------GKFT------VPLH----PVVSDIIYD------------RKA--  172
_XP_037272128.1_  138  -AVRLCFQVFLEGSQP----------GKFT------VPLH----PVVSDIIYD------------RKA--  172
_KAH9380259.1_    138  -AVRLCFQVFLEGSQP----------GKFT------IPLH----PVVSDIIYD------------RKA--  172
_KAI1291715.1_    111  -AIRLCFQVLIMDKES----------NEFL------IPLT----PVVTEPIFD------------KKN--  145
_XP_035209023.1_  135  -SVRLCFQVFITSDNQ----------VR---------PLV----PVVSQPIRN------------KRK--  166
_XP_023332679.1_  144  -AVKLCFHVWYEADVP----------GKCT------KPMK----PVCSDPIYD------------AKA--  178
_XP_046801942.1_  139  -SVRLCFQVFMESDQK----------GRFT------QPLP----PVVSEPIYD------------KKA--  173
_XP_037811120.1_  139  -SVRLCFQVFMESDQK----------GRFT------QPLP----PVVSEPIYD------------KKA--  173
_AFI98401.1_      139  -SVRLCFQVFMESDQK----------GRFT------QPLP----PVVSEPIFD------------KKA--  173
_AFJ54621.1_      139  -SVRLCFQVFMESDQK----------GRFT------QPLP----PVVSEPIFD------------KKA--  173
_KAI8126534.1_    139  -SVRLCFQVFMESDQK----------GRFT------QPLP----PVVSEPIYD------------KKA--  173
_XP_013118266.1_  139  -SVRLCFQVFMESDQK----------GRFT------QPLP----PVVSEPIYD------------KKA--  173
_XP_023293634.2_  139  -SVRLCFQVFMESDQK----------GRFT------QPLP----PVVSEPIYD------------KKA--  173
_XP_037811062.1_  139  -SVRLCFQVFMESDQK----------GRFT------QPLP----PVVSEPIYD------------KKA--  173
_KNC34132.1_      139  -SVRLCFQVFMESDQK----------GRFT------QPLP----PVVSEPIYD------------KKA--  173
_TMW48951.1_      139  -SVRLCFQVFMESDQK----------GRFT------QPLA----PVVSEPIFD------------KKA--  173
_XP_037957363.1_  139  -SVRLCFQVFMESEQK----------GRFT------QPLP----PVVSEPIYD------------KKA--  173
_XP_005175014.1_  139  -SVRLCFQVFMESDQK----------GRFT------QPLP----PVVSEPIYD------------KKA--  173
_XP_037957360.1_  139  -SVRLCFQVFMESEQK----------GRFT------QPLP----PVVSEPIYD------------KKA--  173
_XP_030383330.1_  139  -AVRLCFQVFMESEK-----------GRFT------QALP----PVVSEPIYD------------KKA--  172
_XP_030383328.1_  139  -AVRLCFQVFMESEK-----------GRFT------QALP----PVVSEPIYD------------KKA--  172
_CAH1707987.1_    138  -AVRLCFQVFLEGQQK----------GKFT------VPLK----PIVSDPIYD------------KKS--  172
_TMW54796.1_      139  -SVRLCFQVFMESDQK----------GRFT------QPLA----PVVSEPIFD------------KKA--  173
_XP_026836077.1_  139  -SVRLCFQVFMESEQK----------GRFT------SPLP----PVVSEPIFD------------KKA--  173
_XP_017116803.1_  139  -SVRLCFQVFMESEQK----------GRFT------SPLP----PVVSEPIFD------------KKA--  173
_XP_039480519.1_  139  -SVRLCFQVFMESEQK----------GRFT------SPLP----PVVSEPIFD------------KKA--  173
_XP_043641920.1_  139  -SVRLCFQVFMESEQK----------GRFT------SPLP----PVVSEPIFD------------KKA--  173
_XP_023036748.1_  139  -SVRLCFQVFMESEQK----------GRFT------SPLP----PVVSEPIFD------------KKA--  173
_XP_002090466.1_  139  -SVRLCFQVFMESEQK----------GRFT------SPLP----PVVSEPIFD------------KKA--  173
_XP_034471824.1_  139  -SVRLCFQVFMESEQK----------GRFT------TPLP----PVVSEPIFD------------KKA--  173
_AAT94434.1_      139  -SVRLCFQVFMESEQK----------GRFT------SPLP----PVVSEPIFD------------KKA--  173
_XP_036670985.1_  139  -SVRLCFQVFMESEQK----------GRFT------SPLP----PVVSEPIFD------------KKA--  173
_AAA28479.1_      139  -SVRLCFQVFMESEQK----------GRFT------SPLP----PVVSEPIFD------------KKA--  173
_XP_037708206.1_  139  -SVRLCFQVFMESEQK----------GRFT------SPLP----PVVSEPIFD------------KKA--  173
_XP_002038915.1_  139  -SVRLCFQVFMESEQK----------GRFT------SPLP----PVVSEPIFD------------KKA--  173
_XP_016993636.1_  139  -SVRLCFQVFMESEQK----------GRFT------SPLP----PVVSEPIFD------------KKA--  173
_XP_052852517.1_  139  -SVRLCFQVFMESEQK----------GRFT------SPLP----PVVSEPIFD------------KKA--  173
_XP_016963139.1_  139  -SVRLCFQVFMESEQK----------GRFT------SPLP----PVVSEPIFD------------KKA--  173
```

```
_XP_033156151.1_  139  -SVRLCFQVFMESEQK----------GRFT------SPLP----PVVSEPIFD------------KKA--  173
_XP_001974446.1_  139  -SVRLCFQVFMESEQK----------GRFT------SPLP----PVVSEPIFD------------KKA--  173
_XP_014761886.1_  139  -SVRLCFQVFMESEQK----------GRFT------SPLP----PVVSEPIFD------------KKA--  173
_XP_017107216.2_  139  -SVRLCFQVFMESEQK----------GRFT------SPLP----PVVSEPIFD------------KKA--  173
_XP_017044881.1_  139  -SVRLCFQVFMETE-K----------GRFT------SPLP----PVVSEPIFD------------KKA--  172
_XP_016944798.1_  139  -SVRLCFQVFMESEQK----------GRFT------SPLP----PVVSEPIFD------------KKA--  173
_XP_017116800.1_  139  -SVRLCFQVFMESEQK----------GRFT------SPLP----PVVSEPIFD------------KKA--  173
_XP_016993633.2_  139  -SVRLCFQVFMESEQK----------GRFT------SPLP----PVVSEPIFD------------KKA--  173
_XP_037708198.1_  139  -SVRLCFQVFMESEQK----------GRFT------SPLP----PVVSEPIFD------------KKA--  173
_XP_032583259.1_  139  -SVRLCFQVFMESEQK----------GRFT------SPLP----PVVSEPIFD------------KKA--  173
_AAC35296.1_      139  -SVRLCFQVFMESEQK----------GRFT------SPLP----PVVSEPIFD------------KKA--  173
_NP_001286014.1_  139  -SVRLCFQVFMESEQK----------GRFT------SPLP----PVVSEPIFD------------KKA--  173
_XP_016963136.1_  139  -SVRLCFQVFMESEQK----------GRFT------SPLP----PVVSEPIFD------------KKA--  173
_XP_017063904.2_  139  -SVRLCFQVFMESEQK----------GRFT------SPLP----PVISEPIFD------------KKA--  173
_XP_033156135.1_  139  -SVRLCFQVFMESEQK----------GRFT------SPLP----PVVSEPIFD------------KKA--  173
_XP_052852512.1_  139  -SVRLCFQVFMESEQK----------GRFT------SPLP----PVVSEPIFD------------KKA--  173
_XP_032307758.1_  139  -SVRLCFQVFMESEQK----------GRFT------SPLP----PVVSEPIFD------------KKA--  173
_XP_017107215.2_  139  -SVRLCFQVFMESEQK----------GRFT------SPLP----PVVSEPIFD------------KKA--  173
_AWT86616.1_      138  -AVRLCFQVFLEGKVK----------NQFN------VAIT----PVVSEPIYD------------KKA--  172
_QUF98534.1_      138  -AVRLCFQVFLEGKVK----------NQFN------VAIT----PVVSEPIYD------------KKA--  172
_UNA06287.1_      138  -AVRLCFQVFLEGKVK----------NQFN------VAIT----PVVSEPIYD------------KKA--  172
_AAQ65068.1_      139  -SVRLCFQVFMESEQK----------GRFT------SPLP----PVVSEPIFD------------KKA--  173
_AAQ64916.1_      139  -SVRLCFQVFMESEQK----------GRFT------SPLP----PVVSEPIFD------------KKA--  173
_AAQ64921.1_      139  -SVRLCFQVFMESEQK----------GRFT------SPLP----PVVSEPIFD------------KKA--  173
_AAQ64918.1_      139  -SVRLCFQVFMESEQK----------GRFT------SPLP----PVVSEPIFD------------KKA--  173
_KAH8245459.1_    164  -SVRLCFQVFMESEQK----------GRFT------SPLP----PVVSEPIFD------------KKA--  198
_KAH8283155.1_    161  -SVRLCFQVFMESEQK----------GRFT------SPLP----PVVSEPIFD------------KKA--  195
_KAH8246384.1_    161  -SVRLCFQVFMESEQK----------GRFT------SPLP----PVVSEPIFD------------KKA--  195
_KAH8366418.1_    161  -SVRLCFQVFMESEQK----------GRFT------SPLP----PVVSEPIFD------------KKA--  195
_XP_022195378.2_  138  -AVRLCFQVFLEGKIK----------NQFN------VAIT----PVVSEPIYD------------KKA--  172
_XP_039284931.1_  138  -AVRLCFQVFLEGKIK----------NQFN------VAIT----PVVSEPIYD------------KKA--  172
_ALC40091.1_      161  -SVRLCFQVFMESEQK----------GRFT------SPLP----PVVSEPIFD------------KKA--  195
_XP_036342419.1_  139  -AVRLCFQVFIEGEK-----------GRFT------VPLK----PVVSEPIYD------------KKS--  172
_XP_022903025.1_  138  -AVRLCFQVFLHTQNK----------GKYT-------NLP----PIVSNPIYD------------KKA--  171
_XP_022903017.1_  138  -AVRLCFQVFLHTQNK----------GKYT-------NLP----PIVSNPIYD------------KKA--  171
_XP_036218946.1_  139  -AVRLCFQVFIQGEN-----------GHFT------VPLK----PVVTDPIYD------------KKS--  172
_XP_018790401.1_  139  -SVRLCFQVFIQGES-----------GHFT------VPLK----PVVTDPIYD------------KKS--  172
_KAI9579887.1_    133  -AVRLCFQVYIPPTY---------------------YPLA----PIVSEPIHD------------QKS--  162
_XP_037892537.1_  133  -AVRLCFQVYIPPTY---------------------YPLA----PIVSEPIHD------------QKS--  162
```

```
_XP_018903705.1_  146  -VVRFCFQAFFRTNQW--------------------VTIP----PVVSEPIYD------------KKA--  176
_CAH0395667.1_    146  -VVRFCFQAFFRTNQW--------------------VTIP----PVVSEPIYD------------KKA--  176
_XP_050330165.1_  139  -AVRLCFQVFIQGEN-----------GHFT------VPLK----PVVTEPIYD------------KKS--  172
_XP_039970878.1_  139  -AVRLCFQVFIQGEN-----------GHFT------VPLK----PVVTEPIYD------------KKS--  172
_XP_049305234.1_  139  -AVRLCFQVFIQGEN-----------GHFR------VPLK----PVVTDPIYD------------KKS--  172
_GLH04793.1_      145  -SVRLCFQVFLEGEIK----------GKYT------VPLD----PVVSDPIYD------------KKA--  179
_XP_015835035.1_  138  -VVRLCFQVFLI-DEN----------GRFT------SGLA----PVVSEPIYN------------RKT--  171
_XP_015835036.1_  138  -VVRLCFQVFLI-DEN----------GRFT------SGLA----PVVSEPIYN------------RKT--  171
_XP_039283832.1_  149  -AVRLCFHVFLEGEEE----------GKFN------VPIC----PVVSQPIYD------------KKS--  183
_XP_031788427.1_  137  -SIRLCFQVFLEGKEK----------GKFN------VPLT----PVVSEPIQN------------RRA--  171
_KAJ6646894.1_    140  -CVRLCLQIFLKSDDS----------SKV-------ITVP----PVVSNPITD------------SRY--  173
_XP_026466084.1_   74  -AVRLCFQVILEGDAH----------GKFT------IPLR----PVVSEPIYD------------KKS--  108
_XP_044002069.1_  139  -AIRLCFQIFHYNKVD----------GKVC------LQKLS--NPIVTNPIYN-----------------  172
_XP_046611735.1_  139  -VVRLCFEVYREGTTK----------GLFD------VPFQ----PVVSDPIYN------------KKA--  173
_XP_046417906.1_  139  -VVRLCFEVYREGTTK----------GLFD------VPFQ----PVVSDPIYN------------KKA--  173
_XP_046590309.1_  139  -VVRLCFEVYREGTTK----------GLFD------VPFQ----PVVSDPIYN------------KKA--  173
_XP_046473589.1_  139  -VVRLCFEVYREGTTK----------GLFD------VPFQ----PVVSDPIYN------------KKA--  173
_XP_019892116.1_   50  -SVRLCFQVFMESDQK----------GRFT------QPLP----PVVSEPIYD------------KKA--   84
_XP_005183027.3_   50  -SVRLCFQVFMESDQK----------GRFT------QPLP----PVVSEPIYD------------KKA--   84
_XP_011496598.1_   66  -CIRLCFQVFLEGQES----------GNFN------IPLD----PVVSNPIYD------------KKG--  100
_AAW67216.1_       38  -AIRLCFQVFLEGQQR----------GRFT------EPLQ----PVASDVIYD------------KKA--   72
_AAW67217.1_       38  -AIRLCFQVFLEGQQR----------GRFT------EPLQ----PVASDVIYD------------KKA--   72
_XP_017489788.1_   43  -AVRLCFQVFIEGEK-----------GRFT------VPLK----PVVSEPIYD------------KKS--   76
_BAI67894.1_       42  -SVRLCFQVFMESDQK----------GRFT------QPLA----PVVSEPIFD------------KKA--   76
_XP_054741511.1_  139  -AVRLCFQVFLKGDM-----------GRFT------VPLP----PVVSEPIYD------------KKA--  172
_XP_053961684.1_  139  -AVRLCFQVFLKGDM-----------GRFT------VPLP----PVVSEPIYD------------KKA--  172
_XP_036341313.1_  139  -AVRLCFQVFLKGDM-----------GRFT------VPLP----PVVSEPIYD------------KKA--  172
_XP_036341343.1_  139  -AVRLCFQVFLKGDM-----------GRFT------VPLP----PVVSEPIYD------------KKA--  172
_XP_017471383.1_  139  -AVRLCFQVFLKGDM-----------GRFT------VPLP----PVVSEPIYD------------KKA--  172
_XP_054741498.1_  139  -AVRLCFQVFLKGDM-----------GRFT------VPLP----PVVSEPIYD------------KKA--  172
_XP_053961677.1_  138  -AVRLCFQVFLKGDM-----------GRFT------VPLP----PVVSEPIYD------------KKA--  171
_XP_050320008.1_  139  -SVRLCFQVFLKGDM-----------GRFT------VPLP----PVVSEPIYD------------KKA--  172
_XP_028894490.2_  139  -SVRLCFQVFLKGDM-----------GRFT------VPLP----PVVSEPIYD------------KKA--  172
_XP_050320015.1_  139  -SVRLCFQVFLKGDM-----------GRFT------VPLP----PVVSEPIYD------------KKA--  172
_XP_018787554.1_  139  -SVRLCFQVFLKGDM-----------GRFT------VPLP----PVVSEPIYD------------KKA--  172
_XP_029407438.2_  139  -SVRLCFQVFLKGDM-----------GRFT------VPLP----PVVSEPIYD------------KKA--  172
_XP_036225551.1_  139  -SVRLCFQVFLKGDM-----------GRFT------VPLS----PVVSEPIYD------------KKA--  172
_XP_011178374.2_  139  -SVRLCFQVFLKGDM-----------GRFT------VPLP----PVVSEPIYD------------KKA--  172
_XP_039952593.1_  139  -SVRLCFQVFLKGDM-----------GRFT------VPLP----PVVSEPIYD------------KKA--  172
```

```
_XP_018787542.1_  139  -SVRLCFQVFLKGDM-----------GRFT------VPLP----PVVSEPIYD------------KKA--  172
_XP_011207866.2_  139  -SVRLCFQVFLKGDM-----------GRFT------VPLP----PVVSEPIYD------------KKA--  172
_XP_014086506.1_  139  -SVRLCFQVFLKGDM-----------GRFT------VPLS----PVVSEPIYD------------KKA--  172
_XP_004526638.1_  139  -SVRLCFQVFLKGDL-----------GRFT------VPLP----PVVSEPIYD------------KKA--  172
_XP_017471402.1_  139  -AVRLCFQVFLKGDM-----------GRFT------VPLP----PVVSEPIYD------------KKA--  172
_XP_012157903.1_  139  -SVRLCFQVFLKGDL-----------GRFT------VPLP----PVVSEPIYD------------KKA--  172
_XP_011185661.2_  139  -AVRLCFQVFIQGEN-----------GHFT------VPLK----PVVSEPIYD------------KKS--  172
_XP_050322757.1_  139  -SVRLCFQVFIKGET-----------GHFT------VPLK----PVVTDPIYD------------KKS--  172
_XP_039962922.1_  139  -SVRLCFQVFIKGET-----------GHFT------VPLK----PVVTDPIYD------------KKS--  172
_XP_050322739.1_  139  -SVRLCFQVFIKGET-----------GHFT------VPLK----PVVTDPIYD------------KKS--  172
_XP_050322749.1_  139  -SVRLCFQVFIKGET-----------GHFT------VPLK----PVVTDPIYD------------KKS--  172
_XP_050322724.1_  139  -SVRLCFQVFIKGET-----------GHFT------VPLK----PVVTDPIYD------------KKS--  172
_XP_050322682.1_  139  -SVRLCFQVFIKGET-----------GHFT------VPLK----PVVTDPIYD------------KKS--  172
_CAG9823112.1_    144  -TVRLCFQVFLEGDTP----------RKFS------VPLT----PVVSEPIYD------------KKA--  178
_KAG5873705.1_    139  -TVRLCFQVFLEGDTP----------GKFT------VPLK----PVVSEPIFD------------KKA--  173
_KAH8030364.1_    117  -AVRLCFQVFLEGSQP----------GKFT------VPLH----PVVSDIIYD------------RKA--  151
_XP_023013891.1_  139  -TVRLCFQVFLEGDTP----------GKFT------VPLK----PVVSEPIYD------------KKA--  173
_XP_028130780.2_  145  -VVRLCFQVFLEGDTP----------GKFS------VTVP----PVVSDPIYD------------KKA--  179
_KAG5873704.1_    144  -TVRLCFQVFLEGDTP----------GKFT------VPLK----PVVSEPIFD------------KKA--  178
_CAH1187690.1_    144  -VVRLCFQVFLEGATP----------GRFS------VALP----PVVSEPIYD------------KKA--  178
_CAH1098641.1_    144  -VVRLCFQVFLEGDTP----------GKFS------VPLQ----AVVSEPIYD------------KKA--  178
_XP_023028116.1_  144  -SVRLCFQAFLEGDTP----------GRFD------VPLK----PVISDPIYD------------KKR--  178
_XP_031630227.1_  140  -CVRLCFEVLL--SKT----------GSYSENRNDWKSLG----HVISKPILD------------KKS--  178
_KAG5861047.1_     13  -TVRLCFQVFLEGDTP----------GKFT------VPLK----PVVSEPIFD------------KKA--   47
_KAF2880990.1_    139  -AVRLCFQVFLEGEQR----------GKFT------VPLT----PVVSEPIYD------------KKA--  173
_XP_018326789.1_  139  -AVRLCFQVFLEGER-----------GRFT------IPLT----PVVSESIYD------------KKA--  172
_XP_018326788.1_  139  -AVRLCFQVFLEGER-----------GRFT------IPLT----PVVSESIYD------------KKA--  172
_XP_031339184.1_  139  -SVRLCFQVFLEGDR-----------GKFT------VPLT----PVVSDPIYD------------KKA--  172
_XP_045476069.1_  139  -SVRLCFQVFLEGEKK----------GKFT------VPLP----PVVSEPVYD------------KKT--  173
_XP_044734149.1_  139  -AVRLCFQVFLSTDGK-----------SFT------CPLP----PVVSEPIFD------------KKA--  172
_XP_044734150.1_  139  -AVRLCFQVFLSTDGK-----------SFT------CPLP----PVVSEPIFD------------KKA--  172
_XP_044734148.1_  139  -AVRLCFQVFLSTDGK-----------SFT------CPLP----PVVSEPIFD------------KKA--  172
_XP_044760528.1_  139  -SVRLCFQVFLEGDKK----------GKFT------VPLT----PVVSDPVFD------------KKT--  173
_XP_044747918.1_  139  -AVRLCFQVFLEGDKK----------GKFT------VPLP----PVVSEPILD------------KKA--  173
_XP_044747917.1_  139  -AVRLCFQVFLEGDKK----------GKFT------VPLP----PVVSEPILD------------KKA--  173
_XP_045481017.1_  139  -AVRLCFQVFLEGDKK----------GKFT------VPLS----PVVSEPILD------------KKA--  173
_XP_045481018.1_  139  -AVRLCFQVFLEGDKK----------GKFT------VPLS----PVVSEPILD------------KKA--  173
_KAF5302274.1_    139  -AVRLCFQVFLEGEQR----------GKFT------VPLT----PVVSEPIYD------------KKA--  173
_KAF5308883.1_    139  -AVRLCFQVFLEGEQR----------GKFT------VPLT----PVISEPIYD------------KKA--  173
```

```
_KRT79621.1_       138  -AVRLCFQVFLQGEQR----------GRY-------IDLP----PVVSTPIYD------------KKA--  171
_BAD20728.1_       138  -AVRLCFQVFLQGEQR----------GRY-------TDLP----PVVSTPIYD------------KKA--  171
_KAF2880988.1_     138  -AVRLCFQAFLEGEQK----------GKFT------VPLT----PVVSEPIYD------------KKA--  172
_CAH1403258.1_     131  -SVRLCFQAFLEGPQR----------GKFT------RALP----PVVSDPIYD------------KK---  164
_CAD6992094.1_     139  -AVRLCFQVFIE-DSS----------GRFT------IPLK----PVVSEPIYD------------SRA--  172
_XP_004537184.1_   139  -AVRLCFQVFIE-DSS----------GRFT------IPLK----PVVSEPIYD------------SRA--  172
_XP_018572644.1_   139  -AVRLCFQVFLEGNTP----------GKFS------VPLP----AVVSEPIYD------------KKA--  173
_RZB54393.1__      139  -AVRLCFQVFLEGDRR----------GKFT------VPLQ----PVVSEPIYD------------KKA--  173
_XP_044255725.1_   139  -AVRLCFQVFLEGERR----------GKFT------VPLT----PVVSEPIYD------------KKA--  173
_EFA02850.1_       139  -AVRLCFQVFLEGERR----------GKFT------VPLT----PVVSEPIYD------------KKA--  173
_NP_001034507.1_   139  -AVRLCFQVFLEGERR----------GKFT------VPLT----PVVSEPIYD------------KKA--  173
_CAH1370311.1_     139  -AVRLCFQVFLEGERR----------GKFT------VPLT----PVVSEPIYD------------KKA--  173
_KAJ3630692.1_     139  -AVRLCFQVFLEGERR----------GKFT------VPLT----PVVSEPIYD------------KKA--  173
_XP_044255724.1_   139  -AVRLCFQVFLEGERR----------GKFT------VPLT----PVVSEPIYD------------KKA--  173
_KAH0811528.1_     139  -AVRLCFQVFLEGERR----------GKFT------VPLT----PVVSEPIYD------------KKA--  173
_CAH7716406.1_     139  -SVRLCFQVFLP-DEN----------NKYN------TALK----PVVSEPIYD------------KKA--  172
_CAH7716405.1_     139  -SVRLCFQVFLP-DEN----------NKYN------TALK----PVVSEPIYD------------KKA--  172
_CAH7716404.1_     139  -SVRLCFQVFLP-DEN----------NKYN------TALK----PVVSEPIYD------------KKA--  172
_XP_028130776.2_   139  -VVRLCFQVFLEGDTP----------GKFS------VTVP----PVVSDPIYD------------KKA--  173
_CAH1985684.1_     139  -SVRLCFQVFLP-DEN----------NKFN------TALK----PVVSEPIYD------------KKA--  172
_XP_017781152.1_   139  -SVRLCFQVFTEGDKP----------GKFT------VPLQ----PVVSDPIYD------------KKS--  173
_XP_017781151.1_   139  -SVRLCFQVFTEGDKP----------GKFT------VPLQ----PVVSDPIYD------------KKS--  173
_XP_048522053.1_   138  -AVRLCFQTYLEED------------GKFI------IPLR----AVVSDVIYD------------KKA--  170
_XP_050303070.1_   143  -AVRLCFQVFYLNPENQ--------NGQISRQ-VKYNALR----PVVSNIIYD------------KKA--  184
_CAG9762924.1_     139  -AVRLCFQVFLENERD----------GKFT------TPLR----PVVSDVIYD------------KKA--  173
_CAH0560654.1_     140  -IVRLCFQVFLVEDGK---------NDKYS------VPLA----PVVSEPIYD------------KKS--  175
_CAH0560655.1_     140  -IVRLCFQVFLVEDGK---------NDKYS------VPLA----PVVSEPIYD------------KKS--  175
_XP_018572643.1_   138  -AVRLCFQVFLEGNTP----------GKFS------VPLP----AVVSEPIYD------------KKA--  172
_XP_030763656.1_   139  -AVRLCFQVFIEGDTP----------GKYS------EPLK----PVVSDIIYD------------KKA--  173
_XP_030763654.1_   139  -AVRLCFQVFIEGDTP----------GKYS------EPLK----PVVSDIIYD------------KKA--  173
_XP_018577945.1_   139  -SVRLCFQVFLEGNTP----------GKFS------LPLP----AVVSDPIFD------------KKA--  173
_KAF7283276.1_     139  -AVRLCFQVFLQGDCT----------NKYN------VPLD----PVVSDIIYD------------KKA--  173
_KAF7283275.1_     139  -AVRLCFQVFLQGDCT----------NKYN------VPLD----PVVSDIIYD------------KKA--  173
_XP_030763617.1_   139  -AVRLCFQVLIEGDTP----------GKYR------YPLT----PVVSNIIYD------------KKA--  173
_XP_030763619.1_   142  -AVRLCFQVILEGET-----------GKYT------EPLE----PVVSDVIND------------KRI--  175
_VEN60240.1_        50  -SVRLCFQVFLP-DEN----------NKYN------TALK----PVVSEPIYD------------KKA--   83
_KOB78236.1_       126  -SVRLAFELLPGFQDD---------EGRGQ------LPV-----VTVSNVISG------------TK---  159
_ERL85515.1_        81  -AVRLCFQTYLEED------------GKFI------IPLR----AVVSDVIYD------------KKA--  113
_ENN78766.1_        76  -AVRLCFQTYLEED------------GKFI------IPLR----AVVSDVIYD------------KKA--  108
```

```
_KAH1019371.1_     76  -AVRLCFQTYLEED------------GKFI------IPLR----AVVSDVIYD------------KKA--  108
_KAI9565420.1_    139  -AVRLCFQVFLEGQK-----------GKFT------LALK----PVVSEAIYD------------KKA--  172
_XP_045024905.1_  139  -AVRLCFQVFLEGQK-----------GKFT------LALK----PVVSEAIYD------------KKA--  172
_XP_046447094.1_  139  -AVRLCFQVFLEGQK-----------GKFS------LALK----PVVSEAIYD------------KKA--  172
_KZS08518.1_      139  -AVRLCFQVFLEGQK-----------GKFT------LALK----PVVSEAIYD------------KKA--  172
_XP_046636307.1_  139  -AVRLCFQVFLEGQK-----------GKFS------LALK----PVVSEAIYD------------KKA--  172
_EFX79013.1_      139  -AVRLCFQVFLEGQK-----------GKFS------LALK----PVVSEAIYD------------KKA--  172
_XP_032776606.2_  139  -AVRLCFQVFLEGQK-----------GKFT------LALK----PVVSEAIYD------------KKA--  172
_XP_046447093.1_  139  -AVRLCFQVFLEGQK-----------GKFS------LALK----PVVSEAIYD------------KKA--  172
_XP_046636306.1_  139  -AVRLCFQVFLEGQK-----------GKFS------LALK----PVVSEAIYD------------KKA--  172
_CAH0099799.1_    129  -AVRLCFQVFLEGQK-----------GKFT------MALK----PVVSEAIYD------------KKA--  162
_KAJ1531033.1_    138  -SVRLCFQVFLHDNT----------------------PLK----PVVSQPIFN------------RKL--  166
_ACJ36225.1_      139  -ALRLCFQVFLEGSEK----------GKFT------FPLK----PQVSDPIYD------------KKA--  173
_ACZ98167.1_      139  -ALRLCFQVFLEGSEK----------GKFT------FPLK----PQVSDPIYD------------KKA--  173
_AME17867.1_      139  -ALRLCFQVFLEGSEK----------GKFT------FPLK----PQVSDPIYD------------KKA--  173
_XP_042868409.1_  139  -ALRLCFQVFLEGSEK----------GKFT------FPLK----PQVSDPIYD------------KKA--  173
_XP_045102711.1_  139  -ALRLCFQVFLEGPEK----------GKFT------FPLK----PVVSHAIYD------------KKA--  173
_XP_047487558.1_  139  -ALRLCFQVFLEGSEK----------GKFT------FPLK----PQVSDPIYD------------KKA--  173
_QBJ05651.1_      139  -ALRLCFQVFLEGPEK----------GKFT------FPLK----PVVSHAIYD------------KKA--  173
_AZK36046.1_      139  -ALRLCFQVFLEGPEK----------GKFT------FPLK----PVVSHAIYD------------KKA--  173
_XP_045581920.1_  139  -ALRLCFQVFLEGPDK----------GKFT------FPLK----PVVSDAIYD------------KKA--  173
_XP_050704598.1_  139  -ALRLCFQVFLEGPEK----------GKFT------FPLK----AVVSHPIYD------------KKA--  173
_XP_027237995.1_  139  -ALRLCFQVFLEGSEK----------GKFT------FPLK----PQVSDPIYD------------KKA--  173
_AXB88326.1_      139  -ALRLCFQVFLKGPEK----------GKFT------FPLK----PVVSHAIYD------------KKA--  173
_XP_045102712.1_  139  -ALRLCFQVFLEGPEK----------GKFT------FPLK----PVVSHAIYD------------KKA--  173
_MPC17461.1_      139  -ALRLCFQVFLEGPEK----------GKFT------FPLK----PVVSHAIYD------------KKA--  173
_XP_027237994.1_  139  -ALRLCFQVFLEGSEK----------GKFT------FPLK----PQVSDPIYD------------KKA--  173
_XP_027237996.1_  139  -ALRLCFQVFLEGSEK----------GKFT------FPLK----PQVSDPIYD------------KKA--  173
_XP_045102715.1_  139  -ALRLCFQVFLEGPEK----------GKFT------FPLK----PVVSHAIYD------------KKA--  173
_XP_053627990.1_  139  -ALRLCFQVFLEGPEK----------GKFT------YPLK----AVVSDAIFD------------KKA--  173
_XP_053627989.1_  139  -ALRLCFQVFLEGPEK----------GKFT------YPLK----AVVSDAIFD------------KKA--  173
_XP_053627983.1_  139  -ALRLCFQVFLEGPEK----------GKFT------YPLK----AVVSDAIFD------------KKA--  173
_KAB7495058.1_    139  -AVRLCFQVFLNSSTE----------GRFT------FPLK----PVVSDPIYD------------KKA--  173
_KAF2353654.1_    139  -ALRLCFQVFLEGQTK----------GKFT------FPLK----PVVSEAIYD------------KKA--  173
_ANH22235.1_      139  -SLRLCFQVFLEGSEK----------GKFT------FPLK----PVVSDPIYD------------KKA--  173
_CAD7275741.1_    138  -SLRLCFQVFIEGEQK----------NKYV------VPLK----PVVSEPIYD------------RKA--  172
_XP_042243403.1_  139  -ALRLCFQVFLEGPEK----------GKFT------FPLK----PVVSEPIYD------------KKA--  173
_QTC09942.1_      139  -ALRLCFQVFLEGSEK----------GKFT------FPLK----PQVSDPIYD------------KKA--  173
_XP_018023738.1_  118  -ALRLCFQVFLEGHTK----------GKFT------FPLK----PTVSEPIYD------------KKA--  152
```

```
_XP_019877552.1_   140   -TVRLCFQVFLENENQ---------TGKYT------VQLD----PKVSDPICD------------KKA--   175
_XP_049825875.1_   138   -TVRLCFQVFIENENH---------KGKYN------IQLD----PKVSTPICD------------KKA--   173
_CAD7649074.1_     139   -VVRLCFQVFIEGPEK----------DKFT------VQLP----PIVSDPIYD------------KKA--   173
_CAD7630012.1_     139   -VVRLCFQVFIEGPEK----------DKFT------VQLP----PIVSDPIYD------------KKA--   173
_XP_054165343.1_   140   -VVRLCFQVFIEGPER----------DKFT------VQLP----PIVSDFIYD------------KKA--   174
_XP_044254219.1_   138   -VVRLCFQVFLI-DEN----------RRFT------PVSP----PVVSDPIYN------------RKT--   171
_XP_053201827.1_   139   -VVRLCFQVFVEGPEK----------DKFT------IPLR----PVVSDPIYD------------KKA--   173
_XP_015786990.1_   139   -VVRLCFQVFVEGSEK----------DKFT------VPLK----PIVSDPIYD------------KKA--   173
_KAI1295507.1_     139   -VVRLCFQVFIEGPEK----------DKFT------VPLP----PIVCEPIYD------------KKA--   173
_RWS07527.1_       139   -VVRLCFQVFIEGPEK----------EKFT------VPLM----PVVSDPIYD------------KKA--   173
_RWS29859.1_       139   -VVRLCFQVFVEGPEK----------DKFT------VPLQ----PTVSDPIYD------------KKA--   173
_XP_053201825.1_   139   -VVRLCFQVFVEGPEK----------DKFT------IPLR----PVVSDPIYD------------KTLIG   175
_XP_054713467.1_   141   -VVRLCFQIYIKGQT---------------------DIYA----RTVSEPIYN------------KDV--   170
_XP_046915158.1_   140   -CLRLCFQVFVEGPEK----------GKFT------VQLP----PVVSDAIYD------------KKA--   174
_KAJ6217218.1_     140   -VVRLCFQVFIEGPEK----------GKFT------VQLP----PIVSEAIYD------------KKA--   174
_KAI2801352.1_     140   -VVRLCFQVFIEGPEK----------GKFT------VQLP----PIVSEAIYD------------KKA--   174
_KAH9406651.1_     140   -VVRLCFQVFIEGPEK----------GKFT------VQLP----PIVSEPIYD------------KKA--   174
_XP_017483538.1_   140   -VVRLCFQVFIEGPEK----------GKFT------VQLP----PIVSEPIYD------------KKA--   174
_KPM10359.1_       140   -CLRLCFQVFIEGPEK----------GKFA------VQLP----PVISDAIYD------------KKA--   174
_UXI17582.1_       140   -CLRLCFQVFIEGPEK----------GKFA------VQLP----PVISDAIYD------------KKA--   174
_KAF7488016.1_     140   -CLRLCFQVFIEGPEK----------GKFA------VQLP----PVISDAIYD------------KKA--   174
_KAH9423377.1_     140   -CLRLCFQVFIEGPEK----------GKFT------VQLP----PVVSDAIYD------------KKA--   174
_OTF73464.1_       140   -CLRLCFQVFIEGPEK----------GKFT------VQLP----PVVSDAIYD------------KKA--   174
_XP_037072056.1_   139   -TVRLCFQVFVEGREP----------GKFV------VPLR----PVVSDPIYD------------KKS--   173
_XP_037083492.1_   139   -TVRLCFQVFVEGRER----------GKYV------VPLK----PVVSDAIYD------------KKS--   173
_XP_037083508.1_   139   -TVRLCFQVFVEGRER----------GKYV------VPLK----PVVSDAIYD------------KKS--   173
_XP_043215018.1_   139   -AVRLCFQVFVEGHKR----------DQYV------VPLK----PVISDAIYD------------KKS--   173
_KAF0314765.1_     139   -AVRLCFQVFVEGHKR----------DQYV------VPLK----PVISDAIYD------------KKS--   173
_XP_043215020.1_   139   -AVRLCFQVFVEGHKR----------DQYV------VPLK----PVISDAIYD------------KKS--   173
_XP_043189654.1_   139   -TVRLCFQVFVEGHEP----------GKFV------VPLT----PVVSDPIFD------------KKS--   173
_KAF0293344.1_     125   -TVRLCFQVFVEGHEP----------GKFV------VPLT----PVVSDPIFD------------KKS--   159
_XP_011496609.1_   140   -SLRLCFQVYLEGNEK----------GNFN------LALK----PVVSNPIYD------------KKR--   174
_AGS12619.1_       139   -CVRLCFQVFLEGSEK----------GAFT------FALK----PVVSDPIFD------------KKA--   173
_TRY78048.1_       139   -CVRLCFQVFLEGSEK----------GAFT------FALK----PVVSDPIFD------------KKA--   173
_XP_040576307.1_   139   -SVRLCFQVFLEGSEK----------GSFT------FALK----PVVSEPIYD------------KKA--   173
_CAB4056363.1_     139   -SVRLCFQVFLEGSEK----------GSFT------FALK----PVVSEPIYD------------KKA--   173
_ALF84898.1_       139   -SLRLCFQVFLEGAER----------GAFT------FALK----PVVSDPIYD------------KKA--   173
_XP_023349538.1_   135   -CVRLAFQVFLEGNDA----------GAFT------FALK----PVVSDPIYD------------KKS--   169
_XP_004522582.1_   139   -CVRLCFQVFLKDEM-----------QHYT------IPLP----PVVSEPIYN------------KKS--   172
```

```
_CAD7013622.1_     139  -CVRLCFQVFLKDEM-----------QHYT------MPLP----PVVSEPIYN------------KKS--  172
_XP_042902217.1_   139  -VLRLCFQVFLKDSE-----------NRYS------IPIP----SAVSDPIFD------------KKA--  172
_XP_042902218.1_   139  -VLRLCFQVFLKDSE-----------NRYS------IPIP----SAVSDPIFD------------KKA--  172
_XP_035215470.1_   139  -VLRLCFQVFIRGPD-----------NSYS------LPLQ----PVVSEPIYD------------KKA--  172
_GBN14612.1_       138  -ILRLCFQVFVRGPD-----------NSFS------LPLP----PVVSEPIYD------------KKA--  171
_KAF8795965.1_     139  -VLRLCFQVFIRGPD-----------NAFS------LPLS----PVVSEPIYD------------KKA--  172
_KAF8795964.1_     139  -VLRLCFQVFIRGPD-----------NAFS------LPLS----PVVSEPIYD------------KKA--  172
_GFY66827.1_       139  -VLRLCFQVFIRGPD-----------NSFS------MPLQ----AIVSEPIYD------------KKA--  172
_GFW66965.1_       139  -VLRLCFQVFIRGPD-----------NSFS------MPLQ----AIVSEPIYD------------KKA--  172
_GFQ85823.1_       139  -VLRLCFQVFIRGPD-----------NSFS------MPLQ----AIVSEPIYD------------KKA--  172
_GFT35953.1_       139  -VLRLCFQVFIRGPD-----------NSFS------LPLQ----AIVSEPIYD------------KKA--  172
_XP_054714028.1_   139  -VVRLCFQVFIRGSD-----------NAFS------FPLP----PVVSDPIYD------------KKA--  172
_KAG8194583.1_     138  -VLRLCFQVFIRGPD-----------NSFS------SPLP----PIVSEPIFD------------KKA--  171
_GIY09899.1_       138  -VLRLCFQVFIRGPD-----------NSYS------LPLP----AIVSEPIFD------------KKA--  171
_GIY93593.1_       138  -VLRLCFQVFIRGPD-----------NSYS------LPLQ----AIVSEPIFD------------KKA--  171
_XP_042902221.1_   151  -ILRLCFQVLLKDSE-----------GKYK------IRVP----PVVSEPIYD------------RKS--  184
_XP_046962867.1_   141  -AVRLCFQVFIHDERT----------GKIR------LSLP----PVVSDVIYD------------KKA--  175
_XP_047530540.1_   141  -AVRLCFQVFIHDERT----------GKIR------LSLP----PVVSDVIYD------------KKA--  175
_XP_026486466.1_   141  -AVRLCFQVFIHDERT----------GKIR------LSLP----PVVSDVIYD------------KKA--  175
_XP_047530539.1_   141  -AVRLCFQVFIHDERT----------GKIR------LSLP----PVVSDVIYD------------KKA--  175
_XP_047530538.1_   141  -AVRLCFQVFIHDERT----------GKIR------LSLP----PVVSDVIYD------------KKA--  175
_XP_047530537.1_   141  -AVRLCFQVFIHDERT----------GKIR------LSLP----PVVSDVIYD------------KKA--  175
_XP_026486464.1_   141  -AVRLCFQVFIHDERT----------GKIR------LSLP----PVVSDVIYD------------KKA--  175
_XP_047530541.1_   141  -AVRLCFQVFIHDERT----------GKIR------LSLP----PVVSDVIYD------------KKA--  175
_XP_050347286.1_   141  -AVRLCFQVFIHDERS----------GKIR------LSLP----PVVSDVIYD------------KKA--  175
_XP_050347283.1_   141  -AVRLCFQVFIHDERS----------GKIR------LSLP----PVVSDVIYD------------KKA--  175
_XP_032515351.1_   142  -TVRLCFQVFLPDERT----------GKIK------HSLP----PVVSDVIYD------------KKA--  176
_OWR46016.1_       142  -TVRLCFQVFLPDERT----------GKIK------HSLP----PVVSDVIYD------------KKA--  176
_XP_032515346.1_   142  -TVRLCFQVFLPDERT----------GKIK------HSLP----PVVSDVIYD------------KKA--  176
_CAG9579094.1_     142  -TVRLCFQVFLPDERT----------GKIK------HSLP----PVVSDVIYD------------KKA--  176
_CAG9579091.1_     142  -TVRLCFQVFLPDERT----------GKIK------HSLP----PVVSDVIYD------------KKA--  176
_XP_050668638.1_   140  -AVRLCFQVFLPDERT----------GKIR------QALP----PVVSDIIYD------------KKA--  174
_XP_050668632.1_   140  -AVRLCFQVFLPDERT----------GKIR------QALP----PVVSDIIYD------------KKA--  174
_XP_045495329.1_   140  -AVRLCFQVFIQDERT----------GKVR------HSLP----PVVSDIIYD------------KKA--  174
_XP_038214802.1_   140  -AVRLCFQVFIQDERT----------GKVR------HSLP----PVVSDIIYD------------KKA--  174
_CAH0714714.1_     141  -AVRLCFQVFIPDERT----------GKIR------QSLQ----PVVSDVIYD------------KKA--  175
_CAH0714713.1_     141  -AVRLCFQVFIPDERT----------GKIR------QSLQ----PVVSDVIYD------------KKA--  175
_CAH4029256.1_     140  -AVRLCFQVFLPDEAT----------GKIR------HSLQ----PVVSDVIFD------------KKA--  174
_XP_047513081.1_   140  -AVRLCFQVFLPDEAT----------GKIR------HSLQ----PVVSDVIFD------------KKA--  174
```

```
_XP_045529569.1_  140  -AVRLCFQVFLPDEAT----------GKIR------HSLQ----PVVSDVIFD------------KKA--  174
_XP_045487500.1_  140  -AVRLCFQVFLPDEAT----------GKIR------HSLQ----PVVSDVIFD------------KKA--  174
_XP_047513077.1_  140  -AVRLCFQVFLPDEAT----------GKIR------HSLQ----PVVSDVIFD------------KKA--  174
_CAH4029257.1_    140  -AVRLCFQVFLPDEAT----------GKIR------HSLQ----PVVSDVIFD------------KKA--  174
_XP_045487498.1_  140  -AVRLCFQVFLPDEAT----------GKIR------HSLQ----PVVSDVIFD------------KKA--  174
_CAF4952989.1_    140  -AVRLCFQVFLPDEAT----------GKIR------HSLQ----PVVSDVIFD------------KKA--  174
_XP_045447470.1_  141  -AVRLCFQVFIHDERT----------GKIR------LSLP----PVVSDVIYD------------KKA--  175
_CAH2108688.1_     80  -AVRLCFQVFIHDERK----------NKIR------LSLP----PVVSDVIYD------------KKA--  114
_CAH0714724.1_    140  -AVRLCFLVSIYDKRT----------GEK-------TNLA----PVVSNVIYD------------GKA--  173
_XP_034827218.1_  141  -AVRLCFQVFIQDERT----------NRVR------HSLP----PVVSDIIYD------------KKA--  175
_XP_045768234.1_  141  -AVRLCFQVFIQDERT----------NRVR------HSLP----PVVSDIIYD------------KKA--  175
_XP_034827214.1_  141  -AVRLCFQVFIQDERT----------NRVR------HSLP----PVVSDIIYD------------KKA--  175
_XP_045768230.1_  141  -AVRLCFQVFIQDERT----------NRVR------HSLP----PVVSDIIYD------------KKA--  175
_XP_039751073.1_  141  -AVRLCFQVFIQDERT----------NRVR------HSLP----PVVSDIIYD------------KKA--  175
_XP_023942270.1_  141  -AVRLCFQVFIQDERT----------NRVR------HSLP----PVVSDIIYD------------KKA--  175
_XP_023942268.1_  141  -AVRLCFQVFIQDERT----------NRVR------HSLP----PVVSDIIYD------------KKA--  175
_XP_039751072.1_  141  -AVRLCFQVFIQDERT----------NRVR------HSLP----PVVSDIIYD------------KKA--  175
_XP_023942266.1_  141  -AVRLCFQVFIQDERT----------NRVR------HSLP----PVVSDIIYD------------KKA--  175
_CAH2240541.1_    128  -AVRLCFQVFIQDERT----------NRVR------HSLP----PVVSDIIYD------------KKA--  162
_XP_041985045.1_  141  -AVRLCFQVFIPDERT----------GRIR------RSLQ----PVVSDVIYD------------KKA--  175
_XP_041985024.1_  141  -AVRLCFQVFIPDERT----------GRIR------RSLQ----PVVSDVIYD------------KKA--  175
_XP_049868286.1_  141  -AVRLCFQVFLPDEA-----------GKVR------HSLA----PVVSDVIYD------------KKA--  174
_XP_026314794.1_  141  -AVRLCFQVFLPDEA-----------GKVR------HSLE----PVVSEIIYD------------KKA--  174
_XP_047994271.1_  140  -AVRLCFQVYIPGGNT----------SKIK------QALA----PVVSDVIYD------------KKA--  174
_KAJ2950301.1_    141  -AVRLCFQVFII--------------GRQR------HSLP----PVVSEVIYD------------KKA--  171
_XP_031784347.2_  139  -AVRLCFQVFL-GNN-----------GNFN------IPLD----PVVSVPIYD------------HK---  170
_KAG7305875.1_    139  -AVRLCFQVFLPDERS----------GKIR------HALP----PVVSDVIYD------------KKA--  173
_WDD23886.1_      139  -AVRLCFQVFLPDERS----------GKIR------HALP----PVVSDVIYD------------KKA--  173
_XP_048480424.1_  139  -AVRLCFQVFLPDERS----------GKIR------HALP----PVVSDVIYD------------KKA--  173
_XP_048480423.1_  139  -AVRLCFQVFLPDERS----------GKIR------HALP----PVVSDVIYD------------KKA--  173
_XP_048480422.1_  139  -AVRLCFQVFLPDERS----------GKIR------HALP----PVVSDVIYD------------KKA--  173
_XP_048480421.1_  139  -AVRLCFQVFLPDERS----------GKIR------HALP----PVVSDVIYD------------KKA--  173
_KAG7305876.1_    139  -AVRLCFQVFLPDERS----------GKIR------HALP----PVVSDVIYD------------KKA--  173
_CAG9129242.1_    139  -AVRLCFQVFLPDERS----------GKIR------HALP----PVVSDVIYD------------KKA--  173
_XP_048480420.1_  139  -AVRLCFQVFLPDERS----------GKIR------HALP----PVVSDVIYD------------KKA--  173
_GBP53004.1_       15  -AVRLCFQVFL--EQG----------GKIR------HALP----PVVSDIIYD------------KKA--   47
_XP_045534737.1_  141  -AVRLCFQVFVTGER-----------GKVR------LALA----PVVSDVIYD------------KKA--  174
_XP_013180060.1_  141  -AVRLCFQVFVTGER-----------GKVR------LALA----PVVSDVIYD------------KKA--  174
_XP_013180043.1_  141  -AVRLCFQVFVTGER-----------GKVR------LALA----PVVSDVIYD------------KKA--  174
```

```
_XP_013180068.1_  141  -AVRLCFQVFVTGER-----------GKVR------LALA----PVVSDVIYD------------KKA--  174
_XP_045534736.1_  141  -AVRLCFQVFVTGER-----------GKVR------LALA----PVVSDVIYD------------KKA--  174
_KPJ06840.1_      141  -AVRLCFQVFVTGER-----------GKVR------LALA----PVVSDIIYD------------KKA--  174
_KPJ05518.1_      141  -AVRLCFQVFVTGER-----------GKVR------LALA----PVVSDVIYD------------KKA--  174
_CAG4934519.1_    141  -AVRLCFQVFLTGKG-----------GKVR------LALP----PAVSDVIFD------------KKA--  174
_XP_013149112.1_  141  -AVRLCFQVFVTGER-----------GKVR------LALA----PAVSDVIYD------------KKA--  174
_CAH2077165.1_    153  -ALRLCFQVFVTGEH-----------GKVR------LALA----PLVSDVIYD------------KKA--  186
_CAH2077167.1_    153  -ALRLCFQVFVTGEH-----------GKVR------LALA----PLVSDVIYD------------KKA--  186
_XP_037051415.1_  134  -GVRLCFQVFIRSGDK----------LKPI------YPVY----PIVSDIIRG------------KDS--  168
_XP_037051414.1_  135  -AVRLCFQVFIRSGDK----------YK---------PVY----PIVSDIVRS------------KDS--  166
_XP_022815079.1_  141  -AVRLCFQVFLPDET-----------GRLR------RPLT----PVVSDVIYD------------KKA--  174
_CAB3516522.1_    141  -AVRLCFQVFLPDET-----------GRLR------RPLT----PVVSDVIYD------------KKA--  174
_XP_050562120.1_  141  -AVRLCFQVFLPDET-----------GRLR------RPLT----PVVSDVIYD------------KKA--  174
_XP_050562121.1_  141  -AVRLCFQVFLPDET-----------GRLR------RPLT----PVVSDVIYD------------KKA--  174
_QHD56218.1_      141  -AVRLCFQVFLPDET-----------GRLR------RPLT----PVVSDVIYD------------KKA--  174
_AIA24466.1_      141  -AVRLCFQVFLPDET-----------GRLR------RPLT----PVVSDVIYD------------KKA--  174
_XP_022815076.1_  141  -AVRLCFQVFLPDET-----------GRLR------RPLT----PVVSDVIYD------------KKA--  174
_QHD56217.1_      141  -AVRLCFQVFLPDET-----------GRLR------RPLT----PVVSDVIYD------------KKA--  174
_XP_050562118.1_  141  -AVRLCFQVFLPDET-----------GRLR------RPLT----PVVSDVIYD------------KKA--  174
_AIA24467.1_      141  -AVRLCFQVFLPDET-----------GRLR------RPLT----PVVSDVIYD------------KKA--  174
_XP_047019808.1_  141  -AVRLCFQVFLPDDS-----------GRLR------RPLA----PVVSDVIYD------------KKA--  174
_AEO51736.1_      141  -AVRLCFQVFLPDDS-----------GRLR------RPLA----PVVSDVIYD------------KKA--  174
_XP_049696680.1_  141  -AVRLCFQVFLPDDS-----------GRLR------RPLA----PVVSDVIYD------------KKA--  174
_XP_049696682.1_  141  -AVRLCFQVFLPDDS-----------GRLR------RPLA----PVVSDVIYD------------KKA--  174
_XP_049696676.1_  141  -AVRLCFQVFLPDDS-----------GRLR------RPLA----PVVSDVIYD------------KKA--  174
_XP_026744931.1_  141  -AVRLCFQVFLPDES-----------GRLR------RPLA----PVVSDVIYD------------KKA--  174
_XP_026732895.1_  141  -AVRLCFQVFLPDES-----------GRLR------RPLA----PVVSDVIYD------------KKA--  174
_CAH0581650.1_    141  -AVRLCFQVFLPDES-----------GRLR------RPLA----PVVSDVIYD------------KKA--  174
_KAH9640014.1_    186  -AVRLCFQVFLPDET-----------GRLR------RPLT----PVVSDVIYD------------KKA--  219
_PZC78315.1_       15  -AVRLCFQVFLPDDS-----------GRLR------RPLA----PVVSDVIYD------------KKA--   48
_KAF9795796.1_     15  -AVRLCFQVFLPDET-----------GRLR------RPLT----PVVSDVIYD------------KKA--   48
_AEC52926.1_      141  -AVRLGFQVFVPDAT-----------GKMR------RPLA----PVASEVIYD------------KKA--  174
_KAH7940301.1_    138  -AVRLCFQVFLEGSQP----------GKFT------VPLH----PIVSDIIYD-----------RRFN--  173
_ADK39025.1_      140  -AVRLAFQVFLPHSS-----------GKMR------RTLA----PVVSDVIYD------------KKA--  173
_XP_037298148.1_  140  -AVRLAFQVFLPHSS-----------GKMR------RTLA----PVVSDVIYD------------KKA--  173
_RVE50742.1_      141  -AVRLCFQVFLTDES-----------YKVQ------RALQ----PVVSDVVYD------------KKA--  174
_CAH0692665.1_    141  -AVRLCFQVFLTDES-----------YKVQ------RALQ----PVVSDVVYD------------KKA--  174
_XP_028166351.1_  140  -AVRLCFQVFLTDDA-----------YKVQ------RALP----PVVSDVIYD------------KKA--  173
_XP_028166291.1_  140  -AVRLCFQVFLTDDA-----------YKVQ------RALP----PIVSDVIYD------------KKA--  173
```

```
_CAB3235314.1_     141  -AVRLCFQVFLPDAT-----------GRCR------RPLT----PVVSDIIYD------------KKA--  174
_KAJ0175867.1_     142  -AVRLAFQVFVAKPD-----------GRMR------WQLP----TVASDIIYD------------KKA--  175
_XP_028031027.1_   141  -AVRLAFQVFVPDGA-----------GRMR------RPLP----AVVSDIIYD------------KKA--  174
_NP_001166296.1_   141  -AVRLAFQVFVPDGA-----------GRMR------RPLP----TVVSDIIYD------------KKA--  174
_XP_037870194.1_   141  -AVRLAFQVFVPDGA-----------GRMR------RPLP----AVVSDIIYD------------KKA--  174
_XP_037870193.1_   141  -AVRLAFQVFVPDGA-----------GRMR------RPLP----AVVSDIIYD------------KKA--  174
_XP_028031025.1_   141  -AVRLAFQVFVPDGA-----------GRMR------RPLP----AVVSDIIYD------------KKA--  174
_XP_013187225.1_   139  -AVRLCFQVFMTDEA-----------GKVK------HSLT----PVVSDIIYD------------KKA--  172
_XP_052755011.1_   139  -AVRLCFQVFMTDDT-----------GKVK------QTLT----PVVSDVIYD------------KKA--  172
_XP_053613182.1_   139  -AVRLCFQVFMTDEA-----------GKVK------HSLT----PVVSDIIYD------------KKA--  172
_XP_053613180.1_   139  -AVRLCFQVFMTDEA-----------GKVK------HSLT----PVVSDIIYD------------KKA--  172
_CAG9785863.1_     139  -AVRLCFQVFLTDDT-----------YKVQ------CALQ----PIVSDVVYD------------KKA--  172
_KAG6461275.1_     140  -AVRLAFQVFLPHSS-----------GKMR------RTLA----PVVSDVIYD------------KKA--  173
_KAG6461281.1_     140  -AVRLAFQVFLPHSS-----------GKMR------RTLA----PVVSDVIYD------------KKA--  173
_XP_037302137.1_   139  -AIRLAIQVHPMYPS-----------GSML-------KFR----PVVSHVIHD------------KKN--  171
_KAG6461269.1_     139  -AIRLAIQVHPMYPS-----------GSML-------KFR----PVVSHVIHD------------KKN--  171
_KAG4069896.1_     134  --VRLCFQVFIRS-------------GDKL------KPVY----PIVSDIICC------------K----  162
_XP_044003989.1_   144  -AVRLAIQAFVKVPM-----------INTV------VPIE----TAVTEIIYD------------SKT--  177
_XP_045768240.1_   141  -AVRLCFQVFIRDEHT----------NCML------HSLP----PVVSDIIYD------------KKA--  175
_KAG9509099.1_     145  -ALRLCFQVFIEVPGQ----------EGCT------HQLV----PVVSDVIHD------------KKA--  179
_XP_013118267.1_   139  -IVRLCFQAFIPIGP------------RQY------YKLK----PVVTEPIYD------------KKS--  171
_XP_005183026.2_   139  -IVRLCFQAFIPVGS------------QKY------YKLK----PVVTEPIYD------------KKS--  171
_XP_050303432.1_   148  -AVRLCCQVFYWETE------------NGT------KKLRTFCEPVVSNIIYSK----------NKNT--  186
_TMW48950.1_       139  -VVRLCFQAFIPIPG-----------VKKP------FPIP----PVVSDPIYD------------KKS--  172
_XP_037882085.1_   139  -VVRLCFQAFIQSG------------PRQY------KKLT----PVVSDPVYD------------RKA--  171
_KAI8126541.1_     140  -VVRLCFQAFIPLPG-----------VKKP------YPIP----PVVSDPIHD------------KKS--  173
_KAI8126540.1_     139  -VVRLCFQAFIPLPG-----------VKKP------YPIP----PVVSDPIHD------------KKS--  172
_XP_037811156.1_   139  -VVRLCFQAFIPLPG-----------VKKP------YPIP----PVVSDPIHD------------KKS--  172
_XP_046812595.1_   139  -VVRLCFQAFIPLPG-----------VKKP------YPIP----PVVSDPIHD------------KKS--  172
_XP_046812596.1_   139  -VVRLCFQAFIPLPG-----------VKKP------YPIP----PVVSDPIHD------------KKS--  172
_XP_046812591.1_   139  -VVRLCFQAFIPLPG-----------VKKP------YPIP----PVVSDPIHD------------KKS--  172
_XP_046812590.1_   139  -VVRLCFQAFIPLPG-----------VKKP------YPIP----PVVSDPIHD------------KKS--  172
_KNC34143.1_       139  -VVRLCFQAFIPLPG-----------VKKP------YPIP----PVVSDPIHD------------KKS--  172
_XP_046812589.1_   139  -VVRLCFQAFIPLPG-----------VKKP------YPIP----PVVSDPIHD------------KKS--  172
_KAI8126539.1_     139  -VVRLCFQAFIPLPG-----------VKKP------YPIP----PVVSDPIHD------------KKS--  172
_XP_037958917.1_   139  -VVRLCFQAFLKTES-----------GV--------KPLQ----PVVSEPIYD------------KKA--  170
_XP_037929595.1_   141  -VVRLCFQAFLKTES-----------GV--------KPLQ----PVVSEPVFD------------KKA--  172
_XP_037957513.1_   139  -VVRLCFQAFLKTES-----------GV--------KPLQ----PVVSEPVFD------------KKA--  170
_XP_037958115.1_   141  -VVRLCFQAFLKTES-----------GV--------KPLQ----PVVSEPVFD------------KKA--  172
```

```
_XP_037958114.1_  141  -VVRLCFQAFLKTES-----------GV--------KPLQ----PVVSEPVFD------------KKA--  172
_XP_037957511.1_  139  -VVRLCFQAFLKTES-----------GV--------KPLQ----PVVSEPVFD------------KKA--  170
_XP_037958918.1_  139  -VVRLCFQAFLKTES-----------GV--------KPLQ----PVVSEPIHD------------KKA--  170
_RXG71823.1_      137  -SIRLCFQVYLLDRR-----------SGDR------YPLQ----PIVSEPIYD------------K----  168
_KAB7494622.1_     99  -SVSSRPKVRGSIPS-----------AADR------FRANL-RQNACSSCVTGGKEIILLCDKIVK----  145
_KAI9584339.1_    138  -SLRLCFQVFIDDKK-----------GGFT------NPLN----PVVSEPIYE------------KKA--  171
_XP_037884983.1_  138  -SLRLCFQVFIDDKK-----------GGFT------NPLT----PIVSEPIYE------------KKA--  171
_XP_054744821.1_  139  -SLRLCFQGFLKSANG-------------W------EPLS----PVVSEPLYD------------RKA--  170
_XP_053961676.1_  139  -SLRLCFQGFLKSANG-------------W------EPLS----PVVSEPLYD------------RKA--  170
_XP_053961674.1_  139  -SLRLCFQGFLKSANG-------------W------EPLS----PVVSEPLYD------------RKA--  170
_XP_004526634.2_  139  -SLRLCFQGFLKGANG-------------W------ESLL----PVVSEPLYD------------RKA--  170
_CAD6998451.1_    139  -SLRLCFQGFLKGANG-------------W------ESLL----PVVSEPLYD------------RKA--  170
_XP_012157894.1_  139  -SLRLCFQGFLKGANG-------------W------ESLL----PVVSEPLYD------------RKA--  170
_XP_028894493.1_  139  -SLRLCFQGFLKNENG-------------W------EPLS----PVVSEPLYD------------RKA--  170
_XP_036327693.1_  139  -SLRLCFQGFLKGANG-------------W------EPLS----PVVSEPLYD------------RKA--  170
_XP_017471333.1_  139  -SLRLCFQGFLKGANG-------------W------EPLS----PVVSEPLYD------------RKA--  170
_XP_039952608.1_  139  -SLRLCFQGFIKVGNG-------------W------KALS----PVVSEPLYD------------RKA--  170
_XP_050320023.1_  139  -SLRLCFQGFIKVGNG-------------W------KALS----PVVSEPLYD------------RKA--  170
_XP_036225572.1_  139  -SLRLCFQGFIKVGNG-------------W------KALS----PVVSEPLYD------------RKA--  170
_XP_039952607.1_  139  -SLRLCFQGFIKVGNG-------------W------KALS----PVVSEPLYD------------RKA--  170
_XP_036225564.1_  139  -SLRLCFQGFIKVGNG-------------W------KALS----PVVSEPLYD------------RKA--  170
_XP_036225569.1_  139  -SLRLCFQGFIKVGNG-------------W------KALS----PVVSEPLYD------------RKA--  170
_XP_050320021.1_  139  -SLRLCFQGFIKVGNG-------------W------KALS----PVVSEPLYD------------RKA--  170
_XP_039952606.1_  139  -SLRLCFQGFIKVGNG-------------W------KALS----PVVSEPLYD------------RKA--  170
_XP_011207860.1_  139  -SLRLCFQGFIKVGNG-------------W------KALS----PVVSEPLYD------------RKA--  170
_XP_011207859.1_  139  -SLRLCFQGFIKVGNG-------------W------KALS----PVVSEPLYD------------RKA--  170
_XP_029407466.1_  139  -SLRLCFQGFIKVGNG-------------W------KALS----PVVSEPLYD------------RKA--  170
_XP_019847003.2_  139  -SLRLCFQGFIKVGNG-------------W------KALS----PVVSEPLYD------------RKA--  170
_XP_014086488.2_  139  -SLRLCFQGFIKVGNG-------------W------KALS----PVVSEPLYD------------RKA--  170
_XP_049317393.1_  139  -SLRLCFQGFIKVGNG-------------W------KALS----PVVSEPLYD------------RKA--  170
_XP_039952604.1_  139  -SLRLCFQGFIKVGNG-------------W------KALS----PVVSEPLYD------------RKA--  170
_XP_018787557.1_  139  -SLRLCFQGFIKVGNG-------------W------KALN----PVVSEPLYD------------RKA--  170
_OQR68950.1_      139  -AVRLCFQVFIEPERG---------CGRFS------RALK----PVVSDVIFD------------RKA--  174
_XP_003741763.1_  139  -AVRLCFQVFIESIPG---------SDKFD------RPLD----PVVSDVIYD------------RKA--  174
_XP_022699297.1_  139  -AVRLCFQVFIEPERG---------CGRFN------RPLA----PVVSDVIFD------------RKA--  174
_XP_022665229.1_  139  -AVRLCFQVFIEPERG---------CGRFN------RPLA----PVVSDVIFD------------RKA--  174
_XP_022665225.1_  139  -AVRLCFQVFIEPERG---------CGRFN------RPLA----PVVSDVIFD------------RKA--  174
_XP_022665224.1_  139  -AVRLCFQVFIEPERG---------CGRFN------RPLA----PVVSDVIFD------------RKA--  174
_XP_033219687.1_  139  -AVRLCFQVFIQGSD-----------RKFS------VPLK----PVATTIIYD------------KKA--  172
```

```
_XP_033227990.1_  139  -AVRLCFQVFIQGSD-----------RRFS------VPLK----PVATSIIYN------------KKA--  172
_XP_043466013.1_  138  -SVRLCFQVFIQGPQ-----------KKFN------VPLK----PIVSNPIYD------------KKA--  171
_XP_051172671.1_  138  -SVRLCFQVFIQGPQ-----------KKFN------IPLK----PVVSNPIYD------------KKA--  171
_CAD7643228.1_    137  -VIRLCFQVYLESAP-----------DVFD------VALE----PLVSEPIFD------------KKA--  170
_XP_054157157.1_  138  -VVRLAFQVWIEAEL-----------GSGDY----CLPLE----PVVSEPIYD------------KKA--  173
_CAD7645633.1_    137  -VIRLCFQVFLEGPQ-----------EGQF-----DVPLD----PLVSDPIFD------------KKA--  171
_CAD7632611.1_     79  -VIRLCFQVYLESAP-----------DVFD------VALE----PLVSEPIFD------------KKA--  112
_KAJ6223158.1_    136  -VIRLCFQCYLPNPVD----------GEFT------IALD----PIVSDEIYD------------KKA--  170
_KAI2804035.1_    136  -VIRLCFQCYLPNPVD----------GEFT------IALD----PIVSDEIYD------------KKA--  170
_KAH9398209.1_    128  -VVRLCFQVFLMDNLT----------GKYN------IALD----PVVSTEIFD------------KKA--  162
_GFY77286.1_      139  -ALRLCFQVFLPDEH-----------GKLC------FPLP----PVISNPIFD------------NKT--  172
_GFQ73388.1_      139  -ALRLCFQVFLPDEH-----------GKLC------FALP----PVISNPIFD------------NKT--  172
_GFT25575.1_      139  -ALRLCFQVFLPDEH-----------GKLC------IPLP----PVISNPIFD------------NKT--  172
_KAF8763655.1_    139  -ALRLCFQVFLPDEH-----------GKLC------IPLP----PVISNPIFD------------NKT--  172
_GFX76677.1_      139  -ALRLCFQVFLPDEH-----------GKLC------FPLP----PVISNPIFD------------NKT--  172
_XP_035207052.1_  139  -ALRLCFQVFIPDAN-----------GKLS------IPLS----PVISNPIFD------------NKT--  172
_KFM74898.1_      139  -ALRLCFQVFIPDAN-----------GKLS------IPLS----PVISNPIFD------------NKT--  172
_XP_035207059.1_  139  -ALRLCFQVFIPDAN-----------GKLS------IPLS----PVISNPIFD------------NKT--  172
_XP_035207066.1_  139  -ALRLCFQVFIPDAN-----------GKLS------IPLS----PVISNPIFD------------NKT--  172
_BBD75287.1_      139  -CLRLCFQVFLPNET-----------GKMC------IPLP----PVVSNAIYD------------NKT--  172
_XP_042900952.1_  139  -CLRLCFQVFLPNET-----------GKMC------IPLS----PVVSNAIYD------------NKT--  172
_KAG8201816.1_    138  -ALRLCFQVFLPDES-----------GKLC------IPLS----PVVSNAIND------------NKT--  171
_GIX87435.1_      139  -ALRLCFQVFLPDEQ-----------GKLC------IPLP----PVISNPIFD------------NKT--  172
_GBL81029.1_      139  -ALRLCFQVFLPDEH-----------GKLC------IPLP----PVISNPIFD------------NKT--  172
_GIY01087.1_      139  -ALRLCFQVFLPDEQ-----------GKLC------IPLP----PVISNPIFD------------NKT--  172
_XP_054715436.1_  139  -ALRLCFQVFIPDSN-----------GKLT------VPLH----PVISCPIFD------------NKT--  172
_XP_044576112.1_  136  -AIRLCFQVFLKDDE-----------EKCT------IPLK----PVVSEPIYD------------KKI--  169
_XP_008556435.3_  135  -AVRLCFQVFIEDSK-----------GICS------VPLR----PIVSEPVYD------------KKV--  168
_CAD6234446.1_    136  -AVRLCFQVFLKDDE-----------EKYT------IPLK----PVVSEPIYD------------KKI--  169
_XP_044284598.1_  138  -VVRLCFQAFICDEH-----------GNCT------IPLH----PVVSNPIYD------------NRAP-  172
_XP_033794511.1_  138  -VVRLCFQVFLPDEH-----------GNFT------LALP----PVVSNPIYD------------NRAP-  172
_XP_033794512.1_  138  -VVRLCFQVFLPDEH-----------GNFT------LALP----PVVSNPIYD------------NRAP-  172
_XP_029449578.1_  138  -VVRLCFQVFLPDEH-----------GNFT------IALS----PIVSNPIYD------------NRAP-  172
_XP_030052048.1_  138  -VVRLCFQVFLPDEH-----------GNFT------LALP----PVVSNPIYD------------NRAP-  172
_XP_007423639.1_  138  -VVRLCFQAFISDEH-----------GNCT------RSIC----PVISNPIYD------------NRAP-  172
_XP_039203483.1_  138  -VVRLCFQAFISDEH-----------GNCT------QPIS----PVISNPIYD------------NRAP-  172
_XP_034283206.1_  138  -VVRLCFQAFVSDEH-----------GNCT------QPIS----PVISNPIYD------------NRAP-  172
_XP_033001023.1_  138  -VVRLCFQAFLPDEH-----------GNCT------LPLA----PVISNPIYD------------NRAP-  172
_XP_053239675.1_  138  -VVRLCFQAFLPDEH-----------GNCT------LPLA----PVISNPIYD------------NRAP-  172
```

```
_XP_034966804.1_  138  -VVRLCFQAFLPDEH-----------GNCT------LPLA----PVISNPIYD------------NRAP-  172
_XP_028580982.1_  127  -VVRLCFQAFLPDEH-----------GNCT------LPLA----PVVSNPIYD------------NRAP-  161
_XP_042302822.1_  138  -VVRLCFEAFLCDEH-----------GNCT------LALP----PVISNPIYDN----------RSPN--  173
_XP_015280979.1_  127  -VVRLCFQAFLPNEH-----------GDFT------LALP----PVISNPIYD------------NRAP-  161
_XP_048363544.1_  138  -VVRLCFQGFLPNEH-----------GNFT------LALP----PVISNPVYD------------NRAP-  172
_KAF7252806.1_    112  -VVRLCFQAFICDEH-----------GNCT------IPLH----PVVSNPIYD------------NRAP-  146
_XP_034958398.1_   17  -VVRLCFQAFLPDEH-----------GNCT------LPLA----PVISNPIYD------------NRAP-   51
_CAD5116312.1_    140  -CVRICFQAFLPDPE-----------SNNQF----RKVVP----PIVTQPIFD------------HKT--  175
_KAJ1519398.1_    137  -VVRLCFQVFLGDHN---------------------EPLP----PVVSQPIFD------------GAR--  166
_XP_053103001.1_  138  -VVRLCFQAFLPDEH-----------GHCT------RALC----PIISNPVYD------------NRAP-  172
_XP_032070730.1_  138  -VVRLCFQVFICDEH-----------GNCT------QPIS----PVVSNPIYD------------NRAP-  172
_XP_013922116.1_  138  -VVRLCFQVFICDEH-----------GNCT------QPIS----PVVSNPIYD------------NRAP-  172
_XP_015667999.1_  138  -VVRLCFQAFISDEH-----------GNCT------QPIS----PVISNPIYD------------NRAP-  172
_XP_026551846.1_  138  -VVRLCFQAFLSDEL-----------GNCT------QPIS----PVISNPIYD------------NRAP-  172
_XP_026532518.1_  138  -VVRLCFQAFLSDKL-----------GNCT------QPIS----PVISNPIYD------------NRAP-  172
_KAJ7341301.1_    138  -VVRLCFQAFLADEY-----------DNCT------QPLQ----PLVSNPIYD------------NRAP-  172
_KAJ6667038.1_    127  -VVRLCFQAFIPNEH-----------GNYT------LALH----PVISNPIYD------------NRAP-  161
_XP_003227818.2_  127  -VVRLCFQALVCDEH-----------GNCT------IPLP----PVISNPIYD------------NRAP-  161
_XP_020633036.1_  127  -VVRLCFQAFLTDEH-----------GNCT------RSLP----PVISNPIYDN----------RKFL--  162
_XP_054842059.1_  138  -VVRLCFQVFLPNEH-----------GDFM------LALP----PVISNPIYD------------NRAP-  172
_XP_005238641.2_  127  -VVRLCFQAFLPDEH-----------GNYT------LALP----PLISNPIYD------------NRAP-  161
_XP_005445671.2_  127  -VVRLCFQAFLPDEH-----------GNYT------LALP----PLISNPIYD------------NRAP-  161
_XP_054050029.1_  127  -VVRLCFQAFLPDEH-----------GNYT------IALP----PLISNPIYD------------NRAP-  161
_XP_013810845.1_  127  -VVRLCFQAFLPDEH-----------GNYT------LALP----PLISNPIYD------------NRAP-  161
_XP_026702002.1_  127  -VVRLCFQAFLPDEH-----------GNYT------LALP----PLISNPIYD------------NRAP-  161
_XP_025892242.1_  127  -VVRLCFQAFLPDEH-----------GNYT------LALP----PLISNPIYD------------NRAP-  161
_XP_005507389.1_  127  -VVRLCFQAFLPDEH-----------GNYT------IALP----PLISNPIYD------------NRAP-  161
_XP_032911247.1_  127  -VVRLCFQAFLPDEH-----------GNYT------IALP----PLISNPIYD------------NRAP-  161
_XP_031959657.1_  127  -VVRLCFQAFLPDEH-----------GNYT------RALP----PLISNPIYD------------NRAP-  161
_XP_041905291.1_  127  -VVRLCFQAFLPDEH-----------GNYT------RALP----PLISNPIYD------------NRAP-  161
_XP_051648836.1_  127  -VVRLCFQAFLPDEH-----------GNYT------IALP----PLISNPIYD------------NRAP-  161
_XP_054239488.1_  127  -VVRLCFQAFLPDEH-----------GNYT------LALP----PLISNPIYD------------NRAP-  161
_XP_039914164.1_  127  -VVRLCFQAFLPDEH-----------GNYT------IALP----PLISNPIYD------------NRAP-  161
_XP_005422689.1_  127  -VVRLCFQAFLPDEH-----------GNYS------IALP----PLISNPIYD------------NRAP-  161
_KAF2987757.1_    127  -VVRLCFQAFLPDEH-----------GNYS------IALP----PLISNPIYD------------NRAP-  161
_XP_038665087.1_  138  -VVRLCFQVFLPDEH-----------GSFS------RALP----PVVSNPIYD------------NRAP-  172
_GCB59945.1_      138  -VVRLCFQVFLPDEH-----------GSFS------RALP----PVVSNPIYD------------NRAP-  172
_XP_041065616.1_  134  -VVRLCFQVFLPDEH-----------GSFS------RALP----PVVSNPIYD------------NRAP-  168
_XP_007890564.1_  138  -VVRLCFQVFLPDEH-----------GTFT------RALP----PVISNPIYD------------NRAP-  172
```

```
_XP_051868609.1_  138  -VVRLCFQVFLPDEH-----------GSFS------RALP----PVVSNPIYD------------N----  169
_XP_032881051.1_  137  -VVSLCFQVFLPDEH-----------GSFT------RALP----PVVSNPIFD------------NRAP-  171
_XP_048456938.1_   47  -VVRLCFQVFLPDEH-----------GSFS------RALP----PVVSNPIYD------------NRAP-   81
_KAJ1155552.1_    138  -VVRLCFQVFLPDEH-----------GNCT------IPLA----PVVSNPIYDN----------RSPN--  173
_KAH0622877.1_      7  -VVRLCFQAFLCDEH-----------GNCM------LPLP----PVISNPIYD------------NRAP-   41
_CAI5653329.1_    138  -VVRLCIQVFLQDEN-----------GHYT------RALN----PIVTNPIYD------------NRAP-  172
_CAI5653328.1_    138  -VVRLCIQVFLQDEN-----------GHYT------RALN----PIVTNPIYD------------NRAP-  172
_NXS54806.1_      145  -VVRLCFQAFLPDEH-----------GNYT------LALP----PLISNPIYD------------NRAP-  179
_XP_009504395.1_  135  -VVRLCFQAFLPDEH-----------GNYT------IALP----PLISNPIYD------------NRAP-  169
_NXJ41381.1_      138  -VVRLCFQAFLPDEH-----------GNYT------LALP----PLISNPIYD------------NRAP-  172
_NXT24719.1_      138  -VVRLCFQAFLPDEH-----------GNYT------IALP----PLISNPIYD------------NRAP-  172
_NXK23502.1_      138  -VVRLCFQAFLPDEH-----------GNYT------IALP----PLISNPIYD------------NRAP-  172
_NXK03070.1_      138  -VVRLCFQAFLPDEH-----------GNYT------LALP----PLISNPIYD------------NRAP-  172
_XP_037260944.1_  138  -VVRLCFQAFLPDEH-----------GNYT------LALP----PLISNPIYD------------NRAP-  172
_XP_040467369.1_  138  -VVRLCFQAFLPDEH-----------GNYT------LALP----PLISNPIYD------------NRAP-  172
_NXY71491.1_      138  -VVRLCFQAFLPDEH-----------GNYT------IALP----PLISNPIYD------------NRAP-  172
_NXN54417.1_      138  -VVRLCFQAFLPDEH-----------GNYT------IALP----PLISNPIYD------------NRAP-  172
_NXX19490.1_      138  -VVRLCFQAFLPDEH-----------GNYT------LALP----PLISNPIYD------------NRAP-  172
_NXL47199.1_      138  -VVRLCFQAFLPDEH-----------GNYT------LALP----PLISNPIYD------------NRAP-  172
_XP_050749583.1_  138  -VVRLCFQAFLPDEH-----------GNYT------LALP----PLISNPIYD------------NRAP-  172
_NXD76242.1_      138  -VVRLCFQAFLPDEH-----------GNYT------LALP----PLISNPIYD------------NRAP-  172
_KFZ56827.1_      138  -VVRLCFQAFLPDEH-----------GNYT------LALP----PLISNPIYD------------NRAP-  172
_NXH67691.1_      138  -VVRLCFQAFLPDEH-----------GNYT------LALP----PLISNPIYD------------NRAP-  172
_NXS45828.1_      138  -VVRLCFQAFLPDEH-----------GNYT------LALP----PLISNPIYD------------NRAP-  172
_KFP52034.1_      138  -VVRLCFQAFLPDEH-----------GNYT------LALP----PLISNPIYD------------NRAP-  172
_NWH44485.1_      138  -VVRLCFQAFLPDEH-----------GNYT------LALP----PLISNPIYD------------NRAP-  172
_NWW85416.1_      138  -VVRLCFQAFLPDEH-----------GNYT------LALP----PLISNPIYD------------NRAP-  172
_NWU66320.1_      138  -VVRLCFQAFLPDEH-----------GNYT------IALP----PLISNPIYD------------NRAP-  172
_XP_025919906.1_  138  -VVRLCFQAFLPDEH-----------GNYT------LALP----PLISNPIYD------------NRAP-  172
_KAF1483947.1_    138  -VVRLCFQAFLPDEH-----------GNYT------LALP----PLISNPIYD------------NRAP-  172
_NXQ87688.1_      138  -VVRLCFQAFLPDEH-----------GNYT------LALP----PLISNPIYD------------NRAP-  172
_NXX07562.1_      138  -VVRLCFQAFLPDEH-----------GNYT------IALP----PLISNPIYD------------NRAP-  172
_XP_009280788.2_  138  -VVRLCFQAFLPDEH-----------GNYT------LALP----PLISNPIYD------------NRAP-  172
_XP_014821857.1_  138  -VVRLCFQAFLPDEH-----------GNYT------IALP----PLISNPIYD------------NRAP-  172
_NWU51443.1_      138  -VVRLCFQAFLPDEH-----------GNYT------IALP----PLISNPIYD------------NRAP-  172
_NXV35521.1_      138  -VVRLCFQAFLPDEH-----------GNYT------IALP----PLISNPIYD------------NRAP-  172
_KFV80780.1_      138  -VVRLCFQAFLPDEH-----------GNYT------LALP----PLISNPIYD------------NRAP-  172
_XP_032039522.1_  138  -VVRLCFQAFLPDEH-----------GNYT------LALP----PLISNPIYD------------NRAP-  172
_NWT43027.1_      138  -VVRLCFQAFLPDEH-----------GNYT------IALP----PLISNPIYD------------NRAP-  172
```

```
_KAF1651581.1_     138  -VVRLCFQAFLPDEH-----------GNYT------LALP----PLISNPIYD------------NRAP-  172
_NXU26374.1_       138  -VVRLCFQAFLPDEH-----------GNYT------LALP----PLISNPIYD------------NRAP-  172
_NWX46229.1_       138  -VVRLCFQAFLPDEH-----------GNYT------LALP----PLISNPIYD------------NRAP-  172
_PKU40340.1_       138  -VVRLCFQAFLPDEH-----------GNYT------IALP----PLISNPIYD------------NRAP-  172
_XP_027309078.1_   138  -VVRLCFQAFLPDEH-----------GNYT------LALP----PLISNPIYD------------NRAP-  172
_KAF1573683.1_     138  -VVRLCFQAFLPDEH-----------GNYT------LALP----PLISNPIYD------------NRAP-  172
_KAF1578928.1_     138  -VVRLCFQAFLPDEH-----------GNYT------LALP----PLISNPIYD------------NRAP-  172
_NXN47104.1_       138  -VVRLCFQAFLPDEH-----------GNYT------IALP----PLISNPIYD------------NRAP-  172
_NXV20393.1_       138  -VVRLCFQAFLPDEH-----------GNYT------IALP----PLISNPIYD------------NRAP-  172
_KAF1551267.1_     138  -VVRLCFQAFLPDEH-----------GNYT------LALP----PLISNPIYD------------NRAP-  172
_KAF1406210.1_     138  -VVRLCFQAFLPDEH-----------GNYT------LALP----PLISNPIYD------------NRAP-  172
_NXV43201.1_       138  -VVRLCFQAFLPDEH-----------GNYT------IALP----PLISNPIYD------------NRAP-  172
_NWQ98669.1_       138  -VVRLCFQAFLPDEH-----------GNYT------LALP----PLISNPIYD------------NRAP-  172
_XP_052639968.1_   138  -VVRLCFQAFLPDEH-----------GNYT------LALP----PLISNPIYD------------NRAP-  172
_XP_010573143.1_   138  -VVRLCFQAFLPDEH-----------GNYT------LALP----PLISNPIYD------------NRAP-  172
_KAF1479604.1_     138  -VVRLCFQAFLPDEH-----------GNYT------LALP----PLISNPIYD------------NRAP-  172
_NXG89109.1_       138  -VVRLCFQAFLPDEH-----------GNYT------IALP----PLISNPIYD------------NRAP-  172
_KFQ89757.1_       138  -VVRLCFQAFLPDEH-----------GNYT------LALP----PLISNPIYD------------NRAP-  172
_NWZ21787.1_       138  -VVRLCFQAFLPDEH-----------GNYT------LALP----PLISNPIYD------------NRAP-  172
_NXI74048.1_       138  -VVRLCFQAFLPDEH-----------GNYT------LALP----PLISNPIYD------------NRAP-  172
_NWX76247.1_       138  -VVRLCFQAFLPDEH-----------GNYT------IALP----PLISNPIYD------------NRAP-  172
_NXF29788.1_       138  -VVRLCFQAFLPDEY-----------GNYT------LALP----PLISNPIYD------------NRAP-  172
_KAF1444142.1_     138  -VVRLCFQAFLPDEH-----------GNYT------LALP----PLISNPIYD------------NRAP-  172
_KFV20215.1_       138  -VVRLCFQAFLPDEH-----------GNYT------LALP----PLISNPIYD------------NRAP-  172
_NXI44657.1_       138  -VVRLCFQAFLPDEH-----------GNYT------LALP----PLISNPIYD------------NRAP-  172
_NXJ92321.1_       138  -VVRLCFQAFLPDEH-----------GNYT------LALP----PLISNPIYD------------NRAP-  172
_NWZ49902.1_       138  -VVRLCFQAFLPDEH-----------GNYT------LALP----PLISNPIYD------------NRAP-  172
_NWY59444.1_       138  -VVRLCFQAFLADEH-----------GNYT------IALP----PLISNPIYD------------NRAP-  172
_KFW64019.1_       138  -VVRLCFQAFLPDEH-----------GNYT------LALP----PLISNPIYD------------NRAP-  172
_NXJ55634.1_       138  -VVRLCFQAFLPDEH-----------GNYT------LALP----PLISNPIYD------------NRAP-  172
_KAF1513372.1_     138  -VVRLCFQAFLPDEH-----------GNYT------LALP----PLISNPIYD------------NRAP-  172
_NXW14598.1_       138  -VVRLCFQAFLPDEH-----------GNYT------LALP----PLISNPIYD------------NRAP-  172
_NXC74247.1_       138  -VVRLCFQAFLPDEH-----------GNYT------IALP----PLISNPIYD------------NRAP-  172
_NXT57254.1_       138  -VVRLCFQAFLADEH-----------GNYT------IALP----PLISNPIYD------------NRAP-  172
_XP_047916679.1_   138  -VVRLCFQAFLPDEH-----------GNYT------LALP----PLISNPIYD------------NRAP-  172
_XP_029879190.1_   138  -VVRLCFQAFLPDEH-----------GNYT------LALP----PLISNPIYD------------NRAP-  172
_NXH09241.1_       138  -VVRLCFQAFLPDEH-----------GNYT------LALP----PLISNPIYD------------NRAP-  172
_XP_040407374.1_   138  -VVRLCFQAFLPDEH-----------GNYT------LALP----PLISNPIYD------------NRAP-  172
_KFW76037.1_       138  -VVRLCFQAFLPDEH-----------GNYT------IALP----PLISNPIYD------------NRAP-  172
```

```
_KFP65945.1_       138  -VVRLCFQAFLPDEH-----------GNYT------LALP----PLISNPIYD------------NRAP-  172
_NXX55682.1_       138  -VVRLCFQAFLPDEH-----------GNYT------LALP----PLISNPIYD------------NRAP-  172
_XP_035178333.1_   138  -VVRLCFQAFLPDEH-----------GNYT------LALP----PLISNPIYD------------NRAP-  172
_NXE47317.1_       138  -VVRLCFQAFLPDEH-----------GNYT------LALP----PLISNPIYD------------NRAP-  172
_XP_035408877.1_   138  -VVRLCFQAFLPDEH-----------GNYT------LALP----PLISNPIYD------------NRAP-  172
_KGL94141.1_       138  -VVRLCFQAFLPDEH-----------GNYT------LALP----PLISNPIYD------------NRAP-  172
_NXN68403.1_       138  -VVRLCFQAFLPDEH-----------GNYT------LALP----PLISNPIYD------------NRAP-  172
_NXW28331.1_       138  -VVRLCFQAFLPDEH-----------GNYT------IALP----PLISNPIYD------------NRAP-  172
_XP_049657509.1_   138  -VVRLCFQAFLPDEH-----------GNYT------LALP----PLISNPIYD------------NRAP-  172
_XP_049657511.1_   138  -VVRLCFQAFLPDEH-----------GNYT------LALP----PLISNPIYD------------NRAP-  172
_NXG30098.1_       138  -VVRLCFQAFLPDEH-----------GNYT------LALP----PLISNPIYD------------NRAP-  172
_NXV86645.1_       138  -VVRLCFQAFLPDEH-----------GNYT------LALP----PLISNPIYD------------NRAP-  172
_NXX83200.1_       138  -VVRLCFQAFLPDQH-----------GNYT------LPLP----PLISNPIYD------------NRAP-  172
_NXI59898.1_       138  -VVRLCFQAFLPDEH-----------GNYT------LALP----PLISNPIYD------------NRAP-  172
_NWU89521.1_       138  -VVRLCFQAFLPDEH-----------GNYT------LALP----PLISNPIYD------------NRAP-  172
_NXK45490.1_       138  -VVRLCFQAFLPDEH-----------GNYT------LALP----PLISNPIYD------------NRAP-  172
_NWR55599.1_       138  -VVRLCFQAFLPDEH-----------GNYT------IALP----PLISNPIYD------------NRAP-  172
_NXL37824.1_       138  -VVRLCFQAFLPDEH-----------GNYT------LALP----PLISNPIYD------------NRAP-  172
_NWI33553.1_       138  -VVRLCFQAFLPDEH-----------GNYT------IALP----PLISNPIYD------------NRAP-  172
_KFQ74517.1_       138  -VVRLCFQAFLPDEH-----------GNYT------LALP----PLISNPIYD------------NRAP-  172
_NXV82662.1_       138  -VVRLCFQAFLPDEQ-----------GNYT------LALP----PLISNPIYD------------NRAP-  172
_NWJ10087.1_       138  -VVRLCFQAFLPDEH-----------GNYT------LALP----PLISNPIYD------------NRAP-  172
_NXN24483.1_       138  -VVRLCFQAFLPDEH-----------GNYT------IALP----PLISNPIYD------------NRAP-  172
_NWR80208.1_       138  -VVRLCFQAFLPDKH-----------GNYT------LALP----PLISNPIYD------------NRAP-  172
_NXT37538.1_       138  -VVRLCFQAFLPDEH-----------GNYT------LALP----PLISNPIYD------------NRAP-  172
_XP_032844994.2_   138  -VVRLCFQAFLPDEH-----------GNYT------LALP----PLISNPIYD------------NRAP-  172
_NXW47649.1_       138  -VVRLCFQAFLPDEH-----------GNYT------LALP----PLISNPIYD------------NRAP-  172
_NXA40398.1_       138  -VVRLCFQAFLPDEH-----------GNYT------LALP----PLISNPIYD------------NRAP-  172
_NXW64893.1_       138  -VVRLCFQAFLPDEH-----------GNYT------LALP----PLISNPIYD------------NRAP-  172
_NXX89165.1_       138  -VVRLCFQAFLPDKH-----------GNYT------LALP----PLISNPIYD------------NRAP-  172
_NXQ96911.1_       138  -VVRLCFQAFLPDEH-----------GNYT------LALP----PLISNPIYD------------NRAP-  172
_KFP18273.1_       138  -VVRLCFQAFLPDEH-----------GNYT------IALP----PLISNPIYD------------NRAP-  172
_NXC40882.1_       138  -VVRLCFQAFLPDEH-----------GNYT------LALP----PLISNPIYD------------NRAP-  172
_NXE10305.1_       138  -VVRLCFQAFLPDEH-----------GNYT------LALP----PLISNPIYD------------NRAP-  172
_KFP88087.1_       138  -VVRLCFQAFLPDEH-----------GNYT------LALP----PLISNPIYD------------NRAP-  172
_NWW47871.1_       138  -VVRLCFQAFLPDEH-----------GNYT------IALP----PLISNPIYD------------NRAP-  172
_NXJ61595.1_       138  -VVRLCFQAFLPDEH-----------GNYT------IALP----PLISNPIYD------------NRAP-  172
_NXD13814.1_       138  -VVRLCFQAFLPDEH-----------GNYT------LALP----PLISNPIYD------------NRAP-  172
_KFR16961.1_       138  -VVRLCFQAFLPDEH-----------GNYT------LALP----PVISNPIYD------------NRAP-  172
```

```
_NWX24312.1_       138  -VVRLCFQAFLPDEH-----------GNYT------LALP----PLISNPIYD------------NRAP-  172
_NXA28267.1_       138  -VVRLCFQAFLPDEH-----------GNYT------LALP----PLISNPIYD------------NRAP-  172
_NXL66270.1_       138  -VVRLCFQAFLPDEH-----------GNYT------LALP----PLISNPIYD------------NRAP-  172
_NXA49976.1_       138  -VVRLCFQAFLPDEH-----------GNYT------LALP----PLISNPIYD------------NRAP-  172
_NXT77624.1_       138  -VVRLCFQAFLPDEL-----------GNYT------LALP----PLISNPIYD------------NRAP-  172
_KFQ92336.1_       138  -VVRLCFQAFLPDEH-----------GNYT------LALP----PLISNPIYD------------NRAP-  172
_NXE25013.1_       138  -VVRLCFQAFLPDEH-----------GNYT------LALP----PLISNPIYD------------NRAP-  172
_NXE92240.1_       138  -VVRLCFQAFLPDEH-----------GNYT------IALP----PLISNPIYD------------NRAP-  172
_NWH80943.1_       138  -VVRLCFQAFLPDKH-----------GNYT------IALP----PLISNPIYD------------NRAP-  172
_NWI19058.1_       138  -VVRLCFQAFLPDEH-----------GNYT------LALP----PLISNPIYD------------NRAP-  172
_NXG80610.1_       138  -VVRLCFQAFLPDEH-----------GNYT------LALP----PLISNPIYD------------NRAP-  172
_NWR01486.1_       138  -VVRLCFQAFLPDEH-----------GNYT------IALP----PLISNPIYD------------NRAP-  172
_NXK81337.1_       138  -VVRLCFQAFLPDEH-----------GNYT------LALP----PLISNPIYD------------NRAP-  172
_NXS90563.1_       138  -VVRLCFQAFLPDDH-----------GNYT------IALP----PLISNPIYD------------NRAP-  172
_NXC17430.1_       138  -VVRLCFQAFLPDEH-----------GNYT------LALP----PLISNPIYD------------NRAP-  172
_NXE77245.1_       138  -VVRLCFQAFLPDEH-----------GNYT------IALP----PLISNPIYD------------NRAP-  172
_KFQ29788.1_       139  -VVRLCFQAFLPDEH-----------GNYT------LALP----PLISNPIYD------------NRAP-  173
_KFO89327.1_       138  -VVRLCFQAFLPDEH-----------GNYT------IALP----PLISNPIYD------------NRAP-  172
_NXD64381.1_       138  -VVRLCFQAFLPDEH-----------GNYT------LALP----PLISNPIYD------------NRAP-  172
_NXY45081.1_       138  -VVRLCFQAFLPDKH-----------GNYT------VALP----PLISNPIYD------------NRAP-  172
_KGL72871.1_       138  -VVRLCFQAFLPDEH-----------GNYT------LALP----PLISNPIYD------------NRAP-  172
_XP_053919504.1_   138  -VVRLCFQAFLPDKH-----------GNYT------VALP----PLISNPIYD------------NRAP-  172
_NXL85459.1_       138  -VVRLCFQAFLPDEH-----------GNYT------LALP----PLISNPIYD------------NRAP-  172
_NXO02254.1_       138  -VVRLCFQAFLPDEH-----------GNYT------LALP----PLISNPIYD------------NRAP-  172
_NXJ76034.1_       138  -VVRLCFQAFLPDEH-----------GNYT------LALP----PLISNPIYD------------NRAP-  172
_NXM93491.1_       138  -VVRLCFQAFLPDEH-----------GNYT------IALP----PLISNPIYD------------NRAP-  172
_XP_030345800.1_   138  -VVRLCFQAFLPDEH-----------GNYT------LALP----PLISNPIYD------------NRAP-  172
_XP_030302544.1_   138  -VVRLCFQAFLPDEH-----------GNYT------LALP----PLISNPIYD------------NRAP-  172
_XP_030345801.1_   138  -VVRLCFQAFLPDEH-----------GNYT------LALP----PLISNPIYD------------NRAP-  172
_KQK77724.1_       138  -VVRLCFQAFLPDEH-----------GNYT------LALP----PLISNPIYD------------NXAP-  172
_NXP09913.1_       138  -VVRLCFQAFLPDEH-----------GNYT------IALP----PLISNPIYD------------NRAP-  172
_NXR33018.1_       138  -VVRLCFQAFLPDEH-----------GNYT------IALP----PLISNPIYD------------NRAP-  172
_NXM76881.1_       138  -VVRLCFQAFLPDEH-----------GNYT------IALP----PLISNPIYD------------NRAP-  172
_NXS27423.1_       138  -VVRLCFQAFLPDEH-----------GNYT------IALP----PLISNPIYD------------NRAP-  172
_NXM55821.1_       138  -VVRLCFQAFLPDEH-----------GNYT------IALP----PLISNPIYD------------NRAP-  172
_XP_054678500.1_   138  -VVRLCFQAFLPDEH-----------GNYT------LALP----PLISNPIYD------------NRAP-  172
_XP_015712248.1_   138  -VVRLCFQAFLPDEH-----------GNYT------LALP----PLISNPIYD------------NRAP-  172
_NWX92058.1_       138  -VVRLCFQAFLPDEH-----------GNYT------LALP----PLISNPIYD------------NRAP-  172
_XP_048793646.1_   138  -VVRLCFQAFLPDEH-----------GNYT------LALP----PLISNPIYD------------NRAP-  172
```

```
_NXF04247.1_       138  -VVRLCFQAFLPDEH-----------GNYT------IALP----PLISNPIYD------------NRAP-  172
_XP_042683331.1_   138  -VVRLCFQAFLPDEH-----------GNYT------LALP----PLISNPIYD------------NRAP-  172
_NWV22147.1_       138  -VVRLCFQAFLPDEH-----------GNYT------IALP----PLISNPIYD------------NRAP-  172
_NXA04361.1_       138  -VVRLCFQAFLPDEH-----------GNYT------IALP----PLISNPIYD------------NRAP-  172
_XP_010116198.1_   144  -VVRLCFQAFLPDEH-----------GNYT------LALP----PLISNPIYD------------NRAP-  178
_NXG59741.1_       138  -VVRLCFQAFLPDEH-----------GNYT------LALP----PLISNPIYD------------NRAP-  172
_XP_042735326.1_   138  -VVRLCFQAFLPDEH-----------GNYT------LALP----PLISNPIYD------------NRAP-  172
_NXM29886.1_       138  -VVRLCFQAFLPDEH-----------GNYT------IALP----PLISNPIYD------------NRAP-  172
_NP_001161198.2_   138  -VVRLCFQAFLPDEH-----------GNYT------LALP----PLISNPIYD------------NRAP-  172
_XP_031451029.1_   138  -VVRLCFQAFLPDEH-----------GNYT------LALR----PLISNPIYD------------NRAP-  172
_NXR49262.1_       138  -VVRLCFQAFLPDEH-----------GNYT------IALP----PLISNPIYD------------NRAP-  172
_XP_052555416.1_   138  -VVRLCFQAFLPDEH-----------GNYT------LALP----PLISNPIYD------------NRAP-  172
_NWZ67085.1_       138  -VVRLCFQAFLPDEH-----------GNYT------IALP----PLISNPIYD------------NRAP-  172
_P16236.2_         138  -VVRLCFQAFLPDEH-----------GNYT------LALP----PLISNPIYD------------NRAP-  172
_XP_050566010.1_   138  -VVRLCFQAFLPDEH-----------GNYT------LALP----PLISNPIYD------------NRAP-  172
_NWS67030.1_       138  -VVRLCFQAFLPDKH-----------GNYT------LALP----PLISNPIYD------------NRAP-  172
_XP_005143850.3_   138  -VVRLCFQAFLPDEH-----------GNYT------LALP----PLISNPIYD------------NRAP-  172
_XP_050566011.1_   138  -VVRLCFQAFLPDEH-----------GNYT------LALP----PLISNPIYD------------NRAP-  172
_XP_050566009.1_   138  -VVRLCFQAFLPDEH-----------GNYT------LALP----PLISNPIYD------------NRAP-  172
_NWR30963.1_       138  -VVRLCFQAFLPDEH-----------GNYT------IALP----PLISNPIYD------------NRAP-  172
_NWS17896.1_       138  -VVRLCFQAFLPDEH-----------GNYT------IALP----PLISNPIYD------------NRAP-  172
_XP_021248574.1_   138  -VVRLCFQAFLPDEH-----------GNYT------LALP----PLISNPIYD------------NRAP-  172
_AAA48721.1_       138  -VVRLCFQAFLPDEH-----------GNYT------LALP----PLISNPIYD------------NRAP-  172
_NWI54208.1_       138  -VVRLCFQAFLPDEH-----------GNYT------IALP----PLISNPIYD------------NRAP-  172
_XP_027509532.1_   138  -VVRLCFQAFLPDEH-----------GNYT------IALP----PLISNPIYD------------NRAP-  172
_NXP71979.1_       138  -VVRLCFQAFLPDEH-----------GNYT------LALP----PLISNPIYD------------NRAP-  172
_NXX25352.1_       138  -VVRLCFQAFLPDEH-----------GNYT------IALP----PLISNPIYD------------NRAP-  172
_NXI18618.1_       138  -VVRLCFQAFLPDEH-----------GNYT------IALP----PLISNPIYD------------NRAP-  172
_NXS09623.1_       138  -VVRLCFQAFLPDEH-----------GNYT------IALP----PVISNPIYD------------NRAP-  172
_NXU77980.1_       138  -VVRLCFQAFLPDEH-----------GNYT------IALP----PLISNPIYD------------NRAP-  172
_XP_027509533.1_   138  -VVRLCFQAFLPDEH-----------GNYT------IALP----PLISNPIYD------------NRAP-  172
_NWV71036.1_       138  -VVRLCFQAFLPDEH-----------GNYT------VALP----PLISNPIYD------------NRAP-  172
_NXF89482.1_       138  -VVRLCFQAFLPDEQ-----------GNYT------LALP----PLISNPIYD------------NRAP-  172
_NWY39470.1_       138  -VVRLCFQAFLPDEH-----------GNYT------IALP----PLISNPIYD------------NRAP-  172
_NXT59960.1_       138  -VVRLCFQAFLPDEH-----------GNYT------IALP----PLISNPIYD------------NRAP-  172
_NWU74340.1_       138  -VVRLCFQAFLPDEH-----------GNYT------IALP----PLISNPIYD------------NRAP-  172
_NXB84481.1_       138  -VVRLCFQAFLPDEH-----------GNYT------MALP----PLISNPIYD------------NRAP-  172
_NWV73885.1_       138  -VVRLCFQAFLPDEH-----------GNYT------IALP----PLISNPIYD------------NRAP-  172
_OPJ58894.1_       138  -VVRLCFQAFLPDEH-----------GSYT------IALP----PLISNPIYD------------NRAP-  172
```

```
_XP_048152104.1_  138  -VVRLCFQAFLPDEH-----------GNYT------RALP----PLISNPIYD------------NRAP-  172
_XP_054036037.1_  138  -VVRLCFQAFLPDEH-----------GNYT------LALP----PLISNPIYD------------NRAP-  172
_NXR18149.1_      138  -VVRLCFQAFLPDEH-----------GNYT------IALP----PLISNPIYD------------NRAP-  172
_NXP97533.1_      138  -VVRLCFQAFLPDEH-----------GNYT------MALP----PLISNPIYD------------NRAP-  172
_NXJ05495.1_      138  -VVRLCFQAFLPDEH-----------GNYT------LALP----PLISNPIYD------------NRAP-  172
_XP_032538932.1_  138  -VVRLCFQAFLPDEH-----------GNYT------IALP----PLISNPIYD------------NRAP-  172
_NWI96387.1_      138  -VVRLCFQAFLPDEH-----------GNYT------IALP----PLISNPIYD------------NRAP-  172
_NXR65995.1_      138  -VVRLCFQAFLPDEH-----------GNYT------IALP----PLISNPIYD------------NRAP-  172
_NXK42398.1_      138  -VVRLCFQAFLPDEH-----------GNYT------IALP----PLISNPIYD------------NRAP-  172
_XP_032538931.1_  138  -VVRLCFQAFLPDEH-----------GNYT------IALP----PLISNPIYD------------NRAP-  172
_NXY33181.1_      138  -VVRLCFQAFLPDEH-----------GNYT------IALP----PLISNPIYD------------NRAP-  172
_XP_050171009.1_  138  -VVRLCFQAFLPDEH-----------GNYT------IALP----PLISNPIYD------------NRAP-  172
_NXO50939.1_      138  -VVRLCFQAFLPDEH-----------GNYT------LALP----PLISNPIYD------------NRAP-  172
_NWZ32937.1_      138  -VVRLCFQAFLPDEH-----------GNYT------IALP----PLISNPIYD------------NRAP-  172
_NXP01828.1_      138  -VVRLCFQAFLPDEH-----------GNYT------IALP----PLISNPIYD------------NRAP-  172
_NXL00458.1_      138  -VVRLCFQAFLPDEH-----------GNYT------LPLP----PLISNPIYD------------NRAP-  172
_XP_010401310.4_  138  -VVRLCFQAFLPDEH-----------GNYT------RALP----PLISNPIYD------------NRAP-  172
_NXH62149.1_      138  -VVRLCFQAFLPDEH-----------GNYT------IALP----PLISNPIYD------------NRAP-  172
_NXG43824.1_      138  -VVRLCFQAFLPDEH-----------GNYT------LALP----PLISNPIYD------------NRAP-  172
_XP_017677337.1_  138  -VVRLCFQAFLPDEH-----------GNYT------IALP----PLISNPIYD------------NRAP-  172
_NXU09156.1_      138  -VVRLCFQAFLPDEH-----------GNYT------IALP----PLISNPIYD------------NRAP-  172
_NXB38645.1_      138  -VVRLCFQAFLPDEH-----------GNYT------IALP----PLISNPIYD------------NRAP-  172
_XP_027594504.1_  138  -VVRLCFQAFLPDEH-----------GNYT------IALP----PLISNPIYD------------NRAP-  172
_NXM46269.1_      138  -VVRLCFQAFLPDEH-----------GNYT------IALP----PLISNPIYD------------NRAP-  172
_NWS79257.1_      138  -VVRLCFQAFLPDEH-----------GNYT------IALP----PLISNPIYD------------NRAP-  172
_NXB04839.1_      138  -VVRLCFQAFLPDEH-----------GNYT------IALP----PLISNPIYD------------NRAP-  172
_NXC62865.1_      138  -VVRLCFQAFLPDEH-----------GNYT------IALP----PLISNPIYD------------NRAP-  172
_NXN18277.1_      138  -VVRLCFQAFLPDEH-----------GNYT------LALP----PLISNPIYD------------NRAP-  172
_XP_027760039.1_  138  -VVRLCFQAFLPDEH-----------GNYT------IALP----PLISNPIYD------------NRAP-  172
_NXI90311.1_      138  -VVRLCFQAFLPDKH-----------GNYT------LALP----PLISNPIYD------------NRAP-  172
_NWY17003.1_      138  -VVRLCFQAFLPDEH-----------GNYT------RALP----PLISNPIYD------------NRAP-  172
_NXH97099.1_      138  -VVRLCFQAFLPDEH-----------GNYT------IALP----PLISNPIYD------------NRAP-  172
_NWS97308.1_      138  -VVRLCFQAFLPDEH-----------GNYT------IALP----PLISNPIYD------------NRAP-  172
_NWU45160.1_      138  -VVRLCFQAFLPDEH-----------GNYT------IALP----PLISNPIYD------------NRAP-  172
_NXQ43004.1_      138  -VVRLCFQAFLPDEH-----------GNYT------IALP----PLISNPIYD------------NRAP-  172
_XP_027549142.1_  138  -VVRLCFQAFLPDEH-----------GNYT------IALP----PLISNPIYD------------NRAP-  172
_XP_017933082.2_  138  -VVRLCFQAFLPDEH-----------GNYT------IALP----PLISNPIYD------------NRAP-  172
_NWV96326.1_      138  -VVRLCFQAFLPDEH-----------GNYT------IALP----PLISNPIYD------------NRAP-  172
_NXP57783.1_      138  -VVRLCFQAFLPDEH-----------GNYT------IALP----PLISNPIYD------------NRAP-  172
```

```
_NXY57955.1_      138  -VVRLCFQAFLPDEH-----------GNYT------IALP----PLISNPIYD------------NRAP-  172
_AAA99433.1_      138  -VVRLCFQAFLPDEH-----------GNYT------LALP----PLISNPIYD------------NRAP-  172
_NXY21218.1_      138  -VVRLCFQAFLPDEH-----------GNYT------IALP----PLISNPIYD------------NRAP-  172
_NXX50824.1_      138  -VVRLCFQAFLPDEH-----------GNYT------LALP----PLISNPIYD------------NRAP-  172
_XP_030124623.3_  138  -VVRLCFQAFLPDEH-----------GNYT------IALP----PLISNPIYD------------NRAP-  172
_NXA92235.1_      138  -VVRLCFQAFLPDEH-----------GNYT------IALP----PLISNPIYD------------NRAP-  172
_NWI05522.1_      138  -VVRLCFQAFLPDEH-----------GNYT------IALP----PLISNPIYD------------NRAP-  172
_NWX29009.1_      138  -VVRLCFQAFLPDEH-----------GNYT------IALP----PLISNPIYD------------NRAP-  172
_XP_027549144.1_  138  -VVRLCFQAFLPDEH-----------GNYT------IALP----PLISNPIYD------------NRAP-  172
_NWW35420.1_      138  -VVRLCFQAFLPDEH-----------GNYT------IALP----PLISNPIYD------------NRAP-  172
_NWV07969.1_      138  -VVRLCFQAFLPDEH-----------GNYT------IALP----PLISNPIYD------------NRAP-  172
_NXH42214.1_      138  -VVRLCFQAFLPDEH-----------GNYT------IALP----PLISNPIYD------------NRAP-  172
_NXQ18773.1_      138  -VVRLCFQAFLPDEH-----------GNYT------IALP----PLISNPIYD------------NRAP-  172
_NWX10672.1_      138  -VVRLCFQAFLPDEH-----------GNYT------IALP----PLISNPIYD------------NRAP-  172
_NXU50687.1_      138  -VVRLCFQAFLPDEH-----------GNYT------IALP----PLISNPIYD------------NRAP-  172
_KFU84784.1_      138  -VVRLCFQAFLPDEH-----------GNYT------LALP----PLISNPIYD------------NRAP-  172
_NXQ52438.1_      138  -VVRLCFQAFLPDER-----------GNYT------IALP----PLISNPIYD------------NRAP-  172
_NWW24536.1_      138  -VVRLCFQAFLPDEH-----------GNYT------IALP----PLISNPIYD------------NRAP-  172
_NXF73449.1_      138  -VVRLCFQAFLPDEH-----------GNYT------IALP----PLISNPIYD------------NRAP-  172
_XP_050827906.1_  138  -VVRLCFQAFLPDEH-----------GNYS------IALP----PLISNPIYD------------NRAP-  172
_NWX65205.1_      138  -VVRLCFQAFLPDEH-----------GNYT------IALP----PLISNPIYD------------NRAP-  172
_KFW81749.1_      138  -VVRLCFQAFLPDEH-----------GNYT------IALP----PLISNPIYD------------NRAP-  172
_XP_014750593.1_  138  -VVRLCFQAFLPDEH-----------GNYT------IALP----PLISNPIYD------------NRAP-  172
_NXJ17066.1_      138  -VVRLCFQAFLPDEH-----------GNYT------IALP----PLISNPIYD------------NRAP-  172
_NWW67019.1_      138  -VVRLCFQAFLPDEH-----------GNYT------IALP----PLISNPIYD------------NRAP-  172
_NXH03360.1_      138  -VVRLCFQAFLPDEH-----------GNYS------IALP----PLISNPIYD------------NRAP-  172
_XP_037987525.1_  138  -VVRLCFQAFLPDEH-----------GNYT------IALP----PLISNPIYD------------NRAP-  172
_NXL74522.1_      138  -VVRLCFQAFLPDEH-----------GNYT------IALP----PLISNPIYD------------NRAP-  172
_NWQ60824.1_      138  -VVRLCFQAFLPDEH-----------GNYT------IALP----PLISNPIYD------------NRAP-  172
_NXR70971.1_      138  -VVRLCFQAFLPDEH-----------GNYT------IALP----PLISNPIYD------------NRAP-  172
_XP_051468947.1_  138  -VVRLCFQAFLPDEH-----------GNYT------LALP----PLISNPIYD------------NRAP-  172
_NXB50199.1_      138  -VVRLCFQAFLPDEH-----------GNYT------IALP----PLISNPIYD------------NRAP-  172
_NXP18380.1_      138  -VVRLCFQAFLPDEH-----------GNYT------IALP----PLISNPIYD------------NRAP-  172
_NXI84319.1_      138  -VVRLCFQAFLPDEH-----------GNYT------IALP----PLISNPIYD------------NRAP-  172
_NWH33757.1_      138  -VVRLCFQAFLPDEH-----------GNYT------IALP----PLISNPIYD------------NRAP-  172
_NWR99781.1_      138  -VVRLCFQAFLPDEH-----------GNYT------IALP----PLISNPIYD------------NRAP-  172
_NXW94404.1_      138  -VVRLCFQAFLPDEH-----------GNYT------IALP----PLISNPIYD------------NRAP-  172
_CAA27220.1_      138  -VVRLCFQAFLPDEH-----------GNYT------LALP----PLISNPIYD------------NGAP-  172
_XP_015477679.1_  138  -VVRLCFQAFLPDEH-----------GNYT------IALP----PLISNPIYD------------NRAP-  172
```

```
_NWS32597.1_       138  -VVRLCFQAFLPDEH-----------GNYT------IALP----PLISNPIYD------------NRAP-  172
_NXG22126.1_       138  -VVRLCFQAFLPDEH-----------GNYT------IALP----PLISNPIYD------------NRAP-  172
_NWY97127.1_       138  -VVRLCFQAFLPDEH-----------GNYS------IALP----PLISNPIYD------------NRAP-  172
_NXW80148.1_       138  -VVRLCFQAFLPDEH-----------GNYT------IALP----PLISNPIYD------------NRAP-  172
_NXC27367.1_       138  -VVRLCFQAFLPDEH-----------GNFT------IALP----PLISNPIYD------------NRAP-  172
_NWH83726.1_       138  -VVRLCFQAFLPDEH-----------GNYT------IALP----PLISNPIYD------------NRAP-  172
_NXA73512.1_       138  -VVRLCFQAFLPDEH-----------GNYT------IALP----PLISNPIYD------------NRAP-  172
_NWZ83230.1_       138  -VVRLCFQAFLPDEH-----------GNYT------IALP----PLISNPIYD------------NRAP-  172
_XP_005525572.2_   138  -VVRLCFQAFLPDEH-----------GNYT------IALP----PLISNPIYD------------NRAP-  172
_NXD20033.1_       138  -VVRLCFQAFLPDEH-----------GNYT------IALP----PLISNPIYD------------NRAP-  172
_NXE37177.1_       138  -VVRLCFQAFLPDEH-----------GNYT------IALP----PLISNPIYD------------NRAP-  172
_NWV44369.1_       138  -VVRLCFQAFLPDEL-----------GNYT------IALP----PLISNPIYD------------NRAP-  172
_NXC81400.1_       138  -VVRLCFQAFLPDEH-----------GNYT------IALP----PLISNPIYD------------NRAP-  172
_NXA67345.1_       138  -VVRLCFQAFLPDEH-----------GNYT------IALP----PLISNPIYD------------NRAP-  172
_NXB55155.1_       138  -VVRLCFQAFLPDKH-----------GNYT------IALP----PLISNPIYD------------NRAP-  172
_NXN81229.1_       138  -VVRLCFQAFLPDEH-----------GNYT------IALP----PLISNPIYD------------NRAP-  172
_NXM15601.1_       138  -VVRLCFQAFIPDEH-----------GNYT------IALP----PLISNPIYD------------NRAP-  172
_XP_054484850.1_   138  -VVRLCFQAFLPDEH-----------GNYS------IALP----PLISNPIYD------------NRAP-  172
_NWU02685.1_       138  -VVRLCFQAFLPDEH-----------GSYT------IALP----PLISNPIYD------------NRAP-  172
_XP_041273165.1_   138  -VVRLCFQAFLPDEH-----------GNYS------IALP----PLISNPIYD------------NRAP-  172
_NWW80397.1_       138  -VVRLCFQAFLPDEH-----------GNYT------IALP----PLISNPIYD------------NRAP-  172
_XP_036236540.1_   138  -VVRLCFQAFLPDEH-----------GNYS------IALP----PLISNPIYD------------NRAP-  172
_NWV50957.1_       138  -VVRLCFQAFLPDEH-----------GNYT------IALP----PLISNPIYD------------NRAP-  172
_NXT97287.1_       138  -VVRLCFQAFLPDEH-----------GNYT------IALP----PLISNPIYD------------NRAP-  172
_XP_023778876.1_   138  -VVRLCFQAFLPDEH-----------GNYT------IALP----PLISNPIYD------------NRAP-  172
_NXD42381.1_       138  -VVRLCFQAFLPDEH-----------GNYT------IALP----PLISNPIYD------------NRAP-  172
_NXK69295.1_       138  -VVRLCFQAFLPDEH-----------GNYT------IALP----PLISNPIYD------------NRAP-  172
_XP_049657510.1_   138  -VVRLCFQAFLPDEH-----------GNYT------LALP----PLISNPIYD------------NRAP-  172
_NXV62539.1_       138  -VVRLCFQAFLPDEH-----------GNYS------IALP----PLISNPIYD------------NRAP-  172
_NXB80231.1_       138  -VVRLCFQAFLPDEH-----------GNYT------IALP----PLISNPIYD------------NRAP-  172
_NWU15026.1_       138  -VVRLCFQAFLPDEQ-----------GNYT------IALP----PLISNPIYD------------NRAP-  172
_NXL26104.1_       138  -VVRLCFQAFLPDEH-----------GNYS------IALP----PLISNPIYD------------NRAP-  172
_NWW05001.1_       138  -VVRLCFQAFLPDEH-----------GNYT------IALP----PLISNPIYD------------NRAP-  172
_RLW07261.1_       138  -VVRLCFQAFLPDEH-----------GNYT------IALP----PLISNPIYD------------NRAP-  172
_NXR96625.1_       138  -VVRLCFQAFLPDEH-----------GNYT------IALP----PLISNPIYD------------NRAP-  172
_XP_021383204.1_   138  -VVRLCFQAFLPDEH-----------GNYT------IALP----PLISNPIYD------------NRAP-  172
_NXQ25764.1_       138  -VVRLCFQAFLPDEH-----------GNYT------IALP----PLISNPIYD------------NRAP-  172
_NXO08412.1_       138  -VVRLCFQAFLPDEL-----------GNYT------IALP----PLISNPIYD------------NRAP-  172
_NXI32280.1_       138  -VVRLCFQAFLPDEH-----------GNYT------IALP----PLISNPIYD------------NRAP-  172
```

```
_NXG00288.1_       138  -VVRLCFQAFLPDEH-----------GNYS------IALP----PLISNPIYD------------NRAP-  172
_XP_041329879.1_   138  -VVRLCFQAFLPDEH-----------GNYS------IALP----PLISNPIYD------------NRAP-  172
_NXV03759.1_       138  -VVRLCFQAFLPDEH-----------GNYT------MALP----PLISNPIYD------------NRAP-  172
_NWT16975.1_       138  -VVRLCFQAFLPDEH-----------GNYT------ITLP----PLISNPIYD------------NRAP-  172
_NWT67953.1_       138  -VVRLCFQAFLPDEH-----------GNYA------IALP----PLISNPIYD------------NRAP-  172
_XP_005042765.2_   138  -VVRLCFQAFLPDEH-----------GNYT------IALP----PLISNPIYD------------NRAP-  172
_XP_017600715.1_   138  -VVRLCFQAFLPDEH-----------GNYT------RALP----PLISNPIYD------------NRAP-  172
_NXD93279.1_       138  -VVRLCFQAFLPDEH-----------GNYT------IALP----PLISNPIYD------------NRAP-  172
_NXY02260.1_       138  -VVRLCFQAFLPDEH-----------GNYT------IALP----PLISNPIYD------------NRAP-  172
_NXO84677.1_       138  -VVRLCFQAFLPDEH-----------GNYT------RALP----PLISNPIYD------------NRAP-  172
_NXO64371.1_       138  -VVRLCFQAFLPDEH-----------GNYT------IALP----PLISNPIYD------------NRAP-  172
_NXO42022.1_       138  -VVRLCFQAFLPDEH-----------GNYT------IALP----PLISNPIYD------------NRAP-  172
_NWY65013.1_       138  -VVRLCFQAFLPDEH-----------GNYT------IALP----PLISNPIYD------------NRAP-  172
_NXP90563.1_       138  -VVRLCFQAFLPDEH-----------GNYS------IALP----PLISNPIYD------------NRAP-  172
_NWI73723.1_       138  -VVRLCFQAFLPDEH-----------GNYT------IALP----PLISNPIYD------------NRAP-  172
_NXH30511.1_       138  -VVRLCFQAFLPDEH-----------GNYT------IALP----PLISNPIYD------------NRAP-  172
_NWT77800.1_       138  -VVRLCFQAFLPDEH-----------GNYT------IALP----PLISNPIYD------------NRAP-  172
_NWT34209.1_       138  -VVRLCFQAFLPDEH-----------GNYS------IALP----PLISNPIYD------------NRAP-  172
_NWY29369.1_       138  -VVRLCFQAFLPDEH-----------GNYS------IALP----PLISNPIYD------------NRAP-  172
_NXQ73821.1_       138  -VVRLCFQAFLPDEH-----------GNYS------IALP----PLISNPIYD------------NRAP-  172
_XP_039572786.1_   138  -VVRLCFQAFLPDEH-----------GNYT------IALP----PLISNPIYD------------NRAP-  172
_XP_030802035.1_   138  -VVRLCFQAFLPDEH-----------GNYS------IALP----PLISNPIYD------------NRAP-  172
_NXE68211.1_       138  -VVRLCFQAFLPDKH-----------GTYS------IALP----PLISNPIYD------------NRAP-  172
_NXS80652.1_       138  -VVRLCFQAFLPDEH-----------GNYT------IALP----PLISNPIYD------------NRAP-  172
_NXO32431.1_       138  -VVRLCFQAFLPDEH-----------GNYT------IALP----PLISNPIYD------------NRAP-  172
_NWR17317.1_       138  -VVRLCFQAFLPDEH-----------GNYS------IALP----PLISNPIYD------------NRAP-  172
_NWZ89732.1_       138  -VVRLCFQAFLPDEH-----------GSYS------IALP----PLISNPIYD------------NRAP-  172
_XP_032039523.1_   138  -VVRLCFQAFLPDEH-----------GNYT------LALP----PLISNPIYD------------NRAP-  172
_KFO04362.1_       138  -VVRLCFQAFLPDEH-----------GNYT------LALP----PLISNPIYD------------NRAP-  172
_XP_054146213.1_   138  -VVRLCFQAFLPDEH-----------GNYS------IALP----PLISNPIYD------------NRAP-  172
_XP_005486916.2_   138  -VVRLCFQAFLPDEH-----------GNYS------IALP----PLISNPIYD------------NRAP-  172
_KAJ7422946.1_     138  -VVRLCFQAFLPDEH-----------GNYS------IALP----PLISNPIYD------------NRAP-  172
_NXC99388.1_       138  -VVRLCFQAFLPDEH-----------GNYT------IALP----PLISNPIYD------------NRAP-  172
_RMC07975.1_       138  -VVRLCFQAFLPDEH-----------GNYT------IALP----PLISNPIYD------------NRAP-  172
_NXT95159.1_        96  -VVRLCFQAFLPDEH-----------GNYT------IALP----PLISNPIYD------------NRAP-  130
_XP_009957589.1_    96  -VVRLCFQAFLPDEH-----------GNYT------LALP----PLISNPIYD------------NRAP-  130
_TRZ13602.1_        96  -VVRLCFQAFLPDEH-----------GNYT------IALP----PLISNPIYD------------NRAP-  130
_NWR45911.1_        87  -VVRLCFQAFLPDQH-----------GNYT------IALP----PLISNPIYD------------NRAP-  121
_KAF4799649.1_      96  -VVRLCFQAFLPDEH-----------GNYT------IALP----PLISNPIYD------------NRAP-  130
```

```
_KAJ7420138.1_     132  -VVRLCFQAFLPDEH-----------GNYT------IALP----PLISNPIYD------------NRAP-  166
_XP_054678501.1_   138  -VVRLCFQAFLPDEH-----------GNYT------LALP----PLISNPIYD------------N----  169
_NXP48685.1_       138  -VVRLCFQAFLPDEQ-----------GNYT------LALP----PLISNPIYD------------NRA--  171
_XP_010704701.1_    55  -VVRLCFQAFLPDEH-----------GNYT------LALP----PLISNPIYD------------NRAP-   89
_KFV10805.1_        47  -VVRLCFQAFLPDEH-----------GNYT------IALP----PLISNPIYD------------NRAP-   81
_XP_009572567.1_    17  -VVRLCFQAFLPDEH-----------GNYT------LALP----PLISNPIYD------------NRAP-   51
_NXF64277.1_        16  -VVRLCFQAFLPDEH-----------GNYT------LALP----PLISNPIYD------------NRAP-   50
_KFV54465.1_        16  -VVRLCFQAFLPDEH-----------GNYT------LALP----PLISNPIYD------------NRAP-   50
_KFV90717.1_        16  -VVRLCFQAFLPDEH-----------GNYT------LALP----PLISNPIYD------------NRAP-   50
_KFQ03674.1_        16  -VVRLCFQAFLPDEH-----------GNYT------LALP----PLISNPIYD------------NRAP-   50
_NXW01028.1_        16  -VVRLCFQAFLPDEH-----------GNYT------LALP----PLISNPIYD------------NRAP-   50
_KFP32908.1_        16  -VVRLCFQAFLPDQH-----------GNYT------LPLP----PLISNPIYD------------NRAP-   50
_KFV93389.1_        16  -VVRLCFQAFLPDEH-----------GNYT------LALP----PLISNPIYD------------NRAP-   50
_NWI63728.1_        16  -VVRLCFQAFLPDEH-----------GNYT------LALP----PLISNPIYD------------NRAP-   50
_KAI6072063.1_      17  -VVRLCFQAFLPDEH-----------GNYT------LALP----PLISNPIYD------------NRAP-   51
_KFQ58901.1_        16  -VVRLCFQAFLPDEH-----------GNYT------LALP----PLISNPIYD------------NRAP-   50
_NXP33753.1_        16  -VVRLCFQAFLPDEH-----------GNYT------IALP----PLISNPIYD------------NRAP-   50
_XP_009476353.1_    15  -VVRLCFQAFLPDEH-----------GNYT------LALP----PLISNPIYD------------NRAP-   49
_KFQ55903.1_        16  -VVRLCFQAFLPDEH-----------GNYT------LALP----PLISNPIYD------------NRAP-   50
_XP_042683349.1_    16  -VVRLCFQAFLPDEH-----------GNYT------LALP----PLISNPIYD------------NRAP-   50
_NXR08991.1_        16  -VVRLCFQAFLPDEH-----------GNYT------LALP----PLISNPIYD------------NRAP-   50
_XP_008947250.1_    16  -VVRLCFQAFLPDEH-----------GNYT------IALP----PLISNPIYD------------NRAP-   50
_KFP72464.1_        16  -VVRLCFQAFLPDEH-----------GNYT------LALP----PLISNPIYD------------NRAP-   50
_KFV60523.1_        16  -VVRLCFQAFLLDEH-----------GNYT------LALP----PLISNPIYD------------NRAP-   50
_NXT13165.1_        16  -VVRLCFQAFLPDEH-----------GNYA------IALP----PLISNPIYD------------NRAP-   50
_NWT50506.1_        16  -VVRLCFQAFLPDEH-----------GNYT------IALP----PLISNPIYD------------NRAP-   50
_OXB76139.1_        14  -VVRLCFQAFLPDEH-----------GNYT------LALP----PLISNPIYD------------NRAP-   48
_XP_050827907.1_    29  -VVRLCFQAFLPDEH-----------GNYS------IALP----PLISNPIYD------------NRAP-   63
_XP_030302545.1_    24  -VVRLCFQAFLPDEH-----------GNYT------LALP----PLISNPIYD------------NRAP-   58
_XP_054036038.1_    28  -VVRLCFQAFLPDEH-----------GNYT------LALP----PLISNPIYD------------NRAP-   62
_XP_012788337.1_   138  -VVRLCFQVFLPDER-----------GNLT------TALP----PIVSNPIYD------------NRAP-  172
_XP_008070154.1_   127  -VVRLCFQVFLPDEL-----------GNLT------TALP----PVVSNPIYD------------NRAP-  161
_XP_027477445.1_   127  -VVRLCFQVFLPDEH-----------GNLT------TALP----PVVSNPIYD------------NRAP-  161
_XP_025848473.1_   127  -VVRLCFQVFLPDEH-----------GNLT------TALP----PVVSNPIYD------------NRAP-  161
_XP_049640716.1_   138  -VVRLCFQVFLPDEC-----------GNLT------AALP----PIVSNPIYD------------NRAP-  172
_XP_023084089.1_   138  -VVRLCFQVFLPDEH-----------GNLT------TALP----PVVSNPIYD------------NRAP-  172
_EHH22143.1_       138  -VVRLCFQVFLPDEH-----------GNLT------TALP----PVVSNPIYD------------NRAP-  172
_XP_038504711.1_   138  -VVRLCFQVFLPDEH-----------GNLT------TALP----PVVSNPIYD------------NRAP-  172
_XP_038504703.1_   138  -VVRLCFQVFLPDEH-----------GNLT------TALP----PVVSNPIYD------------NRAP-  172
```

```
_XP_038504718.1_  138  -VVRLCFQVFLPDEH-----------GNLT------TALP----PVVSNPIYD------------NRAP-  172
_XP_048971856.1_  138  -VVRLCFQVFLPDEH-----------GNLT------TALP----PVVSNPIYD------------NRAP-  172
_KAB1266826.1_    138  -VVRLCFQVFLPDEH-----------GNLT------TALP----PLVSNPIYD------------NRAP-  172
_XP_048391424.1_  138  -VVRLCFQVFLPDEH-----------GSFS------RALP----PVVSNPIYD------------NRAP-  172
_XP_048391425.1_  138  -VVRLCFQVFLPDEH-----------GSFS------RALP----PVVSNPIYD------------NRAP-  172
_GCC28026.1_      138  -VVRLCFQVFLPDER-----------GSFS------RALP----PVVSNPIYD------------NRAP-  172
_XP_043551991.1_  138  -VVRLCFQVFLPDER-----------GSFS------RALP----PVVSNPIYD------------NRAP-  172
_XP_043551989.1_  138  -VVRLCFQVFLPDER-----------GSFS------RALP----PVVSNPIYD------------NRAP-  172
_XP_054534389.1_   48  -VVRLCFQVFLPDEH-----------GNLT------TALP----PVVSNPIYD------------NRAP-   82
_XP_011531312.1_   48  -VVRLCFQVFLPDEH-----------GNLT------TALP----PVVSNPIYD------------NRAP-   82
_XP_021780541.1_   47  -VVRLCFQVFLPDEH-----------GNLT------TALP----PVVSNPIYD------------NRAP-   81
_XP_037862912.1_   68  -VVRLCFQVFLPDEH-----------GNLT------TALP----PVVSNPIYD------------NRAP-  102
_XP_015413428.1_  127  -VVRLCFQVFLYDEH-----------GNLT------TTLP----PVVSNPIYD------------NRAP-  161
_XP_005884642.1_  127  -VVRLCFQVFLYDEH-----------GNLT------TTLP----PVVSNPIYD------------NRAP-  161
_KAF6105903.1_    127  -VVRLCFQVFLYDEH-----------GNLT------TTLP----PAVSNPIYD------------NRAP-  161
_XP_015413426.1_  127  -VVRLCFQVFLYDEH-----------GNLT------TTLP----PVVSNPIYD------------NRAP-  161
_XP_014387539.1_  127  -VVRLCFQVFLYDEH-----------GNLT------TTLP----PVVSNPIYD------------NRAP-  161
_XP_014444723.1_  127  -VVRLCFQVFLLDEH-----------GNLT------TALP----PVISNPIYD------------NRAP-  161
_XP_023448058.1_  127  -VVRLCFQVFLPDEH-----------GNLT------TALP----PVVSNPIYD------------NRAP-  161
_EGW11604.1_      127  -VVRLCFQVFLPDEH-----------GNLT------TALP----PVVSNPIYD------------NRAP-  161
_XP_015985129.1_  127  -VVRLCFQVFLYDEH-----------GNLT------TTLP----PVVSNPIYD------------NRAP-  161
_XP_012878014.1_  127  -VVRLCFQVFLPDEC-----------GNLT------KALP----PVVSNPIYD------------NRAP-  161
_KAG5211506.1_    106  -VVRLCFQVFLPDEH-----------GNLT------TALP----PVVSNPIYD------------NRAP-  140
_MXQ82539.1_      102  -VVRLCFQVFLPDEH-----------GNLT------TALP----PVVSNPIYD------------NRAP-  136
_XP_032729748.1_  122  -----------------------------------------------------------------NGAP-  125
_XP_032208113.1_  122  -----------------------------------------------------------------NGAP-  125
_XP_048208817.1_  138  -VVRLCFQVFLPDEC-----------GNLT------TALP----PVVSNPIYD-----------------  168
_XP_038200333.1_  138  -VVRLCFQVFLPDEH-----------GNLT------TALP----PIVSNPIYD-----------------  168
_P15307.2_        138  -VVRCVFMFFLPDED-----------GNFT------TALP----PIVSNPIYD------------NRAP-  172
_XP_041524635.1_  127  -VVRLCFQVFLSDEH-----------GNVT------TALP----PIVSNPIYD------------NRAP-  161
_CAA33843.1_      138  -VVRCVFMFFLPDED-----------GNFT------TAVP----PIVSNPIYD------------NRAP-  172
_XP_028732210.2_  127  -VVRLCFQVFLPDEH-----------GDLT------TALP----PVVSNPIYD------------NRAP-  161
_XP_025848474.1_  127  -VVRLCFQVFLPDEH-----------GNLT------TALP----PVVSNPIYD------------NRAP-  161
_EDL97996.1_      127  -VVRLCFQVFLPDEH-----------GNFT------TALP----PIVSNPIYD------------NRAP-  161
_KAI4577886.1_    127  -VVRLCFQVFLPDEH-----------GNLT------TALP----PVVSNPIYD------------NRAP-  161
_KAG8512309.1_    138  -VVRLCFQVFLPDEH-----------GNLT------TALP----PVVSNPIYD------------NRKYF  173
_XP_047637971.1_  138  -VVRLCFQVFLPDEH-----------GNLT------SALP----PVVSNPIYD------------N----  169
_XP_019491454.1_  138  -VVRLCFQVFLHDEH-----------GNLT------TALP----PVVSNPIYD------------NRAP-  172
_XP_043842482.1_  140  -VVRLCFQVFLLDEH-----------GNLT------SALP----PVVSNPIYD------------NRAP-  174
```

```
_XP_027721170.1_  140  -VVRLCFQVFLLDEH-----------GNLT------SALP----PVVSNPIYD------------NRAP-  174
_XP_036604632.1_  140  -VVRLCFQVFLLDEH-----------GNLT------SALP----PVVSNPIYD------------NRAP-  174
_XP_020841164.1_  140  -VVRLCFQVFLLDEH-----------GNLI------SALP----PVVSNPIYD------------NRAP-  174
_XP_051831295.1_  140  -VVRLCFQVFLLDEH-----------GNLT------SALP----PVVSNPIYD------------NRAP-  174
_XP_031806378.1_  140  -VVRLCFQVFLLDEH-----------GNLT------SALP----PVVSNPIYD------------NRAP-  174
_XP_051831294.1_  140  -VVRLCFQVFLLDEH-----------GNLT------SALP----PVVSNPIYD------------NRAP-  174
_XP_003339776.1_  140  -VVRLCFQVFLLDEH-----------GNLT------SALP----PVVSNPIYD------------NRAP-  174
_XP_044515413.1_  140  -VVRLCFQVFLLDEH-----------GNLT------SALP----PVVSNPIYD------------NRAP-  174
_XP_028928361.1_  140  -VVRLCFQVFLLDEH-----------GNLT------SALP----PVVSNPIYD------------NRAP-  174
_XP_038607403.1_  140  -VVRLCFQVFLLDEH-----------GNLT------SALP----PVVSNPIYD------------NRAP-  174
_XP_040849334.1_   16  -VVRLCFQVFLPDEH-----------GNLT------TALP----PVVSNPIYD------------NRAP-   50
_TKC46270.1_       14  -VVRLCFQVFLPDER-----------GNLT------TALH----PVVSNPIYD------------NRAP-   48
_OWK10634.1_       15  -VVRLCFQVFLPDEH-----------GNLA------TALP----PVVSNPIYD------------NRAP-   49
_XP_036190342.1_   14  -VVRLCFQVFLYDEH-----------GNLT------TTLP----PVVSNPIYD------------NRAP-   48
_KAB0395596.1_     11  -VVRLCFQVFLPDEH-----------GNLT------TALP----PIVSNPIYD------------NRAP-   45
_KAF6430170.1_     15  -VVRLCFQVFLYDEH-----------GNLT------TTLP----PVVSNPIYD------------NRAP-   49
_XP_038948811.1_   16  -VVRLCFQVFLPDEH-----------GNFT------TALP----PIVSNPIYD------------NRAP-   50
_KAB1266828.1_    138  -VVRLCFQVFLPDEH-----------GNLT------TALP----PLVSNPIYDNRKQLILFSSCICAP--  183
_NP_002899.1_     138  -VVRLCFQVFLPDEH-----------GNLT------TALP----PVVSNPIYDNR-----------AP--  172
_XP_001159537.3_  138  -VVRLCFQVFLPDEH-----------GNLT------TALP----PVVSNPIYDNR-----------AP--  172
_PNI66333.1_      138  -VVRLCFQVFLPDEH-----------GNLT------TALP----PVVSNPIYDNR-----------AP--  172
_EHH55592.1_      138  -VVRLCFQVFLPDEH-----------GNLT------TALP----PVVSNPIYDNR-----------AP--  172
_XP_003262470.1_  138  -VVRLCFQVFLPDEH-----------GNLT------TALP----PVVSNPIYDNR-----------AP--  172
_BAG64854.1_      138  -VVRLCFQVFLPDEH-----------GNLT------TALP----PVVSNPIYDNR-----------AP--  172
_XP_032034471.1_  138  -VVRLCFQVFLPDEH-----------GNLT------TALP----PVVSNPIYDNR-----------AP--  172
_XP_037862911.1_  138  -VVRLCFQVFLPDEH-----------GNLT------TALP----PVVSNPIYDNR-----------AP--  172
_XP_003830911.1_  138  -VVRLCFQVFLPDEH-----------GNLT------TALP----PVVSNPIYDNR-----------AP--  172
_XP_004029331.1_  138  -VVRLCFQVFLPDEH-----------GNLT------TALP----PVVSNPIYDNR-----------AP--  172
_XP_002812051.1_  138  -VVRLCFQVFLPDEH-----------GNLT------TALP----PVVSNPIYDNR-----------AP--  172
_XP_011711623.1_  138  -VVRLCFQVFLPDEH-----------GNLT------TALP----PVVSNPIYDNR-----------AP--  172
_XP_033095023.1_  138  -VVRLCFQVFLPDEH-----------GNLT------TALP----PVVSNPIYDNR-----------AP--  172
_XP_002799280.1_  138  -VVRLCFQVFLPDEH-----------GNLT------TALP----PVVSNPIYDNR-----------AP--  172
_XP_011853172.1_  138  -VVRLCFQVFLPDEH-----------GNLT------TALP----PVVSNPIYDNR-----------AP--  172
_XP_050609443.1_  138  -VVRLCFQVFLPDEH-----------GNLT------TALP----PVVSNPIYDNR-----------AP--  172
_XP_003908735.1_  138  -VVRLCFQVFLPDEH-----------GNLT------TALP----PVVSNPIYDNR-----------AP--  172
_XP_025209693.1_  138  -VVRLCFQVFLPDEH-----------GNLT------TALP----PVVSNPIYDNR-----------AP--  172
_XP_023084087.1_  138  -VVRLCFQVFLPDEH-----------GNLT------TALP----PVVSNPIYDNR-----------AP--  172
_XP_021780539.1_  138  -VVRLCFQVFLPDEH-----------GNLT------TALP----PVVSNPIYDNR-----------AP--  172
_XP_017704568.1_  138  -VVRLCFQVFLPDEH-----------GNLT------TALP----PVVSNPIYDNR-----------AP--  172
```

```
_XP_010375811.1_  138  -VVRLCFQVFLPDEH-----------GNLT------TALP----PVVSNPIYDNR-----------AP--  172
_XP_030776399.1_  138  -VVRLCFQVFLPDEH-----------GNLT------TALP----PVVSNPIYDNR-----------AP--  172
_XP_011797211.1_  138  -VVRLCFQVFLPDEH-----------GNLT------TALP----PVVSNPIYDNR-----------AP--  172
_XP_012332682.1_  138  -VVRLCFQVFLPDEH-----------GNLT------TALP----PVVSNPIYDNR-----------AP--  172
_XP_010331875.2_  138  -VVRLCFQVFLPDEH-----------GNLT------TALP----PVVSNPIYDNR-----------AP--  172
_XP_012332683.1_  138  -VVRLCFQVFLPDEH-----------GNLT------TALP----PVVSNPIYDNR-----------AP--  172
_XP_017400475.1_  138  -VVRLCFQVFLPDEH-----------GNLT------TALP----PVVSNPIYDNR-----------AP--  172
_XP_008568357.1_  138  -VVRLCFQVFLPDEH-----------GNLT------TALP----PVVSNPIYDNR-----------AP--  172
_XP_012620284.1_  138  -VVRLCFQVFLPDEH-----------GNLA------TVLP----PVVSNPIYDNR-----------AP--  172
_XP_002709741.2_  138  -VVRLCFQVFLPDEH-----------GNLT------TALP----PVVSNPIYDNR-----------AP--  172
_XP_022452943.1_  138  -VVRLCFQVFLPDER-----------GNLT------TALH----PVVSNPIYDNR-----------AP--  172
_XP_045405625.1_  138  -VVRLCFQVFLPDEH-----------GNLA------TVLP----PVVSNPIYDNR-----------AP--  172
_XP_004280707.1_  138  -VVRLCFQVFLPDER-----------GNLT------TALR----PIVSNPIYDNR-----------AP--  172
_XP_030733169.1_  138  -VVRLCFQVFLPDER-----------GNLT------TALR----PIVSNPIYDNR-----------AP--  172
_TEA23022.1_      138  -VVRLCFQVFLPDER-----------GNLT------TALR----PIVSNPIYDNR-----------AP--  172
_XP_012494629.1_  138  -VVRLCFQVFLPDEH-----------GNLA------TVLP----PVVSNPIYDNR-----------AP--  172
_XP_010950191.1_  138  -VVRLCFQVFLPDEH-----------GNLT------TALP----PLVSNPIYDNR-----------AP--  172
_XP_024608785.1_  138  -VVRLCFQVFLPDER-----------GNLT------TALR----PVVSNPIYDNR-----------AP--  172
_XP_006201637.1_  138  -VVRLCFQVFLPDEH-----------GNLT------TALP----PLVSNPIYDNR-----------AP--  172
_XP_019285354.1_  138  -VVRLCFQVFLPDEH-----------GNLT------TALP----PVVSNPIYDNR-----------AP--  172
_XP_032509224.1_  138  -VVRLCFQVFLPDER-----------GNLT------TALR----PVVSNPIYDNR-----------AP--  172
_XP_047379355.1_  138  -VVRLCFQVFLPDEL-----------GNLT------TALP----PVVSNPIYDNR-----------AP--  172
_XP_032981134.1_  138  -VVRLCFQVFLHDEH-----------GNLT------TTLP----PVVSNPIYDNR-----------AP--  172
_XP_005600018.1_  138  -VVRLCFQVFLPDEH-----------GNLT------TALP----PVVSNPIYDNR-----------AP--  172
_XP_046517051.1_  138  -VVRLCFQVFLPDEH-----------GNLT------TALP----PVVSNPIYDNR-----------AP--  172
_XP_007464205.1_  138  -VVRLCFQVFLPDER-----------GNLT------TAVP----PVVSNPIYDNR-----------AP--  172
_XP_019572903.1_  138  -VVRLCFQVFLHDEH-----------GNLT------TTLP----PVVSNPIYDNR-----------AP--  172
_XP_007189755.1_  138  -VVRLCFQVFLPDEH-----------GNLT------TALP----PIVSNPIYDNR-----------AP--  172
_XP_004397771.1_  138  -VVRLCFQVFLPDEH-----------GNLT------TALP----PVVSNPIYDNR-----------AP--  172
_XP_004435713.1_  138  -VVRLCFQVFLPDEH-----------GNLT------TALP----PVVSNPIYDNR-----------AP--  172
_XP_008698124.1_  138  -VVRLCFQVFLPDEH-----------GNLT------TALP----PVVSNPIYDNR-----------AP--  172
_XP_023989255.1_  138  -VVRLCFQVFLPDEH-----------GNLT------TALP----PVVSNPIYDNR-----------AP--  172
_XP_011233012.2_  138  -VVRLCFQVFLPDEH-----------GNLT------TALP----PVVSNPIYDNR-----------AP--  172
_XP_025724444.1_  138  -VVRLCFQVFLPDEH-----------GNLT------TALP----PVVSNPIYDNR-----------AP--  172
_XP_005626186.1_  138  -VVRLCFQVFLPDEH-----------GNLT------TALP----PVVSNPIYDNR-----------AP--  172
_XP_045643991.1_  138  -VVRLCFQVFLPDEH-----------GNLT------TALP----PVVSNPIYDNR-----------AP--  172
_XP_034857383.1_  138  -VVRLCFQVFLPDEH-----------GNLT------TALP----PVVSNPIYDNR-----------AP--  172
_XP_027945501.1_  138  -VVRLCFQVFLPDEH-----------GNLT------TALP----PVVSNPIYDNR-----------AP--  172
_XP_026341094.1_  138  -VVRLCFQVFLPDEH-----------GNLT------TALP----PVVSNPIYDNR-----------AP--  172
```

```
_EFB29923.1_       138  -VVRLCFQVFLPDEH-----------GNLT------TALP----PVVSNPIYDNR-----------AP--  172
_XP_045740897.1_   138  -VVRLCFQVFLPDEH-----------GNLT------TALP----PVVSNPIYDNR-----------AP--  172
_CAD7677454.1_     138  -VVRLCFQVFLPDEH-----------GNLT------TALP----PVVSNPIYDNR-----------AP--  172
_XP_035577553.1_   138  -VVRLCFQVFLPDEH-----------GNLT------TALP----PVVSNPIYDNR-----------AP--  172
_XP_006733255.1_   138  -VVRLCFQVFLPDEH-----------GNLT------TALP----PVVSNPIYDNR-----------AP--  172
_XP_032266820.1_   138  -VVRLCFQVFLPDEH-----------GNLT------TALP----PVVSNPIYDNR-----------AP--  172
_XP_041611730.1_   138  -VVRLCFQVFLPDEH-----------GNLT------TALP----PVVSNPIYDNR-----------AP--  172
_XP_048971854.1_   138  -VVRLCFQVFLPDEH-----------GNLT------TALP----PVVSNPIYDNR-----------AP--  172
_XP_021554840.1_   138  -VVRLCFQVFLPDEH-----------GNLT------TALP----PVVSNPIYDNR-----------AP--  172
_VCW78562.1_       138  -VVRLCFQVFLPDEH-----------GNLT------MALP----PVVSNPIYDNR-----------AP--  172
_XP_034857381.1_   138  -VVRLCFQVFLPDEH-----------GNLT------TALP----PVVSNPIYDNR-----------AP--  172
_XP_008524231.1_   135  -VVRLCFQVFLPDEH-----------GNLT------TALP----PVVSNPIYDNR-----------AP--  169
_XP_045740895.1_   138  -VVRLCFQVFLPDEH-----------GNLT------TALP----PVVSNPIYDNR-----------AP--  172
_XP_045834746.1_   138  -VVRLCFQVFLPDEH-----------GNLT------MALP----PVVSNPIYDNR-----------AP--  172
_XP_035932906.1_   138  -VVRLCFQVFLPDEH-----------GNLT------TALP----PVVSNPIYDNR-----------AP--  172
_XP_030883859.1_   138  -VVRLCFQVFLPDEH-----------GNLT------TALP----PVVSNPIYDNR-----------AP--  172
_XP_032266818.1_   138  -VVRLCFQVFLPDEH-----------GNLT------TALP----PVVSNPIYDNR-----------AP--  172
_XP_024408528.2_   138  -VVRLCFQVFLYDEH-----------GNLT------TTLP----PVVSNPIYDNR-----------AP--  172
_XP_004580347.1_   138  -VVRLCFQVFLPDEH-----------GNLT------TALP----PVVSNPIYDNR-----------AP--  172
_XP_030166525.1_   138  -VVRLCFQVFLPDEH-----------GNLT------TALP----PVVSNPIYDNR-----------AP--  172
_XP_035932904.1_   138  -VVRLCFQVFLPDEH-----------GNLT------TALP----PVVSNPIYDNR-----------AP--  172
_XP_020040690.1_   138  -VVRLCFQVFLPDEH-----------GNLT------TTLP----PVVSNPIYDNR-----------AP--  172
_XP_053445604.1_   138  -VVRLCFQVFLPDEH-----------GNLT------TALP----PVVSNPIYDNR-----------AP--  172
_XP_049507482.1_   138  -VVRLCFQVFLPDEH-----------GNLT------TALP----PVVSNPIYDNR-----------AP--  172
_XP_015335127.1_   138  -VVRLCFQVFLPDEL-----------GNLT------TALP----PVVSNPIYDNR-----------AP--  172
_XP_027790245.1_   138  -VVRLCFQVFLPDEL-----------GNLT------TALP----PVVSNPIYDNR-----------AP--  172
_XP_003984077.1_   138  -VVRLCFQVFLPDEH-----------GNLT------TALP----PVVSNPIYDNR-----------AP--  172
_XP_039096259.1_   138  -VVRLCFQVFLPDEH-----------GNLT------TALP----PVVSNPIYDNR-----------AP--  172
_XP_007075434.1_   138  -VVRLCFQVFLPDEH-----------GNLT------TALP----PVVSNPIYDNR-----------AP--  172
_XP_022360130.1_   138  -VVRLCFQVFLPDEH-----------GNLT------MALP----PVVSNPIYDNR-----------AP--  172
_XP_047707378.1_   138  -VVRLCFQVFLPDEH-----------GNLT------TALP----PVVSNPIYDNR-----------AP--  172
_XP_026243031.1_   138  -VVRLCFQVFLPDEL-----------GNLT------TALP----PVVSNPIYDNR-----------AP--  172
_XP_037383340.1_   138  -VVRLCFQVFLPDAH-----------GNLT------TALP----PVVSNPIYDNR-----------AP--  172
_XP_005322095.1_   138  -VVRLCFQVFLPDEL-----------GNLT------TALP----PVVSNPIYDNR-----------AP--  172
_XP_005662584.1_   138  -VVRLCFQVFLPDEH-----------GNLT------SALP----PVVSNPIYDNR-----------AP--  172
_XP_040080101.1_   138  -VVRLCFQVFLPDDH-----------GNLT------TALP----PVVSNPIYDNR-----------AP--  172
_XP_017503999.1_   138  -VVRLCFQVFLPDEH-----------GNLT------TALP----PVVSNPIYDNR-----------AP--  172
_XP_044119954.1_   138  -VVRLCFQVFLPDEH-----------GNLT------MALP----PVVSNPIYDNR-----------AP--  172
_XP_032208110.1_   138  -VVRLCFQVFLPDEH-----------GNLT------MALP----PVVSNPIYDNR-----------AP--  172
```

_XP_012586936.1_	138	-VVRLCFQVFLPDEH-----------GNLT------TALP----PVVSNPIYDNR-----------AP--	172
_XP_032729745.1_	138	-VVRLCFQVFLPDEH-----------GNLT------MALP----PVVSNPIYDNR-----------AP--	172
_XP_052504199.1_	138	-VVRLCFQVFLPDEH-----------GNLT------TALP----PVVSNPIYDNR-----------AP--	172
_XP_054431389.1_	138	-VVRLCFQVFLYDEH-----------GNLT------TTLP----PIVSNPIYDNR-----------AP--	172
_XP_023576632.1_	138	-VVRLCFQVFLPDEH-----------GNLT------TALP----PVVSNPIYDNR-----------AP--	172
_XP_004742070.1_	138	-VVRLCFQVFLPDEH-----------GNLT------MALP----PVVSNPIYDNR-----------AP--	172
_XP_037663267.1_	138	-VVRLCFQVFLPDEH-----------GNLT------TALP----PVVSNPIYDNR-----------AP--	172
_XP_004005978.1_	138	-VVRLCFQVFLPDEH-----------GNLT------TALP----PVVSNPIYDNR-----------AP--	172
_XP_036781908.1_	138	-VVRLCFQVFLPDEH-----------GNLT------AALP----PVVSNPIYDNR-----------AP--	172
_XP_040849333.1_	138	-VVRLCFQVFLPDEH-----------GNLT------TALP----PVVSNPIYDNR-----------AP--	172
_XP_005686691.1_	138	-VVRLCFQVFLPDEH-----------GNLT------TALR----PVVSNPIYDNR-----------AP--	172
_XP_023367900.1_	138	-VVRLCFQVFLPDEH-----------GNLT------TALP----PVVSNPIYDNR-----------AP--	172
KAF0879576.1	138	-VVRLCFQVFLPDEH-----------GNLT------TALP----PVVSNPIYDNR-----------AP--	172
_XP_029793284.1_	138	-VVRLCFQVFLPDEH-----------GNLT------TALP----PVVSNPIYDNR-----------AP--	172
_NP_001179899.1_	138	-VVRLCFQVFLPDEH-----------GNLT------TALP----PVVSNPIYDNR-----------AP--	172
_XP_020764718.1_	138	-VVRLCFQVFLPDEH-----------GNLA------TALP----PVVSNPIYDNR-----------AP--	172
_XP_054431388.1_	138	-VVRLCFQVFLYDEH-----------GNLT------TTLP----PIVSNPIYDNR-----------AP--	172
_XP_037663265.1_	138	-VVRLCFQVFLPDEH-----------GNLT------TALP----PVVSNPIYDNR-----------AP--	172
_XP_019825763.1_	138	-VVRLCFQVFLPDEH-----------GNLT------TALP----PVVSNPIYDNR-----------AP--	172
_XP_005889096.1_	138	-VVRLCFQVFLPDEH-----------GNLT------TALP----PVVSNPIYDNR-----------AP--	172
_XP_043324571.1_	138	-VVRLCFQVFLPDEH-----------GNLA------TALP----PVVSNPIYDNR-----------AP--	172
KAJ1078272.1	138	-VVRLCFQVFLPDEH-----------GNLT------TALR----PVVSNPIYDNR-----------AP--	172
_XP_036190339.1_	138	-VVRLCFQVFLYDEH-----------GNLT------TTLP----PVVSNPIYDNR-----------AP--	172
_XP_013005522.1_	138	-VVRLCFQVFLPDEH-----------GNLT------TALP----PVVSNPIYDNR-----------AP--	172
_XP_006073936.1_	138	-VVRLCFQVFLPDEH-----------GNLT------TALP----PVVSNPIYDNR-----------AP--	172
_XP_016069529.1_	138	-VVRLCFQVFLYDDH-----------GNLT------TTLP----PVVSNPIYDNR-----------AP--	172
KAB0354136.1	138	-VVRLCFQVFLPDEH-----------GNLA------TALP----PVVSNPIYDNR-----------AP--	172
_XP_029424871.1_	138	-VVRLCFQVFLPDEH-----------GNLT------TALP----PVVSNPIYDNR-----------AP--	172
_XP_036118792.1_	138	-VVRLCFQVFLYDEH-----------GNLT------TTLP----PVVSNPIYDNR-----------AP--	172
_XP_037019725.2_	137	-VVRLCFQVFLYDEH-----------GNLT------TTLP----PAVSNPIYDNR-----------AP--	171
_XP_047602572.1_	138	-VVRLCFQVFLPDEH-----------GNLT------MALP----PVVSNPIYDNR-----------AP--	172
_XP_014586639.1_	138	-VVRLCFQVFLPDEH-----------GNLT------TALP----PVVSNPIYDNR-----------AP--	172
_XP_046517052.1_	138	-VVRLCFQVFLPDEH-----------GNLT------TALP----PVVSNPIYDNR-----------AP--	172
_XP_006909635.2_	138	-VVRLCFQVFLYDEH-----------GNLT------TTLP----PVVSNPIYDNR-----------AP--	172
_XP_011362222.1_	138	-VVRLCFQVFLYDEH-----------GNLT------TTLP----PVVSNPIYDNR-----------AP--	172
_XP_045684409.1_	138	-VVRLCFQVFLYDEH-----------GNLT------TTLP----PVVSNPIYDNR-----------AP--	172
_XP_048208816.1_	138	-VVRLCFQVFLPDEC-----------GNLT------TALP----PVVSNPIYDNR-----------AP--	172
_XP_052590629.1_	138	-VVRLCFQVFLPDEH-----------GNLT------TALP----PVVSNPIYDNR-----------AP--	172
_XP_032353499.1_	138	-VVRLCFQVFLPDEH-----------GNLT------TALP----PLVSNPIYDNR-----------AP--	172

_XP_038200327.1_	138	-VVRLCFQVFLPDEH-----------GNLT------TALP----PIVSNPIYDNR-----------AP--	172
_XP_051056550.1_	138	-VVRLCFQVFLPDEH-----------GNLT------TALP----PVVSNPIYDNR-----------AP--	172
_XP_012805850.2_	138	-VVRLCFQVFLPDEH-----------GHLT------TALP----PIVSNPIYDNR-----------AP--	172
_XP_016050906.1_	138	-VVRLCFQVFLPDEH-----------GNLT------TALP----PIISNPIYDNR-----------AP--	172
_XP_030166526.1_	138	-VVRLCFQVFLPDEH-----------GNLT------TALP----PVVSNPIYDNR-----------AP--	172
_XP_036903878.1_	137	-VVRLCFQVFLYDEH-----------GDLT------TTLP----PAVSNPIYDNR-----------AP--	171
CAH7004032.1	138	-VVRLCFQVFLPDEH-----------GNLT------TALP----PVVSNPIYDNR-----------AP--	172
_XP_051056549.1_	138	-VVRLCFQVFLPDEH-----------GNLT------TALP----PVVSNPIYDNR-----------AP--	172
_XP_042547166.1_	138	-VVRLCFQVFLPDEC-----------GNLT------KALP----PVVSNPIYDNR-----------AP--	172
_XP_054584456.1_	138	-VVRLCFQVFLYDEH-----------GNLT------TTLP----PVVSNPIYDNR-----------AP--	172
_XP_049996768.1_	138	-VVRLCFQVFLPDEH-----------GNLT------TALP----PIVSNPIYDNR-----------AP--	172
_XP_027243805.1_	138	-VVRLCFQVFLPDEH-----------GNLT------TALP----PVVSNPIYDNR-----------AP--	172
_XP_047707379.1_	138	-VVRLCFQVFLPDEH-----------GNLT------TALP----PVVSNPIYDNR-----------AP--	172
_XP_026645370.1_	138	-VVRLCFQVFLLDEH-----------GNVT------TALP----PIVSNPIYDNR-----------AP--	172
_XP_014302519.1_	138	-VVRLCFQVFLYDEH-----------GNLT------TTLP----PVVSNPIYDNR-----------AP--	172
_XP_049726303.1_	138	-VVRLCFQVFLPDEH-----------GDLT------TALT----PVVSNPIYDNR-----------AP--	172
_XP_049726302.1_	138	-VVRLCFQVFLPDEH-----------GDLT------TALT----PVVSNPIYDNR-----------AP--	172
_XP_012969026.1_	138	-VVRLCFQVFLPDEH-----------GNLT------TALP----PVVSNPIYDNR-----------AP--	172
_XP_008524232.1_	135	-VVRLCFQVFLPDEH-----------GNLT------TALP----PVVSNPIYDNR-----------AP--	169
_XP_051020904.1_	138	-VVRLCFQVFLLDEH-----------GNLT------TALP----PIVSNPIYDNR-----------AP--	172
_XP_023590712.1_	138	-VVRLCFQVFLPDEH-----------GNLT------TALT----PVVSNPIYDNR-----------AP--	172
_XP_027243803.1_	138	-VVRLCFQVFLPDEH-----------GNLT------TALP----PVVSNPIYDNR-----------AP--	172
_XP_040589151.1_	138	-VVRLCFQVFLPDEH-----------GNLT------TALP----PVVSNPIYDNR-----------AP--	172
_XP_023412959.1_	138	-VVRLCFQVFLPDEH-----------GDLT------TALT----PVVSNPIYDNR-----------AP--	172
_XP_036057111.1_	138	-VVRLCFQVFLPDEH-----------GNLT------IALP----PVVSNPIYDNR-----------AP--	172
_XP_027822602.1_	138	-VVRLCFQVFLPDEH-----------GNLT------TALP----PVVSNPIYDNR-----------AP--	172
_XP_031322949.1_	138	-VVRLCFQVFLPDEH-----------GNLT------TALP----PLVSNPIYDNR-----------AP--	172
_XP_028634966.1_	138	-VVRLCFQVFLPDEH-----------GNFT------TALP----PIVSNPIYDNR-----------AP--	172
_XP_031531529.1_	138	-VVRLCFQVFLPDEH-----------GNLT------TALP----PLVSNPIYDNR-----------AP--	172
_XP_006073937.1_	138	-VVRLCFQVFLPDEH-----------GNLT------TALP----PVVSNPIYDNR-----------AP--	172
_XP_034364887.1_	138	-VVRLCFQVFLPDEH-----------GNFT------TALP----PIVSNPIYDNR-----------AP--	172
_XP_021482246.1_	138	-VVRLCFQVFLPDEH-----------GNLT------TALP----PVVSNPIYDNR-----------AP--	172
KAF4016326.1	138	-VVRLCFQVFLPDEH-----------GNLA------TALP----PVVSNPIYDNR-----------AP--	172
_XP_023602319.1_	138	-VVRLCFQVFLYDEH-----------GNLT------TTLP----PVVSNPIYDNR-----------AP--	172
_XP_052054164.1_	138	-VVRLCFQVFLPDEL-----------GNFT------TALP----PIVSNPIYDNR-----------AP--	172
_XP_036291310.1_	138	-VVRLCFQVFLYDEH-----------GNLT------TTLP----PVVSNPIYDNR-----------AP--	172
_XP_013370168.1_	138	-VVRLCFQVFLPDEH-----------GNLT------TALP----PVVSNPIYDNR-----------AP--	172
_XP_023590722.1_	138	-VVRLCFQVFLPDEH-----------GNLT------TALT----PVVSNPIYDNR-----------AP--	172
_XP_048291328.1_	138	-VVRLCFQVFLLDEH-----------GNLT------TALP----PIVSNPIYDNR-----------AP--	172

```
_XP_032772910.1_  138  -VVRLCFQVFLPDEH-----------GNFT------TALP----PIVSNPIYDNR-----------AP--  172
_XP_008768670.2_  138  -VVRLCFQVFLPDEH-----------GNFT------TALP----PIVSNPIYDNR-----------AP--  172
_XP_014302521.1_  138  -VVRLCFQVFLYDEH-----------GNLT------TTLP----PVVSNPIYDNR-----------AP--  172
_XP_031194882.1_  138  -VVRLCFQVFLPDEH-----------GNFT------AALP----PIVSNPIYDNR-----------AP--  172
_NP_033070.2_     138  -VVRLCFQVFLPDEH-----------GNFT------TALP----PIVSNPIYDNR-----------AP--  172
_XP_036291312.1_  138  -VVRLCFQVFLYDEH-----------GNLT------TTLP----PVVSNPIYDNR-----------AP--  172
_XP_021033157.1_  138  -VVRLCFQVFLPDEH-----------GNFT------TALP----PIVSNPIYDNR-----------AP--  172
_XP_015849136.1_  138  -VVRLCFQVFLPDEH-----------GNLT------TALP----PVVSNPIYDNR-----------AP--  172
_XP_045150027.1_  138  -VVRLCFQVFLPDEH-----------GNLT------TALA----PVVSNPIYDNR-----------AP--  172
_XP_021066684.1_  138  -VVRLCFQVFLPDEH-----------GNFT------TALP----PIVSNPIYDNR-----------AP--  172
_XP_006881088.1_  138  -VVRLCFQVFLPDEH-----------GNLT------AALP----PVFSNPIYDNR-----------AP--  172
_XP_008524234.1_  135  -VVRLCFQVFLPDEH-----------GNLT------TALP----PVVSNPIYDNR-----------AP--  169
_XP_021482260.1_  138  -VVRLCFQVFLPDEH-----------GNLT------TALP----PVVSNPIYDNR-----------AP--  172
_XP_007125500.1_  138  -VVRLCFQVFLPDEH-----------GNLT------TALP----PVVSNPIYDNR-----------AP--  172
_XP_028352731.1_  138  -VVRLCFQVFLPDEH-----------GNLT------TALP----PVVSNPIYDNR-----------AP--  172
_XP_028352733.1_  138  -VVRLCFQVFLPDEH-----------GNLT------TALP----PVVSNPIYDNR-----------AP--  172
_XP_028352734.1_  138  -VVRLCFQVFLPDEH-----------GNLT------TALP----PVVSNPIYDNR-----------AP--  172
_XP_028352736.1_  138  -VVRLCFQVFLPDEH-----------GNLT------TALP----PVVSNPIYDNR-----------AP--  172
_XP_028352735.1_  138  -VVRLCFQVFLPDEH-----------GNLT------TALP----PVVSNPIYDNR-----------AP--  172
_ERE88482.1_      138  -VVRLCFQVFLPDEH-----------GNLT------TALP----PVVSNPIYDNR-----------AP--  172
_KAF3826750.1_     17  -VVRLCFQVFLPDEH-----------GNLT------TALP----PVVSNPIYD------------NRAP-   51
_OBS70420.1_      127  -VVRLCFQVFLPDEH-------------------------------------------------------  140
_XP_006878094.1_  101  -VVRLCFQVFLPDEH-----------GNLT------TVLT----PVVSNPIYD------------NRAP-  135
_XP_008978905.3_   95  -VVRLCFQVFLPDEH-----------GNLT------TALP----PVVSNPIYD------------NRAP-  129
_XP_032147941.1_   95  -VVRLCFQVFLPDEH-----------GNLT------TALP----PVVSNPIYD------------NRAP-  129
_XP_008524233.1_   97  -VVRLCFQVFLPDEH-----------GNLT------TALP----PVVSNPIYD------------NRAP-  131
_XP_044628438.1_   95  -VVRLCFQVFLPDEH-----------GNLT------TALP----PVVSNPIYD------------NRAP-  129
_XP_010608101.1_   95  -VVRLCFQVFLPDEH-----------GNLT------TALP----PVVSNPIYD------------NRAP-  129
_KAF5918007.1_     97  -VVRLCFQVFLPDEH-----------GNLT------TALP----PVVSNPIYD------------NRAP-  131
_XP_021101942.1_   95  -VVRLCFQVFLPGEH-----------GNLT------TVLP----PVVSNPIYD------------NRAP-  129
_ABM89308.1_       14  -VVRLCFQVFLPDEH-----------GNLT------TALP----PVVSNPIYD------------NRAP-   48
_MBW00678.1_       30  -----------------------------------------------------------------NGAP-   33
_NP_001096177.1_  138  -VVRLCFQVFLPDEH-----------GSYT------RALP----PVVSNPIYD------------NRAP-  172
_OCT77375.1_      138  -VVRLCLQVFLPDEH-----------GNYT------RALT----PVVSNPIYD------------NRAP-  172
_AAI70187.1_      138  -VVRLCLQVFLPDEH-----------GNYT------RALT----PVVSNPIYD------------NRAP-  172
_AAI69362.1_      138  -VVRLCFQVFLPDEH-----------GSYT------RALG----PVVSNPIYD------------NRAP-  172
_XP_041418586.1_  138  -VVRLCFQVFLPDEH-----------GSYT------RALG----PVVSNPIYD------------NRAP-  172
_XP_041418585.1_  138  -VVRLCFQVFLPDEH-----------GSYT------RALG----PVVSNPIYD------------NRAP-  172
_XP_041418587.1_  138  -VVRLCFQVFLPDEH-----------GSYT------RALG----PVVSNPIYD------------NRAP-  172
```

```
_XP_053316221.1_  138  -VVRLCFQVFLPDEH-----------GNYT------RALA----PVVSNPIYD------------NRAP-  172
_XP_053568018.1_  138  -VVRLCFEVFLADEH-----------GNYT------HALA----PAVSNPIYD------------NRAP-  172
_KAG8443975.1_    138  -VVRLCFQVFLADKH-----------GNYT------RALQ----PVISNPIYD------------NRAP-  172
_XP_044145067.1_  140  -VVRLCFQVFLADEH-----------GNYT------RALH----PVVSNPIYD------------NRAP-  174
_KAG9484268.1_    140  -VVRLCFQVFLADEH-----------GNYT------RALH----PIVSNPIYD------------NRAP-  174
_XP_040285660.1_  140  -VVRLCFQVFLADEH-----------GNYT------RALH----PVVSNPIYD------------NRAP-  174
_XP_040285659.1_  140  -VVRLCFQVFLADEH-----------GNYT------RALH----PVVSNPIYD------------NRAP-  174
_KAG8583825.1_    140  -VVRLCFQVFLADEH-----------GNFT------RALH----PVVSNPIYD------------NRAP-  174
_XP_018411072.1_  140  -VVRLCFQVYLADEQ-----------GNYT------RALV----PVVSNPIYD------------NRAP-  174
_XP_040207073.1_  140  -VVRLCFQVFLADEQ-----------GKYT------RALT----PIVSNPIYD------------NRAP-  174
_NP_001081750.1_  136  -VVRLCLQVFLPDEH-----------GNYT------RALT----PVVST-IFD------------NRAP-  169
_PIO39092.1_       16  -VVRLCFQVFLADEQ-----------GKYT------RALT----PVVSNPIYD------------NRAP-   50
_PIO39091.1_       16  -VVRLCFQVFLADEQ-----------GKYT------RALT----PVVSNPIYD------------NRAP-   50
_XP_041035877.1_  137  -VVRLCFQVFLPNEV-----------GTCN------VPLL----PVVSNPIYD------------NRAP-  171
_XP_042202691.1_  137  -VVKLCFQVFIPDPS-----------GRCT------VPLT----PVVSNPIYD------------NRAP-  171
_XP_048475509.1_  126  -VVRLCFQVFLPNKL-----------GVCN------VPLP----PVVSNPIYD------------NRAP-  160
_XP_048380947.1_  126  -VVRLCFQVFLPNKL-----------GVCN------VPLP----PVVSNPIYD------------NRAP-  160
_XP_051901152.1_  130  -VVRLCFQVFLPNEM-----------GICS------VPLQ----PVVSNPIYD------------NRAP-  164
_BAD60879.1_      123  -SVRLCFQVFLPDAN-----------KKFT------HIVQ----PIVSQPIID------------KKS--  156
_XP_050405370.1_  135  -AVRLCFQVFLPDSN-----------GKIT------KVLQ----PVVSHAIHD------------KKA--  168
_XP_009063713.1_  135  -VVRLCFQVFLPDGS-----------GKIT------KVLQ----PVVSQPIHD------------KKA--  168
_CAB0044847.1_    153  -CVRMCFQVFLKNKVT----------QKFD------VMLD----PVVSEPILD------------TSA--  187
_XP_048259911.1_  137  -VVRLCFQVFLPDAS-----------GKIT------RVVP----PVVSQAIHD------------KKS--  170
_ADI72431.1_      137  -VVRLCFQVFLPDAS-----------GKIT------RVVP----PVVSQAIHD------------KKS--  170
_CAG5133522.1_    138  -VVRLCFQVFLPNEH-----------GKVT------RVVT----PVVSQPIHD------------KKS--  171
_KAG5688355.1_     48  -VVRLCFQVFLPDVN-----------GKIT------RVVP----PVVSLSIHD------------KKA--   81
_AMT84567.1_      136  -VVRLCFQVFLPDEK-----------NKYT------RIVD----PVVSQPIFD------------KKA--  169
_XP_052276378.1_  135  -VVRLCFQVFLPDEQ-----------GKIT------RIVK----PVVSQAIHD------------KKA--  168
_XP_052276377.1_  135  -VVRLCFQVFLPDEQ-----------GKIT------RIVK----PVVSQAIHD------------KKA--  168
_AAK72690.1_      137  -VVRLCFQVFLPDEN-----------GKIT------RIVP----PVVSHCIHD------------KKS--  170
_XP_011414731.2_  137  -VVRLCFQVFLPDEN-----------GKIT------RIVP----PVVSHCIHD------------KKS--  170
_XP_052699807.1_  137  -VVRLCFQVFLPDEN-----------GKIT------RIVP----PVVSHCIHD------------KKS--  170
_NP_001292261.1_  137  -VVRLCFQVFLPDEN-----------GKIT------RIVP----PVVSHCIHD------------KKS--  170
_XP_048738054.1_  137  -VVRLCFQVFLPDEN-----------GKIT------RIVP----PVVSHGIHD------------KKS--  170
_XP_052699808.1_  137  -VVRLCFQVFLPDEN-----------GKIT------RIVP----PVVSHCIHD------------KKS--  170
_XP_022332457.1_  137  -VVRLCFQVFLPDEN-----------GKIT------RIVP----PVVSQCIHD------------KKA--  170
_ABL63469.1_      136  -VVRLCFQVFLPDDQ-----------GKIT------RIVP----PVVSHPIHD------------KKS--  169
_XP_033733822.1_  137  -VVRLCFQVFLPDES-----------GKIS------RIVP----PVVSLPIHD------------KKA--  170
_XP_021347355.1_  137  -VVRLCFQVFLPDES-----------GKIS------RIVP----PVVSLPIHD------------KKA--  170
```

```
_UOA58607.1_       137  -VVRLCFQVFLPDES-----------GKIS------RIVP----PVVSLPIHD------------KKA--  170
_XP_021347356.1_   137  -VVRLCFQVFLPDES-----------GKIS------RIVP----PVVSLPIHD------------KKA--  170
_CAG2186872.1_     136  -VVRLCFQVFLPDEN-----------GKIT------RIVP----PVVSHPIHD------------KKS--  169
_XP_052058915.1_   136  -VVRLCFQVFLPDEN-----------GKIT------RIVP----PVVSHPIHD------------KKS--  169
_KAJ8309232.1_     137  -VVRLCFQVFLPDEN-----------GKIT------RIIP----PVVSHPIHD------------KKS--  170
_UZH84475.1_       135  -VVRLCFQVFLPDET-----------GKIT------RIVP----PVVSQCIHD------------KKA--  168
_ALA99939.1_       135  -VVRLCFQVFLPDDQ-----------GKIT------RIVP----PVVSHPIHD------------KKA--  168
_XP_052767764.1_   135  -VVRLCFQVFLPDEQ-----------GKIT------RIVQ----PVVSQAIHD------------KKA--  168
_ADD25211.1_       137  -VVRLCFQVFLPDES-----------GKIS------RIVP----PVVSLPIHD------------KKA--  170
_XP_052224245.1_   135  -VVRLCFQVFLPDEQ-----------GKIT------GIVK----PVVSQAIHD------------KKA--  168
_KAJ8314234.1_     122  ------------------------------------------------------------------KS--  123
_VDH95719.1_        95  -VVRLCFQVFLPDEN-----------GKIT------RIVP----PVVSHPIHD------------KKS--  128
_XP_041352913.1_   137  -VVRLCFQVFLPDAN-----------GKIT------RVVP----PVTSQVIHD------------KKS--  170
_XP_046555158.1_   137  -VVRLCFQVFLPDAS-----------GKIT------RVVP----PVVSQLIHD------------KKS--  170
_AAW33559.1_       137  -MVRLCFQVFLPDAS-----------GKIT------RVVP----PVVSQLIHD------------KKS--  170
_ABY87435.1_       124  -VVRLCFQVFLPDAS-----------GKIT------RVVP----PVVSQLIHD------------KKS--  157
_XP_009028834.1_   137  -SVRLCFQVFLPDAN-----------KKFT------HIVP----PIVSQPIID------------KKS--  170
_XP_025106109.1_   137  -VVRLCFQVFLPDAS-----------GQVT------RVVP----PVSSHPIHD------------KKA--  170
_NP_001298191.1_   138  -VVRLCFQVFLPNEQ-----------GKVT------RVVP----PVCSHPIHD------------KKS--  171
_KAH9519545.1_     138  -VVRLCFQVFLPNEQ-----------GKVT------RVVP----PVCSHPIHD------------KKS--  171
_RUS74561.1_       138  -VVRLCFQVFLPNEQ-----------GKVT------RVVP----PVCSHAIHD------------KKS--  171
_GFO11973.1_       138  -VVRLCFQVFLPNEQ-----------GKVT------RVVP----PVCSHAIHD------------KKS--  171
_CAG5132534.1_     138  -VVRLCFQVFLPNEQ-----------GKIT------RVVP----PVVSQAIHD------------KKS--  171
_GFR69641.1_       138  -VVRLCFQVFLPNDQ-----------GRVT------RVVP----PVCSHAIHD------------KKS--  171
_XP_035824873.1_   138  -VVRLCFQVFLSNEQ-----------GRVS------KVVP----PVCSHAIHD------------KKS--  171
_AAY27981.1_       136  -TVRLCFQVFIADMD-----------GNLK------KPLQ----PVSSQNIID------------KKA--  169
_XP_052827753.1_   135  -VIRLCFQTFIQDAQ-----------GQYR------IPLN----AVVSQPIID------------KKT--  168
_XP_029642523.1_   135  -VIRLCFQTFIQDSQ-----------GQYR------IPLN----AVVSQPIID------------KKT--  168
_CAE1288013.1_      95  -TVRLCFQVFIADKD-----------GNLK------RPLP----PVSSQNIID------------KKA--  128
_ELU04547.1_       136  -AVCLCFQVFLPDDQ-----------SKFT------RVVR----PIVSQCIYD------------KKA--  169
_KAI0227899.1_     134  -TVRLCFQGFLPDQN-----------GKFT------RTVR----PAVSQCIYD------------KKA--  167
_XP_044002103.1_   141  -VVRLCFEVYLPNEK-----------DEFK------FRLE----PIVSEPIFD------------EKT--  174
_XP_044002102.1_   140  -VVRLCFEVYLPNEK-----------DEFK------IRLE----PIVSDPIYD------------KKA--  173
_XP_044002099.1_   140  -VVRLCFEVYLPNEK-----------DEFK------IRLE----PIVSDPIYD------------KKA--  173
_XP_037026629.1_   140  -QMRLCFQVFLIPDP-----------AKNL------IPLE----PVVSDIICD------------KKT--  173
_CAD7632570.1_     141  -AIRLCFQVYVEDPIT----------RKFT------KPLE----PVVSDAVYD------------KKL--  175
_CAD7650738.1_     141  -AIRLCFQAYIEDPVT----------RKFT------RPLA----PVVSDPVYD------------KKL--  175
_XP_054163335.1_   139  -AVRLCFQILIEDPKT----------SQYS------IPLQ----PVVSDVIFD------------KKS--  173
_CAD7629985.1_     146  -AVRLCFQVLLEDPKT----------GQYS------IPLQ----PVVSDVIFD------------KKS--  180
```

```
_CAD7652069.1_     139  -AVRLCFQVLLEDPKT----------GQYS------IPLQ----PVVSDVIFD------------KKS--  173
_XP_017954168.1_   142  -ELRLCYQAFIKVG-------------NNN------VPLD----PVVSSPIYG------------KSN--  173
_XP_017858583.1_   142  -ELRLCYQAFIKVG-------------NNN------VPLD----PVVSSPIYG------------KSN--  173
_XP_002001588.1_   142  -ELRLCYQAFIKVG-------------NNN------VPLD----PVVSSPIYG------------KSN--  173
_XP_017954163.1_   142  -ELRLCYQAFIKVG-------------NNN------VPLD----PVVSSPIYG------------KSN--  173
_XP_023164563.2_   142  -ELRLCYQAFIKVG-------------NNN------VPLD----PVVSSPIYG------------KSN--  173
_TDG46702.1_       142  -ELRLCYQAFIKVG-------------NNN------VPLD----PVVSSPIYG------------KSN--  173
_XP_030564898.1_   142  -ELRLCYQAFIKVG-------------NNN------VPLD----PVVSSPIYG------------KSN--  173
_XP_015024496.1_   142  -ELRLCYQAFIKVG-------------NNN------VPLD----PVVSSPIYG------------KSN--  173
_XP_034475238.1_   143  -QLRLCYQAYIKVD-------------NNY------VSLD----PVVSSPIYG------------KSN--  174
_XP_034475235.1_   143  -QLRLCYQAYIKVD-------------NNY------VSLD----PVVSSPIYG------------KSN--  174
_XP_017854415.1_   142  -ALRLCYQAFLKVG-------------SNK------IPLD----PVVSEPIFG------------KSN--  173
_XP_017854416.1_   142  -ALRLCYQAFLKVG-------------SNK------IPLD----PVVSEPIFG------------KSN--  173
_ALC40089.1_       142  -ALRLCYQAFLKVG-------------SNK------IPLD----PVVSEPIFG------------KSN--  173
_XP_030382983.1_   143  -ELRLCYQVFLPDA------------KNDF-----RIPLN----PVVSSPIIG------------KSK--  176
_XP_030382981.1_   143  -ELRLCYQVFLPDA------------KNDF-----RIPLN----PVVSSPIIG------------KSK--  176
_XP_001989397.1_   142  -ELRLCYQAFIKVG-------------NNH------VPLD----PVVSSPIYG------------KSN--  173
_XP_002067135.2_   143  -ELRLCYQAFITVG-------------NSK------VPLD----PVVSSPIYG------------KSN--  174
_XP_034475239.1_   141  -QLRLCYQAYIKVD-------------NNY------VSLD----PVVSSPIYG------------KSN--  172
_KAH8411901.1_     142  -ELRLCYQVFMKIGG------------NNS------VPLD----PVVSAPIYG------------KSN--  174
_XP_016963143.1_   143  -ELRLCYQAFITVG-------------NSK------VPLD----PIVSSPIYG------------KSN--  174
_XP_037712361.1_   143  -ELRLCYQAFITVG-------------NSK------VPLD----PIVSSPIYG------------KSN--  174
_XP_016993627.2_   143  -ELRLCYQAFITVG-------------NSK------VPLE----PIVSSPIYG------------KSN--  174
_XP_016963141.1_   143  -ELRLCYQAFITVG-------------NSK------VPLD----PIVSSPIYG------------KSN--  174
_XP_016993624.2_   143  -ELRLCYQAFITVG-------------NSK------VPLE----PIVSSPIYG------------KSN--  174
_XP_037712358.1_   143  -ELRLCYQAFITVG-------------NSK------VPLD----PIVSSPIYG------------KSN--  174
_XP_001974449.1_   143  -ELRLCYQAFITVG-------------NSK------VPLK----PIVSSPIYG------------KSN--  174
_AAM48404.1_       143  -ELRLCYQAFITVG-------------NSK------VPLD----PIVSSPIYG------------KSS--  174
_NP_001162999.1_   143  -ELRLCYQAFITVG-------------NSK------VPLD----PIVSSPIYG------------KSS--  174
_XP_026835878.1_   143  -ELRLCYQAFITVG-------------NSK------VPLK----PIVSSPIYG------------KSN--  174
_AAA28465.1_       143  -ELRLCYQAFITVG-------------NSK------VPLD----PIVSSPIYG------------KSS--  174
_XP_002038912.1_   143  -ELRLCYQAFITVG-------------NSK------VPLD----PIVSSPIYG------------KSS--  174
_XP_033169558.1_   143  -ELRLCYQAFITVG-------------NSK------VPLD----PIVSSPIYG------------KSS--  174
_EDX05310.1_       143  -ELRLCYQAFITVG-------------NSK------VPLD----PIVSSPIYG------------NSS--  174
_NP_001162998.1_   143  -ELRLCYQAFITVG-------------NSK------VPLD----PIVSSPIYG------------KSS--  174
_XP_032579766.1_   143  -ELRLCYQAFITVG-------------NSK------VPLD----PIVSSPIYG------------KSS--  174
_XP_033169541.1_   143  -ELRLCYQAFITVG-------------NSK------VPLD----PIVSSPIYG------------KSS--  174
_XP_034669502.1_   143  -ELRLCYQAFITVG-------------NTR------VPLD----PIVSSPIYG------------KSN--  174
_XP_034125957.1_   143  -ELRLCYQAFITVG-------------NTR------VPLD----PIVSSPIYG------------KSN--  174
```

```
_XP_034669501.1_  143  -ELRLCYQAFITVG-------------NTR------VPLD----PIVSSPIYG------------KSN--  174
_XP_034125956.1_  143  -ELRLCYQAFITVG-------------NTR------VPLD----PIVSSPIYG------------KSN--  174
_XP_034125955.1_  143  -ELRLCYQAFITVG-------------NTR------VPLD----PIVSSPIYG------------KSN--  174
_XP_017152033.1_  143  -ELRLCYQAFITVG-------------NTR------VALD----PIVSSPIYG------------KSN--  174
_XP_017152031.1_  143  -ELRLCYQAFITVG-------------NTR------VALD----PIVSSPIYG------------KSN--  174
_XP_002014799.1_  143  -ELRLCYQAFITVG-------------NTR------VALD----PIVSSPIYG------------KSN--  174
_XP_026844902.1_  143  -ELRLCYQAFITVG-------------NTR------VALD----PIVSSPIYG------------KSN--  174
_XP_015035983.2_  143  -ELRLCYQAFITVG-------------NTR------VALD----PIVSSPIYG------------KSN--  174
_XP_017063896.1_  143  -ELRLCYQAFITVG-------------NAK------VPLD----PIVSSPIYG------------KSN--  174
_XP_016935332.1_  143  -ELRLCYQAFITVG-------------NSK------VPLD----PIVSSPIYG------------KSN--  174
_XP_017063894.2_  143  -ELRLCYQAFITVG-------------NAK------VPLD----PIVSSPIYG------------KSN--  174
_XP_036668637.1_  143  -ELRLCYQAFITVG-------------NSK------VPLD----PIVSSPIYG------------KSN--  174
_XP_016935310.1_  143  -ELRLCYQAFITVG-------------NSK------VPLD----PIVSSPIYG------------KSN--  174
_KAH8298318.1_    143  -ELRLCYQAFITVG-------------NTR------VALD----PVVSSPIYG------------KSN--  174
_KAH8412187.1_    143  -ELRLCYQAFITVG-------------NTR------VALD----PIVSSPIYG------------KSN--  174
_KAH8278042.1_    142  -ELRLCYQGFITVN-------------NKK------VPLD----PIVSSPIYG------------KSN--  173
_KAH8366423.1_    143  -ELRLCYQAFITVG-------------NAK------VPME----PIVSSPIYG------------KSN--  174
_XP_017044860.1_  142  -ELRLCYQAFIRIKEG-----------DPV------VALK----PIVSAPIFG------------KSN--  175
_KAH8324526.1_    141  -ELRLCYQAFITVN-------------NKK------VPLD----PIVSSPIYG------------KSN--  172
_XP_017116817.1_  143  -ELRLCYQALIQVG-------------KTW------VALD----PVVSSPIYG------------KSN--  174
_XP_041566118.1_  143  -ELRLCYQALIQVG-------------KTW------VALD----PVVSSPIYG------------KSN--  174
_XP_052852584.1_  143  -ELRLCYQALIQVG-------------KTW------VAMD----PVVSSPIYG------------KSN--  174
_XP_052852582.1_  143  -ELRLCYQALIQVG-------------KTW------VAMD----PVVSSPIYG------------KSN--  174
_XP_017107132.2_  142  -ELRLCYQGFIKK-----------------------VPLD----PIVSSPIYG------------KSN--  169
_XP_043069650.1_  142  -ELRLCYQGFIKK-----------------------VPLD----PIVSSPIYG------------KSN--  169
_KAH8283158.1_    143  -ELRLCYQAFITVG-------------HTR------VALD----PIVSSPIYG------------KSN--  174
_XP_017025640.1_  142  -ELRLCYQAFITVG-------------HTR------VALD----PIVSSPIYG------------KSN--  173
_XP_020803071.1_  143  -ELRLCYQAFITVG-------------HTR------VALD----PIVSSPIYG------------KSN--  174
_XP_017025638.1_  143  -ELRLCYQAFITVG-------------HTR------VALD----PIVSSPIYG------------KSN--  174
_KAH8308067.1_    143  -ELRLCYQAFITVG-------------HTR------VALD----PIVSSPIYG------------KSN--  174
_KAH8362606.1_    143  -ELRLCYQAFITVG-------------HTR------VALD----PIVSSPIYG------------KSN--  174
_XP_020803068.1_  143  -ELRLCYQAFITVG-------------HTR------VALD----PIVSSPIYG------------KSN--  174
_XP_015052364.1_  142  -ELRLCFQAFITVD-------------KKK------VPLK----PIVSSPIHG------------KSY--  173
_XP_002090469.1_  142  -ELRLCFQAFITVD-------------KKK------VPLK----PIVSSPIHG------------KSY--  173
_XP_039480354.1_  142  -ELRLCFQAFITVD-------------KKK------VPLK----PIVSSPIYG------------KSY--  173
_XP_039480352.1_  142  -ELRLCFQAFITVD-------------KKK------VPLK----PIVSSPIYG------------KSY--  173
_XP_041450795.1_  143  -ELRLCYQAFIKVG-------------GTR------VALD----PIVSSPIYG------------KSN--  174
_XP_044315461.1_  143  -ELRLCYQALFKVG-------------ETW------MAFE----PIVSSPIYG------------KSN--  174
_XP_044315463.1_  143  -ELRLCYQALFKVG-------------ETW------MAFE----PIVSSPIYG------------KSN--  174
```

```
_XP_001961710.2_  143  -ELRLCYQGFITVN-------------NKP------VPLD----PVVSTPIFG------------KSN--  174
_XP_014761890.1_  143  -ELRLCYQGFITVN-------------NKP------VPLD----PVVSTPIFG------------KSN--  174
_KAH8323309.1_    143  -ELRLCYQGFITVN-------------NKP------VPLD----PIVSTPIFG------------KSN--  174
_XP_043646206.1_  142  -ELRLCFQAFITVD-------------DKK------VPLK----PIVSSPIYG------------KNL--  173
_XP_043646204.1_  142  -ELRLCFQAFITVD-------------DKK------VPLK----PIVSSPIYG------------KNL--  173
_AAQ65066.1_      143  -ELRLCYQAFITVG-------------NSK------VPLD----PIVSSPIYG------------KSS--  174
_AAQ64900.1_      143  -ELRLCYQAFITVG-------------NSK------VPLD----PIVSSPIYG------------KSS--  174
_AAQ64905.1_      143  -ELRLCYQAFITVG-------------NSK------VPLD----PIVSSPIYG------------KSS--  174
_KAH8378209.1_    142  -QLRLCYHVVIMDDR-----------TKAN------VALE----PVISSPIYG------------KSN--  175
_KAH8293207.1_    142  -HLRLCFHVLVVDQQ-----------TKEY------MALP----PIVSTVIAG------------KSN--  175
_KAH8391978.1_    141  -QLRLCYHVVIMDEK-----------TKSN------IALE----PVVSSPIYG------------KSN--  174
_XP_034101352.1_  141  -QLRLCYHVVIMDEK-----------TKSN------IALE----PVVSSPIYG------------KSN--  174
_KAJ3658269.1_    138  -VVRLCVQVVLLGEN-----------RSQR------INIS----PVVSEHVYN------------KKT--  171
_KAJ3658270.1_    138  -VVRLCVQVVLLGDN-----------GDQR------IKIR----PVVSNPIYD------------GKA--  171
_KAJ3658271.1_    138  -VVRLCVQVVLLGEN-----------GNTR------IGIS----PIVSDPVYN------------KRL--  171
_XP_015117085.1_  140  -VVRLCFQVFLFDHTT----------EKYT------KKLT----PVVSDPIYD------------KQT--  174
_XP_011307643.1_  140  -VVRLCFQVFLYDRDT----------EKYT------KKLT----PIVSDPIYD------------KQT--  174
_XP_011307741.1_  138  -TVRLCFQVYLRPPGAN--------SRQGE------IKLK----PVVSDPIHA------------KQS--  174
_XP_015119445.1_  140  -VVRLCFQVFLFDRTT----------EKCT------RKLT----PVVSDPIYD------------KQT--  174
_XP_052128594.1_  141  -AIRLCLQVFTLTKKKFESKTEKNLADKHW------KQLG----VAVSEPIYN------------KKA--  185
_XP_052128597.1_  133  -AIRLCLQVFTLTKKKFESKTEKNLADKHW------KQLG----VAVSEPIYN------------KKA--  177
_KAE8741315.1_     87  -AIRLCLQVFTLTKKKFESKTEKNLADKHW------KQLG----VAVSEPIYN------------KKA--  131
_XP_028982160.1_  145  -SVRLCFQLVTENPI-----------TKKP------QAVC----SVVSEPIYD------------KKK--  178
_XP_011307645.1_  145  -AVRLCFQVFTEHPE-----------TRKP------QAVC----LAVSEPIFD------------KKR--  178
_XP_054725384.1_  136  -VVRLCFQVFIADEN-----------GHLT------VPLK----PVVTKPIYD------------KRS--  169
_XP_053965385.1_  136  -VVRLCFQVFIADEN-----------GHLT------VPLK----PAVTKPIYD------------KRS--  169
_XP_013149158.1_  147  -ALRLCFQVFIKDEK-----------NRIR------LTLP----PVVTSVIHD------------KKT--  180
_XP_045534655.1_  147  -ALRLCFQVFVMDEK-----------DGIR------LRLP----PVVTSVIHD------------KKT--  180
_KPJ06852.1_      147  -ALRLCFQVFVMDEK-----------DGIR------LRLP----PVVTSVIHD------------KKT--  180
_XP_013176418.1_  147  -ALRLCFQVFVMDKK-----------DNIR------LRLP----PVVTNVIHD------------KKT--  180
_OXU28425.1_      143  -SVRLCFQVTMHSFA------------CGS------IEVD----PVVSDPIYNTCSTVLFRTINHVYN--  187
_XP_008216913.1_  135  -SVRLCFQVILHSFA------------SGS------IEVD----PVVSDPIHNTCKRL--NIKTLRKK--  177
_XP_008216914.1_  131  -SVRLCFQVILHSFA------------SGS------IEVD----PVVSDPIHNTCKRL--NIKTLRKK--  173
_XP_040572301.1_  138  -ILRLCFQAFLKRESS----------Y---------IPLC----PVVSNIIAD------------SRA--  169
_CAB4068408.1_     64  --LKLCFQAFLKRESS----------Y---------IPLC----PVVSNIIAD------------SRA--   94
_KAF7993284.1_    102  -SLRLCFQVFLIDEEE----------T---------KSLT----PVVTNVIYN------------KSRGF  135
_KAF7987489.1_    145  -AVRFCFHVILINDD-----------EGKT------IVLD----PIVTHVMSS-------------IL--  177
_KAF7994520.1_    147  -CVRFCFQATLTSDD-----------GKKI------ITLD----PVVSNVMNS-------------IL--  179
_XP_044019368.1_  150  -SVRICFQAILTNTD-----------EKKS------IVLD----PVVSTVIKN------------YVG--  183
```

```
_XP_044019332.1_    51  -AVRFCFHVILINDD-----------EGKT------IVLD----PIVTHVMSS-------------IL--   83
_XP_044003986.1_    20  -CVRFCFQATLTSDD-----------GKKI------ITLD----PVVSNVMNS-------------IL--   52
_XP_037042732.1_   140  -TVRLCFQVLISSDPK---------NPTPA------AQLE----PKVSQPIIN------------NKA--  175
_CAD7223243.1_     140  -AVRLCFQAFIGKEKR---------------------PLY----PVVSDIIYD------------RKA--  169
_XP_044732233.1_   146  -CLRLCFEVFIGKREN----------P---------FPHF----RLVSNPITD------------TK---  176
_XP_012231885.1_   134  -VVRLCFQVFLEGSQK----------GKFN------KPLQ----PIVSDPIYN-----------SRTV--  169
_XP_022814699.1    145  -ELRLCFEVKLRRCK---------------------KVLE----PIVSNPIYN------------TRR--  174
_CAB3516523.1_     145  -ELRLCFEVKLRRCK---------------------KVLE----PIVSNPIYN------------TRR--  174
_XP_035458245.2_   144  -ELRLCFQVKLRRCK---------------------KVLE----PIVSTPIYN------------TRR--  173
_KAF9795795.1_     133  -ELRLCFQVKLRRCK---------------------KVLE----PIVSTPIYN------------TRR--  162
_XP_014233257.1_   152  -SARLCVTAYLDHE------------GRFD------YCLL----PAVSEPIHD------------SRD--  184
_XP_034324400.1_   122  KNVKECKCVSLFVEAFY-------KVGKQT------FMLS----DTTH-SLLN------------AKE--  159
_XP_052699907.1_   122  KNVKECKCVSLFVEAFY-------KVGKQT------FMLS----DATH-SLLN------------AKE--  159
Consensus_aa:           .slRLCFQhhl.s.p..........sp.........l.....PlVSpsIhs...........pcs..
Consensus_ss:            eeeeeeeeeee                ee      ee       ee

Conservation:                                                    7                    5
_XP_001652840.2_   174  -----------------------MSD-L-------------VICKLSDV---------TAPVAG-GRE-II  196
_AAW67214.1_       174  -----------------------MSD-L-------------VICKLSDV---------TAPVAG-GRE-II  196
_XP_021704027.1_   174  -----------------------MSD-L-------------VICKLSDV---------TAPVAG-GRE-II  196
_XP_019540833.2_   174  -----------------------MSD-L-------------VICKLSDV---------TAPVAG-GRE-II  196
_XP_029720231.1_   174  -----------------------MSD-L-------------VICKLSDV---------TAPVAG-GRE-II  196
_AAW67215.1_       174  -----------------------MSD-L-------------VICKLSDV---------TAPVAG-GRE-II  196
_XP_021704025.1_   174  -----------------------MSD-L-------------VICKLSDV---------TAPVAG-GRE-II  196
_XP_019540831.2_   174  -----------------------MSD-L-------------VICKLSDV---------TAPVAG-GRE-II  196
_KXJ81105.1_       174  -----------------------MSD-L-------------VICKLSDV---------TAPVAG-GRE-II  196
_KXJ76497.1_       174  -----------------------MSD-L-------------VICKLSDV---------TAPVAG-GRE-II  196
_XP_023240428.1_   173  -----------------------MSD-L-------------SIYKLSHY---------SAPVSG-GTE-VI  195
_CAD7241875.1_     174  -----------------------VKD-L-------------FIYRLSHY---------NSSVAG-GRE-MI  196
_TRY80291.1_       171  -----------------------YCD-L-------------QIVDYSDD---------SCTVDG-GKK-IL  193
_ALF84899.1_       168  -----------------------HSD-L-------------AILNYSDN---------WSPVQG-GKK-IL  190
_KAF4528208.1_     174  -----------------------MSD-L-------------VICRLSDV---------SCYASG-GKE-II  196
_XP_054260955.1_   175  -----------------------LCD-L-------------AICRLSHS---------SATVAG-GLE-MI  197
_XP_054264401.1_   175  -----------------------LCD-L-------------AICRLSHS---------SATVAG-GLE-MI  197
_XP_054260954.1_   175  -----------------------LCD-L-------------AICRLSHS---------SATVAG-GLE-MI  197
_XP_038114042.1_   174  -----------------------MSD-L-------------VICKLSDA---------CASVAG-GKE-II  196
_XP_039437558.1_   174  -----------------------MSD-L-------------VICKLSDA---------CASVAG-GKE-II  196
_XP_001844078.2_   174  -----------------------MSD-L-------------VICKLSDA---------CASVAG-GKE-II  196
```

```
_EDS35747.1_       174  ----------------------MSD-L-------------VICKLSDA---------CASVAG-GKE-II  196
_XP_053696925.1_   174  ----------------------MSD-L-------------VICKLSDI---------TASVAG-GKE-II  196
_XP_001869619.2_   174  ----------------------MSE-L-------------VICKLSHV---------TAPTAG-GKE-II  196
_XP_039434954.1_   174  ----------------------MSE-L-------------VICKLSHV---------TAPTAG-GKE-II  196
_EDS29879.1_       174  ----------------------MSE-L-------------VICKLSHV---------TAPTAG-GKE-II  196
_XP_019550742.2_   174  ----------------------MSE-L-------------VICKLSHV---------AATMAG-GKE-VI  196
_KXJ74587.1_       174  ----------------------MSE-L-------------VICKLSHV---------AATMAG-GKE-VI  196
_XP_035908773.1_   175  ----------------------MSD-L-------------IICRLSDC---------CAPVSG-GKE-II  197
_AJC98655.1_       175  ----------------------MSD-L-------------IICRLSDC---------TAPVSG-GKE-II  197
_AJC98650.1_       175  ----------------------MSD-L-------------IICRLSDC---------TAPVSG-GKE-II  197
_AJC98644.1_       175  ----------------------MSD-L-------------IICRLSDC---------TAPVSG-GKE-II  197
_AJC98657.1_       175  ----------------------MSD-L-------------IICRLSDC---------TAPVSG-GKE-II  197
_AJC98648.1_       175  ----------------------MSD-L-------------IICRLSDC---------TAPVSG-GKE-II  197
_AJC98647.1_       175  ----------------------MSD-L-------------IICRLSDC---------TAPVSG-GKE-II  197
_AJC98651.1_       175  ----------------------MSD-L-------------IICRLSDC---------TAPVSG-GKE-II  197
_AJC98643.1_       175  ----------------------MSD-L-------------IICRLSDC---------TAPVSG-GKE-II  197
_AJC98656.1_       175  ----------------------MSD-L-------------IICRLSDC---------TAPVSG-GKE-II  197
_AJC98641.1_       175  ----------------------MSD-L-------------IICRLSDC---------TAPVSG-GKE-II  197
_AJC98640.1_       175  ----------------------MSD-L-------------IICRLSDC---------TAPVSG-GKE-II  197
_AJC98653.1_       175  ----------------------MSD-L-------------IICRLSDC---------TAPVSG-GKE-II  197
_AJC98654.1_       175  ----------------------MSD-L-------------IICRLSDC---------TAPVSG-GKE-II  197
_AJC98652.1_       175  ----------------------MSD-L-------------IICRLSDC---------TAPVSG-GKE-II  197
_AJC98646.1_       175  ----------------------MSD-L-------------IICRLSDC---------TAPVSG-GKE-II  197
_AJC98658.1_       175  ----------------------MSD-L-------------IICRLSDC---------TAPVSG-GKE-II  197
_AJC98639.1_       175  ----------------------MSD-L-------------IICRLSDC---------TAPVSG-GKE-II  197
_XP_035908772.1_   175  ----------------------MSD-L-------------IICRLSDC---------CAPVSG-GKE-II  197
_XP_050076803.1_   175  ----------------------MSD-L-------------IICRLSDC---------CAPVSG-GKE-II  197
_XP_049282441.1_   175  ----------------------MSD-L-------------IICRLSDC---------CAPTSG-GKE-II  197
_XP_040170080.1_   175  ----------------------MSD-L-------------IICRLSDC---------TAPVSG-GKE-II  197
_XP_050076802.1_   175  ----------------------MSD-L-------------IICRLSDC---------CAPVSG-GKE-II  197
_XP_041780138.1_   175  ----------------------MSD-L-------------IICRLSDC---------TAPVSG-GKE-II  197
_XP_040239229.2_   175  ----------------------MSD-L-------------IICRLSDC---------TAPVSG-GKE-II  197
_XP_040239222.2_   175  ----------------------MSD-L-------------IICRLSDC---------TAPVSG-GKE-II  197
_XP_035908780.1_   175  ----------------------MSD-L-------------IICRLSDC---------CAPVSG-GKE-II  197
_XP_040239221.2_   175  ----------------------MSD-L-------------IICRLSDC---------TAPVSG-GKE-II  197
_XP_052897786.1_   175  ----------------------MSD-L-------------IICRLSDC---------CAPVSG-GKE-II  197
_XP_052895493.1_   175  ----------------------MSD-L-------------IICRLSDC---------CAPVSG-GKE-II  197
_XP_053664395.1_   175  ----------------------MSD-L-------------IICRLSDC---------CAPASG-GKE-II  197
_XP_050076801.1_   175  ----------------------MSD-L-------------IICRLSDC---------CAPVSG-GKE-II  197
```

```
_XP_310177.3_     175  ----------------------MSD-L-------------IICRLSDC---------TAPVSG-GKE-II  197
_XP_001652339.1_  174  ----------------------MSE-L-------------VICKLSHV---------AAPMSG-GKE-VI  196
_XP_053686256.1_  174  ----------------------MSD-L-------------VICKLSEV---------TAPVSG-GKE-II  196
_XP_053675540.1_  175  ----------------------MSD-L-------------IIVRLSDC---------TAPVSG-GKD-II  197
_ETN66814.1_      174  ----------------------MSD-L-------------TICRLSDC---------SAPVSG-GKN-II  196
_XP_049534501.1_  174  ----------------------MSD-L-------------TICRLSDC---------SAPVSG-GKN-II  196
_XP_050084431.1_  174  ----------------------MSD-L-------------TICRLSDC---------SAPVSG-GKN-II  196
_XP_035782874.1_  174  ----------------------MSD-L-------------TICRLSDC---------SAPVSG-GKN-II  196
_XP_035782869.1_  174  ----------------------MSD-L-------------TICRLSDC---------SAPVSG-GKN-II  196
_KFB39849.1_      182  ----------------------MSD-L-------------IICRLSDC---------TAPVSG-GKE-II  204
_XP_052871375.1_  175  ----------------------MSD-L-------------IICRLSDS---------TAPVCG-GKE-II  197
_AAR13743.1_      160  ----------------------MSD-L-------------IICRLSDC---------TAPVSG-GKE-II  182
_AAR13744.1_      160  ----------------------MSD-L-------------IICRLSDC---------TAPVSG-GKE-II  182
_CAA65157.1_      154  ----------------------MSD-L-------------IICRLSDC---------TAPVSG-GKE-II  176
_XP_022170156.1_  173  ----------------------MSD-L-------------VICRLSDA---------AASVVG-GRE-II  195
_XP_025205168.1_  173  ----------------------MSD-L-------------VICRLSDA---------AASVVG-GRE-II  195
_XP_026817162.1_  173  ----------------------MSD-L-------------VICRLSDA---------AASVVG-GRE-II  195
_XP_027846205.1_  173  ----------------------MSD-L-------------VICRLSDA---------AASVVG-GRE-II  195
_XP_015370635.1_  173  ----------------------MSD-L-------------VICRLSDA---------AASVVG-GRE-II  195
_CAH1723797.1_    173  ----------------------MSD-L-------------VICRLSDA---------AASVVG-GRE-II  195
_KAF0769940.1_    173  ----------------------MSD-L-------------VICRLSDA---------AASVVG-GRE-II  195
_CAB4065202.1_    122  ----------------------HTD-L-------------HIYDQSDD---------WSSVEG-GKK-II  144
_XP_050440903.1_  173  ----------------------MSD-L-------------VICRLSDA---------AASVAG-GRE-II  195
_XP_050533799.1_  173  ----------------------MSD-L-------------VICRLSDA---------AASVAG-GRE-II  195
_XP_025410632.1_  173  ----------------------MSD-L-------------VICRLSSA---------SATVSG-DNE-VI  195
_XP_008180746.1_  173  ----------------------MSD-L-------------SICRLSDA---------AASVVG-DKE-II  195
_XP_026465450.1_  175  ----------------------TSD-L-------------VICELSQC---------TASAAG-GQK-II  197
_VVC36026.1_      173  ----------------------MSD-L-------------VICRLSDA---------SASVAG-GKD-II  195
_XP_016661522.1_  173  ----------------------MSD-L-------------SICRLSDA---------AASIVG-DKE-II  195
_XP_001947429.3_  173  ----------------------MSD-L-------------SICRLSDA---------AASIVG-DKE-II  195
_XP_026466007.1_  175  ----------------------TSD-L-------------VICELSQC---------TASAAG-GQK-II  197
_KRT81254.1_      170  ----------------------VCD-L-------------TIVELSDC---------VCPVDG-GNKNII  193
_XP_033224832.1_  179  ----------------------TSG-L-------------NIFKMSHY---------SASVAG-GLE-MI  201
_XP_025407590.1_  166  ----------------------MTG-L-------------TIYRLSHA---------SAPITG-NTE-II  188
_XP_045476729.1_  169  ----------------------LPN-L-------------VITRISTC---------ICAVDE-EPE-II  191
_XP_034174253.1_  174  ----------------------MSD-L-------------VICKLSHC---------SASVAG-GME-MI  196
_XP_029047866.1_  174  ----------------------MSD-L-------------VICKLSHC---------SASVAG-GME-MI  196
_XP_015430648.1_  174  ----------------------MSD-L-------------VICKLSHC---------SASVAG-GME-MI  196
_XP_017798081.1_  174  ----------------------MSD-L-------------VICKLSHC---------SASVAG-GME-MI  196
```

```
_XP_017756471.1_  174  ----------------------MSD-L-------------VICKLSHC---------SASVAG-GME-MI  196
_XP_043248447.1_  174  ----------------------MSD-L-------------VICKLSHC---------SASVAG-GME-MI  196
_XP_029047855.1_  174  ----------------------MSD-L-------------VICKLSHC---------SASVAG-GME-MI  196
_XP_034174252.1_  174  ----------------------MSD-L-------------VICKLSHC---------SASVAG-GME-MI  196
_XP_031829337.1_  174  ----------------------MSD-L-------------VICKLSHC---------SASVAG-GME-MI  196
_KZC09125.1_      174  ----------------------MSD-L-------------VICKLSHC---------SASVAG-GME-MI  196
_XP_033327213.1_  174  ----------------------MSD-L-------------VISKLSHC---------SASVAG-GME-MI  196
_XP_033327212.1_  174  ----------------------MSD-L-------------VISKLSHC---------SASVAG-GME-MI  196
_XP_017756445.1_  174  ----------------------MSD-L-------------VICKLSHC---------SASVAG-GME-MI  196
_OAD62393.1_      174  ----------------------MSD-L-------------VICKLSHC---------SASVAG-GME-MI  196
_XP_043248413.1_  174  ----------------------MSD-L-------------VICKLSHC---------SASVAG-GME-MI  196
_XP_033327208.1_  174  ----------------------MSD-L-------------VISKLSHC---------SASVAG-GME-MI  196
_XP_034950477.1_  174  ----------------------MSD-L-------------VICKLSHY---------SASVAG-GME-MI  196
_XP_034950475.1_  174  ----------------------MSD-L-------------VICKLSHY---------SASVAG-GME-MI  196
_XP_012343274.1_  174  ----------------------MSD-L-------------VICKLSHS---------NASVAG-GME-MI  196
_XP_016909449.1_  174  ----------------------MSD-L-------------VICKLSHS---------NASVAG-GME-MI  196
_XP_012343273.1_  174  ----------------------MSD-L-------------VICKLSHS---------NASVAG-GME-MI  196
_XP_006619742.1_  174  ----------------------MSD-L-------------VICKLSHS---------NASVAG-GME-MI  196
_XP_006567065.1_  174  ----------------------MSD-L-------------VICKLSHS---------NASVAG-GME-MI  196
_XP_006619741.1_  174  ----------------------MSD-L-------------VICKLSHS---------NASVAG-GME-MI  196
_NP_001011577.1_  174  ----------------------MSD-L-------------VICKLSHS---------NASVAG-GME-MI  196
_XP_016909448.1_  174  ----------------------MSD-L-------------VICKLSHS---------NASVAG-GME-MI  196
_XP_043802365.1_  174  ----------------------MSD-L-------------VICKLSHS---------NASVAG-GME-MI  196
_NP_001164477.1_  174  ----------------------MSD-L-------------VICKLSHS---------NASVAG-GME-MI  196
_XP_016909443.1_  174  ----------------------MSD-L-------------VICKLSHS---------NASVAG-GME-MI  196
_XP_043802361.1_  174  ----------------------MSD-L-------------VICKLSHS---------NASVAG-GME-MI  196
_XP_012343269.1_  174  ----------------------MSD-L-------------VICKLSHS---------NASVAG-GME-MI  196
_XP_006619738.1_  174  ----------------------MSD-L-------------VICKLSHS---------NASVAG-GME-MI  196
_PBC29878.1_      174  ----------------------MSD-L-------------VICKLSHS---------NASVAG-GME-MI  196
_XP_046663984.1_  173  ----------------------LCD-L-------------AICKLSHC---------SAPVAG-GME-MI  195
_XP_046663985.1_  173  ----------------------LCD-L-------------AICKLSHC---------SAPVAG-GME-MI  195
_XP_043274771.1_  174  ----------------------MSD-L-------------VICKLSHC---------SASVAG-GLE-MI  196
_XP_043274769.1_  174  ----------------------MSD-L-------------VICKLSHC---------SASVAG-GLE-MI  196
_XP_043289571.1_  173  ----------------------MSD-L-------------MICKLSHY---------SGTVAG-GME-MI  195
_XP_032665873.1_  174  ----------------------MSD-L-------------VICKLSHC---------SASVAG-GME-MI  196
_XP_032665875.1_  174  ----------------------MSD-L-------------VICKLSHC---------SASVAG-GME-MI  196
_XP_032665874.1_  174  ----------------------MSD-L-------------VICKLSHC---------SASVAG-GME-MI  196
_XP_032665869.1_  174  ----------------------MSD-L-------------VICKLSHC---------SASVAG-GME-MI  196
_XP_014470289.1_  174  ----------------------MSD-L-------------VICKLSHC---------SAPVAG-GME-MI  196
```

```
_XP_011149046.1_  174  ----------------------MSD-L-------------VICKLSHC---------SASVAG-GME-MI  196
_XP_017877914.1_  172  ----------------------MSD-L-------------VICKLSHC---------SASVAG-GME-MI  194
_XP_017877911.1_  172  ----------------------MSD-L-------------VICKLSHC---------SASVAG-GME-MI  194
_XP_033174774.1_  174  ----------------------MSD-L------------VVICKLSHS---------NASVAG-GME-MI  197
_XP_043581013.1_  174  ----------------------MSD-L-------------VICKLSHS---------NASVAG-GME-MI  196
_XP_048261507.1_  174  ----------------------MSD-L-------------VICKLSHS---------NASVAG-GME-MI  196
_XP_050573946.1_  174  ----------------------MSD-L-------------VICKLSHS---------NASVAG-GME-MI  196
_XP_050478075.1_  174  ----------------------MSD-L-------------VICKLSHS---------NASVAG-GME-MI  196
_XP_033204249.1_  174  ----------------------MSD-L-------------VICKLSHS---------NASVAG-GME-MI  196
_XP_033358451.1_  174  ----------------------MSD-L-------------VICKLSHS---------NASVAG-GME-MI  196
_XP_033358450.1_  174  ----------------------MSD-L-------------VICKLSHS---------NASVAG-GME-MI  196
_XP_033204244.1_  174  ----------------------MSD-L-------------VICKLSHS---------NASVAG-GME-MI  196
_XP_033312956.1_  174  ----------------------MSD-L-------------VICKLSHS---------NASVAG-GME-MI  196
_XP_012236639.1_  174  ----------------------MSD-L-------------VICKLSHS---------NASVAG-GME-MI  196
_XP_050573940.1_  174  ----------------------MSD-L-------------VICKLSHS---------NASVAG-GME-MI  196
_XP_012174118.1_  174  ----------------------MSD-L-------------VICKLSHS---------NASVAG-GME-MI  196
_XP_043581010.1_  174  ----------------------MSD-L-------------VICKLSHS---------NASVAG-GME-MI  196
_EZA51108.1_      174  ----------------------MSD-L-------------VICKLSHC---------SASVAG-GMD-MI  196
_XP_011344085.1_  174  ----------------------MSD-L-------------VICKLSHC---------SASVAG-GMD-MI  196
_XP_043519475.1_  174  ----------------------MSD-L-------------VICKLSHC---------SASVAG-GME-MI  196
_CAD1481114.1_    174  ----------------------MSD-L-------------VICKLSHC---------SASVAG-GME-MI  196
_XP_043519473.1_  174  ----------------------MSD-L-------------VICKLSHC---------SASVAG-GME-MI  196
_KOX75360.1_      174  ----------------------MSD-L-------------VICKLSHC---------SASVAG-GME-MI  196
_KAF3423741.1_    174  ----------------------MSD-L-------------VICKLSHC---------SASVAG-GME-MI  196
_KOC69050.1_      174  ----------------------MSD-L-------------VICKLSHC---------SASVAG-GME-MI  196
_XP_053980095.1_  174  ----------------------MSD-L-------------VICKLSHC---------SASVAG-GME-MI  196
_XP_054002893.1_  174  ----------------------MSD-L-------------VICKLSHC---------SASVAG-GME-MI  196
_XP_053980091.1_  174  ----------------------MSD-L-------------VICKLSHC---------SASVAG-GME-MI  196
_XP_028049144.1_  174  ----------------------MSD-L-------------VICKLSHC---------SASVAG-SMD-MI  196
_XP_012541458.1_  174  ----------------------MSD-L-------------VICKLSHC---------SASVAG-SMD-MI  196
_XP_003708412.1_  174  ----------------------MSD-L-------------VICKLSHS---------SASVAG-GME-MI  196
_XP_012152807.1_  174  ----------------------MSD-L-------------VICKLSHS---------SASVAG-GME-MI  196
_KAH0945556.1_    174  ----------------------MSD-L-------------VICKLSHC---------SASVAG-GMD-MI  196
_XP_024871832.1_  173  ----------------------MCD-L-------------VICKLSHC---------SASVAG-GMD-MI  195
_XP_011307641.1_  173  ----------------------KSD-L--------------ISKLSHC---------SASVAG-GTE-VI  194
_XP_015118260.1_  173  ----------------------KSD-LI-------------ISKLSHC---------SASVAG-GTE-VI  195
_XP_011307640.1_  173  ----------------------KSD-LI-------------ISKLSHC---------SASVAG-GTE-VI  195
_XP_015118252.1_  173  ----------------------KSD-LI-------------ISKLSHC---------SASVAG-GTE-VI  195
_EFN68841.1_      178  ----------------------MSD-L-------------VICKLSHC---------SASVAG-SMD-MI  200
```

```
_XP_011647340.1_  174  ----------------------MSD-L-------------VICKLSHC---------SASVAG-SID-MI  196
_XP_029166122.1_  178  ----------------------MSD-L-------------VICKLSHC---------SASVAG-GMD-MI  200
_KMQ96453.1_      178  ----------------------MSD-L-------------VICKLSHC---------SSSVAG-GMD-MI  200
_XP_050464492.1_  178  ----------------------MSD-L-------------VICKLSHC---------SASVAG-GMD-MI  200
_XP_029673315.1_  178  ----------------------MSD-L-------------VICKLSHC---------SASVAG-GMD-MI  200
_XP_025265518.1_  178  ----------------------MSD-L-------------VICKLSHC---------SASVAG-SMD-MI  200
_XP_011255523.1_  178  ----------------------MSD-L-------------VICKLSHC---------SASVAG-SMD-MI  200
_XP_011698133.1_  174  ----------------------MAD-L-------------VICKLSHC---------SATVAG-GMN-MI  196
_XP_011698131.1_  174  ----------------------MAD-L-------------VICKLSHC---------SATVAG-GMN-MI  196
_XP_018357420.1_  174  ----------------------MSD-L-------------VICKLSHC---------SAPVAG-GIN-MI  196
_KYM76140.1_      174  ----------------------MSD-L-------------VICKLSHC---------SAPVAG-GIN-MI  196
_XP_011647339.1_  174  ----------------------MSD-L-------------VICKLSHC---------SASVAG-SID-MI  196
_KAG5315434.1_    174  ----------------------MSD-L-------------VICKLSHC---------SAPVAG-GIN-MI  196
_XP_011055463.1_  174  ----------------------MSD-L-------------VICKLSHC---------SAPVAG-GIN-MI  196
_XP_018342550.1_  174  ----------------------MSD-L-------------VICKLSHC---------SAPVAG-GIN-MI  196
_XP_018403565.1_  174  ----------------------MSD-L-------------VICKLSHC---------SAPVAG-GIN-MI  196
_KAG5322912.1_    174  ----------------------MSD-L-------------VICKLSHC---------SAPVAG-GIN-MI  196
_XP_018357417.1_  174  ----------------------MSD-L-------------VICKLSHC---------SAPVAG-GIN-MI  196
_XP_018314560.1_  174  ----------------------MSD-L-------------VICKLSHC---------SAPVAG-GIN-MI  196
_XP_018057217.1_  174  ----------------------MSD-L-------------VICKLSHC---------SAPVAG-GIN-MI  196
_KAG5346558.1_    174  ----------------------MSD-L-------------VICKLSHC---------SAPVAG-GIN-MI  196
_XP_047348408.1_  174  ----------------------MSD-L------------VIINKLSHC---------NAPVAG-GMG-MI  197
_XP_047348412.1_  174  ----------------------MSD-L------------V-INKLSHC---------NAPVAG-GMG-MI  196
_XP_046820611.1_  174  ----------------------MSD-L------------V-INKLSHC---------NAPVAG-GMG-MI  196
_XP_050848254.1_  174  ----------------------MSD-L------------V-INKLSHC---------NAPVAG-GMG-MI  196
_XP_046820612.1_  174  ----------------------MSD-L------------V-INKLSHC---------NAPVAG-GMG-MI  196
_XP_050848252.1_  174  ----------------------MSD-L------------V-INKLSHC---------NAPVAG-GMG-MI  196
_XP_043667564.1_  174  ----------------------MSD-L------------V-INKLSHC---------NAPVAG-GMG-MI  196
_XP_035725337.1_  174  ----------------------MSD-L------------V-INKLSHC---------NAPVAG-GMG-MI  196
_XP_043667562.1_  174  ----------------------MSD-L------------V-INKLSHC---------NAPVAG-GMG-MI  196
_XP_046820605.1_  174  ----------------------MSD-L------------V-INKLSHC---------NAPVAG-GMG-MI  196
_XP_050848249.1_  174  ----------------------MSD-L------------V-INKLSHC---------NAPVAG-GMG-MI  196
_XP_043667560.1_  174  ----------------------MSD-L------------V-INKLSHC---------NAPVAG-GMG-MI  196
_XP_035725333.1_  174  ----------------------MSD-L------------V-INKLSHC---------NAPVAG-GMG-MI  196
_KAF7405659.1_    174  ----------------------MSD-L------------V-INKLSHC---------NAPVAG-GMG-MI  196
_KAF7402294.1_    174  ----------------------MSD-L------------V-INKLSHC---------NAPVAG-GMG-MI  196
_XP_015172825.1_  174  ----------------------MSD-L-------------SINHLSHN---------RAPSCG-GMK-MI  196
_XP_015172824.1_  174  ----------------------MSD-L-------------SINHLSHN---------RAPSCG-GMK-MI  196
_XP_015172823.1_  174  ----------------------MSD-L-------------SINHLSHN---------RAPSCG-GMK-MI  196
```

```
_XP_014609306.1_  174  ----------------------MSD-L-------------SINHLSHN---------RAPSCG-GMK-MI  196
_XP_043491219.1_  174  ----------------------MSD-L-------------SINHLSHN---------RAPSCG-GMK-MI  196
_XP_014609305.1_  174  ----------------------MSD-L-------------SINHLSHN---------RAPSCG-GMK-MI  196
_XP_043491218.1_  174  ----------------------MSD-L-------------SINHLSHN---------RAPSCG-GMK-MI  196
_KAI4498556.1_    174  ----------------------MSD-L-------------SINHLSHN---------RAPSCG-GMK-MI  196
_XP_015172818.1_  174  ----------------------MSD-L-------------SINHLSHN---------RAPSCG-GMK-MI  196
_XP_043491211.1_  174  ----------------------MSD-L-------------SINHLSHN---------RAPSCG-GMK-MI  196
_KAI4495142.1_    174  ----------------------MSD-L-------------SINHLSHN---------RAPSCG-GMK-MI  196
_XP_014609300.1_  174  ----------------------MSD-L-------------SINHLSHN---------RAPSCG-GMK-MI  196
_ADK91833.1_      174  ----------------------MSD-L-------------VICKLSHS---------NASVAG-GME-MI  196
_KAG7213837.1_    174  ----------------------MSD-L-------------VICKLSHC---------SASVAG-GME-MI  196
_XP_014226628.1_  173  ----------------------NSD-L-------------MICKMSHC---------SASVHG-GTE-MI  195
_XP_020287946.1_  174  ----------------------MSD-L-------------VICKLSHC---------SASVAG-GMD-MI  196
_XP_012232007.1_  174  ----------------------MSD-L-------------VICKLSHC---------SASVAG-GLD-MI  196
_XP_011874538.1_  174  ----------------------MSD-L-------------VICKLSHC---------SASVAG-SMD-MI  196
_XP_011874541.1_  174  ----------------------MSD-L-------------VICKLSHC---------SASVAG-SMD-MI  196
_XP_011173619.1_  174  ----------------------MSD-L-------------VICKLSHC---------SAPVAG-SMD-MI  196
_XP_034950793.1_  171  ----------------------LNE-L-------------VICKLSHS---------SAPTDG-GME-MI  193
_XP_034950792.1_  167  ----------------------LNE-L-------------VICKLSHS---------SAPTDG-GME-MI  189
_XP_012340880.2_  171  ----------------------IPD-L-------------MICELSHY---------SASVAG-GMN-MI  193
_XP_031771859.1_  171  ----------------------IPD-L-------------MICELSHY---------SASVAG-GMN-MI  193
_XP_006618953.1_  173  ----------------------IPD-L-------------MICELSHY---------SASVAG-GMH-MI  195
_XP_031367904.1_  173  ----------------------IPD-L-------------MICELSHY---------SASVAG-GMH-MI  195
_XP_043795948.1_  173  ----------------------IPD-L-------------MICELSHY---------SASVAG-GMH-MI  195
_XP_395180.5_     173  ----------------------IPD-L-------------MICELSHY---------SASVAG-GMH-MI  195
_KAG6799500.1_    173  ----------------------IPD-L-------------MICELSHY---------SASVAG-GMH-MI  195
_XP_016768607.2_  173  ----------------------IPD-L-------------MICELSHY---------SASVAG-GMH-MI  195
_PBC31989.1_      182  ----------------------IPD-L-------------MICELSHY---------SASVAG-GMH-MI  204
_XP_016914450.1_  173  ----------------------IPD-L-------------MICELSHY---------SASVAG-GMH-MI  195
_KAG5344958.1_    171  ----------------------FQN-L-------------VICKLSHC---------SAPVAG-GIN-MI  193
_KAJ3630188.1_    172  ----------------------IPE-P-------------QIHRLSHC---------NCYADG-GKPDII  195
_QHN75199.1_      172  ----------------------IPE-P-------------QIHRLSHC---------NCYADG-GKPDII  195
_XP_051171660.1_  179  ----------------------TCT-L-------------SIHKLSHY---------CASVAG-GME-MI  201
_XP_016909447.1_  132  ----------------------MSD-L-------------VICKLSHS---------NASVAG-GME-MI  154
_XP_031773030.1_  132  ----------------------MSD-L-------------VICKLSHS---------NASVAG-GME-MI  154
_KAH0956427.1_    179  ----------------------YLN-L-------------VIRELCQF---------SSSVAG-GEN-MI  201
_XP_012283784.1_  174  ----------------------MSD-L-------------VICKLSTC---------SASVAG-GTE-MI  196
_XP_023224928.1_  173  ----------------------HSD-L-------------SIHKLSHY---------SAPVSG-GTE-VI  195
_XP_008556434.1_  174  ----------------------MSD-L-------------VICKLSHY---------SAPVAG-GME-MI  196
```

```
_XP_008556433.1_   174  ----------------------MSD-L-------------VICKLSHY---------SAPVAG-GME-MI  196
_XP_014299728.1_   174  ----------------------MSD-L-------------VICKLSHY---------SAPVAG-GME-MI  196
_CAD6234449.1_     174  ----------------------MSD-L-------------VICKLSHY---------SAPVAG-GME-ML  196
_KAG8037552.1_     174  ----------------------MSD-L-------------VICKLSHY---------SAPVAG-GME-ML  196
_XP_044576138.1_   174  ----------------------MSD-L-------------VICKLSHY---------SAPVAG-GME-ML  196
_XP_044576125.1_   174  ----------------------MSD-L-------------VICKLSHY---------SAPVAG-GME-ML  196
_XP_026465448.1_   172  ----------------------MSD-L-------------VICTMSDC---------TASVAG-GQR-II  194
_XP_026465446.1_   172  ----------------------MSD-L-------------VICTMSDC---------TASVAG-GQR-II  194
_XP_031785072.1_   174  ----------------------MSD-L-------------VICKLSHC---------SASVAG-GTE-MV  196
_XP_015607185.1_   174  ----------------------MSD-L-------------VICKLSTC---------SASVAG-GTE-MM  196
_XP_011496611.1_   174  ----------------------MSD-L-------------VICKLSHC---------SASVAG-GTE-MV  196
_XP_018903707.1_   170  ----------------------MSN-L-------------QICRICPP---------SASVLG-GTE-VI  192
_CAB3365562.1_     173  ----------------------NTD-L-------------QITRLSDV---------SCSVAG-GKE-II  195
_XP_011496447.1_    44  ----------------------VSD-L-------------KICWLSHH---------SASVTG-GTQ-MC   66
_KAG8230996.1_     173  ----------------------MSD-L-------------VICKLSDC---------TCSVAG-GKE-II  195
_XP_040567422.1_   170  ----------------------HTD-L-------------HIYDQSDD---------WSSVEG-GKK-II  192
_XP_014250205.1_   174  ----------------------MAD-L-------------IICKLSHC---------SASVAG-GKE-II  196
_XP_014250206.1_   174  ----------------------MAD-L-------------IICKLSHC---------SASVAG-GKE-II  196
_XP_047119528.1_   174  ----------------------MSD-L-------------VICKLSDC---------TATVAG-GKE-II  196
_XP_047001845.1_   174  ----------------------MSD-L-------------VICKLSDC---------TATVAG-GKE-II  196
_XP_049939433.1_   174  ----------------------MSD-L-------------VICKLSDC---------TATVAG-GKE-II  196
_XP_049764197.1_   174  ----------------------MSD-L-------------VICKLSDC---------TATVAG-GKE-II  196
_XP_049833656.1_   174  ----------------------MSD-L-------------VICKLSDC---------TATVAG-GKE-II  196
_XP_049790026.1_   174  ----------------------MSD-L-------------VICKLSDC---------TATVAG-GKE-II  196
_XP_049833651.1_   174  ----------------------MSD-L-------------VICKLSDC---------TATVAG-GKE-II  196
_XP_047001842.1_   174  ----------------------MSD-L-------------VICKLSDC---------TATVAG-GKE-II  196
_XP_049790025.1_   174  ----------------------MSD-L-------------VICKLSDC---------TATVAG-GKE-II  196
_XP_049833654.1_   174  ----------------------MSD-L-------------VICKLSDC---------TATVAG-GKE-II  196
_XP_049833659.1_   174  ----------------------MSD-L-------------VICKLSDC---------TATVAG-GKE-II  196
_XP_021925503.1_   174  ----------------------MSD-L-------------VICKLSDC---------SCTVAG-GKE-II  196
_KDR23857.1_       174  ----------------------MSD-L-------------VICKLSDC---------SCTVAG-GKE-II  196
_ABU96700.1_       174  ----------------------MAD-L-------------VICKLSHC---------SGSVAG-GNE-II  196
_ABU96699.1_       174  ----------------------MAD-L-------------VICKLSHC---------SGSVAG-GNE-II  196
_XP_014275495.1_   174  ----------------------MAD-L-------------VICKLSHS---------AASVAG-GQD-VI  196
_BBE08137.1_       174  ----------------------MAD-L-------------VICKLSHC---------SASVAG-GQD-VI  196
_XP_014275494.1_   174  ----------------------MAD-L-------------VICKLSHS---------AASVAG-GQD-VI  196
_XP_014275493.1_   174  ----------------------MAD-L-------------VICKLSHS---------AASVAG-GQD-VI  196
_BBE08136.1_       174  ----------------------MAD-L-------------VICKLSHC---------SASVAG-GQD-VI  196
_KAF6207519.1_     174  ----------------------MAD-L-------------VICKLSHC---------SGSVIG-GNE-II  196
```

```
_CAB0015508.1_     174  ----------------------MAD-L-------------VICKLSHC---------SGSVVG-GNE-II  196
_CAB0015504.1_     174  ----------------------MAD-L-------------VICKLSHC---------SGSVVG-GNE-II  196
_PNF18474.1_       174  ----------------------MSD-L-------------VICKLSDC---------TCTVAG-GKE-II  196
_CEG62431.1_       174  ----------------------MSD-L-------------VICKLSDC---------SCTVAG-GKE-II  196
_PSN36690.1_       174  ----------------------MSD-L-------------VICKLSDC---------SCTVAG-GKE-II  196
_XP_046387412.1_   174  ----------------------MSD-L-------------VICKLSDC---------TCSVAG-GKE-II  196
_XP_046387414.1_   174  ----------------------MSD-L-------------VICKLSDC---------TCSVAG-GKE-II  196
_XP_031634874.1_   174  ----------------------MSD-L-------------VICKLSKP---------SCTVDG-GEE-II  196
_GFG29240.1_       174  ----------------------MSD-L-------------VICKLSDC---------SCTVAG-GKE-II  196
_BBG28464.1_       174  ----------------------MCD-L-------------VICKLSDC---------SATVGG-GRE-II  196
_WCL52334.1_       174  ----------------------MCD-L-------------VICKLSDC---------SATVGG-GRE-II  196
_GLH00135.1_       174  ----------------------MCD-L-------------VICKLSDC---------SATVGG-GRE-II  196
_CAD7205239.1_     174  ----------------------MSD-L------------VIICRLSDC---------SCSVAG-GKE-II  197
_CAD7431383.1_     174  ----------------------MSD-L------------V-ICRLSDC---------SCTVAG-GKE-II  196
_CAD7410836.1_     174  ----------------------MSD-L------------V-ICRLSDC---------SCSVAG-GKE-II  196
_CAD7415345.1_     174  ----------------------MSD-L------------V-ICRLSDC---------SCTVAG-GKE-II  196
_CAD7606635.1_     174  ----------------------MSD-L------------V-ICRLSDC---------SCTVAG-GKE-II  196
_CAD7462957.1_     174  ----------------------MSD-L------------V-ICRLSDC---------SCTVAG-GKE-II  196
_XP_012265400.2_   174  ----------------------MSD-L--------------ICKLSDC---------CCTVAG-GKE-MI  195
_XP_046473587.1_   174  ----------------------MSD-L-------------VICKLSDC---------CCTVAG-GKE-II  196
_XP_046611732.1_   174  ----------------------MSD-L-------------VICKLSDC---------CCTVAG-GKE-II  196
_XP_015519750.2_   174  ----------------------MSD-L-------------VICKLSDC---------CCTVAG-GKE-II  196
_XP_046611731.1_   174  ----------------------MSD-L-------------VICKLSDC---------CCTVAG-GKE-II  196
_XP_046417903.1_   174  ----------------------MSD-L-------------VICKLSDC---------CCTVAG-GKE-II  196
_GLG94364.1_       173  ----------------------MSD-L-------------VICKMSDC---------SASVAG-GKE-II  195
_GLG94363.1_       173  ----------------------MSD-L-------------VICKMSDC---------SASVAG-GKE-II  195
_BBG28463.1_       173  ----------------------MSD-L-------------VICKMSDC---------SASVAG-GKE-II  195
_WCL52335.1_       173  ----------------------MSD-L-------------VICKMSDC---------SASVAG-GKE-II  195
_GLG94365.1_       173  ----------------------MSD-L-------------VICKMSDC---------SASVAG-GKE-II  195
_XP_002431518.1_   172  ----------------------MSD-L-------------TICKLSDC---------SASVAG-GKE-II  194
_KAE8746525.1_     174  ----------------------MSD-L-------------VICKLSEA---------SASVGG-GKE-MI  196
_XP_026277770.1_   174  ----------------------MSD-L-------------VICKLSEA---------SASVGG-GKE-MI  196
_KAJ1519446.1_     174  ----------------------MSD-L-------------VICKLSDA---------SASVAG-GKE-MI  196
_ALC78349.1_       173  ----------------------MSD-L-------------VICKLSHC---------SCSVAG-GQE-MI  195
_XP_046738772.1_   174  ----------------------MSD-L-------------VICKLSDC---------CCTVAG-GKE-MI  196
_XP_046417904.1_   174  ----------------------MSD----------------ICKLSDC---------CCTVAG-GKE-II  194
_CRK94170.1_       174  ----------------------MSD-L-------------VITKLSHC---------NALCNG-GQE-MI  196
_CRK94167.1_       174  ----------------------MSD-L-------------VITKLSHC---------NALCNG-GQE-MI  196
_KAG5676160.1_     174  ----------------------MSD-L-------------VITKLSHC---------NAFCNG-GQE-II  196
```

```
_XP_034245464.1_  175  ----------------------MSD-L-------------VICKLSHA---------SAPAIG-GTE-MI  197
_XP_034245457.1_  175  ----------------------MSD-L-------------VICKLSHA---------SAPAIG-GTE-MI  197
_KAG8287980.1_         ----------------------------------------------------------------------
_XP_044759045.1_  169  ----------------------KTD-L-------------IIYRISHC---------VTRVDE-QQE-II  191
_XP_017305229.1_  168  ----------------------YSD-L-------------TIHTLSHV---------SAPVVG-DMK-MI  190
_XP_026675878.1_  168  ----------------------YSD-L-------------TIHTLSHV---------SAPVVG-DMK-MI  190
_KAE8574083.1_    126  ----------------------MAD-L-------------VICKLSHS---------AASVAG-GQD-VI  148
_XP_037926497.1_  174  ----------------------MSD-L-------------VICKLCKC---------SSTVAG-GKE-II  196
_CAD7079567.1_    174  ----------------------MSD-L-------------VICKLCKC---------SSTVAG-GKE-II  196
_XP_037882485.1_  174  ----------------------MSD-L-------------VICRLCSC---------SASVLG-NNQ-II  196
_XP_037037597.1_  174  ----------------------MSD-L-------------VICKLSDA---------SCTVAG-GKE-MI  196
_XP_037037592.1_  174  ----------------------MSD-L-------------VICKLSDA---------SCTVAG-GKE-MI  196
_KAG4075389.1_    174  ----------------------MSD-L-------------VICKLSDA---------SCTVAG-GKE-MI  196
_XP_034101357.1_  174  ----------------------MSD-L-------------VICRLCSC---------SASVLG-NTQ-II  196
_XP_026844963.1_  174  ----------------------MSD-L-------------VICRLCSC---------SATVLG-NTQ-II  196
_EDW28798.1_      174  ----------------------MSD-L-------------VICRLCSC---------SATVLG-NTQ-II  196
_XP_034125966.1_  174  ----------------------MSD-L-------------VICRLCSC---------SATVLG-NTQ-II  196
_XP_034666519.1_  174  ----------------------MSD-L-------------VICRLCSC---------SATVLG-NTQ-II  196
_XP_001356570.3_  174  ----------------------MSD-L-------------VICRLCSC---------SATVLG-NTQ-II  196
_XP_023164577.2_  174  ----------------------MSD-L-------------VICRLCSC---------SASVLG-NTQ-II  196
_XP_017153206.1_  174  ----------------------MSD-L-------------VICRLCSC---------SATVLG-NTQ-II  196
_XP_030564932.1_  174  ----------------------MSD-L-------------VICRLCSC---------SASVLG-NTQ-II  196
_XP_002059145.1_  174  ----------------------MSD-L-------------VICRLCSC---------SASVLG-NTQ-II  196
_EDW04266.1_      174  ----------------------MSD-L-------------VICRLCSC---------SASVLG-NTQ-II  196
_XP_017025862.1_  174  ----------------------MSD-L-------------VICRLCSC---------SATVLG-NTQ-II  196
_XP_017859907.1_  174  ----------------------MSD-L-------------VICRLCSC---------SASVLG-NTQ-II  196
_XP_032584327.1_  174  ----------------------MSD-L-------------VICRLCSC---------SASVLG-NTQ-II  196
_XP_016987288.2_  174  ----------------------MSD-L-------------VICRLCSC---------SATVLG-NTQ-II  196
_XP_017954173.1_  174  ----------------------MSD-L-------------VICRLCSC---------SASVLG-NTQ-II  196
_XP_017854504.1_  174  ----------------------MSD-L-------------VICRLCSC---------SATVLG-NTQ-II  196
_XP_034125965.1_  174  ----------------------MSD-L-------------VICRLCSC---------SATVLG-NTQ-II  196
_XP_034666515.1_  174  ----------------------MSD-L-------------VICRLCSC---------SATVLG-NTQ-II  196
_XP_043641917.1_  174  ----------------------MSD-L-------------VICRLCSC---------SATVLG-NTQ-II  196
_XP_026844960.1_  174  ----------------------MSD-L-------------VICRLCSC---------SATVLG-NTQ-II  196
_XP_015035988.2_  174  ----------------------MSD-L-------------VICRLCSC---------SATVLG-NTQ-II  196
_XP_041450744.1_  174  ----------------------MSD-L-------------VICRLCSC---------SATVLG-NTQ-II  196
_XP_015052361.1_  174  ----------------------MSD-L-------------VICRLCSC---------SATVLG-NTQ-II  196
_XP_039480515.1_  174  ----------------------MSD-L-------------VICRLCSC---------SATVLG-NTQ-II  196
_XP_033248563.1_  174  ----------------------MSD-L-------------VICRLCSC---------SATVLG-NTQ-II  196
```

```
_KAH8412150.1_     174  ----------------------MSD-L-------------VICRLCSC---------SATVLG-NTQ-II  196
_XP_043864974.1_   174  ----------------------MSD-L-------------VICRLCSC---------SASVLG-NTQ-II  196
_XP_020809119.1_   174  ----------------------MSD-L-------------VICRLCSC---------SATVLG-NTQ-II  196
_KAH8298353.1_     174  ----------------------MSD-L-------------VICRLCSC---------SATVLG-NTQ-II  196
_XP_030564927.1_   174  ----------------------MSD-L-------------VICRLCSC---------SASVLG-NTQ-II  196
_XP_041631592.1_   174  ----------------------MSD-L-------------VICRLCSC---------SATVLG-NTQ-II  196
_XP_017859909.1_   174  ----------------------MSD-L-------------VICRLCSC---------SASVLG-NTQ-II  196
_XP_032591602.1_   174  ----------------------MSD-L-------------VICRLCSC---------SASVLG-NTQ-II  196
_XP_023164572.2_   174  ----------------------MSD-L-------------VICRLCSC---------SASVLG-NTQ-II  196
_TDG46699.1_       174  ----------------------MSD-L-------------VICRLCSC---------SASVLG-NTQ-II  196
_KAH8411895.1_     174  ----------------------MSD-L-------------VICRLCSC---------SASVLG-NTQ-II  196
_KAH8377496.1_     174  ----------------------MSD-L-------------VICRLCSC---------SASVLG-NTQ-II  196
_XP_017854500.2_   174  ----------------------MSD-L-------------VICRLCSC---------SATVLG-NTQ-II  196
_XP_016987284.2_   174  ----------------------MSD-L-------------VICRLCSC---------SATVLG-NTQ-II  196
_KAH8293209.1_     174  ----------------------MSD-L-------------VICRLCSC---------SASVLG-NTQ-II  196
_EEC00225.1_       173  ----------------------MSD-L-------------TITKLSHT---------CAPVSG-GLE-MI  195
_XP_029825505.2_   173  ----------------------MSD-L-------------TITKLSHT---------CAPVSG-GLE-MI  195
_KAG0424790.1_     173  ----------------------MSD-L-------------TITKLSHT---------CAPVSG-GLE-MI  195
_XP_037272127.1_   173  ----------------------MSD-L-------------TITKLSHT---------CAPMSG-GLE-MI  195
_XP_054934017.1_   173  ----------------------MSD-L-------------TITKLSHT---------CAPMSG-GLE-MI  195
_XP_050048524.1_   173  ----------------------MSD-L-------------TITKLSHT---------CAPMSG-GLE-MI  195
_XP_037272126.1_   173  ----------------------MSD-L-------------TITKLSHT---------CAPMSG-GLE-MI  195
_XP_037524244.1_   173  ----------------------MSD-L-------------TITKLSHT---------CAPMSG-GLE-MI  195
_XP_037524245.1_   173  ----------------------MSD-L-------------TITKLSHT---------CAPMSG-GLE-MI  195
_XP_049512094.1_   173  ----------------------MSD-L-------------TITKLSHT---------CAPMSG-GLE-MI  195
_XP_037524243.1_   173  ----------------------MSD-L-------------TITKLSHT---------CAPMSG-GLE-MI  195
_XP_037580160.1_   173  ----------------------MSD-L-------------TITKLSHT---------CAPMSG-GLE-MI  195
_XP_037272128.1_   173  ----------------------MSD-L-------------TITKLSHT---------CAPMSG-GLE-MI  195
_KAH9380259.1_     173  ----------------------MSD-L-------------TITKLSHT---------CAPMSG-GLE-MI  195
_KAI1291715.1_     146  ----------------------VPD-L-------------SIMKVSHS---------SGPASG-GTE-II  168
_XP_035209023.1_   167  ----------------------VSD-L-------------NIARLSHY---------SAPVDG-GTE-II  189
_XP_023332679.1_   179  ----------------------KKE-L-------------QICDISDT---------SSPALG-DKK-II  201
_XP_046801942.1_   174  ----------------------MSD-L-------------VICRLCSC---------SATVLG-NTQ-II  196
_XP_037811120.1_   174  ----------------------MSD-L-------------VICRLCSC---------SATVLG-NTQ-II  196
_AFI98401.1_       174  ----------------------MSD-L-------------VICRLCSC---------SASVLG-NTQ-II  196
_AFJ54621.1_       174  ----------------------MSD-L-------------VICRLCSC---------SASVLG-NTQ-II  196
_KAI8126534.1_     174  ----------------------MSD-L-------------VICRLCSC---------SATVLG-NTQ-II  196
_XP_013118266.1_   174  ----------------------MSD-L-------------VICRLCSC---------SATVLG-NTQ-II  196
_XP_023293634.2_   174  ----------------------MSD-L-------------VICRLCSC---------SATVLG-NTQ-II  196
```

```
_XP_037811062.1_  174  ----------------------MSD-L-------------VICRLCSC---------SATVLG-NTQ-II  196
_KNC34132.1_      174  ----------------------MSD-L-------------VICRLCSC---------SATVLG-NTQ-II  196
_TMW48951.1_      174  ----------------------MSD-L-------------VICRLCSC---------SASVLG-NTQ-II  196
_XP_037957363.1_  174  ----------------------MSD-L-------------VICRLCSC---------SASVLG-NTQ-II  196
_XP_005175014.1_  174  ----------------------MSD-L-------------VICRLCSC---------SASVLG-NTQ-II  196
_XP_037957360.1_  174  ----------------------MSD-L-------------VICRLCSC---------SASVLG-NTQ-II  196
_XP_030383330.1_  173  ----------------------MSD-L-------------VICRLCSC---------SASVLG-NTQ-II  195
_XP_030383328.1_  173  ----------------------MSD-L-------------VICRLCSC---------SASVLG-NTQ-II  195
_CAH1707987.1_    173  ----------------------MSD-L-------------VITKLSHC---------NAFCNG-GQE-MI  195
_TMW54796.1_      174  ----------------------MSE-L-------------VISRLSSR---------SASVLG-NTR-II  196
_XP_026836077.1_  174  ----------------------MSD-L-------------VICRLCSC---------SATVLG-NTQ-II  196
_XP_017116803.1_  174  ----------------------MSD-L-------------VICRLCSC---------SATVLG-NTQ-II  196
_XP_039480519.1_  174  ----------------------MSD-L-------------VICRLCSC---------SATVLG-NTQ-II  196
_XP_043641920.1_  174  ----------------------MSD-L-------------VICRLCSC---------SATVLG-NTQ-II  196
_XP_023036748.1_  174  ----------------------MSD-L-------------VICRLCSC---------SASVLG-NTQ-II  196
_XP_002090466.1_  174  ----------------------MSD-L-------------VICRLCSC---------SATVLG-NTQ-II  196
_XP_034471824.1_  174  ----------------------MSD-L-------------VICRLCSC---------SASVLG-NTQ-II  196
_AAT94434.1_      174  ----------------------MSD-L-------------VICRLCSC---------SASVFG-NTQ-II  196
_XP_036670985.1_  174  ----------------------MSD-L-------------VICRLCSC---------SATVLG-NTQ-II  196
_AAA28479.1_      174  ----------------------MSD-L-------------VICRLCSC---------SATVFG-NTQ-II  196
_XP_037708206.1_  174  ----------------------MSD-L-------------VICRLCSC---------SATVLG-NTQ-II  196
_XP_002038915.1_  174  ----------------------MSD-L-------------VICRLCSC---------SATVFG-NTQ-II  196
_XP_016993636.1_  174  ----------------------MSD-L-------------VICRLCSC---------SATVLG-NTQ-II  196
_XP_052852517.1_  174  ----------------------MSD-L-------------VICRLCSC---------SATVLG-NTQ-II  196
_XP_016963139.1_  174  ----------------------MSD-L-------------VICRLCSC---------SATVLG-NTQ-II  196
_XP_033156151.1_  174  ----------------------MSD-L-------------VICRLCSC---------SATVFG-NTQ-II  196
_XP_001974446.1_  174  ----------------------MSD-L-------------VICRLCSC---------SATVLG-NTQ-II  196
_XP_014761886.1_  174  ----------------------MSD-L-------------VICRLCSC---------SATVLG-NTQ-II  196
_XP_017107216.2_  174  ----------------------MSD-L-------------VICRLCSC---------SATVLG-NTQ-II  196
_XP_017044881.1_  173  ----------------------MSD-L-------------VICRLCSC---------SASVLG-NTQ-II  195
_XP_016944798.1_  174  ----------------------MSD-L-------------VICRLCSC---------SATVLG-NTQ-II  196
_XP_017116800.1_  174  ----------------------MSD-L-------------VICRLCSC---------SATVLG-NTQ-II  196
_XP_016993633.2_  174  ----------------------MSD-L-------------VICRLCSC---------SATVLG-NTQ-II  196
_XP_037708198.1_  174  ----------------------MSD-L-------------VICRLCSC---------SATVLG-NTQ-II  196
_XP_032583259.1_  174  ----------------------MSD-L-------------VICRLCSC---------SATVFG-NTQ-II  196
_AAC35296.1_      174  ----------------------MSD-L-------------VICRLCSC---------SATVFG-NTQ-II  196
_NP_001286014.1_  174  ----------------------MSD-L-------------VICRLCSC---------SATVFG-NTQ-II  196
_XP_016963136.1_  174  ----------------------MSD-L-------------VICRLCSC---------SATVLG-NTQ-II  196
_XP_017063904.2_  174  ----------------------MSD-L-------------VICRLCSC---------SATVLG-NTQ-II  196
```

```
_XP_033156135.1_  174  ----------------------MSD-L-------------VICRLCSC---------SATVFG-NTQ-II  196
_XP_052852512.1_  174  ----------------------MSD-L-------------VICRLCSC---------SATVLG-NTQ-II  196
_XP_032307758.1_  174  ----------------------MSD-L-------------VICRLCSC---------SATVLG-NTQ-II  196
_XP_017107215.2_  174  ----------------------MSD-L-------------VICRLCSC---------SATVLG-NTQ-II  196
_AWT86616.1_      173  ----------------------MSD-L-------------VICKLSED---------SAPVVG-GKQ-II  195
_QUF98534.1_      173  ----------------------MSD-L-------------VICKLSED---------SAPVIG-GKQ-II  195
_UNA06287.1_      173  ----------------------MSD-L-------------VICKLSED---------SAPVVG-GKQ-II  195
_AAQ65068.1_      174  ----------------------MSD-L-------------VICRLCSC---------SATVLG-NTQ-II  196
_AAQ64916.1_      174  ----------------------MSD-L-------------VICRLCSC---------SATVFG-NTQ-II  196
_AAQ64921.1_      174  ----------------------MSD-L-------------VICRLCXC---------SATVFG-NTQ-II  196
_AAQ64918.1_      174  ----------------------MSD-L-------------VICRLCSC---------SATVFG-NTQ-II  196
_KAH8245459.1_    199  ----------------------MSD-L-------------VICRLCSC---------SATVLG-NTQ-II  221
_KAH8283155.1_    196  ----------------------MSD-L-------------VICRLCSC---------SATVLG-NTQ-II  218
_KAH8246384.1_    196  ----------------------MSD-L-------------VICRLCSC---------SATVLG-NTQ-II  218
_KAH8366418.1_    196  ----------------------MSD-L-------------VICRLCSC---------SATVLG-NTQ-II  218
_XP_022195378.2_  173  ----------------------MSD-L-------------LICKLSED---------SAPVTG-GKQ-II  195
_XP_039284931.1_  173  ----------------------MSD-L-------------LICKLSED---------SAPVTG-GKQ-II  195
_ALC40091.1_      196  ----------------------MSD-L-------------VICRLCSC---------SATVLG-NTQ-II  218
_XP_036342419.1_  173  ----------------------KLD-L-------------VIHQVCS----------SATVLG-NTQ-IM  194
_XP_022903025.1_  172  ----------------------MSD-L-------------LIVKLSDC---------VCPVDG-GSKDII  195
_XP_022903017.1_  172  ----------------------MSD-L-------------LIVKLSDC---------VCPVDG-GSKDII  195
_XP_036218946.1_  173  ----------------------KSD-L-------------VIREMCS----------SATVLG-NTT-IM  194
_XP_018790401.1_  173  ----------------------KSD-L-------------VIREMCS----------SGTVLG-NTT-IM  194
_KAI9579887.1_    163  ----------------------KTD-L-------------VIRRLCTC---------AGSVKG-NSE-MI  185
_XP_037892537.1_  163  ----------------------KTD-L--------------IRRLCTC---------AGSVKG-NSE-MI  184
_XP_018903705.1_  177  ----------------------MSN-L-------------QICRICPP---------SASVLG-GTE-VI  199
_CAH0395667.1_    177  ----------------------MSN-L-------------QICRICPP---------SASVLG-GTE-VI  199
_XP_050330165.1_  173  ----------------------KSD-L-------------VIREMCS----------SGTVLG-NTT-IM  194
_XP_039970878.1_  173  ----------------------KSD-L-------------VIREMCS----------SGTVLG-NTT-IM  194
_XP_049305234.1_  173  ----------------------KSD-L-------------VIREMCS----------SGTVLG-NKT-IM  194
_GLH04793.1_      180  ----------------------TSE-L-------------LICKMSDC---------SASVAG-GRE-II  202
_XP_015835035.1_  172  ----------------------FPD-P-------------DIFRLSHC---------NASVDG-GKNDII  195
_XP_015835036.1_  172  ----------------------FPD-P-------------DIFRLSHC---------NASVDG-GKNDII  195
_XP_039283832.1_  184  ----------------------TSD-F-------------AIVELSRC---------SAPVDG-GEK-II  206
_XP_031788427.1_  172  ----------------------KAA-P-------------WIDYISHR---------SATADG-GTE-MC  194
_KAJ6646894.1_    174  ----------------------ASE-L-------------RICDISDV---------TSSTSG-GKK-II  196
_XP_026466084.1_  109  ----------------------TSD-L-------------VICELSQC---------TASAAG-GQK-II  131
_XP_044002069.1_  173  -----------------------ND-L-------------KICKLSPD---------NGPACG-GTD-VI  194
_XP_046611735.1_  174  ----------------------LSK-L-------------IIIDLSSN---------YCNVEG-GKQ-II  196
```

```
_XP_046417906.1_  174  ----------------------LSK-L-------------IIVDLSSN---------YCNVEG-GKQ-II  196
_XP_046590309.1_  174  ----------------------LSK-L-------------VIVDLSSN---------YCNVEG-GKQ-II  196
_XP_046473589.1_  174  ----------------------LSK-L-------------VIVDLSSN---------YCNVEG-GKQ-II  196
_XP_019892116.1_   85  ----------------------MSD-L-------------VICRLCSC---------SASVLG-NTQ-II  107
_XP_005183027.3_   85  ----------------------MSD-L-------------VICRLCSC---------SASVLG-NTQ-II  107
_XP_011496598.1_  101  ----------------------TAE-L-------------TICWLSHA---------SASVVG-GKV-MC  123
_AAW67216.1_       73  ----------------------MSD-L-------------VICKLSDV---------TAPVAG-GRE-II   95
_AAW67217.1_       73  ----------------------MSD-L-------------VICKLSDV---------TAPVAG-GRE-II   95
_XP_017489788.1_   77  ----------------------KLD-L-------------VIHQVCS----------SATVLG-NTQ-IM   98
_BAI67894.1_       77  ----------------------MSD-L-------------VICRLCSC---------SASVLG-NTQ-II   99
_XP_054741511.1_  173  ----------------------MSD-L-------------VIRRICSC---------SATVLG-NTK-II  195
_XP_053961684.1_  173  ----------------------MSD-L-------------VIRRICSC---------SATVLG-NTK-II  195
_XP_036341313.1_  173  ----------------------MSD-L-------------VISRICSC---------SATVLG-NTK-II  195
_XP_036341343.1_  173  ----------------------MSD-L-------------VISRICSC---------SATVLG-NTK-II  195
_XP_017471383.1_  173  ----------------------MSD-L-------------VISRICSC---------SATVLG-NTK-II  195
_XP_054741498.1_  173  ----------------------MSD-L-------------VIRRICSC---------SATVLG-NTK-II  195
_XP_053961677.1_  172  ----------------------MSD-L-------------VIRRICSC---------SATVLG-NTK-II  194
_XP_050320008.1_  173  ----------------------MSD-L-------------VICRICEC---------SASVLG-NTK-II  195
_XP_028894490.2_  173  ----------------------MSD-L-------------VICRVCSC---------SATVLG-NTK-II  195
_XP_050320015.1_  173  ----------------------MSD-L-------------VICRICEC---------SASVLG-NTK-II  195
_XP_018787554.1_  173  ----------------------MSD-L-------------VICRICEC---------SASVLG-NTK-II  195
_XP_029407438.2_  173  ----------------------MSD-L-------------VICRICEC---------SASVLG-NTR-II  195
_XP_036225551.1_  173  ----------------------MSD-L-------------VICRICEC---------SASVLG-NTK-II  195
_XP_011178374.2_  173  ----------------------MSD-L-------------VICRVCSC---------SATVLG-NTK-II  195
_XP_039952593.1_  173  ----------------------MSD-L-------------VICRICEC---------SASVLG-NTK-II  195
_XP_018787542.1_  173  ----------------------MSD-L-------------VICRICEC---------SASVLG-NTK-II  195
_XP_011207866.2_  173  ----------------------MSD-L-------------VICRICEC---------SASVLG-NTR-II  195
_XP_014086506.1_  173  ----------------------MSD-L-------------VICRICEC---------SASVLG-NTK-II  195
_XP_004526638.1_  173  ----------------------MSD-LA-----------IVIVRICSC---------SASVLG-NTA-II  197
_XP_017471402.1_  173  ----------------------MSD-LV-------------ISRICSC---------SATVLG-NTK-II  195
_XP_012157903.1_  173  ----------------------MSD-LA-------------IVRICSC---------SASVLG-NTA-II  195
_XP_011185661.2_  173  ----------------------KLD-L-------------VIIQMCS----------SATV-G-NTH-IM  193
_XP_050322757.1_  173  ----------------------KAD-L-------------IIREMCS----------SGTVLG-NTT-IM  194
_XP_039962922.1_  173  ----------------------KAD-L-------------IIREMCS----------SGTVLG-NTT-IM  194
_XP_050322739.1_  173  ----------------------KAD-L-------------IIREMCS----------SGTVLG-NTT-IM  194
_XP_050322749.1_  173  ----------------------KAD-L-------------IIREMCS----------SGTVLG-NTT-IM  194
_XP_050322724.1_  173  ----------------------KAD-L-------------IIREMCS----------SGTVLG-NTT-IM  194
_XP_050322682.1_  173  ----------------------KAD-L-------------IIREMCS----------SGTVLG-NTT-IM  194
_CAG9823112.1_    179  ----------------------MSD-L-------------SITKLSDC---------VSYVDG-GRKDMI  202
```

```
_KAG5873705.1_     174  ----------------------MSD-L-------------CILKLSDC---------VSSVDG-GKKDII  197
_KAH8030364.1_     152  ----------------------MSD-L-------------TITKLSHT---------CAPMSG-GLE-MI  174
_XP_023013891.1_   174  ----------------------MSD-L-------------SIIKLSDC---------VSPVDG-GQKDII  197
_XP_028130780.2_   180  ----------------------MSD-L-------------TIIKLSDC---------SSYVDG-GRKDII  203
_KAG5873704.1_     179  ----------------------MSD-L-------------CILKLSDC---------VSSVDG-GKKDII  202
_CAH1187690.1_     179  ----------------------MSD-L-------------TIIKLSDC---------VSYVDG-GRKDII  202
_CAH1098641.1_     179  ----------------------MSD-L-------------TIIKLSDC---------VSYVDG-GRKDII  202
_XP_023028116.1_   179  ----------------------ACD-L-------------KIVKLSDC---------VSSVAG-GRKEII  202
_XP_031630227.1_   179  ----------------------NGL-L-------------KITEISSQ---------KAPVTG-GKQ-II  201
_KAG5861047.1_      48  ----------------------MSD-L-------------CILKLSDC---------VSSVDG-GKKDII   71
_KAF2880990.1_     174  ----------------------MCD-L-------------VIVKLSDC---------VSSVNG-GTKDII  197
_XP_018326789.1_   173  ----------------------MSD-L-------------VIVKLSDC---------VTTVDG-GKKDII  196
_XP_018326788.1_   173  ----------------------MSD-L-------------VIVKLSDC---------VTTVDG-GKKDII  196
_XP_031339184.1_   173  ----------------------MCD-L-------------VIVKLSDC---------TSSVAG-GTKDVI  196
_XP_045476069.1_   174  ----------------------ASD-L-------------VIVKLSHC---------VSAADGDRPE-II  197
_XP_044734149.1_   173  ----------------------MSD-L-------------AIFQLSTD---------YSSVAG-GQK-VL  195
_XP_044734150.1_   173  ----------------------MSD-L-------------AIFQLSTD---------YSSVAG-GQK-VL  195
_XP_044734148.1_   173  ----------------------MSD-L-------------AIFQLSTD---------YSSVAG-GQK-VL  195
_XP_044760528.1_   174  ----------------------ASD-L-------------VIVKLSHC---------VCPADGDRTE-II  197
_XP_044747918.1_   174  ----------------------SAD-L-------------VIVKLSDC---------NSHVDG-GRKDII  197
_XP_044747917.1_   174  ----------------------SAD-L-------------VIVKLSDC---------NSHVDG-GRKDII  197
_XP_045481017.1_   174  ----------------------SAD-L-------------VIVKLSDC---------NSHVDG-GRKDII  197
_XP_045481018.1_   174  ----------------------SAD-L-------------VIVKLSDC---------NSHVDG-GRKDII  197
_KAF5302274.1_     174  ----------------------MCD-L-------------IIVKLSDC---------VSCVDG-GRKDII  197
_KAF5308883.1_     174  ----------------------MCD-L-------------VIVKLSDC---------VSCVDG-GRKDII  197
_KRT79621.1_       172  ----------------------MCD-L-------------IIVKLSDC---------VCPVDG-GNKDII  195
_BAD20728.1_       172  ----------------------MCD-L-------------VIVKLSDC---------VCPVDG-GNKDLI  195
_KAF2880988.1_     173  ----------------------MCD-L-------------VIVKLSDC---------VSPVNG-GQKDII  196
_CAH1403258.1_          ----------------------------------------------------------------------
_CAD6992094.1_     173  ----------------------VAD-L-------------KIYQMC-----------SSSTAL-GDKPII  194
_XP_004537184.1_   173  ----------------------VAD-L-------------KIYQMC-----------SSSTAL-GDKPII  194
_XP_018572644.1_   174  ----------------------MSD-L-------------SIIKLSDC---------VSFVDG-GRKDII  197
_RZB54393.1__      174  ----------------------MSD-L-------------LIVKLSHC---------VSYVDG-GRNEII  197
_XP_044255725.1_   174  ----------------------MSD-L-------------MIVKLSHC---------NSYVDG-GRNEII  197
_EFA02850.1_       174  ----------------------MSD-L-------------MIVKLSHC---------NSYVDG-GRNEII  197
_NP_001034507.1_   174  ----------------------MSD-L-------------MIVKLSHC---------NSYVDG-GRNEII  197
_CAH1370311.1_     174  ----------------------MSD-L-------------LIVKLSHC---------VSYVDG-GRNEII  197
_KAJ3630692.1_     174  ----------------------MSD-L-------------LIVKLSHC---------VSYVDG-GRNEII  197
_XP_044255724.1_   174  ----------------------MSD-L-------------MIVKLSHC---------NSYVDG-GRNEII  197
```

```
_KAH0811528.1_     174  ----------------------MSD-L-------------LIVKLSHC---------VSYVDG-GRNEII  197
_CAH7716406.1_     173  ----------------------MSD-L-------------SITKLSHC---------NSYVDG-GRKHII  196
_CAH7716405.1_     173  ----------------------MSD-L-------------SITKLSHC---------NSYVDG-GRKHII  196
_CAH7716404.1_     173  ----------------------MSD-L-------------SITKLSHC---------NSYVDG-GRKHII  196
_XP_028130776.2_   174  ----------------------MSD-L-------------TIIKLSDC---------SSYVDG-GRKDII  197
_CAH1985684.1_     173  ----------------------MSD-L-------------CITKLSNC---------NTYVDG-GRKHMI  196
_XP_017781152.1_   174  ----------------------MTD-L-------------TIVKLSDC---------VSSVDG-GRKDII  197
_XP_017781151.1_   174  ----------------------MTD-L-------------TIVKLSDC---------VSSVDG-GRKDII  197
_XP_048522053.1_   171  ----------------------MAD-L-------------SIVQLCSC---------VSHVNG-GKQ-MI  193
_XP_050303070.1_   185  ----------------------MSE-M-------------TIIKLSDC---------VSYVDG-GKKDII  208
_CAG9762924.1_     174  ----------------------MSD-L-------------TIIKLSDC---------TSYVDG-GRKDII  197
_CAH0560654.1_     176  ----------------------MND-L-------------SIIKLSDC---------VSYVDG-GRKDII  199
_CAH0560655.1_     176  ----------------------MND-L-------------SIIKLSDC---------VSYVDG-GRKDII  199
_XP_018572643.1_   173  ----------------------MSD-L-------------SIIKLSDC---------VSFVDG-GRKDII  196
_XP_030763656.1_   174  ----------------------MSD-L-------------TIIKLSHC---------VSYVNGGGKD-II  197
_XP_030763654.1_   174  ----------------------MSD-L-------------TIIKLSHC---------VSYVNGGGKD-II  197
_XP_018577945.1_   174  ----------------------MSD-L-------------AIAELSDC---------VSYVDG-GRKNII  197
_KAF7283276.1_     174  ----------------------MSD-L-------------SIVKLSDC---------VSYVDG-GRKDII  197
_KAF7283275.1_     174  ----------------------MSD-L-------------SIVKLSDC---------VSYVDG-GRKDII  197
_XP_030763617.1_   174  ----------------------VSD-L-------------TIVKLSSR---------VGYVDI-PKN-II  196
_XP_030763619.1_   176  ----------------------VSS-L-------------TIVSLSDC---------VGSVSG-GEEDCI  199
_VEN60240.1_        84  ----------------------MSD-L-------------SITKLSHC---------NSYVDG-GRKHII  107
_KOB78236.1_       160  -----------------------SQ-L-------------VIFDTCPP---------CGLTRG-GDK-MI  181
_ERL85515.1_       114  ----------------------MAD-L-------------SIVQLCS-----------------------  124
_ENN78766.1_       109  ----------------------MAD-L-------------CIVQLCS-----------------------  119
_KAH1019371.1_     109  ----------------------MAD-L-------------CIVQLCS-----------------------  119
_KAI9565420.1_     173  ----------------------MCE-L-------------TICKLSDC---------SSPVSG-GKE-II  195
_XP_045024905.1_   173  ----------------------MCE-L-------------TICKLSDC---------SSPVSG-GKE-II  195
_XP_046447094.1_   173  ----------------------MCE-L-------------TICKLSDC---------TSPANG-GKE-II  195
_KZS08518.1_       173  ----------------------MCE-L-------------TICKLSDC---------SSPVSG-GKE-II  195
_XP_046636307.1_   173  ----------------------MCE-L-------------TICKLSDC---------TSPVNG-GKE-II  195
_EFX79013.1_       173  ----------------------MCE-L-------------TICKLSDC---------TSPVNG-GKE-II  195
_XP_032776606.2_   173  ----------------------MCE-L-------------TICKLSDC---------SSPVSG-GKE-II  195
_XP_046447093.1_   173  ----------------------MCE-L-------------TICKLSDC---------TSPANG-GKE-II  195
_XP_046636306.1_   173  ----------------------MCE-L-------------TICKLSDC---------TSPVNG-GKE-II  195
_CAH0099799.1_     163  ----------------------MCE-L-------------TICKLSDC---------TSPVNG-GKE-II  185
_KAJ1531033.1_     167  ----------------------FSN-L-------------NICEISYS---------SASVEG-GKK-RM  189
_ACJ36225.1_       174  ----------------------TSD-L-------------IICKLSDC---------ASSVAG-GKE-II  196
_ACZ98167.1_       174  ----------------------TSD-L-------------IICKLSDC---------TSSVAG-GKE-II  196
```

```
_AME17867.1_       174  ----------------------TSD-L-------------IICKLSDC---------TSSVAG-GKE-II  196
_XP_042868409.1_   174  ----------------------TSD-L-------------IICKLSDC---------TSSVAG-GKE-II  196
_XP_045102711.1_   174  ----------------------TSD-L-------------IICKLSDC---------TSSVAG-GKE-II  196
_XP_047487558.1_   174  ----------------------TSD-L-------------IICKLSDC---------TSSVAG-GKE-II  196
_QBJ05651.1_       174  ----------------------TSD-L-------------IICKLSDC---------TSSVAG-GKE-II  196
_AZK36046.1_       174  ----------------------TSD-L-------------IICKLSDC---------TSSVAG-GKE-II  196
_XP_045581920.1_   174  ----------------------TSD-L-------------IICKLSDC---------TSSVAG-GKE-II  196
_XP_050704598.1_   174  ----------------------TSD-L-------------IICKLSDC---------TSSVAG-GKE-II  196
_XP_027237995.1_   174  ----------------------TSD-L-------------IICKLSDC---------TSSVAG-GKE-II  196
_AXB88326.1_       174  ----------------------TSD-L-------------IICKLSDC---------TSSVAG-GKE-II  196
_XP_045102712.1_   174  ----------------------TSD-L-------------IICKLSDC---------TSSVAG-GKE-II  196
_MPC17461.1_       174  ----------------------TSD-L-------------IICKLSDC---------TSSVAG-GKE-II  196
_XP_027237994.1_   174  ----------------------TSD-L-------------IICKLSDC---------TSSVAG-GKE-II  196
_XP_027237996.1_   174  ----------------------TSD-L-------------IICKLSDC---------TSSVAG-GKE-II  196
_XP_045102715.1_   174  ----------------------TSD-L-------------IICKLSDC---------TSSVAG-GKE-II  196
_XP_053627990.1_   174  ----------------------TSD-L-------------IICKLSDC---------TSSVAG-GKE-II  196
_XP_053627989.1_   174  ----------------------TSD-L-------------IICKLSDC---------TSSVAG-GKE-II  196
_XP_053627983.1_   174  ----------------------TSD-L-------------IICKLSDC---------TSSVAG-GKE-II  196
_KAB7495058.1_     174  ----------------------TSD-L-------------NICKLSDY---------TSCVSG-GKE-II  196
_KAF2353654.1_     174  ----------------------TCD-L-------------LICKLSDC---------TSSVAG-GKE-VI  196
_ANH22235.1_       174  ----------------------ACD-L-------------NICKLSDC---------TSSVAG-GKE-II  196
_CAD7275741.1_     173  ----------------------VSD-L-------------NICKMSHC---------NDTVAG-GRE-VI  195
_XP_042243403.1_   174  ----------------------TSD-L-------------IICKLSDC---------TSSVAG-GKE-II  196
_QTC09942.1_       174  ----------------------TSD-L-------------IICKLSDC---------TSSVAG-GKE-II  196
_XP_018023738.1_   153  ----------------------TCD-L-------------LICKLSDC---------TSSVAG-GKE-VI  175
_XP_019877552.1_   176  ----------------------MSD-L-------------NIVMLSDA---------VSPCNG-GKL-VI  198
_XP_049825875.1_   174  ----------------------MID-L-------------NIVRMSDS---------VSPCDG-GKE-IL  196
_CAD7649074.1_     174  ----------------------MTE-L-------------IITKLSHC---------SAPCNG-GQE-MI  196
_CAD7630012.1_     174  ----------------------MTE-L-------------IITKLSHC---------SAPCSG-GKE-VI  196
_XP_054165343.1_   175  ----------------------MTE-L-------------IITKLSHC---------SAPCIG-GKE-MI  197
_XP_044254219.1_   172  ----------------------FPD-P-------------DIFRLSHC---------NTCVNG-GKTDII  195
_XP_053201827.1_   174  ----------------------MSD-L-------------IITKLSHF---------SAPVIG-GRE-II  196
_XP_015786990.1_   174  ----------------------MSD-L-------------IITKLSHF---------SASVTG-GKE-II  196
_KAI1295507.1_     174  ----------------------MSE-L-------------VITKLSHC---------SASVQG-GTD-MI  196
_RWS07527.1_       174  ----------------------MSD-L-------------VITKLSHC---------SAPVTG-GRE-VI  196
_RWS29859.1_       174  ----------------------MSD-L-------------VITKLSHC---------SAPVTG-GKE-II  196
_XP_053201825.1_   176  FCFCISF-----ILLLFHILEAMSD-L-------------IITKLSHF---------SAPVIG-GRE-II  215
_XP_054713467.1_   171  ----------------------VSD-L-------------KIAYLSSD---------NAPVEG-SED-FI  193
_XP_046915158.1_   175  ----------------------MAE-L-------------IIVKLSHY---------SAPCTG-GKE-II  197
```

```
_KAJ6217218.1_     175  ----------------------MAE-L-------------VITKLSHY---------SAPCTG-GKE-II  197
_KAI2801352.1_     175  ----------------------MAE-L-------------VITKLSHY---------SAPCTG-GKE-II  197
_KAH9406651.1_     175  ----------------------MAE-L-------------IITKLSHY---------AAPCTG-GKE-II  197
_XP_017483538.1_   175  ----------------------MAE-L-------------IITKLSHY---------AAPCTG-GKE-II  197
_KPM10359.1_       175  ----------------------MAE-L-------------IIAKLSHY---------SAPCTG-GKE-II  197
_UXI17582.1_       175  ----------------------MAE-L-------------IIAKLSHY---------SAPCTG-GKE-II  197
_KAF7488016.1_     175  ----------------------MAE-L-------------IIAKLSHY---------SAPCTG-GKE-II  197
_KAH9423377.1_     175  ----------------------MAE-L-------------IIVKLSHY---------SAPCTG-GKE-II  197
_OTF73464.1_       175  ----------------------MAE-L-------------IIVKLSHY---------SAPCTG-GKE-II  197
_XP_037072056.1_   174  ----------------------MCD-L-------------IIYKMSDC---------SASVAG-GRD-II  196
_XP_037083492.1_   174  ----------------------MCD-L-------------IIYKMSDC---------SASVAG-GKD-II  196
_XP_037083508.1_   174  ----------------------MCD-L-------------IIYKMSDC---------SASVAG-GKD-II  196
_XP_043215018.1_   174  ----------------------MCD-L-------------VIYKMSDC---------SASVAG-GKD-II  196
_KAF0314765.1_     174  ----------------------MCD-L-------------VIYKMSDC---------SASVAG-GKD-II  196
_XP_043215020.1_   174  ----------------------MCD-L-------------VIYKMSDC---------SASVAG-GKD-II  196
_XP_043189654.1_   174  ----------------------MCD-L-------------VIYKMSDC---------SASAAG-GRD-II  196
_KAF0293344.1_     160  ----------------------MCD-L-------------VIYKMSDC---------SASAAG-GRD-II  182
_XP_011496609.1_   175  ----------------------RMK-P-------------IINFISHE---------SASVAG-GTQ-MC  197
_AGS12619.1_       174  ----------------------KND-L-------------TICRMTEY---------SASVAG-GKE-ML  196
_TRY78048.1_       174  ----------------------KND-L-------------TICRMTEY---------SASVAG-GKE-ML  196
_XP_040576307.1_   174  ----------------------RSD-L-------------TICRMTEY---------SAPVTG-GKE-ML  196
_CAB4056363.1_     174  ----------------------RSD-L-------------TICRMTEY---------SAPVTG-GKE-ML  196
_ALF84898.1_       174  ----------------------RSD-L-------------TICRMTEC---------TASVAG-GKE-IL  196
_XP_023349538.1_   170  ----------------------KCD-L-------------TICRLTET---------SASVAG-GKE-IL  192
_XP_004522582.1_   173  ----------------------LPD-L-------------TIIRICSC---------SASVLG-NTN-II  195
_CAD7013622.1_     173  ----------------------LPD-L-------------TIIRICSC---------SASVLG-NTN-II  195
_XP_042902217.1_   173  ----------------------MSD-L-------------TIMKLSHY---------SAPVTG-GTE-VI  195
_XP_042902218.1_   173  ----------------------MSD-L-------------TIMKLSHY---------SAPVTG-GTE-VI  195
_XP_035215470.1_   173  ----------------------MSD-L-------------TIMKLSHY---------SAPVTG-GTE-VI  195
_GBN14612.1_       172  ----------------------MSD-L-------------TIMKLSHY---------SAPVTG-GTE-VI  194
_KAF8795965.1_     173  ----------------------MSD-L-------------TIMKLSHY---------SAPVTG-GTE-VI  195
_KAF8795964.1_     173  ----------------------MSD-L-------------TIMKLSHY---------SAPVTG-GTE-VI  195
_GFY66827.1_       173  ----------------------MSD-L-------------TIMKLSHY---------SAPVTG-GTE-VI  195
_GFW66965.1_       173  ----------------------MSD-L-------------TIMKLSHY---------SAPVTG-GTE-VI  195
_GFQ85823.1_       173  ----------------------MSD-L-------------TIMKLSHY---------SAPVTG-GTE-VI  195
_GFT35953.1_       173  ----------------------MSD-L-------------TIMKLSHY---------SAPVTG-GTE-VI  195
_XP_054714028.1_   173  ----------------------MSD-L-------------TIIRLSHY---------SAPVTG-GTE-VI  195
_KAG8194583.1_     172  ----------------------MSD-L-------------TIMKISHY---------SAPVTG-GTE-VI  194
_GIY09899.1_       172  ----------------------MSD-L-------------TIMKLSHY---------SAPVSG-GTE-VI  194
```

```
_GIY93593.1_        172  ----------------------MSD-L-------------TIMKLSHY---------SASVSG-GTE-VI  194
_XP_042902221.1_    185  ----------------------ISD-L-------------TIMKFSHY---------SAPATG-GTE-VI  207
_XP_046962867.1_    176  ----------------------MSD-L-------------VIMRLSQC---------SDFHKG-GTE-LI  198
_XP_047530540.1_    176  ----------------------MSD-L-------------VIMRLSQC---------SDFHKG-GTE-LI  198
_XP_026486466.1_    176  ----------------------MSD-L-------------VIMRLSQC---------SDFHKG-GTE-LI  198
_XP_047530539.1_    176  ----------------------MSD-L-------------VIMRLSQC---------SDFHKG-GTE-LI  198
_XP_047530538.1_    176  ----------------------MSD-L-------------VIMRLSQC---------SDFHKG-GTE-LI  198
_XP_047530537.1_    176  ----------------------MSD-L-------------VIMRLSQC---------SDFHKG-GTE-LI  198
_XP_026486464.1_    176  ----------------------MSD-L-------------VIMRLSQC---------SDFHKG-GTE-LI  198
_XP_047530541.1_    176  ----------------------MSD-L-------------VIMRLSQC---------SDFHKG-GTE-LI  198
_XP_050347286.1_    176  ----------------------MSD-L-------------VIMRLSQC---------SDFHKG-GTE-LI  198
_XP_050347283.1_    176  ----------------------MSD-L-------------VIMRLSQC---------SDFHKG-GTE-LI  198
_XP_032515351.1_    177  ----------------------MSD-L-------------VIMRVSQC---------SDFVKG-GAE-II  199
_OWR46016.1_        177  ----------------------MSD-L-------------VIMRVSQC---------SDFVKG-GAE-II  199
_XP_032515346.1_    177  ----------------------MSD-L-------------VIMRVSQC---------SDFVKG-GAE-II  199
_CAG9579094.1_      177  ----------------------MSD-L-------------VIMRVSQC---------SDFVKG-GAE-II  199
_CAG9579091.1_      177  ----------------------MSD-L-------------VIMRVSQC---------SDFVKG-GAE-II  199
_XP_050668638.1_    175  ----------------------MSD-L-------------LIMRISQC---------SASPRG-GTE-IL  197
_XP_050668632.1_    175  ----------------------MSD-L-------------LIMRISQC---------SASPRG-GTE-IL  197
_XP_045495329.1_    175  ----------------------MSD-L-------------VIMRMSQC---------SATPRG-GTE-IF  197
_XP_038214802.1_    175  ----------------------MSD-L-------------VIMRMSQC---------SATPRG-GTE-IF  197
_CAH0714714.1_      176  ----------------------MSD-L-------------TIMRLSQC---------SDFSKG-GTE-LI  198
_CAH0714713.1_      176  ----------------------MSD-L-------------TIMRLSQC---------SDFSKG-GTE-LI  198
_CAH4029256.1_      175  ----------------------MSD-L-------------VIMRLSHC---------SAPPQG-GME-MF  197
_XP_047513081.1_    175  ----------------------MSD-L-------------VIMRLSHC---------SAPPQG-GME-MF  197
_XP_045529569.1_    175  ----------------------MSD-L-------------VIMRLSHC---------SAPPQG-GME-MF  197
_XP_045487500.1_    175  ----------------------MSD-L-------------VIMRLSHC---------SAPPQG-GME-MF  197
_XP_047513077.1_    175  ----------------------MSD-L-------------VIMRLSHC---------SAPPQG-GME-MF  197
_CAH4029257.1_      175  ----------------------MSD-L-------------VIMRLSHC---------SAPPQG-GME-MF  197
_XP_045487498.1_    175  ----------------------MSD-L-------------VIMRLSHC---------SAPPQG-GME-MF  197
_CAF4952989.1_      175  ----------------------MSD-L-------------VIMRLSHC---------SAPPQG-GME-MF  197
_XP_045447470.1_    176  ----------------------MSD-L-------------VIMRLSQC---------SDYHRG-GTE-LI  198
_CAH2108688.1_      115  ----------------------MSD-L-------------VIMRLSQC---------SDFHKG-GTE-LI  137
_CAH0714724.1_      174  ----------------------LMN-L-------------TIRELSIT---------SDYHFG-GRR-LI  196
_XP_034827218.1_    176  ----------------------LSD-L-------------IITRISHC---------SGSAKG-DTE-VI  198
_XP_045768234.1_    176  ----------------------LSD-L-------------IITRISHC---------SGSAKG-DTE-VI  198
_XP_034827214.1_    176  ----------------------LSD-L-------------IITRISHC---------SGSAKG-DTE-VI  198
_XP_045768230.1_    176  ----------------------LSD-L-------------IITRISHC---------SGSAKG-DTE-VI  198
_XP_039751073.1_    176  ----------------------LSD-L-------------IITRVSHC---------SGHAKG-DTE-VI  198
```

```
_XP_023942270.1_  176  ----------------------LSD-L-------------IITRVSHC---------SGYAKG-DTE-VI  198
_XP_023942268.1_  176  ----------------------LSD-L-------------IITRVSHC---------SGYAKG-DTE-VI  198
_XP_039751072.1_  176  ----------------------LSD-L-------------IITRVSHC---------SGHAKG-DTE-VI  198
_XP_023942266.1_  176  ----------------------LSD-L-------------IITRVSHC---------SGYAKG-DTE-VI  198
_CAH2240541.1_    163  ----------------------LSD-L-------------IITRVSHC---------SGHAKG-DTE-VI  185
_XP_041985045.1_  176  ----------------------MSD-L-------------FITKLSHC---------SASPKG-GTE-VI  198
_XP_041985024.1_  176  ----------------------MSD-L-------------FITKLSHC---------SASPKG-GTE-VI  198
_XP_049868286.1_  175  ----------------------MSD-L-------------HILRISSS---------AGAARG-GTE-LI  197
_XP_026314794.1_  175  ----------------------MTD-L-------------QILRISRS---------SGSARG-GTE-LI  197
_XP_047994271.1_  175  ----------------------MCE-L-------------QINRLSTC---------AASVRG-GTE-LI  197
_KAJ2950301.1_    172  ----------------------MSD-L-------------QILRISSS---------AGPARG-GQD-LI  194
_XP_031784347.2_  171  -----------------------KK-L-------------QIVWRSHF---------SVPVSG-GTV-VC  192
_KAG7305875.1_    174  ----------------------MSD-L-------------VITRLSHC---------SAPAQG-GKQ-VI  196
_WDD23886.1_      174  ----------------------MSD-L-------------VITRLSHC---------SAPAQG-GKQ-VI  196
_XP_048480424.1_  174  ----------------------MSD-L-------------VITRLSHC---------SAPAQG-GKQ-VI  196
_XP_048480423.1_  174  ----------------------MSD-L-------------VITRLSHC---------SAPAQG-GKQ-VI  196
_XP_048480422.1_  174  ----------------------MSD-L-------------VITRLSHC---------SAPAQG-GKQ-VI  196
_XP_048480421.1_  174  ----------------------MSD-L-------------VITRLSHC---------SAPAQG-GKQ-VI  196
_KAG7305876.1_    174  ----------------------MSD-L-------------VITRLSHC---------SAPAQG-GKQ-VI  196
_CAG9129242.1_    174  ----------------------MSD-L-------------VITRLSHC---------SAPAQG-GKQ-VI  196
_XP_048480420.1_  174  ----------------------MSD-L-------------VITRLSHC---------SAPAQG-GKQ-VI  196
_GBP53004.1_       48  ----------------------MSD-L-------------VICRLSHC---------SAPVTG-GQQ-VI   70
_XP_045534737.1_  175  ----------------------MSD-L-------------HIARVSHC---------TGPAGG-RMT-MI  197
_XP_013180060.1_  175  ----------------------MSD-L-------------HIARVSHC---------AGPAGG-RMT-MI  197
_XP_013180043.1_  175  ----------------------MSD-L-------------HIARVSHC---------AGPAGG-RMT-MI  197
_XP_013180068.1_  175  ----------------------MSD-L-------------HIARVSHC---------AGPAGG-RMT-MI  197
_XP_045534736.1_  175  ----------------------MSD-L-------------HIARVSHC---------TGPAGG-RMT-MI  197
_KPJ06840.1_      175  ----------------------MSD-L-------------HIARVSHC---------TGPAGG-RMT-MI  197
_KPJ05518.1_      175  ----------------------MSD-L-------------HIARVSHC---------AGPAGG-RMT-MI  197
_CAG4934519.1_    175  ----------------------MSD-L-------------HITRISHC---------AGPVRG-RMT-MI  197
_XP_013149112.1_  175  ----------------------MSD-L-------------HIARVSHC---------AGPAAG-RMT-MI  197
_CAH2077165.1_    187  ----------------------MSE-L-------------HIARISHC---------SGPVRG-RMT-MI  209
_CAH2077167.1_    187  ----------------------MSE-L-------------HIARISHC---------SGPVRG-RMT-MI  209
_XP_037051415.1_  169  --------------------------L-------------EISDISDT---------TSPTTG-GKK-II  188
_XP_037051414.1_  167  --------------------------F-------------EILDISDT---------TSPTTG-GKK-II  186
_XP_022815079.1_  175  ----------------------MSD-L-------------LIMRSSHC---------SGPAKG-GTQ-VI  197
_CAB3516522.1_    175  ----------------------MSD-L-------------LIMRSSHC---------SGPAKG-GTQ-VI  197
_XP_050562120.1_  175  ----------------------MSD-L-------------LIMRSSHC---------SGPAKG-GTQ-VI  197
_XP_050562121.1_  175  ----------------------MSD-L-------------LIMRSSHC---------SGPAKG-GTQ-VI  197
```

```
_QHD56218.1_       175  ----------------------MSD-L-------------LIMRSSHC---------SGPAKG-GTQ-VI  197
_AIA24466.1_       175  ----------------------MSD-L-------------LIMRSSHC---------SGPAKG-GTQ-VI  197
_XP_022815076.1_   175  ----------------------MSD-L-------------LIMRSSHC---------SGPAKG-GTQ-VI  197
_QHD56217.1_       175  ----------------------MSD-L-------------LIMRSSHC---------SGPAKG-GTQ-VI  197
_XP_050562118.1_   175  ----------------------MSD-L-------------LIMRSSHC---------SGPAKG-GTQ-VI  197
_AIA24467.1_       175  ----------------------MSD-L-------------LIMRSSHC---------SGPAKG-GTQ-VI  197
_XP_047019808.1_   175  ----------------------MSD-L-------------LIMRSSHC---------SGTARG-GTQ-VI  197
_AEO51736.1_       175  ----------------------MSD-L-------------LIMRSSHC---------SGTARG-GTQ-VI  197
_XP_049696680.1_   175  ----------------------MSD-L-------------LIMRSSHC---------SGTARG-GTQ-VI  197
_XP_049696682.1_   175  ----------------------MSD-L-------------LIMRSSHC---------SGTARG-GTQ-VI  197
_XP_049696676.1_   175  ----------------------MSD-L-------------LIMRSSHC---------SGTARG-GTQ-VI  197
_XP_026744931.1_   175  ----------------------MSD-L-------------HIMRSSHC---------SGPARG-GTQ-VI  197
_XP_026732895.1_   175  ----------------------MSD-L-------------HIMRSSHC---------SGPARG-GTQ-VI  197
_CAH0581650.1_     175  ----------------------MSD-L-------------HIMRSSHC---------SGPARG-GTQ-VI  197
_KAH9640014.1_     220  ----------------------MSD-L-------------LIMRSSHC---------SGPAKG-GTQ-VI  242
_PZC78315.1_        49  ----------------------MSD-L-------------LIMRSSHC---------SGTARG-GTQ-VI   71
_KAF9795796.1_      49  ----------------------MSD-L-------------LIMRSSHC---------SGPAKG-GTQ-VI   71
_AEC52926.1_       175  ----------------------MSD-L-------------LIMRASHC---------SGRARG-GTQ-VI  197
_KAH7940301.1_     174  ----------------------FSN-L-------------------C----------ESPS---------  182
_ADK39025.1_       174  ----------------------MSD-L-------------LIVRASHC---------AGPARG-GTQ-VV  196
_XP_037298148.1_   174  ----------------------MSD-L-------------LIMRASHC---------AGPARG-GTQ-VI  196
_RVE50742.1_       175  ----------------------MSE-L-------------LIVRSSHC---------SGPVRG-GTT-VI  197
_CAH0692665.1_     175  ----------------------MSE-L-------------LIVRSSHC---------SGPVRG-GTT-VI  197
_XP_028166351.1_   174  ----------------------MSD-L-------------LIVRSSHC---------SGSVKG-GTT-VI  196
_XP_028166291.1_   174  ----------------------MSD-L-------------LIVRSSHC---------SGSVKG-GTT-VI  196
_CAB3235314.1_     175  ----------------------MSD-L-------------LIVRASHC---------SGTAKG-GTQ-VI  197
_KAJ0175867.1_     176  ----------------------MSD-L-------------QIMRASHC---------SGTARG-GNH-VI  198
_XP_028031027.1_   175  ----------------------MSD-L-------------LIMRSSHC---------SGTIRG-GTQ-VI  197
_NP_001166296.1_   175  ----------------------MSD-L-------------LIMRSSHC---------SGTIRG-GTQ-VI  197
_XP_037870194.1_   175  ----------------------MSD-L-------------LIMRSSHC---------SGTIRG-GTQ-VI  197
_XP_037870193.1_   175  ----------------------MSD-L-------------LIMRSSHC---------SGTIRG-GTQ-VI  197
_XP_028031025.1_   175  ----------------------MSD-L-------------LIMRSSHC---------SGTIRG-GTQ-VI  197
_XP_013187225.1_   173  ----------------------MSD-L-------------QIAQISHC---------SGPAKG-GTR-VI  195
_XP_052755011.1_   173  ----------------------MSE-L-------------QIAQISHC---------SGPAKG-GTR-VI  195
_XP_053613182.1_   173  ----------------------MSD-L-------------QIAQISHC---------SGPMKG-GTR-VI  195
_XP_053613180.1_   173  ----------------------MSD-L-------------QIAQISHC---------SGPMKG-GTR-VI  195
_CAG9785863.1_     173  ----------------------MSD-L-------------LIVRSSHC---------SGPVRG-GTT-VI  195
_KAG6461275.1_     174  ----------------------MSD-L-------------LIMRASHC---------AGPARG-GTQ-VI  196
_KAG6461281.1_     174  ----------------------MSD-L-------------LIMRASHC---------AGPARG-GTQ-VI  196
```

```
_XP_037302137.1_  172  ----------------------AGE-L-------------HISYPSHC---------SGPACG-GIQ-VT  194
_KAG6461269.1_    172  ----------------------AGE-L-------------HISYPSHC---------SGPACG-GIQ-VT  194
_KAG4069896.1_    163  -----------------------DN-L-------------EIRDISDT---------TSTTTG-RKK-II  184
_XP_044003989.1_  178  ---------------------ANNG-M-------------TICSVSHD---------SVPASG-GQK-II  201
_XP_045768240.1_  176  ----------------------MNE-L-------------IITCISRC---------SGSMNG-DTR-VI  198
_KAG9509099.1_    180  ----------------------MSD-L-------------IITKLSHV---------TAPATG-GRE-II  202
_XP_013118267.1_  172  ----------------------IND-L-------------VICRLCSC---------SAKASG-GDT-IF  194
_XP_005183026.2_  172  ----------------------INE-L-------------VICRLCSC---------SAKASG-GDT-IF  194
_XP_050303432.1_  187  ----------------------VPD-L-------------AITRISDC---------ISDADGNGKE-II  210
_TMW48950.1_      173  ----------------------IND-L-------------VICRLCSC---------AAKVAG-GDK-II  195
_XP_037882085.1_  172  ----------------------VND-L-------------AINRLCSC---------SAKMSG-GDE-II  194
_KAI8126541.1_    174  ----------------------IND-L-------------VICRLCSC---------AAKVTG-GDT-II  196
_KAI8126540.1_    173  ----------------------IND-L-------------VICRLCSC---------AAKVTG-GDT-II  195
_XP_037811156.1_  173  ----------------------IND-L-------------VICRLCSC---------AAKVTG-GDS-II  195
_XP_046812595.1_  173  ----------------------IND-L-------------VICRLCSC---------AAKVTG-GDT-II  195
_XP_046812596.1_  173  ----------------------IND-L-------------VICRLCSC---------AAKVTG-GDT-II  195
_XP_046812591.1_  173  ----------------------IND-L-------------VICRLCSC---------AAKVTG-GDT-II  195
_XP_046812590.1_  173  ----------------------IND-L-------------VICRLCSC---------AAKVTG-GDT-II  195
_KNC34143.1_      173  ----------------------IND-L-------------VICRLCSC---------AAKVTG-GDT-II  195
_XP_046812589.1_  173  ----------------------IND-L-------------VICRLCSC---------AAKVTG-GDT-II  195
_KAI8126539.1_    173  ----------------------IND-L-------------VICRLCSC---------AAKVTG-GDT-II  195
_XP_037958917.1_  171  ----------------------MSD-L-------------VVIRLCCR---------SAKVGG-GNE-II  193
_XP_037929595.1_  173  ----------------------MSD-L-------------VICRLCCC---------SAKVGG-GDE-II  195
_XP_037957513.1_  171  ----------------------MSD-L-------------VICRLCCC---------SAKVGG-GDE-II  193
_XP_037958115.1_  173  ----------------------MSD-L-------------VICRLCCC---------SAKVGG-GDE-II  195
_XP_037958114.1_  173  ----------------------MSD-L-------------VICRLCCC---------SAKVGG-GDE-II  195
_XP_037957511.1_  171  ----------------------MSD-L-------------VICRLCCC---------SAKVGG-GDE-II  193
_XP_037958918.1_  171  ----------------------ILD-L-------------VIYRLCCC---------SAKVDG-GDR-II  193
_RXG71823.1_           ----------------------------------------------------------------------
_KAB7494622.1_         ----------------------------------------------------------------------
_KAI9584339.1_    172  ----------------------MSE-L-------------EICRVCSC---------TASVDG-GTE-NI  194
_XP_037884983.1_  172  ----------------------MSE-L-------------KICHVCAC---------PASVDG-GTE-HI  194
_XP_054744821.1_  171  ----------------------KSE-L-------------VISRLCSC---------AATIDG-GDE-II  193
_XP_053961676.1_  171  ----------------------KSE-L-------------VISRLCSC---------AATIDG-GDE-II  193
_XP_053961674.1_  171  ----------------------KSE-L-------------VISRLCSC---------AATIDG-GDE-II  193
_XP_004526634.2_  171  ----------------------KSE-L-------------VISRLCSC---------AATIDG-GDE-II  193
_CAD6998451.1_    171  ----------------------KSE-L-------------VISRLCSC---------AATIDG-GDE-II  193
_XP_012157894.1_  171  ----------------------KSE-L-------------VISRLCSC---------AATIDG-GDE-II  193
_XP_028894493.1_  171  ----------------------KSE-L-------------LISRLCSC---------AASIDG-CDE-II  193
```

```
_XP_036327693.1_   171  ----------------------KSE-L-------------VISRLCSC---------SATIEG-GQE-II  193
_XP_017471333.1_   171  ----------------------KSE-L-------------VISRLCSC---------SATIEG-GQE-II  193
_XP_039952608.1_   171  ----------------------KSE-L-------------LISRLCSC---------AATIDG-GDE-II  193
_XP_050320023.1_   171  ----------------------KSE-L-------------LISRLCSC---------AATIDG-GDE-II  193
_XP_036225572.1_   171  ----------------------KSE-L-------------LISRLCSC---------AATIDG-GDE-II  193
_XP_039952607.1_   171  ----------------------KSE-L-------------LISRLCSC---------AATIDG-GDE-II  193
_XP_036225564.1_   171  ----------------------KSE-L-------------LISRLCSC---------AATIDG-GDE-II  193
_XP_036225569.1_   171  ----------------------KSE-L-------------LISRLCSC---------AATIDG-GDE-II  193
_XP_050320021.1_   171  ----------------------KSE-L-------------LISRLCSC---------AATIDG-GDE-II  193
_XP_039952606.1_   171  ----------------------KSE-L-------------LISRLCSC---------AATIDG-GDE-II  193
_XP_011207860.1_   171  ----------------------KSE-L-------------LISRLCSC---------AATIDG-GDE-II  193
_XP_011207859.1_   171  ----------------------KSE-L-------------LISRLCSC---------AATIDG-GDE-II  193
_XP_029407466.1_   171  ----------------------KSE-L-------------LISRLCSC---------AATIDG-GDE-II  193
_XP_019847003.2_   171  ----------------------KSE-L-------------LISRLCSC---------AATIDG-GDE-II  193
_XP_014086488.2_   171  ----------------------KSE-L-------------LISRLCSC---------AATIDG-GDE-II  193
_XP_049317393.1_   171  ----------------------KSE-L-------------LISRLCSC---------AATIDG-GDE-II  193
_XP_039952604.1_   171  ----------------------KSE-L-------------LISRLCSC---------AATIDG-GDE-II  193
_XP_018787557.1_   171  ----------------------KSE-L-------------LISRLCSC---------AATIDG-GDE-II  193
_OQR68950.1_       175  ----------------------MSD-L-------------QITKLSHS---------LAPLHG-GLE-MV  197
_XP_003741763.1_   175  ----------------------MSD-L-------------HITKLSHP---------VAPMAG-GLE-MI  197
_XP_022699297.1_   175  ----------------------MSD-L-------------QITKLSHI---------LAPLRG-GLE-MI  197
_XP_022665229.1_   175  ----------------------MSD-L-------------QITKLSHI---------LAPLRG-GLE-MI  197
_XP_022665225.1_   175  ----------------------MSD-L-------------QITKLSHI---------LAPLRG-GLE-MI  197
_XP_022665224.1_   175  ----------------------MSD-L-------------QITKLSHI---------LAPLRG-GLE-MI  197
_XP_033219687.1_   173  ----------------------VEE-P-------------VIHKLSHC---------SASVAG-GME-MI  195
_XP_033227990.1_   173  ----------------------VEE-P-------------VIHKLSHC---------GAPVDG-DME-MI  195
_XP_043466013.1_   172  ----------------------NED-L-------------VICKLSVC---------SAPVSG-GTE-VI  194
_XP_051172671.1_   172  ----------------------NED-L-------------IICKLSVC---------SAPVSG-GTE-VI  194
_CAD7643228.1_     171  ----------------------MSE-L-------------AIAKLSHY---------SAPVGG-GQD-IV  193
_XP_054157157.1_   174  ----------------------MSE-L-------------AIGKLSHC---------SAPVTG-GQE-VI  196
_CAD7645633.1_     172  ----------------------MSE-L-------------SIAKLSHY---------SAPASG-GQE-II  194
_CAD7632611.1_     113  ----------------------MSE-L-------------AIAKLSHY---------SAPVGG-GQD-IV  135
_KAJ6223158.1_     171  ----------------------MSE-L-------------SIIRLSHY---------SATTTG-GEQ-VI  193
_KAI2804035.1_     171  ----------------------MSE-L-------------SIIRLSHY---------SATTTG-GEQ-VI  193
_KAH9398209.1_     163  ----------------------MSE-L-------------SIAKLSHY---------SAHIGG-GQS-VV  185
_GFY77286.1_       173  ----------------------ISD-L-------------TITKLSHY---------SAPVTG-GQE-VI  195
_GFQ73388.1_       173  ----------------------ISD-L-------------TITKLSHY---------SAPVTG-GQE-VI  195
_GFT25575.1_       173  ----------------------ISD-L-------------TITKLSHY---------SAPVTG-GQE-VI  195
_KAF8763655.1_     173  ----------------------ISD-L-------------TITKLSHY---------SAPVTG-GQE-VI  195
```

```
_GFX76677.1_       173  ----------------------ISD-L-------------TITKLSHC---------SAPVTG-GQE-VI  195
_XP_035207052.1_   173  ----------------------VSD-L-------------TITKLSHS---------SAPVTG-GQE-II  195
_KFM74898.1_       173  ----------------------VSD-L-------------TITKLSHS---------SAPVTG-GQE-II  195
_XP_035207059.1_   173  ----------------------VSD-L-------------TITKLSHS---------SAPVTG-GQE-II  195
_XP_035207066.1_   173  ----------------------VSD-L-------------TITKLSHS---------SAPVTG-GQE-II  195
_BBD75287.1_       173  ----------------------VSD-L-------------TITKLSHS---------SAPVTG-GQE-VI  195
_XP_042900952.1_   173  ----------------------VSD-L-------------TITKLSHS---------SAPVTG-GQE-VI  195
_KAG8201816.1_     172  ----------------------ISP-L-------------TITKLSHY---------SSAVIG-GQE-VI  194
_GIX87435.1_       173  ----------------------ISD-L-------------TITKLSHY---------SAPVTG-GQE-VL  195
_GBL81029.1_       173  ----------------------ISD-L-------------TITKLSHY---------SAPVTG-GQE-VI  195
_GIY01087.1_       173  ----------------------ISD-L-------------TITKLSHY---------SAPVTG-GQE-VI  195
_XP_054715436.1_   173  ----------------------VSD-L-------------TIIRLSHY---------CAPVTG-GQE-II  195
_XP_044576112.1_   170  ----------------------LND-L-------------VIIKLSHY---------SAPVTG-GLP-MI  192
_XP_008556435.3_   169  ----------------------LGD-L-------------SILKLSHY---------SAPATG-GTP-MI  191
_CAD6234446.1_     170  ----------------------LND-L-------------VIIKLSHC---------SAPVTG-GVP-MI  192
_XP_044284598.1_   173  ---------------------NSAE-L-------------RICRVNKN---------CGSVKG-GDE-IF  196
_XP_033794511.1_   173  ---------------------NTAE-L-------------KICRVNKN---------CGSVKG-GDE-IF  196
_XP_033794512.1_   173  ---------------------NTAE-L-------------KICRVNKN---------CGSVKG-GDE-IF  196
_XP_029449578.1_   173  ---------------------NTAE-L-------------RICRVNKN---------CGSIRG-GDE-IF  196
_XP_030052048.1_   173  ---------------------NTAE-L-------------RICRVNKN---------CGSVRG-GDE-IF  196
_XP_007423639.1_   173  ---------------------NSAE-L-------------RICRVNKN---------CGTVKG-GDE-IF  196
_XP_039203483.1_   173  ---------------------NSAE-L-------------RICRVNKN---------CGTVKG-GDE-IF  196
_XP_034283206.1_   173  ---------------------NSAE-L-------------RICRVNKN---------CGTAKG-GDE-IF  196
_XP_033001023.1_   173  ---------------------NSAE-L-------------RICRVNKN---------CGSVKG-GDE-IF  196
_XP_053239675.1_   173  ---------------------NSAE-L-------------RICRVNKN---------CGSVKG-GDE-IF  196
_XP_034966804.1_   173  ---------------------NSAE-L-------------RICRVNKN---------CGSVKG-GDE-IF  196
_XP_028580982.1_   162  ---------------------NSAE-L-------------RICRVNKN---------CGSVKG-GDE-IF  185
_XP_042302822.1_   174  ----------------------SAE-L------------RRICRVNKN---------CGSVKG-GDE-IF  197
_XP_015280979.1_   162  ---------------------NSAE-L-------------RICRVNKN---------CGTAKG-GDE-IF  185
_XP_048363544.1_   173  ---------------------KSAE-L-------------RICRVNKN---------CGTVKG-ADE-IF  196
_KAF7252806.1_     147  ---------------------NSAE-L-------------RICRVNKN---------CGSVKG-GDE-IF  170
_XP_034958398.1_    52  ---------------------NSAE-L-------------RICRVNKN---------CGSVKG-GDE-IF   75
_CAD5116312.1_     176  ----------------------PGE-M-------------KICRVSAC---------AGSVNG-GTE-IF  198
_KAJ1519398.1_     167  -----------------------PP-L-------------RIHALSQP---------GASVAG-HHH-MM  188
_XP_053103001.1_   173  ---------------------NSAE-L-------------RICRVNKN---------CGSVKG-GDE-IF  196
_XP_032070730.1_   173  ---------------------NSAE-L-------------RICRVNKN---------CGTAKG-GDE-IF  196
_XP_013922116.1_   173  ---------------------NSAE-L-------------RICRVNKN---------CGTAKG-GDE-IF  196
_XP_015667999.1_   173  ---------------------NSAE-L-------------RICRVNKN---------CGTVKG-GDE-IF  196
_XP_026551846.1_   173  ---------------------NSAE-L-------------RICRVNKN---------CGTVKG-GDE-IF  196
```

```
_XP_026532518.1_  173  ---------------------NSAE-L-------------RICRVNKN---------CGTATG-GDE-IF  196
_KAJ7341301.1_    173  ---------------------NSAE-L-------------RICRVNKN---------CGSVKG-GDE-IF  196
_KAJ6667038.1_    162  ---------------------NSAE-L-------------RICRVNKN---------CGNVKG-GDE-IF  185
_XP_003227818.2_  162  ---------------------KSAE-L-------------RICRVNKN---------CGSVKG-GDE-IF  185
_XP_020633036.1_  163  ----------------------CSG-IFSIQKELLSTYVCLICRVNKN---------CGSVKG-GDE-IF  198
_XP_054842059.1_  173  ---------------------NSAE-L-------------RICRVNKN---------CGTVKG-GDE-IF  196
_XP_005238641.2_  162  ---------------------NTAE-L-------------RICRVNKN---------CGSVKG-GDE-IF  185
_XP_005445671.2_  162  ---------------------NTAE-L-------------RICRVNKN---------CGSVKG-GDE-IF  185
_XP_054050029.1_  162  ---------------------NTAE-L-------------RICRVNKN---------CGSVKG-GDE-IF  185
_XP_013810845.1_  162  ---------------------NTAE-L-------------RICRVNKN---------CGSVKG-GDE-IF  185
_XP_026702002.1_  162  ---------------------NTAE-L-------------RICRVNKN---------CGSVKG-GDE-IF  185
_XP_025892242.1_  162  ---------------------NTAE-L-------------RICRVNKN---------CGSVKG-GDE-IF  185
_XP_005507389.1_  162  ---------------------NTAE-L-------------RICRVNKN---------CGSVKG-GDE-IF  185
_XP_032911247.1_  162  ---------------------NTAE-L-------------RICRVNKN---------CGSVKG-GDE-IF  185
_XP_031959657.1_  162  ---------------------NTAE-L-------------RICRVNKN---------CGSVKG-GDE-IF  185
_XP_041905291.1_  162  ---------------------NTAE-L-------------RICRVNKN---------CGSVKG-GDE-IF  185
_XP_051648836.1_  162  ---------------------NTAE-L-------------RICRVNKN---------CGSVKG-GDE-IF  185
_XP_054239488.1_  162  ---------------------NTAE-L-------------RICRVNKN---------CGSVKG-GDE-IF  185
_XP_039914164.1_  162  ---------------------NTAE-L-------------RICRVNKN---------CGSVKG-GDE-IF  185
_XP_005422689.1_  162  ---------------------NTAE-L-------------RICRVNKN---------CGSVKG-GDE-IF  185
_KAF2987757.1_    162  ---------------------NTAE-L-------------RICRVNKN---------CGSVKG-GDE-IF  185
_XP_038665087.1_  173  ---------------------NTAE-L-------------KICRVNKN---------TGTVKG-GDE-IF  196
_GCB59945.1_      173  ---------------------NTAE-L-------------KICRVNKN---------TGTVKG-GDE-IF  196
_XP_041065616.1_  169  ---------------------NTAE-L-------------KICRVNKN---------TGTVKG-GDE-IF  192
_XP_007890564.1_  173  ---------------------NTAE-L-------------KICRINKN---------TGSVKG-GDE-IF  196
_XP_051868609.1_  170  ----------------------------------------KICRVNKN---------TGTVKG-GDE-IF  188
_XP_032881051.1_  172  ---------------------NTAE-L-------------KICRVNKN---------TGTVKG-GDE-VF  195
_XP_048456938.1_   82  ---------------------NTAE-L-------------KICRVNKN---------TGTVKG-GDE-IF  105
_KAJ1155552.1_    174  ----------------------TAE-L-------------RICRVNKN---------CGGVRG-QEE-IF  196
_KAH0622877.1_     42  ---------------------NSAE-L-------------RICRVNKN---------CGSVKG-GDE-IF   65
_CAI5653329.1_    173  ---------------------NTAE-L-------------RICRVNRN---------SGSVRG-GDE-IF  196
_CAI5653328.1_    173  ---------------------NTAE-L-------------RICRVNRN---------SGSVRG-GDE-IF  196
_NXS54806.1_      180  ---------------------NTAE-L-------------RICRVNKN---------CGSVKG-GDE-IF  203
_XP_009504395.1_  170  ---------------------NTAE-L-------------RICRVNKN---------CGSVKG-GDE-IF  193
_NXJ41381.1_      173  ---------------------NTAE-L-------------RICRVNKN---------CGSVKG-GDE-IF  196
_NXT24719.1_      173  ---------------------NTAE-L-------------RICRVNKN---------CGSVKG-GDE-IF  196
_NXK23502.1_      173  ---------------------NTAE-L-------------RICRVNKN---------CGSVKG-GDE-IF  196
_NXK03070.1_      173  ---------------------NTAE-L-------------RICRVNKN---------CGSVKG-GDE-IF  196
_XP_037260944.1_  173  ---------------------NTAE-L-------------RICRVNKN---------CGSVKG-GDE-IF  196
```

```
_XP_040467369.1_  173  ---------------------NTAE-L-------------RICRVNKN---------CGSVKG-GDE-IF  196
_NXY71491.1_      173  ---------------------NTAE-L-------------RICRVNKN---------CGSVKG-GDE-IF  196
_NXN54417.1_      173  ---------------------NTAE-L-------------RICRVNKN---------CGSVKG-GDE-IF  196
_NXX19490.1_      173  ---------------------NTAE-L-------------RICRVNKN---------CGSVKG-GDE-IF  196
_NXL47199.1_      173  ---------------------NTAE-L-------------RICRVNKN---------CGSVKG-GDE-IF  196
_XP_050749583.1_  173  ---------------------NTAE-L-------------RICRVNKN---------CGSVKG-GDE-IF  196
_NXD76242.1_      173  ---------------------NTAE-L-------------RICRVNKN---------CGSVKG-GDE-IF  196
_KFZ56827.1_      173  ---------------------NTAE-L-------------RICRVNKN---------CGSVKG-GDE-IF  196
_NXH67691.1_      173  ---------------------NTAE-L-------------RICRVNKN---------CGSVKG-GDE-IF  196
_NXS45828.1_      173  ---------------------NTAE-L-------------RICRVNKN---------CGSVKG-GDE-IF  196
_KFP52034.1_      173  ---------------------NTAE-L-------------RICRVNKN---------CGSVKG-GDE-IF  196
_NWH44485.1_      173  ---------------------NTAE-L-------------RICRVNKN---------CGSVKG-GDE-IF  196
_NWW85416.1_      173  ---------------------NTAE-L-------------RICRVNKN---------CGSVKG-GDE-IF  196
_NWU66320.1_      173  ---------------------NTAE-L-------------RICRVNKN---------CGSVKG-GDE-IF  196
_XP_025919906.1_  173  ---------------------NTAE-L-------------RICRVNKN---------CGSVKG-GDE-IF  196
_KAF1483947.1_    173  ---------------------NTAE-L-------------RICRVNKN---------CGSVKG-GDE-IF  196
_NXQ87688.1_      173  ---------------------NTAE-L-------------RICRVNKN---------CGSVKG-GDE-IF  196
_NXX07562.1_      173  ---------------------NTAE-L-------------RICRVNKN---------CGSVKG-GDE-IF  196
_XP_009280788.2_  173  ---------------------NTAE-L-------------RICRVNKN---------CGSVKG-GDE-IF  196
_XP_014821857.1_  173  ---------------------NTAE-L-------------RICRVNKN---------CGSVKG-GDE-IF  196
_NWU51443.1_      173  ---------------------NTAE-L-------------RICRVNKN---------CGSVKG-GDE-IF  196
_NXV35521.1_      173  ---------------------NTAE-L-------------RICRVNKN---------CGSVKG-GDE-IF  196
_KFV80780.1_      173  ---------------------NTAE-L-------------RICRVNKN---------CGSVKG-GDE-IF  196
_XP_032039522.1_  173  ---------------------NTAE-L-------------RICRVNKN---------CGSVKG-GDE-IF  196
_NWT43027.1_      173  ---------------------NTAE-L-------------RICRVNKN---------CGSVKG-GDE-IF  196
_KAF1651581.1_    173  ---------------------NTAE-L-------------RICRVNKN---------CGSVKG-GDE-IF  196
_NXU26374.1_      173  ---------------------NTAE-L-------------RICRVNKN---------CGSVKG-GDE-IF  196
_NWX46229.1_      173  ---------------------NTAE-L-------------RICRVNKN---------CGSVKG-GDE-IF  196
_PKU40340.1_      173  ---------------------NTAE-L-------------RICRVNKN---------CGSVKG-GDE-IF  196
_XP_027309078.1_  173  ---------------------NTAE-L-------------RICRVNKN---------CGSVKG-GDE-IF  196
_KAF1573683.1_    173  ---------------------NTAE-L-------------RICRVNKN---------CGSVKG-GDE-IF  196
_KAF1578928.1_    173  ---------------------NTAE-L-------------RICRVNKN---------CGSVKG-GDE-IF  196
_NXN47104.1_      173  ---------------------NTAE-L-------------RICRVNKN---------CGSVKG-GDE-IF  196
_NXV20393.1_      173  ---------------------NTAE-L-------------RICRVNKN---------CGSVKG-GDE-IF  196
_KAF1551267.1_    173  ---------------------NTAE-L-------------RICRVNKN---------CGSVKG-GDE-IF  196
_KAF1406210.1_    173  ---------------------NTAE-L-------------RICRVNKN---------CGSVKG-GDE-IF  196
_NXV43201.1_      173  ---------------------NTAE-L-------------RICRVNKN---------CGSVKG-GDE-IF  196
_NWQ98669.1_      173  ---------------------NTAE-L-------------RICRVNKN---------CGSVKG-GDE-IF  196
_XP_052639968.1_  173  ---------------------NTAE-L-------------RICRVNKN---------CGSVKG-GDE-IF  196
```

```
_XP_010573143.1_  173  ---------------------NTAE-L-------------RICRVNKN---------CGSVKG-GDE-IF  196
_KAF1479604.1_    173  ---------------------NTAE-L-------------RICRVNKN---------CGSVKG-GDE-IF  196
_NXG89109.1_      173  ---------------------NTAE-L-------------RICRVNKN---------CGSVKG-GDE-IF  196
_KFQ89757.1_      173  ---------------------NTAE-L-------------RICRVNKN---------CGSVKG-GEE-IF  196
_NWZ21787.1_      173  ---------------------NTAE-L-------------RICRVNKN---------CGSVKG-GDE-IF  196
_NXI74048.1_      173  ---------------------NTAE-L-------------RICRVNKN---------CGSVKG-GDE-IF  196
_NWX76247.1_      173  ---------------------NTAE-L-------------RICRVNKN---------CGSVKG-GDE-IF  196
_NXF29788.1_      173  ---------------------NTAE-L-------------RICRVNKN---------CGSVKG-GDE-IF  196
_KAF1444142.1_    173  ---------------------NTAE-L-------------RICRVNKN---------CGSVKG-GDE-IF  196
_KFV20215.1_      173  ---------------------NTAE-L-------------RICRVNKN---------CGSVKG-GDE-IF  196
_NXI44657.1_      173  ---------------------NTAE-L-------------RICRVNKN---------CGSVKG-GDE-IF  196
_NXJ92321.1_      173  ---------------------NTAE-L-------------RICRVNKN---------CGSVKG-GDE-IF  196
_NWZ49902.1_      173  ---------------------NTAE-L-------------RICRVNKN---------CGSVKG-GDE-IF  196
_NWY59444.1_      173  ---------------------NTAE-L-------------RICRVNKN---------CGSVKG-GDE-IF  196
_KFW64019.1_      173  ---------------------NTAE-L-------------RICRVNKN---------CGSVKG-GDE-IF  196
_NXJ55634.1_      173  ---------------------NTAE-L-------------RICRVNKN---------CGSVKG-GDE-IF  196
_KAF1513372.1_    173  ---------------------NTAE-L-------------RICRVNKN---------CGSVKG-GDE-IF  196
_NXW14598.1_      173  ---------------------NTAE-L-------------RICRVNKN---------CGSVKG-GDE-IF  196
_NXC74247.1_      173  ---------------------NTAE-L-------------RICRVNKN---------CGSVKG-GDE-IF  196
_NXT57254.1_      173  ---------------------NTAE-L-------------RICRVNKN---------CGSVKG-GDE-IF  196
_XP_047916679.1_  173  ---------------------NTAE-L-------------RICRVNKN---------CGSVKG-GDE-IF  196
_XP_029879190.1_  173  ---------------------NTAE-L-------------RICRVNKN---------CGSVKG-GDE-IF  196
_NXH09241.1_      173  ---------------------NTAE-L-------------RICRVNKN---------CGSVKG-GDE-IF  196
_XP_040407374.1_  173  ---------------------NTAE-L-------------RICRVNKN---------CGSVKG-GDE-IF  196
_KFW76037.1_      173  ---------------------NTAE-L-------------RICRVNKN---------CGSVKG-GDE-IF  196
_KFP65945.1_      173  ---------------------NTAE-L-------------RICRVNKN---------CGSVKG-GDE-IF  196
_NXX55682.1_      173  ---------------------NTAE-L-------------RICRVNKN---------CGSVKG-GDE-IF  196
_XP_035178333.1_  173  ---------------------NTAE-L-------------RICRVNKN---------CGSVKG-GDE-IF  196
_NXE47317.1_      173  ---------------------NTAE-L-------------RICRVNKN---------CGSVKG-GDE-IF  196
_XP_035408877.1_  173  ---------------------NTAE-L-------------RICRVNKN---------CGSVKG-GDE-IF  196
_KGL94141.1_      173  ---------------------NTAE-L-------------RICRVNKN---------CGSVKG-GDE-IF  196
_NXN68403.1_      173  ---------------------NTAE-L-------------RICRVNKN---------CGSVKG-GDE-IF  196
_NXW28331.1_      173  ---------------------NTAE-L-------------RICRVNKN---------CGSVKG-GDE-IF  196
_XP_049657509.1_  173  ---------------------NTAE-L-------------RICRVNKN---------CGSVKG-GDE-IF  196
_XP_049657511.1_  173  ---------------------NTAE-L-------------RICRVNKN---------CGSVKG-GDE-IF  196
_NXG30098.1_      173  ---------------------NTAE-L-------------RICRVNKN---------CGSVKG-GDE-IF  196
_NXV86645.1_      173  ---------------------NTAE-L-------------RICRVNKN---------CGSVKG-GDE-IF  196
_NXX83200.1_      173  ---------------------NTAE-L-------------RICRVNKN---------CGSVKG-GDE-IF  196
_NXI59898.1_      173  ---------------------NTAE-L-------------RICRVNKN---------CGSVKG-GDE-IF  196
```

```
_NWU89521.1_      173  ---------------------NTAE-L-------------RICRVNKN---------CGSVKG-GDE-IF  196
_NXK45490.1_      173  ---------------------NTAE-L-------------RICRVNKN---------CGSVKG-GDE-IF  196
_NWR55599.1_      173  ---------------------NTAE-L-------------RICRVNKN---------CGSVKG-GDE-IF  196
_NXL37824.1_      173  ---------------------NTAE-L-------------RICRVNKN---------CGSVKG-GDE-IF  196
_NWI33553.1_      173  ---------------------NTAE-L-------------RICRVNKN---------CGSVKG-GDE-IF  196
_KFQ74517.1_      173  ---------------------NTAE-L-------------RICRVNKN---------CGSVKG-GDE-IF  196
_NXV82662.1_      173  ---------------------NTAE-L-------------RICRVNKN---------CGSVKG-GDE-IF  196
_NWJ10087.1_      173  ---------------------NTAE-L-------------RICRVNKN---------CGSVKG-GDE-IF  196
_NXN24483.1_      173  ---------------------NTAE-L-------------RICRVNKN---------CGSVKG-GDE-IF  196
_NWR80208.1_      173  ---------------------NTAE-L-------------RICRVNKN---------CGSVKG-GDE-IF  196
_NXT37538.1_      173  ---------------------NTAE-L-------------RICRVNKN---------CGSVKG-GDE-IF  196
_XP_032844994.2_  173  ---------------------NTAE-L-------------RICRVNKN---------CGSVKG-GDE-IF  196
_NXW47649.1_      173  ---------------------NTAE-L-------------RICRVNKN---------CGSVKG-GDE-IF  196
_NXA40398.1_      173  ---------------------NTAE-L-------------RICRVNKN---------CGSVKG-GDE-IF  196
_NXW64893.1_      173  ---------------------NTAE-L-------------RICRVNKN---------CGSVKG-GDE-IF  196
_NXX89165.1_      173  ---------------------NTAE-L-------------RICRVNKN---------CGSVKG-GDE-IF  196
_NXQ96911.1_      173  ---------------------NTAE-L-------------RICRVNKN---------CGSVKG-GDE-IF  196
_KFP18273.1_      173  ---------------------NTAE-L-------------RICRVNKN---------CGSVKG-GDE-IF  196
_NXC40882.1_      173  ---------------------NTAE-L-------------RICRVNKN---------CGSVKG-GDE-IF  196
_NXE10305.1_      173  ---------------------NTAE-L-------------RICRVNKN---------CGSVKG-GDE-IF  196
_KFP88087.1_      173  ---------------------NTAE-L-------------RICRVNKN---------CGSVKG-GDE-IF  196
_NWW47871.1_      173  ---------------------NTAE-L-------------RICRVNKN---------CGSVKG-GDE-IF  196
_NXJ61595.1_      173  ---------------------NTAE-L-------------RICRVNKN---------CGSVKG-GDE-IF  196
_NXD13814.1_      173  ---------------------NTAE-L-------------RICRVNKN---------CGSVKG-GDE-IF  196
_KFR16961.1_      173  ---------------------NTAE-L-------------RICRVNKN---------CGSVKG-GDE-IF  196
_NWX24312.1_      173  ---------------------NTAE-L-------------RICRVNKN---------CGSVKG-GDE-IF  196
_NXA28267.1_      173  ---------------------NTAE-L-------------RICRVNKN---------CGSVKG-GDE-IF  196
_NXL66270.1_      173  ---------------------NTAE-L-------------RICRVNKN---------CGSVKG-GDE-IF  196
_NXA49976.1_      173  ---------------------NTAE-L-------------RICRVNKN---------CGSVKG-GDE-IF  196
_NXT77624.1_      173  ---------------------NTAE-L-------------RICRVNKN---------CGSVKG-GDE-IF  196
_KFQ92336.1_      173  ---------------------NTAE-L-------------RICRVNKN---------CGSVKG-GDE-IF  196
_NXE25013.1_      173  ---------------------NTAE-L-------------RICRVNKN---------CGSVKG-GDE-IF  196
_NXE92240.1_      173  ---------------------NTAE-L-------------RICRVNKN---------CGSVKG-GDE-IF  196
_NWH80943.1_      173  ---------------------NTAE-L-------------RICRVNKN---------CGSVKG-GDE-IF  196
_NWI19058.1_      173  ---------------------NTAE-L-------------RICRVNKN---------CGSVKG-GDE-IF  196
_NXG80610.1_      173  ---------------------NTAE-L-------------RICRVNKN---------CGSVKG-GDE-IF  196
_NWR01486.1_      173  ---------------------NTAE-L-------------RICRVNKN---------CGSVKG-GDE-IF  196
_NXK81337.1_      173  ---------------------NTAE-L-------------RICRVNKN---------CGSVKG-GDE-IF  196
_NXS90563.1_      173  ---------------------NTAE-L-------------RICRVNKN---------CGSVKG-GDE-IF  196
```

```
_NXC17430.1_       173  ---------------------NTAE-L-------------RICRVNKN---------CGSVKG-GDE-IF  196
_NXE77245.1_       173  ---------------------NTAE-L-------------RICRVNKN---------CGSVKG-GDE-IF  196
_KFQ29788.1_       174  ---------------------NTAE-L-------------RICRVNKN---------CGSVKG-GDE-IF  197
_KFO89327.1_       173  ---------------------NTAE-L-------------RICRVNKN---------CGSVKG-GDE-IF  196
_NXD64381.1_       173  ---------------------NTAE-L-------------RICRVNKN---------CGSVKG-GDE-IF  196
_NXY45081.1_       173  ---------------------NTAE-L-------------RICRVNKN---------CGSVKG-GDE-IF  196
_KGL72871.1_       173  ---------------------NTAE-L-------------RICRVNKN---------CGSVKG-GDE-IF  196
_XP_053919504.1_   173  ---------------------NTAE-L-------------RICRVNKN---------CGSVKG-GDE-IF  196
_NXL85459.1_       173  ---------------------NTAE-L-------------RICRVNKN---------CGSVKG-GDE-IF  196
_NXO02254.1_       173  ---------------------NTAE-L-------------RICRVNKN---------CGSVKG-GDE-IF  196
_NXJ76034.1_       173  ---------------------NTAE-L-------------RICRVNKN---------CGSVKG-GDE-IF  196
_NXM93491.1_       173  ---------------------NTAE-L-------------RICRVNKN---------CGSVKG-GDE-IF  196
_XP_030345800.1_   173  ---------------------NTAE-L-------------RICRVNKN---------CGSVKG-GDE-IF  196
_XP_030302544.1_   173  ---------------------NTAE-L-------------RICRVNKN---------CGSVKG-GDE-IF  196
_XP_030345801.1_   173  ---------------------NTAE-L-------------RICRVNKN---------CGSVKG-GDE-IF  196
_KQK77724.1_       173  ---------------------NTAE-L-------------RICRVNKN---------CGSVKG-GDE-IF  196
_NXP09913.1_       173  ---------------------NTAE-L-------------RICRVNKN---------CGSVKG-GDE-IF  196
_NXR33018.1_       173  ---------------------NTAE-L-------------RICRVNKN---------CGSVKG-GDE-IF  196
_NXM76881.1_       173  ---------------------NTAE-L-------------RICRVNKN---------CGSVKG-GDE-IF  196
_NXS27423.1_       173  ---------------------NTAE-L-------------RICRVNKN---------CGSVKG-GDE-IF  196
_NXM55821.1_       173  ---------------------NTAE-L-------------RICRVNKN---------CGSVKG-GDE-IF  196
_XP_054678500.1_   173  ---------------------NTAE-L-------------RICRVNKN---------CGSVKG-GDE-IF  196
_XP_015712248.1_   173  ---------------------NTAE-L-------------RICRVNKN---------CGSVKG-GDE-IF  196
_NWX92058.1_       173  ---------------------NTAE-L-------------RICRVNKN---------CGSVKG-GDE-IF  196
_XP_048793646.1_   173  ---------------------NTAE-L-------------RICRVNKN---------CGSVKG-GDE-IF  196
_NXF04247.1_       173  ---------------------NTAE-L-------------RICRVNKN---------CGSVKG-GDE-IF  196
_XP_042683331.1_   173  ---------------------NTAE-L-------------RICRVNKN---------CGSVKG-GDE-IF  196
_NWV22147.1_       173  ---------------------NTAE-L-------------RICRVNKN---------CGSVKG-GDE-IF  196
_NXA04361.1_       173  ---------------------NTAE-L-------------RICRVNKN---------CGSVKG-GDE-IF  196
_XP_010116198.1_   179  ---------------------NTAE-L-------------RICRVNKN---------CGSVKG-GDE-IF  202
_NXG59741.1_       173  ---------------------NTAE-L-------------RICRVNKN---------CGSVKG-GDE-IF  196
_XP_042735326.1_   173  ---------------------NTAE-L-------------RICRVNKN---------CGSVKG-GDE-IF  196
_NXM29886.1_       173  ---------------------NTAE-L-------------RICRVNKN---------CGSVKG-GDE-IF  196
_NP_001161198.2_   173  ---------------------NTAE-L-------------RICRVNKN---------CGSVKG-GDE-IF  196
_XP_031451029.1_   173  ---------------------NTAE-L-------------RICRVNKN---------CGSVKG-GDE-IF  196
_NXR49262.1_       173  ---------------------NTAE-L-------------RICRVNKN---------CGSVKG-GDE-IF  196
_XP_052555416.1_   173  ---------------------NTAE-L-------------RICRVNKN---------CGSVKG-GDE-IF  196
_NWZ67085.1_       173  ---------------------NTAE-L-------------RICRVNKN---------CGSVKG-GDE-IF  196
_P16236.2_         173  ---------------------NTAE-L-------------RICRVNKN---------CGSVKG-GDE-IF  196
```

```
_XP_050566010.1_  173  ---------------------NTAE-L-------------RICRVNKN---------CGSVKG-GDE-IF  196
_NWS67030.1_      173  ---------------------NTAE-L-------------RICRVNKN---------CGSVKG-GDE-IF  196
_XP_005143850.3_  173  ---------------------NTAE-L-------------RICRVNKN---------CGSVKG-GDE-IF  196
_XP_050566011.1_  173  ---------------------NTAE-L-------------RICRVNKN---------CGSVKG-GDE-IF  196
_XP_050566009.1_  173  ---------------------NTAE-L-------------RICRVNKN---------CGSVKG-GDE-IF  196
_NWR30963.1_      173  ---------------------NTAE-L-------------RICRVNKN---------CGSVKG-GDE-IF  196
_NWS17896.1_      173  ---------------------NTAE-L-------------RICRVNKN---------CGSVKG-GDE-IF  196
_XP_021248574.1_  173  ---------------------NTAE-L-------------RICRVNKN---------CGSVKG-GDE-IF  196
_AAA48721.1_      173  ---------------------NTAE-L-------------RICRVNKN---------CGSVKG-GDE-IF  196
_NWI54208.1_      173  ---------------------NTAE-L-------------RICRVNKN---------CGSVKG-GDE-IF  196
_XP_027509532.1_  173  ---------------------NTAE-L-------------RICRVNKN---------CGSVKG-GDE-IF  196
_NXP71979.1_      173  ---------------------NTAE-L-------------RICRVNKN---------CGSVKG-GDE-IF  196
_NXX25352.1_      173  ---------------------NTAE-L-------------RICRVNKN---------CGSVKG-GDE-IF  196
_NXI18618.1_      173  ---------------------NTAE-L-------------RICRVNKN---------CGSVKG-GDE-IF  196
_NXS09623.1_      173  ---------------------NTAE-L-------------RICRVNKN---------CGSVKG-GDE-IF  196
_NXU77980.1_      173  ---------------------NTAE-L-------------RICRVNKN---------CGSVKG-GDE-IF  196
_XP_027509533.1_  173  ---------------------NTAE-L-------------RICRVNKN---------CGSVKG-GDE-IF  196
_NWV71036.1_      173  ---------------------NTAE-L-------------RICRVNKN---------CGSVKG-GDE-IF  196
_NXF89482.1_      173  ---------------------NTAE-L-------------RICRVNKN---------CGSVKG-GDE-IF  196
_NWY39470.1_      173  ---------------------NTAE-L-------------RICRVNKN---------CGSVKG-GDE-IF  196
_NXT59960.1_      173  ---------------------NTAE-L-------------RICRVNKN---------CGSVKG-GDE-IF  196
_NWU74340.1_      173  ---------------------NTAE-L-------------RICRVNKN---------CGSVKG-GDE-IF  196
_NXB84481.1_      173  ---------------------NTAE-L-------------RICRVNKN---------CGSVKG-GDE-IF  196
_NWV73885.1_      173  ---------------------NTAE-L-------------RICRVNKN---------CGSVKG-GDE-IF  196
_OPJ58894.1_      173  ---------------------NTAE-L-------------RICRVNKN---------CGSVKG-GDE-IF  196
_XP_048152104.1_  173  ---------------------NTAE-L-------------RICRVNKN---------CGSVKG-GDE-IF  196
_XP_054036037.1_  173  ---------------------NTAE-L-------------RICRVNKN---------CGSVKG-GDE-IF  196
_NXR18149.1_      173  ---------------------NTAE-L-------------RICRVNKN---------CGSVKG-GDE-IF  196
_NXP97533.1_      173  ---------------------NTAE-L-------------RICRVNKN---------CGSVKG-GDE-IF  196
_NXJ05495.1_      173  ---------------------NTAE-L-------------RICRVNKN---------CGSVKG-GDE-IF  196
_XP_032538932.1_  173  ---------------------NTAE-L-------------RICRVNKN---------CGSVKG-GDE-IF  196
_NWI96387.1_      173  ---------------------NTAE-L-------------RICRVNKN---------CGSVKG-GDE-IF  196
_NXR65995.1_      173  ---------------------NTAE-L-------------RICRVNKN---------CGSVKG-GDE-IF  196
_NXK42398.1_      173  ---------------------NTAE-L-------------RICRVNKN---------CGSVKG-GDE-IF  196
_XP_032538931.1_  173  ---------------------NTAE-L-------------RICRVNKN---------CGSVKG-GDE-IF  196
_NXY33181.1_      173  ---------------------NTAE-L-------------RICRVNKN---------CGSVKG-GDE-IF  196
_XP_050171009.1_  173  ---------------------NTAE-L-------------RICRVNKN---------CGSVKG-GDE-IF  196
_NXO50939.1_      173  ---------------------NTAE-L-------------RICRVNKN---------CGSVKG-GDE-IF  196
_NWZ32937.1_      173  ---------------------NTAE-L-------------RICRVNKN---------CGSVKG-GDE-IF  196
```

```
_NXP01828.1_       173  ---------------------NTAE-L-------------RICRVNKN---------CGSVKG-GDE-IF  196
_NXL00458.1_       173  ---------------------NTAE-L-------------RICRVNKN---------CGSVKG-GDE-IF  196
_XP_010401310.4_   173  ---------------------NTAE-L-------------RICRVNKN---------CGSVKG-GDE-IF  196
_NXH62149.1_       173  ---------------------NTAE-L-------------RICRVNKN---------CGSVKG-GDE-IF  196
_NXG43824.1_       173  ---------------------NTAE-L-------------RICRVNKN---------CGSVKG-GDE-IF  196
_XP_017677337.1_   173  ---------------------NTAE-L-------------RICRVNKN---------CGSVKG-GDE-IF  196
_NXU09156.1_       173  ---------------------NTAE-L-------------RICRVNKN---------CGSVKG-GDE-IF  196
_NXB38645.1_       173  ---------------------NTAE-L-------------RICRVNKN---------CGSVKG-GDE-IF  196
_XP_027594504.1_   173  ---------------------NTAE-L-------------RICRVNKN---------CGSVKG-GDE-IF  196
_NXM46269.1_       173  ---------------------NTAE-L-------------RICRVNKN---------CGSVKG-GDE-IF  196
_NWS79257.1_       173  ---------------------NTAE-L-------------RICRVNKN---------CGSVKG-GDE-IF  196
_NXB04839.1_       173  ---------------------NTAE-L-------------RICRVNKN---------CGSVKG-GDE-IF  196
_NXC62865.1_       173  ---------------------NTAE-L-------------RICRVNKN---------CGSVKG-GDE-IF  196
_NXN18277.1_       173  ---------------------NTAE-L-------------RICRVNKN---------CGSVKG-GDE-IF  196
_XP_027760039.1_   173  ---------------------NTAE-L-------------RICRVNKN---------CGSVKG-GDE-IF  196
_NXI90311.1_       173  ---------------------NTAE-L-------------RICRVNKN---------CGSVKG-GDE-IF  196
_NWY17003.1_       173  ---------------------NTAE-L-------------RICRVNKN---------CGSVKG-GDE-IF  196
_NXH97099.1_       173  ---------------------NTAE-L-------------RICRVNKN---------CGSVKG-GDE-IF  196
_NWS97308.1_       173  ---------------------NTAE-L-------------RICRVNKN---------CGSVKG-GDE-IF  196
_NWU45160.1_       173  ---------------------NTAE-L-------------RICRVNKN---------CGSVKG-GDE-IF  196
_NXQ43004.1_       173  ---------------------NTAE-L-------------RICRVNKN---------CGSVKG-GDE-IF  196
_XP_027549142.1_   173  ---------------------NTAE-L-------------RICRVNKN---------CGSVKG-GDE-IF  196
_XP_017933082.2_   173  ---------------------NTAE-L-------------RICRVNKN---------CGSVKG-GDE-IF  196
_NWV96326.1_       173  ---------------------NTAE-L-------------RICRVNKN---------CGSVKG-GDE-IF  196
_NXP57783.1_       173  ---------------------NTAE-L-------------RICRVNKN---------CGSVKG-GDE-IF  196
_NXY57955.1_       173  ---------------------NTAE-L-------------RICRVNKN---------CGSVKG-GDE-IF  196
_AAA99433.1_       173  ---------------------NTAE-L-------------RICRVNKN---------CGSVKG-GDE-IF  196
_NXY21218.1_       173  ---------------------NTAE-L-------------RICRVNKN---------CGSVKG-GDE-IF  196
_NXX50824.1_       173  ---------------------NTAE-L-------------RICRVNKN---------CGSVKG-GDE-IF  196
_XP_030124623.3_   173  ---------------------NTAE-L-------------RICRVNKN---------CGSVKG-GDE-IF  196
_NXA92235.1_       173  ---------------------NTAE-L-------------RICRVNKN---------CGSVKG-GDE-IF  196
_NWI05522.1_       173  ---------------------NTAE-L-------------RICRVNKN---------CGSVKG-GDE-IF  196
_NWX29009.1_       173  ---------------------NTAE-L-------------RICRVNKN---------CGSVKG-GDE-IF  196
_XP_027549144.1_   173  ---------------------NTAE-L-------------RICRVNKN---------CGSVKG-GDE-IF  196
_NWW35420.1_       173  ---------------------NTAE-L-------------RICRVNKN---------CGSVKG-GDE-IF  196
_NWV07969.1_       173  ---------------------NTAE-L-------------RICRVNKN---------CGSVKG-GDE-IF  196
_NXH42214.1_       173  ---------------------NTAE-L-------------RICRVNKN---------CGSVKG-GDE-IF  196
_NXQ18773.1_       173  ---------------------NTAE-L-------------RICRVNKN---------CGSVKG-GDE-IF  196
_NWX10672.1_       173  ---------------------NTAE-L-------------RICRVNKN---------CGSVKG-GDE-IF  196
```

```
_NXU50687.1_       173  ---------------------NTAE-L-------------RICRVNKN---------CGSVKG-GDE-IF  196
_KFU84784.1_       173  ---------------------NTAE-L-------------RICRVNKN---------CGSVKG-GDE-IF  196
_NXQ52438.1_       173  ---------------------NTAE-L-------------RICRVNKN---------CGSVKG-GDE-IF  196
_NWW24536.1_       173  ---------------------NTAE-L-------------RICRVNKN---------CGSVKG-GDE-IF  196
_NXF73449.1_       173  ---------------------NTAE-L-------------RICRVNKN---------CGSVKG-GDE-IF  196
_XP_050827906.1_   173  ---------------------NTAE-L-------------RICRVNKN---------CGSVKG-GDE-IF  196
_NWX65205.1_       173  ---------------------NTAE-L-------------RICRVNKN---------CGSVKG-GDE-IF  196
_KFW81749.1_       173  ---------------------NTAE-L-------------RICRVNKN---------CGSVKG-GDE-IF  196
_XP_014750593.1_   173  ---------------------NTAE-L-------------RICRVNKN---------CGSVKG-GDE-IF  196
_NXJ17066.1_       173  ---------------------NTAE-L-------------RICRVNKN---------CGSVKG-GDE-IF  196
_NWW67019.1_       173  ---------------------NTAE-L-------------RICRVNKN---------CGSVKG-GDE-IF  196
_NXH03360.1_       173  ---------------------NTAE-L-------------RICRVNKN---------CGSVKG-GDE-IF  196
_XP_037987525.1_   173  ---------------------NTAE-L-------------RICRVNKN---------CGSVKG-GDE-IF  196
_NXL74522.1_       173  ---------------------NTAE-L-------------RICRVNKN---------CGSVKG-GDE-IF  196
_NWQ60824.1_       173  ---------------------NTAE-L-------------RICRVNKN---------CGSVKG-GDE-IF  196
_NXR70971.1_       173  ---------------------NTAE-L-------------RICRVNKN---------CGSVKG-GDE-IF  196
_XP_051468947.1_   173  ---------------------NTAE-L-------------RICRVNKN---------CGSVKG-GDE-IF  196
_NXB50199.1_       173  ---------------------NTAE-L-------------RICRVNKN---------CGSVKG-GDE-IF  196
_NXP18380.1_       173  ---------------------NTAE-L-------------RICRVNKN---------CGSVKG-GDE-IF  196
_NXI84319.1_       173  ---------------------NTAE-L-------------RICRVNKN---------CGSVKG-GDE-IF  196
_NWH33757.1_       173  ---------------------NTAE-L-------------RICRVNKN---------CGSVKG-GDE-IF  196
_NWR99781.1_       173  ---------------------NTAE-L-------------RICRVNKN---------CGSVKG-GDE-IF  196
_NXW94404.1_       173  ---------------------NTAE-L-------------RICRVNKN---------CGSVKG-GDE-IF  196
_CAA27220.1_       173  ---------------------NTAE-L-------------RICRVNKN---------CGSVKG-GDE-IF  196
_XP_015477679.1_   173  ---------------------NTAE-L-------------RICRVNKN---------CGSVKG-GDE-IF  196
_NWS32597.1_       173  ---------------------NTAE-L-------------RICRVNKN---------CGSVKG-GDE-IF  196
_NXG22126.1_       173  ---------------------NTAE-L-------------RICRVNKN---------CGSVKG-GDE-IF  196
_NWY97127.1_       173  ---------------------NTAE-L-------------RICRVNKN---------CGSVKG-GDE-IF  196
_NXW80148.1_       173  ---------------------NTAE-L-------------RICRVNKN---------CGSVKG-GDE-IF  196
_NXC27367.1_       173  ---------------------NTAE-L-------------RICRVNKN---------CGSVKG-GDE-IF  196
_NWH83726.1_       173  ---------------------NTAE-L-------------RICRVNKN---------CGSVKG-GDE-IF  196
_NXA73512.1_       173  ---------------------NTAE-L-------------RICRVNKN---------CGSVKG-GDE-IF  196
_NWZ83230.1_       173  ---------------------NTAE-L-------------RICRVNKN---------CGSVKG-GDE-IF  196
_XP_005525572.2_   173  ---------------------NTAE-L-------------RICRVNKN---------CGSVKG-GDE-IF  196
_NXD20033.1_       173  ---------------------NTAE-L-------------RICRVNKN---------CGSVKG-GDE-IF  196
_NXE37177.1_       173  ---------------------NTAE-L-------------RICRVNKN---------CGSVKG-GDE-IF  196
_NWV44369.1_       173  ---------------------NTAE-L-------------RICRVNKN---------CGSVKG-GDE-IF  196
_NXC81400.1_       173  ---------------------NTAE-L-------------RICRVNKN---------CGSVKG-GDE-IF  196
_NXA67345.1_       173  ---------------------NTAE-L-------------RICRVNKN---------CGSVKG-GDE-IF  196
```

```
_NXB55155.1_      173  ---------------------NTAE-L-------------RICRVNKN---------CGSVKG-GDE-IF  196
_NXN81229.1_      173  ---------------------NTAE-L-------------RICRVNKN---------CGSVKG-GDE-IF  196
_NXM15601.1_      173  ---------------------NTAE-L-------------RICRVNKN---------CGSVKG-GDE-IF  196
_XP_054484850.1_  173  ---------------------NTAE-L-------------RICRVNKN---------CGSVKG-GDE-IF  196
_NWU02685.1_      173  ---------------------NTAE-L-------------RICRVNKN---------CGSVKG-GDE-IF  196
_XP_041273165.1_  173  ---------------------NTAE-L-------------RICRVNKN---------CGSVKG-GDE-IF  196
_NWW80397.1_      173  ---------------------NTAE-L-------------RICRVNKN---------CGSVKG-GDE-IF  196
_XP_036236540.1_  173  ---------------------NTAE-L-------------RICRVNKN---------CGSVKG-GDE-IF  196
_NWV50957.1_      173  ---------------------NTAE-L-------------RICRVNKN---------CGSVKG-GDE-IF  196
_NXT97287.1_      173  ---------------------NTAE-L-------------RICRVNKN---------CGSVKG-GDE-IF  196
_XP_023778876.1_  173  ---------------------NTAE-L-------------RICRVNKN---------CGSVKG-GDE-IF  196
_NXD42381.1_      173  ---------------------NTAE-L-------------RICRVNKN---------CGSVKG-GDE-IF  196
_NXK69295.1_      173  ---------------------NTAE-L-------------RICRVNKN---------CGSVKG-GDE-IF  196
_XP_049657510.1_  173  ---------------------NTAE-L-------------RICRVNKN---------CGSVKG-GDE-IF  196
_NXV62539.1_      173  ---------------------NTAE-L-------------RICRVNKN---------CGSVKG-GDE-IF  196
_NXB80231.1_      173  ---------------------NTAE-L-------------RICRVNKN---------CGSVKG-GDE-IF  196
_NWU15026.1_      173  ---------------------NTAE-L-------------RICRVNKN---------CGSVKG-GDE-IF  196
_NXL26104.1_      173  ---------------------NTAE-L-------------RICRVNKN---------CGSVKG-GDE-IF  196
_NWW05001.1_      173  ---------------------NTAE-L-------------RICRVNKN---------CGSVKG-GDE-IF  196
_RLW07261.1_      173  ---------------------NTAE-L-------------RICRVNKN---------CGSVKG-GDE-IF  196
_NXR96625.1_      173  ---------------------NTAE-L-------------RICRVNKN---------CGSVKG-GDE-IF  196
_XP_021383204.1_  173  ---------------------NTAE-L-------------RICRVNKN---------CGSVKG-GDE-IF  196
_NXQ25764.1_      173  ---------------------NTAE-L-------------RICRVNKN---------CGSVKG-GDE-IF  196
_NXO08412.1_      173  ---------------------NTAE-L-------------RICRVNKN---------CGSVKG-GDE-IF  196
_NXI32280.1_      173  ---------------------NTAE-L-------------RICRVNKN---------CGSVKG-GDE-IF  196
_NXG00288.1_      173  ---------------------NTAE-L-------------RICRVNKN---------CGSVKG-GDE-IF  196
_XP_041329879.1_  173  ---------------------NTAE-L-------------RICRVNKN---------CGSVKG-GDE-IF  196
_NXV03759.1_      173  ---------------------NTAE-L-------------RICRVNKN---------CGSVKG-GDE-IF  196
_NWT16975.1_      173  ---------------------NTAE-L-------------RICRVNKN---------CGSVKG-GDE-IF  196
_NWT67953.1_      173  ---------------------NTAE-L-------------RICRVNKN---------CGSVKG-GDE-IF  196
_XP_005042765.2_  173  ---------------------NTAE-L-------------RICRVNKN---------CGSVKG-GDE-IF  196
_XP_017600715.1_  173  ---------------------NTAE-L-------------RICRVNKN---------CGSVKG-GDE-IF  196
_NXD93279.1_      173  ---------------------NTAE-L-------------RICRVNKN---------CGSVKG-GDE-IF  196
_NXY02260.1_      173  ---------------------NTAE-L-------------RICRVNKN---------CGSVKG-GDE-IF  196
_NXO84677.1_      173  ---------------------NTAE-L-------------RICRVNKN---------CGSVKG-GDE-IF  196
_NXO64371.1_      173  ---------------------NTAE-L-------------RICRVNKN---------CGSVKG-GDE-IF  196
_NXO42022.1_      173  ---------------------NTAE-L-------------RICRVNKN---------CGSVKG-GDE-IF  196
_NWY65013.1_      173  ---------------------NTAE-L-------------RICRVNKN---------CGSVKG-GDE-IF  196
_NXP90563.1_      173  ---------------------NTAE-L-------------RICRVNKN---------CGSVKG-GDE-IF  196
```

```
_NWI73723.1_      173  ---------------------NTAE-L-------------RICRVNKN---------CGSVKG-GDE-IF  196
_NXH30511.1_      173  ---------------------NTAE-L-------------RICRVNKN---------CGSVKG-GDE-IF  196
_NWT77800.1_      173  ---------------------NTAE-L-------------RICRVNKN---------CGSVKG-GDE-IF  196
_NWT34209.1_      173  ---------------------NTAE-L-------------RICRVNKN---------CGSVKG-GDE-IF  196
_NWY29369.1_      173  ---------------------NTAE-L-------------RICRVNKN---------CGSVKG-GDE-IF  196
_NXQ73821.1_      173  ---------------------NTAE-L-------------RICRVNKN---------CGSVKG-GDE-IF  196
_XP_039572786.1_  173  ---------------------NTAE-L-------------RICRVNKN---------CGSVKG-GDE-IF  196
_XP_030802035.1_  173  ---------------------NTAE-L-------------RICRVNKN---------CGSVKG-GDE-IF  196
_NXE68211.1_      173  ---------------------NTAE-L-------------RICRVNKN---------CGSVKG-GDE-IF  196
_NXS80652.1_      173  ---------------------NTAE-L-------------RICRVNKN---------CGSVKG-GDE-IF  196
_NXO32431.1_      173  ---------------------NTAE-L-------------RICRVNKN---------CGSVKG-GDE-IF  196
_NWR17317.1_      173  ---------------------NTAE-L-------------RICRVNKN---------CGSVKG-GDE-IF  196
_NWZ89732.1_      173  ---------------------NTAE-L-------------RICRVNKN---------CGSVKG-GDE-IF  196
_XP_032039523.1_  173  ---------------------NTAE-L-------------RICRVNKN---------CGSVKG-GDE-IF  196
_KFO04362.1_      173  ---------------------NTAE-L-------------RICRVNKN---------CGSVKG-GDE-IF  196
_XP_054146213.1_  173  ---------------------NTAE-L-------------RICRVNKN---------CGSVKG-GDE-IF  196
_XP_005486916.2_  173  ---------------------NTAE-L-------------RICRVNKN---------CGSVKG-GDE-IF  196
_KAJ7422946.1_    173  ---------------------NTAE-L-------------RICRVNKN---------CGSVKG-GDE-IF  196
_NXC99388.1_      173  ---------------------NTAE-L-------------RICRVNKN---------CGSVKG-GDE-IF  196
_RMC07975.1_      173  ---------------------NTAE-L-------------RICRVNKN---------CGSVKG-GDE-IF  196
_NXT95159.1_      131  ---------------------NTAE-L-------------RICRVNKN---------CGSVKG-GDE-IF  154
_XP_009957589.1_  131  ---------------------NTAE-L-------------RICRVNKN---------CGSVKG-GDE-IF  154
_TRZ13602.1_      131  ---------------------NTAE-L-------------RICRVNKN---------CGSVKG-GDE-IF  154
_NWR45911.1_      122  ---------------------NTAE-L-------------RICRVNKN---------CGSVKG-GDE-IF  145
_KAF4799649.1_    131  ---------------------NTAE-L-------------RICRVNKN---------CGSVKG-GDE-IF  154
_KAJ7420138.1_    167  ---------------------NTAE-L-------------RICRVNKN---------CGSVKG-GDE-IF  190
_XP_054678501.1_       ----------------------------------------------------------------------
_NXP48685.1_      172  ----------------------PNT---------------RICRVNKN---------CGSVKG-GDE-IF  193
_XP_010704701.1_   90  ---------------------NTAE-L-------------RICRVNKN---------CGSVKG-GDE-IF  113
_KFV10805.1_       82  ---------------------NTAE-L-------------RICRVNKN---------CGSVKG-GDE-IF  105
_XP_009572567.1_   52  ---------------------NTAE-L-------------RICRVNKN---------CGSVKG-GDE-IF   75
_NXF64277.1_       51  ---------------------NTAE-L-------------RICRVNKN---------CGSVKG-GDE-IF   74
_KFV54465.1_       51  ---------------------NTAE-L-------------RICRVNKN---------CGSVKG-GDE-IF   74
_KFV90717.1_       51  ---------------------NTAE-L-------------RICRVNKN---------CGSVKG-GDE-IF   74
_KFQ03674.1_       51  ---------------------NTAE-L-------------RICRVNKN---------CGSVKG-GDE-IF   74
_NXW01028.1_       51  ---------------------NTAE-L-------------RICRVNKN---------CGSVKG-GDE-IF   74
_KFP32908.1_       51  ---------------------NTAE-L-------------RICRVNKN---------CGSVKG-GDE-IF   74
_KFV93389.1_       51  ---------------------NTAE-L-------------RICRVNKN---------CGSVKG-GDE-IF   74
_NWI63728.1_       51  ---------------------NTAE-L-------------RICRVNKN---------CGSVKG-GDE-IF   74
```

```
_KAI6072063.1_      52  ---------------------NTAE-L-------------RICRVNKN---------CGSVKG-GDE-IF   75
_KFQ58901.1_        51  ---------------------NTAE-L-------------RICRVNKN---------CGSVKG-GDE-IF   74
_NXP33753.1_        51  ---------------------NTAE-L-------------RICRVNKN---------CGSVKG-GDE-IF   74
_XP_009476353.1_    50  ---------------------NTAE-L-------------RICRVNKN---------CGSVKG-GDE-IF   73
_KFQ55903.1_        51  ---------------------NTAE-L-------------RICRVNKN---------CGSVKG-GDE-IF   74
_XP_042683349.1_    51  ---------------------NTAE-L-------------RICRVNKN---------CGSVKG-GDE-IF   74
_NXR08991.1_        51  ---------------------NTAE-L-------------RICRVNKN---------CGSVKG-GDE-IF   74
_XP_008947250.1_    51  ---------------------NTAE-L-------------RICRVNKN---------CGSVKG-GDE-IF   74
_KFP72464.1_        51  ---------------------NTAE-L-------------RICRVNKN---------CGSVKG-GDE-IF   74
_KFV60523.1_        51  ---------------------NTAE-L-------------RICRVNKN---------CGSVKG-GDE-IF   74
_NXT13165.1_        51  ---------------------NTAE-L-------------RICRVNKN---------CGSVKG-GDE-IF   74
_NWT50506.1_        51  ---------------------NTAE-L-------------RICRVNKN---------CGSVKG-GDE-IF   74
_OXB76139.1_        49  ---------------------NTAE-L-------------RICRVNKN---------CGSVKG-GDE-IF   72
_XP_050827907.1_    64  ---------------------NTAE-L-------------RICRVNKN---------CGSVKG-GDE-IF   87
_XP_030302545.1_    59  ---------------------NTAE-L-------------RICRVNKN---------CGSVKG-GDE-IF   82
_XP_054036038.1_    63  ---------------------NTAE-L-------------RICRVNKN---------CGSVKG-GDE-IF   86
_XP_012788337.1_   173  ---------------------NTAE-L-------------RICRVNKN---------CGSVRG-GDE-IF  196
_XP_008070154.1_   162  ---------------------NTAE-L-------------RICRVNKN---------CGSVRG-GDE-IF  185
_XP_027477445.1_   162  ---------------------NTAE-L-------------RICRVNKN---------CGSVRG-GDE-IF  185
_XP_025848473.1_   162  ---------------------NTAE-L-------------RICRVNKN---------CGSVRG-GDE-IF  185
_XP_049640716.1_   173  ---------------------NTAE-L-------------RICRVNKN---------CGSVRG-GDE-IF  196
_XP_023084089.1_   173  ---------------------NTAE-L-------------RICRVNKN---------CGSVRG-GDE-IF  196
_EHH22143.1_       173  ---------------------NTAE-L-------------RICRVNKN---------CGSVRG-GDE-IF  196
_XP_038504711.1_   173  ---------------------NTAE-L-------------RICRVNKN---------CGSVRG-GDE-IF  196
_XP_038504703.1_   173  ---------------------NTAE-L-------------RICRVNKN---------CGSVRG-GDE-IF  196
_XP_038504718.1_   173  ---------------------NTAE-L-------------RICRVNKN---------CGSVRG-GDE-IF  196
_XP_048971856.1_   173  ---------------------NTAE-L-------------RICRVNKN---------CGSVRG-GDE-IF  196
_KAB1266826.1_     173  ---------------------NTAE-L-------------RICRVNKN---------CGSVRG-GDE-IF  196
_XP_048391424.1_   173  ---------------------NTAE-L-------------KICRVNKN---------TGTVKG-GDE-IF  196
_XP_048391425.1_   173  ---------------------NTAE-L-------------KICRVNKN---------TGTVKG-GDE-IF  196
_GCC28026.1_       173  ---------------------NTAE-L-------------KICRVNKN---------TGTVKG-GDE-IF  196
_XP_043551991.1_   173  ---------------------NTAE-L-------------KICRVNKN---------TGTVKG-GDE-IF  196
_XP_043551989.1_   173  ---------------------NTAE-L-------------KICRVNKN---------TGTVKG-GDE-IF  196
_XP_054534389.1_    83  ---------------------NTAE-L-------------RICRVNKN---------CGSVRG-GDE-IF  106
_XP_011531312.1_    83  ---------------------NTAE-L-------------RICRVNKN---------CGSVRG-GDE-IF  106
_XP_021780541.1_    82  ---------------------NTAE-L-------------RICRVNKN---------CGSVRG-GDE-IF  105
_XP_037862912.1_   103  ---------------------NTAE-L-------------RICRVNKN---------CGSVRG-GDE-IF  126
_XP_015413428.1_   162  ---------------------NTAE-L-------------RICRVNKN---------CGSVRG-GDE-IF  185
_XP_005884642.1_   162  ---------------------NTAE-L-------------RICRVNKN---------CGSVRG-GDE-IF  185
```

```
_KAF6105903.1_     162  ---------------------NTAE-L-------------RICRVNKN---------CGSVRG-GDE-IF  185
_XP_015413426.1_   162  ---------------------NTAE-L-------------RICRVNKN---------CGSVRG-GDE-IF  185
_XP_014387539.1_   162  ---------------------NTAE-L-------------RICRVNKN---------CGSVRG-GDE-IF  185
_XP_014444723.1_   162  ---------------------NTAE-L-------------RICRVNKN---------CGSVRG-GDE-IF  185
_XP_023448058.1_   162  ---------------------NTAE-L-------------RICRVNKN---------CGSVRG-GDE-IF  185
_EGW11604.1_       162  ---------------------NTAE-L-------------RICRVNKN---------CGSVRG-GDE-IF  185
_XP_015985129.1_   162  ---------------------NTAE-L-------------RICRVNKN---------CGSVRG-GDE-IF  185
_XP_012878014.1_   162  ---------------------NTAE-L-------------RICRVNKN---------CGSVKG-GDE-IF  185
_KAG5211506.1_     141  ---------------------NTAE-L-------------RICRVNKN---------CGSVKG-GDE-IF  164
_MXQ82539.1_       137  ---------------------NTAE-L-------------RICRVNKN---------CGSVKG-GDE-IF  160
_XP_032729748.1_   126  ---------------------NTAE-L-------------RICRVNKN---------CGSVRG-GDE-IF  149
_XP_032208113.1_   126  ---------------------NTAE-L-------------RICRVNKN---------CGSVRG-GDE-IF  149
_XP_048208817.1_        ----------------------------------------------------------------------
_XP_038200333.1_        ----------------------------------------------------------------------
_P15307.2_         173  ---------------------NTAE-L-------------RICRVNKN---------CGSVRG-GDE-IF  196
_XP_041524635.1_   162  ---------------------NTAE-L-------------RICRVNKN---------CGSVRG-GDE-IF  185
_CAA33843.1_       173  ---------------------NTAE-L---------------ARVNKN---------CGSVRG-GDE-IF  194
_XP_028732210.2_   162  ---------------------NTAE-L-------------RICRVNKN---------CGSVRG-GDE-IF  185
_XP_025848474.1_   162  ---------------------NTAE-L-------------RICRVNKN---------CGSVRG-GDE-IF  185
_EDL97996.1_       162  ---------------------NTAE-L-------------RICRVNKN---------CGSVRG-GDE-IF  185
_KAI4577886.1_     162  ---------------------NTAE-L-------------RICRVNKN---------CGSVKG-GDE-IF  185
_KAG8512309.1_     174  --------------------------------------------------------------------VF  175
_XP_047637971.1_        ----------------------------------------------------------------------
_XP_019491454.1_   173  ---------------------NTAE-L-------------RICRVNKN---------CGSVRG-GDE-IF  196
_XP_043842482.1_   175  ---------------------NTAE-L-------------RICRVNKN---------CGSVRG-GDE-IF  198
_XP_027721170.1_   175  ---------------------NTAE-L-------------RICRVNKN---------CGSVRG-GDE-IF  198
_XP_036604632.1_   175  ---------------------NTAE-L-------------RICRVNKN---------CGSVRG-GDE-IF  198
_XP_020841164.1_   175  ---------------------NTAE-L-------------RICRVNKN---------CGSVRG-GDE-IF  198
_XP_051831295.1_   175  ---------------------NTAE-L-------------RICRVNKN---------CGSVKG-GDE-IF  198
_XP_031806378.1_   175  ---------------------NTAE-L-------------RICRVNKN---------CGSVKG-GDE-IF  198
_XP_051831294.1_   175  ---------------------NTAE-L-------------RICRVNKN---------CGSVKG-GDE-IF  198
_XP_003339776.1_   175  ---------------------NTAE-L-------------RICRVNKN---------CGSVRG-GDE-IF  198
_XP_044515413.1_   175  ---------------------NTAE-L-------------RICRVNKN---------CGSVRG-GDE-IF  198
_XP_028928361.1_   175  ---------------------NTAE-L-------------RICRVNRN---------CGSVRG-GDE-IF  198
_XP_038607403.1_   175  ---------------------NTAE-L-------------RICRVNRN---------CGSVRG-GDE-IF  198
_XP_040849334.1_    51  ---------------------NTAE-L-------------RICRVNKN---------CGSVRG-GDE-IF   74
_TKC46270.1_        49  ---------------------NTAE-L-------------RICRVNKN---------CGSVRG-GDE-IF   72
_OWK10634.1_        50  ---------------------NTAE-L-------------RICRVNKN---------CGSVKG-GDE-IF   73
_XP_036190342.1_    49  ---------------------NTAE-L-------------RICRVNKN---------CGSVRG-GDE-IF   72
```

```
_KAB0395596.1_      46  ---------------------NTAE-L-------------RICRVNKN---------CGSVRG-GDE-IF   69
_KAF6430170.1_      50  ---------------------NTAE-L-------------RICRVNKN---------CGSVRG-GDE-IF   73
_XP_038948811.1_    51  ---------------------NTAE-L-------------RICRVNKN---------CGSVRG-GDE-IF   74
_KAB1266828.1_     184  ---------------------NTAE-L-------------RICRVNKN---------CGSVRG-GDE-IF  207
_NP_002899.1_      173  ---------------------NTAE-L-------------RICRVNKN---------CGSVRG-GDE-IF  196
_XP_001159537.3_   173  ---------------------NTAE-L-------------RICRVNKN---------CGSVRG-GDE-IF  196
_PNI66333.1_       173  ---------------------NTAE-L-------------RICRVNKN---------CGSVRG-GDE-IF  196
_EHH55592.1_       173  ---------------------NTAE-L-------------RICRVNKN---------CGSVRG-GDE-IF  196
_XP_003262470.1_   173  ---------------------NTAE-L-------------RICRVNKN---------CGSVRG-GDE-IF  196
_BAG64854.1_       173  ---------------------NTAE-L-------------RICRVNKN---------CGSVRG-GDE-IF  196
_XP_032034471.1_   173  ---------------------NTAE-L-------------RICRVNKN---------CGSVRG-GDE-IF  196
_XP_037862911.1_   173  ---------------------NTAE-L-------------RICRVNKN---------CGSVRG-GDE-IF  196
_XP_003830911.1_   173  ---------------------NTAE-L-------------RICRVNKN---------CGSVRG-GDE-IF  196
_XP_004029331.1_   173  ---------------------NTAE-L-------------RICRVNKN---------CGSVRG-GDE-IF  196
_XP_002812051.1_   173  ---------------------NTAE-L-------------RICRVNKN---------CGSVRG-GDE-IF  196
_XP_011711623.1_   173  ---------------------NTAE-L-------------RICRVNKN---------CGSVRG-GDE-IF  196
_XP_033095023.1_   173  ---------------------NTAE-L-------------RICRVNKN---------CGSVRG-GDE-IF  196
_XP_002799280.1_   173  ---------------------NTAE-L-------------RICRVNKN---------CGSVRG-GDE-IF  196
_XP_011853172.1_   173  ---------------------NTAE-L-------------RICRVNKN---------CGSVRG-GDE-IF  196
_XP_050609443.1_   173  ---------------------NTAE-L-------------RICRVNKN---------CGSVRG-GDE-IF  196
_XP_003908735.1_   173  ---------------------NTAE-L-------------RICRVNKN---------CGSVRG-GDE-IF  196
_XP_025209693.1_   173  ---------------------NTAE-L-------------RICRVNKN---------CGSVRG-GDE-IF  196
_XP_023084087.1_   173  ---------------------NTAE-L-------------RICRVNKN---------CGSVRG-GDE-IF  196
_XP_021780539.1_   173  ---------------------NTAE-L-------------RICRVNKN---------CGSVRG-GDE-IF  196
_XP_017704568.1_   173  ---------------------NTAE-L-------------RICRVNKN---------CGSVRG-GDE-IF  196
_XP_010375811.1_   173  ---------------------NTAE-L-------------RICRVNKN---------CGSVRG-GDE-IF  196
_XP_030776399.1_   173  ---------------------NTAE-L-------------RICRVNKN---------CGSVRG-GDE-IF  196
_XP_011797211.1_   173  ---------------------NTAE-L-------------RICRVNKN---------CGSVRG-GDE-IF  196
_XP_012332682.1_   173  ---------------------NTAE-L-------------RICRVNKN---------CGSVRG-GDE-IF  196
_XP_010331875.2_   173  ---------------------NTAE-L-------------RICRVNKN---------CGSVRG-GDE-IF  196
_XP_012332683.1_   173  ---------------------NTAE-L-------------RICRVNKN---------CGSVRG-GDE-IF  196
_XP_017400475.1_   173  ---------------------NTAE-L-------------RICRVNKN---------CGSVRG-GDE-IF  196
_XP_008568357.1_   173  ---------------------NTAE-L-------------RICRVNKN---------CGSVRG-GDE-IF  196
_XP_012620284.1_   173  ---------------------NTAE-L-------------KICRVNKN---------CGSVRG-GDE-IF  196
_XP_002709741.2_   173  ---------------------NTAE-L-------------RICRVNKN---------CGSVRG-GDE-IF  196
_XP_022452943.1_   173  ---------------------NTAE-L-------------RICRVNKN---------CGSVRG-GDE-IF  196
_XP_045405625.1_   173  ---------------------NTAE-L-------------KICRVNKN---------CGSVRG-GDE-IF  196
_XP_004280707.1_   173  ---------------------NTAE-L-------------RICRVNKN---------CGSVRG-GDE-IF  196
_XP_030733169.1_   173  ---------------------NTAE-L-------------RICRVNKN---------CGSVRG-GDE-IF  196
```

```
_TEA23022.1_       173  ---------------------NTAE-L-------------RICRVNKN---------CGSVRG-GDE-IF  196
_XP_012494629.1_   173  ---------------------NTAE-L-------------KICRVNKN---------CGSVRG-GDE-IF  196
_XP_010950191.1_   173  ---------------------NTAE-L-------------RICRVNKN---------CGSVRG-GDE-IF  196
_XP_024608785.1_   173  ---------------------NTAE-L-------------RICRVNKN---------CGSVRG-GDE-IF  196
_XP_006201637.1_   173  ---------------------NTAE-L-------------RICRVNKN---------CGSVRG-GDE-IF  196
_XP_019285354.1_   173  ---------------------NTAE-L-------------RICRVNKN---------CGSVRG-GDE-IF  196
_XP_032509224.1_   173  ---------------------NTAE-L-------------RICRVNKN---------CGSVRG-GDE-IF  196
_XP_047379355.1_   173  ---------------------NTAE-L-------------RICRVNKN---------CGSVRG-GDE-IF  196
_XP_032981134.1_   173  ---------------------NTAE-L-------------RICRVNKN---------CGSVRG-GDE-IF  196
_XP_005600018.1_   173  ---------------------NTAE-L-------------RICRVNKN---------CGSVRG-GDE-IF  196
_XP_046517051.1_   173  ---------------------NTAE-L-------------RICRVNKN---------CGSVRG-GDE-IF  196
_XP_007464205.1_   173  ---------------------NTAE-L-------------RICRVNKN---------CGSVRG-GDE-IF  196
_XP_019572903.1_   173  ---------------------NTAE-L-------------RICRVNKN---------CGSVRG-GDE-IF  196
_XP_007189755.1_   173  ---------------------NTAE-L-------------RICRVNKN---------CGSVRG-GDE-IF  196
_XP_004397771.1_   173  ---------------------NTAE-L-------------RICRVNKN---------CGSVRG-GDE-IF  196
_XP_004435713.1_   173  ---------------------NTAELL-------------RICRVNKN---------CGSVRG-GDE-IF  197
_XP_008698124.1_   173  ---------------------NTAE-L-------------RICRVNKN---------CGSVRG-GDE-IF  196
_XP_023989255.1_   173  ---------------------NTAE-L-------------RICRVNKN---------CGSVRG-GDE-IF  196
_XP_011233012.2_   173  ---------------------NTAE-L-------------RICRVNKN---------CGSVRG-GDE-IF  196
_XP_025724444.1_   173  ---------------------NTAE-L-------------RICRVNKN---------CGSVRG-GDE-IF  196
_XP_005626186.1_   173  ---------------------NTAE-L-------------RICRVNKN---------CGSVRG-GDE-IF  196
_XP_045643991.1_   173  ---------------------NTAE-L-------------RICRVNKN---------CGSVRG-GDE-IF  196
_XP_034857383.1_   173  ---------------------NTAE-L-------------RICRVNKN---------CGSVRG-GDE-IF  196
_XP_027945501.1_   173  ---------------------NTAE-L-------------RICRVNKN---------CGSVRG-GDE-IF  196
_XP_026341094.1_   173  ---------------------NTAE-L-------------RICRVNKN---------CGSVRG-GDE-IF  196
_EFB29923.1_       173  ---------------------NTAE-L-------------RICRVNKN---------CGSVRG-GDE-IF  196
_XP_045740897.1_   173  ---------------------NTAE-L-------------RICRVNKN---------CGSVRG-GDE-IF  196
_CAD7677454.1_     173  ---------------------NTAE-L-------------RICRVNKN---------CGSVRG-GDE-IF  196
_XP_035577553.1_   173  ---------------------NTAE-L-------------RICRVNKN---------CGSVRG-GDE-IF  196
_XP_006733255.1_   173  ---------------------NTAE-L-------------RICRVNKN---------CGSVRG-GDE-IF  196
_XP_032266820.1_   173  ---------------------NTAE-L-------------RICRVNKN---------CGSVRG-GDE-IF  196
_XP_041611730.1_   173  ---------------------NTAE-L-------------RICRVNKN---------CGSVRG-GDE-IF  196
_XP_048971854.1_   173  ---------------------NTAE-L-------------RICRVNKN---------CGSVRG-GDE-IF  196
_XP_021554840.1_   173  ---------------------NTAE-L-------------RICRVNKN---------CGSVRG-GDE-IF  196
_VCW78562.1_       173  ---------------------NTAE-L-------------RICRVNKN---------CGSVRG-GDE-IF  196
_XP_034857381.1_   173  ---------------------NTAE-L-------------RICRVNKN---------CGSVRG-GDE-IF  196
_XP_008524231.1_   170  ---------------------NTAE-L-------------RICRVNKN---------CGSVRG-GDE-IF  193
_XP_045740895.1_   173  ---------------------NTAE-L-------------RICRVNKN---------CGSVRG-GDE-IF  196
_XP_045834746.1_   173  ---------------------NTAE-L-------------RICRVNKN---------CGSVRG-GDE-IF  196
```

```
_XP_035932906.1_  173  ---------------------NTAE-L-------------RICRVNKN---------CGSVRG-GDE-IF  196
_XP_030883859.1_  173  ---------------------NTAE-L-------------RICRVNKN---------CGSVRG-GDE-IF  196
_XP_032266818.1_  173  ---------------------NTAE-L-------------RICRVNKN---------CGSVRG-GDE-IF  196
_XP_024408528.2_  173  ---------------------NTAE-L-------------RICRVNKN---------CGSVRG-GDE-IF  196
_XP_004580347.1_  173  ---------------------NTAE-L-------------RICRVNKN---------CGSVRG-GDE-IF  196
_XP_030166525.1_  173  ---------------------NTAE-L-------------RICRVNKN---------CGSVRG-GDE-IF  196
_XP_035932904.1_  173  ---------------------NTAE-L-------------RICRVNKN---------CGSVRG-GDE-IF  196
_XP_020040690.1_  173  ---------------------NTAE-L-------------RICRVNKN---------CGSVRG-GDE-IF  196
_XP_053445604.1_  173  ---------------------NTAE-L-------------KICRVNKN---------CGSVRG-GDE-IF  196
_XP_049507482.1_  173  ---------------------NTAE-L-------------RICRVNKN---------CGSVRG-GDE-IF  196
_XP_015335127.1_  173  ---------------------NTAE-L-------------RICRVNKN---------CGSVRG-GDE-IF  196
_XP_027790245.1_  173  ---------------------NTAE-L-------------RICRVNKN---------CGSVRG-GDE-IF  196
_XP_003984077.1_  173  ---------------------NTAE-L-------------RICRVNKN---------CGSVRG-GDE-IF  196
_XP_039096259.1_  173  ---------------------NTAE-L-------------RICRVNKN---------CGSVRG-GDE-IF  196
_XP_007075434.1_  173  ---------------------NTAE-L-------------RICRVNKN---------CGSVRG-GDE-IF  196
_XP_022360130.1_  173  ---------------------NTAE-L-------------RICRVNKN---------CGSVRG-GDE-IF  196
_XP_047707378.1_  173  ---------------------NTAE-L-------------RICRVNKN---------CGSVRG-GDE-IF  196
_XP_026243031.1_  173  ---------------------NTAE-L-------------RICRVNKN---------CGSVRG-GDE-IF  196
_XP_037383340.1_  173  ---------------------NTAE-L-------------RICRVNKN---------CGSVSG-GDE-IF  196
_XP_005322095.1_  173  ---------------------NTAE-L-------------RICRVNKN---------CGSVRG-GDE-IF  196
_XP_005662584.1_  173  ---------------------NTAE-L-------------RICRVNKN---------CGSVRG-GDE-IF  196
_XP_040080101.1_  173  ---------------------NTAE-L-------------RICRVNKN---------CGSVKG-GDE-IF  196
_XP_017503999.1_  173  ---------------------NTAE-L-------------RICRVNKN---------CGSVRG-GDE-IF  196
_XP_044119954.1_  173  ---------------------NTAE-L-------------RICRVNKN---------CGSVRG-GDE-IF  196
_XP_032208110.1_  173  ---------------------NTAE-L-------------RICRVNKN---------CGSVRG-GDE-IF  196
_XP_012586936.1_  173  ---------------------NTAE-L-------------RICRVNKN---------CGSVSG-GDE-IF  196
_XP_032729745.1_  173  ---------------------NTAE-L-------------RICRVNKN---------CGSVRG-GDE-IF  196
_XP_052504199.1_  173  ---------------------NTAE-L-------------RICRVNKN---------CGSVKG-GDE-IF  196
_XP_054431389.1_  173  ---------------------NTAE-L-------------RICRVNKN---------CGSVRG-GDE-IF  196
_XP_023576632.1_  173  ---------------------NTAE-L-------------RICRVNKN---------CGSVRG-GDE-IF  196
_XP_004742070.1_  173  ---------------------NTAE-L-------------RICRVNKN---------CGSVRG-GDE-IF  196
_XP_037663267.1_  173  ---------------------NTAE-L-------------RICRVNKN---------CGSVRG-GDE-IF  196
_XP_004005978.1_  173  ---------------------NTAE-L-------------RICRVNKN---------CGSVKG-GDE-IF  196
_XP_036781908.1_  173  ---------------------NTAE-L-------------RICRVNKN---------CGSVRG-GDE-IF  196
_XP_040849333.1_  173  ---------------------NTAE-L-------------RICRVNKN---------CGSVRG-GDE-IF  196
_XP_005686691.1_  173  ---------------------NTAE-L-------------RICRVNKN---------CGSVKG-GDE-IF  196
_XP_023367900.1_  173  ---------------------NTAE-L-------------KICRVNKN---------CGSARG-GDE-IF  196
_KAF0879576.1_    173  ---------------------NTAE-L-------------RICRVNKN---------CGSVRG-GDE-IF  196
_XP_029793284.1_  173  ---------------------NTAE-L-------------RICRVNKN---------CGSVRG-GDE-IF  196
```

```
_NP_001179899.1_  173  ---------------------NTAE-L-------------RICRVNKN---------CGSVKG-GDE-IF  196
_XP_020764718.1_  173  ---------------------NTAE-L-------------RICRVNKN---------CGSVKG-GDE-IF  196
_XP_054431388.1_  173  ---------------------NTAE-L-------------RICRVNKN---------CGSVRG-GDE-IF  196
_XP_037663265.1_  173  ---------------------NTAE-L-------------RICRVNKN---------CGSVRG-GDE-IF  196
_XP_019825763.1_  173  ---------------------NTAE-L-------------RICRVNKN---------CGSVKG-GDE-IF  196
_XP_005889096.1_  173  ---------------------NTAE-L-------------RICRVNKN---------CGSVKG-GDE-IF  196
_XP_043324571.1_  173  ---------------------NTAE-L-------------RICRVNKN---------CGSVKG-GDE-IF  196
_KAJ1078272.1_    173  ---------------------NTAE-L-------------RICRVNKN---------CGSVKG-GDE-IF  196
_XP_036190339.1_  173  ---------------------NTAE-L-------------RICRVNKN---------CGSVRG-GDE-IF  196
_XP_013005522.1_  173  ---------------------NTAE-L-------------RICRVNKN---------CGSVRG-GDE-IF  196
_XP_006073936.1_  173  ---------------------NTAE-L-------------RICRVNKN---------CGSVKG-GDE-IF  196
_XP_016069529.1_  173  ---------------------NTAE-L-------------RICRVNKN---------CGSVRG-GDE-IF  196
_KAB0354136.1_    173  ---------------------NTAE-L-------------RICRVNKN---------CGSVKG-GDE-IF  196
_XP_029424871.1_  173  ---------------------NTAE-L-------------RICRVNKN---------CGSVRG-GDE-IF  196
_XP_036118792.1_  173  ---------------------NTAE-L-------------RICRVNKN---------CGSVRG-GDE-IF  196
_XP_037019725.2_  172  ---------------------NTAE-L-------------RICRVNKN---------CGSVRG-GDE-IF  195
_XP_047602572.1_  173  ---------------------NTAE-L-------------RICRVNKN---------CGSVRG-GDE-IF  196
_XP_014586639.1_  173  ---------------------NTAE-L-------------RICRVNKN---------CGSVRG-GDE-IF  196
_XP_046517052.1_  173  ---------------------NTAE-L-------------RICRVNKN---------CGSVRG-GDE-IF  196
_XP_006909635.2_  173  ---------------------NTAE-L-------------RICRVNKN---------CGSVRG-GDE-IF  196
_XP_011362222.1_  173  ---------------------NTAE-L-------------RICRVNKN---------CGSVRG-GDE-IF  196
_XP_045684409.1_  173  ---------------------NTAE-L-------------RICRVNKN---------CGSVRG-GDE-IF  196
_XP_048208816.1_  173  ---------------------NTAE-L-------------RICRVNKN---------CGSVKG-GDE-IF  196
_XP_052590629.1_  173  ---------------------NTAE-L-------------RICRVNKN---------CGSVRG-GDE-IF  196
_XP_032353499.1_  173  ---------------------NTAE-L-------------RICRVNKN---------CGSVRG-GDE-IF  196
_XP_038200327.1_  173  ---------------------NTAE-L-------------RICRVNKN---------CGSVRG-GDE-IF  196
_XP_051056550.1_  173  ---------------------NTAE-L-------------RICRVNKN---------CGSVRG-GDE-IF  196
_XP_012805850.2_  173  ---------------------NTAE-L-------------RICRVNKN---------CGSVSG-GDE-IF  196
_XP_016050906.1_  173  ---------------------NTAE-L-------------RICRVNKN---------CGSVRG-GDE-IF  196
_XP_030166526.1_  173  ---------------------NTAE-L-------------RICRVNKN---------CGSVRG-GDE-IF  196
_XP_036903878.1_  172  ---------------------NTAE-L-------------RICRVNKN---------CGSVRG-GDE-IF  195
_CAH7004032.1_    173  ---------------------NTAE-L-------------RICRVNKN---------CGSVRG-GDE-IF  196
_XP_051056549.1_  173  ---------------------NTAE-L-------------RICRVNKN---------CGSVRG-GDE-IF  196
_XP_042547166.1_  173  ---------------------NTAE-L-------------RICRVNKN---------CGSVKG-GDE-IF  196
_XP_054584456.1_  173  ---------------------NTAE-L-------------RICRVNKN---------CGSVRG-GDE-IF  196
_XP_049996768.1_  173  ---------------------NTAE-L-------------RICRVNKN---------CGSVRG-GDE-IF  196
_XP_027243805.1_  173  ---------------------NTAE-L-------------RICRVNKN---------CGSVRG-GDE-IF  196
_XP_047707379.1_  173  ---------------------NTAE-L-------------RICRVNKN---------CGSVRG-GDE-IF  196
_XP_026645370.1_  173  ---------------------NTAE-L-------------RICRVNKN---------CGSVRG-GDE-IF  196
```

```
_XP_014302519.1_  173  ---------------------NTAE-L-------------RICRVNKN---------CGSVRG-GDE-IF  196
_XP_049726303.1_  173  ---------------------NTAE-L-------------RICRVNKN---------CGSVRG-GDE-IF  196
_XP_049726302.1_  173  ---------------------NTAE-L-------------RICRVNKN---------CGSVRG-GDE-IF  196
_XP_012969026.1_  173  ---------------------NTAE-L-------------RICRVNKN---------CGSVRG-GDE-IF  196
_XP_008524232.1_  170  ---------------------NTAE-L-------------RICRVNKN---------CGSVRG-GDE-IF  193
_XP_051020904.1_  173  ---------------------NTAE-L-------------RICRVNKN---------CGSVRG-GDE-IF  196
_XP_023590712.1_  173  ---------------------NTAE-L-------------RICRVNKN---------CGSVRG-GDE-IF  196
_XP_027243803.1_  173  ---------------------NTAE-L-------------RICRVNKN---------CGSVRG-GDE-IF  196
_XP_040589151.1_  173  ---------------------NTAE-L-------------RICRVNKN---------CGSVRG-GDE-IF  196
_XP_023412959.1_  173  ---------------------NTAE-L-------------RICRVNKN---------CGSVRG-GDE-IF  196
_XP_036057111.1_  173  ---------------------NTAE-L-------------RICRVNKN---------CGSVRG-GDE-IF  196
_XP_027822602.1_  173  ---------------------NTAE-L-------------RICRVNKN---------CGSVKG-GDE-IF  196
_XP_031322949.1_  173  ---------------------NTAE-L-------------RICRVNKN---------CGSVRG-GDE-IF  196
_XP_028634966.1_  173  ---------------------NTAE-L-------------RICRVNKN---------CGSVRG-GDE-IF  196
_XP_031531529.1_  173  ---------------------NTAE-L-------------RICRVNKN---------CGSVRG-GDE-IF  196
_XP_006073937.1_  173  ---------------------NTAE-L-------------RICRVNKN---------CGSVKG-GDE-IF  196
_XP_034364887.1_  173  ---------------------NTAE-L-------------RICRVNKN---------CGSVRG-GDE-IF  196
_XP_021482246.1_  173  ---------------------NTAE-L-------------RICRVNKN---------CGSVRG-GDE-IF  196
_KAF4016326.1_    173  ---------------------NTAE-L-------------RICRVNKN---------CGSVKG-GDE-IF  196
_XP_023602319.1_  173  ---------------------NTAE-L-------------RICRVNKN---------CGSVRG-GDE-IF  196
_XP_052054164.1_  173  ---------------------NTAE-L-------------RICRVNKN---------CGSVRG-GDE-IF  196
_XP_036291310.1_  173  ---------------------NTAE-L-------------RICRVNKN---------CGSVKG-GDE-IF  196
_XP_013370168.1_  173  ---------------------NTAE-L-------------RICRVNKN---------CGSVRG-GDE-IF  196
_XP_023590722.1_  173  ---------------------NTAE-L-------------RICRVNKN---------CGSVRG-GDE-IF  196
_XP_048291328.1_  173  ---------------------NTAE-L-------------RICRVNKN---------CGSVRG-GDE-IF  196
_XP_032772910.1_  173  ---------------------NTAE-L-------------RICRVNKN---------CGSVRG-GDE-IF  196
_XP_008768670.2_  173  ---------------------NTAE-L-------------RICRVNKN---------CGSVRG-GDE-IF  196
_XP_014302521.1_  173  ---------------------NTAE-L-------------RICRVNKN---------CGSVRG-GDE-IF  196
_XP_031194882.1_  173  ---------------------NTAE-L-------------RICRVNKN---------CGSVRG-GDE-IF  196
_NP_033070.2_     173  ---------------------NTAE-L-------------RICRVNKN---------CGSVRG-GDE-IF  196
_XP_036291312.1_  173  ---------------------NTAE-L-------------RICRVNKN---------CGSVKG-GDE-IF  196
_XP_021033157.1_  173  ---------------------NTAE-L-------------RICRVNKN---------CGSVRG-GDE-IF  196
_XP_015849136.1_  173  ---------------------NTAE-L-------------RICRVNKN---------CGSVRG-GDE-IF  196
_XP_045150027.1_  173  ---------------------NTAE-L-------------RICRVNKN---------CGSVRG-GDE-IF  196
_XP_021066684.1_  173  ---------------------NTAE-L-------------RICRVNKN---------CGSVRG-GDE-IF  196
_XP_006881088.1_  173  ---------------------NTAE-L-------------RICRVNKN---------CGSVRG-GDE-IF  196
_XP_008524234.1_  170  ---------------------NTAE-L-------------RICRVNKN---------CGSVRG-GDE-IF  193
_XP_021482260.1_  173  ---------------------NTAE-L-------------RICRVNKN---------CGSVRG-GDE-IF  196
_XP_007125500.1_  173  ---------------------NTAE-L-------------RICRVNKN---------CGSVRG-GDE-IF  196
```

```
_XP_028352731.1_  173  ---------------------NTAE-L-------------RICRVNKN---------CGSVRG-GDE-IF  196
_XP_028352733.1_  173  ---------------------NTAE-L-------------RICRVNKN---------CGSVRG-GDE-IF  196
_XP_028352734.1_  173  ---------------------NTAE-L-------------RICRVNKN---------CGSVRG-GDE-IF  196
_XP_028352736.1_  173  ---------------------NTAE-L-------------RICRVNKN---------CGSVRG-GDE-IF  196
_XP_028352735.1_  173  ---------------------NTAE-L-------------RICRVNKN---------CGSVRG-GDE-IF  196
_ERE88482.1_      173  ---------------------NTAE-L-------------RICRVNKN---------CGSVRG-GDE-IF  196
_KAF3826750.1_     52  ---------------------NTAE-L-------------------------------------------   56
_OBS70420.1_           ----------------------------------------------------------------------
_XP_006878094.1_  136  ---------------------NTAE-L-------------RICRVNKN---------CGSVRG-GDE-IF  159
_XP_008978905.3_  130  ---------------------NTAE-L-------------RICRVNKN---------CGSVRG-GDE-IF  153
_XP_032147941.1_  130  ---------------------NTAE-L-------------RICRVNKN---------CGSVRG-GDE-IF  153
_XP_008524233.1_  132  ---------------------NTAE-L-------------RICRVNKN---------CGSVRG-GDE-IF  155
_XP_044628438.1_  130  ---------------------NTAE-L-------------RICRVNKN---------CGSVRG-GDE-IF  153
_XP_010608101.1_  130  ---------------------NTAE-L-------------RICRVNKN---------CGSVRG-GDE-IF  153
_KAF5918007.1_    132  ---------------------NTAE-L-------------RICRVNKN---------CGSVRG-GDE-IF  155
_XP_021101942.1_  130  ---------------------NTAE-L-------------RICRVNKN---------CGSVRG-GDE-IF  153
_ABM89308.1_       49  ---------------------NTAE-L-------------RICRVNKN---------CGSVRG-GDE-IF   72
_MBW00678.1_       34  ---------------------NTAE-L-------------RICRVNKN---------CGSVRG-GDE-IF   57
_NP_001096177.1_  173  ---------------------NTAE-L-------------RICRVNKN---------CGSVNG-GDE-IF  196
_OCT77375.1_      173  ---------------------NTAE-L-------------RICRVNKN---------CGSVNG-GDE-IF  196
_AAI70187.1_      173  ---------------------NTAE-L-------------RICRVNKN---------CGSVNG-GDE-IF  196
_AAI69362.1_      173  ---------------------NTAE-L-------------RICRVNKN---------CGNVNG-GDE-IF  196
_XP_041418586.1_  173  ---------------------NTAE-L-------------RICRVNKN---------CGNVNG-GDE-IF  196
_XP_041418585.1_  173  ---------------------NTAE-L-------------RICRVNKN---------CGNVNG-GDE-IF  196
_XP_041418587.1_  173  ---------------------NTAE-L-------------RICRVNKN---------CGNVNG-GDE-IF  196
_XP_053316221.1_  173  ---------------------NTAE-L-------------RICRVNKN---------CGSVRG-DDE-IF  196
_XP_053568018.1_  173  ---------------------NTAE-L-------------RICRVNKN---------CGSVRG-GDE-IF  196
_KAG8443975.1_    173  ---------------------NTAE-L-------------RICRVNKN---------CGSVNG-GDE-IF  196
_XP_044145067.1_  175  ---------------------NTAE-L-------------RICRVNKN---------CGSAAG-GDE-IF  198
_KAG9484268.1_    175  ---------------------NTAE-L-------------RICRVNKN---------CGSAAG-GDE-IF  198
_XP_040285660.1_  175  ---------------------NTAE-L-------------RICRVNKN---------CGSAAG-GDE-IF  198
_XP_040285659.1_  175  ---------------------NTAE-L-------------RICRVNKN---------CGSAAG-GDE-IF  198
_KAG8583825.1_    175  ---------------------NTAE-L-------------RICRVNKN---------CGSAAG-GDE-IF  198
_XP_018411072.1_  175  ---------------------NTAE-L-------------RICRVNKN---------CGSAAG-GDE-IF  198
_XP_040207073.1_  175  ---------------------NTAE-L-------------RICRVNKN---------CGTAAG-GDE-IF  198
_NP_001081750.1_  170  ---------------------NTAE-L-------------RICRVNKN---------CGSANG-GDE-IF  193
_PIO39092.1_       51  ---------------------NTAE-L-------------RICRVNKN---------CGSAAG-GDE-IF   74
_PIO39091.1_       51  ---------------------NTAE-L-------------RICRVNKN---------CGSAAG-GDE-IF   74
_XP_041035877.1_  172  ---------------------NTAE-L-------------KICRVNKN---------SGSCKG-GEE-IF  195
```

```
_XP_042202691.1_  172  ---------------------NTAE-L-------------KICRINKN---------SGSCRG-GDE-IF  195
_XP_048475509.1_  161  ---------------------NTAE-L-------------KICRVNKN---------SGSCRG-GEE-IF  184
_XP_048380947.1_  161  ---------------------NTAE-L-------------KICRVNKN---------SGSCRG-GEE-IF  184
_XP_051901152.1_  165  ---------------------NTAE-L-------------KICRVNRN---------SGSCRG-GEE-IF  188
_BAD60879.1_      157  ----------------------IHD-L-------------VICRLSRQ---------SGYAGG-GDE-VF  179
_XP_050405370.1_  169  ----------------------LND-L-------------VICRLDRS---------SGKAQG-GDE-VF  191
_XP_009063713.1_  169  ----------------------LND-L-------------VICRVDKS---------SGRAKG-GDE-VF  191
_CAB0044847.1_    188  ----------------------END-I-------------SIQFASHA---------SDSVIG-GTE-MC  210
_XP_048259911.1_  171  ----------------------VNE-L-------------TICRVDRS---------SGKAKG-GDE-IF  193
_ADI72431.1_      171  ----------------------VNE-L-------------TICRVDRS---------SGKAKG-GDE-IF  193
_CAG5133522.1_    172  ----------------------SKD-L-------------VICRVDKS---------SGKARG-GDE-VF  194
_KAG5688355.1_     82  ----------------------LND-L--------------ICRVDKS---------SGKARG-GDE-VF  103
_AMT84567.1_      170  ----------------------VSD-L-------------VICRVSRS---------AGTAKG-GDE-VF  192
_XP_052276378.1_  169  ----------------------LND-L-------------VICRIDKT---------SGKAKG-GDE-VF  191
_XP_052276377.1_  169  ----------------------LND-L-------------VICRIDKT---------SGKAKG-GDE-VF  191
_AAK72690.1_      171  ----------------------LNE-L-------------VICRVDRH---------SGKAKG-GDE-VF  193
_XP_011414731.2_  171  ----------------------LNE-L-------------VICRVDRH---------SGKAKG-GDE-VF  193
_XP_052699807.1_  171  ----------------------LNE-L-------------VICRVDRH---------SGKAKG-GDE-VF  193
_NP_001292261.1_  171  ----------------------LNE-L-------------VICRVDRH---------SGKAKG-GDE-VF  193
_XP_048738054.1_  171  ----------------------LNE-L-------------VICRVDRS---------SGKAKG-GDE-VF  193
_XP_052699808.1_  171  ----------------------LNE-L-------------VICRVDRH---------SGKAKG-GDE-VF  193
_XP_022332457.1_  171  ----------------------MNE-L-------------VICRVDKS---------SGKAKG-GDE-VF  193
_ABL63469.1_      170  ----------------------LNE-L-------------VICRVDRS---------SGKAKG-GDE-VF  192
_XP_033733822.1_  171  ----------------------LNE-L-------------VICRVDKS---------SGKAKG-GDE-VF  193
_XP_021347355.1_  171  ----------------------LNE-L-------------VICRVDRS---------SGKAKG-GDE-VF  193
_UOA58607.1_      171  ----------------------LNE-L-------------VICRVDKS---------SGKAKG-GDE-VF  193
_XP_021347356.1_  171  ----------------------LNE-L-------------VICRVDRS---------SGKAKG-GDE-VF  193
_CAG2186872.1_    170  ----------------------LNE-L-------------VICRVDKS---------SGRAKG-GDE-VF  192
_XP_052058915.1_  170  ----------------------LNE-L-------------VICRVDKS---------SGRAKG-GDE-VF  192
_KAJ8309232.1_    171  ----------------------LNE-L-------------VICRVDKS---------SGKAKG-GDE-VF  193
_UZH84475.1_      169  ----------------------LND-L-------------VICRVDRT---------SGKSKG-GDE-VF  191
_ALA99939.1_      169  ----------------------LND-L-------------VICRVDRT---------SGKPRG-GDE-VF  191
_XP_052767764.1_  169  ----------------------LND-L-------------VICRVDKT---------SGKSKG-GDE-VF  191
_ADD25211.1_      171  ----------------------LNE-L-------------VICRVDKS---------SGKAKG-GDE-VF  193
_XP_052224245.1_  169  ----------------------LND-L-------------VICQPT-----------SGKAKG-GDE-VV  189
_KAJ8314234.1_    124  ----------------------LNE-L-------------MICRVSKS---------SGSAKG-GDE-IF  146
_VDH95719.1_      129  ----------------------LNE-L-------------VICRVDKS---------SGRAKG-GDE-VF  151
_XP_041352913.1_  171  ----------------------LNE-L-------------VICRVDRS---------SGRARG-GDE-IF  193
_XP_046555158.1_  171  ----------------------VNE-L-------------TICRVDRS---------SGKAKG-GDE-IF  193
```

```
_AAW33559.1_       171  ----------------------VNE-L-------------TICRVDRS---------SGKAKG-GDE-IF  193
_ABY87435.1_       158  ----------------------VNE-L-------------TICRVDRS---------SGKAKG-GDE-IF  180
_XP_009028834.1_   171  ----------------------VHD-L-------------VICRLSRQ---------SGYAVG-GDE-VF  193
_XP_025106109.1_   171  ----------------------LND-L-------------VICRVDKS---------SGRARG-GDE-VF  193
_NP_001298191.1_   172  ----------------------SKD-L-------------VICRVDKS---------SGKARG-GDE-VF  194
_KAH9519545.1_     172  ----------------------SKD-L-------------VICRVDKS---------SGKARG-GDE-VF  194
_RUS74561.1_       172  ----------------------SKD-L-------------VICRVDKS---------SGKARG-KDE-VF  194
_GFO11973.1_       172  ----------------------SKD-L-------------VICRVDKS---------SGKARG-KEE-VF  194
_CAG5132534.1_     172  ----------------------SKD-L-------------VICRVDRS---------SGKARG-GDE-IF  194
_GFR69641.1_       172  ----------------------SKD-L-------------VICRVDKS---------SGKARG-RDE-VF  194
_XP_035824873.1_   172  ----------------------SKD-L-------------VICRVDKT---------SGKARG-GDE-VF  194
_AAY27981.1_       170  ----------------------QTD-L-------------SIFRMDKC---------SGKSTG-HDE-VF  192
_XP_052827753.1_   169  ----------------------QNE-L-------------SIVRMDKC---------SGRACG-RDE-VF  191
_XP_029642523.1_   169  ----------------------QNE-L-------------SIVRMDKC---------SGRACG-RDE-VF  191
_CAE1288013.1_     129  ----------------------QTD-L-------------SICRMDKS---------SGKSTG-HDE-VF  151
_ELU04547.1_       170  ----------------------VND-L-------------VICRLSRQ---------AGSAKG-GDE-VI  192
_KAI0227899.1_     168  ----------------------VSE-L-------------VICRISKQ---------AGSVNG-GDQ-VF  190
_XP_044002103.1_   175  ----------------------LSS-L-------------RIHRVEPC---------CASAAG-SKT-VV  197
_XP_044002102.1_   174  ----------------------MSD-L-------------VICRLSHC---------CASAAG-GTS-VI  196
_XP_044002099.1_   174  ----------------------MSD-L-------------VICRLSHC---------CASAAG-GTS-VI  196
_XP_037026629.1_   174  ----------------------NVK-L-------------RIFEKSDN---------YSAACG-DKK-II  196
_CAD7632570.1_     176  ----------------------LPK-L-------------IITDISTT---------SCRMTG-GK--VM  197
_CAD7650738.1_     176  ----------------------LPK-L-------------IITDISTT---------CAPITG-GK--VM  197
_XP_054163335.1_   174  ----------------------CNE-L-------------NIIDISSH---------TISSNG---SKLM  195
_CAD7629985.1_     181  ----------------------CNE-L-------------NVIDISSN---------TISTNG---SKLM  202
_CAD7652069.1_     174  ----------------------CNE-L-------------NVIDISSH---------TISTNG---SKLM  195
_XP_017954168.1_   174  ------------------------E-L-------------TINRLCSC---------SSKLSG-GDQ-II  194
_XP_017858583.1_   174  ------------------------E-L-------------TINRLCSC---------SSKLSG-GDQ-II  194
_XP_002001588.1_   174  ------------------------E-L-------------TINRLCSC---------SSKLSG-GDQ-II  194
_XP_017954163.1_   174  ------------------------E-L-------------TINRLCSC---------SSKLSG-GDQ-II  194
_XP_023164563.2_   174  ------------------------E-L-------------TINRLCSC---------SSKLSG-GDQ-II  194
_TDG46702.1_       174  ------------------------E-L-------------TINRLCSC---------SSKLSG-GDQ-II  194
_XP_030564898.1_   174  ------------------------E-L-------------TINRLCSC---------SSKLSG-GDQ-II  194
_XP_015024496.1_   174  ------------------------E-L-------------TINRLCSC---------SSKLSG-GDQ-II  194
_XP_034475238.1_   175  ------------------------E-L-------------TITRLCSC---------SSKMSG-GDE-II  195
_XP_034475235.1_   175  ------------------------E-L-------------TITRLCSC---------SSKMSG-GDE-II  195
_XP_017854415.1_   174  ------------------------E-L-------------TINRICSC---------SSKLSG-GDR-II  194
_XP_017854416.1_   174  ------------------------E-L-------------TINRICSC---------SSKLSG-GDR-II  194
_ALC40089.1_       174  ------------------------E-L-------------TINRICSC---------SSKLSG-GDR-II  194
```

```
_XP_030382983.1_   177  ------------------------E-P-------------LIARLCSC---------AAKVTG-GDE-II  197
_XP_030382981.1_   177  ------------------------E-P-------------LIARLCSC---------AAKVTG-GDE-II  197
_XP_001989397.1_   174  ------------------------E-L-------------TINRLCSC---------SSKLSG-GDQ-II  194
_XP_002067135.2_   175  ------------------------E-L-------------TITRLCSC---------AAKVSG-GDE-II  195
_XP_034475239.1_   173  ------------------------E-L-------------TITRLCCG---------FSKMSG-GDE-II  193
_KAH8411901.1_     175  ------------------------E-L-------------TISGLCSC---------SGKLSG-GDK-II  195
_XP_016963143.1_   175  ------------------------E-L-------------TITRLCSC---------SAQVTG-GNE-II  195
_XP_037712361.1_   175  ------------------------E-L-------------TITRLCSC---------SAQATG-GSE-II  195
_XP_016993627.2_   175  ------------------------E-L-------------TITRLCSC---------SAQVTG-GSE-II  195
_XP_016963141.1_   175  ------------------------E-L-------------TITRLCSC---------SAQVTG-GNE-II  195
_XP_016993624.2_   175  ------------------------E-L-------------TITRLCSC---------SAQVTG-GSE-II  195
_XP_037712358.1_   175  ------------------------E-L-------------TITRLCSC---------SAQATG-GSE-II  195
_XP_001974449.1_   175  ------------------------E-L-------------TITRLCSC---------AATANG-GDE-II  195
_AAM48404.1_       175  ------------------------E-L-------------TITRLCSC---------AATANG-GDE-II  195
_NP_001162999.1_   175  ------------------------E-L-------------TITRLCSC---------AATANG-GDE-II  195
_XP_026835878.1_   175  ------------------------E-L-------------TITRLCSC---------AATANG-GDE-II  195
_AAA28465.1_       175  ------------------------E-L-------------TITRLCSC---------AATANG-GDE-II  195
_XP_002038912.1_   175  ------------------------E-L-------------TITRLCSC---------AASANG-GNE-II  195
_XP_033169558.1_   175  ------------------------E-L-------------TITRLCSC---------AASANG-GNE-II  195
_EDX05310.1_       175  ------------------------E-L-------------TITRLCSC---------AASANG-GNE-II  195
_NP_001162998.1_   175  ------------------------E-L-------------TITRLCSC---------AATANG-GDE-II  195
_XP_032579766.1_   175  ------------------------E-L-------------TITRLCSC---------AASANG-GNE-II  195
_XP_033169541.1_   175  ------------------------E-L-------------TITRLCSC---------AASANG-GNE-II  195
_XP_034669502.1_   175  ------------------------E-L-------------TISRLCSC---------SAKVSG-GDE-II  195
_XP_034125957.1_   175  ------------------------E-L-------------TISRLCSC---------SAKVSG-GDE-II  195
_XP_034669501.1_   175  ------------------------E-L-------------TISRLCSC---------SAKVSG-GDE-II  195
_XP_034125956.1_   175  ------------------------E-L-------------TISRLCSC---------SAKVSG-GDE-II  195
_XP_034125955.1_   175  ------------------------E-L-------------TISRLCSC---------SAKVSG-GDE-II  195
_XP_017152033.1_   175  ------------------------E-L-------------TISRLCSC---------SAKVSG-GDE-II  195
_XP_017152031.1_   175  ------------------------E-L-------------TISRLCSC---------SAKVSG-GDE-II  195
_XP_002014799.1_   175  ------------------------E-L-------------TISRLCSC---------SAKVSG-GDE-II  195
_XP_026844902.1_   175  ------------------------E-L-------------TISRLCSC---------SAKVSG-GDE-II  195
_XP_015035983.2_   175  ------------------------E-L-------------TISRLCSC---------SAKVSG-GDE-II  195
_XP_017063896.1_   175  ------------------------E-L-------------TISRLCSC---------SAQVTG-GAE-II  195
_XP_016935332.1_   175  ------------------------E-L-------------TITRLCSC---------SAAVTG-GSE-II  195
_XP_017063894.2_   175  ------------------------E-L-------------TISRLCSC---------SAQVTG-GAE-II  195
_XP_036668637.1_   175  ------------------------E-L-------------TITRLCSC---------SAAVTG-GSE-II  195
_XP_016935310.1_   175  ------------------------E-L-------------TITRLCSC---------SAAVTG-GSE-II  195
_KAH8298318.1_     175  ------------------------E-L-------------TITRLCSC---------SAQVSG-GTE-II  195
```

```
_KAH8412187.1_     175  ------------------------E-L-------------TITRLCSC---------AAQVSG-GTE-II  195
_KAH8278042.1_     174  ------------------------E-L-------------TITRLCSC---------SAPASG-GTE-II  194
_KAH8366423.1_     175  ------------------------E-L-------------TITRLCSC---------SAQVTG-GSE-II  195
_XP_017044860.1_   176  ------------------------E-L-------------TICKICSC---------SAQVTG-GNT-IM  196
_KAH8324526.1_     173  ------------------------E-L-------------TIVRLCSC---------AAPASG-GTE-II  193
_XP_017116817.1_   175  ------------------------E-L-------------TINRLCSC---------SAQVTG-GSE-II  195
_XP_041566118.1_   175  ------------------------E-L--------------INRLCSC---------SAQVTG-GSE-II  194
_XP_052852584.1_   175  ------------------------E-L-------------TINRLCSC---------SAQVTG-GSE-II  195
_XP_052852582.1_   175  ------------------------E-L-------------TINRLCSC---------SAQVTG-GSE-II  195
_XP_017107132.2_   170  ------------------------E-L-------------AIVRLCSC---------SAPASG-GTG-II  190
_XP_043069650.1_   170  ------------------------E-L-------------AIVRLCSC---------SAPASG-GTG-II  190
_KAH8283158.1_     175  ------------------------E-L------------TTITRLCSC---------SAPVTG-GNE-II  196
_XP_017025640.1_   174  ------------------------E-L-------------TITRLCSC---------SAPVTG-GNE-II  194
_XP_020803071.1_   175  ------------------------E-L-------------TITRLCSC---------SAPVTG-GNE-II  195
_XP_017025638.1_   175  ------------------------E-L-------------TITRLCSC---------SAPVTG-GNE-II  195
_KAH8308067.1_     175  ------------------------E-L-------------TITRLCSC---------SAPVTG-GNE-II  195
_KAH8362606.1_     175  ------------------------E-L-------------TITRLCSC---------SAPVTG-GNE-II  195
_XP_020803068.1_   175  ------------------------E-L-------------TITRLCSC---------SAPVTG-GNE-II  195
_XP_015052364.1_   174  ------------------------E-L------------TTIIRLCSC---------AATANG-GDD-II  195
_XP_002090469.1_   174  ------------------------E-L-------------TIIRLCSC---------AATANG-GDD-II  194
_XP_039480354.1_   174  ------------------------E-L-------------TIIRLCSC---------AATANG-GDD-II  194
_XP_039480352.1_   174  ------------------------E-L-------------TIIRLCSC---------AATANG-GDD-II  194
_XP_041450795.1_   175  ------------------------E-L-------------MISRLCSC---------SAKVSG-GDE-II  195
_XP_044315461.1_   175  ------------------------E-L------------TTITRLCSC---------SAQVTG-GNE-IM  196
_XP_044315463.1_   175  ------------------------E-L-------------TITRLCSC---------SAQVTG-GNE-IM  195
_XP_001961710.2_   175  ------------------------E-L-------------TISKICTC---------SAPASG-GSE-II  195
_XP_014761890.1_   175  ------------------------E-L-------------TISKICTC---------SAPASG-GSE-II  195
_KAH8323309.1_     175  ------------------------E-L-------------TISKICTC---------SAPASG-GSE-II  195
_XP_043646206.1_   174  ------------------------D-M-------------TITRLCSC---------SATVNG-GDE-II  194
_XP_043646204.1_   174  ------------------------D-M-------------TITRLCSC---------SATVNG-GDE-II  194
_AAQ65066.1_       175  ------------------------E-L-------------TITRLCSC---------AASANG-GNE-II  195
_AAQ64900.1_       175  ------------------------E-L-------------TITRLCSC---------AASANG-GNE-II  195
_AAQ64905.1_       175  ------------------------E-L-------------TITRLCSC---------AASANG-GNE-II  195
_KAH8378209.1_     176  ------------------------D-L-------------TITKMCKC---------SSKSSG-GDE-II  196
_KAH8293207.1_     176  ------------------------D-L-------------TITRMCQC---------SSNPSG-GDE-II  196
_KAH8391978.1_     175  ------------------------D-L-------------TITKMCKC---------SSKSSG-GDE-II  195
_XP_034101352.1_   175  ------------------------D-L-------------TITKMCKC---------SSKSSG-GDE-II  195
_KAJ3658269.1_     172  ----------------------LPD-P-------------LIYRLSHCNS-----YVNGKNQN-SEQ-II  198
_KAJ3658270.1_     172  ----------------------PIE-P-------------IIYKLSHGNS-----YVDGKIHS-SEQ-II  198
```

```
_KAJ3658271.1_     172  ----------------------FFY-P-------------FIHKLSHCSS-----YVDGKNHN-SEQ-II  198
_XP_015117085.1_   175  ----------------------AVN-L-------------SISKLSTE---------VASVAG-GTS-LV  197
_XP_011307643.1_   175  ----------------------VSN-L-------------SISKLSTE---------MALAVG-GTS-LT  197
_XP_011307741.1_   175  ----------------------TLD-L-------------GICQLSSA---------VASVAG-GTP-LM  197
_XP_015119445.1_   175  ----------------------ATN-L-------------KISKLSAK---------VASTAG-GTS-LI  197
_XP_052128594.1_   186  ----------------------CTN-L-------------KILKLSHS---------SAFVDG-GKE-IL  208
_XP_052128597.1_   178  ----------------------CTN---------------KILKLSHS---------SAFVDG-GKE-IL  199
_KAE8741315.1_     132  ----------------------CTN-L-------------KILKLSHS---------SAFVDG-GKE-IL  154
_XP_028982160.1_   179  ----------------------FNV-L-------------EICRLTTH---------TGSAAG-GTE-LT  201
_XP_011307645.1_   179  ----------------------FNV-L-------------EICRLTTH---------TASMAG-GTE-LT  201
_XP_054725384.1_   170  ----------------------LLR-L-------------AIHEICC----------SGSVLG-CTE-II  191
_XP_053965385.1_   170  ----------------------LFR-L-------------AIHEICC----------SGSVLG-CTE-II  191
_XP_013149158.1_   181  ----------------------QGE-L-------------HISRLSHD---------AVPASG-GTF-VI  203
_XP_045534655.1_   181  ----------------------QGE-L-------------HITRLSHC---------TGPATG-GTT-VI  203
_KPJ06852.1_       181  ----------------------QGE-L-------------HITRLSHC---------TGPATG-GTT-VI  203
_XP_013176418.1_   181  ----------------------QGE-L-------------QIVRLSCQ---------ESPATG-GRF-VI  203
_OXU28425.1_       188  ----------------------SNR-L-------------GLIKTEDKYIAKSNNHNSASVAG-GLK-IC  219
_XP_008216913.1_   178  ----------------------TNY-L-------------NICYLSHN---------SATVAG-GLK-IC  200
_XP_008216914.1_   174  ----------------------TNY-L-------------NICYLSHN---------SATVAG-GLK-IC  196
_XP_040572301.1_   170  ----------------------HAI-P-------------KIHDISDD---------WSYTDG-GKK-II  192
_CAB4068408.1_      95  ----------------------HAI-P-------------KIHDISDD---------WSYTD--------  111
_KAF7993284.1_     136  FFILLKTILKINRIYFNEFFCVLES-P-------------VIYNFSPT---------IGSSLG-GTQ-VT  180
_KAF7987489.1_     178  ----------------------TNN-L-------------RIYRLNRC---------SAPANG-GTE-IT  200
_KAF7994520.1_     180  ----------------------SKS-L-------------RISRLNIC---------SAPASG-GKE-VT  202
_XP_044019368.1_   184  ----------------------KYK-L-------------KIYRLSRH---------SALATG-GTE-LT  206
_XP_044019332.1_    84  ----------------------TNN-L-------------RIYRLNRC---------SAPANG-GTE-IT  106
_XP_044003986.1_    53  ----------------------SKS-L-------------RISRLNIC---------SAPASG-GKE-VT   75
_XP_037042732.1_   176  ----------------------AAE-V-------------KIHDISDT---------FSPASG-GKK-IM  198
_CAD7223243.1_     170  ----------------------VSD-L-------------SITELSHC---------NSLCTG-GRR-MI  192
_XP_044732233.1_   177  ------------------------D-L-------------IIYKLSHV---------SGAVEG-NEE-IL  197
_XP_012231885.1_   170  ----------------------MPD-L-------------VICKLSHC---------SASVAG-GLD-MV  192
_XP_022814699.1    175  ----------------------STK-P-------------QIHHYAPG---------SGSAKG-GAK-VI  197
_CAB3516523.1_     175  ----------------------STK-P-------------QIHHYAPG---------SGSAKG-GAK-LL  197
_XP_035458245.2_   174  ----------------------STK-P-------------QIHHYSPG---------SGSARG-GTK-II  196
_KAF9795795.1_     163  ----------------------STK-P-------------QIHHYSPG---------SGSARG-GTK-II  185
_XP_014233257.1_   185  ----------------------KPD-L-------------TIVWLSHV---------AGPPAG-HQI-CC  207
_XP_034324400.1_   160  ----------------------GRS-FS-----------IHTIRPFAHS-------CLGSRSPNEYMLIV  188
_XP_052699907.1_   160  ----------------------GRS-FS-----------IHTIRPFAHS-------CLGSRSPNEYMLIV  188
Consensus_aa:           .......................sc.L.............Ihclsps..........stsh.G.tpp.lh
```

```
Consensus ss:                                   -              eeeeee            ee      e ee

Conservation:               5                    5                     7                   5
_XP_001652840.2_  197  LLCEKVA----------K-ED---IAVRFY-EEQH---------GNIVWEEYGEFQHT---NVHKQVAIC  239
_AAW67214.1_      197  LLCEKVA----------K-ED---IAVRFY-EEQH---------GNIVWEEYGEFQHT---NVHKQVAIC  239
_XP_021704027.1_  197  LLCEKVA----------K-ED---IAVRFY-EEQH---------GNIVWEEYGEFQHT---NVHKQVAIC  239
_XP_019540833.2_  197  LLCEKVA----------K-ED---IAVRFY-EEQH---------GNIVWEEYGEFQHT---NVHKQVAIC  239
_XP_029720231.1_  197  LLCEKVA----------K-ED---IAVRFY-EEQH---------GNIVWEEYGEFQHT---NVHKQVAIC  239
_AAW67215.1_      197  LLCEKVA----------K-ED---IAVRFY-EEQH---------GNIVWEEYGEFQHT---NVHKQVAIC  239
_XP_021704025.1_  197  LLCEKVA----------K-ED---IAVRFY-EEQH---------GNIVWEEYGEFQHT---NVHKQVAIC  239
_XP_019540831.2_  197  LLCEKVA----------K-ED---IAVRFY-EEQH---------GNIVWEEYGEFQHT---NVHKQVAIC  239
_KXJ81105.1_      197  LLCEKVA----------K-ED---IAVRFY-EEQH---------GNIVWEEYGEFQHT---NVHKQVAIC  239
_KXJ76497.1_      197  LLCEKVA----------K-ED---IAVRFY-EEQH---------GNIVWEEYGEFQHT---NVHKQVAIC  239
_XP_023240428.1_  196  MLCDKIV----------K-ND---IQIRFF-EEKN---------EEVVWEGWAEFQPC---DVYRLVAIC  238
_CAD7241875.1_    197  ILCEKVN----------K-DD---IAVKFF-EKNKA--------NEVVWEAYGDFQAS---DVHKQYAII  240
_TRY80291.1_      194  LFTEQIK----------K-DD---IEVHFT-QYTK---------GGEQIIAKGSFTAN---DVHRQYGIS  236
_ALF84899.1_      191  LFCEKVS----------K-DD---IEVHFS-YQDQ---------NSKTQVIKGHFTPN---DVHKQYGIS  233
_KAF4528208.1_    197  LLCEKVA----------K-ED---ISIRFY-EEKD---------GQLEWEGMADFQPT---DVHKQVAIG  239
_XP_054260955.1_  198  LLCEKVL----------K-ED---IAVRLY-EEKN---------GEIVWAANCEFTPA---QVHKQVAIT  240
_XP_054264401.1_  198  LLCEKVL----------K-ED---IAVRLY-EEKN---------GEIVWAANCEFTPA---QVHKQVAIT  240
_XP_054260954.1_  198  LLCEKVL----------K-ED---IAVRLY-EEKN---------GEIVWAANCEFTPA---QVHKQVAIT  240
_XP_038114042.1_  197  LLCEKVA----------K-ED---ISVRFY-EEQH---------GHILWEDVGEFQHS---NVHKQVAIS  239
_XP_039437558.1_  197  LLCEKVA----------K-ED---ISVRFY-EEQH---------GHILWEDVGEFQHS---NVHKQVAIS  239
_XP_001844078.2_  197  LLCEKVA----------K-ED---ISVRFY-EEQH---------GHILWEDVGEFQHS---NVHKQVAIS  239
_EDS35747.1_      197  LLCEKVA----------K-ED---ISVRFY-EEQH---------GHILWEDVGEFQHS---NVHKQVAIS  239
_XP_053696925.1_  197  LLCEKVA----------K-ED---ISVRFY-EEQH---------GKILWEDIGEFQHT---NVHKQVAIC  239
_XP_001869619.2_  197  ILCEKVT----------K-ED---ISVRFY-EERD---------GRIVWEDVGEFQHN---NVHKQVAIC  239
_XP_039434954.1_  197  ILCEKVT----------K-ED---ISVRFY-EERE---------GRIVWEDVGEFQHN---NVHKQVAIC  239
_EDS29879.1_      197  ILCEKVT----------K-ED---ISVRFY-EERE---------GRIVWEDVGEFQHN---NVHKQVAIC  239
_XP_019550742.2_  197  LLCEKVV----------K-ED---IAVRFY-EEQQ---------GKIVWEDHGEFPHS---NVHKQVAIS  239
_KXJ74587.1_      197  LLCEKVV----------K-ED---IAVRFY-EEQQ---------GKIVWEDHGEFPHS---NVHKQVAIS  239
_XP_035908773.1_  198  LLCEKVV----------K-ED---IKVRFF-EKKG---------NITVWENYAEFSHT---DVHKQVAIS  240
_AJC98655.1_      198  LLCEKVV----------K-ED---IKVRFF-EKKG---------NATVWENYAEFSHT---DVHKQVAIS  240
_AJC98650.1_      198  LLCEKVV----------K-ED---IKVRFF-EKKG---------NATVWENYAEFSHT---DVHKQVAIS  240
_AJC98644.1_      198  LLCEKVV----------K-ED---IKVRFF-EKKG---------NATVWENYAEFSHT---DVHKQVAIS  240
_AJC98657.1_      198  LLCEKVV----------K-ED---IKVRFF-EKKG---------NATVWENYAEFSHT---DVHKQVAIS  240
_AJC98648.1_      198  LLCEKVV----------K-ED---IKVRFF-EKKG---------NATVWENYAEFSHT---DVHKQVAIS  240
_AJC98647.1_      198  LLCEKVV----------K-ED---IKVRFF-EKKG---------NATVWENYAEFSHT---DVHKQVAIS  240
```

```
_AJC98651.1_      198  LLCEKVV----------K-ED---IKVRFF-EKKG---------NATVWENYAEFSHT---DVHKQVAIS  240
_AJC98643.1_      198  LLCEKVV----------K-ED---IKVRFF-EKKG---------NATVWENYAEFSHT---DVHKQVAIS  240
_AJC98656.1_      198  LLCEKVV----------K-ED---IKVRFF-EKKG---------NATVWENYAEFSHT---DVHKQVAIS  240
_AJC98641.1_      198  LLCEKVV----------K-ED---IKVRFF-EKKG---------NATVWENYAEFSHT---DVHKQVAIS  240
_AJC98640.1_      198  LLCEKVV----------K-ED---IKVRFF-EKKG---------NATVWENYAEFSHT---DVHKQVAIS  240
_AJC98653.1_      198  LLCEKVV----------K-ED---IKVRFF-EKKG---------NATVWENYAEFSHT---DVHKQVAIS  240
_AJC98654.1_      198  LLCEKVV----------K-ED---IKVRFF-EKKG---------NATVWENYAEFSHT---DVHKQVAIS  240
_AJC98652.1_      198  LLCEKVV----------K-ED---IKVRFF-EKKG---------NATVWENYAEFSHT---DVHKQVAIS  240
_AJC98646.1_      198  LLCEKVV----------K-ED---IKVRFF-EKKG---------NAMVWENYAEFSHT---DVHKQVAIS  240
_AJC98658.1_      198  LLCEKVV----------K-ED---IKVRFF-EKKG---------NTMVWENYAEFSHT---DVHKQVAIS  240
_AJC98639.1_      198  LLCEKVV----------K-ED---IKVRFF-EKKG---------NTTVWENYAEFSHT---DVHKQVAIS  240
_XP_035908772.1_  198  LLCEKVV----------K-ED---IKVRFF-EKKG---------NITVWENYAEFSHT---DVHKQVAIS  240
_XP_050076803.1_  198  LLCEKVV----------K-ED---IKVRFF-EKKG---------NITVWESYAEFSHS---DVHKQVAIS  240
_XP_049282441.1_  198  LLCEKVV----------K-ED---IKVRFF-EKKG---------NITVWENYAEFSHT---DVHKQVAIS  240
_XP_040170080.1_  198  LLCEKVV----------K-ED---IKVRFF-EKKG---------NATVWENYAEFSHT---DVHKQVAIS  240
_XP_050076802.1_  198  LLCEKVV----------K-ED---IKVRFF-EKKG---------NITVWESYAEFSHS---DVHKQVAIS  240
_XP_041780138.1_  198  LLCEKVV----------K-ED---IKVRFF-EKKG---------NTMVWENYAEFSHT---DVHKQVAIS  240
_XP_040239229.2_  198  LLCEKVV----------K-ED---IKVRFF-EKKG---------NATVWENYAEFSHT---DVHKQVAIS  240
_XP_040239222.2_  198  LLCEKVV----------K-ED---IKVRFF-EKKG---------NATVWENYAEFSHT---DVHKQVAIS  240
_XP_035908780.1_  198  LLCEKVV----------K-ED---IKVRFF-EKKG---------NITVWENYAEFSHT---DVHKQVAIS  240
_XP_040239221.2_  198  LLCEKVV----------K-ED---IKVRFF-EKKG---------NATVWENYAEFSHT---DVHKQVAIS  240
_XP_052897786.1_  198  LLCEKVV----------K-ED---IKVRFF-EKKG---------NITVWENYAEFSHT---DVHKQVAIS  240
_XP_052895493.1_  198  LLCEKVV----------K-ED---IKVRFF-EKKG---------NITVWENYAEFSHT---DVHKQVAIS  240
_XP_053664395.1_  198  LLCEKVV----------K-ED---IKVRFF-EKKG---------NITVWENYAEFSHT---DVHKQVAIS  240
_XP_050076801.1_  198  LLCEKVV----------K-ED---IKVRFF-EKKG---------NITVWESYAEFSHS---DVHKQVAIS  240
_XP_310177.3_     198  LLCEKVV----------K-ED---IKVRFF-EKKG---------NATVWENYAEFSHT---DVHKQVAIS  240
_XP_001652339.1_  197  LLCEKVA----------K-ED---IAVRFY-EEHQ---------GKIVWEDQGEFQHS---NVHKQVAIS  239
_XP_053686256.1_  197  LLCEKVT----------K-ED---ISVRFY-EERN---------GRIVWTDSGEFQHN---NVHKQVAIC  239
_XP_053675540.1_  198  LLCEKVV----------K-ED---IKVRFF-EKKG---------DKTVWENYAEFSHT---DVHKQVAIG  240
_ETN66814.1_      197  MLCEKVV----------K-ED---IRVRFY-ETQN---------NMLIWEGNGEFTHT---DVHKQVAIS  239
_XP_049534501.1_  197  MLCEKVV----------K-ED---IRVRFY-ETQN---------NMLIWEGNGEFTHT---DVHKQVAIS  239
_XP_050084431.1_  197  MLCEKVV----------K-ED---IRVRFY-EMQN---------NGLVWEGNGEFTHT---DVHKQVAIS  239
_XP_035782874.1_  197  MLCEKVV----------K-ED---IRVRFY-EMQN---------NGLVWEGNGEFTHT---DVHKQVAIS  239
_XP_035782869.1_  197  MLCEKVV----------K-ED---IRVRFY-EMQN---------NGLVWEGNGEFTHT---DVHKQVAIS  239
_KFB39849.1_      205  LLCEKVV----------K-ED---IRVRFF-EKKG---------NITVWQGYGEFTHT---DVHKQVAIS  247
_XP_052871375.1_  198  LLCEKVV----------K-ED---IKVRFF-EKKG---------NMTVWENY------------------  225
_AAR13743.1_      183  LLCEKVV----------K-E--------------------------------------------------  191
_AAR13744.1_      183  LLCEKVV----------K-E--------------------------------------------------  191
_CAA65157.1_      177  L---------------------------------------------------------------------  177
```

```
_XP_022170156.1_   196  LLCEKVT----------K-DD---IQVRFF-EEKD---------GVCIWEGYADFQAS---DVHKQVAIS  238
_XP_025205168.1_   196  LLCEKVT----------K-DD---IQVRFF-EEKD---------GVCTWEGYADFQPS---DVHKQVAIS  238
_XP_026817162.1_   196  LLCEKVT----------K-DD---IQVRFF-EEKD---------GVCTWEGYADFQPS---DVHKQVAIS  238
_XP_027846205.1_   196  LLCEKVT----------K-DD---IQVRFF-EEKD---------GVCTWEGYADFQPS---DVHKQVAIS  238
_XP_015370635.1_   196  LLCEKVT----------K-DD---IQVRFF-EEKD---------GVCTWEGYADFQAS---DVHKQVAIS  238
_CAH1723797.1_     196  LLCEKVT----------K-DD---IQVRFF-EEKD---------GVCTWEGYADFQPS---DVHKQVAIS  238
_KAF0769940.1_     196  LLCEKVT----------K-DD---IQIRFF-EEKD---------GICTWEGYADFQPS---DVHKQVAIS  238
_CAB4065202.1_     145  ILSEKVC----------K-DN---IEVRFT-QYAED--------GTILWHSKAEFGPS---AVHKQYAIS  188
_XP_050440903.1_   196  LLCEKVT----------K-DD---IQVRFY-EEQD---------GLCTWEGYADFQPS---DVHKQVAIS  238
_XP_050533799.1_   196  LLCEKVT----------K-DD---IQVRFF-EEQD---------GLCTWEGYADFQPS---DVHKQVAIT  238
_XP_025410632.1_   196  LLCEKVI----------K-DD---IQIRFF-EEKDD--------GTCVWEGYADFQPS---DVHKQVAIY  239
_XP_008180746.1_   196  LLCEKVT----------K-DD---IQVRFF-EEKD---------GKCIWEDYGVFKAS---DVHKQVAIW  238
_XP_026465450.1_   198  LLCEKVN----------K-DD---ITVRFF-EEQD---------GLVCWEDYGYFKFS---NVHKQTAIS  240
_VVC36026.1_       196  LLCEKVT----------K-DD---IQVRFY-EEKD---------GKCIWESYADFQPQ---DVHKQVAIY  238
_XP_016661522.1_   196  LLCDKIT----------K-DD---IQVRFF-EEKD---------GKCIWEDYGVFKAS---DVHKQVAIW  238
_XP_001947429.3_   196  LLCDKIT----------K-DD---IQVRFF-EEKD---------GKCIWEDYGVFKAS---DVHKQVAIW  238
_XP_026466007.1_   198  LLCEKVN----------K-DD---ITVRFF-EEQD---------GLVCWEDYGYFKLA----VHKQTAIS  239
_KRT81254.1_       194  LLCEKVA----------K-EN---IQIRFF-EEKN---------NEVVWEGFGSFQPS---QVHKQVAIS  236
_XP_033224832.1_   202  ILCDKVS----------K-ED---IQIRFF-EERP----------DSVWEAYADFQPT---QVHYQHAIY  243
_XP_025407590.1_   189  LLCDKVD----------K-ND---IEIRFF-EMKN---------GKIIWECVADFNPS---DVHKHTAIC  231
_XP_045476729.1_   192  ILCGKIF----------E-DD---IQVRFY-KEQL---------GVLTWEEYG--SPS---EVHRNVALA  232
_XP_034174253.1_   197  LLCEKVA----------K-ED---IQVRFF-EEKD---------GQVVWEGFGDFQPT---HVHKQTAIA  239
_XP_029047866.1_   197  LLCEKVA----------K-ED---IQVRFF-EEKD---------GQVVWEGFGDFQPT---HVHKQTAIA  239
_XP_015430648.1_   197  LLCEKVA----------K-ED---IQVRFF-EEKD---------GQMVWEGFGDFQPT---HVHKQTAIA  239
_XP_017798081.1_   197  LLCEKVA----------K-ED---IQVRFF-EEKD---------GQVVWEGLGDFQPT---HVHKQTAIA  239
_XP_017756471.1_   197  LLCEKVA----------K-ED---IQVRFF-EKKE---------GQVVWEGFGDFQPA---HVHKQTAIA  239
_XP_043248447.1_   197  LLCEKVA----------K-ED---IQVRFF-EEKD---------GQVIWEGFGDFQPT---HVHKQTAIA  239
_XP_029047855.1_   197  LLCEKVA----------K-ED---IQVRFF-EEKD---------GQVVWEGFGDFQPT---HVHKQTAIA  239
_XP_034174252.1_   197  LLCEKVA----------K-ED---IQVRFF-EEKD---------GQVVWEGFGDFQPT---HVHKQTAIA  239
_XP_031829337.1_   197  LLCEKVA----------K-ED---IQVRFF-EEKD---------GQMVWEGFGDFQPT---HVHKQTAIA  239
_KZC09125.1_       197  LLCEKVA----------K-ED---IQVRFF-EEKD---------GQMVWEGFGDFQPT---HVHKQTAIA  239
_XP_033327213.1_   197  LLCEKVA----------K-ED---IQVRFF-EEKD---------GQVVWEGLGDFQPT---HVHKQTAIA  239
_XP_033327212.1_   197  LLCEKVA----------K-ED---IQVRFF-EEKD---------GQVVWEGLGDFQPT---HVHKQTAIA  239
_XP_017756445.1_   197  LLCEKVA----------K-ED---IQVRFF-EKKE---------GQVVWEGFGDFQPA---HVHKQTAIA  239
_OAD62393.1_       197  LLCEKVA----------K-ED---IQVRFF-EKKE---------GQVVWEGFGDFQPA---HVHKQVSIA  239
_XP_043248413.1_   197  LLCEKVA----------K-ED---IQVRFF-EEKD---------GQVIWEGFGDFQPT---HVHKQTAIA  239
_XP_033327208.1_   197  LLCEKVA----------K-ED---IQVRFF-EEKD---------GQVVWEGLGDFQPT---HVHKQTAIA  239
_XP_034950477.1_   197  LLCEKVA----------K-ED---IQVRFF-EERD---------GQVVWEGFGDFQPS---HVHKQVAIA  239
_XP_034950475.1_   197  LLCEKVA----------K-ED---IQVRFF-EERD---------GQVVWEGFGDFQPS---HVHKQVAIA  239
```

```
_XP_012343274.1_  197  LLCEKVA----------K-ED---IQVRFF-EEKD---------GQVLWEGFGDFQPV---HVHKQTAIA  239
_XP_016909449.1_  197  LLCEKVA----------K-ED---IQVRFF-EEKD---------GQVLWEGFGDFQPV---HVHKQTAIA  239
_XP_012343273.1_  197  LLCEKVA----------K-ED---IQVRFF-EEKD---------GQVLWEGFGDFQPV---HVHKQTAIA  239
_XP_006619742.1_  197  LLCEKVA----------K-ED---IQVRFF-EEKD---------GQVLWEGFGDFQPV---HVHKQTAIA  239
_XP_006567065.1_  197  LLCEKVA----------K-ED---IQVRFF-EEKD---------GQVLWEGFGDFQPV---HVHKQTAIA  239
_XP_006619741.1_  197  LLCEKVA----------K-ED---IQVRFF-EEKD---------GQVLWEGFGDFQPV---HVHKQTAIA  239
_NP_001011577.1_  197  LLCEKVA----------K-ED---IQVRFF-EEKD---------GQVLWEGFGDFQPV---HVHKQTAIA  239
_XP_016909448.1_  197  LLCEKVA----------K-ED---IQVRFF-EEKD---------GQVLWEGFGDFQPV---HVHKQTAIA  239
_XP_043802365.1_  197  LLCEKVA----------K-ED---IQVRFF-EEKD---------GQVLWEGFGDFQPV---HVHKQTAIA  239
_NP_001164477.1_  197  LLCEKVA----------K-ED---IQVRFF-EEKD---------GQVLWEGFGDFQPV---HVHKQTAIA  239
_XP_016909443.1_  197  LLCEKVA----------K-ED---IQVRFF-EEKD---------GQVLWEGFGDFQPV---HVHKQTAIA  239
_XP_043802361.1_  197  LLCEKVA----------K-ED---IQVRFF-EEKD---------GQVLWEGFGDFQPV---HVHKQTAIA  239
_XP_012343269.1_  197  LLCEKVA----------K-ED---IQVRFF-EEKD---------GQVLWEGFGDFQPV---HVHKQTAIA  239
_XP_006619738.1_  197  LLCEKVA----------K-ED---IQVRFF-EEKD---------GQVLWEGFGDFQPV---HVHKQTAIA  239
_PBC29878.1_      197  LLCEKVA----------K-ED---IQVRFF-EEKD---------GQVLWEGFGDFQPV---HVHKQTAIA  239
_XP_046663984.1_  196  LLCEKVA----------K-ED---ISVRLY-EERN---------GQVLWEGMGDFTPT---QVHKQVAIT  238
_XP_046663985.1_  196  LLCEKVA----------K-ED---ISVRLY-EERN---------GQVLWEGMGDFTPT---QVHKQVAIT  238
_XP_043274771.1_  197  LLCEKVA----------K-ED---IQVKFY-EERD---------GQLVWEGYGDFQPS---QVHKQVAIA  239
_XP_043274769.1_  197  LLCEKVA----------K-ED---IQVKFY-EERD---------GQLVWEGYGDFQPS---QVHKQVAIA  239
_XP_043289571.1_  196  MLCEKVA----------K-DN---IQVRFF-EKRD---------GLEVWEGYGDFKSI---DVHKQTAIA  238
_XP_032665873.1_  197  LLCEKVA----------K-ED---IQVRFF-EEDD---------GQLTWEGFGDFQPT---HVHKQTAIA  239
_XP_032665875.1_  197  LLCEKVA----------K-ED---IQVRFF-EEDD---------GQLTWEGFGDFQPT---HVHKQTAIA  239
_XP_032665874.1_  197  LLCEKVA----------K-ED---IQVRFF-EEDD---------GQLTWEGFGDFQPT---HVHKQTAIA  239
_XP_032665869.1_  197  LLCEKVA----------K-ED---IQVRFF-EEDD---------GQLTWEGFGDFQPT---HVHKQTAIA  239
_XP_014470289.1_  197  LLCEKVA----------K-ED---IQVRFF-EEDD---------GQLLWEGFGDFQPT---HVHKQTAIA  239
_XP_011149046.1_  197  LLCEKVA----------K-ED---IQVRFF-EEDD---------GQLLWEGFGDFQPT---HVHKQTAIA  239
_XP_017877914.1_  195  LLCEKVA----------K-ED---IQVRFI-EEKD---------GQVVWEGFGDFQPT---HVHKQTAIA  237
_XP_017877911.1_  195  LLCEKVA----------K-ED---IQVRFI-EEKD---------GQVVWEGFGDFQPT---HVHKQTAIA  237
_XP_033174774.1_  198  LLCEKVA----------K-ED---IQVRFF-EEKE---------GQVIWEGFGDFQPV---HVHKQTAIA  240
_XP_043581013.1_  197  LLCEKVA----------K-ED---IQIRFF-EEKE---------GQVIWEGFGDFQPV---HVHKQTAIA  239
_XP_048261507.1_  197  LLCEKVA----------K-ED---IQVRFF-EEKE---------GQVIWEGFGDFQPV---HVHKQTAIA  239
_XP_050573946.1_  197  LLCEKVA----------K-ED---IQVRFF-EEKE---------GQVIWEGFGDFQPV---HVHKQTAIA  239
_XP_050478075.1_  197  LLCEKVA----------K-ED---IQVRFF-EEKE---------GQVIWEGFGDFQPV---HVHKQTAIA  239
_XP_033204249.1_  197  LLCEKVA----------K-ED---IQVRFF-EEKE---------GQVIWEGFGDFQPV---HVHKQTAIA  239
_XP_033358451.1_  197  LLCEKVA----------K-ED---IQVRFF-EEKE---------GQVIWEGFGDFQPV---HVHKQTAIA  239
_XP_033358450.1_  197  LLCEKVA----------K-ED---IQVRFF-EEKE---------GQVIWEGFGDFQPV---HVHKQTAIA  239
_XP_033204244.1_  197  LLCEKVA----------K-ED---IQVRFF-EEKE---------GQVIWEGFGDFQPV---HVHKQTAIA  239
_XP_033312956.1_  197  LLCEKVA----------K-ED---IQVRFF-EEKE---------GQVIWEGFGDFQPV---HVHKQTAIA  239
_XP_012236639.1_  197  LLCEKVA----------K-ED---IQVRFF-EEKE---------GQVIWEGFGDFQPV---HVHKQTAIA  239
```

```
_XP_050573940.1_  197  LLCEKVA----------K-ED---IQVRFF-EEKE---------GQVIWEGFGDFQPV---HVHKQTAIA  239
_XP_012174118.1_  197  LLCEKVA----------K-ED---IQVRFF-EEKE---------GQVIWEGFGDFQPV---HVHKQTAIA  239
_XP_043581010.1_  197  LLCEKVA----------K-ED---IQIRFF-EEKE---------GQVIWEGFGDFQPV---HVHKQTAIA  239
_EZA51108.1_      197  LLCEKVA----------K-ED---IQVRFF-EERD---------GQLYWEGYGDFQPT---HVHKQTAIA  239
_XP_011344085.1_  197  LLCEKVA----------K-ED---IQVRFF-EERD---------GQLYWEGYGDFQPT---HVHKQTAIA  239
_XP_043519475.1_  197  LLCEKVA----------K-ED---IQVRFF-EEKD---------RQVVWEGIGDFQPA---HVHKQTAIA  239
_CAD1481114.1_    197  LLCEKVA----------K-ED---IQVRFF-EEKD---------GQVVWEGIGDFQPA---HVHKQTAIA  239
_XP_043519473.1_  197  LLCEKVA----------K-ED---IQVRFF-EEKD---------RQVVWEGIGDFQPA---HVHKQTAIA  239
_KOX75360.1_      197  LLCEKVA----------K-ED---IQVRFF-EEKD---------GQVVWEGIGDFQPA---HVHKQTAIA  239
_KAF3423741.1_    197  LLCEKVA----------K-ED---IQVRFF-EEKD---------RQVVWEGIGDFQPA---HVHKQTAIA  239
_KOC69050.1_      197  LLCEKVA----------K-ED---IQVRFF-EEKD---------GQVVWEGLGDFQPT---HVHKQTAIA  239
_XP_053980095.1_  197  LLCEKVA----------K-ED---IQVRFF-EEKD---------GKLCWEGLGDFQPA---HVHKQTAIA  239
_XP_054002893.1_  197  LLCEKVA----------K-ED---IQVRFF-EEKD---------GKLCWEGLGDFQPA---HVHKQTAIA  239
_XP_053980091.1_  197  LLCEKVA----------K-ED---IQVRFF-EEKD---------GKLCWEGLGDFQPA---HVHKQTAIA  239
_XP_028049144.1_  197  LLCEKVA----------K-ED---IQVRFF-EERD---------GQLVWEGYGDFQPS---HVHKQTAIA  239
_XP_012541458.1_  197  LLCEKVA----------K-ED---IQVRFF-EERD---------GQLVWEGYGDFQPS---HVHKQTAIA  239
_XP_003708412.1_  197  LLCEKVA----------K-ED---IQVRFF-EEKD---------GQLVWEGFGDFQPT---HVHKQTAIA  239
_XP_012152807.1_  197  LLCEKVA----------K-ED---IQVRFF-EEKD---------GQLVWEGFGDFQPT---HVHKQTAIA  239
_KAH0945556.1_    197  LLCEKIA----------K-ED---IQVRFF-EERD---------GQLCWEGYGDFQPT---HVHKQTAIT  239
_XP_024871832.1_  196  LLCEKVA----------K-ED---IQVRFF-EERD---------GQLLWEGYGDFQPT---HVHKQTAIA  238
_XP_011307641.1_  195  LLCEKIA----------K-ED---IQVRFF-EEKD---------DKVVWEGLADFQPA---NVHKQFAIA  237
_XP_015118260.1_  196  LLCEKIA----------K-ED---IQVRFF-EEKD---------DKVVWEGLADFQPA---NVHKQFAIA  238
_XP_011307640.1_  196  LLCEKIA----------K-ED---IQVRFF-EEKD---------DKVVWEGLADFQPA---NVHKQFAIA  238
_XP_015118252.1_  196  LLCEKIA----------K-ED---IQVRFF-EEKD---------DKVVWEGLADFQPA---NVHKQFAIA  238
_EFN68841.1_      201  LLCEKVA----------K-ED---IQVRFF-EERD---------GQLLWEGYGDFQPT---HVHKQTAIA  243
_XP_011647340.1_  197  LLCEKVA----------K-ED---IQVRFF-EERD---------GQLLWEGYGDFQPT---HVHKQTAIA  239
_XP_029166122.1_  201  LLCEKVA----------K-ED---IQVRFF-EERD---------GQLLWEGYGDFQPT---HVHKQTAIA  243
_KMQ96453.1_      201  LLCEKVA----------K-ED---IQVRFF-EERD---------GQLLWEGYGDFQPT---HVHKQTAIA  243
_XP_050464492.1_  201  LLCEKVA----------K-ED---IQVRFF-EERD---------GQLLWEGYGDFQPT---HVHKQTAIA  243
_XP_029673315.1_  201  LLCEKVA----------K-ED---IQVRFF-EERD---------GQLLWEGYGDFQPT---HVHKQTAIA  243
_XP_025265518.1_  201  LLCEKVA----------K-ED---IQVRFF-EERD---------GQLLWEGYGDFQPT---HVHKQTAIA  243
_XP_011255523.1_  201  LLCEKVA----------K-ED---IQVRFF-EERD---------GQLLWEGYGDFQPT---HVHKQTAIA  243
_XP_011698133.1_  197  LLCEKVA----------K-ED---IQVRFF-EERN---------GQLVWEGYGDFQPT---HVHKQTAIA  239
_XP_011698131.1_  197  LLCEKVA----------K-ED---IQVRFF-EERN---------GQLVWEGYGDFQPT---HVHKQTAIA  239
_XP_018357420.1_  197  LLCEKVA----------K-ED---IQVRFF-EERD---------GQLIWEGYGDFQPT---HVHKQTAIA  239
_KYM76140.1_      197  LLCEKVA----------K-ED---IQVRFF-EERD---------GQLIWEGYGDFQPT---HVHKQTAIA  239
_XP_011647339.1_  197  LLCEKVA----------K-ED---IQVRFF-EERD---------GQLLWEGYGDFQPT---HVHKQTAIA  239
_KAG5315434.1_    197  LLCEKVA----------K-ED---IQVRFF-EERD---------GQLIWEGYGDFQPT---HVHKQTAIA  239
_XP_011055463.1_  197  LLCEKVA----------K-ED---IQVRFF-EERD---------GQLIWEGYGDFQPT---HVHKQTAIA  239
```

```
_XP_018342550.1_  197  LLCEKVA----------K-ED---IQVRFF-EERD---------GQLIWEGYGDFQPT---HVHKQTAIA  239
_XP_018403565.1_  197  LLCEKVA----------K-ED---IQVRFF-EERD---------GQLIWEGYGDFQPT---HVHKQTAIA  239
_KAG5322912.1_    197  LLCEKVA----------K-ED---IQVRFF-EERD---------GQLIWEGYGDFQPT---HVHKQTAIA  239
_XP_018357417.1_  197  LLCEKVA----------K-ED---IQVRFF-EERD---------GQLIWEGYGDFQPT---HVHKQTAIA  239
_XP_018314560.1_  197  LLCEKVA----------K-ED---IQVRFF-EERD---------GQLIWEGYGDFQPT---HVHKQTAIA  239
_XP_018057217.1_  197  LLCEKVA----------K-ED---IQVRFF-EERD---------GQLIWEGYGDFQPT---HVHKQTAIA  239
_KAG5346558.1_    197  LLCEKVA----------K-ED---IQVRFF-EERD---------GQLIWEGYGDFQPT---HVHKQTAIA  239
_XP_047348408.1_  198  LLCEKVA----------K-ED---IQIRFF-EEKD---------GHVTWEGFGDFQPS---QVHKQTAIA  240
_XP_047348412.1_  197  LLCEKVA----------K-ED---IQIRFF-EEKD---------GHVTWEGFGDFQPS---QVHKQTAIA  239
_XP_046820611.1_  197  LLCEKVA----------K-ED---IQIRFF-EEKD---------GHVTWEGFGDFQPS---QVHKQTAIA  239
_XP_050848254.1_  197  LLCEKVA----------K-ED---IQIRFF-EEKD---------GHVTWEGFGDFQPS---QVHKQTAIA  239
_XP_046820612.1_  197  LLCEKVA----------K-ED---IQIRFF-EEKD---------GHVTWEGFGDFQPS---QVHKQTAIA  239
_XP_050848252.1_  197  LLCEKVA----------K-ED---IQIRFF-EEKD---------GHVTWEGFGDFQPS---QVHKQTAIA  239
_XP_043667564.1_  197  LLCEKVA----------K-ED---IQIRFF-EEKD---------GHVTWEGFGDFQPS---QVHKQTAIA  239
_XP_035725337.1_  197  LLCEKVA----------K-ED---IQIRFF-EEKD---------GHVTWEGFGDFQPS---QVHKQTAIA  239
_XP_043667562.1_  197  LLCEKVA----------K-ED---IQIRFF-EEKD---------GHVTWEGFGDFQPS---QVHKQTAIA  239
_XP_046820605.1_  197  LLCEKVA----------K-ED---IQIRFF-EEKD---------GHVTWEGFGDFQPS---QVHKQTAIA  239
_XP_050848249.1_  197  LLCEKVA----------K-ED---IQIRFF-EEKD---------GHVTWEGFGDFQPS---QVHKQTAIA  239
_XP_043667560.1_  197  LLCEKVA----------K-ED---IQIRFF-EEKD---------GHVTWEGFGDFQPS---QVHKQTAIA  239
_XP_035725333.1_  197  LLCEKVA----------K-ED---IQIRFF-EEKD---------GHVTWEGFGDFQPS---QVHKQTAIA  239
_KAF7405659.1_    197  LLCEKVA----------K-ED---IQIRFF-EEKD---------GHVTWEGFGDFQPS---QVHKQTAIA  239
_KAF7402294.1_    197  LLCEKVA----------K-ED---IQIRFF-EEKD---------GHVTWEGFGDFQPS---QVHKQTAIA  239
_XP_015172825.1_  197  LLCEKVA----------K-ED---IQVRFF-EEKN---------GHVIWEGYGDFAPT---DVHKQTAIV  239
_XP_015172824.1_  197  LLCEKVA----------K-ED---IQVRFF-EEKN---------GHVIWEGYGDFAPT---DVHKQTAIV  239
_XP_015172823.1_  197  LLCEKVA----------K-ED---IQVRFF-EEKN---------GHVIWEGYGDFAPT---DVHKQTAIV  239
_XP_014609306.1_  197  LLCEKVA----------K-ED---IQVRFF-EEKN---------GHVFWEGYGDFAPT---DVHKQTAIV  239
_XP_043491219.1_  197  LLCEKVA----------K-ED---IQVRFF-EEKN---------GHVFWEGYGDFAPT---DVHKQTAIV  239
_XP_014609305.1_  197  LLCEKVA----------K-ED---IQVRFF-EEKN---------GHVFWEGYGDFAPT---DVHKQTAIV  239
_XP_043491218.1_  197  LLCEKVA----------K-ED---IQVRFF-EEKN---------GHVFWEGYGDFAPT---DVHKQTAIV  239
_KAI4498556.1_    197  LLCEKVA----------K-ED---IQVRFF-EEKN---------GHVIWEGYGDFAPT---DVHKQTAIV  239
_XP_015172818.1_  197  LLCEKVA----------K-ED---IQVRFF-EEKN---------GHVIWEGYGDFAPT---DVHKQTAIV  239
_XP_043491211.1_  197  LLCEKVA----------K-ED---IQVRFF-EEKN---------GHVFWEGYGDFAPT---DVHKQTAIV  239
_KAI4495142.1_    197  LLCEKVA----------K-ED---IQVRFF-EEKN---------GHVFWEGYGDFAPT---DVHKQTAIV  239
_XP_014609300.1_  197  LLCEKVA----------K-ED---IQVRFF-EEKN---------GHVFWEGYGDFAPT---DVHKQTAIV  239
_ADK91833.1_      197  LLCEKVA----------K-ED---IQVRFF-EEKE---------GQVIWEGFGDFQPV---HVHKQTAIA  239
_KAG7213837.1_    197  LLCEKVA----------K-ED---IQVRFF-EEKD---------GQILWEGFADFQPT---HVHKQTAIA  239
_XP_014226628.1_  196  ILCERVN----------K-DD---IQIVFY-EESN---------GKVIWEGLGDFTPP---QVHKQVGIS  238
_XP_020287946.1_  197  LLCEKVA----------K-ED---IQVRFF-EERD---------GQLVWEGYGDFQPT---HVHKQTAIA  239
_XP_012232007.1_  197  LLCEKVA----------K-ED---IKVRFF-EERD---------GQLVWEGFGEFQPT---HVHKQTAIA  239
```

```
_XP_011874538.1_  197  LLCEKVA----------K-ED---IQVRFF-EMKD---------GQLLWEGYGDFQPT---HVHKQTAIS  239
_XP_011874541.1_  197  LLCEKVA----------K-ED---IQVRFF-EMKD---------GQLLWEGYGDFQPT---HVHKQTAIS  239
_XP_011173619.1_  197  LLCEKVA----------K-ED---IQVRFF-EEKD---------GQLIWEGYGDFQPT---HVHKQTAIA  239
_XP_034950793.1_  194  LLCEKVV----------K-DN---IQIVFF-EMKN---------DKMVWRDFGVFTPS---DVHKQTAIA  236
_XP_034950792.1_  190  LLCEKVV----------K-DN---IQIVFF-EMKN---------DKMVWRDFGVFTPS---DVHKQTAIA  232
_XP_012340880.2_  194  LLCEKVV----------K-DD---IQIRFF-EEKN---------GKVVWEGFGDFQPA---HVHKQTAIR  236
_XP_031771859.1_  194  LLCEKVV----------K-DD---IQIRFF-EEKN---------GKVVWEGFGDFQPA---HVHKQVSIR  236
_XP_006618953.1_  196  LLCEKVV----------K-DD---IQVRFF-EEKN---------GKVVWEGFGDFHPA---HVHKQTAIR  238
_XP_031367904.1_  196  LLCEKVV----------K-DD---IQVRFF-EEKN---------GKVVWEGFGDFHPA---HVHKQVSIR  238
_XP_043795948.1_  196  LLCEKVV----------K-DD---IQVRFF-EEKN---------GKVVWEGFGDFHPA---HVHKQTAIR  238
_XP_395180.5_     196  LLCEKVV----------K-DD---IQVRFF-EEKN---------GKVVWEAFGDFHPA---HVHKQTAIR  238
_KAG6799500.1_    196  LLCEKVV----------K-DD---IQVRFF-EEKN---------GKVVWEAFGDFHPA---HVHKQVSIR  238
_XP_016768607.2_  196  LLCEKVV----------K-DD---IQVRFF-EEKN---------GKVVWEAFGDFHPA---HVHKQVSIR  238
_PBC31989.1_      205  LLCEKVV----------K-DD---IQVRFF-EEKN---------GKVVWEGFGDFHPA---HVHKQTAIR  247
_XP_016914450.1_  196  LLCEKVV----------K-DD---IQVRFF-EEKN---------GKVVWEGFGDFHPA---HVHKQTAIR  238
_KAG5344958.1_    194  LLCEKVA----------K-ED---IQVRFF-EERD---------GQLIWEGYGDFQPT---HVHKQTAIA  236
_KAJ3630188.1_    196  LLCTRVI----------K-ED---IKVRFF-ETKD---------GQVVWEGFGDFKPS---EVYNQTAIC  238
_QHN75199.1_      196  LLCTRVI----------K-ED---IKVRFF-ETKD---------GQVVWEGFGDFKPS---EVYNQTAIC  238
_XP_051171660.1_  202  LLCDKVP----------R-DD---IQIRFY-EERD----------GRDWEGYGEFQPA---HVHHQYAIS  243
_XP_016909447.1_  155  LLCEKVA----------K-ED---IQVRFF-EEKD---------GQVLWEGFGDFQPV---HVHKQTAIA  197
_XP_031773030.1_  155  LLCEKVA----------K-ED---IQVRFF-EEKD---------GQVLWEGFGDFQPV---HVHKQTAIA  197
_KAH0956427.1_    202  LLCDKVA----------K-HD---IQIKFF-EVQN---------GVRCWEAYAAFSPL-------DTAIT  240
_XP_012283784.1_  197  LLCEKVA----------K-ED---IQVRFF-EERD---------GQVIWEGFGDFQPT---HVHKQVAIA  239
_XP_023224928.1_  196  LLCEKVN----------K--D---VQIRFF-EKKD---------GQEIWSANAEFQPS---DIHKQVAIS  237
_XP_008556434.1_  197  LLCEKVA----------K-ED---IQIRFF-EERD---------GQFVWEGFGDFQPS---HVHKQVAIA  239
_XP_008556433.1_  197  LLCEKVA----------K-ED---IQIRFF-EERD---------GQFVWEGFGDFQPS---HVHKQVAIA  239
_XP_014299728.1_  197  LLCEKVA----------K-ED---IQIRFF-EERD---------GQFVWEGFGDFQPS---HVHKQVAIA  239
_CAD6234449.1_    197  LLCEKVA----------K-ED---IQVRFT-EERD---------GQIVWEGFGDFQPS---HVHKQVAIS  239
_KAG8037552.1_    197  LLCEKVA----------K-ED---IQIRFT-EERD---------GQIVWEGFGDFQPS---HVHKQVAIS  239
_XP_044576138.1_  197  LLCEKVA----------K-ED---IQIRFT-EERD---------GQIVWEGFGDFQPS---HVHKQVAIS  239
_XP_044576125.1_  197  LLCEKVA----------K-ED---IQIRFT-EERD---------GQIVWEGFGDFQPS---HVHKQVAIS  239
_XP_026465448.1_  195  LLCEKVA----------K-ED---IQVRLF-EERD---------GQLVWEGFADFQHT---DVHKQVAIT  237
_XP_026465446.1_  195  LLCEKVA----------K-ED---IQVRLF-EERD---------GQLVWEGFADFQHT---DVHKQVAIT  237
_XP_031785072.1_  197  LLCEKVA----------K-ED---IEVRFF-EERD---------GQTCWEGKGDFQPT---HVHKQVAIS  239
_XP_015607185.1_  197  LFCEKVA----------K-ED---IQVRFF-EERD---------GQLVWEGFGDFQPT---HVHKQVGIA  239
_XP_011496611.1_  197  LLCEKVA----------K-ED---IQVRFF-EEQD---------GHTYWEGFGDFQPT---HVHKQVAIA  239
_XP_018903707.1_  193  LTCEKVT----------R-DD---IEIWIY-EKNSS--------GEIVWEEKTEFQPQ---DVHHQYAIA  236
_CAB3365562.1_    196  LLCEKVA----------K-DD---IDIHFT-EEKD---------GQIVWHGKGEFQPS---DVHRQYAIP  238
_XP_011496447.1_   67  VLCEKVS----------K-LD---IEVRFF-HEES------------NWEAIAILTKP---EVFRQVAIV  106
```

```
_KAG8230996.1_     196  LLCEKVA----------K-ED---IQVRFY-EEKD---------GQIVWEGFGDFQPT---HVHKQVAIS  238
_XP_040567422.1_   193  ILSEKVC----------K-DN---IEVRFT-QYAED--------GTILWHSKAEFGPS---AVHKQYAIS  236
_XP_014250205.1_   197  LLCEKVA----------K-ED---ISVRFY-EEND---------GVVVWEGLGDFTPT---QVHKQVAIA  239
_XP_014250206.1_   197  LLCEKVA----------K-ED---ISVRFY-EEND---------GVVVWEGLGDFTPT---QVHKQVAIA  239
_XP_047119528.1_   197  LLCEKVA----------K-ED---IQVRFF-EEQG---------GQVMWEGFGEFQPT---QVHKQVAIC  239
_XP_047001845.1_   197  LLCEKVA----------K-ED---IQVRFF-EEQG---------GQVMWEGFGEFQPT---QVHKQVAIC  239
_XP_049939433.1_   197  LLCEKVA----------K-ED---IQVRFF-EEQG---------GQVMWEGFGEFQPT---QVHKQVAIC  239
_XP_049764197.1_   197  LLCEKVA----------K-ED---IQVRFF-EEQG---------GQVMWEGFGEFQPT---QVHKQVAIC  239
_XP_049833656.1_   197  LLCEKVA----------K-ED---IQVRFF-EEQG---------GQVLWEGFGEFQPT---QVHKQVAIC  239
_XP_049790026.1_   197  LLCEKVA----------K-ED---IQVRFF-EEQG---------GQVMWEGFGEFQPT---QVHKQVAIC  239
_XP_049833651.1_   197  LLCEKVA----------K-ED---IQVRFF-EEQG---------GQVLWEGFGEFQPT---QVHKQVAIC  239
_XP_047001842.1_   197  LLCEKVA----------K-ED---IQVRFF-EEQG---------GQVMWEGFGEFQPT---QVHKQVAIC  239
_XP_049790025.1_   197  LLCEKVA----------K-ED---IQVRFF-EEQG---------GQVMWEGFGEFQPT---QVHKQVAIC  239
_XP_049833654.1_   197  LLCEKVA----------K-ED---IQVRFF-EEQG---------GQVLWEGFGEFQPT---QVHKQVAIC  239
_XP_049833659.1_   197  LLCEKVA----------K-ED---IQVRFF-EEQG---------GQVLWEGFGEFQPT---QVHKQVAIC  239
_XP_021925503.1_   197  LLCEKVT----------K-ED---IQLHFY-EERD---------GNILWEGFGDFQPT---HVHKQVAIS  239
_KDR23857.1_       197  LLCEKVT----------K-ED---IQLHFY-EERD---------GNILWEGFGDFQPT---HVHKQVAIS  239
_ABU96700.1_       197  LLCEKVA----------K-ED---ISVRFY-EEKD---------GQVVWEGLGDFTPT---QVHKQVAIS  239
_ABU96699.1_       197  LLCEKVA----------K-ED---ISVRFY-EEKD---------GQVVWEGLGDFTPT---QVHKQVAIS  239
_XP_014275495.1_   197  LLCEKVA----------K-ED---ISVRFF-EEKD---------GKIFWEGYGDFTPT---QVHKQTAIA  239
_BBE08137.1_       197  LLCEKVA----------K-ED---ISVRFF-EEKD---------GKIFWEGYGDFTPT---QVHKQTAIA  239
_XP_014275494.1_   197  LLCEKVA----------K-ED---ISVRFF-EEKD---------GKIFWEGYGDFTPT---QVHKQTAIA  239
_XP_014275493.1_   197  LLCEKVA----------K-ED---ISVRFF-EEKD---------GKIFWEGYGDFTPT---QVHKQTAIA  239
_BBE08136.1_       197  LLCEKVA----------K-ED---ISVRFF-EEKD---------GKIFWEGYGDFTPT---QVHKQTAIA  239
_KAF6207519.1_     197  LLCEKVA----------K-ED---ISVRFF-EEKD---------GIIVWEGYGDFTPT---QVHKQVAIS  239
_CAB0015508.1_     197  LLCEKVA----------K-ED---ISVRFF-EEKD---------GIVVWEGYGDFTPT---QVHKQVAIA  239
_CAB0015504.1_     197  LLCEKVA----------K-ED---ISVRFF-EEKD---------GIVVWEGYGDFTPT---QVHKQVAIA  239
_PNF18474.1_       197  LLCEKVA----------K-ED---IQIHFY-EERD---------GNIVWEGFGDFQPT---HVHKQVAIS  239
_CEG62431.1_       197  LLCEKVA----------K-ED---IQVYFY-EEKD---------GNEVWNGLADFQPT---HVHKQVAIS  239
_PSN36690.1_       197  LLCEKVA----------K-ED---IQVYFY-EEKD---------GNEVWNGLADFQPT---HVHKQVAIS  239
_XP_046387412.1_   197  LLCEKVA----------K-ED---IQIRFY-EESD---------GQLVWEGFGDFQPT---HVHKQVAIS  239
_XP_046387414.1_   197  LLCEKVA----------K-ED---IQIRFY-EESD---------GQLVWEGFGDFQPT---HVHKQVAIS  239
_XP_031634874.1_   197  LLCEKVS----------K-ED---IQVRFY-EENQ---------GVILWEGFGEFQHT---NVHKQVAIS  239
_GFG29240.1_       197  LLCEKVA----------K-ED---IQIHFF-EEQD---------GNMVWEGFGDFQPT---HVHKQVAIS  239
_BBG28464.1_       197  LLCEKVA----------K-ED---IQVRFF-TEED---------GRITWEGLGDFTPA---SVHKQVAIS  239
_WCL52334.1_       197  LLCEKVA----------K-ED---IQVRFF-TEED---------GRITWEGLGDFTPA---SVHKQVAIS  239
_GLH00135.1_       197  LLCEKVA----------K-ED---IQVRFF-TEED---------GRITWEGLGDFTPA---SVHKQVAIS  239
_CAD7205239.1_     198  LLCEKVA----------K-ED---IQVRFY-EERE---------GHMVWEGFADFQPT---HVHKQVAIT  240
_CAD7431383.1_     197  LLCEKVA----------K-ED---IQVRFY-EERD---------GHMVWEGFADFQPT---HVHKQVAIT  239
```

```
_CAD7410836.1_     197  LLCEKVA----------K-ED---IQVRFY-EERE---------GHMVWEGFADFQPT---HVHKQVAIT  239
_CAD7415345.1_     197  LLCEKVA----------K-ED---IQVRFY-EERD---------GHMVWEGFADFQPT---HVHKQVAIT  239
_CAD7606635.1_     197  LLCEKVA----------K-ED---IQVRFY-EERE---------GHMVWEGFADFQPT---HVHKQVAIT  239
_CAD7462957.1_     197  LLCEKVA----------K-ED---IQVRFY-EERE---------GHMVWEGFADFQPT---HVHKQVAIT  239
_XP_012265400.2_   196  LLCEKVA----------K-ED---IQIRFF-EERD---------GQFVWEGFADFQPT---HVHKQVAIS  238
_XP_046473587.1_   197  LLCEKVA----------K-ED---IKVRFF-EERD---------GQVLWEGFADFQPM---NVHKQVAIT  239
_XP_046611732.1_   197  LLCEKVA----------K-ED---IKVRFF-EERD---------GQVLWEGFADFQPM---NVHKQVAIT  239
_XP_015519750.2_   197  LLCEKVA----------K-ED---IKVRFF-EERD---------GQVLWEGFADFQPM---NVHKQVAIT  239
_XP_046611731.1_   197  LLCEKVA----------K-ED---IKVRFF-EERD---------GQVLWEGFADFQPM---NVHKQVAIT  239
_XP_046417903.1_   197  LLCEKVA----------K-ED---IKVRFF-EERD---------GQVLWEGFADFQPM---NVHKQVAIT  239
_GLG94364.1_       196  LLCEKVA----------K-ED---IQVRFF-EQDHG--------GRTLWEAQGDFTPA---HVHKQVAIS  239
_GLG94363.1_       196  LLCEKVA----------K-ED---IQVRFF-EQDHG--------GRTLWEAQGDFTPA---HVHKQVAIS  239
_BBG28463.1_       196  LLCEKVA----------K-ED---IQVRFF-EQDHG--------GRTLWEAQGDFTPA---HVHKQVAIS  239
_WCL52335.1_       196  LLCEKVA----------K-ED---IQVRFF-EQDHA--------GRTLWEAQGDFTPA---HVHKQVAIS  239
_GLG94365.1_       196  LLCEKVA----------K-ED---IQVRFF-EQDHG--------GRTLWEAQGDFTPA---HVHKQVAIS  239
_XP_002431518.1_   195  LLCEKVA----------K-ED---IQIRFY-EERD---------GDLFWEGLGDFQPT---QVHKQVAIS  237
_KAE8746525.1_     197  LLCEKVA----------K-ED---IQIRFY-EERD---------NQVKWEAYADFQPS---QVHKQVAIS  239
_XP_026277770.1_   197  LLCEKVA----------K-ED---IQIRFY-EERD---------NQVKWEAYADFQPS---QVHKQVAIS  239
_KAJ1519446.1_     197  LLCEKVS----------K-DD---IEIRFY-EERD---------NLVVWEAKADFQPS---QVHKQVAIS  239
_ALC78349.1_       196  LLCEKVA----------K-ED---IQVRFY-EEKD---------AQVVWEAYGDFQHT---NVHKQVAIS  238
_XP_046738772.1_   197  LLCEKVA----------K-ED---IKVRFF-EERD---------GQVFWEGFADFQPI---NVHKQVAIT  239
_XP_046417904.1_   195  LLCEKVA----------K-ED---IKVRFF-EERD---------GQVLWEGFADFQPM---NVHKQVAIT  237
_CRK94170.1_       197  LLCEKVA----------K-ED---IGIRFY-EEA-----------ADGWEGFGEFQHS---QVHKQVAIS  237
_CRK94167.1_       197  LLCEKVA----------K-ED---IGIRFY-EEA-----------ADGWEGFGEFQHS---QVHKQVAIS  237
_KAG5676160.1_     197  LLCEKVA----------K-ED---IQIRFY-EESMM-------PEQEPWEGYGEFQHS---QVHKQVAIS  241
_XP_034245464.1_   198  LLCEKVA----------K-ED---IQVRFF-EERD---------NEVKWEAYANFQPS---QVHKQVAIT  240
_XP_034245457.1_   198  LLCEKVA----------K-ED---IQVRFF-EERD---------NEVKWEAYANFQPS---QVHKQVAIT  240
_KAG8287980.1_     172  --------------------D---ISVRLY-EERN---------GQVLWEGMGDFTPT---QVHKQVAIT  205
_XP_044759045.1_   192  ILCGKVA----------K-DD---VQVRFY-EEKN---------GVLVWEDYGMFTSS---QIHRQVAIS  234
_XP_017305229.1_   191  ILCDKVN----------K-DD---IEVRFY-EEQD---------GVVVWD--ERVKTL---EVHKQYAIV  231
_XP_026675878.1_   191  ILCDKVN----------K-DD---IEVRFY-EEQD---------GVVVWD--ERVKTL---EVHKQYAIV  231
_KAE8574083.1_     149  LLCEKVA----------K-ED---ISVRFF-EEKD---------GKIFWEGYGDFTPT---QVHKQTAIA  191
_XP_037926497.1_   197  LLCEKVA----------K-ED---IAVRFF-EEKD---------GQTVWEAYGEFQHT---DVHKQTAIN  239
_CAD7079567.1_     197  LLCEKVA----------K-ED---IAVRFF-EEKD---------GQTVWEAYGEFQHT---DVHKQTAIN  239
_XP_037882485.1_   197  LLCEKVA----------K-ED---ISIRFF-EEKD---------EQVIWEGYGDFQHT---DVHKQTAIT  239
_XP_037037597.1_   197  LLCEKVA----------K-ED---ISVRFF-EEID---------DNIVWEGYGDFQHT---NVHKQVAIW  239
_XP_037037592.1_   197  LLCEKVA----------K-ED---ISVRFF-EEID---------DNIVWEGYGDFQHT---NVHKQVAIW  239
_KAG4075389.1_     197  LLCEKVA----------K-ED---ISVRFF-EEID---------DNIVWEGYGDFQHT---NVHKQVAIW  239
_XP_034101357.1_   197  LLCEKVA----------K-ED---ITVRFY-EEKN---------GQMVWEALGDFQHT---DVHKQTAIT  239
```

```
_XP_026844963.1_  197  LLCEKVA----------K-ED---ISVRFF-EEKN---------GQSVWEAFGDFQHT---DVHKQTAIT  239
_EDW28798.1_      197  LLCEKVA----------K-ED---ISVRFF-EEKN---------GQSVWEAFGDFQHT---DVHKQTAIT  239
_XP_034125966.1_  197  LLCEKVA----------K-ED---ISVRFF-EEKN---------GQSVWEAFGDFQHT---DVHKQTAIT  239
_XP_034666519.1_  197  LLCEKVA----------K-ED---ISVRFF-EEKN---------GQSVWEAFGDFQHT---DVHKQTAIT  239
_XP_001356570.3_  197  LLCEKVA----------K-ED---ISVRFF-EEKN---------GQSVWEAFGDFQHT---DVHKQTAIT  239
_XP_023164577.2_  197  LLCEKVA----------K-ED---ITVRFF-EEKN---------GQTVWEALGDFQHT---DVHKQTAIT  239
_XP_017153206.1_  197  LLCEKVA----------K-ED---ISVRFF-EEKN---------GQSVWEAFGDFQHT---DVHKQTAIT  239
_XP_030564932.1_  197  LLCEKVA----------K-ED---ITVRFF-EEKN---------GQTVWEALGDFQHT---DVHKQTAIT  239
_XP_002059145.1_  197  LLCEKVA----------K-ED---ITVRFF-EEKN---------GQTVWEALGDFQHT---DVHKQTAIT  239
_EDW04266.1_      197  LLCEKVA----------K-ED---ITVRFF-EERN---------GQTAWEALGDFQHT---DVHKQTAIT  239
_XP_017025862.1_  197  LLCEKVA----------K-ED---ISVRFF-EEKN---------GQTVWEAFGDFQHT---DVHKQTAIT  239
_XP_017859907.1_  197  LLCEKVA----------K-ED---ITVRFF-EEKN---------GQTVWEALGDFQHT---DVHKQTAIT  239
_XP_032584327.1_  197  LLCEKVA----------K-ED---ITVRFF-EEKN---------GQTVWEALGDFQHT---DVHKQTAIT  239
_XP_016987288.2_  197  LLCEKVA----------K-ED---ITVRFF-EEKN---------GQCVWEALGEFQHT---DVHKQTAIT  239
_XP_017954173.1_  197  LLCEKVA----------K-ED---ITVRFF-EEKN---------GQTVWEALGDFQHT---DVHKQTAIT  239
_XP_017854504.1_  197  LLCEKVA----------K-ED---ITVRFF-EEKN---------GQTVWEALGDFQHT---DVHKQTAIT  239
_XP_034125965.1_  197  LLCEKVA----------K-ED---ISVRFF-EEKN---------GQSVWEAFGDFQHT---DVHKQTAIT  239
_XP_034666515.1_  197  LLCEKVA----------K-ED---ISVRFF-EEKN---------GQSVWEAFGDFQHT---DVHKQTAIT  239
_XP_043641917.1_  197  LLCEKVA----------K-ED---IAVRFF-EEKN---------GQSVWEAFGDFQHT---DVHKQTAIT  239
_XP_026844960.1_  197  LLCEKVA----------K-ED---ISVRFF-EEKN---------GQSVWEAFGDFQHT---DVHKQTAIT  239
_XP_015035988.2_  197  LLCEKVA----------K-ED---ISVRFF-EEKN---------GQSVWEAFGDFQHT---DVHKQTAIT  239
_XP_041450744.1_  197  LLCEKVA----------K-ED---ISVRFF-EEKN---------GQSVWEAFGDFQHT---DVHKQTAIT  239
_XP_015052361.1_  197  LLCEKVA----------K-ED---IAVRFF-EEKN---------GQSVWEAFGDFQHT---DVHKQTAIT  239
_XP_039480515.1_  197  LLCEKVA----------K-ED---IAVRFF-EEKN---------GQSVWEAFGDFQHT---DVHKQTAIT  239
_XP_033248563.1_  197  LLCEKVA----------K-ED---ISVRFF-EEKN---------GQSVWEAFGDFQHT---DVHKQTAIT  239
_KAH8412150.1_    197  LLCEKVA----------K-ED---ISVRFF-EEKN---------GQSVWEAFGDFQHT---DVHKQTAIT  239
_XP_043864974.1_  197  LLCEKVA----------K-ED---ITVRFF-EEKN---------GQTVWEALGDFQHT---DVHKQTAIT  239
_XP_020809119.1_  197  LLCEKVA----------K-ED---ISVRFF-EEKN---------GQTVWEAFGDFQHT---DVHKQTAIT  239
_KAH8298353.1_    197  LLCEKVA----------K-ED---ISVRFF-EEKN---------GQSVWEAFGDFQHT---DVHKQTAIT  239
_XP_030564927.1_  197  LLCEKVA----------K-ED---ITVRFF-EEKN---------GQTVWEALGDFQHT---DVHKQTAIT  239
_XP_041631592.1_  197  LLCEKVA----------K-ED---ISVRFF-EEKN---------GQTVWEAFGDFQHT---DVHKQTAIT  239
_XP_017859909.1_  197  LLCEKVA----------K-ED---ITVRFF-EEKN---------GQTVWEALGDFQHT---DVHKQTAIT  239
_XP_032591602.1_  197  LLCEKVA----------K-ED---ITVRFF-EERN---------GQTAWEALGDFQHT---DVHKQTAIT  239
_XP_023164572.2_  197  LLCEKVA----------K-ED---ITVRFF-EEKN---------GQTVWEALGDFQHT---DVHKQTAIT  239
_TDG46699.1_      197  LLCEKVA----------K-ED---ITVRFF-EEKN---------GQTVWEALGDFQHT---DVHKQTAIT  239
_KAH8411895.1_    197  LLCEKVA----------K-ED---ITVRFF-EEKN---------GHIVWEALGDFQHT---DVHKQTAIT  239
_KAH8377496.1_    197  LLCEKVA----------K-ED---ITVRFY-EENN---------GVIVWEALGDFQHT---DVHKQTAIT  239
_XP_017854500.2_  197  LLCEKVA----------K-ED---ITVRFF-EEKN---------GQTVWEALGDFQHT---DVHKQTAIT  239
_XP_016987284.2_  197  LLCEKVA----------K-ED---ITVRFF-EEKN---------GQCVWEALGEFQHT---DVHKQTAIT  239
```

```
_KAH8293209.1_     197  LLCEKVA----------K-ED---ITVRFY-EEKN---------GVMVWEALGDFQHT---DVHKQTAIT  239
_EEC00225.1_       196  LLCDKVA----------K-DD---IEVWFE-EERE---------GLTVWKERAEILPN---GVHKQVAIC  238
_XP_029825505.2_   196  LLCDKVA----------K-DD---IEVWFE-EERE---------GLTVWKERAEILPN---GVHKQVAIC  238
_KAG0424790.1_     196  LLCDKVA----------K-DD---IEVWFE-EERE---------GLTVWKERAEILPN---GVHKQVAIC  238
_XP_037272127.1_   196  LLCDKVAK--------GK-YD---IEVWFE-EERD---------GQTVWKERAELLPN---GVHKQVAIC  240
_XP_054934017.1_   196  LLCDKVAK--------D---D---IEVWFE-EERD---------GQTVWKERAELLPN---GVHKQVAIC  238
_XP_050048524.1_   196  LLCDKVAK--------D---D---IEVWFE-EERD---------GQTVWKERAELLPN---GVHKQVAIC  238
_XP_037272126.1_   196  LLCDKVAK--------D---D---IEVWFE-EERD---------GQTVWKERAELLPN---GVHKQVAIC  238
_XP_037524244.1_   196  LLCDKVAK--------D---D---IEVWFE-EERD---------GQTVWKERAELLPN---GVHKQVAIC  238
_XP_037524245.1_   196  LLCDKVAK--------D---D---IEVWFE-EERD---------GQTVWKERAELLPN---GVHKQVAIC  238
_XP_049512094.1_   196  LLCDKVAK--------D---D---IEVWFE-EERD---------GQTVWKERAELLPN---GVHKQVAIC  238
_XP_037524243.1_   196  LLCDKVAK--------D---D---IEVWFE-EERD---------GQTVWKERAELLPN---GVHKQVAIC  238
_XP_037580160.1_   196  LLCDKVAK--------D---D---IEVWFE-EERD---------GQTVWKERAELLPN---GVHKQVAIC  238
_XP_037272128.1_   196  LLCDKVAK--------GK-YD---IEVWFE-EERD---------GQTVWKERAELLPN---GVHKQVAIC  240
_KAH9380259.1_     196  LLCDKVAK--------D---D---IEVWFE-EERE---------GQTLWKERAELLPN---GVHKQVAIC  238
_KAI1291715.1_     169  ILCDKVI----------K-DD---LEVRFY-QEQN---------GKTVWEGLGLFDCS---DIHKQIALS  211
_XP_035209023.1_   190  ILCNLIT------------ED---MQLWFY-EEES---------GEVIWECESRFSSN---DVFKNVALS  231
_XP_023332679.1_   202  ILCEKIS----------R-DD---IRVRFY-DEAG-------------WEAYANFSPS---EVHKQYAIS  240
_XP_046801942.1_   197  LLCEKVA----------K-ED---ISVRFY-EENN---------GKTIWEAYGEFQHT---DVHKQTAIA  239
_XP_037811120.1_   197  LLCEKVA----------K-ED---ISVRFY-EENN---------GQTIWEAYGEFQHT---DVHKQTAIA  239
_AFI98401.1_       197  LLCEKVA----------K-ED---ISVRFY-EEKN---------GQTIWEALGEFQHT---DVHKQTAIA  239
_AFJ54621.1_       197  LLCEKVA----------K-ED---ISVRFY-EEKN---------GQTIWEALGEFQHT---DVHKQTAIA  239
_KAI8126534.1_     197  LLCEKVA----------K-ED---ISVRFY-EENN---------GKTIWEAYGEFQHT---DVHKQTAIA  239
_XP_013118266.1_   197  LLCEKVA----------K-ED---IAVRFF-EEKN---------GQLVWESYGEFQHT---DVHKQTAIA  239
_XP_023293634.2_   197  LLCEKVA----------K-ED---ISVRFY-EENN---------GKTIWEAYGEFQHT---DVHKQTAIA  239
_XP_037811062.1_   197  LLCEKVA----------K-ED---ISVRFY-EENN---------GQTIWEAYGEFQHT---DVHKQTAIA  239
_KNC34132.1_       197  LLCEKVA----------K-ED---ISVRFY-EENN---------GKTIWEAYGEFQHT---DVHKQTAIA  239
_TMW48951.1_       197  LLCEKVA----------K-ED---ISVRFY-EENN---------GQTIWEAYGDFQHT---DVHKQTAIT  239
_XP_037957363.1_   197  LLCEKVA----------K-ED---IAVRFF-EEKN---------GQVFWEAFGEFQHA---DVHKQTAIT  239
_XP_005175014.1_   197  LLCEKVA----------K-ED---IAVRFF-EEKN---------GQLFWEAYGEFQHT---DVHKQTAIA  239
_XP_037957360.1_   197  LLCEKVA----------K-ED---IAVRFF-EEKN---------GQVFWEAFGEFQHA---DVHKQTAIT  239
_XP_030383330.1_   196  LLCEKVA----------K-ED---ISVRFF-EEKN---------GKCVWEALGDFQHT---DVHKQTAIT  238
_XP_030383328.1_   196  LLCEKVA----------K-ED---ISVRFF-EEKN---------GKCVWEALGDFQHT---DVHKQTAIT  238
_CAH1707987.1_     196  LLCEKVA----------K-ED---IQVRFY-EEIMN--------PEEAWEGNGEFQHS---QVHKQVAIS  239
_TMW54796.1_       197  LLCEKVV----------N-KD---ISVRFY-EENN---------GQTIWEAYGDFKHT---DVHKQTAIT  239
_XP_026836077.1_   197  LLCEKVA----------K-ED---ISVRFF-EEKN---------GQSVWEAYGDFQHT---DVHKQTAIT  239
_XP_017116803.1_   197  LLCEKVA----------K-ED---ISVRFF-EEKN---------GQSVWEAFGDFQHT---DVHKQTAIT  239
_XP_039480519.1_   197  LLCEKVA----------K-ED---IAVRFF-EEKN---------GQSVWEAFGDFQHT---DVHKQTAIT  239
_XP_043641920.1_   197  LLCEKVA----------K-ED---IAVRFF-EEKN---------GQSVWEAFGDFQHT---DVHKQTAIT  239
```

```
_XP_023036748.1_  197  LLCEKVA----------K-ED---ISVRFF-EEKN---------GQSVWEAFGDFQHT---DVHKQTAIT  239
_XP_002090466.1_  197  LLCEKVA----------K-ED---IAVRFF-EEKN---------GQSVWEAFGDFQHT---DVHKQTAIT  239
_XP_034471824.1_  197  LLCEKVA----------K-ED---ITVRFY-EEKN---------GVAVWEALGDFQHT---DVHKQTAIT  239
_AAT94434.1_      197  LLCEKVA----------K-ED---ISVRFF-EEKN---------GQSVWEAFGDFQHT---DVHKQTAIT  239
_XP_036670985.1_  197  LLCEKVA----------K-ED---ISVRFF-EEKN---------GQSVWEAFGDFQHT---DVHKQTAIT  239
_AAA28479.1_      197  LLCEKVA----------K-ED---ISVRFF-EEKN---------GQSVWEAFGDFQHT---DVHKQTAIT  239
_XP_037708206.1_  197  LLCEKVA----------K-ED---ISVRFF-EEKN---------GQSVWEAFGDFQHT---DVHKQTAIT  239
_XP_002038915.1_  197  LLCEKVA----------K-ED---ISVRFF-EEKN---------GQSVWEAFGDFQHT---DVHKQTAIT  239
_XP_016993636.1_  197  LLCEKVA----------K-ED---ISVRFF-EEKN---------GQSVWEAFGDFQHT---DVHKQTAIT  239
_XP_052852517.1_  197  LLCEKVA----------K-ED---ISVRFF-EEKN---------GQTVWEAFGDFQHT---DVHKQTAIT  239
_XP_016963139.1_  197  LLCEKVA----------K-ED---ISVRFF-EEKN---------GQSVWEAFGDFQHT---DVHKQTAIT  239
_XP_033156151.1_  197  LLCEKVA----------K-ED---ISVRFF-EEKN---------GQSVWEAFGDFQHT---DVHKQTAIT  239
_XP_001974446.1_  197  LLCEKVA----------K-ED---ISVRFF-EEKN---------GQSVWEAYGDFQHT---DVHKQTAIT  239
_XP_014761886.1_  197  LLCEKVA----------K-ED---ISVRFF-EEKN---------GQSVWEAFGDFQHT---DVHKQTAIT  239
_XP_017107216.2_  197  LLCEKVA----------K-ED---ISVRFF-EENN---------GQTVWEAYGEFQHT---DVHKQTAIA  239
_XP_017044881.1_  196  LLCEKVA----------K-ED---ISVRFF-EEKN---------GQSVWEGFGDFQHT---DVHKQTAIT  238
_XP_016944798.1_  197  LLCEKVA----------K-ED---ISVRFF-EEKN---------GQSVWEAFGDFQHT---DVHKQTAIT  239
_XP_017116800.1_  197  LLCEKVA----------K-ED---ISVRFF-EEKN---------GQSVWEAFGDFQHT---DVHKQTAIT  239
_XP_016993633.2_  197  LLCEKVA----------K-ED---ISVRFF-EEKN---------GQSVWEAFGDFQHT---DVHKQTAIT  239
_XP_037708198.1_  197  LLCEKVA----------K-ED---ISVRFF-EEKN---------GQSVWEAFGDFQHT---DVHKQTAIT  239
_XP_032583259.1_  197  LLCEKVA----------K-ED---ISVRFF-EEKN---------GQSVWEAFGDFQHT---DVHKQTAIT  239
_AAC35296.1_      197  LLCEKVA----------K-ED---ISVRFF-EEKN---------GQSVWEAFGDFQHT---DVHKQTAIT  239
_NP_001286014.1_  197  LLCEKVA----------K-ED---ISVRFF-EEKN---------GQSVWEAFGDFQHT---DVHKQTAIT  239
_XP_016963136.1_  197  LLCEKVA----------K-ED---ISVRFF-EEKN---------GQSVWEAFGDFQHT---DVHKQTAIT  239
_XP_017063904.2_  197  LLCEKVA----------K-ED---ISVRFF-EEKN---------GQSVWEAFGEFQHT---DVHKQTAIT  239
_XP_033156135.1_  197  LLCEKVA----------K-ED---ISVRFF-EEKN---------GQSVWEAFGDFQHT---DVHKQTAIT  239
_XP_052852512.1_  197  LLCEKVA----------K-ED---ISVRFF-EEKN---------GQTVWEAFGDFQHT---DVHKQTAIT  239
_XP_032307758.1_  197  LLCEKVA----------K-ED---ISVRFF-EEKN---------GQSVWEAFGDFQHT---DVHKQTAIT  239
_XP_017107215.2_  197  LLCEKVA----------K-ED---ISVRFF-EENN---------GQTVWEAYGEFQHT---DVHKQTAIA  239
_AWT86616.1_      196  LLCEKVA----------K-ED---ISVRLF-EEVN---------NQTVWEGQCDFQTT---DVHKQVAIT  238
_QUF98534.1_      196  LLCEKVA----------K-ED---ISVRLY-EELN---------NQIVWEGQCDFQTT---DVHKQVAIT  238
_UNA06287.1_      196  LLCEKVA----------K-ED---ISVRLF-EEVN---------NQTVWEGQCDFQTT---DVHKQVAIT  238
_AAQ65068.1_      197  LLCEKVA----------K-ED---IAVRFF-EEKN---------GQSVWEAFGDFQHT---DVHKQTAIT  239
_AAQ64916.1_      197  LLCEKVA----------K-ED---ISVRFF-EEKN---------GQSVWEAFGDFQHT---DVHKQTAIT  239
_AAQ64921.1_      197  LLCEXVA----------K-ED---ISVRFF-EEKN---------GQSVXEAFGDFQHT---DVHKQTAIT  239
_AAQ64918.1_      197  LLCEKVA----------K-ED---ISVRFF-EEKN---------GQSVWEAFGDFQHT---DVHKQTAIT  239
_KAH8245459.1_    222  LLCEKVA----------K-ED---ISVRFF-EEKN---------GQTVWEAFGDFQHT---DVHKQTAIT  264
_KAH8283155.1_    219  LLCEKVA----------K-ED---ISVRFF-EEKN---------GQTVWEAFGDFQHT---DVHKQTAIT  261
_KAH8246384.1_    219  LLCEKVA----------K-ED---ISVRFF-EEKN---------GQTVWEAFGDFQHT---DVHKQTAIT  261
```

```
_KAH8366418.1_     219  LLCEKVA----------K-ED---ISVRFF-EEKN---------GQSVWEAFGEFQHT---DVHKQTAIT  261
_XP_022195378.2_   196  LLCEKVA----------K-ED---ISVKLY-EVVN---------NQIVWEGECEFNTT---DVHKQVAIT  238
_XP_039284931.1_   196  LLCEKVA----------K-ED---ISVKLY-EVVN---------NQIVWEGECEFNTT---DVHKQVAIT  238
_ALC40091.1_       219  LLCEKVA----------K-ED---ITVRFF-EEKN---------GQTVWEALGDFQHT---DVHKQTAIT  261
_XP_036342419.1_   195  LLCEKVA----------K-ED---IAVRFF-EERN---------GVTVWQAYGEFHPS---DVHKQTSIR  237
_XP_022903025.1_   196  LLCEKVA----------K-ED---IQVRFF-EEQN---------GRVVWEGFGEFQPA---QVHKQTAIW  238
_XP_022903017.1_   196  LLCEKVA----------K-ED---IQVRFF-EEQN---------GRVVWEGFGEFQPA---QVHKQTAIW  238
_XP_036218946.1_   195  LLCEKVA----------K-ED---IAVRFF-EEIN---------GVVVWQAYGDFEPS---SVHKQTAIR  237
_XP_018790401.1_   195  LLCEKVA----------K-ED---IAVRFF-EEKN---------GAVVWQAYGEFEPS---SVHKQTAIR  237
_KAI9579887.1_     186  LLCDKVS----------K-TD---IKVRFY-EQNT------------GWTAYGLFEQK---DVHKQTAIV  225
_XP_037892537.1_   185  LLCDKVS----------K-TD---IKVRFY-EQNT------------GWTAYGLFEQK---DVHKQTAIV  224
_XP_018903705.1_   200  LTCEKVT----------R-DD---IEIWIY-EKNSS--------GEIVWEEKTEFQPQ---DVHHQYAIA  243
_CAH0395667.1_     200  LTCEKVT----------R-DD---IEVWIY-EKNSS--------GEIVWEEKTEFQPQ---DVHHQYAIA  243
_XP_050330165.1_   195  LLCEKVT----------K-ED---IAVRFF-EEKN---------GAVVWQAYGEFDPS---SVHKQTAIG  237
_XP_039970878.1_   195  LLCEKVT----------K-ED---IAVRFF-EEKN---------GAVVWQAYGEFDPS---SVHKQTAIG  237
_XP_049305234.1_   195  LLCEKVT----------K-ED---IAVRFF-EEKN---------GAVVWQAYGEFDPS--MHVHKQTAIT  238
_GLH04793.1_       203  LLCEKVA----------K-DD---IEVRFF-EQDVE--------GNVCWEAAGDFTPA---HVHRQVAIC  246
_XP_015835035.1_   196  LLCPKVD----------K-DD---IRIRIY-ETAN---------GQTLWQYFINGDQT---QIYSSTAIC  238
_XP_015835036.1_   196  LLCPKVD----------K-DD---IRIRIY-ETAN---------GQTLWQYFINGDQT---QIYSSTAIC  238
_XP_039283832.1_   207  LLCHKVM----------K-DD---IKIHFY-EEVD---------GQIVWQDYGEFEPN---NVHYQYAIS  249
_XP_031788427.1_   195  ILCEKAT----------R-ED---IEVRFY-RQEN------------NWEAIAELIPP---NVYKQIAIV  234
_KAJ6646894.1_     197  LLCNRVT----------K-NN---IKIRFY-QNGKD--------DSLKWEAWADFRPT---NVHNHVAIS  240
_XP_026466084.1_   132  LLCEKVN----------K-DD---ITVRFF-EEQD---------XLVCWEDYGYFKFS---NVHKQTAIS  174
_XP_044002069.1_   195  LLCEKIQ----------K-ND---IKIRFF-VKDSN--------NKIIWESFGGFNPV--IDIHKQYAIF  239
_XP_046611735.1_   197  LLCDKVP----------K-ED---VQVRFF-EKQT------------GWKCQATIK-----NIHRQVAIS  234
_XP_046417906.1_   197  LLCDKVP----------K-ED---VQVRFF-EKQT------------GWKCQATIK-----NIHRQVAIS  234
_XP_046590309.1_   197  LLCDKVP----------K-ED---VQVRFF-EKQT------------GWKCQATIK-----NIHRQVAIS  234
_XP_046473589.1_   197  LLCDKVP----------K-ED---VQVRFF-EKQT------------GWKCQATIK-----NIHRQVAIS  234
_XP_019892116.1_   108  LLCEKVA----------K-ED---IAVRFF-EEKN---------GQLFWEAYGEFQHT---DVHKQTAIA  150
_XP_005183027.3_   108  LLCEKVA----------K-ED---IAVRFF-EEKN---------GQLFWEAYGEFQHT---DVHKQTAIA  150
_XP_011496598.1_   124  ILCEKIA----------K-DD---VQVRFF-HEEN------------RWEVIAESVYP---HYHKQVAII  163
_AAW67216.1_        96  LLCEKVA----------K-ED---IAVRFY-EEQH---------GNIVWEEYGEFQHT---NVHKQVAIC  138
_AAW67217.1_        96  LLCEKVA----------K-ED---IAVRFY-EEQH---------GNIVWEEYGEFQHT---NVHKQVAIC  138
_XP_017489788.1_    99  LLCEKVA----------K-ED---IAVRFF-EERN---------GVTVWQAYGEFHPS---DVHKQTSIR  141
_BAI67894.1_       100  LLCEKVA----------K-ED---I---------------------------------------------  110
_XP_054741511.1_   196  LLCEKVA----------K-ED---IAVRFF-EEKD---------GVTIWQAFGEFEQN---DVHKQTAIS  238
_XP_053961684.1_   196  LLCEKVA----------K-ED---IAVRFF-EEKD---------GVTIWQAFGEFEQN---DVHKQTAIS  238
_XP_036341313.1_   196  LLCEKVA----------K-ED---IAVRFF-EEKD---------GVTIWQAFGEFEHN---DVHKQTAIT  238
_XP_036341343.1_   196  LLCEKVA----------K-ED---IAVRFF-EEKD---------GVTIWQAFGEFEHN---DVHKQTAIT  238
```

```
_XP_017471383.1_  196  LLCEKVA----------K-ED---IAVRFF-EEKD---------GVTIWQAFGEFEHN---DVHKQTAIT  238
_XP_054741498.1_  196  LLCEKVA----------K-ED---IAVRFF-EEKD---------GVTIWQAFGEFEQN---DVHKQTAIS  238
_XP_053961677.1_  195  LLCEKVA----------K-ED---IAVRFF-EEKD---------GVTIWQAFGEFEQN---DVHKQTAIS  237
_XP_050320008.1_  196  LLCEKVA----------K-ED---IAVRFF-EEKD---------GVTIWQAYGQFEHS---DVHKQTAIT  238
_XP_028894490.2_  196  LLCEKVA----------K-ED---IAVRFF-EEKD---------GVTIWQGFGEFEHN---DVHKQTAIT  238
_XP_050320015.1_  196  LLCEKVA----------K-ED---IAVRFF-EEKD---------GVTIWQAYGQFEHS---DVHKQTAIT  238
_XP_018787554.1_  196  LLCEKVA----------K-ED---IAVRFF-EEKD---------GVTIWQAYGQFEHS---DVHKQTAIT  238
_XP_029407438.2_  196  LLCEKVA----------K-ED---IAVRFF-EEKD---------GVTIWQAYGQFEHS---DVHKQTAIT  238
_XP_036225551.1_  196  LLCEKVA----------K-ED---IAVRFF-EEKD---------GVTIWQAYGQFEHS---DVHKQTAIT  238
_XP_011178374.2_  196  LLCEKVA----------K-ED---IAVRFF-EEKD---------GVTIWQGFGEFEHN---DVHKQTAIT  238
_XP_039952593.1_  196  LLCEKVA----------K-ED---IAVRFF-EEKD---------GVTIWQAYGQFEHS---DVHKQTAIT  238
_XP_018787542.1_  196  LLCEKVA----------K-ED---IAVRFF-EEKD---------GVTIWQAYGQFEHS---DVHKQTAIT  238
_XP_011207866.2_  196  LLCEKVA----------K-ED---IAVRFF-EEKD---------GVTIWQAYGQFEHS---DVHKQTAIT  238
_XP_014086506.1_  196  LLCEKVA----------K-ED---IAVRFF-EEKD---------GVTIWQAYGQFEHS---DVHKQTAIT  238
_XP_004526638.1_  198  LLCEKVA----------K-ED---IAVRFF-EEKD---------GVIVWQGYGEFEHN---DVHKQTAIA  240
_XP_017471402.1_  196  LLCEKVA----------K-ED---IAVRFF-EEKD---------GVTIWQAFGEFEHN---DVHKQTAIT  238
_XP_012157903.1_  196  LLCEKVA----------K-ED---IAVRFF-EEKD---------GVIVWQGYGEFEHN---DVHKQTAIA  238
_XP_011185661.2_  194  LLCEKVA----------K-ED---IAVRFF-EEKN---------GVVVWQAYGDFLSS---DVHKQTSIR  236
_XP_050322757.1_  195  LLCEKVT----------K-ED---IAVRFF-EEKN---------GAVVWQAYGEFDPS---SVHKQTAIT  237
_XP_039962922.1_  195  LLCEKVT----------K-ED---ISVRFF-EEKN---------GVVVWQAYGEFDAS---SVHKQTAIT  237
_XP_050322739.1_  195  LLCEKVT----------K-ED---IAVRFF-EEKN---------GAVVWQAYGEFDPS---SVHKQTAIT  237
_XP_050322749.1_  195  LLCEKVT----------K-ED---IAVRFF-EEKN---------GAVVWQAYGEFDPS---SVHKQTAIT  237
_XP_050322724.1_  195  LLCEKVT----------K-ED---IAVRFF-EEKN---------GAVVWQAYGEFDPS---SVHKQTAIT  237
_XP_050322682.1_  195  LLCEKVT----------K-ED---IAVRFF-EEKN---------GAVVWQAYGEFDPS---SVHKQTAIT  237
_CAG9823112.1_    203  LLCEKVA----------K-ED---IQVRFF-EDPG------------DWEAYADFQPS---QVHKQHAIW  242
_KAG5873705.1_    198  LLCEKVA----------K-ED---IQVRFY-EENG------------DWEGLADFQPS---QVHKQHAIW  237
_KAH8030364.1_    175  LLCDKVA----------K-DD---IEVWF--EEERD--------GQTVWKERAELLPN---GVHKQVAIC  217
_XP_023013891.1_  198  LLCEKVA----------K-ED---IQIRFY-EETS------------DWEAFADFQPS---QVHKQHAIW  237
_XP_028130780.2_  204  LLCEKVA----------K-ED---IQVRFF-EENG------------DWEAFADFQPS---QVHKQHAIW  243
_KAG5873704.1_    203  LLCEKVA----------K-ED---IQVRFY-EENG------------DWEGLADFQPS---QVHKQHAIW  242
_CAH1187690.1_    203  LLCEKVA----------K-ED---IQVRFF-EDSS------------DWEAFADIQPS---QVHKQHAIW  242
_CAH1098641.1_    203  LLCEKVA----------K-ED---IQVRFF-EENS------------DWEAFADIQPA---QVHKQHAIW  242
_XP_023028116.1_  203  LLCEKVV----------K-ED---IQVWFH-EENS------------DWEAQADVQPS---QVHKSFAIS  242
_XP_031630227.1_  202  LLCDKVK----------R-ED---ISVVFY-EEDAD--------RNQIWREEIQYKNSTTMKVHHQYAIS  248
_KAG5861047.1_     72  LLCEKVA----------K-ED---IQVRFY-EENG------------DWEGLADFQPS---QVHKQHAIW  111
_KAF2880990.1_    198  LLCEKVA----------K-ED---IQIRFY-EEKD---------GRVEWEGFGDFQPS---QVHKQTAIW  240
_XP_018326789.1_  197  LLCEKVA----------K-ED---IQVRFY-EEKE---------NQVVWEGFADFQPS---QVHKQTAIW  239
_XP_018326788.1_  197  LLCEKVA----------K-ED---IQVRFY-EEKE---------NQVVWEGFADFQPS---QVHKQTAIW  239
_XP_031339184.1_  197  LLCEKVS----------K-ED---IEIRFY-EERD---------GQVVWEGRGDFQPS---QVHKQTAIW  239
```

```
_XP_045476069.1_  198  MLCEKIT----------K-ED---IQIRFY-EERD---------GMLIWEDFGEFQPA---QVHKQVAIC  240
_XP_044734149.1_  196  LFCEKVT----------K-DD---INVRFY-EEKN---------GQMVWEGFADFQPS---DVHRQVGIA  238
_XP_044734150.1_  196  LFCEKVT----------K-DD---INVRFY-EEKN---------GQMVWEGFADFQPS---DVHRQVGIA  238
_XP_044734148.1_  196  LFCEKVT----------K-DD---INVRFY-EEKN---------GQMVWEGFADFQPS---DVHRQVGIA  238
_XP_044760528.1_  198  LLCEKIT----------K-ED---IQIRYY-EERD---------GLLVWEDYGEFQPA---QVHKQVAIC  240
_XP_044747918.1_  198  LLCEKVA----------K-ED---IQIRFF-EEKD---------GKTVWEAYGDFQSN---QVHKQTSIW  240
_XP_044747917.1_  198  LLCEKVA----------K-ED---IQIRFF-EEKD---------GKTVWEAYGDFQSN---QVHKQTSIW  240
_XP_045481017.1_  198  LLCEKVA----------K-ED---IQIRFF-EEKD---------GKTVWEGYADFQSN---QVHKQTSIW  240
_XP_045481018.1_  198  LLCEKVA----------K-ED---IQIRFF-EEKD---------GKTVWEGYADFQSN---QVHKQTSIW  240
_KAF5302274.1_    198  LLCEKVA----------K-ED---IQIRFY-EEKD---------GQVVWEGFGDFQPS---QVHKQTAIW  240
_KAF5308883.1_    198  LLCEKVA----------K-ED---IQIRFY-EEKD---------GQVVWEGFGDFQPS---QVHKQTAIW  240
_KRT79621.1_      196  LLCEKVA----------K-ED---IQIRFF-EEKN---------NEVVWEGFGEFQPS---QVHKQTAIW  238
_BAD20728.1_      196  LLCEKVA----------K-ED---IQIRFF-EEKN---------NEVVWEGYGEFQPN---QVHKQTAIA  238
_KAF2880988.1_    197  LLCEKVA----------K-DD---IQIRFY-EEKD---------GRVEWEVFADFPPS---QVHKETAIW  239
_CAH1403258.1_    165  --------------------D---ISVRFF-EIRD---------GKLFWEGYGDFTPT---QVHKQTAIA  198
_CAD6992094.1_    195  LLCDKII----------R-EK---TMVRFY-EEKG---------SDVIWQSYGDYRPS---DIHRQTAIK  237
_XP_004537184.1_  195  LLCDKII----------R-EK---TMVRFY-EEKG---------SDVIWQSYGDYRPS---DIHRQTAIK  237
_XP_018572644.1_  198  LLCEKVA----------K-ED---IQVRFY-EEKG---------D---WEAFADFQPS---QVHKQHAIW  237
_RZB54393.1__     198  LLCEKVA----------K-ED---IQVRFF-EEKD---------GRVVWEGFGDFQPS---QVHKQTAIS  240
_XP_044255725.1_  198  LLCEKVA----------K-ED---IQVRFF-EEIN---------GKVVWEGFGDFQPS---QVHKQTAIC  240
_EFA02850.1_      198  LLCEKVA----------K-ED---IQVRFF-EEKN---------GKVVWEGFGDFQPS---QVHKQTAIC  240
_NP_001034507.1_  198  LLCEKVA----------K-ED---IQVRFF-EEKN---------GKVVWEGFGDFQPS---QVHKQTAIC  240
_CAH1370311.1_    198  LLCEKVA----------K-ED---IQVRFF-EEKD---------GRVVWEGYGDFQPS---QVHKQTAIC  240
_KAJ3630692.1_    198  LLCEKVA----------K-ED---IQVRFF-EEKD---------GRVVWEGYGDFQPS---QVHKQTAIC  240
_XP_044255724.1_  198  LLCEKVA----------K-ED---IQVRFF-EEIN---------GKVVWEGFGDFQPS---QVHKQTAIC  240
_KAH0811528.1_    198  LLCEKVA----------K-ED---IQVRFF-EEKD---------GRVVWEGYGDFQPS---QVHKQTAIC  240
_CAH7716406.1_    197  LLCEKVA----------K-DD---IQIRFF-EKHG------------DWEAYADIQPS---NVHHQYAIW  236
_CAH7716405.1_    197  LLCEKVA----------K-DD---IQIRFF-EKHG------------DWEAYADIQPS---NVHHQYAIW  236
_CAH7716404.1_    197  LLCEKVA----------K-DD---IQIRFF-EKHG------------DWEAYADIQPS---NVHHQYAIW  236
_XP_028130776.2_  198  LLCEKVA----------K-ED---IQVRFF-EENG------------DWEAFADFQPS---QVHKQHAIW  237
_CAH1985684.1_    197  LLCEKVA----------K-DD---IQVRFF-EKHG------------DWEAYADIQPN---NVHHQYAIW  236
_XP_017781152.1_  198  VLCEKVS----------K-ED---IQIRFY-EEKD---------GLVVWEGLGDFQPS---QVHKQTAIW  240
_XP_017781151.1_  198  VLCEKVS----------K-ED---IQIRFY-EEKD---------GLVVWEGLGDFQPS---QVHKQTAIW  240
_XP_048522053.1_  194  LLCEKVA----------K-ED---VEIHFY-EEDLQ--------GNRVWQAKADFQPS---QVHKQTAIS  237
_XP_050303070.1_  209  LLCEKVA----------K-ED---IEVRFF-EEDDA--------GNRVWEEKADFQPT---QVHKQHAIW  252
_CAG9762924.1_    198  LLCEKVA----------K-ED---IEVHFY-EKNQQ--------GEEVWHGKADFQPG---QVHKQHAIW  241
_CAH0560654.1_    200  LLCEKVA----------K-ED---IQVRFY-EEVG---------GQIVWEALADIQPA---QVHKQHAIW  242
_CAH0560655.1_    200  LLCEKVA----------K-ED---IQVRFY-EEVG---------GQIVWEALADIQPA---QVHKQHAIW  242
_XP_018572643.1_  197  LLCEKVA----------K-ED---IQVRFY-EEKG------------DWEAFADFQPS---QVHKQHAIW  236
```

```
_XP_030763656.1_  198  LLCEKVA----------K-ED---IEIHFF-EMEN---------DKRVWSAKADFQPS---QVHKQHAIW  240
_XP_030763654.1_  198  LLCEKVA----------K-ED---IEIHFF-EMEN---------DKRVWSAKADFQPS---QVHKQHAIW  240
_XP_018577945.1_  198  LLCEKVT----------K-ED---ISVRFY-ENEG------------EWEAY--VKPI---RVHKQHAIC  235
_KAF7283276.1_    198  LLCEKVA----------K-ED---IEIHFY-ELEN---------DKKVWEAKADFQPS---QVHKQHAIW  240
_KAF7283275.1_    198  LLCEKVA----------K-ED---IEIHFY-ELEN---------DKKVWEAKADFQPS---QVHKQHAIW  240
_XP_030763617.1_  197  LVCDKVA----------K-DD---IEIHFF-EMKN---------DEIVWSAKADFLQH---QVHKHHAIW  239
_XP_030763619.1_  200  LLCEKAS----------K-DD---IEVHFF-ELEN---------DRVVWSAKPDIRPT---QVYKQVVIW  242
_VEN60240.1_      108  LLCEKVA----------K-DD---IQIRFF-EKHG------------DWEAYADIQPS---NVHHQYAIW  147
_KOB78236.1_      182  LLTEKLV----------S-SD---VLSVVF-AEKGR-------KLEEIWKESVEITSK---SVHKQHALL  226
_ERL85515.1_      125  -CVSHVA----------K-ED---VEIHFY-EEDLQ--------GNRVWQAKADFQPS---QVHKQTAIS  167
_ENN78766.1_      120  -C---VA----------K-ED---VEIHFY-EEDLQ--------GNRVWQAKADFQPS---QVHKQTAIS  159
_KAH1019371.1_    120  -C---VA----------K-ED---VEIHFY-EEDLQ--------GNRVWQAKADFQPS---QVHKQTAIS  159
_KAI9565420.1_    196  LLCDKVT----------K-DD---IQVFFY-HEEE---------GRTIWEGYGDFSAS---DVHKQVAIT  238
_XP_045024905.1_  196  LLCDKVT----------K-DD---IQVVFY-HEEE---------GRLIWEGIGDFSAS---DVHKQVAIT  238
_XP_046447094.1_  196  LLCDKVT----------K-DD---IQVVFY-HEEE---------GHLIWEGTGEFSAS---DVHKQVAIT  238
_KZS08518.1_      196  LLCDKVT----------K-DD---IQVVFY-HEEE---------GRLIWEGIGDFSAS---DVHKQVAIT  238
_XP_046636307.1_  196  LLCDKVT----------K-DD---IQVVFY-HEEE---------GHLIWEGTGEFSAS---DVHKQVAIT  238
_EFX79013.1_      196  LLCDKVT----------K-DD---IQVVFY-HEEE---------GHLIWEGTGEFSAS---DVHKQVAIT  238
_XP_032776606.2_  196  LLCDKVT----------K-DD---IQVVFY-HEEE---------GRLIWEGIGDFSAS---DVHKQVAIT  238
_XP_046447093.1_  196  LLCDKVT----------K-DD---IQVVFY-HEEE---------GHLIWEGTGEFSAS---DVHKQVAIT  238
_XP_046636306.1_  196  LLCDKVT----------K-DD---IQVVFY-HEEE---------GHLIWEGTGEFSAS---DVHKQVAIT  238
_CAH0099799.1_    186  LLCDKVT----------K-DD---IQVVFY-HEEE---------GHLIWEGIGEFSAS---DVHKQVAIT  228
_KAJ1531033.1_    190  LFCDKIN----------K-DD---IEIVFF-EETD---------GIRTWEACVDIKPS---HVHRGCGII  232
_ACJ36225.1_      197  LLCDKVT----------K-ED---IHVRFY-EVKD---------GMIEWEAFGDFQAS---DVHKQVAIS  239
_ACZ98167.1_      197  LLCDKVT----------K-ED---IHVRFY-EVKD---------GMIEWEAFGDFQAS---DVHKQVAIS  239
_AME17867.1_      197  LLCDKVT----------K-ED---IHVRFY-EVKD---------GMIEWEAFGDFQAS---DVHKQVAIS  239
_XP_042868409.1_  197  LLCDKVT----------K-ED---IHVRFY-EVKD---------GMIEWEAFGDFQAS---DVHKQVAIS  239
_XP_045102711.1_  197  LLCDKVT----------K-ED---IHVRFY-EVKD---------GMIEWEGFGDFQAS---DVHKQVAIS  239
_XP_047487558.1_  197  LLCDKVT----------K-ED---IHVRFY-EVKD---------GMIEWEAFGDFQAS---DVHKQVAIS  239
_QBJ05651.1_      197  LLCDKVT----------K-ED---IHVRFY-EVKD---------GMIEWEGFGDFQAS---DVHKQVAIS  239
_AZK36046.1_      197  LLCDKVT----------K-ED---IHVRFY-EVKD---------GMIEWEGFGDFQAS---DVHKQVAIS  239
_XP_045581920.1_  197  LLCDKVT----------K-ED---ILVRFY-EVKD---------GMIEWESYGDFQAS---DVHKQVAIS  239
_XP_050704598.1_  197  LLCDKVT----------K-ED---IQVRFY-EEKD---------GLLEWEGYGDFQAS---DVHKQVAIS  239
_XP_027237995.1_  197  LLCDKVT----------K-ED---IHVRFY-EVKD---------GMIEWEAFGDFQAS---DVHKQVAIS  239
_AXB88326.1_      197  LLCDKVT----------K-ED---IHVRFY-EVKD---------GMIEWEGFGDFQAS---DVHKQVAIS  239
_XP_045102712.1_  197  LLCDKVT----------K-ED---IHVRFY-EVKD---------GMIEWEGFGDFQAS---DVHKQVAIS  239
_MPC17461.1_      197  LLCDKVT----------K-ED---IHVRFY-EVKD---------GMIEWEGFGDFQAS---DVHKQVAIS  239
_XP_027237994.1_  197  LLCDKVT----------K-ED---IHVRFY-EVKD---------GMIEWEAFGDFQAS---DVHKQVAIS  239
_XP_027237996.1_  197  LLCDKVT----------K-ED---IHVRFY-EVKD---------GMIEWEAFGDFQAS---DVHKQVAIS  239
```

```
_XP_045102715.1_   197  LLCDKVT----------K-ED---IHVRFY-EVKD---------GMIEWEGFGDFQAS---DVHKQVAIS  239
_XP_053627990.1_   197  LLCDKVT----------K-ED---ILVRFY-EVKD---------GMIDWEAYGDFQAS---DVHKQVAIS  239
_XP_053627989.1_   197  LLCDKVT----------K-ED---ILVRFY-EVKD---------GMIDWEAYGDFQAS---DVHKQVAIS  239
_XP_053627983.1_   197  LLCDKVT----------K-ED---ILVRFY-EVKD---------GMIDWEAYGDFQAS---DVHKQVAIS  239
_KAB7495058.1_     197  LLCDKVT----------K-ED---IQIRFT-EVKD---------GHIEWEAYGDFQAS---DVHKQVAIS  239
_KAF2353654.1_     197  LLCDKVT----------K-ED---IQVRFY-EVTD---------GLISWEAFGDFQPS---DVHKQVAIA  239
_ANH22235.1_       197  LLCEKVT----------K-ED---IQVRFY-EVKD---------GRIEWEAFGDFQAS---DVHKQVAIS  239
_CAD7275741.1_     196  LLCEKVA----------K-EN---ISVRFYEESPQ---------GEVTWEDFGKFQPS---DVHKQVAIC  239
_XP_042243403.1_   197  LLCDKVT----------K-ED---IHVRFF-EVKD---------GMVEWEAFGDFQAS---DVHKQVAIS  239
_QTC09942.1_       197  LLCDKVT----------K-ED---IHVRFY-EVKD---------GMIEWEAFGDFQAS---DVHKQVAIS  239
_XP_018023738.1_   176  LLCDKVT----------K-ED---IQVRFY-EVTD---------GLISWEALGEFQPS---DVHKQVAIA  218
_XP_019877552.1_   199  LLCEKVA----------K-ED---IQVRFY-DDAD------------GWETFAEIQPA---QVHKQHAIW  238
_XP_049825875.1_   197  LFCEKVS----------K-ED---IQVRFY-DDAD------------GWETSIDIQPS---QVHKQTGIW  236
_CAD7649074.1_     197  LLCDRVT----------K-DD---IQVRFF-QELN---------GQVVWDALADFGPG---DVHKQVAIC  239
_CAD7630012.1_     197  LLCDRVT----------K-DD---IQVRFF-QEHN---------GQVVWDAFADFGPG---DVHKQVAIC  239
_XP_054165343.1_   198  LLCDRVT----------K-DD---IQVRFY-EEQN---------GQLVWEALAEFAPT---DVHKQVAIC  240
_XP_044254219.1_   196  LLCPKVD----------K-DD---IRIRIY-EVTN---------GQTTWQEFVTGDQM---QIYSSTAIC  238
_XP_053201827.1_   197  LLCDRVA----------K-DD---IQIRFF-EESN---------GQLIWEGYADFQPN---DVHKQVAIS  239
_XP_015786990.1_   197  LLCDRVA----------K-DD---IQVRFY-EESK---------GQIIWEGYADFQPN---DVHKQVAIS  239
_KAI1295507.1_     197  LLCDRVT----------K-DD---IQVRFY-EERN---------NQLVWEAYGEFQPN---DVHKQVAIC  239
_RWS07527.1_       197  LLCDRVT----------K-DD---IQVRFF-EEKN---------GQLVWEGFGDMQPN---DVHKQVAIS  239
_RWS29859.1_       197  LLCDRVT----------K-DD---IQVRFF-EEKN---------GQLIWEGFGDLQPN---DVHKQVAIS  239
_XP_053201825.1_   216  LLCDRVA----------K-DD---IQIRFF-EESN---------GQLIWEGYADFQPN---DVHKQVAIS  258
_XP_054713467.1_   194  ILCPRV-----------N-EG---AEVWFF-EECQ---------SQITWAKQATVKC-----IYKKVAVM  233
_XP_046915158.1_   198  LLCDRVA----------K-DD---IQVRFV-QEGNN-------QQGIIWEAYGEFLAN---DVHKQVAIS  242
_KAJ6217218.1_     198  LLCDRVA----------K-DD---IQVRFY-HESID-------SAHLLWEAYGEFLPN---DVHKQVAIS  242
_KAI2801352.1_     198  LLCDRVA----------K-DD---IQVRFY-HESID-------SAHLLWEAYGEFLPN---DVHKQVAIS  242
_KAH9406651.1_     198  LLCDRVA----------K-DD---IQVRFF-HETVD--------CALIWEAYGEFLPN---DVHKQVAIS  241
_XP_017483538.1_   198  LLCDRVA----------K-DD---IQVRFF-HETVD--------CALIWEAYGEFLPN---DVHKQVAIS  241
_KPM10359.1_       198  LLCDRVA----------K-DD---IQVRFF-QESE---------KGIVWEAFGEFSAN---DVHKQVAIA  240
_UXI17582.1_       198  LLCDRVA----------K-DD---IQVRFF-QESE---------KGIVWEAFGEFSAN---DVHKQVAIA  240
_KAF7488016.1_     198  LLCDRVA----------K-DD---IQVRFF-QESE---------KGIVWEAFGEFSAN---DVHKQVAIA  240
_KAH9423377.1_     198  LLCDRVA----------K-DD---IQVRFV-QENQQK------QSGIIWEAYGDFLAN---DVHKQVAIS  243
_OTF73464.1_       198  LLCDRVA----------K-DD---IQVRFV-QEGNNQ------Q-GIIWEAYGEFLAN---DVHKQVAIS  242
_XP_037072056.1_   197  LLCDKVT----------K-DD---IEVRFF-EVRD---------NRLCWEAFGEFLPA---DVHKQVAIC  239
_XP_037083492.1_   197  LLCDKVT----------K-DD---IEVRFF-EVRD---------NRLHWEAFGEFLPA---DVHKQVAIS  239
_XP_037083508.1_   197  LLCDKVT----------K-DD---IEVRFF-EVRD---------NRLHWEAFGEFLPA---DVHKQVAIS  239
_XP_043215018.1_   197  LLCDKVT----------K-DD---IEVRFF-EVRD---------NRLHWEAFGEFLPA---DVHKQVAIC  239
_KAF0314765.1_     197  LLCDKVT----------K-DD---IEVRFF-EVRD---------NRLYWEAFGEFLPA---DVHKQVAIC  239
```

```
_XP_043215020.1_   197  LLCDKVT----------K-DD---IEVRFF-EVRD---------NRLHWEAFGEFLPA---DVHKQVAIC  239
_XP_043189654.1_   197  LLCDKVT----------K-DD---IEVRFF-EVRD---------NRLYWEGFGEFLPA---DVHKQVAIC  239
_KAF0293344.1_     183  LLCDKVT----------K-DD---IEVRFF-EVRD---------NRLYWEGFGEFLPA---DVHKQVAIC  225
_XP_011496609.1_   198  ILCDKVT----------R-EN---IQVRFF-HDDN------------NWQSLAEFTPP---GIFKQA---  234
_AGS12619.1_       197  LFCDKVI----------K-DD---IQVRFY-QEMN---------GQTVWEGLGDFQPC---DVHKQFGIA  239
_TRY78048.1_       197  LFCDKVI----------K-DD---IQVRFY-QEMN---------GQLVWEGLGDFQPC---DVHKQFGIA  239
_XP_040576307.1_   197  IFCDKVT----------K-DD---IQVRFY-EEKD---------GKVIWEGLGDFQLT---DVHKQYGIC  239
_CAB4056363.1_     197  IFCDKVT----------K-DD---IQVRFY-EEKD---------GKVIWEGLGDFQLT---DVHKQYGIC  239
_ALF84898.1_       197  LFTEKVT----------K-DD---IEVRFY-QEIN---------GKVVWEGLGEFQPS---DVHKQYGIC  239
_XP_023349538.1_   193  LFCDKVT----------K-DD---IQVRFY-EESSN--------DSFTWEGFGDFQPA---DVHKQYGIC  236
_XP_004522582.1_   196  LMCEKIV----------R-ND---IAVRFF-EEKD---------NTTLWQAFGALEPS--KDIHKLTAIS  239
_CAD7013622.1_     196  LMCEKIV----------R-ND---IAVRFF-EEKD---------NTTLWQAFGALEPS--KDIHKLTAIS  239
_XP_042902217.1_   196  LLCDRVA----------K-ED---IQIWFY-EEQN---------SRIVWESPAEFQPS---DVHKQVAIS  238
_XP_042902218.1_   196  LLCDRVA----------K-ED---IQIWFY-EEQN---------SRIVWESPAEFQPS---DVHKQVAIS  238
_XP_035215470.1_   196  LLCDRVA----------K-ED---IQIWFY-EEQG---------SRIVWESQAEFQPS---DVHKQVAIS  238
_GBN14612.1_       195  LLCDRVA----------K-ED---IQIWFY-EEQG---------SRIVWESQAEFQPS---DVHKQVAIS  237
_KAF8795965.1_     196  LLCDRVA----------K-ED---IQIWFY-EEQG---------SRIVWESQAEFQPS---DVHKQVAIS  238
_KAF8795964.1_     196  LLCDRVA----------K-ED---IQIWFY-EEQG---------SRIVWESQAEFQPS---DVHKQVAIS  238
_GFY66827.1_       196  LLCDRVA----------K-ED---IQIWFY-EEQG---------SRILWESQAEFQPS---DVHKQVAIS  238
_GFW66965.1_       196  LLCDRVA----------K-ED---IQIWFY-EEQG---------SRILWESQAEFQPS---DVHKQVAIS  238
_GFQ85823.1_       196  LLCDRVA----------K-ED---IQIWFY-EEQG---------SRILWESQAEFQPS---DVHKQVAIS  238
_GFT35953.1_       196  LLCDRVA----------K-ED---IQIWFY-EEQG---------SRILWESQAEFQPS---DVHKQVAIS  238
_XP_054714028.1_   196  LLCDRVA----------K-DD---IQVWFY-EEQS---------SQILWESQAEFQPS---DVHKQVAIT  238
_KAG8194583.1_     195  LLCDRVA----------K-ED---IQIWFY-EEQG---------SMILWESQAEFQPS---DVHKQVAIS  237
_GIY09899.1_       195  LLCDRVA----------K-ED---IQIWFY-EEQG---------SRILWESQAEFQPS---DVHKQVAIS  237
_GIY93593.1_       195  LLCDRVA----------K-ED---IQIWFY-EEQG---------SRILWESQAEFQPS---DVHKQVAIS  237
_XP_042902221.1_   208  LLCDRVP----------K-ES---IQIWFY-EVHD---------SNVIWESPAEFHPS---DIHKQVAIS  250
_XP_046962867.1_   199  LLCEKVT----------R-ED---IAVVFF-QKEG---------EHVVWEESANIV-----LVHRQVAIA  239
_XP_047530540.1_   199  LLCEKVT----------R-ED---IAVVFF-QKEG---------EHVVWEESANIV-----LVHRQVAIA  239
_XP_026486466.1_   199  LLCEKVT----------R-ED---IAVVFF-QKEG---------EHVVWEESANIV-----LVHRQVAIA  239
_XP_047530539.1_   199  LLCEKVT----------R-ED---IAVVFF-QKEG---------EHVVWEESANIV-----LVHRQVAIA  239
_XP_047530538.1_   199  LLCEKVT----------R-ED---IAVVFF-QKEG---------EHVVWEESANIV-----LVHRQVAIA  239
_XP_047530537.1_   199  LLCEKVT----------R-ED---IAVVFF-QKEG---------EHVVWEESANIV-----LVHRQVAIA  239
_XP_026486464.1_   199  LLCEKVT----------R-ED---IAVVFF-QKEG---------EHVVWEESANIV-----LVHRQVAIA  239
_XP_047530541.1_   199  LLCEKVT----------R-ED---IAVVFF-QKEG---------EHVVWEESANIV-----LVHRQVAIA  239
_XP_050347286.1_   199  LLCEKVT----------R-ED---IAVVFF-QKEG---------EHVVWEESANIV-----LVHRQVAIA  239
_XP_050347283.1_   199  LLCEKVT----------R-ED---IAVVFF-QKEG---------EHVVWEESANIV-----LVHRQVAIA  239
_XP_032515351.1_   200  LLCERVT----------R-ED---ISVVFF-QKEG---------DNVVWEESAHIV-----LVHRQYAIA  240
_OWR46016.1_       200  LLCERVT----------R-ED---ISVVFF-QKEG---------DNVVWEESAHIV-----LVHRQYAIA  240
```

```
_XP_032515346.1_  200  LLCERVT----------R-ED---ISVVFF-QKEG---------DNVVWEESAHIV-----LVHRQYAIA  240
_CAG9579094.1_    200  LLCERVT----------R-ED---IAVVFF-QKEG---------DSVVWEESAHIV-----LVHRQYAIA  240
_CAG9579091.1_    200  LLCERVT----------R-ED---IAVVFF-QKEG---------DSVVWEESAHIV-----LVHRQYAIA  240
_XP_050668638.1_  198  LLCEKVV----------R-ED---VAVVFF-EKDG---------ERVVWEENGNIT-----LVHKQVAIA  238
_XP_050668632.1_  198  LLCEKVV----------R-ED---VAVVFF-EKDG---------ERVVWEENGNIT-----LVHKQVAIA  238
_XP_045495329.1_  198  LLCEKVV----------R-ED---IAVVFF-QKEG---------DTVVWEESANIV-----LVHKQVAIA  238
_XP_038214802.1_  198  LLCEKVV----------R-ED---IAVVFF-QKEG---------DAVVWEESANIV-----LVHKQVAIA  238
_CAH0714714.1_    199  LLCEKVT----------R-ED---IAIVFF-QKEG---------ERVVWEEHANIV-----LVHKLTAIV  239
_CAH0714713.1_    199  LLCEKVT----------R-ED---IAIVFF-QKEG---------ERVVWEEHANIV-----LVHKLTAIV  239
_CAH4029256.1_    198  LLCEKVV----------R-ED---IAVVFY-QKEG---------EMVVWEETANIQ-----LVHKQYAIA  238
_XP_047513081.1_  198  LLCEKVV----------R-ED---IAVVFY-QKEG---------EMVVWEETANIQ-----LVHKQYAIA  238
_XP_045529569.1_  198  LLCEKVV----------R-ED---IAVVFY-QKEG---------EMVVWEETANIQ-----LVHKQYAIA  238
_XP_045487500.1_  198  LLCEKVV----------R-ED---IAVVFY-QKEG---------EMVVWEETANIQ-----LVHKQYAIA  238
_XP_047513077.1_  198  LLCEKVV----------R-ED---IAVVFY-QKEG---------EMVVWEETANIQ-----LVHKQYAIA  238
_CAH4029257.1_    198  LLCEKVV----------R-ED---IAVVFY-QKEG---------EMVVWEETANIQ-----LVHKQYAIA  238
_XP_045487498.1_  198  LLCEKVV----------R-ED---IAVVFY-QKEG---------EMVVWEETANIQ-----LVHKQYAIA  238
_CAF4952989.1_    198  LLCEKVV----------R-ED---IAVVFY-QKEG---------EMVVWEETANIQ-----LVHKQYAIA  238
_XP_045447470.1_  199  LLCEKVT----------R-ED---IAVVFF-EKEG---------EQVVWEESANIV-----LVHRQVAIA  239
_CAH2108688.1_    138  LLCEKVT----------R-ED---IAVVFF-QKEG---------ERVVWEESANIV-----LVHRQVAIA  178
_CAH0714724.1_    197  ILCGWVT------------ND---ISVVFF-EKVE---------DKIVWEKHAKVD-----YVHNHVAIA  236
_XP_034827218.1_  199  LLCERVT----------K-ED---VMVVFF-QKDG---------DRTVWEANANVV-----LVHRQYAVV  239
_XP_045768234.1_  199  LLCERVT----------K-ED---VMVVFF-QKEG---------DRTVWEANANVV-----LVHRQYAVV  239
_XP_034827214.1_  199  LLCERVT----------K-ED---VMVVFF-QKDG---------DRTVWEANANVV-----LVHRQYAVV  239
_XP_045768230.1_  199  LLCERVT----------K-ED---VMVVFF-QKEG---------DRTVWEANANVV-----LVHRQYAVV  239
_XP_039751073.1_  199  LLCERVT----------K-ED---VMVVFF-QKEG---------DNVVWEANANVL-----MVHRQYAVV  239
_XP_023942270.1_  199  LLCERVT----------K-ED---VMVVFF-QKEG---------ERVVWEANANVL-----MVHRQYAVV  239
_XP_023942268.1_  199  LLCERVT----------K-ED---VMVVFF-QKEG---------ERVVWEANANVL-----MVHRQYAVV  239
_XP_039751072.1_  199  LLCERVT----------K-ED---VMVVFF-QKEG---------DNVVWEANANVL-----MVHRQYAVV  239
_XP_023942266.1_  199  LLCERVT----------K-ED---VMVVFF-QKEG---------ERVVWEANANVL-----MVHRQYAVV  239
_CAH2240541.1_    186  LLCERVT----------K-ED---VMVVFF-QKEG---------DNVVWEANANVL-----MVHRQYAVV  226
_XP_041985045.1_  199  LLCERIT----------K-ED---ISVVFF-ERGDG--------DRIVWEKSAHVM-----LVHRQYAIV  240
_XP_041985024.1_  199  LLCERIT----------K-ED---ISVVFF-ERGDG--------DRIVWEKSAHVM-----LVHRQYAIV  240
_XP_049868286.1_  198  LLCEKVT----------R-ED---IAVVFY-EERRDQGAGGGGCAAVVWEESANIV-----MVHKQVAIA  247
_XP_026314794.1_  198  LLCEKVT----------R-ED---VAVVLY-EERREVGAGGGGASCVVWEDNATVV-----MVHKQAAIA  247
_XP_047994271.1_  198  LLCEKVT----------R-DD---VDVVFS-QAFD---------DGSVWEASANIV-----LVHKQVAIA  238
_KAJ2950301.1_    195  LLCEKVT----------R-ED---IAVVFY-EERRMEGAGDGGASAVVWEAPANVI-----LVHKQYAIA  244
_XP_031784347.2_  193  LLCEKVS----------K-GD---IQVCFF-DENS------------TWKAIAEFSPP---KYHKQYALI  232
_KAG7305875.1_    197  LLCEKVA----------R-ED---ITVTFF-EKSG---------ERVTWQADAADV-----FVHKQVAIC  237
_WDD23886.1_      197  LLCEKVA----------R-ED---ITVTFF-EKSG---------ERVTWQADAADV-----FVHKQVAIC  237
```

```
_XP_048480424.1_  197  LLCEKVA----------R-ED---ITVTFF-EKSG---------ERVTWQADAADV-----FVHKQVAIC  237
_XP_048480423.1_  197  LLCEKVA----------R-ED---ITVTFF-EKSG---------ERVTWQADAADV-----FVHKQVAIC  237
_XP_048480422.1_  197  LLCEKVA----------R-ED---ITVTFF-EKSG---------ERVTWQADAADV-----FVHKQVAIC  237
_XP_048480421.1_  197  LLCEKVA----------R-ED---ITVTFF-EKSG---------ERVTWQADAADV-----FVHKQVAIC  237
_KAG7305876.1_    197  LLCEKVA----------R-ED---ITVTFF-EKSG---------ERVTWQADAADV-----FVHKQVAIC  237
_CAG9129242.1_    197  LLCEKVA----------R-ED---ITVTFF-EKSG---------ERVTWQADAADV-----FVHKQVAIC  237
_XP_048480420.1_  197  LLCEKVA----------R-ED---ITVTFF-EKSG---------ERVTWQADAADV-----FVHKQVAIC  237
_GBP53004.1_       71  LLCEKVT----------K-ED---IAVTFF-EKVG---------EQVTWQADADNV-----NVHKQVAIC  111
_XP_045534737.1_  198  LLCEKVT----------G-QD---TAVVFF-EKEN---------EEVVWEAAATNV-----HVHKQAAIA  238
_XP_013180060.1_  198  LLCEKVT----------G-QD---TAVVFF-EKAN---------EEVVWEAAATNV-----HVHKQAAIA  238
_XP_013180043.1_  198  LLCEKVT----------G-QD---TAVVFF-EKAN---------EEVVWEAAATNV-----HVHKQAAIA  238
_XP_013180068.1_  198  LLCEKVT----------G-QD---TAVVFF-EKAN---------EEVVWEAAATNV-----HVHKQAAIA  238
_XP_045534736.1_  198  LLCEKVT----------G-QD---TAVVFF-EKEN---------EEVVWEAAATNV-----HVHKQAAIA  238
_KPJ06840.1_      198  LLCEKVT----------G-QD---TAVVFF-EKEN---------EEVVWEAAATNV-----HVHKQAAIA  238
_KPJ05518.1_      198  LLCEKVT----------G-QD---TAVVFF-EKAN---------EEVVWEAAATNV-----HVHKQAAIA  238
_CAG4934519.1_    198  LLCEKVT----------R-ED---TAVVFF-EKEN---------EQVIWEAAANNV-----HVHKQVAIA  238
_XP_013149112.1_  198  LLCEKVT----------G-QD---TAVVFF-EKKN---------EKVVWEAAATNV-----HVHKQAAIA  238
_CAH2077165.1_    210  LLCEKVS----------R-DD---TAVVFF-EKEN---------EQVVWEAAATNV-----HVHKQAAIA  250
_CAH2077167.1_    210  LLCEKVS----------R-DD---TAVVFF-EKEN---------EQVVWEAAATNV-----HVHKQAAIA  250
_XP_037051415.1_  189  VFCSKVS----------Q-GD---IKVRFYRQNEN---------TMVEWEAWVQKPI----LVHRQAAIV  231
_XP_037051414.1_  187  VFCSNVT----------K-GD---LKVRFYREDEN---------GMLEWEAWVQKPI----IVHRQAAIV  229
_XP_022815079.1_  198  LLCEKVT----------R-ED---IEVVFY-QEED---------GIVVWEEIAIRI-----LVHKQVAIA  238
_CAB3516522.1_    198  LLCEKVT----------R-ED---IEVVFY-QEED---------GIIVWEEIAIRI-----LVHKQVAIA  238
_XP_050562120.1_  198  LLCEKVT----------R-ED---IEVVFY-QEED---------GIIVWEETAIRI-----LVHKQVAIA  238
_XP_050562121.1_  198  LLCEKVT----------R-ED---IEVVFY-QEED---------GIIVWEETAIRI-----LVHKQVAIA  238
_QHD56218.1_      198  LLCEKVT----------R-ED---IEVVFY-QEED---------GIVVWEETAIRI-----LVHKQVAIA  238
_AIA24466.1_      198  LLCEKVT----------R-ED---IEVVFY-QEED---------GIIVWEEMAIRI-----LVHKQVAIA  238
_XP_022815076.1_  198  LLCEKVT----------R-ED---IEVVFY-QEED---------GIVVWEEIAIRI-----LVHKQVAIA  238
_QHD56217.1_      198  LLCEKVT----------R-ED---IEVVFY-QEED---------GIVVWEETAIRI-----LVHKQVAIA  238
_XP_050562118.1_  198  LLCEKVT----------R-ED---IEVVFY-QEED---------GIIVWEETAIRI-----LVHKQVAIA  238
_AIA24467.1_      198  LLCEKVT----------R-ED---IEVVFY-QEED---------GIIVWEEMAIRI-----LVHKQVAIA  238
_XP_047019808.1_  198  LLCEKVT----------R-ED---IEVVFY-QEEN---------GIVVWEEMAIRI-----LVHKQVAIA  238
_AEO51736.1_      198  LLCEKVT----------R-ED---IEVVFY-QEEN---------GIVVWEEMAIRI-----LVHKQVAIA  238
_XP_049696680.1_  198  LLCEKVT----------R-ED---IEVVFY-QEEN---------GIVVWEEMAIRI-----LVHKQVAIA  238
_XP_049696682.1_  198  LLCEKVT----------R-ED---IEVVFY-QEEN---------GIVVWEEMAIRI-----LVHKQVAIA  238
_XP_049696676.1_  198  LLCEKVT----------R-ED---IEVVFY-QEEN---------GIVVWEEMAIRI-----LVHKQVAIA  238
_XP_026744931.1_  198  LLCEKVT----------R-ED---IEVVFY-QEEN---------GHVVWEEMAIRI-----LVHKQVAIA  238
_XP_026732895.1_  198  LLCEKVT----------R-ED---IEVVFY-QEEN---------GHVVWEEMAIRI-----LVHKQVAIA  238
_CAH0581650.1_    198  LLCEKVT----------R-ED---IEVVFY-QEEN---------GHVVWEEMAIRI-----LVHKQVAIA  238
```

```
_KAH9640014.1_    243  LLCEKVT----------R-ED---IEVVFY-QEED---------GIVVWEETAIRI-----LVHKQVAIA  283
_PZC78315.1_       72  LLCEKVT----------R-ED---IEVVFY-QEEN---------GIVVWEEMAIRI-----LVHKQVAIA  112
_KAF9795796.1_     72  LLCEK---------------------------EED---------GIIVWEETAIRI-----LVHKQVAIA  100
_AEC52926.1_      198  LLCEKVT----------R-ED---IAVVFY-LEDK---------NQVIWEETAIIL-----LVHKQVAIA  238
_KAH7940301.1_    183  VVCFLLC----------A-DD---IEVWFE-EERD---------GQTVWKERAELLPN---GVHKQVAIC  225
_ADK39025.1_      197  LLCEKVT----------R-ED---TVVVFY-QEDN---------NRVLWEEMAIII-----VVHKQYAIA  237
_XP_037298148.1_  197  LLCEKVT----------R-ED---TVVVFY-QEDN---------NRVLWEEMAIII-----VVHKQYAIA  237
_RVE50742.1_      198  LLCEKVT----------R-ED---TEIWFF-HED--------------WVEKVPPE-----FVHKQLAVV  233
_CAH0692665.1_    198  LLCEKVT----------R-ED---TEIWFF-HED--------------WVEKVPPE-----FVHKQLAVV  233
_XP_028166351.1_  197  LLCEKVT----------R-ED---TEVWFF-LEEN---------GAVVWSERGHID-----YVHKQLAIV  237
_XP_028166291.1_  197  LLCEKVT----------R-ED---TEVWFF-LEEN---------GAVVWSERGHID-----YVHKQLAIV  237
_CAB3235314.1_    198  LLCEKVT----------R-ED---TVVVFY-LEEN---------NMVVWEEPANTI-----LVHKQVAIA  238
_KAJ0175867.1_    199  LLCEKVT----------R-EE---VVVVFY-EDDNN--------DQILWEEPAITL-----LVHRQCAIA  240
_XP_028031027.1_  198  LLCEKVS----------K-ED---TAVIFY-QEVN---------EQIVWEETVTPS-----YVHKQVAVV  238
_NP_001166296.1_  198  LLCEKVS----------K-ED---TAVIFY-QEVN---------EQIVWEETVTPS-----YVHKQVAVV  238
_XP_037870194.1_  198  LLCEKVS----------K-ED---TAVIFY-QEVN---------EQIVWEETVTPS-----YVHKQVAVV  238
_XP_037870193.1_  198  LLCEKVS----------K-ED---TAVIFY-QEVN---------EQIVWEETVTPS-----YVHKQVAVV  238
_XP_028031025.1_  198  LLCEKVS----------K-ED---TAVIFY-QEVN---------EQIVWEETVTPS-----YVHKQVAVV  238
_XP_013187225.1_  196  LLCEKVN----------R-ED---TAVVFF-QEES---------GQLVWEERAHIE-----AVHKQVAIV  236
_XP_052755011.1_  196  LLCEKVN----------R-ED---TAVVFF-QEEN---------KRVVWEERAHIE-----AVHKLVAIV  236
_XP_053613182.1_  196  LLCEKVN----------R-ED---TAVVFY-QEEN---------GQEVWRERAHIE-----AVHKQVAIV  236
_XP_053613180.1_  196  LLCEKVN----------R-ED---TAVVFY-QEEN---------GQEVWRERAHIE-----AVHKQVAIV  236
_CAG9785863.1_    196  LLCEKVT----------R-ED---TEIWFF-LDD--------------WVEKVPPE-----FVHKQLAVV  231
_KAG6461275.1_    197  LLCEKVS---------------------------------------------------------------  203
_KAG6461281.1_    197  LLCEKVS---------------------------------------------------------------  203
_XP_037302137.1_  195  VLSEKVI----------R-KN---IVVVFY-QDDE----------QMWWEQEASIK-----RVHKQVTIL  234
_KAG6461269.1_    195  VLSEKVI----------R-KN---IVVVFY-QDDE----------QMWWEQEASIK-----RVHKQVTIL  234
_KAG4069896.1_    185  VFCNKVV----------K-GD---IKVRFY-RENEN--------AMVEWEAWVQRPI----IVHRQAAIV  227
_XP_044003989.1_  202  ILTEKIY----------K-TD---IEVWFY-QLNDE--------DDVIWKAPGIFEPE---DIHYQRAIT  245
_XP_045768240.1_  199  LLCERVT----------K-ED---VMVVFF-QKEG---------DRTVWEANANVV-----LVHRQYAVV  239
_KAG9509099.1_    203  MLCDKVS----------R-DD---IQIRFY-EERD---------GKLVWETYSEF------EIHKQVAIC  242
_XP_013118267.1_  195  LLCEKLP----------K-DD---IKIRFY-EEKD---------NKVIWEDYGEFQQT---DIHKQTA-A  236
_XP_005183026.2_  195  LLCEKLP----------K-DD---IKIRFY-EEKD---------GKVIWEDYGEFQQT---DIHKQTAIA  237
_XP_050303432.1_  211  LWCGDVK----------K-GD---IEVKFV-KENEN--------GEVVWEARATKIEV----QKKNSSIA  253
_TMW48950.1_      196  LLCEKVP----------K-DD---IKVRFF-EIRN---------NELYWEGFGEFQQT---DVHKQAAIT  238
_XP_037882085.1_  195  MLCEKVP----------K--D---IKIKFY-ELSKE--------GTIVWEDYAVFQHT---DIHKHTAIA  237
_KAI8126541.1_    197  MLCEKVP----------K-DD---IKIRFF-ELRN---------NEVYWEDVAQFQPT---DVHKQAAIT  239
_KAI8126540.1_    196  MLCEKVP----------K-DD---IKIRFF-ELRN---------NEVYWEDVAQFQPT---DVHKQAAIT  238
_XP_037811156.1_  196  LLCEKVP----------K-DD---IKIRFF-ELRN---------NEVYWEDFAQFQPT---DVHKQAAIT  238
```

```
_XP_046812595.1_  196  MLCEKVP----------K-DD---IKIRFF-ELRN---------NEVYWEDVAQFQPT---DVHKQAAIT  238
_XP_046812596.1_  196  MLCEKVP----------K-DD---IKIRFF-ELRN---------NEVYWEDVAQFQPT---DVHKQAAIT  238
_XP_046812591.1_  196  MLCEKVP----------K-DD---IKIRFF-ELRN---------NEVYWEDVAQFQPT---DVHKQAAIT  238
_XP_046812590.1_  196  MLCEKVP----------K-DD---IKIRFF-ELRN---------NEVYWEDVAQFQPT---DVHKQAAIT  238
_KNC34143.1_      196  MLCEKVP----------K-DD---IKIRFF-ELRN---------NEVYWEDVAQFQPT---DVHKQAAIT  238
_XP_046812589.1_  196  MLCEKVP----------K-DD---IKIRFF-ELRN---------NEVYWEDVAQFQPT---DVHKQAAIT  238
_KAI8126539.1_    196  MLCEKVP----------K-DD---IKIRFF-ELRN---------NEVYWEDVAQFQPT---DVHKQAAIT  238
_XP_037958917.1_  194  LLCEKIA----------K-DDVKLIKIRFY-ELGND--------NELIWEDYAEFQPT---DIHKQTAIA  240
_XP_037929595.1_  196  LLCEKIA----------K-NDIKTIKIRFY-ELNND--------NQLVWEDFAKFRPA---DIHKQTAIT  242
_XP_037957513.1_  194  LLCEKIA----------K-DDIKIIKIRFY-ELSND--------NQLVWEDFAEFLPT---DIHKQTAIA  240
_XP_037958115.1_  196  LLCEKIA----------K-NDIKTIKIRFY-ELNND--------NQLVWEDFAKFRPA---DIHKQTAIT  242
_XP_037958114.1_  196  LLCEKIA----------K-NDIKTIKIRFY-ELNND--------NQLVWEDFAKFRPA---DIHKQTAIT  242
_XP_037957511.1_  194  LLCEKIA----------K-DDIKIIKIRFY-ELSND--------NQLVWEDFAEFLPT---DIHKQTAIA  240
_XP_037958918.1_  194  LLCEKIA----------K-DDIKFIKIRFY-ELDND--------NQLIWEDYAEFQPT---DVHKQTAIA  240
_RXG71823.1_      169  -------------------KD---IKVRFF-EMKE---------NQVEWEAFGDFQSS---DVHKQVAIS  203
_KAB7494622.1_    146  -------------------ED---IKVRFF-EMKE---------NQVEWEAFGDFQSS---DVHKQVAIS  180
_KAI9584339.1_    195  LLCEKIV----------R-DD---ILIRFY-ETKN---------DQIIWEDYASFEIT---DVHKQTAIA  237
_XP_037884983.1_  195  LLCEKII----------K-HD---ISIRLY-EKKN---------DQIIWEDYAAFDFT---DVHKQTAIA  237
_XP_054744821.1_  194  LLCSKVN----------K-DD---VKIRFK-AIDN---------NQEVWHAYAEFEPS---NVHKQTAIA  236
_XP_053961676.1_  194  LLCSKVN----------K-DD---VKIRFK-AIDN---------NQEVWHAYAEFEPS---NVHKQTAIA  236
_XP_053961674.1_  194  LLCSKVN----------K-DD---VKIRFK-AIDN---------NQEVWHAYAEFEPS---NVHKQTAIA  236
_XP_004526634.2_  194  LLCSKVN----------K-DD---IRIRFK-AIDN---------NREVWYSYAEFEPS---NVHKQTAIV  236
_CAD6998451.1_    194  LLCSKVN----------K-DD---IRIRFK-AIDN---------NREVWYSYAEFEPS---NVHKQTAIV  236
_XP_012157894.1_  194  LLCSKVN----------K-DD---IRIRFK-AIDN---------NREVWYSYAEFEPS---NVHKQTAIV  236
_XP_028894493.1_  194  LLCSKVN----------K-DD---VRIRFK-AIDN---------NHEVWHAYADFEPS---NVHKQTAIV  236
_XP_036327693.1_  194  LLCSKVN----------K-DD---IKVRFK-AIDN---------NQEVWHAYADFEPS---NVHKQTAIA  236
_XP_017471333.1_  194  LLCSKVN----------K-DD---IKVRFK-AIDN---------NQEVWHAYADFEPS---NVHKQAAIA  236
_XP_039952608.1_  194  LLCSQVN----------K-DD---VKIRFK-AIDN---------NREVWHAYADFEPS---NVHKQTAIA  236
_XP_050320023.1_  194  LLCSQVN----------K-DD---VKIRFK-AIDN---------NREVWHAYADFEPS---NVHKQTAIA  236
_XP_036225572.1_  194  LLCSQVN----------K-DD---VKIRFK-AIDN---------NREVWHAYADFEPS---NVHKQTAIV  236
_XP_039952607.1_  194  LLCSQVN----------K-DD---VKIRFK-AIDN---------NREVWHAYADFEPS---NVHKQTAIA  236
_XP_036225564.1_  194  LLCSQVN----------K-DD---VKIRFK-AIDN---------NREVWHAYADFEPS---NVHKQTAIV  236
_XP_036225569.1_  194  LLCSQVN----------K-DD---VKIRFK-AIDN---------NREVWHAYADFEPS---NVHKQTAIV  236
_XP_050320021.1_  194  LLCSQVN----------K-DD---VKIRFK-AIDN---------NREVWHAYADFEPS---NVHKQTAIA  236
_XP_039952606.1_  194  LLCSQVN----------K-DD---VKIRFK-AIDN---------NREVWHAYADFEPS---NVHKQTAIA  236
_XP_011207860.1_  194  LLCSQVN----------K-DD---VRIRFK-AIDN---------NREVWHAYADFEPS---NVHKQTAIA  236
_XP_011207859.1_  194  LLCSQVN----------K-DD---VRIRFK-AIDN---------NREVWHAYADFEPS---NVHKQTAIA  236
_XP_029407466.1_  194  LLCSQVN----------K-DD---VRIRFK-AIDN---------NREVWHAYADFEPS---NVHKQTAIA  236
_XP_019847003.2_  194  LLCSQVN----------K-DD---VRIRFK-AIDN---------NREVWHAYADFEPS---NVHKQTAIA  236
```

```
_XP_014086488.2_   194   LLCSQVN----------K-DD---VKIRFK-AIDN---------NREVWHAYADFEPS---NVHKQTAIV   236
_XP_049317393.1_   194   LLCSQVN----------K-DD---VRIRFK-AIDN---------NREVWHAYADFEPS---NVHKQTAIA   236
_XP_039952604.1_   194   LLCSQVN----------K-DD---VKIRFK-AIDN---------NREVWHAYADFEPS---NVHKQTAIA   236
_XP_018787557.1_   194   LLCSQVN----------K-DD---VKIRFK-AIDN---------NREVWHAYADFEPS---NVHKQTAIA   236
_OQR68950.1_       198   VLCDKVS----------K-DD---IEVYFY-QEDSA--------GNHLWEEKADIV-----SVHKQVAVC   239
_XP_003741763.1_   198   VLCDKVS----------K-DD---IEVFFY-EKDEA--------GRLIWEEKADNL-----TVHKQVAVC   239
_XP_022699297.1_   198   VLCDKVS----------K-DD---IEVYFY-QEDSA--------GNHLWEKKAEIQ-----SVHKQVAVC   239
_XP_022665229.1_   198   VLCDKVS----------K-DD---IEVYFY-QEDSA--------GNHLWEKKAEIQ-----SVHKQVAVC   239
_XP_022665225.1_   198   VLCDKVS----------K-DD---IEVYFY-QEDSA--------GNHLWEKKAEIQ-----SVHKQVAVC   239
_XP_022665224.1_   198   VLCDKVS----------K-DD---IEVYFY-QEDSA--------GNHLWEKKAEIQ-----SVHKQVAVC   239
_XP_033219687.1_   196   LLCGKVL----------K-DD---IQVRFF-EMRD---------NWLFWDSYGEFSPT---DVHKQTAIV   238
_XP_033227990.1_   196   LLCGKVL----------K-DD---IQVRFF-EMRD---------NSLFWHDYGEFLPT---DVHKQTAIV   238
_XP_043466013.1_   195   LLCGKIS----------R-DD---IRIRFF-EVRN---------DWLFWEGFADFQPV---NVHKQVAIV   237
_XP_051172671.1_   195   LLCGKIS----------R-DD---IQVRFF-EVRN---------EWLFWEGFGEFQPV---NVHKQVAIV   237
_CAD7643228.1_     194   LLCDRVV----------K-DD---IRIRFW-EERD---------DCLYWEAFADFKAS---DIHKHFAIS   236
_XP_054157157.1_   197   LLCDRVV----------K-DD---IQVRFY-EETAD--------GILVWEAYAEFRAQ---DIHKHFAIS   240
_CAD7645633.1_     195   LLCDRVI----------K-DD---IQIRFF-EERE---------DAVYWEAFADFKAS---DIHKHFAIS   237
_CAD7632611.1_     136   LLCDRVV----------K-DD---IRIRFW-EERD---------DCLYWEAFADFKAS---DIHKHFAIS   178
_KAJ6223158.1_     194   MLCDRVL----------K-DD---IQIRFF-RNCPR-------SGQLIWERYAQFGPN---DIHKHFAIT   238
_KAI2804035.1_     194   MLCDRVL----------K-DD---IQIRFF-RNCPR-------SGQLIWERYAQFGPN---DIHKHFAIT   238
_KAH9398209.1_     186   MLCDRVL----------K-ED---IQIRFY-QTDCA-------TGQTVWERMAHFNAS---DIHKHFAIT   230
_GFY77286.1_       196   LLCDQVN----------K-DD---IQILFY-EEND---------NKIIWCDHGKFAPH---HVHRKAAIS   238
_GFQ73388.1_       196   LLCDQVN----------K-DD---IQILFY-EEND---------NKIVWCDHGKFAPH---HVHRKAAIS   238
_GFT25575.1_       196   LLCDQVN----------K-DD---IQILFY-EEND---------NKVVWCDHGKFAPH---HVHRKAAIS   238
_KAF8763655.1_     196   LLCDQVN----------K-DD---IQILFF-EEND---------NKIVWCDHGKFAPH---HVHRKAAIS   238
_GFX76677.1_       196   LLCDQVN----------K-DD---IQILFY-EEND---------NKIVWCDHGKFAPH---HVHRKAAIS   238
_XP_035207052.1_   196   LLCDQVN----------K-DD---IQIIFY-EEND---------GKVTWSALGKFAPH---HVHRKAAIS   238
_KFM74898.1_       196   LLCDQVN----------K-DD---IQIIFY-EENE---------GKITWSALGKFAPH---HVHRKAAIS   238
_XP_035207059.1_   196   LLCDQVN----------K-DD---IQIIFY-EEND---------GKVTWSALGKFAPH---HVHRKAAIS   238
_XP_035207066.1_   196   LLCDQVN----------K-DD---IQIIFY-EEND---------GKVTWSALGKFAPH---HVHRKAAIS   238
_BBD75287.1_       196   LLCDQVN----------K-DD---IQILFY-EEVN---------NKIIWCANGKFAPH---HVHRKAAIS   238
_XP_042900952.1_   196   LLCDQVN----------K-DD---IQILFY-EEVN---------NKIIWCANGKFAPH---HVHRKAAIS   238
_KAG8201816.1_     195   LLCDQVN----------K-DD---IQILVY-EKNQ---------DKVTWCDQGKFAPH---HVHRKSAIC   237
_GIX87435.1_       196   ENLQTVNFVNAKTWLIVA-DD---IQILFF-EEND---------NKVVWCDHGKFAPH---HVHRKAAIS   248
_GBL81029.1_       196   LLCDQVN----------K-DD---IQILFY-EEND---------NKVVWCDHGKFAPH---HVHRKAAIS   238
_GIY01087.1_       196   LLCDQVN----------K-DD---IQILFF-EEND---------NKVVWCDHGKFAPH---HVHRKAAIS   238
_XP_054715436.1_   196   LLCNQVN----------K-DD---IQILFY-EESG---------SNIIWSGQGLFSPH---HVHRKAAIT   238
_XP_044576112.1_   193   LLCDKVS----------K-DN---INIVFF-EQKN---------GKNVWEAPAKFNPS---DVFRQVSIS   235
_XP_008556435.3_   192   LLCERVA----------K-DD---ISIVFF-EKRN---------NKIVWEGLAKFNSN---DVYRQISIA   234
```

```
_CAD6234446.1_     193  LLCDKVP----------K-DN---INIVFF-EQKN---------GQNVWEAPAKFNPS---DVFRQVSIS  235
_XP_044284598.1_   197  LLCDKVQ----------K-DD---IEVRFF-MND--------------WEAKGVFSQA---DVHRQVAIV  234
_XP_033794511.1_   197  LLCDRVQ----------K-DD---IEVRFF-FNT--------------WEAKGVFSQA---DVHRQVAIV  234
_XP_033794512.1_   197  LLCDRVQ----------K-DD---IEVRFF-FNT--------------WEAKGVFSQA---DVHRQVAIV  234
_XP_029449578.1_   197  LLCDKVQ----------K-DD---IEVRFF-SST--------------WEAKGVFSQA---DVHRQVAIV  234
_XP_030052048.1_   197  LLCDKVQ----------K-DD---IEVRFF-SNT--------------WEAKGVFSQA---DVHRQVAIV  234
_XP_007423639.1_   197  LLCDKVQ----------K-DD---IEVRFV-MND--------------WEAKGIFSQA---DVHRQVAIV  234
_XP_039203483.1_   197  LLCDKVQ----------K-DD---IEVRFF-MNN--------------WEAKGIFSQA---DVHRQVAIV  234
_XP_034283206.1_   197  LLCDKVQ----------K-DD---IEVRFF-MND--------------WEAKGIFSQA---DVHRQVAIV  234
_XP_033001023.1_   197  LLCDKVQ----------K-DD---IEVRFV-MDE--------------WEAKGIFSQA---DVHRQVAIV  234
_XP_053239675.1_   197  LLCDKVQ----------K-DD---IEVRFV-MNE--------------WEAKGIFSQA---DVHRQVAIV  234
_XP_034966804.1_   197  LLCDKVQ----------K-DD---IEVRFV-MNE--------------WEAKGIFSQA---DVHRQVAIV  234
_XP_028580982.1_   186  LLCDKVQ----------K-DD---IEVRFV-MNE--------------WEAKGIFSQA---DVHRQVAIV  223
_XP_042302822.1_   198  LLCDKVQ----------K-DD---IEVRFV-MDD--------------WEAKGIFSQA---DVHRQVAIV  235
_XP_015280979.1_   186  LLCDKVQ----------K-DD---IEVRFS-NNE--------------WEGKGIFSQA---DVHRQVAIV  223
_XP_048363544.1_   197  LLCDKVQ----------K-DD---IEVRFS-NNE--------------WEAKGIFSQA---DVHRQVAIV  234
_KAF7252806.1_     171  LLCDKVQ----------K-DD---IEVRFF-MND--------------WEAKGVFSQA---DVHRQVAIV  208
_XP_034958398.1_    76  LLCDKVQ----------K----------------------------------------------------   83
_CAD5116312.1_     199  MLTDKLT---------SK-DD---VEIIFE-CKD--------------WMDKGNFGST---DVHHQVAIV  237
_KAJ1519398.1_     189  LLCGRVR---------ER-EV---PEVWFY-EEADG--------VHT-WSARA--DVL---AVHNNTAIA  230
_XP_053103001.1_   197  LLCDKVQ----------K-DD---IEVRFV-MNE--------------WEAKGVFSQA---DVHRQVAIV  234
_XP_032070730.1_   197  LLCDKVQ----------K-DD---IEVRFF-MND--------------WEAKGIFSQA---DVHRQVAIV  234
_XP_013922116.1_   197  LLCDKVQ----------K-DD---IEVRFF-MND--------------WEAKGIFSQA---DVHRQVAIV  234
_XP_015667999.1_   197  LLCDKVQ----------K-DD---IEVRFF-MNN--------------WEAKGIFSQA---DVHRQVAIV  234
_XP_026551846.1_   197  LLCDKVQ----------K-DD---IEVRFF-MND--------------WEARGIFSQA---DVHRQVAIV  234
_XP_026532518.1_   197  LLCDKVQ----------K-DD---IEVRFF-MND--------------WEDRGIFSQA---DVHRQVAIV  234
_KAJ7341301.1_     197  LLCDKVQ----------K-DD---IEVRFA-MNE--------------WEAKGIFSQA---DVHRQVAIV  234
_KAJ6667038.1_     186  LLCDKVQ----------K-DD---IEVRFF-MNE--------------WEAKGIFSQA---DVHRQVAIV  223
_XP_003227818.2_   186  LLCDKVL----------K-DD---IEVRFV-MND--------------WEAKGVFSQA---DVHRQVAIV  223
_XP_020633036.1_   199  LLCDKVQ----------K-DD---IEVRFV-MND--------------WEAKGIFSQA---DVHRQVAIV  236
_XP_054842059.1_   197  LLCDKVQ----------K-DD---IEVRFS-INE--------------WEAKGIFSQA---DVHRQVAIV  234
_XP_005238641.2_   186  LLCDKVQ----------K-DD---IEVRFV-LDN--------------WEAKGSFSQA---DVHRQVAIV  223
_XP_005445671.2_   186  LLCDKVQ----------K-DD---IEVRFV-LDN--------------WEAKGSFSQA---DVHRQVAIV  223
_XP_054050029.1_   186  LLCDKVQ----------K-DD---IEVRFV-LDN--------------WEAKGSFSQA---DVHRQVAIV  223
_XP_013810845.1_   186  LLCDKVQ----------K-DD---IEVRFV-LDN--------------WEAKGSFSQA---DVHRQVAIV  223
_XP_026702002.1_   186  LLCDKVQ----------K-DD---IEVRFV-LDN--------------WEAKGSFSQA---DVHRQVAIV  223
_XP_025892242.1_   186  LLCDKVQ----------K-DD---IEVRFV-LDN--------------WEAKGSFSQA---DVHRQVAIV  223
_XP_005507389.1_   186  LLCDKVQ----------K-DD---IEVRFV-LDN--------------WEAKGSFSQA---DVHRQVAIV  223
_XP_032911247.1_   186  LLCDKVQ----------K-DD---IEVRFV-LDN--------------WEAKGSFSQA---DVHRQVAIV  223
```

```
_XP_031959657.1_  186  LLCDKVQ----------K-DD---IEVRFV-LDN--------------WEAKGSFSQA---DVHRQVAIV  223
_XP_041905291.1_  186  LLCDKVQ----------K-DD---IEVRFV-LDN--------------WEAKGSFSQA---DVHRQVAIV  223
_XP_051648836.1_  186  LLCDKVQ----------K-DD---IEVRFV-LDN--------------WEAKGSFSQA---DVHRQVAIV  223
_XP_054239488.1_  186  LLCDKVQ----------K-DD---IEVRFV-LDN--------------WEAKGSFSQA---DVHRQVAIV  223
_XP_039914164.1_  186  LLCDKVQ----------K-DD---IEVRFV-LDN--------------WEAKGSFSQA---DVHRQVAIV  223
_XP_005422689.1_  186  LLCDKVQ----------K-DD---IEVRFV-LDN--------------WEAKGSFSQA---DVHRQVAIV  223
_KAF2987757.1_    186  LLCDKVQ----------K-DD---IEVRFV-LDN--------------WEAKGSFSQA---DVHRQVAIV  223
_XP_038665087.1_  197  LLCDKVQ----------K-DD---IEVRFF-TQN--------------WEAKGSFSQA---DVHRQVAIV  234
_GCB59945.1_      197  LLCDKVQ----------K-DD---IEVRFF-TQN--------------WEAKGSFSQA---DVHRQVAIV  234
_XP_041065616.1_  193  LLCDKVQ----------K-DD---IEIRFF-THN--------------WEAKGSFSQA---DVHRQVAIV  230
_XP_007890564.1_  197  LLCDKVQ----------K-DD---IEVRFF-THN--------------WEAKGSFSQA---DVHRQVAIV  234
_XP_051868609.1_  189  LLCDKVQ----------K-DD---IEVRFF-TPN--------------WEAKGSFSQA---DVHRQVAIV  226
_XP_032881051.1_  196  LLCDKVQ----------K-DD---IEVRFF-TTN--------------WEAKGSFSQA---DVHRQVAIV  233
_XP_048456938.1_  106  LLCDKVQ----------K-DD---IEVRFF-THD--------------WEAKGSFSQA---DVHRQVAIV  143
_KAJ1155552.1_    197  LLCDKVQ----------K-DD---IEVRFF-TSE--------------WEGKGTFSQA---DVHRQVAIV  234
_KAH0622877.1_     66  LLCDKVQ----------K-D--------------------------------------------------   74
_CAI5653329.1_    197  LLCDKVQ----------K-DD---IEVRFF-SPDG-------------WEAKGSFSQA---DVHRQVAIV  235
_CAI5653328.1_    197  LLCDKVQ----------K-DD---IEVRFF-SPDG-------------WEAKGSFSQA---DVHRQVAIV  235
_NXS54806.1_      204  LLCDKVQ----------K-DD---IEVRFV-LDN--------------WEAKGSFSQA---DVHRQVAIV  241
_XP_009504395.1_  194  LLCDKVQ----------K-DD---IEVRFV-LDN--------------WEAKGSFSQA---DVHRQVAIV  231
_NXJ41381.1_      197  LLCDKVQ----------K-DD---IEVRFV-LDN--------------WEAKGSFSQA---DVHRQVAIV  234
_NXT24719.1_      197  LLCDKVQ----------K-DD---IEVRFV-LDN--------------WEAKGSFSQA---DVHRQVAIV  234
_NXK23502.1_      197  LLCDKVQ----------K-DD---IEVRFV-LDN--------------WEAKGSFSQA---DVHRQVAIV  234
_NXK03070.1_      197  LLCDKVQ----------K-DD---IEVRFV-LDN--------------WEAKGSFSQA---DVHRQVAIV  234
_XP_037260944.1_  197  LLCDKVQ----------K-DD---IEVRFV-LDN--------------WEAKGSFSQA---DVHRQVAIV  234
_XP_040467369.1_  197  LLCDKVQ----------K-DD---IEVRFV-LDN--------------WEAKGSFSQA---DVHRQVAIV  234
_NXY71491.1_      197  LLCDKVQ----------K-DD---IEVRFV-LDN--------------WEAKGSFSQA---DVHRQVAIV  234
_NXN54417.1_      197  LLCDKVQ----------K-DD---IEVRFV-LDN--------------WEAKGSFSQA---DVHRQVAIV  234
_NXX19490.1_      197  LLCDKVQ----------K-DD---IEVRFV-LDN--------------WEAKGSFSQA---DVHRQVAIV  234
_NXL47199.1_      197  LLCDKVQ----------K-DD---IEVRFV-LDN--------------WEAKGSFSQA---DVHRQVAIV  234
_XP_050749583.1_  197  LLCDKVQ----------K-DD---IEVRFV-LDN--------------WEAKGSFSQA---DVHRQVAIV  234
_NXD76242.1_      197  LLCDKVQ----------K-DD---IEVRFV-LDN--------------WEAKGSFSQA---DVHRQVAIV  234
_KFZ56827.1_      197  LLCDKVQ----------K-DD---IEVRFV-LDN--------------WEAKGSFSQA---DVHRQVAIV  234
_NXH67691.1_      197  LLCDKVQ----------K-DD---IEVRFV-LDN--------------WEAKGSFSQA---DVHRQVAIV  234
_NXS45828.1_      197  LLCDKVQ----------K-DD---IEVRFV-LDN--------------WEAKGSFSQA---DVHRQVAIV  234
_KFP52034.1_      197  LLCDKVQ----------K-DD---IEVRFV-LDN--------------WEAKGSFSQA---DVHRQVAIV  234
_NWH44485.1_      197  LLCDKVQ----------K-DD---IEVRFV-LDN--------------WEAKGSFSQA---DVHRQVAIV  234
_NWW85416.1_      197  LLCDKVQ----------K-DD---IEVRFV-LDN--------------WEAKGSFSQA---DVHRQVAIV  234
_NWU66320.1_      197  LLCDKVQ----------K-DD---IEVRFV-LDN--------------WEAKGSFSQA---DVHRQVAIV  234
```

```
_XP_025919906.1_  197  LLCDKVQ----------K-DD---IEVRFV-LDN--------------WEAKGSFSQA---DVHRQVAIV  234
_KAF1483947.1_    197  LLCDKVQ----------K-DD---IEVRFV-LDN--------------WEAKGSFSQA---DVHRQVAIV  234
_NXQ87688.1_      197  LLCDKVQ----------K-DD---IEVRFV-LDN--------------WEAKGSFSQA---DVHRQVAIV  234
_NXX07562.1_      197  LLCDKVQ----------K-DD---IEVRFV-LDN--------------WEAKGSFSQA---DVHRQVAIV  234
_XP_009280788.2_  197  LLCDKVQ----------K-DD---IEVRFV-LDN--------------WEAKGSFSQA---DVHRQVAIV  234
_XP_014821857.1_  197  LLCDKVQ----------K-DD---IEVRFV-LDN--------------WEAKGSFSQA---DVHRQVAIV  234
_NWU51443.1_      197  LLCDKVQ----------K-DD---IEVRFV-LDN--------------WEAKGSFSQA---DVHRQVAIV  234
_NXV35521.1_      197  LLCDKVQ----------K-DD---IEVRFV-LDN--------------WEAKGSFSQA---DVHRQVAIV  234
_KFV80780.1_      197  LLCDKVQ----------K-DD---IEVRFV-LDN--------------WEAKGSFSQA---DVHRQVAIV  234
_XP_032039522.1_  197  LLCDKVQ----------K-DD---IEVRFV-LDN--------------WEAKGSFSQA---DVHRQVAIV  234
_NWT43027.1_      197  LLCDKVQ----------K-DD---IEVRFV-LDN--------------WEAKGSFSQA---DVHRQVAIV  234
_KAF1651581.1_    197  LLCDKVQ----------K-DD---IEVRFV-LDN--------------WEAKGSFSQA---DVHRQVAIV  234
_NXU26374.1_      197  LLCDKVQ----------K-DD---IEVRFV-LDN--------------WEAKGSFSQA---DVHRQVAIV  234
_NWX46229.1_      197  LLCDKVQ----------K-DD---IEVRFV-LDN--------------WEAKGSFSQA---DVHRQVAIV  234
_PKU40340.1_      197  LLCDKVQ----------K-DD---IEVRFV-LDN--------------WEAKGSFSQA---DVHRQVAIV  234
_XP_027309078.1_  197  LLCDKVQ----------K-DD---IEVRFV-LDN--------------WEAKGSFSQA---DVHRQVAIV  234
_KAF1573683.1_    197  LLCDKVQ----------K-DD---IEVRFV-LDN--------------WEAKGSFSQA---DVHRQVAIV  234
_KAF1578928.1_    197  LLCDKVQ----------K-DD---IEVRFV-LDN--------------WEAKGSFSQA---DVHRQVAIV  234
_NXN47104.1_      197  LLCDKVQ----------K-DD---IEVRFV-LDN--------------WEAKGSFSQA---DVHRQVAIV  234
_NXV20393.1_      197  LLCDKVQ----------K-DD---IEVRFV-LDN--------------WEAKGSFSQA---DVHRQVAIV  234
_KAF1551267.1_    197  LLCDKVQ----------K-DD---IEVRFV-LDN--------------WEAKGSFSQA---DVHRQVAIV  234
_KAF1406210.1_    197  LLCDKVQ----------K-DD---IEVRFV-LDN--------------WEAKGSFSQA---DVHRQVAIV  234
_NXV43201.1_      197  LLCDKVQ----------K-DD---IEVRFV-LDN--------------WEAKGSFSQA---DVHRQVAIV  234
_NWQ98669.1_      197  LLCDKVQ----------K-DD---IEVRFV-LDN--------------WEAKGSFSQA---DVHRQVAIV  234
_XP_052639968.1_  197  LLCDKVQ----------K-DD---IEVRFV-LDN--------------WEAKGSFSQA---DVHRQVAIV  234
_XP_010573143.1_  197  LLCDKVQ----------K-DD---IEVRFV-LDN--------------WEAKGSFSQA---DVHRQVAIV  234
_KAF1479604.1_    197  LLCDKVQ----------K-DD---IEVRFV-LDN--------------WEAKGSFSQA---DVHRQVAIV  234
_NXG89109.1_      197  LLCDKVQ----------K-DD---IEVRFV-LDN--------------WEAKGSFSQA---DVHRQVAIV  234
_KFQ89757.1_      197  LLCDKVQ----------K-DD---IEVRFV-LDN--------------WEAKGSFSQA---DVHRQVAIV  234
_NWZ21787.1_      197  LLCDKVQ----------K-DD---IEVRFV-LDN--------------WEAKGSFSQA---DVHRQVAIV  234
_NXI74048.1_      197  LLCDKVQ----------K-DD---IEVRFV-LDN--------------WEAKGSFSQA---DVHRQVAIV  234
_NWX76247.1_      197  LLCDKVQ----------K-DD---IEVRFV-LDN--------------WEAKGSFSQA---DVHRQVAIV  234
_NXF29788.1_      197  LLCDKVQ----------K-DD---IEVRFV-LDN--------------WEAKGSFSQA---DVHRQVAIV  234
_KAF1444142.1_    197  LLCDKVQ----------K-DD---IEVRFV-LDN--------------WEAKGSFSQA---DVHRQVAIV  234
_KFV20215.1_      197  LLCDKVQ----------K-DD---IEVRFV-LDN--------------WEAKGSFSQA---DVHRQVAIV  234
_NXI44657.1_      197  LLCDKVQ----------K-DD---IEVRFV-LDN--------------WEAKGSFSQA---DVHRQVAIV  234
_NXJ92321.1_      197  LLCDKVQ----------K-DD---IEVRFV-LDN--------------WEAKGSFSQA---DVHRQVAIV  234
_NWZ49902.1_      197  LLCDKVQ----------K-DD---IEVRFV-LDN--------------WEAKGSFSQA---DVHRQVAIV  234
_NWY59444.1_      197  LLCDKVQ----------K-DD---IEVRFV-LDN--------------WEAKGSFSQA---DVHRQVAIV  234
```

```
_KFW64019.1_       197  LLCDKVQ----------K-DD---IEVRFV-LDN--------------WEAKGSFSQA---DVHRQVAIV  234
_NXJ55634.1_       197  LLCDKVQ----------K-DD---IEVRFV-LDN--------------WEAKGSFSQA---DVHRQVAIV  234
_KAF1513372.1_     197  LLCDKVQ----------K-DD---IEVRFV-LDN--------------WEAKGSFSQA---DVHRQVAIV  234
_NXW14598.1_       197  LLCDKVQ----------K-DD---IEVRFV-LDN--------------WEAKGSFSQA---DVHRQVAIV  234
_NXC74247.1_       197  LLCDKVQ----------K-DD---IEVRFV-LDN--------------WEAKGSFSQA---DVHRQVAIV  234
_NXT57254.1_       197  LLCDKVQ----------K-DD---IEVRFV-LDN--------------WEAKGSFSQA---DVHRQVAIV  234
_XP_047916679.1_   197  LLCDKVQ----------K-DD---IEVRFV-LDN--------------WEAKGSFSQA---DVHRQVAIV  234
_XP_029879190.1_   197  LLCDKVQ----------K-DD---IEVRFV-LDN--------------WEAKGSFSQA---DVHRQVAIV  234
_NXH09241.1_       197  LLCDKVQ----------K-DD---IEVRFV-LDN--------------WEAKGSFSQA---DVHRQVAIV  234
_XP_040407374.1_   197  LLCDKVQ----------K-DD---IEVRFV-LDN--------------WEAKGSFSQA---DVHRQVAIV  234
_KFW76037.1_       197  LLCDKVQ----------K-DD---IEVRFV-LDN--------------WEAKGSFSQA---DVHRQVAIV  234
_KFP65945.1_       197  LLCDKVQ----------K-DD---IEVRFV-LDN--------------WEAKGSFSQA---DVHRQVAIV  234
_NXX55682.1_       197  LLCDKVQ----------K-DD---IEVRFV-LDN--------------WEAKGSFSQA---DVHRQVAIV  234
_XP_035178333.1_   197  LLCDKVQ----------K-DD---IEVRFV-LDN--------------WEAKGSFSQA---DVHRQVAIV  234
_NXE47317.1_       197  LLCDKVQ----------K-DD---IEVRFV-LDN--------------WEAKGSFSQA---DVHRQVAIV  234
_XP_035408877.1_   197  LLCDKVQ----------K-DD---IEVRFV-LDN--------------WEAKGSFSQA---DVHRQVAIV  234
_KGL94141.1_       197  LLCDKVQ----------K-DD---IEVRFV-LDN--------------WEAKGSFSQA---DVHRQVAIV  234
_NXN68403.1_       197  LLCDKVQ----------K-DD---IEVRFV-LDN--------------WEAKGSFSQA---DVHRQVAIV  234
_NXW28331.1_       197  LLCDKVQ----------K-DD---IEVRFV-LDN--------------WEAKGSFSQA---DVHRQVAIV  234
_XP_049657509.1_   197  LLCDKVQ----------K-DD---IEVRFV-LDN--------------WEAKGSFSQA---DVHRQVAIV  234
_XP_049657511.1_   197  LLCDKVQ----------K-DD---IEVRFV-LDN--------------WEAKGSFSQA---DVHRQVAIV  234
_NXG30098.1_       197  LLCDKVQ----------K-DD---IEVRFV-LDN--------------WEAKGSFSQA---DVHRQVAIV  234
_NXV86645.1_       197  LLCDKVQ----------K-DD---IEVRFV-LDN--------------WEAKGSFSQA---DVHRQVAIV  234
_NXX83200.1_       197  LLCDKVQ----------K-DD---IEVRFV-LDN--------------WEAKGSFSQA---DVHRQVAIV  234
_NXI59898.1_       197  LLCDKVQ----------K-DD---IEVRFV-LDS--------------WEAKGSFSQA---DVHRQVAIV  234
_NWU89521.1_       197  LLCDKVQ----------K-DD---IEVRFV-LDN--------------WEAKGSFSQA---DVHRQVAIV  234
_NXK45490.1_       197  LLCDKVQ----------K-DD---IEVRFV-LDN--------------WEAKGSFSQA---DVHRQVAIV  234
_NWR55599.1_       197  LLCDKVQ----------K-DD---IEVRFV-LDN--------------WEAKGSFSQA---DVHRQVAIV  234
_NXL37824.1_       197  LLCDKVQ----------K-DD---IEVRFV-LDN--------------WEAKGSFSQA---DVHRQVAIV  234
_NWI33553.1_       197  LLCDKVQ----------K-DD---IEVRFV-LDN--------------WEAKGSFSQA---DVHRQVAIV  234
_KFQ74517.1_       197  LLCDKVQ----------K-DD---IEVRFV-LDN--------------WEAKGSFSQA---DVHRQVAIV  234
_NXV82662.1_       197  LLCDKVQ----------K-DD---IEVRFV-LDN--------------WEAKGSFSQA---DVHRQVAIV  234
_NWJ10087.1_       197  LLCDKVQ----------K-DD---IEVRFV-LDN--------------WEAKGSFSQA---DVHRQVAIV  234
_NXN24483.1_       197  LLCDKVQ----------K-DD---IEVRFV-LDN--------------WEAKGSFSQA---DVHRQVAIV  234
_NWR80208.1_       197  LLCDKVQ----------K-DD---IEVRFV-LDN--------------WEAKGSFSQA---DVHRQVAIV  234
_NXT37538.1_       197  LLCDKVQ----------K-DD---IEVRFV-LDN--------------WEAKGSFSQA---DVHRQVAIV  234
_XP_032844994.2_   197  LLCDKVQ----------K-DD---IEVRFV-LDN--------------WEAKGSFSQA---DVHRQVAIV  234
_NXW47649.1_       197  LLCDKVQ----------K-DD---IEVRFV-LDN--------------WEAKGSFSQA---DVHRQVAIV  234
_NXA40398.1_       197  LLCDKVQ----------K-DD---IEVRFV-LDN--------------WEAKGSFSQA---DVHRQVAIV  234
```

```
_NXW64893.1_      197  LLCDKVQ----------K-DD---IEVRFV-LDN--------------WEAKGSFSQA---DVHRQVAIV  234
_NXX89165.1_      197  LLCDKVQ----------K-DD---IEVRFV-LDN--------------WEAKGSFSQA---DVHRQVAIV  234
_NXQ96911.1_      197  LLCDKVQ----------K-DD---IEVRFV-LDN--------------WEAKGSFSQA---DVHRQVAIV  234
_KFP18273.1_      197  LLCDKVQ----------K-DD---IEVRFV-LDN--------------WEAKGSFSQA---DVHRQVAIV  234
_NXC40882.1_      197  LLCDKVQ----------K-DD---IEVRFV-LDN--------------WEAKGSFSQA---DVHRQVAIV  234
_NXE10305.1_      197  LLCDKVQ----------K-DD---IEVRFV-LDN--------------WEAKGSFSQA---DVHRQVAIV  234
_KFP88087.1_      197  LLCDKVQ----------K-DD---IEVRFV-LDN--------------WEAKGSFSQA---DVHRQVAIV  234
_NWW47871.1_      197  LLCDKVQ----------K-DD---IEVRFV-LDN--------------WEAKGSFSQA---DVHRQVAIV  234
_NXJ61595.1_      197  LLCDKVQ----------K-DD---IEVRFV-LDN--------------WEAKGSFSQA---DVHRQVAIV  234
_NXD13814.1_      197  LLCDKVQ----------K-DD---IEVRFV-LDN--------------WEAKGSFSQA---DVHRQVAIV  234
_KFR16961.1_      197  LLCDKVQ----------K-DD---IEVRFV-LDN--------------WEAKGSFSQA---DVHRQVAIV  234
_NWX24312.1_      197  LLCDKVQ----------K-DD---IEVRFV-LDN--------------WEAKGSFSQA---DVHRQVAIV  234
_NXA28267.1_      197  LLCDKVQ----------K-DD---IEVRFV-LDN--------------WEAKGSFSQA---DVHRQVAIV  234
_NXL66270.1_      197  LLCDKVQ----------K-DD---IEVRFV-LDN--------------WEAKGSFSQA---DVHRQVAIV  234
_NXA49976.1_      197  LLCDKVQ----------K-DD---IEVRFV-LDN--------------WEAKGSFSQA---DVHRQVAIV  234
_NXT77624.1_      197  LLCDKVQ----------K-DD---IEVRFV-LDN--------------WEAKGSFSQA---DVHRQVAIV  234
_KFQ92336.1_      197  LLCDKVQ----------K-DD---IEVRFV-LDN--------------WEAKGSFSQA---DVHRQVAIV  234
_NXE25013.1_      197  LLCDKVQ----------K-DD---IEVRFV-LDN--------------WEAKGSFSQA---DVHRQVAIV  234
_NXE92240.1_      197  LLCDKVQ----------K-DD---IEVRFV-LDN--------------WEAKGSFSQA---DVHRQVAIV  234
_NWH80943.1_      197  LLCDKVQ----------K-DD---IEVRFV-LDN--------------WEAKGSFSQA---DVHRQVAIV  234
_NWI19058.1_      197  LLCDKVQ----------K-DD---IEVRFV-LDN--------------WEAKGSFSQA---DVHRQVAIV  234
_NXG80610.1_      197  LLCDKVQ----------K-DD---IEVRFV-LDN--------------WEAKGSFSQA---DVHRQVAIV  234
_NWR01486.1_      197  LLCDKVQ----------K-DD---IEVRFV-LDN--------------WEAKGSFSQA---DVHRQVAIV  234
_NXK81337.1_      197  LLCDKVQ----------K-DD---IEVRFV-LDN--------------WEAKGSFSQA---DVHRQVAIV  234
_NXS90563.1_      197  LLCDKVQ----------K-DD---IEVRFV-LDN--------------WEAKGSFSQA---DVHRQVAIV  234
_NXC17430.1_      197  LLCDKVQ----------K-DD---IEVRFV-LDN--------------WEAKGSFSQA---DVHRQVAIV  234
_NXE77245.1_      197  LLCDKVQ----------K-DD---IEVRFV-LDN--------------WEAKGSFSQA---DVHRQVAIV  234
_KFQ29788.1_      198  LLCDKVQ----------K-DD---IEVRFV-LDN--------------WEAKGSFSQA---DVHRQVAIV  235
_KFO89327.1_      197  LLCDKVQ----------K-DD---IEVRFV-LDN--------------WEAKGSFSQA---DVHRQVAIV  234
_NXD64381.1_      197  LLCDKVQ----------K-DD---IEVRFV-LDN--------------WEAKGSFSQA---DVHRQVAIV  234
_NXY45081.1_      197  LLCDKVQ----------K-DD---IEVRFV-LDN--------------WEAKGSFSQA---DVHRQVAIV  234
_KGL72871.1_      197  LLCDKVQ----------K-DD---IEVRFV-LDN--------------WEAKGSFSQA---DVHRQVAIV  234
_XP_053919504.1_  197  LLCDKVQ----------K-DD---IEVRFV-LDN--------------WEAKGSFSQA---DVHRQVAIV  234
_NXL85459.1_      197  LLCDKVQ----------K-DD---IEVRFV-LDN--------------WEAKGSFSQA---DVHRQVAIV  234
_NXO02254.1_      197  LLCDKVQ----------K-DD---IEVRFV-LDN--------------WEAKGSFSQA---DVHRQVAIV  234
_NXJ76034.1_      197  LLCDKVQ----------K-DD---IEVRFV-LDN--------------WEAKGSFSQA---DVHRQVAIV  234
_NXM93491.1_      197  LLCDKVQ----------K-DD---IEVRFV-LDN--------------WEAKGSFSQA---DVHRQVAIV  234
_XP_030345800.1_  197  LLCDKVQ----------K-DD---IEVRFV-LDN--------------WEAKGSFSQA---DVHRQVAIV  234
_XP_030302544.1_  197  LLCDKVQ----------K-DD---IEVRFV-LDN--------------WEAKGSFSQA---DVHRQVAIV  234
```

_XP_030345801.1_	197	LLCDKVQ----------K-DD---IEVRFV-LDN--------------WEAKGSFSQA---DVHRQVAIV	234
KQK77724.1	197	LLCDKVQ----------K-DD---IEVRFV-LDN--------------WEAKGSFSQA---XVHRQVAIV	234
NXP09913.1	197	LLCDKVQ----------K-DD---IEVRFV-LDN--------------WEAKGSFSQA---DVHRQVAIV	234
NXR33018.1	197	LLCDKVQ----------K-DD---IEVRFV-LDN--------------WEAKGSFSQA---DVHRQVAIV	234
NXM76881.1	197	LLCDKVQ----------K-DD---IEVRFV-LDN--------------WEAKGSFSQA---DVHRQVAIV	234
NXS27423.1	197	LLCDKVQ----------K-DD---IEVRFV-LDN--------------WEAKGSFSQA---DVHRQVAIV	234
NXM55821.1	197	LLCDKVQ----------K-DD---IEVRFV-LDN--------------WEAKGSFSQA---DVHRQVAIV	234
_XP_054678500.1_	197	LLCDKVQ----------K-DD---IEVRFV-LDN--------------WEAKGSFSQA---DVHRQVAIV	234
_XP_015712248.1_	197	LLCDKVQ----------K-DD---IEVRFV-LDN--------------WEAKGSFSQA---DVHRQVAIV	234
NWX92058.1	197	LLCDKVQ----------K-DD---IEVRFV-LDN--------------WEAKGSFSQA---DVHRQVAIV	234
_XP_048793646.1_	197	LLCDKVQ----------K-DD---IEVRFV-LDN--------------WEAKGSFSQA---DVHRQVAIV	234
NXF04247.1	197	LLCDKVQ----------K-DD---IEVRFV-LDN--------------WEAKGSFSQA---DVHRQVAIV	234
_XP_042683331.1_	197	LLCDKVQ----------K-DD---IEVRFV-LDN--------------WEAKGSFSQA---DVHRQVAIV	234
NWV22147.1	197	LLCDKVQ----------K-DD---IEVRFV-LDN--------------WEAKGSFSQA---DVHRQVAIV	234
NXA04361.1	197	LLCDKVQ----------K-DD---IEVRFV-LDN--------------WEAKGSFSQA---DVHRQVAIV	234
_XP_010116198.1_	203	LLCDKVQ----------K-DD---IEVRFV-LDN--------------WEAKGSFSQA---DVHRQVAIV	240
NXG59741.1	197	LLCDKVQ----------K-DD---IEVRFV-LDN--------------WEAKGSFSQA---DVHRQVAIV	234
_XP_042735326.1_	197	LLCDKVQ----------K-DD---IEVRFV-LDN--------------WEAKGSFSQA---DVHRQVAIV	234
NXM29886.1	197	LLCDKVQ----------K-DD---IEVRFV-LDN--------------WEAKGSFSQA---DVHRQVAIV	234
_NP_001161198.2_	197	LLCDKVQ----------K-DD---IEVRFV-LDN--------------WEAKGSFSQA---DVHRQVAIV	234
_XP_031451029.1_	197	LLCDKVQ----------K-DD---IEVRFV-LDN--------------WEAKGSFSQA---DVHRQVAIV	234
NXR49262.1	197	LLCDKVQ----------K-DD---IEVRFV-LDN--------------WEAKGSFSQA---DVHRQVAIV	234
_XP_052555416.1_	197	LLCDKVQ----------K-DD---IEVRFV-LDN--------------WEAKGSFSQA---DVHRQVAIV	234
NWZ67085.1	197	LLCDKVQ----------K-DD---IEVRFV-LDN--------------WEAKGSFSQA---DVHRQVAIV	234
_P16236.2_	197	ILCDKVQ----------K-DD---IEVRFV-LDN--------------WEAKGSFSQA---DVHRQVAIV	234
_XP_050566010.1_	197	LLCDKVQ----------K-DD---IEVRFV-LDN--------------WEAKGSFSQA---DVHRQVAIV	234
NWS67030.1	197	LLCDKVQ----------K-DD---IEVRFV-LDN--------------WEAKGSFSQA---DVHRQVAIV	234
_XP_005143850.3_	197	LLCDKVQ----------K-DD---IEVRFV-LDN--------------WEAKGSFSQA---DVHRQVAIV	234
_XP_050566011.1_	197	LLCDKVQ----------K-DD---IEVRFV-LDN--------------WEAKGSFSQA---DVHRQVAIV	234
_XP_050566009.1_	197	LLCDKVQ----------K-DD---IEVRFV-LDN--------------WEAKGSFSQA---DVHRQVAIV	234
NWR30963.1	197	LLCDKVQ----------K-DD---IEVRFV-LDN--------------WEAKGSFSQA---DVHRQVAIV	234
NWS17896.1	197	LLCDKVQ----------K-DD---IEVRFV-LDN--------------WEAKGSFSQA---DVHRQVAIV	234
_XP_021248574.1_	197	LLCDKVQ----------K-DD---IEVRFV-LDN--------------WEAKGSFSQA---DVHRQVAIV	234
AAA48721.1	197	LLCDKVQ----------K-DD---IEVRFV-LDN--------------WEAKGSFSQA---DVHRQVAIV	234
NWI54208.1	197	LLCDKVQ----------K-DD---IEVRFV-LDN--------------WEAKGSFSQA---DVHRQVAIV	234
_XP_027509532.1_	197	LLCDKVQ----------K-DD---IEVRFV-LDN--------------WEAKGSFSQA---DVHRQVAIV	234
NXP71979.1	197	LLCDKVQ----------K-DD---IEVRFV-LDN--------------WEAKGSFSQA---DVHRQVAIV	234
NXX25352.1	197	LLCDKVQ----------K-DD---IEVRFV-LDN--------------WEAKGSFSQA---DVHRQVAIV	234
NXI18618.1	197	LLCDKVQ----------K-DD---IEVRFV-LDN--------------WEAKGSFSQA---DVHRQVAIV	234

```
_NXS09623.1_      197  LLCDKVQ----------K-DD---IEVRFV-LDN--------------WEAKGSFSQA---DVHRQVAIV  234
_NXU77980.1_      197  LLCDKVQ----------K-DD---IEVRFV-LDN--------------WEAKGSFSQA---DVHRQVAIV  234
_XP_027509533.1_  197  LLCDKVQ----------K-DD---IEVRFV-LDN--------------WEAKGSFSQA---DVHRQVAIV  234
_NWV71036.1_      197  LLCDKVQ----------K-DD---IEVRFV-LDN--------------WEAKGSFSQA---DVHRQVAIV  234
_NXF89482.1_      197  LLCDKVQ----------K-DD---IEVRFV-LDN--------------WEAKGSFSQA---DVHRQVAIV  234
_NWY39470.1_      197  LLCDKVQ----------K-DD---IEVRFV-LDN--------------WEAKGSFSQA---DVHRQVAIV  234
_NXT59960.1_      197  LLCDKVQ----------K-DD---IEVRFV-LDN--------------WEAKGSFSQA---DVHRQVAIV  234
_NWU74340.1_      197  LLCDKVQ----------K-DD---IEVRFV-LDN--------------WEAKGSFSQA---DVHRQVAIV  234
_NXB84481.1_      197  LLCDKVQ----------K-DD---IEVRFV-LDS--------------WEAKGSFSQA---DVHRQVAIV  234
_NWV73885.1_      197  LLCDKVQ----------K-DD---IEVRFV-LDN--------------WEAKGSFSQA---DVHRQVAIV  234
_OPJ58894.1_      197  LLCDKVQ----------K-DD---IEVRFV-LDN--------------WEAKGSFSQA---DVHRQVAIV  234
_XP_048152104.1_  197  LLCDKVQ----------K-DD---IEVRFV-LDN--------------WEAKGSFSQA---DVHRQVAIV  234
_XP_054036037.1_  197  LLCDKVQ----------K-DD---IEVRFV-LDN--------------WEAKGSFSQA---DVHRQVAIV  234
_NXR18149.1_      197  LLCDKVQ----------K-DD---IEVRFV-LDN--------------WEAKGSFSQA---DVHRQVAIV  234
_NXP97533.1_      197  LLCDKVQ----------K-DD---IEVRFV-LDN--------------WEAKGSFSQA---DVHRQVAIV  234
_NXJ05495.1_      197  LLCDKVQ----------K-DD---IEVRFV-LDN--------------WEAKGSFSQA---DVHRQVAIV  234
_XP_032538932.1_  197  LLCDKVQ----------K-DD---IEVRFV-LDN--------------WEAKGSFSQA---DVHRQVAIV  234
_NWI96387.1_      197  LLCDKVQ----------K-DD---IEVRFV-LDN--------------WEAKGSFSQA---DVHRQVAIV  234
_NXR65995.1_      197  LLCDKVQ----------K-DD---IEVRFV-LDN--------------WEAKGSFSQA---DVHRQVAIV  234
_NXK42398.1_      197  LLCDKVQ----------K-DD---IEVRFV-LDN--------------WEAKGSFSQA---DVHRQVAIV  234
_XP_032538931.1_  197  LLCDKVQ----------K-DD---IEVRFV-LDN--------------WEAKGSFSQA---DVHRQVAIV  234
_NXY33181.1_      197  LLCDKVQ----------K-DD---IEVRFV-LDN--------------WEAKGSFSQA---DVHRQVAIV  234
_XP_050171009.1_  197  LLCDKVQ----------K-DD---IEVRFV-LDN--------------WEAKGSFSQA---DVHRQVAIV  234
_NXO50939.1_      197  LLCDKVQ----------K-DD---IEVRFV-LDN--------------WEAKGSFSQA---DVHRQVAIV  234
_NWZ32937.1_      197  LLCDKVQ----------K-DD---IEVRFV-LDN--------------WEAKGSFSQA---DVHRQVAIV  234
_NXP01828.1_      197  LLCDKVQ----------K-DD---IEVRFV-LDN--------------WEAKGSFSQA---DVHRQVAIV  234
_NXL00458.1_      197  LLCDKVQ----------KADD---IEVRFV-LDN--------------WEAKGSFSQA---DVHRQVAIV  235
_XP_010401310.4_  197  LLCDKVQ----------K-DD---IEVRFV-LDN--------------WEAKGSFSQA---DVHRQVAIV  234
_NXH62149.1_      197  LLCDKVQ----------K-DD---IEVRFV-LDN--------------WEAKGSFSQA---DVHRQVAIV  234
_NXG43824.1_      197  LLCDKVQ----------K-DD---IEVRFV-LDN--------------WEAKGSFSQA---DVHRQVAIV  234
_XP_017677337.1_  197  LLCDKVQ----------K-DD---IEVRFV-LDN--------------WEAKGSFSQA---DVHRQVAIV  234
_NXU09156.1_      197  LLCDKVQ----------K-DD---IEVRFV-LDN--------------WEAKGSFSQA---DVHRQVAIV  234
_NXB38645.1_      197  LLCDKVQ----------K-DD---IEVRFV-LDN--------------WEAKGSFSQA---DVHRQVAIV  234
_XP_027594504.1_  197  LLCDKVQ----------K-DD---IEVRFV-LDN--------------WEAKGSFSQA---DVHRQVAIV  234
_NXM46269.1_      197  LLCDKVQ----------K-DD---IEVRFV-LDN--------------WEAKGSFSQA---DVHRQVAIV  234
_NWS79257.1_      197  LLCDKVQ----------K-DD---IEVRFV-LDN--------------WEAKGSFSQA---DVHRQVAIV  234
_NXB04839.1_      197  LLCDKVQ----------K-DD---IEVRFV-LDN--------------WEAKGSFSQA---DVHRQVAIV  234
_NXC62865.1_      197  LLCDKVQ----------K-DD---IEVRFV-LDN--------------WEAKGSFSQA---DVHRQVAIV  234
_NXN18277.1_      197  LLCDKVQ----------K-DD---IEVRFV-LDN--------------WEAKGSFSQA---DVHRQVAIV  234
```

```
_XP_027760039.1_  197  LLCDKVQ----------K-DD---IEVRFV-LDN--------------WEAKGSFSQA---DVHRQVAIV  234
_NXI90311.1_      197  LLCDKVQ----------K-DD---IEVRFV-LDN--------------WEAKGSFSQA---DVHRQVAIV  234
_NWY17003.1_      197  LLCDKVQ----------K-DD---IEVRFV-LDN--------------WEAKGSFSQA---DVHRQVAIV  234
_NXH97099.1_      197  LLCDKVQ----------K-DD---IEVRFV-LDN--------------WEAKGSFSQA---DVHRQVAIV  234
_NWS97308.1_      197  LLCDKVQ----------K-DD---IEVRFV-LDN--------------WEAKGSFSQA---DVHRQVAIV  234
_NWU45160.1_      197  LLCDKVQ----------K-DD---IEVRFV-LDN--------------WEAKGSFSQA---DVHRQVAIV  234
_NXQ43004.1_      197  LLCDKVQ----------K-DD---IEVRFV-LDN--------------WEAKGSFSQA---DVHRQVAIV  234
_XP_027549142.1_  197  LLCDKVQ----------K-DD---IEVRFV-LDN--------------WEAKGSFSQA---DVHRQVAIV  234
_XP_017933082.2_  197  LLCDKVQ----------K-DD---IEVRFV-LDN--------------WEAKGSFSQA---DVHRQVAIV  234
_NWV96326.1_      197  LLCDKVQ----------K-DD---IEVRFV-LDN--------------WEAKGSFSQA---DVHRQVAIV  234
_NXP57783.1_      197  LLCDKVQ----------K-DD---IEVRFV-LDN--------------WEAKGSFSQA---DVHRQVAIV  234
_NXY57955.1_      197  LLCDKVQ----------K-DD---IEVRFV-LDN--------------WEAKGSFSQA---DVHRQVAIV  234
_AAA99433.1_      197  LLCDKVQ----------K-DD---IEVRFV-LDN--------------WEAKGSFSQA---DVHRQVAIV  234
_NXY21218.1_      197  LLCDKVQ----------K-DD---IEVRFV-LDN--------------WEAKGSFSQA---DVHRQVAIV  234
_NXX50824.1_      197  LLCDKVQ----------K-DD---IEVRFV-LDN--------------WEAKGSFSQA---DVHRQVAIV  234
_XP_030124623.3_  197  LLCDKVQ----------K-DD---IEVRFV-LDN--------------WEAKGSFSQA---DVHRQVAIV  234
_NXA92235.1_      197  LLCDKVQ----------K-DD---IEVRFV-LDN--------------WEAKGSFSQA---DVHRQVAIV  234
_NWI05522.1_      197  LLCDKVQ----------K-DD---IEVRFV-LDN--------------WEAKGSFSQA---DVHRQVAIV  234
_NWX29009.1_      197  LLCDKVQ----------K-DD---IEVRFV-LDN--------------WEAKGSFSQA---DVHRQVAIV  234
_XP_027549144.1_  197  LLCDKVQ----------K-DD---IEVRFV-LDN--------------WEAKGSFSQA---DVHRQVAIV  234
_NWW35420.1_      197  LLCDKVQ----------K-DD---IEVRFV-LDN--------------WEAKGSFSQA---DVHRQVAIV  234
_NWV07969.1_      197  LLCDKVQ----------K-DD---IEVRFV-LDN--------------WEAKGSFSQA---DVHRQVAIV  234
_NXH42214.1_      197  LLCDKVQ----------K-DD---IEVRFV-LDN--------------WEAKGSFSQA---DVHRQVAIV  234
_NXQ18773.1_      197  LLCDKVQ----------K-DD---IEVRFV-LDN--------------WEAKGSFSQA---DVHRQVAIV  234
_NWX10672.1_      197  LLCDKVQ----------K-DD---IEVRFV-LDN--------------WEAKGSFSQA---DVHRQVAIV  234
_NXU50687.1_      197  LLCDKVQ----------K-DD---IEVRFV-LDN--------------WEAKGSFSQA---DVHRQVAIV  234
_KFU84784.1_      197  LLCDKVQ----------K-DD---IEVRFV-LDN--------------WEAKGSFSQA---DVHRQVAIV  234
_NXQ52438.1_      197  LLCDKVQ----------K-DD---IEVRFV-LDN--------------WEAKGSFSQA---DVHRQVAIV  234
_NWW24536.1_      197  LLCDKVQ----------K-DD---IEVRFV-LDN--------------WEAKGSFSQA---DVHRQVAIV  234
_NXF73449.1_      197  LLCDKVQ----------K-DD---IEVRFV-LDN--------------WEAKGSFSQA---DVHRQVAIV  234
_XP_050827906.1_  197  LLCDKVQ----------K-DD---IEVRFV-LDN--------------WEAKGSFSQA---DVHRQVAIV  234
_NWX65205.1_      197  LLCDKVQ----------K-DD---IEVRFV-LDN--------------WEAKGSFSQA---DVHRQVAIV  234
_KFW81749.1_      197  LLCDKVQ----------K-DD---IEVRFV-LDN--------------WEAKGSFSQA---DVHRQVAIV  234
_XP_014750593.1_  197  LLCDKVQ----------K-DD---IEVRFV-LDN--------------WEAKGSFSQA---DVHRQVAIV  234
_NXJ17066.1_      197  LLCDKVQ----------K-DD---IEVRFV-LDN--------------WEAKGSFSQA---DVHRQVAIV  234
_NWW67019.1_      197  LLCDKVQ----------K-DD---IEVRFV-LDN--------------WEAKGSFSQA---DVHRQVAIV  234
_NXH03360.1_      197  LLCDKVQ----------K-DD---IEVRFV-LDN--------------WEAKGSFSQA---DVHRQVAIV  234
_XP_037987525.1_  197  LLCDKVQ----------K-DD---IEVRFV-LDN--------------WEAKGSFSQA---DVHRQVAIV  234
_NXL74522.1_      197  LLCDKVQ----------K-DD---IEVRFV-LDN--------------WEAKGSFSQA---DVHRQVAIV  234
```

```
_NWQ60824.1_      197  LLCDKVQ----------K-DD---IEVRFV-LDN--------------WEAKGSFSQA---DVHRQVAIV  234
_NXR70971.1_      197  LLCDKVQ----------K-DD---IEVRFV-LDN--------------WEAKGSFSQA---DVHRQVAIV  234
_XP_051468947.1_  197  LLCDKVQ----------K-DD---IEVRFV-LDN--------------WEAKGSFSQA---DVHRQVAIV  234
_NXB50199.1_      197  LLCDKVQ----------K-DD---IEVRFV-LDN--------------WEAKGSFSQA---DVHRQVAIV  234
_NXP18380.1_      197  LLCDKVQ----------K-DD---IEVRFV-LDN--------------WEAKGSFSQA---DVHRQVAIV  234
_NXI84319.1_      197  LLCDKVQ----------K-DD---IEVRFV-LDN--------------WEAKGSFSQA---DVHRQVAIV  234
_NWH33757.1_      197  LLCDKVQ----------K-DD---IEVRFV-LDN--------------WEAKGSFSQA---DVHRQVAIV  234
_NWR99781.1_      197  LLCDKVQ----------K-DD---IEVRFV-LDN--------------WEAKGSFSQA---DVHRQVAIV  234
_NXW94404.1_      197  LLCDKVQ----------K-DD---IEVRFV-LDN--------------WEAKGSFSQA---DVHRQVAIV  234
_CAA27220.1_      197  LLCDKVQ----------K-DD---IEVRFV-LGN--------------WEAKGSFSQA---DVHRQVAIV  234
_XP_015477679.1_  197  LLCDKVQ----------K-DD---IEVRFV-LDN--------------WEAKGSFSQA---DVHRQVAIV  234
_NWS32597.1_      197  LLCDKVQ----------K-DD---IEVRFV-LDN--------------WEAKGSFSQA---DVHRQVAIV  234
_NXG22126.1_      197  LLCDKVQ----------K-DD---IEVRFV-LDN--------------WEAKGSFSQA---DVHRQVAIV  234
_NWY97127.1_      197  LLCDKVQ----------K-DD---IEVRFV-LDN--------------WEAKGSFSQA---DVHRQVAIV  234
_NXW80148.1_      197  LLCDKVQ----------K-DD---IEVRFV-LDN--------------WEAKGSFSQA---DVHRQVAIV  234
_NXC27367.1_      197  LLCDKVQ----------K-DD---IEVRFV-LDN--------------WEAKGSFSQA---DVHRQVAIV  234
_NWH83726.1_      197  LLCDKVQ----------K-DD---IEVRFV-LDN--------------WEAKGSFSQA---DVHRQVAIV  234
_NXA73512.1_      197  LLCDKVQ----------K-DD---IEVRFV-LDN--------------WEAKGSFSQA---DVHRQVAIV  234
_NWZ83230.1_      197  LLCDKVQ----------K-DD---IEVRFV-LDN--------------WEAKGSFSQA---DVHRQVAIV  234
_XP_005525572.2_  197  LLCDKVQ----------K-DD---IEVRFV-LDN--------------WEAKGSFSQA---DVHRQVAIV  234
_NXD20033.1_      197  LLCDKVQ----------K-DD---IEVRFV-LDN--------------WEAKGSFSQA---DVHRQVAIV  234
_NXE37177.1_      197  LLCDKVQ----------K-DD---IEVRFV-LDN--------------WEAKGSFSQA---DVHRQVAIV  234
_NWV44369.1_      197  LLCDKVQ----------K-DD---IEVRFV-LDN--------------WEAKGSFSQA---DVHRQVAIV  234
_NXC81400.1_      197  LLCDKVQ----------K-DD---IEVRFV-LDN--------------WEAKGSFSQA---DVHRQVAIV  234
_NXA67345.1_      197  LLCDKVQ----------K-DD---IEVRFV-LDN--------------WEAKGSFSQA---DVHRQVAIV  234
_NXB55155.1_      197  LLCDKVQ----------K-DD---IEVRFV-LDN--------------WEAKGSFSQA---DVHRQVAIV  234
_NXN81229.1_      197  LLCDKVQ----------K-DD---IEVRFV-LDN--------------WEAKGSFSQA---DVHRQVAIV  234
_NXM15601.1_      197  LLCDKVQ----------K-DD---IEVRFV-LDN--------------WEAKGSFSQA---DVHRQVAIV  234
_XP_054484850.1_  197  LLCDKVQ----------K-DD---IEVRFV-LDN--------------WEAKGSFSQA---DVHRQVAIV  234
_NWU02685.1_      197  LLCDKVQ----------K-DD---IEVRFV-LDN--------------WEAKGSFSQA---DVHRQVAIV  234
_XP_041273165.1_  197  LLCDKVQ----------K-DD---IEVRFV-LDN--------------WEAKGSFSQA---DVHRQVAIV  234
_NWW80397.1_      197  LLCDKVQ----------K-DD---IEVRFV-LDN--------------WEAKGSFSQA---DVHRQVAIV  234
_XP_036236540.1_  197  LLCDKVQ----------K-DD---IEVRFV-LDN--------------WEAKGSFSQA---DVHRQVAIV  234
_NWV50957.1_      197  LLCDKVQ----------K-DD---IEVRFV-LDN--------------WEAKGSFSQA---DVHRQVAIV  234
_NXT97287.1_      197  LLCDKVQ----------K-DD---IEVRFV-LDN--------------WEAKGSFSQA---DVHRQVAIV  234
_XP_023778876.1_  197  LLCDKVQ----------K-DD---IEVRFV-LDN--------------WEAKGSFSQA---DVHRQVAIV  234
_NXD42381.1_      197  LLCDKVQ----------K-DD---IEVRFV-LDN--------------WEAKGSFSQA---DVHRQVAIV  234
_NXK69295.1_      197  LLCDKVQ----------K-DD---IEVRFV-LDN--------------WEAKGSFSQA---DVHRQVAIV  234
_XP_049657510.1_  197  LLCDKVQ----------K-DD---IEVRFV-LDN--------------WEAKGSFSQA---DVHRQVAIV  234
```

```
_NXV62539.1_      197  LLCDKVQ----------K-DD---IEVRFV-LDN--------------WEAKGSFSQA---DVHRQVAIV  234
_NXB80231.1_      197  LLCDKVQ----------K-DD---IEVRFV-LDN--------------WEAKGSFSQA---DVHRQVAIV  234
_NWU15026.1_      197  LLCDKVQ----------K-DD---IEVRFV-LDN--------------WEAKGSFSQA---DVHRQVAIV  234
_NXL26104.1_      197  LLCDKVQ----------K-DD---IEVRFV-LDN--------------WEAKGSFSQA---DVHRQVAIV  234
_NWW05001.1_      197  LLCDKVQ----------K-DD---IEVRFV-LDN--------------WEAKGSFSQA---DVHRQVAIV  234
_RLW07261.1_      197  LLCDKVQ----------K-DD---IEVRFV-LDN--------------WEAKGSFSQA---DVHRQVAIV  234
_NXR96625.1_      197  LLCDKVQ----------K-DD---IEVRFV-LDN--------------WEAKGSFSQA---DVHRQVAIV  234
_XP_021383204.1_  197  LLCDKVQ----------K-DD---IEVRFV-LDN--------------WEAKGSFSQA---DVHRQVAIV  234
_NXQ25764.1_      197  LLCDKVQ----------K-DD---IEVRFV-LDN--------------WEAKGSFSQA---DVHRQVAIV  234
_NXO08412.1_      197  LLCDKVQ----------K-DD---IEVRFV-LDN--------------WEAKGSFSQA---DVHRQVAIV  234
_NXI32280.1_      197  LLCDKVQ----------K-DD---IEVRFV-LDN--------------WEAKGSFSQA---DVHRQVAIV  234
_NXG00288.1_      197  LLCDKVQ----------K-DD---IEVRFV-LDN--------------WEAKGSFSQA---DVHRQVAIV  234
_XP_041329879.1_  197  LLCDKVQ----------K-DD---IEVRFV-LDN--------------WEAKGSFSQA---DVHRQVAIV  234
_NXV03759.1_      197  LLCDKVQ----------K-DD---IEVRFV-LDN--------------WEAKGSFSQA---DVHRQVAIV  234
_NWT16975.1_      197  LLCDKVQ----------K-DD---IEVRFV-LDN--------------WEAKGSFSQA---DVHRQVAIV  234
_NWT67953.1_      197  LLCDKVQ----------K-DD---IEVRFV-LDN--------------WEAKGSFSQA---DVHRQVAIV  234
_XP_005042765.2_  197  LLCDKVQ----------K-DD---IEVRFV-LDN--------------WEAKGSFSQA---DVHRQVAIV  234
_XP_017600715.1_  197  LLCDKVQ----------K-DD---IEVRFV-LDN--------------WEAKGSFSQA---DVHRQVAIV  234
_NXD93279.1_      197  LLCDKVQ----------K-DD---IEVRFV-LDN--------------WEAKGSFSQA---DVHRQVAIV  234
_NXY02260.1_      197  LLCDKVQ----------K-DD---IEVRFV-LDN--------------WEAKGSFSQA---DVHRQVAIV  234
_NXO84677.1_      197  LLCDKVQ----------K-DD---IEVRFV-LDN--------------WEAKGSFSQA---DVHRQVAIV  234
_NXO64371.1_      197  LLCDKVQ----------K-DD---IEVRFV-LDN--------------WEAKGSFSQA---DVHRQVAIV  234
_NXO42022.1_      197  LLCDKVQ----------K-DD---IEVRFV-LDN--------------WEAKGSFSQA---DVHRQVAIV  234
_NWY65013.1_      197  LLCDKVQ----------K-DD---IEVRFV-LDN--------------WEAKGSFSQA---DVHRQVAIV  234
_NXP90563.1_      197  LLCDKVQ----------K-DD---IEVRFV-LDN--------------WEAKGSFSQA---DVHRQVAIV  234
_NWI73723.1_      197  LLCDKVQ----------K-DD---IEVRFV-LDN--------------WEAKGSFSQA---DVHRQVAIV  234
_NXH30511.1_      197  LLCDKVQ----------K-DD---IEVRFV-LDN--------------WEAKGSFSQA---DVHRQVAIV  234
_NWT77800.1_      197  LLCDKVQ----------K-DD---IEVRFV-LDN--------------WEAKGSFSQA---DVHRQVAIV  234
_NWT34209.1_      197  LLCDKVQ----------K-DD---IEVRFV-LDN--------------WEAKGSFSQA---DVHRQVAIV  234
_NWY29369.1_      197  LLCDKVQ----------K-DD---IEVRFV-LDN--------------WEAKGSFSQA---DVHRQVAIV  234
_NXQ73821.1_      197  LLCDKVQ----------K-DD---IEVRFV-LDN--------------WEAKGSFSQA---DVHRQVAIV  234
_XP_039572786.1_  197  LLCDKVQ----------K-DD---IEVRFV-LDN--------------WEAKGSFSQA---DVHRQVAIV  234
_XP_030802035.1_  197  LLCDKVQ----------K-DD---IEVRFV-LDN--------------WEAKGSFSQA---DVHRQVAIV  234
_NXE68211.1_      197  LLCDKVQ----------K-DD---IEVRFV-LDN--------------WEAKGSFSQA---DVHRQVAIV  234
_NXS80652.1_      197  LLCDKVQ----------K-DD---IEVRFV-LDN--------------WEAKGSFSQA---DVHRQVAIV  234
_NXO32431.1_      197  LLCDKVQ----------K-DD---IEVRFV-LDN--------------WEAKGSFSQA---DVHRQVAIV  234
_NWR17317.1_      197  LLCDKVQ----------K-DD---IEVRFV-LDN--------------WEAKGSFAQA---DVHRQVAIV  234
_NWZ89732.1_      197  LLCDKVQ----------K-DD---IEVRFV-LDN--------------WEAKGSFSQA---DVHRQVAIV  234
_XP_032039523.1_  197  LLCDKVQ----------K-DD---IEVRFV-LDN--------------WEAKGSFSQA---DVHRQVAIV  234
```

```
_KFO04362.1_       197  LLCDKVQ----------K-DD---IEVRFV-LDN--------------WEAKGSFSQA---DVHRQVAIV  234
_XP_054146213.1_   197  LLCDKVQ----------K-DD---IEVRFV-LDN--------------WEAKGSFSQA---DVHRQVAIV  234
_XP_005486916.2_   197  LLCDKVQ----------K-DD---IEVRFV-LDN--------------WEAKGSFSQA---DVHRQVAIV  234
_KAJ7422946.1_     197  LLCDKVQ----------K-DD---IEVRFV-LDN--------------WEAKGSFSQA---DVHRQVAIV  234
_NXC99388.1_       197  LLCDKVQ----------K-DD---IEVRFV-LDN--------------WEAKGSFSQA---DVHRQVAIV  234
_RMC07975.1_       197  LLCDKVQ----------K-DD---IEVRFV-LDN--------------WEAKGSFSQA---DVHRQVAIV  234
_NXT95159.1_       155  LLCDKVQ----------K-DD---IEVRFV-LDN--------------WEAKGSFSQA---DVHRQVAIV  192
_XP_009957589.1_   155  LLCDKVQ----------K-DD---IEVRFV-LDN--------------WEAKGSFSQA---DVHRQVAIV  192
_TRZ13602.1_       155  LLCDKVQ----------K-DD---IEVRFV-LDN--------------WEAKGSFSQA---DVHRQVAIV  192
_NWR45911.1_       146  LLCDKVQ----------K-DD---IEVRFV-LDN--------------WEAKGSFSQA---DVHRQVAIV  183
_KAF4799649.1_     155  LLCDKVQ----------K-DD---IEVRFV-LDN--------------WEAKGSFSQA---DVHRQVAIV  192
_KAJ7420138.1_     191  LLCDKVQ----------K-DD---IEVRFV-LDN--------------WEAKGSFSQA---DVHRQVAIV  228
_XP_054678501.1_   170  -----------------N-ND---IEVRFV-LDN--------------WEAKGSFSQA---DVHRQVAIV  200
_NXP48685.1_       194  LLCDKVQ----------K-AE---L---------------------------------------------  204
_XP_010704701.1_   114  LLCDKVQ----------K-DD---IEVRFV-LDN--------------WEAKGSFSQA---DVHRQVAIV  151
_KFV10805.1_       106  LLCDKVQ----------K-DD---IEVRFV-LDN--------------WEAKGSFSQA---DVHRQVAIV  143
_XP_009572567.1_    76  LLCDKVQ----------K-DD---IEVRFV-LDN--------------WEAKGSFSQA---DVHRQVAIV  113
_NXF64277.1_        75  LLCDKVQ----------K-DD---IEVRFV-LDN--------------WEAKGSFSQA---DVHRQVAIV  112
_KFV54465.1_        75  LLCDKVQ----------K-DD---IEVRFV-LDN--------------WEAKGSFSQA---DVHRQVAIV  112
_KFV90717.1_        75  LLCDKVQ----------K-DD---IEVRFV-LDN--------------WEAKGSFSQA---DVHRQVAIV  112
_KFQ03674.1_        75  LLCDKVQ----------K-DD---IEVRFV-LDN--------------WEAKGSFSQA---DVHRQVAIV  112
_NXW01028.1_        75  LLCDKVQ----------K-DD---IEVRFV-LDN--------------WEAKGSFSQA---DVHRQVAIV  112
_KFP32908.1_        75  LLCDKVQ----------K-DD---IEVRFV-LDN--------------WEAKGSFSQA---DVHRQVAIV  112
_KFV93389.1_        75  LLCDKVQ----------K-DD---IEVRFV-LDN--------------WEAKGSFSQA---DVHRQVAIV  112
_NWI63728.1_        75  LLCDKVQ----------K-DD---IEVRFV-LDN--------------WEAKGSFSQA---DVHRQVAIV  112
_KAI6072063.1_      76  LLCDKVQ----------K-DD---IEVRFV-LDN--------------WEAKGSFSQA---DVHRQVAIV  113
_KFQ58901.1_        75  LLCDKVQ----------K-DD---IEVRFV-LDN--------------WEAKGSFSQA---DVHRQVAIV  112
_NXP33753.1_        75  LLCDKVQ----------K-DD---IEVRFV-LDN--------------WEAKGSFSQA---DVHRQVAIV  112
_XP_009476353.1_    74  LLCDKVQ----------K-DD---IEVRFV-LDN--------------WEAKGSFSQA---DVHRQVAIV  111
_KFQ55903.1_        75  LLCDKVQ----------K-DD---IEVRFV-LDN--------------WEAKGSFSQA---DVHRQVAIV  112
_XP_042683349.1_    75  LLCDKVQ----------K-DD---IEVRFV-LDN--------------WEAKGSFSQA---DVHRQVAIV  112
_NXR08991.1_        75  LLCDKVQ----------K-DD---IEVRFV-LDN--------------WEAKGSFSQA---DVHRQVAIV  112
_XP_008947250.1_    75  LLCDKVQ----------K-DD---IEVRFV-LDN--------------WEAKGSFSQA---DVHRQVAIV  112
_KFP72464.1_        75  LLCDKVQ----------K-DD---IEVRFV-LDN--------------WEAKGSFSQA---DVHRQVAIV  112
_KFV60523.1_        75  LLCDKVQ----------K-DD---IEVRFV-LDN--------------WEAKGSFSQA---DVHRQVAIV  112
_NXT13165.1_        75  LLCDKVQ----------K-DD---IEVRFV-LDN--------------WEAKGSFSQA---DVHRQVAIV  112
_NWT50506.1_        75  LLCDKVQ----------K-DD---IEVRFV-LDN--------------WEAKGSFSQA---DVHRQVAIV  112
_OXB76139.1_        73  LLCDKVQ----------K-DD---IEVRFV-LDN--------------WEAKGSFSQA---DVHRQVAIV  110
_XP_050827907.1_    88  LLCDKVQ----------K-DD---IEVRFV-LDN--------------WEAKGSFSQA---DVHRQVAIV  125
```

```
_XP_030302545.1_    83  LLCDKVQ----------K-DD---IEVRFV-LDN--------------WEAKGSFSQA---DVHRQVAIV  120
_XP_054036038.1_    87  LLCDKVQ----------K-DD---IEVRFV-LDN--------------WEAKGSFSQA---DVHRQVAIV  124
_XP_012788337.1_   197  LLCDRVQ----------K-DD---IEVRFV-LNN--------------WEAKGIFSQA---DVHRQVAIV  234
_XP_008070154.1_   186  LLCDKVQ----------K-DD---IEVRFV-LND--------------WEAKGIFSQA---DVHRQVAIV  223
_XP_027477445.1_   186  LLCDKVQ----------K-DD---IEVRFV-LND--------------WEAKGIFSQA---DVHRQVAIV  223
_XP_025848473.1_   186  LLCDKVQ----------K-DD---IEVRFV-LND--------------WEAKGIFSQA---DVHRQVAIV  223
_XP_049640716.1_   197  LLCDKVQ----------K-DD---IEVQFL-SNN--------------WEAKGIFSQA---DVHRQVAIV  234
_XP_023084089.1_   197  LLCDKVQ----------K-DD---IEVRFV-LND--------------WEAKGIFSQA---DVHRQVAIV  234
_EHH22143.1_       197  LLCDKVQ----------K-DD---IEVRFV-LND--------------WEAKGIFSQA---DVHRQVAIV  234
_XP_038504711.1_   197  LLCDKVQ----------K-DD---IEVRFV-LND--------------WEAKGIFSQA---DVHRQVAIV  234
_XP_038504703.1_   197  LLCDKVQ----------K-DD---IEVRFV-LND--------------WEAKGIFSQA---DVHRQVAIV  234
_XP_038504718.1_   197  LLCDKVQ----------K-DD---IEVRFV-LND--------------WEAKGIFSQA---DVHRQVAIV  234
_XP_048971856.1_   197  LLCDKVQ----------K-DD---IEVRFV-LND--------------WEAKGIFSQA---DVHRQVAIV  234
_KAB1266826.1_     197  LLCDKVQ----------K-DD---IEVRFV-LND--------------WEAKGIFSQA---DVHRQVAIV  234
_XP_048391424.1_   197  LLCDKVQ----------K-DD---IEVRFF-THN--------------WEAKGSFSQA---DVHRQVAIV  234
_XP_048391425.1_   197  LLCDKVQ----------K-DD---IEVRFF-THN--------------WEAKGSFSQA---DVHRQVAIV  234
_GCC28026.1_       197  LLCDKVQ----------K-DD---IEVRFF-THN--------------WEAKGSFSQA---DVHRQVAIV  234
_XP_043551991.1_   197  LLCDKVQ----------K-DD---IEVRFF-THN--------------WEAKGSFSQA---DVHRQVAIV  234
_XP_043551989.1_   197  LLCDKVQ----------K-DD---IEVRFF-THN--------------WEAKGSFSQA---DVHRQVAIV  234
_XP_054534389.1_   107  LLCDKVQ----------K-DD---IEVRFV-LND--------------WEAKGIFSQA---DVHRQVAIV  144
_XP_011531312.1_   107  LLCDKVQ----------K-DD---IEVRFV-LND--------------WEAKGIFSQA---DVHRQVAIV  144
_XP_021780541.1_   106  LLCDKVQ----------K-DD---IEVRFV-LND--------------WEAKGIFSQA---DVHRQVAIV  143
_XP_037862912.1_   127  LLCDKVQ----------K-DD---IEVRFV-LND--------------WEAKGIFSQA---DVHRQVAIV  164
_XP_015413428.1_   186  LLCDKVQ----------K-DD---IEVRFA-LND--------------WEAKGIFSQA---DVHRQVAIV  223
_XP_005884642.1_   186  LLCDKVQ----------K-DD---IEVRFA-LND--------------WEAKGIFSQA---DVHRQVAIV  223
_KAF6105903.1_     186  LLCDKVQ----------K-DD---IEVRFV-LND--------------WEAKGIFSQA---DVHRQVAIV  223
_XP_015413426.1_   186  LLCDKVQ----------K-DD---IEVRFA-LND--------------WEAKGIFSQA---DVHRQVAIV  223
_XP_014387539.1_   186  LLCDKVQ----------K-DD---IEVRFA-LND--------------WEAKGIFSQA---DVHRQVAIV  223
_XP_014444723.1_   186  LLCDKVQ----------K-DD---IEVRFV-LND--------------WEAKGIFSQA---DVHRQVAIV  223
_XP_023448058.1_   186  LLCDKVQ----------K-DD---IEVRFV-LNN--------------WEAKGIFSQA---DVHRQVAIV  223
_EGW11604.1_       186  LLCDKVQ----------K-DD---IEVRFV-LND--------------WEAKGIFSQA---DVHRQVAIV  223
_XP_015985129.1_   186  LLCDKVQ----------K-DD---IEVRFA-LND--------------WEAKGIFSQA---DVHRQVAIV  223
_XP_012878014.1_   186  LLCDKVQ----------K-DD---IEVCFV-LND--------------WEAKGVFSQA---DVHRQVAIV  223
_KAG5211506.1_     165  LLCDKVQ----------K-DD---IEVRFV-LNE--------------WEAKGVFSQA---DVHRQVAIV  202
_MXQ82539.1_       161  LLCDKVQ----------K-DD---IEVRFV-LND--------------WEAKGVFSQA---DVHRQVAIV  198
_XP_032729748.1_   150  LLCDKVQ----------K-DD---IEVRFV-LND--------------WEAKGIFSQA---DVHRQVAIV  187
_XP_032208113.1_   150  LLCDKVQ----------K-DD---IEVRFV-LND--------------WEAKGIFSQA---DVHRQVAIV  187
_XP_048208817.1_   169  -----------------N-HD---IEVRFV-LND--------------WEAKGIFSQA---DVHRQVAIV  199
_XP_038200333.1_   169  -----------------N-HD---IEVRFV-LND--------------WEAKGIFSQA---DVHRQVAIV  199
```

```
_P15307.2_         197  LLCDKVQ----------K-DD---IEVRFV-LND--------------WEARGVFSQA---DVHRQVAIV  234
_XP_041524635.1_   186  LLCDKVQ----------K-DD---IEVRFV-LND--------------WEAKGIFSQA---DVHRQVAIV  223
_CAA33843.1_       195  LLCDKVQ----------K-DD---IEVRFV-LND--------------WEARGVFSQA---DVHRQVAIV  232
_XP_028732210.2_   186  LLCDKVQ----------K-DD---IEVRFV-LND--------------WEAKGIFSQA---DVHRQVAIV  223
_XP_025848474.1_   186  LLCDKVQ----------K-DD---IEVRFV-LND--------------WEAKGIFSQA---DVHRQVAIV  223
_EDL97996.1_       186  LLCDKVQ----------K-DD---IEVRFV-LND--------------WEAKGIFSQA---DVHRQVAIV  223
_KAI4577886.1_     186  LLCDKVQ----------K-DD---IEVRFV-LNE--------------WEAKGVFSQA---DVHRQVAIV  223
_KAG8512309.1_     176  LY------------------D---IEVRFV-LND--------------WEAKGIFSQA---DVHRQVAIV  206
_XP_047637971.1_   170  -H------------------D---IEVRFV-LND--------------WEAKGIFSQA---DVHRQVAIV  199
_XP_019491454.1_   197  LLCDKVQ----------K-DD---IEVRFV-LND--------------WEAKGIFSQA---DVHRQVAIV  234
_XP_043842482.1_   199  LLCDKVQ----------K-DD---IEVRFV-LND--------------WEAKGSFSQA---DVHRQVAIV  236
_XP_027721170.1_   199  LLCDKVQ----------K-DD---IEVRFV-LND--------------WEAKGSFSQA---DVHRQVAIV  236
_XP_036604632.1_   199  LLCDKVQ----------K-DD---IEVRFV-LND--------------WEAKGSFSQA---DVHRQVAIV  236
_XP_020841164.1_   199  LLCDKVQ----------K-DD---IEVRFV-LND--------------WEAKGSFSQA---DVHRQVAIV  236
_XP_051831295.1_   199  LLCDKVQ----------K-DD---IEVRFV-LNE--------------WEAKGSFSQA---DVHRQVAIV  236
_XP_031806378.1_   199  LLCDKVQ----------K-DD---IEVRFA-LNE--------------WEAKGSFSQA---DVHRQVAIV  236
_XP_051831294.1_   199  LLCDKVQ----------K-DD---IEVRFV-LNE--------------WEAKGSFSQA---DVHRQVAIV  236
_XP_003339776.1_   199  LLCDKVQ----------K-DD---IEVRFV-LND--------------WEAKGSFSQA---DVHRQVAIV  236
_XP_044515413.1_   199  LLCDKVQ----------K-DD---IEVRFV-LND--------------WEAKGSFSQA---DVHRQVAIV  236
_XP_028928361.1_   199  LLCDKVQ----------K-DD---IEVRFV-LND--------------WEAKGSFSQA---DVHRQVAIV  236
_XP_038607403.1_   199  LLCDKVQ----------K-DD---IEVRFV-LND--------------WEAKGSFSQA---DVHRQVAIV  236
_XP_040849334.1_    75  LLCDKVQ----------K-DD---IEVRFV-LND--------------WEAKGIFSQA---DVHRQVAIV  112
_TKC46270.1_        73  LLCDKVQ----------K-DD---IEVRFV-LND--------------WEAKGIFSQA---DVHRQVAIV  110
_OWK10634.1_        74  LLCDKVQ----------K-DD---IEVRFV-LNE--------------WEAKGVFSQA---DVHRQVAIV  111
_XP_036190342.1_    73  LLCDKVQ----------K-DD---IEVRFA-LND--------------WEAKGIFSQA---DVHRQVAIV  110
_KAB0395596.1_      70  LLCDKVQ----------K-DD---IEVRFV-LND--------------WEAKGIFSQA---DVHRQVAIV  107
_KAF6430170.1_      74  LLCDKVQ----------K-DD---IEVRFV-LND--------------WEAKGIFSQA---DVHRQVAIV  111
_XP_038948811.1_    75  LLCDKVQ----------K-DD---IEVRFV-LND--------------WEAKGIFSQA---DVHRQVAIV  112
_KAB1266828.1_     208  LLCDKVQ----------K-DD---IEVRFV-LND--------------WEAKGIFSQA---DVHRQVAIV  245
_NP_002899.1_      197  LLCDKVQ----------K-DD---IEVRFV-LND--------------WEAKGIFSQA---DVHRQVAIV  234
_XP_001159537.3_   197  LLCDKVQ----------K-DD---IEVRFV-LND--------------WEAKGIFSQA---DVHRQVAIV  234
_PNI66333.1_       197  LLCDKVQ----------K-DD---IEVRFV-LND--------------WEAKGIFSQA---DVHRQVAIV  234
_EHH55592.1_       197  LLCDKVQ----------K-DD---IEVRFV-LND--------------WEAKGIFSQA---DVHRQVAIV  234
_XP_003262470.1_   197  LLCDKVQ----------K-DD---IEVRFV-LND--------------WEAKGIFSQA---DVHRQVAIV  234
_BAG64854.1_       197  LLCDKVQ----------K-DD---IEVRFV-LND--------------WEAKGIFSQA---DVHRQVAIV  234
_XP_032034471.1_   197  LLCDKVQ----------K-DD---IEVRFV-LND--------------WEAKGIFSQA---DVHRQVAIV  234
_XP_037862911.1_   197  LLCDKVQ----------K-DD---IEVRFV-LND--------------WEAKGIFSQA---DVHRQVAIV  234
_XP_003830911.1_   197  LLCDKVQ----------K-DD---IEVRFV-LND--------------WEAKGIFSQA---DVHRQVAIV  234
_XP_004029331.1_   197  LLCDKVQ----------K-DD---IEVRFV-LND--------------WEAKGIFSQA---DVHRQVAIV  234
```

```
_XP_002812051.1_  197  LLCDKVQ----------K-DD---IEVRFV-LND--------------WEAKGIFSQA---DVHRQVAIV  234
_XP_011711623.1_  197  LLCDKVQ----------K-DD---IEVRFV-LND--------------WEAKGIFSQA---DVHRQVAIV  234
_XP_033095023.1_  197  LLCDKVQ----------K-DD---IEVRFV-LND--------------WEAKGIFSQA---DVHRQVAIV  234
_XP_002799280.1_  197  LLCDKVQ----------K-DD---IEVRFV-LND--------------WEAKGIFSQA---DVHRQVAIV  234
_XP_011853172.1_  197  LLCDKVQ----------K-DD---IEVRFV-LND--------------WEAKGIFSQA---DVHRQVAIV  234
_XP_050609443.1_  197  LLCDKVQ----------K-DD---IEVRFV-LND--------------WEAKGIFSQA---DVHRQVAIV  234
_XP_003908735.1_  197  LLCDKVQ----------K-DD---IEVRFV-LND--------------WEAKGIFSQA---DVHRQVAIV  234
_XP_025209693.1_  197  LLCDKVQ----------K-DD---IEVRFV-LND--------------WEAKGIFSQA---DVHRQVAIV  234
_XP_023084087.1_  197  LLCDKVQ----------K-DD---IEVRFV-LND--------------WEAKGIFSQA---DVHRQVAIV  234
_XP_021780539.1_  197  LLCDKVQ----------K-DD---IEVRFV-LND--------------WEAKGIFSQA---DVHRQVAIV  234
_XP_017704568.1_  197  LLCDKVQ----------K-DD---IEVRFV-LND--------------WEAKGIFSQA---DVHRQVAIV  234
_XP_010375811.1_  197  LLCDKVQ----------K-DD---IEVRFV-LND--------------WEAKGIFSQA---DVHRQVAIV  234
_XP_030776399.1_  197  LLCDKVQ----------K-DD---IEVRFV-LND--------------WEAKGIFSQA---DVHRQVAIV  234
_XP_011797211.1_  197  LLCDKVQ----------K-DD---IEVRFV-LND--------------WEAKGIFSQA---DVHRQVAIV  234
_XP_012332682.1_  197  LLCDKVQ----------K-DD---IEVRFV-LND--------------WEAKGIFSQA---DVHRQVAIV  234
_XP_010331875.2_  197  LLCDKVQ----------K-DD---IEVRFV-LND--------------WEAKGIFSQA---DVHRQVAIV  234
_XP_012332683.1_  197  LLCDKVQ----------K-DD---IEVRFV-LND--------------WEAKGIFSQA---DVHRQVAIV  234
_XP_017400475.1_  197  LLCDKVQ----------K-DD---IEVRFV-LND--------------WEAKGIFSQA---DVHRQVAIV  234
_XP_008568357.1_  197  LLCDKVQ----------K-DD---IEVRFV-LND--------------WEAKGIFSQA---DVHRQVAIV  234
_XP_012620284.1_  197  LLCDKVQ----------K-DD---IEVRFV-LND--------------WEAKGIFSQA---DVHRQVAIV  234
_XP_002709741.2_  197  LLCDKVQ----------K-DD---IEVRFV-LND--------------WEAKGIFSQA---DVHRQVAIV  234
_XP_022452943.1_  197  LLCDKVQ----------K-DD---IEVRFV-LND--------------WEAKGIFSQA---DVHRQVAIV  234
_XP_045405625.1_  197  LLCDKVQ----------K-DD---IEVRFV-LND--------------WEAKGIFSQA---DVHRQVAIV  234
_XP_004280707.1_  197  LLCDKVQ----------K-DD---IEVRFV-LND--------------WEAKGIFSQA---DVHRQVAIV  234
_XP_030733169.1_  197  LLCDKVQ----------K-DD---IEVRFV-LND--------------WEAKGIFSQA---DVHRQVAIV  234
_TEA23022.1_      197  LLCDKVQ----------K-DD---IEVRFV-LND--------------WEAKGIFSQA---DVHRQVAIV  234
_XP_012494629.1_  197  LLCDKVQ----------K-DD---IEVRFV-LND--------------WEAKGIFSQA---DVHRQVAIV  234
_XP_010950191.1_  197  LLCDKVQ----------K-DD---IEVRFV-LND--------------WEAKGIFSQA---DVHRQVAIV  234
_XP_024608785.1_  197  LLCDKVQ----------K-DD---IEVRFV-LND--------------WEAKGIFSQA---DVHRQVAIV  234
_XP_006201637.1_  197  LLCDKVQ----------K-DD---IEVRFV-LND--------------WEAKGIFSQA---DVHRQVAIV  234
_XP_019285354.1_  197  LLCDKVQ----------K-DD---IEVRFV-LND--------------WEAKGIFSQA---DVHRQVAIV  234
_XP_032509224.1_  197  LLCDKVQ----------K-DD---IEVRFV-LND--------------WEAKGIFSQA---DVHRQVAIV  234
_XP_047379355.1_  197  LLCDKVQ----------K-DD---IEVRFV-LND--------------WEAKGNFSQA---DVHRQVAIV  234
_XP_032981134.1_  197  LLCDKVQ----------K-DD---IEVRFV-LND--------------WEAKGIFSQA---DVHRQVAIV  234
_XP_005600018.1_  197  LLCDKVQ----------K-DD---IEVRFV-LND--------------WEAKGIFSQA---DVHRQVAIV  234
_XP_046517051.1_  197  LLCDKVQ----------K-DD---IEVRFV-LND--------------WEAKGIFSQA---DVHRQVAIV  234
_XP_007464205.1_  197  LLCDKVQ----------K-DD---IEVRFV-LND--------------WEAKGIFSQA---DVHRQVAIV  234
_XP_019572903.1_  197  LLCDKVQ----------K-DD---IEVRFV-LND--------------WEAKGIFSQA---DVHRQVAIV  234
_XP_007189755.1_  197  LLCDKVQ----------K-DD---IEVRFV-LND--------------WEAKGIFSQA---DVHRQVAIV  234
```

```
_XP_004397771.1_  197  LLCDKVQ----------K-DD---IEVRFV-LND--------------WEAKGIFSQA---DVHRQVAIV  234
_XP_004435713.1_  198  LLCDKVQ----------K-DD---IEVRFV-LND--------------WEAKGIFSQA---DVHRQVAIV  235
_XP_008698124.1_  197  LLCDKVQ----------K-DD---IEVRFV-LND--------------WEAKGIFSQA---DVHRQVAIV  234
_XP_023989255.1_  197  LLCDKVQ----------K-DD---IEVRFV-LND--------------WEAKGIFSQA---DVHRQVAIV  234
_XP_011233012.2_  197  LLCDKVQ----------K-DD---IEVRFV-LND--------------WEAKGIFSQA---DVHRQVAIV  234
_XP_025724444.1_  197  LLCDKVQ----------K-DD---IEVRFV-LND--------------WEAKGIFSQA---DVHRQVAIV  234
_XP_005626186.1_  197  LLCDKVQ----------K-DD---IEVRFV-LND--------------WEAKGIFSQA---DVHRQVAIV  234
_XP_045643991.1_  197  LLCDKVQ----------K-DD---IEVRFV-LND--------------WEAKGIFSQA---DVHRQVAIV  234
_XP_034857383.1_  197  LLCDKVQ----------K-DD---IEVRFV-LND--------------WEAKGIFSQA---DVHRQVAIV  234
_XP_027945501.1_  197  LLCDKVQ----------K-DD---IEVRFV-LND--------------WEAKGIFSQA---DVHRQVAIV  234
_XP_026341094.1_  197  LLCDKVQ----------K-DD---IEVRFV-LND--------------WEAKGIFSQA---DVHRQVAIV  234
_EFB29923.1_      197  LLCDKVQ----------K-DD---IEVRFV-LND--------------WEAKGIFSQA---DVHRQVAIV  234
_XP_045740897.1_  197  LLCDKVQ----------K-DD---IEVRFV-LND--------------WEAKGIFSQA---DVHRQVAIV  234
_CAD7677454.1_    197  LLCDKVQ----------K-DD---IEVRFV-LND--------------WEAKGIFSQA---DVHRQVAIV  234
_XP_035577553.1_  197  LLCDKVQ----------K-DD---IEVRFV-LND--------------WEAKGIFSQA---DVHRQVAIV  234
_XP_006733255.1_  197  LLCDKVQ----------K-DD---IEVRFV-LND--------------WEAKGIFSQA---DVHRQVAIV  234
_XP_032266820.1_  197  LLCDKVQ----------K-DD---IEVRFA-LND--------------WEAKGIFSQA---DVHRQVAIV  234
_XP_041611730.1_  197  LLCDKVQ----------K-DD---IEVRFV-LND--------------WEAKGIFSQA---DVHRQVAIV  234
_XP_048971854.1_  197  LLCDKVQ----------K-DD---IEVRFV-LND--------------WEAKGIFSQA---DVHRQVAIV  234
_XP_021554840.1_  197  LLCDKVQ----------K-DD---IEVRFV-LND--------------WEAKGIFSQA---DVHRQVAIV  234
_VCW78562.1_      197  LLCDKVQ----------K-DD---IEVRFV-LND--------------WEAKGIFSQA---DVHRQVAIV  234
_XP_034857381.1_  197  LLCDKVQ----------K-DD---IEVRFV-LND--------------WEAKGIFSQA---DVHRQVAIV  234
_XP_008524231.1_  194  LLCDKVQ----------K-DD---IEVRFV-LND--------------WEAKGIFSQA---DVHRQVAIV  231
_XP_045740895.1_  197  LLCDKVQ----------K-DD---IEVRFV-LND--------------WEAKGIFSQA---DVHRQVAIV  234
_XP_045834746.1_  197  LLCDKVQ----------K-DD---IEVRFV-LND--------------WEAKGIFSQA---DVHRQVAIV  234
_XP_035932906.1_  197  LLCDKVQ----------K-DD---IEVRFA-LND--------------WEAKGIFSQA---DVHRQVAIV  234
_XP_030883859.1_  197  LLCDKVQ----------K-DD---IEVRFV-LND--------------WEAKGIFSQA---DVHRQVAIV  234
_XP_032266818.1_  197  LLCDKVQ----------K-DD---IEVRFA-LND--------------WEAKGIFSQA---DVHRQVAIV  234
_XP_024408528.2_  197  LLCDKVQ----------K-DD---IEVRFV-LND--------------WEAKGIFSQA---DVHRQVAIV  234
_XP_004580347.1_  197  LLCDKVQ----------K-DD---IEVRFV-LND--------------WEAKGIFSQA---DVHRQVAIV  234
_XP_030166525.1_  197  LLCDKVQ----------K-DD---IEVRFV-LND--------------WEAKGIFSQA---DVHRQVAIV  234
_XP_035932904.1_  197  LLCDKVQ----------K-DD---IEVRFA-LND--------------WEAKGIFSQA---DVHRQVAIV  234
_XP_020040690.1_  197  LLCDKVQ----------K-DD---IEVRFV-LND--------------WEAKGIFSQA---DVHRQVAIV  234
_XP_053445604.1_  197  LLCDKVQ----------K-DD---IEVRFV-LND--------------WEAKGIFSQA---DVHRQVAIV  234
_XP_049507482.1_  197  LLCDKVQ----------K-DD---IEVRFV-LND--------------WEAKGIFSQA---DVHRQVAIV  234
_XP_015335127.1_  197  LLCDKVQ----------K-DD---IEVRFV-LND--------------WEAKGNFSQA---DVHRQVAIV  234
_XP_027790245.1_  197  LLCDKVQ----------K-DD---IEVRFV-LND--------------WEAKGNFSQA---DVHRQVAIV  234
_XP_003984077.1_  197  LLCDKVQ----------K-DD---IEVRFV-LND--------------WEAKGIFSQA---DVHRQVAIV  234
_XP_039096259.1_  197  LLCDKVQ----------K-DD---IEVRFV-LND--------------WEAKGIFSQA---DVHRQVAIV  234
```

```
_XP_007075434.1_  197  LLCDKVQ----------K-DD---IEVRFV-LND--------------WEAKGIFSQA---DVHRQVAIV  234
_XP_022360130.1_  197  LLCDKVQ----------K-DD---IEVRFV-LND--------------WEAKGIFSQA---DVHRQVAIV  234
_XP_047707378.1_  197  LLCDKVQ----------K-DD---IEVRFV-LND--------------WEAKGIFSQA---DVHRQVAIV  234
_XP_026243031.1_  197  LLCDKVQ----------K-DD---IEVRFV-LND--------------WEAKGNFSQA---DVHRQVAIV  234
_XP_037383340.1_  197  LLCDKVQ----------K-DD---IEVRFV-LND--------------WEAKGIFSQA---DVHRQVAIV  234
_XP_005322095.1_  197  LLCDKVQ----------K-DD---IEVRFV-LND--------------WEAKGNFSQA---DVHRQVAIV  234
_XP_005662584.1_  197  LLCDKVQ----------K-DD---IEVRFV-LND--------------WEAKGIFSQA---DVHRQVAIV  234
_XP_040080101.1_  197  LLCDKVQ----------K-DD---IEVRFV-LNE--------------WEAKGVFSQA---DVHRQVAIV  234
_XP_017503999.1_  197  LLCDKVQ----------K-DD---IEVRFV-LND--------------WEAKGIFSQA---DVHRQVAIV  234
_XP_044119954.1_  197  LLCDKVQ----------K-DD---IEVRFV-LND--------------WEAKGIFSQA---DVHRQVAIV  234
_XP_032208110.1_  197  LLCDKVQ----------K-DD---IEVRFV-LND--------------WEAKGIFSQA---DVHRQVAIV  234
_XP_012586936.1_  197  LLCDKVQ----------K-DD---IEVRFV-LND--------------WEAKGIFSQA---DVHRQVAIV  234
_XP_032729745.1_  197  LLCDKVQ----------K-DD---IEVRFV-LND--------------WEAKGIFSQA---DVHRQVAIV  234
_XP_052504199.1_  197  LLCDKVQ----------K-DD---IEVRFV-LNE--------------WEAKGVFSQA---DVHRQVAIV  234
_XP_054431389.1_  197  LLCDKVQ----------K-DD---IEVRFV-LND--------------WEAKGTFSQA---DVHRQVAIV  234
_XP_023576632.1_  197  LLCDKVQ----------K-DD---IEVRFV-LND--------------WEAKGVFSQA---DVHRQVAIV  234
_XP_004742070.1_  197  LLCDKVQ----------K-DD---IEVRFV-LND--------------WEAKGIFSQA---DVHRQVAIV  234
_XP_037663267.1_  197  LLCDKVQ----------K-DD---IEVRFV-LNN--------------WEAKGIFSQA---DVHRQVAIV  234
_XP_004005978.1_  197  LLCDKVQ----------K-DD---IEVRFV-LNE--------------WEAKGVFSQA---DVHRQVAIV  234
_XP_036781908.1_  197  LLCDKVQ----------K-DD---IEVRFV-FND--------------WEAKGIFSQA---DVHRQVAIV  234
_XP_040849333.1_  197  LLCDKVQ----------K-DD---IEVRFV-LND--------------WEAKGIFSQA---DVHRQVAIV  234
_XP_005686691.1_  197  LLCDKVQ----------K-DD---IEVRFV-LNE--------------WEAKGVFSQA---DVHRQVAIV  234
_XP_023367900.1_  197  LLCDKVQ----------K-DD---IEVRFV-LND--------------WEAKGIFSQA---DVHRQVAIV  234
_KAF0879576.1_    197  LLCDKVQ----------K-DD---IEVRFV-LND--------------WEAKGIFSQA---DVHRQVAIV  234
_XP_029793284.1_  197  LLCDKVQ----------K-DD---IEVRFV-LND--------------WEAKGIFSQA---DVHRQVAIV  234
_NP_001179899.1_  197  LLCDKVQ----------K-DD---IEVRFV-LND--------------WEAKGVFSQA---DVHRQVAIV  234
_XP_020764718.1_  197  LLCDKVQ----------K-DD---IEVRFV-LNE--------------WEAKGVFSQA---DVHRQVAIV  234
_XP_054431388.1_  197  LLCDKVQ----------K-DD---IEVRFV-LND--------------WEAKGTFSQA---DVHRQVAIV  234
_XP_037663265.1_  197  LLCDKVQ----------K-DD---IEVRFV-LNN--------------WEAKGIFSQA---DVHRQVAIV  234
_XP_019825763.1_  197  LLCDKVQ----------K-DD---IEVRFV-LND--------------WEAKGVFSQA---DVHRQVAIV  234
_XP_005889096.1_  197  LLCDKVQ----------K-DD---IEVRFV-LND--------------WEAKGVFSQA---DVHRQVAIV  234
_XP_043324571.1_  197  LLCDKVQ----------K-DD---IEVRFV-LNE--------------WEAKGVFSQA---DVHRQVAIV  234
_KAJ1078272.1_    197  LLCDKVQ----------K-DD---IEVRFV-LNE--------------WEAKGVFSQA---DVHRQVAIV  234
_XP_036190339.1_  197  LLCDKVQ----------K-DD---IEVRFA-LND--------------WEAKGIFSQA---DVHRQVAIV  234
_XP_013005522.1_  197  LLCDKVQ----------K-DD---IEVRFV-LND--------------WEAKGIFSQA---DVHRQVAIV  234
_XP_006073936.1_  197  LLCDKVQ----------K-DD---IEVRFV-LND--------------WEAKGVFSQA---DVHRQVAIV  234
_XP_016069529.1_  197  LLCDKVQ----------K-DD---IEVRFV-LND--------------WEAKGIFSQA---DVHRQVAIV  234
_KAB0354136.1_    197  LLCDKVQ----------K-DD---IEVRFV-LNE--------------WEAKGVFSQA---DVHRQVAIV  234
_XP_029424871.1_  197  LLCDKVQ----------K-DD---IEVRFV-LND--------------WEAKGIFSQA---DVHRQVAIV  234
```

```
_XP_036118792.1_  197  LLCDKVQ----------K-DD---IEVRFV-LND--------------WEAKGIFSQA---DVHRQVAIV  234
_XP_037019725.2_  196  LLCDKVQ----------K-DD---IEVRFV-LND--------------WEAKGIFSQA---DVHRQVAIV  233
_XP_047602572.1_  197  LLCDKVQ----------K-DD---IEVRFV-LND--------------WEAKGIFSQA---DVHRQVAIV  234
_XP_014586639.1_  197  LLCDKVQ----------K-DD---IEVRFV-LND--------------WEAKGIFSQA---DVHRQVAIV  234
_XP_046517052.1_  197  LLCDKVQ----------K-DD---IEVRFV-LND--------------WEAKGIFSQA---DVHRQVAIV  234
_XP_006909635.2_  197  LLCDKVQ----------K-DD---IEVRFA-LND--------------WEAKGIFSQA---DVHRQVAIV  234
_XP_011362222.1_  197  LLCDKVQ----------K-DD---IEVRFA-LND--------------WEAKGIFSQA---DVHRQVAIV  234
_XP_045684409.1_  197  LLCDKVQ----------K-DD---IEVRFV-LND--------------WEAKGIFSQA---DVHRQVAIV  234
_XP_048208816.1_  197  LLCDKVQ----------K-DD---IEVRFV-LND--------------WEAKGIFSQA---DVHRQVAIV  234
_XP_052590629.1_  197  LLCDKVQ----------K-DD---IEVRFV-LND--------------WEAKGIFSQA---DVHRQVAIV  234
_XP_032353499.1_  197  LLCDKVQ----------K-DD---IEVRFV-LND--------------WEAKGIFSQA---DVHRQVAIV  234
_XP_038200327.1_  197  LLCDKVQ----------K-DD---IEVRFV-LND--------------WEAKGIFSQA---DVHRQVAIV  234
_XP_051056550.1_  197  LLCDKVQ----------K-DD---IEVRFV-LND--------------WEAKGIFSQA---DVHRQVAIV  234
_XP_012805850.2_  197  LLCDKVQ----------K-DD---IEVRFV-LND--------------WEAKGIFSQA---DVHRQVAIV  234
_XP_016050906.1_  197  LLCDKVQ----------K-DD---IEVRFV-LND--------------WEAKGIFSQA---DVHRQVAIV  234
_XP_030166526.1_  197  LLCDKVQ----------K-DD---IEVRFV-LND--------------WEAKGIFSQA---DVHRQVAIV  234
_XP_036903878.1_  196  LLCDKVQ----------K-DD---IEVRFV-LND--------------WEAKGIFSQA---DVHRQVAIV  233
_CAH7004032.1_    197  LLCDKVQ----------K-DD---IEVRFV-LND--------------WEAKGIFSQA---DVHRQVAIV  234
_XP_051056549.1_  197  LLCDKVQ----------K-DD---IEVRFV-LND--------------WEAKGIFSQA---DVHRQVAIV  234
_XP_042547166.1_  197  LLCDKVQ----------K-DD---IEVRFV-LND--------------WEAKGIFSQA---DVHRQVAIV  234
_XP_054584456.1_  197  LLCDKVQ----------K-DD---IEVRFA-LND--------------WEAKGIFSQA---DVHRQVAIV  234
_XP_049996768.1_  197  LLCDKVQ----------K-DD---IEVRFV-LND--------------WEAKGIFSQA---DVHRQVAIV  234
_XP_027243805.1_  197  LLCDKVQ----------K-DD---IEVRFV-LND--------------WEAKGIFSQA---DVHRQVAIV  234
_XP_047707379.1_  197  LLCDKVQ----------K-DD---IEVRFV-LND--------------WEAKGIFSQA---DVHRQVAIV  234
_XP_026645370.1_  197  LLCDKVQ----------K-DD---IEVRFV-LND--------------WEAKGIFSQA---DVHRQVAIV  234
_XP_014302519.1_  197  LLCDKVQ----------K-DD---IEVRFA-LND--------------WEAKGIFSQA---DVHRQVAIV  234
_XP_049726303.1_  197  LLCDKVQ----------K-DD---IEVRFV-LND--------------WEAKGIFSQA---DVHRQVAIV  234
_XP_049726302.1_  197  LLCDKVQ----------K-DD---IEVRFV-LND--------------WEAKGIFSQA---DVHRQVAIV  234
_XP_012969026.1_  197  LLCDKVQ----------K-DD---IEVRFV-LND--------------WEAKGIFSQA---DVHRQVAIV  234
_XP_008524232.1_  194  LLCDKVQ----------K-DD---IEVRFV-LND--------------WEAKGIFSQA---DVHRQVAIV  231
_XP_051020904.1_  197  LLCDKVQ----------K-DD---IEVRFV-LND--------------WEAKGIFSQA---DVHRQVAIV  234
_XP_023590712.1_  197  LLCDKVQ----------K-DD---IEVRFV-LND--------------WEAKGIFSQA---DVHRQVAIV  234
_XP_027243803.1_  197  LLCDKVQ----------K-DD---IEVRFV-LND--------------WEAKGIFSQA---DVHRQVAIV  234
_XP_040589151.1_  197  LLCDKVQ----------K-DD---IEVRFV-LND--------------WEAKGIFSQA---DVHRQVAIV  234
_XP_023412959.1_  197  LLCDKVQ----------K-DD---IEVRFV-LND--------------WEAKGIFSQA---DVHRQVAIV  234
_XP_036057111.1_  197  LLCDKVQ----------K-DD---IEVRFV-LND--------------WEAKGIFSQA---DAQRQVAIA  234
_XP_027822602.1_  197  LLCDKVQ----------K-DD---IEVRFV-LNE--------------WEAKGVFSQA---DVHRQVAIV  234
_XP_031322949.1_  197  LLCDKVQ----------K-DD---IEVRFV-LND--------------WEAKGIFSQA---DVHRQVAIV  234
_XP_028634966.1_  197  LLCDKVQ----------K-DD---IEVRFV-LND--------------WEAKGVFSQA---DVHRQVAIV  234
```

```
_XP_031531529.1_  197  LLCDKVQ----------K-DD---IEVRFV-LND--------------WEAKGIFSQA---DVHRQVAIV  234
_XP_006073937.1_  197  LLCDKVQ----------K-DD---IEVRFV-LND--------------WEAKGVFSQA---DVHRQVAIV  234
_XP_034364887.1_  197  LLCDKVQ----------K-DD---IEVRFV-LND--------------WEAKGVFSQA---DVHRQVAIV  234
_XP_021482246.1_  197  LLCDKVQ----------K-DD---IEVRFV-LND--------------WEAKGIFSQA---DVHRQVAIV  234
_KAF4016326.1_    197  LLCDKVQ----------K-DD---IEVRFV-LNE--------------WEAKGVFSQA---DVHRQVAIV  234
_XP_023602319.1_  197  LLCDKVQ----------K-DD---IEVRFA-LND--------------WEAKGIFSQA---DVHRQVAIV  234
_XP_052054164.1_  197  LLCDKVQ----------K-DD---IEVRFV-LND--------------WEAKGVFSQA---DVHRQVAIV  234
_XP_036291310.1_  197  LLCDKVQ----------K-DD---IEVRFA-LND--------------WEAKGIFSQA---DVHRQVAIV  234
_XP_013370168.1_  197  LLCDKVQ----------K-DD---IEVRFV-LND--------------WEAKGIFSQA---DVHRQVAIV  234
_XP_023590722.1_  197  LLCDKVQ----------K-DD---IEVRFV-LND--------------WEAKGIFSQA---DVHRQVAIV  234
_XP_048291328.1_  197  LLCDKVQ----------K-DD---IEVRFV-LND--------------WEAKGIFSQA---DVHRQVAIV  234
_XP_032772910.1_  197  LLCDKVQ----------K-DD---IEVRFV-LND--------------WEAKGIFSQA---DVHRQVAIV  234
_XP_008768670.2_  197  LLCDKVQ----------K-DD---IEVRFV-LND--------------WEAKGIFSQA---DVHRQVAIV  234
_XP_014302521.1_  197  LLCDKVQ----------K-DD---IEVRFA-LND--------------WEAKGIFSQA---DVHRQVAIV  234
_XP_031194882.1_  197  LLCDKVQ----------K-DD---IEVRFV-LND--------------WEAKGVFSQA---DVHRQVAIV  234
_NP_033070.2_     197  LLCDKVQ----------K-DD---IEVRFV-LND--------------WEARGVFSQA---DVHRQVAIV  234
_XP_036291312.1_  197  LLCDKVQ----------K-DD---IEVRFA-LND--------------WEAKGIFSQA---DVHRQVAIV  234
_XP_021033157.1_  197  LLCDKVQ----------K-DD---IEVRFV-LND--------------WEAKGVFSQA---DVHRQVAIV  234
_XP_015849136.1_  197  LLCDKVQ----------K-DD---IEVRFV-LND--------------WEAKGIFSQA---DVHRQVAIV  234
_XP_045150027.1_  197  LLCDKVQ----------K-DD---IEVRFV-LND--------------WEAKGVFSQA---DVHRQVAIV  234
_XP_021066684.1_  197  LLCDKVQ----------K-DD---IEVRFV-LND--------------WEAKGVFSQA---DVHRQVAIV  234
_XP_006881088.1_  197  LLCDKVQ----------K-DD---IEVRFA-LND--------------WEAKGIFSQA---DVHRQVAIV  234
_XP_008524234.1_  194  LLCDKVQ----------K-DD---IEVRFV-LND--------------WEAKGIFSQA---DVHRQVAIV  231
_XP_021482260.1_  197  LLCDKVQ----------K-DD---IEVRFV-LND--------------WEAKGIFSQA---DVHRQVAIV  234
_XP_007125500.1_  197  LLCDKVQ----------K-DD---IEVRFV-LND--------------WEAKGIFSQA---DVHRQVAIV  234
_XP_028352731.1_  197  LLCDKVQ----------K-DD---IEVRFV-LND--------------WEAKGIFSQA---DVHRQVAIV  234
_XP_028352733.1_  197  LLCDKVQ----------K-DD---IEVRFV-LND--------------WEAKGIFSQA---DVHRQVAIV  234
_XP_028352734.1_  197  LLCDKVQ----------K-DD---IEVRFV-LND--------------WEAKGIFSQA---DVHRQVAIV  234
_XP_028352736.1_  197  LLCDKVQ----------K-DD---IEVRFV-LND--------------WEAKGIFSQA---DVHRQVAIV  234
_XP_028352735.1_  197  LLCDKVQ----------K-DD---IEVRFV-LND--------------WEAKGIFSQA---DVHRQVAIV  234
_ERE88482.1_      197  LLCDKVQ----------K-DD---IEVRFV-LND--------------WEAKGIFSQA---DVHRQVAIV  234
_KAF3826750.1_     57  -------------------------EVRFV-LND--------------WEAKGIFSQA---DVHRQVAIV   83
_OBS70420.1_      141  -------------------DD---IEVRFV-LND--------------WEAKGIFSQA---DVHRQVAIV  170
_XP_006878094.1_  160  LLCDKVQ----------K-DD---IEVRFV-LND--------------WEAKGIFSQA---DVHRQVAIV  197
_XP_008978905.3_  154  LLCDKVQ----------K-DD---IEVRFV-LND--------------WEAKGIFSQA---DVHRQVAIV  191
_XP_032147941.1_  154  LLCDKVQ----------K-DD---IEVRFV-LND--------------WEAKGIFSQA---DVHRQVAIV  191
_XP_008524233.1_  156  LLCDKVQ----------K-DD---IEVRFV-LND--------------WEAKGIFSQA---DVHRQVAIV  193
_XP_044628438.1_  154  LLCDKVQ----------K-DD---IEVRFV-LND--------------WEAKGIFSQA---DVHRQVAIV  191
_XP_010608101.1_  154  LLCDKVQ----------K-DD---IEVRFV-LND--------------WEAKGIFSQA---DVHRQVAIV  191
```

```
_KAF5918007.1_     156  LLCDKVQ----------K-DD---IEVRFV-LND--------------WEAKGIFSQA---DVHRQVAIV  193
_XP_021101942.1_   154  LLCDKVQ----------K-DD---IEVRFV-LND--------------WEAKGIFSQA---DVHRQVAIV  191
_ABM89308.1_        73  LLCDKVQ----------K----------------------------------------------------   80
_MBW00678.1_        58  LLCDKVQ----------K-GV-------------------------------------------------   67
_NP_001096177.1_   197  LLCDKVQ----------K-DD---IEVRFF-TDN--------------WEAKGTFGQA---DVHRQVAIV  234
_OCT77375.1_       197  LLCDKVQ----------K-DD---IEVRFF-TDN--------------WEAKGTFGQA---DVHRQVAIV  234
_AAI70187.1_       197  LLCDKVQ----------K-DD---IEVRFF-TDN--------------WEAKGTFGQA---DVHRQVAIV  234
_AAI69362.1_       197  LLCDKVQ----------K-DD---IEVRFF-TDN--------------WEAKGTFGQA---DVHRQVAIV  234
_XP_041418586.1_   197  LLCDKVQ----------K-DD---IEVRFF-TDN--------------WEAKGTFGQA---DVHRQVAIV  234
_XP_041418585.1_   197  LLCDKVQ----------K-DD---IEVRFF-TDN--------------WEAKGTFGQA---DVHRQVAIV  234
_XP_041418587.1_   197  LLCDKVQ----------K-DD---IEVRFF-TDN--------------WEAKGTFGQA---DVHRQVAIV  234
_XP_053316221.1_   197  LLCDKVQ----------K-DD---IEVRFF-SDN--------------WEAKGTFGQA---DVHRQVAIV  234
_XP_053568018.1_   197  LLCDKVQ----------K-DD---IEVRFF-TDD--------------WEAKGTFSQA---DVHRQVAIV  234
_KAG8443975.1_     197  LLCDKVQ----------R-DD---IEVRFF-TDN--------------WEDKGTFGQA---DVHRQVAIV  234
_XP_044145067.1_   199  LLCDKVQ----------K-DD---IEVRFF-SNT--------------WEDKGQFGQA---DVHRQVAIV  236
_KAG9484268.1_     199  LLCDKVQ----------K-DD---IEVRFF-TNT--------------WEDKGQFAQA---DVHRQVAIV  236
_XP_040285660.1_   199  LLCDKVQ----------K-DD---IEVRFF-TNT--------------WEDKGQFGQA---DVHRQVAIV  236
_XP_040285659.1_   199  LLCDKVQ----------K-DD---IEVRFF-TNT--------------WEDKGQFGQA---DVHRQVAIV  236
_KAG8583825.1_     199  LLCDKVQ----------K-DD---IEVRFF-TST--------------WEDKGHFGQA---DVHRQVAIV  236
_XP_018411072.1_   199  LLCDKVQ----------K-DD---IEVRFF-SDN--------------WENKGIFGQA---DVHRQVAIV  236
_XP_040207073.1_   199  LLCDKVQ----------K-DD---IEVRFF-TDN--------------WENKGIFGQA---DVHRQVAIV  236
_NP_001081750.1_   194  LLCDKVQ----------K-DD---IEVRFF-TDN--------------WEAKGTFGQA---DVHRQVANV  231
_PIO39092.1_        75  LLCDKVQ----------K-DD---IEVRFF-TDN--------------WENKGIFGQA---DVHRQVAIV  112
_PIO39091.1_        75  LLCDKVQ----------K-DD---IEVRFF-TDN--------------WENKGIFGQA---DVHRQVAIV  112
_XP_041035877.1_   196  LLCDKVQ----------K-ED---IEVRFF-TSS--------------WEGKGSFSQA---DVHRQVAIV  233
_XP_042202691.1_   196  LLCDKVQ----------K-ED---IEVRFF-TSD--------------WEGRGSFSQA---DVHRQVAIV  233
_XP_048475509.1_   185  LLCDKVQ----------K-ED---IEVRFF-TNE--------------WEGRGSFSQA---DVHRQVAIV  222
_XP_048380947.1_   185  LLCDKVQ----------K-ED---IEVRFF-TNE--------------WEGKGSFSQA---DVHRQVAIV  222
_XP_051901152.1_   189  LLCDKVQ----------K-ED---IEVRFF-TSD--------------WEGKGSFSQA---DVHRQVAIV  226
_BAD60879.1_       180  LLCEKIN----------K-DD---IQVRFF-EETE---------DGISWEAFGDFASH---DVRRQYAIV  222
_XP_050405370.1_   192  LLCEKIN----------K-DD---IGVRFY-EESVD--------GTVVWENYGEFGAG---DVHRQYAIV  235
_XP_009063713.1_   192  ILCEKIN----------K-DD---IGIKFY-EERKD--------GTIEWEAFGEFGAG---DVHRQYAIV  235
_CAB0044847.1_     211  LLCKRLD----------R-DD---IKVVFF-DDES------------GWKSEARLSSP---GVWNRCALL  250
_XP_048259911.1_   194  LLCEKVN----------K-DD---IQIRFF-KDTAA--------G-CMWEDYGDFGQG---DVHRQFAIV  236
_ADI72431.1_       194  LLCEKVN----------K-DD---IQVRFF-KDTAA--------G-CMWEDYGDFGQG---DVHRQFAIV  236
_CAG5133522.1_     195  LLCDKVN----------K-ED---IKIRLF-EENDQ--------GITIWEDFGDFGQG---DVHRQYAIV  238
_KAG5688355.1_     104  LLCEKVG----------K-DD---IRVRFF-EDVD---------GEVVWEGFGDFGQG---DVHRQYAIV  146
_AMT84567.1_       193  MLVEKVN----------K-DD---IHVRFF-ELSET--------GDTIWEGYADLSPS---DVHRQFAIV  236
_XP_052276378.1_   192  LLCEKIN----------R-DD---VRVRFY-LEQQ---------GHVVWEAFGDFGQN---DVHRQFAIV  234
```

```
_XP_052276377.1_   192  LLCEKIN----------R-DD---VRVRFY-LEQQ---------GHVVWEAFGDFGQN---DVHRQFAIV  234
_AAK72690.1_       194  LLCEKIN----------R-DD---IVVRFY-EETDC--------GECVWEDFADFSTN---DIHRQYAIV  237
_XP_011414731.2_   194  LLCEKIN----------R-DD---IVVRFY-EETEC--------GECLWEDFADFSTN---DIHRQYAIV  237
_XP_052699807.1_   194  LLCEKIN----------R-DD---IVVRFY-EETEC--------GECVWEDFADFSTN---DIHRQYAIV  237
_NP_001292261.1_   194  LLCEKIN----------R-DD---IVVRFY-EETEC--------GECLWEDFADFSTN---DIHRQYAIV  237
_XP_048738054.1_   194  LLCEKIN----------R-DD---IVVRFF-EENEF--------GECLWEDFADFSTN---DIHRQYAIV  237
_XP_052699808.1_   194  LLCEKIN----------R-DD---IVVRFY-EETEC--------GECVWEDFADFSTN---DIHRQYAIV  237
_XP_022332457.1_   194  LLCEKIN----------R-DD---IVVRFY-EETQF--------GECIWEDFADFSTN---DIHRQYAIV  237
_ABL63469.1_       193  LLCEKIN----------R-DD---IGVRFY-QENSS--------GDTIWEDYGEFSVN---DIHRQYAIV  236
_XP_033733822.1_   194  LLCEKIN----------R-ED---IGVRFY-KENEVN------ESLLDWEDYGDFSQN---DIHRQYAIV  239
_XP_021347355.1_   194  LLCEKIN----------R-ED---IGVRFY-KENDAN------ESVVEWEEYGDFSQN---DIHRQYAIV  239
_UOA58607.1_       194  LLCEKIN----------R-ED---IGVRFY-QENEAT------ET-VEWEDYGDFSQN---DIHRQYAIV  238
_XP_021347356.1_   194  LLCEKIN----------R-ED---IGVRFY-KENDAN------ESVVEWEEYGDFSQN---DIHRQYAIV  239
_CAG2186872.1_     193  LLCEKIN----------K-DD---VRIRFY-QENDN--------GDVVWEDSGDFSAN---DIHRQYAIV  236
_XP_052058915.1_   193  LLCEKIN----------K-DD---VRIRFY-QENDN--------GDVVWEDSGDFSAN---DIHRQYAIV  236
_KAJ8309232.1_     194  LLCEKIN----------R-DD---IGIRFF-QEVDD--------GSVEWEDFGDFSAN---DIHRQYAIV  237
_UZH84475.1_       192  LLCEKIN----------R-DD---IRIRFF-EERD---------EETVWENFGDFSQN---DVHRQFAIV  234
_ALA99939.1_       192  LLCEKIN----------R-DD---IRVRFY-EERD---------GQIVWEGFGDFGQN---DVHRQFAIV  234
_XP_052767764.1_   192  LLCEKIN----------R-DD---VRVRFF-EERE---------GQIIWEAFGDFGQN---DVHRQYAIV  234
_ADD25211.1_       194  LLCEKIN----------R-ED---IGVRFY-QENEA-------TETVEWEDYGDFSQN---DIHRQYAIV  238
_XP_052224245.1_   190  LLCEKIN----------R-DD---VRVRFY-LEQQ---------GHVVWEAFGDFGQN---EVHRQFAIK  232
_KAJ8314234.1_     147  LLCEKIN----------R-ED---IDVRFY-EVAKG--------GVVEWEDYGIFSSY---DIHRQFL--  188
_VDH95719.1_       152  LLCEKIN----------K-DD---VRIRFY-QENDN--------GDVVWEDSGDFSAN---DIHRQYAIV  195
_XP_041352913.1_   194  LLCEKIN----------K-DD---IQVRFY-EENF---------TSVVWEAFGDFGAG---DVHRQYAIV  236
_XP_046555158.1_   194  LLCEKVN----------K-DD---IQVRFY-KDTA---------AGCMWEEFGDFGQG---DVHRQFAVV  236
_AAW33559.1_       194  LLCEKIN----------K-DD---IQVRFF-KDTA---------AGCMWEDFGDFGQG---DVHRQFAVV  236
_ABY87435.1_       181  LLCEKIN----------K-DD---IQVRFF-KDTA---------AGCMWEDFGDFGQG---DVHRQFAVV  223
_XP_009028834.1_   194  LLCEKIN----------K-DD---IQVRFF-EETD---------DREVWEGNGDFAPN---DVHRQYAIV  236
_XP_025106109.1_   194  LLCEKVG----------K-DD---IRVRFF-EE-EN--------GEPVWESFGDFGQG---DVHRQYAIV  236
_NP_001298191.1_   195  LLCDKVN----------K-ED---IKVRFY-EENEQ--------GMVVWEDLGDFGQG---DVHRQYAIV  238
_KAH9519545.1_     195  LLCDKVN----------K-ED---IKIRFY-EENEQ--------GIVVWEDFGDFGQG---DVHRQYAIV  238
_RUS74561.1_       195  LLCDKVN----------K-ED---IRIRFF-EENEN-------NGTVVWEDYGDFGQG---DVHRQYAIV  239
_GFO11973.1_       195  LLCDKVN----------K-ED---IKVRFF-EENEN--------GSVLWEDYGDFGQG---DVHRQYAIV  238
_CAG5132534.1_     195  LLCDKVN----------K-ED---IKIKFY-EEDNQ--------GVTLWEDHADFGQG---DVHRQ----  234
_GFR69641.1_       195  LLCDKVN----------K-ED---IKIRFF-EENDN--------GTLNWEDYGDFGQG---DVHRQVSV-  237
_XP_035824873.1_   195  LLCDKVN----------K-ED---IKVRFY-EENEQ--------GIVSWEDWGDFGQG---DVHKQVRVI  238
_AAY27981.1_       193  LLCEKVS----------K-DD---IKVRFF-ENDKE--------GNCIWEDFGSFGHS---DVHRQYAIV  236
_XP_052827753.1_   192  LLCEKVA----------K-DN---IKVRFF-EEDS------------TWEAHGEFNPS---DVHHQYAIV  231
_XP_029642523.1_   192  LLCEKVA----------K-DN---IRVRFF-EEDS------------PWEALGEFNPS---DVHHQYAIV  231
```

```
_CAE1288013.1_      152  LLCEKVS----------K-DD---IKVRFF-ENDKE--------GNCLWEDFGSFGHT---DVHRQCAIV  195
_ELU04547.1_        193  LLCEKVT----------K-DD---IQVRFY-EENNL--------KEVIWEEYGEFAPA---DIHRQFAIV  236
_KAI0227899.1_      191  IFTEKIQ----------K-DN---IQVRFF-ETTQD---------GMVWEAFADFGQG---DVHRQFAIV  233
_XP_044002103.1_    198  LLCERVW----------E-ND---IQVRFY-GEKN---------NEIIWDKNAVVEDI---YIHKKVTII  240
_XP_044002102.1_    197  LLCEKVL----------K-DD---IQVRFF-QKIN---------DEVIWEKNAEFTTN---DVHKQVAIT  239
_XP_044002099.1_    197  LLCEKVL----------K-DD---IQVRFF-QKIN---------DEVIWEKNAEFTTN---DVHKQVAIT  239
_XP_037026629.1_    197  ILCDKVD----------K-RD---IRVRFY-ETGTD--------NSVIWLDWAKFQPK---DVHKQL---  237
_CAD7632570.1_      198  LFCNYID----------E-ED---IEVRFY-ETDAA-------TGLVVWESIADLKPPV-GEVHKRVGIS  244
_CAD7650738.1_      198  LFCNYID----------E-ED---IEVRFY-ELDTT--------GAVVWESIADLKPPV-GEVHKRVGIS  243
_XP_054163335.1_    196  LFTSFVL----------R-DD---IEVRFY-ELDDQ--------NQVKWQDSGK----------------  226
_CAD7629985.1_      203  LFTSFVL----------R-DD---IEVRFY-ELDDQ--------NAVKWQDFG--VIA---FIYRHIGIS  244
_CAD7652069.1_      196  LFTSFVL----------R-DD---IEVRFY-ELDDQ--------NGVKWQDFG--VIA---FIYRHIGIS  237
_XP_017954168.1_    195  LLCEKIS----------K-DD---IRIRFF-ETNEA--------GVEVWEAYADFQPT---DIYKQTAIV  238
_XP_017858583.1_    195  LLCEKIS----------K-DD---IRIRFF-ETNEA--------GVEVWEAYADFQPT---DIYKQTAIV  238
_XP_002001588.1_    195  LLCEKIS----------K-DD---IRIRFF-ETNEA--------GVEVWEAYADFQPT---DIYKQTAIV  238
_XP_017954163.1_    195  LLCEKIS----------K-DD---IRIRFF-ETNEA--------GVEVWEAYADFQPT---DIYKQTAIV  238
_XP_023164563.2_    195  LLCEKIS----------K-DD---IRIRFF-ETNEA--------GVEVWEAYADFQPT---DIYKQTAIV  238
_TDG46702.1_        195  LLCEKIS----------K-DD---IRIRFF-ETNEA--------GVEVWEAYADFQPT---DIYKQTAIV  238
_XP_030564898.1_    195  MLCEKIS----------K-DD---IRIRFF-ETNEA--------GVEIWEAYADFQPT---DIYKQTAIV  238
_XP_015024496.1_    195  MLCEKIS----------K-DD---IRIRFF-ETNEA--------GVEIWEAYADFQPT---DIYKQTAIV  238
_XP_034475238.1_    196  MLCDKVS----------K-ND---IRIRFF-ETNDE--------GDVVWEADATFQNQ---DVYKQMAIV  239
_XP_034475235.1_    196  MLCDKVS----------K-ND---IRIRFF-ETNDE--------GDVVWEADATFQNQ---DVYKQMAIV  239
_XP_017854415.1_    195  MLCEKIN----------K-DD---IKIRFF-ETNEV--------GEEVWHDFAEFQAT---DVYKQVAIA  238
_XP_017854416.1_    195  MLCEKIN----------K-DD---IKIRFF-ETNEV--------GEEVWHDFAEFQAT---DVYKQVAIA  238
_ALC40089.1_        195  MLCEKIN----------K-DD---IKIRFF-ETNEV--------GEEVWHDFAEFQAT---DVYKQVAIA  238
_XP_030382983.1_    198  MLCENIN----------K-ND---IRVRFF-ETNEQ--------NDVIWEADAEFQKT---DIYKQMAIA  241
_XP_030382981.1_    198  MLCENIN----------K-ND---IRVRFF-ETNEQ--------NDVIWEADAEFQKT---DIYKQMAIA  241
_XP_001989397.1_    195  MLCEKIN----------K-DD---IRIRFF-ETNEA--------GVEIWEAYAEFQPT---DIYKQTAIV  238
_XP_002067135.2_    196  MLCEKIA----------K-DD---IQIRFF-ETNDD--------GVEVWEAYAEFQQT---DIFKQMAIA  239
_XP_034475239.1_    194  MLCDKVS----------K-ND---IRIRFF-ETNDE--------GDVVWEADATFQNQ---DVYKQMAIV  237
_KAH8411901.1_      196  MLCEKVT----------R-GD---IKIRFF-ETNDD--------GVEIWEDYASFQPH---DIHKQSAIA  239
_XP_016963143.1_    196  MLCEKIA----------K-DD---IEVRFY-ETDRD--------GRETWFANAEFQPT---DVFKQMAIA  239
_XP_037712361.1_    196  MLCEKIA----------K-DD---IEVRFY-ETDRD--------GRETWFANAEFLPT---DVFKQMAIT  239
_XP_016993627.2_    196  MLCEKIA----------K-DD---IEVRFY-ETDRD--------GRETWFANAEFQPT---DVFKQMAIA  239
_XP_016963141.1_    196  MLCEKIA----------K-DD---IEVRFY-ETDRD--------GRETWFANAEFQPT---DVFKQMAIA  239
_XP_016993624.2_    196  MLCEKIA----------K-DD---IEVRFY-ETDRD--------GRETWFANAEFQPT---DVFKQMAIA  239
_XP_037712358.1_    196  MLCEKIA----------K-DD---IEVRFY-ETDRD--------GRETWFANAEFLPT---DVFKQMAIT  239
_XP_001974449.1_    196  MLCEKIA----------K-DD---IEVRFY-ETDKD--------GRETWFANAEFQPT---DVFKQMAIA  239
_AAM48404.1_        196  MLCEKIA----------K-DD---IEVRFY-ETDKD--------GRETWFANAEFQPT---DVFKQMAIA  239
```

```
_NP_001162999.1_  196  MLCEKIA----------K-DD---IEVRFY-ETDKD--------GRETWFANAEFQPT---DVFKQMAIA  239
_XP_026835878.1_  196  MLCEKIA----------K-DD---IEVRFY-ETDKD--------GRETWFANAEFQPT---DVFKQMAIA  239
_AAA28465.1_      196  MLCEKIA----------K-DD---IEVRFY-ETDKD--------GRETWFANAEFQPT---DVFKQMAIA  239
_XP_002038912.1_  196  MLCEKIA----------K-DD---IEVRFY-ETDKD--------GRETWYANAEFQPT---DVFKQMAIA  239
_XP_033169558.1_  196  MLCEKIA----------K-DD---IEVRFY-ETDKD--------GRETWYANAEFQPT---DVFKQMAIA  239
_EDX05310.1_      196  MLCEKIA----------K-DD---IEVRFY-ETDKD--------GRETWYANAEFQPT---DVFKQMAIA  239
_NP_001162998.1_  196  MLCEKIA----------K-DD---IEVRFY-ETDKD--------GRETWFANAEFQPT---DVFKQMAIA  239
_XP_032579766.1_  196  MLCEKIA----------K-DD---IEVRFY-ETDKD--------GRETWYANAEFQPT---DVFKQMAIA  239
_XP_033169541.1_  196  MLCEKIA----------K-DD---IEVRFY-ETDKD--------GRETWYANAEFQPT---DVFKQMAIA  239
_XP_034669502.1_  196  MLCEKIS----------K-DD---IEIRFY-ETDED--------GRELWHANAEFQPT---DVFKQMAIA  239
_XP_034125957.1_  196  MLCEKIS----------K-DD---IEIRFY-ETDED--------GRELWHANAEFQPT---DVFKQMAIA  239
_XP_034669501.1_  196  MLCEKIS----------K-DD---IEIRFY-ETDED--------GRELWHANAEFQPT---DVFKQMAIA  239
_XP_034125956.1_  196  MLCEKIS----------K-DD---IEIRFY-ETDED--------GRELWHANAEFQPT---DVFKQMAIA  239
_XP_034125955.1_  196  MLCEKIS----------K-DD---IEIRFY-ETDED--------GRELWHANAEFQPT---DVFKQMAIA  239
_XP_017152033.1_  196  MLCEKIS----------K-DD---IEIRFY-ETDDD--------GRELWHANAEFQPT---DVFKQMAIA  239
_XP_017152031.1_  196  MLCEKIS----------K-DD---IEIRFY-ETDDD--------GRELWHANAEFQPT---DVFKQMAIA  239
_XP_002014799.1_  196  MLCEKIS----------K-DD---IEIRFY-ETDDD--------GRELWHANAEFQPT---DVFKQMAIA  239
_XP_026844902.1_  196  MLCEKIS----------K-DD---IEIRFY-ETDDD--------GRELWHANAEFQPT---DVFKQMAIA  239
_XP_015035983.2_  196  MLCEKIS----------K-DD---IEIRFY-ETDDD--------GRELWHANAEFQPT---DVFKQMAIA  239
_XP_017063896.1_  196  MLCEKIA----------K-DD---IEVRFY-ETDRD--------GRETWFANAEFHPT---DVYKQMAIA  239
_XP_016935332.1_  196  MLCEKIA----------K-DD---IEVRFY-ETDRD--------GRETWFANAEFQPT---DVFKQMAIA  239
_XP_017063894.2_  196  MLCEKIA----------K-DD---IEVRFY-ETDRD--------GRETWFANAEFHPT---DVYKQMAIA  239
_XP_036668637.1_  196  MLCEKIA----------K-DD---IEVRFY-ETDRD--------GRETWFANAEFQPT---DVFKQMAIA  239
_XP_016935310.1_  196  MLCEKIA----------K-DD---IEVRFY-ETDRD--------GRETWFANAEFQPT---DVFKQMAIA  239
_KAH8298318.1_    196  MLCEKIA----------K-DD---IEVRFY-ETDED--------GRELWHANAEFQPT---DVFKQMAIA  239
_KAH8412187.1_    196  MLCEKIA----------K-DD---IEVRFY-ELDED--------GREIWHANAEFQPT---DVFKQMAIA  239
_KAH8278042.1_    195  MLCDRVA----------K-DD---IEVVFY-ETDRD--------GHEIWSENAEFKPT---DVFKQMAIA  238
_KAH8366423.1_    196  MLCEKIA----------K-DD---IEVRFY-ETDGD--------GRETWFANAEFQPT---DVFKQMAIA  239
_XP_017044860.1_  197  MLCEKIL----------K-DD---IEVRFY-ETDKD--------GQETWFANAEFQPT---DVFKQTAIT  240
_KAH8324526.1_    194  MLCDRVA----------K-DD---IDVVFY-ETDRD--------GREIWSEKADFKPT---DVFKQMAIA  237
_XP_017116817.1_  196  MLCEKIA----------K-DD---IEVRFY-ETDSN--------GRETWFANAEFQPT---DVFKQMAIA  239
_XP_041566118.1_  195  MLCEKIA----------K-DD---IEVRFY-ETDSN--------GRETWFANAEFQPT---DVFKQMAIA  238
_XP_052852584.1_  196  MLCEKIA----------K-DD---IEVRFY-ETDSN--------GRETWFANAEFQPT---DVFKQMAIA  239
_XP_052852582.1_  196  MLCEKIA----------K-DD---IEVRFY-ETDSN--------GRETWFANAEFQPT---DVFKQMAIA  239
_XP_017107132.2_  191  MWCERVA----------K-DD---IEVVFY-ETDRD--------GHEIWSEIAEFKPT---DVFKQTSIA  234
_XP_043069650.1_  191  MWCERVA----------K-DD---IEVVFY-ETDRD--------GHEIWSEIAEFKPT---DVFKQTSIA  234
_KAH8283158.1_    197  MLCEKIA----------K-DD---IEVRFY-ETDGD--------GRETWHANAEFLPT---DVFKQMAIA  240
_XP_017025640.1_  195  MLCERIG----------K-DD---IEVRFY-ETDAD--------GRETWHANAEFLPT---DVFKQMAIA  238
_XP_020803071.1_  196  MLCERVA----------K-DD---IEVRFY-ETDGD--------GRETWHANAEFLPT---DVFKQMAIA  239
```

```
_XP_017025638.1_  196  MLCERIG----------K-DD---IEVRFY-ETDAD--------GRETWHANAEFLPT---DVFKQMAIA  239
_KAH8308067.1_    196  MLCERIG----------K-DD---IEVRFY-ETDAD--------GRETWHANAEFLPT---DVFKQMAIA  239
_KAH8362606.1_    196  MLCERVA----------K-DD---IEVRFY-ETDGD--------GRETWHANAEFLPT---DVFKQMAIA  239
_XP_020803068.1_  196  MLCERVA----------K-DD---IEVRFY-ETDGD--------GRETWHANAEFLPT---DVFKQMAIA  239
_XP_015052364.1_  196  MLCDNIT----------K-DD---IEVHFY-ETDED--------GRETWIAKADFKST---DVFRQMAIA  239
_XP_002090469.1_  195  MLCDNIT----------K-DD---IEVHFY-ETDED--------GRETWIAKADFKST---DVFRQMAIA  238
_XP_039480354.1_  195  MLCDNIT----------K-DD---IEVHFY-ETDED--------GRETWIAKAEFKST---DVFRQMAIA  238
_XP_039480352.1_  195  MLCDNIT----------K-DD---IEVHFY-ETDED--------GRETWIAKAEFKST---DVFRQMAIA  238
_XP_041450795.1_  196  MLCEKIS----------K-DD---IEIRFY-ETDDD--------GRELWHANGEFQPT---DVFKQMAIT  239
_XP_044315461.1_  197  MLCEKIA----------K-DD---IEVRFY-ETDRD--------GRETWFANAEFQPT---DVFKQMAIT  240
_XP_044315463.1_  196  MLCEKIA----------K-DD---IEVRFY-ETDRD--------GRETWFANAEFQPT---DVFKQMAIT  239
_XP_001961710.2_  196  MLCDRVA----------K-DD---IEVVFY-ETDSH--------GREIWSQNAEFKPT---DVFKQMAIT  239
_XP_014761890.1_  196  MLCDRVA----------K-DD---IEVVFY-ETDSH--------GREIWSQNAEFKPT---DVFKQMAIT  239
_KAH8323309.1_    196  MLCDRVA----------K-DD---IEVVFY-ETDSH--------GREIWSQNAEFKPT---DVFKQMAIT  239
_XP_043646206.1_  195  MLCDNII----------K-DD---VEVRFY-ETDEE--------GRETWSANAEFKPT---DVFRQMAIA  238
_XP_043646204.1_  195  MLCDNII----------K-DD---VEVRFY-ETDEE--------GRETWSANAEFKPT---DVFRQMAIA  238
_AAQ65066.1_      196  MLCEKIA----------K-DD---IEVRFY-ET--------------------------------------  213
_AAQ64900.1_      196  MLCEK-----------------------------------------------------------------  200
_AAQ64905.1_      196  MLCEK-----------------------------------------------------------------  200
_KAH8378209.1_    197  LLCEKVN----------R-ND---IKIRFF-ETNAD--------DVVVWEAYGIFGNH---NVYKQTAIA  240
_KAH8293207.1_    197  LLCEKVN----------S-KD---IHIRFY-ETDEA--------QNVVWEDFGVSL-----YVHKQTAIA  238
_KAH8391978.1_    196  LLCEKVN----------R-ND---IKIRFF-ETNAD--------DVVVWEAYGIFGNH---NVYKQTAIA  239
_XP_034101352.1_  196  LLCEKVN----------R-ND---IKIRFF-ETNAD--------DVVVWEAYGIFGNH---NVYKQTAIA  239
_KAJ3658269.1_    199  LLCTKVT----------K-DD---IKIRFF-ETRN---------NQVVWEDFGTFRAN---DIYNNTAIC  241
_KAJ3658270.1_    199  LLCTKVK----------T-DD---LKIRFF-EMKD---------NEVVWEDFGTFHSD---DIYNNTAIC  241
_KAJ3658271.1_    199  LLGCKVV----------H-GD---IRIRFF-ETMN---------DQVVWEDFGSFQSD---DIYNKTAIC  241
_XP_015117085.1_  198  IFGQKII----------K-ED---VKVRFF-ETQE---------DKVIWEASEDSLPR--NNYDNQMSIT  241
_XP_011307643.1_  198  IFGQKII----------K-ED---IKVRFF-EIQE---------DKVVWEASEDSLPR--NNHDNQTTIT  241
_XP_011307741.1_  198  IFCQKIM----------K-DD---IKVRFF-EERK---------GRVVWETFGNFHPS--RDVFEQSAII  241
_XP_015119445.1_  198  ISGHKVI----------E-ED---VKIRFF-EMKK---------DQVVWAALKDPLPR--NNHDDQTTIS  241
_XP_052128594.1_  209  MFCEKVV----------K-DD---VEIRFF-EQRDQ-------TGPVEWEALGQFHPS---NVHKGFGIA  253
_XP_052128597.1_  200  MFCEKVV----------K-DD---VEIRFF-EQRDQ-------TGPVEWEALGQFHPS---NVHKGFGIA  244
_KAE8741315.1_    155  MFCEKVV----------K-DD---VEIRFF-EQRDQ-------TGPVEWEALGQFHPS---NVHKGFGIA  199
_XP_028982160.1_  202  MLCRKVA----------R-SD---IQVRFF-EEKE---------GRVVWQAFADPKYL---YFFKQISIK  244
_XP_011307645.1_  202  MLCKKVA----------K-SD---IQVRFY-EEKG---------GRVVWQAFADPKYL---HFFKQISIK  244
_XP_054725384.1_  192  LLCNKVV----------K-ED---VSVRFY-EEQN---------GVTTWEAFGSFEIY---DVHKQSSIR  234
_XP_053965385.1_  192  LLCNRVV----------K-ED---VSVRFY-EVQN---------GVRTWEAFGSFEIY---DVHKQSSIR  234
_XP_013149158.1_  204  LLCKKVK----------P-SD---TTVVFV-ERRG----------DKDWEMPASNI-----SVHQNVAFC  243
_XP_045534655.1_  204  LLCKKVK----------P-SD---TTVVFV-ERGAG--------GGAVWEAAASDV-----SAHQHVAFC  245
```

```
_KPJ06852.1_        204  LLCKKVK----------P-SD---TTVVFV-ERGAG--------GGAVWEAAASDV-----SAHQHVAFC  245
_XP_013176418.1_    204  LLCKKVK----------P-SD---TTVVFV---QG----------SEDWEVAASDI-----SSHQHVAFC  241
_OXU28425.1_        220  ILCEKVP----------S-ND---IEVRFF-HKDI------------KWEALGKFVGP---SIFQKVAII  259
_XP_008216913.1_    201  ILCERVP----------R-ND---IEVRFF-HKDS------------KWEAFGRFVGP---SIFQKVAII  240
_XP_008216914.1_    197  ILCERVP----------R-ND---IEVRFF-HKDS------------KWEAFGRFVGP---SIFQKVAII  236
_XP_040572301.1_    193  ILTNKVY----------K-DD---IEVHFT-NESEG--------RRESWHAKGISL-----SVHKQHALS  234
_CAB4068408.1_      112  --------------------------VHFT-NESEG--------RRESWHAKGVSL-----SVHKQHAIS  141
_KAF7993284.1_      181  LVGDNIS----------K-ND---KVVMFE-VNQN---------GKRVWEGVSSFRQS---DLIKKSAVT  223
_KAF7987489.1_      201  LLCDKVS----------K-KD---IKVLLY-QKDDN--------NKIIWKRYADII-----GVHRQIGIT  242
_KAF7994520.1_      203  LLCDKVS----------K-HD---IKVLFY-QQDND--------SNIIWKKYAEIV-----GVHRQIGIT  244
_XP_044019368.1_    207  LLCNKVS----------K-N----IRVLFY-QKDND--------DNIIWQRYAYII-----DVYRQVAIT  247
_XP_044019332.1_    107  LLCDKVS----------K-KD---IKVLLY-QKDDN--------NKIIWKRYADII-----GVHRQIGIT  148
_XP_044003986.1_     76  LLCDKVS----------K-HD---IKVLFY-QQDND--------SNIIWKKYAEIV-----GVHRQIGIT  117
_XP_037042732.1_    199  IFCNKIA----------Q-DD---IEIRFY-EEDAK--------KNQKLIGLGSFQPR---NIHRNVGIA  242
_CAD7223243.1_      193  ILCEKVS----------K-DD---IHVEFY-EEDIA--------GKIIWSEEGKFTPN---DVHKQYAIS  236
_XP_044732233.1_    198  MFCEKIV----------K-SD---TIKIRF-IEEQD--------NQIVWESCGTFGPS---DVHKQYGIA  241
_XP_012231885.1_    193  LLCDKVV----------K-ED----IKVIF-FEKRD--------EKLIWKGFGNVQ-----YVHRQVAIA  233
_XP_022814699.1     198  ILCS-VV----------K-EE---IEVVFF-TKPTD--------HEPAWESHAMGL-----KVHGTQSLY  238
_CAB3516523.1_      198  ILCS-VV----------K-EE---IEVVFF-TKPSD--------SEPAWESHAMGL-----KVHGTQSLY  238
_XP_035458245.2_    197  ILCT-VV----------K-EE---TEVVFY-TKPTE--------NEPAWERHAMGL-----KVHGTQSLY  237
_KAF9795795.1_      186  ILCT-VV----------K-EE---TEVVFY-TKPAK--------NGPAWERHAMGV-----KVHGTQSLY  226
_XP_014233257.1_    208  LLAGRVE----------R-DD---VEVHFY-DDAN------------EWQAVGLPYPP---GVYENTAIV  247
_XP_034324400.1_    189  LTADSFI----------P-KG---IEVRFT-DNE-------------GWNVKAEKP-----NVIKN-VLE  224
_XP_052699907.1_    189  LTGDSFI----------P-KG---IEVRFT-DNE-------------GWNVKAEKP-----NVIKN-VLE  224
Consensus_aa:            lLCp+l...........+.cD...Ipl.Fh.ppp..............Wps.tph..s...pV++phAIs
Consensus_ss:            eee            -         eeeee ee             eeeeeeeee hh   hh     eee


Conservation:            5  8       5                             5                         5
_XP_001652840.2_    240  FRTP------RYRTLE------------VEHP---VMVNIQLK---RPSDG-------ATSE-PLP-FEL  276
_AAW67214.1_        240  FRTP------RYRTLE------------VEHP---VMVNIQLK---RPSDG-------ATSE-PLP-FEL  276
_XP_021704027.1_    240  FRTP------RYRTLE------------VEHP---VMVNIQLK---RPSDG-------ATSE-PLP-FEL  276
_XP_019540833.2_    240  FRTP------RYRTLD------------VEHH---VMVNIQLK---RPSDG-------ATSE-PLP-FEL  276
_XP_029720231.1_    240  FRTP------RYRTLD------------VEHH---VMVNIQLK---RPSDG-------ATSE-PLP-FEL  276
_AAW67215.1_        240  FRTP------RYRTLE------------VEHP---VMVNIQLK---RPSDG-------ATSE-PLP-FEL  276
_XP_021704025.1_    240  FRTP------RYRTLE------------VEHP---VMVNIQLK---RPSDG-------ATSE-PLP-FEL  276
_XP_019540831.2_    240  FRTP------RYRTLD------------VEHH---VMVNIQLK---RPSDG-------ATSE-PLP-FEL  276
_KXJ81105.1_        240  FRTP------RYRTLD------------VEHH---VMVNIQLK---RPSDG-------ATSE-PLP-FEL  276
_KXJ76497.1_        240  FRTP------RYRTLD------------VEHH---VMVNIQLK---RPSDG-------ATSE-PLP-FEL  276
```

```
_XP_023240428.1_  239  FRTP------RYHDES------------VKYP---VNVNVQLV---RPSNN-------EKSL-PRP-FQL  275
_CAD7241875.1_    241  LRTP------RYKSLE------------VKDL---VLCQIQLE---RPSDG-------EHSE-PRD-FQY  277
_TRY80291.1_      237  LKTP------EYVDKD------------ITEP---VLGAMYLY---KPKTQ-------ESSE-PVN-FYY  273
_ALF84899.1_      234  FVTP------PFTDQK------------ISQE---VLTSMYLF---KPSEQ-------AQSD-AID-FYF  270
_KAF4528208.1_    240  FRTP------AYKDLQ------------ITKE---VDVKIQLR---RPSDN-------ATSE-PKS-FKY  276
_XP_054260955.1_  241  FKTP------RYHTMQ------------VDKP---VQVFIQLQ---RPSDH-------ETSE-PLP-FQL  277
_XP_054264401.1_  241  FKTP------RYHTMQ------------VDKP---VQVFIQLQ---RPSDH-------ETSE-PLP-FQL  277
_XP_054260954.1_  241  FKTP------RYHTMQ------------VDKP---VQVFIQLQ---RPSDH-------ETSE-PLP-FQL  277
_XP_038114042.1_  240  FRTP------RYRTLE------------IEQS---VMVNIQLR---RPSDG-------ATSE-PLP-FEL  276
_XP_039437558.1_  240  FRTP------RYRTLE------------IEQS---VMVNIQLR---RPSDG-------ATSE-PLP-FEL  276
_XP_001844078.2_  240  FRTP------RYRTLE------------IEQS---VMVNIQLR---RPSDG-------ATSE-PLP-FEL  276
_EDS35747.1_      240  FRTP------RYRTLE------------IEQS---VMVNIQLR---RPSDG-------ATSE-PLP-FEL  276
_XP_053696925.1_  240  FRTP------RYRTIE------------VEHS---VMVNIQLR---RPSDG-------ATSE-PLP-FEL  276
_XP_001869619.2_  240  FRTP------RYHTLE------------TSQP---VMVNIQLR---RPSDG-------ATSE-PLP-FEL  276
_XP_039434954.1_  240  FRTP------RYHTLE------------TSQP---VMVNIQLR---RPSDG-------ATSE-PLP-FEL  276
_EDS29879.1_      240  FRTP------RYHTLE------------TSQP---VMVNIQLR---RPSDG-------ATSE-PLP-FEL  276
_XP_019550742.2_  240  FRTP------RYRSLD------------VEHS---VMVHIQLK---RPSDG-------ATSE-PVP-FEL  276
_KXJ74587.1_      240  FRTP------RYRSLD------------FEHS---VMVHIQLK---RPSDG-------ATSE-PVP-FEL  276
_XP_035908773.1_  241  FRTP------PYKAID------------ISDP---VRVYVQLE---RPSDN-------TYSD-ARD-FQF  277
_AJC98655.1_      241  FRTP------PYRTID------------ISDP---VRVYVQLE---RPSDN-------TYSE-ARD-FQF  277
_AJC98650.1_      241  FRTP------PYRTID------------ISDP---VRVYVQLE---RPSDN-------TYSE-ARD-FQF  277
_AJC98644.1_      241  FRTP------PYRTID------------ISDP---VRVYVQLE---RPSDN-------TYSE-ARD-FQF  277
_AJC98657.1_      241  FRTP------PYRTID------------ISDP---VRVYVQLE---RPSDN-------TYSE-ARD-FQF  277
_AJC98648.1_      241  FRTP------PYRTID------------ISDP---VRVYVQLE---RPSDN-------TYSE-ARD-FQF  277
_AJC98647.1_      241  FRTP------PYRTID------------ISDP---VRVFVQLE---RPSDN-------TYSE-ARD-FQF  277
_AJC98651.1_      241  FRTP------PYRTID------------ISDP---VRVFVQLE---RPSDN-------TYSE-ARD-FQF  277
_AJC98643.1_      241  FRTP------PYRTID------------ISDP---VRVFVQLE---RPSDN-------TYSE-ARD-FQF  277
_AJC98656.1_      241  FRTP------PYRTID------------ISDP---VRVFVQLE---RPSDN-------TYSE-ARD-FQF  277
_AJC98641.1_      241  FRTP------PYRTID------------ISDP---VRVFVQLE---RPSDN-------TYSE-ARD-FQF  277
_AJC98640.1_      241  FRTP------PYRTID------------ISDP---VRVFVQLE---RPSDN-------TYSE-ARD-FQF  277
_AJC98653.1_      241  FRTP------PYRTID------------ISDP---VRVFVQLE---RPSDN-------TYSE-ARD-FQF  277
_AJC98654.1_      241  FRTP------PYRTID------------ISDP---VRVFVQLE---RPSDN-------TYSE-ARD-FQF  277
_AJC98652.1_      241  FRTP------PYRTID------------ISDP---VRVFVQLE---RPSDN-------TYSE-ARD-FQF  277
_AJC98646.1_      241  FRTP------PYRTID------------ISDP---VRVFVQLE---RPSDN-------TYSE-ARD-FQF  277
_AJC98658.1_      241  FRTP------PYRTID------------ISDP---VRVFVQLE---RPSDN-------TYSE-ARD-FQF  277
_AJC98639.1_      241  FRTP------PYRTID------------ISDP---VRVFVQLE---RPSDN-------TYSE-ARD-FQF  277
_XP_035908772.1_  241  FRTP------PYKAID------------ISDP---VRVYVQLE---RPSDN-------TYSD-ARD-FQF  277
_XP_050076803.1_  241  FRTP------QYKPMD------------ITDP---VRVYVQLE---RPSDN-------TYSE-ARD-FQF  277
_XP_049282441.1_  241  FRTP------PYKAIE------------ISDP---VRVYVQLE---RPSDN-------TYSD-ARD-FQF  277
```

```
_XP_040170080.1_   241  FRTP------PYRTID------------ISDP---VRVFVQLE---RPSDN-------TYSE-ARD-FQF  277
_XP_050076802.1_   241  FRTP------QYKPMD------------ITDP---VRVYVQLE---RPSDN-------TYSE-ARD-FQF  277
_XP_041780138.1_   241  FRTP------PYRTID------------ISDP---VRVFVQLE---RPSDN-------TYSE-ARD-FQF  277
_XP_040239229.2_   241  FRTP------PYRTID------------ISDP---VRVFVQLE---RPSDN-------TYSE-ARD-FQF  277
_XP_040239222.2_   241  FRTP------PYRTID------------ISDP---VRVFVQLE---RPSDN-------TYSE-ARD-FQF  277
_XP_035908780.1_   241  FRTP------PYKAID------------ISDP---VRVYVQLE---RPSDN-------TYSD-ARD-FQF  277
_XP_040239221.2_   241  FRTP------PYRTID------------ISDP---VRVFVQLE---RPSDN-------TYSE-ARD-FQF  277
_XP_052897786.1_   241  FRTP------PYKAID------------ISDP---ARVYVQLE---RPSDN-------TYSE-ARD-FQF  277
_XP_052895493.1_   241  FRTP------PYKAID------------ISDP---ARVYVQLE---RPSDN-------TYSE-ARD-FQF  277
_XP_053664395.1_   241  FRTP------PYKAIE------------ISDP---VRVYVQLE---RPSDN-------TYSE-VRD-FQF  277
_XP_050076801.1_   241  FRTP------QYKPMD------------ITDP---VRVYVQLE---RPSDN-------TYSE-ARD-FQF  277
_XP_310177.3_      241  FRTP------PYRTID------------ISDP---VRVFVQLE---RPSDN-------TYSE-ARD-FQF  277
_XP_001652339.1_   240  FRTP------RYRSLD------------VDFP---VMVHIQLQ---RPSDG-------ATSE-PVP-FEL  276
_XP_053686256.1_   240  FRTP------RYRTLD------------VEHS---VMVNIQLR---RLSDG-------ATSE-PLP-FEL  276
_XP_053675540.1_   241  FRTP------PYKSLN------------ITDP---VKVYVQLE---RPSDN-------TYSE-ARD-FQL  277
_ETN66814.1_       240  FRTP------QYRTLD------------ITEP---VTVFVRLE---RPSDN-------ACSE-SLE-FMF  276
_XP_049534501.1_   240  FRTP------QYRTLD------------LTEP---VTVFVRLE---RPSDN-------ACSE-SLE-FMF  276
_XP_050084431.1_   240  FRTP------PYRTLD------------ITEP---VTVFVRLE---RPSDN-------ACSE-SLE-FLM  276
_XP_035782874.1_   240  FRTP------QYRTQD------------ITEP---VTVFVRLE---RPSDN-------ACSE-SLE-FLM  276
_XP_035782869.1_   240  FRTP------QYRTQD------------ITEP---VTVFVRLE---RPSDN-------ACSE-SLE-FLM  276
_KFB39849.1_       248  FRTP------TYRAMD------------VTDP---VKVYVQLE---RPSDG-------AQSD-ARD-FLF  284
_XP_052871375.1_        ----------------------------------------------------------------------
_AAR13743.1_            ----------------------------------------------------------------------
_AAR13744.1_            ----------------------------------------------------------------------
_CAA65157.1_            ----------------------------------------------------------------------
_XP_022170156.1_   239  FRTP------KYKIMQ------------IDGP---VNVWIQLK---RPSDG-------MCSE-ARP-FLF  275
_XP_025205168.1_   239  FRTP------KYKVMQ------------IDGP---INVWIQLK---RPTDG-------MCSE-ARP-FLF  275
_XP_026817162.1_   239  FRTP------KYKVMQ------------IDGP---INVWIQLK---RPTDG-------MCSE-ARP-FLF  275
_XP_027846205.1_   239  FRTP------KYKVMQ------------IDGP---INVWIQLK---RPTDG-------MCSE-ARP-FLF  275
_XP_015370635.1_   239  FRTP------KYKILQ------------IDGP---VNVWIQLK---RPSDG-------MSSE-ARP-FLF  275
_CAH1723797.1_     239  FRTP------KYKVMQ------------IDGP---INVWIQLK---RPTDG-------MCSE-ARP-FLF  275
_KAF0769940.1_     239  FRTP------KYKVMQ------------IDGP---INVWIQLK---RPTDG-------MCSE-ARP-FLF  275
_CAB4065202.1_     189  FLTP------PYFDKD------------IKQP---IKVSMYLY---KPCEN-------VASE-HVE-FQY  225
_XP_050440903.1_   239  FRTP------KYKTLQ------------VDYP---VKVWIQLK---RPTDG-------MCSD-ARP-FLL  275
_XP_050533799.1_   239  FRTP------KYKTLQ------------IDYP---IKVWIQLK---RPTDG-------MCSD-ARP-FLL  275
_XP_025410632.1_   240  FRTP------KYRTTD------------IQNR---VNVKIQLR---RPSDG-------MCSD-SRP-FIF  276
_XP_008180746.1_   239  FKTP------KYKITE------------IDSP---VNVWIQLK---RPSDG-------MCSD-ALP-FTF  275
_XP_026465450.1_   241  FRTP------SYKNQD------------IEKP---VKAFIQLR---RPSDG-------AVSE-PLA-FQF  277
_VVC36026.1_       239  FRTP------KYRTTD------------INST---VHVWIQLK---RPSDN-------MCSE-PRP-FML  275
```

```
_XP_016661522.1_   239  FKTP------KYKITE------------IDSP---VNVWIQLK---RPSDG-------MCSD-ALP-FTF  275
_XP_001947429.3_   239  FKTP------KYKITE------------IDSP---VNVWIQLK---RPSDG-------MCSD-ALP-FTF  275
_XP_026466007.1_   240  FRTP------SYKNQD------------IEKT--------------------------------------  253
_KRT81254.1_       237  FRPP------KYKTLD------------VIEP---VQMFIHLR---RPSDN-------ATSA-FLP-FQM  273
_XP_033224832.1_   244  FRTP------KYNNQE------------ITEP---VTVQVQLT---LRNKP-------ERGP-PRD-FEM  280
_XP_025407590.1_   232  FKTP------TYYLRN------------IENP---VHVKIQLR---RISDG-------KYGE-CYP-FIL  268
_XP_045476729.1_   233  VKVP------KYCNLE------------IETE---VEVMVQLK---RPSDN-------AFSA-AIP-FQY  269
_XP_034174253.1_   240  FRTP------TYRIQQ------------VDQP---VQVYIQLK---RPSDG-------ATSE-PLP-FQM  276
_XP_029047866.1_   240  FRTP------TYRIQQ------------VDQP---VQVYIQLK---RPSDG-------ATSE-PLP-FQM  276
_XP_015430648.1_   240  FRTP------TYRIQQ------------VDQP---VQVYIQLK---RPSDG-------ATSE-PLP-FQM  276
_XP_017798081.1_   240  FRTP------TYRIQQ------------VEQP---VQVSIQLK---RPSDG-------ATSE-PLP-FQM  276
_XP_017756471.1_   240  FRTP------TYRMQQ------------VEQP---VQVYIQLK---RPSDG-------ATSE-PLP-FQM  276
_XP_043248447.1_   240  FRTP------TYRIQQ------------VEQP---VQVFIQLR---RPSDG-------VASE-PLP-FEM  276
_XP_029047855.1_   240  FRTP------TYRIQQ------------VDQP---VQVYIQLK---RPSDG-------ATSE-PLP-FQM  276
_XP_034174252.1_   240  FRTP------TYRIQQ------------VDQP---VQVYIQLK---RPSDG-------ATSE-PLP-FQM  276
_XP_031829337.1_   240  FRTP------TYRIQQ------------VDQP---VQVYIQLK---RPSDG-------ATSE-PLP-FQM  276
_KZC09125.1_       240  FRTP------TYRIQQ------------VDQP---VQVYIQLK---RPSDG-------ATSE-PLP-FQM  276
_XP_033327213.1_   240  FRTP------TYRLQQ------------VDQP---VQVYIQLK---RPSDG-------ATSE-PLP-FQM  276
_XP_033327212.1_   240  FRTP------TYRLQQ------------VDQP---VQVYIQLK---RPSDG-------ATSE-PLP-FQM  276
_XP_017756445.1_   240  FRTP------TYRMQQ------------VEQP---VQVYIQLK---RPSDG-------ATSE-PLP-FQM  276
_OAD62393.1_       240  FRTP------TYRMQQ------------VEQP---VQVYIQLK---RPSDG-------ATSE-PLP-FQM  276
_XP_043248413.1_   240  FRTP------TYRIQQ------------VEQP---VQVFIQLR---RPSDG-------VASE-PLP-FEM  276
_XP_033327208.1_   240  FRTP------TYRLQQ------------VDQP---VQVYIQLK---RPSDG-------ATSE-PLP-FQM  276
_XP_034950477.1_   240  FRTP------TYRIQQ------------VDQP---VQVYIQLK---RPSDN-------ATSD-ALP-FQM  276
_XP_034950475.1_   240  FRTP------TYRIQQ------------VDQP---VQVYIQLK---RPSDN-------ATSD-ALP-FQM  276
_XP_012343274.1_   240  FRTP------TYRMQQ------------VEQP---VQVYIQLK---RPSDG-------ATSE-PFP-FLM  276
_XP_016909449.1_   240  FRTP------TYRMQQ------------VEQP---VQVYIQLK---RPSDG-------ATSE-PFP-FLM  276
_XP_012343273.1_   240  FRTP------TYRMQQ------------VEQP---VQVYIQLK---RPSDG-------ATSE-PFP-FLM  276
_XP_006619742.1_   240  FRTP------TYRMQQ------------VEQP---VQVYIQLK---RPSDG-------ATSE-PFP-FLM  276
_XP_006567065.1_   240  FRTP------TYRMQQ------------VEQP---VQVYIQLK---RPSDG-------ATSE-PFP-FLM  276
_XP_006619741.1_   240  FRTP------TYRMQQ------------VEQP---VQVYIQLK---RPSDG-------ATSE-PFP-FLM  276
_NP_001011577.1_   240  FRTP------TYRMQQ------------VEQP---VQVYIQLK---RPSDG-------ATSE-PFP-FLM  276
_XP_016909448.1_   240  FRTP------TYRMQQ------------VEQP---VQVYIQLK---RPSDG-------ATSE-PFP-FLM  276
_XP_043802365.1_   240  FRTP------TYRMQQ------------VEQP---VQVYIQLK---RPSDG-------ATSE-PFP-FLM  276
_NP_001164477.1_   240  FRTP------TYRMQQ------------VEQP---VQVYIQLK---RPSDG-------ATSE-PFP-FLM  276
_XP_016909443.1_   240  FRTP------TYRMQQ------------VEQP---VQVYIQLK---RPSDG-------ATSE-PFP-FLM  276
_XP_043802361.1_   240  FRTP------TYRMQQ------------VEQP---VQVYIQLK---RPSDG-------ATSE-PFP-FLM  276
_XP_012343269.1_   240  FRTP------TYRMQQ------------VEQP---VQVYIQLK---RPSDG-------ATSE-PFP-FLM  276
_XP_006619738.1_   240  FRTP------TYRMQQ------------VEQP---VQVYIQLK---RPSDG-------ATSE-PFP-FLM  276
```

```
_PBC29878.1_       240  FRTP------TYRMQQ------------VEQP---VQVYIQLK---RPSDG-------ATSE-PFP-FLM  276
_XP_046663984.1_   239  FKTP------RYRTLQ------------VEDP---VQVFIQLR---RPSDG-------ETSD-SLP-FQL  275
_XP_046663985.1_   239  FKTP------RYRTLQ------------VEDP---VQVFIQLR---RPSDG-------ETSD-SLP-FQL  275
_XP_043274771.1_   240  FRTP------TYRIQQ------------VEQP---VQVFIQLK---RPSDG-------AISE-SLP-FQM  276
_XP_043274769.1_   240  FRTP------TYRIQQ------------VEQP---VQVFIQLK---RPSDG-------AISE-SLP-FQM  276
_XP_043289571.1_   239  FRTP------SYRYQA------------IEEP---LPVYIQLK---RPSDG-------ATSE-PLP-FQY  275
_XP_032665873.1_   240  FRTP------TYRMQQ------------VEQP---VQVHIQLK---RPSDG-------ATSE-PLP-FQM  276
_XP_032665875.1_   240  FRTP------TYRMQQ------------VEQP---VQVHIQLK---RPSDG-------ATSE-PLP-FQM  276
_XP_032665874.1_   240  FRTP------TYRMQQ------------VEQP---VQVHIQLK---RPSDG-------ATSE-PLP-FQM  276
_XP_032665869.1_   240  FRTP------TYRMQQ------------VEQP---VQVHIQLK---RPSDG-------ATSE-PLP-FQM  276
_XP_014470289.1_   240  FRTP------TYRMQQ------------VEQP---VQVHIQLK---RPSDG-------ATSE-PLP-FQM  276
_XP_011149046.1_   240  FRTP------TYRMQQ------------VEQP---VQVHIQLK---RPSDG-------ATSE-PLP-FQM  276
_XP_017877914.1_   238  FRTP------TYRIQQ------------VEQP---VQVFIQLR---RPSDG-------ATSE-PLP-FQM  274
_XP_017877911.1_   238  FRTP------TYRIQQ------------VEQP---VQVFIQLR---RPSDG-------ATSE-PLP-FQM  274
_XP_033174774.1_   241  FKTP------TYRMQQ------------VEQP---VQICIQLK---RPSDG-------ATSE-PLP-FQM  277
_XP_043581013.1_   240  FKTP------TYRMQQ------------VEQP---VQICIQLK---RPSDG-------ATSE-PLP-FQM  276
_XP_048261507.1_   240  FKTP------TYRMQQ------------VEQP---VQICIQLK---RPSDG-------ATSE-PLP-FQM  276
_XP_050573946.1_   240  FKTP------TYRMQQ------------VEQP---VQICIQLK---RPSDG-------ATSE-PLP-FQM  276
_XP_050478075.1_   240  FKTP------TYRMQQ------------VEQP---VQICIQLK---RPSDG-------ATSE-PLP-FQM  276
_XP_033204249.1_   240  FKTP------TYRMQQ------------VEQP---VQICIQLK---RPSDG-------ATSE-PLP-FQM  276
_XP_033358451.1_   240  FKTP------TYRMQQ------------VEQP---VQICIQLK---RPSDG-------ATSE-PLP-FQM  276
_XP_033358450.1_   240  FKTP------TYRMQQ------------VEQP---VQICIQLK---RPSDG-------ATSE-PLP-FQM  276
_XP_033204244.1_   240  FKTP------TYRMQQ------------VEQP---VQICIQLK---RPSDG-------ATSE-PLP-FQM  276
_XP_033312956.1_   240  FKTP------TYRMQQ------------VEQP---VQICIQLK---RPSDG-------ATSE-PLP-FQM  276
_XP_012236639.1_   240  FKTP------TYRMQQ------------VEQP---VQICIQLK---RPSDG-------ATSE-PLP-FQM  276
_XP_050573940.1_   240  FKTP------TYRMQQ------------VEQP---VQICIQLK---RPSDG-------ATSE-PLP-FQM  276
_XP_012174118.1_   240  FKTP------TYRMQQ------------VEQP---VQICIQLK---RPSDG-------ATSE-PLP-FQM  276
_XP_043581010.1_   240  FKTP------TYRMQQ------------VEQP---VQICIQLK---RPSDG-------ATSE-PLP-FQM  276
_EZA51108.1_       240  FRTP------SYRTQQ------------VEQP---MQVYIQLK---RPSDG-------ATSE-PLP-FQM  276
_XP_011344085.1_   240  FRTP------SYRTQQ------------VEQP---MQVYIQLK---RPSDG-------ATSE-PLP-FQM  276
_XP_043519475.1_   240  FRTP------TYRIQQ------------VDQP---VQVYIQLK---RPSDG-------ATSE-PLP-FQM  276
_CAD1481114.1_     240  FRTP------TYRIQQ------------VEQP---VQVYIQLK---RPSDG-------ATSE-PLP-FQM  276
_XP_043519473.1_   240  FRTP------TYRIQQ------------VDQP---VQVYIQLK---RPSDG-------ATSE-PLP-FQM  276
_KOX75360.1_       240  FRTP------TYRIQQ------------VDQP---VQVYIQLK---RPSDG-------ATSE-PLP-FQM  276
_KAF3423741.1_     240  FRTP------TYRIQQ------------VDQP---VQVYIQLK---RPSDG-------ATSE-PLP-FQM  276
_KOC69050.1_       240  FRTP------TYRIQQ------------VEQP---VQVSIQLK---RPSDG-------ATSE-PLP-FQM  276
_XP_053980095.1_   240  FRTP------SYNNRI-----------QVDQP---VQVYIQLR---RPSDG-------TTSE-PLP-FQM  277
_XP_054002893.1_   240  FRTP------SYNNRI-----------QVDQP---VQVYIQLR---RPSDG-------TTSE-PLP-FQM  277
_XP_053980091.1_   240  FRTP------SYNNRI-----------QVDQP---VQVYIQLR---RPSDG-------TTSE-PLP-FQM  277
```

```
_XP_028049144.1_  240  FRTP------SYRTQQ------------VEQP---VQVYIQLR---RPSDG-------ATSE-PLP-FQM  276
_XP_012541458.1_  240  FRTP------SYRTQQ------------VEQP---VQVYIQLR---RPSDG-------ATSE-PLP-FQM  276
_XP_003708412.1_  240  FRTP------TYRIQQ------------VDQP---VQVYIQLK---RPSDG-------ATSE-PLP-FQM  276
_XP_012152807.1_  240  FRTP------TYRIQQ------------VDQP---VQVYIQLK---RPSDG-------ATSE-PLP-FQM  276
_KAH0945556.1_    240  FKTP------SYRIQQ------------VEQP---VQVYIQLK---RPSDG-------ATSE-PLP-FQM  276
_XP_024871832.1_  239  FRTP------SYRTQQ------------VEQP---VQVYIQLR---RPSDG-------TTSE-PLP-FQM  275
_XP_011307641.1_  238  FRTP------TYRIQQ------------VDQP---VQVYIQLR---RPSDG-------SASE-PLP-FQM  274
_XP_015118260.1_  239  FRTP------TYRIQQ------------VDQP---VQVYIQLR---RPSDG-------TASE-PLP-FQM  275
_XP_011307640.1_  239  FRTP------TYRIQQ------------VDQP---VQVYIQLR---RPSDG-------SASE-PLP-FQM  275
_XP_015118252.1_  239  FRTP------TYRIQQ------------VDQP---VQVYIQLR---RPSDG-------TASE-PLP-FQM  275
_EFN68841.1_      244  FRTP------SYRTQQ------------VEQP---VQVYIQLR---RPSDG-------ATSE-PLP-FQM  280
_XP_011647340.1_  240  FRTP------SYRTQQ------------VEQP---VQVYIQLR---RPSDG-------ATSE-ALP-FQM  276
_XP_029166122.1_  244  FRTP------SYRTQQ------------VEQP---VQVYIQLR---RPSDG-------ATSE-PLP-FQM  280
_KMQ96453.1_      244  FRTP------SYRTQQ------------VEQP---VQVYIQLR---RPSDG-------ATSE-PLP-FQM  280
_XP_050464492.1_  244  FRTP------SYRTQQ------------VEQP---VQVYIQLR---RPSDR-------ATSE-PLP-FQM  280
_XP_029673315.1_  244  FRTP------SYRTQQ------------VEQP---VQVYIQLR---RPSDG-------TTSE-PLP-FQM  280
_XP_025265518.1_  244  FRTP------SYRTQQ------------VEQP---VQVYIQLR---RPSDG-------ATSE-PLP-FQM  280
_XP_011255523.1_  244  FRTP------SYRTQQ------------VEQP---VQVYIQLR---RPSDG-------ATSE-PLP-FQM  280
_XP_011698133.1_  240  FRTP------SYHIQQ------------VEEP---VQVCIQLR---RPSDN-------ATSE-PLP-FQM  276
_XP_011698131.1_  240  FRTP------SYHIQQ------------VEEP---VQVCIQLR---RPSDN-------ATSE-PLP-FQM  276
_XP_018357420.1_  240  FRTP------SYRTQE------------VEQP---VQAFIQLR---RPSDG-------ATSE-PLP-FQM  276
_KYM76140.1_      240  FRTP------SYRTQE------------VEQP---VQAFIQLR---RPSDG-------ATSE-PLP-FQM  276
_XP_011647339.1_  240  FRTP------SYRTQQ------------VEQP---VQVYIQLR---RPSDG-------ATSE-ALP-FQM  276
_KAG5315434.1_    240  FRTP------SYRTQE------------VEQP---VQAFIQLR---RPSDG-------ATSE-PLP-FQM  276
_XP_011055463.1_  240  FRTP------SYRTQE------------VEQP---VQAFIQLR---RPSDG-------ATSE-PLP-FQM  276
_XP_018342550.1_  240  FRTP------SYRTQE------------VEQP---VQAFIQLR---RPSDG-------ATSE-PLP-FQM  276
_XP_018403565.1_  240  FRTP------SYRTQE------------VEQP---VQAFIQLR---RPSDG-------ATSE-PLP-FQM  276
_KAG5322912.1_    240  FRTP------SYRTQE------------VEQP---VQAFIQLR---RPSDG-------ATSE-PLP-FQM  276
_XP_018357417.1_  240  FRTP------SYRTQE------------VEQP---VQAFIQLR---RPSDG-------ATSE-PLP-FQM  276
_XP_018314560.1_  240  FRTP------SYRTQE------------VEQP---VQAFIQLR---RPSDG-------ATSE-PLP-FQM  276
_XP_018057217.1_  240  FRTP------SYRTQE------------VEQP---VQAFIQLR---RPSDG-------ATSE-PLP-FQM  276
_KAG5346558.1_    240  FRTP------SYRTQE------------VEQP---VQAFIQLR---RPSDG-------AMSE-PLP-FQM  276
_XP_047348408.1_  241  FRTP------SYCTQE------------IEQP---VQVYIQLK---RPSDG-------VTSD-PLP-FEM  277
_XP_047348412.1_  240  FRTP------SYCTQE------------IEQP---VQVYIQLK---RPSDG-------VTSD-PLP-FEM  276
_XP_046820611.1_  240  FRTP------SYCTQE------------IEQP---VQVYIQLK---RPSDG-------VTSD-PLP-FEM  276
_XP_050848254.1_  240  FRTP------SYCTQE------------IEQP---VQVYIQLK---RPSDG-------VTSD-PLP-FEM  276
_XP_046820612.1_  240  FRTP------SYCTQE------------IEQP---VQVYIQLK---RPSDG-------VTSD-PLP-FEM  276
_XP_050848252.1_  240  FRTP------SYCTQE------------IEQP---VQVYIQLK---RPSDG-------VTSD-PLP-FEM  276
_XP_043667564.1_  240  FRTP------SYCTQE------------IEQP---VQVYIQLK---RPSDG-------VTSD-PLP-FEM  276
```

```
_XP_035725337.1_  240  FRTP------SYCTQE------------IEQP---VQVYIQLK---RPSDG-------VTSD-PLP-FEM  276
_XP_043667562.1_  240  FRTP------SYCTQE------------IEQP---VQVYIQLK---RPSDG-------VTSD-PLP-FEM  276
_XP_046820605.1_  240  FRTP------SYCTQE------------IEQP---VQVYIQLK---RPSDG-------VTSD-PLP-FEM  276
_XP_050848249.1_  240  FRTP------SYCTQE------------IEQP---VQVYIQLK---RPSDG-------VTSD-PLP-FEM  276
_XP_043667560.1_  240  FRTP------SYCTQE------------IEQP---VQVYIQLK---RPSDG-------VTSD-PLP-FEM  276
_XP_035725333.1_  240  FRTP------SYCTQE------------IEQP---VQVYIQLK---RPSDG-------VTSD-PLP-FEM  276
_KAF7405659.1_    240  FRTP------SYCTQE------------IEQP---VQVYIQLK---RPSDG-------VTSD-PLP-FEM  276
_KAF7402294.1_    240  FRTP------SYCTQE------------IEQP---VQVYIQLK---RPSDG-------VTSD-PLP-FEM  276
_XP_015172825.1_  240  FKTP------SFRLLQ------------IEEP---VQIYIQLK---RPSDG-------VTSE-PHP-FEM  276
_XP_015172824.1_  240  FKTP------SFRLLQ------------IEEP---VQIYIQLK---RPSDG-------VTSE-PHP-FEM  276
_XP_015172823.1_  240  FKTP------SFRLLQ------------IEEP---VQIYIQLK---RPSDG-------VTSE-PHP-FEM  276
_XP_014609306.1_  240  FKTP------SYRILQ------------IEEP---VQIYIQLK---RPSDG-------VISE-PHP-FEM  276
_XP_043491219.1_  240  FKTP------SYRVLQ------------IEEP---VQIYIQLK---RPSDG-------VISE-PHP-FEM  276
_XP_014609305.1_  240  FKTP------SYRILQ------------IEEP---VQIYIQLK---RPSDG-------VISE-PHP-FEM  276
_XP_043491218.1_  240  FKTP------SYRVLQ------------IEEP---VQIYIQLK---RPSDG-------VISE-PHP-FEM  276
_KAI4498556.1_    240  FKTP------SYRILQ------------IEEQ---VQIYIQLK---RPSDG-------VTSE-PHP-FEM  276
_XP_015172818.1_  240  FKTP------SFRLLQ------------IEEP---VQIYIQLK---RPSDG-------VTSE-PHP-FEM  276
_XP_043491211.1_  240  FKTP------SYRVLQ------------IEEP---VQIYIQLK---RPSDG-------VISE-PHP-FEM  276
_KAI4495142.1_    240  FKTP------SYRILQ------------IEEP---VQIYIQLK---RPSDG-------VISE-PHP-FEM  276
_XP_014609300.1_  240  FKTP------SYRILQ------------IEEP---VQIYIQLK---RPSDG-------VISE-PHP-FEM  276
_ADK91833.1_      240  FKTP------TYRMQQ------------VEQP---VQI--------------------------------  256
_KAG7213837.1_    240  FRTP------SYRVQQ------------IEQP---VQVYIQLK---RPSDG-------ATSE-PLP-FQM  276
_XP_014226628.1_  239  FRTP------AYHDPM------------IDRT---VHAYVQLR---RPSDG-------MTSE-SIQ-FNF  275
_XP_020287946.1_  240  FRTP------GYRTQQ------------VEQP---VQVHIQLR---RPSDG-------ITSE-PLP-FQM  276
_XP_012232007.1_  240  FKTP------SYRPQQ------------VEDV---VQVYIQLQ---RPSDN-------ATSE-PLP-FQM  276
_XP_011874538.1_  240  FRTP------SYRTQQ------------VEQP---VEAYIQLK---RPSDG-------ATSE-PLP-FQM  276
_XP_011874541.1_  240  FRTP------SYRTQQ------------VEQP---VEAYIQLK---RPSDG-------ATSE-PLP-FQM  276
_XP_011173619.1_  240  FRTP------SYRSQQ------------VEQP---VQVYIQLR---RPSDG-------ATSEPPLP-FQM  277
_XP_034950793.1_  237  FRTP------RYPIVD------------VPNP---VQVYIQLR---RPLDG-------TTSE-PMP-FTL  273
_XP_034950792.1_  233  FRTP------RYPIVD------------VPNP---VQVYIQLR---RPLDG-------TTSE-PMP-FTL  269
_XP_012340880.2_  237  FKTS------SYHTQE------------IEEP---VPVHIQLK---RPSDG-------ALSE-PLP-FLM  273
_XP_031771859.1_  237  FKTS------SYHTQE------------IEEP---VPVHIQLK---RPSDG-------ALSE-PLP-FLM  273
_XP_006618953.1_  239  FKTS------SYHTQE------------IEEP---VPVHIQLK---RPSDG-------ALSE-PLP-FLM  275
_XP_031367904.1_  239  FKTS------SYHTQE------------IEEP---VPVHIQLK---RPSDG-------ALSE-PLP-FLM  275
_XP_043795948.1_  239  FKTS------SYHTQE------------IEEP---VPVHIQLK---RPSDG-------ALSE-PLP-FLM  275
_XP_395180.5_     239  FKTS------SYHTQE------------IEEP---VPVHIQLK---RPSDG-------ALSE-PLP-FLM  275
_KAG6799500.1_    239  FKTS------SYHTQE------------IEEP---VPVHIQLK---RPSDG-------ALSE-PLP-FLM  275
_XP_016768607.2_  239  FKTS------SYHTQE------------IEEP---VPVHIQLK---RPSDG-------ALSE-PLP-FLM  275
_PBC31989.1_      248  FKTS------SYHTQE------------IEEP---VSVQIQLK---RPSDG-------ALSE-PLP-FLM  284
```

```
_XP_016914450.1_   239   FKTS------SYHTQE------------IEEP---VSVQIQLK---RPSDG-------ALSE-PLP-FLM   275
_KAG5344958.1_     237   FRTP------SYRTQE------------VEQP---VQAFIQLR---RPSDG-------ATSE-PLP-FQM   273
_KAJ3630188.1_     239   FKPP------SYHTPE------------ITNP---VEVFIQLQ---RPSDG-------AHSK-ALP-FEF   275
_QHN75199.1_       239   FKPP------SYHTPE------------ITNP---VEVFIQLQ---RPSDG-------AHSK-ALP-FEF   275
_XP_051171660.1_   244   FRTP------KYDNMD------------ITEP---VKVKIQLK---LKKKD-------QGGP-PLD-FEM   280
_XP_016909447.1_   198   FRTP------TYRMQQ------------VEQP---VQVYIQLK---RPSDG-------ATSE-PFP-FLM   234
_XP_031773030.1_   198   FRTP------TYRMQQ------------VEQP---VQVYIQLK---RPSDG-------ATSE-PFP-FLM   234
_KAH0956427.1_     241   FKTP------KFYKQD------------LAKP---VQVYIELR---RPSDG-------ATSD-PLP-FQM   277
_XP_012283784.1_   240   FRTP------TYRMQQ------------VDHP---VQVYIHLR---RPSDG-------ATSE-PLP-FQL   276
_XP_023224928.1_   238   FITP------SYFDLN------------VSHE---VNVWIQLI---RPSDN-------SAGK-PRK-FQL   274
_XP_008556434.1_   240   FRTP------TYRIQQ------------VEHP---VQIYVQLR---RPSDN-------ATSE-ALP-FQF   276
_XP_008556433.1_   240   FRTP------TYRIQQ------------VEHP---VQIYVQLR---RPSDN-------ATSE-ALP-FQF   276
_XP_014299728.1_   240   FRTP------TYRIQQ------------VEHP---VQIYVQLR---RPSDN-------ATSE-ALP-FQF   276
_CAD6234449.1_     240   FRTP------TYRIQQ------------VEHP---VQVYIQLR---RPSDS-------ATSE-ALP-FQF   276
_KAG8037552.1_     240   FRTP------TYRIQQ------------VEHP---VQVYIQLR---RPSDS-------ATSE-ALP-FQF   276
_XP_044576138.1_   240   FRTP------TYRIQQ------------VEHP---VQVYIQLR---RPSDS-------ATSE-ALP-FQF   276
_XP_044576125.1_   240   FRTP------TYRIQQ------------VEHP---VQVYIQLR---RPSDS-------ATSE-ALP-FQF   276
_XP_026465448.1_   238   FRTP------KYRTLE------------VDRP---VNAQIQLR---RPSDS-------ATST-PLK-FQL   274
_XP_026465446.1_   238   FRTP------KYRTLE------------VDRP---VNAQIQLR---RPSDS-------ATST-PLK-FQL   274
_XP_031785072.1_   240   FRTP------SYMTHQ------------VDQP---VQAFIQLR---RPSDG-------AVSE-ALP-FQL   276
_XP_015607185.1_   240   FRTP------SYRMQQ------------VDQP---VQIFIQLK---RPSDG-------STSD-PLP-FQL   276
_XP_011496611.1_   240   FRTP------SYKSQQ------------VEHP---VQAYIQLR---RPSDG-------ATSE-PLH-FQL   276
_XP_018903707.1_   237   FRLP------TYKSLE------------VENP---VQAYLELR---RPSDQ-------ASSV-PRP-FQL   273
_CAB3365562.1_     239   FRTP------AYHNLQ------------VQHE---VKLKIQLV---RPSDG-------ANSE-KRD-FTY   275
_XP_011496447.1_   107   FETP------EYRTATCS-------PELVHEP---LNVRIELR---RISDK-------ETSQ-TVP-FQL   148
_KAG8230996.1_     239   FRTP------RYKKLE------------VDAP---VKVCIQLR---RPSDG-------ATSE-PLP-FEY   275
_XP_040567422.1_   237   FLTP------PYFDKD------------IKQP---IKVSMYLY---KPCEN-------VASE-HVE-FQY   273
_XP_014250205.1_   240   FRTP------RYRTLE------------IDQP---VQALIQLR---RPSDN-------ALSD-PLP-FLI   276
_XP_014250206.1_   240   FRTP------RYRTLE------------IDQP---VQALIQLR---RPSDN-------ALSD-PLP-FLI   276
_XP_047119528.1_   240   LRTP------RYRTLD------------VDRP---VRAHIQLR---RPSDG-------ATSE-PLP-FEL   276
_XP_047001845.1_   240   LRTP------RYRTLD------------VDRP---VRAHIQLR---RPSDG-------ATSE-PLP-FEL   276
_XP_049939433.1_   240   LRTP------RYRTLD------------VDRP---VRAHIQLR---RPSDG-------ATSE-PLP-FEL   276
_XP_049764197.1_   240   LRTP------RYRTLD------------VDRP---VRAHIQLR---RPSDG-------ATSE-PLP-FEL   276
_XP_049833656.1_   240   LRTP------RYRTLD------------VDRP---VRAHIQLR---RPSDG-------ATSE-PLP-FEL   276
_XP_049790026.1_   240   LRTP------RYRTLD------------VDRP---VRAHIQLR---RPSDG-------ATSE-PLP-FEL   276
_XP_049833651.1_   240   LRTP------RYRTLD------------VDRP---VRAHIQLR---RPSDG-------ATSE-PLP-FEL   276
_XP_047001842.1_   240   LRTP------RYRTLD------------VDRP---VRAHIQLR---RPSDG-------ATSE-PLP-FEL   276
_XP_049790025.1_   240   LRTP------RYRTLD------------VDRP---VRAHIQLR---RPSDG-------ATSE-PLP-FEL   276
_XP_049833654.1_   240   LRTP------RYRTLD------------VDRP---VRAHIQLR---RPSDG-------ATSE-PLP-FEL   276
```

```
_XP_049833659.1_  240  LRTP------RYRTLD------------VDRP---VRAHIQLR---RPSDG-------ATSE-PLP-FEL  276
_XP_021925503.1_  240  FRTP------RYKSLE------------VEAP---IKVNIQLR---RPSDG-------ATSE-PLP-FEY  276
_KDR23857.1_      240  FRTP------RYKSLE------------VEAP---IKVNIQLR---RPSDG-------ATSE-PLP-FEY  276
_ABU96700.1_      240  FRTP------RYKTLE------------IEQP---VQVLIQLR---RPSDN-------ATSE-ALP-FQI  276
_ABU96699.1_      240  FRTP------RYKTLE------------IEQP---VQVLIQLR---RPSDN-------ATSE-ALP-FQI  276
_XP_014275495.1_  240  FRTP------RYKSLE------------IDQP---VQAFIQLR---RPSDG-------ATSE-ALP-FQI  276
_BBE08137.1_      240  FRTP------RYKSLE------------IDHP---VQAFIQLR---RPSDG-------ATSE-ALP-FQI  276
_XP_014275494.1_  240  FRTP------RYKSLE------------IDQP---VQAFIQLR---RPSDG-------ATSE-ALP-FQI  276
_XP_014275493.1_  240  FRTP------RYKSLE------------IDQP---VQAFIQLR---RPSDG-------ATSE-ALP-FQI  276
_BBE08136.1_      240  FRTP------RYKSLE------------IDHP---VQAFIQLR---RPSDG-------ATSE-ALP-FQI  276
_KAF6207519.1_    240  FRTP------RYRTLE------------IDQP---VQALIQLR---RPSDN-------ATSE-ALP-FQI  276
_CAB0015508.1_    240  FRTP------RYRTLE------------IEQP---VQALIQLR---RPSDN-------ATSD-ALP-FQI  276
_CAB0015504.1_    240  FRTP------RYRTLE------------IEQP---VQALIQLR---RPSDN-------ATSD-ALP-FQI  276
_PNF18474.1_      240  FRTP------RYKLLE------------VEAP---VKVNIQLR---RPSDG-------ATSE-PLP-FEY  276
_CEG62431.1_      240  FRTP------SYKTLH------------VDSP---VTVNIQLR---RPSDG-------ATSE-PLP-FQY  276
_PSN36690.1_      240  FRTP------SYKTLH------------VDSP---VTVNIQLR---RPSDG-------ATSE-PLP-FQY  276
_XP_046387412.1_  240  FRTP------RYKKLE------------VESS---VKVCIQLR---RPSDG-------ATSE-PLP-FEF  276
_XP_046387414.1_  240  FRTP------RYKKLE------------VESS---VKVCIQLR---RPSDG-------ATSE-PLP-FEF  276
_XP_031634874.1_  240  FRTP------QYKTID------------ISQP---VKCFIQLR---RPSDS-------ATSD-SLP-FEY  276
_GFG29240.1_      240  FRTP------RYKSLE------------VEAP---VKVHIQLR---RPSDG-------ATSE-PLP-FEY  276
_BBG28464.1_      240  FRTP------RYRVLN------------VDRP---VQAFIQLR---RPSDG-------ATSE-ALP-FTF  276
_WCL52334.1_      240  FRTP------RYRVLN------------VDRP---VQAFIQLR---RPSDG-------ATSE-ALP-FTF  276
_GLH00135.1_      240  FRTP------RYRVLN------------VDRP---VQAFIQLR---RPSDG-------ATSE-ALP-FTF  276
_CAD7205239.1_    241  FRTP------HYRTIE------------VDAP---VKAFVQLR---RPSDG-------ATSE-PLP-FEF  277
_CAD7431383.1_    240  FRTP------HYRTIE------------VDAP---VKAFVQLR---RPSDG-------ATSE-PLP-FEF  276
_CAD7410836.1_    240  FRTP------HYRTIE------------VDAP---VKAFVQLR---RPSDG-------ATSE-PLP-FEF  276
_CAD7415345.1_    240  FRTP------HYRTIE------------VDAP---VKAFVQLR---RPSDG-------ATSE-PLP-FEF  276
_CAD7606635.1_    240  FRTP------HYRTIE------------VDAP---VKAFVQLR---RPSDG-------ATSE-PLP-FEF  276
_CAD7462957.1_    240  FRTP------HYRTIE------------VDAP---VKAFVQLR---RPSDG-------ATSE-PLP-FEF  276
_XP_012265400.2_  239  FRTP------RYRVLE------------VDQP---VKVFIQLR---RPSDG-------ATSD-PLP-FEF  275
_XP_046473587.1_  240  FRTP------RYRTLE------------VDHP---VKVFIQLL---RPTDG-------ATSE-PLA-FEF  276
_XP_046611732.1_  240  FRTP------RYRTLE------------VDHP---VKVFIQLL---RPTDG-------ATSE-PLA-FEF  276
_XP_015519750.2_  240  FRTP------RYRTLE------------VDHP---VKVFIQLL---RPTDG-------ATSE-PLA-FEF  276
_XP_046611731.1_  240  FRTP------RYRTLE------------VDHP---VKVFIQLL---RPTDG-------ATSE-PLA-FEF  276
_XP_046417903.1_  240  FRTP------RYRTLE------------VDHP---VKVFIQLL---RPTDG-------ATSE-PLA-FEF  276
_GLG94364.1_      240  LRTP------RYRTLD------------VPQP---VDACIQLR---RPSDG-------ASSE-PLP-FQL  276
_GLG94363.1_      240  LRTP------RYRTLD------------VPQP---VDACIQLR---RPSDG-------ASSE-PLP-FQL  276
_BBG28463.1_      240  LRTP------RYRTLD------------VPQP---VDACIQLR---RPSDG-------ASSE-PLP-FQL  276
_WCL52335.1_      240  LRTP------RYRTLD------------VPQP---VDACIQLR---RPSDG-------ASSE-PLP-FQL  276
```

```
_GLG94365.1_       240  LRTP------RYRTLD------------VPQP---VDACIQLR---RPSDG-------ASSE-PLP-FQL  276
_XP_002431518.1_   238  FRTP------KYKNID------------VEKP---VKVWVQLR---RPSDG-------AVSE-SVP-FQF  274
_KAE8746525.1_     240  FRTP------RYERLE------------VDGP---VNVFIQLK---RPSDG-------ATSD-PLP-FQM  276
_XP_026277770.1_   240  FRTP------RYERLE------------VDGP---VNVFIQLK---RPSDG-------ATSD-PLP-FQM  276
_KAJ1519446.1_     240  FRTP------RYERLE------------VDGP---VSVYIQLR---RPSDN-------ATSE-PQP-FQM  276
_ALC78349.1_       239  FRTP------RYRTLE------------VDQI---VKGFIQLK---RPSDG-------ATSD-PLP-FDY  275
_XP_046738772.1_   240  FRTP------RYRTLE------------VDHP---VKVFIQLL---RPTDG-------ATSE-PLP-FQF  276
_XP_046417904.1_   238  FRTP------RYRTLE------------VDHP---VKVFIQLL---RPTDG-------ATSE-PLA-FEF  274
_CRK94170.1_       238  FRTP------RFKAID------------ISEP---VRVKVQLR---RPSDG-------ATSE-PLD-FEM  274
_CRK94167.1_       238  FRTP------RFKAID------------ISEP---VRVKVQLR---RPSDG-------ATSE-PLD-FEM  274
_KAG5676160.1_     242  FRTP------RYKSIN------------LTDP---VKVKVQLR---RPSDG-------ATSD-PLD-FEM  278
_XP_034245464.1_   241  FETP------RYERIE------------FSEP---VTVYIQLK---RPSDG-------ATSE-PHI-FEM  277
_XP_034245457.1_   241  FETP------RYERIE------------FSEP---VTVYIQLK---RPSDG-------ATSE-PHI-FEM  277
_KAG8287980.1_     206  FKTP------RYRTLQ------------VEDP---VQVFIQLR---RPSDG-------ETSD-SLP-FQL  242
_XP_044759045.1_   235  FRAP------KFYNLE------------IENP---VRVLVQLR---RPSDN-------AVSE-SIP-FEY  271
_XP_017305229.1_   232  LKTP------AYKSFE------------INDP---VHVKIQLV---TKKD---------ISE-PYN-FML  266
_XP_026675878.1_   232  LKTP------AYKSFE------------INDP---VHVKIQLV---TKKD---------ISE-PYN-FML  266
_KAE8574083.1_     192  FRTP------RYKSLE------------IDQP---VQAFIQLR---RPSDG-------ATSE-ALP-FQI  228
_XP_037926497.1_   240  FRTP------RYRTLD------------IEQP---VKVFIQLK---RPSDG-------ATSE-PIP-FDY  276
_CAD7079567.1_     240  FRTP------RYRTLD------------IEQP---VKVFIQLK---RPSDG-------ATSE-PIP-FDY  276
_XP_037882485.1_   240  FKTP------RYHKLD------------ITEP---AKVFIQLK---RPSDG-------VTSE-ALP-FQY  276
_XP_037037597.1_   240  FRTP------RYKSID------------IDQP---VRCYVQLK---RPSDG-------VTSE-PLP-FDY  276
_XP_037037592.1_   240  FRTP------RYKSID------------IDQP---VRCYVQLK---RPSDG-------VTSE-PLP-FDY  276
_KAG4075389.1_     240  FRTP------RYKSID------------IDQP---IRCYVQLK---RPSDG-------VTSE-PLP-FDY  276
_XP_034101357.1_   240  FKTP------RYRSLE------------ITEP---AKVFIQLR---RPSDG-------VTSE-PLP-FEY  276
_XP_026844963.1_   240  FKTP------RYHTLD------------ITEP---AKVFIQLR---RPSDG-------VTSE-SLP-FEY  276
_EDW28798.1_       240  FKTP------RYHTLD------------ITEP---AKVFIQLR---RPSDG-------VTSE-SLP-FEY  276
_XP_034125966.1_   240  FKTP------RYHTLD------------ITEP---AKVFIQLR---RPSDG-------VTSE-SLP-FEY  276
_XP_034666519.1_   240  FKTP------RYHTLD------------ITEP---AKVFIQLR---RPSDG-------VTSE-SLP-FEY  276
_XP_001356570.3_   240  FKTP------RYHTLD------------ITEP---AKVFIQLR---RPSDG-------VTSE-SLP-FEY  276
_XP_023164577.2_   240  FKTP------RYHTLE------------ITEP---AKVFIQLR---RPSDG-------VTSE-ALP-FEY  276
_XP_017153206.1_   240  FKTP------RYHTLD------------ITEP---AKVFIQLR---RPSDG-------VTSE-SLP-FEY  276
_XP_030564932.1_   240  FKTP------RYHTLE------------ITEP---AKVFIQLR---RPSDG-------VTSE-ALP-FEY  276
_XP_002059145.1_   240  FKTP------RYHTLE------------ITEP---AKVFIQLR---RPSDG-------VTSE-ALP-FEY  276
_EDW04266.1_       240  FKTP------RYHTLE------------ITEP---AKVFIQLR---RPSDG-------VTSE-ALP-FEY  276
_XP_017025862.1_   240  FKTP------RYHTLD------------ITEP---AKVFIQLR---RPSDG-------VTSE-ALP-FEY  276
_XP_017859907.1_   240  FKTP------RYHTLE------------ITEP---AKVFIQLR---RPSDG-------VTSE-ALP-FEY  276
_XP_032584327.1_   240  FKTP------RYHTLE------------ITEP---AKVFIQLR---RPSDG-------VTSE-ALP-FEY  276
_XP_016987288.2_   240  FKTP------RYHSPE------------ITEP---AKVFIQLR---RPSDG-------VTSE-ALP-FEY  276
```

```
_XP_017954173.1_  240  FKTP------RYHTLD------------ITEP---AKVFIQLR---RPSDG-------VTSE-ALP-FEY  276
_XP_017854504.1_  240  FKTP------RYHTLD------------ITEP---AKVFIQLR---RPSDG-------VTSE-ALP-FEY  276
_XP_034125965.1_  240  FKTP------RYHTLD------------ITEP---AKVFIQLR---RPSDG-------VTSE-SLP-FEY  276
_XP_034666515.1_  240  FKTP------RYHTLD------------ITEP---AKVFIQLR---RPSDG-------VTSE-SLP-FEY  276
_XP_043641917.1_  240  FKTP------RYHTLE------------ITEP---AKVFIQLR---RPSDG-------VTSE-ALP-FEY  276
_XP_026844960.1_  240  FKTP------RYHTLD------------ITEP---AKVFIQLR---RPSDG-------VTSE-SLP-FEY  276
_XP_015035988.2_  240  FKTP------RYHTLD------------ITEP---AKVFIQLR---RPSDG-------VTSE-SLP-FEY  276
_XP_041450744.1_  240  FKTP------RYHTLD------------ITEP---AKVFIQLR---RPSDG-------VTSE-SLP-FEY  276
_XP_015052361.1_  240  FKTP------RYHTLE------------ITEP---AKVFIQLR---RPSDG-------VTSE-ALP-FEY  276
_XP_039480515.1_  240  FKTP------RYHTLE------------ITEP---AKVFIQLR---RPSDG-------VTSE-ALP-FEY  276
_XP_033248563.1_  240  FKTP------RYHTLD------------ITEP---AKVFIQLR---RPSDG-------VTSE-SLP-FEY  276
_KAH8412150.1_    240  FKTP------RYQTLD------------ITEP---AKVFIQLR---RPSDG-------VTSE-ALP-FEY  276
_XP_043864974.1_  240  FKTP------RYHTLE------------ITEP---AKVFIQLR---RPSDG-------VTSE-ALP-FEY  276
_XP_020809119.1_  240  FKTP------RYQTLD------------ITEP---AKVFIQLR---RPSDG-------VTSE-ALP-FEY  276
_KAH8298353.1_    240  FKTP------RYHTLD------------ITEP---AKVFIQLR---RPSDG-------VTSE-ALP-FEY  276
_XP_030564927.1_  240  FKTP------RYHTLE------------ITEP---AKVFIQLR---RPSDG-------VTSE-ALP-FEY  276
_XP_041631592.1_  240  FKTP------RYHTLD------------ITEP---AKVFIQLR---RPSDG-------VTSE-ALP-FEY  276
_XP_017859909.1_  240  FKTP------RYHTLE------------ITEP---AKVFIQLR---RPSDG-------VTSE-ALP-FEY  276
_XP_032591602.1_  240  FKTP------RYHTLE------------ITEP---AKVFIQLR---RPSDG-------VTSE-ALP-FEY  276
_XP_023164572.2_  240  FKTP------RYHTLE------------ITEP---AKVFIQLR---RPSDG-------VTSE-ALP-FEY  276
_TDG46699.1_      240  FKTP------RYHTLD------------ITEP---AKVFIQLR---RPSDG-------VTSE-ALP-FEY  276
_KAH8411895.1_    240  FKTP------RYHSID------------ITEP---AKVFIQLR---RPSDG-------VTSE-PLP-FEY  276
_KAH8377496.1_    240  FKTP------RYRSLE------------ITEP---AKVFIQLR---RPSDG-------VTSE-PLP-FEY  276
_XP_017854500.2_  240  FKTP------RYHTLD------------ITEP---AKVFIQLR---RPSDG-------VTSE-ALP-FEY  276
_XP_016987284.2_  240  FKTP------RYHSPE------------ITEP---AKVFIQLR---RPSDG-------VTSE-ALP-FEY  276
_KAH8293209.1_    240  FKTP------RYHSLE------------ITEP---AKVFIQLR---RPSDG-------VTSE-PLP-FEY  276
_EEC00225.1_      239  FRTP------RYREPP------------AEHP---VDVHLLLR---RPSDG-------ALSE-PRA-FTL  275
_XP_029825505.2_  239  FRTP------RYREPP------------AEHP---VDVHLLLR---RPSDG-------ALSE-PRA-FTL  275
_KAG0424790.1_    239  FRTP------RYREPP------------AEHP---VDVHLLLR---RPSDG-------ALSE-PRA-FTL  275
_XP_037272127.1_  241  FRTP------RYRDPV-----------MADVP---VDVHLLLR---RPSDG-------ALSE-PRA-FTL  278
_XP_054934017.1_  239  FRTP------RYRDPI-----------MADVP---VDVHLLLR---RPSDG-------ALSE-ARA-FTL  276
_XP_050048524.1_  239  FRTP------RYRDPI-----------MADVP---VDVHLLLR---RPSDG-------ALSE-ARA-FTL  276
_XP_037272126.1_  239  FRTP------RYRDPV-----------MADVP---VDVHLLLR---RPSDG-------ALSE-PRA-FTL  276
_XP_037524244.1_  239  FRTP------RYRDPI-----------MADVP---VDVHLLLR---RPSDG-------ALSE-PRA-FTL  276
_XP_037524245.1_  239  FRTP------RYRDPI-----------MADVP---VDVHLLLR---RPSDG-------ALSE-PRA-FTL  276
_XP_049512094.1_  239  FRTP------RYRDPI-----------MADVP---VDVHLLLR---RPSDG-------ALSE-PRA-FTL  276
_XP_037524243.1_  239  FRTP------RYRDPI-----------MADVP---VDVHLLLR---RPSDG-------ALSE-PRA-FTL  276
_XP_037580160.1_  239  FRTP------RYRDPI-----------MADVP---VDVHLLLR---RPSDG-------ALSE-PRA-FTL  276
_XP_037272128.1_  241  FRTP------RYRDPV-----------MADVP---VDVHLLLR---RPSDG-------ALSE-PRA-FTL  278
```

```
_KAH9380259.1_     239  FRTP------RYREPL-----------AADLP---VDVHLQLR---RPSDG-------ALSE-PRA-FTL  276
_KAI1291715.1_     212  LRTP------PYHDSA------------IKQP---VQILLQLR---RPSDM-------HTSN-SVK-FKF  248
_XP_035209023.1_   232  CRVP------RYHDIN------------IRSY---RDIFIQFR-----DGN-------RIGT-PRC-FRY  266
_XP_023332679.1_   241  FKTP------KYRDLN------------IKEK---VRVQVELC----KVNG-------ETSE-PQD-FYY  276
_XP_046801942.1_   240  FKTP------RYRKIE------------ITEP---AKVFIQLK---RPSDG-------VTSE-PLP-FEY  276
_XP_037811120.1_   240  FKTP------RYRKID------------ITEP---AKVFIQLK---RPSDG-------VTSE-PLP-FEY  276
_AFI98401.1_       240  FKTP------RYRKID------------ITEP---AKVFIQLK---RPSDG-------ATSE-PLP-FEY  276
_AFJ54621.1_       240  FKTP------RYRKID------------ITEP---AKVFIQLK---RPSDG-------ATSE-PLP-FEY  276
_KAI8126534.1_     240  FKTP------RYRKIE------------ITEP---AKVFIQLK---RPSDG-------VTSE-PLP-FEY  276
_XP_013118266.1_   240  FKTP------RYHTMD------------ITEP---AKVFIQLK---RPSDG-------VTSE-ALP-FEY  276
_XP_023293634.2_   240  FKTP------RYRKIE------------ITEP---AKVFIQLK---RPSDG-------VTSE-PLP-FEY  276
_XP_037811062.1_   240  FKTP------RYRKID------------ITEP---AKVFIQLK---RPSDG-------VTSE-PLP-FEY  276
_KNC34132.1_       240  FKTP------RYRKIE------------ITEP---AKVFIQLK---RPSDG-------VTSE-PLP-FEY  276
_TMW48951.1_       240  FKTP------RYRKID------------ITEP---AKVFIQLK---RPSDG-------VTSE-PLP-FEY  276
_XP_037957363.1_   240  FKTP------RYHTLD------------ITEP---AKVNIQLK---RPSDG-------VTSD-PLP-FEY  276
_XP_005175014.1_   240  FKTP------RYRTID------------ITEP---AKVFIQLK---RPSDG-------VTSE-ALP-FEY  276
_XP_037957360.1_   240  FKTP------RYHTLD------------ITEP---AKVNIQLK---RPSDG-------VTSD-PLP-FEY  276
_XP_030383330.1_   239  FKTP------RYHSLE------------ITEP---AKVFIQLR---RPSDG-------ATSE-ALP-FEY  275
_XP_030383328.1_   239  FKTP------RYHSLE------------ITEP---AKVFIQLR---RPSDG-------ATSE-ALP-FEY  275
_CAH1707987.1_     240  FRTP------RYKRID------------IAEP---VKVKIQLR---RPSDG-------ATSD-PLD-FEM  276
_TMW54796.1_       240  FRTP------RYHKID------------ITEP---TKVFIQLK---RPSDG-------VTSK-PFP-FKY  276
_XP_026836077.1_   240  FKTP------RYHTLD------------ITEP---AKVFIQLR---RPSDG-------VTSE-ALP-FEY  276
_XP_017116803.1_   240  FKTP------RYHTLD------------ITEP---AKVFIQLR---RPSDG-------VTSE-ALP-FEY  276
_XP_039480519.1_   240  FKTP------RYHTLE------------ITEP---AKVFIQLR---RPSDG-------VTSE-ALP-FEY  276
_XP_043641920.1_   240  FKTP------RYHTLE------------ITEP---AKVFIQLR---RPSDG-------VTSE-ALP-FEY  276
_XP_023036748.1_   240  FKTP------RYHSIE------------ITEP---AKVFIQLR---RPSDG-------VTSE-ALP-FEY  276
_XP_002090466.1_   240  FKTP------RYHTLE------------ITEP---AKVFIQLR---RPSDG-------VTSE-ALP-FEY  276
_XP_034471824.1_   240  FKTP------RYHSLE------------ITEP---AKVFIQLR---RPSDG-------VTSE-PLP-FEY  276
_AAT94434.1_       240  FKTP------RYHTLD------------ITEP---AKVFIQLR---RPSDG-------VTSE-ALP-FEY  276
_XP_036670985.1_   240  FKTP------RYHTLD------------ITEP---AKVFIQLR---RPSDG-------VTSE-SLP-FEY  276
_AAA28479.1_       240  FKTP------RYHTLD------------ITEP---AKVFIQLR---RPSDG-------VTSE-ALP-FEY  276
_XP_037708206.1_   240  FKTP------RYHTLD------------ITEP---AKVFIQLR---RPSDG-------VTSE-ALP-FEY  276
_XP_002038915.1_   240  FKTP------RYHTLD------------ITEP---AKVFIQLR---RPSDG-------VTSE-ALP-FEY  276
_XP_016993636.1_   240  FKTP------RYHTLD------------ITEP---AKVFIQLR---RPSDG-------VTSE-ALP-FEY  276
_XP_052852517.1_   240  FKTP------RYHTLD------------ITEP---AKVFIQLR---RPSDG-------VTSE-ALP-FEY  276
_XP_016963139.1_   240  FKTP------RYHTLD------------ITEP---AKVFIQLR---RPSDG-------VTSE-ALP-FEY  276
_XP_033156151.1_   240  FKTP------RYHTLD------------ITEP---AKVFIQLR---RPSDG-------VTSE-ALP-FEY  276
_XP_001974446.1_   240  FKTP------RYHTLD------------ITEP---AKVFIQLR---RPSDG-------VTSE-ALP-FEY  276
_XP_014761886.1_   240  FKTP------RYHTLD------------ITEP---AKVFIQLR---RPSDG-------VTSE-ALA-FEY  276
```

_XP_017107216.2_	240	FKTP------RYHTLD------------ITEP---AKVFIQLK---RPSDG-------VLSE-ALA-FEY	276
_XP_017044881.1_	239	FKTP------RYHTLD------------ITEP---AKVFVQLR---RPSDG-------VTSE-ALP-FEY	275
_XP_016944798.1_	240	FKTP------RYHTLD------------ITEP---AKVFIQLR---RPSDG-------VTSE-SLP-FEY	276
_XP_017116800.1_	240	FKTP------RYHTLD------------ITEP---AKVFIQLR---RPSDG-------VTSE-ALP-FEY	276
_XP_016993633.2_	240	FKTP------RYHTLD------------ITEP---AKVFIQLR---RPSDG-------VTSE-ALP-FEY	276
_XP_037708198.1_	240	FKTP------RYHTLD------------ITEP---AKVFIQLR---RPSDG-------VTSE-ALP-FEY	276
_XP_032583259.1_	240	FKTP------RYHTLD------------ITEP---AKVFIQLR---RPSDG-------VTSE-ALP-FEY	276
AAC35296.1	240	FKTP------RYHTLD------------ITEP---AKVFIQLR---RPSDG-------VTSE-ALP-FEY	276
_NP_001286014.1_	240	FKTP------RYHTLD------------ITEP---AKVFIQLR---RPSDG-------VTSE-ALP-FEY	276
_XP_016963136.1_	240	FKTP------RYHTLD------------ITEP---AKVFIQLR---RPSDG-------VTSE-ALP-FEY	276
_XP_017063904.2_	240	FKTP------RYHTLE------------ITEP---AKVFIQLR---RPSDG-------VTSE-ALP-FEY	276
_XP_033156135.1_	240	FKTP------RYHTLD------------ITEP---AKVFIQLR---RPSDG-------VTSE-ALP-FEY	276
_XP_052852512.1_	240	FKTP------RYHTLD------------ITEP---AKVFIQLR---RPSDG-------VTSE-ALP-FEY	276
_XP_032307758.1_	240	FKTP------RYHTLD------------ITEP---AKVFIQLR---RPSDG-------VTSE-ALA-FEY	276
_XP_017107215.2_	240	FKTP------RYHTLD------------ITEP---AKVFIQLK---RPSDG-------VLSE-ALA-FEY	276
AWT86616.1	239	FRTP------RYRNID------------VTDP---ILVQIQLV---RPSDK-------AASE-PLP-FQL	275
QUF98534.1	239	FRTP------RYKNID------------VTEP---ILVQIQLV---RPSDK-------AASE-PLP-FQL	275
UNA06287.1	239	FRTP------RYRNID------------VTDP---ILVQIQLV---RPSDK-------AASE-PLP-FQL	275
AAQ65068.1	240	FKTP------RYHTLE------------ITEP---A----------------------------------	254
AAQ64916.1	240	FKTP------RYHTLE------------ITEP---A----------------------------------	254
AAQ64921.1	240	FKTP------RYHTLD------------ITXP---A----------------------------------	254
AAQ64918.1	240	FKTP------RYHTLD------------ITEP---A----------------------------------	254
KAH8245459.1	265	FKTP------RYQTLD------------ITEP---AKVFIQLR---RPSDG-------VTSE-ALP-FEY	301
KAH8283155.1	262	FKTP------RYQTLD------------ITEP---AKVFIQLR---RPSDG-------VTSE-ALP-FEY	298
KAH8246384.1	262	FKTP------RYQTLD------------ITEP---AKVFIQLR---RPSDG-------VTSE-ALP-FEY	298
KAH8366418.1	262	FKTP------RYYSLD------------ITEP---AKVFIQLR---RPSDG-------VTSE-ALP-FEY	298
_XP_022195378.2_	239	FKTP------RYKNIE------------VTEP---IVAQIQLV---RPSDK-------AASD-PLP-FQL	275
_XP_039284931.1_	239	FKTP------RYKNIE------------VTEP---IVAQIQLV---RPSDK-------AASD-PLP-FQL	275
ALC40091.1	262	FKTP------RYHTLD------------ITEP---AKVFIQLR---RPSDG-------VTSE-ALP-FEY	298
_XP_036342419.1_	238	LKTP------RYRNVD------------ITEP---VKLFVQLK---RPSDG-------AVSE-PWV-FKY	274
_XP_022903025.1_	239	FRPP------RYRTLE------------VIDP---VKMFVQLR---RPSDG-------ATSA-PVP-FQM	275
_XP_022903017.1_	239	FRPP------RYRTLE------------VIDP---VKMFVQLR---RPSDG-------ATSA-PVP-FQM	275
_XP_036218946.1_	238	LRTP------KYHNTD------------IEEP---VQIFVQLQ---RPSDG-------AVSK-PCE-FKY	274
_XP_018790401.1_	238	LRTP------QYHKTD------------IEQP---VMVFVQLQ---RPSDG-------AVSE-PCE-FKY	274
KAI9579887.1	226	FRTP------KYHNLN------------ITKP---ATVTIQLE---RISDG-------ALSE-SYA-FDY	262
_XP_037892537.1_	225	FRTP------KYHNLN------------ITKP---ATVTIQLE---RISDG-------ALSE-SYA-FDY	261
_XP_018903705.1_	244	FRLP------TYKSLE------------VENP---VQAYLELR---RPSDQ-------ASSV-PRP-FQL	280
CAH0395667.1	244	FRLP------TYKSLE------------VENP---VQAYLELR---RPSDQ-------ASSV-PRP-FQL	280
_XP_050330165.1_	238	LRTP------QYHNTD------------IEEP---VQVFVQLQ---RPSDG-------AVSE-PCE-FNY	274

```
_XP_039970878.1_  238  LRTP------QYHNTD------------IEEP---VQVFVQLQ---RPSDG-------AVSE-PCE-FNY  274
_XP_049305234.1_  239  LRTP------QYHNTD------------IEET---VQVFVQLQ---RPSDG-------AVSE-PWQ-FNY  275
_GLH04793.1_      247  LRTP------RYCRLD------------VSVP---VSAFVQLR---RPSDG-------ACSV-PLP-FEL  283
_XP_015835035.1_  239  FKPP------PYKNLN------------IVEP---VPVFIQLV---RPSDE-------VVSK-SVQ-FEY  275
_XP_015835036.1_  239  FKPP------PYKNLN------------IVEP---VPVFIQLV---RPSDE-------VVSK-SVQ-FEY  275
_XP_039283832.1_  250  FKTP------PYKDLN------------VTGP---VQVKIQLV---RPSDN-------EVGP-ERE-FQL  286
_XP_031788427.1_  235  FKSP------KYPDTE------------ISEP---VIVMVQLR---RISDG-------EMSD-PLS-FRI  271
_KAJ6646894.1_    241  FKTP------PYVTND------------ITEP---VTVMMQLV---RTTDG-------HQSE-PRL-FYY  277
_XP_026466084.1_  175  FRTP------SYKNQD------------IENQ---FKAFIQLR---RPSDG-------AVSE-PLA-FQF  211
_XP_044002069.1_  240  FKTP------AYKNQT------------IYET---VEVFVELI---RPSNG-------DVSN-TCS-FKY  276
_XP_046611735.1_  235  FTAP------PYHNFE------------VNDS---VPVFVELF---RPSNG-------DISN-AFP-FVF  271
_XP_046417906.1_  235  FTAP------PYHNFE------------VNDS---VPVFVELF---RPSNG-------DISN-AFP-FVF  271
_XP_046590309.1_  235  FTAP------PYHNFD------------VNDS---VPVFVELF---RPSNG-------DISN-AFP-FVF  271
_XP_046473589.1_  235  FTAP------PYHNFD------------VNDS---VPVFVELF---RPSNG-------DISN-AFP-FVF  271
_XP_019892116.1_  151  FKTP------RYRTID------------ITEP---AKVFIQLK---RPSDG-------VTSE-ALP-FEY  187
_XP_005183027.3_  151  FKTP------RYRTID------------ITEP---AKVFIQLK---RPSDG-------VTSE-ALP-FEY  187
_XP_011496598.1_  164  FKSP------KYIEQD------------IGEP---ISVQIQLK---RFSDG-------ETSK-PVP-FQF  200
_AAW67216.1_      139  FRTP------RYRTXE------------VEHP---VMVNIQLK---RPSDG-------ATSE-PLP-FEL  175
_AAW67217.1_      139  FRTP------RYRTXE------------VEHP---VMVNIQLK---RPSDG-------ATSE-PLP-FEL  175
_XP_017489788.1_  142  LKTP------RYRNVD------------ITEP---VKLFVQLK---RPSDG-------AVSE-PWV-FKY  178
_BAI67894.1_           ----------------------------------------------------------------------
_XP_054741511.1_  239  FRTP------KYHNTN------------ITKP---IEVFVQLQ---RPSDG-------AVSD-PEK-FQY  275
_XP_053961684.1_  239  FRTP------KYHNIN------------ITKP---IEVFVQLQ---RPSDG-------AVSD-PEK-FQY  275
_XP_036341313.1_  239  FRTP------KFHRTN------------ITEP---VEVFVQLQ---RPSDG-------AVSD-RVK-FEY  275
_XP_036341343.1_  239  FRTP------KFHRTN------------ITEP---VEVFVQLQ---RPSDG-------AVSD-RVK-FEY  275
_XP_017471383.1_  239  FRTP------KFHRTN------------ITEP---VEVFVQLQ---RPSDG-------AVSD-PVK-FEY  275
_XP_054741498.1_  239  FRTP------KYHNTN------------ITKP---IEVFVQLQ---RPSDG-------AVSD-PEK-FQY  275
_XP_053961677.1_  238  FRTP------KYHNIN------------ITKP---IEVFVQLQ---RPSDG-------AVSD-PEK-FQY  274
_XP_050320008.1_  239  FRTP------KYHNTS------------ITMP---VEVLVQLQ---RPSDG-------AVSD-PAT-FEY  275
_XP_028894490.2_  239  FRTP------KYHKTS------------ITEP---VEVLVQLQ---RPSDG-------AVSD-PVK-FEY  275
_XP_050320015.1_  239  FRTP------KYHNTS------------ITMP---VEVLVQLQ---RPSDG-------AVSD-PAT-FEY  275
_XP_018787554.1_  239  FRTP------KYHNTS------------ITMP---VEVLVQLQ---RPSDG-------AVSD-PAT-FEY  275
_XP_029407438.2_  239  FRTP------KYHNTS------------ITMP---VEVLVQLQ---RPSDG-------AVSD-PAR-FEY  275
_XP_036225551.1_  239  FRTP------KYHNTS------------ITTP---VEVLVQLQ---RPSDG-------AVSD-PAT-FEY  275
_XP_011178374.2_  239  FRTP------KYHKTS------------ITEP---VEVLVQLQ---RPSDG-------AVSD-PVK-FEY  275
_XP_039952593.1_  239  FRTP------KYHNTS------------ITMP---VEVLVQLQ---RPSDG-------AVSD-PAT-FEY  275
_XP_018787542.1_  239  FRTP------KYHNTS------------ITMP---VEVLVQLQ---RPSDG-------AVSD-PAT-FEY  275
_XP_011207866.2_  239  FRTP------KYHNTS------------ITMP---VEVLVQLQ---RPSDG-------AVSD-PAR-FEY  275
_XP_014086506.1_  239  FRTP------KYHNTS------------ITTP---VEVLVQLQ---RPSDG-------AVSD-PAT-FEY  275
```

```
_XP_004526638.1_   241  FRTP------KYHKTN------------ITEP---VEVFVQLQ---RPSDG-------AVSD-SVR-FEY  277
_XP_017471402.1_   239  FRTP------KFHRTN------------ITEP---VEVFVQLQ---RPSDG-------AVSD-PVK-FEY  275
_XP_012157903.1_   239  FRTP------KYHKTN------------ITEP---VEVFVQLQ---RPSDG-------AVSD-SVR-FEY  275
_XP_011185661.2_   237  LKTP------KYHKTD------------ITEP---VQVLVQLL---RPSDG-------AVSE-PWE-FKY  273
_XP_050322757.1_   238  LRTP------QYHNTD------------IEEP---VPVFVQLQ---RPSDG-------AVGE-PWQ-FKY  274
_XP_039962922.1_   238  LRTP------QYHNTD------------IEEP---VPVFVQLQ---RPSDG-------AVGE-PWQ-FKY  274
_XP_050322739.1_   238  LRTP------QYHNTD------------IEEP---VPVFVQLQ---RPSDG-------AVGE-PWQ-FKY  274
_XP_050322749.1_   238  LRTP------QYHNTD------------IEEP---VPVFVQLQ---RPSDG-------AVGE-PWQ-FKY  274
_XP_050322724.1_   238  LRTP------QYHNTD------------IEEP---VPVFVQLQ---RPSDG-------AVGE-PWQ-FKY  274
_XP_050322682.1_   238  LRTP------QYHNTD------------IEEP---VPVFVQLQ---RPSDG-------AVGE-PWQ-FKY  274
_CAG9823112.1_     243  FRTP------RYKTLD------------VTEP---VKVFIQLK---RPSDG-------AVSE-PLP-FEL  279
_KAG5873705.1_     238  FRTP------RYKTLD------------VTEP---VKVLIQLR---RPSDG-------ATSE-SLP-FEL  274
_KAH8030364.1_     218  FRTP------RYRDPV-----------MADVP---VDVHLLLR---RPSDG-------ALSE-PRA-FTL  255
_XP_023013891.1_   238  FKTP------RYKTLD------------VTEP---VKVFIQLR---RPSDG-------ATSE-SLP-FEM  274
_XP_028130780.2_   244  FRTP------RYKTLD------------VTDP---VDVFIQLR---RPSDG-------ATSE-ALP-FKL  280
_KAG5873704.1_     243  FRTP------RYKTLD------------VTEP---VKVLIQLR---RPSDG-------ATSE-SLP-FEL  279
_CAH1187690.1_     243  FRTP------RYKTID------------VTEP---VKVFIQLR---RPTDG-------ATSD-PLP-FEF  279
_CAH1098641.1_     243  FKTP------RYKTID------------VRDP---VKVFIQLR---RPSDG-------ATSE-AHQ-FEL  279
_XP_023028116.1_   243  FKTP------KYRTVN------------ITEP---VKVLIQLR---RPSDG-------STSE-SLP-FQF  279
_XP_031630227.1_   249  FKTP------RYDRFD------------FGGT---RRTFIQLC---RPSDG-------ACSD-PIA-FEF  285
_KAG5861047.1_     112  FRTP------RYKTLD------------VTEP---VKVLIQLR---RPSDG-------ATSE-SLP-FEL  148
_KAF2880990.1_     241  FRAP------RYKTLD------------VTEP---VKVFIQLR---RPSDG-------ATSE-PLP-FEF  277
_XP_018326789.1_   240  FRPP------RYRTLE------------ITEP---VKVYIQLR---RPSDG-------STSE-PLP-FQF  276
_XP_018326788.1_   240  FRPP------RYRTLE------------ITEP---VKVYIQLR---RPSDG-------STSE-PLP-FQF  276
_XP_031339184.1_   240  FKTP------PYRRLD------------VTEP---VKVFIQLR---RPSDT-------ATSE-PLP-FEL  276
_XP_045476069.1_   241  FKPP------RYHNSE------------ISDP---TKVFIQLR---RPSDG-------ATSE-PIP-FQF  277
_XP_044734149.1_   239  FTTP------KYHSLE------------VTEP---VKVFIQLF---RPSDR-------ACSE-PLP-FFL  275
_XP_044734150.1_   239  FTTP------KYHSLE------------VTEP---VKVFIQLF---RPSDR-------ACSE-PLP-FFL  275
_XP_044734148.1_   239  FTTP------KYHSLE------------VTEP---VKVFIQLF---RPSDR-------ACSE-PLP-FFL  275
_XP_044760528.1_   241  FKPP------RYHNTE------------ITDP---VKVLVQLK---RPSDG-------ATSE-SVP-FEF  277
_XP_044747918.1_   241  FRAP------KYRTLE------------VTEP---VKAFIQLK---RPSDG-------ATSE-PLP-FEF  277
_XP_044747917.1_   241  FRAP------KYRTLE------------VTEP---VKAFIQLK---RPSDG-------ATSE-PLP-FEF  277
_XP_045481017.1_   241  FRAP------KYRTLE------------VTEP---VKAFIQLK---RPSDG-------ATSE-SLP-FEF  277
_XP_045481018.1_   241  FRAP------KYRTLE------------VTEP---VKAFIQLK---RPSDG-------ATSE-SLP-FEF  277
_KAF5302274.1_     241  FRPP------RYKTLD------------ISEP---VKVLVQLR---RPSDG-------ATSE-PYH-FEL  277
_KAF5308883.1_     241  FRPP------RYKTLD------------VSEP---VKVFIQLR---RPSDG-------ATSE-PYH-FEL  277
_KRT79621.1_       239  FRPP------KYKTLD------------VIEP---VKMFIQLR---RPSDN-------ATSA-PVP-FQM  275
_BAD20728.1_       239  FRPP------KY------------------------KMFIQLR---RPSDN-------ATSA-PLA-FQM  266
_KAF2880988.1_     240  FRAP------QYKTLD------------VTEP---IKVLIQLR---RPSDG-------ATSE-PLP-FQF  276
```

```
_CAH1403258.1_     199  FRTP------RYKTLE------------IDHP---VQASIQLR---RPSDG-------ATSD-AKP-FQI  235
_CAD6992094.1_     238  LYPP------QYHNLD------------ITEP---VQVYVQLV----NVDG-------FASE-PCP-FKY  273
_XP_004537184.1_   238  LYPP------QYHNLD------------ITEP---VQVYVQLV----NVDG-------FASE-PCP-FKY  273
_XP_018572644.1_   238  FRTP------RYKTLE------------VTEP---VKVFIQLR---RPSDG-------ATSE-PLP-FEL  274
_RZB54393.1__      241  FKAP------RYHTLD------------ITEP---VKVFIQLR---RPSDG-------AASE-ALP-FEL  277
_XP_044255725.1_   241  FKAP------RYHTLD------------ITEP---VKVFIQLR---RPSDG-------ATSE-ALP-FEL  277
_EFA02850.1_       241  FKAP------RYHTLD------------ITEP---VKVFIQLR---RPSDG-------ATSE-ALP-FEL  277
_NP_001034507.1_   241  FKAP------RYHTLD------------ITEP---VKVFIQLR---RPSDG-------ATSE-ALP-FEL  277
_CAH1370311.1_     241  FKAP------RYHTLD------------ITEP---VKVFVQLK---RPSDG-------ATSE-PLP-FEL  277
_KAJ3630692.1_     241  FKAP------RYHTLD------------ITEP---VKVFVQLK---RPSDG-------ATSE-PLP-FEL  277
_XP_044255724.1_   241  FKAP------RYHTLD------------ITEP---VKVFIQLR---RPSDG-------ATSE-ALP-FEL  277
_KAH0811528.1_     241  FKAP------RYHTLD------------ITEP---VKVFVQLK---RPSDG-------ATSE-PLP-FEL  277
_CAH7716406.1_     237  FQPP------KYKTLD------------VTEP---VPVCLQLR---RPSDG-------ACSE-PVD-FEF  273
_CAH7716405.1_     237  FQPP------KYKTLD------------VTEP---VPVCLQLR---RPSDG-------ACSE-PVD-FEF  273
_CAH7716404.1_     237  FQPP------KYKTLD------------VTEP---VPVCLQLR---RPSDG-------ACSE-PVD-FEF  273
_XP_028130776.2_   238  FRTP------RYKTLD------------VTDP---VDVFIQLR---RPSDG-------ATSE-ALP-FKL  274
_CAH1985684.1_     237  FTPP------KYKTLD------------VTEP---VPVCIQLR---RPSDG-------ACSE-PVD-FEF  273
_XP_017781152.1_   241  FKTP------RYRTLD------------ITEP---VKVNIQLR---RPSDG-------AISE-PLP-FEL  277
_XP_017781151.1_   241  FKTP------RYRTLD------------ITEP---VKVNIQLR---RPSDG-------AISE-PLP-FEL  277
_XP_048522053.1_   238  FKVP------PYRTLD------------VSEP---VRVYVQLY---RPSDR-------LTSE-PRP-FEY  274
_XP_050303070.1_   253  FRAP------RYRNIE------------ITEP---VKAFIQLY---RPSDH-------ATSE-ALP-FQF  289
_CAG9762924.1_     242  FRPP------RYKTLD------------ISEP---VRVFIQLY---RPSDQ-------LKSD-PLP-FEL  278
_CAH0560654.1_     243  FKTP------RYKTLD------------VTEA---VNVHLQLR---RPSDG-------ATSE-SRP-FQL  279
_CAH0560655.1_     243  FKTP------RYKTLD------------VTEA---VNVHLQLR---RPSDG-------ATSE-SRP-FQL  279
_XP_018572643.1_   237  FRTP------RYKTLE------------VTEP---VKVFIQLR---RPSDG-------ATSE-PLP-FEL  273
_XP_030763656.1_   241  FKTP------RYKTLD------------VTEP---VKVFIQLY---RPSDQ-------VTSE-PLP-FEL  277
_XP_030763654.1_   241  FKTP------RYKTLD------------VTEP---VKVFIQLY---RPSDQ-------VTSE-PLP-FEL  277
_XP_018577945.1_   236  FKPP------KYRDLD------------VKEP---VRVFIQLY---RPSDG-------ATSK-PLP-FEL  272
_KAF7283276.1_     241  FRTP------RYKKLD------------VTEP---VKVFIQLF---RPSDQ-------VRSE-PLP-FEL  277
_KAF7283275.1_     241  FRTP------RYKKLD------------VTEP---VKVFIQLF---RPSDQ-------VRSE-PLP-FEL  277
_XP_030763617.1_   240  FKTP------RYKTLD------------VTES---VKVFIQLY---RPSDQ-------ATSE-PLP-FEL  276
_XP_030763619.1_   243  FKSP------PYKTTD------------VEEP---VRVFLQLY---RPSDR-------ATSE-PVP-FEL  279
_VEN60240.1_       148  FQPP------KYKTLD------------VTEP---VPVCLQLR---RPSDG-------ACSE-PVD-FEF  184
_KOB78236.1_       227  IETP------PYSGTT------------TTGP---IKVVVYLQ---RALTGDEAMDRNRTSN-SVP-YEY  270
_ERL85515.1_       168  FKVP------PYRTLD------------VSEP---VRVYVQLY---RPSDR-------LTSE-PRP-FEY  204
_ENN78766.1_       160  FKVP------PYRTLD------------VSEP---VRVYVQLY---RPSDR-------LTSE-PRP-FEY  196
_KAH1019371.1_     160  FKVP------PYRTLD------------VSEP---VRVYVQLY---RPSDR-------LTSE-PRP-FEY  196
_KAI9565420.1_     239  FRTP------RYRVTD------------VEEP---ISVYVQLR---RPSDG-------ACSE-SRR-FEY  275
_XP_045024905.1_   239  FRTP------RYRVTD------------VEEP---ISLYVQLR---RPSDG-------ACSE-SRR-FEY  275
```

```
_XP_046447094.1_  239  FRTP------RYRVTD------------VEEP---ISVYVQLR---RPSDG-------ACSE-SRR-FEY  275
_KZS08518.1_      239  FRTP------RYRVTD------------VEEP---ISLYVQLR---RPSDG-------ACSE-SRR-FEY  275
_XP_046636307.1_  239  FRTP------RYRVTD------------VEEP---ISVYVQLR---RPSDG-------ACSE-SRR-FEY  275
_EFX79013.1_      239  FRTP------RYRVTD------------VEEP---ISVYVQLR---RPSDG-------ACSE-SRR-FEY  275
_XP_032776606.2_  239  FRTP------RYRVTD------------VEEP---ISLYVQLR---RPSDG-------ACSE-SRR-FEY  275
_XP_046447093.1_  239  FRTP------RYRVTD------------VEEP---ISVYVQLR---RPSDG-------ACSE-SRR-FEY  275
_XP_046636306.1_  239  FRTP------RYRVTD------------VEEP---ISVYVQLR---RPSDG-------ACSE-SRR-FEY  275
_CAH0099799.1_    229  FRTP------RYRVTDVSYCKDDYVHSLVEEP---ISLYVQLR---RPSDG-------ACSE-SRR-FEY  277
_KAJ1531033.1_    233  FETP------AYKNIH------------VDEP---VLVYMQLR---QRTTK-------ATGE-PRV-FTL  269
_ACJ36225.1_      240  FKTP------RYKTLE------------IENP---IKVYVQLL---RPSDK-------STSE-PRP-FQY  276
_ACZ98167.1_      240  FKTP------RYKTLE------------IENP---IKVYVQLL---RPPDK-------STSE-PRP-FQY  276
_AME17867.1_      240  FKTP------RYKTLE------------IENP---IKVYVQLL---RPSDK-------STSD-PRP-FQY  276
_XP_042868409.1_  240  FKTP------RYKTLE------------IENP---IKVYVQLL---RPSDK-------STSD-PRP-FQY  276
_XP_045102711.1_  240  FKTP------RYKSLE------------VENP---VKVYVQLL---RPSDK-------STSE-PRP-FQY  276
_XP_047487558.1_  240  FKTP------RYKTLE------------IENP---IKVYVQLL---RPSDK-------STSE-PRP-FQY  276
_QBJ05651.1_      240  FKTP------RYKSLE------------VENP---VKVYVQLL---RPSDK-------STSE-PRP-FQY  276
_AZK36046.1_      240  FKTP------RYKSLE------------VENP---VKVYVQLL---RPSDK-------STSE-PRP-FQY  276
_XP_045581920.1_  240  FKTP------RYKSLE------------IENP---VKVYVQLL---RPSDK-------STSE-PRP-FQY  276
_XP_050704598.1_  240  FKTP------RYKSLE------------VENP---VKVYVQLL---RPSDK-------SMSE-PRP-FQY  276
_XP_027237995.1_  240  FKTP------RYKTLE------------IENP---IKVYVQLL---RPSDK-------STSE-PRP-FQY  276
_AXB88326.1_      240  FKTP------RYKSLE------------VENP---VKVYVQLL---RPSDK-------SISE-PRP-FQY  276
_XP_045102712.1_  240  FKTP------RYKSLE------------VENP---VKVYVQLL---RPSDK-------STSE-PRP-FQY  276
_MPC17461.1_      240  FKTP------RYKSLE------------VENP---VKVYVQLL---RPSDK-------STSE-PRP-FQY  276
_XP_027237994.1_  240  FKTP------RYKTLE------------IENP---IKVYVQLL---RPSDK-------STSE-PRP-FQY  276
_XP_027237996.1_  240  FKTP------RYKTLE------------IENP---IKVYVQLL---RPSDK-------STSE-PRP-FQY  276
_XP_045102715.1_  240  FKTP------RYKSLE------------VENP---VKVYVQLL---RPSDK-------STSE-PRP-FQY  276
_XP_053627990.1_  240  FKTP------RYKSLE------------IENP---VKVYVQLL---RPSDK-------STSE-PRP-FQY  276
_XP_053627989.1_  240  FKTP------RYKSLE------------IENP---VKVYVQLL---RPSDK-------STSE-PRP-FQY  276
_XP_053627983.1_  240  FKTP------RYKSLE------------IENP---VKVYVQLL---RPSDK-------STSE-PRP-FQY  276
_KAB7495058.1_    240  FKTP------KYKDIQ------------VDHS---VKVFIQLQ---RPSDK-------STSE-PRP-FEY  276
_KAF2353654.1_    240  FKTP------RYRTIE------------IENP---VKVFIQLQ---RPSDK-------TTSE-PRP-FEY  276
_ANH22235.1_      240  FKTP------RYKTLE------------VENP---VKVFVQLL---RPTDK-------STSE-PRN-FHF  276
_CAD7275741.1_    240  LRTP------RYRTLD------------VTDV---VKCRIQLM---RPSDK-------ATSA-PRA-FEY  276
_XP_042243403.1_  240  FKTP------RYKSLE------------IENP---VKVYVQLL---RPSDK-------STSE-SRP-FQY  276
_QTC09942.1_      240  FKTP------RYKTLE------------IENP---IKVYV------------------------------  258
_XP_018023738.1_  219  FKTP------RYKTLE------------IENP---VKVYIQLQ---RPSDK-------TTSE-PRP-FEY  255
_XP_019877552.1_  239  FKTP------AYKRVD------------IVDK---VRVQVQLR---RPSDG-------SVSD-SRP-FEF  275
_XP_049825875.1_  237  FNTP------AYKRMD------------IEEA---VRVQMQLR---RPSDG-------AVSE-SRP-FEF  273
_CAD7649074.1_    240  FRTP------RYFMDN------------LTQP---VSVQLQLR---RPSDG-------CLSD-ARV-FQL  276
```

```
_CAD7630012.1_     240  FRTP------RYYSEN------------LTQA---VSVQLQLR---RPSDG-------CLSD-ARV-FQI  276
_XP_054165343.1_   241  FRTP------RYCRDD------------LSQP---VSVMLQLR---RPSDG-------CLSD-PRA-FQL  277
_XP_044254219.1_   239  FKPP------PYKNLN------------IVDP---APVFMQLI---RPSDG-------VVSK-SVQ-FEY  275
_XP_053201827.1_   240  FRSP------KYYNEN------------ISSP---VTVNIQLR---RPSDG-------HTSE-SRS-FQF  276
_XP_015786990.1_   240  FRSP------KYYNEN------------ISQP---VTVNIQLR---RPSDG-------HTSE-PRQ-FQF  276
_KAI1295507.1_     240  FRTP------RYFDET------------IQQP---VLVQIQMR---RPSDS-------HTSD-PRP-FQL  276
_RWS07527.1_       240  FRTP------RYYNEN------------IQQP---VVVNIQLR---RPSDG-------QTSD-PRP-FQF  276
_RWS29859.1_       240  FRSP------RYYNDN------------IQQP---VHLNIQLR---RPSDG-------QTSE-ARA-FQF  276
_XP_053201825.1_   259  FRSP------KYYNEN------------ISSP---VTVNIQLR---RPSDG-------HTSE-SRS-FQF  295
_XP_054713467.1_   234  CNVP------PYFRPD------------ATKT---VDVFVHLR---NPVED-------IVGE-KRP-FRY  270
_XP_046915158.1_   243  LRTP------KYFDEN------------ISQP---VTINIQLR---RPSDG-------CLSL-PRS-FQI  279
_KAJ6217218.1_     243  LRTP------KYYDDS------------IQQP---VTVQIQLR---RPSDG-------CTSL-SRS-FQL  279
_KAI2801352.1_     243  LRTP------KYYDDS------------IQQP---VTVQIQLR---RPSDG-------CTSL-SRS-FQL  279
_KAH9406651.1_     242  LRTP------KYYDES------------IQQP---VTIQIQLR---RPSDG-------CTSL-SRS-FQL  278
_XP_017483538.1_   242  LRTP------KYYDES------------IQQP---VTIQIQLR---RPSDG-------CTSL-SRS-FQL  278
_KPM10359.1_       241  LRTP------RYYDET------------ISQP---VTVQMQLR---RPSDC-------CLSM-PRS-FQI  277
_UXI17582.1_       241  LRTP------RYYDET------------ISQP---VTVQMQLR---RPSDC-------CLSM-PRS-FQI  277
_KAF7488016.1_     241  LRTP------RYYDET------------ISQP---VTVQMQLR---RPSDC-------CLSM-PRS-FQI  277
_KAH9423377.1_     244  LRTP------KYFDET------------ISQP---VTVNIQLR---RPSDG-------CLSL-PRS-FQI  280
_OTF73464.1_       243  LRTP------KYFDEN------------ISQP---VTINIQLR---RPSDG-------CLSL-PRS-FQI  279
_XP_037072056.1_   240  FKTP------RYVNPE------------CDTP---VRVQIQLR---RPSDK-------QYSD-PRA-FTL  276
_XP_037083492.1_   240  FKTP------RYATLE------------LEAP---VRAQIQLR---RPSDR-------QYSE-ARP-FTF  276
_XP_037083508.1_   240  FKTP------RYATLE------------LEAP---VRAQIQLR---RPSDR-------QYSE-ARP-FTF  276
_XP_043215018.1_   240  FKTP------RYASLE------------LESP---VRAQIQLR---RPSDK-------QCSE-ARP-FTF  276
_KAF0314765.1_     240  FKTP------RYASLE------------LESP---VRAQIQLR---RPSDK-------QCSE-ARP-FTF  276
_XP_043215020.1_   240  FKTP------RYASLE------------LESP---VRAQIQLR---RPSDK-------QCSE-ARP-FTF  276
_XP_043189654.1_   240  FKTP------RYANPD------------LDTP---VRVQIQLR---RPSDK-------QYSE-SRA-FTL  276
_KAF0293344.1_     226  FKTP------RYANPD------------LDTP---VRVQIQLR---RPSDK-------QYSE-SRA-FTL  262
_XP_011496609.1_        ----------------------------------------------------------------------
_AGS12619.1_       240  FKTP------RYKDLE------------IEQP---VKVKIHLR---RPSDG-------AVSE-SRN-FEF  276
_TRY78048.1_       240  FKTP------RYKDLE------------IEQS---VKVKIHLR---RPSDG-------AVSE-SRN-FEF  276
_XP_040576307.1_   240  FRTP------RYENLE------------IDRP---VKVSMQLR---RPSDG-------TVSE-SRA-FEF  276
_CAB4056363.1_     240  FRTP------RYENLE------------IDRP---VKVSMQLR---RPSDG-------TVSE-SRA-FEF  276
_ALF84898.1_       240  LRTP------RYHDLE------------VEEN---IHVKIHLR---RPSDG-------AISD-PRP-FEL  276
_XP_023349538.1_   237  FKTP------KYHNIE------------IETP---VKVLLQLR---RPSDG-------AVSE-PRS-FEF  273
_XP_004522582.1_   240  FRTP------KYHKTD------------ITEP---VQVYIQLQ---RPSDG-------ATSK-PVK-FEF  276
_CAD7013622.1_     240  FRTP------KYHKTD------------ITEP---VQVYIQLQ---RPSDG-------ATSK-PVK-FEF  276
_XP_042902217.1_   239  FRTP------RYQNEN------------IQQP---VPVYIELR---KPSEN-------KRGD-PRP-FQY  275
_XP_042902218.1_   239  FRTP------RYQNEN------------IQQP---VPVYIELR---KPSEN-------KRGD-PRP-FQY  275
```

```
_XP_035215470.1_   239  FRTP------RYHNEN------------VQQP---VPVFVELR---KPSEN-------KRGE-PRP-FQY  275
_GBN14612.1_       238  FRTP------RYPNEN------------IQQP---VPVFVELR---KPSEN-------KRGE-PRP-FQY  274
_KAF8795965.1_     239  FRTP------RYPNEN------------IQQP---VPVFVELR---KPSEN-------KRGD-PRP-FQY  275
_KAF8795964.1_     239  FRTP------RYPNEN------------IQQP---VPVFVELR---KPSEN-------KRGD-PRP-FQY  275
_GFY66827.1_       239  FRTP------RYHNEN------------IQQP---VPVFVELR---KPSEN-------KRGE-PRP-FQY  275
_GFW66965.1_       239  FRTP------RYHNEN------------IQQP---VPVFVELR---KPSEN-------KRGE-PRP-FQY  275
_GFQ85823.1_       239  FRTP------RYHNEN------------IQQP---VPVFVELR---KPSEN-------KRGE-PRP-FQY  275
_GFT35953.1_       239  FRTP------RYHNEN------------IQQP---VPVFVELR---KPSEN-------KRGE-PRP-FQY  275
_XP_054714028.1_   239  FRTP------RYHNEN------------VQQP---VPVFVELR---KPSEN-------KRGE-PRP-FQY  275
_KAG8194583.1_     238  FRTP------RYPKEN------------IQQP---VPVFVELR---KPSEN-------KRGE-PRP-FQF  274
_GIY09899.1_       238  FRTP------RYPNEN------------IQQP---VPVFVELR---KPSEN-------KRGE-PRA-FQY  274
_GIY93593.1_       238  FRTP------RYPNEN------------IQQP---VPVFVELR---KPSEN-------KRGE-PRA-FQY  274
_XP_042902221.1_   251  FRIP------RYHNEN------------LQQP---VSVYVELR-----DEN-------RKGK-PRP-FQY  285
_XP_046962867.1_   240  FHTP------PYRDPS------------ERRH---VQVYLQLK---RTSDN-------ARSN-AVP-FEY  276
_XP_047530540.1_   240  FHTP------PYRDPS------------ERRH---VQVYLQLK---RTSDN-------ARSN-AVP-FEY  276
_XP_026486466.1_   240  FHTP------PYRDPS------------ERRH---VQVYLQLK---RTSDN-------ARSN-AVP-FEY  276
_XP_047530539.1_   240  FHTP------PYRDPS------------ERRH---VQVYLQLK---RTSDN-------ARSN-AVP-FEY  276
_XP_047530538.1_   240  FHTP------PYRDPS------------ERRH---VQVYLQLK---RTSDN-------ARSN-AVP-FEY  276
_XP_047530537.1_   240  FHTP------PYRDPS------------ERRH---VQVYLQLK---RTSDN-------ARSN-AVP-FEY  276
_XP_026486464.1_   240  FHTP------PYRDPS------------ERRH---VQVYLQLK---RTSDN-------ARSN-AVP-FEY  276
_XP_047530541.1_   240  FHTP------PYRDPS------------ERRH---VQVYLQLK---RTSDN-------ARSN-AVP-FEY  276
_XP_050347286.1_   240  FHTP------PYRDPS------------ERRH---VQVYLQLK---RTSDN-------ARSN-AVP-FEY  276
_XP_050347283.1_   240  FHTP------PYRDPS------------ERRH---VQVYLQLK---RTSDN-------ARSN-AVP-FEY  276
_XP_032515351.1_   241  FHTP------PYRDQA------------ETGH---VQVYLQLK---RISDN-------ARSN-AVP-FEY  277
_OWR46016.1_       241  FHTP------PYRDQA------------ETGH---VQVYLQLK---RISDN-------ARSN-AVP-FEY  277
_XP_032515346.1_   241  FHTP------PYRDQA------------ETGH---VQVYLQLK---RISDN-------ARSN-AVP-FEY  277
_CAG9579094.1_     241  FHTP------PYRDQA------------ETGH---VQVYLQLK---RISDN-------ARSN-AVP-FEY  277
_CAG9579091.1_     241  FHTP------PYRDQA------------ETGH---VQVYLQLK---RISDN-------ARSN-AVP-FEY  277
_XP_050668638.1_   239  FYTP------PYRDPH------------SQHH---VQVYVQLK---RTSDN-------ARSN-AIP-FEF  275
_XP_050668632.1_   239  FYTP------PYRDPH------------SQHH---VQVYVQLK---RTSDN-------ARSN-AIP-FEF  275
_XP_045495329.1_   239  FHAP------PYQNPH------------ATHH---VQVYIQLK---RTSDN-------ARSN-AVP-FEY  275
_XP_038214802.1_   239  FHAP------PYQNPH------------ATHN---VQVYIQLK---RTSDN-------ARSN-AVP-FEY  275
_CAH0714714.1_     240  FHTP------PYRDPH------------VGHH---VQVYLQLK---RTSDN-------ARSN-AIP-FEY  276
_CAH0714713.1_     240  FHTP------PYRDPH------------VGHH---VQVYLQLK---RTSDN-------ARSN-AIP-FEY  276
_CAH4029256.1_     239  FHVP------PYHKPT------------TPHH---VQVYIQLK---RISDN-------ARSN-PVP-FEY  275
_XP_047513081.1_   239  FHVP------PYHKPN------------NPHP---VQVYIQLK---RISDN-------ARSN-PVP-FEY  275
_XP_045529569.1_   239  FHVP------PYHKPT------------TPHH---VQVYIQLK---RISDN-------ARSN-PVP-FEY  275
_XP_045487500.1_   239  FHVP------PYHKPN------------NPHH---VQVYIQLK---RISDN-------ARSN-PVP-FEY  275
_XP_047513077.1_   239  FHVP------PYHKPN------------NPHP---VQVYIQLK---RISDN-------ARSN-PVP-FEY  275
```

```
_CAH4029257.1_     239  FHVP------PYHKPT------------TPHH---VQVYIQLK---RISDN-------ARSN-PVP-FEY  275
_XP_045487498.1_   239  FHVP------PYHKPN------------NPHH---VQVYIQLK---RISDN-------ARSN-PVP-FEY  275
_CAF4952989.1_     239  FHVP------PYHKPN------------NPHH---VQVYIQLK---RISDN-------ARSN-PVP-FEY  275
_XP_045447470.1_   240  FQTP------PYRDPR------------GRDH---VQVYLQLK---RTSDN-------ARSN-AVP-FEY  276
_CAH2108688.1_     179  FQTP------PYRDPQ------------GRDH---VQVYLQLK---RTSDN-------ARSN-AVP-FEY  215
_CAH0714724.1_     237  CFVP------RYH---------------SNRD---AEVGVELRMSLKTEEG-------ERNN-VMS-FNY  273
_XP_034827218.1_   240  FQTP------PYRNPN------------EQNH---VQVYLQLK---RTSDN-------ARSN-AIP-FEY  276
_XP_045768234.1_   240  FQTP------PYRNPN------------EQNH---VQVYLQLK---RTSDN-------ARSN-AIP-FEY  276
_XP_034827214.1_   240  FQTP------PYRNPN------------EQNH---VQVYLQLK---RTSDN-------ARSN-AIP-FEY  276
_XP_045768230.1_   240  FQTP------PYRNPN------------EQNH---VQVYLQLK---RTSDN-------ARSN-AIP-FEY  276
_XP_039751073.1_   240  FQTP------PYRNPH------------EQSH---VQVFLQLK---RTSDN-------ARSN-AIP-FEY  276
_XP_023942270.1_   240  FQTP------PYRNPH------------EQSH---VQVYLQLK---RSSDN-------ARSN-AIP-FEY  276
_XP_023942268.1_   240  FQTP------PYRNPH------------EQSH---VQVYLQLK---RSSDN-------ARSN-AIP-FEY  276
_XP_039751072.1_   240  FQTP------PYRNPH------------EQSH---VQVFLQLK---RTSDN-------ARSN-AIP-FEY  276
_XP_023942266.1_   240  FQTP------PYRNPH------------EQSH---VQVYLQLK---RSSDN-------ARSN-AIP-FEY  276
_CAH2240541.1_     227  FQTP------PYRNPH------------EQSH---VQVFLQLK---RTSDN-------ARSN-AIP-FEY  263
_XP_041985045.1_   241  FQTP------AYDRSD------------ISYN---VQVYLQLV---RNSDN-------ARSN-ALP-FEY  277
_XP_041985024.1_   241  FQTP------AYDRSD------------ISYN---VQVYLQLV---RNSDN-------ARSN-ALP-FEY  277
_XP_049868286.1_   248  FTAP------AYRDPH------------TQEH---VEVYIQLK---RPTDG-------ARSL-GIP-FTY  284
_XP_026314794.1_   248  FTTP------PYRDPA------------TGQH---VDVFIQLK---RPSDG-------TRSV-GVP-FTY  284
_XP_047994271.1_   239  FQAP------PYRDPT------------CMEN---VKVKIFLK---RVSDG-------ARSA-PVD-FEY  275
_KAJ2950301.1_     245  FTAP------PYRDPQ------------AREH---VEVFIQLK---RPTDN-------ARST-GIA-YTY  281
_XP_031784347.2_   233  FKTP------RYVAQN------------IDEP---VKVFLQLK---RPSDG-------ELSS-PVP-FLF  269
_KAG7305875.1_     238  FTTP------PYRDPH------------VQDH---VQAYIQLR---RPTDN-------ATSE-PLP-FEL  274
_WDD23886.1_       238  FTTP------PYRDPH------------VQDH---VQAYIQLR---RPTDN-------ATSE-PLP-FEL  274
_XP_048480424.1_   238  FTTP------PYRDPH------------VQDH---VQAYIQLR---RPTDN-------ATSE-PLP-FEL  274
_XP_048480423.1_   238  FTTP------PYRDPH------------VQDH---VQAYIQLR---RPTDN-------ATSE-PLP-FEL  274
_XP_048480422.1_   238  FTTP------PYRDPH------------VQDH---VQAYIQLR---RPTDN-------ATSE-PLP-FEL  274
_XP_048480421.1_   238  FTTP------PYRDPH------------VQDH---VQAYIQLR---RPTDN-------ATSE-PLP-FEL  274
_KAG7305876.1_     238  FTTP------PYRDPH------------VQDH---VQAYIQLR---RPTDN-------ATSE-PLP-FEL  274
_CAG9129242.1_     238  FTTP------PYRDPH------------VQDH---VQAYIQLR---RPTDN-------ATSE-PLP-FEL  274
_XP_048480420.1_   238  FTTP------PYRDPH------------VQDH---VQAYIQLR---RPTDN-------ATSE-PLP-FEL  274
_GBP53004.1_       112  FNTP------RYHNDR------------VEHD---VQAYIQLR---RPSDN-------AVSE-SVP-FEF  148
_XP_045534737.1_   239  FDIP------PYRDPA------------PRDN---VKVYLQLK---RVTDN-------ARSN-AVN-FEY  275
_XP_013180060.1_   239  FDIP------PYRDPA------------PHDN---VKVYLQLK---RVTDN-------ARSN-AVD-FEY  275
_XP_013180043.1_   239  FDIP------PYRDPA------------PHDN---VKVYLQLK---RVTDN-------ARSN-AVD-FEY  275
_XP_013180068.1_   239  FDIP------PYRDPA------------PHDN---VKVYLQLK---RVTDN-------ARSN-AVD-FEY  275
_XP_045534736.1_   239  FDIP------PYRDPA------------PRDN---VKVYLQLK---RVTDN-------ARSN-AVN-FEY  275
_KPJ06840.1_       239  FDIP------PYRDPA------------PRDN---VKVYLQLK---RVTDN-------ARSN-AVN-FEY  275
```

```
_KPJ05518.1_       239  FDIP------PYRDPA------------PHDN---VKVYLQLK---RVTDN-------ARSN-AVD-FEY  275
_CAG4934519.1_     239  FEIP------PYRNPD------------TTKN---VKVYLQLK---RTTDN-------ARSN-ALD-FEY  275
_XP_013149112.1_   239  FDIP------PYQDPT------------PRDN---VKV--------------------------------  255
_CAH2077165.1_     251  FDTP------PYRDPD------------VAAN---VKVFLQLK---RTTDN-------ARSN-ALD-FEY  287
_CAH2077167.1_     251  FDTP------PYRDPD------------VAAN---VKVFLQLK---RTTDN-------ARSN-ALD-FEY  287
_XP_037051415.1_   232  FETP------AYATHD------------IIEP---VDVLFELV---RPSDG-------RRSE-PVRYFRY  269
_XP_037051414.1_   230  FETP------AYATHD------------ISEP---VVVLFELV---RPSDD-------KRSDPARY-FRY  267
_XP_022815079.1_   239  FETP------AYKYPN------------TTDH---VNVYFQLK---RLSDN-------ARSN-SLP-FEY  275
_CAB3516522.1_     239  FETP------AYKYPN------------TTDH---VNVYFQLK---RLSDN-------ARSN-SLP-FEY  275
_XP_050562120.1_   239  FETP------AYRIPN------------TTDH---VNVYFQLK---RLSDN-------ARSN-SLP-FEY  275
_XP_050562121.1_   239  FETP------AYRIPN------------TTDH---VNVYFQLK---RLSDN-------ARSN-SLP-FEY  275
_QHD56218.1_       239  FETP------AYKYPN------------TTDH---VNVYFQLK---RLSDN-------ARSN-SLP-FEY  275
_AIA24466.1_       239  FETP------AYKYPN------------TTGH---VNVYFQLK---RLSDN-------ARSN-SLP-FEY  275
_XP_022815076.1_   239  FETP------AYKYPN------------TTDH---VNVYFQLK---RLSDN-------ARSN-SLP-FEY  275
_QHD56217.1_       239  FETP------AYKYPN------------TTDH---VNVYFQLK---RLSDN-------ARSN-SLP-FEY  275
_XP_050562118.1_   239  FETP------AYRIPN------------TTDH---VNVYFQLK---RLSDN-------ARSN-SLP-FEY  275
_AIA24467.1_       239  FETP------AYKYPN------------TTGH---VNVYFQLK---RLSDN-------ARSN-SLA-FEY  275
_XP_047019808.1_   239  FETP------AYKYPN------------TTDH---VHVHFQLK---RLSDN-------ARSN-SLP-FEY  275
_AEO51736.1_       239  FETP------AYKYPN------------TTDH---VHVHFQLK---RLSDN-------ARSN-SLP-FEY  275
_XP_049696680.1_   239  FETP------AYKYPN------------TTDH---VHVHFQLK---RLSDN-------ARSN-SLP-FEY  275
_XP_049696682.1_   239  FETP------AYKYPN------------TTDH---VHVHFQLK---RLSDN-------ARSN-SLP-FEY  275
_XP_049696676.1_   239  FETP------AYKYPN------------TTDH---VHVHFQLK---RLSDN-------ARSN-SLP-FEY  275
_XP_026744931.1_   239  FETP------AYRFPN------------ITDH---VNVHFQLK---RMSDN-------ARSN-SLP-FEY  275
_XP_026732895.1_   239  FETP------AYRFPN------------ITDH---VNVHFQLK---RMSDN-------ARSN-SLP-FEY  275
_CAH0581650.1_     239  FETP------AYKFPN------------ITDH---VNVHFQLK---RMSDN-------ARSN-SLP-FEY  275
_KAH9640014.1_     284  FETP------AYKYPN------------TTDH---VNVYFQLK---RLSDN-------ARSN-SLP-FEY  320
_PZC78315.1_       113  FETP------AYKYPN------------TTDH---VHVHFQLK---RLSDN-------ARSN-SLP-FEY  149
_KAF9795796.1_     101  FETP------AYRIPN------------TTDH---VNVYFQLK---RLSDN-------ARSN-SLP-FEY  137
_AEC52926.1_       239  FETP------AYRIPN------------ITEP---VNVRFQLK---RLTDN-------ARSN-SLP-FEY  275
_KAH7940301.1_     226  FRTP------RYRDPI-----------MADVP---VDVHLLLR---RPSDG-------ALSE-PRA-FTL  263
_ADK39025.1_       238  FETP------PYKNPN------------ITDN---VNVRFQLR---RLSDK-------MTSN-SLP-FEY  274
_XP_037298148.1_   238  FETP------PYKNPN------------ITDN---VNVRFQLR---RLSDK-------MTSN-SLP-FEY  274
_RVE50742.1_       234  FKTP------PYRDQS------------IARP---ITVQFQLH---RVSDH-------AKSN-VYS-FEY  270
_CAH0692665.1_     234  FKTP------PYRDQS------------IARP---ITVQFQLH---RVSDH-------AKSN-VYS-FEY  270
_XP_028166351.1_   238  LKTP------PYRDQR------------LDHH---VTVQFQLQ---RLSDG-------AKSN-AYS-FEY  274
_XP_028166291.1_   238  LKTP------PYRDQR------------LDHH---VTVQFQLQ---RLSDG-------AKSN-AYS-FEY  274
_CAB3235314.1_     239  FETP------AFRDPR------------TSEH---VTVRFQLK---RVTDN-------ARSN-SFP-FEY  275
_KAJ0175867.1_     241  FETP------PYRNPQ------------ITDH---VKVKFQLK---RTTDN-------ARSN-SLP-FEY  277
_XP_028031027.1_   239  FNTP------PYRDPQ------------RDEH---VTVHFQLK---RLTDD-------ARSN-SFA-FEY  275
```

```
_NP_001166296.1_  239  FNTP------PYRDPQ------------RDEH---VTVHFQLK---RLTDD-------ARSN-SFA-FEY  275
_XP_037870194.1_  239  FNTP------PYRDPQ------------RDEH---VTVHFQLK---RLTDD-------ARSN-SFA-FEY  275
_XP_037870193.1_  239  FNTP------PYRDPQ------------RDEH---VTVHFQLK---RLTDD-------ARSN-SFA-FEY  275
_XP_028031025.1_  239  FNTP------PYRDPQ------------RDEH---VTVHFQLK---RLTDD-------ARSN-SFA-FEY  275
_XP_013187225.1_  237  FTTP------RYFRP-------------TQHH---VTVQLHLQ---RLTDK-------ACSA-PVN-FEY  272
_XP_052755011.1_  237  FHTP------PYRHPD------------TADH---VTVQLQLQ---RLTDN-------ARSS-PVS-FEY  273
_XP_053613182.1_  237  FHTP------RYHRP-------------SQQH---VTVQLLLQ---RLTDG-------AKST-PVN-FEY  272
_XP_053613180.1_  237  FHTP------RYHRP-------------SQQH---VTVQLLLQ---RLTDG-------AKST-PVN-FEY  272
_CAG9785863.1_    232  FKTP------PYRDQT------------IPQP---VTVQFQLH---RVSDH-------AKSN-VYS-FEY  268
_KAG6461275.1_         ----------------------------------------------------------------------
_KAG6461281.1_         ----------------------------------------------------------------------
_XP_037302137.1_  235  FNTP------PYVYPN------------ITNK---VKVHFQLR---RLSDG-------AVSN-SLP-YIY  271
_KAG6461269.1_    235  FNTP------PYVYPN------------ITNK---VKVHFQLR---RLSDG-------AVSN-SLP-YIY  271
_KAG4069896.1_    228  FETP------AYATHD------------ISEP---VVVLLELV---RPSDG-------RHSKRACS-FRY  265
_XP_044003989.1_  246  LKTP------AYSNLN------------IKEP---VTIFIQLY---RRSNK-------ANSE-FHE-IKF  282
_XP_045768240.1_  240  FQTP------PYRNPN------------EQNH---VQVYLQLK---RTSDG-------VRSN-AIP-FEY  276
_KAG9509099.1_    243  IKTP------KYYDES------------IQQP---VIVRVQLR---RPSDN-------QVSE-PMN-FQF  279
_XP_013118267.1_  237  FKTP------RYHNTD------------IVKR---AQVFIQLV---RFSAE-------TT----------  265
_XP_005183026.2_  238  FKTP------RYHNTE------------IEKR---AQVFIQLV---RFSSE-------PVAN-GQT-PTF  274
_XP_050303432.1_  254  FQAP------PYQMSD------------GEDM---VNVKLKLY---RPSDG---------SE-GEA-RDF  288
_TMW48950.1_      239  FKTP------RYINPD------------VNHK---VQVYIQLY---RPSDG-------ETSE-ALP-FEY  275
_XP_037882085.1_  238  FKTP------RYHNTE------------AIHR---PQVYIQLV---RPSDG-------ATSE-PLP-FEY  274
_KAI8126541.1_    240  FKTP------RYINPD------------VKHS---VQVYIQLF---RPSDG-------ETSE-ALP-FEF  276
_KAI8126540.1_    239  FKTP------RYINPD------------VKHS---VQVYIQLF---RPSDG-------ETSE-ALP-FEF  275
_XP_037811156.1_  239  FKTP------RYINPD------------VKHS---VQVYIQLF---RPSDG-------ETSE-ALP-FEF  275
_XP_046812595.1_  239  FKTP------RYINPD------------VKHS---VQVYIQLF---RPSDG-------ETSE-ALP-FEF  275
_XP_046812596.1_  239  FKTP------RYINPD------------VKHS---VQVYIQLF---RPSDG-------ETSE-ALP-FEF  275
_XP_046812591.1_  239  FKTP------RYINPD------------VKHS---VQVYIQLF---RPSDG-------ETSE-ALP-FEF  275
_XP_046812590.1_  239  FKTP------RYINPD------------VKHS---VQVYIQLF---RPSDG-------ETSE-ALP-FEF  275
_KNC34143.1_      239  FKTP------RYINPD------------VKHS---VQVYIQLF---RPSDG-------ETSE-ALP-FEF  275
_XP_046812589.1_  239  FKTP------RYINPD------------VKHS---VQVYIQLF---RPSDG-------ETSE-ALP-FEF  275
_KAI8126539.1_    239  FKTP------RYINPD------------VKHS---VQVYIQLF---RPSDG-------ETSE-ALP-FEF  275
_XP_037958917.1_  241  FKAP------RYHNQD------------ILEK---AKVFIQLV---RPSDG-------VQSD-SLP-FEY  277
_XP_037929595.1_  243  FKTP------PYHNQD------------VLEK---AKVFIQLV---RPSDG-------VQSE-SIP-FEY  279
_XP_037957513.1_  241  FKTP------RYHNQD------------VLEK---AKVFIQLV---RPSDG-------VQSE-SIP-FEY  277
_XP_037958115.1_  243  FKTP------PYHNQD------------VLEK---AKVFIQLV---RPSDG-------VQSE-SIP-FEY  279
_XP_037958114.1_  243  FKTP------PYHNQD------------VLEK---AKVFIQLV---RPSDG-------VQSE-SIP-FEY  279
_XP_037957511.1_  241  FKTP------RYHNQD------------VLEK---AKVFIQLV---RPSDG-------VQSE-SIP-FEY  277
_XP_037958918.1_  241  FKTP------RYYNED------------VSEK---AKIFIQLV---RSSDG-------VQSK-PLP-FEY  277
```

```
_RXG71823.1_       204  FKTP------RYKDLE------------VTQT---VKVSLQLF---RPSDK-------SVSE-TVP-FEY  240
_KAB7494622.1_     181  FKTP------RYKDLE------------VTQT---VKVSLQLF---RPSDK-------SVSE-TVP-FEY  217
_KAI9584339.1_     238  FTTP------PYRIKN------------ITEP---VEVYIQLK---RPSDG-------LLSE-PFP-FRY  274
_XP_037884983.1_   238  FTPP------PYRIKN------------ITEP---VEVFIQLK---RPSDG-------LLSE-PVT-FRY  274
_XP_054744821.1_   237  FRTP------RYPQAD------------LPKT---AKPYIELE---CPSLD-------KRSD-PLE-FVF  273
_XP_053961676.1_   237  FRTP------RYPQAD------------LPKT---AKPYIELE---CPSLD-------KRSD-PLE-FVF  273
_XP_053961674.1_   237  FRTP------RYPQAD------------LPKT---AKPYIELE---CPSLD-------KRSD-PLE-FVF  273
_XP_004526634.2_   237  FRTP------RFPQSD------------LPKT---VKSYIELE---CPSLE-------KKSD-ALE-FVF  273
_CAD6998451.1_     237  FRTP------RFPQSD------------LPKT---VKSYIELE---CPSLE-------KKSD-ALE-FVF  273
_XP_012157894.1_   237  FRTP------RFPQSD------------LPKT---VKSYIELE---CPSLE-------KKSD-ALE-FVF  273
_XP_028894493.1_   237  FRTP------RYPQAD------------LPKT---AKTYIELE---CPSLD-------KRSD-ALE-FVF  273
_XP_036327693.1_   237  FRTP------RYPQAD------------LPKT---AKPYIELE---CPSLD-------KRSE-ALE-FVF  273
_XP_017471333.1_   237  FRTP------RYPQAD------------LPKT---AKPYIELE---CPSLD-------KRSE-ALE-FVF  273
_XP_039952608.1_   237  FRTP------RYPQAD------------LPKT---AKTYIELE---RPSLD-------KRSD-ALE-FVF  273
_XP_050320023.1_   237  FRTP------RYPQAD------------LPKT---AKTYIELE---RPSLD-------KRSD-ALE-FVF  273
_XP_036225572.1_   237  FRTP------RYPQAD------------LPKT---AKTYIELE---RPSLD-------KRSD-ALE-FVF  273
_XP_039952607.1_   237  FRTP------RYPQAD------------LPKT---AKTYIELE---RPSLD-------KRSD-ALE-FVF  273
_XP_036225564.1_   237  FRTP------RYPQAD------------LPKT---AKTYIELE---RPSLD-------KRSD-ALE-FVF  273
_XP_036225569.1_   237  FRTP------RYPQAD------------LPKT---AKTYIELE---RPSLD-------KRSD-ALE-FVF  273
_XP_050320021.1_   237  FRTP------RYPQAD------------LPKT---AKTYIELE---RPSLD-------KRSD-ALE-FVF  273
_XP_039952606.1_   237  FRTP------RYPQAD------------LPKT---AKTYIELE---RPSLD-------KRSD-ALE-FVF  273
_XP_011207860.1_   237  FRTP------RYPQAD------------LPKT---AKTYIELE---RPSLD-------KRSD-ALE-FVF  273
_XP_011207859.1_   237  FRTP------RYPQAD------------LPKT---AKTYIELE---RPSLD-------KRSD-ALE-FVF  273
_XP_029407466.1_   237  FRTP------RYPQAD------------LPKT---AKTYIELE---RPSLD-------KRSD-ALE-FVF  273
_XP_019847003.2_   237  FRTP------RYPQAD------------LPKT---AKTYIELE---RPSLD-------KRSD-ALE-FVF  273
_XP_014086488.2_   237  FRTP------RYPQAD------------LPKT---AKTYIELE---RPSLD-------KRSD-ALE-FVF  273
_XP_049317393.1_   237  FRTP------RYPQAD------------LPKT---AKTYIELE---RPSLD-------KRSD-ALE-FVF  273
_XP_039952604.1_   237  FRTP------RYPQAD------------LPKT---AKTYIELE---RPSLD-------KRSD-ALE-FVF  273
_XP_018787557.1_   237  FRTP------RYPQAD------------LPKT---AKTYIELE---RPSLD-------KRSD-ALE-FVF  273
_OQR68950.1_       240  FRSPVCSQHLPYSEGD------------QLHA---VKVHL-----------------------------  264
_XP_003741763.1_   240  FRSPDC--RKHFTAGN------------NLQTCIKIEVHLRRS---R----------------------  269
_XP_022699297.1_   240  FKSPNC-LDHVVTERD------------QLYP---VKIHLRRT---RVN--------------------  269
_XP_022665229.1_   240  FKSPNC-LDHVVTERD------------QLYP---VKIHLRRT---RVN--------------------  269
_XP_022665225.1_   240  FKSPNC-LDHVVTERD------------QLYP---VKIHLRRT---RVN--------------------  269
_XP_022665224.1_   240  FKSPNC-LDHVVTERD------------QLYP---VKIHLRRT---RV---------------------  268
_XP_033219687.1_   239  FKTP------KYCNQD------------IAEP---VTVLVQLR---KKSDG-------TTSK-AVP-FQM  275
_XP_033227990.1_   239  FKTP------KYRDQK------------IEEP---VTVLIQLR---KKSDG-------TTSK-AVP-FQM  275
_XP_043466013.1_   238  FRTP------AYCNQD------------ITEP---VGVQIQLQ---KKSDG-------STSK-PLP-FIM  274
_XP_051172671.1_   238  FRTP------AYCNQD------------ISEP---VGVQIQLR---KKSDG-------STSK-PLP-FIM  274
```

```
_CAD7643228.1_    237  FRSP------KYCDLS------------VQNA---IPVNVELV---RPS---------------------  261
_XP_054157157.1_  241  FRTP------RYADTN------------VLSP---VAVAVQLR---RPSDG-------QMSE-SMP-FWL  277
_CAD7645633.1_    238  FRSP------KYCDVS------------IATP---VAVNVELR---RPSDG-------GTSE-SLP-FYL  274
_CAD7632611.1_    179  FRSP------KYCDLS------------VQNA---IPVNVELV---RPSDG-------GTSE-SLP-FYL  215
_KAJ6223158.1_    239  FVVP------AYDMVM------------SADEY-QVTVNVQLR---RPSDG-------CLSE-PIT-FDY  277
_KAI2804035.1_    239  FVVP------AYDMVM------------SADEY-QVTVNVQLR---RPSDG-------CLSE-PIT-FDY  277
_KAH9398209.1_    231  FTVP------PYEL--------------------------------------------------------  238
_GFY77286.1_      239  FQTP------AYKNIN------------LQHS---AECFIQLR---RPSDG-------AVGK-PRN-FTF  275
_GFQ73388.1_      239  FQTP------AYKNIN------------LQHS---AECFIQLR---RPSDG-------AVGK-PRN-FTF  275
_GFT25575.1_      239  FQTP------AYKNIN------------LQHS---AECFIQLR---RPSDG-------AVGK-PRN-FTF  275
_KAF8763655.1_    239  FQTP------AYKNIN------------LQHS---LDCFIQLR---RPSDG-------AVGK-PRT-FTF  275
_GFX76677.1_      239  FQTP------AYKNIN------------LQHS---AECFIQLR---RPSDG-------AVGK-PRN-FTF  275
_XP_035207052.1_  239  FQTP------AYKSTN------------VNQS---IDCFIQLR---RPSDG-------AVGK-PRA-FIL  275
_KFM74898.1_      239  FQTP------AYKSTT------------VNQS---IDCFIQLR---RPSDG-------AVGK-PRA-FIL  275
_XP_035207059.1_  239  FQTP------AYKSTN------------VNQS---IDCFIQLR---RPSDG-------AVGK-PRA-FIL  275
_XP_035207066.1_  239  FQTP------AYKSTN------------VNQS---IDCFIQLR---RPSDG-------AVGK-PRA-FIL  275
_BBD75287.1_      239  FQTP------AYRDVT------------VPQG---VDCFVQLR---RPSDG-------AVGK-PRT-FCF  275
_XP_042900952.1_  239  FQTP------PYRDVT------------VPQG---VDCFVQLR---RPSDG-------AVGK-PRT-FCF  275
_KAG8201816.1_    238  FETP------PYKTTD------------IQQP---VECYIQLR---RPSDG-------AVGD-PIN-FTY  274
_GIX87435.1_      249  FQTP------AYKNIN------------LQQS---AECFVQLR---RPSDG-------AVGK-PRT-FTF  285
_GBL81029.1_      239  FQTP------AYKNIN------------LQHS---LECFIQLR---RPSDG-------AVGK-PRT-FTF  275
_GIY01087.1_      239  FQTP------AYKNIN------------LQQS---AECFVQLR---RPSDG-------AVGK-PRT-FSF  275
_XP_054715436.1_  239  FRTP------VYRDCE------------LQGR--NVECFVQLR---RPSDG-------AVGK-ARP-FTL  276
_XP_044576112.1_  236  FESP------AYKDLS------------IKEP---VQVFIQLR---KMTGR------IDKSE-PLP-FTL  273
_XP_008556435.3_  235  FESP------AYHNVN------------IEEP---AQVFIQLR---KLSDH-------YASE-PLP-FTF  271
_CAD6234446.1_    236  FESP------AYSDLS------------IEEP---VQVFIQLR---KLTGR------IDRSE-PLP-FTF  273
_XP_044284598.1_  235  FRTP------PFHKD-------------IFEP---VTVKMQLR---RPSDQ-------EVSD-PMD-FRY  270
_XP_033794511.1_  235  FRTP------AYFKS-------------IMEA---VTVKMQLR---RPSDQ-------EVSE-PMD-FRY  270
_XP_033794512.1_  235  FRTP------AYFKS-------------IMEA---VTVKMQLR---RPSDQ-------EVSE-PMD-FRY  270
_XP_029449578.1_  235  FRTP------AYFKP-------------ITEA---VTVKMQLR---RPSDQ-------EVSE-PMD-FRY  270
_XP_030052048.1_  235  FRTP------TYFKP-------------ITEA---VTVKMQLR---RPSDQ-------EVSE-PVD-FRY  270
_XP_007423639.1_  235  FKTP------PFYKD-------------IMDP---VTVKMQLR---RPSDQ-------EVSD-PMD-FRY  270
_XP_039203483.1_  235  FKTP------PFFKD-------------ITDP---VTVKMQLR---RPSDQ-------EVSD-PMD-FRY  270
_XP_034283206.1_  235  FKTP------PFFKD-------------ISDP---VTVKMQLR---RPSDQ-------EVSD-PMD-FRY  270
_XP_033001023.1_  235  FRTP------PFYKD-------------IGEP---VTVKMQLR---RPSDQ-------EVSD-PMD-FRY  270
_XP_053239675.1_  235  FRTP------PFYKD-------------IAEP---VTVKMQLR---RPSDQ-------EVSD-PMD-FRY  270
_XP_034966804.1_  235  FRTP------PFYKD-------------IGEP---VTVKMQLR---RPSDQ-------EVSD-PMD-FRY  270
_XP_028580982.1_  224  FRTP------PFYKD-------------IAEP---VTVKMQLC---RPSDQ-------EVSD-PMD-FRY  259
_XP_042302822.1_  236  FRTP------PFYKD-------------ISEP---ITVKMQLR---RPSDQ-------EVSH-PMD-FRY  271
```

```
_XP_015280979.1_   224  FRTP------SFYKD-------------LSEP---VTVKMQLR---RPSDQ-------EVSD-PMD-FRY  259
_XP_048363544.1_   235  FRTP------PFYKD-------------ITEP---VTVKMQLR---RPSDQ-------EVSD-PMD-FRY  270
_KAF7252806.1_     209  FRTP------PFHKD-------------IFEP---VTVKMQLR---RPSDQ-------EVSD-PMD-FRY  244
_XP_034958398.1_        ----------------------------------------------------------------------
_CAD5116312.1_     238  FKTP------PFPFQ-------------ITEP---CKVFMYLK---RMSED-------LKSE-PIE-FTY  273
_KAJ1519398.1_     231  ILTP------AFRTVQ------------TSEP---VQVFVQLR---YADAP------RMASE-PWP-FTL  268
_XP_053103001.1_   235  FRTP------PFYEE-------------ITEP---VTVKMQLR---RPSDQ-------EVSD-PMD-FRY  270
_XP_032070730.1_   235  FRTP------PFFKD-------------IPDP---VTVKMQLR---RPSDQ-------EVSD-PMD-FRY  270
_XP_013922116.1_   235  FRTP------PFFKD-------------IPDP---VTVKMQLR---RPSDQ-------EVSD-PMD-FRY  270
_XP_015667999.1_   235  FKTP------PFFKD-------------ITDP---ATVKMQLR---RPSDQ-------EVSD-PMD-FRY  270
_XP_026551846.1_   235  FKTP------PFFKD-------------ISDP---VTVKMQLR---RPSDQ-------EVSD-PMD-FRY  270
_XP_026532518.1_   235  FKTP------PFFKD-------------IPDP---VTVKMQLR---RPSDQ-------EVSD-PMD-FRY  270
_KAJ7341301.1_     235  FRTP------PFYKD-------------ISEP---VTVKMQLR---RPSDQ-------EV----------  262
_KAJ6667038.1_     224  FRTP------AFYRD-------------IIEP---VTVKMQLR---RPSDQ-------EVSD-PMD-FRY  259
_XP_003227818.2_   224  FRTP------PFYKD-------------ISEP---ITVQMQLR---RPSDQ-------ELSL-PMD-FRY  259
_XP_020633036.1_   237  FRTP------PFYKD-------------ISEP---VTVKMQLR---RPSDQ-------EVSD-PMD-FRY  272
_XP_054842059.1_   235  FRTP------PFYKD-------------ITEP---MTVKMQLR---RPSDQ-------EVSD-PMD-FRY  270
_XP_005238641.2_   224  FRTP------PFLKD-------------ITEP---ITVKMQLR---RPSDQ-------EVSE-PMD-FRY  259
_XP_005445671.2_   224  FRTP------PFLKD-------------ITEP---VTVKMQLR---RPSDQ-------EVSE-PMD-FRY  259
_XP_054050029.1_   224  FRTP------PFLKD-------------ITEP---VTVKMQLR---RPSDQ-------EVSE-PMD-FRY  259
_XP_013810845.1_   224  FRTP------PFLKD-------------IAEP---VTVKMQLR---RPSDQ-------EVSE-PMD-FRY  259
_XP_026702002.1_   224  FRTP------PFLKD-------------ITEP---VTVKMQLR---RPSDQ-------EVSE-PMD-FRY  259
_XP_025892242.1_   224  FRTP------PFLRD-------------ITEP---VTVKMQLR---RPSDQ-------EVSE-PMD-FRY  259
_XP_005507389.1_   224  FRTP------PFLRD-------------ITEP---VTVKMQLR---RPSDQ-------EVSE-PMD-FRY  259
_XP_032911247.1_   224  FRTP------PFLRD-------------ITEP---VTVKMQLR---RPSDQ-------EVSE-PMD-FRY  259
_XP_031959657.1_   224  FRTP------PFLKD-------------ITEP---VTVKMQLR---RPSDQ-------EVSE-PMD-FRY  259
_XP_041905291.1_   224  FRTP------PFLKD-------------ITEP---VTVKMQLR---RPSDQ-------EVSE-PMD-FRY  259
_XP_051648836.1_   224  FRTP------PFLKD-------------ITEP---VTVKMQLR---RPSDQ-------EVSE-PMD-FRY  259
_XP_054239488.1_   224  FRTP------PFLKD-------------ITEP---VTVKMQLR---RPSDQ-------EVSE-PMD-FRY  259
_XP_039914164.1_   224  FRTP------PFLRD-------------ITEP---VTVKMQLR---RPSDQ-------EVSE-PMD-FRY  259
_XP_005422689.1_   224  FRTP------PFLRD-------------ISEP---VTVKMQLR---RPSDQ-------EVSE-PMD-FRY  259
_KAF2987757.1_     224  FRTP------PFLRD-------------ISEP---VTVKMQLR---RPSDQ-------EVSE-PMD-FRY  259
_XP_038665087.1_   235  FRTP------AYYQIN------------ITEP---VSVKMQLR---RPSDQ-------EVSE-SME-FRY  271
_GCB59945.1_       235  FRTP------AYYQIN------------ITEP---VSVKMQLR---RPSDQ-------EVSE-FME-FRY  271
_XP_041065616.1_   231  FRTP------AYCQTN------------ITEP---VSVKMQLR---RPSDQ-------EVSE-SME-FRY  267
_XP_007890564.1_   235  FRTP------AYCQTN------------ISEP---MTVKMQLR---RPSDQ-------EVSE-PME-FRY  271
_XP_051868609.1_   227  FRTP------AYCQSN------------ITEP---VSVKMQLR---RPSDQ-------EVSE-PME-FRY  263
_XP_032881051.1_   234  FRTP------AYCQTN------------ITEP---VSVKMQLR---RPSDQ-------EVSQ-PME-FRY  270
_XP_048456938.1_   144  FRTP------AYCNTN------------ITEP---VSVKMQLR---RPSDQ-------QVSE-SME-FRY  180
```

```
_KAJ1155552.1_     235  FKTP------AFYKN-------------ITEP---VTVKMQLR---RPSDQ-------EVSE-PID-FRY  270
_KAH0622877.1_          ----------------------------------------------------------------------
_CAI5653329.1_     236  FKTP------PYHNTS------------ITDS---VTVHMQLR---RPSDQ-------EVSE-PMD-FRY  272
_CAI5653328.1_     236  FKTP------PYHNTS------------ITDS---VTVHMQLR---RPSDQ-------EVSE-PMD-FRY  272
_NXS54806.1_       242  FRTP------PFLKD-------------ITEP---VTVKMQLR---RPSDQ-------EVSE-PMD-FRY  277
_XP_009504395.1_   232  FRTP------PFLKD-------------ITEP---VTVKMQLR---RPSDQ-------EVSE-PMD-FRY  267
_NXJ41381.1_       235  FRTP------PFLKD-------------ITEP---VTVKMQLR---RPSDQ-------EVSE-PMD-FRY  270
_NXT24719.1_       235  FRTP------PFLKD-------------ITEP---ITVKMQLR---RPSDQ-------EVSE-PMD-FRY  270
_NXK23502.1_       235  FRTP------PFLKD-------------ITEP---VTVKMQLR---RPSDQ-------EVSE-PMD-FRY  270
_NXK03070.1_       235  FRTP------PFLKD-------------ITEP---VTVKMQLR---RPSDQ-------EVSE-PMD-FRY  270
_XP_037260944.1_   235  FRTP------PFLKD-------------ITEP---VTVKMQLR---RPSDQ-------EVSE-PMD-FRY  270
_XP_040467369.1_   235  FRTP------PFLKD-------------ITEP---VTVKMQLR---RPSDQ-------EVSE-PMD-FRY  270
_NXY71491.1_       235  FRTP------PFLKD-------------ITEP---VTVKMQLR---RPSDQ-------EVSE-PMD-FRY  270
_NXN54417.1_       235  FRTP------PFLKD-------------ITEP---VTVKMQLR---RPSDQ-------EVSE-PMD-FRY  270
_NXX19490.1_       235  FRTP------PFLKD-------------ITEP---VTVKMQLR---RPSDQ-------EVSE-PMD-FRY  270
_NXL47199.1_       235  FRTP------PFLKD-------------ITEP---ITVKMQLR---RPSDQ-------EVSE-PMD-FRY  270
_XP_050749583.1_   235  FRTP------PFLKD-------------ITEP---VTVKMQLR---RPSDQ-------EVSE-PMD-FRY  270
_NXD76242.1_       235  FRTP------PFLKD-------------ITEP---VTVKMQLR---RPSDQ-------EVSE-PMD-FRY  270
_KFZ56827.1_       235  FRTP------PFLKD-------------ITEP---ITVKMQLR---RPSDQ-------EVSE-PMD-FRY  270
_NXH67691.1_       235  FRTP------PFLKD-------------ITEP---VTVKMQLR---RPSDQ-------EVSE-PMD-FRY  270
_NXS45828.1_       235  FRTP------PFLKD-------------ITEP---VTVKMQLR---RPSDQ-------EVSE-PMD-FRY  270
_KFP52034.1_       235  FRTP------PFLKD-------------ITEP---VTVKMQLR---RPSDQ-------EVSE-PMD-FRY  270
_NWH44485.1_       235  FRTP------PFLKD-------------ITEP---VTVKMQLR---RPSDQ-------EVSE-PMD-FRY  270
_NWW85416.1_       235  FRTP------PFLKD-------------ITEP---VTVKMQLR---RPSDQ-------EVSE-PMD-FRY  270
_NWU66320.1_       235  FRTP------PFLKD-------------ITEP---VTVKMQLR---RPSDQ-------EVSE-PMD-FRY  270
_XP_025919906.1_   235  FRTP------PFLKD-------------IAEP---VTVKMQLR---RPSDQ-------EVSE-PMD-FRY  270
_KAF1483947.1_     235  FRTP------PFLKD-------------ITEP---VTVKMQLR---RPSDQ-------EVSE-PMD-FRY  270
_NXQ87688.1_       235  FRTP------PFLKD-------------ITEP---VTVKMQLR---RPSDQ-------EVSE-PMD-FRY  270
_NXX07562.1_       235  FRTP------PFLKD-------------ITEP---VTVKMQLR---RPSDQ-------EVSE-PMD-FRY  270
_XP_009280788.2_   235  FRTP------PFLKD-------------ITEP---VTVKMQLR---RPSDQ-------EVSE-PMD-FRY  270
_XP_014821857.1_   235  FRTP------PFLKD-------------ITEP---VTVKMQLR---RPSDQ-------EVSE-PMD-FRY  270
_NWU51443.1_       235  FRTP------PFLKD-------------ITEP---VTVKMQLR---RPSDQ-------EVSE-PMD-FRY  270
_NXV35521.1_       235  FRTP------PFLKD-------------ITEP---VTVKMQLR---RPSDQ-------EVSE-PMD-FRY  270
_KFV80780.1_       235  FRTP------PFLKD-------------ITEP---VTVKMQLR---RPSDQ-------EVSE-PMD-FRY  270
_XP_032039522.1_   235  FRTP------PFLKD-------------ITEP---VTVKMQLR---RPSDQ-------EVSE-PMD-FRY  270
_NWT43027.1_       235  FRTP------PFLKD-------------ITEP---VTVKMQLR---RPSDQ-------EVSE-PMD-FRY  270
_KAF1651581.1_     235  FRTP------PFLKD-------------ITEP---VTVKMQLR---RPSDQ-------EVSE-PMD-FRY  270
_NXU26374.1_       235  FRTP------PFLKD-------------ITEP---VTVKMQLR---RPSDQ-------EVSE-PMD-FRY  270
_NWX46229.1_       235  FRTP------PFLKD-------------ITEP---VTVKMQLR---RPSDQ-------EVSE-PMD-FRY  270
```

```
_PKU40340.1_       235  FRTP------PFLKD-------------ITEP---VTVKMQLR---RPSDQ-------EVSE-PMD-FRY  270
_XP_027309078.1_   235  FRTP------PFLKD-------------ITEP---VTVKMQLR---RPSDQ-------EVSE-PMD-FRY  270
_KAF1573683.1_     235  FRTP------PFLKD-------------ITEP---VTVKMQLR---RPSDQ-------EVSE-PMD-FRY  270
_KAF1578928.1_     235  FRTP------PFLKD-------------ITEP---VTVKMQLR---RPSDQ-------EVSE-PMD-FRY  270
_NXN47104.1_       235  FRTP------PFLKD-------------ITEP---VTVKMQLR---RPSDQ-------EVSE-PMD-FRY  270
_NXV20393.1_       235  FRTP------PFLKD-------------ITEP---VTVKMQLR---RPSDQ-------EVSE-PMD-FRY  270
_KAF1551267.1_     235  FRTP------PFLKD-------------ITEP---ITVKMQLR---RPSDQ-------EVSE-PMD-FRY  270
_KAF1406210.1_     235  FRTP------PFLKD-------------ITEP---VTVKMQLR---RPSDQ-------EVSE-PMD-FRY  270
_NXV43201.1_       235  FRTP------PFLKD-------------ITEP---VTVKMQLR---RPSDQ-------EVSE-PMD-FRY  270
_NWQ98669.1_       235  FRTP------PFLKD-------------ITEP---VTVKMQLR---RPSDQ-------EVSE-PMD-FRY  270
_XP_052639968.1_   235  FRTP------PFLKD-------------ITEP---VTVKMQLR---RPSDQ-------EVSE-PMD-FRY  270
_XP_010573143.1_   235  FRTP------PFLKD-------------ITEP---VTVKMQLR---RPSDQ-------EVSE-PMD-FRY  270
_KAF1479604.1_     235  FRTP------PFLKD-------------ITEP---VTVKMQLR---RPSDQ-------EVSE-PMD-FRY  270
_NXG89109.1_       235  FRTP------PFLKD-------------ITEP---VTVKMQLR---RPSDQ-------EVSE-PMD-FRY  270
_KFQ89757.1_       235  FKTP------PFLKD-------------ITEP---VTVKMQLR---RPSDQ-------EVSE-PMD-FRY  270
_NWZ21787.1_       235  FRTP------PFLKD-------------ITEP---VTVKMQLR---RPSDQ-------EVSE-PMD-FRY  270
_NXI74048.1_       235  FRTP------PFLKD-------------ITEP---VTVKMQLR---RPSDQ-------EVSE-PMD-FRY  270
_NWX76247.1_       235  FRTP------PFLKD-------------ITEP---VTVKMQLR---RPSDQ-------EVSE-PMD-FRY  270
_NXF29788.1_       235  FRTP------PFLKD-------------ITEP---VTVKMQLR---RPSDQ-------EVSE-PMD-FRY  270
_KAF1444142.1_     235  FRTP------PFLKD-------------ITEP---ITVKMQLR---RPSDQ-------EVSE-PMD-FRY  270
_KFV20215.1_       235  FRTP------PFLKD-------------ITEP---VTVKMQLR---RPSDQ-------EVSE-PMD-FRY  270
_NXI44657.1_       235  FRTP------PFHKD-------------ITEP---VTVKMQLR---RPSDQ-------EVSE-PMD-FRY  270
_NXJ92321.1_       235  FRTP------PFLKD-------------ITEP---VTVKMQLR---RPSDQ-------EVSE-PMD-FRY  270
_NWZ49902.1_       235  FRTP------PFLKD-------------ITEP---VTVKMQLR---RPSDQ-------EVSE-PMD-FRY  270
_NWY59444.1_       235  FRTP------PFLKD-------------ITEP---VTVKMQLR---RPSDQ-------EVSE-PMD-FRY  270
_KFW64019.1_       235  FRTP------PFLKD-------------ITEP---VTVKMQLR---RPSDQ-------EVSE-PMD-FRY  270
_NXJ55634.1_       235  FRTP------PFLKD-------------ITEP---VTVKMQLR---RPSDQ-------EVSE-PMD-FRY  270
_KAF1513372.1_     235  FRTP------PFLKD-------------ITEP---VTVKMQLR---RPSDQ-------EVSE-PMD-FRY  270
_NXW14598.1_       235  FRTP------PFLKD-------------ITEP---ITVKMQLR---RPSDQ-------EVSE-PMD-FRY  270
_NXC74247.1_       235  FRTP------PFLKD-------------ITEP---VTVKMQLR---RPSDQ-------EVSE-PMD-FRY  270
_NXT57254.1_       235  FRTP------PFLKD-------------ITEP---VTVKMQLR---RPSDQ-------EVSE-PMD-FRY  270
_XP_047916679.1_   235  FRTP------PFLKD-------------ITEP---VTVKMQLR---RPSDQ-------EVSE-PMD-FRY  270
_XP_029879190.1_   235  FRTP------PFLKD-------------ITEP---VTVKMQLR---RPSDQ-------EVSE-PMD-FRY  270
_NXH09241.1_       235  FRTP------PFHKD-------------ITEP---VTVKMQLR---RPSDQ-------EVSE-PMD-FRY  270
_XP_040407374.1_   235  FRTP------PFLKD-------------ITEP---VTVKMQLR---RPSDQ-------EVSE-PMD-FRY  270
_KFW76037.1_       235  FRTP------PFLKD-------------ITEP---VTVKMQLR---RPSDQ-------EVSE-PMD-FRY  270
_KFP65945.1_       235  FRTP------PFLKD-------------ITEP---VTVKMQLR---RPSDQ-------EVSE-PMD-FRY  270
_NXX55682.1_       235  FRTP------PFLKD-------------ITEP---VTVKMQLR---RPSDQ-------EVSE-PMD-FRY  270
_XP_035178333.1_   235  FRTP------PFLKD-------------ITEP---VTVKMQLR---RPSDQ-------EVSE-PMD-FRY  270
```

```
_NXE47317.1_       235  FRTP------PFLKD-------------ITEP---VTVKMQLR---RPSDQ-------EVSE-PMD-FRY  270
_XP_035408877.1_   235  FRTP------PFLKD-------------ITEP---VTVKMQLR---RPSDQ-------EVSE-PMD-FRY  270
_KGL94141.1_       235  FRTP------PFLKD-------------ITEP---VTVKMQLR---RPSDQ-------EVSE-PMD-FRY  270
_NXN68403.1_       235  FRTP------PFLKD-------------ITEP---VTVKMQLR---RPSDQ-------EVSE-PMD-FRY  270
_NXW28331.1_       235  FRTP------PFLKD-------------ITEP---VTVKMQLR---RPSDQ-------EVSE-PMD-FRY  270
_XP_049657509.1_   235  FRTP------PFLKD-------------ITEP---VTVKMQLR---RPSDQ-------EVSE-PMD-FRY  270
_XP_049657511.1_   235  FRTP------PFLKD-------------ITEP---VTVKMQLR---RPSDQ-------EVSE-PMD-FRY  270
_NXG30098.1_       235  FRTP------PFLKD-------------ITEP---VTVKMQLR---RPSDQ-------EVSE-PMD-FRY  270
_NXV86645.1_       235  FRTP------PFLKD-------------ITEP---VTVKMQLR---RPSDQ-------EVSE-PMD-FRY  270
_NXX83200.1_       235  FRTP------PFHKD-------------ITEP---VTVKMQLR---RPSDQ-------EVSE-PMD-FRY  270
_NXI59898.1_       235  FRTP------PFLKD-------------ITEP---VTVKMQLR---RPSDQ-------EVSE-PMD-FRY  270
_NWU89521.1_       235  FKTP------PFLKD-------------ITEP---VTVKMQLR---RPSDQ-------EVSE-PMD-FRY  270
_NXK45490.1_       235  FRTP------PFLKD-------------ITEP---VTVKMQLR---RPSDQ-------EVSE-PMD-FRY  270
_NWR55599.1_       235  FRTP------PFLKD-------------ITEP---VTVKMQLR---RPSDQ-------EVSE-PMD-FRY  270
_NXL37824.1_       235  FRTP------PFLKD-------------ITEP---ITVKMQLR---RPSDQ-------EVSE-PMD-FRY  270
_NWI33553.1_       235  FRTP------PFLKD-------------ITEP---VTVKMQLR---RPSDQ-------EVSE-PMD-FRY  270
_KFQ74517.1_       235  FRTP------PFLKD-------------ITEP---VTVKMQLR---RPSDQ-------EVSE-PMD-FRY  270
_NXV82662.1_       235  FRTP------PFLKD-------------ITEP---VTVKMQLR---RPSDQ-------EVSE-PMD-FRY  270
_NWJ10087.1_       235  FRTP------PFLRD-------------ISEP---VTVKMQLR---RPSDQ-------EVSE-PMD-FRY  270
_NXN24483.1_       235  FRTP------PFLKD-------------ITEP---VTVKMQLR---RPSDQ-------EVSE-PMD-FRY  270
_NWR80208.1_       235  FRTP------PFLKD-------------ITEP---VTVKMQLR---RPSDQ-------EVSE-PMD-FRY  270
_NXT37538.1_       235  FKTP------PFLKD-------------ITEP---VTVKMQLR---RPSDQ-------EVSE-PMD-FRY  270
_XP_032844994.2_   235  FRTP------PFLKD-------------ITEP---VTVKMQLR---RPSDQ-------EVSE-PMD-FRY  270
_NXW47649.1_       235  FRTP------PFLKD-------------ITEP---VTVKMQLR---RPSDQ-------EVSE-PMD-FRY  270
_NXA40398.1_       235  FRTP------PFLRD-------------ITEP---VTVKMQLR---RPSDQ-------EVSE-PMD-FRY  270
_NXW64893.1_       235  FRTP------PFLKD-------------ITEP---VTVKMQLR---RPSDQ-------EVSE-PMD-FRY  270
_NXX89165.1_       235  FRTP------PFLKD-------------ITEP---VTVKMQLR---RPSDQ-------EVSE-PMD-FRY  270
_NXQ96911.1_       235  FRTP------PFLKD-------------ITEP---VTVKMQLR---RPSDQ-------EVSE-PMD-FRY  270
_KFP18273.1_       235  FRTP------PFLKD-------------ITEP---VTVKMQLR---RPSDQ-------EVSE-PMD-FRY  270
_NXC40882.1_       235  FRTP------PFLRD-------------ITEP---VTVKMQLR---RPSDQ-------EVSE-PMD-FRY  270
_NXE10305.1_       235  FRTP------PFLKD-------------ITEP---VTVKMQLR---RPSDQ-------EVSE-PMD-FRY  270
_KFP88087.1_       235  FRTP------PFLKD-------------ITEP---VTVKMQLR---RPSDQ-------EVSE-PMD-FRY  270
_NWW47871.1_       235  FRTP------PFLKD-------------ITEP---VTVKMQLR---RPSDQ-------EVSE-PMD-FRY  270
_NXJ61595.1_       235  FRTP------PFLKD-------------ITEP---VTVKMQLR---RPSDQ-------EVSE-PMD-FRY  270
_NXD13814.1_       235  FRTP------PFLRD-------------ITEP---VTVKMQLR---RPSDQ-------EVSE-PMD-FRY  270
_KFR16961.1_       235  FRTP------PFLKD-------------ITEP---VTVKMQLR---RPSDQ-------EVSE-PMD-FRY  270
_NWX24312.1_       235  FRTP------PFLKD-------------ITEP---VTVKMQLR---RPSDQ-------EVSE-PMD-FRY  270
_NXA28267.1_       235  FRTP------PFLKD-------------ITEP---VTVKMQLR---RPSDQ-------EVSE-PMD-FRY  270
_NXL66270.1_       235  FRTP------PFLKD-------------ITEP---VTVKMQLR---RPSDQ-------EVSE-PMD-FRY  270
```

NXA49976.1	235	FRTP------PFLRD-------------ITEP---VTVKMQLR---RPSDQ-------EVSE-PMD-FRY	270
NXT77624.1	235	FRTP------PFLKD-------------ITEP---VTVKMQLR---RPSDQ-------EVSE-PMD-FRY	270
KFQ92336.1	235	FRTP------PFLKD-------------ITEP---VTVKMQLR---RPSDQ-------EVSE-PMD-FRY	270
NXE25013.1	235	FRTP------PFLKD-------------ITEP---VTVKMQLR---RPSDQ-------EVSE-PMD-FRY	270
NXE92240.1	235	FRTP------PFLKD-------------ITEP---VTVKMQLR---RPSDQ-------EVSE-PMD-FRY	270
NWH80943.1	235	FRTP------PFLKD-------------ITEP---VTVKMQLR---RPSDQ-------EVSE-PMD-FRY	270
NWI19058.1	235	FRTP------PFLRD-------------ISEP---VTVKMQLR---RPSDQ-------EVSE-PMD-FRY	270
NXG80610.1	235	FRTP------PFLKD-------------ITEP---VTVKMQLR---RPSDQ-------EVSE-PMD-FRY	270
NWR01486.1	235	FRTP------PFLRD-------------ITEP---ITVKMQLR---RPSDQ-------EVSE-PMD-FRY	270
NXK81337.1	235	FRTP------PFLRD-------------ITEP---VTVKMQLR---RPSDQ-------EVSE-PMD-FRY	270
NXS90563.1	235	FRTP------PFLKD-------------ITEP---VTVKMQLR---RPSDQ-------EVSE-PMD-FRY	270
NXC17430.1	235	FRTP------PFLKD-------------ITEP---VTVKMQLR---RPSDQ-------EVSE-PMD-FRY	270
NXE77245.1	235	FRTP------PFLKD-------------ITEP---VTVKMQLR---RPSDQ-------EVSE-PMD-FRY	270
KFQ29788.1	236	FRTP------PFLKD-------------ITEP---VTVKMQLR---RPSDQ-------EVSE-PMD-FRY	271
KFO89327.1	235	FRTP------PFLKD-------------ITEP---VTVKMQLR---RPSDQ-------EVSE-PMD-FRY	270
NXD64381.1	235	FRTP------PFLRD-------------ITEP---VTVKMQLR---RPSDQ-------EVSE-PMD-FRY	270
NXY45081.1	235	FRTP------PFLKD-------------ITEP---VTVKMQLR---RPSDQ-------EVSE-PMD-FRY	270
KGL72871.1	235	FRTP------PFLRD-------------ISEP---VTVKMQLR---RPSDQ-------EVSE-PMD-FRY	270
_XP_053919504.1_	235	FRTP------PFLKD-------------ITEP---VTVKMQLR---RPSDQ-------EVSE-PMD-FRY	270
NXL85459.1	235	FRTP------PFVRD-------------ITEP---VTVKMQLR---RPSDQ-------EVSE-PMD-FRY	270
NXO02254.1	235	FKTP------PFLKD-------------ITEP---VTVKMQLR---RPSDQ-------EVSE-PMD-FRY	270
NXJ76034.1	235	FRTP------PFLKD-------------ITEP---VTVKMQLR---RPSDQ-------EVSE-PMD-FRY	270
NXM93491.1	235	FRTP------PFLRD-------------ITEP---VTVKMQLR---RPSDQ-------EVSE-PMD-FRY	270
_XP_030345800.1_	235	FRTP------PFLRD-------------ITEP---VTVKMQLR---RPSDQ-------EVSE-PMD-FRY	270
_XP_030302544.1_	235	FRTP------PFLRD-------------ITEP---VTVKMQLR---RPSDQ-------EVSE-PMD-FRY	270
_XP_030345801.1_	235	FRTP------PFLRD-------------ITEP---VTVKMQLR---RPSDQ-------EVSE-PMD-FRY	270
KQK77724.1	235	FRTP------PFLRD-------------ITEP---VTVKMQLR---RPSDQ-------EVSE-PMD-FRY	270
NXP09913.1	235	FRTP------PFLKD-------------ITEP---VTVKMQLR---RPSDQ-------EVSE-PMD-FRY	270
NXR33018.1	235	FRTP------PFLRD-------------ITEP---VTVKMQLR---RPSDQ-------EVSE-PMD-FRY	270
NXM76881.1	235	FRTP------PFLKD-------------ITEP---VTVKMQLR---RPSDQ-------EVSE-PMD-FRY	270
NXS27423.1	235	FRTP------PFLKD-------------ITEP---VTVKMQLR---RPSDQ-------EVSE-PMD-FRY	270
NXM55821.1	235	FRTP------PFLRD-------------ITEP---VTVKMQLR---RPSDQ-------EVSE-PMD-FRY	270
_XP_054678500.1_	235	FRTP------PFLKD-------------ITEP---VTVKMQLR---RPSDQ-------EVSE-PMD-FRY	270
_XP_015712248.1_	235	FRTP------PFLRD-------------ITEP---ITVKMQLR---RPSDQ-------EVSE-PMD-FRY	270
NWX92058.1	235	FRTP------PFLRD-------------ITEP---VTVKMQLR---RPSDQ-------EVSE-PMD-FRY	270
_XP_048793646.1_	235	FRTP------PFLRD-------------ITEP---ITVKMQLR---RPSDQ-------EVSE-PMD-FRY	270
NXF04247.1	235	FRTP------PFLKD-------------ITEP---VTVKMQLR---RPSDQ-------EVSE-PMD-FRY	270
_XP_042683331.1_	235	FRTP------PFLRD-------------ITEP---ITVKMQLR---RPSDQ-------EVSE-PMD-FRY	270
NWV22147.1	235	FRTP------PFLKD-------------ITEP---VTVKMQLR---RPSDQ-------EVSE-PMD-FRY	270

NXA04361.1	235	FRTP------PFLKD-------------ITEP---VTVKMQLR---RPSDQ-------EVSE-PMD-FRY	270
_XP_010116198.1_	241	FRTP------PFLKD-------------ITEP---VTVKMQLR---RPSDQ-------EVSE-PMD-FRY	276
NXG59741.1	235	FRTP------PFLRD-------------ITEP---VTVKMQLR---RPSDQ-------EVSE-PMD-FRY	270
_XP_042735326.1_	235	FRTP------PFLRD-------------ITEP---ITVKMQLR---RPSDQ-------EVSE-PMD-FRY	270
NXM29886.1	235	FRTP------PFLKD-------------ITEP---VTVKMQLR---RPSDQ-------EVSE-PMD-FRY	270
_NP_001161198.2_	235	FRTP------PFLRD-------------ITEP---ITVKMQLR---RPSDQ-------EVSE-PMD-FRY	270
_XP_031451029.1_	235	FRTP------PFLRD-------------ITEP---ITVKMQLR---RPSDQ-------EVSE-PMD-FRY	270
NXR49262.1	235	FRTP------PFLRD-------------ITEP---VTVKMQLR---RPSDQ-------EVSE-PMD-FRY	270
_XP_052555416.1_	235	FRTP------PFLRD-------------ITEP---ITVKMQLR---RPSDQ-------EVSE-PMD-FRY	270
NWZ67085.1	235	FRTP------PFLRD-------------ITEP---VTVKMQLR---RPSDQ-------EVSE-PMD-FRY	270
_P16236.2_	235	FRTP------PFLRD-------------ITEP---ITVKMQLR---RPSDQ-------EVSE-PMD-FRY	270
_XP_050566010.1_	235	FRTP------PFLKD-------------ITEP---VTVKMQLR---RPSDQ-------EVSE-PMD-FRY	270
NWS67030.1	235	FRTP------PFLKD-------------ITEP---VTVKMQLR---RPSDQ-------EVSE-PMD-FRY	270
_XP_005143850.3_	235	FRTP------PFLRD-------------ITEP---VTVKMQLR---RPSDQ-------EVSE-PMD-FRY	270
_XP_050566011.1_	235	FRTP------PFLKD-------------ITEP---VTVKMQLR---RPSDQ-------EVSE-PMD-FRY	270
_XP_050566009.1_	235	FRTP------PFLKD-------------ITEP---VTVKMQLR---RPSDQ-------EVSE-PMD-FRY	270
NWR30963.1	235	FRTP------PFLKD-------------ITEP---VTVKMQLR---RPSDQ-------EVSE-PMD-FRY	270
NWS17896.1	235	FRTP------PFLKD-------------ITEP---VTVKMQLR---RPSDQ-------EVSE-PMD-FRY	270
_XP_021248574.1_	235	FRTP------PFLRD-------------ITEP---ITVKMQLR---RPSDQ-------EVSE-PMD-FRY	270
AAA48721.1	235	FRTP------PFLRD-------------ITEP---ITVKMQLR---RPSDQ-------EVSE-PMD-FRY	270
NWI54208.1	235	FRTP------PFLKD-------------ITEP---VTVKMQLR---RPSDQ-------EVSE-PMD-FRY	270
_XP_027509532.1_	235	FRTP------PFLKD-------------ITEP---VTVKMQLR---RPSDQ-------EVSE-PMD-FRY	270
NXP71979.1	235	FKTP------PFLKD-------------ITEP---VTVKMQLR---RPSDQ-------EVSE-PMD-FRY	270
NXX25352.1	235	FRTP------PFLRD-------------ITEP---ITVKMQLR---RPSDQ-------EVSE-PMD-FRY	270
NXI18618.1	235	FRTP------PFLRD-------------ITEP---VTVKMQLR---RPSDQ-------EVSE-PMD-FRY	270
NXS09623.1	235	FRTP------PFLKD-------------ITEP---VTVKMQLR---RPSDQ-------EVSE-PMD-FRY	270
NXU77980.1	235	FRTP------PFLRD-------------ITEP---VTVKMQLR---RPSDQ-------EVSE-PMD-FRY	270
_XP_027509533.1_	235	FRTP------PFLKD-------------ITEP---VTVKMQLR---RPSDQ-------EVSE-PMD-FRY	270
NWV71036.1	235	FRTP------PFLRD-------------ITEP---VTVKMQLR---RPSDQ-------EVSE-PMD-FRY	270
NXF89482.1	235	FKTP------PFLKD-------------ITEP---VTVKMQLR---RPSDQ-------EVSE-PMD-FRY	270
NWY39470.1	235	FRTP------PFLRD-------------ITEP---VTVKMQLR---RPSDQ-------EVSE-PMD-FRY	270
NXT59960.1	235	FRTP------PFLRD-------------ITEP---VTVKMQLR---RPSDQ-------EVSE-PMD-FRY	270
NWU74340.1	235	FRTP------PFLKD-------------ITEP---VTVKMQLR---RPSDQ-------EVSE-PMD-FRY	270
NXB84481.1	235	FRTP------PFLRD-------------ISEP---VTVKMQLR---RPSDQ-------EVSE-PMD-FRY	270
NWV73885.1	235	FRTP------PFLKD-------------ITEP---VTVKMQLR---RPSDQ-------EVSE-PMD-FRY	270
OPJ58894.1	235	FRTP------PFLRD-------------ITEP---VTVKMQLR---RPSDQ-------EVSE-PMD-FRY	270
_XP_048152104.1_	235	FRTP------PFLKD-------------ITEP---VTVKMQLR---RPSDQ-------EVSE-PMD-FRY	270
_XP_054036037.1_	235	FRTP------PFLKD-------------ITEP---VTVKMQLR---RPSDQ-------EVSE-PMD-FRY	270
NXR18149.1	235	FRTP------PFLRD-------------ITEP---VTVKMQLR---RPSDQ-------EVSE-PMD-FRY	270

```
_NXP97533.1_       235  FRTP------PFLRD-------------ISEP---VTVKMQLR---RPSDQ-------EVSE-PMD-FRY  270
_NXJ05495.1_       235  FRTP------PFLRD-------------ITEP---VTVKMQLR---RPSDQ-------EVSE-PMD-FRY  270
_XP_032538932.1_   235  FRTP------PFLKD-------------ITEP---VTVKMQLR---RPSDQ-------EVSE-PMD-FRY  270
_NWI96387.1_       235  FRTP------PFLKD-------------ITEP---VTVKMQLR---RPSDQ-------EVSE-PMD-FRY  270
_NXR65995.1_       235  FRTP------PFLRD-------------ITEP---VTVKMQLR---RPSDQ-------EVSE-PMD-FRY  270
_NXK42398.1_       235  FRTP------PFLKD-------------ITEP---VTVKMQLR---RPSDQ-------EVSE-PMD-FRY  270
_XP_032538931.1_   235  FRTP------PFLKD-------------ITEP---VTVKMQLR---RPSDQ-------EVSE-PMD-FRY  270
_NXY33181.1_       235  FRTP------PFLRD-------------ITEP---VTVKMQLR---RPSDQ-------EVSE-PMD-FRY  270
_XP_050171009.1_   235  FRTP------PFLKD-------------ITEP---VTVKMQLR---RPSDQ-------EVSE-PMD-FRY  270
_NXO50939.1_       235  FRTP------PFLKD-------------ITEP---VTVKMQLR---RPSDQ-------EVSE-PMD-FRY  270
_NWZ32937.1_       235  FRTP------PFLRD-------------ITEP---VTVKMQLR---RPSDQ-------EVSE-PMD-FRY  270
_NXP01828.1_       235  FRTP------PFLRD-------------ITEP---VTVKMQLR---RPSDQ-------EVSE-PMD-FRY  270
_NXL00458.1_       236  FRTP------PFLKD-------------ITEP---VTVKMQLR---RPSDQ-------EVSE-PMD-FRY  271
_XP_010401310.4_   235  FRTP------PFLKD-------------ITEP---VTVKMQLR---RPSDQ-------EVSE-PMD-FRY  270
_NXH62149.1_       235  FRTP------PFLRD-------------ITEP---VTVKMQLR---RPSDQ-------EVSE-PMD-FRY  270
_NXG43824.1_       235  FRTP------PFLKD-------------ITEP---VTVKMQLR---RPSDQ-------EVSE-PMD-FRY  270
_XP_017677337.1_   235  FRTP------PFLKD-------------ITEP---VTVKMQLR---RPSDQ-------EVSE-PMD-FRY  270
_NXU09156.1_       235  FRTP------PFLKD-------------ITEP---VTVKMQLR---RPSDQ-------EVSE-PMD-FRY  270
_NXB38645.1_       235  FRTP------PFLRD-------------ITEP---VTVKMQLR---RPSDQ-------EVSE-PMD-FRY  270
_XP_027594504.1_   235  FRTP------PFLKD-------------ITEP---VTVKMQLR---RPSDQ-------EVSE-PMD-FRY  270
_NXM46269.1_       235  FRTP------PFLKD-------------ITEP---VTVKMQLR---RPSDQ-------EVSE-PMD-FRY  270
_NWS79257.1_       235  FRTP------PFLRD-------------ITEP---VTVKMQLR---RPSDQ-------EVSE-PMD-FRY  270
_NXB04839.1_       235  FRTP------PFLRD-------------ITEP---VTVKMQLR---RPSDQ-------EVSE-PMD-FRY  270
_NXC62865.1_       235  FRTP------PFLRD-------------ITEP---VTVKMQLR---RPSDQ-------EVSE-PMD-FRY  270
_NXN18277.1_       235  FRTP------PFLKD-------------ITEP---VTVKMQLR---RPSDQ-------EVSE-PMD-FRY  270
_XP_027760039.1_   235  FRTP------PFLKD-------------ITEP---VTVKMQLR---RPSDQ-------EVSE-PMD-FRY  270
_NXI90311.1_       235  FRTP------PFLKD-------------ITEP---VTVKMQLR---RPSDQ-------EVSE-PMD-FRY  270
_NWY17003.1_       235  FRTP------PFLKD-------------ITEP---VTVKMQLR---RPSDQ-------EVSE-PMD-FRY  270
_NXH97099.1_       235  FRTP------PFLRD-------------ITEP---VTVKMQLR---RPSDQ-------EVSE-PMD-FRY  270
_NWS97308.1_       235  FRTP------PFLKD-------------ITEP---VTVKMQLR---RPSDQ-------EVSE-PMD-FRY  270
_NWU45160.1_       235  FRTP------PFLRD-------------ITEP---VTVKMQLR---RPSDQ-------EVSE-PMD-FRY  270
_NXQ43004.1_       235  FRTP------PFLRD-------------ITEP---VTVKMQLR---RPSDQ-------EVSE-PMD-FRY  270
_XP_027549142.1_   235  FRTP------PFLKD-------------ITEP---VTVKMQLR---RPSDQ-------EVSE-PMD-FRY  270
_XP_017933082.2_   235  FRTP------PFLKD-------------ITEP---VTVKMQLR---RPSDQ-------EVSE-PMD-FRY  270
_NWV96326.1_       235  FRTP------PFLKD-------------ITEP---VTVKMQLR---RPSDQ-------EVSE-PMD-FRY  270
_NXP57783.1_       235  FRTP------PFLRD-------------ITEP---VTVKMQLR---RPSDQ-------EVSE-PMD-FRY  270
_NXY57955.1_       235  FRTP------PFLRD-------------ITEP---VTVKMQLR---RPSDQ-------EVSE-PMD-FRY  270
_AAA99433.1_       235  FRTP------PFLRD-------------ITEP---ITVKMQLR---RPSDQ-------EVSE-PMD-FRY  270
_NXY21218.1_       235  FRTP------PFLKD-------------ITEP---VTVKMQLR---RPSDQ-------EVSE-PMD-FRY  270
```

```
_NXX50824.1_       235  FKTP------PFLKD-------------ITEP---VTVKMQLR---RPSDQ-------EVSE-PMD-FRY  270
_XP_030124623.3_   235  FRTP------PFLRD-------------ISEP---VTVKMQLR---RPSDQ-------EVSE-PMD-FRY  270
_NXA92235.1_       235  FRTP------PFLRD-------------ITEP---VTVKMQLR---RPSDQ-------EVSE-PMD-FRY  270
_NWI05522.1_       235  FRTP------PFLRD-------------ITEP---VTVKMQLR---RPSDQ-------EVSE-PMD-FRY  270
_NWX29009.1_       235  FRTP------PFLRD-------------ITEP---VTVKMQLR---RPSDQ-------EVSE-PMD-FRY  270
_XP_027549144.1_   235  FRTP------PFLKD-------------ITEP---VTVKMQLR---RPSDQ-------EVSE-PMD-FRY  270
_NWW35420.1_       235  FRTP------PFLRD-------------ITEP---VTVKMQLR---RPSDQ-------EVSE-PMD-FRY  270
_NWV07969.1_       235  FRTP------PFLKD-------------ITEP---VTVKMQLR---RPSDQ-------EVSE-PMD-FRY  270
_NXH42214.1_       235  FRTP------PFLRD-------------ITEP---VTVKMQLR---RPSDQ-------EVSE-PMD-FRY  270
_NXQ18773.1_       235  FRTP------PFLRD-------------ISEP---ITVKMQLR---RPSDQ-------EVSE-PMD-FRY  270
_NWX10672.1_       235  FRTP------PFLRD-------------ITEP---VTVKMQLR---RPSDQ-------EVSE-PMD-FRY  270
_NXU50687.1_       235  FRTP------PFLRD-------------ITEP---VTVKMQLR---RPSDQ-------EVSE-PMD-FRY  270
_KFU84784.1_       235  FRTP------PFQRD-------------ITEP---VTVKMQLR---RPSDQ-------EVSE-PMD-FRY  270
_NXQ52438.1_       235  FRTP------PFLRD-------------ITEP---VTVKMQLR---RPSDQ-------EVSE-PMD-FRY  270
_NWW24536.1_       235  FRTP------PFLRD-------------ITEP---VTVKMQLR---RPSDQ-------EVSE-PMD-FRY  270
_NXF73449.1_       235  FRTP------PFLKD-------------ITEP---VTVKMQLR---RPSDQ-------EVSE-PMD-FRY  270
_XP_050827906.1_   235  FRTP------PFLRD-------------ISEP---VTVKMQLR---RPSDQ-------EVSE-PMD-FRY  270
_NWX65205.1_       235  FRTP------PFLRD-------------ITEP---VTVKMQLR---RPSDQ-------EVSE-PMD-FRY  270
_KFW81749.1_       235  FRTP------PFLKD-------------ITEP---VTVKMQLR---RPSDQ-------EVSE-PMD-FRY  270
_XP_014750593.1_   235  FRTP------PFLRD-------------ITEP---VTVKMQLR---RPSDQ-------EVSE-PMD-FRY  270
_NXJ17066.1_       235  FRTP------PFLKD-------------ITEP---VTVKMQLR---RPSDQ-------EVSE-PMD-FRY  270
_NWW67019.1_       235  FRTP------PFLKD-------------ITEP---VTVKMQLR---RPSDQ-------EVSE-PMD-FRY  270
_NXH03360.1_       235  FRTP------PFLRD-------------ISEP---VTVKMQLR---RPSDQ-------EVSE-PMD-FRY  270
_XP_037987525.1_   235  FRTP------PFLRD-------------ISEP---VTVKMQLR---RPSDQ-------EVSE-PMD-FRY  270
_NXL74522.1_       235  FRTP------PFLRD-------------ITEP---VTVKMQLR---RPSDQ-------EVSE-PMD-FRY  270
_NWQ60824.1_       235  FRTP------PFLKD-------------ITEP---VTVKMQLR---RPSDQ-------EVSE-PMD-FRY  270
_NXR70971.1_       235  FRTP------PFLRD-------------ITEP---VTVKMQLR---RPSDQ-------EVSE-PMD-FRY  270
_XP_051468947.1_   235  FRTP------PFQRD-------------ITEP---ITVKMQLR---RPSDQ-------EVSE-PMD-FRY  270
_NXB50199.1_       235  FRTP------PFLRD-------------ITEP---VTVKMQLR---RPSDQ-------EVSE-PMD-FRY  270
_NXP18380.1_       235  FRTP------PFLKD-------------ISEP---VTVKMQLR---RPSDQ-------EVSE-PMD-FRY  270
_NXI84319.1_       235  FKTP------PFLRD-------------ITEP---VTVKMQLR---RPSDQ-------EVSE-PMD-FRY  270
_NWH33757.1_       235  FRTP------PFLRD-------------ITEP---VTVKMQLR---RPSDQ-------EVSE-PMD-FRY  270
_NWR99781.1_       235  FRTP------PFLRD-------------ISEP---VTVKMQLR---RPSDQ-------EVSE-PMD-FRY  270
_NXW94404.1_       235  FRTP------PFLRD-------------ITEP---VTVKMQLR---RPSDQ-------EVSE-PMD-FRY  270
_CAA27220.1_       235  FRTP------PFLRD-------------ITEP---ITVKMQLR---RPSDQ-------EVSE-PVD-FRY  270
_XP_015477679.1_   235  FRTP------PFLRD-------------ITEP---VTVKMQLR---RPSDQ-------EVSE-PMD-FRY  270
_NWS32597.1_       235  FRTP------PFLRD-------------ISEP---VTVKMQLR---RPSDQ-------EVSE-PMD-FRY  270
_NXG22126.1_       235  FRTP------PFLKD-------------ITEP---VTVKMQLR---RPSDQ-------EVSE-PMD-FRY  270
_NWY97127.1_       235  FRTP------PFLRD-------------ISEP---VTVKMQLR---RPSDQ-------EVSE-PMD-FRY  270
```

```
_NXW80148.1_       235  FRTP------PFLRD-------------ITEP---VTVKMQLR---RPSDQ-------EVSE-PMD-FRY  270
_NXC27367.1_       235  FRTP------PFLKD-------------ITEP---VTVKMQLR---RPSDQ-------EVSE-PMD-FRY  270
_NWH83726.1_       235  FRTP------PFLRD-------------ITEP---VTVKMQLR---RPSDQ-------EVSE-PMD-FRY  270
_NXA73512.1_       235  FRTP------PFLRD-------------ITEP---VTVKMQLR---RPSDQ-------EVSE-PMD-FRY  270
_NWZ83230.1_       235  FRTP------PFLRD-------------ITEP---VTVKMQLR---RPSDQ-------EVSE-PMD-FRY  270
_XP_005525572.2_   235  FRTP------PFLRD-------------ITEP---VTVKMQLR---RPSDQ-------EVSE-PMD-FRY  270
_NXD20033.1_       235  FRTP------PFLRD-------------ISEP---VTVKMQLR---RPSDQ-------EVSE-PMD-FRY  270
_NXE37177.1_       235  FRTP------PFLKD-------------ITEP---VTVKMQLR---RPSDQ-------EVSE-PMD-FRY  270
_NWV44369.1_       235  FRTP------PFLRD-------------ITEP---VTVKMQLR---RPSDQ-------EVSE-PMD-FRY  270
_NXC81400.1_       235  FRTP------PFLRD-------------ITEP---VTVKMQLR---RPSDQ-------EVSE-PMD-FRY  270
_NXA67345.1_       235  FRTP------PFLKD-------------ITEP---VTVKMQLR---RPSDQ-------EVSE-PMD-FRY  270
_NXB55155.1_       235  FRTP------PFLKD-------------ITEP---VTVKMQLR---RPSDQ-------EVSE-PMD-FRY  270
_NXN81229.1_       235  FRTP------PFLRD-------------ITEP---VTVKMQLR---RPSDQ-------EVSE-PMD-FRY  270
_NXM15601.1_       235  FRTP------PFLRD-------------ISEP---VTVKMQLR---RPSDQ-------EVSE-PMD-FRY  270
_XP_054484850.1_   235  FRTP------PFLRD-------------ISEP---VTVKMQLR---RPSDQ-------EVSE-PMD-FRY  270
_NWU02685.1_       235  FRTP------PFLRD-------------ISEP---VTVKMQLR---RPSDQ-------EVSE-PMD-FRY  270
_XP_041273165.1_   235  FRTP------PFLRD-------------ISEP---VTVKMQLR---RPSDQ-------EVSE-PMD-FRY  270
_NWW80397.1_       235  FRTP------PFLRD-------------ITEP---VTVKMQLR---RPSDQ-------EVSE-PMD-FRY  270
_XP_036236540.1_   235  FRTP------PFLRD-------------ISEP---VTVKMQLR---RPSDQ-------EVSE-PMD-FRY  270
_NWV50957.1_       235  FRTP------PFLKD-------------ITEP---VTVKMQLR---RPSDQ-------EVSE-PMD-FRY  270
_NXT97287.1_       235  FRTP------PFLRD-------------ITEP---VTVKMQLR---RPSDQ-------EVSE-PMD-FRY  270
_XP_023778876.1_   235  FRTP------PFLRD-------------ISEP---VTVKMQLR---RPSDQ-------EVSE-PMD-FRY  270
_NXD42381.1_       235  FRTP------PFLRD-------------ITEP---VTVKMQLR---RPSDQ-------EVSE-PMD-FRY  270
_NXK69295.1_       235  FRTP------PFLRD-------------ITEP---VTVKMQLR---RPSDQ-------EVSE-PMD-FRY  270
_XP_049657510.1_   235  FRTP------PFLKD-------------ITEP---VTVKMQLR---RPSDQ-------EVSE-PMD-FRY  270
_NXV62539.1_       235  FRTP------PFLRD-------------ISEP---VTVKMQLR---RPSDQ-------EVSE-PMD-FRY  270
_NXB80231.1_       235  FRTP------PFLRD-------------ITEP---VTVKMQLR---RPSDQ-------EVSE-PMD-FRY  270
_NWU15026.1_       235  FRTP------PFLKD-------------ITEP---VTVKMQLR---RPSDQ-------EVSE-PMD-FRY  270
_NXL26104.1_       235  FRTP------PFLRD-------------ISEP---VTVKMQLR---RPSDQ-------EVSE-PMD-FRY  270
_NWW05001.1_       235  FRTP------PFLRD-------------ITEP---VTVKMQLR---RPSDQ-------EVSE-PMD-FRY  270
_RLW07261.1_       235  FRTP------PFLRD-------------ISEP---VTVKMQLR---RPSDQ-------EVSE-PMD-FRY  270
_NXR96625.1_       235  FRTP------PFLRD-------------ISEP---VTVKMQLR---RPSDQ-------EVSE-PMD-FRY  270
_XP_021383204.1_   235  FRTP------PFLRD-------------ISEP---VTVKMQLR---RPSDQ-------EVSE-PMD-FRY  270
_NXQ25764.1_       235  FRTP------PFLRD-------------ITEP---VTVKMQLR---RPSDQ-------EVSE-PMD-FRY  270
_NXO08412.1_       235  FKTP------PFLRD-------------ITEP---VTVKMQLR---RPSDQ-------EVSE-PMD-FRY  270
_NXI32280.1_       235  FRTP------PFLRD-------------ITEP---VTVKMQLR---RPSDQ-------EVSE-PMD-FRY  270
_NXG00288.1_       235  FRTP------PFLRD-------------ISEP---VTVKMQLR---RPSDQ-------EVSE-PMD-FRY  270
_XP_041329879.1_   235  FRTP------PFLRD-------------ISEP---VTVKMQLR---RPSDQ-------EVSE-PMD-FRY  270
_NXV03759.1_       235  FRTP------PFLRD-------------ISEP---VTVKMQLR---RPSDQ-------EVSE-PMD-FRY  270
```

```
_NWT16975.1_      235  FRTP------PFLRD-------------ITEP---VTVKMQLR---RPSDQ-------EVSE-PMD-FRY  270
_NWT67953.1_      235  FRTP------PFLRD-------------ISEP---VTVKMQLR---RPSDQ-------EVSE-PMD-FRY  270
_XP_005042765.2_  235  FRTP------PFLRD-------------ITEP---VTVKMQLR---RPSDQ-------EVSE-PMD-FRY  270
_XP_017600715.1_  235  FRTP------PFLKD-------------ITEP---VTVKMQLR---RPSDQ-------EVSE-PMD-FRY  270
_NXD93279.1_      235  FRTP------PFLRD-------------ITEP---VTVKMQLR---RPSDQ-------EVSE-PMD-FRY  270
_NXY02260.1_      235  FRTP------PFLKD-------------ITEP---VTVKMQLR---RPSDQ-------EVSE-PMD-FRY  270
_NXO84677.1_      235  FRTP------PFLRD-------------ITEP---VTVKMQLR---RPSDQ-------EVSE-PMD-FRY  270
_NXO64371.1_      235  FRTP------PFLRD-------------ITEP---VTVKMQLR---RPSDQ-------EVSE-PMD-FRY  270
_NXO42022.1_      235  FRTP------PFLRD-------------ISEP---VTVKMQLR---RPSDQ-------EVSE-PMD-FRY  270
_NWY65013.1_      235  FRTP------PFLRD-------------ITEP---VTVKMQLR---RPSDQ-------EVSE-PMD-FRY  270
_NXP90563.1_      235  FRTP------PFLRD-------------ISEP---VTVKMQLR---RPSDQ-------EVSE-PMD-FRY  270
_NWI73723.1_      235  FRTP------PFLKD-------------ITEP---VTVKMQLR---RPSDQ-------EVSE-PMD-FRY  270
_NXH30511.1_      235  FRTP------PFLRD-------------ITEP---VTVKMQLR---RPSDQ-------EVSE-PMD-FRY  270
_NWT77800.1_      235  FRTP------PFLKD-------------ITEP---VTVKMQLR---RPSDQ-------EVSE-PMD-FRY  270
_NWT34209.1_      235  FRTP------PFLRD-------------ISEP---VTVKMQLR---RPSDQ-------EVSE-PMD-FRY  270
_NWY29369.1_      235  FRTP------PFLRD-------------ISEP---VTVKMQLR---RPSDQ-------EVSE-PMD-FRY  270
_NXQ73821.1_      235  FRTP------PFLRD-------------ISEP---VTVKMQLR---RPSDQ-------EVSE-PMD-FRY  270
_XP_039572786.1_  235  FRTP------PFLRD-------------ISEP---VTVKMQLR---RPSDQ-------EVSE-PMD-FRY  270
_XP_030802035.1_  235  FRTP------PFLRD-------------ISEP---VTVKMQLR---RPSDQ-------EVSE-PMD-FRY  270
_NXE68211.1_      235  FRTP------PFLRD-------------ISEP---VTVKMQLR---RPSDQ-------EVSE-PMD-FRY  270
_NXS80652.1_      235  FRTP------PFLRD-------------ITEP---VTVKMQLR---RPSDQ-------EVSE-PMD-FRY  270
_NXO32431.1_      235  FRTP------PFLRD-------------ITEP---VTVKMQLR---RPSDQ-------EVSE-PMD-FRY  270
_NWR17317.1_      235  FRTP------PFLRD-------------ISEP---VTVKMQLR---RPSDQ-------EVSE-PMD-FRY  270
_NWZ89732.1_      235  FRTP------PFLRD-------------ISEP---VTVKMQLR---RPSDQ-------EVSE-PMD-FRY  270
_XP_032039523.1_  235  FRTP------PFLKD-------------ITEP---VTVKMQLR---RPSDQ-------EVSE-PMD-FRY  270
_KFO04362.1_      235  FRTP------PFLKD-------------ITEP---VTVKMQLR---RPSDQ-------EVSE-PMD-FRY  270
_XP_054146213.1_  235  FRTP------PFLRD-------------ISEP---VTVKMQLR---RPSDQ-------EVSE-PMD-FRY  270
_XP_005486916.2_  235  FRTP------PFLRD-------------ISEP---VTVKMQLR---RPSDQ-------EVSE-PMD-FRY  270
_KAJ7422946.1_    235  FRTP------PFLRD-------------ISEP---VTVKMQLR---RPSDQ-------EVSE-PMD-FRY  270
_NXC99388.1_      235  FRTP------PFLRD-------------ITEP---VTVKMQLR---RPSDQ-------EVSE-PMD-FRY  270
_RMC07975.1_      235  FRTP------PFLRD-------------ITEP---VTVKMQLR---RPSDQ-------EVSE-PMD-FRY  270
_NXT95159.1_      193  FRTP------PFLKD-------------ITEP---VTVKMQLR---RPSDQ-------EVSE-PMD-FRY  228
_XP_009957589.1_  193  FRTP------PFLKD-------------ITEP---VTVKMQLR---RPSDQ-------EVSE-PMD-FRY  228
_TRZ13602.1_      193  FRTP------PFLRD-------------ITEP---VTVKMQLR---RPSDQ-------EVSE-PMD-FRY  228
_NWR45911.1_      184  FRTP------PFLRD-------------ITEP---VTVKMQLR---RPSDQ-------EVSE-PMD-FRY  219
_KAF4799649.1_    193  FRTP------PFLRD-------------ITEP---VTVKMQLR---RPSDQ-------EVSE-PMD-FRY  228
_KAJ7420138.1_    229  FRTP------PFLKD-------------ITEP---VTVKMQLR---RPSDQ-------EVSE-PMD-FRY  264
_XP_054678501.1_  201  FRTP------PFLKD-------------ITEP---VTVKMQLR---RPSDQ-------EVSE-PMD-FRY  236
_NXP48685.1_           ----------------------------------------------------------------------
```

```
_XP_010704701.1_  152  FRTP------PFLRD-------------ITEP---ITVKMQLR---RPSDQ-------EVSE-PMD-FRY  187
_KFV10805.1_      144  FRTP------PFLKD-------------ITEP---ITVKMQLR---RPSDQ-------EVSE-PMD-FRY  179
_XP_009572567.1_  114  FRTP------PFLKD-------------ITEP---VTVKMQLR---RPSDQ-------EVSE-PMD-FRY  149
_NXF64277.1_      113  FRTP------PFLKD-------------ITEP---VTVKMQLR---RPSDQ-------EVSE-PMD-FRY  148
_KFV54465.1_      113  FRTP------PFLKD-------------ITEP---VTVKMQLR---RPSDQ-------EVSE-PMD-FRY  148
_KFV90717.1_      113  FRTP------PFLKD-------------ITEP---VTVKMQLR---RPSDQ-------EVSE-PMD-FRY  148
_KFQ03674.1_      113  FRTP------PFLKD-------------ITEP---VTVKMQLR---RPSDQ-------EVSE-PMD-FRY  148
_NXW01028.1_      113  FRTP------PFLKD-------------ITEP---VTVKMQLR---RPSDQ-------EVSE-PMD-FRY  148
_KFP32908.1_      113  FRTP------PFLKD-------------ITEP---VTVKMQLR---RPSDQ-------EVSE-PMD-FRY  148
_KFV93389.1_      113  FRTP------PFLKD-------------ITEP---VTVKMQLR---RPSDQ-------EVSE-PMD-FRY  148
_NWI63728.1_      113  FRTP------PFLKD-------------ITEP---VTVKMQLR---RPSDQ-------EVSE-PMD-FRY  148
_KAI6072063.1_    114  FRTP------PFLKD-------------ITEP---VTVKMQLR---RPSDQ-------EVSE-PMD-FRY  149
_KFQ58901.1_      113  FRTP------PFLKD-------------ITEP---VTVKMQLR---RPSDQ-------EVSE-PMD-FRY  148
_NXP33753.1_      113  FRTP------PFLRD-------------ITEP---VTVKMQLR---RPSDQ-------EVSE-PMD-FRY  148
_XP_009476353.1_  112  FRTP------PFLKD-------------ITEP---VTVKMQLR---RPSDQ-------EVSE-PMD-FRY  147
_KFQ55903.1_      113  FRTP------PFLRD-------------ITEP---VTVKMQLR---RPSDQ-------EVSE-PMD-FRY  148
_XP_042683349.1_  113  FRTP------PFLRD-------------ITEP---ITVKMQLR---RPSDQ-------EVSE-PMD-FRY  148
_NXR08991.1_      113  FKTP------PFLKD-------------ITEP---VTVKMQLR---RPSDQ-------EVSE-PMD-FRY  148
_XP_008947250.1_  113  FRTP------PFLRD-------------ITEP---VTVKMQLR---RPSDQ-------EVSE-PMD-FRY  148
_KFP72464.1_      113  FRTP------PFLKD-------------ITEP---VTVKMQLR---RPSDQ-------EVSE-PMD-FRY  148
_KFV60523.1_      113  FRTP------PFLKD-------------ITEP---VTVKMQLR---RPSDQ-------EVSE-PMD-FRY  148
_NXT13165.1_      113  FRTP------PFLRD-------------ISEP---ITVKMQLR---RPSDQ-------EVSE-PMD-FRY  148
_NWT50506.1_      113  FRTP------PFLRD-------------ITEP---VTVKMQLR---RPSDQ-------EVSE-PMD-FRY  148
_OXB76139.1_      111  FRTP------PFLRD-------------ITEP---VTVKMQLR---RPSDQ-------EVSE-PMD-FRY  146
_XP_050827907.1_  126  FRTP------PFLRD-------------ISEP---VTVKMQLR---RPSDQ-------EVSE-PMD-FRY  161
_XP_030302545.1_  121  FRTP------PFLRD-------------ITEP---VTVKMQLR---RPSDQ-------EVSE-PMD-FRY  156
_XP_054036038.1_  125  FRTP------PFLKD-------------ITEP---VTVKMQLR---RPSDQ-------EVSE-PMD-FRY  160
_XP_012788337.1_  235  FKTP------PYCKP-------------IMEP---VTVKMQLR---RPSDQ-------EVSE-SMD-FRY  270
_XP_008070154.1_  224  FKTP------PYCKA-------------IMEP---VTVKMQLR---RPSDQ-------EVSE-SMD-FRY  259
_XP_027477445.1_  224  FKTP------PYCRV-------------ITEP---VTVKMQLR---RPSDQ-------EVSE-SMD-FRY  259
_XP_025848473.1_  224  FKTP------PYCRA-------------ITEP---VTVKMQLR---RPSDQ-------EVSE-SMD-FRY  259
_XP_049640716.1_  235  FKTP------PYCKP-------------IMEP---VTVKMQLR---RPSDQ-------EVSE-SMD-FRY  270
_XP_023084089.1_  235  FKTP------PYCKA-------------ITEP---VTVKMQLR---RPSDQ-------EVSE-SMD-FRY  270
_EHH22143.1_      235  FKTP------PYCKA-------------ITEP---VTVKMQLR---RPSDQ-------EVSE-SMD-FRY  270
_XP_038504711.1_  235  FKTP------PYCRA-------------ITEP---VTVKMQLR---RPSDQ-------EVSE-SMD-FRY  270
_XP_038504703.1_  235  FKTP------PYCRA-------------ITEP---VTVKMQLR---RPSDQ-------EVSE-SMD-FRY  270
_XP_038504718.1_  235  FKTP------PYCRA-------------ITEP---VTVKMQLR---RPSDQ-------EVSE-SMD-FRY  270
_XP_048971856.1_  235  FKTP------PYCRA-------------ITEP---VTVKMQLR---RPSDQ-------EVSE-SMD-FRY  270
_KAB1266826.1_    235  FKTP------PYCKA-------------ITEP---VTVKMQLR---RPSDQ-------EVSE-SMD-FRY  270
```

```
_XP_048391424.1_  235  FRTP------AYCNTN------------ITEP---VSVKMQLR---RPSDQ-------QVSE-SME-FRY  271
_XP_048391425.1_  235  FRTP------AYCNTN------------ITEP---VSVKMQLR---RPSDQ-------QVSE-SME-FRY  271
_GCC28026.1_      235  FRTP------AYCKTN------------ITEP---VSVKMQLR---RPSDQ-------QVSE-SME-FRY  271
_XP_043551991.1_  235  FRTP------AYCKTN------------VTEP---VSVKMQLR---RPSDQ-------QVSE-SME-FRY  271
_XP_043551989.1_  235  FRTP------AYCKTN------------VTEP---VSVKMQLR---RPSDQ-------QVSE-SME-FRY  271
_XP_054534389.1_  145  FKTP------PYCKA-------------ITEP---VTVKMQLR---RPSDQ-------EVSE-SMD-FRY  180
_XP_011531312.1_  145  FKTP------PYCKA-------------ITEP---VTVKMQLR---RPSDQ-------EVSE-SMD-FRY  180
_XP_021780541.1_  144  FKTP------PYCKA-------------ITEP---VTVKMQLR---RPSDQ-------EVSE-SMD-FRY  179
_XP_037862912.1_  165  FKTP------PYCKA-------------ITEP---VTVKMQLR---RPSDQ-------EVSE-SMD-FRY  200
_XP_015413428.1_  224  FKTP------PYCKV-------------ITEP---VTVKMQLR---RPSDQ-------EVSE-SMD-FRY  259
_XP_005884642.1_  224  FKTP------PYCKV-------------ITEP---VTVKMQLR---RPSDQ-------EVSE-SMD-FRY  259
_KAF6105903.1_    224  FKTP------PYCKA-------------ITEP---VTVKMQLR---RPSDQ-------EVSE-SMD-FRY  259
_XP_015413426.1_  224  FKTP------PYCKV-------------ITEP---VTVKMQLR---RPSDQ-------EVSE-SMD-FRY  259
_XP_014387539.1_  224  FKTP------PYCKV-------------ITEP---VTVKMQLR---RPSDQ-------EVSE-SMD-FRY  259
_XP_014444723.1_  224  FKTP------PYCKA-------------ITEP---VTVKMQLR---RPSDQ-------EVSE-SMD-FRY  259
_XP_023448058.1_  224  FKTP------PYCKA-------------ITEP---VTVKMQLR---RPSDQ-------EVSE-SMD-FRY  259
_EGW11604.1_      224  FKTP------PYCKA-------------ILEP---VTVKMQLR---RPSDQ-------EVSE-SMD-FRY  259
_XP_015985129.1_  224  FKTP------PYCKA-------------ITEP---VTVKMQLR---RPSDQ-------EVSE-SMD-FRY  259
_XP_012878014.1_  224  FKTP------PYCKV-------------ISEP---VTVKMQLR---RPSDQ-------EVSK-SMD-FRY  259
_KAG5211506.1_    203  FKTP------PYCKA-------------ITEP---VTVKMQLR---RPSDQ-------EVSE-SMD-FRY  238
_MXQ82539.1_      199  FKTP------PYCKA-------------IIEP---VTVKMQLR---RPSDQ-------EVSE-SMD-FRY  234
_XP_032729748.1_  188  FKTP------PYCRA-------------ITEP---VTVKMQLR---RPSDQ-------EVSE-SMD-FRY  223
_XP_032208113.1_  188  FKTP------PYCRA-------------ITEP---VTVKMQLR---RPSDQ-------EVSE-SMD-FRY  223
_XP_048208817.1_  200  FKTP------PFCKV-------------ISEP---VTVKMQLR---RPSDQ-------EVSK-SMD-FRY  235
_XP_038200333.1_  200  FKTP------PYCKA-------------ILEP---VTVKMQLR---RPSDQ-------EVSE-SMD-FRY  235
_P15307.2_        235  FKTP------PYCKA-------------ILEP---VTVKMQLR---RPSDQ-------EVSE-SMD-FRY  270
_XP_041524635.1_  224  FKTP------PYCKA-------------ILEP---VTVKMQLR---RPSDQ-------EVSE-SMD-FRY  259
_CAA33843.1_      233  FKTP------PYCKA-------------ILEP---VTVKMQLR---RPSDQ-------EVSE-SMD-FRY  268
_XP_028732210.2_  224  FKTP------PYCKA-------------ILEP---VTVRMQLR---RPSDQ-------EVSE-SMD-FRY  259
_XP_025848474.1_  224  FKTP------PYCRA-------------ITEP---VTVKMQLR---RPSDQ-------EVSE-SMD-FRY  259
_EDL97996.1_      224  FRTP------PYCRA-------------IVEP---VTVKMQLR---RPSDQ-------EVSE-SMD-FRY  259
_KAI4577886.1_    224  FKTP------PYCKA-------------ITEP---VTVKMQLR---RPSDQ-------EVSE-SMD-FRY  259
_KAG8512309.1_    207  FKTP------PYCKT-------------IMEP---VTVKMQLR---RPSDQ-------EVSE-SMD-FRY  242
_XP_047637971.1_  200  FKTP------PYCKA-------------IIEP---VTVKMQLR---RPSDQ-------EVSE-SMD-FRY  235
_XP_019491454.1_  235  FKTP------PYCKA-------------ITEP---VTVKMQLR---RHTYG-------NKAK--------  264
_XP_043842482.1_  237  FRTP------PFYKA-------------ITEP---VTVKMQLR---RPSDQ-------EVSE-SMD-FRY  272
_XP_027721170.1_  237  FRTP------PFYKA-------------ITEP---VTVKMQLR---RPSDQ-------EVSE-SMD-FRY  272
_XP_036604632.1_  237  FRTP------PFYKA-------------ITEP---VTVKMQLR---RPSDQ-------EVSE-SMD-FRY  272
_XP_020841164.1_  237  FRTP------PFYKA-------------ITEP---VTVKMQLR---RPSDQ-------EVSE-SMD-FRY  272
```

```
_XP_051831295.1_  237  FRTP------PFYKE-------------ITEP---ITVKMQLR---RPSDQ-------EVSE-SMD-FRY  272
_XP_031806378.1_  237  FRTP------PFYKE-------------ITEP---ITVKMQLR---RPSDQ-------EVSE-SMD-FRY  272
_XP_051831294.1_  237  FRTP------PFYKE-------------ITEP---ITVKMQLR---RPSDQ-------EVSE-SMD-FRY  272
_XP_003339776.1_  237  FRTP------PFYKA-------------ITEP---VTVKMQLR---RPSDQ-------EVSE-SMD-FRY  272
_XP_044515413.1_  237  FRTP------PFYKA-------------ITEP---VTVKMQLR---RPSDQ-------EVSE-SMD-FRY  272
_XP_028928361.1_  237  FRTP------PYNKV-------------ITEP---VTVKMQLR---RPSDQ-------EVSD-PMD-FRY  272
_XP_038607403.1_  237  FRTP------PYNKV-------------ITEP---VTVKMQLR---RPSDQ-------EVSD-PMD-FRY  272
_XP_040849334.1_  113  FKTP------PYCKA-------------ITEP---VTVKMQLR---RPSDQ-------EVSE-SMD-FRY  148
_TKC46270.1_      111  FKTP------PYCKA-------------ITEP---VTVKMQLR---RPSDQ-------EVSE-SMD-FRY  146
_OWK10634.1_      112  FKTP------PYCKA-------------ITEP---VTVKMQLR---RPSDQ-------EVSE-SMD-FRY  147
_XP_036190342.1_  111  FKTP------PYCKV-------------ITEP---VTVKMQLR---RPSDQ-------EVSE-SMD-FRY  146
_KAB0395596.1_    108  FKTP------PYCKA-------------ITEP---VTVKMQLR---RPSDQ-------EVSE-SMD-FRY  143
_KAF6430170.1_    112  FKTP------PYCKA-------------ITDP---VTVKMQLR---RPSDQ-------EVSE-SMD-FRY  147
_XP_038948811.1_  113  FRTP------PYCRA-------------IVEP---VTVKMQLR---RPSDQ-------EVSE-SMD-FRY  148
_KAB1266828.1_    246  FKTP------PYCKA-------------ITEP---VTVKMQLR---RPSDQ-------EVSE-SMD-FRY  281
_NP_002899.1_     235  FKTP------PYCKA-------------ITEP---VTVKMQLR---RPSDQ-------EVSE-SMD-FRY  270
_XP_001159537.3_  235  FKTP------PYCKA-------------ITEP---VTVKMQLR---RPSDQ-------EVSE-SMD-FRY  270
_PNI66333.1_      235  FKTP------PYCKA-------------ITEP---VTVKMQLR---RPSDQ-------EVSE-SMD-FRY  270
_EHH55592.1_      235  FKTP------PYCKA-------------ITEP---VTVKMQLR---RPSDQ-------EVSE-SMD-FRY  270
_XP_003262470.1_  235  FKTP------PYCKA-------------ITEP---VTVKMQLR---RPSDQ-------EVSE-SMD-FRY  270
_BAG64854.1_      235  FKTP------PYCKA-------------ITEP---VTVKMQLR---RPSDQ-------EVSE-SMD-FRY  270
_XP_032034471.1_  235  FKTP------PYCKA-------------ITEP---VTVKMQLR---RPSDQ-------EVSE-SMD-FRY  270
_XP_037862911.1_  235  FKTP------PYCKA-------------ITEP---VTVKMQLR---RPSDQ-------EVSE-SMD-FRY  270
_XP_003830911.1_  235  FKTP------PYCKA-------------ITEP---VTVKMQLR---RPSDQ-------EVSE-SMD-FRY  270
_XP_004029331.1_  235  FKTP------PYCKA-------------ITEP---VTVKMQLR---RPSDQ-------EVSE-SMD-FRY  270
_XP_002812051.1_  235  FKTP------PYCKA-------------ITEP---VTVKMQLR---RPSDQ-------EVSE-SMD-FRY  270
_XP_011711623.1_  235  FKTP------PYCKA-------------ITEP---VTVKMQLR---RPSDQ-------EVSE-SMD-FRY  270
_XP_033095023.1_  235  FKTP------PYCKA-------------ITEP---VTVKMQLR---RPSDQ-------EVSE-SMD-FRY  270
_XP_002799280.1_  235  FKTP------PYCKA-------------ITEP---VTVKMQLR---RPSDQ-------EVSE-SMD-FRY  270
_XP_011853172.1_  235  FKTP------PYCKA-------------ITEP---VTVKMQLR---RPSDQ-------EVSE-SMD-FRY  270
_XP_050609443.1_  235  FKTP------PYCKA-------------ITEP---VTVKMQLR---RPSDQ-------EVSE-SMD-FRY  270
_XP_003908735.1_  235  FKTP------PYCKA-------------ITEP---VTVKMQLR---RPSDQ-------EVSE-SMD-FRY  270
_XP_025209693.1_  235  FKTP------PYCKA-------------ITEP---VTVKMQLR---RPSDQ-------EVSE-SMD-FRY  270
_XP_023084087.1_  235  FKTP------PYCKA-------------ITEP---VTVKMQLR---RPSDQ-------EVSE-SMD-FRY  270
_XP_021780539.1_  235  FKTP------PYCKA-------------ITEP---VTVKMQLR---RPSDQ-------EVSE-SMD-FRY  270
_XP_017704568.1_  235  FKTP------PYCKA-------------ITEP---VTVKMQLR---RPSDQ-------EVSE-SMD-FRY  270
_XP_010375811.1_  235  FKTP------PYCKA-------------ITEP---VTVKMQLR---RPSDQ-------EVSE-SMD-FRY  270
_XP_030776399.1_  235  FKTP------PYCKA-------------ITEP---VTVKMQLR---RPSDQ-------EVSE-SMD-FRY  270
_XP_011797211.1_  235  FKTP------PYCKA-------------ITEP---VTVKMQLR---RPSDQ-------EVSE-SMD-FRY  270
```

```
_XP_012332682.1_   235  FKTP------PYCKV-------------ITEP---VTVKMQLR---RPSDQ-------EVSE-SMD-FRY  270
_XP_010331875.2_   235  FKTP------PYCKT-------------ITEP---VTVKMQLR---RPSDQ-------EVSE-SMD-FRY  270
_XP_012332683.1_   235  FKTP------PYCKV-------------ITEP---VTVKMQLR---RPSDQ-------EVSE-SMD-FRY  270
_XP_017400475.1_   235  FKTP------PYCKT-------------IIEP---VTVKMQLR---RPSDQ-------EVSE-SMD-FRY  270
_XP_008568357.1_   235  FKTP------PYCKA-------------ITEP---VTVKMQLR---RPSDQ-------EVSE-SMD-FRY  270
_XP_012620284.1_   235  FKTP------PYCKA-------------IMEP---VTVKMQLR---RPSDQ-------EVSE-SMD-FRY  270
_XP_002709741.2_   235  FKTP------PYCKA-------------ITEP---VTVKMQLR---RPSDQ-------EVSE-SMD-FRY  270
_XP_022452943.1_   235  FKTP------PYCKA-------------ITEP---VTVKMQLR---RPSDQ-------EVSE-SMD-FRY  270
_XP_045405625.1_   235  FKTP------PYCKA-------------IMEP---VTVKMQLR---RPSDQ-------EVSE-SMD-FRY  270
_XP_004280707.1_   235  FKTP------PYCKA-------------ITEP---VTVKMQLR---RPSDQ-------EVSE-SMD-FRY  270
_XP_030733169.1_   235  FKTP------PYCKA-------------ITEP---VTVKMQLR---RPSDQ-------EVSE-SMD-FRY  270
_TEA23022.1_       235  FKTP------PYCKA-------------ITEP---VTVKMQLR---RPSDQ-------EVSE-SMD-FRY  270
_XP_012494629.1_   235  FKTP------PYCKA-------------IMEP---VTVKMQLR---RPSDQ-------EVSE-SMD-FRY  270
_XP_010950191.1_   235  FKTP------PYCKA-------------ITEP---VTVKMQLR---RPSDQ-------EVSE-SMD-FRY  270
_XP_024608785.1_   235  FKTP------PYCKA-------------ITEP---VTVKMQLR---RPSDQ-------EVSE-SMD-FRY  270
_XP_006201637.1_   235  FKTP------PYCKA-------------ITEP---VTVKMQLR---RPSDQ-------EVSE-SMD-FRY  270
_XP_019285354.1_   235  FKTP------PYCRA-------------ITEP---VTVKMQLR---RPSDQ-------EVSE-SMD-FRY  270
_XP_032509224.1_   235  FKTP------PYCKA-------------ITEP---VTVKMQLR---RPSDQ-------EVSE-SMD-FRY  270
_XP_047379355.1_   235  FKTP------PYCKA-------------ITEP---VTVKMQLR---RPSDQ-------EVSE-SMD-FRY  270
_XP_032981134.1_   235  FKTP------PYCKA-------------ITEP---VTVKMQLR---RPSDQ-------EVSE-SMD-FRY  270
_XP_005600018.1_   235  FKTP------PYCKA-------------ITEP---VTVKMQLR---RPSDQ-------EVSE-SMD-FRY  270
_XP_046517051.1_   235  FKTP------PYCKA-------------ITEP---VTVKMQLR---RPSDQ-------EVSE-SMD-FRY  270
_XP_007464205.1_   235  FKTP------PYCKA-------------ITEP---VTVKMQLR---RPSDQ-------EVSE-SMD-FRY  270
_XP_019572903.1_   235  FKTP------PYCKA-------------ITEP---VTVKMQLR---RPSDQ-------EVSE-SMD-FRY  270
_XP_007189755.1_   235  FKTP------PYCKA-------------ITEP---VTVKMQLR---RPSDQ-------EVSE-SMD-FRY  270
_XP_004397771.1_   235  FKTP------PYCRA-------------ITEP---VTVKMQLR---RPSDQ-------EVSE-SMD-FRY  270
_XP_004435713.1_   236  FKTP------PYCKA-------------ITEP---VTVKMQLR---RPSDQ-------EVSE-SMD-FRY  271
_XP_008698124.1_   235  FKTP------PYCRA-------------ITEP---VTVKMQLR---RPSDQ-------EVSE-SMD-FRY  270
_XP_023989255.1_   235  FKTP------PYCKA-------------ITEP---VTVKMQLR---RPSDQ-------EVSE-SMD-FRY  270
_XP_011233012.2_   235  FKTP------PYCRA-------------ITEP---VTVKMQLR---RPSDQ-------EVSE-SMD-FRY  270
_XP_025724444.1_   235  FKTP------PYCRA-------------ITEP---VTVKMQLR---RPSDQ-------EVSE-SMD-FRY  270
_XP_005626186.1_   235  FKTP------PYCRA-------------ITEP---VTVKMQLR---RPSDQ-------EVSE-SMD-FRY  270
_XP_045643991.1_   235  FKTP------PYCRA-------------ITEP---VTVKMQLR---RPSDQ-------EVSE-SMD-FRY  270
_XP_034857383.1_   235  FKTP------PYCRA-------------ITEP---VTVKMQLR---RPSDQ-------EVSE-SMD-FRY  270
_XP_027945501.1_   235  FKTP------PYCRA-------------ITEP---VTVKMQLR---RPSDQ-------EVSE-SMD-FRY  270
_XP_026341094.1_   235  FKTP------PYCRA-------------ITEP---VTVKMQLR---RPSDQ-------EVSE-SMD-FRY  270
_EFB29923.1_       235  FKTP------PYCRA-------------ITEP---VTVKMQLR---RPSDQ-------EVSE-SMD-FRY  270
_XP_045740897.1_   235  FKTP------PYCRA-------------ITEP---VTVKMQLR---RPSDQ-------EVSE-SMD-FRY  270
_CAD7677454.1_     235  FKTP------PYCRA-------------ITEP---VTVKMQLR---RPSDQ-------EVSE-SMD-FRY  270
```

```
_XP_035577553.1_   235  FKTP------PYCRA-------------ITEP---VTVKMQLR---RPSDQ-------EVSE-SMD-FRY  270
_XP_006733255.1_   235  FKTP------PYCRA-------------ITEP---VTVKMQLR---RPSDQ-------EVSE-SMD-FRY  270
_XP_032266820.1_   235  FKTP------PYCRA-------------ITEP---VTVKMQLR---RPSDQ-------EVSE-SMD-FRY  270
_XP_041611730.1_   235  FKTP------PYCRA-------------ITEP---VTVKMQLR---RPSDQ-------EVSE-SMD-FRY  270
_XP_048971854.1_   235  FKTP------PYCRA-------------ITEP---VTVKMQLR---RPSDQ-------EVSE-SMD-FRY  270
_XP_021554840.1_   235  FKTP------PYCRA-------------ITEP---VTVKMQLR---RPSDQ-------EVSE-SMD-FRY  270
_VCW78562.1_       235  FKTP------PYCRA-------------ITEP---VTVKMQLR---RPSDQ-------EVSE-SMD-FRY  270
_XP_034857381.1_   235  FKTP------PYCRA-------------ITEP---VTVKMQLR---RPSDQ-------EVSE-SMD-FRY  270
_XP_008524231.1_   232  FKTP------PYCKA-------------ITEP---VTVKMQLR---RPSDQ-------EVSE-SMD-FRY  267
_XP_045740895.1_   235  FKTP------PYCRA-------------ITEP---VTVKMQLR---RPSDQ-------EVSE-SMD-FRY  270
_XP_045834746.1_   235  FKTP------PYCRA-------------ITEP---VTVKMQLR---RPSDQ-------EVSE-SMD-FRY  270
_XP_035932906.1_   235  FKTP------PYCRA-------------ITEP---VTVKMQLR---RPSDQ-------EVSE-SMD-FRY  270
_XP_030883859.1_   235  FKTP------PYCRA-------------ITEP---VTVKMQLR---RPSDQ-------EVSE-SMD-FRY  270
_XP_032266818.1_   235  FKTP------PYCRA-------------ITEP---VTVKMQLR---RPSDQ-------EVSE-SMD-FRY  270
_XP_024408528.2_   235  FKTP------PYCKA-------------ITEP---VTVKMQLR---RPSDQ-------EVSE-SMD-FRY  270
_XP_004580347.1_   235  FKTP------PYCKA-------------ITEP---VTVKMQLR---RPSDQ-------EVSE-SMD-FRY  270
_XP_030166525.1_   235  FKTP------PYCRA-------------ITEP---VTVKMQLR---RPSDQ-------EVSE-SMD-FRY  270
_XP_035932904.1_   235  FKTP------PYCRA-------------ITEP---VTVKMQLR---RPSDQ-------EVSE-SMD-FRY  270
_XP_020040690.1_   235  FKTP------PYCKA-------------ISEP---VTVKMQLR---RPSDQ-------EVSE-SMD-FRY  270
_XP_053445604.1_   235  FKTP------PYCKA-------------IMEP---VTVKMHLR---RPSDQ-------EVSE-SMD-FRY  270
_XP_049507482.1_   235  FKTP------PYCRA-------------ITEP---VTVKMQLR---RPSDQ-------EVSE-SMD-FRY  270
_XP_015335127.1_   235  FKTP------PYCKA-------------ITEP---VTVKMQLR---RPSDQ-------EVSE-SMD-FRY  270
_XP_027790245.1_   235  FKTP------PYCKA-------------ITEP---VTVKMQLR---RPSDQ-------EVSE-SMD-FRY  270
_XP_003984077.1_   235  FKTP------PYCRA-------------ITEP---VTVKMQLR---RPSDQ-------EVSE-SMD-FRY  270
_XP_039096259.1_   235  FKTP------PYCRA-------------ITEP---VTVKMQLR---RPSDQ-------EVSE-SMD-FRY  270
_XP_007075434.1_   235  FKTP------PYCRA-------------ITEP---VTVKMQLR---RPSDQ-------EVSE-SMD-FRY  270
_XP_022360130.1_   235  FKTP------PYCRA-------------ITEP---VTVKMQLR---RPSDQ-------EVSE-SMD-FRY  270
_XP_047707378.1_   235  FKTP------PYCRA-------------ITEP---VTVKMQLR---RPSDQ-------EVSE-SMD-FRY  270
_XP_026243031.1_   235  FKTP------PYCKA-------------ITEP---VTVKMQLR---RPSDQ-------EVSE-SMD-FRY  270
_XP_037383340.1_   235  FKTP------PYCKA-------------IMEP---VTVKMQLR---RPSDQ-------EVSE-SMD-FRY  270
_XP_005322095.1_   235  FKTP------PYCKA-------------ITEP---VTVKMQLR---RPSDQ-------EVSE-SMD-FRY  270
_XP_005662584.1_   235  FKTP------PYCKA-------------IIEP---VTVKMQLR---RPSDQ-------EVSE-SMD-FRY  270
_XP_040080101.1_   235  FKTP------PYCKA-------------ITEP---VTVKMQLR---RPSDQ-------EVSE-SMD-FRY  270
_XP_017503999.1_   235  FKTP------PYCKA-------------ITEP---VTVKMQLR---RPSDQ-------EVSE-SMD-FRY  270
_XP_044119954.1_   235  FKTP------PYCRA-------------ITEP---VTVKMQLR---RPSDQ-------EVSE-SMD-FRY  270
_XP_032208110.1_   235  FKTP------PYCRA-------------ITEP---VTVKMQLR---RPSDQ-------EVSE-SMD-FRY  270
_XP_012586936.1_   235  FKTP------PYCKA-------------IVEP---VTVKMQLR---RPSDQ-------EVSE-SMD-FRY  270
_XP_032729745.1_   235  FKTP------PYCRA-------------ITEP---VTVKMQLR---RPSDQ-------EVSE-SMD-FRY  270
_XP_052504199.1_   235  FKTP------PYCKA-------------ITEP---VTVKMQLR---RPSDQ-------EVSE-SMD-FRY  270
```

```
_XP_054431389.1_  235  FKTP------PYCKA-------------ITEP---VTVKMQLR---RPSDQ-------EVSE-SMD-FRY  270
_XP_023576632.1_  235  FKTP------PYCKA-------------IAEP---VTVQMQLR---RPSDQ-------EVSE-SMD-FRY  270
_XP_004742070.1_  235  FKTP------PYCRA-------------ITEP---VTVKMQLR---RPSDQ-------EVSE-SMD-FRY  270
_XP_037663267.1_  235  FKTP------PYCKA-------------IMEP---VTVKMQLR---RPSDQ-------EVSE-SMD-FRY  270
_XP_004005978.1_  235  FKTP------PYCKA-------------ITEP---VTVKMQLR---RPSDQ-------EVSE-SMD-FRY  270
_XP_036781908.1_  235  FKTP------PYCKA-------------ITEP---VTVKMQLR---RPSDQ-------EVSE-SMD-FRY  270
_XP_040849333.1_  235  FKTP------PYCKA-------------ITEP---VTVKMQLR---RPSDQ-------EVSE-SMD-FRY  270
_XP_005686691.1_  235  FKTP------PYCKA-------------ITEP---VTVKMQLR---RPSDQ-------EVSE-SMD-FRY  270
_XP_023367900.1_  235  FKTP------PYSKA-------------ILEP---VTVKMHLR---RPSDQ-------EVSE-SMD-FRY  270
_KAF0879576.1_    235  FKTP------PYCRA-------------ITEP---VTVKMQLR---RPSDQ-------EVSE-SMD-FRY  270
_XP_029793284.1_  235  FKTP------PYCRD-------------ITEP---VTVKMQLR---RPSDQ-------EVSE-SMD-FRY  270
_NP_001179899.1_  235  FKTP------PYCKA-------------IIEP---VTVKMQLR---RPSDQ-------EVSE-SMD-FRY  270
_XP_020764718.1_  235  FKTP------PYCKA-------------ITEP---VTVKMQLR---RPSDQ-------EVSE-SMD-FRY  270
_XP_054431388.1_  235  FKTP------PYCKA-------------ITEP---VTVKMQLR---RPSDQ-------EVSE-SMD-FRY  270
_XP_037663265.1_  235  FKTP------PYCKA-------------IMEP---VTVKMQLR---RPSDQ-------EVSE-SMD-FRY  270
_XP_019825763.1_  235  FKTP------PYCKA-------------IIEP---VTVKMQLR---RPSDQ-------EVSE-SMD-FRY  270
_XP_005889096.1_  235  FKTP------PYCKA-------------IIEP---VTVKMQLR---RPSDQ-------EVSE-SMD-FRY  270
_XP_043324571.1_  235  FKTP------PYCKA-------------ITEP---VTVKMQLR---RPSDQ-------EVSE-SMD-FRY  270
_KAJ1078272.1_    235  FKTP------PYCKA-------------ITEP---VTVKMQLR---RPSDQ-------EVSE-SMD-FRY  270
_XP_036190339.1_  235  FKTP------PYCKV-------------ITEP---VTVKMQLR---RPSDQ-------EVSE-SMD-FRY  270
_XP_013005522.1_  235  FKTP------PYCKA-------------ITEP---VTVKMQLR---RPSDQ-------EVSE-SMD-FRY  270
_XP_006073936.1_  235  FKTP------PYCKA-------------ITEP---VTVKMQLR---RPSDQ-------EVSE-SMD-FRY  270
_XP_016069529.1_  235  FKTP------PYCKA-------------ITEP---VTVKMHLR---RPSDQ-------EVSE-SMD-FRY  270
_KAB0354136.1_    235  FKTP------PYCKA-------------ITEP---VTVKMQLR---RPSDQ-------EVSE-SMD-FRY  270
_XP_029424871.1_  235  FKTP------PYCRA-------------ILEP---VTVKMQLR---RPSDQ-------EVSE-SMD-FRY  270
_XP_036118792.1_  235  FKTP------PYCKA-------------ITDP---VTVKMQLR---RPSDQ-------EVSE-SMD-FRY  270
_XP_037019725.2_  234  FKTP------PYCKA-------------ITEP---VTVKMQLR---RPSDQ-------EVSE-SMD-FRY  269
_XP_047602572.1_  235  FKTP------PYCRA-------------ITEP---VTVKMQLR---RPSDQ-------EVSE-SMD-FRY  270
_XP_014586639.1_  235  FKTP------PYCKA-------------ITEP---VTVKMQLR---RPSDQ-------EVSE-SMD-FRY  270
_XP_046517052.1_  235  FKTP------PYCKA-------------ITEP---VTVKMQLR---RPSDQ-------EVSE-SMD-FRY  270
_XP_006909635.2_  235  FKTP------PYCKA-------------ITEP---VTVKMQLR---RPSDQ-------EVSE-SMD-FRY  270
_XP_011362222.1_  235  FKTP------PYCKA-------------ITEP---VTVKMQLR---RPSDQ-------EVSE-SMD-FRY  270
_XP_045684409.1_  235  FKTP------PYCKA-------------ITEP---VTVKMQLR---RPSDQ-------EVSE-SMD-FRY  270
_XP_048208816.1_  235  FKTP------PFCKV-------------ISEP---VTVKMQLR---RPSDQ-------EVSK-SMD-FRY  270
_XP_052590629.1_  235  FKTP------PYCKA-------------ILEP---VTVRMQLR---RPSDQ-------EVSE-SMD-FRY  270
_XP_032353499.1_  235  FKTP------PYCKA-------------ITEP---VTVKMQLR---RPSDQ-------EVSE-SMD-FRY  270
_XP_038200327.1_  235  FKTP------PYCKA-------------ILEP---VTVKMQLR---RPSDQ-------EVSE-SMD-FRY  270
_XP_051056550.1_  235  FKTP------PYCKA-------------ILEP---VTVKMQLR---RPSDQ-------EVSE-SMD-FRY  270
_XP_012805850.2_  235  FKTP------PYCKA-------------ISEP---VTVKMQLR---RPSDQ-------EVSE-SMD-FRY  270
```

_XP_016050906.1_	235	FKTP------PYCKA-------------IMEP---VTVKMQLR---RPSDQ-------EVSE-SMD-FRY	270
_XP_030166526.1_	235	FKTP------PYCRA-------------ITEP---VTVKMQLR---RPSDQ-------EVSE-SMD-FRY	270
_XP_036903878.1_	234	FKTP------PYCKA-------------ITEP---VTVKMQLR---RPSDQ-------EVSE-SMD-FRY	269
CAH7004032.1	235	FKTP------PYCKA-------------ILEP---VTVKMQLR---RPSDQ-------EVSE-SMD-FRY	270
_XP_051056549.1_	235	FKTP------PYCKA-------------ILEP---VTVKMQLR---RPSDQ-------EVSE-SMD-FRY	270
_XP_042547166.1_	235	FKTP------PYCKV-------------ISEP---VTVKMQLR---RPSDQ-------EVSK-SMD-FRY	270
_XP_054584456.1_	235	FKTP------PYCKV-------------ITEP---VTVKMQLR---RPSDQ-------EVSE-PMD-FRY	270
_XP_049996768.1_	235	FKTP------PYCKA-------------ILEP---VTVKMQLR---RPSDQ-------EVSE-SMD-FRY	270
_XP_027243805.1_	235	FKTP------PYCKA-------------ILEP---VTVKMQLR---RPSDQ-------EVSE-SMD-FRY	270
_XP_047707379.1_	235	FKTP------PYCRA-------------ITEP---VTVKMQLR---RPSDQ-------EVSE-SMD-FRY	270
_XP_026645370.1_	235	FKTP------PYCKA-------------ILEP---VTVKMQLR---RPSDQ-------EVSE-SMD-FRY	270
_XP_014302519.1_	235	FKTP------PYCKV-------------ITEP---VTVKMQLR---RPSDQ-------EVSE-SMD-FRY	270
_XP_049726303.1_	235	FKTP------PYCKA-------------ITEP---VTVKMQLR---RPSDQ-------EVSE-SMD-FRY	270
_XP_049726302.1_	235	FKTP------PYCKA-------------ITEP---VTVKMQLR---RPSDQ-------EVSE-SMD-FRY	270
_XP_012969026.1_	235	FKTP------PYCKA-------------ILEP---VTVKMQLR---RPSDQ-------EVSE-SMD-FRY	270
_XP_008524232.1_	232	FKTP------PYCKA-------------ITEP---VTVKMQLR---RPSDQ-------EVSE-SMD-FRY	267
_XP_051020904.1_	235	FKTP------PYCKA-------------ILEP---VTVKMQLR---RPSDQ-------EVSE-SMD-FRY	270
_XP_023590712.1_	235	FKTP------PYCKA-------------ITEP---VTVKMQLR---RPSDQ-------EVSE-SMD-FRY	270
_XP_027243803.1_	235	FKTP------PYCKA-------------ILEP---VTVKMQLR---RPSDQ-------EVSE-SMD-FRY	270
_XP_040589151.1_	235	FKTP------PYCKA-------------ILEP---VTVKMQLR---RPSDQ-------EVSE-SMD-FRY	270
_XP_023412959.1_	235	FKTP------PYCKA-------------ITEP---VTVKMQLR---RPSDQ-------EVSE-SMD-FRY	270
_XP_036057111.1_	235	FMTP------PYCKA-------------ILVP---VTVKMQLR---RPSDQ-------EVSE-SMD-FRY	270
_XP_027822602.1_	235	FKTP------PYCKA-------------ITEP---VTVKMQLR---RPSDQ-------EVSE-SMD-FRY	270
_XP_031322949.1_	235	FKTP------PYCKA-------------ITEP---VTVKMQLR---RPSDQ-------EVSE-SMD-FRY	270
_XP_028634966.1_	235	FKTP------PYCKA-------------ILEP---VTVKMQLR---RPSDQ-------EVSE-SMD-FRY	270
_XP_031531529.1_	235	FKTP------PYCKA-------------ITEP---VTVKMQLR---RPSDQ-------EVSE-SMD-FRY	270
_XP_006073937.1_	235	FKTP------PYCKA-------------ITEP---VTVKMQLR---RPSDQ-------EVSE-SMD-FRY	270
_XP_034364887.1_	235	FKTP------PYCKA-------------ILEP---VTVKMQLR---RPSDQ-------EVSE-SMD-FRY	270
_XP_021482246.1_	235	FKTP------PYCRA-------------ILEP---VTVKMQLR---RPSDQ-------EVSE-SMD-FRY	270
KAF4016326.1	235	FKTP------PYCKA-------------ITEP---VTVKMQLR---RPSDQ-------EVSE-SMD-FRY	270
_XP_023602319.1_	235	FKTP------PYCKV-------------ITEP---VTVKMQLR---RPSDQ-------EVSE-SMD-FRY	270
_XP_052054164.1_	235	FKTP------PYCKA-------------ILEP---VTVKMQLR---RPSDQ-------EVSE-SMD-FRY	270
_XP_036291310.1_	235	FRTP------PYCKV-------------ITEP---VTVKMQLR---RPSDQ-------EVSE-PMD-FRY	270
_XP_013370168.1_	235	FKTP------PYCKA-------------ITEP---VTVKMQLR---RPSDQ-------EVSE-SMD-FRY	270
_XP_023590722.1_	235	FKTP------PYCKA-------------ITEP---VTVKMQLR---RPSDQ-------EVSE-SMD-FRY	270
_XP_048291328.1_	235	FKTP------PYCKA-------------ILEP---VTVKMQLR---RPSDQ-------EVSE-SMD-FRY	270
_XP_032772910.1_	235	FRTP------PYCRA-------------IVEP---VTVKMQLR---RPSDQ-------EVSE-SMD-FRY	270
_XP_008768670.2_	235	FRTP------PYCRA-------------IVEP---VTVKMQLR---RPSDQ-------EVSE-SMD-FRY	270
_XP_014302521.1_	235	FKTP------PYCKV-------------ITEP---VTVKMQLR---RPSDQ-------EVSE-SMD-FRY	270

```
_XP_031194882.1_  235  FKTP------PYCKA-------------ILEP---VTVKMQLR---RPSDQ-------EVSE-SMD-FRY  270
_NP_033070.2_     235  FKTP------PYCKA-------------ILEP---VTVKMQLR---RPSDQ-------EVSE-SMD-FRY  270
_XP_036291312.1_  235  FRTP------PYCKV-------------ITEP---VTVKMQLR---RPSDQ-------EVSE-PMD-FRY  270
_XP_021033157.1_  235  FKTP------PYCKA-------------ILEP---VTVKMQLR---RPSDQ-------EVSE-SMD-FRY  270
_XP_015849136.1_  235  FKTP------PYCKA-------------ILEP---VTVRMQLR---RPSDQ-------EVSE-SMD-FRY  270
_XP_045150027.1_  235  FKTP------PYCKA-------------IIEP---VTVKMQLR---RPSDQ-------EVSE-SMD-FRY  270
_XP_021066684.1_  235  FKTP------PYCKA-------------ILEP---VTVKMQLR---RPSDQ-------EVSE-SMD-FRY  270
_XP_006881088.1_  235  FKTP------PYGRT-------------ITEP---VTVKMQLR---RPSDQ-------EVSE-SMD-FRY  270
_XP_008524234.1_  232  FKTP------PYCKA-------------ITEP---VTVKMQLR---RPSDQ-------EVSE-SMD-FRY  267
_XP_021482260.1_  235  FKTP------PYCRA-------------ILEP---VTVKMQLR---RPSDQ-------EVSE-SMD-FRY  270
_XP_007125500.1_  235  FKTP------PYCKA-------------ITEP---VTVKMQLR---RPSDQ-------EVSE-SMD-FRY  270
_XP_028352731.1_  235  FKTP------PYCKA-------------ITEP---VTVKMQLR---RPSDQ-------EVSE-SMD-FRY  270
_XP_028352733.1_  235  FKTP------PYCKA-------------ITEP---VTVKMQLR---RPSDQ-------EVSE-SMD-FRY  270
_XP_028352734.1_  235  FKTP------PYCKA-------------ITEP---VTVKMQLR---RPSDQ-------EVSE-SMD-FRY  270
_XP_028352736.1_  235  FKTP------PYCKA-------------ITEP---VTVKMQLR---RPSDQ-------EVSE-SMD-FRY  270
_XP_028352735.1_  235  FKTP------PYCKA-------------ITEP---VTVKMQLR---RPSDQ-------EVSE-SMD-FRY  270
_ERE88482.1_      235  FKTP------PYCKA-------------ILEP---VTVKMQLR---RPSDQ-------EVSE-SMD-FRY  270
_KAF3826750.1_     84  FKTP------PYCRA-------------ITEP---VTVKMQLR---RPSDQ-------EVSE-SMD-FRY  119
_OBS70420.1_      171  FKTP------PYCKA-------------IVEP---VTVRMQLR---RPSDQ-------EVSE-SMD-FRY  206
_XP_006878094.1_  198  FKTP------PYCKV-------------ITEP---VTVKMQLR---RPSDQ-------EVSE-SMD-FRY  233
_XP_008978905.3_  192  FKTP------PYCKA-------------ITEP---VTVKMQLR---RPSDQ-------EVSE-SMD-FRY  227
_XP_032147941.1_  192  FKTP------PYCKT-------------IIEP---VTVKMQLR---RPSDQ-------EVSE-SMD-FRY  227
_XP_008524233.1_  194  FKTP------PYCKA-------------ITEP---VTVKMQLR---RPSDQ-------EVSE-SMD-FRY  229
_XP_044628438.1_  192  FKTP------PYCKA-------------ITEP---VTVKMQLR---RPSDQ-------EVSE-SMD-FRY  227
_XP_010608101.1_  192  FKTP------PYCKA-------------ITEP---VTVNMQLR---RPSDQ-------EVSE-PMD-FRY  227
_KAF5918007.1_    194  FKTP------PYCKA-------------ITEP---VTVKMQLR---RPSDQ-------EVSE-SMD-FRY  229
_XP_021101942.1_  192  FKTP------PYCKA-------------ITEP---VTVKMQLR---RPSDQ-------EVSE-SMD-FRY  227
_ABM89308.1_           ----------------------------------------------------------------------
_MBW00678.1_           ----------------------------------------------------------------------
_NP_001096177.1_  235  FKTP------PFHRS-------------ITDA---VTVKMQLR---RPSDQ-------EVSE-PMD-FRY  270
_OCT77375.1_      235  FKTP------PFLRS-------------IADA---VTVKMQLR---RPSDQ-------EVSE-PMD-FRY  270
_AAI70187.1_      235  FKTP------PFLRS-------------IADA---VTVKMQLR---RPSDQ-------EVSE-PMD-FRY  270
_AAI69362.1_      235  FKTP------PFHRS-------------ITDV---VTVKMQLR---RPSDQ-------EVSE-PMD-FRY  270
_XP_041418586.1_  235  FKTP------PFHRS-------------ITDV---VTVKMQLR---RPSDQ-------EVSE-PMD-FRY  270
_XP_041418585.1_  235  FKTP------PFHRS-------------ITDV---VTVKMQLR---RPSDQ-------EVSE-PMD-FRY  270
_XP_041418587.1_  235  FKTP------PFHRS-------------ITDV---VTVKMQLR---RPSDQ-------EVSE-PMD-FRY  270
_XP_053316221.1_  235  FKTP------AFPFN-------------ITEP---TTVKMQLR---RPSDR-------EVSE-PMD-FRY  270
_XP_053568018.1_  235  FRTP------PFHRP-------------VSEP---TTVKMQLR---RPSDQ-------EVSE-PMD-FRY  270
_KAG8443975.1_    235  FKTP------PFPRP-------------LTEA---IMVKMQLR---RPSDQ-------EVSE-PMD-FRY  270
```

_XP_044145067.1_	237	FKTP------TFPFD-------------ITEV---TTVKMQLR---RPSDQ-------EVSE-PMD-FRY	272
KAG9484268.1	237	FKTP------PIPFD-------------ITEV---TTVKMQLR---RPSDQ-------EVSE-PMD-FRY	272
_XP_040285660.1_	237	FKSP------TFPFD-------------ITEV---TTVKMQLR---RPSDQ-------EVSE-PMD-FRY	272
_XP_040285659.1_	237	FKSP------TFPFD-------------ITEV---TTVKMQLR---RPSDQ-------EVSE-PMD-FRY	272
KAG8583825.1	237	FKTP------PFPFD-------------ITEV---TTVKMQLR---RPSDQ-------EVSE-PMD-FRY	272
_XP_018411072.1_	237	FKTP------SFPFP-------------ITEV---TTVKMQLK---RPSDQ-------EVSE-PMD-FRY	272
_XP_040207073.1_	237	FRTP------PFPFP-------------ITEV---TTVKMQLK---RPSDQ-------EVSE-PMD-FRY	272
_NP_001081750.1_	232	FKTP------PFLRS-------------IADA---VTVKMQLR---RPSDE-------EVSE-PMD-FRY	267
PIO39092.1	113	FRTP------PFPFP-------------ITEV---TTVKMQLK---RPSDQ-------EVSE-PMD-FRY	148
PIO39091.1	113	FRTP------PFPFP-------------ITEV---TTVKMQLK---RPSDQ-------EVSE-PMD-FRY	148
_XP_041035877.1_	234	FKTP------PYKDIT------------LSQP---VTVHMQLR---RPSDK-------EVSD-AME-FQY	270
_XP_042202691.1_	234	FRTP------AYRDLS------------LERP---QLVHLQLR---RPSDK-------EVSD-TME-FQY	270
_XP_048475509.1_	223	FKTP------PYKDVT------------LSEP---VTVRMQLR---RPSDK-------EVSD-AME-FQY	259
_XP_048380947.1_	223	FKTP------PYKDVT------------LSEP---VTVRMQLR---RPSDK-------EVSD-AME-FQY	259
_XP_051901152.1_	227	FKTP------PYKDVM------------LSQP---VTVHMQLR---RPYRX------GSQAT-PME-FQY	264
BAD60879.1	223	FRTP------PYRDPH------------IQSS---VQVFIQLR---RPSDD-------EIGD-PKP-FQY	259
_XP_050405370.1_	236	FRTP------PYRECY------------ISRP---FDVHMQLQ---RPSDG-------ETSE-PIS-FTY	272
_XP_009063713.1_	236	FKTP------EYRNCY------------INRP---VQVFMQLH---RPSDA-------ETSE-PIN-FIY	272
CAB0044847.1	251	FKVP------TYCDPG------------IEEP---RQVTVQLR---RPSDG-------AVSQ-AVP-FTY	287
_XP_048259911.1_	237	FKTP------AYKEAY------------IQQP---VEVQLQLR---RPSDN-------ETSE-SIP-FTY	273
ADI72431.1	237	FKTP------AYKEAY------------IQQP---VEVQLQLR---RPSDN-------ETSE-SIP-FTY	273
CAG5133522.1	239	FRTP------AYHSQD------------ITKP---VEVNMQLH---RPSDG-------ETSE-SIP-FTY	275
KAG5688355.1	147	FKTP------KYKEEY------------ITRP---VNVNMQLL---RPSDM-------EASD-PIQ-FTY	183
AMT84567.1	237	FKTP------RYRDPY------------IKQP---VSVHVQLK---RPSDN-------ETSD-AKP-FLF	273
_XP_052276378.1_	235	FKTP------PYMDED------------IQQP---VEVEMQLQ---RSSDL-------EGSD-PIR-FTY	271
_XP_052276377.1_	235	FKTP------PYMDED------------IQQP---VEVEMQLQ---R-SDL-------EGSD-PIR-FTY	270
AAK72690.1	238	FRTP------PYKDTM------------ITRP---KEVKMQLK---RNNEP-------ETSD-SIP-FIY	274
_XP_011414731.2_	238	FRTP------PYKDTM------------ITRP---KEVKMQLK---RNNEP-------ETSD-SIP-FIY	274
_XP_052699807.1_	238	FRTP------PYKDTM------------ITRP---KEVKMQLK---RNNEP-------ETSD-SIP-FIY	274
_NP_001292261.1_	238	FRTP------PYKDTM------------ITRP---KEVKMQLK---RNNEP-------ETSD-SIP-FIY	274
_XP_048738054.1_	238	FRTP------PYRDTM------------ITRP---KEVKMQLK---RNNEP-------ETSD-AIP-FIY	274
_XP_052699808.1_	238	FRTP------PYKDTM------------ITRP---KEVKMQLK---RNNEP-------ETSD-SIP-FIY	274
_XP_022332457.1_	238	FRTP------PYKDTM------------ITRP---REVKMQLK---RNNEP-------ETSD-SIP-FMY	274
ABL63469.1	237	FKTP------PYKDTL------------ISQP---VEVRMQLK---RQNDA-------ETSE-AIP-FIY	273
_XP_033733822.1_	240	FKTP------PYKDTF------------ITRP---VEVKMQLK---RMNDT-------ETSD-PIP-FTY	276
_XP_021347355.1_	240	FKTP------PYKDTY------------ITRP---VEVKMQLK---RMNDT-------ETSD-PIP-FTY	276
UOA58607.1	239	FKTP------PYKDTF------------ISRP---VEVKMQLK---RMNDT-------ETSD-SIP-FTY	275
_XP_021347356.1_	240	FKTP------PYKDTY------------ITRP---VEVKMQLK---RMNDT-------ETSD-PIP-FTY	276
CAG2186872.1	237	FKTP------PYKDQY------------ITRP---EQVKMQLQ---RLNDP------AETSD-PIP-FIY	274

```
_XP_052058915.1_  237  FKTP------PYKDQY------------ITRP---EQVKMQLQ---RLNDP------AETSD-PIP-FIY  274
_KAJ8309232.1_    238  FKTP------PYKDPY------------ITRP---VEVKMQLK---RTNDT-------ETSD-SIP-FIY  274
_UZH84475.1_      235  FRTP------AYKDQY------------ISKP---VEVKMQLQ---RISDL-------DGSD-PIP-FTY  271
_ALA99939.1_      235  FKTP------PYQDQY------------LTQP---VEVQMQIQ---RSSDL-------EGSD-PIA-FTY  271
_XP_052767764.1_  235  FKTP------PYSNQY------------IQQP---VEVEMQLQ---RSSDL-------EGSD-PIK-FTY  271
_ADD25211.1_      239  FKTP------PYKDTF------------ISRP---VEVKMQLK---RMND--------------------  264
_XP_052224245.1_  233  FKTP------PFMDQN------------IRQP---VVVEMQLQ---RSSDL-------EGCD-SIW-FTY  269
_KAJ8314234.1_         ----------------------------------------------------------------------
_VDH95719.1_      196  FKTP------PYKDQY------------ITRP---EQVKMQLQ---RLNDP------AETSD-PIP-FIY  233
_XP_041352913.1_  237  FKTP------AFREKL------------IDRP---VEVQMQLR---RPSDQ-------EMSE-PIP-FTY  273
_XP_046555158.1_  237  FKTP------PYKEAF------------IQQP---VDVLMQLK---RPSDN-------ETSE-SIP-FTY  273
_AAW33559.1_      237  FKTP------PYKEAF------------IQQP---VDVQMQLR---RPSDN-------ETSE-SIP-FTY  273
_ABY87435.1_      224  FKTP------PYKEAF------------IQQP---VDVQTQLR---RPSDN-------ETSE-SIP-LTY  260
_XP_009028834.1_  237  FKTP------PYRNPD------------IQEP---VTVFVQLR---RPSDD-------EVGD-PKP-FQY  273
_XP_025106109.1_  237  FKTP------KYREEY------------ITRP---VNVSMQLM---RPSDG-------ELSD-AIQ-FTY  273
_NP_001298191.1_  239  FRTP------SYHNTE------------ITRP---AEVLMQLQ---RPSDG-------ETSE-PIP-FTY  275
_KAH9519545.1_    239  FRTP------AHHNPE------------ITRP---AEVLMQLQ---RPSDG-------ETSE-PIP-FTY  275
_RUS74561.1_      240  FRTP------AYVNQD------------ITKP---LVVQMQLQ---RPSDG-------ETSE-SIP-FTY  276
_GFO11973.1_      239  FRTP------AYVNQE------------ITKP---QVVHIQLQ---RPSDG-------ETSE-SIP-FTY  275
_CAG5132534.1_         ----------------------------------------------------------------------
_GFR69641.1_           ----------------------------------------------------------------------
_XP_035824873.1_  239  V---------------------------------------------------------------------  239
_AAY27981.1_      237  FQTP------AYKDPF------------ITKS---VSVSLQLF---RPTDQ-------ACSE-PKE-FLY  273
_XP_052827753.1_  232  FKTP------PYRNIR------------ITKD---VTVLLQLV---RPSDF-------AYSN-PIK-FTY  268
_XP_029642523.1_  232  FKTP------PYRNIR------------ITKD---VTVLLQLV---RPSDF-------ASSN-PIK-FTY  268
_CAE1288013.1_    196  FQTP------AYKDPF------------ITKS---VSVSLQLF---RPSDQ-------ACSE-PRE-FLY  232
_ELU04547.1_      237  FKTP------CFRDPY------------IRDP---AQVFVQLR---RPSDE-------ETSD-SKP-FQF  273
_KAI0227899.1_    234  IRTP------AYRDTR------------IKQP---VTISFQLR---RPSDG-------EVSE-PKP-FQY  270
_XP_044002103.1_  241  LKTP------KYDLNP------------DDKA---VKVFVQLR---RPSDG-------AVGE-PRP-FEM  277
_XP_044002102.1_  240  FKTP------KYDLNP------------DDKA---VKVFVQLR---RPSDG-------AVGE-PRP-FEM  276
_XP_044002099.1_  240  FKTP------KYDLNP------------DDKA---VKVFVQLR---RPSDG-------AVGE-PRP-FEM  276
_XP_037026629.1_       ----------------------------------------------------------------------
_CAD7632570.1_    245  LMAP------PYPRPE------------CRHP---VDVYLQLR---RPSDQ-------RASE-AMI-FTY  281
_CAD7650738.1_    244  LMAP------GYPYSDD---------MSGGKP---VPVYLQLR---RPSDQ-------RSSD-PVV-FTY  283
_XP_054163335.1_       ----------------------------------------------------------------------
_CAD7629985.1_    245  LLTP------KYIEPI------------NEEK---V-VYLQLR---RPSDH-------ELSA-PIK-L--  278
_CAD7652069.1_    238  LLTP------KYAEPI------------NQDK---I-IYLQLR---RPSDQ-------ELSA-PIK-LQM  273
_XP_017954168.1_  239  FRTP------RYRNTE------------IQHS---VQVELQLE---RPSDR-------ATSA-ALP-FEY  275
_XP_017858583.1_  239  FRTP------RYRNTE------------IQHS---VQVELQLE---RPSDR-------ATSA-ALP-FEY  275
```

```
_XP_002001588.1_   239  FRTP------RYRNTE------------IQHS---VQVELQLE---RPSDR-------ATSA-ALP-FEY  275
_XP_017954163.1_   239  FRTP------RYRNTE------------IQHS---VQVELQLE---RPSDR-------ATSA-ALP-FEY  275
_XP_023164563.2_   239  FKTP------RYRNTE------------IQHS---VQVELQLE---RPSDR-------ATSA-ALP-FEY  275
_TDG46702.1_       239  FRTP------RYRNTE------------IQHS---VQVELQLE---RPSDR-------ATSA-ALP-FEY  275
_XP_030564898.1_   239  FRTP------RYRNPE------------IQHS---VQVELQLE---RPSDR-------ATSA-ALP-FEY  275
_XP_015024496.1_   239  FRTP------RYRNPE------------IQHS---VQVELQLE---RPSDR-------ATSA-ALP-FEY  275
_XP_034475238.1_   240  FRTP------RYRNPE------------TQKG---VQVELQLV---RPSDG-------ATSE-PRP-FEY  276
_XP_034475235.1_   240  FRTP------RYRNPE------------TQKG---VQVELQLV---RPSDG-------ATSE-PRP-FEY  276
_XP_017854415.1_   239  FRTP------RYRNTD------------LTHS---VQVSIRLE---RPSDN-------ATSA-SVP-FEY  275
_XP_017854416.1_   239  FRTP------RYRNTD------------LTHS---VQVSIRLE---RPSDN-------ATSA-SVP-FEY  275
_ALC40089.1_       239  FRTP------RYRNTD------------LTHS---VQVSIRLE---RPSDN-------ATSA-SVP-FEY  275
_XP_030382983.1_   242  FRTP------RYRDPD------------VTHS---VFVYLQLV---RPTDM-------ATSD-PVA-FEY  278
_XP_030382981.1_   242  FRTP------RYRDPD------------VTHS---VFVYLQLV---RPTDM-------ATSD-PVA-FEY  278
_XP_001989397.1_   239  FKTP------RYRNTE------------IQHS---VQVDLQLQ---RPSDG-------ATSA-ALP-FEY  275
_XP_002067135.2_   240  FKTP------RYRNTE------------ITHC---VNVELQLV---RPSDR-------ATSA-ALH-FEY  276
_XP_034475239.1_   238  FRTP------RYRNPE------------VRHI---VQVQLQLV---RPSDG-------ATSE-PRP-FEY  274
_KAH8411901.1_     240  FKTP------RYRDPE------------IQHS---VKVELQLV---RPSDN-------ATSA-AVE-FEY  276
_XP_016963143.1_   240  FKTP------RYKNTE------------ITQS---VNVELKLV---RPSDG-------ATSA-PLQ-FEY  276
_XP_037712361.1_   240  FKTP------RYRNTE------------ITQS---VNVELKLV---RPSDG-------ATSA-PMQ-FEY  276
_XP_016993627.2_   240  FKTP------RYKNTE------------ITQS---VNVELKLV---RPSDG-------ATSA-PLQ-FEY  276
_XP_016963141.1_   240  FKTP------RYKNTE------------ITQS---VNVELKLV---RPSDG-------ATSA-PLQ-FEY  276
_XP_016993624.2_   240  FKTP------RYKNTE------------ITQS---VNVELKLV---RPSDG-------ATSA-PLQ-FEY  276
_XP_037712358.1_   240  FKTP------RYRNTE------------ITQS---VNVELKLV---RPSDG-------ATSA-PMQ-FEY  276
_XP_001974449.1_   240  FKTP------RYRNTE------------ITQS---VNVELKLV---RPSDG-------ATSA-PLP-FEY  276
_AAM48404.1_       240  FKTP------RYRNTE------------ITQS---VNVELKLV---RPSDG-------ATSA-PLP-FEY  276
_NP_001162999.1_   240  FKTP------RYRNTE------------ITQS---VNVELKLV---RPSDG-------ATSA-PLP-FEY  276
_XP_026835878.1_   240  FKTP------RYRNTE------------ITQS---VNVELKLV---RPSDG-------ATSA-PLP-FEY  276
_AAA28465.1_       240  FKTP------RYRNTE------------ITQS---VNVELKLV---RPSDG-------ATSA-PLP-FEY  276
_XP_002038912.1_   240  FKTP------RYRNTE------------ITQS---VNVELKLV---RPSDG-------ATSA-PLP-FEY  276
_XP_033169558.1_   240  FKTP------RYRNTE------------ITQS---VNVELKLV---RPSDG-------ATSA-PLP-FEY  276
_EDX05310.1_       240  FKTP------RYRNTE------------ITQS---VNVELKLV---RPSDG-------ATSA-PLP-FEY  276
_NP_001162998.1_   240  FKTP------RYRNTE------------ITQS---VNVELKLV---RPSDG-------ATSA-PLP-FEY  276
_XP_032579766.1_   240  FKTP------RYRNTE------------ITQS---VNVELKLV---RPSDG-------ATSA-PLP-FEY  276
_XP_033169541.1_   240  FKTP------RYRNTE------------ITQS---VNVELKLV---RPSDG-------ATSA-PLP-FEY  276
_XP_034669502.1_   240  FKTP------RYRNPE------------ITQS---VNVELKLV---RPSDG-------ATSA-PLQ-FEY  276
_XP_034125957.1_   240  FKTP------RYRNPE------------ITQS---VNVELKLV---RPSDG-------ATSA-PLQ-FEY  276
_XP_034669501.1_   240  FKTP------RYRNPE------------ITQS---VNVELKLV---RPSDG-------ATSA-PLQ-FEY  276
_XP_034125956.1_   240  FKTP------RYRNPE------------ITQS---VNVELKLV---RPSDG-------ATSA-PLQ-FEY  276
_XP_034125955.1_   240  FKTP------RYRNPE------------ITQS---VNVELKLV---RPSDG-------ATSA-PLQ-FEY  276
```

```
_XP_017152033.1_  240  FKTP------RYRNPE------------ITQS---VNVELKLV---RPSDG-------ATSA-PLQ-FEY  276
_XP_017152031.1_  240  FKTP------RYRNPE------------ITQS---VNVELKLV---RPSDG-------ATSA-PLQ-FEY  276
_XP_002014799.1_  240  FKTP------RYRNPE------------ITQS---VNVELKLV---RPSDG-------ATSA-PLQ-FEY  276
_XP_026844902.1_  240  FKTP------RYRNPE------------ITQS---VNVELKLV---RPSDG-------ATSA-PLQ-FEY  276
_XP_015035983.2_  240  FKTP------RYRNPE------------ITQS---VNVELKLV---RPSDG-------ATSA-PLQ-FEY  276
_XP_017063896.1_  240  FKTP------RYRNTE------------ITQS---VNVELKLV---RPSDG-------ATSA-PLK-FEY  276
_XP_016935332.1_  240  FKTP------RYRNTE------------ITQS---VNVELKLV---RPSDG-------ATSA-PLQ-FEY  276
_XP_017063894.2_  240  FKTP------RYRNTE------------ITQS---VNVELKLV---RPSDG-------ATSA-PLK-FEY  276
_XP_036668637.1_  240  FKTP------RYRNTE------------ITQS---VNVELKLV---RPSDG-------ATSA-PLQ-FEY  276
_XP_016935310.1_  240  FKTP------RYRNTE------------ITQS---VNVELKLV---RPSDG-------ATSA-PLQ-FEY  276
_KAH8298318.1_    240  FKTP------RYRNTE------------ITQS---VNVELKLV---RPSDG-------ATSA-PLQ-FEY  276
_KAH8412187.1_    240  FKTP------RYRNTE------------ITQS---VNVELKLV---RPSDG-------ATSA-PLQ-FEY  276
_KAH8278042.1_    239  FKTP------RYKNPE------------ITTN---VNVKLKLV---RPSDK-------ATSA-PRQ-FEY  275
_KAH8366423.1_    240  FKTP------RYRNTE------------ITQS---VNVALKLV---RPSDG-------ATSA-PLE-FEY  276
_XP_017044860.1_  241  FKTP------RYKNTE------------ITHS---VNVELKLV---RPSDG-------ATSA-PIP-FEF  277
_KAH8324526.1_    238  FKTP------RYRNPE------------ITTN---VNVKLKLV---RTSDK-------ATSA-SLP-FEY  274
_XP_017116817.1_  240  FKTP------RYKNTE------------ITHS---VNVELKLV---RPSDG-------ATSA-PLP-FEY  276
_XP_041566118.1_  239  FKTP------RYKNTE------------ITHS---VNVELKLV---RPSDG-------ATSA-PLP-FEY  275
_XP_052852584.1_  240  FKTP------RYKNTE------------ITHS---VNVELKLV---RPSDG-------ATSA-PLQ-FEY  276
_XP_052852582.1_  240  FKTP------RYKNTE------------ITHS---VNVELKLV---RPSDG-------ATSA-PLQ-FEY  276
_XP_017107132.2_  235  FKTP------RYKNPE------------ITTS---VNVKLKLV---RPSDK-------ATSA-PLQ-FEY  271
_XP_043069650.1_  235  FKTP------RYKNPE------------ITTS---VNVKLKLV---RPSDK-------ATSA-PLQ-FEY  271
_KAH8283158.1_    241  FKTP------RYRNPE------------ITQS---VHVELKLV---RPSDG-------ATSA-PLK-FEY  277
_XP_017025640.1_  239  FKTP------RYRNPE------------ITQS---VHVELKLV---RPSDG-------ATSA-PLK-FEY  275
_XP_020803071.1_  240  FKTP------RYRNPE------------ITQS---VHVELKLV---RPSDG-------ATSA-PLK-FEY  276
_XP_017025638.1_  240  FKTP------RYRNPE------------ITQS---VHVELKLV---RPSDG-------ATSA-PLK-FEY  276
_KAH8308067.1_    240  FKTP------RYRNPE------------ITQS---VHVELKLV---RPSDG-------ATSA-PLK-FEY  276
_KAH8362606.1_    240  FKTP------RYRNPE------------ITQS---VHVELKLV---RPSDG-------ATSA-PLK-FEY  276
_XP_020803068.1_  240  FKTP------RYRNPE------------ITQS---VHVELKLV---RPSDG-------ATSA-PLK-FEY  276
_XP_015052364.1_  240  FKTP------RYRNTE------------ITKS---VYVNLKLV---RPSDG-------ATSS-PLL-FEY  276
_XP_002090469.1_  239  FKTP------RYRNTE------------ITKS---VYVNLKLV---RPSDG-------ATSS-PLL-FEY  275
_XP_039480354.1_  239  FKTP------RYRNTE------------ITKS---VYVNLKLV---RPSDG-------ATSS-PLL-FEY  275
_XP_039480352.1_  239  FKTP------RYRNTE------------ITKS---VYVNLKLV---RPSDG-------ATSS-PLL-FEY  275
_XP_041450795.1_  240  FKTP------RYRNTE------------ITQS---VYVELKLV---RPSDG-------ATSA-PLQ-FEY  276
_XP_044315461.1_  241  FKTP------RYKNTE------------ITHS---VNVELKLV---RPSDG-------ATSA-PLP-FEF  277
_XP_044315463.1_  240  FKTP------RYKNTE------------ITHS---VNVELKLV---RPSDG-------ATSA-PLP-FEF  276
_XP_001961710.2_  240  FKVP------RYKNPE------------ITAN---VNVELKLV---RPSDK-------ATSA-PLQ-FEY  276
_XP_014761890.1_  240  FKVP------RYKNPE------------ITAN---VNVELKLV---RPSDK-------ATSA-PLQ-FEY  276
_KAH8323309.1_    240  FKVP------RYKNPE------------ITAN---VNVELKLV---RPSDK-------ATSA-PLQ-FEY  276
```

```
_XP_043646206.1_  239  FKTP------RYRNTE------------ITKS---VFVYLKLV---KPSDG-------ATSP-PRE-FEY  275
_XP_043646204.1_  239  FKTP------RYRNTE------------ITKS---VFVYLKLV---KPSDG-------ATSP-PRE-FEY  275
_AAQ65066.1_           -----------------------------------------------------------------------
_AAQ64900.1_           -----------------------------------------------------------------------
_AAQ64905.1_           -----------------------------------------------------------------------
_KAH8378209.1_    241  FRTP------SYHNKN------------IQNC---VEVEVQLV---RPTDG-------ATSA-PRP-FIY  277
_KAH8293207.1_    239  FQTP------SYRNKN------------IQNP---VEVQVQLV---RRTDG-------ATSA-SLP-FIY  275
_KAH8391978.1_    240  FKTP------SYHNKN------------IQNC---VDVEVQLV---RPSDG-------ATSA-PRV-FTY  276
_XP_034101352.1_  240  FKTP------SYHNKN------------IQNC---VDVEVQLV---RPSDG-------ATSA-PRV-FTY  276
_KAJ3658269.1_    242  FRPP------KYHDQN------------ITDA---VKVWVQLV---RPTDE-------AASP-PAE-FEY  278
_KAJ3658270.1_    242  FRPP------KYHDQN------------ITYA---VKVWVQLV---RPTDQ-------MVSQ-PIE-FMY  278
_KAJ3658271.1_    242  FKPP------KYHNQD------------IVEP---VTVWVQLV---RPSDE-------AASS-PVE-FEY  278
_XP_015117085.1_  242  VKTP------AYPTKD------------TGEP---VQLMIQLT---KPSTG-------EAGK-AIP-FTF  278
_XP_011307643.1_  242  IKTP------AYPLKE------------TDDC---VHLMVQLT---KPSTG-------EAGK-AIP-FTF  278
_XP_011307741.1_  242  LQTP------PYYTQN------------VDNP---VNIFVQLV---KPTTN-------DAGK-PVS-LQL  278
_XP_015119445.1_  242  VITP------TYTRRD------------TESP---VRVMIQLT---KPSTG-------EAGK-AIP-FIL  278
_XP_052128594.1_  254  FRTP------PYKTLE------------VEAP---VDVFIQLR---RPSDN-------SVGD-AVP-FQL  290
_XP_052128597.1_  245  FRTP------PYKTLE------------VEAP---VDVFIQLR---RPSDN-------SVGD-AVP-FQL  281
_KAE8741315.1_    200  FRTP------PYKTLE------------L------------------------------------------  210
_XP_028982160.1_  245  LRTP------AYHNVN------------VSEP---VSVFIQLV---RPSDG-------DMSE-PVP-FQF  281
_XP_011307645.1_  245  LRTP------AYHDVD------------FPRP---VPILMQLV---RPSDG-------DVSE-AVP-FQL  281
_XP_054725384.1_  235  LKTP------RYHNIN------------ITKP---VTVFVQLV---RASDG-------IASD-PVQ-FTY  271
_XP_053965385.1_  235  LRTP------RYHNIN------------ITKP---VTVFVQLV---RVSDG-------IASD-PVQ-FTY  271
_XP_013149158.1_  244  FNTP------PYHDTD------------ITTN---ATVYVQLK---RLSDN-------ARSD-PIE-IQY  280
_XP_045534655.1_  246  FTTP------PYRHTD------------ITQN---VTVFVQLK---RLSDN-------ARSE-PVE-FVY  282
_KPJ06852.1_      246  FTTP------PYRHTD------------ITQN---VTVFVQLK---RLSDN-------ARSE-PVE-FVY  282
_XP_013176418.1_  242  FNTP------PYRHTD------------ITTN---VTVSVQLK---RLSDN-------ARSE-PVQ-FVY  278
_OXU28425.1_      260  IETP------EFRTDTLS-------PENINEP---IEVNIQLK---RPSNG-------HVSD-PIP-FQL  301
_XP_008216913.1_  241  IETP------EFKTDTLS-------PESIKEP---IEVNVQLK---QPSNG-------HVSD-PIP-FQL  282
_XP_008216914.1_  237  IETP------EFKTDTLS-------PESIKEP---IEVNVQLK---QPSNG-------HVSD-PIP-FQL  278
_XP_040572301.1_  235  FLTP------PYKDEE------------LTHP---VTVYIYLY---KSGLR-------QRSE-PLK-FQF  271
_CAB4068408.1_    142  FLTP------PYKDEE------------LTHP---VTVYIYLY---KSGLR-------QRSE-PLK-FQF  178
_KAF7993284.1_    224  FLTP------AYERV-------------IQQP---VDVYVELR----KSDG-------ETSE-AWP-FTI  258
_KAF7987489.1_    243  IKTP------KYNRQ-------------STEI---IKGFIQLV---KPSDG-------QSSQ-PHE-FVI  278
_KAF7994520.1_    245  IVTP------KYDRQ-------------LSDV---IEVFIKLV---RPSDG-------QSSE-PRQ-FEI  280
_XP_044019368.1_  248  IKTP------EYNGL-------------QFGI---IKVFIQLV---RKTDG-------VTSE-PHK-YS-  282
_XP_044019332.1_  149  IKTP------KYNRQ-------------STEI---IKGFIQLV---KPSDV-------IKEN--------  178
_XP_044003986.1_  118  IVTP------KYDRQ-------------LSDV---IEVFIKLV---RPSDG-------QSSE-PRQ-FEI  153
_XP_037042732.1_  243  FTTP------KCDLKS------------VDTP---QNIFIEMV---RPSDD-------VRRN-RFP-FQY  279
```

```
_CAD7223243.1_     237  LETP------PYRDEN------------ITEP---VNCKIRLR---QLKEP-------LDGK-PRA-FQY  273
_XP_044732233.1_   242  FKTP------KYRKTD------------ITLP---VKVKIRLE---RTDGDV-----VTASA-PRD-FYY  280
_XP_012231885.1_   234  FKTP------KYHSRE-----------VVIRP---VQIYIQLQ---RPSDN-------ATSE-PRP-FQM  271
_XP_022814699.1    239  CFAP------PYRDTD------------IKES---VTVYFELR---RKSDD-------TRSN-AEK-FHY  275
_CAB3516523.1_     239  CFAP------PYRDTN------------IKES---VTVYFELR---RKSDD-------TRSN-AEK-FHY  275
_XP_035458245.2_   238  CFAP------PYRDTD------------IKEN---VTVYFELR---RKSDG-------TRSS-PEK-FHY  274
_KAF9795795.1_     227  CFAP------PYRDTD------------IKEN---VTVYFELR---RKSDG-------TRSS-PEK-FHY  263
_XP_014233257.1_   248  FSTP------AYRGSG-----------ELEQP---VTVNVRLR---LKSDP-------SVTS-PRV-YRY  285
_XP_034324400.1_   225  VKIP------PYKNLE------------IEVP---KQIQVYVK---SDGKV-------DLEAKPMD-FLY  262
_XP_052699907.1_   225  VKIP------PYKNSE------------IENP---KQIQVYVK---SDGKV-------DLEAKPMD-FMY  262
Consensus_aa:           hpoP.......Y.p..............lpps...lpl.hpL....RsSD..........Sp.s.s.Fph
Consensus_ss:           ee                                 eeeeeeee   e              ee  eee

Conservation:            6
_XP_001652840.2_   277  TPL----------  279
_AAW67214.1_       277  TPL----------  279
_XP_021704027.1_   277  TPL----------  279
_XP_019540833.2_   277  TPL----------  279
_XP_029720231.1_   277  TPL----------  279
_AAW67215.1_       277  TPL----------  279
_XP_021704025.1_   277  TPL----------  279
_XP_019540831.2_   277  TPL----------  279
_KXJ81105.1_       277  TPL----------  279
_KXJ76497.1_       277  TPL----------  279
_XP_023240428.1_   276  LPL----------  278
_CAD7241875.1_     278  LPL----------  280
_TRY80291.1_       274  YPR----------  276
_ALF84899.1_       271  RPL----------  273
_KAF4528208.1_     277  LPL----------  279
_XP_054260955.1_   278  TPL----------  280
_XP_054264401.1_   278  TPL----------  280
_XP_054260954.1_   278  TPL----------  280
_XP_038114042.1_   277  FPL----------  279
_XP_039437558.1_   277  FPL----------  279
_XP_001844078.2_   277  FPL----------  279
_EDS35747.1_       277  FPL----------  279
_XP_053696925.1_   277  IPL----------  279
_XP_001869619.2_   277  LPL----------  279
```

```
_XP_039434954.1_  277  LPL----------  279
_EDS29879.1_      277  LPL----------  279
_XP_019550742.2_  277  IPV----------  279
_KXJ74587.1_      277  IPV----------  279
_XP_035908773.1_  278  IPL----------  280
_AJC98655.1_      278  IPL----------  280
_AJC98650.1_      278  IPL----------  280
_AJC98644.1_      278  IPL----------  280
_AJC98657.1_      278  IPL----------  280
_AJC98648.1_      278  IPL----------  280
_AJC98647.1_      278  IPL----------  280
_AJC98651.1_      278  IPL----------  280
_AJC98643.1_      278  IPL----------  280
_AJC98656.1_      278  IPL----------  280
_AJC98641.1_      278  IPL----------  280
_AJC98640.1_      278  IPL----------  280
_AJC98653.1_      278  IPL----------  280
_AJC98654.1_      278  IPL----------  280
_AJC98652.1_      278  IPL----------  280
_AJC98646.1_      278  IPL----------  280
_AJC98658.1_      278  IPL----------  280
_AJC98639.1_      278  IPL----------  280
_XP_035908772.1_  278  IPL----------  280
_XP_050076803.1_  278  IPL----------  280
_XP_049282441.1_  278  IPL----------  280
_XP_040170080.1_  278  IPL----------  280
_XP_050076802.1_  278  IPL----------  280
_XP_041780138.1_  278  IPL----------  280
_XP_040239229.2_  278  IPL----------  280
_XP_040239222.2_  278  IPL----------  280
_XP_035908780.1_  278  IPL----------  280
_XP_040239221.2_  278  IPL----------  280
_XP_052897786.1_  278  IPL----------  280
_XP_052895493.1_  278  I-A----------  279
_XP_053664395.1_  278  IPA----------  280
_XP_050076801.1_  278  IPL----------  280
_XP_310177.3_     278  IPL----------  280
_XP_001652339.1_  277  IPV----------  279
_XP_053686256.1_  277  LPV----------  279
```

```
_XP_053675540.1_  278  IPL----------  280
_ETN66814.1_      277  TPL----------  279
_XP_049534501.1_  277  TPL----------  279
_XP_050084431.1_  277  TPL----------  279
_XP_035782874.1_  277  TPL----------  279
_XP_035782869.1_  277  TPL----------  279
_KFB39849.1_      285  TPL----------  287
_XP_052871375.1_       -------------
_AAR13743.1_           -------------
_AAR13744.1_           -------------
_CAA65157.1_           -------------
_XP_022170156.1_  276  TPV----------  278
_XP_025205168.1_  276  TPV----------  278
_XP_026817162.1_  276  TPV----------  278
_XP_027846205.1_  276  TPV----------  278
_XP_015370635.1_  276  TPV----------  278
_CAH1723797.1_    276  TPV----------  278
_KAF0769940.1_    276  TPV----------  278
_CAB4065202.1_    226  VP-----------  227
_XP_050440903.1_  276  TPV----------  278
_XP_050533799.1_  276  TPV----------  278
_XP_025410632.1_  277  TPD----------  279
_XP_008180746.1_  276  TPD----------  278
_XP_026465450.1_  278  APT----------  280
_VVC36026.1_      276  TPV----------  278
_XP_016661522.1_  276  TPD----------  278
_XP_001947429.3_  276  TPD----------  278
_XP_026466007.1_       -------------
_KRT81254.1_      274  IPL----------  276
_XP_033224832.1_  281  LP-----------  282
_XP_025407590.1_  269  TPD----------  271
_XP_045476729.1_  270  LPA----------  272
_XP_034174253.1_  277  LPL----------  279
_XP_029047866.1_  277  LPL----------  279
_XP_015430648.1_  277  LPL----------  279
_XP_017798081.1_  277  LPL----------  279
_XP_017756471.1_  277  LPL----------  279
_XP_043248447.1_  277  LPL----------  279
_XP_029047855.1_  277  LPL----------  279
```

```
_XP_034174252.1_  277  LPL----------  279
_XP_031829337.1_  277  LPL----------  279
_KZC09125.1_      277  LPL----------  279
_XP_033327213.1_  277  LPL----------  279
_XP_033327212.1_  277  LPL----------  279
_XP_017756445.1_  277  LPL----------  279
_OAD62393.1_      277  LPL----------  279
_XP_043248413.1_  277  LPL----------  279
_XP_033327208.1_  277  LPL----------  279
_XP_034950477.1_  277  LPL----------  279
_XP_034950475.1_  277  LPL----------  279
_XP_012343274.1_  277  LPL----------  279
_XP_016909449.1_  277  LP-----------  278
_XP_012343273.1_  277  LP-----------  278
_XP_006619742.1_  277  LP-----------  278
_XP_006567065.1_  277  LP-----------  278
_XP_006619741.1_  277  LP-----------  278
_NP_001011577.1_  277  LPL----------  279
_XP_016909448.1_  277  LP-----------  278
_XP_043802365.1_  277  LP-----------  278
_NP_001164477.1_  277  LP-----------  278
_XP_016909443.1_  277  LPL----------  279
_XP_043802361.1_  277  LP-----------  278
_XP_012343269.1_  277  LP-----------  278
_XP_006619738.1_  277  LP-----------  278
_PBC29878.1_      277  LP-----------  278
_XP_046663984.1_  276  TPL----------  278
_XP_046663985.1_  276  TPL----------  278
_XP_043274771.1_  277  LPL----------  279
_XP_043274769.1_  277  LPL----------  279
_XP_043289571.1_  276  LPL----------  278
_XP_032665873.1_  277  LPL----------  279
_XP_032665875.1_  277  LPL----------  279
_XP_032665874.1_  277  LPL----------  279
_XP_032665869.1_  277  LPL----------  279
_XP_014470289.1_  277  LPL----------  279
_XP_011149046.1_  277  LPL----------  279
_XP_017877914.1_  275  LPL----------  277
_XP_017877911.1_  275  LPL----------  277
```

```
_XP_033174774.1_  278  LPL----------  280
_XP_043581013.1_  277  LPL----------  279
_XP_048261507.1_  277  LPL----------  279
_XP_050573946.1_  277  LPL----------  279
_XP_050478075.1_  277  LPL----------  279
_XP_033204249.1_  277  LPL----------  279
_XP_033358451.1_  277  LPL----------  279
_XP_033358450.1_  277  LPL----------  279
_XP_033204244.1_  277  LPL----------  279
_XP_033312956.1_  277  LPL----------  279
_XP_012236639.1_  277  LPL----------  279
_XP_050573940.1_  277  LPL----------  279
_XP_012174118.1_  277  LPL----------  279
_XP_043581010.1_  277  LPL----------  279
_EZA51108.1_      277  LPL----------  279
_XP_011344085.1_  277  LPL----------  279
_XP_043519475.1_  277  LPL----------  279
_CAD1481114.1_    277  LPL----------  279
_XP_043519473.1_  277  LPL----------  279
_KOX75360.1_      277  LPL----------  279
_KAF3423741.1_    277  LPL----------  279
_KOC69050.1_      277  LPL----------  279
_XP_053980095.1_  278  LPL----------  280
_XP_054002893.1_  278  LPL----------  280
_XP_053980091.1_  278  LPL----------  280
_XP_028049144.1_  277  LPL----------  279
_XP_012541458.1_  277  LPL----------  279
_XP_003708412.1_  277  LPL----------  279
_XP_012152807.1_  277  LPL----------  279
_KAH0945556.1_    277  LPL----------  279
_XP_024871832.1_  276  LPL----------  278
_XP_011307641.1_  275  LPLI---------  278
_XP_015118260.1_  276  LPL----------  278
_XP_011307640.1_  276  LPL----------  278
_XP_015118252.1_  276  LPL----------  278
_EFN68841.1_      281  LPL----------  283
_XP_011647340.1_  277  LPL----------  279
_XP_029166122.1_  281  LPL----------  283
_KMQ96453.1_      281  LPL----------  283
```

```
_XP_050464492.1_  281  LPL----------  283
_XP_029673315.1_  281  LPL----------  283
_XP_025265518.1_  281  LPL----------  283
_XP_011255523.1_  281  LPL----------  283
_XP_011698133.1_  277  LPL----------  279
_XP_011698131.1_  277  LPL----------  279
_XP_018357420.1_  277  LPL----------  279
_KYM76140.1_      277  LPL----------  279
_XP_011647339.1_  277  LPL----------  279
_KAG5315434.1_    277  LPL----------  279
_XP_011055463.1_  277  LPL----------  279
_XP_018342550.1_  277  LPL----------  279
_XP_018403565.1_  277  LPL----------  279
_KAG5322912.1_    277  LPL----------  279
_XP_018357417.1_  277  LPL----------  279
_XP_018314560.1_  277  LPL----------  279
_XP_018057217.1_  277  LPL----------  279
_KAG5346558.1_    277  LPL----------  279
_XP_047348408.1_  278  LPL----------  280
_XP_047348412.1_  277  LPL----------  279
_XP_046820611.1_  277  LPL----------  279
_XP_050848254.1_  277  LPL----------  279
_XP_046820612.1_  277  LPL----------  279
_XP_050848252.1_  277  LPL----------  279
_XP_043667564.1_  277  LPL----------  279
_XP_035725337.1_  277  LPL----------  279
_XP_043667562.1_  277  LPL----------  279
_XP_046820605.1_  277  LPL----------  279
_XP_050848249.1_  277  LPL----------  279
_XP_043667560.1_  277  LPL----------  279
_XP_035725333.1_  277  LPL----------  279
_KAF7405659.1_    277  LPL----------  279
_KAF7402294.1_    277  LPL----------  279
_XP_015172825.1_  277  SPL----------  279
_XP_015172824.1_  277  SPL----------  279
_XP_015172823.1_  277  SPL----------  279
_XP_014609306.1_  277  SPL----------  279
_XP_043491219.1_  277  SPL----------  279
_XP_014609305.1_  277  SPL----------  279
```

```
_XP_043491218.1_  277  SPL----------  279
_KAI4498556.1_    277  SPL----------  279
_XP_015172818.1_  277  SPL----------  279
_XP_043491211.1_  277  SPL----------  279
_KAI4495142.1_    277  SPL----------  279
_XP_014609300.1_  277  SPL----------  279
_ADK91833.1_           -------------
_KAG7213837.1_    277  LPL----------  279
_XP_014226628.1_  276  LPS----------  278
_XP_020287946.1_  277  LPL----------  279
_XP_012232007.1_  277  LPL----------  279
_XP_011874538.1_  277  LPL----------  279
_XP_011874541.1_  277  LP-----------  278
_XP_011173619.1_  278  LP-----------  279
_XP_034950793.1_  274  IPL----------  276
_XP_034950792.1_  270  IPL----------  272
_XP_012340880.2_  274  LPL----------  276
_XP_031771859.1_  274  LPL----------  276
_XP_006618953.1_  276  LPL----------  278
_XP_031367904.1_  276  LPL----------  278
_XP_043795948.1_  276  LPL----------  278
_XP_395180.5_     276  LPL----------  278
_KAG6799500.1_    276  LPL----------  278
_XP_016768607.2_  276  LPL----------  278
_PBC31989.1_      285  LPL----------  287
_XP_016914450.1_  276  LPL----------  278
_KAG5344958.1_    274  LPL----------  276
_KAJ3630188.1_    276  LPT----------  278
_QHN75199.1_      276  LPT----------  278
_XP_051171660.1_  281  LP-----------  282
_XP_016909447.1_  235  LP-----------  236
_XP_031773030.1_  235  LP-----------  236
_KAH0956427.1_    278  LP-----------  279
_XP_012283784.1_  277  LPM----------  279
_XP_023224928.1_  275  LPL----------  277
_XP_008556434.1_  277  LPL----------  279
_XP_008556433.1_  277  LPL----------  279
_XP_014299728.1_  277  LPL----------  279
_CAD6234449.1_    277  LPL----------  279
```

KAG8037552.1	277	LPL----------	279
_XP_044576138.1_	277	LPL----------	279
_XP_044576125.1_	277	LPL----------	279
_XP_026465448.1_	275	LPL----------	277
_XP_026465446.1_	275	LPL----------	277
_XP_031785072.1_	277	LP-----------	278
_XP_015607185.1_	277	LP-----------	278
_XP_011496611.1_	277	LP-----------	278
_XP_018903707.1_	274	TPL----------	276
CAB3365562.1	276	LPL----------	278
_XP_011496447.1_	149	LPL----------	151
KAG8230996.1	276	IPL----------	278
_XP_040567422.1_	274	VP-----------	275
_XP_014250205.1_	277	TPL----------	279
_XP_014250206.1_	277	TPL----------	279
_XP_047119528.1_	277	IPL----------	279
_XP_047001845.1_	277	IPL----------	279
_XP_049939433.1_	277	IPL----------	279
_XP_049764197.1_	277	IPL----------	279
_XP_049833656.1_	277	IPL----------	279
_XP_049790026.1_	277	IPL----------	279
_XP_049833651.1_	277	IPL----------	279
_XP_047001842.1_	277	IPL----------	279
_XP_049790025.1_	277	IPL----------	279
_XP_049833654.1_	277	IPL----------	279
_XP_049833659.1_	277	IPL----------	279
_XP_021925503.1_	277	LPL----------	279
KDR23857.1	277	LPL----------	279
ABU96700.1	277	TPL----------	279
ABU96699.1	277	TPL----------	279
_XP_014275495.1_	277	TPL----------	279
BBE08137.1	277	TPL----------	279
_XP_014275494.1_	277	TPL----------	279
_XP_014275493.1_	277	TPL----------	279
BBE08136.1	277	TPL----------	279
KAF6207519.1	277	TPL----------	279
CAB0015508.1	277	TPL----------	279
CAB0015504.1	277	TPL----------	279
PNF18474.1	277	LPL----------	279

```
_CEG62431.1_       277  HPI----------  279
_PSN36690.1_       277  HPI----------  279
_XP_046387412.1_   277  IPL----------  279
_XP_046387414.1_   277  IPL----------  279
_XP_031634874.1_   277  IPS----------  279
_GFG29240.1_       277  LPL----------  279
_BBG28464.1_       277  TPS----------  279
_WCL52334.1_       277  TPS----------  279
_GLH00135.1_       277  TPS----------  279
_CAD7205239.1_     278  LPL----------  280
_CAD7431383.1_     277  LPL----------  279
_CAD7410836.1_     277  LPL----------  279
_CAD7415345.1_     277  LPL----------  279
_CAD7606635.1_     277  LPL----------  279
_CAD7462957.1_     277  LPL----------  279
_XP_012265400.2_   276  LPL----------  278
_XP_046473587.1_   277  LPL----------  279
_XP_046611732.1_   277  LPL----------  279
_XP_015519750.2_   277  LPL----------  279
_XP_046611731.1_   277  LPL----------  279
_XP_046417903.1_   277  LPL----------  279
_GLG94364.1_       277  LPL----------  279
_GLG94363.1_       277  LPL----------  279
_BBG28463.1_       277  LPL----------  279
_WCL52335.1_       277  LPL----------  279
_GLG94365.1_       277  LPL----------  279
_XP_002431518.1_   275  IPL----------  277
_KAE8746525.1_     277  LPLD---------  280
_XP_026277770.1_   277  LPL----------  279
_KAJ1519446.1_     277  TPL----------  279
_ALC78349.1_       276  LPL----------  278
_XP_046738772.1_   277  RPL----------  279
_XP_046417904.1_   275  LPL----------  277
_CRK94170.1_       275  LPL----------  277
_CRK94167.1_       275  LPL----------  277
_KAG5676160.1_     279  LPL----------  281
_XP_034245464.1_   278  IPV----------  280
_XP_034245457.1_   278  IPV----------  280
_KAG8287980.1_     243  TPL----------  245
```

```
_XP_044759045.1_  272  LPS----------  274
_XP_017305229.1_  267  TPL----------  269
_XP_026675878.1_  267  TPL----------  269
_KAE8574083.1_    229  TPL----------  231
_XP_037926497.1_  277  LPL----------  279
_CAD7079567.1_    277  LPL----------  279
_XP_037882485.1_  277  IPL----------  279
_XP_037037597.1_  277  LPL----------  279
_XP_037037592.1_  277  LPL----------  279
_KAG4075389.1_    277  LPL----------  279
_XP_034101357.1_  277  VPL----------  279
_XP_026844963.1_  277  VPL----------  279
_EDW28798.1_      277  VPL----------  279
_XP_034125966.1_  277  VPL----------  279
_XP_034666519.1_  277  VPL----------  279
_XP_001356570.3_  277  VPL----------  279
_XP_023164577.2_  277  VPL----------  279
_XP_017153206.1_  277  VPL----------  279
_XP_030564932.1_  277  VPL----------  279
_XP_002059145.1_  277  VPL----------  279
_EDW04266.1_      277  VPL----------  279
_XP_017025862.1_  277  VPL----------  279
_XP_017859907.1_  277  VPL----------  279
_XP_032584327.1_  277  VPL----------  279
_XP_016987288.2_  277  VPL----------  279
_XP_017954173.1_  277  VPL----------  279
_XP_017854504.1_  277  VPL----------  279
_XP_034125965.1_  277  VPL----------  279
_XP_034666515.1_  277  VPL----------  279
_XP_043641917.1_  277  VPM----------  279
_XP_026844960.1_  277  VPL----------  279
_XP_015035988.2_  277  VPL----------  279
_XP_041450744.1_  277  VPL----------  279
_XP_015052361.1_  277  VPM----------  279
_XP_039480515.1_  277  VPM----------  279
_XP_033248563.1_  277  VPL----------  279
_KAH8412150.1_    277  VPL----------  279
_XP_043864974.1_  277  VPL----------  279
_XP_020809119.1_  277  VPL----------  279
```

```
_KAH8298353.1_    277  VPL----------  279
_XP_030564927.1_  277  VPL----------  279
_XP_041631592.1_  277  VPL----------  279
_XP_017859909.1_  277  VPL----------  279
_XP_032591602.1_  277  VPL----------  279
_XP_023164572.2_  277  VPL----------  279
_TDG46699.1_      277  VPL----------  279
_KAH8411895.1_    277  VPM----------  279
_KAH8377496.1_    277  VPL----------  279
_XP_017854500.2_  277  VPL----------  279
_XP_016987284.2_  277  VPL----------  279
_KAH8293209.1_    277  VPL----------  279
_EEC00225.1_      276  HPN----------  278
_XP_029825505.2_  276  HPN----------  278
_KAG0424790.1_    276  HPN----------  278
_XP_037272127.1_  279  HPN----------  281
_XP_054934017.1_  277  HPN----------  279
_XP_050048524.1_  277  HPN----------  279
_XP_037272126.1_  277  HPN----------  279
_XP_037524244.1_  277  HPN----------  279
_XP_037524245.1_  277  HPN----------  279
_XP_049512094.1_  277  HPN----------  279
_XP_037524243.1_  277  HPN----------  279
_XP_037580160.1_  277  HPN----------  279
_XP_037272128.1_  279  HPN----------  281
_KAH9380259.1_    277  HPN----------  279
_KAI1291715.1_    249  NPV----------  251
_XP_035209023.1_  267  LPI----------  269
_XP_023332679.1_  277  LPT----------  279
_XP_046801942.1_  277  VPL----------  279
_XP_037811120.1_  277  VPL----------  279
_AFI98401.1_      277  VPL----------  279
_AFJ54621.1_      277  VPL----------  279
_KAI8126534.1_    277  VPL----------  279
_XP_013118266.1_  277  VPL----------  279
_XP_023293634.2_  277  VPL----------  279
_XP_037811062.1_  277  VPL----------  279
_KNC34132.1_      277  VPL----------  279
_TMW48951.1_      277  VPL----------  279
```

```
_XP_037957363.1_  277  VPL----------  279
_XP_005175014.1_  277  VPL----------  279
_XP_037957360.1_  277  VPL----------  279
_XP_030383330.1_  276  VPL----------  278
_XP_030383328.1_  276  VPL----------  278
_CAH1707987.1_    277  LPL----------  279
_TMW54796.1_      277  VPL----------  279
_XP_026836077.1_  277  VPMD---------  280
_XP_017116803.1_  277  LPL----------  279
_XP_039480519.1_  277  VPM----------  279
_XP_043641920.1_  277  VPM----------  279
_XP_023036748.1_  277  VPL----------  279
_XP_002090466.1_  277  VPM----------  279
_XP_034471824.1_  277  VPM----------  279
_AAT94434.1_      277  VPM----------  279
_XP_036670985.1_  277  VPM----------  279
_AAA28479.1_      277  VPM----------  279
_XP_037708206.1_  277  VPM----------  279
_XP_002038915.1_  277  VPM----------  279
_XP_016993636.1_  277  VPM----------  279
_XP_052852517.1_  277  VPL----------  279
_XP_016963139.1_  277  VPM----------  279
_XP_033156151.1_  277  VPM----------  279
_XP_001974446.1_  277  VPM----------  279
_XP_014761886.1_  277  VPL----------  279
_XP_017107216.2_  277  VPL----------  279
_XP_017044881.1_  276  VPL----------  278
_XP_016944798.1_  277  VPM----------  279
_XP_017116800.1_  277  LPL----------  279
_XP_016993633.2_  277  VPM----------  279
_XP_037708198.1_  277  VPM----------  279
_XP_032583259.1_  277  VPM----------  279
_AAC35296.1_      277  VPM----------  279
_NP_001286014.1_  277  VPM----------  279
_XP_016963136.1_  277  VPM----------  279
_XP_017063904.2_  277  VPM----------  279
_XP_033156135.1_  277  VPM----------  279
_XP_052852512.1_  277  VPL----------  279
_XP_032307758.1_  277  VPL----------  279
```

```
_XP_017107215.2_  277  VPL----------  279
_AWT86616.1_      276  TPI----------  278
_QUF98534.1_      276  TPI----------  278
_UNA06287.1_      276  TPI----------  278
_AAQ65068.1_           -------------
_AAQ64916.1_           -------------
_AAQ64921.1_           -------------
_AAQ64918.1_           -------------
_KAH8245459.1_    302  VPL----------  304
_KAH8283155.1_    299  VPL----------  301
_KAH8246384.1_    299  VPL----------  301
_KAH8366418.1_    299  VPM----------  301
_XP_022195378.2_  276  TPI----------  278
_XP_039284931.1_  276  TPI----------  278
_ALC40091.1_      299  VPL----------  301
_XP_036342419.1_  275  EPV----------  277
_XP_022903025.1_       -------------
_XP_022903017.1_       -------------
_XP_036218946.1_  275  EPV----------  277
_XP_018790401.1_  275  EPV----------  277
_KAI9579887.1_    263  IPL----------  265
_XP_037892537.1_  262  IPL----------  264
_XP_018903705.1_  281  TPL----------  283
_CAH0395667.1_    281  TPL----------  283
_XP_050330165.1_  275  EPV----------  277
_XP_039970878.1_  275  EPV----------  277
_XP_049305234.1_  276  EPV----------  278
_GLH04793.1_      284  TPL----------  286
_XP_015835035.1_  276  LPL----------  278
_XP_015835036.1_  276  LPL----------  278
_XP_039283832.1_  287  TPI----------  289
_XP_031788427.1_  272  MPT----------  274
_KAJ6646894.1_    278  LPD----------  280
_XP_026466084.1_  212  APT----------  214
_XP_044002069.1_  277  IPD----------  279
_XP_046611735.1_  272  HPK----------  274
_XP_046417906.1_  272  HPK----------  274
_XP_046590309.1_  272  HPK----------  274
_XP_046473589.1_  272  HPK----------  274
```

```
_XP_019892116.1_  188  VPL----------  190
_XP_005183027.3_  188  VPL----------  190
_XP_011496598.1_  201  LPV----------  203
_AAW67216.1_      176  TPL----------  178
_AAW67217.1_      176  TPL----------  178
_XP_017489788.1_  179  EPV----------  181
_BAI67894.1_           -------------
_XP_054741511.1_  276  FPA----------  278
_XP_053961684.1_  276  FPA----------  278
_XP_036341313.1_  276  VPA----------  278
_XP_036341343.1_  276  VPA----------  278
_XP_017471383.1_  276  VPA----------  278
_XP_054741498.1_  276  FPA----------  278
_XP_053961677.1_  275  FPA----------  277
_XP_050320008.1_  276  KPAK---------  279
_XP_028894490.2_  276  VPA----------  278
_XP_050320015.1_  276  KPA----------  278
_XP_018787554.1_  276  KPA----------  278
_XP_029407438.2_  276  KPA----------  278
_XP_036225551.1_  276  KPA----------  278
_XP_011178374.2_  276  VPA----------  278
_XP_039952593.1_  276  KPA----------  278
_XP_018787542.1_  276  KPA----------  278
_XP_011207866.2_  276  KPA----------  278
_XP_014086506.1_  276  KPA----------  278
_XP_004526638.1_  278  VPA----------  280
_XP_017471402.1_  276  VPA----------  278
_XP_012157903.1_  276  VPA----------  278
_XP_011185661.2_  274  EPI----------  276
_XP_050322757.1_  275  EPV----------  277
_XP_039962922.1_  275  EPV----------  277
_XP_050322739.1_  275  EPV----------  277
_XP_050322749.1_  275  EPV----------  277
_XP_050322724.1_  275  EPV----------  277
_XP_050322682.1_  275  EPV----------  277
_CAG9823112.1_    280  WPL----------  282
_KAG5873705.1_    275  WPA----------  277
_KAH8030364.1_    256  HPN----------  258
_XP_023013891.1_  275  WPS----------  277
```

```
_XP_028130780.2_  281  WP-----------  282
_KAG5873704.1_    280  WPA----------  282
_CAH1187690.1_    280  WPP----------  282
_CAH1098641.1_    280  WPP----------  282
_XP_023028116.1_  280  WPD----------  282
_XP_031630227.1_  286  T------------  286
_KAG5861047.1_    149  WPA----------  151
_KAF2880990.1_    278  LPL----------  280
_XP_018326789.1_  277  LPL----------  279
_XP_018326788.1_  277  LPL----------  279
_XP_031339184.1_  277  LPL----------  279
_XP_045476069.1_  278  LP-----------  279
_XP_044734149.1_  276  TPL----------  278
_XP_044734150.1_  276  TPL----------  278
_XP_044734148.1_  276  TPL----------  278
_XP_044760528.1_  278  LP-----------  279
_XP_044747918.1_  278  LPL----------  280
_XP_044747917.1_  278  LPD----------  280
_XP_045481017.1_  278  LPL----------  280
_XP_045481018.1_  278  LPL----------  280
_KAF5302274.1_    278  LPL----------  280
_KAF5308883.1_    278  LPL----------  280
_KRT79621.1_           -------------
_BAD20728.1_           -------------
_KAF2880988.1_    277  LPL----------  279
_CAH1403258.1_    236  TPL----------  238
_CAD6992094.1_    274  MPI----------  276
_XP_004537184.1_  274  MPI----------  276
_XP_018572644.1_  275  LPL----------  277
_RZB54393.1__     278  LPL----------  280
_XP_044255725.1_  278  LPL----------  280
_EFA02850.1_      278  LPL----------  280
_NP_001034507.1_  278  LPL----------  280
_CAH1370311.1_    278  LPL----------  280
_KAJ3630692.1_    278  LPL----------  280
_XP_044255724.1_  278  LPL----------  280
_KAH0811528.1_    278  LPL----------  280
_CAH7716406.1_    274  LPL----------  276
_CAH7716405.1_    274  LPL----------  276
```

CAH7716404.1	274	LPL----------	276
_XP_028130776.2_	275	WP-----------	276
CAH1985684.1	274	LPL----------	276
_XP_017781152.1_	278	LPL----------	280
_XP_017781151.1_	278	LPL----------	280
_XP_048522053.1_	275	LPL----------	277
_XP_050303070.1_	290	LPL----------	292
CAG9762924.1	279	LPL----------	281
CAH0560654.1	280	LPL----------	282
CAH0560655.1	280	LPL----------	282
_XP_018572643.1_	274	LPL----------	276
_XP_030763656.1_	278	LPV----------	280
_XP_030763654.1_	278	LPV----------	280
_XP_018577945.1_	273	LPL----------	275
KAF7283276.1	278	LPI----------	280
KAF7283275.1	278	LPI----------	280
_XP_030763617.1_	277	LPA----------	279
_XP_030763619.1_	280	HPH----------	282
VEN60240.1	185	LPL----------	187
KOB78236.1	271	MPE----------	273
ERL85515.1	205	LPL----------	207
ENN78766.1	197	LPL----------	199
KAH1019371.1	197	LPL----------	199
KAI9565420.1	276	LPL----------	278
_XP_045024905.1_	276	LPL----------	278
_XP_046447094.1_	276	LPM----------	278
KZS08518.1	276	LPL----------	278
_XP_046636307.1_	276	LPM----------	278
EFX79013.1	276	LPM----------	278
_XP_032776606.2_	276	LPL----------	278
_XP_046447093.1_	276	LPM----------	278
_XP_046636306.1_	276	LPM----------	278
CAH0099799.1	278	LPM----------	280
KAJ1531033.1	270	TPK----------	272
ACJ36225.1	277	LPL----------	279
ACZ98167.1	277	LPL----------	279
AME17867.1	277	LPL----------	279
_XP_042868409.1_	277	LPL----------	279
_XP_045102711.1_	277	LPL----------	279

```
_XP_047487558.1_  277  LPL----------  279
_QBJ05651.1_      277  LPL----------  279
_AZK36046.1_      277  LPL----------  279
_XP_045581920.1_  277  LPL----------  279
_XP_050704598.1_  277  LPL----------  279
_XP_027237995.1_  277  LPL----------  279
_AXB88326.1_      277  LPL----------  279
_XP_045102712.1_  277  LPL----------  279
_MPC17461.1_      277  LPL----------  279
_XP_027237994.1_  277  LPL----------  279
_XP_027237996.1_  277  LPL----------  279
_XP_045102715.1_  277  LPL----------  279
_XP_053627990.1_  277  LPL----------  279
_XP_053627989.1_  277  LPL----------  279
_XP_053627983.1_  277  LPL----------  279
_KAB7495058.1_    277  LPM----------  279
_KAF2353654.1_    277  LPL----------  279
_ANH22235.1_      277  LPL----------  279
_CAD7275741.1_    277  HPL----------  279
_XP_042243403.1_  277  LPL----------  279
_QTC09942.1_           -------------
_XP_018023738.1_  256  LPL----------  258
_XP_019877552.1_  276  WPL----------  278
_XP_049825875.1_  274  WPS----------  276
_CAD7649074.1_    277  LPK----------  279
_CAD7630012.1_    277  LPK----------  279
_XP_054165343.1_  278  LPK----------  280
_XP_044254219.1_  276  LPL----------  278
_XP_053201827.1_  277  LPK----------  279
_XP_015786990.1_  277  LPK----------  279
_KAI1295507.1_    277  IPR----------  279
_RWS07527.1_      277  LPR----------  279
_RWS29859.1_      277  FPR----------  279
_XP_053201825.1_  296  LPK----------  298
_XP_054713467.1_  271  IA-----------  272
_XP_046915158.1_  280  TPR----------  282
_KAJ6217218.1_    280  TPR----------  282
_KAI2801352.1_    280  TPR----------  282
_KAH9406651.1_    279  TPR----------  281
```

_XP_017483538.1_	279	TPR----------	281
KPM10359.1	278	TPR----------	280
UXI17582.1	278	TPR----------	280
KAF7488016.1	278	TPR----------	280
KAH9423377.1	281	TPR----------	283
OTF73464.1	280	TPR----------	282
_XP_037072056.1_	277	TPV----------	279
_XP_037083492.1_	277	TPL----------	279
_XP_037083508.1_	277	TPL----------	279
_XP_043215018.1_	277	TPL----------	279
KAF0314765.1	277	TPL----------	279
_XP_043215020.1_	277	TPL----------	279
_XP_043189654.1_	277	TPV----------	279
KAF0293344.1	263	TPV----------	265
_XP_011496609.1_		-------------	
AGS12619.1	277	LPL----------	279
TRY78048.1	277	LPL----------	279
_XP_040576307.1_	277	LPL----------	279
CAB4056363.1	277	LPL----------	279
ALF84898.1	277	TPI----------	279
_XP_023349538.1_	274	LPL----------	276
_XP_004522582.1_	277	MPA----------	279
CAD7013622.1	277	MPA----------	279
_XP_042902217.1_	276	LPM----------	278
_XP_042902218.1_	276	LPM----------	278
_XP_035215470.1_	276	LPM----------	278
GBN14612.1	275	LPM----------	277
KAF8795965.1	276	LPM----------	278
KAF8795964.1	276	LPM----------	278
GFY66827.1	276	LPM----------	278
GFW66965.1	276	LPM----------	278
GFQ85823.1	276	LPM----------	278
GFT35953.1	276	LPM----------	278
_XP_054714028.1_	276	LPM----------	278
KAG8194583.1	275	LPM----------	277
GIY09899.1	275	LPM----------	277
GIY93593.1	275	LPM----------	277
_XP_042902221.1_	286	LPM----------	288
_XP_046962867.1_	277	IPE----------	279

```
_XP_047530540.1_  277  IPE----------  279
_XP_026486466.1_  277  IPE----------  279
_XP_047530539.1_  277  IPE----------  279
_XP_047530538.1_  277  IPE----------  279
_XP_047530537.1_  277  IPE----------  279
_XP_026486464.1_  277  IPE----------  279
_XP_047530541.1_  277  IPE----------  279
_XP_050347286.1_  277  IPE----------  279
_XP_050347283.1_  277  IPE----------  279
_XP_032515351.1_  278  IPE----------  280
_OWR46016.1_      278  IPE----------  280
_XP_032515346.1_  278  IPE----------  280
_CAG9579094.1_    278  IPE----------  280
_CAG9579091.1_    278  IPE----------  280
_XP_050668638.1_  276  VPD----------  278
_XP_050668632.1_  276  VPD----------  278
_XP_045495329.1_  276  IPD----------  278
_XP_038214802.1_  276  IPD----------  278
_CAH0714714.1_    277  IPE----------  279
_CAH0714713.1_    277  IPE----------  279
_CAH4029256.1_    276  IAD----------  278
_XP_047513081.1_  276  IA-----------  277
_XP_045529569.1_  276  IA-----------  277
_XP_045487500.1_  276  IA-----------  277
_XP_047513077.1_  276  IA-----------  277
_CAH4029257.1_    276  IA-----------  277
_XP_045487498.1_  276  IA-----------  277
_CAF4952989.1_    276  IA-----------  277
_XP_045447470.1_  277  IPE----------  279
_CAH2108688.1_    216  IPE----------  218
_CAH0714724.1_    274  MA-----------  275
_XP_034827218.1_  277  IPS----------  279
_XP_045768234.1_  277  IPS----------  279
_XP_034827214.1_  277  IPS----------  279
_XP_045768230.1_  277  IPS----------  279
_XP_039751073.1_  277  IPN----------  279
_XP_023942270.1_  277  IPN----------  279
_XP_023942268.1_  277  IPN----------  279
_XP_039751072.1_  277  IPN----------  279
```

```
_XP_023942266.1_  277  IPN----------  279
_CAH2240541.1_    264  IPN----------  266
_XP_041985045.1_  278  TPD----------  280
_XP_041985024.1_  278  TPD----------  280
_XP_049868286.1_  285  IPE----------  287
_XP_026314794.1_  285  IPE----------  287
_XP_047994271.1_  276  TPD----------  278
_KAJ2950301.1_    282  IPD----------  284
_XP_031784347.2_  270  IPE----------  272
_KAG7305875.1_    275  LPS----------  277
_WDD23886.1_      275  LPS----------  277
_XP_048480424.1_  275  LPS----------  277
_XP_048480423.1_  275  LPS----------  277
_XP_048480422.1_  275  LPS----------  277
_XP_048480421.1_  275  LPS----------  277
_KAG7305876.1_    275  LPS----------  277
_CAG9129242.1_    275  LPS----------  277
_XP_048480420.1_  275  LPS----------  277
_GBP53004.1_      149  I------------  149
_XP_045534737.1_  276  IA-----------  277
_XP_013180060.1_  276  TA-----------  277
_XP_013180043.1_  276  TA-----------  277
_XP_013180068.1_  276  TA-----------  277
_XP_045534736.1_  276  IA-----------  277
_KPJ06840.1_      276  IA-----------  277
_KPJ05518.1_      276  TA-----------  277
_CAG4934519.1_    276  T------------  276
_XP_013149112.1_       -------------
_CAH2077165.1_    288  T------------  288
_CAH2077167.1_    288  T------------  288
_XP_037051415.1_  270  VPD----------  272
_XP_037051414.1_  268  VPD----------  270
_XP_022815079.1_  276  YPD----------  278
_CAB3516522.1_    276  YPD----------  278
_XP_050562120.1_  276  YPD----------  278
_XP_050562121.1_  276  YPD----------  278
_QHD56218.1_      276  YPD----------  278
_AIA24466.1_      276  YPD----------  278
_XP_022815076.1_  276  YPD----------  278
```

```
_QHD56217.1_       276  YPD----------  278
_XP_050562118.1_   276  YPD----------  278
_AIA24467.1_       276  YPD----------  278
_XP_047019808.1_   276  IPE----------  278
_AEO51736.1_       276  IPE----------  278
_XP_049696680.1_   276  IPE----------  278
_XP_049696682.1_   276  IPE----------  278
_XP_049696676.1_   276  IPE----------  278
_XP_026744931.1_   276  IPD----------  278
_XP_026732895.1_   276  IPD----------  278
_CAH0581650.1_     276  IPD----------  278
_KAH9640014.1_     321  YPD----------  323
_PZC78315.1_       150  IPE----------  152
_KAF9795796.1_     138  YPD----------  140
_AEC52926.1_       276  YPE----------  278
_KAH7940301.1_     264  HPN----------  266
_ADK39025.1_       275  IPE----------  277
_XP_037298148.1_   275  IPE----------  277
_RVE50742.1_       271  IPE----------  273
_CAH0692665.1_     271  IPE----------  273
_XP_028166351.1_   275  IPN----------  277
_XP_028166291.1_   275  IPN----------  277
_CAB3235314.1_     276  TPE----------  278
_KAJ0175867.1_     278  IPE----------  280
_XP_028031027.1_   276  IPD----------  278
_NP_001166296.1_   276  IPD----------  278
_XP_037870194.1_   276  IPD----------  278
_XP_037870193.1_   276  IPD----------  278
_XP_028031025.1_   276  IPD----------  278
_XP_013187225.1_   273  IPE----------  275
_XP_052755011.1_   274  IPA----------  276
_XP_053613182.1_   273  IPD----------  275
_XP_053613180.1_   273  IPD----------  275
_CAG9785863.1_     269  IPQ----------  271
_KAG6461275.1_     204  --T----------  204
_KAG6461281.1_     204  --T----------  204
_XP_037302137.1_   272  HPN----------  274
_KAG6461269.1_     272  HPN----------  274
_KAG4069896.1_     266  VPD----------  268
```

_XP_044003989.1_	283	VPD----------	285
_XP_045768240.1_	277	IPS----------	279
KAG9509099.1	280	LP-----------	281
_XP_013118267.1_		-------------	
_XP_005183026.2_	275	SQVSDPLHFEFYP	287
_XP_050303432.1_	289	LYLS---------	292
TMW48950.1	276	YPD----------	278
_XP_037882085.1_	275	YS-----------	276
KAI8126541.1	277	HPD----------	279
KAI8126540.1	276	HPD----------	278
_XP_037811156.1_	276	HPD----------	278
_XP_046812595.1_	276	HPD----------	278
_XP_046812596.1_	276	HPD----------	278
_XP_046812591.1_	276	HPD----------	278
_XP_046812590.1_	276	HPD----------	278
KNC34143.1	276	HPD----------	278
_XP_046812589.1_	276	HPD----------	278
KAI8126539.1	276	HPD----------	278
_XP_037958917.1_	278	YPD----------	280
_XP_037929595.1_	280	HPD----------	282
_XP_037957513.1_	278	HPD----------	280
_XP_037958115.1_	280	HPD----------	282
_XP_037958114.1_	280	HPD----------	282
_XP_037957511.1_	278	HPD----------	280
_XP_037958918.1_	278	YS-----------	279
RXG71823.1	241	YP-----------	242
KAB7494622.1	218	YPI----------	220
KAI9584339.1	275	SPS----------	277
_XP_037884983.1_	275	SPS----------	277
_XP_054744821.1_	274	H------------	274
_XP_053961676.1_		-------------	
_XP_053961674.1_		-------------	
_XP_004526634.2_	274	H------------	274
CAD6998451.1	274	H------------	274
_XP_012157894.1_		-------------	
_XP_028894493.1_		-------------	
_XP_036327693.1_		-------------	
_XP_017471333.1_		-------------	
_XP_039952608.1_	274	H------------	274

_XP_050320023.1_	274	H-------------	274
_XP_036225572.1_	274	H-------------	274
_XP_039952607.1_	274	H-------------	274
_XP_036225564.1_	274	H-------------	274
_XP_036225569.1_	274	H-------------	274
_XP_050320021.1_	274	H-------------	274
_XP_039952606.1_	274	H-------------	274
_XP_011207860.1_	274	H-------------	274
_XP_011207859.1_	274	H-------------	274
_XP_029407466.1_	274	H-------------	274
_XP_019847003.2_		--------------	
_XP_014086488.2_		--------------	
_XP_049317393.1_		--------------	
_XP_039952604.1_		--------------	
_XP_018787557.1_		--------------	
OQR68950.1		--------------	
_XP_003741763.1_		--------------	
_XP_022699297.1_		--------------	
_XP_022665229.1_		--------------	
_XP_022665225.1_		--------------	
_XP_022665224.1_		--------------	
_XP_033219687.1_	276	LPL-----------	278
_XP_033227990.1_	276	LPL-----------	278
_XP_043466013.1_	275	LPM-----------	277
_XP_051172671.1_	275	LPV-----------	277
CAD7643228.1		--------------	
_XP_054157157.1_	278	TPV-----------	280
CAD7645633.1	275	LPR-----------	277
CAD7632611.1	216	LPD-----------	218
KAJ6223158.1	278	F-------------	278
KAI2804035.1	278	F-------------	278
KAH9398209.1		--------------	
GFY77286.1	276	LPL-----------	278
GFQ73388.1	276	HPL-----------	278
GFT25575.1	276	LPL-----------	278
KAF8763655.1	276	LPL-----------	278
GFX76677.1	276	LPL-----------	278
_XP_035207052.1_	276	HPL-----------	278
KFM74898.1	276	HPL-----------	278

```
_XP_035207059.1_  276  HPL----------  278
_XP_035207066.1_  276  HPL----------  278
_BBD75287.1_      276  IPL----------  278
_XP_042900952.1_  276  IPL----------  278
_KAG8201816.1_    275  FP-----------  276
_GIX87435.1_      286  LPL----------  288
_GBL81029.1_      276  LPL----------  278
_GIY01087.1_      276  LPL----------  278
_XP_054715436.1_  277  LPL----------  279
_XP_044576112.1_  274  TP-----------  275
_XP_008556435.3_  272  TP-----------  273
_CAD6234446.1_    274  TP-----------  275
_XP_044284598.1_  271  LPE----------  273
_XP_033794511.1_  271  LPD----------  273
_XP_033794512.1_  271  LPD----------  273
_XP_029449578.1_  271  LPD----------  273
_XP_030052048.1_  271  LPD----------  273
_XP_007423639.1_  271  LPE----------  273
_XP_039203483.1_  271  LPE----------  273
_XP_034283206.1_  271  LPD----------  273
_XP_033001023.1_  271  LPE----------  273
_XP_053239675.1_  271  LPE----------  273
_XP_034966804.1_  271  LPE----------  273
_XP_028580982.1_  260  LPE----------  262
_XP_042302822.1_  272  LPE----------  274
_XP_015280979.1_  260  LPE----------  262
_XP_048363544.1_  271  LPE----------  273
_KAF7252806.1_    245  LPE----------  247
_XP_034958398.1_       -------------
_CAD5116312.1_    274  HPN----------  276
_KAJ1519398.1_    269  LPA----------  271
_XP_053103001.1_  271  LPE----------  273
_XP_032070730.1_  271  LPD----------  273
_XP_013922116.1_  271  LPD----------  273
_XP_015667999.1_  271  LPE----------  273
_XP_026551846.1_  271  LPV----------  273
_XP_026532518.1_  271  LPV----------  273
_KAJ7341301.1_         -------------
_KAJ6667038.1_    260  LPE----------  262
```

```
_XP_003227818.2_  260  LPE----------  262
_XP_020633036.1_  273  LPE----------  275
_XP_054842059.1_  271  LPE----------  273
_XP_005238641.2_  260  LPD----------  262
_XP_005445671.2_  260  LPD----------  262
_XP_054050029.1_  260  LPD----------  262
_XP_013810845.1_  260  LPD----------  262
_XP_026702002.1_  260  LPD----------  262
_XP_025892242.1_  260  LPD----------  262
_XP_005507389.1_  260  LPD----------  262
_XP_032911247.1_  260  LPD----------  262
_XP_031959657.1_  260  LPD----------  262
_XP_041905291.1_  260  LPD----------  262
_XP_051648836.1_  260  LPD----------  262
_XP_054239488.1_  260  LPD----------  262
_XP_039914164.1_  260  LPD----------  262
_XP_005422689.1_  260  LPD----------  262
_KAF2987757.1_    260  LPD----------  262
_XP_038665087.1_  272  LPD----------  274
_GCB59945.1_      272  LPD----------  274
_XP_041065616.1_  268  LPD----------  270
_XP_007890564.1_  272  LPD----------  274
_XP_051868609.1_  264  LPD----------  266
_XP_032881051.1_  271  LPD----------  273
_XP_048456938.1_  181  LPD----------  183
_KAJ1155552.1_    271  LPD----------  273
_KAH0622877.1_         -------------
_CAI5653329.1_    273  LPD----------  275
_CAI5653328.1_    273  LPD----------  275
_NXS54806.1_      278  LPD----------  280
_XP_009504395.1_  268  LPD----------  270
_NXJ41381.1_      271  LPD----------  273
_NXT24719.1_      271  LPD----------  273
_NXK23502.1_      271  LPD----------  273
_NXK03070.1_      271  LPD----------  273
_XP_037260944.1_  271  LPD----------  273
_XP_040467369.1_  271  LPD----------  273
_NXY71491.1_      271  LPD----------  273
_NXN54417.1_      271  LPD----------  273
```

```
_NXX19490.1_      271  LPD----------  273
_NXL47199.1_      271  LPD----------  273
_XP_050749583.1_  271  LPD----------  273
_NXD76242.1_      271  LPD----------  273
_KFZ56827.1_      271  LPD----------  273
_NXH67691.1_      271  LPD----------  273
_NXS45828.1_      271  LPD----------  273
_KFP52034.1_      271  LPD----------  273
_NWH44485.1_      271  LPD----------  273
_NWW85416.1_      271  LPD----------  273
_NWU66320.1_      271  LPD----------  273
_XP_025919906.1_  271  LPD----------  273
_KAF1483947.1_    271  LPD----------  273
_NXQ87688.1_      271  LPD----------  273
_NXX07562.1_      271  LPD----------  273
_XP_009280788.2_  271  LPD----------  273
_XP_014821857.1_  271  LPD----------  273
_NWU51443.1_      271  LPD----------  273
_NXV35521.1_      271  LPD----------  273
_KFV80780.1_      271  LPD----------  273
_XP_032039522.1_  271  LPD----------  273
_NWT43027.1_      271  LPD----------  273
_KAF1651581.1_    271  LPD----------  273
_NXU26374.1_      271  LPD----------  273
_NWX46229.1_      271  LPD----------  273
_PKU40340.1_      271  LPD----------  273
_XP_027309078.1_  271  LPD----------  273
_KAF1573683.1_    271  LPD----------  273
_KAF1578928.1_    271  LPD----------  273
_NXN47104.1_      271  LPD----------  273
_NXV20393.1_      271  LPD----------  273
_KAF1551267.1_    271  LPD----------  273
_KAF1406210.1_    271  LPD----------  273
_NXV43201.1_      271  LPD----------  273
_NWQ98669.1_      271  LPD----------  273
_XP_052639968.1_  271  LPD----------  273
_XP_010573143.1_  271  LPD----------  273
_KAF1479604.1_    271  LPD----------  273
_NXG89109.1_      271  LPD----------  273
```

```
_KFQ89757.1_      271  LPD----------  273
_NWZ21787.1_      271  LPD----------  273
_NXI74048.1_      271  LPD----------  273
_NWX76247.1_      271  LPD----------  273
_NXF29788.1_      271  LPD----------  273
_KAF1444142.1_    271  LPD----------  273
_KFV20215.1_      271  LPD----------  273
_NXI44657.1_      271  LPD----------  273
_NXJ92321.1_      271  LPD----------  273
_NWZ49902.1_      271  LPD----------  273
_NWY59444.1_      271  LPD----------  273
_KFW64019.1_      271  LPD----------  273
_NXJ55634.1_      271  LPD----------  273
_KAF1513372.1_    271  LPD----------  273
_NXW14598.1_      271  LPD----------  273
_NXC74247.1_      271  LPD----------  273
_NXT57254.1_      271  LPD----------  273
_XP_047916679.1_  271  LPD----------  273
_XP_029879190.1_  271  LPD----------  273
_NXH09241.1_      271  LPD----------  273
_XP_040407374.1_  271  LPD----------  273
_KFW76037.1_      271  LPD----------  273
_KFP65945.1_      271  LPD----------  273
_NXX55682.1_      271  LPD----------  273
_XP_035178333.1_  271  LPD----------  273
_NXE47317.1_      271  LPD----------  273
_XP_035408877.1_  271  LPD----------  273
_KGL94141.1_      271  LPD----------  273
_NXN68403.1_      271  LPD----------  273
_NXW28331.1_      271  LPD----------  273
_XP_049657509.1_  271  LPD----------  273
_XP_049657511.1_  271  LPD----------  273
_NXG30098.1_      271  LPD----------  273
_NXV86645.1_      271  LPD----------  273
_NXX83200.1_      271  LPD----------  273
_NXI59898.1_      271  LPD----------  273
_NWU89521.1_      271  LPD----------  273
_NXK45490.1_      271  LPD----------  273
_NWR55599.1_      271  LPD----------  273
```

```
_NXL37824.1_      271  LPD----------  273
_NWI33553.1_      271  LPD----------  273
_KFQ74517.1_      271  LPD----------  273
_NXV82662.1_      271  LPD----------  273
_NWJ10087.1_      271  LPD----------  273
_NXN24483.1_      271  LPD----------  273
_NWR80208.1_      271  LPD----------  273
_NXT37538.1_      271  LPD----------  273
_XP_032844994.2_  271  LPD----------  273
_NXW47649.1_      271  LPD----------  273
_NXA40398.1_      271  LPD----------  273
_NXW64893.1_      271  LPD----------  273
_NXX89165.1_      271  LPD----------  273
_NXQ96911.1_      271  LPD----------  273
_KFP18273.1_      271  LPD----------  273
_NXC40882.1_      271  LPD----------  273
_NXE10305.1_      271  LPD----------  273
_KFP88087.1_      271  LPD----------  273
_NWW47871.1_      271  LPD----------  273
_NXJ61595.1_      271  LPD----------  273
_NXD13814.1_      271  LPD----------  273
_KFR16961.1_      271  LPD----------  273
_NWX24312.1_      271  LPD----------  273
_NXA28267.1_      271  LPD----------  273
_NXL66270.1_      271  LPD----------  273
_NXA49976.1_      271  LPD----------  273
_NXT77624.1_      271  LPD----------  273
_KFQ92336.1_      271  LPD----------  273
_NXE25013.1_      271  LPD----------  273
_NXE92240.1_      271  LPD----------  273
_NWH80943.1_      271  LPD----------  273
_NWI19058.1_      271  LPD----------  273
_NXG80610.1_      271  LPD----------  273
_NWR01486.1_      271  LPD----------  273
_NXK81337.1_      271  LPD----------  273
_NXS90563.1_      271  LPD----------  273
_NXC17430.1_      271  LPD----------  273
_NXE77245.1_      271  LPD----------  273
_KFQ29788.1_      272  LPD----------  274
```

```
_KFO89327.1_       271  LPD----------  273
_NXD64381.1_       271  LPD----------  273
_NXY45081.1_       271  LPD----------  273
_KGL72871.1_       271  LPD----------  273
_XP_053919504.1_   271  LPD----------  273
_NXL85459.1_       271  LPD----------  273
_NXO02254.1_       271  LPD----------  273
_NXJ76034.1_       271  LPD----------  273
_NXM93491.1_       271  LPD----------  273
_XP_030345800.1_   271  LPD----------  273
_XP_030302544.1_   271  LPD----------  273
_XP_030345801.1_   271  LPD----------  273
_KQK77724.1_       271  LPD----------  273
_NXP09913.1_       271  LPD----------  273
_NXR33018.1_       271  LPD----------  273
_NXM76881.1_       271  LPD----------  273
_NXS27423.1_       271  LPD----------  273
_NXM55821.1_       271  LPD----------  273
_XP_054678500.1_   271  LPD----------  273
_XP_015712248.1_   271  LPD----------  273
_NWX92058.1_       271  LPD----------  273
_XP_048793646.1_   271  LPD----------  273
_NXF04247.1_       271  LPD----------  273
_XP_042683331.1_   271  LPD----------  273
_NWV22147.1_       271  LPD----------  273
_NXA04361.1_       271  LPD----------  273
_XP_010116198.1_   277  LPD----------  279
_NXG59741.1_       271  LPD----------  273
_XP_042735326.1_   271  LPD----------  273
_NXM29886.1_       271  LPD----------  273
_NP_001161198.2_   271  LPD----------  273
_XP_031451029.1_   271  LPD----------  273
_NXR49262.1_       271  LPD----------  273
_XP_052555416.1_   271  LPD----------  273
_NWZ67085.1_       271  LPD----------  273
_P16236.2_         271  LPD----------  273
_XP_050566010.1_   271  LPD----------  273
_NWS67030.1_       271  LPD----------  273
_XP_005143850.3_   271  LPD----------  273
```

```
_XP_050566011.1_  271  LPD----------  273
_XP_050566009.1_  271  LPD----------  273
_NWR30963.1_      271  LPD----------  273
_NWS17896.1_      271  LPD----------  273
_XP_021248574.1_  271  LPD----------  273
_AAA48721.1_      271  LPD----------  273
_NWI54208.1_      271  LPD----------  273
_XP_027509532.1_  271  LPD----------  273
_NXP71979.1_      271  LPD----------  273
_NXX25352.1_      271  LPD----------  273
_NXI18618.1_      271  LPD----------  273
_NXS09623.1_      271  LPD----------  273
_NXU77980.1_      271  LPD----------  273
_XP_027509533.1_  271  LPD----------  273
_NWV71036.1_      271  LPD----------  273
_NXF89482.1_      271  LPD----------  273
_NWY39470.1_      271  LPD----------  273
_NXT59960.1_      271  LPD----------  273
_NWU74340.1_      271  LPD----------  273
_NXB84481.1_      271  LPD----------  273
_NWV73885.1_      271  LPD----------  273
_OPJ58894.1_      271  LPD----------  273
_XP_048152104.1_  271  LPD----------  273
_XP_054036037.1_  271  LPD----------  273
_NXR18149.1_      271  LPD----------  273
_NXP97533.1_      271  LPD----------  273
_NXJ05495.1_      271  LPD----------  273
_XP_032538932.1_  271  LPD----------  273
_NWI96387.1_      271  LPD----------  273
_NXR65995.1_      271  LPD----------  273
_NXK42398.1_      271  LPD----------  273
_XP_032538931.1_  271  LPD----------  273
_NXY33181.1_      271  LPD----------  273
_XP_050171009.1_  271  LPD----------  273
_NXO50939.1_      271  LPD----------  273
_NWZ32937.1_      271  LPD----------  273
_NXP01828.1_      271  LPD----------  273
_NXL00458.1_      272  LPD----------  274
_XP_010401310.4_  271  LPD----------  273
```

```
_NXH62149.1_       271  LPD----------  273
_NXG43824.1_       271  LPD----------  273
_XP_017677337.1_   271  LPD----------  273
_NXU09156.1_       271  LPD----------  273
_NXB38645.1_       271  LPD----------  273
_XP_027594504.1_   271  LPD----------  273
_NXM46269.1_       271  LPD----------  273
_NWS79257.1_       271  LPD----------  273
_NXB04839.1_       271  LPD----------  273
_NXC62865.1_       271  LPD----------  273
_NXN18277.1_       271  LPD----------  273
_XP_027760039.1_   271  LPD----------  273
_NXI90311.1_       271  LPD----------  273
_NWY17003.1_       271  LPD----------  273
_NXH97099.1_       271  LPD----------  273
_NWS97308.1_       271  LPD----------  273
_NWU45160.1_       271  LPD----------  273
_NXQ43004.1_       271  LPD----------  273
_XP_027549142.1_   271  LPD----------  273
_XP_017933082.2_   271  LPD----------  273
_NWV96326.1_       271  LPD----------  273
_NXP57783.1_       271  LPD----------  273
_NXY57955.1_       271  LPD----------  273
_AAA99433.1_       271  LPD----------  273
_NXY21218.1_       271  LPD----------  273
_NXX50824.1_       271  LPD----------  273
_XP_030124623.3_   271  LPD----------  273
_NXA92235.1_       271  LPD----------  273
_NWI05522.1_       271  LPD----------  273
_NWX29009.1_       271  LPD----------  273
_XP_027549144.1_   271  LPD----------  273
_NWW35420.1_       271  LPD----------  273
_NWV07969.1_       271  LPD----------  273
_NXH42214.1_       271  LPD----------  273
_NXQ18773.1_       271  LPD----------  273
_NWX10672.1_       271  LPD----------  273
_NXU50687.1_       271  LPD----------  273
_KFU84784.1_       271  LPD----------  273
_NXQ52438.1_       271  LPD----------  273
```

```
_NWW24536.1_      271  LPD----------  273
_NXF73449.1_      271  LPD----------  273
_XP_050827906.1_  271  LPD----------  273
_NWX65205.1_      271  LPD----------  273
_KFW81749.1_      271  LPD----------  273
_XP_014750593.1_  271  LPD----------  273
_NXJ17066.1_      271  LPD----------  273
_NWW67019.1_      271  LPD----------  273
_NXH03360.1_      271  LPD----------  273
_XP_037987525.1_  271  LPD----------  273
_NXL74522.1_      271  LPD----------  273
_NWQ60824.1_      271  LPD----------  273
_NXR70971.1_      271  LPD----------  273
_XP_051468947.1_  271  LPD----------  273
_NXB50199.1_      271  LPD----------  273
_NXP18380.1_      271  LPD----------  273
_NXI84319.1_      271  LPD----------  273
_NWH33757.1_      271  LPD----------  273
_NWR99781.1_      271  LPD----------  273
_NXW94404.1_      271  LPD----------  273
_CAA27220.1_      271  LPD----------  273
_XP_015477679.1_  271  LPD----------  273
_NWS32597.1_      271  LPD----------  273
_NXG22126.1_      271  LPD----------  273
_NWY97127.1_      271  LPD----------  273
_NXW80148.1_      271  LPD----------  273
_NXC27367.1_      271  LPD----------  273
_NWH83726.1_      271  LPD----------  273
_NXA73512.1_      271  LPD----------  273
_NWZ83230.1_      271  LPD----------  273
_XP_005525572.2_  271  LPD----------  273
_NXD20033.1_      271  LPD----------  273
_NXE37177.1_      271  LPD----------  273
_NWV44369.1_      271  LPD----------  273
_NXC81400.1_      271  LPD----------  273
_NXA67345.1_      271  LPD----------  273
_NXB55155.1_      271  LPD----------  273
_NXN81229.1_      271  LPD----------  273
_NXM15601.1_      271  LPD----------  273
```

```
_XP_054484850.1_  271  LPD----------  273
_NWU02685.1_      271  LPD----------  273
_XP_041273165.1_  271  LPD----------  273
_NWW80397.1_      271  LPD----------  273
_XP_036236540.1_  271  LPD----------  273
_NWV50957.1_      271  LPD----------  273
_NXT97287.1_      271  LPD----------  273
_XP_023778876.1_  271  LPD----------  273
_NXD42381.1_      271  LPD----------  273
_NXK69295.1_      271  LPD----------  273
_XP_049657510.1_  271  LPD----------  273
_NXV62539.1_      271  LPD----------  273
_NXB80231.1_      271  LPD----------  273
_NWU15026.1_      271  LPD----------  273
_NXL26104.1_      271  LPD----------  273
_NWW05001.1_      271  LPD----------  273
_RLW07261.1_      271  LPD----------  273
_NXR96625.1_      271  LPD----------  273
_XP_021383204.1_  271  LPD----------  273
_NXQ25764.1_      271  LPD----------  273
_NXO08412.1_      271  LPD----------  273
_NXI32280.1_      271  LPD----------  273
_NXG00288.1_      271  LPD----------  273
_XP_041329879.1_  271  LPD----------  273
_NXV03759.1_      271  LPD----------  273
_NWT16975.1_      271  LPD----------  273
_NWT67953.1_      271  LPD----------  273
_XP_005042765.2_  271  LPD----------  273
_XP_017600715.1_  271  LPD----------  273
_NXD93279.1_      271  LPD----------  273
_NXY02260.1_      271  LPD----------  273
_NXO84677.1_      271  LPD----------  273
_NXO64371.1_      271  LPD----------  273
_NXO42022.1_      271  LPD----------  273
_NWY65013.1_      271  LPD----------  273
_NXP90563.1_      271  LPD----------  273
_NWI73723.1_      271  LPD----------  273
_NXH30511.1_      271  LPD----------  273
_NWT77800.1_      271  LPD----------  273
```

```
_NWT34209.1_      271  LPD----------  273
_NWY29369.1_      271  LPD----------  273
_NXQ73821.1_      271  LPD----------  273
_XP_039572786.1_  271  LPD----------  273
_XP_030802035.1_  271  LPD----------  273
_NXE68211.1_      271  LPD----------  273
_NXS80652.1_      271  LPD----------  273
_NXO32431.1_      271  LPD----------  273
_NWR17317.1_      271  LPD----------  273
_NWZ89732.1_      271  LPD----------  273
_XP_032039523.1_  271  LPD----------  273
_KFO04362.1_      271  LPD----------  273
_XP_054146213.1_  271  LPD----------  273
_XP_005486916.2_  271  LPD----------  273
_KAJ7422946.1_    271  LPD----------  273
_NXC99388.1_      271  LPD----------  273
_RMC07975.1_      271  LPD----------  273
_NXT95159.1_      229  LPD----------  231
_XP_009957589.1_  229  LPD----------  231
_TRZ13602.1_      229  LPD----------  231
_NWR45911.1_      220  LPD----------  222
_KAF4799649.1_    229  LPD----------  231
_KAJ7420138.1_    265  LPD----------  267
_XP_054678501.1_  237  LPD----------  239
_NXP48685.1_           -------------
_XP_010704701.1_  188  LPD----------  190
_KFV10805.1_      180  LPD----------  182
_XP_009572567.1_  150  LPD----------  152
_NXF64277.1_      149  LPD----------  151
_KFV54465.1_      149  LPD----------  151
_KFV90717.1_      149  LPD----------  151
_KFQ03674.1_      149  LPD----------  151
_NXW01028.1_      149  LPD----------  151
_KFP32908.1_      149  LPD----------  151
_KFV93389.1_      149  LPD----------  151
_NWI63728.1_      149  LPD----------  151
_KAI6072063.1_    150  LPD----------  152
_KFQ58901.1_      149  LPD----------  151
_NXP33753.1_      149  LPD----------  151
```

_XP_009476353.1_	148	LPD----------	150
KFQ55903.1	149	LPD----------	151
_XP_042683349.1_	149	LPD----------	151
NXR08991.1	149	LPD----------	151
_XP_008947250.1_	149	LPD----------	151
KFP72464.1	149	LPD----------	151
KFV60523.1	149	LPD----------	151
NXT13165.1	149	LPD----------	151
NWT50506.1	149	LPD----------	151
OXB76139.1	147	LPD----------	149
_XP_050827907.1_	162	LPD----------	164
_XP_030302545.1_	157	LPD----------	159
_XP_054036038.1_	161	LPD----------	163
_XP_012788337.1_	271	LPD----------	273
_XP_008070154.1_	260	LPD----------	262
_XP_027477445.1_	260	LPD----------	262
_XP_025848473.1_	260	LPD----------	262
_XP_049640716.1_	271	LPD----------	273
_XP_023084089.1_	271	LPD----------	273
EHH22143.1	271	LPD----------	273
_XP_038504711.1_	271	LPD----------	273
_XP_038504703.1_	271	LPD----------	273
_XP_038504718.1_	271	LPD----------	273
_XP_048971856.1_	271	LPD----------	273
KAB1266826.1	271	LPD----------	273
_XP_048391424.1_	272	LPD----------	274
_XP_048391425.1_	272	LPD----------	274
GCC28026.1	272	LPD----------	274
_XP_043551991.1_	272	LPD----------	274
_XP_043551989.1_	272	LPD----------	274
_XP_054534389.1_	181	LPD----------	183
_XP_011531312.1_	181	LPD----------	183
_XP_021780541.1_	180	LPD----------	182
_XP_037862912.1_	201	LPD----------	203
_XP_015413428.1_	260	LPD----------	262
_XP_005884642.1_	260	LPD----------	262
KAF6105903.1	260	LPD----------	262
_XP_015413426.1_	260	LPD----------	262
_XP_014387539.1_	260	LPD----------	262

```
_XP_014444723.1_  260  LPD----------  262
_XP_023448058.1_  260  LPD----------  262
_EGW11604.1_      260  LPD----------  262
_XP_015985129.1_  260  LPD----------  262
_XP_012878014.1_  260  LPD----------  262
_KAG5211506.1_    239  LPD----------  241
_MXQ82539.1_      235  LPD----------  237
_XP_032729748.1_  224  LPD----------  226
_XP_032208113.1_  224  LPD----------  226
_XP_048208817.1_  236  LPD----------  238
_XP_038200333.1_  236  LPD----------  238
_P15307.2_        271  LPD----------  273
_XP_041524635.1_  260  LPD----------  262
_CAA33843.1_      269  LPD----------  271
_XP_028732210.2_  260  LPD----------  262
_XP_025848474.1_  260  LPD----------  262
_EDL97996.1_      260  LPD----------  262
_KAI4577886.1_    260  LPD----------  262
_KAG8512309.1_    243  LPD----------  245
_XP_047637971.1_  236  LPD----------  238
_XP_019491454.1_       -------------
_XP_043842482.1_  273  LPD----------  275
_XP_027721170.1_  273  LPD----------  275
_XP_036604632.1_  273  LPD----------  275
_XP_020841164.1_  273  LPD----------  275
_XP_051831295.1_  273  LPD----------  275
_XP_031806378.1_  273  LPD----------  275
_XP_051831294.1_  273  LPD----------  275
_XP_003339776.1_  273  LPD----------  275
_XP_044515413.1_  273  LPD----------  275
_XP_028928361.1_  273  LPD----------  275
_XP_038607403.1_  273  LPD----------  275
_XP_040849334.1_  149  LPD----------  151
_TKC46270.1_      147  LPD----------  149
_OWK10634.1_      148  LPD----------  150
_XP_036190342.1_  147  LPD----------  149
_KAB0395596.1_    144  LPD----------  146
_KAF6430170.1_    148  LPD----------  150
_XP_038948811.1_  149  LPD----------  151
```

```
_KAB1266828.1_    282  LPD----------  284
_NP_002899.1_     271  LPD----------  273
_XP_001159537.3_  271  LPD----------  273
_PNI66333.1_      271  LPD----------  273
_EHH55592.1_      271  LPD----------  273
_XP_003262470.1_  271  LPD----------  273
_BAG64854.1_      271  LPD----------  273
_XP_032034471.1_  271  LPD----------  273
_XP_037862911.1_  271  LPD----------  273
_XP_003830911.1_  271  LPD----------  273
_XP_004029331.1_  271  LPD----------  273
_XP_002812051.1_  271  LPD----------  273
_XP_011711623.1_  271  LPD----------  273
_XP_033095023.1_  271  LPD----------  273
_XP_002799280.1_  271  LPD----------  273
_XP_011853172.1_  271  LPD----------  273
_XP_050609443.1_  271  LPD----------  273
_XP_003908735.1_  271  LPD----------  273
_XP_025209693.1_  271  LPD----------  273
_XP_023084087.1_  271  LPD----------  273
_XP_021780539.1_  271  LPD----------  273
_XP_017704568.1_  271  LPD----------  273
_XP_010375811.1_  271  LPD----------  273
_XP_030776399.1_  271  LPD----------  273
_XP_011797211.1_  271  LPD----------  273
_XP_012332682.1_  271  LPD----------  273
_XP_010331875.2_  271  LPD----------  273
_XP_012332683.1_  271  LPD----------  273
_XP_017400475.1_  271  LPD----------  273
_XP_008568357.1_  271  LPD----------  273
_XP_012620284.1_  271  LPD----------  273
_XP_002709741.2_  271  LPD----------  273
_XP_022452943.1_  271  LPD----------  273
_XP_045405625.1_  271  LPD----------  273
_XP_004280707.1_  271  LPD----------  273
_XP_030733169.1_  271  LPD----------  273
_TEA23022.1_      271  LPD----------  273
_XP_012494629.1_  271  LPD----------  273
_XP_010950191.1_  271  LPD----------  273
```

```
_XP_024608785.1_  271  LPD----------  273
_XP_006201637.1_  271  LPD----------  273
_XP_019285354.1_  271  LPD----------  273
_XP_032509224.1_  271  LPD----------  273
_XP_047379355.1_  271  LPD----------  273
_XP_032981134.1_  271  LPD----------  273
_XP_005600018.1_  271  LPD----------  273
_XP_046517051.1_  271  LPD----------  273
_XP_007464205.1_  271  LPD----------  273
_XP_019572903.1_  271  LPD----------  273
_XP_007189755.1_  271  LPD----------  273
_XP_004397771.1_  271  LPD----------  273
_XP_004435713.1_  272  LPD----------  274
_XP_008698124.1_  271  LPD----------  273
_XP_023989255.1_  271  LPD----------  273
_XP_011233012.2_  271  LPD----------  273
_XP_025724444.1_  271  LPD----------  273
_XP_005626186.1_  271  LPD----------  273
_XP_045643991.1_  271  LPD----------  273
_XP_034857383.1_  271  LPD----------  273
_XP_027945501.1_  271  LPD----------  273
_XP_026341094.1_  271  LPD----------  273
_EFB29923.1_      271  LPD----------  273
_XP_045740897.1_  271  LPD----------  273
_CAD7677454.1_    271  LPD----------  273
_XP_035577553.1_  271  LPD----------  273
_XP_006733255.1_  271  LPD----------  273
_XP_032266820.1_  271  LPD----------  273
_XP_041611730.1_  271  LPD----------  273
_XP_048971854.1_  271  LPD----------  273
_XP_021554840.1_  271  LPD----------  273
_VCW78562.1_      271  LPD----------  273
_XP_034857381.1_  271  LPD----------  273
_XP_008524231.1_  268  LPD----------  270
_XP_045740895.1_  271  LPD----------  273
_XP_045834746.1_  271  LPD----------  273
_XP_035932906.1_  271  LPD----------  273
_XP_030883859.1_  271  LPD----------  273
_XP_032266818.1_  271  LPD----------  273
```

```
_XP_024408528.2_  271  LPD----------  273
_XP_004580347.1_  271  LPD----------  273
_XP_030166525.1_  271  LPD----------  273
_XP_035932904.1_  271  LPD----------  273
_XP_020040690.1_  271  LPD----------  273
_XP_053445604.1_  271  LPD----------  273
_XP_049507482.1_  271  LPD----------  273
_XP_015335127.1_  271  LPD----------  273
_XP_027790245.1_  271  LPD----------  273
_XP_003984077.1_  271  LPD----------  273
_XP_039096259.1_  271  LPD----------  273
_XP_007075434.1_  271  LPD----------  273
_XP_022360130.1_  271  LPD----------  273
_XP_047707378.1_  271  LPD----------  273
_XP_026243031.1_  271  LPD----------  273
_XP_037383340.1_  271  LPD----------  273
_XP_005322095.1_  271  LPD----------  273
_XP_005662584.1_  271  LPD----------  273
_XP_040080101.1_  271  LPD----------  273
_XP_017503999.1_  271  LPD----------  273
_XP_044119954.1_  271  LPD----------  273
_XP_032208110.1_  271  LPD----------  273
_XP_012586936.1_  271  LPD----------  273
_XP_032729745.1_  271  LPD----------  273
_XP_052504199.1_  271  LPD----------  273
_XP_054431389.1_  271  LPD----------  273
_XP_023576632.1_  271  LPD----------  273
_XP_004742070.1_  271  LPD----------  273
_XP_037663267.1_  271  LPD----------  273
_XP_004005978.1_  271  LPD----------  273
_XP_036781908.1_  271  LPD----------  273
_XP_040849333.1_  271  LPD----------  273
_XP_005686691.1_  271  LPD----------  273
_XP_023367900.1_  271  LPD----------  273
_KAF0879576.1_    271  LPD----------  273
_XP_029793284.1_  271  LPD----------  273
_NP_001179899.1_  271  LPD----------  273
_XP_020764718.1_  271  LPD----------  273
_XP_054431388.1_  271  LPD----------  273
```

```
_XP_037663265.1_  271  LPD----------  273
_XP_019825763.1_  271  LPD----------  273
_XP_005889096.1_  271  LPD----------  273
_XP_043324571.1_  271  LPD----------  273
_KAJ1078272.1_    271  LPD----------  273
_XP_036190339.1_  271  LPD----------  273
_XP_013005522.1_  271  LPD----------  273
_XP_006073936.1_  271  LPD----------  273
_XP_016069529.1_  271  LPD----------  273
_KAB0354136.1_    271  LPD----------  273
_XP_029424871.1_  271  LPD----------  273
_XP_036118792.1_  271  LPD----------  273
_XP_037019725.2_  270  LPD----------  272
_XP_047602572.1_  271  LPD----------  273
_XP_014586639.1_  271  LPD----------  273
_XP_046517052.1_  271  LPD----------  273
_XP_006909635.2_  271  LPD----------  273
_XP_011362222.1_  271  LPD----------  273
_XP_045684409.1_  271  LPD----------  273
_XP_048208816.1_  271  LPD----------  273
_XP_052590629.1_  271  LPD----------  273
_XP_032353499.1_  271  LPD----------  273
_XP_038200327.1_  271  LPD----------  273
_XP_051056550.1_  271  LPD----------  273
_XP_012805850.2_  271  LPD----------  273
_XP_016050906.1_  271  LPD----------  273
_XP_030166526.1_  271  LPD----------  273
_XP_036903878.1_  270  LPD----------  272
_CAH7004032.1_    271  LPD----------  273
_XP_051056549.1_  271  LPD----------  273
_XP_042547166.1_  271  LPD----------  273
_XP_054584456.1_  271  LPD----------  273
_XP_049996768.1_  271  LPD----------  273
_XP_027243805.1_  271  LPD----------  273
_XP_047707379.1_  271  LPD----------  273
_XP_026645370.1_  271  LPD----------  273
_XP_014302519.1_  271  LPD----------  273
_XP_049726303.1_  271  LPD----------  273
_XP_049726302.1_  271  LPD----------  273
```

```
_XP_012969026.1_  271  LPD----------  273
_XP_008524232.1_  268  LPD----------  270
_XP_051020904.1_  271  LPD----------  273
_XP_023590712.1_  271  LPD----------  273
_XP_027243803.1_  271  LPD----------  273
_XP_040589151.1_  271  LPD----------  273
_XP_023412959.1_  271  LPD----------  273
_XP_036057111.1_  271  LPD----------  273
_XP_027822602.1_  271  LPD----------  273
_XP_031322949.1_  271  LPD----------  273
_XP_028634966.1_  271  LPD----------  273
_XP_031531529.1_  271  LPD----------  273
_XP_006073937.1_  271  LPD----------  273
_XP_034364887.1_  271  LPD----------  273
_XP_021482246.1_  271  LPD----------  273
_KAF4016326.1_    271  LPD----------  273
_XP_023602319.1_  271  LPD----------  273
_XP_052054164.1_  271  LPD----------  273
_XP_036291310.1_  271  LPD----------  273
_XP_013370168.1_  271  LPD----------  273
_XP_023590722.1_  271  LPD----------  273
_XP_048291328.1_  271  LPD----------  273
_XP_032772910.1_  271  LPD----------  273
_XP_008768670.2_  271  LPD----------  273
_XP_014302521.1_  271  LPD----------  273
_XP_031194882.1_  271  LPD----------  273
_NP_033070.2_     271  LPD----------  273
_XP_036291312.1_  271  LPD----------  273
_XP_021033157.1_  271  LPD----------  273
_XP_015849136.1_  271  LPD----------  273
_XP_045150027.1_  271  LPD----------  273
_XP_021066684.1_  271  LPD----------  273
_XP_006881088.1_  271  LPD----------  273
_XP_008524234.1_  268  LPD----------  270
_XP_021482260.1_  271  LPD----------  273
_XP_007125500.1_  271  LPD----------  273
_XP_028352731.1_  271  LPD----------  273
_XP_028352733.1_  271  LPD----------  273
_XP_028352734.1_  271  LPD----------  273
```

```
_XP_028352736.1_  271  LPD----------  273
_XP_028352735.1_  271  LPD----------  273
_ERE88482.1_      271  LPD----------  273
_KAF3826750.1_    120  LPD----------  122
_OBS70420.1_      207  LPD----------  209
_XP_006878094.1_  234  LPD----------  236
_XP_008978905.3_  228  LPD----------  230
_XP_032147941.1_  228  LPD----------  230
_XP_008524233.1_  230  LPD----------  232
_XP_044628438.1_  228  LPD----------  230
_XP_010608101.1_  228  VPD----------  230
_KAF5918007.1_    230  LPD----------  232
_XP_021101942.1_  228  VPD----------  230
_ABM89308.1_           -------------
_MBW00678.1_           -------------
_NP_001096177.1_  271  LPD----------  273
_OCT77375.1_      271  LPD----------  273
_AAI70187.1_      271  LPD----------  273
_AAI69362.1_      271  LPD----------  273
_XP_041418586.1_  271  LPD----------  273
_XP_041418585.1_  271  LPD----------  273
_XP_041418587.1_  271  LPD----------  273
_XP_053316221.1_  271  LPD----------  273
_XP_053568018.1_  271  LAD----------  273
_KAG8443975.1_    271  LPD----------  273
_XP_044145067.1_  273  IP-----------  274
_KAG9484268.1_    273  IP-----------  274
_XP_040285660.1_  273  IP-----------  274
_XP_040285659.1_  273  IP-----------  274
_KAG8583825.1_    273  IP-----------  274
_XP_018411072.1_  273  IPY----------  275
_XP_040207073.1_  273  IP-----------  274
_NP_001081750.1_  268  LPD----------  270
_PIO39092.1_      149  IP-----------  150
_PIO39091.1_      149  IP-----------  150
_XP_041035877.1_  271  LPD----------  273
_XP_042202691.1_  271  LPH----------  273
_XP_048475509.1_  260  LPD----------  262
_XP_048380947.1_  260  LPD----------  262
```

_XP_051901152.1_	265	LPE----------	267
BAD60879.1	260	IPQ----------	262
_XP_050405370.1_	273	MPE----------	275
_XP_009063713.1_	273	MPD----------	275
CAB0044847.1	288	RPA----------	290
_XP_048259911.1_	274	MPE----------	276
ADI72431.1	274	MPE----------	276
CAG5133522.1	276	MPD----------	278
KAG5688355.1	184	MPE----------	186
AMT84567.1	274	TPD----------	276
_XP_052276378.1_	272	QPE----------	274
_XP_052276377.1_	271	QPE----------	273
AAK72690.1	275	MPE----------	277
_XP_011414731.2_	275	MPE----------	277
_XP_052699807.1_	275	MPE----------	277
_NP_001292261.1_	275	MPE----------	277
_XP_048738054.1_	275	MPE----------	277
_XP_052699808.1_	275	MPE----------	277
_XP_022332457.1_	275	MPE----------	277
ABL63469.1	274	MPE----------	276
_XP_033733822.1_	277	MPE----------	279
_XP_021347355.1_	277	MPE----------	279
UOA58607.1	276	MPE----------	278
_XP_021347356.1_	277	MPE----------	279
CAG2186872.1	275	MPE----------	277
_XP_052058915.1_	275	MPE----------	277
KAJ8309232.1	275	MPE----------	277
UZH84475.1	272	MPE----------	274
ALA99939.1	272	QPE----------	274
_XP_052767764.1_	272	QPE----------	274
ADD25211.1		-------------	
_XP_052224245.1_	270	EPE----------	272
KAJ8314234.1		-------------	
VDH95719.1	234	MPE----------	236
_XP_041352913.1_	274	MPE----------	276
_XP_046555158.1_	274	MPE----------	276
AAW33559.1	274	MPE----------	276
ABY87435.1	261	MPE----------	263
_XP_009028834.1_	274	IPQ----------	276

```
_XP_025106109.1_  274  MPE----------  276
_NP_001298191.1_  276  MPE----------  278
_KAH9519545.1_    276  MPE----------  278
_RUS74561.1_      277  MPE----------  279
_GFO11973.1_      276  MPE----------  278
_CAG5132534.1_         -------------
_GFR69641.1_           -------------
_XP_035824873.1_       -------------
_AAY27981.1_      274  QPQ----------  276
_XP_052827753.1_  269  QPE----------  271
_XP_029642523.1_  269  QPE----------  271
_CAE1288013.1_    233  QPQ----------  235
_ELU04547.1_      274  IPD----------  276
_KAI0227899.1_    271  IPE----------  273
_XP_044002103.1_       -------------
_XP_044002102.1_  277  IP-----------  278
_XP_044002099.1_  277  IP-----------  278
_XP_037026629.1_       -------------
_CAD7632570.1_    282  LPE----------  284
_CAD7650738.1_    284  LPD----------  286
_XP_054163335.1_       -------------
_CAD7629985.1_         -------------
_CAD7652069.1_    274  L------------  274
_XP_017954168.1_  276  YPN----------  278
_XP_017858583.1_  276  YPN----------  278
_XP_002001588.1_  276  YPN----------  278
_XP_017954163.1_  276  YPN----------  278
_XP_023164563.2_  276  YPN----------  278
_TDG46702.1_      276  YPN----------  278
_XP_030564898.1_  276  YPN----------  278
_XP_015024496.1_  276  YPN----------  278
_XP_034475238.1_  277  YPN----------  279
_XP_034475235.1_  277  YPN----------  279
_XP_017854415.1_  276  YPN----------  278
_XP_017854416.1_  276  YPN----------  278
_ALC40089.1_      276  YPN----------  278
_XP_030382983.1_  279  YPN----------  281
_XP_030382981.1_  279  YPN----------  281
_XP_001989397.1_  276  YPN----------  278
```

```
_XP_002067135.2_  277  YPN----------  279
_XP_034475239.1_  275  YPN----------  277
_KAH8411901.1_    277  YPN----------  279
_XP_016963143.1_  277  YPN----------  279
_XP_037712361.1_  277  YPN----------  279
_XP_016993627.2_  277  YPN----------  279
_XP_016963141.1_  277  YPN----------  279
_XP_016993624.2_  277  YPN----------  279
_XP_037712358.1_  277  YPN----------  279
_XP_001974449.1_  277  YPN----------  279
_AAM48404.1_      277  YPN----------  279
_NP_001162999.1_  277  YPN----------  279
_XP_026835878.1_  277  YPN----------  279
_AAA28465.1_      277  YPN----------  279
_XP_002038912.1_  277  YPN----------  279
_XP_033169558.1_  277  YPN----------  279
_EDX05310.1_      277  YPN----------  279
_NP_001162998.1_  277  YPN----------  279
_XP_032579766.1_  277  YPN----------  279
_XP_033169541.1_  277  YPN----------  279
_XP_034669502.1_  277  YPN----------  279
_XP_034125957.1_  277  YPN----------  279
_XP_034669501.1_  277  YPN----------  279
_XP_034125956.1_  277  YPN----------  279
_XP_034125955.1_  277  YPN----------  279
_XP_017152033.1_  277  YPN----------  279
_XP_017152031.1_  277  YPN----------  279
_XP_002014799.1_  277  YPN----------  279
_XP_026844902.1_  277  YPN----------  279
_XP_015035983.2_  277  YPN----------  279
_XP_017063896.1_  277  YPN----------  279
_XP_016935332.1_  277  YPN----------  279
_XP_017063894.2_  277  YPN----------  279
_XP_036668637.1_  277  YPN----------  279
_XP_016935310.1_  277  YPN----------  279
_KAH8298318.1_    277  YPN----------  279
_KAH8412187.1_    277  YPN----------  279
_KAH8278042.1_    276  YPK----------  278
_KAH8366423.1_    277  YPN----------  279
```

```
_XP_017044860.1_  278  YPN----------  280
_KAH8324526.1_    275  YPQ----------  277
_XP_017116817.1_  277  YPN----------  279
_XP_041566118.1_  276  YPN----------  278
_XP_052852584.1_  277  YPN----------  279
_XP_052852582.1_  277  YPN----------  279
_XP_017107132.2_  272  YPK----------  274
_XP_043069650.1_  272  YPK----------  274
_KAH8283158.1_    278  YPN----------  280
_XP_017025640.1_  276  YPN----------  278
_XP_020803071.1_  277  YPN----------  279
_XP_017025638.1_  277  YPN----------  279
_KAH8308067.1_    277  YPN----------  279
_KAH8362606.1_    277  YPN----------  279
_XP_020803068.1_  277  YPN----------  279
_XP_015052364.1_  277  FPN----------  279
_XP_002090469.1_  276  FPN----------  278
_XP_039480354.1_  276  FPN----------  278
_XP_039480352.1_  276  FPN----------  278
_XP_041450795.1_  277  FPN----------  279
_XP_044315461.1_  278  YPN----------  280
_XP_044315463.1_  277  YPN----------  279
_XP_001961710.2_  277  YPK----------  279
_XP_014761890.1_  277  YPK----------  279
_KAH8323309.1_    277  YPK----------  279
_XP_043646206.1_  276  FPN----------  278
_XP_043646204.1_  276  FPN----------  278
_AAQ65066.1_           -------------
_AAQ64900.1_           -------------
_AAQ64905.1_           -------------
_KAH8378209.1_    278  KPN----------  280
_KAH8293207.1_    276  RPN----------  278
_KAH8391978.1_    277  KPN----------  279
_XP_034101352.1_  277  KPN----------  279
_KAJ3658269.1_    279  LPL----------  281
_KAJ3658270.1_    279  LPV----------  281
_KAJ3658271.1_    279  LPL----------  281
_XP_015117085.1_  279  L------------  279
_XP_011307643.1_  279  V------------  279
```

```
_XP_011307741.1_  279  LPS----------  281
_XP_015119445.1_  279  LP-----------  280
_XP_052128594.1_  291  RPP----------  293
_XP_052128597.1_  282  RPP----------  284
_KAE8741315.1_         -------------
_XP_028982160.1_  282  EPA----------  284
_XP_011307645.1_  282  EP-----------  283
_XP_054725384.1_  272  KPV----------  274
_XP_053965385.1_  272  KPV----------  274
_XP_013149158.1_  281  TPA----------  283
_XP_045534655.1_  283  TPA----------  285
_KPJ06852.1_      283  TPA----------  285
_XP_013176418.1_  279  TPP----------  281
_OXU28425.1_      302  LPL----------  304
_XP_008216913.1_  283  LPL----------  285
_XP_008216914.1_  279  LPL----------  281
_XP_040572301.1_  272  IPP----------  274
_CAB4068408.1_    179  IPP----------  181
_KAF7993284.1_    259  HPE----------  261
_KAF7987489.1_    279  IPN----------  281
_KAF7994520.1_    281  TPN----------  283
_XP_044019368.1_       -------------
_XP_044019332.1_  179  VNF----------  181
_XP_044003986.1_  154  TPN----------  156
_XP_037042732.1_  280  VPD----------  282
_CAD7223243.1_    274  LPK----------  276
_XP_044732233.1_  281  KPN----------  283
_XP_012231885.1_  272  LPL----------  274
_XP_022814699.1   276  LPY----------  278
_CAB3516523.1_    276  LPY----------  278
_XP_035458245.2_  275  LPY----------  277
_KAF9795795.1_    264  LPY----------  266
_XP_014233257.1_  286  T------------  286
_XP_034324400.1_  263  TPD----------  265
_XP_052699907.1_  263  TPD----------  265
Consensus_aa:          hP...........
Consensus_ss:
```

RelA/p65

```
Conservation:              7775799959999979 99997799999759 75 777777        7  7 9  579779999  7
_XP_028917328.1_     1  VEIIEQPKQRGMRFRYKCEGRSAGSIPGERSTDTTKTHPTIK-------INGYVGPGKVRISLVTKEPPH   63
_XP_038621028.1_     1  VEIIEQPKQRGMRFRYKCEGRSAGSIPGERSTDTTKTHPTIK-------ISGYVGPGKVRISLVTKEPPH   63
_RLV71517.1_         1  -----------MRFRYKCEGRSAGSIPGEHSSESTKTHPTIR-------VNNYRGPGRVRVSLVTKDPPH   52
_XP_033367665.1_     1  -----------MRFRYKCEGRSAGSIPGEHSTETTKTHPTIR-------VNNYRGPGRVRVSLVTKDPPH   52
_OWK50979.1_            ----------------------------------------------------------------------
_XP_031363427.1_     1  --------------------------------------PTIR-------VNNYRGPGRVRVSLVTKDPPH   25
_XP_041319512.1_        ----------------------------------------------------------------------
_XP_054509575.1_     1  -----------MRFRYKCEGRSAGSIPGEHSTESTKTHPTIR-------VNNYRGPGRVRVSLVTKDPPH   52
_XP_039909596.1_     1  -----------MRFRYKCEGRSAGSIPGEHSTESTKTHPTIR-------VNNYRGPGRVRVSLVTKDPPH   52
_TRZ10035.1_         1  -----------MRFRYKCEGRSAGSIPGEHSTESTKTHPTIR-------VNNYRGPGRVRVSLVTKDPPH   52
_XP_031950790.1_     1  -----------MRFRYKCEGRSAGSIPGEHSTETTKTHPTIR-------VNNYRGPGRVRVSLVTKDPPH   52
_XP_050163562.1_     1  -----------MRFRYKCEGRSAGSIPGEHSSETTRTHPTIR-------VNNYRGPARVRVSLVTKDPPH   52
_XP_039428642.1_     1  -----------MRFRYKCEGRSAGSIPGEHSTETTKTHPTIR-------VKQLPGPGRVRVSLVTKDPPH   52
_XP_050840410.1_     1  VEILEQPKQRGMRFRYKCEGRSAGSIPGEHSTESTKTHPTIRVS---APVNNYRGPGRVRVSLVTKDPPH   67
_XP_054151317.1_     1  VEILEQPKQRGMRFRYKCEGRSAGSIPGERSTESAKTHPTIR-------VNNYRGPGRVRVSLVTKDPPH   63
_XP_050840409.1_     1  VEILEQPKQRGMRFRYKCEGRSAGSIPGEHSTESTKTHPTIR-------VNNYRGPGRVRVSLVTKDPPH   63
_XP_030825825.1_     1  VEILEQPKQRGMRFRYKCEGRSAGSIPGEHSAESTKTHPTIR-------VNNYRGPGRVRVSLVTKDPPH   63
_XP_032939479.1_     1  VEILEQPKQRGMRFRYKCEGRSAGSIPGEHSSETTKTHPTIR-------VNNYRGPGRVRVSLVTKDPPH   63
_XP_041567964.1_     1  VEILEQPKQRGMRFRYKCEGRSAGSIPGEHSSESTRTHPTIR-------VNNYRGPGRVRVSLVTKDPPH   63
_XP_048149083.1_     1  VEILEQPKQRGMRFRYKCEGRSAGSIPGEHSTETTKTHPTIR-------VNNYRGPGRVRVSLVTKDPPH   63
_XP_054374241.1_     1  VEILEQPKQRGMRFRYKCEGRSAGSIPGEHSTESTKTHPTIR-------VNNYRGPGRVRVSLVTKDPPH   63
_NXV55901.1_         1  VEILEQPKQRGMRFRYKCEGRSAGSIPGEHSTESTKTHPTIR-------VNNYRGPGRVRVSLVTKDPPH   63
_XP_005533697.1_     1  VEILEQPKQRGMRFRYKCEGRSAGSIPGEHSTETTKTHPTIR-------VNNYRGPGRVRVSLVTKDPPH   63
_XP_014748521.1_     1  VEILEQPKQRGMRFRYECEGRSAGSIPGEHSTETTKTHPTIR-------VNNYRGPGRVRVSLVTKDPPH   63
_RMB90491.1_         1  VEILEQPKQRGMRFRYKCEGRSAGSIPGEHSTESTKTHPTIR-------VNNYRGPGRVRVSLVTKDPPH   63
_XP_053857772.1_     1  VEILEQPKQRGMRFRYKCEGRSAGSIPGEHSTESTKTHPTIR-------VNNYRGPGRVRVSLVTKDPPH   63
_XP_029862760.1_        ----------------------------------------------------------------------
_XP_039575719.1_     1  -------------------------IPGEHSSESTRTHPTIR-------VNNYRGPGRVRVSLVTKDPPH   38
_XP_054664313.1_     1  VEILEQPKPRGMRFRYKCEGRSAGSIPGEHSTDTTKTHPTIR-------VNNYRGPGRVRVSLVTKEPPH   63
_XP_049691648.1_     1  VEILEQPKPRGMRFRYKCEGRSAGSIPGEHSTDTTKTHPTIR-------VNNYRGPGRVRVSLVTKEPPH   63
_XP_030331239.1_     1  VEILEQPKARGMRFRYKCEGRSAGSIPGEHSTESARTHPTIR-------VNNYRGPSRVRVSLVTKDPPH   63
_XP_010726496.2_        ----------------------------------------------------------------------
_XP_042747731.1_        ----------------------------------------------------------------------
_XP_031466227.1_     1  VEILEQPKQRGMRFRYKCEGRSAGSIPGEHSTDSARTHPTIR-------VNHYRGPGRVRVSLVTKDPPH   63
_XP_032297702.1_     1  VEILEQPKQRGMRFRYKCEGRSAGSIPGEHSTDSARTHPTIR-------VNHYRGPGRVRVSLVTKDPPH   63
_RMB90505.1_         1  -------------------------------ADGHKVYGAIL-------VNNYRGPGRVRVSLVTKDPRA   32
_KAI2561003.1_       1  -----------MRFRYKCEGRSAGSIPGERSTDTTKTHPTIK-------INGYTGPGTVRISLVTKDPPH   52
```

```
_KAF6105893.1_      1  VEIIEQPKQRGMRFRYKCEGRSAGSIPGERSTDTTKTHPTIK-------INGYTGPGTVRISLVTKDPPH   63
_PNJ38430.1_        1  VEIIEQPKQRGMRFRYKCEGRSAGSIPGERSTDTTKTHPTIK-------INGYTGPGTVRISLVTKDPPH   63
_PNI93749.1_        1  VEIIEQPKQRGMRFRYKCEGRSAGSIPGERSTDTTKTHPTIK-------INGYTGPGTVRISLVTKDPPH   63
_KAF6334124.1_      1  VEIIEQPKQRGMRFRYKCEGRSAGSIPGERSTDTTKTHPTIK-------INGYTGPGTVRISLVTKDPPH   63
_KAF6467432.1_      1  VEIIEQPKQRGMRFRYKCEGRSAGSIPGERSTDTTKTHPTIK-------INGYTGPGTVRISLVTKDPPH   63
_PNI93759.1_        1  VEIIEQPKQRGMRFRYKCEGRSAGSIPGERSTDTTKTHPTIK-------INGYTGPGTVRISLVTKDPPH   63
_PNJ38434.1_        1  VEIIEQPKQRGMRFRYKCEGRSAGSIPGERSTDTTKTHPTIK-------INGYTGPGTVRISLVTKDPPH   63
_KAH0508019.1_      1  VEIIEQPKQRGMRFRYKCEGRSAGSIPGERSTDTTKTHPTIKSTLYFGQINGYTGPGTVRISLVTKDPPH   70
_AAA36408.1_        1  VEIIEQPKQRGMRFRYKCEGRSAGSIPGERSTDTTKTHPTIK-------INGYTGPGTVRISLVTKDPPH   63
_XP_003274247.2_    1  VEIIEQPKQRGMRFRYKCEGRSAGSIPGERSTDTTKTHPTIK-------INGYTGPGTVRISLVTKDPPH   63
_XP_007981896.1_    1  VEIIEQPKQRGMRFRYKCEGRSAGSIPGERSTDTTKTHPTIK-------INGYTGPGTVRISLVTKDPPH   63
_XP_054296639.1_    1  VEIIEQPKQRGMRFRYKCEGRSAGSIPGERSTDTTKTHPTIK-------INGYTGPGTVRISLVTKDPPH   63
_XP_023042293.1_    1  VEIIEQPKQRGMRFRYKCEGRSAGSIPGERSTDTTKTHPTIK-------INGYTGPGTVRISLVTKDPPH   63
_XP_025211457.1_    1  VEIIEQPKQRGMRFRYKCEGRSAGSIPGERSTDTTKTHPTIK-------INGYTGPGTVRISLVTKDPPH   63
_XP_039326898.1_    1  VEIIEQPKQRGMRFRYKCEGRSAGSIPGERSTDTTKTHPTIK-------INGYTGPGTVRISLVTKDPPH   63
_XP_021781237.2_    1  VEIIEQPKQRGMRFRYKCEGRSAGSIPGERSTDTTKTHPTIK-------INGYTGPGTVRISLVTKDPPH   63
_XP_010364772.1_    1  VEIIEQPKQRGMRFRYKCEGRSAGSIPGERSTDTTKTHPTIK-------INGYTGPGTVRISLVTKDPPH   63
_XP_033063134.1_    1  VEIIEQPKQRGMRFRYKCEGRSAGSIPGERSTDTTKTHPTIK-------INGYTGPGTVRISLVTKDPPH   63
_XP_017392927.1_    1  VEIIEQPKQRGMRFRYKCEGRSAGSIPGERSTDTTKTHPTIK-------INGYTGPGTVRISLVTKDPPH   63
_XP_011897055.1_    1  VEIIEQPKQRGMRFRYKCEGRSAGSIPGERSTDTTKTHPTIK-------INGYTGPGTVRISLVTKDPPH   63
_XP_032138725.1_    1  VEIIEQPKQRGMRFRYKCEGRSAGSIPGERSTDTTKTHPTIK-------INGYTGPGTVRISLVTKDPPH   63
_CAA80524.2_        1  VEIIEQPKQRGMRFRYKCEGRSAGSIPGRRSTDTTKTHPTIK-------INGYTGPGTVRISLVTKDPPH   63
_XP_005577305.2_    1  VEIIEQPKQRGMRFRYKCEGRSAGSIPGERSTDTTKTHPTIK-------INGYTGPGTVRISLVTKDPPH   63
_BAE87977.1_        1  VEIIEQPKQRGMRFRYKCEGRSAGSIPGERSTDTTKTHPTIK-------INGYTGPGTVRISLVTKDPPH   63
_XP_023042294.1_    1  VEIIEQPKQRGMRFRYKCEGRSAGSIPGERSTDTTKTHPTIK-------INGYTGPGTVRISLVTKDPPH   63
_XP_050612617.1_    1  VEIIEQPKQRGMRFRYKCEGRSAGSIPGERSTDTTKTHPTIK-------INGYTGPGTVRISLVTKDPPH   63
_EHH56082.1_        1  VEIIEQPKQRGMRFRYKCEGRSAGSIPGERSTDTTKTHPTIK-------INGYTGPGTVRISLVTKDPPH   63
_XP_028689064.1_    1  VEIIEQPKQRGMRFRYKCEGRSAGSIPGERSTDTTKTHPTIK-------INGYTGPGTVRISLVTKDPPH   63
_XP_045411957.1_    1  VEIIEQPKQRGMRFRYKCEGRSAGSIPGERSTDTTKTHPTIK-------INGYTGPGTVRISLVTKDPPH   63
_XP_012520963.1_    1  VEIIEQPKQRGMRFRYKCEGRSAGSIPGERSTDTTKTHPTIK-------INGYTGPGTVRISLVTKDPPH   63
_XP_012633553.1_    1  VEIIEQPKQRGMRFRYKCEGRSAGSIPGERSTDTTKTHPTIK-------INGYTGPGTVRISLVTKDPPH   63
_XP_053417694.1_    1  VEIIEQPKQRGMRFRYKCEGRSAGSIPGERSTDTTKTHPTIK-------INGYTGPGTVRISLVTKDPPH   63
_XP_032976853.1_    1  VEIIEQPKQRGMRFRYKCEGRSAGSIPGERSTDTTKTHPTIK-------INGYTGPGTVRISLVTKDPPH   63
_XP_008576943.1_    1  VEIIEQPKQRGMRFRYKCEGRSAGSIPGERSTDTTKTHPTIK-------INGYTGPGTVRISLVTKDPPH   63
_XP_032345730.1_    1  VEIIEQPKQRGMRFRYKCEGRSAGSIPGERSTDTTKTHPTIK-------INGYTGPGTVRISLVTKDPPH   63
_XP_010955442.1_    1  VEIIEQPKQRGMRFRYKCEGRSAGSIPGERSTDTTKTHPTIK-------INGYTGPGTVRISLVTKDPPH   63
_XP_031303872.1_    1  VEIIEQPKQRGMRFRYKCEGRSAGSIPGERSTDTTKTHPTIK-------INGYTGPGTVRISLVTKDPPH   63
_XP_032976854.1_    1  VEIIEQPKQRGMRFRYKCEGRSAGSIPGERSTDTTKTHPTIK-------INGYTGPGTVRISLVTKDPPH   63
_XP_053780986.1_    1  VEIIEQPKQRGMRFRYKCEGRSAGSIPGERSTDTTKTHPTIK-------INGYTGPGTVRISLVTKDPPH   63
```

_XP_036115166.1_	1	VEIIEQPKQRGMRFRYKCEGRSAGSIPGERSTDTTKTHPTIK-------VNGYTGPGTVRISLVTKDPPH	63
_XP_043307320.1_	1	VEIIEQPKQRGMRFRYKCEGRSAGSIPGERSTDTTKTHPTIK-------INGYTGPGTVRISLVTKDPPH	63
EQB77721.1	1	VEIIEQPKQRGMRFRYKCEGRSAGSIPGERSTDTTKTHPTIK-------INGYTGPGTVRISLVTKDPPH	63
_NP_001107753.1_	1	VEIIEQPKQRGMRFRYKCEGRSAGSIPGERSTDTTKTHPTIK-------INGYTGPGTVRISLVTKDPPH	63
_XP_036785182.1_	1	VEIIEQPKQRGMRFRYKCEGRSAGSIPGERSTDTTKTHPTIK-------INGYTGPGTVRISLVTKDPPH	63
_XP_020762118.1_	1	VEIIEQPKQRGMRFRYKCEGRSAGSIPGERSTDTTKTHPTIK-------INGYTGPGTVRISLVTKDPPH	63
_XP_031546756.1_	1	VEIIEQPKQRGMRFRYKCEGRSAGSIPGERSTDTTKTHPTIK-------INGYTGPGTVRISLVTKDPPH	63
_XP_023989099.1_	1	VEIIEQPKQRGMRFRYKCEGRSAGSIPGERSTDTTKTHPTIK-------INGYTGPGTVRISLVTKDPPH	63
KAF4009900.1	1	VEIIEQPKQRGMRFRYKCEGRSAGSIPGERSTDTTKTHPTIK-------INGYTGPGTVRISLVTKDPPH	63
KAB0376704.1	1	VEIIEQPKQRGMRFRYKCEGRSAGSIPGERSTDTTKTHPTIK-------INGYTGPGTVRISLVTKDPPH	63
KAB0364713.1	1	VEIIEQPKQRGMRFRYKCEGRSAGSIPGERSTDTTKTHPTIK-------INGYTGPGTVRISLVTKDPPH	63
CBT01657.1	1	VEIIEQPKQRGMRFRYKCEGRSAGSIPGERSTDTTKTHPTIK-------INGYTGPGTVRISLVTKDPPH	63
_XP_036851744.1_	1	VEIIEQPKQRGMRFRYKCEGRSAGSIPGERSTDTTKTHPTIK-------INGYTGPGTVRISLVTKDPPH	63
_XP_007128247.1_	1	VEIIEQPKQRGMRFRYKCEGRSAGSIPGERSTDTTKTHPTIK-------INGYTGPGTVRISLVTKDPPH	63
_XP_003798713.1_	1	VEIIEQPKQRGMRFRYKCEGRSAGSIPGERSTDTTKTHPTIK-------INGYTGPGTVRISLVTKDPPH	63
_NP_001073711.1_	1	VEIIEQPKQRGMRFRYKCEGRSAGSIPGERSTDTTKTHPTIK-------INGYTGPGTVRISLVTKDPPH	63
_XP_006056522.1_	1	VEIIEQPKQRGMRFRYKCEGRSAGSIPGERSTDTTKTHPTIK-------INGYTGPGTVRISLVTKDPPH	63
_XP_019593326.1_	1	VEIIEQPKQRGMRFRYKCEGRSAGSIPGERSTDTTKTHPTIK-------INGYTGPGTVRISLVTKDPPH	63
_XP_005894159.1_	1	VEIIEQPKQRGMRFRYKCEGRSAGSIPGERSTDTTKTHPTIK-------INGYTGPGTVRISLVTKDPPH	63
ELR57541.1	1	VEIIEQPKQRGMRFRYKCEGRSAGSIPGERSTDTTKTHPTIK-------INGYTGPGTVRISLVTKDPPH	63
_XP_010836092.1_	1	VEIIEQPKQRGMRFRYKCEGRSAGSIPGERSTDTTKTHPTIK-------INGYTGPGTVRISLVTKDPPH	63
_XP_051049621.1_	1	VEIIEQPKQRGMRFRYKCEGRSAGSIPGERSTDTTKTHPTIK-------INGYTGPGTVRISLVTKDPPH	63
CBT01658.1	1	VEIIEQPKQRGMRFRYKCEGRSAGSIPGERSTDTTKTHPTIK-------INGYTGPGTVRISLVTKDPPH	63
_XP_015974930.1_	1	VEIIEQPKQRGMRFRYKCEGRSAGSIPGERSTDTTKTHPTIK-------INGYTGPGTVRISLVTKDPPH	63
_XP_027264386.1_	1	VEIIEQPKQRGMRFRYKCEGRSAGSIPGERSTDTTKTHPTIK-------INGYTGPGTVRISLVTKDPPH	63
_XP_040112158.1_	1	VEIIEQPKQRGMRFRYKCEGRSAGSIPGERSTDTTKTHPTIK-------INGYTGPGTVRISLVTKDPPH	63
_XP_052517633.1_	1	VEIIEQPKQRGMRFRYKCEGRSAGSIPGERSTDTTKTHPTIK-------INGYTGPGTVRISLVTKDPPH	63
_XP_017898873.1_	1	VEIIEQPKQRGMRFRYKCEGRSAGSIPGERSTDTTKTHPTIK-------INGYTGPGTVRISLVTKDPPH	63
_XP_020020484.1_	1	VEIIEQPKQRGMRFRYKCEGRSAGSIPGERSTDTTKTHPTIK-------INGYTGPGTVRISLVTKDPPH	63
_XP_047631223.1_	1	VEIIEQPKQRGMRFRYKCEGRSAGSIPGERSTDTTKTHPTIK-------INGYTGPGTVRISLVTKDPPH	63
_XP_007651509.2_	1	VEIIEQPKQRGMRFRYKCEGRSAGSIPGERSTDTTKTHPTIK-------INGYTGPGTVRISLVTKDPPH	63
_XP_027815096.1_	1	VEIIEQPKQRGMRFRYKCEGRSAGSIPGERSTDTTKTHPTIK-------INGYTGPGTVRISLVTKDPPH	63
_XP_034377707.1_	1	VEIIEQPKQRGMRFRYKCEGRSAGSIPGERSTDTTKTHPTIK-------INGYTGPGTVRISLVTKDPPH	63
ERE77884.1	1	VEIIEQPKQRGMRFRYKCEGRSAGSIPGERSTDTTKTHPTIK-------INGYTGPGTVRISLVTKDPPH	63
_XP_028626823.1_	1	VEIIEQPKQRGMRFRYKCEGRSAGSIPGERSTDTTKTHPTIK-------INGYTGPGTVRISLVTKDPPH	63
KAF5928161.1	1	VEIIEQPKQRGMRFRYKCEGRSAGSIPGERSTDTTKTHPTIK-------INGYTGPGTVRISLVTKDPPH	63
KAI5933031.1	1	VEIIEQPKQRGMRFRYKCEGRSAGSIPGERSTDTTKTHPTIK-------INGYTGPGTVRISLVTKDPPH	63
_XP_021007720.1_	1	VEIIEQPKQRGMRFRYKCEGRSAGSIPGERSTDTTKTHPTIK-------INGYTGPGTVRISLVTKDPPH	63
_XP_026252329.1_	1	VEIIEQPKQRGMRFRYKCEGRSAGSIPGERSTETTKTHPTIK-------INGYTGPGTVRISLVTKDPPH	63

_XP_054426842.1_ 1 VEIIEQPKQRGMRFRYKCEGRSAGSIPGERSTDTTKTHPTIK-------INGYTGPGTVRISLVTKDPPH 63
_XP_032747030.1_ 1 VEIIEQPKQRGMRFRYKCEGRSAGSIPGERSTDTTKTHPTIK-------INGYTGPGTVRISLVTKDPPH 63
_XP_049748463.1_ 1 VEIIEQPKQRGMRFRYKCEGRSAGSIPGERSTDTTKTHPTIK-------INGYTGPGTVRISLVTKDPPH 63
KAJ1065952.1 1 VEIIEQPKQRGMRFRYKCEGRSAGSIPGERSTDTTKTHPTIK-------INGYTGPGTVRISLVTKDPPH 63
_XP_052048317.1_ 1 VEIIEQPKQRGMRFRYKCEGRSAGSIPGERSTDTTKTHPTIK-------INGYTGPGTVRISLVTKDPPH 63
_XP_012876614.1_ 1 VEIMEQPKQRGMRFRYKCEGRSAGSIPGERSTDTTKTHPTIK-------INGYTGPGTVRISLVTKDPPH 63
_XP_020020482.1_ 1 VEIIEQPKQRGMRFRYKCEGRSAGSIPGERSTDTTKTHPTIK-------INGYTGPGTVRISLVTKDPPH 63
_XP_010597340.1_ 1 VEIIEQPKQRGMRFRYKCEGRSAGSIPGERSTDTTKTHPTIK-------INGYTGPGTVRISLVTKDPPH 63
_XP_026339000.2_ 1 VEIIEQPKQRGMRFRYKCEGRSAGSIPGERSTDTTKTHPTIK-------INGYTGPGTVRISLVTKDPPH 63
_XP_030689468.1_ 1 VEIIEQPKQRGMRFRYKCEGRSAGSIPGERSTDTTKTHPTIK-------INGYTGPGTVRISLVTKDPPH 63
_XP_049993276.1_ 1 VEIIEQPKQRGMRFRYKCEGRSAGSIPGERSTDTTKTHPTIK-------INGYTGPGTVRISLVTKDPPH 63
_XP_014700317.1_ 1 VEIIEQPKQRGMRFRYKCEGRSAGSIPGERSTDTTKTHPTIK-------INGYTGPGTVRISLVTKDPPH 63
_XP_021494126.1_ 1 VEIIEQPKQRGMRFRYKCEGRSAGSIPGERSTDTTKTHPTIK-------INGYTGPGTVRISLVTKDPPH 63
_XP_022447167.1_ 1 VEIIEQPKQRGMRFRYKCEGRSAGSIPGERSTDTTKTHPTIK-------INGYTGPGTVRISLVTKDPPH 63
_XP_042532137.1_ 1 VEIMEQPKQRGMRFRYKCEGRSAGSIPGERSTDTTKTHPTIK-------INGYTGPGTVRISLVTKDPPH 63
_XP_008820421.1_ 1 VEIIEQPKQRGMRFRYKCEGRSAGSIPGERSTDTTKTHPTIK-------INGYTGPGTVRISLVTKDPPH 63
_XP_023415352.1_ 1 VEIIEQPKQRGMRFRYKCEGRSAGSIPGERSTDTTKTHPTIK-------INGYTGPGTVRISLVTKDPPH 63
_XP_029414056.1_ 1 VEIIEQPKQRGMRFRYKCEGRSAGSIPGERSTDTTKTHPTIK-------INGYTGPGTVRISLVTKDPPH 63
_XP_024604853.1_ 1 VEIIEQPKQRGMRFRYKCEGRSAGSIPGERSTDTTKTHPTIK-------INGYTGPGTVRISLVTKDPPH 63
_XP_007462052.1_ 1 VEIIEQPKQRGMRFRYKCEGRSAGSIPGERSTDTTKTHPTIK-------INGYTGPGTVRISLVTKDPPH 63
_XP_006101621.1_ 1 VEIIEQPKQRGMRFRYKCEGRSAGSIPGERSTDTTKTHPTIK-------IIGYTGPGTVRISLVTKDPPH 63
_XP_005064049.1_ 1 VEIIEQPKQRGMRFRYKCEGRSAGSIPGERSTDTTKTHPTIK-------INGYTGPGTVRISLVTKDPPH 63
_XP_031245193.1_ 1 VEIIEQPKQRGMRFRYKCEGRSAGSIPGERSTDTTKTHPTIK-------INGYTGPGTVRISLVTKDPPH 63
ELK24332.1 1 VEIIEQPKQRGMRFRYKCEGRSAGSIPGERSTDTTKTHPTIK-------IIGYTGPGTVRISLVTKDPPH 63
_XP_041518057.1_ 1 VEIIEQPKQRGMRFRYKCEGRSAGSIPGERSTDTTKTHPTIK-------INGYTGPGTVRISLVTKDPPH 63
_XP_005351804.2_ 1 VEIIEQPKQRGMRFRYKCEGRSAGSIPGERSTDTTKTHPTIK-------INGYTGPGTVRISLVTKDPPH 63
EGW13067.1 1 VEIIEQPKQRGMRFRYKCEGRSAGSIPGERSTDTTKTHPTIK-------INGYTGPGTVRISLVTKDPPH 63
ERE77883.1 1 VEIIEQPKQRGMRFRYKCEGRSAGSIPGERSTDTTKTHPTIK-------INGYTGPGTVRISLVTKDPPH 63
KFO28401.1 1 VEIIEQPKQRGMRFRYKCEGRSAGSIPGERSTDTTKTHPTIK-------INGYTGPGTVRISLVTKDPPH 63
_XP_010634478.1_ 1 VEIIEQPKQRGMRFRYKCEGRSAGSIPGERSTDTTKTHPTIK-------INGYTGPGTVRISLVTKDPPH 63
OBS81518.1 1 VEIIEQPKQRGMRFRYKCEGRSAGSIPGERSTDTTKTHPTIK-------INGYTGPGTVRISLVTKDPPH 63
_XP_007462051.1_ 1 VEIIEQPKQRGMRFRYKCEGRSAGSIPGERSTDTTKTHPTIK-------INGYTGPGTVRISLVTKDPPH 63
_XP_014393535.1_ 1 VEIIEQPKQRGMRFRYKCEGRSAGSIPGERSTDTTKTHPTIK-------IIGYTGPGTVRISLVTKDPPH 63
_XP_048216512.1_ 1 VEIMEQPKQRGMRFRYKCEGRSAGSIPGERSTDTTKTHPTIK-------INGYTGPGTVRISLVTKDPPH 63
_XP_015424455.1_ 1 VEIIEQPKQRGMRFRYKCEGRSAGSIPGERSTDTTKTHPTIK-------IIGYTGPGTVRISLVTKDPPH 63
_XP_019501147.1_ 1 VEIIEQPKQRGMRFRYKCEGRSAGSIPGERSTDTTKTHPTIK-------INGYTGPGTVRISLVTKDPPH 63
_XP_004656738.2_ 1 VEIIEQPKQRGMRFRYKCEGRSAGSIPGERSTDTTKTHPTIK-------INGYTGPGTVRISLVTKDPPH 63
_XP_036909111.1_ 1 VEIIEQPKQRGMRFRYKCEGRSAGSIPGERSTDTTKTHPTIK-------INGYTGPGTVRISLVTKDPPH 63
_XP_036182518.1_ 1 VEIIEQPKQRGMRFRYKCEGRSAGSIPGERSTDTTKTHPTIK-------IIGYTGPGTVRISLVTKDPPH 63

```
_XP_012580591.1_     1  VEIIEQPKQRGMRFRYKCEGRSAGSIPGERSTDTTKTHPTIK-------INGYTGPGTVRISLVTKDPPH   63
_XP_016063765.1_     1  VEIIEQPKPRGMRFRYKCEGRSAGSIPGERSTDTTKTHPTIK-------INGYTGPGTVRISLVTKDPPH   63
_XP_021047054.1_     1  VEIIEQPKQRGMRFRYKCEGRSAGSIPGERSTDTTKTHPTIK-------INGYTGPGTVRISLVTKDPPH   63
_XP_023095836.1_     1  VEIIEQPKQRGMRFRYKCEGRSAGSIPGERSTDTTKTHPTIK-------INGYTGPGTVRISLVTKDPPH   63
_XP_045342734.1_     1  VEIIEQPKQRGMRFRYKCEGRSAGSIPGERSTDTTKTHPTIK-------INGYTGPGTVRISLVTKDPPH   63
_XP_053755429.1_     1  VEIIEQPKQRGMRFRYKCEGRSAGSIPGERSTDTTKTHPTIK-------INGYTGPGTVRISLVTKDPPH   63
_XP_022447166.1_     1  VEIIEQPKQRGMRFRYKCEGRSAGSIPGERSTDTTKTHPTIK-------INGYTGPGTVRISLVTKDPPH   63
_XP_004713741.1_     1  VEIIEQPKQRGMRFRYKCEGRSAGSIPGERSTDTTKTHPTIK-------INGYTGPGTVRISLVTKDPPH   63
_NP_954888.1_        1  VEIIEQPKQRGMRFRYKCEGRSAGSIPGERSTDTTKTHPTIK-------INGYTGPGTVRISLVTKDPPH   63
_XP_037002389.2_     1  VEIIEQPKQRGMRFRYKCEGRSAGSIPGERSTDTTKTHPTIK-------INGYTGPGTVRISLVTKDPPH   63
_XP_039100808.1_     1  VEIIEQPKQRGMRFRYKCEGRSAGSIPGERSTDTTKTHPTIK-------INGYTGPGTVRISLVTKDPPH   63
_XP_038170688.1_     1  VEIIEQPKQRGMRFRYKCEGRSAGSIPGERSTDTTKTHPTIK-------INGYTGPGTVRISLVTKDPPH   63
_XP_040495345.1_     1  VEIIEQPKQRGMRFRYKCEGRSAGSIPGERSTDTTKTHPTIK-------INGYTGPGTVRISLVTKDPPH   63
_XP_052568261.1_     1  VEIIEQPKQRGMRFRYKCEGRSAGSIPGERSTDTTKTHPTIK-------INGYTGPGTVRISLVTKDPPH   63
_XP_042760134.1_     1  VEIIEQPKQRGMRFRYKCEGRSAGSIPGERSTDTTKTHPTIK-------INGYTGPGTVRISLVTKDPPH   63
_XP_049472392.1_     1  VEIIEQPKQRGMRFRYKCEGRSAGSIPGERSTDTTKTHPTIK-------INGYTGPGTVRISLVTKDPPH   63
_XP_023593974.1_     1  VEIIEQPKQRGMRFRYKCEGRSAGSIPGERSTDTTKTHPTIK-------INGYTGPGTVRISLVTKDPPH   63
_XP_034501886.1_     1  VEIIEQPKQRGMRFRYKCEGRSAGSIPGERSTDTTKTHPTIK-------INGYTGPGTVRISLVTKDPPH   63
_XP_004393991.1_     1  VEIIEQPKQRGMRFRYKCEGRSAGSIPGERSTDTTKTHPTIK-------INGYTGPGTVRISLVTKDPPH   63
_XP_025860214.1_     1  VEIIEQPKQRGMRFRYKCEGRSAGSIPGERSTDTTKTHPTIK-------INGYTGPGTVRISLVTKDPPH   63
_XP_019501146.1_     1  VEIIEQPKQRGMRFRYKCEGRSAGSIPGERSTDTTKTHPTIK-------INGYTGPGTVRISLVTKDPPH   63
_XP_025734327.1_     1  VEIIEQPKQRGMRFRYKCEGRSAGSIPGERSTDTTKTHPTIK-------INGYTGPGTVRISLVTKDPPH   63
_XP_025301454.1_     1  VEIIEQPKQRGMRFRYKCEGRSAGSIPGERSTDTTKTHPTIK-------INGYTGPGTVRISLVTKDPPH   63
_KAG3284687.1_       1  VEIIEQPKQRGMRFRYKCEGRSAGSIPGERSTETTKTHPTIK-------INGYTGPGTVRISLVTKDPPH   63
_VFV23136.1_         1  VEIIEQPKQRGMRFRYKCEGRSAGSIPGERSTDTTKTHPTIK-------INGYTGPGTVRISLVTKDPPH   63
_XP_025771173.1_     1  VEIIEQPKQRGMRFRYKCEGRSAGSIPGERSTDTTKTHPTIK-------INGYTGPGTVRISLVTKDPPH   63
_XP_054580928.1_     1  VEIIEQPKQRGMRFRYKCEGRSAGSIPGERSTDTTKTHPTIK-------INGYTGPGTVRISLVTKDPPH   63
_EPQ06533.1_         1  VEIIEQPKQRGMRFRYKCEGRSAGSIPGERSTDTTKTHPTIK-------IIGYTGPGTVRISLVTKDPPH   63
_XP_034869141.1_     1  VEIIEQPKQRGMRFRYKCEGRSAGSIPGERSTDTTKTHPTIK-------INGYTGPGTVRISLVTKDPPH   63
_XP_042813589.1_     1  VEIIEQPKQRGMRFRYKCEGRSAGSIPGERSTDTTKTHPTIK-------INGYTGPGTVRISLVTKDPPH   63
_XP_040322176.1_     1  VEIIEQPKQRGMRFRYKCEGRSAGSIPGERSTDTTKTHPTIK-------INGYTGPGTVRISLVTKDPPH   63
_XP_006861137.1_     1  VEIIEQPKQRGMRFRYKCEGRSAGSIPGERSTDTTKTHPTIK-------INGYTGPGTVRISLVTKDPPH   63
_XP_007943155.2_     1  VEIIEQPKQRGMRFRYKCEGRSAGSIPGERSTDTTKTHPTIK-------INGYTGPGTVRISLVTKDPPH   63
_BAQ95593.1_         1  VEIIEQPKQRGMRFRYKCEGRSAGSIPGERSTDTTKTHPTIK-------INGYTGPGTVRISLVTKDPPH   63
_XP_019652454.2_     1  VEIIEQPKQRGMRFRYKCEGRSAGSIPGERSTDTTKTHPTIK-------INGYTGPGTVRISLVTKDPPH   63
_XP_016041658.1_     1  VEVIEQPKQRGMRFRYKCEGRSAGSIPGERSTDTTKTHPTIK-------INGYTGPGTVRISLVTKDPPH   63
_XP_047546997.1_     1  VEIIEQPKQRGMRFRYKCEGRSAGSIPGERSTDTTKTHPTIK-------INGYTGPGTVRISLVTKDPPH   63
_XP_042115231.1_     1  VEIIEQPKQRGMRFRYKCEGRSAGSIPGERSTDTTKTHPTIK-------INGYTGPGTVRISLVTKDPPH   63
_XP_042115227.1_     1  VEIIEQPKQRGMRFRYKCEGRSAGSIPGERSTDTTKTHPTIK-------INGYTGPGTVRISLVTKDPPH   63
```

```
_XP_015854576.1_     1  VEIIEQPKQRGMRFRYKCEGRSAGSIPGERSTDTTKTHPTIK-------INGYTGPGTVRISLVTKDPPH   63
_XP_025301452.1_     1  VEIIEQPKQRGMRFRYKCEGRSAGSIPGERSTDTTKTHPTIK-------INGYTGPGTVRISLVTKDPPH   63
_XP_044920951.1_     1  VEIIEQPKQRGMRFRYKCEGRSAGSIPGERSTDTTKTHPTIK-------INGYTGPGTVRISLVTKDPPH   63
_KAF3822254.1_       1  VEIIEQPKQRGMRFRYKCEGRSAGSIPGERSTDTTKTHPTIK-------INGYTGPGTVRISLVTKDPPH   63
_XP_021540718.1_     1  VEIIEQPKQRGMRFRYKCEGRSAGSIPGERSTDTTKTHPTIK-------INGYTGPGTVRISLVTKDPPH   63
_XP_022362935.1_     1  VEIIEQPKQRGMRFRYKCEGRSAGSIPGERSTDTTKTHPTIK-------INGYTGPGTVRISLVTKDPPH   63
_XP_044115623.1_     1  VEIIEQPKQRGMRFRYKCEGRSAGSIPGERSTDTTKTHPTIK-------INGYTGPGTVRISLVTKDPPH   63
_XP_032735359.1_     1  VEIIEQPKQRGMRFRYKCEGRSAGSIPGERSTDTTKTHPTIK-------INGYTGPGTVRISLVTKDPPH   63
_XP_032270304.1_     1  VEIIEQPKQRGMRFRYKCEGRSAGSIPGERSTDTTKTHPTIK-------INGYTGPGTVRISLVTKDPPH   63
_XP_032213216.1_     1  VEIIEQPKQRGMRFRYKCEGRSAGSIPGERSTDTTKTHPTIK-------INGYTGPGTVRISLVTKDPPH   63
_XP_004478661.1_     1  VEIIEQPKQRGMRFRYKCEGRSAGSIPGERSTDTTKTHPTIK-------INGYTGPGTVRISLVTKDPPH   63
_XP_035933519.1_     1  VEIIEQPKQRGMRFRYKCEGRSAGSIPGERSTDTTKTHPTIK-------INGYTGPGTVRISLVTKDPPH   63
_KAI5761432.1_       1  VEIIEQPKQRGMRFRYKCEGRSAGSIPGERSTDTTKTHPTIK-------INGYTGPGTVRISLVTKDPPH   63
_XP_029813244.1_     1  VEIIEQPKQRGMRFRYKCEGRSAGSIPGERSTDTTKTHPTIK-------INGYTGPGTVRISLVTKDPPH   63
_XP_027800377.1_     1  VEIIEQPKQRGMRFRYKCEGRSAGSIPGERSTETTKTHPTIK-------INGYTGPGTVRISLVTKDPPH   63
_XP_004852440.1_     1  VEIIEQPKQRGMRFRYKCEGRSAGSIPGERSTDTTKTHPTIK-------INGYTGPGTVRISLVTKDPPH   63
_XP_045873061.1_     1  VEIIEQPKQRGMRFRYKCEGRSAGSIPGERSTDTTKTHPTIK-------INGYTGPGTVRISLVTKDPPH   63
_XP_030891354.1_     1  VEIIEQPKQRGMRFRYKCEGRSAGSIPGERSTDTTKTHPTIK-------INGYTGPGTVRISLVTKDPPH   63
_CAD7670066.1_       1  VEIIEQPKQRGMRFRYKCEGRSAGSIPGERSTDTTKTHPTIK-------INGYTGPGTVRISLVTKDPPH   63
_XP_046301192.1_     1  VEIIEQPKQRGMRFRYKCEGRSAGSIPGERSTETTKTHPTIK-------INGYTGPGTVRISLVTKDPPH   63
_XP_036059776.1_     1  VEIIEQPKQRGMRFRYKCEGRSAGSIPGERSTDTTKTHPTIK-------INGYTGPGTVRISLVTKDPPH   63
_XP_028711748.1_     1  VEIIEQPKQRGMRFRYKCEGRSAGSIPGERSTDTTKTHPTIK-------INGYTGPGTVRISLVTKDPPH   63
_XP_037696258.1_     1  VEIIEQPKQRGMRFRYKCEGRSAGSIPGERSTDTTKTHPTIK-------INGYTGPGTVRISLVTKDPPH   63
_XP_004627287.1_     1  VEIIEQPKQRGMRFRYKCEGRSAGSIPGERSTDTTKTHPTIK-------INGYTGPGTVRISLVTKDPPH   63
_XP_037371589.1_     1  VEIIEQPKQRGMRFRYKCEGRSAGSIPGERSTDTTKTHPTIK-------INGYTGPGTVRISLVTKDPPH   63
_XP_003468122.1_     1  VEIIEQPKQRGMRFRYKCEGRSAGSIPGERSTDTTKTHPTIK-------INGYTGPGTVRISLVTKDPPH   63
_XP_040826731.1_     1  VEIIEQPKQRGMRFRYKCEGRSAGSIPGERSTDTTKTHPTIK-------INGYTGPGTVRISLVTKDPPH   63
_XP_005384525.1_     1  VEIIEQPKQRGMRFRYKCEGRSAGSIPGERSTDTTKTHPTIK-------INGYTGPGTVRISLVTKDPPH   63
_XP_045711286.1_     1  VEIIEQPKQRGMRFRYKCEGRSAGSIPGERSTDTTKTHPTIK-------INGYTGPGTVRISLVTKDPPH   63
_XP_044920955.1_     1  VEIIEQPKQRGMRFRYKCEGRSAGSIPGERSTDTTKTHPTIK-------INGYTGPGTVRISLVTKDPPH   63
_XP_047375326.1_     1  VEIIEQPKQRGMRFRYKCEGRSAGSIPGERSTDTTKTHPTIK-------INGYTGPGTVRISLVTKDPPH   63
_XP_036272436.1_     1  VEIIEQPKQRGMRFRYKCEGRSAGSIPGERSTDTTKTHPTIK-------INGYTGPGTVRISLVTKDPPH   63
_ELK13339.1_         1  VEIIEQPKQRGMRFRYKCEGRSAGSIPGERSTDTTKTHPTIK-------INGYTGPGTVRISLVTKDPPH   63
_XP_021564615.1_     1  VEIIEQPKQRGMRFRYKCEGRSAGSIPGERSTDTTKTHPTIK-------INGYTGPGTVRISLVTKDPPH   63
_XP_024902067.1_     1  VEIIEQPKQRGMRFRYKCEGRSAGSIPGERSTDTTKTHPTIK-------INGYTGPGTVRISLVTKDPPH   63
_XP_036349005.1_     1  VEIIEQPKQRGMRFRYKCEGRSAGSIPGERSTDTTKTHPTIK-------INGYTGPGTVRISLVTKDPPH   63
_XP_051683930.1_     1  VEIIEQPKQRGMRFRYKCEGRSAGSIPGERSTDTTKTHPTIK-------INGYTGPGTVRISLVTKDPPH   63
_XP_013006227.1_     1  VEIIEQPKQRGMRFRYKCEGRSAGSIPGERSTDTTKTHPTIK-------INGYTGPGTVRISLVTKDPPH   63
_XP_006893486.1_     1  VEIIEQPKQRGMRFRYKCEGRSAGSIPGERSTDTTKTHPTIK-------INGYTGPGTVRISLVTKDPPH   63
```

_XP_036272437.1_	1	VEIIEQPKQRGMRFRYKCEGRSAGSIPGERSTDTTKTHPTIK-------INGYTGPGTVRISLVTKDPPH	63
ELW65121.1	1	VEIIEQPKQRGMRFRYKCEGRSAGSIPGERSTDTTKTHPTIK-------INGYTGPGTVRISLVTKDPPH	63
EHB04873.1	1	VEIIEQPKQRGMRFRYKCEGRSAGSIPGERSTDTTKTHPTIK-------INGYTGPGTVRISLVTKDPPH	63
KAB0407447.1	1	VEIIEQPKQRGMRFRYKCEGRSAGSIPGERSTDTTKTHPTIK-------INGYTGPGTVRISLVTKDPPH	63
KAF6105901.1	1	VEIIEQPKQRGMRFRYKCEGRSAGSIPGERSTDTTKTHPTIK-------INGYTGPGTVRISLVTKDPPH	63
MXQ90627.1	1	VEIIEQPKQRGMRFRYKCEGRSAGSIPGERSTDTTKTHPTIK-------INGYTGPGTVRISLVTKDPPH	63
PNJ38432.1	1	VEIIEQPKQRGMRFRYKCEGRSAGSIPGERSTDTTKTHPTIK-------INGYTGPGTVRISLVTKDPPH	63
PNI93760.1	1	VEIIEQPKQRGMRFRYKCEGRSAGSIPGERSTDTTKTHPTIK-------INGYTGPGTVRISLVTKDPPH	63
KAF6438864.1	1	VEIIEQPKQRGMRFRYKCEGRSAGSIPGERSTDTTKTHPTIK-------VNGYTGPGTVRISLVTKDPPH	63
KAF6281766.1	1	VEIIEQPKQRGMRFRYKCEGRSAGSIPGERSTDTTKTHPTIK-------INGYTGPGTVRISLVTKDPPH	63
_XP_035119201.1_	1	VEIIEQPKQRGMRFRYKCEGRSAGSIPGERSTDTTKTHPTIK-------INGYTGPGTVRISLVTKDPPH	63
KAF6334125.1	1	VEIIEQPKQRGMRFRYKCEGRSAGSIPGERSTDTTKTHPTIK-------INGYTGPGTVRISLVTKDPPH	63
_XP_036785207.1_	1	VEIIEQPKQRGMRFRYKCEGRSAGSIPGERSTDTTKTHPTIK-------INGYTGPGTVRISLVTKDPPH	63
_XP_036851747.1_	1	VEIIEQPKQRGMRFRYKCEGRSAGSIPGERSTDTTKTHPTIK-------INGYTGPGTVRISLVTKDPPH	63
_XP_054367814.1_	1	VEIIEQPKQRGMRFRYKCEGRSAGSIPGERSTDTTKTHPTIK-------INGYTGPGTVRISLVTKDPPH	63
_XP_030689470.1_	1	VEIIEQPKQRGMRFRYKCEGRSAGSIPGERSTDTTKTHPTIK-------INGYTGPGTVRISLVTKDPPH	63
_XP_026946120.1_	1	VEIIEQPKQRGMRFRYKCEGRSAGSIPGERSTDTTKTHPTIK-------INGYTGPGTVRISLVTKDPPH	63
_XP_039696841.1_	1	VEIIEQPKQRGMRFRYKCEGRSAGSIPGERSTDTTKTHPTIK-------INGYTGPGTVRISLVTKDPPH	63
_1IKN_A_	1	VEIIEQPKQRGMRFRYKCEGRSAGSIPGERSTDTTKTHPTIK-------INGYTGPGTVRISLVTKDPPH	63
_XP_016775352.1_	1	VEIIEQPKQRGMRFRYKCEGRSAGSIPGERSTDTTKTHPTIK-------INGYTGPGTVRISLVTKDPPH	63
PNI93761.1	1	-----------MRFRYKCEGRSAGSIPGERSTDTTKTHPTIK-------INGYTGPGTVRISLVTKDPPH	52
_XP_054381607.1_	1	-----------MRFRYKCEGRSAGSIPGERSTDTTKTHPTIK-------INGYTGPGTVRISLVTKDPPH	52
_XP_011797882.1_	1	-----------MRFRYKCEGRSAGSIPGERSTDTTKTHPTIK-------INGYTGPGTVRISLVTKDPPH	52
_XP_011820578.1_	1	-----------MRFRYKCEGRSAGSIPGERSTDTTKTHPTIK-------INGYTGPGTVRISLVTKDPPH	52
_XP_024644798.1_	1	-----------MRFRYKCEGRSAGSIPGERSTDTTKTHPTIK-------INGYTGPGTVRISLVTKDPPH	52
_XP_012318394.1_	1	-----------MRFRYKCEGRSAGSIPGERSTDTTKTHPTIK-------INGYTGPGTVRISLVTKDPPH	52
_XP_035119199.1_	1	-----------MRFRYKCEGRSAGSIPGERSTDTTKTHPTIK-------INGYTGPGTVRISLVTKDPPH	52
_XP_036717345.1_	1	-----------MRFRYKCEGRSAGSIPGERSTDTTKTHPTIK-------INGYTGPGTVRISLVTKDPPH	52
_XP_051001869.1_	1	-----------MRFRYKCEGRSAGSIPGERSTDTTKTHPTIK-------INGYTGPGTVRISLVTKDPPH	52
_XP_007171011.2_	1	-----------MRFRYKCEGRSAGSIPGERSTDTTKTHPTIK-------INGYTGPGTVRISLVTKDPPH	52
_XP_033272588.1_	1	-----------MRFRYKCEGRSAGSIPGERSTDTTKTHPTIK-------INGYTGPGTVRISLVTKDPPH	52
_XP_026946119.1_	1	-----------MRFRYKCEGRSAGSIPGERSTDTTKTHPTIK-------INGYTGPGTVRISLVTKDPPH	52
_XP_048314002.1_	1	-----------MRFRYKCEGRSAGSIPGERSTDTTKTHPTIK-------INGYTGPGTVRISLVTKDPPH	52
_XP_014648508.1_	1	-----------MRFRYKCEGRSAGSIPGERSTDTTKTHPTIK-------INGYTGPGTVRISLVTKDPPH	52
_NP_001389477.1_	1	-----------MRFRYKCEGRSAGSIPGERSTDTTKTHPTIK-------INGYTGPGTVRISLVTKDPPH	52
_XP_049637117.1_	1	-----------MRFRYKCEGRSAGSIPGERSTDTTKTHPTIK-------INGYTGPGTVRISLVTKDPPY	52
_XP_054367813.1_	1	-----------MRFRYKCEGRSAGSIPGERSTDTTKTHPTIK-------INGYTGPGTVRISLVTKDPPH	52
_XP_041577696.1_	1	-----------MRFRYKCEGRSAGSIPGERSTDTTKTHPTIK-------INGYTGPGTVRISLVTKDPPH	52
_XP_005631531.1_	1	-----------MRFRYKCEGRSAGSIPGERSTDTTKTHPTIK-------INGYTGPGTVRISLVTKDPPH	52

```
_KAF6105898.1_      1  -----------MRFRYKCEGRSAGSIPGERSTDTTKTHPTIK-------INGYTGPGTVRISLVTKDPPH   52
_XP_012790426.1_    1  -----------MRFRYKCEGRSAGSIPGERSTDTTKTHPTIK-------INGYTGPGTVRISLVTKDPPH   52
_XP_031245195.1_       ----------------------------------------------------------------------
_NP_001391590.1_       ----------------------------------------------------------------------
_XP_053417695.1_       ----------------------------------------------------------------------
_XP_027240591.1_       ----------------------------------------------------------------------
_XP_027264387.1_       ----------------------------------------------------------------------
_XP_051001870.1_       ----------------------------------------------------------------------
_XP_023415353.1_       ----------------------------------------------------------------------
_XP_032747031.1_       ----------------------------------------------------------------------
_XP_052048334.1_       ----------------------------------------------------------------------
_XP_028019514.1_       ----------------------------------------------------------------------
_XP_049993277.1_       ----------------------------------------------------------------------
_XP_044617925.1_       ----------------------------------------------------------------------
_XP_048216513.1_       ----------------------------------------------------------------------
_XP_029414057.1_       ----------------------------------------------------------------------
_XP_049569046.1_       ----------------------------------------------------------------------
_XP_040605354.1_       ----------------------------------------------------------------------
_XP_048314004.1_       ----------------------------------------------------------------------
_XP_041518059.1_       ----------------------------------------------------------------------
_NP_001351996.1_       ----------------------------------------------------------------------
_XP_038935449.1_       ----------------------------------------------------------------------
_XP_038170707.1_       ----------------------------------------------------------------------
_XP_040140051.1_       ----------------------------------------------------------------------
_XP_052568262.1_       ----------------------------------------------------------------------
_XP_042115241.1_       ----------------------------------------------------------------------
_XP_054580929.1_       ----------------------------------------------------------------------
_XP_054367811.1_       ----------------------------------------------------------------------
_XP_027628238.1_       ----------------------------------------------------------------------
_XP_044536462.1_    1  VEIIEQPKQRGMRFRYKCEGRSAGSIPGERSTDTTKTHPTIK-------IHGYSGPGTVRISLVTKEPPH   63
_XP_043827224.1_    1  VEIIEQPKQRGMRFRYKCEGRSAGSIPGERSTDTTKTHPTIK-------INGYTGPGTVRISLVTKEPPH   63
_XP_027711838.1_    1  VEIIEQPKQRGMRFRYKCEGRSAGSIPGERSTDTTKTHPTIK-------IIGYTGPGTVRISLVTKEPPH   63
_XP_020824046.1_    1  VEIIEQPKQRGMRFRYKCEGRSAGSIPGERSTDTTKTHPTIK-------IVGYTGPGTVRISLVTKEPPH   63
_XP_036619952.1_    1  VEIIEQPKQRGMRFRYKCEGRSAGSIPGERSTDTTKTHPTIK-------IVGYTGPGTVRISLVTKEPPH   63
_XP_031799278.1_    1  VEIIEQPKQRGMRFRYKCEGRSAGSIPGERSTDTTKTHPTIK-------INGYTGPGTVRISLVTKEPPH   63
_XP_007502748.1_    1  VEIIEQPKQRGMRFRYKCEGRSAGSIPGERSTDTTKTHPTIK-------IHGYLGPGTVRISLVTKEPPH   63
_XP_051821546.1_    1  VEIIEQPKQRGMRFRYKCEGRSAGSIPGERSTDTTKTHPTIK-------INGYTGPGTVRISLVTKEPPH   63
_XP_051821547.1_    1  VEIIEQPKQRGMRFRYKCEGRSAGSIPGERSTDTTKTHPTIK-------INGYTGPGTVRISLVTKEPPH   63
_KAF6105894.1_      1  ------------------------------------------------------------------IPPR    4
```

```
_XP_008508783.1_        ------------------------------------------------------------
_MBV98069.1_            ------------------------------------------------------------
_XP_045411956.1_    1   -----------------------------------------QINGYTGPGTVRISLVTKDPPH   22
_XP_012520962.1_    1   -----------------------------------------QINGYTGPGTVRISLVTKDPPH   22
_XP_043307319.1_    1   -----------------------------------------QINGYTGPGTVRISLVTKDPPH   22
_XP_012666134.1_    1   -----------------------------------------QINGYTGPGTVRISLVTKDPPH   22
_XP_011367280.1_    1   -----------------------------------------QINGYTGPGTVRISLVTKDPPH   22
_KAF7238569.1_          ------------------------------------------------------------
_KAG8146725.1_      1   VEIIEQPKQRGMRFRYQCEGRLAGSIPGERSTDTTKTHPTIK-------IHNYQGPGKVRISLVTKEAPH   63
_XP_048371352.1_    1   VEIIEQPKQRGMRFRYQCEGRLAGSIPGERSTDTTKTHPTIK-------IHNYVGPGKVRISLVTKESPH   63
_XP_015272351.1_    1   VEIIEQPKQRGMRFRYQCEGRLAGSIPGERSTDTTKTHPTIK-------IHNYVGPGKVRISLVTKETPH   63
_XP_054845680.1_    1   VEIIEQPKQRGMRFRYQCEGRLAGSIPGERSTDTTKTHPTIK-------IHNYVGPGKVRISLVTKEAPH   63
_XP_015272352.1_    1   VEIIEQPKQRGMRFRYQCEGRLAGSIPGERSTDTTKTHPTIK-------IHNYVGPGKVRISLVTKETPH   63
_XP_042295897.1_    1   VEIIEQPKQRGMRFRYQCEGRLAGSIPGERSTDTTKTHPTIK-------VHNYVGPGKVRISLVTKEVPY   63
_XP_053133861.1_    1   VEIIEQPKQRGMRFRYQCEGRLAGSIPGERSTDTTKTHPTIK-------IHNYVGPGKVRISLVTKEVPH   63
_XP_053133862.1_    1   VEIIEQPKQRGMRFRYQCEGRLAGSIPGERSTDTTKTHPTIK-------IHNYVGPGKVRISLVTKEVPH   63
_KAJ6654174.1_      1   VEIIEQPKQRGMRFRYQCEGRLAGSIPGERSTDTTKTHPTIK-------IHNYMGPGKVRISLVTKETPH   63
_XP_044304454.1_    1   VEIIEQPKQRGMRFRYQCEGRLAGSIPGERSTDTTKTHPTIK-------VHNYMGPGKIRISLVTKESPH   63
_XP_020633015.1_    1   -EIIEQPKQRGMRFRYQCEGRLAGSIPGERSTDTTKTHPTIK-------IHNYVGPGKVRISLVTKESPY   62
_XP_028566864.1_    1   VEIIEQPKQRGMRFRYQCEGRLAGSIPGERSTDTTKTHPTIK-------IHNYTGPGKVRISLVTKEVPY   63
_XP_033030397.1_    1   VEIIEQPKQRGMRFRYQCEGRLAGSIPGERSTDTTKTHPTIK-------IHNYTGPGKVRISLVTKELPY   63
_XP_044304455.1_    1   VEIIEQPKQRGMRFRYQCEGRLAGSIPGERSTDTTKTHPTIK-------VHNYMGPGKIRISLVTKESPH   63
_XP_053225758.1_    1   VEIIEQPKQRGMRFRYQCEGRLAGSIPGERSTDTTKTHPTIK-------IHNYTGPGKVRISLVTKEVPY   63
_XP_033030398.1_    1   VEIIEQPKQRGMRFRYQCEGRLAGSIPGERSTDTTKTHPTIK-------IHNYTGPGKVRISLVTKELPY   63
_XP_034953722.1_    1   VEIIEQPKQRGMRFRYQCEGRLAGSIPGERSTDTTKTHPTIK-------IHNYTGPGKVRISLVTKEAPY   63
_XP_008123768.1_    1   VEIMEQPKQRGMRFRYQCEGRLAGSIPGERSTETTKTHPTIK-------IHNYVGPGKVRISLVTKETPY   63
_KAJ7307406.1_      1   -----------MRFRYQCEGRLAGSIPGERSTDTTKTHPTIK-------IHNYVGPGKVRISLVTKEAPY   52
_XP_013931335.1_    1   VEIIEQPKQRGMRFRYQCEGRLAGSIPGERSTDTTKTHPTIK-------IHNYQGPGKVRISLVTKEAPH   63
_XP_032089926.1_    1   VEIIEQPKQRGMRFRYQCEGRLAGSIPGERSTDTTKTHPTIK-------IHNYQGPGKVRISLVTKEAPH   63
_XP_026572203.1_    1   VEIIEQPKQRGMRFRYQCEGRLAGSIPGERSTDTTKTHPTIK-------IHNYQGPGKVRISLVTKEAPH   63
_XP_026572204.1_    1   VEIIEQPKQRGMRFRYQCEGRLAGSIPGERSTDTTKTHPTIK-------IHNYQGPGKVRISLVTKEAPH   63
_XP_034280315.1_    1   VEIIEQPKQRGMRFRYQCEGRLAGSIPGERSTDTTKTHPTIK-------IHNYQGPGKVRISLVTKEAPH   63
_XP_039222066.1_    1   VEIIEQPKQRGMRFRYQCEGRLAGSIPGERSTDTTKTHPTIK-------IHNYQGPGRVRISLVTKEAPH   63
_XP_015744179.1_    1   -----------MRFRYQCEGRLAGSIPGERSTDTTKTHPTIK-------IHNYQGPGKVRISLVTKEAPH   52
_XP_029142061.1_    1   LEIIEQPKQRGMRFRYQCEGRLAGSIPGERSTDTTKTHPTIK-------IHNYQGPGKVRISLVTKEAPH   63
_AAH41188.1_        1   VEIIEQPKQRGMRFRYKCEGRSAGSIPGERSTDTSKTHPTIK-------INNYQGPARIRISLVTKDSPH   63
_XP_018112002.1_    1   VEIIEQPKQRGMRFRYKCEGRSAGSIPGERSTDTSKTHPTIK-------INNYQGPARIRISLVTKDSPH   63
_NP_001081048.1_    1   VEIIEQPKQRGMRFRYKCEGRSAGSIPGERSTDTSKTHPTIK-------INNYQGPARIRISLVTKDSPH   63
_XP_031755832.1_    1   VEIIEQPKQRGMRFRYKCEGRSAGSIPGERSTDTTKTHPTIK-------INHYQGSARIRISLVTKDSPH   63
```

```
_KAG8437862.1_       1  VEIIEQPKQRGMRFRYKCEGRSAGSIPGERSTDTTKTHPTVK-------INNYQGPARIRISLVTKDAPF   63
_XP_053304358.1_     1  VEIIEQPKQRGMRFRYKCEGRSAGSIPGERSTDTTKTHPTIK-------VHNYQGPARIRISLVTKDAPH   63
_KAG9464550.1_       1  VEIIEQPKQRGMRFRYKCEGRSAGSIPGERSTDTTKTHPTIK-------VNGYQGPARIRISLVTKDAPY   63
_XP_040265411.1_     1  VEIIEQPKQRGMRFRYKCEGRSAGSIPGERSTDTTKTHPTIK-------VNNYQGPARIRISLVTKDAPY   63
_XP_044125715.1_     1  VEIIEQPKQRGMRFRYKCEGRSAGSIPGERSTDTTKTHPTIK-------VNNYQGPARIRISLVTKEAPY   63
_XP_040265414.1_     1  VEIIEQPKQRGMRFRYKCEGRSAGSIPGERSTDTTKTHPTIK-------VNNYQGPARIRISLVTKDAPY   63
_XP_044125717.1_     1  VEIIEQPKQRGMRFRYKCEGRSAGSIPGERSTDTTKTHPTIK-------VNNYQGPARIRISLVTKEAPY   63
_XP_040184801.1_     1  VEIIEQPKQRGMRFRYKCEGRSAGSIPGERSTDTTKTHPTIK-------VHNYQGPARIRISLVTKDVPH   63
_ALA55869.1_         1  VEIIEQPKQRGMRFRYKCEGRSAGSIPGERSTDTTKTHPTIK-------VHNYQGPARIRISLVTKDVPH   63
_XP_018408357.1_     1  VEIIEQPKQRGMRFRYKCEGRSAGSIPGERSTDTTKTHPTIK-------VNNYQGPARIRISLVTKDAPH   63
_XP_053575987.1_     1  VDIIEQPKQRGMRFRYKCEGRSAGSIPGERSTDTNKTHPTIK-------INKYQGPARIRISLVTKDSPH   63
_XP_033812535.1_     1  VEIIEQPKQRGMRFRYKCEGRSAGSIPGERSTDTNKTHPTIK-------VQNYVGPARIRISLVTKDTPH   63
_XP_030074553.1_     1  VEIIEQPXQRGMRFRYRCEGRSAGSIPGERSTDTNKTHPTVK-------VQNYVGPARIRISLVTKDIPH   63
_XP_029468059.1_     1  VEIIEQPKQRGMRFRYKCEGRSAGSIPGERSTDTNKTHPTIK-------VKNYVGPARVRISLVTKDLPH   63
_XP_029468060.1_     1  VEIIEQPKQRGMRFRYKCEGRSAGSIPGERSTDTNKTHPTIK-------VKNYVGPARVRISLVTKDLPH   63
_KAJ1106220.1_       1  VEIIEQPKQRGMRFRYKCEGRSAGSIPGERSTDTNKTHPTIK-------INNYSGSARVRISLVTKEQPY   63
_CAI5672275.1_       1  -----------MRFRYKCEGRSAGSIPGEKSNDTTKTHPAIK-------VNNYNGPLRVRISLVTKNAPY   52
_GCB80996.1_         1  -----------MRFRYKCEGRSAGSIPGEKSTDSTRTYPAIE-------IGNYVGQAKIRICLVTKKEPY   52
_XP_038618162.1_     1  VEIIKQPKQRGMRFHYKCEGRSVGSIPGERSTDTTKTHPTIK-------ISGYVGLGKVRISLVTKEPPH   63
Consensus_aa:           VEIIEQPKQRGMRFRYKCEGRSAGSIPGE+STDooKTHPTI+.......lssYpGPtpVRlSLVTK-sP@
Consensus_ss:            eeeee      eeeeee           ee       eeee       ee      eeeeeeeee


Conservation:            5799599755 7  7559779  57 77977777999979777  95  9  7 7977
_XP_028917328.1_    64  RPHPHELVGKD-CRDGFYEADLCPDRSIHSFQNLGIQCVKKRDLEQAITQRMQTNNNPFNVPL------E  126
_XP_038621028.1_    64  RPHPHELVGKD-CRDGFYEADLCPDRCIHSFQNLGIQCVKKRDLEQAITQRMQTNNNPFNVPL------E  126
_RLV71517.1_        53  RPHPHELVGKD-CKDGFYEAELCPERSIHSFQNLGIQCVKKRELEAAVAERIRTNNNPFNVPA------E  115
_XP_033367665.1_    53  RPHPHELVGKD-CKDGFYEAELCPERSIHSFQNLGIQCVKKRELEAAVAERIRTNNNPFNGTGSTGRDWE  121
_OWK50979.1_            ----------------------------------------------------------------------
_XP_031363427.1_    26  RPHPHELVGKD-CRDGFYEAELSPERSVHSFQNLGIQCVKKRELEAAVAERIRTNNXXXXVPA------E   88
_XP_041319512.1_     1  ------------------------------FQNLGIQCVKKRELEAAVAERIRTNNNPFNVPA------E   34
_XP_054509575.1_    53  RPHPHELVGKD-CRDGFYEAELCPERSIHSFQNLGIQCVKKRELEAAVAERIRTNNNPFNVPA------E  115
_XP_039909596.1_    53  RAHPHELVGKD-CKDGFYEAELCPERNIHSFQNLGIQCVKKRELDAAVAERIRTNNNPFNVPA------E  115
_TRZ10035.1_        53  RAHPHELVGKD-CKDGFYEAELCPERNIHSFQNLGIQCVKKRELEAAVAERIRTNNNPFNVPA------E  115
_XP_031950790.1_    53  RPHPHELVGKD-CKDGFYEAELSPERSIHSFQNLGIQCVKKRELEAAVAERIRTNNNPFNVPA------E  115
_XP_050163562.1_    53  RPHPHELVGKD-CRDGFYEAELPPERSVHSFQNLGIQCVKKRELEAAVAERIRTNNNPFNVPA------E  115
_XP_039428642.1_    53  RPHPHELVGKD-CKDGFYEAELXPERSIHSFQNLGIQCVKKRELEAAVAERIRTNNNPFNVPA------E  115
_XP_050840410.1_    68  RPHPHELVGKD-CKDGFYEAELCPERSIHSFQNLGIQCVKKRELEAAVAERIRTNNNPFNVPA------E  130
_XP_054151317.1_    64  RPHPHELVGKD-CRDGFYEAELSPERNIHSFQNLGIQCVKKRELEAAVAERIRTNNNPFNVPA------E  126
```

```
_XP_050840409.1_   64  RPHPHELVGKD-CKDGFYEAELCPERSIHSFQNLGIQCVKKRELEAAVAERIRTNNNPFNVPA------E  126
_XP_030825825.1_   64  RPHPHELVGKD-CRDGFYEAELCPERSIHSFQNLGIQCVKKRELEAAVAERIRTNNNPFNVPA------E  126
_XP_032939479.1_   64  RPHPHELVGKD-CKDGFYEAELCPERNIHSFQNLGIQCVKKRELEAAVAERIRTNNNPFNVPP------E  126
_XP_041567964.1_   64  RPHPHELVGKD-CRDGFYEAELCPERSVHSFQNLGIQCVKKRELEAAVAERIRTNNNPFNVPA------E  126
_XP_048149083.1_   64  RPHPHELVGKD-CKDGFYEAELSPERSIHSFQNLGIQCVKKRELEAAVAERIRTNNNPFNVPA------E  126
_XP_054374241.1_   64  RPHPHELVGKD-CRDGFYEAELCPERSIHSFQNLGIQCVKKRELEAAVAERIRTNNNPFNVPA------E  126
_NXV55901.1_       64  RPHPHELVGKD-CRDGFYEAELCPERSIHSFQNLGIQCVKKRELEAAVAERIRTNNNPFNVPA------E  126
_XP_005533697.1_   64  RPHPHELVGKD-CKDGFYEAELCPERSIHSFQNLGIQCVKKRELEAAVAERIRTNNNPFNVPA------E  126
_XP_014748521.1_   64  RPHPHELVGKD-CKDGFYEAELCPERSIHSFQNLGIQCVKKRELEAAVAERIRTNNNPFNVPP------E  126
_RMB90491.1_       64  RAHPHELVGKD-CKDGFYEAELCPERNIHSFQNLGIQCVKKRELDAAVAERIRTNNNPFNVPA------E  126
_XP_053857772.1_   64  RPHPHELVGKD-CKDGFYEAELCPERSIHSFQNLGIQCVKKRELEAAVAERIRTNNNPFNVPA------E  126
_XP_029862760.1_       ----------------------------------------------------------------------
_XP_039575719.1_   39  RPHPHELVGKD-CRDGFYEAELSPERSVHSFQNLGIQCVKKRELEAAVAERIRTNNNPFNVPA------E  101
_XP_054664313.1_   64  RPHPHELVGKD-CRDGYYEAELPPERNVHSFQNLGIQCVKKRELEAAVAERIRTNNNPFNVPA------E  126
_XP_049691648.1_   64  RPHPHELVGKD-CRDGYYEAELPPERNVHSFQNLGIQCVKKRELEAAVAERIRTNNNPFNVPA------E  126
_XP_030331239.1_   64  RPHPHELVGKD-CHDGYYEAELPPERSVHSFQNLGIQCVKKRELEAAVAERIRTNNNPFNVPA------E  126
_XP_010726496.2_       ----------------------------------------------------------------------
_XP_042747731.1_       ----------------------------------------------------------------------
_XP_031466227.1_   64  RPHPHELVGRH-CQHGYYEAELSPERCVHSFQNLGIQCVKKRELEAAVAERIRTNNNPFNVPM------E  126
_XP_032297702.1_   64  RPHPHELVGRH-CQHGYYEAELSPERSVHSFQNLGIQCVKKRELEAAVAERIRTNNNPFNVPM------E  126
_RMB90505.1_       33  RAHPHELVGKDCCKDGFYEAELCPERNIHSAGGAA-----------------------------------   67
_KAI2561003.1_     53  RPHPHELVGKD-CRDGFYEAELCPDRCIHSFQNLGIQCVKKRDLEQAISQRIQTNNNPFQVPI------E  115
_KAF6105893.1_     64  RPHPHELVGKD-CRDGYYEAELCPDRCIHSFQNLGIQCVKKRDLEQAVRQRIQTNNNPFQVPI------E  126
_PNJ38430.1_       64  RPHPHELVGKD-CRDGFYEAELCPDRCIHSFQNLGIQCVKKRDLEQAINQRIQTNNNPFQVPI------E  126
_PNI93749.1_       64  RPHPHELVGKD-CRDGFYEAELCPDRCIHSFQNLGIQCVKKRDLEQAISQRIQTNNNPFQVPI------E  126
_KAF6334124.1_     64  RPHPHELVGKD-CRDGFYEAELCPDRCIHSFQNLGIQCVKKRDLEQAISQRIQTNNNPFQVPI------E  126
_KAF6467432.1_     64  RPHPHELVGKD-CRDGFYEAELCPDRCIHSFQNLGIQCVKKRDLEQAISQRIQTNNNPFQVPI------E  126
_PNI93759.1_       64  RPHPHELVGKD-CRDGFYEAELCPDRCIHSFQNLGIQCVKKRDLEQAISQRIQTNNNPFQVPI------E  126
_PNJ38434.1_       64  RPHPHELVGKD-CRDGFYEAELCPDRCIHSFQNLGIQCVKKRDLEQAINQRIQTNNNPFQVPI------E  126
_KAH0508019.1_     71  RPHPHELVGKD-CRDGFYEAELCPDRCIHSFQNLGIQCVKKRDLEQAISQRIQTNNNPFQVPM------E  133
_AAA36408.1_       64  RPHPHELVGKD-CRDGFYEAELCPDRCIHSFQNLGIQCVKKRDLEQAISQRIQTNNNPFQVPI------E  126
_XP_003274247.2_   64  RPHPHELVGKD-CRDGFYEAELCPDRCIHSFQNLGIQCVKKRDLEQAINQRIQTNNNPFQVPI------E  126
_XP_007981896.1_   64  RPHPHELVGKD-CRDGFYEAELCPDRCIHSFQNLGIQCVKKRDLEQAITQRIQTNNNPFQVPI------E  126
_XP_054296639.1_   64  RPHPHELVGKD-CRDGFYEAELCPDRCIHSFQNLGIQCVKKRDLEQAINQRIQTNNNPFQVPI------E  126
_XP_023042293.1_   64  RPHPHELVGKD-CRDGFYEAELCPDRCIHSFQNLGIQCVKKRDLEQAITQRIQTNNNPFQVPI------E  126
_XP_025211457.1_   64  RPHPHELVGKD-CRDGFYEAELCPDRCIHSFQNLGIQCVKKRDLEQAITQRIQTNNNPFQVPI------E  126
_XP_039326898.1_   64  RPHPHELVGKD-CRDGFYEAELCPDRCIHSFQNLGIQCVKKRDLEQAINQRIQTNNNPFQVPI------E  126
_XP_021781237.2_   64  RPHPHELVGKD-CRDGFYEAELCPDRCIHSFQNLGIQCVKKRDLEQAITQRIQTNNNPFQVPI------E  126
_XP_010364772.1_   64  RPHPHELVGKD-CRDGFYEAELCPDRCIHSFQNLGIQCVKKRDLEQAITQRIQTNNNPFQVPI------E  126
```

```
_XP_033063134.1_    64  RPHPHELVGKD-CRDGFYEAELCPDRCIHSFQNLGIQCVKKRDLEQAITQRIQTNNNPFQVPI------E  126
_XP_017392927.1_    64  RPHPHELVGKD-CRDGFYEAELCPDRCIHSFQNLGIQCVKKRDLEQAINQRIQTNNNPFQVPI------E  126
_XP_011897055.1_    64  RPHPHELVGKD-CRDGFYEAELCPDRCIHSFQNLGIQCVKKRDLEQAITQRIQTNNNPFQVPI------E  126
_XP_032138725.1_    64  RPHPHELVGKD-CRDGFYEAELCPDRCIHSFQNLGIQCVKKRDLEQAINQRIQTNNNPFQVPI------E  126
_CAA80524.2_        64  RPHPHELVGKD-CRDGFYEAELCPDRCIHSFQNLGIQCVKKRDLEQAISQRIQTNNNPFQVPI------E  126
_XP_005577305.2_    64  RPHPHELVGKD-CRDGFYEAELCPDRCIHSFQNLGIQCVKKRDLEQAITQRIQTNNNPFQVPI------E  126
_BAE87977.1_        64  RPHPHELVGKD-CRDGFYEAELCPDRCIHSFQNLGIQCVKKRDLEQAITQRIQTNNNPFQVPI------E  126
_XP_023042294.1_    64  RPHPHELVGKD-CRDGFYEAELCPDRCIHSFQNLGIQCVKKRDLEQAITQRIQTNNNPFQVPI------E  126
_XP_050612617.1_    64  RPHPHELVGKD-CRDGFYEAELCPDRCIHSFQNLGIQCVKKRDLEQAITQRIQTNNNPFQVPI------E  126
_EHH56082.1_        64  RPHPHELVGKD-CRDGFYEAELCPDRCIHSFQNLGIQCVKKRDLEQAITQRIQTNNNPFQVPI------E  126
_XP_028689064.1_    64  RPHPHELVGKD-CRDGFYEAELCPDRCIHSFQNLGIQCVKKRDLEQAITQRIQTNNNPFQVPI------E  126
_XP_045411957.1_    64  RPHPHELVGKD-CRDGFYEAELCPDRCIHSFQNLGIQCVKKRDLEQAISQRIQTNNNPFQVPI------E  126
_XP_012520963.1_    64  RPHPHELVGKD-CRDGFYEAELCPDRCIHSFQNLGIQCVKKRDLEQAISQRIQTNNNPFQVPI------E  126
_XP_012633553.1_    64  RPHPHELVGKD-CRDGFYEAELCPDRCIHSFQNLGIQCVKKRDLEQAISQRIQTNNNPFQVPI------E  126
_XP_053417694.1_    64  RPHPHELVGKD-CRDGFYEADLCPDRCIHSFQNLGIQCVKKRDLEQAISQRIQTNNNPFQVPI------E  126
_XP_032976853.1_    64  RPHPHELVGKD-CRDGFYEAELCPDRCIHSFQNLGIQCVKKRDLEQAISQRIQTNNNPFQVPI------E  126
_XP_008576943.1_    64  RPHPHELVGKD-CRDGFYEAELCPDRCIHSFQNLGIQCVKKRDLEQAISQRIQTNNNPFQVPI------E  126
_XP_032345730.1_    64  RPHPHELVGKD-CRDGFYEAELCPDRCIHSFQNLGIQCVKKRDLEQAISQRIQTNNNPFQVPI------E  126
_XP_010955442.1_    64  RPHPHELVGKD-CRDGFYEAELCPDRCIHSFQNLGIQCVKKRDLEQAISQRIQTNNNPFQVPI------E  126
_XP_031303872.1_    64  RPHPHELVGKD-CRDGFYEAELCPDRCIHSFQNLGIQCVKKRDLEQAISQRIQTNNNPFQVPI------E  126
_XP_032976854.1_    64  RPHPHELVGKD-CRDGFYEAELCPDRCIHSFQNLGIQCVKKRDLEQAISQRIQTNNNPFQVPI------E  126
_XP_053780986.1_    64  RPHPHELVGKD-CRDGYYEAELCPDRCIHSFQNLGIQCVKKRDLEQAISQRIQTNNNPFQVPI------E  126
_XP_036115166.1_    64  RPHPHELVGKD-CRDGFYEAELCPDRCIHSFQNLGIQCVKKRDLEQAISQRIQTNNNPFQVPI------E  126
_XP_043307320.1_    64  RPHPHELVGKD-CRDGFYEAELCPDRCIHSFQNLGIQCVKKRDLEQAISQRIQTNNNPFQVPI------E  126
_EQB77721.1_        64  RPHPHELVGKD-CRDGFYEAELCPDRCIHSFQNLGIQCVKKRDLEQAISQRIQTNNNPFQVPI------E  126
_NP_001107753.1_    64  RPHPHELVGKD-CRDGFYEAELCPDRCIHSFQNLGIQCVKKRDLEQAINQRIQTNNNPFQVPI------E  126
_XP_036785182.1_    64  RPHPHELVGKD-CRDGFYEAELCPDRCIHSFQNLGIQCVKKRDLEQAISQRIQTNNNPFQVPI------E  126
_XP_020762118.1_    64  RPHPHELVGKD-CRDGFYEAELCPDRCIHSFQNLGIQCVKKRDLEQAISQRIQTNNNPFQVPI------E  126
_XP_031546756.1_    64  RPHPHELVGKD-CRDGFYEAELCPDRCIHSFQNLGIQCVKKRDLEQAISQRIQTNNNPFQVPI------E  126
_XP_023989099.1_    64  RPHPHELVGKD-CRDGFYEAELCPDRCIHSFQNLGIQCVKKRDLEQAISQRIQTNNNPFQVPV------E  126
_KAF4009900.1_      64  RPHPHELVGKD-CRDGFYEAELCPDRCIHSFQNLGIQCVKKRDLEQAISQRIQTNNNPFQVPI------E  126
_KAB0376704.1_      64  RPHPHELVGKD-CRDGFYEAELCPDRCIHSFQNLGIQCVKKRDLEQAISQRIQTNNNPFQVPI------E  126
_KAB0364713.1_      64  RPHPHELVGKD-CRDGFYEAELCPDRCIHSFQNLGIQCVKKRDLEQAISQRIQTNNNPFQVPI------E  126
_CBT01657.1_        64  RPHPHELVGKD-CRDGFYEAELCPDRCIHSFQNLGIQCVKKRDLEQAINQRIQTNNNPFQVPI------E  126
_XP_036851744.1_    64  RPHPHELVGKD-CRDGFYEAELCPDRCIHSFQNLGIQCVKKRDLEQAISQRIQTNNNPFQVPI------E  126
_XP_007128247.1_    64  RPHPHELVGKD-CRDGFYEAELCPDRCIHSFQNLGIQCVKKRDLEQAISQRIQTNNNPFQVPV------E  126
_XP_003798713.1_    64  RPHPHELVGKD-CRDGFYEADLCPDRCIHSFQNLGIQCVKKRDLEQAISQRIQTNNNPFQVPI------E  126
_NP_001073711.1_    64  RPHPHELVGKD-CRDGFYEAELCPDRCIHSFQNLGIQCVKKRDLEQAISQRIQTNNNPFQVPI------E  126
_XP_006056522.1_    64  RPHPHELVGKD-CRDGFYEAELCPDRCIHSFQNLGIQCVKKRDLEQAISQRIQTNNNPFQVPI------E  126
```

```
_XP_019593326.1_   64  RPHPHELVGKD-CRDGFYEAELCPDRCIHSFQNLGIQCVKKRDLEQAISQRIQTNNNPFQVPI------E  126
_XP_005894159.1_   64  RPHPHELVGKD-CRDGFYEAELCPDRCIHSFQNLGIQCVKKRDLEQAISQRIQTNNNPFQVPI------E  126
_ELR57541.1_       64  RPHPHELVGKD-CRDGFYEAELCPDRCIHSFQNLGIQCVKKRDLEQAISQRIQTNNNPFQVPI------E  126
_XP_010836092.1_   64  RPHPHELVGKD-CRDGFYEAELCPDRCIHSFQNLGIQCVKKRDLEQAISQRIQTNNNPFQVPI------E  126
_XP_051049621.1_   64  RPHPHELVGKD-CRDGFYEAELCPDRCIHSFQNLGIQCVKKRDLEQAISQRIQTNNNPFQVPI------E  126
_CBT01658.1_       64  RPHPHELVGKD-CRDGFYEAELCPDRCIHSFQNLGIQCVKKRDLEQAISQRIQTNNNPFQVPI------E  126
_XP_015974930.1_   64  RPHPHELVGKD-CRDGFYEAELCPDRCIHSFQNLGIQCVKKRDLEQAISQRIQTNNNPFQVPI------E  126
_XP_027264386.1_   64  RPHPHELVGKD-CRDGFYEAELCPDRCIHSFQNLGIQCVKKRDLEQAISQRIQTNNNPFQVPI------E  126
_XP_040112158.1_   64  RPHPHELVGKD-CRDGFYEAELCPDRCIHSFQNLGIQCVKKRDLEQAISQRIQTNNNPFQVPI------E  126
_XP_052517633.1_   64  RPHPHELVGKD-CRDGFYEAELCPDRCIHSFQNLGIQCVKKRDLEQAISQRIQTNNNPFQVPI------E  126
_XP_017898873.1_   64  RPHPHELVGKD-CRDGFYEAELCPDRCIHSFQNLGIQCVKKRDLEQAISQRIQTNNNPFQVPI------E  126
_XP_020020484.1_   64  RPHPHELVGKD-CRDGFYEAELCPDRCIHSFQNLGIQCVKKRDLEQAISQRIQTNNNPFQVPI------E  126
_XP_047631223.1_   64  RPHPHELVGKD-CRDGFYEAELCPDRCIHSFQNLGIQCVKKRDLEQAINQRIQTNNNPFQVPI------E  126
_XP_007651509.2_   64  RPHPHELVGKD-CRDGFYEAELCPDRCIHSFQNLGIQCVKKRDLEQAISQRIQTNNNPFQVPI------E  126
_XP_027815096.1_   64  RPHPHELVGKD-CRDGFYEAELCPDRCIHSFQNLGIQCVKKRDLEQAISQRIQTNNNPFQVPI------E  126
_XP_034377707.1_   64  RPHPHELVGKD-CRDGFYEAELCPDRCIHSFQNLGIQCVKKRDLEQAISQRIQTNNNPFQVPI------E  126
_ERE77884.1_       64  RPHPHELVGKD-CRDGFYEAELCPDRCIHSFQNLGIQCVKKRDLEQAISQRIQTNNNPFQVPI------E  126
_XP_028626823.1_   64  RPHPHELVGKD-CRDGFYEAELCPDRCIHSFQNLGIQCVKKRDLEQAISQRIQTNNNPFQVPI------E  126
_KAF5928161.1_     64  RPHPHELVGKD-CRDGFYEAELCPDRCIHSFQNLGIQCVKKRDLEQAISQRIQTNNNPFQVPI------E  126
_KAI5933031.1_     64  RPHPHELVGKD-CRDGFYEAELCPDRCIHSFQNLGIQCVKKRDLEQAISQRIQTNNNPFQVPI------E  126
_XP_021007720.1_   64  RPHPHELVGKD-CRDGYYEADLCPDRCIHSFQNLGIQCVKKRDLEQAISQRIQTNNNPFHVPI------E  126
_XP_026252329.1_   64  RPHPHELVGKD-CRDGFYEAELCPDRCIHSFQNLGIQCVKKRDLEQAISQRIQTNNNPFQVPI------E  126
_XP_054426842.1_   64  RPHPHELVGKD-CRDGYYEAELCPDRCIHSFQNLGIQCVKKRDLEQAISQRIQTNNNPFQVPI------E  126
_XP_032747030.1_   64  RPHPHELVGKD-CRDGFYEAELCPDRCIHSFQNLGIQCVKKRDLEQAISQRIQTNNNPFQVPI------E  126
_XP_049748463.1_   64  RPHPHELVGKD-CRDGFYEAELCPDRCIHSFQNLGIQCVKKRDLEQAISQRIQTNNNPFQVPI------E  126
_KAJ1065952.1_     64  RPHPHELVGKD-CRDGFYEAELCPDRCIHSFQNLGIQCVKKRDLEQAISQRIQTNNNPFQVPI------E  126
_XP_052048317.1_   64  RPHPHELVGKD-CRDGFYEAELCPDRCIHSFQNLGIQCVKKRDLEQAISQRIQTNNNPFQVPI------E  126
_XP_012876614.1_   64  RPHPHELVGKD-CHDGFYEAELCPDRSIHSFQNLGIQCVKKRDLEQAISQRIQTNNNPFQVPI------E  126
_XP_020020482.1_   64  RPHPHELVGKD-CRDGFYEAELCPDRCIHSFQNLGIQCVKKRDLEQAISQRIQTNNNPFQVPI------E  126
_XP_010597340.1_   64  RPHPHELVGKD-CRDGFYEAELCPDRCIHSFQNLGIQCVKKRDLEQAISQRIQTNNNPFQVPI------E  126
_XP_026339000.2_   64  RPHPHELVGKD-CRDGFYEAELCPDRCIHSFQNLGIQCVKKRDLEQAISQRIQTNNNPFQVPI------E  126
_XP_030689468.1_   64  RPHPHELVGKD-CRDGFYEAELCPDRCIHSFQNLGIQCVKKRDLEQAISQRIQTNNNPFQVPV------E  126
_XP_049993276.1_   64  RPHPHELVGKD-CRDGFYEAELCPDRCIHSFQNLGIQCVKKRDLEQAISQRIQTNNNPFQVPM------E  126
_XP_014700317.1_   64  RPHPHELVGKD-CRDGFYEAELCPDRCIHSFQNLGIQCVKKRDLEQAISQRIQTNNNPFQVPI------E  126
_XP_021494126.1_   64  RPHPHELVGKD-CRDGFYEAELCPDRCIHSFQNLGIQCVKKRDLEQAISQRIQTNNNPFQVPI------E  126
_XP_022447167.1_   64  RPHPHELVGKD-CRDGFYEAELCPDRCIHSFQNLGIQCVKKRDLEQAISQRIQTNNNPFQVPV------E  126
_XP_042532137.1_   64  RPHPHELVGKD-CHDGFYEAELCPDRSIHSFQNLGIQCVKKRDLEQAISQRIQTNNNPFQVPI------E  126
_XP_008820421.1_   64  RPHPHELVGKD-CRDGFYEAELCPDRCIHSFQNLGIQCVKKRDLEQAISQRIQTNNNPFQVPI------E  126
_XP_023415352.1_   64  RPHPHELVGKD-CRDGFYEAELCPDRCIHSFQNLGIQCVKKRDLEQAISQRIQTNNNPFQVPI------E  126
```

_XP_029414056.1_	64	RPHPHELVGKD-CRDGFYEAELCPDRCIHSFQNLGIQCVKKRDLEQAISQRIQTNNNPFQVPI------E	126
_XP_024604853.1_	64	RPHPHELVGKD-CRDGFYEAELCPDRCIHSFQNLGIQCVKKRDLEQAISQRIQTNNNPFQVPV------E	126
_XP_007462052.1_	64	RPHPHELVGKD-CRDGFYEAELCPDRCIHSFQNLGIQCVKKRDLEQAISQRIQTNNNPFQVPV------E	126
_XP_006101621.1_	64	RPHPHELVGKD-CRDGFYEAELCPDRCIHSFQNLGIQCVKKRDLEQAINQRIQTNNNPFQVPM------E	126
_XP_005064049.1_	64	RPHPHELVGKD-CRDGFYEAELCPDRCIHSFQNLGIQCVKKRDLEQAISQRIQTNNNPFQVPI------E	126
_XP_031245193.1_	64	RPHPHELVGKD-CRDGYYEAELCPDRCIHSFQNLGIQCVKKRDLEQAISQRIQTNNNPFHVPI------E	126
ELK24332.1	64	RPHPHELVGKD-CRDGFYEAELCPDRCIHSFQNLGIQCVKKRDLEQAISQRIQTNNNPFQVPM------E	126
_XP_041518057.1_	64	RPHPHELVGKD-CRDGFYEAELCPDRCIHSFQNLGIQCVKKRDLEQAISQRIQTNNNPFQVPM------E	126
_XP_005351804.2_	64	RPHPHELVGKD-CRDGFYEAELCPDRCIHSFQNLGIQCVKKRDLEQAISQRIQTNNNPFQVPM------E	126
EGW13067.1	64	RPHPHELVGKD-CRDGFYEAELCPDRCIHSFQNLGIQCVKKRDLEQAISQRIQTNNNPFQVPI------E	126
ERE77883.1	64	RPHPHELVGKD-CRDGFYEAELCPDRCIHSFQNLGIQCVKKRDLEQAISQRIQTNNNPFQVPI------E	126
KFO28401.1	64	RPHPHELVGKD-CRDGFYEAELCPDRCIHSFQNLGIQCVKKRDLEQAISQRIQTNNNPFQVPI------E	126
_XP_010634478.1_	64	RPHPHELVGKD-CRDGFYEAELCPDRCIHSFQNLGIQCVKKRDLEQAISQRIQTNNNPFQVPI------E	126
OBS81518.1	64	RPHPHELVGKD-CRDGFYEAELCPDRCIHSFQNLGIQCVKKRDLEQAISQRIQTNNNPFQVPI------E	126
_XP_007462051.1_	64	RPHPHELVGKD-CRDGFYEAELCPDRCIHSFQNLGIQCVKKRDLEQAISQRIQTNNNPFQVPV------E	126
_XP_014393535.1_	64	RPHPHELVGKD-CRDGFYEAELCPDRCIHSFQNLGIQCVKKRDLEQAINQRIQTNNNPFQVPM------E	126
_XP_048216512.1_	64	RPHPHELVGKD-CHDGFYEAELCPDRCIHSFQNLGIQCVKKRDLEQAISQRIQTNNNPFQVPI------E	126
_XP_015424455.1_	64	RPHPHELVGKD-CRDGFYEAELCPDRCIHSFQNLGIQCVKKRDLEQAISQRIQTNNNPFQVPM------E	126
_XP_019501147.1_	64	RPHPHELVGKD-CRDGFYEAELCPDRCIHSFQNLGIQCVKKRDLEQAISQRIQTNNNPFQVPI------E	126
_XP_004656738.2_	64	RPHPHELVGKD-CRDGFYEADLCPDRCIHSFQNLGIQCVKKRDLEQAISQRIQTNNNPFQVPI------E	126
_XP_036909111.1_	64	RPHPHELVGKD-CRDGYYEAELCPDRCIHSFQNLGIQCVKKRDLEQAVRQRIQTNNNPFQVPI------E	126
_XP_036182518.1_	64	RPHPHELVGKD-CRDGFYEAELCPDRCIHSFQNLGIQCVKKRDLEQAISQRIQTNNNPFQVPM------E	126
_XP_012580591.1_	64	RPHPHELVGKD-CRDGFYEAELCPDRCIHSFQNLGIQCVKKRDLEQAINQRIQTNNNPFQVPI------E	126
_XP_016063765.1_	64	RPHPHELVGKD-CRDGFYEAELCPDRCIHSFQNLGIQCVKKRDLEQAISQRIQTNNNPFQVPI------E	126
_XP_021047054.1_	64	RPHPHELVGKD-CRDGYYEADLCPDRCIHSFQNLGIQCVKKRDLEQAISQRIQTNNNPFHVPI------E	126
_XP_023095836.1_	64	RPHPHELVGKD-CRDGFYEAELCPDRCIHSFQNLGIQCVKKRDLEQAISQRIQTNNNPFQVPI------E	126
_XP_045342734.1_	64	RPHPHELVGKD-CRDGFYEAELCPDRCIHSFQNLGIQCVKKRDLEQAISQRIQTNNNPFQVPI------E	126
_XP_053755429.1_	64	RPHPHELVGKD-CRDGFYEAELCPDRCIHSFQNLGIQCVKKRDLEQAISQRIQTNNNPFQVPI------E	126
_XP_022447166.1_	64	RPHPHELVGKD-CRDGFYEAELCPDRCIHSFQNLGIQCVKKRDLEQAISQRIQTNNNPFQVPV------E	126
_XP_004713741.1_	64	RPHPHELVGKD-CRDGFYEAELCPDRCIHSFQNLGIQCVKKRDLEQAISQRIQTSNNSFQVPI------E	126
_NP_954888.1_	64	RPHPHELVGKD-CRDGFYEAELCPDRCIHSFQNLGIQCVKKRDLEQAISQRIQTNNNPFQVPI------E	126
_XP_037002389.2_	64	RPHPHELVGKD-CRDGYYEAELCPDRCIHSFQNLGIQCVKKRDLEQAVRQRIQTNNNPFQVPI------E	126
_XP_039100808.1_	64	RPHPHELVGKD-CRDGFYEAELCPDRCIHSFQNLGIQCVKKRDLEQAISQRIQTNNNPFQVPI------E	126
_XP_038170688.1_	64	RPHPHELVGKD-CRDGFYEAELCPDRCIHSFQNLGIQCVKKRDLEQAISQRIQTNNNPFQVPI------E	126
_XP_040495345.1_	64	RPHPHELVGKD-CRDGFYEAELCPDRCIHSFQNLGIQCVKKRDLEQAISQRIQTNNNPFQVPI------E	126
_XP_052568261.1_	64	RPHPHELVGKD-CRDGFYEAELCPDRCIHSFQNLGIQCVKKRDLEQAISQRIQTNNNPFQVPI------E	126
_XP_042760134.1_	64	RPHPHELVGKD-CRDGFYEAELCPDRCIHSFQNLGIQCVKKRDLEQAISQRIQTNNNPFQVPI------E	126
_XP_049472392.1_	64	RPHPHELVGKD-CRDGFYEAELCPDRCIHSFQNLGIQCVKKRDLEQAISQRIQTNNNPFQVPI------E	126
_XP_023593974.1_	64	RPHPHELVGKD-CRDGFYEAELCPDRCIHSFQNLGIQCVKKRDLEQAISQRIQTNNNPFQVPI------E	126

_XP_034501886.1_	64	RPHPHELVGKD-CRDGFYEAELCPDRCIHSFQNLGIQCVKKRDLEQAISQRIQTNNNPFQVPI------E	126
_XP_004393991.1_	64	RPHPHELVGKD-CRDGFYEAELCPDRCIHSFQNLGIQCVKKRDLEQAISQRIQTNNNPFQVPI------E	126
_XP_025860214.1_	64	RPHPHELVGKD-CRDGFYEAELCPDRCIHSFQNLGIQCVKKRDLEQAISQRIQTNNNPFQVPI------E	126
_XP_019501146.1_	64	RPHPHELVGKD-CRDGFYEAELCPDRCIHSFQNLGIQCVKKRDLEQAISQRIQTNNNPFQVPI------E	126
_XP_025734327.1_	64	RPHPHELVGKD-CRDGFYEAELCPDRCIHSFQNLGIQCVKKRDLEQAISQRIQTNNNPFQVPI------E	126
_XP_025301454.1_	64	RPHPHELVGKD-CRDGFYEAELCPDRCIHSFQNLGIQCVKKRDLEQAISQRIQTNNNPFQVPI------E	126
KAG3284687.1	64	RPHPHELVGKD-CRDGFYEAELCPDRCIHSFQNLGIQCVKKRDLEQAISQRIQTNNNPFQVPI------E	126
VFV23136.1	64	RPHPHELVGKD-CRDGFYEAELCPDRCIHSFQNLGIQCVKKRDLEQAISQRIQTNNNPFQVPI------E	126
_XP_025771173.1_	64	RPHPHELVGKD-CRDGFYEAELCPDRCIHSFQNLGIQCVKKRDLEQAISQRIQTNNNPFQVPI------E	126
_XP_054580928.1_	64	RPHPHELVGKD-CRDGFYEAELCPDRCIHSFQNLGIQCVKKRDLEQAISQRIQTNNNPFQVPI------E	126
EPQ06533.1	64	RPHPHELVGKD-CRDGFYEAELCPDRCIHSFQNLGIQCVKKRDLEQAINQRIQTNNNPFQVPM------E	126
_XP_034869141.1_	64	RPHPHELVGKD-CRDGFYEAELCPDRCIHSFQNLGIQCVKKRDLEQAISQRIQTNNNPFQVPV------E	126
_XP_042813589.1_	64	RPHPHELVGKD-CRDGFYEAELCPDRCIHSFQNLGIQCVKKRDLEQAISQRIQTNNNPFQVPI------E	126
_XP_040322176.1_	64	RPHPHELVGKD-CRDGFYEAELCPDRCIHSFQNLGIQCVKKRDLEQAISQRIQTNNNPFQVPI------E	126
_XP_006861137.1_	64	RPHPHELVGKD-CRDGFYEAELCPDRCIHSFQNLGIQCVKKRDLEQAISQRIQTNNNPFQVPI------E	126
_XP_007943155.2_	64	RPHPHELVGKD-CRDGFYEAELSPDRCIHSFQNLGIQCVKKRDLEQAISQRIQTNNNPFQVPI------E	126
BAQ95593.1	64	RPHPHELVGKD-CRDGFYEAELCPDRCIHSFQNLGIQCVKKRDLEQAISQRIQTNNNPFQVPI------E	126
_XP_019652454.2_	64	RPHPHELVGKD-CRDGFYEAELCPDRCIHSFQNLGIQCVKKRDLEQAISQRIQTNNNPFQVPI------E	126
_XP_016041658.1_	64	RPHPHELVGKD-CRDGFYEAELCPDRCIHSFQNLGIQCVKKRDLEQAISQRIQTNNNPFQVPV------E	126
_XP_047546997.1_	64	RPHPHELVGKD-CRDGFYEAELCPDRCIHSFQNLGIQCVKKRDLEQAISQRIQTNNNPFQVPI------E	126
_XP_042115231.1_	64	RPHPHELVGKD-CRDGFYEAELCPDRCIHSFQNLGIQCVKKRDLEQAISQRIQTNNNPFQVPI------E	126
_XP_042115227.1_	64	RPHPHELVGKD-CRDGFYEAELCPDRCIHSFQNLGIQCVKKRDLEQAISQRIQTNNNPFQVPI------E	126
_XP_015854576.1_	64	RPHPHELVGKD-CRDGFYEAELCPDRCIHSFQNLGIQCVKKRDLEQAISQRIQTNNNPFQVPI------E	126
_XP_025301452.1_	64	RPHPHELVGKD-CRDGFYEAELCPDRCIHSFQNLGIQCVKKRDLEQAISQRIQTNNNPFQVPI------E	126
_XP_044920951.1_	64	RPHPHELVGKD-CRDGFYEAELCPDRCIHSFQNLGIQCVKKRDLEQAISQRIQTNNNPFQVPI------E	126
KAF3822254.1	64	RPHPHELVGKD-CRDGFYEAELCPDRCIHSFQNLGIQCVKKRDLEQAISQRIQTNNNPFQVPV------E	126
_XP_021540718.1_	64	RPHPHELVGKD-CRDGFYEAELCPDRCIHSFQNLGIQCVKKRDLEQAISQRIQTNNNPFQVPV------E	126
_XP_022362935.1_	64	RPHPHELVGKD-CRDGFYEAELCPDRCIHSFQNLGIQCVKKRDLEQAISQRIQTNNNPFQVPI------E	126
_XP_044115623.1_	64	RPHPHELVGKD-CRDGFYEAELCPDRCIHSFQNLGIQCVKKRDLEQAISQRIQTNNNPFQVPI------E	126
_XP_032735359.1_	64	RPHPHELVGKD-CRDGFYEAELCPDRCIHSFQNLGIQCVKKRDLEQAISQRIQTNNNPFQVPI------E	126
_XP_032270304.1_	64	RPHPHELVGKD-CRDGFYEAELCPDRCIHSFQNLGIQCVKKRDLEQAISQRIQTNNNPFQVPI------E	126
_XP_032213216.1_	64	RPHPHELVGKD-CRDGFYEAELCPDRCIHSFQNLGIQCVKKRDLEQAISQRIQTNNNPFQVPI------E	126
_XP_004478661.1_	64	RPHPHELVGKD-CRDGFYEAELCPDRCIHSFQNLGIQCVKKRDLEQAISQRIQTNNNPFQVPI------E	126
_XP_035933519.1_	64	RPHPHELVGKD-CRDGFYEAELCPDRCIHSFQNLGIQCVKKRDLEQAISQRIQTNNNPFQVPI------E	126
KAI5761432.1	64	RPHPHELVGKD-CRDGFYEAELCPDRCIHSFQNLGIQCVKKRDLEQAISQRIQTNNNPFQVPI------E	126
_XP_029813244.1_	64	RPHPHELVGKD-CRDGFYEAELCPDRCIHSFQNLGIQCVKKRDLEQAISQRIQTNNNPFQVPI------E	126
_XP_027800377.1_	64	RPHPHELVGKD-CRDGFYEAELCPDRCIHSFQNLGIQCVKKRDLEQAISQRIQTNNNPFQVPI------E	126
_XP_004852440.1_	64	RPHPHELVGKD-CRDGFYEAELCPDRCIHSFQNLGIQCVKKRDLEQAISQRIQTNNNPFQVPI------E	126
_XP_045873061.1_	64	RPHPHELVGKD-CRDGFYEAELCPDRCIHSFQNLGIQCVKKRDLEQAISQRIQTNNNPFQVPI------E	126

```
_XP_030891354.1_   64  RPHPHELVGKD-CRDGFYEAELCPDRCIHSFQNLGIQCVKKRDLEQAISQRIQTNNNPFQVPV------E  126
_CAD7670066.1_     64  RPHPHELVGKD-CRDGFYEAELCPDRCIHSFQNLGIQCVKKRDLEQAISQRIQTNNNPFQVPI------E  126
_XP_046301192.1_   64  RPHPHELVGKD-CRDGFYEAELCPDRCIHSFQNLGIQCVKKRDLEQAISQRIQTNNNPFQVPI------E  126
_XP_036059776.1_   64  RPHPHELVGKD-CRDGFYEAELCPDRCIHSFQNLGIQCVKKRDLEQAISQRIQTNNNPFQVPI------E  126
_XP_028711748.1_   64  RPHPHELVGKD-CRDGFYEAELCPDRCIHSFQNLGIQCVKKRDLEQAISQRIQTNNNPFQVPI------E  126
_XP_037696258.1_   64  RPHPHELVGKD-CRDGFYEAELCPDRCIHSFQNLGIQCVKKRDLEQAITQRIQTNNNPFQVPI------E  126
_XP_004627287.1_   64  RPHPHELVGKD-CRDGFYEAELCPDRCIHSFQNLGIQCVKKRDLEQAISQRIQTNNNPFQVPI------E  126
_XP_037371589.1_   64  RPHPHELVGKD-CRDGFYEAELCPDRSIHSFQNLGIQCVKKRDLEQAINQRIQTNNNPFQVPI------E  126
_XP_003468122.1_   64  RPHPHELVGKD-CRDGFYEAELCPDRCIHSFQNLGIQCVKKRDLEQAINQRIQTNNNPFQVPI------E  126
_XP_040826731.1_   64  RPHPHELVGKD-CRDGFYEAELCPDRCIHSFQNLGIQCVKKRDLEQAINQRIQTNNNPFQVPI------E  126
_XP_005384525.1_   64  RPHPHELVGKD-CRDGFYEAELCPDRCIHSFQNLGIQCVKKRDLEQAISQRIQTNNNPFQVPI------E  126
_XP_045711286.1_   64  RPHPHELVGKD-CRDGYYEAELCPDRCIHSFQNLGIQCVKKRDLEQAVRQRIQTNNNPFQVPI------E  126
_XP_044920955.1_   64  RPHPHELVGKD-CRDGFYEAELCPDRCIHSFQNLGIQCVKKRDLEQAISQRIQTNNNPFQVPI------E  126
_XP_047375326.1_   64  RPHPHELVGKD-CRDGFYEAELCPDRCIHSFQNLGIQCVKKRDLEQAISQRIQTNNNPFQVPI------E  126
_XP_036272436.1_   64  RPHPHELVGKD-CRDGFYEAELCPDRCIHSFQNLGIQCVKKRDLEQAISQRIQTNNNPFQVPI------E  126
_ELK13339.1_       64  RPHPHELVGKD-CRDGFYEAELCPDRCIHSFQNLGIQCVKKRDLEQAINQRIQTNNNPFQVPI------E  126
_XP_021564615.1_   64  RPHPHELVGKD-CRDGFYEAELCPDRCIHSFQNLGIQCVKKRDLEQAISQRIQTNNNPFQVPV------E  126
_XP_024902067.1_   64  RPHPHELVGKD-CRDGFYEAELCPDRCIHSFQNLGIQCVKKRDLEQAINQRIQTNNNPFQVPI------E  126
_XP_036349005.1_   64  RPHPHELVGKD-CRDGFYEAELCPDRCIHSFQNLGIQCVKKRDLEQAINQRIQTNNNPFQVPI------E  126
_XP_051683930.1_   64  RPHPHELVGKD-CRDGFYEAELCPDRCIHSFQNLGIQCVKKRDLEQAISQRIQTNNNPFQVPI------E  126
_XP_013006227.1_   64  RPHPHELVGKD-CRDGFYEAELCPDRCIHSFQNLGIQCVKKRDLEQAINQRIQTNNNPFQVPI------E  126
_XP_006893486.1_   64  RPHPHELVGKD-CRDGFYEAELCPDRCIHSFQNLGIQCVKKRDLEQAISQRIQTNNNPFQVPI------E  126
_XP_036272437.1_   64  RPHPHELVGKD-CRDGFYEAELCPDRCIHSFQNLGIQCVKKRDLEQAISQRIQTNNNPFQVPI------E  126
_ELW65121.1_       64  RPHPHELVGKD-CRDGFYEAELCPDRCIHSFQNLGIQCVKKRDLEQAISQRIQTNNNPFQVPI------E  126
_EHB04873.1_       64  RPHPHELVGKD-CRDGFYEAELCPDRCIHSFQNLGIQCVKKRDLEQAISQRIQTNNNPFQVPI------E  126
_KAB0407447.1_     64  RPHPHELVGKD-CRDGFYEAELCPDRCIHSFQNLGIQCVKKRDLEQAISQRIQTNNNPFQVPI------E  126
_KAF6105901.1_     64  RPHPHELVGKD-CRDGYYEAELCPDRCIHSFQNLGIQCVKKRDLEQAVRQRIQTNNNPFQVPI------E  126
_MXQ90627.1_       64  RPHPHELVGKD-CRDGFYEAELCPDRCIHSFQNLGIQCVKKRDLEQAISQRIQTNNNPFQVPI------E  126
_PNJ38432.1_       64  RPHPHELVGKD-CRDGFYEAELCPDRCIHSFQNLGIQCVKKRDLEQAINQRIQTNNNPFQVPI------E  126
_PNI93760.1_       64  RPHPHELVGKD-CRDGFYEAELCPDRCIHSFQNLGIQCVKKRDLEQAISQRIQTNNNPFQVPI------E  126
_KAF6438864.1_     64  RPHPHELVGKD-CRDGFYEAELCPDRCIHSFQNLGIQCVKKRDLEQAISQRIQTNNNPFQVPI------E  126
_KAF6281766.1_     64  RPHPHELVGKD-CRDGFYEAELCPDRCIHSFQNLGIQCVKKRDLEQAISQRIQTNNNPFQVPI------E  126
_XP_035119201.1_   64  RPHPHELVGKD-CRDGFYEAELCPDRCIHSFQNLGIQCVKKRDLEQAINQRIQTNNNPFQVPI------E  126
_KAF6334125.1_     64  RPHPHELVGKD-CRDGFYEAELCPDRCIHSFQNLGIQCVKKRDLEQAISQRIQTNNNPFQVPI------E  126
_XP_036785207.1_   64  RPHPHELVGKD-CRDGFYEAELCPDRCIHSFQNLGIQCVKKRDLEQAISQRIQTNNNPFQVPI------E  126
_XP_036851747.1_   64  RPHPHELVGKD-CRDGFYEAELCPDRCIHSFQNLGIQCVKKRDLEQAISQRIQTNNNPFQVPI------E  126
_XP_054367814.1_   64  RPHPHELVGKD-CRDGFYEAELCPDRCIHSFQNLGIQCVKKRDLEQAISQRIQTNNNPFQVPV------E  126
_XP_030689470.1_   64  RPHPHELVGKD-CRDGFYEAELCPDRCIHSFQNLGIQCVKKRDLEQAISQRIQTNNNPFQVPV------E  126
_XP_026946120.1_   64  RPHPHELVGKD-CRDGFYEAELCPDRCIHSFQNLGIQCVKKRDLEQAISQRIQTNNNPFQVPV------E  126
```

```
_XP_039696841.1_   64  RPHPHELVGKD-CRDGFYEAELCPDRCIHSFQNLGIQCVKKRDLEQAINQRIQTNNNPFQVPI------E  126
_1IKN_A_           64  RPHPHELVGKD-CRDGYYEADLCPDRSIHSFQNLGIQCVKKRDLEQAISQRIQTNNNPFHVPI------E  126
_XP_016775352.1_   64  RPHPHELVGKD-CRDGFYEAELCPDRCIHSFQNLGIQCVKKRDLEQAISQRIQTNNNPFQVPI------E  126
_PNI93761.1_       53  RPHPHELVGKD-CRDGFYEAELCPDRCIHSFQNLGIQCVKKRDLEQAISQRIQTNNNPFQVPI------E  115
_XP_054381607.1_   53  RPHPHELVGKD-CRDGFYEAELCPDRCIHSFQNLGIQCVKKRDLEQAINQRIQTNNNPFQVPI------E  115
_XP_011797882.1_   53  RPHPHELVGKD-CRDGFYEAELCPDRCIHSFQNLGIQCVKKRDLEQAITQRIQTNNNPFQVPI------E  115
_XP_011820578.1_   53  RPHPHELVGKD-CRDGFYEAELCPDRCIHSFQNLGIQCVKKRDLEQAITQRIQTNNNPFQVPI------E  115
_XP_024644798.1_   53  RPHPHELVGKD-CRDGFYEAELCPDRCIHSFQNLGIQCVKKRDLEQAITQRIQTNNNPFQVPI------E  115
_XP_012318394.1_   53  RPHPHELVGKD-CRDGFYEAELCPDRCIHSFQNLGIQCVKKRDLEQAINQRIQTNNNPFQVPI------E  115
_XP_035119199.1_   53  RPHPHELVGKD-CRDGFYEAELCPDRCIHSFQNLGIQCVKKRDLEQAINQRIQTNNNPFQVPI------E  115
_XP_036717345.1_   53  RPHPHELVGKD-CRDGFYEAELCPDRCIHSFQNLGIQCVKKRDLEQAISQRIQTNNNPFQVPI------E  115
_XP_051001869.1_   53  RPHPHELVGKD-CRDGFYEAELCPDRSIHSFQNLGIQCVKKRDLEQAISQRIQTNNNPFQVPI------E  115
_XP_007171011.2_   53  RPHPHELVGKD-CRDGFYEAELCPDRCIHSFQNLGIQCVKKRDLEQAISQRIQTNNNPFQVPI------E  115
_XP_033272588.1_   53  RPHPHELVGKD-CRDGFYEAELCPDRCIHSFQNLGIQCVKKRDLEQAISQRIQTNNNPFQVPV------E  115
_XP_026946119.1_   53  RPHPHELVGKD-CRDGFYEAELCPDRCIHSFQNLGIQCVKKRDLEQAISQRIQTNNNPFQVPV------E  115
_XP_048314002.1_   53  RPHPHELVGKD-CRDGFYEAELCPDRCIHSFQNLGIQCVKKRDLEQAISQRIQTNNNPFQVPV------E  115
_XP_014648508.1_   53  RPHPHELVGKD-CRDGFYEAELCPDRCIHSFQNLGIQCVKKRDLEQAISQRIQTNNNPFQVPI------E  115
_NP_001389477.1_   53  RPHPHELVGKD-CRDGYYEADLCPDRSIHSFQNLGIQCVKKRDLEQAISQRIQTNNNPFHVPI------E  115
_XP_049637117.1_   53  RPHPHELVGKD-CRDGFYEAELCPDRCIHSFQNLGIQCVKKRDLEQAISQRIQTNNNPFQVPI------E  115
_XP_054367813.1_   53  RPHPHELVGKD-CRDGFYEAELCPDRCIHSFQNLGIQCVKKRDLEQAISQRIQTNNNPFQVPV------E  115
_XP_041577696.1_   53  RPHPHELVGKD-CRDGFYEAELCPDRCIHSFQNLGIQCVKKRDLEQAISQRIQTNNNPFQVPI------E  115
_XP_005631531.1_   53  RPHPHELVGKD-CRDGFYEAELCPDRCIHSFQNLGIQCVKKRDLEQAISQRIQTNNNPFQVPI------E  115
_KAF6105898.1_     53  RPHPHELVGKD-CRDGYYEAELCPDRCIHSFQNLGIQCVKKRDLEQAVRQRIQTNNNPFQVPI------E  115
_XP_012790426.1_   53  RAHPHELVGKD-CRDGYYEAELCPDRCIHSFQNLGIQCVKKRDLEQAISQRIRTDNNPFHVPI------E  115
_XP_031245195.1_    1  ------------------------------------------------------------VPI------E    4
_NP_001391590.1_    1  ---------------------------------------------------------------------E    1
_XP_053417695.1_    1  ---------------------------------------------------------------------E    1
_XP_027240591.1_    1  --------------------------------------------------------------I------E    2
_XP_027264387.1_    1  --------------------------------------------------------------I------E    2
_XP_051001870.1_    1  ---------------------------------------------------------------------E    1
_XP_023415353.1_    1  ---------------------------------------------------------------------E    1
_XP_032747031.1_    1  ---------------------------------------------------------------------E    1
_XP_052048334.1_    1  ---------------------------------------------------------------------E    1
_XP_028019514.1_    1  ---------------------------------------------------------------------E    1
_XP_049993277.1_    1  ---------------------------------------------------------------------E    1
_XP_044617925.1_    1  ---------------------------------------------------------------------E    1
_XP_048216513.1_       ----------------------------------------------------------------------
_XP_029414057.1_    1  ---------------------------------------------------------------------E    1
_XP_049569046.1_    1  ---------------------------------------------------------------------E    1
```

```
_XP_040605354.1_    1  --------------------------------------------------------------I------E    2
_XP_048314004.1_    1  ---------------------------------------------------------------------E    1
_XP_041518059.1_    1  ---------------------------------------------------------------------E    1
_NP_001351996.1_    1  ---------------------------------------------------------------------E    1
_XP_038935449.1_    1  --------------------------------------------------------------I------E    2
_XP_038170707.1_    1  ---------------------------------------------------------------------E    1
_XP_040140051.1_    1  ---------------------------------------------------------------------E    1
_XP_052568262.1_    1  ---------------------------------------------------------------------E    1
_XP_042115241.1_    1  --------------------------------------------------------------I------E    2
_XP_054580929.1_    1  ---------------------------------------------------------------------E    1
_XP_054367811.1_    1  --------------------------------------------------------------V------E    2
_XP_027628238.1_    1  ---------------------------------------------------------------------E    1
_XP_044536462.1_   64  RPHPHELVGKD-CRDGFYEAELCPDHSIHSFQNLGIQCVKKRDLEQAIAHRMQTNNNPFNVPL------E  126
_XP_043827224.1_   64  RPHPHELVGKD-CRDGFYEAELCPDHCIHSFQNLGIQCVKKRDLEQAIAHRMQTNNNPFNVPL------E  126
_XP_027711838.1_   64  RPHPHELVGKD-CRDGFYEAELCPDHCIHSFQNLGIQCVKKRDLEQAIAHRVQTNNNPFNVPL------E  126
_XP_020824046.1_   64  RPHPHELVGKD-CRDGFYEAELCPDHCIHSFQNLGIQCVKKRDLEQAIAHRVQTNNNPFNVPL------E  126
_XP_036619952.1_   64  RPHPHELVGKD-CRDGFYEAELCPDHCIHSFQNLGIQCVKKRDLEQAIAHRMQTNNNPFNVPL------E  126
_XP_031799278.1_   64  RPHPHELVGKD-CRDGFYEADLCPDHCIHSFQNLGIQCVKKRDLEQAIAHRMQTNNNPFNVPL------E  126
_XP_007502748.1_   64  RPHPHELVGKD-CRDGFYEAELCPDHSIHSFQNLGIQCVKKRDLEQAIAHRMQTNNNPFNVPL------E  126
_XP_051821546.1_   64  RPHPHELVGKD-CRDGFYEADLSPDHCIHSFQNLGIQCVKKRDLEQAIAHRMQTNNNPFNVPL------E  126
_XP_051821547.1_   64  RPHPHELVGKD-CRDGFYEADLSPDHCIHSFQNLGIQCVKKRDLEQAIAHRMQTNNNPFNVPL------E  126
_KAF6105894.1_      5  PTLP------------------------LSFQNLGIQCVKKRDLEQAVRQRIQTNNNPFQVPI------E   44
_XP_008508783.1_    1  --------GGD-----------------LSFQNLGIQCVKKRDLEQAISQRIQTNNNPFQVPI------E   39
_MBV98069.1_        1  -AHPHPL-ERA-LGSALGEAELCPDRCIHSFQNLGIQCVKKRDLEQAISQRIQTNNNPFQVPI------E   61
_XP_045411956.1_   23  RPHPHELVGKD-CRDGFYEAELCPDRCIHSFQNLGIQCVKKRDLEQAISQRIQTNNNPFQVPI------E   85
_XP_012520962.1_   23  RPHPHELVGKD-CRDGFYEAELCPDRCIHSFQNLGIQCVKKRDLEQAISQRIQTNNNPFQVPI------E   85
_XP_043307319.1_   23  RPHPHELVGKD-CRDGFYEAELCPDRCIHSFQNLGIQCVKKRDLEQAISQRIQTNNNPFQVPI------E   85
_XP_012666134.1_   23  RPHPHELVGKD-CRDGFYEADLCPDRCIHSFQNLGIQCVKKRDLEQAISQRIQTNNNPFQVPI------E   85
_XP_011367280.1_   23  RPHPHELVGKD-CRDGFYEAELCPDRCIHSFQNLGIQCVKKRDLEQAINQRIQTNNNPFQVPI------E   85
_KAF7238569.1_      1  -----------------------------SFQNLGIQCVRKRDLDKAVAKRIETCNNPFKVPV------E   35
_KAG8146725.1_     64  RPHPHDLVGKD-CKEGYYEAELSPERSIHSFQNLGIQCVRKRDLEKAVAKRIETGNNPFNVPM------E  126
_XP_048371352.1_   64  RPHPHDLVGKD-CKDGFYEAELAAERSIHSFQNLGIQCVRKRDLDKAVAKRIETSNNPFNVPL------E  126
_XP_015272351.1_   64  RPHPHDLVGKD-CKEGFYEAELASERSIHSFQNLGIQCVRKRDLEKAIAKRIETNNNPFSVPL------E  126
_XP_054845680.1_   64  RPHPHDLVGKD-CKEGFYEAELAPERSIHSFQNLGIQCVRKRDLDKAVAKRIETNNNPFNVAV------E  126
_XP_015272352.1_   64  RPHPHDLVGKD-CKEGFYEAELASERSIHSFQNLGIQCVRKRDLEKAIAKRIETNNNPFSVPL------E  126
_XP_042295897.1_   64  RPHPHDLVGKD-CKEGYYEAELSPERSIHSFQNLGIQCVRKRDLDKAVAKRIETSNNPFNVPV------E  126
_XP_053133861.1_   64  RPHPHDLVGKD-CREGYYEAELAPERSIHSFQNLGIQCVRKRDLDKAVSKRIETSNNPFNVPM------E  126
_XP_053133862.1_   64  RPHPHDLVGKD-CREGYYEAELAPERSIHSFQNLGIQCVRKRDLDKAVSKRIETSNNPFNVPM------E  126
_KAJ6654174.1_     64  RPHPHDLVGKD-CREGYYEAELAPERSIHSFQNLGIQCVRKRDLDKAIAKRIETSNNPFNVPA------E  126
```

_XP_044304454.1_	64	RPHPHDLVGKD-CKEGFYEAELSPERSIHSFQNLGIQCVRKRDLDKAVAKRIETCNNPFKVPV------E	126
_XP_020633015.1_	63	RPHPHDLVGKD-CREGYYEAELSPERSIHSFQNLGIQCVRKRDLDKAVARRLETANNPFNVSA------E	125
_XP_028566864.1_	64	RPHPHDLVGKD-CKEGYYEAELSPERSIHSFQNLGIQCVRKRDLDKAVAKRIETNNNPFNVSM------E	126
_XP_033030397.1_	64	RPHPHDLVGKD-CREGYYEAELSPERSIHSFQNLGIQCVRKRDLDKAVAKRIETNNNPFSVSM------E	126
_XP_044304455.1_	64	RPHPHDLVGKD-CKEGFYEAELSPERSIHSFQNLGIQCVRKRDLDKAVAKRIETCNNPFKVPV------E	126
_XP_053225758.1_	64	RPHPHDLVGKD-CKEGYYEAELSPERSIHSFQNLGIQCVRKRDLDKAVAKRIETNNNPFNVSM------E	126
_XP_033030398.1_	64	RPHPHDLVGKD-CREGYYEAELSPERSIHSFQNLGIQCVRKRDLDKAVAKRIETNNNPFSVSM------E	126
_XP_034953722.1_	64	RPHPHDLVGKD-CKEGYYEAELSPERSIHSFQNLGIQCVRKRDLDKAVTKRIETNNNPFNVSM------E	126
_XP_008123768.1_	64	RPHPHDLVGKD-CKEGYYEAELSPERSIHSFQNLGIQCVRKRDLDKAVAKRIETGNNPFNVSV------E	126
KAJ7307406.1	53	RPHPHDLVGKD-CKEGYYEAELSPERSIHSFQNLGIQCVRKRDLDKAVSRRIETANNPFNVPA------E	115
_XP_013931335.1_	64	RPHPHDLVGKD-CKEGYYEAELSPERSIHSFQNLGIQCVRKRDLEKAVAKRIETGNNPYNVPM------E	126
_XP_032089926.1_	64	RPHPHDLVGKD-CKEGYYEAELSPERSIHSFQNLGIQCVRKRDLEKAVAKRIETGNNPYNVPM------E	126
_XP_026572203.1_	64	RPHPHDLVGKD-CKEGYYEAELSPERSIHSFQNLGIQCVRKRDLEKAVAKRIETGNNPFNVPM------E	126
_XP_026572204.1_	64	RPHPHDLVGKD-CKEGYYEAELSPERSIHSFQNLGIQCVRKRDLEKAVAKRIETGNNPFNVPM------E	126
_XP_034280315.1_	64	RPHPHDLVGKD-CKEGYYEAELSPERSIHSFQNLGIQCVRKRDLEKAVAKRIETGNNPFSVPM------D	126
_XP_039222066.1_	64	RPHPHDLVGKD-CKEGYYEAELSPERSIHSFQNLGIQCVRKRDLEKAVTKRIETGNNPFSVPK------E	126
_XP_015744179.1_	53	RPHPHDLVGKD-CKEGYYEAELSPERSIHSFQNLGIQCVRKRDLDKAVAKRIETGNNPYNVPM------E	115
_XP_029142061.1_	64	RPHPHDLVGKD-CKEGYYEAELSPERSIHSFQNLGIQCVRKRDLEKAVTKRIETGNNPF-MPM------E	125
AAH41188.1	64	KPHPHELVGKD-CKDGYYEAELSPDRSIHSFQNLGIQCVKKREVEDAVAHRIRTNNNPFNVSP------E	126
_XP_018112002.1_	64	KPHPHELVGKD-CKDGYYEAELSPDRSIHSFQNLGIQCVKKREVEDAVAHRIRTNNNPFNVSP------E	126
_NP_001081048.1_	64	KPHPHELVGKD-CKDGYYEAELSPDRSIHSFQNLGIQCVKKREVEDAVAHRIRTNNNPFNVSP------E	126
_XP_031755832.1_	64	KPHPHELVGKD-CKDGYYEAELSPDRSIHSFQNLGIQCVKKREVEDAIAHRIRTNNNPFNVPP------E	126
KAG8437862.1	64	KPHPHELVGKD-CKDGYYEAELSPDRNIHSFQNLGIQCVKKREVEEAIAHRIRTNNNPFNVPH------E	126
_XP_053304358.1_	64	KPHPHELVGKD-CKDGYYEADLSADRNIHSFQNLGIQCVKKREVEDAITHRIRTNNNPFNIPQ------E	126
KAG9464550.1	64	KPHPHELVGKD-CKDGYYEADLSSDRNIHSFQNLGIQCVKKREVDEAIAQRILTKNNPFNVSV------E	126
_XP_040265411.1_	64	KPHPHELVGKD-CKDGYYEADLSLDRNIHSFQNLGIQCVKKREVDDAIAQRIQTNNNPFNVPV------E	126
_XP_044125715.1_	64	KPHPHELVGKD-CKDGYYEADLSLDRNIHSFQNLGIQCVKKREVDDAIAQRIQTNNNPFNVPV------E	126
_XP_040265414.1_	64	KPHPHELVGKD-CKDGYYEADLSLDRNIHSFQNLGIQCVKKREVDDAIAQRIQTNNNPFNVPV------E	126
_XP_044125717.1_	64	KPHPHELVGKD-CKDGYYEADLSLDRNIHSFQNLGIQCVKKREVDDAIAQRIQTNNNPFNVPV------E	126
_XP_040184801.1_	64	KPHPHELVGKD-CKDGYFEADLSLDRNIHSFQNLGIQCVKKREVEEAIAQRMRTNNNPFNVSL------E	126
ALA55869.1	64	KPHPHELVGKD-CKDGYFEADLSLDRNIHSFQNLGIQCVKKREVEEAIAQRMRTNNNPFNVSV------E	126
_XP_018408357.1_	64	KPHPHELVGKD-CKDGYYEADLSPDRNIHSFQNLGIQCVKKREVEDAIAQRIRTNNNPFNVPV------E	126
_XP_053575987.1_	64	KPHPHELVGKD-CMDGYYEADLSADRNIHSFQNLGIQCVKKREVEEAIKHRIRTNNNPFNVPM------E	126
_XP_033812535.1_	64	RPHPHELVGKD-CKDGYYEAELAPERSIHSFQNLGIQCVKKRELDEAISERIRTKNNPFNVPL------E	126
_XP_030074553.1_	64	RPHPHELVGKD-CMDGYYEAELAPERSIHSFQNLGIQCVKKRELDEAISNRIRTKNNPFNVPL------E	126
_XP_029468059.1_	64	RPHPHELVGKD-CKDGYYEADLAPERSIHSFQNLGIQCVKKRELDDAISERIRTKNNPFNVSL------E	126
_XP_029468060.1_	64	RPHPHELVGKD-CKDGYYEADLAPERSIHSFQNLGIQCVKKRELDDAISERIRTKNNPFNVSL------E	126
KAJ1106220.1	64	RPHPHELVGKD-CKDGYFETDLPPERSIHSFQNLGIQCVKKRELDGAVADRIRTNNNPFNVPI------A	126
CAI5672275.1	53	KPHPHELVGKD-CKHGYYEADL-QERRIHSFQNLGIQCVKKKDVNEAITCRLQTNNNPFNIPE-----AK	115

```
_GCB80996.1_          53  KPHPHDLVGKD-CKDGYYEADL-PARSIHSFQNLGIQCVKKREVALAIQSRVSKNVNPF-----------  109
_XP_038618162.1_      64  WPHPHELVGKD-CWDGFYEADLCPDRCIHSFQNLGIQCVKKRDLEQANTQRMQTNNNPFNVLL------E  126
Consensus aa:             +PHPH-LVGKD.C+-G@YEAELtP-RsIHSFQNLGIQCVKKR-l-.AlspRIpTNNNPFpVs.......E
Consensus ss:                 eeee        eeeeee     eeee  eeeee     hhhhhhhhh          h      h

Conservation:                 7577 59799995 5          7    757779979775797599779575759 99797999
_XP_028917328.1_     127  EQRGDYDLNAVRLCFQVTVQDSA-GRPLALPPVLSQPIYDNRAPNTAELKICRVNRNSGSCLGGDEIFLL  195
_XP_038621028.1_     127  EQRGDYDLNAVRLCFQVTVQDPA-GRPLALPPVLSQPIYDNRAPNTAELKICRVNRNSGSCLGGDEIFLL  195
_RLV71517.1_         116  QRGGEYDLAAVRLCFQV-----------------------------------------CRGGDEIFLL  142
_XP_033367665.1_     122  ETGRDWE----------------------------------RAGSYWEGL-----GSAGGCRGGDEIFLL  152
_OWK50979.1_           1  QRGGEYDLAAVRLCFQVW----------------------------------------------------   18
_XP_031363427.1_      89  QRGGEYDLAAVRLCFQVWVRGP--GGLQPLPPVLSQPIYDNRAPSTAELRICRVNRNSGSCRGGDEIFLL  156
_XP_041319512.1_      35  QRGGEYDLAAVRLCFQVWVRGP--GGLRPLPPVLSQPIYDNRAPSTAELRICRVNRNSGSCRGGDEIFLL  102
_XP_054509575.1_     116  QRGGEYDLAAVRLCFQVWVRGP--GGLRPLPPVLSQPIYDNRAPSTAELRICRVNRNSGSCRGGDEIFLL  183
_XP_039909596.1_     116  QRGGEYDLAAVRLCFQVWVRGP--GGLRPLPPVLSQPIYDNRAPSTAELRICRINRNSGSCRGGDEIFLL  183
_TRZ10035.1_         116  QRGGEYDLAAVRLCFQVWVRGP--GGLRPLPPVLSQPIYDNRAPSTAELRICRVNRNSGSCRGGDEIFLL  183
_XP_031950790.1_     116  QRGGEYDLAAVRLCFQVWVGGP--GGLRPLPPVLSQPVYDNRAPSTAELRICRVNRNSGSCRGGDEIFLL  183
_XP_050163562.1_     116  QRGGEYDLAAVRLCFQVWVRGP--GGLRPLPPVLSQPIYDNRAPSTAELRICRVNRNSGSCRGGDEIFLL  183
_XP_039428642.1_     116  QRGGEYDLAAVRLCFQVWVXGP--GGLRPLPPVLSQPVYDNRAPSTAELRICRVNRNSGSCRGGDEIFLL  183
_XP_050840410.1_     131  QRGGEYDLAAVRLCFQVWVRGP--GGLRPLPPVLSQPIYDNRAPSTAELRICRVNRNSGSCRGGDEIFLL  198
_XP_054151317.1_     127  QRGGEYDLAAVRLCFQVWVRGP--GGLRPLPPVLSQPIYDNRAPSTAELRICRVNRNSGSCRGGDEIFLL  194
_XP_050840409.1_     127  QRGGEYDLAAVRLCFQVWVRGP--GGLRPLPPVLSQPIYDNRAPSTAELRICRVNRNSGSCRGGDEIFLL  194
_XP_030825825.1_     127  QRGGEYDLAAVRLCFQVWVRGP--GGLRPLXPVLSQPIYDNRAPSTAELRICRVNRNSGSCRGGDEIFLL  194
_XP_032939479.1_     127  QRGGEYDLAAVRLCFQVWVRGP--GGLRPLPPVLSQPIYDNRAPSTAELRICRVNRNSGSCRGGDEIFLL  194
_XP_041567964.1_     127  QRGGEYDLAAVRLCFQVWVRGP--GGLQPLPPVLSQPIYDNRAPSTAELRICRVNRNSGSCRGGDEIFLL  194
_XP_048149083.1_     127  QRGGEYDLAAVRLCFQVWVGGP--GGLRPLPPVLSQPVYDNRAPSTAELRICRVNRNSGSCRGGDEIFLL  194
_XP_054374241.1_     127  QRGGEYDLAAVRLCFQVWVRGP--GGLRPLPPVLSQPIYDNRAPSTAELRICRVNRNSGSCRGGDEIFLL  194
_NXV55901.1_         127  QRGGEYDLAAVRLCFQVWVRGP--GGLRPLPPVLSQPIYDNRAPSTAELRICRVNRNSGSCRGGDEIFLL  194
_XP_005533697.1_     127  QRGGEYDLAAVRLCFQVWVRVP--GGLRPLPPVLSQPIYDNRAPSTAELRICRVNRNSGSCRGGDEIFLL  194
_XP_014748521.1_     127  QRGGEYDLAAVRLCFQVWVRGP--GGLRPLPPVLSQPIYDNRAPSTAELRICRVNRNSGSCRGGDEIFLL  194
_RMB90491.1_         127  QRGGEYDLAAVRLCFQVWVRGP--GGLRPLPPVLSQPIYDNRAPSTAELRICRINRNSGSCRGGDEIFLL  194
_XP_053857772.1_     127  QRGGEYDLAAVRLCFQVWVRGP--GGLQPLPPVLSQPIYDNRAPSTAELRICRVNRNSGSCRGGDEIFLL  194
_XP_029862760.1_       1  QRGGEYDLSAVRLCFQVWVRGP--GGLHPLPPVLSQPVYDNRAPSTAELRICRVNRNSGSCRGGDEIFLL   68
_XP_039575719.1_     102  QRGGEYDLAAVRLCFQVWVRGP--GGLRPLPPVLSQPIYDNRAPSTAELRICRVNRNSGSCRGGDEIFLL  169
_XP_054664313.1_     127  QRGGEYDLSAVRLCFQVWVRGP--GGLHPLPPVLSQPVYDNRAPNTAELRICRVNRNSGSCRGGDEIFLL  194
_XP_049691648.1_     127  QRGGEYDLSAVRLCFQVWVRGP--GGLHPLPPVLSQPVYDNRAPSTAELRICRVNRNSGSCRGGDEIFLL  194
_XP_030331239.1_     127  QRGGEYDLSSVRLCFQVWVRGWG-GGWLPLPPVLSQPVYDNRAPSAAELRICRVNRNCGSCRGGDEIFLL  195
_XP_010726496.2_       1  --GAEYDLSAVRLCFQVWLQGP--GGLSPLPPVLSQPIYDNRAPSTAELRICRVNRNSGSCQGGDEIFLL   66
```

```
_XP_042747731.1_     1  --GAEYDLSAVRLCFQVWLPGP--GGLCPLPPVLSQPIYDNRAPSTAELRICRVNRNSGSCQGGDEIFLL   66
_XP_031466227.1_   127  ERGAEYDLSAVRLCFQVWLHSP--GGLCPLPPVLSQPIYDNRAPSTAELRICRVNRNSGSCQGGDEIFLL  194
_XP_032297702.1_   127  ERGAEYDLSSVRLCFQVWLNGP--GGLQMLPPVLSQPIYDNRAPSTAELRICRVNRNSGSCQGGDEIFLL  194
_RMB90505.1_        68  --RGEYDLAAVRLCFQVW-----------------------GAPSTAELRICRINRNSGSCRGGDEIFLL  112
_KAI2561003.1_     116  EQRGDYDLNAVRLCFQVTVRDPS-GRPLRLPPVLSHPIFDNRAPNTAELKICRVNRNSGSCLGGDEIFLL  184
_KAF6105893.1_     127  EQRGDYDLNAVRLCFQVTVRDPS-GRPLRLSPVLSHPIFDNRAPNTAELKICRVNRNSGSCLGGDEIFLL  195
_PNJ38430.1_       127  EQRGDYDLNAVRLCFQVTVRDPS-GRPLRLPPVLSHPIFDNRAPNTAELKICRVNRNSGSCLGGDEIFLL  195
_PNI93749.1_       127  EQRGDYDLNAVRLCFQVTVREPS-GRPLRLPPVLSHPIFDNRAPNTAELKICRVNRNSGSCLGGDEIFLL  195
_KAF6334124.1_     127  EQRGDYDLNAVRLCFQVTVRDPS-GRPLRLSPVLSHPIFDNRAPNTAELKICRVNRNSGSCLGGDEIFLL  195
_KAF6467432.1_     127  EQRGDYDLNAVRLCFQVTVRDPS-GRPLCLSPVLSHPIFDNRAPNTAELKICRVNRNSGSCLGGDEIFLL  195
_PNI93759.1_       127  EQRGDYDLNAVRLCFQVTVREPS-GRPLRLPPVLSHPIFDNRAPNTAELKICRVNRNSGSCLGGDEIFLL  195
_PNJ38434.1_       127  EQRGDYDLNAVRLCFQVTVRDPS-GRPLRLPPVLSHPIFDNRAPNTAELKICRVNRNSGSCLGGDEIFLL  195
_KAH0508019.1_     134  DQRGDYDLNAVRLCFQVTVRDPS-GRPLRLTPVLSHPIFDNRAPNTAELKICRVNRNSGSCLGGDEIFLL  202
_AAA36408.1_       127  EQRGDYDLNAVRLCFQVTVRDPS-GRPLRLPPVLPHPIFDNRAPNTAELKICRVNRNSGSCLGGDEIFLL  195
_XP_003274247.2_   127  EQRGDYDLNAVRLCFQVTVRDPS-GRPLRLPPVLSHPIFDNRAPNTAELKICRVNRNSGSCLGGDEIFLL  195
_XP_007981896.1_   127  EQRGDYDLNAVRLCFQVTVRDPS-GRPLRLPPVLSHPIFDNRAPNTAELKICRVNRNSGSCLGGDEIFLL  195
_XP_054296639.1_   127  EQRGDYDLNAVRLCFQVTVRDPS-GRPLRLPPVLSHPIFDNRAPNTAELKICRVNRNSGSCLGGDEIFLL  195
_XP_023042293.1_   127  EQRGDYDLNAVRLCFQVTVRDPS-GRPLRLPPVLSHPIFDNRAPNTAELKICRVNRNSGSCLGGDEIFLL  195
_XP_025211457.1_   127  EQRGDYDLNAVRLCFQVTVRDPS-GRPLRLPPVLSHPIFDNRAPNTAELKICRVNRNSGSCLGGDEIFLL  195
_XP_039326898.1_   127  EQRGDYDLNAVRLCFQVTVRDPS-GRPLRLPPVLSHPIFDNRAPNTAELKICRVNRNSGSCLGGDEIFLL  195
_XP_021781237.2_   127  EQRGDYDLNAVRLCFQVTVRDPS-GRPLRLPPVLSHPIFDNRAPNTAELKICRVNRNSGSCLGGDEIFLL  195
_XP_010364772.1_   127  EQRGDYDLNAVRLCFQVTVRDPS-GRPLCLPPVLSHPIFDNRAPNTAELKICRVNRNSGSCLGGDEIFLL  195
_XP_033063134.1_   127  EQRGDYDLNAVRLCFQVTVRDPS-GRPLCLPPVLSHPIFDNRAPNTAELKICRVNRNSGSCLGGDEIFLL  195
_XP_017392927.1_   127  EQRGDYDLNAVRLCFQVTVRDPS-GRPLRLPPVLSHPIFDNRAPNTAELKICRVNRNSGSCLGGDEIFLL  195
_XP_011897055.1_   127  EQRGDYDLNAVRLCFQVTVRDPS-GRPLRLPPVLSHPIFDNRAPNTAELKICRVNRNSGSCLGGDEIFLL  195
_XP_032138725.1_   127  EQRGDYDLNAVRLCFQVTVRDPS-GRPLRLPPVLSHPIFDNRAPNTAELKICRVNRNSGSCLGGDEIFLL  195
_CAA80524.2_       127  EQRGDYDLNAVRLCFQVTVRDPS-GRPLRLPPVLSHPIFDNRAPNTAELKICRVNRNSGSCLGGDEIFLL  195
_XP_005577305.2_   127  EQRGDYDLNAVRLCFQVTVRDPS-GRPLRLPPVLSHPIFDNRAPNTAELKICRVNRNSGSCLGGDEIFLL  195
_BAE87977.1_       127  EQRGDYDLNAVRLCFQVTVRDPS-GRPLRLPPVLSHPIFDNRAPNTAELKICRVNRNSGSCLGGDEIFLL  195
_XP_023042294.1_   127  EQRGDYDLNAVRLCFQVTVRDPS-GRPLRLPPVLSHPIFDNRAPNTAELKICRVNRNSGSCLGGDEIFLL  195
_XP_050612617.1_   127  EQRGDYDLNAVRLCFQVTVRDPS-GRPLRLPPVLSHPIFDNRAPNTAELKICRVNRNSGSCLGGDEIFLL  195
_EHH56082.1_       127  EQRGDYDLNAVRLCFQVTVRDPS-GRPLRLPPVLSHPIFDNRAPNTAELKICRVNRNSGSCLGGDEIFLL  195
_XP_028689064.1_   127  EQRGDYDLNAVRLCFQVTVRDPS-GRPLRLPPVLSHPIFDNRAPNTAELKICRVNRNSGSCLGGDEIFLL  195
_XP_045411957.1_   127  EQRGDYDLNAVRLCFQVTVRDPS-GRPLRLPPVLSHPIFDNRAPNTAELKICRVNRNSGSCLGGDEIFLL  195
_XP_012520963.1_   127  EQRGDYDLNAVRLCFQVTVRDPS-GRPLRLPPVLSHPIFDNRAPNTAELKICRVNRNSGSCLGGDEIFLL  195
_XP_012633553.1_   127  EQRGDYDLNAVRLCFQVTVRDSS-GRPLRLPPVLSHPIFDNRAPNTAELKICRVNRNSGSCLGGDEIFLL  195
_XP_053417694.1_   127  EQRGDYDLNAVRLCFQVTVRDPS-GRPLRLPPVLSHPIFDNRAPNTAELKICRVNRNSGSCLGGDEIFLL  195
_XP_032976853.1_   127  EQRGDYDLNAVRLCFQVTVRDPS-GRPLRLSPVLSHPIFDNRAPNTAELKICRVNRNSGSCLGGDEIFLL  195
_XP_008576943.1_   127  EQRGDYDLNAVRLCFQVTVRDPS-GRPLRLPPVLSHPIFDNRAPNTAELKICRVNRNSGSCLGGDEIFLL  195
```

```
_XP_032345730.1_  127  EQRGDYDLNAVRLCFQVTVRDPA-GRPLRLSPVLSHPIFDNRAPNTAELKICRVNRNSGSCLGGDEIFLL  195
_XP_010955442.1_  127  EQRGDYDLNAVRLCFQVTVRDPA-GRPLRLSPVLSHPIFDNRAPNTAELKICRVNRNSGSCLGGDEIFLL  195
_XP_031303872.1_  127  EQRGDYDLNAVRLCFQVTVRDPA-GRPLHLSPVLSHPIFDNRAPNTAELKICRVNRNSGSCLGGDEIFLL  195
_XP_032976854.1_  127  EQRGDYDLNAVRLCFQVTVRDPS-GRPLRLSPVLSHPIFDNRAPNTAELKICRVNRNSGSCLGGDEIFLL  195
_XP_053780986.1_  127  EQRGDYDLNAVRLCFQVTVRDPS-GRPLRLSPVLSHPIFDNRAPNTAELKICRVNRNSGSCLGGDEIFLL  195
_XP_036115166.1_  127  EQRGDYDLNAVRLCFQVTVRDPS-GRPLRLSPVLSHPIFDNRAPNTAELKICRVNRNSGSCLGGDEIFLL  195
_XP_043307320.1_  127  EQRGDYDLNAVRLCFQVTVRDPA-GRPLRLVPVLSHPIFDNRAPNTAELKICRVNRNSGSCLGGDEIFLL  195
_EQB77721.1_      127  EQRGDYDLNAVRLCFQVTVRDPA-GRPLRLSPVLSHPIFDNRAPNTAELKICRVNRNSGSCLGGDEIFLL  195
_NP_001107753.1_  127  EQRGDYDLNAVRLCFQVTVRDPA-GRPLRLPPVLSHPIFDNRAPNTAELKICRVNRNSGSCLGGDEIFLL  195
_XP_036785182.1_  127  EQRGDYDLNAVRLCFQVTVRDPA-GRPLRLSPVLSHPIFDNRAPNTAELKICRVNRNSGSCLGGDEIFLL  195
_XP_020762118.1_  127  EQRGDYDLNAVRLCFQVTVRDPA-GRPLRLVPVLSHPIFDNRAPNTAELKICRVNRNSGSCLGGDEIFLL  195
_XP_031546756.1_  127  EQRGDYDLNAVRLCFQVTVRDPA-GRPLRLSPVLSHPIFDNRAPNTAELKICRVNRNSGSCLGGDEIFLL  195
_XP_023989099.1_  127  EQRGDYDLNAVRLCFQVTVRDPA-GRPRRLPPVLSHPIFDNRAPNTAELKICRVNRNSGSCLGGDEIFLL  195
_KAF4009900.1_    127  EQRGDYDLNAVRLCFQVTVRDPA-GRPLRLVPVLSHPIFDNRAPNTAELKICRVNRNSGSCLGGDEIFLL  195
_KAB0376704.1_    127  EQRGDYDLNAVRLCFQVTVRDPA-GRPLRLVPVLSHPIFDNRAPNTAELKICRVNRNSGSCLGGDEIFLL  195
_KAB0364713.1_    127  EQRGDYDLNAVRLCFQVTVRDPA-GRPLRLVPVLSHPIFDNRAPNTAELKICRVNRNSGSCLGGDEIFLL  195
_CBT01657.1_      127  EQRGDYDLNAVRLCFQVTVRDPA-GRPLRLPPVLSHPIFDNRAPNTAELKICRVNRNSGSCLGGDEIFLL  195
_XP_036851744.1_  127  EQRGDYDLNAVRLCFQVTVRDPA-GRPLRLSPVLSHPIFDNRAPNTAELKICRVNRNSGSCLGGDEIFLL  195
_XP_007128247.1_  127  EQRGDYDLNAVRLCFQVTVRDPA-GRPRRLPPVLSHPIFDNRAPNTAELKICRVNRNSGSCLGGDEIFLL  195
_XP_003798713.1_  127  EQRGDYDLNAVRLCFQVTVRDPS-GRPLRLPPVLSHPIFDNRAPNTAELKICRVNRNSGSCLGGDEIFLL  195
_NP_001073711.1_  127  EQRGDYDLNAVRLCFQVTVRDPA-GRPLRLAPVLSHPIFDNRAPNTAELKICRVNRNSGSCLGGDEIFLL  195
_XP_006056522.1_  127  EQRGDYDLNAVRLCFQVTVRDPA-GRPLRLAPVLSHPIFDNRAPNTAELKICRVNRNSGSCLGGDEIFLL  195
_XP_019593326.1_  127  EQRGDYDLNAVRLCFQVTVRDPS-GRPLRLSPVLSHPIFDNRAPNTAELKICRVNRNSGSCLGGDEIFLL  195
_XP_005894159.1_  127  EQRGDYDLNAVRLCFQVTVRDPA-GRPLRLAPVLSHPIFDNRAPNTAELKICRVNRNSGSCLGGDEIFLL  195
_ELR57541.1_      127  EQRGDYDLNAVRLCFQVTVRDPA-GRPLRLAPVLSHPIFDNRAPNTAELKICRVNRNSGSCLGGDEIFLL  195
_XP_010836092.1_  127  EQRGDYDLNAVRLCFQVTVRDPA-GRPLRLAPVLSHPIFDNRAPNTAELKICRVNRNSGSCLGGDEIFLL  195
_XP_051049621.1_  127  EQRGDYDLNAVRLCFQVTVRDPS-GRPLRLTPVLSHPIFDNRAPNTAELKICRVNRNSGSCLGGDEIFLL  195
_CBT01658.1_      127  EQRGDYDLNAVRLCFQVTVRDPA-GRPLRLPPVLSHPIFDNRAPNTAELKICRVNRNSGSCLGGDEIFLL  195
_XP_015974930.1_  127  EQRGDYDLNAVRLCFQVTVRDPS-GRPLCLSPVLSHPIFDNRAPNTAELKICRVNRNSGSCLGGDEIFLL  195
_XP_027264386.1_  127  EQRGDYDLNAVRLCFQVTVRDPS-GRPLRLTPVLSHPIFDNRAPNTAELKICRVNRNSGSCLGGDEIFLL  195
_XP_040112158.1_  127  EQRGDYDLNAVRLCFQVTVRDPA-GRPLRLAPVLSHPIFDNRAPNTAELKICRVNRNSGSCLGGDEIFLL  195
_XP_052517633.1_  127  EQRGDYDLNAVRLCFQVTVRDPA-GRPLRLAPVLSHPIFDNRAPNTAELKICRVNRNSGSCLGGDEIFLL  195
_XP_017898873.1_  127  EQRGDYDLNAVRLCFQVTVRDPA-GRPLRLAPVLSHPIFDNRAPNTAELKICRVNRNSGSCLGGDEIFLL  195
_XP_020020484.1_  127  EQRGDYDLNAVRLCFQVTVRDPS-GRPLRLPPVLSHPIFDNRAPNTAELKICRVNRNSGSCLGGDEIFLL  195
_XP_047631223.1_  127  EQRGDYDLNAVRLCFQVTVRDPA-GRPLRLPPVLSHPIFDNRAPNTAELKICRVNRNSGSCLGGDEIFLL  195
_XP_007651509.2_  127  EQRGDYDLNAVRLCFQVTVRDPS-GRPLRLTPVLSHPIFDNRAPNTAELKICRVNRNSGSCLGGDEIFLL  195
_XP_027815096.1_  127  EQRGDYDLNAVRLCFQVTVRDPA-GRPLRLAPVLSHPIFDNRAPNTAELKICRVNRNSGSCLGGDEIFLL  195
_XP_034377707.1_  127  EQRGDYDLNAVRLCFQVTVRDPS-GRPLRLTPVLSHPIFDNRAPNTAELKICRVNRNSGSCLGGDEIFLL  195
_ERE77884.1_      127  EQRGDYDLNAVRLCFQVTVRDPS-GRPLRLTPVLSHPIFDNRAPNTAELKICRVNRNSGSCLGGDEIFLL  195
```

```
_XP_028626823.1_  127  EQRGDYDLNAVRLCFQVTVRDPS-GRPLRLTPVLSHPIFDNRAPNTAELKICRVNRNSGSCLGGDEIFLL  195
_KAF5928161.1_    127  EQRGDYDLNAVRLCFQVTVRDPA-GRPLRLSPVLSHPIFDNRAPNTAELKICRVNRNSGSCLGGDEIFLL  195
_KAI5933031.1_    127  EQRGDYDLNAVRLCFQVTVRDPA-GRPLRLSPVLSHPIFDNRAPNTAELKICRVNRNSGSCLGGDEIFLL  195
_XP_021007720.1_  127  EQRGDYDLNAVRLCFQVTVRDSA-GRPLLLTPVLSHPIFDNRAPNTAELKICRVNRNSGSCLGGDEIFLL  195
_XP_026252329.1_  127  EQRGDYDLNAVRLCFQVTVRDPS-GRPLCLPPVLSHPIFDNRAPNTAELKICRVNRNSGSCLGGDEIFLL  195
_XP_054426842.1_  127  EQRGDYDLNAVRLCFQVTVRDPS-GRPLRLSPVLSHPIFDNRAPNTAELKICRVNRNSGSCLGGDEIFLL  195
_XP_032747030.1_  127  EQRGDYDLNAVRLCFQVTVRDPS-GRPLRLTPVLSHPIFDNRAPNTAELKICRVNRNSGSCLGGDEIFLL  195
_XP_049748463.1_  127  EQRGDYDLNAVRLCFQVTVRDPS-GRPLRLPPVLSHPIFDNRAPNTAELKICRVNRNSGSCLGGDEIFLL  195
_KAJ1065952.1_    127  EQRGDYDLNAVRLCFQVTVRDPA-GRPLRLAPVLSHPIFDNRAPNTAELKICRVNRNSGSCLGGDEIFLL  195
_XP_052048317.1_  127  EQRGDYDLNAVRLCFQVTVRDPS-GRPLRLTPVLSHPIFDNRAPNTAELKICRVNRNSGSCLGGDEIFLL  195
_XP_012876614.1_  127  EQRGDYDLNAVRLCFQVTVRDPS-GRPLRLPPVLSHPIFDNRAPNTAELKICRVNRNSGSCLGGDEIFLL  195
_XP_020020482.1_  127  EQRGDYDLNAVRLCFQVTVRDPS-GRPLRLPPVLSHPIFDNRAPNTAELKICRVNRNSGSCLGGDEIFLL  195
_XP_010597340.1_  127  EQRGDYDLNAVRLCFQVTVRDPS-GRPLRLPPVLSHPIFDNRAPNTAELKICRVNRNSGSCLGGDEIFLL  195
_XP_026339000.2_  127  EQRGDYDLNAVRLCFQVTVRDPA-GRPLRLSPVLSHPIFDNRAPNTAELKICRVNRNSGSCLGGDEIFLL  195
_XP_030689468.1_  127  EQRGDYDLNAVRLCFQVTVRDPA-GRPLRLSPALSHPIFDNRAPNTAELKICRVNRNSGSCLGGDEIFLL  195
_XP_049993276.1_  127  DQRGDYDLNAVRLCFQVTVRDPS-GRPLRLTPVLSHPIFDNRAPNTAELKICRVNRNSGSCLGGDEIFLL  195
_XP_014700317.1_  127  EQRGDYDLNAVRLCFQVTVRDPA-GRPLRLSPVLSHPIFDNRAPNTAELKICRVNRNSGSCLGGDEIFLL  195
_XP_021494126.1_  127  EQRGDYDLNAVRLCFQVTVRDPS-GRPLRLPPVLSHPIFDNRAPNTAELKICRVNRNSGSCLGGDEIFLL  195
_XP_022447167.1_  127  EQRGDYDLNAVRLCFQVTVRDPA-GRPLRLSPALSHPIFDNRAPNTAELKICRVNRNSGSCLGGDEIFLL  195
_XP_042532137.1_  127  EQRGDYDLNAVRLCFQVTVRDPS-GRPLRLPPVLSHPIFDNRAPNTAELKICRVNRNSGSCLGGDEIFLL  195
_XP_008820421.1_  127  EQRGDYDLNAVRLCFQVTVCDPS-GRPLRLPPVLSHPIFDNRAPNTAELKICRVNRNSGSCLGGDEIFLL  195
_XP_023415352.1_  127  EQRGDYDLNAVRLCFQVTVRDPS-GRPLRLPPVLSHPIFDNRAPNTAELKICRVNRNSGSCLGGDEIFLL  195
_XP_029414056.1_  127  EQRGDYDLNAVRLCFQVTVCDPS-GRPLRLPPVLSHPIFDNRAPNTAELKICRVNRNSGSCLGGDEIFLL  195
_XP_024604853.1_  127  EQRGDYDLNAVRLCFQVTVRDPA-GRPLRLSPALSHPIFDNRAPNTAELKICRVNRNSGSCLGGDEIFLL  195
_XP_007462052.1_  127  EQRGDYDLNAVRLCFQVTVRDPA-GRPLRLSPALSHPIFDNRAPNTAELKICRVNRNSGSCLGGDEIFLL  195
_XP_006101621.1_  127  EQRGDYDLNAVRLCFQVTVRDPS-GRPLRLSPVLSHPIFDNRAPNTAELKICRVNRNSGSCLGGDEIFLL  195
_XP_005064049.1_  127  EQRGDYDLNAVRLCFQVTVRDPS-GRPLRLTPVLSHPIFDNRAPNTAELKICRVNRNSGSCLGGDEIFLL  195
_XP_031245193.1_  127  EQRGDYDLNAVRLCFQVTVRDPS-GRPLLLTPVLSHPIFDNRAPNTAELKICRVNRNSGSCLGGDEIFLL  195
_ELK24332.1_      127  EQRGDYDLNAVRLCFQVTVRDPS-GRPLRLSPVLSHPIFDNRAPNTAELKICRVNRNSGSCLGGDEIFLL  195
_XP_041518057.1_  127  DQRGDYDLNAVRLCFQVTVRDPS-GRPLRLTPVLSHPIFDNRAPNTAELKICRVNRNSGSCLGGDEIFLL  195
_XP_005351804.2_  127  DQRGDYDLNAVRLCFQVTVRDPS-GRPLRLTPVLSHPIFDNRAPNTAELKICRVNRNSGSCLGGDEIFLL  195
_EGW13067.1_      127  EQRGDYDLNAVRLCFQVTVRDPS-GRPLRLTPVLSHPIFDNRAPNTAELKICRVNRNSGSCLGGDEIFLL  195
_ERE77883.1_      127  EQRGDYDLNAVRLCFQVTVRDPS-GRPLRLTPVLSHPIFDNRAPNTAELKICRVNRNSGSCLGGDEIFLL  195
_KFO28401.1_      127  EQRGDYDLNAVRLCFQVTVRDQS-GRPLRLPPVLSHPIFDNRAPNTAELKICRVNRNSGSCLGGDEIFLL  195
_XP_010634478.1_  127  EQRGDYDLNAVRLCFQVTVRDQS-GRPLRLPPVLSHPIFDNRAPNTAELKICRVNRNSGSCLGGDEIFLL  195
_OBS81518.1_      127  EQRGDYDLNAVRLCFQVTVRDPS-GRPLRLTPVLSHPIFDNRAPNTAELKICRVNRNSGSCLGGDEIFLL  195
_XP_007462051.1_  127  EQRGDYDLNAVRLCFQVTVRDPA-GRPLRLSPALSHPIFDNRAPNTAELKICRVNRNSGSCLGGDEIFLL  195
_XP_014393535.1_  127  EQRGDYDLNAVRLCFQVTVRDPS-GRPLRLSPVLSHPIFDNRAPNTAELKICRVNRNSGSCLGGDEIFLL  195
_XP_048216512.1_  127  EQRGDYDLNAVRLCFQVTVRDPS-GRPLRLPPVLSHPIFDNRAPNTAELKICRVNRNSGSCLGGDEIFLL  195
```

```
_XP_015424455.1_  127  EQRGDYDLNAVRLCFQVTVRDPS-GRPLRLSPVLSHPIFDNRAPNTAELKICRVNRNSGSCLGGDEIFLL  195
_XP_019501147.1_  127  EQRGDYDLNAVRLCFQVTVRDPS-GRPLRLSPVLSHPIFDNRAPNTAELKICRVNRNSGSCLGGDEIFLL  195
_XP_004656738.2_  127  EQRGDYDLNAVRLCFQVTIRDPS-GRPLRLPPVLSHPIFDNRAPNTAELKICRVNRNSGSCLGGDEIFLL  195
_XP_036909111.1_  127  EQRGDYDLNAVRLCFQVTVRDPS-GRPLRLSPVLSHPIFDNRAPNTAELKICRVNRNSGSCLGGDEIFLL  195
_XP_036182518.1_  127  EQRGDYDLNAVRLCFQVTVRDPS-GRPLRLSPVLSHPIFDNRAPNTAELKICRVNRNSGSCLGGDEIFLL  195
_XP_012580591.1_  127  EQRGDYDLNAVRLCFQVTVRDPT-GRPLRLSPVLSHPIFDNRAPNTAELKICRVNRNSGSCAGGDEIFLL  195
_XP_016063765.1_  127  EQRGDYDLNAVRLCFQVTVRDPS-GRPLRLAPVLSHPIFDNRAPNTAELKICRVNRNSGSCLGGDEIFLL  195
_XP_021047054.1_  127  EQRGDYDLNAVRLCFQVTVRDPA-GRPLLLTPVLSHPIFDNRAPNTAELKICRVNRNSGSCLGGDEIFLL  195
_XP_023095836.1_  127  EQRGDYDLNAVRLCFQVTVRDPA-GRPLRLPPVLSHPIFDNRAPNTAELKICRVNRNSGSCLGGDEIFLL  195
_XP_045342734.1_  127  EQRGDYDLNAVRLCFQVTVRDPA-GRPLRLPPVLSHPIFDNRAPNTAELKICRVNRNSGSCLGGDEIFLL  195
_XP_053755429.1_  127  EQRGDYDLNAVRLCFQVTVRDPA-GRPLRLPPVLSHPIFDNRAPNTAELKICRVNRNSGSCLGGDEIFLL  195
_XP_022447166.1_  127  EQRGDYDLNAVRLCFQVTVRDPA-GRPLRLSPALSHPIFDNRAPNTAELKICRVNRNSGSCLGGDEIFLL  195
_XP_004713741.1_  127  EQRGDYDLKAVRLCFQVTVRDPA-GRPLRLLPVLSHPIFDNRAPNTAELKICRVNRNSGSCLGGDEIFLL  195
_NP_954888.1_     127  EQRGDYDLNAVRLCFQVTVRDPS-GRPLRLTPVLSHPIFDNRAPNTAELKICRVNRNSGSCLGGDEIFLL  195
_XP_037002389.2_  127  EQRGDYDLNAVRLCFQVTVRDPS-GRPLRLSPVLSHPIFDNRAPNTAELKICRVNRNSGSCLGGDEIFLL  195
_XP_039100808.1_  127  EQRGDYDLNAVRLCFQVTVRDPA-GRPLRLPPVLSHPIFDNRAPNTAELKICRVNRNSGSCLGGDEIFLL  195
_XP_038170688.1_  127  EQRGDYDLNAVRLCFQVTVRDPS-GRPLRLTPVLSHPIFDNRAPNTAELKICRVNRNSGSCLGGDEIFLL  195
_XP_040495345.1_  127  EQRGDYDLNAVRLCFQVTVRDPA-GRPLRLSPVLSHPIFDNRAPNTAELKICRVNRNSGSCLGGDEIFLL  195
_XP_052568261.1_  127  EQRGDYDLNAVRLCFQVTVRDPS-GRPLRLTPVLSHPIFDNRAPNTAELKICRVNRNSGSCLGGDEIFLL  195
_XP_042760134.1_  127  EQRGDYDLNAVRLCFQVTVRDPA-GRPLRLPPVLSHPIFDNRAPNTAELKICRVNRNSGSCLGGDEIFLL  195
_XP_049472392.1_  127  EQRGDYDLNAVRLCFQVTVRDPA-GRPLRLPPVLSHPIFDNRAPNTAELKICRVNRNSGSCLGGDEIFLL  195
_XP_023593974.1_  127  EQRGDYDLNAVRLCFQVTVRDPS-GRPLRLPPVLSHPIFDNRAPNTAELKICRVNRNSGSCLGGDEIFLL  195
_XP_034501886.1_  127  EQRGDYDLNAVRLCFQVTVRDPA-GRPLRLSPVLSHPIFDNRAPNTAELKICRVNRNSGSCLGGDEIFLL  195
_XP_004393991.1_  127  EQRGDYDLNAVRLCFQVTVRDPA-GRPLRLSPVLSHPIFDNRAPNTAELKICRVNRNSGSCLGGDEIFLL  195
_XP_025860214.1_  127  EQRGDYDLNAVRLCFQVTVRDPA-GRPLRLSPVLSHPIFDNRAPNTAELKICRVNRNSGSCLGGDEIFLL  195
_XP_019501146.1_  127  EQRGDYDLNAVRLCFQVTVRDPS-GRPLRLSPVLSHPIFDNRAPNTAELKICRVNRNSGSCLGGDEIFLL  195
_XP_025734327.1_  127  EQRGDYDLNAVRLCFQVTVRDPA-GRPLRLSPVLSHPIFDNRAPNTAELKICRVNRNSGSCLGGDEIFLL  195
_XP_025301454.1_  127  EQRGDYDLNAVRLCFQVTVRDPA-GRPLRLSPVLSHPIFDNRAPNTAELKICRVNRNSGSCLGGDEIFLL  195
_KAG3284687.1_    127  EQRGDYDLNAVRLCFQVTVRDPS-GRPLCLPPVLSHPIFDNRAPNTAELKICRVNRNSGSCLGGDEIFLL  195
_VFV23136.1_      127  EQRGDYDLNAVRLCFQVTVRDPA-GRPLRLPPVLSHPIFDNRAPNTAELKICRVNRNSGSCLGGDEIFLL  195
_XP_025771173.1_  127  EQRGDYDLNAVRLCFQVTVRDPA-GRPLRLPPVLSHPIFDNRAPNTAELKICRVNRNSGSCLGGDEIFLL  195
_XP_054580928.1_  127  EQRGDYDLNAVRLCFQVTVRDPS-GRPLRLSPVLSHPIFDNRAPNTAELKICRVNRNSGSCLGGDEIFLL  195
_EPQ06533.1_      127  EQRGDYDLNAVRLCFQVTVRDPS-GRPLRLSPVLSHPIFDNRAPNTAELKICRVNRNSGSCLGGDEIFLL  195
_XP_034869141.1_  127  EQRGDYDLNAVRLCFQVTVRDPA-GRPLRLSPVLSHPIFDNRAPNTAELKICRVNRNSGSCLGGDEIFLL  195
_XP_042813589.1_  127  EQRGDYDLNAVRLCFQVTVRDPA-GRPLRLPPVLSHPIFDNRAPNTAELKICRVNRNSGSCLGGDEIFLL  195
_XP_040322176.1_  127  EQRGDYDLNAVRLCFQVTVRDPA-GRPLRLPPVLSHPIFDNRAPNTAELKICRVNRNSGSCLGGDEIFLL  195
_XP_006861137.1_  127  EQRGDYDLNAVRLCFQVTVCDPS-GRPLRLPPVLSHPIFDNRAPNTAELKICRVNRNSGSCLGGDEIFLL  195
_XP_007943155.2_  127  DQRGDYDLNAVRLCFQVTVRDSS-GRPLRLPPVLSHPIFDNRAPNTAELKICRVNRNSGSCLGGDEIFLL  195
_BAQ95593.1_      127  EQRGDYDLNAVRLCFQVTVRDPA-GRPLRLSPVLSHPIFDNRAPNTAELKICRVNRNSGSCLGGDEIFLL  195
```

```
_XP_019652454.2_  127  EQRGDYDLNAVRLCFQVTVRDPA-GRPLRLSPVLSHPIFDNRAPNTAELKICRVNRNSGSCLGGDEIFLL  195
_XP_016041658.1_  127  EQRGDYDLNAVRLCFQVTVRDLA-GRPLRLPPVLSHPIFDNRAPNTAELKICRVNRNSGSCLGGDEIFLL  195
_XP_047546997.1_  127  EQRGDYDLNAVRLCFQVTVRDPA-GRPLRLSPVLSHPIFDNRAPNTAELKICRVNRNSGSCLGGDEIFLL  195
_XP_042115231.1_  127  EQRGDYDLNAVRLCFQVTVRDPA-GRPLRLTPVLSHPIFDNRAPNTAELKICRVNRNSGSCLGGDEIFLL  195
_XP_042115227.1_  127  EQRGDYDLNAVRLCFQVTVRDPA-GRPLRLTPVLSHPIFDNRAPNTAELKICRVNRNSGSCLGGDEIFLL  195
_XP_015854576.1_  127  EQRGDYDLNAVRLCFQVTVRDPA-GRPLRLTPVLSHPIFDNRAPNTAELKICRVNRNSGSCLGGDEIFLL  195
_XP_025301452.1_  127  EQRGDYDLNAVRLCFQVTVRDPA-GRPLRLSPVLSHPIFDNRAPNTAELKICRVNRNSGSCLGGDEIFLL  195
_XP_044920951.1_  127  EQRGDYDLNAVRLCFQVTVRDPA-GRPLRLSPVLSHPIFDNRAPNTAELKICRVNRNSGSCLGGDEIFLL  195
_KAF3822254.1_    127  EQRGDYDLNAVRLCFQVTVRDPA-GRPLRLSPVLSHPIFDNRAPNTAELKICRVNRNSGSCLGGDEIFLL  195
_XP_021540718.1_  127  EQRGDYDLNAVRLCFQVTVRDPA-GRPLRLSPVLSHPIFDNRAPNTAELKICRVNRNSGSCLGGDEIFLL  195
_XP_022362935.1_  127  EQRGDYDLNAVRLCFQVTVRDPA-GRPLRLSPVLSHPIFDNRAPNTAELKICRVNRNSGSCLGGDEIFLL  195
_XP_044115623.1_  127  EQRGDYDLNAVRLCFQVTVRDPA-GRPLRLSPVLSHPIFDNRAPNTAELKICRVNRNSGSCLGGDEIFLL  195
_XP_032735359.1_  127  EQRGDYDLNAVRLCFQVTVRDPA-GRPLRLSPVLSHPIFDNRAPNTAELKICRVNRNSGSCLGGDEIFLL  195
_XP_032270304.1_  127  EQRGDYDLNAVRLCFQVTVRDPA-GRPLRLSPVLSHPIFDNRAPNTAELKICRVNRNSGSCLGGDEIFLL  195
_XP_032213216.1_  127  EQRGDYDLNAVRLCFQVTVRDPA-GRPLRLSPVLSHPIFDNRAPNTAELKICRVNRNSGSCRGGDEIFLL  195
_XP_004478661.1_  127  EQRGDYDLNAVRLCFQVTVQDPA-GRPLHLPPVLSHPIFDNRAPNTAELKICRVNRNSGSCLGGDEIFLL  195
_XP_035933519.1_  127  EQRGDYDLNAVRLCFQVTVRDPA-GRPLRLSPVLSHPIFDNRAPNTAELKICRVNRNSGSCLGGDEIFLL  195
_KAI5761432.1_    127  EQRGDYDLNAVRLCFQVTVRDPA-GRPLRLSPVLSHPIFDNRAPNTAELKICRVNRNSGSCLGGDEIFLL  195
_XP_029813244.1_  127  EQRGDYDLNAVRLCFQVTVRDPA-GRPLRLPPVLSHPIFDNRAPNTAELKICRVNRNSGSCLGGDEIFLL  195
_XP_027800377.1_  127  EQRGDYDLNAVRLCFQVTVRDPS-GRPLCLPPVLSHPIFDNRAPNTAELKICRVNRNSGSCLGGDEIFLL  195
_XP_004852440.1_  127  EQRGDYDLNAVRLCFQVTVRDQS-GRPLRLPPVLSNPIFDNRAPNTAELKICRVNRNSGSCLGGDEIFLL  195
_XP_045873061.1_  127  EQRGDYDLNAVRLCFQVTVRDPA-GRPLRLSPVLSHPIFDNRAPNTAELKICRVNRNSGSCLGGDEIFLL  195
_XP_030891354.1_  127  EQRGDYDLNAVRLCFQVTVRDPA-GRPLRLSPVLSHPIFDNRAPNTAELKICRVNRNSGSCLGGDEIFLL  195
_CAD7670066.1_    127  EQRGDYDLNAVRLCFQVTVRDPA-GRPLRLSPVLSHPIFDNRAPNTAELKICRVNRNSGSCLGGDEIFLL  195
_XP_046301192.1_  127  EQRGDYDLNAVRLCFQVTVRDPS-GRPLCLPPVLSHPIFDNRAPNTAELKICRVNRNSGSCLGGDEIFLL  195
_XP_036059776.1_  127  EQRGDYDLNAVRLCFQVTVRDPS-GRPLRLTPALSHPIFDNRAPNTAELKICRVNRNSGSCLGGDEIFLL  195
_XP_028711748.1_  127  EQRGDYDLNAVRLCFQVTVRDPA-GRPLRLTPVLSHPIFDNRAPNTAELKICRVNRNSGSCLGGDEIFLL  195
_XP_037696258.1_  127  EQRGDYDLNAVRLCFQVTVRDPA-GRPLRLPPVLSHPIFDNRAPNTAELKICRVNRNSGSCLGGDEIFLL  195
_XP_004627287.1_  127  EQRGDYDLNAVRLCFQVTVRDQS-GRPLRLPPVLSHPIFDNRAPNTAELKICRVNRNSGSCLGGDEIFLL  195
_XP_037371589.1_  127  EQRGDYDLNAVRLCFQVTVRDPA-GRPLRLSPVLSHPIFDNRAPNTAELKICRVNRNSGSCLGGDEIFLL  195
_XP_003468122.1_  127  EQRGDYDLNAVRLCFQVTVRDQS-GRPLRLPPVLSHPIFDNRAPNTAELKICRVNRNSGSCLGGDEIFLL  195
_XP_040826731.1_  127  EQRGDYDLNAVRLCFQVTVRDPS-GRPLRLPPVLSHPIFDNRAPNTAELKICRVNRNSGSCLGGDEIFLL  195
_XP_005384525.1_  127  EQRGDYDLNAVRLCFQVTVRDQS-GRPLRLPPVLSHPIFDNRAPNTAELKICRVNRNSGSCLGGDEIFLL  195
_XP_045711286.1_  127  EQRGDYDLNAVRLCFQVTVRDSS-GRPLRLSPVLSHPIFDNRAPNTAELKICRVNRNSGSCLGGDEIFLL  195
_XP_044920955.1_  127  EQRGDYDLNAVRLCFQVTVRDPA-GRPLRLSPVLSHPIFDNRAPNTAELKICRVNRNSGSCLGGDEIFLL  195
_XP_047375326.1_  127  EQRGDYDLNAVRLCFQVTVRDPS-GRPLCLPPVLSHPIFDNRAPNTAELKICRVNRNSGSCLGGDEIFLL  195
_XP_036272436.1_  127  EQRGDYDLNAVRLCFQVTVRDPS-GRPLRLPPVLSHPIFDNRAPNTAELKICRVNRNSGSCLGGDEIFLL  195
_ELK13339.1_      127  EQRGDYDLNAVRLCFQVTVRDPS-GRPLRLSPVLSHPIFDNRAPNTAELKICRVNRNSGSCLGGDEIFLL  195
_XP_021564615.1_  127  EQRGDYDLNAVRLCFQVTVRDPS-GRPLRLQPVLSHPIFDNRAPNTAELKICRVNRNSGSCLGGDEIFLL  195
```

```
_XP_024902067.1_  127  EQRGDYDLNAVRLCFQVTVRDPS-GRPLRLSPVLSHPIFDNRAPNTAELKICRVNRNSGSCLGGDEIFLL  195
_XP_036349005.1_  127  EQRGDYDLNAVRLCFQVTVRDPS-GRPLRLPPVLSHPIFDNRAPNTAELKICRVNRNSGSCLGGDEIFLL  195
_XP_051683930.1_  127  EQRGDYDLNAVRLCFQVTVRDPA-GRPLRLPPVLSHPIFDNRAPNTAELKICRVNRNSGSCLGGDEIFLL  195
_XP_013006227.1_  127  EQRGDYDLNAVRLCFQVTVRDQS-GRPLRLPPVLSHPIFDNRAPNTAELKICRVNRNSGSCLGGDEIFLL  195
_XP_006893486.1_  127  EQRGDYDLNAVRLCFQVTVRDPA-GRPLRLPPVLSHPIFDNRAPNTAELKICRVNRNSGSCL-GDEIFLL  194
_XP_036272437.1_  127  EQRGDYDLNAVRLCFQVTVRDPS-GRPLRLPPVLSHPIFDNRAPNTAELKICRVNRNSGSCLGGDEIFLL  195
_ELW65121.1_      127  EQRGDYDLNAVRLCFQVTVRDPA-GRPLRLQPVLSHPIFDNRAPNTAELKICRVNRNSGSCLGGDEIFLL  195
_EHB04873.1_      127  EQRGDYDLNAVRLCFQVTVRDQS-GRPLRLPPVLSNPIFDNRAPNTAELKICRVNRNSGSCLGGDEIFLL  195
_KAB0407447.1_    127  EQRGDYDLNAVRLCFQVTVRDPA-GRPLRLSPVLSHPIFDNRAPNTAELKICRVNRNSGSCLGGDEIFLL  195
_KAF6105901.1_    127  EQRGDYDLNAVRLCFQVTVRDPS-GRPLRLSPVLSHPIFDNRAPNTAELKICRVNRNSGSCLGGDEIFLL  195
_MXQ90627.1_      127  EQRGDYDLNAVRLCFQVTVRDPA-GRPLRLAPVLSHPIFDNRAPNTAELKICRVNRNSGSCLGGDEIFLL  195
_PNJ38432.1_      127  EQRGDYDLNAVRLCFQVTVRDPS-GRPLRLPPVLSHPIFDNRAPNTAELKICRVNRNSGSCLGGDEIFLL  195
_PNI93760.1_      127  EQRGDYDLNAVRLCFQVTVREPS-GRPLRLPPVLSHPIFDNRAPNTAELKICRVNRNSGSCLGGDEIFLL  195
_KAF6438864.1_    127  EQRGDYDLNAVRLCFQVTVRDPS-GRPLRLSPVLSHPIFDNRAPNTAELKICRVNRNSGSCLGGDEIFLL  195
_KAF6281766.1_    127  EQRGDYDLNAVRLCFQVTVRDPS-GRPLRLPPVLSHPIFDNRAPNTAELKICRVNRNSGSCLGGDEIFLL  195
_XP_035119201.1_  127  EQRGDYDLNAVRLCFQVTVRDPS-GRPLRLLPVLSHPIFDNRAPNTAELKICRVNRNSGSCLGGDEIFLL  195
_KAF6334125.1_    127  EQRGDYDLNAVRLCFQVTVRDPS-GRPLRLSPVLSHPIFDNRAPNTAELKICRVNRNSGSCLGGDEIFLL  195
_XP_036785207.1_  127  EQRGDYDLNAVRLCFQVTVRDPA-GRPLRLSPVLSHPIFDNRAPNTAELKICRVNRNSGSCLGGDEIFLL  195
_XP_036851747.1_  127  EQRGDYDLNAVRLCFQVTVRDPA-GRPLRLSPVLSHPIFDNRAPNTAELKICRVNRNSGSCLGGDEIFLL  195
_XP_054367814.1_  127  EQRGDYDLNAVRLCFQVTVRDPA-GRPLRLSPVLSHPIFDNRAPNTAELKICRVNRNSGSCLGGDEIFLL  195
_XP_030689470.1_  127  EQRGDYDLNAVRLCFQVTVRDPA-GRPLRLSPALSHPIFDNRAPNTAELKICRVNRNSGSCLGGDEIFLL  195
_XP_026946120.1_  127  EQRGDYDLNAVRLCFQVTVRDPA-GRPLRLSPALSHPIFDNRAPNTAELKICRVNRNSGSCLGGDEIFLL  195
_XP_039696841.1_  127  EQRGDYDLNAVRLCFQVTVRDPS-GRPLRLSPVLSHPIFDNRAPNTAELKICRVNRNSGSCLGGDEIFLL  195
_1IKN_A_          127  EQRGDYDLNAVRLCFQVTVRDPA-GRPLLLTPVLSHPIFDNRAPNTAELKICRVNRNSGSCLGGDEIFLL  195
_XP_016775352.1_  127  EQRGDYDLNAVRLCFQVTVREPS-GRPLRLPPVLSHPIFDN-----------------------------  166
_PNI93761.1_      116  EQRGDYDLNAVRLCFQVTVREPS-GRPLRLPPVLSHPIFDNRAPNTAELKICRVNRNSGSCLGGDEIFLL  184
_XP_054381607.1_  116  EQRGDYDLNAVRLCFQVTVRDPS-GRPLRLPPVLSHPIFDNRAPNTAELKICRVNRNSGSCLGGDEIFLL  184
_XP_011797882.1_  116  EQRGDYDLNAVRLCFQVTVRDPS-GRPLRLPPVLSHPIFDNRAPNTAELKICRVNRNSGSCLGGDEIFLL  184
_XP_011820578.1_  116  EQRGDYDLNAVRLCFQVTVRDPS-GRPLRLPPVLSHPIFDNRAPNTAELKICRVNRNSGSCLGGDEIFLL  184
_XP_024644798.1_  116  EQRGDYDLNAVRLCFQVTVRDPS-GRPLRLPPVLSHPIFDNRAPNTAELKICRVNRNSGSCLGGDEIFLL  184
_XP_012318394.1_  116  EQRGDYDLNAVRLCFQVTVRDPS-GRPLRLPPVLSHPIFDNRAPNTAELKICRVNRNSGSCLGGDEIFLL  184
_XP_035119199.1_  116  EQRGDYDLNAVRLCFQVTVRDPS-GRPLRLLPVLSHPIFDNRAPNTAELKICRVNRNSGSCLGGDEIFLL  184
_XP_036717345.1_  116  EQRGDYDLNAVRLCFQVTVRDPA-GRPLRLSPVLSHPIFDNRAPNTAELKICRVNRNSGSCLGGDEIFLL  184
_XP_051001869.1_  116  EQRGDYDLNAVRLCFQVTVRDTS-GRPLRLTPVLSHPIFDNRAPNTAELKICRVNRNSGSCLGGDEIFLL  184
_XP_007171011.2_  116  EQRGDYDLNAVRLCFQVTVRDPA-GRPLRLSPVLSHPIFDNRAPNTAELKICRVNRNSGSCLGGDEIFLL  184
_XP_033272588.1_  116  EQRGDYDLNAVRLCFQVTVRDPA-GRPLRLSPALSHPIFDNRAPNTAELKICRVNRNSGSCLGGDEIFLL  184
_XP_026946119.1_  116  EQRGDYDLNAVRLCFQVTVRDPA-GRPLRLSPALSHPIFDNRAPNTAELKICRVNRNSGSCLGGDEIFLL  184
_XP_048314002.1_  116  EQRGDYDLNAVRLCFQVTVRDPA-GRPLRLTPVLSHPIFDNRAPNTAELKICRVNRNSGSCLGGDEIFLL  184
_XP_014648508.1_  116  EQRGDYDLNAVRLCFQVTVRDPA-GRPLRLSPVLSHPIFDNRAPNTAELKICRVNRNSGSCLGGDEIFLL  184
```

_NP_001389477.1_	116	EQRGDYDLNAVRLCFQVTVRDPA-GRPLLLTPVLSHPIFDNRAPNTAELKICRVNRNSGSCLGGDEIFLL	184
_XP_049637117.1_	116	EQRGDYDLNAVRLCFQVTVRDPT-GRPLRLAPVLSHPIFDNRAPNTAELKICRVNRNSGSCLGGDEIFLL	184
_XP_054367813.1_	116	EQRGDYDLNAVRLCFQVTVRDPA-GRPLRLSPVLSHPIFDNRAPNTAELKICRVNRNSGSCLGGDEIFLL	184
_XP_041577696.1_	116	EQRGDYDLNAVRLCFQVTVRDPA-GRPLRLSPVLSHPIFDNRAPNTAELKICRVNRNSGSCLGGDEIFLL	184
_XP_005631531.1_	116	EQRGDYDLNAVRLCFQVTVRDPA-GRPLRLSPVLSHPIFDNRAPNTAELKICRVNRNSGSCLGGDEIFLL	184
KAF6105898.1	116	EQRGDYDLNAVRLCFQVTVRDPS-GRPLRLSPVLSHPIFDNRAPNTAELKICRVNRNSGSCLGGDEIFLL	184
_XP_012790426.1_	116	EQRGDYDLNAVRLCFQVTVRDIT-GRPLRLPPVLSHPIFDNRAPNTAELKICRVNRNSGSCLGGDEIFLL	184
_XP_031245195.1_	5	EQRGDYDLNAVRLCFQVTVRDPS-GRPLLLTPVLSHPIFDNRAPNTAELKICRVNRNSGSCLGGDEIFLL	73
_NP_001391590.1_	2	EQRGDYDLNAVRLCFQVTVRDPS-GRPLRLPPVLSHPIFDNRAPNTAELKICRVNRNSGSCLGGDEIFLL	70
_XP_053417695.1_	2	EQRGDYDLNAVRLCFQVTVRDPS-GRPLRLPPVLSHPIFDNRAPNTAELKICRVNRNSGSCLGGDEIFLL	70
_XP_027240591.1_	3	EQRGDYDLNAVRLCFQVTVRDPS-GRPLRLTPVLSHPIFDNRAPNTAELKICRVNRNSGSCLGGDEIFLL	71
_XP_027264387.1_	3	EQRGDYDLNAVRLCFQVTVRDPS-GRPLRLTPVLSHPIFDNRAPNTAELKICRVNRNSGSCLGGDEIFLL	71
_XP_051001870.1_	2	EQRGDYDLNAVRLCFQVTVRDTS-GRPLRLTPVLSHPIFDNRAPNTAELKICRVNRNSGSCLGGDEIFLL	70
_XP_023415353.1_	2	EQRGDYDLNAVRLCFQVTVRDPS-GRPLRLPPVLSHPIFDNRAPNTAELKICRVNRNSGSCLGGDEIFLL	70
_XP_032747031.1_	2	EQRGDYDLNAVRLCFQVTVRDPS-GRPLRLTPVLSHPIFDNRAPNTAELKICRVNRNSGSCLGGDEIFLL	70
_XP_052048334.1_	2	EQRGDYDLNAVRLCFQVTVRDPS-GRPLRLTPVLSHPIFDNRAPNTAELKICRVNRNSGSCLGGDEIFLL	70
_XP_028019514.1_	2	EQRGDYDLNAVRLCFQVTVRDPA-GRPLRLSPVLSHPIFDNRAPNTAELKICRVNRNSGSCLGGDEIFLL	70
_XP_049993277.1_	2	DQRGDYDLNAVRLCFQVTVRDPS-GRPLRLTPVLSHPIFDNRAPNTAELKICRVNRNSGSCLGGDEIFLL	70
_XP_044617925.1_	2	EQRGDYDLNAVRLCFQVTVRDPA-GRPLRLSPVLSHPIFDNRAPNTAELKICRVNRNSGSCLGGDEIFLL	70
_XP_048216513.1_	1	EQRGDYDLNAVRLCFQVTVRDPS-GRPLRLPPVLSHPIFDNRAPNTAELKICRVNRNSGSCLGGDEIFLL	69
_XP_029414057.1_	2	EQRGDYDLNAVRLCFQVTVCDPS-GRPLRLPPVLSHPIFDNRAPNTAELKICRVNRNSGSCLGGDEIFLL	70
_XP_049569046.1_	2	EQRGDYDLNAVRLCFQVTVRDPA-GRPLRLSPALSHPIFDNRAPNTAELKICRVNRNSGSCLGGDEIFLL	70
_XP_040605354.1_	3	EQRGDYDLNAVRLCFQVTVRDPS-GRPLRLTPVLSHPIFDNRAPNTAELKICRVNRNSGSCLGGDEIFLL	71
_XP_048314004.1_	2	EQRGDYDLNAVRLCFQVTVRDPA-GRPLRLTPVLSHPIFDNRAPNTAELKICRVNRNSGSCLGGDEIFLL	70
_XP_041518059.1_	2	DQRGDYDLNAVRLCFQVTVRDPS-GRPLRLTPVLSHPIFDNRAPNTAELKICRVNRNSGSCLGGDEIFLL	70
_NP_001351996.1_	2	EQRGDYDLNAVRLCFQVTVRDPA-GRPLLLTPVLSHPIFDNRAPNTAELKICRVNRNSGSCLGGDEIFLL	70
_XP_038935449.1_	3	EQRGDYDLNAVRLCFQVTVRDPS-GRPLRLTPVLSHPIFDNRAPNTAELKICRVNRNSGSCLGGDEIFLL	71
_XP_038170707.1_	2	EQRGDYDLNAVRLCFQVTVRDPS-GRPLRLTPVLSHPIFDNRAPNTAELKICRVNRNSGSCLGGDEIFLL	70
_XP_040140051.1_	2	EQRGDYDLNAVRLCFQVTVRDPS-GRPLCLPPVLSHPIFDNRAPNTAELKICRVNRNSGSCLGGDEIFLL	70
_XP_052568262.1_	2	EQRGDYDLNAVRLCFQVTVRDPS-GRPLRLTPVLSHPIFDNRAPNTAELKICRVNRNSGSCLGGDEIFLL	70
_XP_042115241.1_	3	EQRGDYDLNAVRLCFQVTVRDPA-GRPLRLTPVLSHPIFDNRAPNTAELKICRVNRNSGSCLGGDEIFLL	71
_XP_054580929.1_	2	EQRGDYDLNAVRLCFQVTVRDPS-GRPLRLSPVLSHPIFDNRAPNTAELKICRVNRNSGSCLGGDEIFLL	70
_XP_054367811.1_	3	EQRGDYDLNAVRLCFQVTVRDPA-GRPLRLSPVLSHPIFDNRAPNTAELKICRVNRNSGSCLGGDEIFLL	71
_XP_027628238.1_	2	EQRGDYDLNAVRLCFQVTVRDPA-GRPLRLQPVLSHPIFDNRAPNTAELKICRVNRNSGSCLGGDEIFLL	70
_XP_044536462.1_	127	EQRGDYDLNAVRLCFQVTIRDST-GRPLVLPPVLSHPIYDNRAPNTAELKICRVNRNSGSCLGGDEIFLL	195
_XP_043827224.1_	127	EQRGDYDLNAVRLCFQVTIRDPA-GRPLVLTPVLSHPIYDNRAPNTAELKICRVNRNSGSCLGGDEIFLL	195
_XP_027711838.1_	127	EQRGDYDLNAVRLCFQVTIRDPA-GRPLVLPPVLSHPIYDNRAPNTAELKICRVNRNSGSCLGGDEIFLL	195
_XP_020824046.1_	127	EQRGDYDLNAVRLCFQVTIRDPA-GRPLVLPPVLSHPIYDNRAPNTAELKICRVNRNSGSCLGGDEIFLL	195
_XP_036619952.1_	127	EQRGDYDLNAVRLCFQVTIRDSA-GRPLVLPPVLSHPIYDNRAPNTAELKICRVNRNSGSCLGGDEIFLL	195

```
_XP_031799278.1_  127  EQRGDYDLNAVRLCFQVTIRDAA-GRPLVLPPVLSHPIYDNRAPNTAELKICRVNRNSGSCLGGDEIFLL  195
_XP_007502748.1_  127  EQRGDYDLNAVRLCFQVTIQDSA-GRPLVLPPVLSHPIYDNRAPNTAELKICRVNRNSGSCLGGDEIFLL  195
_XP_051821546.1_  127  EQRGDYDLNAVRLCFQVTIRDAA-GRPLVLPPVLSHPIYDNRAPNTAELKICRVNRNSGSCLGGDEIFLL  195
_XP_051821547.1_  127  EQRGDYDLNAVRLCFQVTIRDAA-GRPLVLPPVLSHPIYDNRAPNTAELKICRVNRNSGSCLGGDEIFLL  195
_KAF6105894.1_     45  EQRGDYDLNAVRLCFQVTVRDPS-GRPLRLSPVLSHPIFDNRAPNTAELKICRVNRNSGSCLGGDEIFLL  113
_XP_008508783.1_   40  EQRGDYDLNAVRLCFQVTVRDPA-GRPLRLSPVLSHPIFDNRAPNTAELKICRVNRNSGSCLGGDEIFLL  108
_MBV98069.1_       62  EQRGDYDLNAVRLCFQVTVRDPA-GRPLRLSPVLSHPIFDNRAPNTAELKICRVNRNSGSCLGGDEIFLL  130
_XP_045411956.1_   86  EQRGDYDLNAVRLCFQVTVRDPS-GRPLRLPPVLSHPIFDNRAPNTAELKICRVNRNSGSCLGGDEIFLL  154
_XP_012520962.1_   86  EQRGDYDLNAVRLCFQVTVRDPS-GRPLRLPPVLSHPIFDNRAPNTAELKICRVNRNSGSCLGGDEIFLL  154
_XP_043307319.1_   86  EQRGDYDLNAVRLCFQVTVRDPA-GRPLRLVPVLSHPIFDNRAPNTAELKICRVNRNSGSCLGGDEIFLL  154
_XP_012666134.1_   86  EQRGDYDLNAVRLCFQVTVRDPS-GRPLRLPPVLSHPIFDNRAPNTAELKICRVNRNSGSCLGGDEIFLL  154
_XP_011367280.1_   86  EQRGDYDLNAVRLCFQVTVRDPS-GRPLRLSPVLSHPIFDNRAPNTAELKICRVNRNSGSCLGGDEIFLL  154
_KAF7238569.1_     36  ELKGDYDLNAVRLCFQVWIRSAGSGCLAALPLVISQPIYDNRAPNTAELKICRVNRNSGSCLGGDEIFLL  105
_KAG8146725.1_    127  ELKGDYDLNAVRLCFQVWIRDTGTGHLMQLPLVVSQPIYDNR----------------------------  168
_XP_048371352.1_  127  ELKGDYDLNAVRLCFQVWIRDAASGHLVQLPLSVSQPIYDNRAPNTAELKICRVNRNSGSCLGGDEIFLL  196
_XP_015272351.1_  127  ELKGDYDLNAVRLCFQVWIRDAASGHLVQLPLTVSQPIYDNRAPNTAELKICRVNRNSGSCLGGDEIFLL  196
_XP_054845680.1_  127  ELKGDYDLNAVRLCFQVWIRDTASGHLVQLPLTVSQPIYDNRAPNTAELKICRVNRNSGTCLGGDEIFLL  196
_XP_015272352.1_  127  ELKGDYDLNAVRLCFQVWIRDAASGHLVQLPLTVSQPIYDNRAPNTAELKICRVNRNSGSCLGGDEIFLL  196
_XP_042295897.1_  127  ELKGDYDLNAVRLCFQVWIRDPGSGRLVQLPLVISQPIYDNRAPNTAELKICRVNRNSGSCLGGDEIFLL  196
_XP_053133861.1_  127  ELKADYDLNAVRLCFQVWIRETGSGHLVQLPLVISQPIYDNRAPNTAELKICRVNRNSGSCLGGDEIFLL  196
_XP_053133862.1_  127  ELKADYDLNAVRLCFQVWIRETGSGHLVQLPLVISQPIYDNRAPNTAELKICRVNRNSGSCLGGDEIFLL  196
_KAJ6654174.1_    127  ELKGDYDLNAVRLCFQVWIRDTGSGHLVPLPLVTSQPIYDNRAPNTAELKICRVNRNSGSCLGGDEIFLL  196
_XP_044304454.1_  127  ELKGDYDLNAVRLCFQVWIRSAGSGCLAALPLVISQPIYDNRAPNTAELKICRVNRNSGSCLGGDEIFLL  196
_XP_020633015.1_  126  ELKGDYDLNAVRLCFQVWIRDAGSSRLVQLPLVVSQPIYDNRAPNTAELKICRVNRNSGSCLGGDEIFLL  195
_XP_028566864.1_  127  ELKGDYDLNAVRLCFQVWIQDAGSGRLVQLPLAISQPIYDNRAPNTAELKICRVNRNSGSCLGGDEIFLL  196
_XP_033030397.1_  127  ELKGDYDLNAVRLCFQVWIQDAGSGRLVQLPLAISQPIYDNRAPNTAELKICRVNRNSGSCLGGDEIFLL  196
_XP_044304455.1_  127  ELKGDYDLNAVRLCFQVWIRSAGSGCLAALPLVISQPIYDNRAPNTAELKICRVNRNSGSCLGGDEIFLL  196
_XP_053225758.1_  127  ELKGDYDLNAVRLCFQVWIQDAGSGRLVQLPLAISQPIYDNRAPNTAELKICRVNRNSGSCLGGDEIFLL  196
_XP_033030398.1_  127  ELKGDYDLNAVRLCFQVWIQDAGSGRLVQLPLAISQPIYDNRAPNTAELKICRVNRNSGSCLGGDEIFLL  196
_XP_034953722.1_  127  ELKGDYDLNAVRLCFQVWIQDAGSGRLVQLPLAISQPIYDNRAPNTAELKICRVNRNSGSCLGGDEIFLL  196
_XP_008123768.1_  127  ELKGDYDLNAVRLCFQVWIRDAGSGHLVQLPLVVSQPIYDNRAPNTAELKICRVNRNSGSCRGGDEIFLL  196
_KAJ7307406.1_    116  ELKGDYDLNAVRLCFQVWIRDAGSSRLVLLPLVVSQPIYDNRAPNTAELKICRVNRNSGSCLGGDEIFLL  185
_XP_013931335.1_  127  ALKGDYDLNAVRLCFQVWIRDTNTGHLIQLPLVFSQPIYDNRAPNTAELKICRVNRNSGTCLGGDEIFLL  196
_XP_032089926.1_  127  ALKGDYDLNAVRLCFQVWIRDTNTGHLIQLPLVFSQPIYDNRAPNTAELKICRVNRNSGTCLGGDEIFLL  196
_XP_026572203.1_  127  ELKGDYDLNAVRLCFQVWIRDTGTGHLMQLPLVVSQPIYDNRAPNTAELKICRVNRNSGTCLGGDEVFLL  196
_XP_026572204.1_  127  ELKGDYDLNAVRLCFQVWIRDTGTGHLMQLPLVVSQPIYDNRAPNTAELKICRVNRNSGTCLGGDEVFLL  196
_XP_034280315.1_  127  ELKGDYDLNAVRLCFQVWIRDTSTGHLIQLPLVVSQPIYDNRAPNTAELKICRVNRNSGTCLGGDEIFLL  196
_XP_039222066.1_  127  ELK-DYDLNAVRLCFQVWIRDTGTGHLIQLPLVVSQPIYDNRAPNTAELKICRVNRNSGTCVGGDEIFLL  195
_XP_015744179.1_  116  ELKGDYDLNAVRLCFQVWIRDTGTGHLIQLPLVVSQPIYDNRAPNTAELKICRVNRNSGTCLGGDEIFLL  185
```

```
_XP_029142061.1_   126  ELKGDYDLNAVRLCFQVWIRDTGTGHLIQLPLVVSQPIYDNRAPNTAELKICRVNRNSGTCLGGDEIFLL  195
_AAH41188.1_       127  ELKADYDLNTVCLCFQVFIPDQAAGRMLPLPFVVSQPIYDNRAPNTAELKICRVNKNSGSCLGGDEIFLL  196
_XP_018112002.1_   127  ELKADYDLNTVCLCFQVFIPDQAAGRMLPLPFVVSQPIYDNRAPNTAELKICRVNKNSGSCLGGDEIFLL  196
_NP_001081048.1_   127  ELKADYDLNTVCLCFQVFIPDQAAGRMLPLPFVVSQPIYDNRAPNTAELKICRVNKNSGSCLGGDEIFLL  196
_XP_031755832.1_   127  ELKSDYDLNTVCLCFQVFIPDRAAGQVVPLPFVVSQPIYDNRAPNTAELKICRVNRNSGSCMGGDEIFLL  196
_KAG8437862.1_     127  ELKTDFDLNTVCLCFQVFIHDLVSGRIIPLPFVVSQPIYDNRAPNTAELKICRVNRNSGSCLGGDEIFLL  196
_XP_053304358.1_   127  DLKSDFDLNTVCLCFQVFIHDQTGGHLVPLQFVVSQPIYDNRAPNTAELKICRVNKNSGSCMGGDEIFLL  196
_KAG9464550.1_     127  ELKSDYDLNTVCLCFQVYIGEQTS----PLPLVVSQPIYDNRAPNTAELKICRVNKNSGSCLGGDEIFLL  192
_XP_040265411.1_   127  DLKSDYDLNTVCLCFQVYIRDQS-GVLIPLQFVVSQPIYDNRAPNTAELKICRVNKNSGSCLGGDEIFLL  195
_XP_044125715.1_   127  DLKSDYDLNTVCLCFQVYIRDQS-GVLAPLQFVVSQPIYDNRAPNTAELKICRVNKNSGSCLGGDEIFLL  195
_XP_040265414.1_   127  DLKSDYDLNTVCLCFQVYIRDQS-GVLIPLQFVVSQPIYDNRAPNTAELKICRVNKNSGSCLGGDEIFLL  195
_XP_044125717.1_   127  DLKSDYDLNTVCLCFQVYIRDQS-GVLAPLQFVVSQPIYDNRAPNTAELKICRVNKNSGSCLGGDEIFLL  195
_XP_040184801.1_   127  DLKSDFDLNTVCLCFQVYIRDPSGG-LSPLTFVVSHPIYDNRAPNTAELKICRVNKNSGSCLGGDEIFLL  195
_ALA55869.1_       127  DLKSDFDLNTVCLCFQVYIRDPSGG-LSPLSFVVSHPIYDNRAPNTAELKICRVNKNSGSCLGGDEIFLL  195
_XP_018408357.1_   127  DLKSDFDLNTVCLCFQICIRDPTRG-LLPFPLVVSHPIYDNRAPNTAELKICRVNKNSGTCLGGDEIFLL  195
_XP_053575987.1_   127  DLKYEYDLNTVCLCFQVFIRDPSGNHFMPLPLMVSQPIYDNRAPNTAELKICRVNKNSGSCLGGDEIFLL  196
_XP_033812535.1_   127  ELKGEYDVNTVCLCFQVFIRDQSLGQFLPLPPVVSQAIYDNRAPNTAELKICRVNKNSGSCLGGDEIFLL  196
_XP_030074553.1_   127  ELKGEYDVNTVCLCFQVFIRDQSLGQFVPLPPVVSQPIYDNRAPNTAELKICRVNKNSGSCLGGDEIFLL  196
_XP_029468059.1_   127  ELKGDYDVNAVCLCFQIFIRDQSLGQFVPLPLVVSHPIYDNRAPNTAELKICRVNKNSGSCLGGDEIFLL  196
_XP_029468060.1_   127  ELKGDYDVNAVCLCFQIFIRDQSLGQFVPLPLVVSHPIYDNRAPNTAELKICRVNKNSGSCLGGDEIFLL  196
_KAJ1106220.1_     127  DLKGDYDLNAVRLCFQVFIQDPTSGQFMQLPMVVSQPIFDNRAPNTAELKICRVNKNSGSCQGGDEIFLL  196
_CAI5672275.1_     116  VWEEEFDLNSVRLCFQASITLST-GELYPLEPVVSQPIYDNRAPNTAELKICRVNRNSGSCKGGDEIFLL  184
_GCB80996.1_       110  -----------------------------------------NRAPNTAELKICRVNKNSGSCKGGEEIFLL  139
_XP_038618162.1_   127  EQRGDYDLNAVRLCFQVTVQDSA-DRPLALPPVLSQPIYDNRAPNTAELKICHVNRNSRSCLGGDEIFLL  195
Consensus_aa:           p..t-YDLsAVRLCFQVhlR-st.Gp.h.Ls.VlSpPI@DNRAPsTAEL+ICRVN+NSGoC.GGDEIFLL
Consensus_ss:           hh       eeeeeeeeeee      eee   ee               eeeeee          eeeee


Conservation:           99997759997 9    99 79 9779779999999979957    7  77 7 959597997759 557
_XP_028917328.1_   196  CDKVQKEDIEVCFSGQGWEARGSFSQADVHRQVAIVFRTPPYAEPALQAPVRVLMQLRRPSDRELSEPME  265
_XP_038621028.1_   196  CDKVQKEDIEVCFSGQGWEARGSFSQADVHRQVAIVFRTPPYAEPTLQAPVRVLMQLRRPSDRELSEPME  265
_RLV71517.1_       143  CDKVQKEDIEVRFSAEGWEAKGSFAQADVHRQVAIVFRTPPFRDPALRAPVTVRMELQRPSDRQRSAPVD  212
_XP_033367665.1_   153  CDKVQKEDIEVRFSAEGWEAKGSFAQADVHRQVAIVFRTPPFRDPALRAPVTVRMELQRPSDRQRSAPVD  222
_OWK50979.1_        19  --------------------------ADVHRQVAIVFRTPPFRDPALRAPVTVRMELQRPSDRQRSAPLD   62
_XP_031363427.1_   157  CDKVQKEDIEVRFSAEGWEAKGSFAQADVHRQVAIVFRTPPFRDPALRAPVTVRMELQRPSDRQRSAPLD  226
_XP_041319512.1_   103  CDKVQKEDIEVRFSAEGWEAKGSFAQADVHRQVAIVFRTPPFRDPALRAPVTVRMELQRPSDRQRSAPVD  172
_XP_054509575.1_   184  CDKVQKEDIEVRFWAEGWEAKGSFAQADVHRQVAIVFRTPPFRDPALRAPVTVRMELQRPSDRQRSAPVD  253
_XP_039909596.1_   184  CDKVQKEDIEVRFSAEGWEAKGSFAQADVHRQVAIVFRTPPFRDPALRAPVTVRMELQRPSDRQRSAPVD  253
_TRZ10035.1_       184  CDKVQKEDIEVRFSAEGWEAKGSFAQADVHRQVAIVFRTPPFRDPALRAPVTVRMELQRPSDRQRSAPVD  253
```

```
_XP_031950790.1_  184  CDKVQKEDIEVRFSAEGWEAKGSFAQADVHRQVAIVFRTPPFRDPALRAPVTVRMELQRPSDRQRSAPVD  253
_XP_050163562.1_  184  CDKVQKEDIEVRFWAEGWEAKGSFAQADVHRQVAIVFRTPPFRDPSLRQPVTVRMELQRPSDRQRSAPVD  253
_XP_039428642.1_  184  CDKVQKEDIEVRFSAEGWEAKGSFAQADVHRQVAIVFRTPPFRDPALRAPVTVRMELQRPSDRQRSAPVD  253
_XP_050840410.1_  199  CDKVQKEDIEVRFSAEGWEAKGSFAQADVHRQVAIVFRTPPFRDPALRAPVTVRMELQRPSDRQRSAPVD  268
_XP_054151317.1_  195  CDKVQKEDIEVRFWAEGWEAKGSFAQADVHRQVAIVFRTPPFRDPALRAPVTVRMELQRPSDRQRSAPVD  264
_XP_050840409.1_  195  CDKVQKEDIEVRFSAEGWEAKGSFAQADVHRQVAIVFRTPPFRDPALRAPVTVRMELQRPSDRQRSAPVD  264
_XP_030825825.1_  195  CDKVQKEDIEVRFWAEGWEAKGSFAQADVHRQVAIVFRTPPFRDPALRAPVTVRMELQRPSDRQRSAPVD  264
_XP_032939479.1_  195  CDKVQKEDIEVRFWAEGWEAKGSFAQADVHRQVAIVFRTPPFRDPALRAPVTVRMELQRPSDRQRSAPVD  264
_XP_041567964.1_  195  CDKVQKEDIEVRFSAEGWEAKGSFAQADVHRQVAIVFRTPPFRDPALRVPVTVRMELQRPSDRQRSAPLD  264
_XP_048149083.1_  195  CDKVQKEDIEVRFSAEGWEAKGSFAQADVHRQVAIVFRTPPFRDPALRAPVTVRMELQRPSDRQRSAPVD  264
_XP_054374241.1_  195  CDKVQKEDIEVRFWAEGWEAKGSFAQADVHRQVAIVFRTPPFRDPALRAPVTVRMELQRPSDRQRSAPVD  264
_NXV55901.1_      195  CDKVQKEDIEVRFWAEGWEAKGSFAQADVHRQVAIVFRTPPFRDPALRAPVTVRMELQRPSDRQRSAPVD  264
_XP_005533697.1_  195  CDKVQKEDIEVRFSAEGWEAKGSFAQADVHRQVAIVFRTPPFRDPALRAPVTVRMELQRPSDRQRSAPVD  264
_XP_014748521.1_  195  CDKVQKEDIEVRFWAEGWEAKGLFAQADVHRQVAIVFRTPPFRDPALRAPVTVRMELQRPSDRQRSAPVD  264
_RMB90491.1_      195  CDKVQKEDIEVRFSAEGWEAKGSFAQADVHRQVAIVFRTPPFRDPALRAPVTVRMELQRPSDRQRSAPVD  264
_XP_053857772.1_  195  CDKVQKEDIEVRFSAEGWEAKGSFAQADVHRQVAIVFRTPPFRDPALRAPVTVRMELQRPSDRQRSAPVD  264
_XP_029862760.1_   69  CDKVQKEDIEVRFSAEGWEAKGSFAQADVHRQVAIVFRTPPYREPALRQPVTVRMQLQRPSDRERSEPMD  138
_XP_039575719.1_  170  CDKVQKEDIEVRFSAEGWEAKGSFAQADVHRQVAIVFRTPPFRDPALRAPVTVRMELQRPSDRQRSAPVD  239
_XP_054664313.1_  195  CDKVQKEDIEVRFSAEGWEAKGAFAQADVHRQVAIVFRTPPYREPALRQPVTVHMQLQRPSDRERSEPMD  264
_XP_049691648.1_  195  CDKVQKEDIEVRFSAEGWEAKGSFAQADVHRQVAIVFRTPPYREPALRQPVTVRMQLQRPSDRERSEPMD  264
_XP_030331239.1_  196  CDKVQKEDIEVRFWAEGWEARGSFAQADVHRQVAIVFRTPAFLDPGLRQPRSVHMELQRPSDRQRSAAME  265
_XP_010726496.2_   67  CDKVQK----------------------------------------------------------------   72
_XP_042747731.1_   67  CDKVQK----------------------------------------------------------------   72
_XP_031466227.1_  195  CDKVQK----------------------------------------------------------------  200
_XP_032297702.1_  195  CDKVQKEDIEVRFWSDGWEAKGSFAAADVHRQVAIVFRTPPFRERSLLHPVTVRMELQRPSDRQRSPPLD  264
_RMB90505.1_      113  CDKVPE----------------------------------------------------------------  118
_KAI2561003.1_    185  C---------------------------------------------------------------------  185
_KAF6105893.1_    196  CDKVQKEDIELHFH--------------------------------------------------------  209
_PNJ38430.1_      196  CDKVQKEDIE------------------------------------------------------------  205
_PNI93749.1_      196  CDKVQKEDIE------------------------------------------------------------  205
_KAF6334124.1_    196  CDKVQKEDIEL-----------------------------------------------------------  206
_KAF6467432.1_    196  CDKVQKEDIELHFC--------------------------------------------------------  209
_PNI93759.1_      196  CDKVQK----------------------------------------------------------------  201
_PNJ38434.1_      196  CDKVQK----------------------------------------------------------------  201
_KAH0508019.1_    203  CDKVQKEDIEVYFTGPGWEARGSFSQADVHRQVAIVFRTPPYADPSLQAPVRVSMQLRRPSDRELSEPME  272
_AAA36408.1_      196  CDKVQKEDIEVYFTGPGWEARGSFSQADVHRQVAIVFRTPPYADPSLQAPVRVSMQLRRPSDRELSEPME  265
_XP_003274247.2_  196  CDKVQKEDIEVYFTGPGWEARGSFSQADVHRQVAIVFRTPPYADPSLQAPVRVSMQLRRPSDRELSEPME  265
_XP_007981896.1_  196  CDKVQKEDIEVYFTGPGWEARGSFSQADVHRQVAIVFRTPPYADPSLQAPVRVSMQLRRPSDRELSEPME  265
_XP_054296639.1_  196  CDKVQKEDIEVYFTGPGWEARGSFSQADVHRQVAIVFRTPPYADPSLQAPVRVSMQLRRPSDRELSEPME  265
```

_XP_023042293.1_	196	CDKVQKEDIEVYFTGPGWEARGSFSQADVHRQVAIVFRTPPYADPSLQAPVRVSMQLRRPSDRELSEPME	265
_XP_025211457.1_	196	CDKVQKEDIEVYFTGPGWEARGSFSQADVHRQVAIVFRTPPYADPSLQAPVRVSMQLRRPSDRELSEPME	265
_XP_039326898.1_	196	CDKVQKEDIEVYFTGPGWEARGSFSQADVHRQVAIVFRTPPYADPGLQAPVRVSMQLRRPSDRELSEPME	265
_XP_021781237.2_	196	CDKVQKEDIEVYFTGPGWEARGSFSQADVHRQVAIVFRTPPYADPSLQAPVRVSMQLRRPSDRELSEPME	265
_XP_010364772.1_	196	CDKVQKEDIEVYFTGPGWEARGSFSQADVHRQVAIVFRTPPYADPSLQAPVRVSMQLRRPSDRELSEPME	265
_XP_033063134.1_	196	CDKVQKEDIEVYFTGPGWEARGSFSQADVHRQVAIVFRTPPYADPSLQAPVRVSMQLRRPSDRELSEPME	265
_XP_017392927.1_	196	CDKVQKEDIEVYFTGPGWEARGSFSQADVHRQVAIVFRTPPYADPSLQAPVRVSMQLRRPSDRELSEPME	265
_XP_011897055.1_	196	CDKVQKEDIEVYFTGPGWEARGSFSQADVHRQVAIVFRTPPYADPSLQAPVRVSMQLRRPSDRELSEPME	265
_XP_032138725.1_	196	CDKVQKEDIEVYFTGPGWEARGSFSQADVHRQVAIVFRTPPYADPSLQAPVRVSMQLRRPSDRELSEPME	265
CAA80524.2	196	CDKVQKEDIEVYFTGPGWEARGSFSQADVHRQVAIVFRTPPYADPSLQAPVRVSMQLRRPSDRELSEPME	265
_XP_005577305.2_	196	CDKVQKEDIEVYFTGPGWEARGSFSQADVHRQVAIVFRTPPYADPSLQAPVRVSMQLRRPSDRELSEPME	265
BAE87977.1	196	CDKVQKEDIEVYFTGPGWEARGSFSQADVHRQVAIVFRTPPYADPSLQAPVRVSMQLRRPSDRELSEPME	265
_XP_023042294.1_	196	CDKVQKEDIEVYFTGPGWEARGSFSQADVHRQVAIVFRTPPYADPSLQAPVRVSMQLRRPSDRELSEPME	265
_XP_050612617.1_	196	CDKVQKEDIEVYFTGPGWEARGSFSQADVHRQVAIVFRTPPYADPSLQAPVRVSMQLRRPSDRELSEPME	265
EHH56082.1	196	CDKVQKEDIEVYFTGPGWEARGSFSQADVHRQVAIVFRTPPYADPSLQAPVRVSMQLRRPSDRELSEPME	265
_XP_028689064.1_	196	CDKVQKEDIEVYFTGPGWEARGSFSQADVHRQVAIVFRTPPYADPSLQAPVRVSMQLRRPSDRELSEPME	265
_XP_045411957.1_	196	CDKVQKEDIEVYFTGPGWEARGSFSQADVHRQVAIVFRTPPYADPSLQAPVRVSMQLRRPSDRELSEPME	265
_XP_012520963.1_	196	CDKVQKEDIEVYFTGPGWEARGSFSQADVHRQVAIVFRTPPYADPSLQAPVRVSMQLRRPSDRELSEPME	265
_XP_012633553.1_	196	CDKVQKEDIEVYFTGPGWEARGSFSQADVHRQVAIVFRTPPYADPSLQAPVRVSMQLRRPSDRELSEPME	265
_XP_053417694.1_	196	CDKVQKEDIEVYFTGPGWEARGSFSQADVHRQVAIVFRTPPYADPSLQAPVRVSMQLRRPSDRELSEPME	265
_XP_032976853.1_	196	CDKVQKEDIEVYFTGPGWEARGSFSQADVHRQVAIVFRTPPYADPSLQAPVRVSMQLRRPSDRELSEPME	265
_XP_008576943.1_	196	CDKVQKEDIEVYFTGPGWEARGSFSQADVHRQVAIVFRTPPYADPSLQAPVRVSMQLRRPSDRELSEPME	265
_XP_032345730.1_	196	CDKVQKEDIEVYFTGPGWEARGSFSQADVHRQVAIVFRTPPYADPSLQAPVRVSMQLRRPSDRELSEPME	265
_XP_010955442.1_	196	CDKVQKEDIEVYFTGPGWEARGSFSQADVHRQVAIVFRTPPYADPSLQAPVRVSMQLRRPSDRELSEPME	265
_XP_031303872.1_	196	CDKVQKEDIEVYFTGPGWEARGSFSQADVHRQVAIVFRTPPYADPSLQAPVRVSMQLRRPSDRELSEPME	265
_XP_032976854.1_	196	CDKVQKEDIEVYFTGPGWEARGSFSQADVHRQVAIVFRTPPYADPSLQAPVRVSMQLRRPSDRELSEPME	265
_XP_053780986.1_	196	CDKVQKEDIEVYFTGPGWEARGSFSQADVHRQVAIVFRTPPYADSSLQAPVRVSMHLRRPSDRELSEPME	265
_XP_036115166.1_	196	CDKVQKEDIEVYFTGPGWEARGSFSQADVHRQVAIVFRTPPYADPSLQAPVRVSMQLRRPSDRELSEPME	265
_XP_043307320.1_	196	CDKVQKEDIEVYFTGPGWEARGSFSQADVHRQVAIVFRTPPYADPSLQAPVRVSMQLRRPSDRELSEPME	265
EQB77721.1	196	CDKVQKEDIEVYFTGPGWEARGSFSQADVHRQVAIVFRTPPYADPSLQAPVRVSMQLRRPSDRELSEPME	265
_NP_001107753.1_	196	CDKVQKEDIEVYFTGPGWEARGSFSQADVHRQVAIVFRTPPYADPSLQAPVRVSMQLRRPSDRELSEPME	265
_XP_036785182.1_	196	CDKVQKEDIEVYFTGPGWEARGSFSQADVHRQVAIVFRTPPYADPGLQAPVRVSMQLRRPSDRELSEPME	265
_XP_020762118.1_	196	CDKVQKEDIEVYFTGPGWEARGSFSQADVHRQVAIVFRTPPYADPSLQAPVRVSMQLRRPSDRELSEPME	265
_XP_031546756.1_	196	CDKVQKEDIEVYFTGPGWEARGSFSQADVHRQVAIVFRTPPYADPSLQAPVRVSMQLRRPSDRELSEPME	265
_XP_023989099.1_	196	CDKVQKEDIEVYFTGPGWEARGSFSQADVHRQVAIVFRTPPYADSSLQAPVRVSMQLRRPSDRELSEPME	265
KAF4009900.1	196	CDKVQKEDIEVYFTGPGWEARGSFSQADVHRQVAIVFRTPPYADPSLQAPVRVSMQLRRPSDRELSEPME	265
KAB0376704.1	196	CDKVQKEDIEVYFTGPGWEARGSFSQADVHRQVAIVFRTPPYADPSLQAPVRVSMQLRRPSDRELSEPME	265
KAB0364713.1	196	CDKVQKEDIEVYFTGPGWEARGSFSQADVHRQVAIVFRTPPYADPSLQAPVRVSMQLRRPSDRELSEPME	265
CBT01657.1	196	CDKVQKEDIEVYFTGPGWEARGSFSQADVHRQVAIVFRTPPYADPSLQAPVRVSMQLRRPSDRELSEPME	265

_XP_036851744.1_	196	CDKVQKEDIEVYFTGPGWEARGSFSQADVHRQVAIVFRTPPYADPGLQAPVRVSMQLRRPSDRELSEPME	265
_XP_007128247.1_	196	CDKVQKEDIEVYFTGPGWEARGSFSQADVHRQVAIVFRTPPYADSSLQAPVRVSMQLRRPSDRELSEPME	265
_XP_003798713.1_	196	CDKVQKEDIEVYFTGPGWEARGSFSQADVHRQVAIVFRTPPYVDPSLQAPVRVSMQLRRPSDRELSEPME	265
_NP_001073711.1_	196	CDKVQKEDIEVYFTGPGWEARGSFSQADVHRQVAIVFRTPPYADPGLQAPVRVSMQLRRPSDRELSEPME	265
_XP_006056522.1_	196	CDKVQKEDIEVYFTGPGWEARGSFSQADVHRQVAIVFRTPPYADPGLQAPVRVSMQLRRPSDRELSEPME	265
_XP_019593326.1_	196	CDKVQKEDIEVYFTGPGWEARGSFSQADVHRQVAIVFRTPPYADPSLQAPVRVSMQLRRPSDRELSEPME	265
_XP_005894159.1_	196	CDKVQKEDIEVYFTGPGWEARGSFSQADVHRQVAIVFRTPPYADPGLQAPVRVSMQLRRPSDRELSEPME	265
ELR57541.1	196	CDKVQKEDIEVYFTGPGWEARGSFSQADVHRQVAIVFRTPPYADPGLQAPVRVSMQLRRPSDRELSEPME	265
_XP_010836092.1_	196	CDKVQKEDIEVYFTGPGWEARGSFSQADVHRQVAIVFRTPPYADPGLQAPVRVSMQLRRPSDRELSEPME	265
_XP_051049621.1_	196	CDKVQKEDIEVYFTGPGWEARGSFSQADVHRQVAIVFRTPPYADPSLQAPVRVSMQLRRPSDRELSEPME	265
CBT01658.1	196	CDKVQEEDIEVYFTGPGWEARGSFSQADVHRQVAIVFRTPPYSDPSLQAPVRVSMQLRRPSDRELSEPME	265
_XP_015974930.1_	196	CDKVQKEDIEVYFTGPGWEARGSFSQADVHRQVAIVFRTPPYADPSLQAPVRVSMQLRRPSDRELSEPME	265
_XP_027264386.1_	196	CDKVQKEDIEVYFTGPGWEARGSFSQADVHRQVAIVFRTPPYADPSLQAPVRVSMQLRRPSDRELSEPME	265
_XP_040112158.1_	196	CDKVQKEDIEVYFTGPGWEARGSFSQADVHRQVAIVFRTPPYADPSLQAPVRVSMQLRRPSDRELSEPME	265
_XP_052517633.1_	196	CDKVQKEDIEVYFTGPGWEARGSFSQADVHRQVAIVFRTPPYADPSLQAPVRVSMQLRRPSDRELSEPME	265
_XP_017898873.1_	196	CDKVQKEDIEVYFTGPGWEARGSFSQADVHRQVAIVFRTPPYADPSLQAPVRVSMQLRRPSDRELSEPME	265
_XP_020020484.1_	196	CDKVQKEDIEVYFTGPGWEARGSFSQADVHRQVAIVFRTPPYADPGLQAPVRVSMQLRRPSDRELSEPME	265
_XP_047631223.1_	196	CDKVQKEDIEVYFTGPGWEARGSFSQADVHRQVAIVFRTPPYADPSLQAPVRVSMQLRRPSDRELSEPME	265
_XP_007651509.2_	196	CDKVQKEDIEVYFTGPGWEARGSFSQADVHRQVAIVFRTPPYADPSLQAPVRVSMQLRRPSDRELSEPME	265
_XP_027815096.1_	196	CDKVQKEDIEVYFTGPGWEARGSFSQADVHRQVAIVFRTPPYADPSLQAPVRVSMQLRRPSDRELSEPME	265
_XP_034377707.1_	196	CDKVQKEDIEVYFTGPGWEARGSFSQADVHRQVAIVFRTPPYADPSLQAPVRVSMQLRRPSDRELSEPME	265
ERE77884.1	196	CDKVQKEDIEVYFTGPGWEARGSFSQADVHRQVAIVFRTPPYADPSLQAPVRVSMQLRRPSDRELSEPME	265
_XP_028626823.1_	196	CDKVQKEDIEVYFTGPGWEARGSFSQADVHRQVAIVFRTPPYADPSLQAPVRVSMQLRRPSDRELSEPME	265
KAF5928161.1	196	CDKVQKEDIEVYFTGPGWEARGSFSQADVHRQVAIVFRTPPYADPSLQAPVRVSMQLRRPSDRELSEPME	265
KAI5933031.1	196	CDKVQKEDIEVYFTGPGWEARGSFSQADVHRQVAIVFRTPPYADPGLQAPVRVSMQLRRPSDRELSEPME	265
_XP_021007720.1_	196	CDKVQKEDIEVYFTGPGWEARGSFSQADVHRQVAIVFRTPPYADPSLQAPVRVSMQLRRPSDRELSEPME	265
_XP_026252329.1_	196	CDKVQKEDIEVYFTGPGWEARGSFSQADVHRQVAIVFRTPPYADPSLQAPVRVSMQLRRPSDRELSEPME	265
_XP_054426842.1_	196	CDKVQKEDIEVYFTGPGWEARGSFSQADVHRQVAIVFRTPPYADPSLQAPVRVSMHLRRPSDRELSEPME	265
_XP_032747030.1_	196	CDKVQKEDIEVYFTGPGWEARGSFSQADVHRQVAIVFRTPPYADPSLQAPVRVSMQLRRPSDRELSEPME	265
_XP_049748463.1_	196	CDKVQKEDIEVCFTGPGWEARGSFSQADVHRQVAIVFRTPPYADPSLQAPVRVSMQLRRPSDRELSEPME	265
KAJ1065952.1	196	CDKVQKEDIEVYFTGPGWEARGSFSQADVHRQVAIVFRTPPYADPSLQAPVRVSMQLRRPSDRELSEPME	265
_XP_052048317.1_	196	CDKVQKEDIEVYFTGPGWEARGSFSQADVHRQVAIVFRTPPYADPGLQAPVRVSMQLRRPSDRELSEPME	265
_XP_012876614.1_	196	CDKVQKEDIEVYFTGPGWEARGSFSQADVHRQVAIVFRTPPYADPSLQAPVRVSMQLRRPSDRELSEPME	265
_XP_020020482.1_	196	CDKVQKEDIEVYFTGPGWEARGSFSQADVHRQVAIVFRTPPYADPGLQAPVRVSMQLRRPSDRELSEPME	265
_XP_010597340.1_	196	CDKVQKEDIEVCFTGPGWEARGSFSQADVHRQVAIVFRTPPYADPSLQAPVRVSMQLRRPSDRELSEPME	265
_XP_026339000.2_	196	CDKVQKEDIEVYFTGPGWEARGSFSQADVHRQVAIVFRTPPYADPSLQAPVRVSMQLRRPSDRELSEPME	265
_XP_030689468.1_	196	CDKVQKEDIEVYFTGPGWEARGSFSQADVHRQVAIVFRTPPYADPSLQAPVRVSMQLRRPSDRELSEPME	265
_XP_049993276.1_	196	CDKVQKEDIEVYFTGPGWEARGSFSQADVHRQVAIVFRTPPYADPSLQAPVRVSMQLRRPSDRELSEPME	265
_XP_014700317.1_	196	CDKVQKEDIEVYFTGPGWEARGSFSQADVHRQVAIVFRTPPYADPSLQAPVRVSMQLRRPSDRELSEPME	265

_XP_021494126.1_	196	CDKVQKEDIEVYFTGPGWEARGSFSQADVHRQVAIVFRTPPYADPSLQAPVRVSMQLRRPSDRELSEPME	265
_XP_022447167.1_	196	CDKVQKEDIEVYFTGPGWEARGSFSQADVHRQVAIVFRTPPYADPSLQAPVRVSMQLRRPSDRELSEPME	265
_XP_042532137.1_	196	CDKVQKEDIEVYFTGPGWEARGSFSQADVHRQVAIVFRTPPYADPSLQAPVRVSMQLRRPSDRELSEPME	265
_XP_008820421.1_	196	CDKVQKEDIEVYFTGPGWEARGSFSQADVHRQVAIVFRTPPYADPSLQAPVRVSMQLRRPSDRELSEPME	265
_XP_023415352.1_	196	CDKVQKEDIEVCFTGPGWEARGSFSQADVHRQVAIVFRTPPYADPSLQAPVRVSMQLRRPSDRELSEPME	265
_XP_029414056.1_	196	CDKVQKEDIEVYFTGPGWEARGSFSQADVHRQVAIVFRTPPYADPSLQAPVRVSMQLRRPSDRELSEPME	265
_XP_024604853.1_	196	CDKVQKEDIEVYFTGPGWEARGSFSQADVHRQVAIVFRTPPYADPSLQAPVRVSMQLRRPSDRELSEPME	265
_XP_007462052.1_	196	CDKVQKEDIEVYFTGPGWEARGSFSQADVHRQVAIVFRTPPYADPSLQAPVRVSMQLRRPSDRELSEPME	265
_XP_006101621.1_	196	CDKVQKEDIEVYFTGPGWEARGSFSQADVHRQVAIVFRTPPYADPSLQAPVRVSMQLRRPSDRELSEPME	265
_XP_005064049.1_	196	CDKVQKEDIEVYFTGPGWEARGSFSQADVHRQVAIVFRTPPYADPSLQAPVRVSMQLRRPSDRELSEPME	265
_XP_031245193.1_	196	CDKVQKEDIEVYFTGPGWEARGSFSQADVHRQVAIVFRTPPYADPSLQAPVRVSMQLRRPSDRELSEPME	265
ELK24332.1	196	CDKVQKEDIEVYFTGPGWEARGSFSQADVHRQVAIVFRTPPYADPSLQAPVRVSMQLRRPSDRELSEPME	265
_XP_041518057.1_	196	CDKVQKEDIEVYFTGPGWEARGSFSQADVHRQVAIVFRTPPYADPSLQAPVRVSMQLRRPSDRELSEPME	265
_XP_005351804.2_	196	CDKVQKEDIEVYFTGPGWEARGSFSQADVHRQVAIVFRTPPYADPSLQAPVRVSMQLRRPSDRELSEPME	265
EGW13067.1	196	CDKVQKEDIEVYFTGPGWEARGSFSQADVHRQVAIVFRTPPYADPSLQAPVRVSMQLRRPSDRELSEPME	265
ERE77883.1	196	CDKVQKEDIEVYFTGPGWEARGSFSQADVHRQVAIVFRTPPYADPSLQAPVRVSMQLRRPSDRELSEPME	265
KFO28401.1	196	CDKVQKEDIEVYFTGPGWEARGSFSQADVHRQVAIVFRTPPYADPSLQAPVRVSMQLRRPSDRELSEPME	265
_XP_010634478.1_	196	CDKVQKEDIEVYFTGPGWEARGSFSQADVHRQVAIVFRTPPYADPSLQAPVRVSMQLRRPSDRELSEPME	265
OBS81518.1	196	CDKVQKEDIEVYFTGPGWEARGSFSQADVHRQVAIVFRTPPYADPSLQAPVRVSMQLRRPSDRELSEPME	265
_XP_007462051.1_	196	CDKVQKEDIEVYFTGPGWEARGSFSQADVHRQVAIVFRTPPYADPSLQAPVRVSMQLRRPSDRELSEPME	265
_XP_014393535.1_	196	CDKVQKEDIEVYFTGPGWEARGSFSQADVHRQVAIVFRTPPYADPSLQAPVRVSMQLRRPSDRELSEPME	265
_XP_048216512.1_	196	CDKVQKEDIEVYFTGPGWEARGSFSQADVHRQVAIVFRTPPYADPGLQAPVRVSMQLRRPSDRELSEPME	265
_XP_015424455.1_	196	CDKVQKEDIEVYFTGPGWEARGSFSQADVHRQVAIVFRTPPYADPSLQAPVRVSMQLRRPSDRELSEPME	265
_XP_019501147.1_	196	CDKVQKEDIEVYFTGPGWEARGSFSQADVHRQVAIVFRTPPYADPRLQAPVRVSMQLRRPSDRELSEPME	265
_XP_004656738.2_	196	CDKVQKEDIEVYFTGPGWEARGSFSQADVHRQVAIVFRTPPYADPSLQAPVRVSMQLRRPSDRELSEPME	265
_XP_036909111.1_	196	CDKVQKEDIEVYFTGPGWEARGSFSQADVHRQVAIVFRTPPYADPSLQAPVRVSMHLRRPSDRELSEPME	265
_XP_036182518.1_	196	CDKVQKEDIEVYFTGPGWEARGSFSQADVHRQVAIVFRTPPYADPSLQAPVRVSMQLRRPSDRELSEPME	265
_XP_012580591.1_	196	CDKVQKEDIEVYFTGPGWEARGFFSQADVHRQVAIVFRTPPYMNPRLQAPVRVSMQLRRPSDRELSEPME	265
_XP_016063765.1_	196	CDKVQKEDIEVYFTGPGWEARGSFSQADVHRQVAIVFRTPPYADPSLQAPVRVSMQLRRPSDRELSEPME	265
_XP_021047054.1_	196	CDKVQKEDIEVYFTGPGWEARGSFSQADVHRQVAIVFRTPPYADPSLQAPVRVSMQLRRPSDRELSEPME	265
_XP_023095836.1_	196	CDKVQKEDIEVYFTGPGWEARGSFSQADVHRQVAIVFRTPPYADPSLQAPVRVSMQLRRPSDRELSEPME	265
_XP_045342734.1_	196	CDKVQKEDIEVYFTGPGWEARGSFSQADVHRQVAIVFRTPPYADPSLQAPVRVSMQLRRPSDRELSEPME	265
_XP_053755429.1_	196	CDKVQKEDIEVYFTGPGWEARGSFSQADVHRQVAIVFRTPPYADPSLQAPVRVSMQLRRPSDRELSEPME	265
_XP_022447166.1_	196	CDKVQKEDIEVYFTGPGWEARGSFSQADVHRQVAIVFRTPPYADPSLQAPVRVSMQLRRPSDRELSEPME	265
_XP_004713741.1_	196	CDKVQKEDIEVCFTGPGWEARGAFSQADVHRQVAIVFRTPPYADPSLQAPVRVSMQLRRPSDRELSEPME	265
_NP_954888.1_	196	CDKVQKEDIEVYFTGPGWEARGSFSQADVHRQVAIVFRTPPYADPSLQAPVRVSMQLRRPSDRELSEPME	265
_XP_037002389.2_	196	CDKVQKEDIEVYFTGPGWEARGSFSQADVHRQVAIVFRTPPYADPSLQVPVRVSMHLRRPSDRELSEPME	265
_XP_039100808.1_	196	CDKVQKEDIEVYFTGPGWEARGSFSQADVHRQVAIVFRTPPYADPSLQAPVRVSMQLRRPSDRELSEPME	265
_XP_038170688.1_	196	CDKVQKEDIEVYFTGPGWEARGSFSQADVHRQVAIVFRTPPYADPSLQAPVRVSMQLRRPSDRELSEPME	265

```
_XP_040495345.1_  196  CDKVQKEDIEVYFTGPGWEARGSFSQADVHRQVAIVFRTPPYADPSLQAPVRVSMQLRRPSDRELSEPME  265
_XP_052568261.1_  196  CDKVQKEDIEVYFTGPGWEARGSFSQADVHRQVAIVFRTPPYADPSLQAPVRVSMQLRRPSDRELSEPME  265
_XP_042760134.1_  196  CDKVQKEDIEVYFTGPGWEARGSFSQADVHRQVAIVFRTPPYADPSLQAPVRVSMQLRRPSDRELSEPME  265
_XP_049472392.1_  196  CDKVQKEDIEVYFTGPGWEARGSFSQADVHRQVAIVFRTPPYADPSLQAPVRVSMQLRRPSDRELSEPME  265
_XP_023593974.1_  196  CDKVQKEDIEVCFTGPGWEARGSFSQADVHRQVAIVFRTPPYADPSLQAPVRVSMQLRRPSDRELSEPME  265
_XP_034501886.1_  196  CDKVQKEDIEVYFTGPGWEARGSFSQADVHRQVAIVFRTPPYADPSLQAPVRVSMQLRRPSDRELSEPME  265
_XP_004393991.1_  196  CDKVQKEDIEVYFTGPGWEARGSFSQADVHRQVAIVFRTPPYADPSLQAPVRVSMQLRRPSDRELSEPME  265
_XP_025860214.1_  196  CDKVQKEDIEVYFTGPGWEARGSFSQADVHRQVAIVFRTPPYADPSLQAPVRVSMQLRRPSDRELSEPME  265
_XP_019501146.1_  196  CDKVQKEDIEVYFTGPGWEARGSFSQADVHRQVAIVFRTPPYADPRLQAPVRVSMQLRRPSDRELSEPME  265
_XP_025734327.1_  196  CDKVQKEDIEVYFTGPGWEARGSFSQADVHRQVAIVFRTPPYADPSLQAPVRVSMQLRRPSDRELSEPME  265
_XP_025301454.1_  196  CDKVQKEDIEVYFTGPGWEARGSFSQADVHRQVAIVFRTPPYADPSLQAPVRVTMQLRRPSDRELSEPME  265
_KAG3284687.1_    196  CDKVQKEDIEVYFTGPGWEARGSFSQADVHRQVAIVFRTPPYADPSLQAPVRVSMQLRRPSDRELSEPME  265
_VFV23136.1_      196  CDKVQKEDIEVYFTGPGWEARGSFSQADVHRQVAIVFRTPPYADPSLQAPVRVSMQLRRPSDRELSEPME  265
_XP_025771173.1_  196  CDKVQKEDIEVYFTGPGWEARGSFSQADVHRQVAIVFRTPPYADPSLQAPVRVSMQLRRPSDRELSEPME  265
_XP_054580928.1_  196  CDKVQKEDIEVYFTGPGWEARGSFSQADVHRQVAIVFRTPPYADPTLQAPVRVSMQLRRPSDRELSEPME  265
_EPQ06533.1_      196  CDKVQKEDIEVYFTGPGWEARGSFSQADVHRQVAIVFRTPPYADPSLQAPVRVSMQLRRPSDRELSEPME  265
_XP_034869141.1_  196  CDKVQKEDIEVYFTGPGWEARGSFSQADVHRQVAIVFRTPPYADPSLQAPVRVSMQLRRPSDRELSEPME  265
_XP_042813589.1_  196  CDKVQKEDIEVYFTGPGWEARGSFSQADVHRQVAIVFRTPPYADPSLQAPVRVSMQLRRPSDRELSEPME  265
_XP_040322176.1_  196  CDKVQKEDIEVYFTGPGWEARGSFSQADVHRQVAIVFRTPPYADPSLQAPVRVSMQLRRPSDRELSEPME  265
_XP_006861137.1_  196  CDKVQKEDIEVCFTGPGWEARGSFSQADVHRQVAIVFRTPPYADPSLQAPVRVSMQLRRPSDRELSEPME  265
_XP_007943155.2_  196  CDKVQKEDIEVCFTGPGWEARGSFSQADVHRQVAIVFRTPPYADPSLQAPVRVSMQLRRPSDRELSEPME  265
_BAQ95593.1_      196  CDKVQKEDIEVYFTGPGWEARGSFSQADVHRQVAIVFRTPPYADPSLQAPVRVTMQLRRLSDRELSEPME  265
_XP_019652454.2_  196  CDKVQKEDIEVYFTGPGWEARGSFSQADVHRQVAIVFRTPPYADPSLQAPVRVSMQLRRPSDRELSEPME  265
_XP_016041658.1_  196  CDKVQKEDIEVYFTGPGWEARGSFSQADVHRQVAIVFRTPPYADPSLQAPVRVSMQLRRPSDRELSEPME  265
_XP_047546997.1_  196  CDKVQKEDIEVYFTGPGWEARGSFSQADVHRQVAIVFRTPPYADPSLQAPVRVSMQLRRPSDRELSEPME  265
_XP_042115231.1_  196  CDKVQKEDIEVYFTGPGWEARGSFSQADVHRQVAIVFRTPPYADPSLQAPVRVSMQLRRPSDRELSEPME  265
_XP_042115227.1_  196  CDKVQKEDIEVYFTGPGWEARGSFSQADVHRQVAIVFRTPPYADPSLQAPVRVSMQLRRPSDRELSEPME  265
_XP_015854576.1_  196  CDKVQKEDIEVYFTGPGWEARGSFSQADVHRQVAIVFRTPPYADPSLQAPVRVSMQLRRPSDRELSEPME  265
_XP_025301452.1_  196  CDKVQKEDIEVYFTGPGWEARGSFSQADVHRQVAIVFRTPPYADPSLQAPVRVTMQLRRPSDRELSEPME  265
_XP_044920951.1_  196  CDKVQKEDIEVYFTGPGWEARGSFSQADVHRQVAIVFRTPPYADPSLQAPVRVSMQLRRPSDRELSEPME  265
_KAF3822254.1_    196  CDKVQKEDIEVYFTGPGWEARGSFSQADVHRQVAIVFRTPPYADPSLQAPVRVSMQLRRPSDRELSEPME  265
_XP_021540718.1_  196  CDKVQKEDIEVYFTGPGWEARGSFSQADVHRQVAIVFRTPPYADPSLQAPVRVSMQLRRPSDRELSEPME  265
_XP_022362935.1_  196  CDKVQKEDIEVYFTGPGWEARGSFSQADVHRQVAIVFRTPPYADPSLQAPVRVSMQLRRPSDRELSEPME  265
_XP_044115623.1_  196  CDKVQKEDIEVYFTGPGWEARGSFSQADVHRQVAIVFRTPPYADPSLQAPVRVSMQLRRPSDRELSEPME  265
_XP_032735359.1_  196  CDKVQKEDIEVYFTGPGWEARGSFSQADVHRQVAIVFRTPPYADPSLQAPVRVSMQLRRPSDRELSEPME  265
_XP_032270304.1_  196  CDKVQKEDIEVYFTGPGWEARGSFSQADVHRQVAIVFRTPPYADPSLQAPVRVSMQLRRPSDRELSEPME  265
_XP_032213216.1_  196  CDKVQKEDIEVYFTGPGWEARGSFSQADVHRQVAIVFRTPPYADPSLQAPVRVSMQLRRPSDRELSEPME  265
_XP_004478661.1_  196  CDKVQKEDIEVCFTGPGWEARGSFSQADVHRQVAIVFRTPPYADPSLQAPVRVSMQLRRPSDRELSEPME  265
_XP_035933519.1_  196  CDKVQKEDIEVYFTGPGWEARGSFSQADVHRQVAIVFRTPPYADPSLQAPVRVSMQLRRPSDRELSEPME  265
```

KAI5761432.1	196	CDKVQKEDIEVYFTGPGWEARGSFSQADVHRQVAIVFRTPPYADPSLQAPVRVSMQLRRPSDRELSEPME	265
_XP_029813244.1_	196	CDKVQKEDIEVYFTGPGWEARGSFSQADVHRQVAIVFRTPPYADPSLQAPVRVSMQLRRPSDRELSEPME	265
_XP_027800377.1_	196	CDKVQKEDIEVYFTGPGWEARGSFSQADVHRQVAIVFRTPPYADPSLQAPVRVSMQLRRPSDRELSEPME	265
_XP_004852440.1_	196	CDKVQKEDIEVYFTGPGWEARGSFSQADVHRQVAIVFRTPPYADSSLQAPVRVSMQLRRPSDRELSEPME	265
_XP_045873061.1_	196	CDKVQKEDIEVYFTGPGWEARGSFSQADVHRQVAIVFRTPPYADPSLQAPVRVSMQLRRPSDRELSEPME	265
_XP_030891354.1_	196	CDKVQKEDIEVYFTGPGWEARGSFSQADVHRQVAIVFRTPPYADPSLQAPVRVSMQLRRPSDRELSEPME	265
CAD7670066.1	196	CDKVQKEDIEVYFTGPGWEARGSFSQADVHRQVAIVFRTPPYADPSLQAPVRVSMQLRRPSDRELSEPME	265
_XP_046301192.1_	196	CDKVQKEDIEVYFTGPGWEARGSFSQADVHRQVAIVFRTPPYADPSLQAPVRVSMQLRRPSDRELSEPME	265
_XP_036059776.1_	196	CDKVQKEDIEVYFTGPGWEARGSFSQADVHRQVAIVFRTPPYADPSLQAPVRVSMQLRRPSDRELSEPME	265
_XP_028711748.1_	196	CDKVQKEDIEVYFTGPGWEARGSFSQADVHRQVAIVFRTPPYADPSLQAPVRVSMQLRRPSDRELSEPME	265
_XP_037696258.1_	196	CDKVQKEDIEVCFTGPGWEARGSFSQADVHRQVAIVFRTPPYADPGLQAPVRVSMQLRRPSDRELSEPME	265
_XP_004627287.1_	196	CDKVQKEDIEVYFTGPGWEARGSFSQADVHRQVAIVFRTPPYADPSLQAPVRVSMQLRRPSDRELSEPME	265
_XP_037371589.1_	196	CDKVQKEDIEVYFTGPGWEARGSFSQADVHRQVAIVFRTPPYMTRSLQAPVRVSMQLRRPSDRELSEPME	265
_XP_003468122.1_	196	CDKVQKEDIEVYFTGPGWEARGSFSQADVHRQVAIVFRTPPYADPSLQAPVRVSMQLRRPSDRELSEPME	265
_XP_040826731.1_	196	CDKVQKEDIEVYFTGPGWEARGSFSQADVHRQVAIVFRTPPYADPSLQAPVRVSMQLRRPSDREVSEPME	265
_XP_005384525.1_	196	CDKVQKEDIEVYFTGPGWEARGSFSQADVHRQVAIVFRTPPYADPSLQAPVRVSMQLRRPSDRELSEPME	265
_XP_045711286.1_	196	CDKVQKEDIEVYFTGPGWEARGSFSQADVHRQVAIVFRTPPYADPGLQAPVRVSMHLRRPSDRELSEPME	265
_XP_044920955.1_	196	CDKVQKEDIEVYFTGPGWEARGSFSQADVHRQVAIVFRTPPYADPSLQAPVRVSMQLRRPSDRELSEPME	265
_XP_047375326.1_	196	CDKVQKEDIEVYFTGPGWEARGSFSQADVHRQVAIVFRTPPYADPSLQAPVRVSMQLRRPSDRELSEPME	265
_XP_036272436.1_	196	CDKVQKEDIEVYFTGPGWEARGSFSQADVHRQVAIVFRTPPYADPSLQAPVRVSMQLRRPSDRELSEPME	265
ELK13339.1	196	CDKVQKEDIEVYFTGPGWEARGSFSQADVHRQVAIVFRTPPYADPSLQAPVRVSMQLRRPSDRELSEPME	265
_XP_021564615.1_	196	CDKVQKEDIEVYFTGPGWEARGSFSQADVHRQVAIVFRTPPYADPSLQAPVRVSMQLRRPSDRELSEPME	265
_XP_024902067.1_	196	CDKVQKEDIEVYFTGPGWEARGSFSQADVHRQVAIVFRTPPYADPSLQAPVRVSMQLRRPSDRELSEPME	265
_XP_036349005.1_	196	CDKVQKEDIEVYFTGPGWEARGSFSQADVHRQVAIVFRTPPYADPSLQAPVRVSMQLRRPSDREVSEPME	265
_XP_051683930.1_	196	CDKVQKEDIEVYFTGPGWEARGSFSQADVHRQVAIVFRTPPYADPGLQAPVRVSMQLRRPSDRELSEPME	265
_XP_013006227.1_	196	CDKVQKEDIEVYFTGPGWEARGSFSQADVHRQVAIVFRTPPYADPSLQAPVRVSMQLRRPSDRELSEPME	265
_XP_006893486.1_	195	CDKVQKEDIEVCFTGPGWEARGSFSQADVHRQVAIVFRTPPYADPGLQAPVRVSMQLRRPSDRELSEPME	264
_XP_036272437.1_	196	CDKVQKEDIEVYFTGPGWEARGSFSQADVHRQVAIVFRTPPYADPSLQAPVRVSMQLRRPSDRELSEPME	265
ELW65121.1	196	CDKVQKEDIEVYFTGPGWEARGSFSQADVHRQVAIVFRTPPYADPSLQAPVRVSMQLRRPSDRELSEPME	265
EHB04873.1	196	CDKVQKEDIEVYFTGPGWEARGSFSQADVHRQVAIVFRTPPYADSSLQAPVRVSMQLRRPSDRELSEPME	265
KAB0407447.1	196	CDKVQKEDIEVYFTGPGWEARGSFSQADVHRQVAIVFRTPPYADPGLQAPVRVSMQLRRPSDRELSEPME	265
KAF6105901.1	196	CDKVQKEDIEVYFTGPGWEARGSFSQADVHRQVAIVFRTPPYADPGLQAPVRVSMHLRRPSDRELSEPME	265
MXQ90627.1	196	CDKVQKEDIEVYFTGPGWEARGSFSQADVHRQVAIVFRTPPYADPGLQAPVRVSMQLRRPSDRELSEPME	265
PNJ38432.1	196	CDKVQKEDIEVYFTGPGWEARGSFSQADVHRQVAIVFRTPPYADPSLQAPVRVSMQLRRPSDRELSEPME	265
PNI93760.1	196	CDKVQKEDIEVYFTGPGWEARGSFSQADVHRQVAIVFRTPPYADPSLQAPVRVSMQLRRPSDRELSEPME	265
KAF6438864.1	196	CDKVQKEDIEVYFTGPGWEARGSFSQADVHRQVAIVFRTPPYADPSLQAPVRVSMQLRRPSDRELSEPME	265
KAF6281766.1	196	CDKVQKEDIEVYFTGPGWEARGSFSQADVHRQVAIVFRTPPYADPSLQAPVRVSMQLRRPSDRELSEPME	265
_XP_035119201.1_	196	CDKVQKEDIEVYFTGPGWEARGSFSQADVHRQVAIVFRTPPYADPSLQAPVRVFMQLRRPSDRELSEPME	265
KAF6334125.1	196	CDKVQKEDIEVYFTGPGWEARGSFSQADVHRQVAIVFRTPPYADPSLQAPVRVSMQLRRPSDRELSEPME	265

```
_XP_036785207.1_  196  CDKVQKEDIEVYFTGPGWEARGSFSQADVHRQVAIVFRTPPYADPGLQAPVRVSMQLRRPSDRELSEPME  265
_XP_036851747.1_  196  CDKVQKEDIEVYFTGPGWEARGSFSQADVHRQVAIVFRTPPYADPGLQAPVRVSMQLRRPSDRELSEPME  265
_XP_054367814.1_  196  CDKVQKEDIEVYFTGPGWEARGSFSQADVHRQVAIVFRTPPYADPSLQAPVRVSMQLRRPSDRELSEPME  265
_XP_030689470.1_  196  CDKVQKEDIEVYFTGPGWEARGSFSQADVHRQVAIVFRTPPYADPSLQAPVRVSMQLRRPSDRELSEPME  265
_XP_026946120.1_  196  CDKVQKEDIEVYFTGPGWEARGSFSQADVHRQVAIVFRTPPYADPSLQAPVRVSMQLRRPSDRELSEPME  265
_XP_039696841.1_  196  CDKVQKEDIEVYFTGPGWEARGSFSQADVHRQVAIVFRTPPYADPSLQAPVRVSMQLRRPSDRELSEPME  265
_1IKN_A_          196  CDKVQKEDIEVYFTGPGWEARGSFSQADVHRQVAIVFRTPPYADPSLQAPVRVSMQLRRPSDRELSEPME  265
_XP_016775352.1_  167  ------QDIEVYFTGPGWEARGSFSQADVHRQVAIVFRTPPYADPSLQAPVRVSMQLRRPSDRELSEPME  230
_PNI93761.1_      185  CDKVQKEDIEVYFTGPGWEARGSFSQADVHRQVAIVFRTPPYADPSLQAPVRVSMQLRRPSDRELSEPME  254
_XP_054381607.1_  185  CDKVQKEDIEVYFTGPGWEARGSFSQADVHRQVAIVFRTPPYADPSLQAPVRVSMQLRRPSDRELSEPME  254
_XP_011797882.1_  185  CDKVQKEDIEVYFTGPGWEARGSFSQADVHRQVAIVFRTPPYADPSLQAPVRVSMQLRRPSDRELSEPME  254
_XP_011820578.1_  185  CDKVQKEDIEVYFTGPGWEARGSFSQADVHRQVAIVFRTPPYADPSLQAPVRVSMQLRRPSDRELSEPME  254
_XP_024644798.1_  185  CDKVQKEDIEVYFTGPGWEARGSFSQADVHRQVAIVFRTPPYADPSLQAPVRVSMQLRRPSDRELSEPME  254
_XP_012318394.1_  185  CDKVQKEDIEVYFTGPGWEARGSFSQADVHRQVAIVFRTPPYADPSLQAPVRVFMQLRRPSDRELSEPME  254
_XP_035119199.1_  185  CDKVQKEDIEVYFTGPGWEARGSFSQADVHRQVAIVFRTPPYADPSLQAPVRVFMQLRRPSDRELSEPME  254
_XP_036717345.1_  185  CDKVQKEDIEVYFTGPGWEARGSFSQADVHRQVAIVFRTPPYADPGLQAPVRVSMQLRRPSDRELSEPME  254
_XP_051001869.1_  185  CDKVQKEDIEVYFTGPGWEARGSFSQADVHRQVAIVFRTPPYADPSLQAPVRVSMQLRRPSDRELSEPME  254
_XP_007171011.2_  185  CDKVQKEDIEVYFTGPGWEARGSFSQAYLHRQVAIVFRTPPYADPGLQAPVRVSMQLRRPSDRELSEPME  254
_XP_033272588.1_  185  CDKVQKEDIEVYFTGPGWEARGSFSQADVHRQVAIVFRTPPYADPSLQAPVRVSMQLRRPSDRELSEPME  254
_XP_026946119.1_  185  CDKVQKEDIEVYFTGPGWEARGSFSQADVHRQVAIVFRTPPYADPSLQAPVRVSMQLRRPSDRELSEPME  254
_XP_048314002.1_  185  CDKVQKEDIEVYFTGPGWEARGSFSQADVHRQVAIVFRTPPYADPSLQAPVRVSMQLRRPSDRELSEPME  254
_XP_014648508.1_  185  CDKVQKEDIEVYFTGPGWEARGSFSQADVHRQVAIVFRTPPYADPSLQVPVRVSMQLRRPSDRELSEPME  254
_NP_001389477.1_  185  CDKVQKEDIEVYFTGPGWEARGSFSQADVHRQVAIVFRTPPYADPSLQAPVRVSMQLRRPSDRELSEPME  254
_XP_049637117.1_  185  CDKVQKEDIEVYFTGPNWEARGSFSQADVHRQVAIVFRTPPYMDPSLQAPVRVSMQLRRPSDRELSEPME  254
_XP_054367813.1_  185  CDKVQKEDIEVYFTGPGWEARGSFSQADVHRQVAIVFRTPPYADPSLQAPVRVSMQLRRPSDRELSEPME  254
_XP_041577696.1_  185  CDKVQKEDIEVYFTGPGWEARGSFSQADVHRQVAIVFRTPPYADPSLQAPVRVSMQLRRPSDRELSEPME  254
_XP_005631531.1_  185  CDKVQKEDIEVYFTGPGWEARGSFSQADVHRQVAIVFRTPPYADPSLQAPVRVTMQLRRPSDRELSEPME  254
_KAF6105898.1_    185  CDKVQKEDIEVYFTGPGWEARGSFSQADVHRQVAIVFRTPPYADPGLQAPVRVSMHLRRPSDRELSEPME  254
_XP_012790426.1_  185  CDKVQKEDIEVYFTGPGWEARGSFSQADVHRQVAIVFRTPPYADPGLQAPVRVSMQLRRPSDRELSEPME  254
_XP_031245195.1_   74  CDKVQKEDIEVYFTGPGWEARGSFSQADVHRQVAIVFRTPPYADPSLQAPVRVSMQLRRPSDRELSEPME  143
_NP_001391590.1_   71  CDKVQKEDIEVYFTGPGWEARGSFSQADVHRQVAIVFRTPPYADPSLQAPVRVSMQLRRPSDRELSEPME  140
_XP_053417695.1_   71  CDKVQKEDIEVYFTGPGWEARGSFSQADVHRQVAIVFRTPPYADPSLQAPVRVSMQLRRPSDRELSEPME  140
_XP_027240591.1_   72  CDKVQKEDIEVYFTGPGWEARGSFSQADVHRQVAIVFRTPPYADPSLQAPVRVSMQLRRPSDRELSEPME  141
_XP_027264387.1_   72  CDKVQKEDIEVYFTGPGWEARGSFSQADVHRQVAIVFRTPPYADPSLQAPVRVSMQLRRPSDRELSEPME  141
_XP_051001870.1_   71  CDKVQKEDIEVYFTGPGWEARGSFSQADVHRQVAIVFRTPPYADPSLQAPVRVSMQLRRPSDRELSEPME  140
_XP_023415353.1_   71  CDKVQKEDIEVCFTGPGWEARGSFSQADVHRQVAIVFRTPPYADPSLQAPVRVSMQLRRPSDRELSEPME  140
_XP_032747031.1_   71  CDKVQKEDIEVYFTGPGWEARGSFSQADVHRQVAIVFRTPPYADPSLQAPVRVSMQLRRPSDRELSEPME  140
_XP_052048334.1_   71  CDKVQKEDIEVYFTGPGWEARGSFSQADVHRQVAIVFRTPPYADPGLQAPVRVSMQLRRPSDRELSEPME  140
_XP_028019514.1_   71  CDKVQKEDIEVYFTGPGWEARGSFSQAYLHRQVAIVFRTPPYADPGLQAPVRVSMQLRRPSDRELSEPME  140
```

```
_XP_049993277.1_    71  CDKVQKEDIEVYFTGPGWEARGSFSQADVHRQVAIVFRTPPYADPSLQAPVRVSMQLRRPSDRELSEPME  140
_XP_044617925.1_    71  CDKVQKEDIEVYFTGPGWEARGSFSQADVHRQVAIVFRTPPYADPSLQAPVRVSMQLRRPSDRELSEPME  140
_XP_048216513.1_    70  CDKVQKEDIEVYFTGPGWEARGSFSQADVHRQVAIVFRTPPYADPGLQAPVRVSMQLRRPSDRELSEPME  139
_XP_029414057.1_    71  CDKVQKEDIEVYFTGPGWEARGSFSQADVHRQVAIVFRTPPYADPSLQAPVRVSMQLRRPSDRELSEPME  140
_XP_049569046.1_    71  CDKVQKEDIEVYFTGPGWEARGSFSQADVHRQVAIVFRTPPYADPSLQAPVRVSMQLRRPSDRELSEPME  140
_XP_040605354.1_    72  CDKVQKEDIEVYFTGPGWEARGSFSQADVHRQVAIVFRTPPYADPSLQAPVRVSMQLRRPSDRELSEPME  141
_XP_048314004.1_    71  CDKVQKEDIEVYFTGPGWEARGSFSQADVHRQVAIVFRTPPYADPSLQAPVRVSMQLRRPSDRELSEPME  140
_XP_041518059.1_    71  CDKVQKEDIEVYFTGPGWEARGSFSQADVHRQVAIVFRTPPYADPSLQAPVRVSMQLRRPSDRELSEPME  140
_NP_001351996.1_    71  CDKVQKEDIEVYFTGPGWEARGSFSQADVHRQVAIVFRTPPYADPSLQAPVRVSMQLRRPSDRELSEPME  140
_XP_038935449.1_    72  CDKVQKEDIEVYFTGPGWEARGSFSQADVHRQVAIVFRTPPYADPSLQAPVRVSMQLRRPSDRELSEPME  141
_XP_038170707.1_    71  CDKVQKEDIEVYFTGPGWEARGSFSQADVHRQVAIVFRTPPYADPSLQAPVRVSMQLRRPSDRELSEPME  140
_XP_040140051.1_    71  CDKVQKEDIEVYFTGPGWEARGSFSQADVHRQVAIVFRTPPYADPSLQAPVRVSMQLRRPSDRELSEPME  140
_XP_052568262.1_    71  CDKVQKEDIEVYFTGPGWEARGSFSQADVHRQVAIVFRTPPYADPSLQAPVRVSMQLRRPSDRELSEPME  140
_XP_042115241.1_    72  CDKVQKEDIEVYFTGPGWEARGSFSQADVHRQVAIVFRTPPYADPSLQAPVRVSMQLRRPSDRELSEPME  141
_XP_054580929.1_    71  CDKVQKEDIEVYFTGPGWEARGSFSQADVHRQVAIVFRTPPYADPTLQAPVRVSMQLRRPSDRELSEPME  140
_XP_054367811.1_    72  CDKVQKEDIEVYFTGPGWEARGSFSQADVHRQVAIVFRTPPYADPSLQAPVRVSMQLRRPSDRELSEPME  141
_XP_027628238.1_    71  CDKVQKEDIEVYFTGPGWEARGSFSQADVHRQVAIVFRTPPYADPSLQAPVRVSMQLRRPSDRELSEPME  140
_XP_044536462.1_   196  CDKVQKEDIEVCFSGPGWEARGSFSQADVHRQVAIVFRTPPYAEAALQAPVRVHMQLRRPSDRELSEPME  265
_XP_043827224.1_   196  CDKVQKEDIEVCFTGPGWEARGSFSQADVHRQVAIVFRTPPYAEPALQAPVRVHMQLRRPSDRELSEPME  265
_XP_027711838.1_   196  CDKVQKEDIEVCFRGPGWEARGSFSQADVHRQVAIVFRTPPYAEPALQAPVRVHMQLRRPSDRELSEPME  265
_XP_020824046.1_   196  CDKVQKEDIEVCFTGPGWEARGSFSQADVHRQVAIVFRTPPYAEPALQAPVRVHMQLRRPSDRELSEPME  265
_XP_036619952.1_   196  CDKVQKEDIEVCFTGPGWEARGSFSQADVHRQVAIVFRTPPYAEPTLQAPVRVHMQLRRPSDRELSEPME  265
_XP_031799278.1_   196  CDKVQKEDIEVCFTGQGWEARGSFSQADVHRQVAIVFRTPPYAEPALAAPVRVHMQLRRPSDRELSEPME  265
_XP_007502748.1_   196  CDKVQKEDIEVCFSGPGWEARGSFSQADVHRQVAIVFRTPPYAEAALQAPVRVHMQLRRPSDRELSEPME  265
_XP_051821546.1_   196  CDKVQKEDIEVCFTGQGWEARGSFSQADVHRQVAIVFRTPPYAEPALAAPVRVHMQLRRPSDRELSEPME  265
_XP_051821547.1_   196  CDKVQKEDIEVCFTGQGWEARGSFSQADVHRQVAIVFRTPPYAEPALAAPVRVHMQLRRPSDRELSEPME  265
_KAF6105894.1_     114  CDKVQKEDIEVYFTGPGWEARGSFSQADVHRQVAIVFRTPPYADPGLQAPVRVSMHLRRPSDRELSEPME  183
_XP_008508783.1_   109  CDKVQKEDIEVYFTGPGWEARGSFSQADVHRQVAIVFRTPPYADPSLQAPVRVSMQLRRPSDRELSEPME  178
_MBV98069.1_       131  CDKVQKEDIEVYFTGPGWEARGSFSQADVHRQVAIVFRTPPYADPGLQAPVRVSMQLRRPSDRELSEPME  200
_XP_045411956.1_   155  CDKVQKEDIEVYFTGPGWEARGSFSQADVHRQVAIVFRTPPYADPSLQAPVRVSMQLRRPSDRELSEPME  224
_XP_012520962.1_   155  CDKVQKEDIEVYFTGPGWEARGSFSQADVHRQVAIVFRTPPYADPSLQAPVRVSMQLRRPSDRELSEPME  224
_XP_043307319.1_   155  CDKVQKEDIEVYFTGPGWEARGSFSQADVHRQVAIVFRTPPYADPSLQAPVRVSMQLRRPSDRELSEPME  224
_XP_012666134.1_   155  CDKVQKEDIEVYFTGPGWEARGSFSQADVHRQVAIVFRTPPYVDPSLQAPVRVSMQLRRPSDRELSEPME  224
_XP_011367280.1_   155  CDKVQKEDIEVYFTGPGWEARGSFSQADVHRQVAIVFRTPPYADPSLQAPVRVSMQLRRPSDRELSEPME  224
_KAF7238569.1_     106  CDKVQKEDIEVRFFKDAWEAKGSFSQADVHRQVAIVFKTPPYMDQALREPVTVQMQLRRPSDKEVSGPME  175
_KAG8146725.1_     169  ----------------------SFSQADVHRQVAIVFKTPPYVDQSIREPVTVQMQLHRPSDKEVSGSME  216
_XP_048371352.1_   197  CDKVQKEDIEVRFFKGSWEAKGSFSQADVHRQVAIVFKTPPYMDHSLREPVTVQMQLRRPSDKEVSGPME  266
_XP_015272351.1_   197  CDKVQKEDIEVRFFKDSWEAKGSFSQADVHRQVAIVFRTPPYMDQTLREPVMVQMQLRRPSDKEVSGPME  266
_XP_054845680.1_   197  CDKVQKEDIEVRFFKDSWEAKGSFSQADVHRQVAIVFKTPPYMDQTLREPVTVQMQLRRPSDKEVSGPME  266
```

```
_XP_015272352.1_  197  CDKVQKEDIEVRFFKDSWEAKGSFSQADVHRQVAIVFRTPPYMDQTLREPVMVQMQLRRPSDKEVSGPME  266
_XP_042295897.1_  197  CDKVQKEDIEVRFFKDSWEAKGSFSQADVHRQVAIVFKTPPYMDQALREPVTVQMQLRRPSDKEVSGPME  266
_XP_053133861.1_  197  CDKVQKEDIEVRFFKDSWEAKGSFSQADVHRQVAIVFKTPPYLDQTLREPVTVQMQLRRPSDKEVSGPME  266
_XP_053133862.1_  197  CDKVQKEDIEVRFFKDSWEAKGSFSQADVHRQVAIVFKTPPYLDQTLREPVTVQMQLRRPSDKEVSGPME  266
_KAJ6654174.1_    197  CDKVQKEDIEVRFFKDSWEAKGSFSQADVHRQVAIVFKTPPYMDQSLREPVTVQMQLRRPSDKEVSGPME  266
_XP_044304454.1_  197  CDKVQKEDIEVRFFKDAWEAKGSFSQADVHRQVAIVFKTPPYMDQALREPVTVQMQLRRPSDKEVSGPME  266
_XP_020633015.1_  196  CDKVQKEDIEVRFFKDSWEARGSFSQADVHRQVAIVFKTPPYMDQTLREPVTVQMQLRRPSDKEVSGPME  265
_XP_028566864.1_  197  CDKVQKEDIEVRFFKDSWEAKGSFSQADVHRQVAIVFKTPPYMDQALREPVTVQMQLRRPSDKEVSGPME  266
_XP_033030397.1_  197  CDKVQKEDIEVRFFKDSWEAKGSFSQADVHRQVAIVFKTPPYMDQTLREPVTVQMQLRRPSDKEVSGPME  266
_XP_044304455.1_  197  CDKVQKEDIEVRFFKDAWEAKGSFSQADVHRQVAIVFKTPPYMDQALREPVTVQMQLRRPSDKEVSGPME  266
_XP_053225758.1_  197  CDKVQKEDIEVRFFKDSWEAKGSFSQADVHRQVAIVFKTPPYMDQALREPVTVQMQLRRPSDKEVSGPME  266
_XP_033030398.1_  197  CDKVQKEDIEVRFFKDSWEAKGSFSQADVHRQVAIVFKTPPYMDQTLREPVTVQMQLRRPSDKEVSGPME  266
_XP_034953722.1_  197  CDKVQKEDIEVRFFKDSWEAKGSFSQADVHRQVAIVFKTPPYMDQTLREPVTVQMQLRRPSDKEVSGPME  266
_XP_008123768.1_  197  CDKVQKEDIEVRFFKDSWEAKGSFSQADVHRQVAIVFKTPPYLDQALREPVTIQMQLRRPSDKEVSGPME  266
_KAJ7307406.1_    186  CDKVQKEDIEVRFFKDSWEARGSFSQADVHRQVAIVFKTPPYMDQALREPVTVQMQLRRPSDKEVSGPME  255
_XP_013931335.1_  197  CDKVQKEDIEVRFFKDSWEAKGSFSQADVHRQVAIVFKTPPYADQTIREPVTVQMQLHRPSDKEVSGSME  266
_XP_032089926.1_  197  CDKVQKEDIEVRFFKDSWEAKGSFSQADVHRQVAIVFKTPPYADQTIREPVTVQMQLHRPSDKEVSGSME  266
_XP_026572203.1_  197  CDKVQKEDIEVRFFKDSWEAKGSFSQADVHRQVAIVFKTPPYADQTIREPVTVQMQLHRPSDKEVSGSME  266
_XP_026572204.1_  197  CDKVQKEDIEVRFFKDSWEAKGSFSQADVHRQVAIVFKTPPYADQTIREPVTVQMQLHRPSDKEVSGSME  266
_XP_034280315.1_  197  CDKVQKEDIEVRFFKDSWEAKGSFSQADVHRQVAIVFKTPPYADQTIREPVTVQMQLHRPSDKEVSGSME  266
_XP_039222066.1_  196  CDKVQKEDIEVRFFKDSWEAKGSFSQADVHRQVAIVFKTPPYADQAIREPVTVQMQLHRPSDKEVSGSME  265
_XP_015744179.1_  186  CDKVQKEDIEVRFFKDSWEAKGSFSQADVHRQVAIVFKTPPYVDQGLREPVTVQMQLRRPSDKEVSGSME  255
_XP_029142061.1_  196  CDKVQKEDIEVRFFKDSWEAKGSFSQADVHRQVAIVFKTPPYVDQGLREPVTVQMQLRRPSDKEVSGSME  265
_AAH41188.1_      197  CDKVQKEDIEVIFGLGNWEARGIFSQADVHRQVAIVFRTPAFQDTKIRQSVKVQMQLRRPSDKEVSEPME  266
_XP_018112002.1_  197  CDKVQKEDIEVIFGLGNWEARGIFSQADVHRQVAIVFRTPAFQDTKIRQSVKVQMQLRRPSDKEVSEPME  266
_NP_001081048.1_  197  CDKVQKEDIEVIFGLGNWEARGIFSQADVHRQVAIVFRTPAFQDTKIRQSVKVQMQLRRPSDKEVSEPME  266
_XP_031755832.1_  197  CDKVQKEDIEVLFVLGNWEARGIFSQADVHRQVAIVFRTPAFHDTKIRQSVKVQMQLRRPSDKEVSEPME  266
_KAG8437862.1_    197  CDKVQKEDIEVVFVLGNWESRGIFSQADVHRQVAIVFKTPPFQDPGIRQSVKVQMQLRRPSDKEVSEPME  266
_XP_053304358.1_  197  CDKVQKEDIEVVFVLGNWEARGSFSQADVHRQVAIVFKTPAYQDQGIRQPVKVQMHLRRPSDKEVSEPME  266
_KAG9464550.1_    193  CDKVQKEDIEVVFVHGNWEARGSFSQADVHRQVAIVFKTPPYQDAEIHQPVKVQMQLRRPSDKEVSEAME  262
_XP_040265411.1_  196  CDKVQKEDIEVVFVHGNWEARGSFSQADVHRQVAIVFKTPPYQDPEIHQPVKVQMQLRRPSDKEVSEPME  265
_XP_044125715.1_  196  CDKVQKEDIEVVFVHGNWEARGSFSQADVHRQVAIVFKTPPYQDPEIHQPVKVQMQLRRPSDKEVSEPME  265
_XP_040265414.1_  196  CDKVQKEDIEVVFVHGNWEARGSFSQADVHRQVAIVFKTPPYQDPEIHQPVKVQMQLRRPSDKEVSEPME  265
_XP_044125717.1_  196  CDKVQKEDIEVVFVHGNWEARGSFSQADVHRQVAIVFKTPPYQDPEIHQPVKVQMQLRRPSDKEVSEPME  265
_XP_040184801.1_  196  CDKVQKEDIEVVFAVGNWEARGSFSQADVHRQVAIVFRTPPYHDPGIRQPVKVQMQLRRPSDKEVSEPME  265
_ALA55869.1_      196  CDKVQKEDIEVVFAVGNWEARGSFSQADVHRQVAIVFRTPPYHDPEIRQPVKVQMQLRRPSDKEVSEPME  265
_XP_018408357.1_  196  CDKVQKEDIEVVFALGNWEARGSFSQADVHRQVAIVFRTPQYHDPGIRQPVKVQMQLRRPSDKEVSEPME  265
_XP_053575987.1_  197  CDKVQKEDIEVVFTLGNWECRGSFSQADVHRQVAIVFKTPPFQDINIRQPVKVQMQLRRPSDKEVSEPME  266
_XP_033812535.1_  197  CDKVQKEDIEVRFFLDFWEAKGNFSQADVHRQVAIVFRTPPYLDQNIREPVKVQMQLRRPSDKEVSEAME  266
```

```
_XP_030074553.1_   197  CDKVQKEDIEVRFFLDSWEAKGNFSQADVHRQVAIVFRTPPYLDRNIREPVKVQMQLRRPSDKEVSEAME  266
_XP_029468059.1_   197  CDKVQKEDIEVRFFLDTWEAKGNFSQADVHRQVAIVFRTPPYLDPNIREPVKVQMQLRRPSDKEVSEAME  266
_XP_029468060.1_   197  CDKVQKEDIEVRFFLDTWEAKGNFSQADVHRQVAIVFRTPPYLDPNIREPVKVQMQLRRPSDKEVSEAME  266
_KAJ1106220.1_     197  CDKVQKEDIEVRFFLGTWEAKGTFSQADVHRQVAIVFRTPAYQNQAIREPVRVQMQLRRPSDKEVSEAME  266
_CAI5672275.1_     185  CDKVQKEDIEVRFFQDSWEGKGTFSQADVHRQVAIVFRTPPYRDTNLTEPVRVKMQLRRPSDREVSEPMD  254
_GCB80996.1_       140  CDKVQKEDIEVRFFTNDWEGKGSFSQADVHRQVAIVFKTPPYKDVTLSQPVTVRMQLRRPSDKEVSDAME  209
_XP_038618162.1_   196  CDKVQKS---------------------------------------------------------------  202
Consensus aa:           CDKVQKEDIEV.Fh..tWEA+GSFtQADVHRQVAIVF+TPP@.D.slp.PVpVpMpLpRPSD+p.S.Ph-
Consensus ss:           e        eeeeee   eeeeee hhhh   eeeeee                eeeeeeeee         ee


Conservation:           9799                                 9
_XP_028917328.1_   266  FQYL---------------------------------PD  271
_XP_038621028.1_   266  FQYL---------------------------------PD  271
_RLV71517.1_       213  FRYL---------------------------------PH  218
_XP_033367665.1_   223  FRYL---------------------------------PH  228
_OWK50979.1_        63  FRYL---------------------------------PH   68
_XP_031363427.1_   227  FRYL---------------------------------PH  232
_XP_041319512.1_   173  FRYL---------------------------------PH  178
_XP_054509575.1_   254  FRYL---------------------------------PH  259
_XP_039909596.1_   254  FRYL---------------------------------PH  259
_TRZ10035.1_       254  FRYL---------------------------------PH  259
_XP_031950790.1_   254  FRYL---------------------------------PH  259
_XP_050163562.1_   254  FRYL---------------------------------PH  259
_XP_039428642.1_   254  FRYL---------------------------------PH  259
_XP_050840410.1_   269  FRYL---------------------------------PH  274
_XP_054151317.1_   265  FRYL---------------------------------PH  270
_XP_050840409.1_   265  FRYL---------------------------------PH  270
_XP_030825825.1_   265  FRYL---------------------------------PH  270
_XP_032939479.1_   265  FRYL---------------------------------PH  270
_XP_041567964.1_   265  FRYL---------------------------------PH  270
_XP_048149083.1_   265  FRYL---------------------------------PH  270
_XP_054374241.1_   265  FRYL---------------------------------PH  270
_NXV55901.1_       265  FRYL---------------------------------PH  270
_XP_005533697.1_   265  FRYL---------------------------------PH  270
_XP_014748521.1_   265  FRYL---------------------------------PH  270
_RMB90491.1_       265  FRYL---------------------------------PH  270
_XP_053857772.1_   265  FRYL---------------------------------PH  270
_XP_029862760.1_   139  FRYL---------------------------------PH  144
```

```
_XP_039575719.1_  240  FRYL---------------------------------PH  245
_XP_054664313.1_  265  FRYL---------------------------------PH  270
_XP_049691648.1_  265  FRYL---------------------------------PH  270
_XP_030331239.1_  266  FRYL---------------------------------PH  271
_XP_010726496.2_       ---------------------------------------
_XP_042747731.1_       ---------------------------------------
_XP_031466227.1_       ---------------------------------------
_XP_032297702.1_  265  FRYL---------------------------------PH  270
_RMB90505.1_           ---------------------------------------
_KAI2561003.1_         ---------------------------------------
_KAF6105893.1_    210  -------------------------------------PQ  211
_PNJ38430.1_           ---------------------------------------
_PNI93749.1_           ---------------------------------------
_KAF6334124.1_         ---------------------------------------
_KAF6467432.1_    210  -------------------------------------PQ  211
_PNI93759.1_           ---------------------------------------
_PNJ38434.1_           ---------------------------------------
_KAH0508019.1_    273  FQYL---------------------------------PD  278
_AAA36408.1_      266  FQYL---------------------------------PD  271
_XP_003274247.2_  266  FQYL---------------------------------PD  271
_XP_007981896.1_  266  FQYL---------------------------------PD  271
_XP_054296639.1_  266  FQYL---------------------------------PD  271
_XP_023042293.1_  266  FQYL---------------------------------PD  271
_XP_025211457.1_  266  FQYL---------------------------------PD  271
_XP_039326898.1_  266  FQYL---------------------------------PD  271
_XP_021781237.2_  266  FQYL---------------------------------PD  271
_XP_010364772.1_  266  FQYL---------------------------------PD  271
_XP_033063134.1_  266  FQYL---------------------------------PD  271
_XP_017392927.1_  266  FQYL---------------------------------PD  271
_XP_011897055.1_  266  FQYL---------------------------------PD  271
_XP_032138725.1_  266  FQYL---------------------------------PD  271
_CAA80524.2_      266  FQYL---------------------------------PD  271
_XP_005577305.2_  266  FQYL---------------------------------PD  271
_BAE87977.1_      266  FQYL---------------------------------PD  271
_XP_023042294.1_  266  FQYL---------------------------------PD  271
_XP_050612617.1_  266  FQYL---------------------------------PD  271
_EHH56082.1_      266  FQYL---------------------------------PD  271
_XP_028689064.1_  266  FQYL---------------------------------PD  271
_XP_045411957.1_  266  FQYL---------------------------------PD  271
```

```
_XP_012520963.1_  266  FQYL---------------------------------PD  271
_XP_012633553.1_  266  FQYL---------------------------------PD  271
_XP_053417694.1_  266  FQYL---------------------------------PD  271
_XP_032976853.1_  266  FQYL---------------------------------PD  271
_XP_008576943.1_  266  FQYL---------------------------------PD  271
_XP_032345730.1_  266  FQYL---------------------------------PD  271
_XP_010955442.1_  266  FQYL---------------------------------PD  271
_XP_031303872.1_  266  FQYL---------------------------------PD  271
_XP_032976854.1_  266  FQYL---------------------------------PD  271
_XP_053780986.1_  266  FQYL---------------------------------PD  271
_XP_036115166.1_  266  FQYL---------------------------------PD  271
_XP_043307320.1_  266  FQYL---------------------------------PD  271
_EQB77721.1_      266  FQYL---------------------------------PD  271
_NP_001107753.1_  266  FQYL---------------------------------PD  271
_XP_036785182.1_  266  FQYL---------------------------------PD  271
_XP_020762118.1_  266  FQYL---------------------------------PD  271
_XP_031546756.1_  266  FQYL---------------------------------PD  271
_XP_023989099.1_  266  FQYL---------------------------------PD  271
_KAF4009900.1_    266  FQYL---------------------------------PD  271
_KAB0376704.1_    266  FQYL---------------------------------PD  271
_KAB0364713.1_    266  FQYL---------------------------------PD  271
_CBT01657.1_      266  FQYL---------------------------------PD  271
_XP_036851744.1_  266  FQYL---------------------------------PD  271
_XP_007128247.1_  266  FQYL---------------------------------PD  271
_XP_003798713.1_  266  FQYL---------------------------------PD  271
_NP_001073711.1_  266  FQYL---------------------------------PD  271
_XP_006056522.1_  266  FQYL---------------------------------PD  271
_XP_019593326.1_  266  FQYL---------------------------------PD  271
_XP_005894159.1_  266  FQYL---------------------------------PD  271
_ELR57541.1_      266  FQYL---------------------------------PD  271
_XP_010836092.1_  266  FQYL---------------------------------PD  271
_XP_051049621.1_  266  FQYL---------------------------------PD  271
_CBT01658.1_      266  FQYL---------------------------------PD  271
_XP_015974930.1_  266  FQYL---------------------------------PD  271
_XP_027264386.1_  266  FQYL---------------------------------PD  271
_XP_040112158.1_  266  FQYL---------------------------------PD  271
_XP_052517633.1_  266  FQYL---------------------------------PD  271
_XP_017898873.1_  266  FQYL---------------------------------PD  271
_XP_020020484.1_  266  FQYL---------------------------------PD  271
```

_XP_047631223.1_ 266 FQYL---------------------------------PD 271
_XP_007651509.2_ 266 FQYL---------------------------------PD 271
_XP_027815096.1_ 266 FQYL---------------------------------PD 271
_XP_034377707.1_ 266 FQYL---------------------------------PD 271
ERE77884.1   266 FQYL---------------------------------PD 271
_XP_028626823.1_ 266 FQYL---------------------------------PD 271
KAF5928161.1   266 FQYL---------------------------------PD 271
KAI5933031.1   266 FQYL---------------------------------PD 271
_XP_021007720.1_ 266 FQYL---------------------------------PD 271
_XP_026252329.1_ 266 FQYL---------------------------------PD 271
_XP_054426842.1_ 266 FQYL---------------------------------PD 271
_XP_032747030.1_ 266 FQYL---------------------------------PD 271
_XP_049748463.1_ 266 FQYL---------------------------------PD 271
KAJ1065952.1   266 FQYL---------------------------------PD 271
_XP_052048317.1_ 266 FQYL---------------------------------PD 271
_XP_012876614.1_ 266 FQYL---------------------------------PD 271
_XP_020020482.1_ 266 FQYL---------------------------------PD 271
_XP_010597340.1_ 266 FQYL---------------------------------PD 271
_XP_026339000.2_ 266 FQYL---------------------------------PD 271
_XP_030689468.1_ 266 FQYL---------------------------------PD 271
_XP_049993276.1_ 266 FQYL---------------------------------PD 271
_XP_014700317.1_ 266 FQYL---------------------------------PD 271
_XP_021494126.1_ 266 FQYL---------------------------------PD 271
_XP_022447167.1_ 266 FQYL---------------------------------PD 271
_XP_042532137.1_ 266 FQYL---------------------------------PD 271
_XP_008820421.1_ 266 FQYL---------------------------------PD 271
_XP_023415352.1_ 266 FQYL---------------------------------PD 271
_XP_029414056.1_ 266 FQYL---------------------------------PD 271
_XP_024604853.1_ 266 FQYL---------------------------------PD 271
_XP_007462052.1_ 266 FQYL---------------------------------PD 271
_XP_006101621.1_ 266 FQYL---------------------------------PD 271
_XP_005064049.1_ 266 FQYL---------------------------------PD 271
_XP_031245193.1_ 266 FQYL---------------------------------PD 271
ELK24332.1   266 FQYL---------------------------------PD 271
_XP_041518057.1_ 266 FQYL---------------------------------PD 271
_XP_005351804.2_ 266 FQYL---------------------------------PD 271
EGW13067.1   266 FQYL---------------------------------PD 271
ERE77883.1   266 FQYL---------------------------------PD 271
KFO28401.1   266 FQYL---------------------------------PD 271

```
_XP_010634478.1_  266  FQYL---------------------------------PD  271
_OBS81518.1_      266  FQYL---------------------------------PD  271
_XP_007462051.1_  266  FQYL---------------------------------PD  271
_XP_014393535.1_  266  FQYL---------------------------------PD  271
_XP_048216512.1_  266  FQYL---------------------------------PD  271
_XP_015424455.1_  266  FQYL---------------------------------PD  271
_XP_019501147.1_  266  FQYL---------------------------------PD  271
_XP_004656738.2_  266  FQYL---------------------------------PD  271
_XP_036909111.1_  266  FQYL---------------------------------PD  271
_XP_036182518.1_  266  FQYL---------------------------------PD  271
_XP_012580591.1_  266  FQYL---------------------------------PD  271
_XP_016063765.1_  266  FQYL---------------------------------PD  271
_XP_021047054.1_  266  FQYL---------------------------------PD  271
_XP_023095836.1_  266  FQYL---------------------------------PD  271
_XP_045342734.1_  266  FQYL---------------------------------PD  271
_XP_053755429.1_  266  FQYL---------------------------------PD  271
_XP_022447166.1_  266  FQYL---------------------------------PD  271
_XP_004713741.1_  266  FQYL---------------------------------PD  271
_NP_954888.1_     266  FQYL---------------------------------PD  271
_XP_037002389.2_  266  FQYL---------------------------------PD  271
_XP_039100808.1_  266  FQYL---------------------------------PD  271
_XP_038170688.1_  266  FQYL---------------------------------PD  271
_XP_040495345.1_  266  FQYL---------------------------------PD  271
_XP_052568261.1_  266  FQYL---------------------------------PD  271
_XP_042760134.1_  266  FQYL---------------------------------PD  271
_XP_049472392.1_  266  FQYL---------------------------------PD  271
_XP_023593974.1_  266  FQYL---------------------------------PD  271
_XP_034501886.1_  266  FQYL---------------------------------PD  271
_XP_004393991.1_  266  FQYL---------------------------------PD  271
_XP_025860214.1_  266  FQYL---------------------------------PD  271
_XP_019501146.1_  266  FQYL---------------------------------PD  271
_XP_025734327.1_  266  FQYL---------------------------------PD  271
_XP_025301454.1_  266  FQYL---------------------------------PD  271
_KAG3284687.1_    266  FQYL---------------------------------PD  271
_VFV23136.1_      266  FQYL---------------------------------PD  271
_XP_025771173.1_  266  FQYL---------------------------------PD  271
_XP_054580928.1_  266  FQYL---------------------------------PD  271
_EPQ06533.1_      266  FQYL---------------------------------PD  271
_XP_034869141.1_  266  FQYL---------------------------------PD  271
```

_XP_042813589.1_   266 FQYL---------------------------------PD 271
_XP_040322176.1_   266 FQYL---------------------------------PD 271
_XP_006861137.1_   266 FQYL---------------------------------PD 271
_XP_007943155.2_   266 FQYL---------------------------------PD 271
BAQ95593.1   266 FQYL---------------------------------PD 271
_XP_019652454.2_   266 FQYL---------------------------------PD 271
_XP_016041658.1_   266 FQYL---------------------------------PD 271
_XP_047546997.1_   266 FQYL---------------------------------PD 271
_XP_042115231.1_   266 FQYL---------------------------------PD 271
_XP_042115227.1_   266 FQYL---------------------------------PD 271
_XP_015854576.1_   266 FQYL---------------------------------PD 271
_XP_025301452.1_   266 FQYL---------------------------------PD 271
_XP_044920951.1_   266 FQYL---------------------------------PD 271
KAF3822254.1   266 FQYL---------------------------------PD 271
_XP_021540718.1_   266 FQYL---------------------------------PD 271
_XP_022362935.1_   266 FQYL---------------------------------PD 271
_XP_044115623.1_   266 FQYL---------------------------------PD 271
_XP_032735359.1_   266 FQYL---------------------------------PD 271
_XP_032270304.1_   266 FQYL---------------------------------PD 271
_XP_032213216.1_   266 FQYL---------------------------------PD 271
_XP_004478661.1_   266 FQYL---------------------------------PD 271
_XP_035933519.1_   266 FQYL---------------------------------PD 271
KAI5761432.1   266 FQYL---------------------------------PD 271
_XP_029813244.1_   266 FQYL---------------------------------PD 271
_XP_027800377.1_   266 FQYL---------------------------------PD 271
_XP_004852440.1_   266 FQYL---------------------------------PD 271
_XP_045873061.1_   266 FQYL---------------------------------PD 271
_XP_030891354.1_   266 FQYL---------------------------------PD 271
CAD7670066.1   266 FQYL---------------------------------PD 271
_XP_046301192.1_   266 FQYL---------------------------------PD 271
_XP_036059776.1_   266 FQYL---------------------------------PD 271
_XP_028711748.1_   266 FQYL---------------------------------PD 271
_XP_037696258.1_   266 FQYL---------------------------------PD 271
_XP_004627287.1_   266 FQYL---------------------------------PD 271
_XP_037371589.1_   266 FQYL---------------------------------PD 271
_XP_003468122.1_   266 FQYL---------------------------------PD 271
_XP_040826731.1_   266 FQYL---------------------------------PD 271
_XP_005384525.1_   266 FQYL---------------------------------PD 271
_XP_045711286.1_   266 FQYL---------------------------------PD 271

```
_XP_044920955.1_  266  FQYL---------------------------------PD  271
_XP_047375326.1_  266  FQYL---------------------------------PD  271
_XP_036272436.1_  266  FQYL---------------------------------PD  271
_ELK13339.1_      266  FQYL---------------------------------PD  271
_XP_021564615.1_  266  FQYL---------------------------------PD  271
_XP_024902067.1_  266  FQYL---------------------------------PD  271
_XP_036349005.1_  266  FQYL---------------------------------PD  271
_XP_051683930.1_  266  FQYL---------------------------------PD  271
_XP_013006227.1_  266  FQYL---------------------------------PD  271
_XP_006893486.1_  265  FQYL---------------------------------PD  270
_XP_036272437.1_  266  FQYL---------------------------------PD  271
_ELW65121.1_      266  FQYL---------------------------------PD  271
_EHB04873.1_      266  FQYL---------------------------------PD  271
_KAB0407447.1_    266  FQYL---------------------------------PD  271
_KAF6105901.1_    266  FQYL---------------------------------PD  271
_MXQ90627.1_      266  FQYL---------------------------------PD  271
_PNJ38432.1_      266  FQYL---------------------------------PD  271
_PNI93760.1_      266  FQYL---------------------------------PD  271
_KAF6438864.1_    266  FQYL---------------------------------PD  271
_KAF6281766.1_    266  FQYL---------------------------------PD  271
_XP_035119201.1_  266  FQYL---------------------------------PD  271
_KAF6334125.1_    266  FQYL---------------------------------PD  271
_XP_036785207.1_  266  FQYL---------------------------------PD  271
_XP_036851747.1_  266  FQYL---------------------------------PD  271
_XP_054367814.1_  266  FQYL---------------------------------PD  271
_XP_030689470.1_  266  FQYL---------------------------------PD  271
_XP_026946120.1_  266  FQYL---------------------------------PD  271
_XP_039696841.1_  266  FQYL---------------------------------PD  271
_1IKN_A_          266  FQYL---------------------------------PD  271
_XP_016775352.1_  231  FQYL---------------------------------PD  236
_PNI93761.1_      255  FQYL---------------------------------PD  260
_XP_054381607.1_  255  FQYL---------------------------------PD  260
_XP_011797882.1_  255  FQYL---------------------------------PD  260
_XP_011820578.1_  255  FQYL---------------------------------PD  260
_XP_024644798.1_  255  FQYL---------------------------------PD  260
_XP_012318394.1_  255  FQYL---------------------------------PD  260
_XP_035119199.1_  255  FQYL---------------------------------PD  260
_XP_036717345.1_  255  FQYL---------------------------------PD  260
_XP_051001869.1_  255  FQYL---------------------------------PD  260
```

```
_XP_007171011.2_  255  FQYL---------------------------------PD  260
_XP_033272588.1_  255  FQYL---------------------------------PD  260
_XP_026946119.1_  255  FQYL---------------------------------PD  260
_XP_048314002.1_  255  FQYL---------------------------------PD  260
_XP_014648508.1_  255  FQYL---------------------------------PD  260
_NP_001389477.1_  255  FQYL---------------------------------PD  260
_XP_049637117.1_  255  FQYL---------------------------------PD  260
_XP_054367813.1_  255  FQYL---------------------------------PD  260
_XP_041577696.1_  255  FQYL---------------------------------PD  260
_XP_005631531.1_  255  FQYL---------------------------------PD  260
_KAF6105898.1_    255  FQYL---------------------------------PD  260
_XP_012790426.1_  255  FQYL---------------------------------PD  260
_XP_031245195.1_  144  FQYL---------------------------------PD  149
_NP_001391590.1_  141  FQYL---------------------------------PD  146
_XP_053417695.1_  141  FQYL---------------------------------PD  146
_XP_027240591.1_  142  FQYL---------------------------------PD  147
_XP_027264387.1_  142  FQYL---------------------------------PD  147
_XP_051001870.1_  141  FQYL---------------------------------PD  146
_XP_023415353.1_  141  FQYL---------------------------------PD  146
_XP_032747031.1_  141  FQYL---------------------------------PD  146
_XP_052048334.1_  141  FQYL---------------------------------PD  146
_XP_028019514.1_  141  FQYL---------------------------------PD  146
_XP_049993277.1_  141  FQYL---------------------------------PD  146
_XP_044617925.1_  141  FQYL---------------------------------PD  146
_XP_048216513.1_  140  FQYL---------------------------------PD  145
_XP_029414057.1_  141  FQYL---------------------------------PD  146
_XP_049569046.1_  141  FQYL---------------------------------PD  146
_XP_040605354.1_  142  FQYL---------------------------------PD  147
_XP_048314004.1_  141  FQYL---------------------------------PD  146
_XP_041518059.1_  141  FQYL---------------------------------PD  146
_NP_001351996.1_  141  FQYL---------------------------------PD  146
_XP_038935449.1_  142  FQYL---------------------------------PD  147
_XP_038170707.1_  141  FQYL---------------------------------PD  146
_XP_040140051.1_  141  FQYL---------------------------------PD  146
_XP_052568262.1_  141  FQYL---------------------------------PD  146
_XP_042115241.1_  142  FQYL---------------------------------PD  147
_XP_054580929.1_  141  FQYL---------------------------------PD  146
_XP_054367811.1_  142  FQYL---------------------------------PD  147
_XP_027628238.1_  141  FQYL---------------------------------PD  146
```

```
_XP_044536462.1_  266  FQYL---------------------------------PD  271
_XP_043827224.1_  266  FQYL---------------------------------PD  271
_XP_027711838.1_  266  FQYL---------------------------------PD  271
_XP_020824046.1_  266  FQYL---------------------------------PD  271
_XP_036619952.1_  266  FQYL---------------------------------PD  271
_XP_031799278.1_  266  FQYL---------------------------------PD  271
_XP_007502748.1_  266  FQYL---------------------------------PD  271
_XP_051821546.1_  266  FQYL---------------------------------PD  271
_XP_051821547.1_  266  FQYL---------------------------------PD  271
_KAF6105894.1_    184  FQYL---------------------------------PD  189
_XP_008508783.1_  179  FQYLHADPSLQAPVRVSMQLRRPSDRELSEPMEFQYLPD  217
_MBV98069.1_      201  FQYL---------------------------------PD  206
_XP_045411956.1_  225  FQYL---------------------------------PD  230
_XP_012520962.1_  225  FQYL---------------------------------PD  230
_XP_043307319.1_  225  FQYL---------------------------------PD  230
_XP_012666134.1_  225  FQYL---------------------------------PD  230
_XP_011367280.1_  225  FQYL---------------------------------PD  230
_KAF7238569.1_    176  FRYL---------------------------------PD  181
_KAG8146725.1_    217  FRYL---------------------------------PD  222
_XP_048371352.1_  267  FRYL---------------------------------PD  272
_XP_015272351.1_  267  FRYL---------------------------------PD  272
_XP_054845680.1_  267  FRYL---------------------------------PD  272
_XP_015272352.1_  267  FRYL---------------------------------PD  272
_XP_042295897.1_  267  FRYL---------------------------------PD  272
_XP_053133861.1_  267  FRYL---------------------------------PD  272
_XP_053133862.1_  267  FRYL---------------------------------PD  272
_KAJ6654174.1_    267  FRYL---------------------------------PD  272
_XP_044304454.1_  267  FRYL---------------------------------PD  272
_XP_020633015.1_  266  FRYL---------------------------------PD  271
_XP_028566864.1_  267  FRYL---------------------------------PD  272
_XP_033030397.1_  267  FRYL---------------------------------PD  272
_XP_044304455.1_  267  FRYL---------------------------------PD  272
_XP_053225758.1_  267  FRYL---------------------------------PD  272
_XP_033030398.1_  267  FRYL---------------------------------PD  272
_XP_034953722.1_  267  FRYL---------------------------------PD  272
_XP_008123768.1_  267  FRYL---------------------------------PD  272
_KAJ7307406.1_    256  FRYL---------------------------------PR  261
_XP_013931335.1_  267  FRYL---------------------------------PD  272
_XP_032089926.1_  267  FRYL---------------------------------PD  272
```

```
_XP_026572203.1_   267  FRYL---------------------------------PD  272
_XP_026572204.1_   267  FRYL---------------------------------PD  272
_XP_034280315.1_   267  FRYL---------------------------------PD  272
_XP_039222066.1_   266  FRYL---------------------------------PD  271
_XP_015744179.1_   256  FRYL---------------------------------PD  261
_XP_029142061.1_   266  FRYL---------------------------------PD  271
_AAH41188.1_       267  FQYL---------------------------------PD  272
_XP_018112002.1_   267  FQYL---------------------------------PD  272
_NP_001081048.1_   267  FQYL---------------------------------PD  272
_XP_031755832.1_   267  FQYL---------------------------------PD  272
_KAG8437862.1_     267  FQYL---------------------------------PD  272
_XP_053304358.1_   267  FQYL---------------------------------PH  272
_KAG9464550.1_     263  FQYL---------------------------------PY  268
_XP_040265411.1_   266  FQYL---------------------------------PY  271
_XP_044125715.1_   266  FQYL---------------------------------PY  271
_XP_040265414.1_   266  FQYL---------------------------------PY  271
_XP_044125717.1_   266  FQYL---------------------------------PY  271
_XP_040184801.1_   266  FQYL---------------------------------PD  271
_ALA55869.1_       266  FQYL---------------------------------PD  271
_XP_018408357.1_   266  FQYL---------------------------------PD  271
_XP_053575987.1_   267  FQYL---------------------------------PD  272
_XP_033812535.1_   267  FQYL---------------------------------PN  272
_XP_030074553.1_   267  FQYL---------------------------------PD  272
_XP_029468059.1_   267  FQYL---------------------------------PD  272
_XP_029468060.1_   267  FQYL---------------------------------PD  272
_KAJ1106220.1_     267  FQYL---------------------------------PN  272
_CAI5672275.1_     255  FQYL---------------------------------PS  260
_GCB80996.1_       210  FQYL---------------------------------PD  215
_XP_038618162.1_        ---------------------------------------
Consensus_aa:           FpYL.................................Pc
Consensus_ss:           eee
```

p50/p105

```
Conservation:            5 7  69   56 7777 57   567 5     8  5      5  9   5               85
_KAH7637713.1_      1   -LTIIEEPIN-R-IRYRYRSEKG-SHGGLN----GVNSCPI--RKTYPTVKIENYHLS-NPVYVRASLAT   59
_XP_023220155.1_    1   -LKIVNQPIN-R-IRFRYGTECD-SHGVLK----GRNSHGN--SKSYPTVHLENYFGN-ENVHIKASLYT   59
```

```
_XP_009016345.1_     1  -LEIIEEPQS-KGFRFRYACEGK-SHGGLQ----GVNHRKD--KKTVPTVKINRYSG---MARLEVTLVT   58
_ELU08708.1_         1  --EIIEQPQA-RGYRFRYHCEGN-SHGGIQ----GVASRKE--GKTYPTIKIHNYRG---PARVRVTLVT   57
_UCK81536.1_         1  --DIVVQPQQ-RGFRFRYECEGS-SHGGIQ----GESNEVS--GKNYPTVKIHGYTG---PARLTVTLVT   57
_CAH1795432.1_       1  -LHIIEEPQQ-RGFRFRYRSENSASHGGLQ----GCNSKKS--HKSYPTVELRNYQG---TARIVVSLVT   59
_CAE1313443.1_       1  -LTIIEQPQS-RGFRFRYKCEGP-SHGGLQ----GVKSEKG--KKSYPTVQIMNYEG---PARILVSLVT   58
_XP_048764075.1_     1  -VEIVEQPQA-RGFRFRYECEGP-SHGGLQ----GEKSEKY--RKTFPSIRIRNYNG---SARIVVTLVT   58
_XP_009060044.1_     1  -VAIIEEPQS-RGFRFRYECEGP-SHGGLQ----GTSSERG--RKTYPSIKIENYDG---PARIEVSLVN   58
_XP_050391129.1_     1  -VTIAEQPQS-RGFRFRYECEGP-SHGGLQ----GQKSERG--RKTYPSIKIDNYRN---SARIIVSLVT   58
_RUS85575.1_         1  -VVITEQPQQ-RGFRFRYACEGG-SHGGLQ----GTRSERS--RKSYPTIKVENYTG---ALKVVVSLVT   58
_GFS09172.1_         1  -VVITEQPQQ-RGFRFRYPCEGG-SHGGLQ----GTRSERS--RKSYPTIKVENYTG---ALKVVVSLVT   58
_XP_005091024.1_     1  -VVISEQPQQ-RGFRFRYECEGP-SHGGLQ----GTKSERS--KKSYPAIKIENYTG---AARVVVSLVT   58
_XP_005091023.1_     1  -VVISEQPQQ-RGFRFRYECEGP-SHGGLQ----GTKSERS--KKSYPAIKIENYTG---AARVVVSLVT   58
_KAH9508414.1_       1  --FITEQPQQ-RGFRFRYECEGP-SHGGLQ----GTKSERS--RKSYPAIKIENYTG---AARVVVSLVT   57
_XP_013071937.1_     1  -VVITEQPQQ-RGFRFRYECEGP-SHGGLQ----GTKSERS--RKSYPSIKIENYSG---AARVVVSLVT   58
_KAI8766750.1_       1  -VVITEQPQQ-RGFRFRYECEGP-SHGGLQ----GTKSERS--RKSYPSIKIENYSG---AARVVVSLVT   58
_KAI8794647.1_       1  -VVITEQPQQ-RGFRFRYECEGP-SHGGLQ----GTKSERS--RKSYPSIKIENYSG---AARVVVSLVT   58
_NP_001298220.1_     1  -VVITEQPQQ-RGFRFRYECEGP-SHGGLQ----GTKSERS--RKSYPSIKIENYSG---AARVVVSLVT   58
_CAG5129174.1_       1  -VEISEQPQQ-RGFRFRYECEGP-SHGGLQ----GTKSERS--RKSYPTIKICNYSG---AARVVVSLVT   58
_KAG5707696.1_          ----------------------------------------------------------------------
_XP_025114345.1_     1  -VVIVEQPQQ-RGFRFRYECEGP-SHGGLQ----GEKTERG--RKTHPAIKIENYSG---PARIVVSLVT   58
_QNL15288.1_         1  -VVIVEQPQQ-RGFRFRYECEGP-SHGGLQ----GEKTERG--RKTYPSIRIENYSG---PAHIEVSLVT   58
_XP_041362807.1_     1  --IIVEQPQQ-RGFRFRYECEGP-SHGGLQ----GEKSRKN--QKSYPAIRVENYSG---SVRMVVTLVT   57
_XP_046550037.1_     1  -IKIVEEPQQ-RGFRFRYECEGP-SHGGLQ----GEKSEKS--RKSYPSIKIENYNG---PAKIVVTLVT   58
_XP_048248211.1_     1  -IKIVEEPQQ-RGFRFRYECEGP-SHGGLQ----GEKSEKS--RKSYPSIKIENYNG---PAKIVVSLVT   58
_XP_046550036.1_     1  -IKIVEEPQQ-RGFRFRYECEGP-SHGGLQ----GEKSEKS--RKSYPSIKIENYNG---PAKIVVTLVT   58
_CAC5363017.1_       1  -IEIVEQPQS-RGFRFRYECEGP-SHGGLQ----GEKSEKY--RKTFPAIKIRNYNG---PSRVVVNLVT   58
_VDI06196.1_         1  -IEIVEQPQS-RGFRFRYECEGP-SHGGLQ----GEKSEKY--RKTFPAIKIRNYNG---PSRVVVNLVT   58
_AHI17304.1_         1  -IEIVEQPQS-RGFRFRYECEGP-SHGGLQ----GEKSEKY--RKTFPAIKIRNYNG---PSRVVVNLVT   58
_XP_052068168.1_     1  -IEIVEQPQS-RGFRFRYECEGP-SHGGLQ----GEKSEKY--RKTFPAIKIRNYNG---PSRVVVNLVT   58
_XP_052068167.1_     1  -IEIVEQPQS-RGFRFRYECEGP-SHGGLQ----GEKSEKY--RKTFPAIKIRNYNG---PSRVVVNLVT   58
_UZH84476.1_         1  -IEIVEQPQA-RGFRFRYECEGP-SHGGLQ----GEKSEKY--RRTYPSIRIRHYTG---AARIVVTLVT   58
_XP_033756865.1_     1  -IEIVEQPQP-RGFRFRYECEGP-SHGGLQ----GEKSEKY--RKTYPAIKIKNYNG---PARVVVTLVT   58
_AKC01669.1_         1  -IEIVEQPQP-RGFRFRYECEGP-SHGGLQ----GEKSEKY--RKTYPAIKIKNYNG---PARVVVTLVT   58
_KAJ8319099.1_       1  -IEISEQPQA-RGFRFRYECEGP-SHGGLQ----GESSEKY--KKTYPAIRIKNYVG---PARVVVSLVT   58
_XP_022318640.1_     1  -IEIVEQPQA-RGFRFRYECEGP-SHGGLQ----GEKSEKY--RKTFPSIRIRNYNG---AARIVVTLVT   58
_XP_011423502.2_     1  -IDIVEQPQA-RGFRFRYECEGP-SHGGLQ----GEKSEKY--RKTFPSIKIRNYNG---AARIVVTLVT   58
_XP_052690447.1_     1  -IDIVEQPQA-RGFRFRYECEGP-SHGGLQ----GEKSEKY--RKTFPSIKIRNYNG---AARIVVTLVT   58
_AEY99616.1_         1  -IAIVEQPQS-RGFRFRYECEGP-SHGGLQ----GERSEKY--RRSFPSIKIQNYNG---PAKIFVKLVT   58
_XP_052263328.1_     1  -LEIVEQPQQ-RGFRFRYECEGP-SHGGLQ----GASSERF--KKTVPTIRIKNYRG---LARVQVSLVA   58
```

```
_XP_052263327.1_     1  -LEIVEQPQQ-RGFRFRYECEGP-SHGGLQ----GASSERF--KKTVPTIRIKNYRG---LARVQVSLVA   58
_XP_052761314.1_     1  -LEIVEQPQQ-RGFRFRYECEGP-SHGGLQ----GASSERY--RKTVPTVRIKNYTG---AAKMTVSLVT   58
_XP_053392566.1_     1  -LEIIEQPQQ-RGFRFRYECEGP-SHGGLQ----GASSERH--RKTVPTVKIRNYNG---PARVVVSLVT   58
_KAI0238513.1_       1  -LEIIEQPKM-RGYRFRYKCEGD-SHGGLQ----GEKSNPG--HKSYPSVKLSNYQG---PARITVSLVT   58
_XP_029638652.1_     1  --VFIEQPQS-RGFRFRYKCEGP-SHGGLQ----GAHSQKS--KRTYPTVQILNYKG---PALFVVSLVT   57
_AUR53646.1_         1  --VFIEQPQS-RGFRFRYKCEGP-SHGGLQ----GAHSQKS--KRTYPTVQILNYKG---PALFVVSLVT   57
_XP_036360935.1_     1  --VFIEQPQS-RGFRFRYKCEGP-SHGGLQ----GAHSQKS--KRTYPTVQILNYKG---PALFVVSLVT   57
_XP_036360934.1_     1  --VFIEQPQS-RGFRFRYKCEGP-SHGGLQ----GAHSQKS--KRTYPTVQILNYKG---PALFVVSLVT   57
_XP_029638651.1_     1  --VFIEQPQS-RGFRFRYKCEGP-SHGGLQ----GAHSQKS--KRTYPTVQILNYKG---PALFVVSLVT   57
_XP_014776042.1_     1  ---FLEQPQS-RGFRFRYKCEGP-SHGGLQ----GTHSQKS--KRTYPTVQILNYKG---PALFVVSLVT   56
_XP_014776041.1_     1  ---FLEQPQS-RGFRFRYKCEGP-SHGGLQ----GTHSQKS--KRTYPTVQILNYKG---PALFVVSLVT   56
_XP_052825578.1_     1  ---FLEQPQS-RGFRFRYKCEGP-SHGGLQ----GTHSQKS--KRTYPTVQILNYKG---PALFVVSLVT   56
_XP_052825580.1_     1  --Y-------------------------------------------------------------------    1
_AEP33644.1_         1  -LKILEQPKS-RGFRFRYGCEGP-SHGGLP----GEHSQKG--KKSYPSVEIVNYKG---DARIVVSLVT   58
_PIK44524.1_         1  -LKITEQPKT-KGFRFRYNCEGL-SHGGLP----GEHNQKG--KRTYPSVEIYNYVG---EARIVVSLVT   58
_KAJ8022831.1_       1  -LVIREQPKS-RGFRFRYPCEGQ-SHGGLP----GEHSQRG--RRTYPSVEIVNYIG---EARIVVSLVT   58
_XP_030838637.1_     1  -------------------------------------------------------TG---LARIVVSLVT   12
_XP_038060093.1_     1  -LRILEQPKQ-RGFRFRYDCEGP-SHGGLP----GQNNQKG--KRTYPSVEILNYTG---LARIVVSLVT   58
_XP_022087493.1_     1  -LRILEQPKQ-RGFRFRYDCEGP-SHGGLP----GQNNQKG--KRTYPSVEIVNHVG---VARIVVSLVT   58
_XP_033643738.1_     1  -LRIIEQPKQ-RGFRFRYDCEGP-SHGGLP----GQHSQRG--KKTFPSVEILNYTG---PARIVVSLVT   58
_XP_033100082.1_     1  -LRIIEQPKQ-YGFRFRYECEGK-SHGGIP----GMSNERG--KKTYPSCEIVNYKG---LARIVVSLVT   58
_CCC55418.2_         1  ------------------------SHGGLP----CQNSQRG--KRSFPAVEICKYKG---PARIVVSLVT   37
_XP_054773081.1_     1  -LKILEQPRQ-RGFRFRYGCEGP-SHGGLP----GHNSQRG--KRSFPSVEICNYKG---PARIVVSLVT   58
_XP_030834883.1_     1  -LKILEQPRQ-RGFRFRYGCEGP-SHGGLP----GQNSQRG--KRSFPSVEICNYKG---SARIVVSLVT   58
_NP_999819.1_        1  -LKILEQPRQ-RGFRFRYGCEGP-SHGGLP----GQNSQRG--KRSFPSVEICNYKG---SARIVVSLVT   58
_XP_030834884.1_     1  -LKILEQPRQ-RGFRFRYGCEGP-SHGGLP----GQNSQRG--KRSFPSVEICNYKG---SARIVVSLVT   58
_XP_041485048.1_     1  -LKILEQPRQ-RGFRFRYGCEGP-SHGGLP----GHNSQRG--KRSFPSVEICNYKG---PARIVVSLVT   58
_QIA61835.1_         1  -LEIVEQPKA-RGFRFRYDCEGQ-SHGGLP----GENSEKNRKQKTYPTVRLIGYRG---RARVMVSLVS   60
_NP_001266238.1_     1  -LEIVEQPKS-RGFRFRYDCEGQ-SHGGLP----GENSEKNRRQKTYPTVHLKGYRG---RARVMVSLVT   60
_QHX41456.1_         1  -L-ILEQPKS-RGFRFRYDCEGQ-SHGGLP----GENSERNRKAKSYPTVQLLNYRG---RARIVVSLVT   59
_XP_022789210.1_     1  -MEILEQPKQ-RGFRFRYPCEGP-SHGGLP----GQYSEKG--KKSYPSVQLCNYQG---PARIVVSLVT   58
_XP_046859971.1_     1  -LEILEQPKQ-RGFRFRYSCEGP-SHGGLP----GENSVKG--KKTSPTVQIVNYHG---KARVEVSLVT   58
_XP_028414684.1_     1  -LEILEQPKQ-RGFRFRYSCEGP-SHGGLP----GERSAKG--RKSFPSVQIINYSG---KARIEVTLVT   58
_XP_028414852.1_     1  -LEILEQPKQ-RGFRFRYSCEGP-SHGGLP----GERSAKG--RKSFPSVQIINYSG---KARIEVTLVT   58
_ADQ57372.1_         1  -LEILEQPKP-RGFRFRYPCEGP-SHGGLP----GQFSTSK--SKSYPSVQVNNYQG---PCRIVVTLVT   58
_XP_048580668.1_     1  -LEILEQPKP-RGFRFRYPCEGP-SHGGLP----GQFSTSK--SKSYPSVQVNNYQG---PCRIVVTLVT   58
_EDO32923.1_         1  -LEILEQPKP-RGFRFRYPSEGP-SHGGLP----GQFSTSK--SKSYPSVQVNNYQG---PCRIVVTLVT   58
_ABU48530.1_         1  -LEILEQPKP-RGFRFRYPSEGP-SHGGLP----GQFSTSK--SKSYPSVQVNNYQG---PCRIVVTLVT   58
_ALG40993.1_         1  -LEILEQPKS-RGFRFRYPCEGP-SHGGLP----GEFSSQK--NKSYPSVQLNNYRG---PCRIVVSLVT   58
```

```
_ALG40994.1_        1  -LEILEQPKS-RGFRFRYPCEGP-SHGGLP----GEFSSQK--TKSYPSVQLNNYRG---PCRIVVSLVT  58
_XP_031565156.1_    1  -LEILEQPKS-RGFRFRYPCEGP-SHGGLP----GEFSTQK--TKSYPSVQLNNYRG---PCRIVVSLVT  58
_ALG40992.1_        1  -LEILEQPKS-RGFRFRYPCEGP-SHGGLP----GQFSSPK--NKSYPSVQLNNYHG---LCRIVVSLVT  58
_KXJ21435.1_        1  -LEILEQPKS-RGFRFRYPCEGP-SHGGLP----GEFSDSK--NKSYPSVQVCNYQG---PCRIVVSLVT  58
_XP_020916759.1_    1  -LEILEQPKS-RGFRFRYPCEGP-SHGGLP----GEFSDSK--NKSYPSVQVCNYQG---PCRIVVSLVT  58
_XP_015766406.1_    1  -LEILEQPKQ-RGFRFRYPCEGP-SHGGLP----GEYSEKG--KKSYPSVQLCNYKG---PARIVVSLVT  58
_UBR69922.1_        1  -LEILEQPKQ-RGFRFRYPCEGP-SHGGLP----GEYSEKG--KKSYPSVQLCNYKG---PARIVVSLVT  58
_XP_029186950.2_    1  -LEILEQPKQ-RGFRFRYPCEGP-SHGGLP----GEYSEKG--KKSYPSVQLCNYKG---PARIVVSLVT  58
_UCR60992.1_        1  -LEILEQPKQ-RGFRFRYPCEGP-SHGGLP----GEYSEKG--KKSYPSVQLCNYKG---PARIVVSLVT  58
_XP_027038010.1_    1  -MEILEQPKQ-RGFRFRYPCEGP-SHGGLP----GQYSEKG--KKSYPSVQLCNYQG---PARIVVSLVT  58
_NWX00991.1_        1  -LQIIEQPKQ-RGFRFRYVCEGP-SHGGLP----GASSEKN--KKSYPQVKICNYVG---PAKVIVQLVT  58
_NXS91232.1_        1  -LQIIEQPKQ-RGFRFRYVCEGP-SHGGLP----GASSEKN--KKSYPQVKICNYVG---PAKVIVQLVT  58
_NXG78778.1_        1  -LQIIEQPKQ-RGFRFRYVCEGP-SHGGLP----GASSEKN--KKSYPQVKICNYVG---PAKVIVQLVT  58
_XP_025964754.1_    1  -LQIIEQPKQ-RGFRFRYVCEGP-SHGGLP----GASSEKN--KKSYPQVKICNYVG---PAKVIVQLVT  58
_XP_014730709.1_    1  -LQIIEQPKQ-RGFRFRYVCEGP-SHGGLP----GASSEKN--KKSYPQVKICNYVG---PAKVIVQLVT  58
_NXW45082.1_        1  -LQIIEQPKQ-RGFRFRYVCEGP-SHGGLP----GASSEKN--KKSYPQVKICNYVG---PAKVIVQLVT  58
_XP_015479466.1_    1  -LQIIEQPKQ-RGFRFRYVCEGP-SHGGLP----GASSEKN--KKSYPQVKICNYVG---PAKVIVQLVT  58
_XP_009577719.1_    1  -LQIIEQPKQ-RGFRFRYVCEGP-SHGGLP----GASSEKN--KKSYPQVKICNYVG---PAKVIVQLVT  58
_NXC45563.1_        1  -LQIIEQPKQ-RGFRFRYVCEGP-SHGGLP----GASSEKN--KKSYPQVKICNYVG---PAKVIVQLVT  58
_XP_009883811.1_    1  -LQIIEQPKQ-RGFRFRYVCEGP-SHGGLP----GASSEKN--KKSYPQVKICNYVG---PAKVIVQLVT  58
_NXH56308.1_        1  -LQIIEQPKQ-RGFRFRYVCEGP-SHGGLP----GASSEKN--KKSYPQVKICNYVG---PAKVIVQLVT  58
_XP_023780422.1_    1  -LQIIEQPKQ-RGFRFRYVCEGP-SHGGLP----GASSEKN--KKSYPQVKICNYVG---PAKVIVQLVT  58
_XP_009271830.1_    1  -LQIIEQPKQ-RGFRFRYVCEGP-SHGGLP----GASSEKN--KKSYPQVKICNYVG---PAKVIVQLVT  58
_XP_009325314.1_    1  -LQIIEQPKQ-RGFRFRYVCEGP-SHGGLP----GASSEKN--KKSYPQVKICNYVG---PAKVIVQLVT  58
_NXM85820.1_        1  -LQIIEQPKQ-RGFRFRYVCEGP-SHGGLP----GASSEKN--KKSYPQVKICNYVG---PAKVIVQLVT  58
_NXI98149.1_        1  -LQIIEQPKQ-RGFRFRYVCEGP-SHGGLP----GASSEKN--KKSYPQVKICNYVG---PAKVIVQLVT  58
_XP_013807107.1_    1  -LQIIEQPKQ-RGFRFRYVCEGP-SHGGLP----GASSEKN--KKSYPQVKICNYVG---PAKVIVQLVT  58
_NWS89770.1_        1  -LQIIEQPKQ-RGFRFRYVCEGP-SHGGLP----GASSEKN--KKSYPQVKICNYVG---PAKVIVQLVT  58
_NXN94237.1_        1  -LQIIEQPKQ-RGFRFRYVCEGP-SHGGLP----GASSEKN--KKSYPQVKICNYVG---PAKVIVQLVT  58
_NXH67539.1_        1  -LQIIEQPKQ-RGFRFRYVCEGP-SHGGLP----GASSEKN--KKSYPQVKICNYVG---PAKVIVQLVT  58
_NXQ49570.1_        1  -LQIIEQPKQ-RGFRFRYVCEGP-SHGGLP----GASSEKN--KKSYPQVKICNYVG---PAKVIVQLVT  58
_NWT07854.1_        1  -LQIIEQPKQ-RGFRFRYVCEGP-SHGGLP----GASSEKN--KKSYPQVKICNYVG---PAKVIVQLVT  58
_XP_032915292.1_    1  -LQIIEQPKQ-RGFRFRYVCEGP-SHGGLP----GASSEKN--KKSYPQVKICNYVG---PAKVIVQLVT  58
_XP_014117319.1_    1  -LQIIEQPKQ-RGFRFRYVCEGP-SHGGLP----GASSEKN--KKSYPQVKICNYVG---PAKVIVQLVT  58
_XP_009816360.1_    1  -LQIIEQPKQ-RGFRFRYVCEGP-SHGGLP----GASSEKN--KKSYPQVKICNYVG---PAKVIVQLVT  58
_NXO31807.1_        1  -LQIIEQPKQ-RGFRFRYVCEGP-SHGGLP----GASSEKN--KKSYPQVKICNYVG---PAKVIVQLVT  58
_NWW07363.1_        1  -LQIIEQPKQ-RGFRFRYVCEGP-SHGGLP----GASSEKN--KKSYPQVKICNYVG---PAKVIVQLVT  58
_XP_054681626.1_    1  -LQIIEQPKQ-RGFRFRYVCEGP-SHGGLP----GASSEKN--KKSYPQVKICNYVG---PAKVIVQLVT  58
_XP_010286301.1_    1  -LQIIEQPKQ-RGFRFRYVCEGP-SHGGLP----GASSEKN--KKSYPQVKICNYVG---PAKVIVQLVT  58
```

NXC54686.1	1	-LQIIEQPKQ-RGFRFRYVCEGP-SHGGLP----GASSEKN--KKSYPQVKICNYVG---PAKVIVQLVT	58
_XP_054059788.1_	1	-LQIIEQPKQ-RGFRFRYVCEGP-SHGGLP----GASSEKN--KKSYPQVKICNYVG---PAKVIVQLVT	58
OPJ75671.1	1	-LQIIEQPKQ-RGFRFRYVCEGP-SHGGLP----GASSEKN--KKSYPQVKICNYVG---PAKVIVQLVT	58
_XP_010311473.1_	1	-LQIIEQPKQ-RGFRFRYVCEGP-SHGGLP----GASSEKN--KKSYPQVKICNYVG---PAKVIVQLVT	58
_XP_053921662.1_	1	-LQIIEQPKQ-RGFRFRYVCEGP-SHGGLP----GASSEKN--KKSYPQVKICNYVG---PAKVIVQLVT	58
NWQ94220.1	1	-LQIIEQPKQ-RGFRFRYVCEGP-SHGGLP----GASSEKN--KKSYPQVKICNYVG---PAKVIVQLVT	58
_XP_025934196.1_	1	-LQIIEQPKQ-RGFRFRYVCEGP-SHGGLP----GASSEKN--KKSYPQVKICNYVG---PAKVIVQLVT	58
NXS57318.1	1	-LQIIEQPKQ-RGFRFRYVCEGP-SHGGLP----GASSEKN--KKSYPQVKICNYVG---PAKVIVQLVT	58
NXF52248.1	1	-LQIIEQPKQ-RGFRFRYVCEGP-SHGGLP----GASSEKN--KKSYPQVKICNYVG---PAKVIVQLVT	58
OPJ75670.1	1	-LQIIEQPKQ-RGFRFRYVCEGP-SHGGLP----GASSEKN--KKSYPQVKICNYVG---PAKVIVQLVT	58
NXD98176.1	1	-LQIIEQPKQ-RGFRFRYVCEGP-SHGGLP----GASSEKN--KKSYPQVKICNYVG---PAKVIVQLVT	58
_XP_009949173.1_	1	-LQIIEQPKQ-RGFRFRYVCEGP-SHGGLP----GASSEKN--KKSYPQVKICNYVG---PAKVIVQLVT	58
NWH32710.1	1	-LQIIEQPKQ-RGFRFRYVCEGP-SHGGLP----GASSEKN--KKSYPQVKICNYVG---PAKVIVQLVT	58
_XP_010178978.1_	1	-LQIIEQPKQ-RGFRFRYVCEGP-SHGGLP----GASSEKN--KKSYPQVKICNYVG---PAKVIVQLVT	58
NXI82337.1	1	-LQIIEQPKQ-RGFRFRYVCEGP-SHGGLP----GASSEKN--KKSYPQVKICNYVG---PAKVIVQLVT	58
NXJ24754.1	1	-LQIIEQPKQ-RGFRFRYVCEGP-SHGGLP----GASSEKN--KKSYPQVKICNYVG---PAKVIVQLVT	58
NXM41500.1	1	-LQIIEQPKQ-RGFRFRYVCEGP-SHGGLP----GASSEKN--KKSYPQVKICNYVG---PAKVIVQLVT	58
NWW61888.1	1	-LQIIEQPKQ-RGFRFRYVCEGP-SHGGLP----GASSEKN--KKSYPQVKICNYVG---PAKVIVQLVT	58
NXE22655.1	1	-LQIIEQPKQ-RGFRFRYVCEGP-SHGGLP----GASSEKN--KKSYPQVKICNYVG---PAKVIVQLVT	58
NXF65753.1	1	-LQIIEQPKQ-RGFRFRYVCEGP-SHGGLP----GASSEKN--KKSYPQVKICNYVG---PAKVIVQLVT	58
NWS57053.1	1	-LQIIEQPKQ-RGFRFRYVCEGP-SHGGLP----GASSEKN--KKSYPQVKICNYVG---PAKVIVQLVT	58
NWU51843.1	1	-LQIIEQPKQ-RGFRFRYVCEGP-SHGGLP----GASSEKN--KKSYPQVKICNYVG---PAKVIVQLVT	58
NXB31773.1	1	-LQIIEQPKQ-RGFRFRYVCEGP-SHGGLP----GASSEKN--KKSYPQVKICNYVG---PAKVIVQLVT	58
NXE37412.1	1	-LQIIEQPKQ-RGFRFRYVCEGP-SHGGLP----GASSEKN--KKSYPQVKICNYVG---PAKVIVQLVT	58
NXM73977.1	1	-LQIIEQPKQ-RGFRFRYVCEGP-SHGGLP----GASSEKN--KKSYPQVKICNYVG---PAKVIVQLVT	58
NXK45099.1	1	-LQIIEQPKQ-RGFRFRYVCEGP-SHGGLP----GASSEKN--KKSYPQVKICNYVG---PAKVIVQLVT	58
NXT11309.1	1	-LQIIEQPKQ-RGFRFRYVCEGP-SHGGLP----GASSEKN--KKSYPQVKICNYVG---PAKVIVQLVT	58
_XP_039920334.1_	1	-LQIIEQPKQ-RGFRFRYVCEGP-SHGGLP----GASSEKN--KKSYPQVKICNYVG---PAKVIVQLVT	58
NXQ27105.1	1	-LQIIEQPKQ-RGFRFRYVCEGP-SHGGLP----GASSEKN--KKSYPQVKICNYVG---PAKVIVQLVT	58
NXN43964.1	1	-LQIIEQPKQ-RGFRFRYVCEGP-SHGGLP----GASSEKN--KKSYPQVKICNYVG---PAKVIVQLVT	58
_XP_025934203.1_	1	-LQIIEQPKQ-RGFRFRYVCEGP-SHGGLP----GASSEKN--KKSYPQVKICNYVG---PAKVIVQLVT	58
KFM03673.1	1	-LQIIEQPKQ-RGFRFRYVCEGP-SHGGLP----GASSEKN--KKSYPQVKICNYVG---PAKVIVQLVT	58
NXN08485.1	1	-LQIIEQPKQ-RGFRFRYVCEGP-SHGGLP----GASSEKN--KKSYPQVKICNYVG---PAKVIVQLVT	58
NXR70665.1	1	-LQIIEQPKQ-RGFRFRYVCEGP-SHGGLP----GASSEKN--KKSYPQVKICNYVG---PAKVIVQLVT	58
_XP_010165409.1_	1	-LQIIEQPKQ-RGFRFRYVCEGP-SHGGLP----GASSEKN--KKSYPQVKICNYVG---PAKVIVQLVT	58
_XP_010584501.1_	1	-LQIIEQPKQ-RGFRFRYVCEGP-SHGGLP----GASSEKN--KKSYPQVKICNYVG---PAKVIVQLVT	58
NWS41193.1	1	-LQIIEQPKQ-RGFRFRYVCEGP-SHGGLP----GASSEKN--KKSYPQVKICNYVG---PAKVIVQLVT	58
KFV51344.1	1	-LQIIEQPKQ-RGFRFRYVCEGP-SHGGLP----GASSEKN--KKSYPQVKICNYVG---PAKVIVQLVT	58
NXE09783.1	1	-LQIIEQPKQ-RGFRFRYVCEGP-SHGGLP----GASSEKN--KKSYPQVKICNYVG---PAKVIVQLVT	58

```
_NXI10557.1_        1  -LQIIEQPKQ-RGFRFRYVCEGP-SHGGLP----GASSEKN--KKSYPQVKICNYVG---PAKVIVQLVT   58
_NXJ43845.1_        1  -LQIIEQPKQ-RGFRFRYVCEGP-SHGGLP----GASSEKN--KKSYPQVKICNYVG---PAKVIVQLVT   58
_NWS05278.1_        1  -LQIIEQPKQ-RGFRFRYVCEGP-SHGGLP----GASSEKN--KKSYPQVKICNYVG---PAKVIVQLVT   58
_XP_027573674.2_    1  -LQIIEQPKQ-RGFRFRYVCEGP-SHGGLP----GASSEKN--KKSYPQVKICNYVG---PAKVIVQLVT   58
_NXQ94576.1_        1  -LQIIEQPKQ-RGFRFRYVCEGP-SHGGLP----GASSEKN--KKSYPQVKICNYVG---PAKVIVQLVT   58
_KAI1238512.1_      1  -LQIIEQPKQ-RGFRFRYVCEGP-SHGGLP----GASSEKN--KKSYPQVKICNYVG---PAKVIVQLVT   58
_NXK36380.1_        1  -LQIIEQPKQ-RGFRFRYVCEGP-SHGGLP----GASSEKN--KKSYPQVKICNYVG---PAKVIVQLVT   58
_NWH51107.1_        1  -LQIIEQPKQ-RGFRFRYVCEGP-SHGGLP----GASSEKN--KKSYPQVKICNYVG---PAKVIVQLVT   58
_NXW80580.1_        1  -LQIIEQPKQ-RGFRFRYVCEGP-SHGGLP----GASSEKN--KKSYPQVKICNYVG---PAKVIVQLVT   58
_NXY08181.1_        1  -LQIIEQPKQ-RGFRFRYVCEGP-SHGGLP----GASSEKN--KKSYPQVKICNYVG---PAKVIVQLVT   58
_NWU97338.1_        1  -LQIIEQPKQ-RGFRFRYVCEGP-SHGGLP----GASSEKN--KKSYPQVKICNYVG---PAKVIVQLVT   58
_XP_027491592.1_    1  -LQIIEQPKQ-RGFRFRYVCEGP-SHGGLP----GASSEKN--KKSYPQVKICNYVG---PAKVIVQLVT   58
_NXU08140.1_        1  -LQIIEQPKQ-RGFRFRYVCEGP-SHGGLP----GASSEKN--KKSYPQVKICNYVG---PAKVIVQLVT   58
_XP_029870423.1_    1  -LQIIEQPKQ-RGFRFRYVCEGP-SHGGLP----GASSEKN--KKSYPQVKICNYVG---PAKVIVQLVT   58
_XP_037992222.1_    1  -LQIIEQPKQ-RGFRFRYVCEGP-SHGGLP----GASSEKN--KKSYPQVKICNYVG---PAKVIVQLVT   58
_XP_019470357.1_    1  -LQIIEQPKQ-RGFRFRYVCEGP-SHGGLP----GASSEKN--KKSYPQVKICNYVG---PAKVIVQLVT   58
_XP_051646449.1_    1  -LQIIEQPKQ-RGFRFRYVCEGP-SHGGLP----GASSEKN--KKSYPQVKICNYVG---PAKVIVQLVT   58
_NXT54739.1_        1  -LQIIEQPKQ-RGFRFRYVCEGP-SHGGLP----GASSEKN--KKSYPQVKICNYVG---PAKVIVQLVT   58
_NXG03656.1_        1  -LQIIEQPKQ-RGFRFRYVCEGP-SHGGLP----GASSEKN--KKSYPQVKICNYVG---PAKVIVQLVT   58
_NWT86202.1_        1  -LQIIEQPKQ-RGFRFRYVCEGP-SHGGLP----GASSEKN--KKSYPQVKICNYVG---PAKVIVQLVT   58
_XP_009687644.1_    1  -LQIIEQPKQ-RGFRFRYVCEGP-SHGGLP----GASSEKN--KKSYPQVKICNYVG---PAKVIVQLVT   58
_NWQ73497.1_        1  -LQIIEQPKQ-RGFRFRYVCEGP-SHGGLP----GASSEKN--KKSYPQVKICNYVG---PAKVIVQLVT   58
_XP_050751358.1_    1  -LQIIEQPKQ-RGFRFRYVCEGP-SHGGLP----GASSEKN--KKSYPQVKICNYVG---PAKVIVQLVT   58
_KFO60593.1_        1  -LQIIEQPKQ-RGFRFRYVCEGP-SHGGLP----GASSEKN--KKSYPQVKICNYVG---PAKVIVQLVT   58
_XP_014133950.1_    1  -LQIIEQPKQ-RGFRFRYVCEGP-SHGGLP----GASSEKN--KKSYPQVKICNYVG---PAKVIVQLVT   58
_NXK26872.1_        1  -LQIIEQPKQ-RGFRFRYVCEGP-SHGGLP----GASSEKN--KKSYPQVKICNYVG---PAKVIVQLVT   58
_NWQ68199.1_        1  -LQIIEQPKQ-RGFRFRYVCEGP-SHGGLP----GASSEKN--KKSYPQVKICNYVG---PAKVIVQLVT   58
_XP_030348000.1_    1  -LQIIEQPKQ-RGFRFRYVCEGP-SHGGLP----GASSEKN--KKSYPQVKICNYVG---PAKVIVQLVT   58
_NWI78033.1_        1  -LQIIEQPKQ-RGFRFRYVCEGP-SHGGLP----GASSEKN--KKSYPMVKICNYVG---PAKVIVQLVT   58
_NXO71082.1_        1  -LQIIEQPKQ-RGFRFRYVCEGP-SHGGLP----GASSEKN--KKSYPQVKICNYVG---PAKVIVQLVT   58
_XP_040454521.1_    1  -LQIIEQPKQ-RGFRFRYVCEGP-SHGGLP----GASSEKN--KKSYPQVKICNYVG---PAKVIVQLVT   58
_NXQ12070.1_        1  -LQIIEQPKQ-RGFRFRYVCEGP-SHGGLP----GASSEKN--KKSYPQVKICNYVG---PAKVIVQLVT   58
_NXB56634.1_        1  -LQIIEQPKQ-RGFRFRYVCEGP-SHGGLP----GASSEKN--KKSYPQVKICNYVG---PAKVIVQLVT   58
_NXO78917.1_        1  -LQIIEQPKQ-RGFRFRYVCEGP-SHGGLP----GASSEKN--KKSYPQVKICNYVG---PAKVIVQLVT   58
_NXR80251.1_        1  -LQIIEQPKQ-RGFRFRYVCEGP-SHGGLP----GASSEKN--KKSYPQVKICNYVG---PAKVIVQLVT   58
_NXP31238.1_        1  -LQIIEQPKQ-RGFRFRYVCEGP-SHGGLP----GASSEKN--KKSYPQVKICNYVG---PAKVIVQLVT   58
_NXE73484.1_        1  -LQIIEQPKQ-RGFRFRYVCEGP-SHGGLP----GASSEKN--KKSYPQVKICNYVG---PAKVIVQLVT   58
_NXM25057.1_        1  -LQIIEQPKQ-RGFRFRYVCEGP-SHGGLP----GASSEKN--KKSYPQVKICNYVG---PAKVIVQLVT   58
_NXA89423.1_        1  -LQIIEQPKQ-RGFRFRYVCEGP-SHGGLP----GASSEKN--KKSYPQVKICNYVG---PAKVIVQLVT   58
```

```
_NXB19967.1_        1  -LQIIEQPKQ-RGFRFRYVCEGP-SHGGLP----GASSEKN--KKSYPQVKICNYVG---PAKVIVQLVT   58
_NXP82485.1_        1  -LQIIEQPKQ-RGFRFRYVCEGP-SHGGLP----GASSEKN--KKSYPQVKICNYVG---PAKVIVQLVT   58
_NWW54706.1_        1  -LQIIEQPKQ-RGFRFRYVCEGP-SHGGLP----GASSEKN--KKSYPQVKICNYVG---PAKVIVQLVT   58
_NXF96805.1_        1  -LQIIEQPKQ-RGFRFRYVCEGP-SHGGLP----GASSEKN--KKSYPQVKICNYVG---PAKVIVQLVT   58
_NXY35925.1_        1  -LQIIEQPKQ-RGFRFRYVCEGP-SHGGLP----GASSEKN--KKSYPQVKICNYVG---PAKVIVQLVT   58
_XP_037236065.1_    1  -LQIIEQPKQ-RGFRFRYVCEGP-SHGGLP----GASSEKN--KKSYPQVKICNYVG---PAKVIVQLVT   58
_NWT99824.1_        1  -LQIIEQPKQ-RGFRFRYVCEGP-SHGGLP----GASSEKN--KKSYPQVKICNYVG---PAKVIVQLVT   58
_XP_052631299.1_    1  -LQIIEQPKQ-RGFRFRYVCEGP-SHGGLP----GASSEKN--KKSYPQVKICNYVG---PAKVIVQLVT   58
_NXO12533.1_        1  -LQIIEQPKQ-RGFRFRYVCEGP-SHGGLP----GASSEKN--KKSYPQVKICNYVG---PAKVIVQLVT   58
_NWZ34533.1_        1  -LQIIEQPKQ-RGFRFRYVCEGP-SHGGLP----GASSEKN--KKSYPQVKICNYVG---PAKVIVQLVT   58
_NXA30360.1_        1  -LQIIEQPKQ-RGFRFRYVCEGP-SHGGLP----GASSEKN--KKSYPQVKICNYVG---PAKVIVQLVT   58
_NWU09820.1_        1  -LQIIEQPKQ-RGFRFRYVCEGP-SHGGLP----GASSEKN--KKSYPQVKICNYVG---PAKVIVQLVT   58
_NXO38268.1_        1  -LQIIEQPKQ-RGFRFRYVCEGP-SHGGLP----GASSEKN--KKSYPQVKICNYVG---PAKVIVQLVT   58
_NXY70755.1_        1  -LQIIEQPKQ-RGFRFRYVCEGP-SHGGLP----GASSEKN--KKSYPQVKICNYVG---PAKVIVQLVT   58
_RLW08972.1_        1  -LQIIEQPKQ-RGFRFRYVCEGP-SHGGLP----GASSEKN--KKSYPQVKICNYVG---PAKVIVQLVT   58
_XP_013155126.2_    1  -LQIIEQPKQ-RGFRFRYVCEGP-SHGGLP----GASSEKN--KKSYPQVKICNYVG---PAKVIVQLVT   58
_XP_053830927.1_    1  -LQIIEQPKQ-RGFRFRYVCEGP-SHGGLP----GASSEKN--KKSYPQVKICNYVG---PAKVIVQLVT   58
_XP_040454531.1_    1  -LQIIEQPKQ-RGFRFRYVCEGP-SHGGLP----GASSEKN--KKSYPQVKICNYVG---PAKVIVQLVT   58
_XP_049671253.1_    1  -LQIIEQPKQ-RGFRFRYVCEGP-SHGGLP----GASSEKN--KKSYPQVKICNYVG---PAKVIVQLVT   58
_XP_050829478.1_    1  -LQIIEQPKQ-RGFRFRYVCEGP-SHGGLP----GASSEKN--KKSYPQVKICNYVG---PAKVIVQLVT   58
_XP_029818864.1_    1  -LQIIEQPKQ-RGFRFRYVCEGP-SHGGLP----GASSEKN--KKSYPQVKICNYVG---PAKVIVQLVT   58
_NWV77110.1_        1  -LQIIEQPKQ-RGFRFRYVCEGP-SHGGLP----GASSEKN--KKSYPQVKICNYVG---PAKVIVQLVT   58
_NXW55588.1_        1  -LQIIEQPKQ-RGFRFRYVCEGP-SHGGLP----GASSEKN--KKSYPQVKICNYVG---PAKVIVQLVT   58
_NXK11347.1_        1  -LQIIEQPKQ-RGFRFRYVCEGP-SHGGLP----GASSEKN--KKSYPQVKICNYVG---PAKVIVQLVT   58
_XP_037236074.1_    1  -LQIIEQPKQ-RGFRFRYVCEGP-SHGGLP----GASSEKN--KKSYPQVKICNYVG---PAKVIVQLVT   58
_NXM20845.1_        1  -LQIIEQPKQ-RGFRFRYVCEGP-SHGGLP----GASSEKN--KKSYPQVKICNYVG---PAKVIVQLVT   58
_XP_009472960.1_    1  -LQIIEQPKQ-RGFRFRYVCEGP-SHGGLP----GASSEKN--KKSYPQVKICNYVG---PAKVIVQLVT   58
_NXP26913.1_        1  -LQIIEQPKQ-RGFRFRYVCEGP-SHGGLP----GASSEKN--KKSYPQVKICNYVG---PAKVIVQLVT   58
_NXB10880.1_        1  -LQIIEQPKQ-RGFRFRYVCEGP-SHGGLP----GASSEKN--KKSYPQVKICNYVG---PAKVIVQLVT   58
_NXQ83786.1_        1  -LQIIEQPKQ-RGFRFRYVCEGP-SHGGLP----GASSEKN--KKSYPQVKICNYVG---PAKVIVQLVT   58
_XP_041571848.1_    1  -LQIIEQPKQ-RGFRFRYVCEGP-SHGGLP----GASSEKN--KKSYPQVKICNYVG---PAKVIVQLVT   58
_NXS86984.1_        1  -LQIIEQPKQ-RGFRFRYVCEGP-SHGGLP----GASSEKN--KKSFPQVKICNYVG---PAKVIVQLVT   58
_XP_031966433.1_    1  -LQIIEQPKQ-RGFRFRYVCEGP-SHGGLP----GASSEKN--KKSYPQVKICNYVG---PAKVIVQLVT   58
_BAA02872.1_        1  -LQIIEQPKQ-RGFRFRYVCEGP-SHGGLP----GASSEKN--KKSYPQVKICNYVG---PAKVIVQLVT   58
_XP_051473650.1_    1  -LQIIEQPKQ-RGFRFRYVCEGP-SHGGLP----GASSEKN--KKSYPQVKICNYVG---PAKVIVQLVT   58
_XP_046771508.1_    1  -LQIIEQPKQ-RGFRFRYVCEGP-SHGGLP----GASSEKN--KKSYPQVKICNYVG---PAKVIVQLVT   58
_XP_033920424.1_    1  -LQIIEQPKQ-RGFRFRYVCEGP-SHGGLP----GASSEKN--KKSYPQVKICNYVG---PAKVIVQLVT   58
_NXK78645.1_        1  -LQIIEQPKQ-RGFRFRYVCEGP-SHGGLP----GASSEKN--KKSYPQVKICNYVG---PAKVIVQLVT   58
_XP_014795980.1_    1  -LQIIEQPKQ-RGFRFRYVCEGP-SHGGLP----GASSEKN--KKSYPQVKICNYVG---PAKVIVQLVT   58
```

NXN79327.1	1	-LQIIEQPKQ-RGFRFRYVCEGP-SHGGLP----GASSEKN--KKSYPQVKICNYVG---PAKVIVQLVT	58
_XP_041905595.1_	1	-LQIIEQPKQ-RGFRFRYVCEGP-SHGGLP----GASSEKN--KKSYPQVKICNYVG---PAKVIVQLVT	58
KQK83110.1	1	-LQIIEQPKQ-RGFRFRYVCEGP-SHGGLP----GASSEKN--KKSYPQVKICNYVG---PAKVIVQLVT	58
_NP_001383324.1_	1	-LQIIEQPKQ-RGFRFRYVCEGP-SHGGLP----GASSEKN--KKSYPQVKICNYVG---PAKVIVQLVT	58
NWX31294.1	1	-LQIIEQPKQ-RGFRFRYVCEGP-SHGGLP----GASSEKN--KKSYPQVKICNYVG---PAKVIVQLVT	58
_XP_050194007.1_	1	-LQIIEQPKQ-RGFRFRYVCEGP-SHGGLP----GASSEKN--KKSYPQVKICNYVG---PAKVIVQLVT	58
NXP05826.1	1	-LQIIEQPKQ-RGFRFRYVCEGP-SHGGLP----GASSEKN--KKSYPQVKICNYVG---PAKVIVQLVT	58
_XP_027759894.1_	1	-LQIIEQPKQ-RGFRFRYVCEGP-SHGGLP----GASSEKN--KKSYPQVKICNYVG---PAKVIVQLVT	58
_XP_009873772.1_	1	-LQIIEQPKQ-RGFRFRYVCEGP-SHGGLP----GASSEKN--KKSYPQVKICNYVG---PAKVIVQLVT	58
_XP_053796910.1_	1	-LQIIEQPKQ-RGFRFRYICEGP-SHGGLP----GASSEKN--KKSYPQVKICNYVG---PAKVIVQLVT	58
_XP_039406722.1_	1	-LQIIEQPKQ-RGFRFRYVCEGP-SHGGLP----GASSEKN--KKSYPQVKICNYVG---PAKVIVQLVT	58
NWU26823.1	1	-LQIIEQPKQ-RGFRFRYVCEGP-SHGGLP----GASSEKN--KKSYPQVKICNYVG---PAKVIVQLVT	58
_XP_051473648.1_	1	-LQIIEQPKQ-RGFRFRYVCEGP-SHGGLP----GASSEKN--KKSYPQVKICNYVG---PAKVIVQLVT	58
_Q04861.2_	1	-LQIIEQPKQ-RGFRFRYVCEGP-SHGGLP----GASSEKN--KKSYPQVKICNYVG---PAKVIVQLVT	58
NXL17972.1	1	-LQIIEQPKQ-RGFRFRYVCEGP-SHGGLP----GASSEKN--KKSYPQVKICNYVG---PAKVIVQLVT	58
_XP_054239251.1_	1	-LQIIEQPKQ-RGFRFRYVCEGP-SHGGLP----GASSEKN--KKSYPQVKICNYVG---PAKVIVQLVT	58
AAB58343.1	1	-LQIIEQPKQ-RGFRFRYVCEGP-SHGGLP----GASSEKN--KKSYPQVKICNYVG---PAKVIVQLVT	58
NXO56761.1	1	-LQIIEQPKQ-RGFRFRYVCEGP-SHGGLP----GASSEKN--KKSYPQVKICNYVG---PAKVIVQLVT	58
_XP_021251730.1_	1	-LQIIEQPKQ-RGFRFRYVCEGP-SHGGLP----GASSEKN--KKSYPQVKICNYVG---PAKVIVQLVT	58
_XP_015717578.1_	1	-LQIIEQPKQ-RGFRFRYVCEGP-SHGGLP----GASSEKN--KKSYPQVKICNYVG---PAKVIVQLVT	58
NWX21242.1	1	-LQIIEQPKQ-RGFRFRYVCEGP-SHGGLP----GASSEKN--KKSYPQVKICNYVG---PAKVIVQLVT	58
NXR39429.1	1	-LQIIEQPKQ-RGFRFRYVCEGP-SHGGLP----GASSEKN--KKSYPQVKICNYVG---PAKVIVQLVT	58
_XP_027532933.1_	1	-LQIIEQPKQ-RGFRFRYVCEGP-SHGGLP----GASSEKN--KKSYPQVKICNYVG---PAKVIVQLVT	58
NXT85173.1	1	-LQIIEQPKQ-RGFRFRYVCEGP-SHGGLP----GASSEKN--KKSYPQVKICNYVG---PAKVIVQLVT	58
_XP_030803683.1_	1	-LQIIEQPKQ-RGFRFRYVCEGP-SHGGLP----GASSEKN--KKSYPQVKICNYVG---PAKVIVQLVT	58
_XP_041340240.1_	1	-LQIIEQPKQ-RGFRFRYVCEGP-SHGGLP----GASSEKN--KKSYPQVKICNYVG---PAKVIVQLVT	58
NWT29590.1	1	-LQIIEQPKQ-RGFRFRYVCEGP-SHGGLP----GASSEKN--KKSYPQVKICNYVG---PAKVIVQLVT	58
NXH28317.1	1	-LQIIEQPKQ-RGFRFRYVCEGP-SHGGLP----GASSEKN--KKSYPQVKICNYVG---PAKVIVQLVT	58
NWS12389.1	1	-LQIIEQPKQ-RGFRFRYVCEGP-SHGGLP----GASSEKN--KKSYPQVKICNYVG---PAKVIVQLVT	58
NXF08296.1	1	-LQIIEQPKQ-RGFRFRYVCEGP-SHGGLP----GASSEKN--KKSYPQVKICNYVG---PAKVIVQLVT	58
NXP45252.1	1	-LQIIEQPKQ-RGFRFRYVCEGP-SHGGLP----GASSEKN--KKSYPQVKICNYVG---PAKVIVQLVT	58
_XP_048159384.1_	1	-LQIIEQPKQ-RGFRFRYVCEGP-SHGGLP----GASSEKN--KKSYPQVKICNYVG---PAKVIVQLVT	58
NXG13678.1	1	-LQIIEQPKQ-RGFRFRYVCEGP-SHGGLP----GASSEKN--KKSYPQVKICNYVG---PAKVIVQLVT	58
NXG81801.1	1	-----------RGFRFRYVCEGP-SHGGLP----GASSEKN--KKSYPQVKICNYVG---PAKVIVQLVT	49
NXM64740.1	1	-LQIIEQPKQ-RGFRFRYVCEGP-SHGGLP----GASSEKN--KKSYPQVKICNYVG---PAKVIVQLVT	58
NXJ10506.1	1	-LQIIEQPKQ-RGFRFRYVCEGP-SHGGLP----GASSEKN--KKSYPQVKICNYVG---PAKVIVQLVT	58
_XP_005418238.1_	1	-LQIIEQPKQ-RGFRFRYVCEGP-SHGGLP----GASSEKN--KKSYPQVKICNYVG---PAKVIVQLVT	58
_XP_009078875.1_	1	-LQIIEQPKQ-RGFRFRYVCEGP-SHGGLP----GASSEKN--KKSYPQVKICNYVG---PAKVIVQLVT	58
_XP_010204515.1_	1	-LQIIEQPKQ-RGFRFRYVCEGP-SHGGLP----GASSEKN--KKSYPQVKICNYVG---PAKVIVQLVT	58

```
_XP_008939135.1_    1  -LQIIEQPKQ-RGFRFRYVCEGP-SHGGLP----GASSEKN--KKSYPQVKICNYVG---PAKVIVQLVT   58
_NXA77498.1_        1  -LQIIEQPKQ-RGFRFRYVCEGP-SHGGLP----GASSEKN--KKSYPQVKICNYVG---PAKVIVQLVT   58
_XP_025906692.1_    1  -LQIIEQPKQ-RGFRFRYVCEGP-SHGGLP----GASSEKN--KKSYPQVKICNYVG---PAKVIVQLVT   58
_NWS73106.1_        1  -LQIIEQPKQ-RGFRFRYVCEGP-SHGGLP----GASSEKN--KKSYPQVKICNYVG---PAKVIVQLVT   58
_XP_042726775.1_    1  -LQIIEQPKQ-RGFRFRYVCEGP-SHGGLP----GASSEKN--KKSYPQVKICNYVG---PAKVIVQLVT   58
_XP_031464691.1_    1  -LQIIEQPKQ-RGFRFRYVCEGP-SHGGLP----GASSEKN--KKSYPQVKICNYVG---PAKVIVQLVT   58
_KFP33485.1_        1  -LQIIEQPKQ-RGFRFRYVCEGP-SHGGLP----GASSEKN--KKSYPQVKICNYVG---PAKVIVQLVT   58
_XP_054146082.1_    1  -LQIIEQPKQ-RGFRFRYVCEGP-SHGGLP----GASSEKN--KKSYPQVKICNYVG---PAKVIVQLVT   58
_XP_041280451.1_    1  -LQIIEQPKQ-RGFRFRYVCEGP-SHGGLP----GASSEKN--KKSYPQVKICNYVG---PAKVIVQLVT   58
_XP_021387126.1_    1  -LQIIEQPKQ-RGFRFRYVCEGP-SHGGLP----GASSEKN--KKSYPQVKICNYVG---PAKVIVQLVT   58
_NXH08922.1_        1  -LQIIEQPKQ-RGFRFRYVCEGP-SHGGLP----GASSEKN--KKSYPQVKICNYVG---PAKVIVQLVT   58
_NWV89860.1_        1  -LQIIEQPKQ-RGFRFRYVCEGP-SHGGLP----GASSEKN--KKSYPQVKICNYVG---PAKVIVQLVT   58
_NWS31855.1_        1  -LQIIEQPKQ-RGFRFRYVCEGP-SHGGLP----GASSEKN--KKSYPQVKICNYVG---PAKVIVQLVT   58
_NXC72334.1_        1  -LQIIEQPKQ-RGFRFRYVCEGP-SHGGLP----GASSEKN--KKSYPQVKICNYVG---PAKVIVQLVT   58
_KAJ7408913.1_      1  -LQIIEQPKQ-RGFRFRYVCEGP-SHGGLP----GASSEKN--KKSYPQVKICNYVG---PAKVIVQLVT   58
_KFV88798.1_        1  -LQIIEQPKQ-RGFRFRYVCEGP-SHGGLP----GASSEKN--KKSYPQVKICNYVG---PAKVIVQLVT   58
_NXF34295.1_        1  -LQIIEQPKQ-RGFRFRYVCEGP-SHGGLP----GASSEKN--KKSYPQVKICNYVG---PAKVIVQLVT   58
_NWR93222.1_        1  -LQIIEQPKQ-RGFRFRYVCEGP-SHGGLP----GASSEKN--KKSYPQVKICNYVG---PAKVIVQLVT   58
_NXR48213.1_        1  -LQIIEQPKQ-RGFRFRYVCEGP-SHGGLP----GASSEKN--KKSYPQVKICNYVG---PAKVIVQLVT   58
_NXY44121.1_        1  ---------Q-RGFRFRYVCEGP-SHGGLP----GASSEKN--KKSYPQVKICNYVG---PAKVIVQLVT   50
_XP_009935286.1_    1  -LQIIEQPKQ-RGFRFRYVCEGP-SHGGLP----GASSEKN--KKSYPQVKICNYVG---PAKVIVQLVT   58
_XP_026704017.1_    1  -LQIIEQPKQ-RGFRFRYVCEGP-SHGGLP----GASSEKN--KKSYPQVKICNYVG---PAKVIVQLVT   58
_XP_009513483.1_    1  -LQIIEQPKQ-RGFRFRYVCEGP-SHGGLP----GASSEKN--KKSYPQVKICNYVG---PAKVIVQLVT   58
_OXB77562.1_        1  -LQIIEQPKQ-RGFRFRYVCEGP-SHGGLP----GASSEKN--KKSYPQVKICNYVG---PAKVIVQLVT   58
_XP_026647812.1_    1  -LQIIEQPKQ-RGFRFRYVCEGP-SHGGLP----GASSEKN--KKSYPQVKICNYVG---PAKVIVQLVT   58
_NXC04840.1_        1  -LQIIEQPKQ-RGFRFRYVCEGP-SHGGLP----GASSEKN--KKSYPQVKICNYVG---PAKVIVQLVT   58
_XP_009990600.1_    1  -LQIIEQPKQ-RGFRFRYVCEGP-SHGGLP----GASSEKN--KKSFPQVKICNYVG---PAKVIVQLVT   58
_NXW31285.1_        1  ---------Q-RGFRFRYVCEGP-SHGGLP----GASSEKN--KKSYPQVKICNYVG---PAKVIVQLVT   50
_AAA49000.1_        1  -LQIIEQPKQ-RGFRFRYVCEGP-SHGGLP----GASSEKN--KKSYPQVKICNYVG---PAKVIVQLVT   58
_XP_032540773.1_    1  -LQIIEQPKQ-RGFRFRYVCEGP-SHGGLP----GASSEKN--KKSYPQVKICNYVG---PAKVIVQLVT   58
_XP_054489767.1_    1  -LQIIEQPKQ-RGFRFRYVCEGP-SHGGLP----GASSEKN--KKSYPQVKICNYVG---PAKVIVQLVT   58
_NXI38931.1_        1  -LEIIEQPKQ-RGFRFRYVCEGP-SHGGLP----GASSEKN--KKSYPQVKICNYVG---PAKVIVQLVT   58
_NWZ75946.1_        1  ---------Q-RGFRFRYVCEGP-SHGGLP----GASSEKN--KKSYPQVKICNYVG---PAKVIVQLVT   50
_TRZ15085.1_        1  -LQIIEQPKQ-RGFRFRYVCEGP-SHGGLP----GASSEKN--KKSYPQVKICNYVG---PAKVIVQLVT   58
_NWT43768.1_        1  ---------Q-RGFRFRYVCEGP-SHGGLP----GASSEKN--KKSYPQVKICNYVG---PAKVIVQLVT   50
_XP_042667699.1_    1  -LQIIEQPKQ-RGFRFRYVCEGP-SHGGLP----GASSEKN--KKSYPQVKICNYVG---PAKVIVQLVT   58
_KFW75731.1_        1  -LQIIEQPKQ-RGFRFRYVCEGP-SHGGLP----GASSEKN--KKSYPQVKICNYVG---PAKVIVQLVT   58
_XP_048796949.1_    1  -LQIIEQPKQ-RGFRFRYVCEGP-SHGGLP----GASSEKN--KKSYPQVKICNYVG---PAKVIVQLVT   58
_NWR08112.1_        1  -LQIIEQPKQ-RGFRFRYVCEGP-SHGGLP----GASSEKN--KKSYPQVKICNYVG---PAKVIVQLVT   58
```

```
_KAJ7414413.1_      1  -LQIIEQPKQ-RGFRFRYVCEGP-SHGGLP----GASSEKN--KKSYPQVKICNYVG---PAKVIVQLVT   58
_NWI28654.1_        1  -LQIIEQPKQ-RGFRFRYVCEGP-SHGGLP----GASSEKN--KKSYPQVKICNYVG---PAKVIVQLVT   58
_NXQ74308.1_        1  -LQIIEQPKQ-RGFRFRYVCEGP-SHGGLP----GASSEKN--KKSYPQVKICNYVG---PAKVIVQLVT   58
_NWR61490.1_        1  -LQIIEQPKQ-RGFRFRYVCEGP-SHGGLP----GASSEKN--KKSYPQVKICNYVG---PAKVIVQLVT   58
_NXW00255.1_        1  ---------Q-RGFRFRYVCEGP-SHGGLP----GASSEKN--KKSYPQVKICNYVG---PAKVIVQLVT   50
_XP_010131965.1_    1  -LQIIEQPKQ-RGFRFRYVCEGP-SHGGLP----GASSEKN--KKSYPQVKICNYVG---PAKVIVQLVT   58
_KAF1499115.1_      1  ---------Q-RGFRFRYVCEGP-SHGGLP----GASSEKN--KKSYPQVKICNYVG---PAKVIVQLVT   50
_XP_054018945.1_    1  -LQIIEQPKQ-RGFRFRYVCEGP-SHGGLP----GASSEKN--KKSYPQVKICNYVG---PAKVIVQLVT   58
_XP_052539264.1_    1  -LQIIEQPKQ-RGFRFRYVCEGP-SHGGLP----GASSEKN--KKSYPQVKICNYVG---PAKVIVQLVT   58
_NXC30330.1_        1  -LQIIEQPKQ-RGFRFRYVCEGP-SHGGLP----GASSEKN--KKSYPQVKICNYVG---PAKVIVQLVT   58
_KFR08581.1_        1  -LQIIEQPKQ-RGFRFRYVCEGP-SHGGLP----GASSEKN--KKSYPQVKICNYVG---PAKVIVQLVT   58
_XP_039557931.1_    1  -LQIIEQPKQ-RGFRFRYVCEGP-SHGGLP----GASSEKN--KKSYPQVKICNYVG---PAKVIVQLVT   58
_KAF1486784.1_      1  ---------Q-RGFRFRYVCEGP-SHGGLP----GASSEKN--KKSYPQVKICNYVG---PAKVIVQLVT   50
_OXB59608.1_        1  -LQIIEQPKQ-RGFRFRYVCEGP-SHGGLP----GASSEKN--KKSYPQVKICNYVG---PAKVIVQLVT   58
_XP_021138721.1_    1  -LQIIEQPKQ-RGFRFRYVCEGP-SHGGLP----GASSEKN--KKSYPQVKICNYVG---PAKVIVQLVT   58
_KAF1514432.1_      1  ---------Q-RGFRFRYVCEGP-SHGGLP----GASSEKN--KKSYPQVKICNYVG---PAKVIVQLVT   50
_NWV14170.1_        1  -LQIIEQPKQ-RGFRFRYVCEGP-SHGGLP----GASSEKN--KKSYPQVKICNYVG---PAKVIVQLVT   58
_KAF1548068.1_      1  ---------Q-RGFRFRYVCEGP-SHGGLP----GASSEKN--KKSYPQVKICNYVG---PAKVIVQLVT   50
_NXI51730.1_        1  -LQIIEQPKQ-RGFRFRYVCEGP-SHGGLP----GASSEKN--KKSYPQVKICNYVG---PAKVIVQLVT   58
_NXE50896.1_        1  ---------Q-RGFRFRYVCEGP-SHGGLP----GASSEKN--KKSYPQVKICNYVG---PAKVIVQLVT   50
_NXT41700.1_        1  ---------Q-RGFRFRYVCEGP-SHGGLP----GASSEKN--KKSYPQVKICNYVG---PAKVIVQLVT   50
_KAF1659387.1_      1  ---------Q-RGFRFRYVCEGP-SHGGLP----GASSEKN--KKSYPQVKICNYVG---PAKVIVQLVT   50
_NWR74867.1_        1  ---------Q-RGFRFRYVCEGP-SHGGLP----GASSEKN--KKSYPQVKICNYVG---PAKVIVQLVT   50
_NXV33343.1_        1  ---------Q-RGFRFRYVCEGP-SHGGLP----GASSEKN--KKSYPQVKICNYVG---PAKVIVQLVT   50
_NXW99502.1_        1  ---------Q-RGFRFRYVCEGP-SHGGLP----GASSEKN--KKSYPQVKICNYVG---PAKVIVQLVT   50
_NXV14161.1_        1  ---------Q-RGFRFRYVCEGP-SHGGLP----GASSEKN--KKSYPQVKICNYVG---PAKVIVQLVT   50
_NWX63354.1_        1  ---------Q-RGFRFRYICEGP-SHGGLP----GASSEKN--KKSYPQVKICNYVG---PAKVIVQLVT   50
_NXL58318.1_        1  ---------Q-RGFRFRYVCEGP-SHGGLP----GASSEKN--KKSYPQVKICNYVG---PAKVIVQLVT   50
_NWV41036.1_        1  ---------Q-RGFRFRYVCEGP-SHGGLP----GASSEKN--KKSYPQVKICNYVG---PAKVIVQLVT   50
_KAF1609011.1_      1  ---------Q-RGFRFRYVCEGP-SHGGLP----GASSEKN--KKSYPQVKICNYVG---PAKVIVQLVT   50
_NWT52190.1_        1  -LQIIEQPKQ-RGFRFRYVCEGP-SHGGLP----GASSEKN--KKSYPQVKICNYVG---PAKVIVQLVT   58
_NXO93770.1_        1  -LQIIEQPKQ-RGFRFRYVCEGP-SHGGLP----GASSEKN--KKSYPQVKICNYVG---PAKVIVQLVT   58
_NXI62681.1_        1  -LQIIEQPKQ-RGFRFRYVCEGP-SHGGLP----GASSEKN--KKSYPQVKICNYVG---PAKVIVQLVT   58
_NXH21058.1_        1  -LQIIEQPKQ-RGFRFRYVCEGP-SHGGLP----GASSEKN--KKSYPQVKICNYVG---PAKVIVQLVT   58
_KFV75676.1_        1  -LQIIEQPKQ-RGFRFRYVCEGP-SHGGLP----GASSEKN--KKSYPQVKICNYVG---PAKVIVQLVT   58
_KAF1435617.1_      1  ---------Q-RGFRFRYVCEGP-SHGGLP----GASSEKN--KKSYPQVKICNYVG---PAKVIVQLVT   50
_KAF1479350.1_      1  ---------Q-RGFRFRYVCEGP-SHGGLP----GASSEKN--KKSYPQVKICNYVG---PAKVIVQLVT   50
_NXW86014.1_        1  ---------Q-RGFRFRYVCEGP-SHGGLP----GASSEKN--KKSYPQVKICNYVG---PAKVIVQLVT   50
_KAF1483835.1_      1  ---------Q-RGFRFRYVCEGP-SHGGLP----GASSEKN--KKSYPQVKICNYVG---PAKVIVQLVT   50
```

NWW87838.1	1	-LQIIEQPKQ-RGFRFRYVCEGP-SHGGLP----GASSEKN--KKSYPQVKICNYVG---PAKVIVQLVT	58
NXF79309.1	1	-LQIIEQPKQ-RGFRFRYVCEGP-SHGGLP----GASSEKN--KKSYPQVKICNYVG---PAKVIVQLVT	58
_XP_030305668.1_	1	-LQIIEQPKQ-RGFRFRYVCEGP-SHGGLP----GASSEKN--KKSYPQVKICNYVG---PAKVIVQLVT	58
NXS27685.1	1	-LQIIEQPKQ-RGFRFRYVCEGP-SHGGLP----GASSEKN--KKSYPQVKICNYVG---PAKVIVQLVT	58
_XP_036237629.1_	1	-LQIIEQPKQ-RGFRFRYVCEGP-SHGGLP----GASSEKN--KKSYPQVKICNYVG---PAKVIVQLVT	58
NXM97765.1	1	-LQIIEQPKQ-RGFRFRYVCEGP-SHGGLP----GASSEKN--KKSYPQVKICNYVG---PAKVIVQLVT	58
KAF2988548.1	1	-LQIIEQPKQ-RGFRFRYVCEGP-SHGGLP----GASSEKN--KKSYPQVKICNYVG---PAKVIVQLVT	58
NWH87727.1	1	-LQIIEQPKQ-RGFRFRYVCEGP-SHGGLP----GASSEKN--KKSYPQVKICNYVG---PAKVIVQLVT	58
NXR25130.1	1	---------Q-RGFRFRYVCEGP-SHGGLP----GASSEKN--KKSYPQVKICNYVG---PAKVIVQLVT	50
NXV50350.1	1	---------Q-RGFRFRYVCEGP-SHGGLP----GASSEKN--KKSYPQVKICNYVG---PAKVIVQLVT	50
NWT72232.1	1	---------Q-RGFRFRYVCEGP-SHGGLP----GASSEKN--KKSYPQVKICNYVG---PAKVIVQLVT	50
KAF1435460.1	1	---------Q-RGFRFRYVCEGP-SHGGLP----GASSEKN--KKSYPQVKICNYVG---PAKVIVQLVT	50
KAF1424376.1	1	---------Q-RGFRFRYVCEGP-SHGGLP----GASSEKN--KKSYPQVKICNYVG---PAKVIVQLVT	50
NWX41614.1	1	---------Q-RGFRFRYVCEGP-SHGGLP----GASSEKN--KKSYPQVKICNYVG---PAKVIVQLVT	50
NXD28887.1	1	---------Q-RGFRFRYVCEGP-SHGGLP----GASSEKN--KKSYPQVKICNYVG---PAKVIVQLVT	50
NXY59218.1	1	---------Q-RGFRFRYVCEGP-SHGGLP----GASSEKN--KKSYPQVKICNYVG---PAKVIVQLVT	50
KAF1399315.1	1	---------Q-RGFRFRYVCEGP-SHGGLP----GASSEKN--KKSYPQVKICNYVG---PAKVIVQLVT	50
NXJ95672.1	1	---------Q-RGFRFRYVCEGP-SHGGLP----GASSEKN--KKSYPQVKICNYVG---PAKVIVQLVT	50
NWW19682.1	1	---------Q-RGFRFRYVCEGP-SHGGLP----GASSEKN--KKSYPQVKICNYVG---PAKVIVQLVT	50
NWU76879.1	1	---------Q-RGFRFRYVCEGP-SHGGLP----GASSEKN--KKSYPQVKICNYVG---PAKVIVQLVT	50
NWY75431.1	1	---------Q-RGFRFRYVCEGP-SHGGLP----GASSEKN--KKSYPQVKICNYVG---PAKVIVQLVT	50
NXX32566.1	1	---------Q-RGFRFRYVCEGP-SHGGLP----GASSEKN--KKSYPQVKICNYVG---PAKVIVQLVT	50
NXL48469.1	1	---------Q-RGFRFRYVCEGP-SHGGLP----GASSEKN--KKSYPQVKICNYVG---PAKVIVQLVT	50
NXN66426.1	1	---------Q-RGFRFRYVCEGP-SHGGLP----GASSEKN--KKSYPQVKICNYVG---PAKVIVQLVT	50
NXV94077.1	1	---------Q-RGFRFRYVCEGP-SHGGLP----GASSEKN--KKSYPQVKICNYVG---PAKVIVQLVT	50
KFO88230.1	1	-LQIIEQPKQ-RGFRFRYVCEGP-SHGGLP----GASSEKN--KKSYPQVKICNYVG---PAKVIVQLVT	58
NXD82005.1	1	-LQIIEQPKQ-RGFRFRYVCEGP-SHGGLP----GASSEKN--KKSYPQVKICNYVG---PAKVIVQLVT	58
NWY36867.1	1	---------Q-RGFRFRYVCEGP-SHGGLP----GASSEKN--KKSYPQVKICNYVG---PAKVIVQLVT	50
NWZ53691.1	1	---------Q-RGFRFRYVCEGP-SHGGLP----GASSEKN--KKSYPQVKICNYVG---PAKVIVQLVT	50
NXE95961.1	1	---------Q-RGFRFRYVCEGP-SHGGLP----GASSEKN--KKSYPQVKICNYVG---PAKVIVQLVT	50
NXB76170.1	1	---------Q-RGFRFRYVCEGP-SHGGLP----GASSEKN--KKSYPQVKICNYVG---PAKVIVQLVT	50
NWR51229.1	1	---------Q-RGFRFRYVCEGP-SHGGLP----GASSEKN--KKSYPQVKICNYVG---PAKVIVQLVT	50
NXB96832.1	1	---------Q-RGFRFRYICEGP-SHGGLP----GASSEKN--KKSYPQVKICNYVG---PAKVIVQLVT	50
NXW19741.1	1	---------Q-RGFRFRYVCEGP-SHGGLP----GASSEKN--KKSYPQVKICNYVG---PAKVIVQLVT	50
NWV61316.1	1	---------Q-RGFRFRYVCEGP-SHGGLP----GASSEKN--KKSYPQVKICNYVG---PAKVIVQLVT	50
NXD68485.1	1	---------Q-RGFRFRYVCEGP-SHGGLP----GASSEKN--KKSYPQVKICNYVG---PAKVIVQLVT	50
_XP_038033784.1_	1	-LQIIEQPKQ-RGFRFRYVCEGP-SHGGLP----GASSEKN--KKSFPQVKICNYVG---PAKVIVQLVT	58
_XP_009918158.1_	1	-LQIIEQPKQ-RGFRFRYVCEGP-SHGGLP----GASSEKN--KKSYPQVKICNYVG---PAKVIVQLVT	58
NWI62302.1	1	---------Q-RGFRFRYVCEGP-SHGGLP----GASSEKN--KKSYPQVKICNYVG---PAKVIVQLVT	50

```
_NXV82871.1_        1  ---------Q-RGFRFRYVCEGP-SHGGLP----GASSEKN--KKSYPQVKICNYVG---PAKVIVQLVT   50
_NXX21988.1_        1  ---------Q-RGFRFRYVCEGP-SHGGLP----GASSEKN--KKSYPQVKICNYVG---PAKVIVQLVT   50
_NWZ28840.1_        1  -LQIIEQPKQ-RGFRFRYVCEGP-SHGGLP----GASSEKN--KKSFPQVKICNYVG---PAKVIVQLVT   58
_NXA68287.1_        1  ---------Q-RGFRFRYVCEGP-SHGGLP----GASSEKN--KKSYPQVKICNYVG---PAKVIVQLVT   50
_NXL73547.1_        1  -----------RGFRFRYVCEGP-SHGGLP----GASSEKN--KKSYPQVKICNYVG---PAKVIVQLVT   49
_NXV08833.1_        1  ---------Q-RGFRFRYVCEGP-SHGGLP----GASSEKN--KKSYPQVKICNYVG---PAKVIVQLVT   50
_NWY20334.1_        1  ---------Q-RGFRFRYVCEGP-SHGGLP----GASSEKN--KKSYPQVKICNYVG---PAKVIVQLVT   50
_NXA13401.1_        1  ---------Q-RGFRFRYVCEGP-SHGGLP----GASSEKN--KKSYPQVKICNYVG---PAKVIVQLVT   50
_NXY15168.1_        1  ---------Q-RGFRFRYVCEGP-SHGGLP----GASSEKN--KKSYPQVKICNYVG---PAKVIVQLVT   50
_NXJ48973.1_        1  -LQIIEQPKQ-RGFRFRYVCEGP-SHGGLP----GASSEKN--KKSYPQVKICNYVG---PAKVIVQLVT   58
_KAI6070014.1_      1  -LQIIEQPKQ-RGFRFRYVCEGP-SHGGLP----GASSEKN--KKSFPQVKICNYVG---PAKVIVQLVT   58
_NXH91756.1_        1  -----------RGFRFRYVCEGP-SHGGLP----GASSEKN--KKSYPQVKICNYVG---PAKVIVQLVT   49
_NXL04686.1_        1  -----------RGFRFRYVCEGP-SHGGLP----GASSEKN--KKSYPQVKICNYVG---PAKVIVQLVT   49
_XP_040411521.1_    1  -LQIIEQPKQ-RGFRFRYVCEGP-SHGGLP----GASSEKN--KKSFPQVKICNYVG---PAKVIVQLVT   58
_XP_035395635.1_    1  -LQIIEQPKQ-RGFRFRYVCEGP-SHGGLP----GASSEKN--KKSFPQVKICNYVG---PAKVIVQLVT   58
_NXU37184.1_        1  ---------Q-RGFRFRYVCEGP-SHGGLP----GASSEKN--KKSYPQVKICNYVG---PAKVIVQLVT   50
_NWV21895.1_        1  ---------Q-RGFRFRYVCEGP-SHGGLP----GASSEKN--KKSYPQVKICNYVG---PAKVIVQLVT   50
_NXY81878.1_        1  -LQIIEQPKQ-RGFRFRYVCEGP-SHGGLP----GASSEKN--KKSYPQVKICNYVG---PAKVIVQLVT   58
_NXH41439.1_        1  -----------RGFRFRYVCEGP-SHGGLP----GASSEKN--KKSYPQVKICNYVG---PAKVIVQLVT   49
_NWW81943.1_        1  ---------Q-RGFRFRYVCEGP-SHGGLP----GASSEKN--KKSYPQVKICNYVG---PAKVIVQLVT   50
_NXJ61295.1_        1  ---------Q-RGFRFRYVCEGP-SHGGLP----GASSEKN--KKSYPQVKICNYVG---PAKVIVQLVT   50
_NXN34085.1_        1  ---------Q-RGFRFRYVCEGP-SHGGLP----GASSEKN--KKSYPQVKICNYVG---PAKVIVQLVT   50
_NXK62748.1_        1  --------KK-RGFRFRYVCEGK-KNGGLP----GASSEKN--KKTYPQVKICNYVG---PAKVIVQLVT   51
_NWH66460.1_        1  ---------Q-RGFRFRYVCEGP-SHGGLP----GASSEKN--KKSYPQVKICNYVG---PAKVIVQLVT   50
_NWS97842.1_        1  ---------Q-RGFRFRYVCEGP-SHGGLP----GASSEKN--KKSYPQVKICNYVG---PAKVIVQLVT   50
_XP_013051206.2_    1  -LQIIEQPKQ-RGFRFRYVCEGP-SHGGLP----GASSEKN--KKSFPQVKICNYVG---PAKVIVQLVT   58
_NWY58828.1_        1  ---------Q-RGFRFRYVCEGP-SHGGLP----GASSEKN--KKSYPQVKICNYVG---PAKVIVQLVT   50
_NWR27345.1_        1  ---------Q-RGFRFRYVCEGP-SHGGLP----GASSEKN--KKSYPQVKICNYVG---PAKVIVQLVT   50
_NXM04963.1_        1  -----------RGFRFRYVCEGP-SHGGLP----GASSEKN--KKSYPQVKICNYVG---PAKVIVQLVT   49
_NWZ13908.1_        1  ---------Q-RGFRFRYVCEGP-SHGGLP----GASSEKN--KKSYPQVKICNYVG---PAKVIVQLVT   50
_NWI56539.1_        1  ---------Q-RGFRFRYVCEGP-SHGGLP----GASSEKN--KKSYPQVKICNYVG---PAKVIVQLVT   50
_NXL35478.1_        1  ---------Q-RGFRFRYVCEGP-SHGGLP----GASSEKN--KKSYPQVKICNYVG---PAKVIVQLVT   50
_NWY90605.1_        1  ---------Q-RGFRFRYVCEGP-SHGGLP----GASSEKN--KKSYPQVKICNYVG---PAKVIVQLVT   50
_NXF23305.1_        1  ---------Q-RGFRFRYVCEGP-SHGGLP----GASSEKN--KKSYPQVKICNYVG---PAKVIVQLVT   50
_XP_035182229.1_    1  -LQIIEQPKQ-RGFRFRYVCEGP-SHGGLP----GASSEKN--KKSFPQVKICNYVG---PAKVIVQLVT   58
_EOB02688.1_        1  -LQIIEQPKQ-RGFRFRYVCEGP-SHGGLP----GASSEKN--KKSFPQVKICNYVG---PAKVIVQLVT   58
_NXP86430.1_        1  ---------Q-RGFRFRYVCEGP-SHGGLP----GASSEKN--KKSYPQVKICNYVG---PAKVIVQLVT   50
_NXK90683.1_        1  -LQIIEQPKQ-RGFRFRYVCEGP-SHGGLP----GASSEKN--KKSYPQVKICNYVG---PAKVIVQLVT   58
_NWU68452.1_        1  ---------Q-RGFRFRYVCEGP-SHGGLP----GASSEKN--KKSYPQVKICNYVG---PAKVIVQLVT   50
```

NXT19433.1	1	---------Q-RGFRFRYVCEGP-SHGGLP----GASSEKN--KKSYPQVKICNYVG---PAKVIVQLVT	50
NXV56948.1	1	---------Q-RGFRFRYVCEGP-SHGGLP----GASSEKN--KKSYPQVKICNYVG---PAKVIVQLVT	50
NXX85020.1	1	---------Q-RGFRFRYVCEGP-SHGGLP----GASSEKN--KKSYPQVKICNYVG---PAKVIVQLVT	50
NWY25110.1	1	---------Q-RGFRFRYVCEGP-SHGGLP----GASSEKN--KKSYPQVKICNYVG---PAKVIVQLVT	50
_XP_032041859.1_	1	-LQIIEQPKQ-RGFRFRYVCEGP-SHGGLP----GASSEKN--KKSFPQVKICNYVG---PAKVIVQLVT	58
NWZ68267.1	1	---------Q-RGFRFRYVCEGP-SHGGLP----GASSEKN--KKSYPQVKICNYVG---PAKVIVQLVT	50
NXG60352.1	1	---------Q-RGFRFRYVCEGP-SHGGLP----GASSEKN--KKSYPQVKICNYVG---PAKVIVQLVT	50
NXT59875.1	1	---------Q-RGFRFRYVCEGP-SHGGLP----GASSEKN--KKSYPQVKICNYVG---PAKVIVQLVT	50
NWY04604.1	1	---------Q-RGFRFRYVCEGP-SHGGLP----GASSEKN--KKSYPQVKICNYVG---PAKVIVQLVT	50
NXC95671.1	1	---------Q-RGFRFRYVCEGP-SHGGLP----GASSEKN--KKSYPQVKICNYVG---PAKVIVQLVT	50
NXD14679.1	1	---------Q-RGFRFRYVCEGP-SHGGLP----GASSEKN--KKSYPQVKICNYVG---PAKVIVQLVT	50
NWX87899.1	1	---------Q-RGFRFRYVCEGP-SHGGLP----GASSEKN--KKSYPQVKICNYVG---PAKVIVQLVT	50
NWZ96914.1	1	---------Q-RGFRFRYVCEGP-SHGGLP----GASSEKN--KKSYPQVKICNYVG---PAKVIVQLVT	50
KFP08036.1	1	-LQIIEQPKQ-RGFRFRYVCEGP-SHGGLP----GASSEKN--KKSYPQVKICNYVG---PAKVIVQLVT	58
NXJ81100.1	1	---------Q-RGFRFRYVCEGP-SHGGLP----GASSEKN--KKSYPQVKICNYVG---PAKVIVQLVT	50
KAF1419467.1	1	---------Q-RGFRFRYVCEGP-SHGGLP----GASSEKN--KKSYPQVKICNYVG---PAKVIVQLVT	50
NXA54431.1	1	---------Q-RGFRFRYVCEGP-SHGGLP----GASSEKN--KKSYPQVKICNYVG---PAKVIVQLVT	50
NXI22500.1	1	-----------RGFRFRYVCEGP-SHGGLP----GASSEKN--KKSYPQVKICNYVG---PAKVIVQLVT	49
_XP_030348001.1_	1	-LQIIEQPKQ-RGFRFRYVCEGP-SHGGLP----GASSEKN--KKSYPQVKICNYVG---PAKVIVQLVT	58
_XP_032041862.1_	1	-LQIIEQPKQ-RGFRFRYVCEGP-SHGGLP----GASSEKN--KKSFPQVKICNYVG---PAKVIVQLVT	58
_XP_054059792.1_	1	-LQIIEQPKQ-RGFRFRYVCEGP-SHGGLP----GASSEKN--KKSYPQVKICNYVG---PAKVIVQLVT	58
NWI98852.1	1	---------Q-RGFRFRYVCEGP-SHGGLP----GASSEKN--KKSYPQVKICNYVG---PAKVIVQLVT	50
_XP_040979578.1_	1	-LQIIEQPKQ-RGFRFRYVCEGP-SHGGLP----GASSEKN--KKSYPQVKICNYVG---PAKVIVQLVT	58
_XP_035182231.1_	1	-LQIIEQPKQ-RGFRFRYVCEGP-SHGGLP----GASSEKN--KKSFPQVKICNYVG---PAKVIVQLVT	58
NWI17161.1	1	---------Q-RGFRFRYVCEGP-SHGGLP----GASSEKN--KKSYPQVKICNYVG---PAKVIVQLVT	50
_XP_017677707.1_	1	-LQIIEQPKQ-RGFRFRYVCEGP-SHGGLP----GASSEKN--KKSYPQVKICNYVG---PAKVIVQLVT	58
_XP_031966434.1_	1	-LQIIEQPKQ-RGFRFRYVCEGP-SHGGLP----GASSEKN--KKSYPQVKICNYVG---PAKVIVQLVT	58
_XP_014795990.1_	1	-LQIIEQPKQ-RGFRFRYVCEGP-SHGGLP----GASSEKN--KKSYPQVKICNYVG---PAKVIVQLVT	58
_XP_041905596.1_	1	-LQIIEQPKQ-RGFRFRYVCEGP-SHGGLP----GASSEKN--KKSYPQVKICNYVG---PAKVIVQLVT	58
_XP_039406723.1_	1	-LQIIEQPKQ-RGFRFRYVCEGP-SHGGLP----GASSEKN--KKSYPQVKICNYVG---PAKVIVQLVT	58
_XP_048159386.1_	1	-LQIIEQPKQ-RGFRFRYVCEGP-SHGGLP----GASSEKN--KKSYPQVKICNYVG---PAKVIVQLVT	58
NXA36485.1	1	---------Q-RGFRFRYVCEGP-SHGGLP----GASSEKN--KKSYPQVKICNYVG---PAKVIVQLVT	50
_XP_010001479.1_	1	-LQIIEQPKQ-RGFRFRYVCEGP-SHGGLP----GASSEKN--KKSYPQVKICNYVG---PAKVIVQLVT	58
KFU93771.1	1	-LQIIEQPKQ-RGFRFRYVCEGP-SHGGLP----GASSEKN--KKSYPQVKICNYVG---PAKVIVQLVT	58
NXE65168.1	1	---------Q-RGFRFRYVCEGP-SHGGLP----GASSEKN--KKSYPQVKICNYVG---PAKVIVQLVT	50
KAF4794888.1	1	-LQIIEQPKQ-RGFRFRYVCEGP-SHGGLP----GASSEKN--KKSYPQVKICNYVG---PAKVIVQLVT	58
_XP_027532934.1_	1	-LQIIEQPKQ-RGFRFRYVCEGP-SHGGLP----GASSEKN--KKSYPQVKICNYVG---PAKVIVQLVT	58
_XP_046795876.1_	1	-LQIIEQPKQ-RGFRFRYVCEGP-SHGGLP----GASSEKN--KKSYPQVKICNYVG---PAKVIVQLVT	58
KFV96277.1	1	-LQIIEQPKQ-RGFRFRYVCEGP-SHGGLP----GASSEKN--KKSYPQVKICNYVG---PAKVIVQLVT	58

```
_XP_010157325.1_    1  -LQIIEQPKQ-RGFRFRYVCEGP-SHGGLP----GASSEKN--KKSYPQVKICNYVG---PAKVIVQLVT  58
_NWI02032.1_        1  -LQIIEQPKQ-RGFRFRYVCEGP-SHGGLP----GASSEKN--KKSYPQVKICNYVG---PAKVIVQLVT  58
_XP_035746225.1_    1  -LQIIEQPKQ-RGFRFRYVCEGP-SHGGLP----GASSEKN--KKSYPQVKICNYVG---PAKVIVQLVT  58
_NWH69936.1_        1  ---------Q-RGFRFRYVCEGP-SHGGLP----GASSEKN--KKSYPQVKICNYVG---PAKVIVQLVT  50
_NXH96653.1_        1  -----------RGFRFRYVCEGP-SHGGLP----GASSEKN--KKSYPQVKICNYVG---PAKVIVQLVT  49
_NXN58828.1_        1  -LQIIEQPKQ-RGFRFRYVCEGP-SHGGLP----GASSEKN--KKSYPQVKICNYVG---PAKVIVQLVT  58
_XP_010083670.1_    1  -LQIIEQPKQ-RGFRFRYVCEGP-SHGGLP----GASSEKN--KKSYPQVKICNYVG---PAKVIVQLVT  58
_RMC13788.1_        1  -LQIIEQPKQ-RGFRFRYVCEGP-SHGGLP----GASSEKN--KKSYPQVKICNYVG---PAKVIVQLVT  58
_XP_023801002.1_       ----------------------------------------------------------------------
_KAJ1206281.1_      1  -IEITEQPKQ-RGFRFRYVCEGP-SHGGLP----GASSERN--KKSFPQIKIHNYVG---HAKVVVQLVT  58
_XP_029452911.1_    1  -IQIMEQPKQ-RGFRFRYVCEGP-SHGGLP----GASSEKN--KKSYPQIKIHNYVG---HAKVIVQLVT  58
_XP_030046614.1_    1  -IQIVEQPKQ-RGFRFRYVCEGP-SHGGLP----GASSEKN--KKSYPQIKIHNYVG---HAKVIVQLVT  58
_XP_033791760.1_    1  -IQITEQPKQ-RGFRFRYVCEGP-SHGGLP----GASSEKN--KKSYPQIKIHNFVG---HAKVIVQLVT  58
_NXS74679.1_           ----------------------------------------------------------------------
_XP_019782049.1_       ----------------------------------------------------------------------
_XP_030700974.1_       ----------------------------------------------------------------------
_XP_026972817.1_       ----------------------------------------------------------------------
_XP_042107134.1_       ----------------------------------------------------------------------
_XP_016807420.1_       ----------------------------------------------------------------------
_XP_031520261.1_       ----------------------------------------------------------------------
_XP_025146162.3_       ----------------------------------------------------------------------
_MXQ81613.1_           ----------------------------------------------------------------------
_XP_014391951.1_       ----------------------------------------------------------------------
_KAF5926647.1_         ----------------------------------------------------------------------
_XP_014315807.1_       ----------------------------------------------------------------------
_XP_044243992.1_       ----------------------------------------------------------------------
_XP_015425475.1_       ----------------------------------------------------------------------
_XP_036196822.1_       ----------------------------------------------------------------------
_XP_036266201.1_       ----------------------------------------------------------------------
_XP_025729301.1_       ----------------------------------------------------------------------
_XP_051041401.1_       ----------------------------------------------------------------------
_XP_029330831.1_       ----------------------------------------------------------------------
_XP_021051020.1_       ----------------------------------------------------------------------
_XP_023493402.1_       ----------------------------------------------------------------------
_XP_021101132.1_       ----------------------------------------------------------------------
_BAC35117.1_           ----------------------------------------------------------------------
_XP_006732650.2_       ----------------------------------------------------------------------
_KAB0368408.1_      1  --------------------------------------------HQQPRECICNYVG---PAKVIVQLVT  23
_XP_030675033.1_    1  -----------------------------------------------PLQQICNYVG---PAKVIVQLVT  20
```

```
_XP_012291953.1_     1  --------------------------------------------------KICNYVG---PAKVIVQLVT   17
_XP_047422690.1_     1  --------------------------------------------------AICNYVG---PAKVIVQLVT   17
_XP_051675507.1_     1  -----------------------------------------------PLQQICKYIG---PAKVIVQLVT   20
_XP_025146160.1_     1  -----------------------------------------------PLQQICNYVG---PAKVIVQLVT   20
_KAB0345267.1_       1  ---------------------------------------------------ICNYVG---PAKVIVQLVT   16
_XP_020014165.1_     1  ------------------------------------------------NILICNYVG---PAKVIVQLVT   19
_XP_026917376.1_     1  --------------------------------------------------QICNYVG---PAKVIVQLVT   17
_XP_019271516.2_     1  --------------------------------------------------QICNYVG---PAKVIVQLVT   17
_XP_045327688.1_     1  --------------------------------------------------QICNYVG---PAKVIVQLVT   17
_XP_023108675.2_     1  --------------------------------------------------QICNYMG---PAKVIVQLVT   17
_XP_014391950.1_     1  --------------------------------------------------------G---PAKVIVQLVT   11
_XP_023614158.1_     1  --------------------------------------------------------G---PAKVIVQLVT   11
_XP_032256797.1_     1  -----------------------------------------------PLQQICNYVG---PAKVIVQLVT   20
_XP_015425474.1_     1  --------------------------------------------------------G---PAKVIVQLVT   11
_XP_027709164.1_     1  -------------------------------------------------LNICNYVG---PAKVIVQLVT   18
_KAG8519429.1_       1  -----------------------------------------------SSVQICNYVG---TAKVIVQLVT   20
_KAB1282111.1_       1  --------------------------------------------------KICNYVG---PAKVIVQLVT   17
_XP_016049280.1_        ----------------------------------------------------------------------
_OBS59044.1_            ----------------------------------------------------------------------
_KAI2535356.1_          ----------------------------------------------------------------------
_XP_054846407.1_     1  -LQIIEQPKQ-RGFRFRYVCEGP-SHGGLP----GASSEKN--KKSYPQVKICNYVG---PAKVIVQLVT   58
_XP_015261255.1_     1  -LQIVEQPKQ-RGFRFRYVCEGP-SHGGLP----GASSEKN--KKSYPQVKICNYVG---PAKVIVQLVT   58
_XP_028599456.1_     1  -LQIVEQPKQ-RGFRFRYVCEGP-SHGGLP----GASSEKN--KKSYPQVKICNYAG---PAKVIVQLVT   58
_QCQ80695.1_         1  -LQIVEQPKQ-RGFRFRYVCEGP-SHGGLP----GASSEKN--KKSYPQVKICNYAG---PAKVIVQLVT   58
_XP_048365177.1_     1  -LQIVEQPKQ-RGFRFRYVCEGP-SHGGLP----GASSEKN--KKSYPQVKIFNYVG---PAKVIVQLVT   58
_EGW14115.1_            ----------------------------------------------------------------------
_KAJ6668884.1_          ----------------------------------------------------------------------
_XP_053108974.1_     1  -IQIVEQPKQ-RGFRFRYVCEGP-SHGGLP----GASSEKN--KKSYPQVKICNYVG---PAKVIVQLVT   58
_XP_007429861.1_     1  -LQIVEQPKQ-RGFRFRYVCEGP-SHGGLP----GASSEKN--KKSYPQVKICNYAG---PAKVIVQLVT   58
_XP_047654926.1_        ----------------------------------------------------------------------
_XP_011884266.1_        ----------------------------------------------------------------------
_KAI2535355.1_          ----------------------------------------------------------------------
_PNJ84388.1_            ----------------------------------------------------------------------
_XP_038167493.1_        ----------------------------------------------------------------------
_ERE89661.1_            ----------------------------------------------------------------------
_XP_038167494.1_        ----------------------------------------------------------------------
_KAH0615883.1_       1  -LQIVEQPKQ-RGFRFRYVCEGP-SHGGLP----GASSEKN--KKSYPQVKICNYTG---TAKVIVQLVT   58
_XP_020661083.1_     1  -LQIVEQPKQ-RGFRFRYVCEGP-SHGGLP----GASSEKN--KKSYPQVKICNYTG---PAKVIVQLVT   58
_XP_053259916.1_     1  -LQIVEQPKQ-RGFRFRYVCEGP-SHGGLP----GASSEKN--KKSYPQVKICNYAG---PAKVIVQLVT   58
```

```
_XP_044284768.1_    1  -LHIVEQPKQ-RGFRFRYVCEGP-SHGGLP----GASSEKN--KKSYPQVKICNYTG---PAKVIVQLVT   58
_XP_034967560.1_    1  -LQIVEQPKQ-RGFRFRYVCEGP-SHGGLP----GASSEKN--KKSYPQVKICNYAG---PAKVIVQLVT   58
_XP_033016273.1_    1  -LQIVEQPKQ-RGFRFRYVCEGP-SHGGLP----GASSEKN--KKSYPQVKICNYAG---PAKVIVQLVT   58
_KAJ7327088.1_      1  -LQIVEQPKQ-RGFRFRYVCEGP-SHGGLP----GASSEKN--KKSYPQVKICNYTG---PAKVIVQLVT   58
_XP_044284772.1_    1  --------AQ-RGFRFRYVCEGP-SHGGLP----GASSEKN--KKSYPQVKICNYTG---PAKVIVQLVT   51
_XP_044284769.1_    1  ---------E-RGFRFRYVCEGP-SHGGLP----GASSEKN--KKSYPQVKICNYTG---PAKVIVQLVT   50
_XP_039206640.1_    1  -LQIVEQPKQ-RGFRFRYVCEGP-SHGGLP----GASSEKN--KKSYPQVKICNYVG---PAKVIVQLVT   58
_XP_042325082.1_    1  -LQIVEQPKQ-RGFRFRYVCEGP-SHGGLP----GASSEKN--KKSYPQVKICNYTG---PAKVIVQLVT   58
_XP_015683855.1_    1  -LQIVEQPKQ-RGFRFRYVCEGP-SHGGLP----GASSEKN--KKSYPQVKICNYVG---PAKVIVQLVT   58
_KAF7252349.1_      1  -LHIVEQPKQ-RGFRFRYVCEGP-SHGGLP----GASSEKN--KKSYPQVKICNYTG---PAKVIVQLVT   58
_XP_053259920.1_    1  --------------------------------------------------QICNYAG---PAKVIVQLVT   17
_XP_028599459.1_    1  --------------------------------------------------QICNYAG---PAKVIVQLVT   17
_XP_044284775.1_       ----------------------------------------------------------------------
_XP_039206641.1_       ----------------------------------------------------------------------
_XP_013807108.1_       ----------------------------------------------------------------------
_XP_042664115.1_       ----------------------------------------------------------------------
_XP_042664114.1_       ----------------------------------------------------------------------
_NXS19671.1_           ----------------------------------------------------------------------
_XP_030305671.1_       ----------------------------------------------------------------------
_PKU34609.1_           ----------------------------------------------------------------------
_KFP37264.1_        1  -------------------------------------------------LQICNYVG---PAKVIVQLVT   18
_NWX68872.1_        1  --------------------------------------------------QICNYVG---PAKVIVQLVT   17
_KFP53260.1_        1  -------------------------------------------------LQICNYVG---PAKVIVQLVT   18
_NXP61177.1_        1  --------------------------------------------------QICNYVG---PAKVIVQLVT   17
_XP_009482766.1_    1  ---------------------------------------------------ICNYVG---PAKVIVQLVT   16
_XP_010215381.1_    1  ---------------------------------------------------ICNYVG---PAKVIVQLVT   16
_NWI90635.1_        1  -------------------------------------------------LQICNYVG---PAKVIVQLVT   18
_NXU77174.1_        1  --------------------------------------------------QICNYVG---PAKVIVQLVT   17
_NWR18823.1_        1  -------------------------------------------------LQICNYVG---PAKVIVQLVT   18
_POI31681.1_        1  ---------------------------------------------------ICNYVG---PAKVIVQLVT   16
_NXR92200.1_        1  --------------------------------------------------QICNYVG---PAKVIVQLVT   17
_NXL85091.1_        1  -LQIIEQPKQVRVLHLETLQDRP-SHGGLP----GASSEKN--KKSYPQVKICNYVG---PAKVIVQLVT   59
_XP_005044951.1_    1  -LQIIEQPKQ-RGFRFRYVCEGP-SHGGLP----GASSEKN--KKSYPQVKICNYVG---PAKVIVQLVT   58
_NXC19638.1_        1  ---------Q-RGFRFRYVCEGP-SHGGLP----GASSEKN--KKSYPQVKVKNFHW---PSYTFTMCV-   49
_ETE71838.1_           ----------------------------------------------------------------------
_KAG8134450.1_      1  -LQIVEQPKQ-RGFRFRYVCEGP-SHGGLP----GASSEKN--KKSYPQVKICSYAG---PAKVIVQLVT   58
_XP_026528490.1_    1  -LQIVEQPKQ-RGFRFRYVCEGP-SHGGLP----GASSEKN--KKSYPQVKICNYAG---PAKVIVQLVT   58
_XP_044284773.1_    1  ---------Q-RGFRFRYVCEGP-SHGGLP----GASSEKN--KKSYPQVKICNYTG---PAKVIVQLVT   50
_XP_026554053.1_    1  -LQIVEQPKQ-RGFRFRYVCEGP-SHGGLP----GASSEKN--KKSYPQVKICNYAG---PAKVIVQLVT   58
```

```
_XP_032080378.1_     1  -LQIIEQPKQ-RGFRFRYVCEGP-SHGGLP----GASSEKN--KKSYPQVKICNYVG---PAKVIVQLVT   58
_XP_034298076.1_     1  -LQIVEQPKQ-RGFRFRYVCEGP-SHGGLP----GASSEKN--KKSYPQVKICNYAG---PAKVIVQLVT   58
_ABR18728.1_         1  ------------------------------------------------------YVG---PAKVIVQLVT   13
_XP_004459938.1_     1  -LQILEQPKQ-RGFRFRYVCEGP-SHGGLP----GASSEKN--KKSYPQVKICNYVG---PAKVIVQLVT   58
_XP_036619706.1_     1  -LQILEQPKQ-RGFRFRYVCEGP-SHGGLP----GASSEKN--KKSYPQVKICNYVG---PAKVIVQLVT   58
_XP_028932520.1_     1  -LQIIEQPKQ-RGFRFRYVCEGP-SHGGLP----GASSEKN--KKSYPQVKICNYVG---PAKVIVQLVT   58
_MBZ3886481.1_       1  -------PEQ-RGFRFRYVCEGP-SHGGLP----GASSEKN--KKSYPQVKICNYVG---PAKVIVQLVT   52
_XP_047574052.1_     1  ---------Q-RGFRFRYVCEGP-SHGGLP----GASSEKN--KKSYPQVKICNYVG---PAKVIVQLVT   50
_XP_032716757.1_     1  ---------Q-RGFRFRYVCEGP-SHGGLP----GASSEKN--KKSYPQVKICNYVG---PAKVIVQLVT   50
_EFB13115.1_         1  ------SPLQ-RGFRFRYVCEGP-SHGGLP----GASSEKN--KKSYPQVKICNYVG---PAKVIVQLVT   53
_XP_044923034.1_     1  ---------Q-RGFRFRYVCEGP-SHGGLP----GASSEKN--KKSYPQVKICNYVG---PAKVIVQLVT   50
_KFO25880.1_         1  -------PTP-RGFRFRYVCEGP-SHGGLP----GASSEKN--KKSYPQVKICNYVG---PAKVIVQLVT   52
_EPY78492.1_         1  --------EQ-RGFRFRYVCEGP-SHGGLP----GASSEKN--KKSYPQVKICNYVG---PAKVIVQLVT   51
_XP_006233422.1_     1  -LQILEQPKQ-RGFRFRYVCEGP-SHGGLP----GASSEKN--KKSYPQVKICNYVG---PAKVIVQLVT   58
_XP_038625183.1_     1  -LQIIEQPKQ-RGFRFRYVCEGP-SHGGLP----GASSEKN--KKSYPQVKICNYVG---PAKVIVQLVT   58
_XP_039769612.1_     1  -LQIIEQPKQ-RGFRFRYVCEGP-SHGGLP----GASSEKN--KKSYPQVKICNYVG---PAKVIVQLVT   58
_XP_026972816.1_     1  -LQILEQPKQ-RGFRFRYVCEGP-SHGGLP----GASSEKN--KKSYPQVKICNYVG---PAKVIVQLVT   58
_XP_030700972.1_     1  -LQILEQPKQ-RGFRFRYVCEGP-SHGGLP----GASSEKN--KKSYPQVKICNYVG---PAKVIVQLVT   58
_XP_019782048.1_     1  -LQILEQPKQ-RGFRFRYVCEGP-SHGGLP----GASSEKN--KKSYPQVKICNYVG---PAKVIVQLVT   58
_XP_044537564.1_     1  -LQILEQPKQ-RGFRFRYVCEGP-SHGGLP----GASSEKN--KKSYPQVKICNYVG---PAKIIVQLVT   58
_EDL12139.1_         1  -LQILEQPKQ-RGFRFRYVCEGP-SHGGLP----GASSEKN--KKSYPQVKICNYVG---PAKVIVQLVT   58
_EDL82274.1_         1  -LQILEQPKQ-RGFRFRYVCEGP-SHGGLP----GASSEKN--KKSYPQVKICNYVG---PAKVIVQLVT   58
_XP_014391954.1_     1  -LQILEQPKQ-RGFRFRYVCEGP-SHGGLP----GASSEKN--KKSYPQVKICNYMG---PAKVIVQLVT   58
_XP_014391955.1_     1  -LQILEQPKQ-RGFRFRYVCEGP-SHGGLP----GASSEKN--KKSYPQVKICNYMG---PAKVIVQLVT   58
_XP_033258015.1_     1  -LQILEQPKQ-RGFRFRYVCEGP-SHGGLP----GASSEKN--KKSYPQVKICNYVG---PAKVIVQLVT   58
_XP_030700975.1_     1  -LQILEQPKQ-RGFRFRYVCEGP-SHGGLP----GASSEKN--KKSYPQVKICNYVG---PAKVIVQLVT   58
_XP_014315810.1_     1  -LQILEQPKQ-RGFRFRYVCEGP-SHGGLP----GASSEKN--KKSYPQVKICNYMG---PAKVIVQLVT   58
_XP_014315809.1_     1  -LQILEQPKQ-RGFRFRYVCEGP-SHGGLP----GASSEKN--KKSYPQVKICNYMG---PAKVIVQLVT   58
_XP_019782050.1_     1  -LQILEQPKQ-RGFRFRYVCEGP-SHGGLP----GASSEKN--KKSYPQVKICNYVG---PAKVIVQLVT   58
_XP_026972818.1_     1  -LQILEQPKQ-RGFRFRYVCEGP-SHGGLP----GASSEKN--KKSYPQVKICNYVG---PAKVIVQLVT   58
_XP_015425477.1_     1  -LQILEQPKQ-RGFRFRYVCEGP-SHGGLP----GASSEKN--KKSYPQVKICNYMG---PAKVIVQLVT   58
_XP_036266227.1_     1  -LQILEQPKQ-RGFRFRYVCEGP-SHGGLP----GASSEKN--KKSYPQVKICNYMG---PAKVIVQLVT   58
_XP_036196846.1_     1  -LQILEQPKQ-RGFRFRYVCEGP-SHGGLP----GASSEKN--KKSYPQVKICNYMG---PAKVIVQLVT   58
_XP_027242102.1_     1  -LQILEQPKQ-RGFRFRYVCEGP-SHGGLP----GASSEKN--KKSYPQVKICNYVG---PAKVIVQLVT   58
_EDL82271.1_         1  -LQILEQPKQ-RGFRFRYVCEGP-SHGGLP----GASSEKN--KKSYPQVKICNYVG---PAKVIVQLVT   58
_XP_035951696.1_     1  -LQILEQPKQ-RGFRFRYVCEGP-SHGGLP----GASSEKN--KKSYPQVKICNYVG---PAKVIVQLVT   58
_XP_024309837.1_     1  -LQILEQPKQ-RGFRFRYVCEGP-SHGGLP----GASSEKN--KKSYPQVKICNYVG---PAKVIVQLVT   58
_CAB94757.1_         1  -LQILEQPKQ-RGFRFRYVCEGP-SHGGLP----GASSEKN--KKSYPQVKICNYVG---PAKVIVQLVT   58
_AAA36361.1_         1  -LQILEQPKQ-RGFRFRYVCEGP-SHGGLP----GASSEKN--KKSYPQVKICNYVG---PAKVIVQLVT   58
```

```
_NP_001369554.1_    1  -LQILEQPKQ-RGFRFRYVCEGP-SHGGLP----GASSEKN--KKSYPQVKICNYVG---PAKVIVQLVT   58
_XP_034814839.1_    1  -LQILEQPKQ-RGFRFRYVCEGP-SHGGLP----GASSEKN--KKSYPQVKICNYVG---PAKVIVQLVT   58
_BAF84139.1_        1  -LQILEQPKQ-RGFRFRYVCEGP-SHGGLP----GASSEKN--KKSYPQVKICNYVG---PAKVIVQLVT   58
_CAH18336.1_        1  -LQILEQPKQ-RGFRFRYVCEGP-SHGGLP----GASSEKN--KKSYPQVKICNYVG---PAKVIVQLVT   58
_XP_032033228.1_    1  -LQILEQPKQ-RGFRFRYVCEGP-SHGGLP----GASSEKN--KKSYPQVKICNYVG---PAKVIVQLVT   58
_XP_054340511.1_    1  -LQILEQPKQ-RGFRFRYVCEGP-SHGGLP----GASSEKN--KKSYPQVKICNYVG---PAKVIVQLVT   58
_XP_024101589.1_    1  -LQILEQPKQ-RGFRFRYVCEGP-SHGGLP----GASSEKN--KKSYPQVKICNYVG---PAKVIVQLVT   58
_XP_003257527.1_    1  -LQILEQPKQ-RGFRFRYVCEGP-SHGGLP----GASSEKN--KKSYPQVKICNYVG---PAKVIVQLVT   58
_XP_009205528.3_    1  -LQILEQPKQ-RGFRFRYVCEGP-SHGGLP----GASSEKN--KKSYPQVKICNYVG---PAKVIVQLVT   58
_XP_014994319.1_    1  -LQILEQPKQ-RGFRFRYVCEGP-SHGGLP----GASSEKN--KKSYPQVKICNYVG---PAKVIVQLVT   58
_XP_005555609.1_    1  -LQILEQPKQ-RGFRFRYVCEGP-SHGGLP----GASSEKN--KKSYPQVKICNYVG---PAKVIVQLVT   58
_XP_024650030.1_    1  -LQILEQPKQ-RGFRFRYVCEGP-SHGGLP----GASSEKN--KKSYPQVKICNYVG---PAKVIVQLVT   58
_XP_023075867.2_    1  -LQILEQPKQ-RGFRFRYVCEGP-SHGGLP----GASSEKN--KKSYPQVKICNYVG---PAKVIVQLVT   58
_XP_017732574.1_    1  -LQILEQPKQ-RGFRFRYVCEGP-SHGGLP----GASSEKN--KKSYPQVKICNYVG---PAKVIVQLVT   58
_XP_010361621.1_    1  -LQILEQPKQ-RGFRFRYVCEGP-SHGGLP----GASSEKN--KKSYPQVKICNYVG---PAKVIVQLVT   58
_XP_007997596.2_    1  -LQILEQPKQ-RGFRFRYVCEGP-SHGGLP----GASSEKN--KKSYPQVKICNYVG---PAKVIVQLVT   58
_EHH53872.1_        1  -LQILEQPKQ-RGFRFRYVCEGP-SHGGLP----GASSEKN--KKSYPQVKICNYVG---PAKVIVQLVT   58
_XP_005555612.2_    1  -LQILEQPKQ-RGFRFRYVCEGP-SHGGLP----GASSEKN--KKSYPQVKICNYVG---PAKVIVQLVT   58
_XP_033070771.1_    1  -LQILEQPKQ-RGFRFRYVCEGP-SHGGLP----GASSEKN--KKSYPQVKICNYVG---PAKVIVQLVT   58
_XP_003929561.1_    1  -LQILEQPKQ-RGFRFRYVCEGP-SHGGLP----GASSEKN--KKSYPQVKICNYVG---PAKVIVQLVT   58
_XP_017364149.1_    1  -LQILEQPKQ-RGFRFRYVCEGP-SHGGLP----GASSEKN--KKSYPQVKICNYVG---PAKVIVQLVT   58
_XP_012291952.1_    1  -LQILEQPKQ-RGFRFRYVCEGP-SHGGLP----GASSEKN--KKSYPQVKICNYVG---PAKVIVQLVT   58
_XP_002745591.1_    1  -LQILEQPKQ-RGFRFRYVCEGP-SHGGLP----GASSEKN--KKSYPQVKICNYVG---PAKVIVQLVT   58
_XP_032117501.1_    1  -LQILEQPKQ-RGFRFRYVCEGP-SHGGLP----GASSEKN--KKSYPQVKICNYVG---PAKVIVQLVT   58
_XP_011798604.1_    1  -LQILEQPKQ-RGFRFRYVCEGP-SHGGLP----GASSEKN--KKSYPQVKICNYVG---PAKVIVQLVT   58
_XP_050647861.1_    1  -LQILEQPKQ-RGFRFRYVCEGP-SHGGLP----GASSEKN--KKSYPQVKICNYVG---PAKVIVQLVT   58
_XP_050647862.1_    1  -LQILEQPKQ-RGFRFRYVCEGP-SHGGLP----GASSEKN--KKSYPQVKICNYVG---PAKVIVQLVT   58
_XP_016807418.1_    1  -LQILEQPKQ-RGFRFRYVCEGP-SHGGLP----GASSEKN--KKSYPQVKICNYVG---PAKVIVQLVT   58
_XP_008070272.2_    1  -LQILEQPKQ-RGFRFRYVCEGP-SHGGLP----GASSEKN--KKSYPQVKICNYVG---PAKVIVQLVT   58
_XP_016079084.1_    1  -LQILEQPKQ-RGFRFRYVCEGP-SHGGLP----GASSEKN--KKSYPQVKICNYVG---PAKVIVQLVT   58
_XP_054447653.1_    1  -LQILEQPKQ-RGFRFRYVCEGP-SHGGLP----GASSEKN--KKSYPQVKICNYVG---PAKVIVQLVT   58
_XP_014643400.1_    1  -LQILEQPKQ-RGFRFRYVCEGP-SHGGLP----GASSEKN--KKSYPQVKICNYVG---PAKVIVQLVT   58
_ABU63584.1_        1  -LQILEQPKQ-RGFRFRYVCEGP-SHGGLP----GASSEKN--KKSYPQVKICNYVG---PAKVIVQLVT   58
_XP_025146157.3_    1  -LQILEQPKQ-RGFRFRYVCEGP-SHGGLP----GASSEKN--KKSYPQVKICNYVG---PAKVIVQLVT   58
_XP_047654925.1_    1  -LQILEQPKQ-RGFRFRYVCEGP-SHGGLP----GASSEKN--KKSYPQVKICNYVG---PAKVIVQLVT   58
_DAA28856.1_        1  -LQILEQPKQ-RGFRFRYVCEGP-SHGGLP----GASSEKN--KKSYPQVKICNYVG---PAKVIVQLVT   58
_XP_025839601.1_    1  -LQILEQPKQ-RGFRFRYVCEGP-SHGGLP----GASSEKN--KKSYPQVKICNYVG---PAKVIVQLVT   58
_XP_005899878.1_    1  -LQILEQPKQ-RGFRFRYVCEGP-SHGGLP----GASSEKN--KKSYPQVKICNYVG---PAKVIVQLVT   58
_XP_043726831.1_    1  -LQILEQPKQ-RGFRFRYVCEGP-SHGGLP----GASSEKN--KKSYPQVKICNYVG---PAKVIVQLVT   58
```

```
_KAF6372160.1_      1  -LQILEQPKQ-RGFRFRYVCEGP-SHGGLP----GASSEKN--KKSYPQVKICNYVG---HAKVIVQLVT   58
_KAF4019754.1_      1  -LQILEQPKQ-RGFRFRYVCEGP-SHGGLP----GASSEKN--KKSYPQVKICNYVG---PAKVIVQLVT   58
_XP_027399763.1_    1  -LQILEQPKQ-RGFRFRYVCEGP-SHGGLP----GASSEKN--KKSYPQVKICNYVG---PAKVIVQLVT   58
_XP_008579050.1_    1  -LQILEQPKQ-RGFRFRYVCEGP-SHGGLP----GASSEKN--KKSYPQVKICNYVG---PAKVIVQLVT   58
_NP_001069877.1_    1  -LQILEQPKQ-RGFRFRYVCEGP-SHGGLP----GASSEKN--KKSYPQVKICNYVG---PAKVIVQLVT   58
_XP_032962399.1_    1  -LQILEQPKQ-RGFRFRYVCEGP-SHGGLP----GASSEKN--KKSYPQVKICNYVG---HAKVIVQLVT   58
_CAD7684753.1_      1  -LQILEQPKQ-RGFRFRYVCEGP-SHGGLP----GASSEKN--KKSYPQVKICNYVG---PAKVIVQLVT   58
_XP_041613896.1_    1  -LQILEQPKQ-RGFRFRYVCEGP-SHGGLP----GASSEKN--KKSYPQVKICNYVG---PAKVIVQLVT   58
_XP_019481431.1_    1  -LQILEQPKQ-RGFRFRYVCEGP-SHGGLP----GASSEKN--KKSYPQVKICNYVG---PAKVIVQLVT   58
_XP_019817941.1_    1  -LQILEQPKQ-RGFRFRYVCEGP-SHGGLP----GASSEKN--KKSYPQVKICNYVG---PAKVIVQLVT   58
_XP_016014547.2_    1  -LQILEQPKQ-RGFRFRYVCEGP-SHGGLP----GASSEKN--KKSYPQVKICNYVG---PAKVIVQLVT   58
_KAI4543101.1_      1  -LQILEQPKQ-RGFRFRYVCEGP-SHGGLP----GASSEKN--KKSYPQVKICNYVG---PAKVIVQLVT   58
_XP_043294197.1_    1  -LQILEQPKQ-RGFRFRYVCEGP-SHGGLP----GASSEKN--KKSYPQVKICNYVG---PAKVIVQLVT   58
_XP_010846585.1_    1  -LQILEQPKQ-RGFRFRYVCEGP-SHGGLP----GASSEKN--KKSYPQVKICNYVG---PAKVIVQLVT   58
_XP_017904753.1_    1  -LQILEQPKQ-RGFRFRYVCEGP-SHGGLP----GASSEKN--KKSYPQVKICNYVG---PAKVIVQLVT   58
_XP_052497597.1_    1  -LQILEQPKQ-RGFRFRYVCEGP-SHGGLP----GASSEKN--KKSYPQVKICNYVG---PAKVIVQLVT   58
_KAJ1061279.1_      1  -LQILEQPKQ-RGFRFRYVCEGP-SHGGLP----GASSEKN--KKSYPQVKICNYVG---PAKVIVQLVT   58
_KAF6372159.1_      1  -LQILEQPKQ-RGFRFRYVCEGP-SHGGLP----GASSEKN--KKSYPQVKICNYVG---HAKVIVQLVT   58
_XP_032962400.1_    1  -LQILEQPKQ-RGFRFRYVCEGP-SHGGLP----GASSEKN--KKSYPQVKICNYVG---HAKVIVQLVT   58
_XP_019481432.1_    1  -LQILEQPKQ-RGFRFRYVCEGP-SHGGLP----GASSEKN--KKSYPQVKICNYVG---PAKVIVQLVT   58
_XP_040093689.1_    1  -LQILEQPKQ-RGFRFRYVCEGP-SHGGLP----GASSEKN--KKSYPQVKICNYVG---PAKVIVQLVT   58
_XP_012034999.2_    1  -LQILEQPKQ-RGFRFRYVCEGP-SHGGLP----GASSEKN--KKSYPQVKICNYVG---PAKVIVQLVT   58
_XP_019594487.1_    1  -LQILEQPKQ-RGFRFRYVCEGP-SHGGLP----GASSEKN--KKSYPQVKICNYVG---HAKVIVQLVT   58
_XP_011359814.1_    1  -LQILEQPKQ-RGFRFRYVCEGP-SHGGLP----GASSEKN--KKSYPQVKICNYVG---PAKVIVQLVT   58
_XP_006918936.1_    1  -LQILEQPKQ-RGFRFRYVCEGP-SHGGLP----GASSEKN--KKSYPQVKICNYVG---PAKVIVQLVT   58
_XP_003796373.2_    1  -LQILEQPKQ-RGFRFRYVCEGP-SHGGLP----GASSEKN--KKSYPQVKICNYVG---PAKVIVQLVT   58
_VFV45675.1_        1  -LQILEQPKQ-RGFRFRYVCEGP-SHGGLP----GASSEKN--KKSYPQVKICNYVG---PAKVIVQLVT   58
_XP_014938375.1_    1  -LQILEQPKQ-RGFRFRYVCEGP-SHGGLP----GASSEKN--KKSYPQVKICNYVG---PAKVIVQLVT   58
_XP_038299711.1_    1  -LQILEQPKQ-RGFRFRYVCEGP-SHGGLP----GASSEKN--KKSYPQVKICNYVG---PAKVIVQLVT   58
_XP_030169211.1_    1  -LQILEQPKQ-RGFRFRYVCEGP-SHGGLP----GASSEKN--KKSYPQVKICNYVG---PAKVIVQLVT   58
_XP_019594488.1_    1  -LQILEQPKQ-RGFRFRYVCEGP-SHGGLP----GASSEKN--KKSYPQVKICNYVG---HAKVIVQLVT   58
_XP_046947838.1_    1  -LQILEQPKQ-RGFRFRYVCEGP-SHGGLP----GASSEKN--KKSYPQVKICNYVG---PAKVIVQLVT   58
_XP_043427583.1_    1  -LQILEQPKQ-RGFRFRYVCEGP-SHGGLP----GASSEKN--KKSYPQVKICNYVG---PAKVIVQLVT   58
_XP_026253918.1_    1  -LQILEQPKQ-RGFRFRYVCEGP-SHGGLP----GASSEKN--KKSYPQVKICNYVG---PAKVIVQLVT   58
_XP_007081014.2_    1  -LQILEQPKQ-RGFRFRYVCEGP-SHGGLP----GASSEKN--KKSYPQVKICNYVG---PAKVIVQLVT   58
_XP_005325802.1_    1  -LQILEQPKQ-RGFRFRYVCEGP-SHGGLP----GASSEKN--KKSYPQVKICNYVG---PAKVIVQLVT   58
_XP_025777165.1_    1  -LQILEQPKQ-RGFRFRYVCEGP-SHGGLP----GASSEKN--KKSYPQVKICNYVG---PAKVIVQLVT   58
_XP_053413718.1_    1  -LQILEQPKQ-RGFRFRYVCEGP-SHGGLP----GASSEKN--KKSYPQVKICNYVG---PAKVIVQLVT   58
_KAF6131715.1_      1  -LQILEQPKQ-RGFRFRYVCEGP-SHGGLP----GASSEKN--KKSYPQVKICNYVG---PAKVIVQLVT   58
```

```
_NP_001041697.1_     1  -LQILEQPKQ-RGFRFRYVCEGP-SHGGLP----GASSEKN--KKSYPQVKICNYVG---PAKVIVQLVT   58
_XP_039076751.1_     1  -LQILEQPKQ-RGFRFRYVCEGP-SHGGLP----GASSEKN--KKSYPQVKICNYVG---PAKVIVQLVT   58
_XP_047711530.1_     1  -LQILEQPKQ-RGFRFRYVCEGP-SHGGLP----GASSEKN--KKSYPQVKICNYVG---PAKVIVQLVT   58
_XP_045648536.1_     1  -LQILEQPKQ-RGFRFRYVCEGP-SHGGLP----GASSEKN--KKSYPQVKICNYVG---PAKVIVQLVT   58
_XP_036708419.1_     1  -LQILEQPKQ-RGFRFRYVCEGP-SHGGLP----GASSEKN--KKSYPQVKICNYVG---PAKVIVQLVT   58
_XP_027791168.1_     1  -LQILEQPKQ-RGFRFRYVCEGP-SHGGLP----GASSEKN--KKSYPQVKICNYVG---PAKVIVQLVT   58
_KAF7487162.1_       1  -LQILEQPKQ-RGFRFRYVCEGP-SHGGLP----GASSEKN--KKSYPQVKICNYVG---PAKVIVQLVT   58
_XP_004380306.2_     1  -LQILEQPKQ-RGFRFRYVCEGP-SHGGLP----GASSEKN--KKSYPQVKICNYVG---PAKVIVQLVT   58
_XP_048960883.1_     1  -LQILEQPKQ-RGFRFRYVCEGP-SHGGLP----GASSEKN--KKSYPQVKICNYVG---PAKVIVQLVT   58
_XP_007172981.1_     1  -LQILEQPKQ-RGFRFRYVCEGP-SHGGLP----GASSEKN--KKSYPQVKICNYVG---PAKVIVQLVT   58
_XP_015340088.1_     1  -LQILEQPKQ-RGFRFRYVCEGP-SHGGLP----GASSEKN--KKSYPQVKICNYVG---PAKVIVQLVT   58
_XP_049488091.1_     1  -LQILEQPKQ-RGFRFRYVCEGP-SHGGLP----GASSEKN--KKSYPQVKICNYVG---PAKVIVQLVT   58
_XP_046314717.1_     1  -LQILEQPKQ-RGFRFRYVCEGP-SHGGLP----GASSEKN--KKSYPQVKICNYVG---PAKVIVQLVT   58
_XP_045327678.1_     1  -LQILEQPKQ-RGFRFRYVCEGP-SHGGLP----GASSEKN--KKSYPQVKICNYVG---PAKVIVQLVT   58
_KAI5775569.1_       1  -LQILEQPKQ-RGFRFRYVCEGP-SHGGLP----GASSEKN--KKSYPQVKICNYVG---PAKVIVQLVT   58
_XP_044080328.1_     1  -LQILEQPKQ-RGFRFRYVCEGP-SHGGLP----GASSEKN--KKSYPQVKICNYVG---PAKVIVQLVT   58
_XP_008154865.2_     1  -LQILEQPKQ-RGFRFRYVCEGP-SHGGLP----GASSEKN--KKSYPQVKICNYMG---PAKVIVQLVT   58
_KAI5937862.1_       1  -LHILEQPKQ-RGFRFRYVCEGP-SHGGLP----GASSEKN--KKSYPQVKICNYVG---PAKVIVQLVT   58
_XP_031546207.1_     1  -LQILEQPKQ-RGFRFRYVCEGP-SHGGLP----GASSEKN--KKSYPQVKICNYVG---PAKVIVQLVT   58
_XP_053779051.1_     1  -LQILEQPKQ-RGFRFRYVCEGP-SHGGLP----GASSEKN--KKSYPQVKICNYVG---PAKVIVQLVT   58
_XP_007463874.1_     1  -LQILEQPKQ-RGFRFRYVCEGP-SHGGLP----GASSEKN--KKSYPQVKICNYVG---PAKVIVQLVT   58
_XP_022368452.1_     1  -LQILEQPKQ-RGFRFRYVCEGP-SHGGLP----GASSEKN--KKSYPQVKICNYVG---PAKVIVQLVT   58
_XP_036876060.1_     1  -LHILEQPKQ-RGFRFRYVCEGP-SHGGLP----GASSEKN--KKSYPQVKICNYVG---PAKVIVQLVT   58
_XP_029798483.1_     1  -LQILEQPKQ-RGFRFRYVCEGP-SHGGLP----GASSEKN--KKSYPQVKICNYVG---PAKVIVQLVT   58
_XP_036109130.1_     1  -LQILEQPKQ-RGFRFRYVCEGP-SHGGLP----GASSEKN--KKSYPQIKICNYSG---PAKVIVQLVT   58
_XP_010951892.1_     1  -LQILEQPKQ-RGFRFRYVCEGP-SHGGLP----GASSEKN--KKSYPQVKICNYVG---PAKVIVQLVT   58
_XP_031324505.1_     1  -LQILEQPKQ-RGFRFRYVCEGP-SHGGLP----GASSEKN--KKSYPQVKICNYVG---PAKVIVQLVT   58
_XP_023982362.1_     1  -LQILEQPKQ-RGFRFRYVCEGP-SHGGLP----GASSEKN--KKSYPQVKICNYVG---PAKVIVQLVT   58
_XP_028362571.1_     1  -LQILEQPKQ-RGFRFRYVCEGP-SHGGLP----GASSEKN--KKSYPQVKICNYVG---PAKVIVQLVT   58
_XP_032316661.1_     1  -LQILEQPKQ-RGFRFRYVCEGP-SHGGLP----GASSEKN--KKSYPQVKICNYVG---PAKVIVQLVT   58
_XP_051675506.1_     1  -LQILEQPKQ-RGFRFRYVCEGP-SHGGLP----GASSEKN--KKSYPQVKICKYIG---PAKVIVQLVT   58
_VCW95948.1_         1  -LQILEQPKQ-RGFRFRYVCEGP-SHGGLP----GASSEKN--KKSYPQVKICNYVG---PAKVIVQLVT   58
_XP_045676060.1_     1  -LQILEQPKQ-RGFRFRYVCEGP-SHGGLP----GASSEKN--KKSYPQVKICNYVG---PAKVIVQLVT   58
_XP_040481442.1_     1  -LQILEQPKQ-RGFRFRYVCEGP-SHGGLP----GASSEKN--KKSYPQVKICNYVG---PAKVIVQLVT   58
_XP_047422689.1_     1  -LQILEQPKQ-RGFRFRYVCEGP-SHGGLP----GASSEKN--KKSYPQVKICNYVG---PAKVIVQLVT   58
_XP_040333287.1_     1  -LQILEQPKQ-RGFRFRYVCEGP-SHGGLP----GASSEKN--KKSYPQVKICNYVG---PAKVIVQLVT   58
_XP_032256795.1_     1  -LQILEQPKQ-RGFRFRYVCEGP-SHGGLP----GASSEKN--KKSYPQVKICNYVG---PAKVIVQLVT   58
_XP_036892131.1_     1  -LQILEQPKQ-RGFRFRYVCEGP-SHGGLP----GASSEKN--KKSYPQVKICNYVG---PAKVIVQLVT   58
_XP_049741557.1_     1  -LQILEQPKQ-RGFRFRYVCEGP-SHGGLP----GASSEKN--KKSYPQVKICNYVG---PAKVIVQLVT   58
```

```
_XP_003410513.1_     1  -LQILEQPKQ-RGFRFRYVCEGP-SHGGLP----GASSEKN--KKSYPQVKICNYVG---PAKVIVQLVT   58
_XP_024591836.1_     1  -LQILEQPKQ-RGFRFRYVCEGP-SHGGLP----GASSEKN--KKSYPQVKICNYVG---PAKVIVQLVT   58
_XP_022438147.1_     1  -LQILEQPKQ-RGFRFRYVCEGP-SHGGLP----GASSEKN--KKSYPQVKICNYVG---PAKVIVQLVT   58
_KAF0884117.1_       1  -LQILEQPKQ-RGFRFRYVCEGP-SHGGLP----GASSEKN--KKSYPQVKICNYVG---PAKVIVQLVT   58
_XP_034886066.1_     1  -LQILEQPKQ-RGFRFRYVCEGP-SHGGLP----GASSEKN--KKSYPQVKICNYVG---PAKVIVQLVT   58
_NP_001003344.1_     1  -LQILEQPKQ-RGFRFRYVCEGP-SHGGLP----GASSEKN--KKSYPQVKICNYVG---PAKVIVQLVT   58
_XP_020757604.1_     1  -LQILEQPKQ-RGFRFRYVCEGP-SHGGLP----GASSEKN--KKSYPQVKICNYVG---PAKVIVQLVT   58
_XP_045736627.1_     1  -LQILEQPKQ-RGFRFRYVCEGP-SHGGLP----GASSEKN--KKSYPQVKICNYVG---PAKVIVQLVT   58
_XP_032488543.1_     1  -LQILEQPKQ-RGFRFRYVCEGP-SHGGLP----GASSEKN--KKSYPQVKICNYVG---PAKVIVQLVT   58
_XP_029090486.1_     1  -LQILEQPKQ-RGFRFRYVCEGP-SHGGLP----GASSEKN--KKSYPQVKICNYVG---PAKVIVQLVT   58
_XP_036735088.1_     1  -LQILEQPKQ-RGFRFRYVCEGP-SHGGLP----GASSEKN--KKSYPQVKICNYVG---PAKVIVQLVT   58
_XP_007951843.2_     1  -LQILEQPKQ-RGFRFRYVCEGP-SHGGLP----GASSEKN--KKSYPQVKICNYVG---PAKVIVQLVT   58
_XP_045392602.1_     1  -LQILEQPKQ-RGFRFRYVCEGP-SHGGLP----GASSEKN--KKSYPQVKICNYMG---PARVIVQLVT   58
_XP_021536093.1_     1  -LQILEQPKQ-RGFRFRYVCEGP-SHGGLP----GASSEKN--KKSYPQVKICNYVG---PAKVIVQLVT   58
_XP_027453602.1_     1  -LQILEQPKQ-RGFRFRYVCEGP-SHGGLP----GASSEKN--KKSYPQVKICNYVG---PAKVIVQLVT   58
_XP_023108674.2_     1  -LQILEQPKQ-RGFRFRYVCEGP-SHGGLP----GASSEKN--KKSYPQVKICNYMG---PAKVIVQLVT   58
_XP_045853066.1_     1  -LQILEQPKQ-RGFRFRYVCEGP-SHGGLP----GASSEKN--KKSYPQVKICNYVG---PAKVIVQLVT   58
_XP_036266160.1_     1  -LQILEQPKQ-RGFRFRYVCEGP-SHGGLP----GASSEKN--KKSYPQVKICNYMG---PAKVIVQLVT   58
_XP_037012773.2_     1  -LQILEQPKQ-RGFRFRYVCEGP-SHGGLP----GASSEKN--KKSYPQVKICNYVG---HAKVIVQLVT   58
_XP_004401911.1_     1  -LQILEQPKQ-RGFRFRYVCEGP-SHGGLP----GASSEKN--KKSYPQVKICNYVG---PAKVIVQLVT   58
_KAI5226086.1_       1  -LQILEQPKQ-RGFRFRYVCEGP-SHGGLP----GASSEKN--KKSYPQVKICNYVG---PAKVIVQLVT   58
_XP_032716751.1_     1  -LQILEQPKQ-RGFRFRYVCEGP-SHGGLP----GASSEKN--KKSYPQVKICNYVG---PAKVIVQLVT   58
_XP_032188916.1_     1  -LQILEQPKQ-RGFRFRYVCEGP-SHGGLP----GASSEKN--KKSYPQVKICNYVG---PAKVIVQLVT   58
_XP_027453603.1_     1  -LQILEQPKQ-RGFRFRYVCEGP-SHGGLP----GASSEKN--KKSYPQVKICNYVG---PAKVIVQLVT   58
_XP_049564805.1_     1  -LQILEQPKQ-RGFRFRYVCEGP-SHGGLP----GASSEKN--KKSYPQVKICNYVG---PAKVIVQLVT   58
_XP_004401912.1_     1  -LQILEQPKQ-RGFRFRYVCEGP-SHGGLP----GASSEKN--KKSYPQVKICNYVG---PAKVIVQLVT   58
_XP_047574051.1_     1  -LQILEQPKQ-RGFRFRYVCEGP-SHGGLP----GASSEKN--KKSYPQVKICNYVG---PAKVIVQLVT   58
_XP_032488539.1_     1  -LQILEQPKQ-RGFRFRYVCEGP-SHGGLP----GASSEKN--KKSYPQVKICNYVG---PAKVIVQLVT   58
_XP_012510833.1_     1  -LQILEQPKQ-RGFRFRYVCEGP-SHGGLP----GASSEKN--KKSYPQVKICNYMG---PAKVIVQLVT   58
_XP_006893175.1_     1  -LQILEQPKQ-RGFRFRYVCEGP-SHGGLP----GASSEKN--KKSYPQVKICNYVG---PAKVIVQLVT   58
_XP_037686273.1_     1  -LQILEQPKQ-RGFRFRYVCEGP-SHGGLP----GASSEKN--KKSYPQVKICNYVG---PAKVIVQLVT   58
_XP_046512032.1_     1  -LQILEQPKQ-RGFRFRYVCEGP-SHGGLP----GASSEKN--KKSYPQVKICNYVG---PAKVIVQLVT   58
_XP_005608646.3_     1  -LQILEQPKQ-RGFRFRYVCEGP-SHGGLP----GASSEKN--KKSYPQVKICNYVG---PAKVIVQLVT   58
_XP_036196809.1_     1  -LQILEQPKQ-RGFRFRYVCEGP-SHGGLP----GASSEKN--KKSYPQVKICNYMG---PAKVIVQLVT   58
_XP_034527338.1_     1  -LQILEQPKQ-RGFRFRYVCEGP-SHGGLP----GASSEKN--KKSYPQVKICNYVG---PAKVIVQLVT   58
_XP_048960884.1_     1  -LQILEQPKQ-RGFRFRYVCEGP-SHGGLP----GASSEKN--KKSYPQVKICNYVG---PAKVIVQLVT   58
_XP_036046011.1_     1  -LQILEQPKQ-RGFRFRYVCEGP-SHGGLP----GASSEKN--KKSYPQVKICNYVG---PAKVIVQLVT   58
_XP_006872788.1_     1  -LQILEQPKQ-RGFRFRYVCEGP-SHGGLP----GASSEKN--KKSYPQVKICNYMG---PAKVIVQLVT   58
_XP_044923033.1_     1  -LQILEQPKQ-RGFRFRYVCEGP-SHGGLP----GASSEKN--KKSYPQVKICNYVG---PAKVIVQLVT   58
```

KAB0398538.1	1	-LQILEQPKQ-RGFRFRYVCEGP-SHGGLP----GASSEKN--KKSYPQVKICNYVG---PAKVIVQLVT	58
TEA30114.1	1	-LQILEQPKQ-RGFRFRYVCEGP-SHGGLP----GASSEKN--KKSYPQVKICNYVG---PAKVIVQLVT	58
_XP_004703501.1_	1	-LQILEQPKQ-RGFRFRYVCEGP-SHGGLP----GASSEKN--KKSYPQVKICNYVG---PAKVIVQLVT	58
_XP_045371697.1_	1	-LQILEQPKQ-RGFRFRYVCEGP-SHGGLP----GASSEKN--KKSYPQVKICNYVG---PAKVIVQLVT	58
_XP_045371695.1_	1	-LQILEQPKQ-RGFRFRYVCEGP-SHGGLP----GASSEKN--KKSYPQVKICNYVG---PAKVIVQLVT	58
_XP_045371698.1_	1	-LQILEQPKQ-RGFRFRYVCEGP-SHGGLP----GASSEKN--KKSYPQVKICNYVG---PAKVIVQLVT	58
_XP_051021942.1_	1	-LQILEQPKQ-RGFRFRYVCEGP-SHGGLP----GASSEKN--KKSYPQVKICNYVG---PAKVIVQLVT	58
_XP_028616215.1_	1	-LQILEQPKQ-RGFRFRYVCEGP-SHGGLP----GASSEKN--KKSYPQVKICNYVG---PAKVIVQLVT	58
_XP_040822498.1_	1	-LQILEQPKQ-RGFRFRYVCEGP-SHGGLP----GASSEKN--KKSYPQVKICKYIG---PAKVIVQLVT	58
_XP_051041400.1_	1	-LQILEQPKQ-RGFRFRYVCEGP-SHGGLP----GASSEKN--KKSYPQVKICNYVG---PAKVIVQLVT	58
_XP_034356238.1_	1	-LQILEQPKQ-RGFRFRYVCEGP-SHGGLP----GASSEKN--KKSYPQVKICNYVG---PAKVIVQLVT	58
_XP_028713251.1_	1	-LQILEQPKQ-RGFRFRYVCEGP-SHGGLP----GASSEKN--KKSYPQVKICNYVG---PAKVIVQLVT	58
ERE89660.1	1	-LQILEQPKQ-RGFRFRYVCEGP-SHGGLP----GASSEKN--KKSYPQVKICNYVG---PAKVIVQLVT	58
_XP_052035128.1_	1	-LQILEQPKQ-RGFRFRYVCEGP-SHGGLP----GASSEKN--KKSYPQVKICNYVG---PAKVIVQLVT	58
_XP_006970398.1_	1	-LQILEQPKQ-RGFRFRYVCEGP-SHGGLP----GASSEKN--KKSYPQVKICNYVG---PAKVIVQLVT	58
_XP_042557701.1_	1	-LQILEQPKQ-RGFRFRYVCEGP-SHGGLP----GASSEKN--KKSYPQVKICNYVG---PAKVIVQLVT	58
_XP_031231473.1_	1	-LQILEQPKQ-RGFRFRYVCEGP-SHGGLP----GASSEKN--KKSYPQVKICNYVG---PAKVIVQLVT	58
_XP_012879285.1_	1	-LQILEQPKQ-RGFRFRYVCEGP-SHGGLP----GASSEKN--KKSYPQVKICNYVG---PAKVIVQLVT	58
_XP_003515839.4_	1	-LQILEQPKQ-RGFRFRYVCEGP-SHGGLP----GASSEKN--KKSYPQVKICNYVG---PAKVIVQLVT	58
_XP_012626422.1_	1	-LQILEQPKQ-RGFRFRYVCEGP-SHGGLP----GASSEKN--KKSYPQVKICNYMG---PAKVIVQLVT	58
_XP_048189722.1_	1	-LQILEQPKQ-RGFRFRYVCEGP-SHGGLP----GASSEKN--KKSYPQVKICNYMG---PAKVIVQLVT	58
_XP_052586690.1_	1	-LQILEQPKQ-RGFRFRYVCEGP-SHGGLP----GASSEKN--KKSYPQVKICNYVG---PAKVIVQLVT	58
_NP_032715.2_	1	-LQILEQPKQ-RGFRFRYVCEGP-SHGGLP----GASSEKN--KKSYPQVKICNYVG---PAKVIVQLVT	58
AAA40415.1	1	-LQILEQPKQ-RGFRFRYVCEGP-SHGGLP----GASSEKN--KKSYPQVKICNYVG---PAKVIVQLVT	58
_XP_049488087.1_	1	-LQILEQPKQ-RGFRFRYVCEGP-SHGGLP----GASSEKN--KKSYPQVKICNYVG---PAKVIVQLVT	58
_XP_044998939.1_	1	-LKILEQPKQ-RGFRFRYVCEGP-SHGGLP----GASSEKN--KKSYPQVKICNYVG---PAKVIVQLVT	58
_XP_049999310.1_	1	-LQILEQPKQ-RGFRFRYVCEGP-SHGGLP----GASSEKN--KKSYPQVKICNYVG---PAKVIVQLVT	58
_XP_040607681.1_	1	-LQILEQPKQ-RGFRFRYVCEGP-SHGGLP----GASSEKN--KKSYPQVKICNYVG---PAKVIVQLVT	58
_XP_045676057.1_	1	-LQILEQPKQ-RGFRFRYVCEGP-SHGGLP----GASSEKN--KKSYPQVKICNYVG---PAKVIVQLVT	58
_XP_032753167.1_	1	-LQILEQPKQ-RGFRFRYVCEGP-SHGGLP----GASSEKN--KKSYPQVKICNYVG---PAKVIVQLVT	58
_XP_028347494.1_	1	-LQILEQPKQ-RGFRFRYVCEGP-SHGGLP----GASSEKN--KKSYPQVKICNYVG---PAKVIVQLVT	58
_XP_031546209.1_	1	-LQILEQPKQ-RGFRFRYVCEGP-SHGGLP----GASSEKN--KKSYPQVKICNYVG---PAKVIVQLVT	58
_XP_010951893.1_	1	-LQILEQPKQ-RGFRFRYVCEGP-SHGGLP----GASSEKN--KKSYPQVKICNYVG---PAKVIVQLVT	58
_XP_005357381.1_	1	-LQILEQPKQ-RGFRFRYVCEGP-SHGGLP----GASSEKN--KKSYPQVKICNYVG---PAKVIVQLVT	58
_XP_031546210.1_	1	-LQILEQPKQ-RGFRFRYVCEGP-SHGGLP----GASSEKN--KKSYPQVKICNYVG---PAKVIVQLVT	58
_XP_031324516.1_	1	-LQILEQPKQ-RGFRFRYVCEGP-SHGGLP----GASSEKN--KKSYPQVKICNYVG---PAKVIVQLVT	58
_XP_031324521.1_	1	-LQILEQPKQ-RGFRFRYVCEGP-SHGGLP----GASSEKN--KKSYPQVKICNYVG---PAKVIVQLVT	58
_XP_041511026.1_	1	-LQILEQPKQ-RGFRFRYVCEGP-SHGGLP----GASSEKN--KKSYPQVKICNYVG---PAKVIVQLVT	58
_XP_032316666.1_	1	-LQILEQPKQ-RGFRFRYVCEGP-SHGGLP----GASSEKN--KKSYPQVKICNYVG---PAKVIVQLVT	58

```
_XP_032316670.1_    1  -LQILEQPKQ-RGFRFRYVCEGP-SHGGLP----GASSEKN--KKSYPQVKICNYVG---PAKVIVQLVT   58
_XP_021794361.1_    1  -LQILEQPKQ-RGFRFRYVCEGP-SHGGLP----GASSEKN--KKSYPQVKICNYVG---PAKVIVQLVT   58
_XP_021509291.1_    1  -LQILEQPKQ-RGFRFRYVCEGP-SHGGLP----GASSEKN--KKSYPQVKICNYVG---PAKVIVQLVT   58
_XP_021536094.1_    1  -LQILEQPKQ-RGFRFRYVCEGP-SHGGLP----GASSEKN--KKSYPQVKICNYVG---PAKVIVQLVT   58
_XP_013360849.1_    1  -LQILEQPKQ-RGFRFRYVCEGP-SHGGLP----GASSEKN--KKSYPQVKICNYVG---PAKVIVQLVT   58
_XP_038959153.1_    1  -LQILEQPKQ-RGFRFRYVCEGP-SHGGLP----GASSEKN--KKSYPQVKICNYVG---PAKVIVQLVT   58
_XP_048312756.1_    1  -LQILEQPKQ-RGFRFRYVCEGP-SHGGLP----GASSEKN--KKSYPQVKICNYVG---PAKVIVQLVT   58
_XP_044622324.1_    1  -LQILEQPKQ-RGFRFRYVCEGP-SHGGLP----GASSEKN--KKSYPQVKICNYVG---PAKVIVQLVT   58
_XP_044622325.1_    1  -LQILEQPKQ-RGFRFRYVCEGP-SHGGLP----GASSEKN--KKSYPQVKICNYVG---PAKVIVQLVT   58
_XP_008527175.1_    1  -LQILEQPKQ-RGFRFRYVCEGP-SHGGLP----GASSEKN--KKSYPQVKICNYVG---PAKVIVQLVT   58
_XP_003468075.1_    1  -LQILEQPKQ-RGFRFRYVCEGP-SHGGLP----GASSEKN--KKSYPQVKICNYVG---PAKVIVQLVT   58
_XP_027628732.1_    1  -LQILEQPKQ-RGFRFRYVCEGP-SHGGLP----GASSEKN--KKSYPQVKICNYVG---PAKVIVQLVT   58
_XP_046512030.1_    1  -LQILEQPKQ-RGFRFRYVCEGP-SHGGLP----GASSEKN--KKSYPQVKICNYVG---PAKVIVQLVT   58
_VFV45676.1_        1  -LQILEQPKQ-RGFRFRYVCEGP-SHGGLP----GASSEKN--KKSYPQVKICNYVG---PAKVIVQLVT   58
_XP_046512031.1_    1  -LQILEQPKQ-RGFRFRYVCEGP-SHGGLP----GASSEKN--KKSYPQVKICNYVG---PAKVIVQLVT   58
_XP_020843959.1_    1  -LQILEQPKQ-RGFRFRYVCEGP-SHGGLP----GASSEKN--KKSYPQVKICNYVG---PAKVIVQLVT   58
_XP_010639891.1_    1  -LQILEQPKQ-RGFRFRYVCEGP-SHGGLP----GASSEKN--KKSYPQVKICNYVG---PAKVIVQLVT   58
_XP_025146161.3_    1  -LQILEQPKQ-RGFRFRYVCEGP-SHGGLP----GASSEKN--KKSYPQVKICNYVG---PAKVIVQLVT   58
_XP_029416093.1_    1  -LQILEQPKQ-RGFRFRYVCEGP-SHGGLP----GASSEKN--KKSYPQVKICNYVG---PAKVIVQLVT   58
_XP_007495939.1_    1  -LQILEQPKQ-RGFRFRYVCEGP-SHGGLP----GASSEKN--KKSYPQVKICNYVG---PAKVIVQLVT   58
_XP_007495937.1_    1  -LQILEQPKQ-RGFRFRYVCEGP-SHGGLP----GASSEKN--KKSYPQVKICNYVG---PAKVIVQLVT   58
_XP_049564806.1_    1  -LQILEQPKQ-RGFRFRYVCEGP-SHGGLP----GASSEKN--KKSYPQVKICNYVG---PAKVIVQLVT   58
_XP_027709162.1_    1  -LQILEQPKQ-RGFRFRYVCEGP-SHGGLP----GASSEKN--KKSYPQVKICNYVG---PAKVIVQLVT   58
_XP_037365527.1_    1  -LQILEQPKQ-RGFRFRYVCEGP-SHGGLP----GASSEKN--KKSYPQVKICNYVG---AAKVIVQLVT   58
_XP_004594353.1_    1  -L--ISLPLQ-RGFRFRYVCEGP-SHGGLP----GASSEKN--KKSYPQVKICKYIG---PAKVIVQLVT   56
_XP_036619704.1_    1  -LQILEQPKQ-RGFRFRYVCEGP-SHGGLP----GASSEKN--KKSYPQVKICNYVG---PAKVIVQLVT   58
_XP_043828877.1_    1  -LQILEQPKQ-RGFRFRYVCEGP-SHGGLP----GASSEKN--KKSYPQVKICNYVG---PAKVIVQLVT   58
_XP_012407475.1_    1  -LQILEQPKQ-RGFRFRYVCEGP-SHGGLP----GASSEKN--KKSYPQVKICNYVG---PAKVIVQLVT   58
_XP_051822145.1_    1  -LQILEQPKQ-RGFRFRYVCEGP-SHGGLP----GASSEKN--KKSYPQVKICNYVG---PAKVIVQLVT   58
_XP_031798171.1_    1  -LQILEQPKQ-RGFRFRYVCEGP-SHGGLP----GASSEKN--KKSYPQVKICNYVG---PAKVIVQLVT   58
_XP_004686510.2_    1  -LQILEQPKQ-RGFRFRYVCEGP-SHGGLP----GASSEKN--KKSYPQVKICNYVG---AAKVIVQLVT   58
_XP_021509292.1_    1  -LQILEQPKQ-RGFRFRYVCEGP-SHGGLP----GASSEKN--KKSYPQVKICNYVG---PAKVIVQLVT   58
_XP_052035129.1_    1  -LQILEQPKQ-RGFRFRYVCEGP-SHGGLP----GASSEKN--KKSYPQVKICNYVG---PAKVIVQLVT   58
_XP_019065790.1_    1  -LQILEQPKQ-RGFRFRYVCEGP-SHGGLP----GASSEKN--KKSYPQVKICNYVG---PAKVIVQLVT   58
_XP_031231475.1_    1  -LQILEQPKQ-RGFRFRYVCEGP-SHGGLP----GASSEKN--KKSYPQVKICNYVG---PAKVIVQLVT   58
_XP_034356239.1_    1  -LQILEQPKQ-RGFRFRYVCEGP-SHGGLP----GASSEKN--KKSYPQVKICNYVG---PAKVIVQLVT   58
_XP_027251570.1_    1  -LQILEQPKQ-RGFRFRYVCEGP-SHGGLP----GASSEKN--KKSYPQVKICNYVG---PAKVIVQLVT   58
_XP_029330830.1_    1  -LQILEQPKQ-RGFRFRYVCEGP-SHGGLP----GASSEKN--KKSYPQVKICNYVG---PAKVIVQLVT   58
_XP_045426343.1_    1  -LQILEQPKQ-RGFRFRYVCEGP-SHGGLP----GASSEKN--KKSYPQVKICNYMG---PAKVIVQLVT   58
```

```
_NP_001397371.1_    1  -LQILEQPKQ-RGFRFRYVCEGP-SHGGLP----GASSEKN--KKSYPQVKICNYVG---PAKVIVQLVT   58
_XP_040607683.1_    1  -LQILEQPKQ-RGFRFRYVCEGP-SHGGLP----GASSEKN--KKSYPQVKICNYVG---PAKVIVQLVT   58
_XP_026639883.1_    1  -LQILEQPKQ-RGFRFRYVCEGP-SHGGLP----GASSEKN--KKSYPQVKICNYVG---PAKVIVQLVT   58
_XP_029393278.1_    1  -LQILEQPKQ-RGFRFRYVCEGP-SHGGLP----GASSEKN--KKSYPQVKICNYVG---PAKVIVQLVT   58
_XP_049642488.1_    1  -LQILEQPKQ-RGFRFRYVCEGP-SHGGLP----GASSEKN--KKSYPQVKICNYVG---PAKVIVQLVT   58
_XP_004617756.1_    1  -LQILEQPKQ-RGFRFRYVCEGP-SHGGLP----GASSEKN--KKSYPQVKICNYVG---PAKVIVQLVT   58
_XP_021536097.1_    1  -LQILEQPKQ-RGFRFRYVCEGP-SHGGLP----GASSEKN--KKSYPQVKICNYVG---PAKVIVQLVT   58
_NWW35472.1_        1  -LQIIEQPKQ-RGFRFRYVCEGP-SHGGLP----GASSEKN--KKSYPQVKICNYVG---PAKVIVQLVT   58
_KAG8543434.1_      1  -LEIVEQPKQ-RGFRFRYVCEGP-SHGGLP----GASSEKN--KKSYPQIKINNYVG---QAKIIVQLVT   58
_KAG8543366.1_      1  -LEIVEQPKQ-RGFRFRYVCEGP-SHGGLP----GASSEKN--KKSYPQIKINNYVG---QAKIIVQLVT   58
_XP_044130636.1_    1  -LEVVEQPKQ-RGFRFRYVCEGP-SHGGLP----GASSEKN--KKSYPQIKINNYVG---QAKIIVQLVT   58
_XP_040274154.1_    1  -LEVVEQPKQ-RGFRFRYVCEGP-SHGGLP----GASSEKN--KKSYPQIKINNYVG---QAKIIVQLVT   58
_XP_053317513.1_    1  -IEILEQPKQ-RGFRFRYVCEGP-SHGGLP----GASSEKN--KKSYPQIKINNYIG---DAKIIVQLVT   58
_ALA55868.1_        1  -LEVTEQPKQ-RGFRFRYVCEGP-SHGGLP----GASSEKN--KKSYPQIKIYNYNG---HAKIIVQLVT   58
_XP_040185842.1_    1  -LEVTEQPKQ-RGFRFRYVCEGP-SHGGLP----GASSEKN--KKSYPQIKIYNYNG---HAKIIVQLVT   58
_XP_040185853.1_    1  -LEVTEQPKQ-RGFRFRYVCEGP-SHGGLP----GASSEKN--KKSYPQIKIYNYNG---HAKIIVQLVT   58
_XP_018414489.1_    1  -LDVTEQPKQ-RGFRFRYVCEGP-SHGGLP----GASSEKN--KKSYPQIKIHNYIG---HAKLIVQLVT   58
_XP_053559470.1_    1  -IEIIEQPKQ-RGFRFRYVCEGP-SHGGLP----GASSEKN--KKSYPQIKIHNYIG---HAKIVVQLVT   58
_XP_053559471.1_    1  -IEIIEQPKQ-RGFRFRYVCEGP-SHGGLP----GASSEKN--KKSYPQIKIHNYIG---HAKIVVQLVT   58
_KAG8454149.1_      1  -IEITEQPKQ-RGFRFRYVCEGP-SHGGLP----GASSEKN--KKSYPQIKIHNYVG---QVKIIVQLVT   58
_NP_001087808.1_    1  -IEITEQPKQ-RGFRFRYVCEGP-SHGGLP----GASSEKN--RKSYPQIKIHNYVG---QVKVVVQLIT   58
_XP_018098880.1_    1  -IEITEQPKQ-RGFRFRYVCEGP-SHGGLP----GASSEKI--KKSYPQIKIHNYVG---QVKIIVQLIT   58
_XP_002934805.3_    1  -IEITEQPKQ-RGFRFRYVCEGP-SHGGLP----GASSEKN--KKSYPQIKIHNYVG---QVKIIVQLIT   58
_OCT97186.1_        1  -IEITEQPKQ-RGFRFRYVCEGP-SHGGLP----GASSEKI--KKSYPQIKIHNYVG---QVKIIVQLIT   58
_CAI5653158.1_      1  -LQIVEQPKQ-RGFRFRYGCEGP-SHGGLP----GATSEKN--RKTYPTVKICNYQG---QARVVVQLVT   58
_XP_032876238.1_    1  -LQIIEQPKQ-RGFRFRYGCEGP-SHGGLP----GASSEKN--KKAYPAVKICNYVG---HAKIVVQLVS   58
_XP_038648821.1_    1  -LQIVEQPKQ-RGFRFRYGCEGP-SHGGLP----GASSEKN--KKTYPTIKICNYVG---QAKIVVQLVT   58
_XP_038648817.1_    1  -LQIVEQPKQ-RGFRFRYGCEGP-SHGGLP----GASSEKN--KKTYPTIKICNYVG---QAKIVVQLVT   58
_GCB77082.1_        1  -LQIVEQPKQ-RGFRFRYGCEGP-SHGGLP----GASSEKN--KKTYPTVKICNYVG---QAKIVVQLVT   58
_XP_051900898.1_    1  -LQILEQPKQ-RGFRFRYGCEGP-SHGGLP----GASSEKN--KKTYPTVKICNYVG---HAKIVVQLVT   58
_XP_048452319.1_    1  -LQILEQPKQ-RGFRFRYGCEGP-SHGGLP----GASSEKN--KKSYPTVKICNYVG---QAKIVVQLVT   58
_XP_048382772.1_    1  -LQILEQPKQ-RGFRFRYGCEGP-SHGGLP----GASSEKN--KKSYPTVKICNYVG---QAKIVVQLVT   58
_XP_043530455.1_    1  -LQILEQPKQ-RGFRFRYGCEGP-SHGGLP----GASSEKN--KKSYPTVKICNYVG---QAKIVVQLVT   58
_GCC38268.1_        1  -LQILEQPKQ-RGFRFRYGCEGP-SHGGLP----GASSEKN--KKSYPTVKICNYVG---QAKIVVQLVT   58
_XP_041045532.1_    1  -LQIVEQPKQ-RGFRFRYGCEGP-SHGGLP----GASSEKN--KKSYPTVKICNYVG---QAKIVVQLVT   58
_XP_007890062.2_    1  -LQIVEQPKQ-RGFRFRYGCEGP-SHGGLP----GASSEKN--KKTYPTVKICNYVG---NAKIVVQLVT   58
_XP_007897340.2_    1  --IITEQPKQ-RGFRFRYGCEGP-SHGGVP----GASSEKN--RKTYPSVKIINYCG---NAKIVVSLVT   57
_XP_007897342.2_    1  ---------Q-RGFRFRYGCEGP-SHGGVP----GASSEKN--RKTYPSVKIINYCG---NAKIVVSLVT   50
_XP_032868306.1_    1  -IDICEQPKQ-RGFRFRYGCEGP-SHGGLP----GASSEKN--RKTYPSVKIYNYTG---NAKVLVQLVT   58
```

```
_XP_051897529.1_    1  -IAICEQPKQ-RGFRFRYGCEGP-SHGGLP----GASSEKN--RKTYPSVKIFNYTG---NAKVVVQLVT  58
_XP_048419270.1_    1  -LTIIEQPKQ-RGFRFRYGCEGP-SHGGLP----GASSEKN--RKTYPTVKIFNYTG---NAKIVVQLVT  58
_XP_048472262.1_    1  -LTIVEQPKQ-RGFRFRYGCEGP-SHGGLP----GASSEKN--RKTYPTIKIFNYTG---NAKIVVQLVT  58
_GCC17239.1_        1  -LAITEQPKQ-RGFRFRYGCEGP-SHGGLP----GASSEKN--RKTYPTVKIFNYTG---NAKIVVQLVT  58
_XP_043534576.1_    1  -LAITEQPKQ-RGFRFRYGCEGP-SHGGLP----GASSEKN--RKTYPTVKIFNYTG---NAKIVVQLVT  58
_XP_041032206.1_    1  -LHISEQPKQ-RGFRFRYGCEGP-SHGGLP----GASSEKN--RKTYPTIKILNYTG---NAKIVVQLVT  58
_XP_038639209.1_    1  ---------Q-RGFRFRYGCEGP-SHGGLP----GASSEKN--RKTYPTVKIFNYTG---NAKIVVQLVT  50
_XP_032825135.1_    1  -LQITEEPKQ-RGFRFRYPCEGP-SHGGLP----GCSSERN--KKTFPTVRICKYFG---LAKVVVQLVT  58
_AUA18026.1_        1  -LQITEEPKQ-RGFRFRYPCEGP-SHGGLP----GCSSERN--KKTFPTIRICKYLG---LAKVVVQLVT  58
_XP_043559628.1_    1  ------------------------------------------------TFQIHNYTG---PVKIIVQLVT  19
_GCC23558.1_        1  --------------------------------------------------QIHNYTG---PVKIIVQLVT  17
_XP_048459926.1_    1  -IEIIEQPKQ-RGFRFRYGCEGP-SHGGLP----GVASEKN--KKTYPTVKIHNYTG---PAKIVVQLVT  58
_XP_048399043.1_    1  -IEIIEQPKQ-RGFRFRYGCEGP-SHGGLP----GVSSEKN--KKTYPTVKIHNYTG---PAKIVVQLVT  58
_XP_043559626.1_    1  -IEIIEQPKQ-RGFRFRYGCEGP-SHGGLP----GVSSEKN--KKTYPTVKIHNYTG---PVKIIVQLVT  58
_XP_043559625.1_    1  -IEIIEQPKQ-RGFRFRYGCEGP-SHGGLP----GVSSEKN--KKTYPTVKIHNYTG---PVKIIVQLVT  58
_XP_043559624.1_    1  -IEIIEQPKQ-RGFRFRYGCEGP-SHGGLP----GVSSEKN--KKTYPTVKIHNYTG---PVKIIVQLVT  58
_XP_043559627.1_    1  -IEIIEQPKQ-RGFRFRYGCEGP-SHGGLP----GVSSEKN--KKTYPTVKIHNYTG---PVKIIVQLVT  58
_XP_041050315.1_    1  -IEIIEQPKQ-RGFRFRYGCEGP-SHGGLP----GVSSEKN--KKTYPKVKIHNYTG---PAKIVVQLVT  58
_GCB72915.1_        1  -IEIIEQPKQ-RGFRFRYGCEGP-SHGGLP----GVSSEKN--KKTYPTVKIQNYTG---PAKIVVQLVT  58
_XP_051870516.1_    1  -IEIIEQPKQ-RGFRFRYGCEGP-SHGGLP----GVSSEKN--RKTYPTVKIHNYTG---PVKVVVQLVT  58
_XP_032885971.1_    1  -IEIIEEPKQ-RGFRFRYGCEGP-SHGGLP----GVSSEKN--NKTYPAVKIHNYTG---PARVIVQLVT  58
_XP_007904454.1_    1  -IEIIEQPRQ-RGFRFRYGCEGP-SHGGLP----GVSSERS--KKTYPTVKIHNYTG---PAKIVVQLVT  58
_CAD5120983.1_      1  --SFVEQPQE-RGYRFRYESEGA-THGGIQ----GDNTKKS--KKTYPSVQVHNFRG---NVKVVVQLVT  57
_KAI6649899.1_      1  -LSICEQPLQ-KGYRFRYLCEGS-AHGPIT----GATSTKD--KKTFPTIKIEQCLP---NTTVYLHVSL  58
_KAI6652168.1_      1  --IISEQPKS-QGYRFRYECEGY-THGGIP----GEYSERG--RKTHPTLLFNDYEG---RVIVVGYLV-  56
_KAI6653779.1_      1  -LEINEQPKS--AFRFRYSCEGS-SHGALL----GAKSEKG--RKSYPSVSVENYFG---HVVVVASLVP  57
_NP_001267795.1_    1  -LKIERQPRK-YGYRFRYKTEGV-CHGGILADTDGAVNCGS--SKSCPKIKVHNLNG--QRAKVVLRLAA  63
_XP_047123977.1_    1  -LKIERQPRK-YGYRFRYKTEGV-CHGGILADTDGAVNCGS--SKSCPKIKVHNLNG--QRAKVVLRLAA  63
_XP_047123978.1_       ----------------------------------------------------------------------
_KAG8178714.1_      1  LLAIENQPCD--LYRFRYKSENE-KGHHGP----LKGSCPE-REQNFPTVKLLNRNYSQDEIKIKASLYT  62
_GFT03926.1_        1  --IITMQPAD--TFRYRYESEKG-SHGGIK----GEKYSRT--FKCFPEVKLNNAPCN-TNIKIKASLYT  58
_KAI6646872.1_      1  -----DEPAP-YGTRFRYVSEGT-CHGPIL----GQSSIQG--NRTHPNVTCIGNFQDGLKAILLVSLVS  57
_XP_033625504.1_    1  -VDIYEQPRS-KGVSFLNQHQMP-SSSTYI---VGERSKAQ--VQTDPSLLLSNFRG---TVKLVWYLVE  59
_XP_038072325.1_    1  -LEVVEQPRS-LGTPFVKDTDLP-SSSSYI---VGEKSTTE--KTTHPSLLLMNYQG---PVNLVWYLVA  59
_XP_038072324.1_    1  -LEVVEQPRS-LGTPFVKDTDLP-SSSSYI---VGEKSTTE--KTTHPSLLLMNYQG---PVNLVWYLVA  59
Consensus_aa:          .l.IhEQPpp.+G@RFRY.tEGs.SHGtl.....G.pSpp...pKo@Pplpl.N@.G....hplhhpLVo
Consensus_ss:            eeee        eeeee                            eeeeee        eeeeeeeee
```

```
Conservation:                     9 5 7 7      9                       6 55 7    55     7  5
_KAH7637713.1_     60  NEL----QPKLHVHKLMGRHCSADGSCTL--PVNLD---NMTA----IFQNLGILFIGKKEVQEILYRRK  116
_XP_023220155.1_   60  NTD----PAKRHVHRLIGPNCK-DGVCTL--ILDE----NKTA----VFQNISIECIGKRRVKDVIYERN  114
_XP_009016345.1_   59  DEK----IPRHHAHELIGKNCE-NGKCVV--NVKGD---NPII----GFPNLGIKHITKKNLVNVLIDKL  114
_ELU08708.1_       58  DEA----VPRPHPHELMGKSCV-DGVCTV--DLKGDN-SNMVA----HFPNLGIRHVTRKRVIESLTQRI  115
_UCK81536.1_       58  DEK----VPKPHAHELVGKHCS-NGICVL--DVESC---AQAI----QFPGLSIRHSTRKRAVSILTDRI  113
_CAH1795432.1_     60  DET----VPKTHAHELVGKNCN-NGICVV--QLDGR--KNMTA----TFPNLGIIHVTKKKVVDTILSRV  116
_CAE1313443.1_     59  DDS----IPKTHAHELVGKSCN-KGICLM--DVKEA--KNPIC----QFQNIGVMHVTKRKVADVLKERI  115
_XP_048764075.1_   59  NEA----IPKPHAHKLVGKNCN-DGSCIV--ELKPS---QNII----TFPNLCIQHVTRRKASEVIEHRI  114
_XP_009060044.1_   59  VDN-----DKNHPHKLVGKNCE-DGICVM--EVKET---YGPI----HFQNMSVQHVTKKKAVEVLQKRI  113
_XP_050391129.1_   59  ADES--DPPKNHPHKLVGKNCE-DGICVM--HVKDG---HGPI----QFQNMSVQHVTKKKAGEVLLKRI  116
_RUS85575.1_       59  DEK----TPRPHAHKLVGTNCK-NGYCSV--EIKAN---SEPI----SFPNICISHVTRRNLIDVLTERF  114
_GFS09172.1_       59  DEK----TPRPHAHKLVGTNCK-NGYCSV--EIKAN---SEPI----TFPNICISHVTRKNLIDVLTERF  114
_XP_005091024.1_   59  DEK----SPRAHAHKLVGTNCK-DGLCTV--EIKAN---TDPI----IFPNLCILHVTRKKLIDVLTGRI  114
_XP_005091023.1_   59  DEK----SPRAHAHKLVGTNCK-DGLCTV--EIKAN---TDPI----IFPNLCILHVTRKKLIDVLTGRI  114
_KAH9508414.1_     58  DEK----TPRPHAHKLVGTNCK-DGLCTV--ELKAN---PEYI----NFPNLCILHVTRKKLIDVLTERN  113
_XP_013071937.1_   59  DEK----VPRPHAHKLVGTNCK-DGLCTV--ELKSC---LDVV----SFPNLCILHVTRKKLIDVLTERN  114
_KAI8766750.1_     59  DEK----VPRPHAHKLVGTNCK-DGLCTV--ELKSC---LDVV----SFPNLCILHVTRKKLIDVLTERN  114
_KAI8794647.1_     59  DEK----VPRPHAHKLVGTNCK-DGLCTV--ELKSC---LDVV----SFPNLCILHVTRKKLIDVLTERN  114
_NP_001298220.1_   59  DEK----VPRPHAHKLVGTNCK-DGLCTV--ELKSC---LDVV----SFPNLCILHVTGKKLIDVLTERN  114
_CAG5129174.1_     59  DEK----TPRAHAHKLVGSNCK-NGLCTV--EVKAN---VEPV----SFANLSILHVTRKKLVDVLTERI  114
_KAG5707696.1_         ----------------------------------------------------------------------
_XP_025114345.1_   59  DEA----HPHDHAHKLVGKNCN-DGICTV--EQQST---AGPI----GFPNLCVLHVTRKKAAEVLFNRI  114
_QNL15288.1_       59  DED----TPKVHAHKLVGKNCI-GGVCKI--EMNST---ASDI----SFPNLCILHVTRKKAAEVLANRL  114
_XP_041362807.1_   58  DET----VPKHHAHKLVGKNCN-NGICAF--DVKAA---NQNI----TFPNLCVLHVTRKKASEVLEKRI  113
_XP_046550037.1_   59  DED----HPKHHAHKLVGRNCQ-NGICVQ--EVQLT---SGII----SFPNLCVLHVTRKKATEVLEGRI  114
_XP_048248211.1_   59  DED----SPKHHAHKLVGRNCQ-NGICVQ--ETQST---SGII----SFPNLCVLHVTRKKATEVLEGRI  114
_XP_046550036.1_   59  DED----HPKHHAHKLVGRNCQ-NGICVQ--EVQLT---SGII----SFPNLCVLHVTRKKATEVLEGRI  114
_CAC5363017.1_     59  DEA----VPRPHAHKLVGKNCS-DGVCTV--DVKTG---QNTV----TFANLCVQHVTRKKSAEVIEQRI  114
_VDI06196.1_       59  DEP----VPRPHAHKLVGKNCN-DGVCTV--DVKTG---QNTV----TFANLCVQHVTRKKSAEVIEQRI  114
_AHI17304.1_       59  DEP----VPRPHAHKLVGKNCN-DGVCTV--DVKTG---QNTV----TFANLCVQHVTRKKSAEVIEQRI  114
_XP_052068168.1_   59  DEP----VPRPHAHKLVGKNCS-DGVCTV--DVKTG---QNTV----TFANLCVQHVTRKKSAEVIEQRI  114
_XP_052068167.1_   59  DEP----VPRPHAHKLVGKNCS-DGVCTV--DVKTG---QNTV----TFANLCVQHVTRKKSAEVIEQRI  114
_UZH84476.1_       59  DDQ----IPSPRAHKLVGKNCH-NGICVM--EVKAG---SPFV----QFPNLCVQHVTRKKASEVLEQRI  114
_XP_033756865.1_   59  DEV----IPRPHAHKLVGRNCN-EGTCIV--ELKGG---QNSL----TFPNLCVQHVTRRKASDVIEQRI  114
_AKC01669.1_       59  DEV----IPRPHAHKLVGRNCN-GGTCIV--ELKGG---QNSL----TFPNLCVQHVTRRKASEVIEQRI  114
_KAJ8319099.1_     59  DEV----IPRPHAHKLVGKNCS-EGSCIV--ELKTG---QNTA----TFPNLCIQHVTRKKAAEVIQQRI  114
_XP_022318640.1_   59  NEN----IPKPHAHKLVGKNCN-DGSCVV--EQKAN---QNTV----TFPNLCIQHVTRRKASEVIEYRI  114
_XP_011423502.2_   59  NEA----IPKPHAHKLVGKNCN-DGSCVV--ELKPN---QNVI----TFPNLCIQHVTRRKASEVIEYRI  114
```

```
_XP_052690447.1_    59  NEA----IPKPHAHKLVGKNCN-DGSCVV--ELKPN---QNVI----TFPNLCIQHVTRRKASEVIEYRI  114
_AEY99616.1_        59  TES----IPRPHAHKLVGKNCQ-DGVCVT--EIKSG----NTV----NFPNLCIQHVTRRKAADVIEQRI  113
_XP_052263328.1_    59  EDS-----TKPHAHKLVGKNCS-NGLCTI--ETKPG---TTQI----PFPNLCIQHVTRRKAAEVLEQRL  113
_XP_052263327.1_    59  EDS-----TKPHAHKLVGKNCS-NGLCTI--ETKPG---TTQI----PFPNLCIQHVTRRKAAEVLEQRL  113
_XP_052761314.1_    59  DEP----SPKPHAHKLVGKNCT-SGACTV--EVKPG---TAQI----AFPNLCIQHVTRRKAGDVLEERI  114
_XP_053392566.1_    59  DES----VPKSHAHKLVGKHCT-DGVCQL--DVKAG---TSQI----SFPNLCIQHVTRRKASEVLEQRI  114
_KAI0238513.1_      59  SNEV--TPLRIHAHELISKNCQ-NGVYVT--ELSNS--DSREV----QFPNLGIQHATRKNVVEVLEKKI  117
_XP_029638652.1_    58  DDP----EPYLHAHELVGKNCS-KGICTI--EVPST--NNPTC----KLQNIGVMHVTKRKVIDILRERI  114
_AUR53646.1_        58  DDP----EPYLHAHELVGKNCS-KGICTI--DVPST--NNPTC----KLQNIGVMHVTKRKVIDILRERI  114
_XP_036360935.1_    58  DDP----EPYLHAHELVGKNCS-KGICTI--EVPST--NNPTC----KLQNIGVMHVTKRKVIDILRERI  114
_XP_036360934.1_    58  DDP----EPYLHAHELVGKNCS-KGICTI--EVPST--NNPTC----KLQNIGVMHVTKRKVIDILRERI  114
_XP_029638651.1_    58  DDP----EPYLHAHELVGKNCS-KGICTI--EVPST--NNPTC----KLQNIGVMHVTKRKVIDILRERI  114
_XP_014776042.1_    57  DDP----EPYLHAHELVGKNCS-KGICTI--EVPST--NNPIC----KLQNIGVMHVTKRKVIDILKERI  113
_XP_014776041.1_    57  DDP----EPYLHAHELVGKNCS-KGICTI--EVPST--NNPIC----KLQNIGVMHVTKRKVIDILKERI  113
_XP_052825578.1_    57  DDP----EPYLHAHELVGKNCS-KGICTI--EVPST--NNPIC----KLQNIGVMHVTKRKVIDILKERI  113
_XP_052825580.1_     2  ---------------------------------------------KLQNIGVMHVTKRKVIDILKERI   24
_AEP33644.1_        59  DEE----VPKPHAHSLVGKHCC-NGICTV--QVGPK---DLTA----CFQNLGIQHVTRKEVIPVLKKRI  114
_PIK44524.1_        59  DEP----EPKPHAHSLVGRNCN-NGICTV--QVGPA---DMTA----CFQNLMIQHATRRDAIPILQRRI  114
_KAJ8022831.1_      59  DEA----QPKPHAHSLVGRMCD-NGICTV--QVGPN---DMTA----SFQSLGIQHATRREVVPVLQKRI  114
_XP_030838637.1_    13  NDE----IPKPHAHKHCGKHCN-DGVCTV--QLGPT---DMTQG-RQPFPNLGILHVTRKKVEKVLKDRL   71
_XP_038060093.1_    59  NDE----IPKPHAHSIVGKHCN-DGVCTV--QLGPT---DMTA----SFPNLGILHVTRKNVEKVLKDRL  114
_XP_022087493.1_    59  NEE----VPRPHAHSLVGKHCN-DGQCMV--QVGPA---DMTA----SFPNLGILHVTRKNVEKVLKDRL  114
_XP_033643738.1_    59  SED----LPKPHAHSIVGKHCT-DGSCTV--QVGPT---DMTA----SFPNLGILHVTRKNVERVLKDRL  114
_XP_033100082.1_    59  NEE----MPRPHAHSLVGKHCT-NGICSV--QVGAS---DMTA----SFPNLGILHITKKNVIPILKKRM  114
_CCC55418.2_        38  NED----TPRPHAHSLVGKHCK-DGLCTV--QVGPN---DMTA----SFPNLGILHVTRKDVVPTLKTRI   93
_XP_054773081.1_    59  SED----TPRPHAHSLVGKHCK-DGLCTV--QVGPK---DMTA----SFPNLGILHVTRKDVVPTLKTRI  114
_XP_030834883.1_    59  NEE----TPRPHAHSLVGKHCK-DGLCTV--QVGPK---DMTARSEMSFPNLGILHVTRKDVVPTLKTRI  118
_NP_999819.1_       59  NEE----TPRPHAHSLVGKHCK-DGLCTV--QVGPK---DMTA----SFPNLGILHVTRKDVVPTLKTRI  114
_XP_030834884.1_    59  NEE----TPRPHAHSLVGKHCK-DGLCTV--QVGPK---DMTARSEMSFPNLGILHVTRKDVVPTLKTRI  118
_XP_041485048.1_    59  NED----TPRPHAHSLVGKHCK-DGLCTV--QVGPK---DMTA----SFPNLGILHVTRKDVVPTLKTRI  114
_QIA61835.1_        61  END----PPIPHAHSIVGKNTQ-DGRCTL--EMGPE--TEMYA----QFPNMGILHVTKKKVPEVLTRRL  117
_NP_001266238.1_    61  DSD----PAMPHAHSIVGKNAI-DGRCVV--EIGPE--TDMYA----QFTSLGILHVTKKKVPEVLTRRL  117
_QHX41456.1_        60  ESD----PPLPHAHSIVGRNTE-NGQCTV--EVGPE--NDMCA----TFPNLGILHVTRKRVVDVLTERF  116
_XP_022789210.1_    59  VDE----PPMPHAHSLIGKNSA-GGVVTV--QIGPE--QGMTA----TFPNLGIEHVTKKMVGKVLMDRY  115
_XP_046859971.1_    59  TSD----PPTPHAHSLVGKNV--DGACVV--EVSED--TGMTA----VFQNLGIQHVTKKNVKNVLKERH  114
_XP_028414684.1_    59  VSD----PPQPHAHSLVGKNVR-DGACIV--EVGPD--CGMTA----SFPNLGVQHVTKKNVLPILKERY  115
_XP_028414852.1_    59  VSD----PPQPHAHSLVGKNVR-DGACIV--EVGPD--CGMTA----SFPNLGVQHVTKKNVLPILKERY  115
_ADQ57372.1_        59  KDE----PYMLHAHSLTGKNANEEGVVTV--QVGPD--QHMTA----SFPNLGIQHVTKKNVVKVLMDRF  116
_XP_048580668.1_    59  KDE----PYMLHAHSLTGKNANEEGVVTV--QVGPD--QHMTA----SFPNLGIKHVTKKNVVKVLMDRF  116
```

```
_EDO32923.1_       59  KDE----PYMLHAHSLTGKNANEEGVVTV--QVGPD--QHMTA----SFPNLGIQHVTKKNVVKVLMDRF  116
_ABU48530.1_       59  KDE----PYMLHAHSLTGKNANEEGVVTV--QVGPD--QHMTA----SFPNLGIQHVTKKNVVKVLMDRF  116
_ALG40993.1_       59  EEE----PYMPHAHSLTGKNASKEGIVTV--QIGPE--QGMSA----SFPNLGIQHVTRKAVARTLLERY  116
_ALG40994.1_       59  EEE----PYMPHAHSLTGKNASKEGIVTV--QIGPE--QGMSA----SFPNLGIQHVTRKAVAKTLLERY  116
_XP_031565156.1_   59  EEE----PYMPHAHSLTGKNASKEGIVTV--QIGPE--QGMSA----SFPNLGIQHVTRKAVAKTLLERY  116
_ALG40992.1_       59  EDE----PYMPHAHSLTGKNASGEGIVTV--QIGPE--QGMSA----SFPNLGIQHVTRKAVSKTLLSRY  116
_KXJ21435.1_       59  EDE----PHMPHAHSLTGKHANNDGIVTV--QIGTE--QGMTA----SFPNLGIQHVTKKKVAKTLTERY  116
_XP_020916759.1_   59  EDE----PHMPHAHSLTGKHANNDGIVTV--QIGTE--QGMTA----SFPNLGIQHVTKKKVAKTLTERY  116
_XP_015766406.1_   59  SDE----NPMPHAHSLIGKNAS-NGVVTV--QIGPE--QGMTA----SFPNLGIQHVTKKNVGYVLMDRY  115
_UBR69922.1_       59  CDE----NPMPHAHSLIGKNAS-NGVVTV--QIGPE--QGMTA----SFPNLGIQHVTKKNVGYVLMDRY  115
_XP_029186950.2_   59  SDE----NPMPHAHSLIGKNAS-NGVVTV--QIGPE--QGMTA----SFPNLGIQHVTKKNVGYVLMDRY  115
_UCR60992.1_       59  CDE----NPMPHAHSLIGKNAS-NGVVTV--QIGPE--QGMTA----SFPNLGIQHVTKKNVGYVLMDRY  115
_XP_027038010.1_   59  VDE----PPMPHAHSLISKNSN-NGVVTV--QIGPE--QGMTA----TFPNLGIEHVTKKMVSKVLMDRY  115
_NWX00991.1_       59  NGK----YVHLHAHSLVGKFCE-DGVCTV--NAGPK---DMVV----GFANLGILHVTKKKVFETLEARM  114
_NXS91232.1_       59  NGK----YVHLHAHSLVGKFCE-DGVCTV--NAGPK---DMVV----GFANLGILHVTKKKVFETLETRM  114
_NXG78778.1_       59  NGK----YVHLHAHSLVGKFCE-DGVCTV--NAGPK---DMVV----GFANLGILHVTKKKVFETLETRM  114
_XP_025964754.1_   59  NGK----YVHLHAHSLVGKFCE-DGVCTV--NAGPK---DMVV----GFANLGILHVTKKKVFETLEARM  114
_XP_014730709.1_   59  NGK----YVHLHAHSLVGKFCE-DGVCTV--TAGPK---DMVV----GFANLGILHVTKKKVFETLEARM  114
_NXW45082.1_       59  NGK----YVHLHAHSLVGKFCE-DGVCTV--NAGPK---DMVV----GFANLGILHVTKKKVFETLESRM  114
_XP_015479466.1_   59  NGK----YVHLHAHSLVGKFCE-DGVCTV--NAGPK---DMVV----GFANLGILHVTKKKVFETLETRM  114
_XP_009577719.1_   59  NGK----YVHLHAHSLVGKFCE-DGVCTV--NAGPK---DMVV----GFANLGILHVTKKKVFETLETRM  114
_NXC45563.1_       59  NGK----YVHLHAHSLVGKFCE-DGVCTV--NAGPK---DMVV----GFANLGILHVTKKKVFETLEARM  114
_XP_009883811.1_   59  NGK----YVHLHAHSLVGKFCE-DGVCTV--NAGPK---DMVV----GFANLGILHVTKKKVFETLETRM  114
_NXH56308.1_       59  NGK----YVHLHAHSLVGKFCE-DGVCTV--TAGPK---DMVV----GFANLGILHVTKKKVFETLEARM  114
_XP_023780422.1_   59  NGK----YVHLHAHSLVGKFCE-DGVCTV--NAGPK---DMVV----GFANLGILHVTKKKVFETLETRM  114
_XP_009271830.1_   59  NGK----YVHLHAHSLVGKFCE-DGVCTV--NAGPK---DMVV----GFANLGILHVTKKKVFETLETRM  114
_XP_009325314.1_   59  NGK----YVHLHAHSLVGKFCE-DGVCTV--NAGPK---DMVV----GFANLGILHVTKKKVFETLETRM  114
_NXM85820.1_       59  NGK----YVHLHAHSLVGKFCE-DGVCTV--TAGPK---DMVV----GFANLGILHVTKKKVFETLETRM  114
_NXI98149.1_       59  NGK----YVHLHAHSLVGKFCE-DGVCTV--NAGPK---DMVV----GFANLGILHVTKKKVFETLETRM  114
_XP_013807107.1_   59  NGK----YVHLHAHSLVGKFCE-DGVCTV--NAGPK---DMVV----GFANLGILHVTKKKVFETLEARM  114
_NWS89770.1_       59  NGK----YVHLHAHSLVGKFCE-DGVCTV--TAGPK---DMVV----GFANLGILHVTKKKVFETLEARM  114
_NXN94237.1_       59  NGK----YVHLHAHSLVGKFCE-DGVCSV--NAGPK---DMVV----GFANLGILHVTKKKVFETLESRM  114
_NXH67539.1_       59  NGK----YVHLHAHSLVGKFCE-DGVCTV--NAGPK---DMVV----GFANLGILHVTKKKVFETLETRM  114
_NXQ49570.1_       59  NGK----YVHLHAHSLVGKFCE-DGVCTV--TAGPK---DMVV----GFANLGILHVTKKKVFETLETRM  114
_NWT07854.1_       59  NGK----YVHLHAHSLVGKFCE-DGVCTV--NAGPK---DMVV----GFANLGILHVTKKKVFETLETRM  114
_XP_032915292.1_   59  NGK----YVHLHAHSLVGKFCE-DGVCTV--TAGPK---DMVV----GFANLGILHVTKKKVFETLETRM  114
_XP_014117319.1_   59  NGK----YVHLHAHSLVGKFCE-DGVCTV--NAGPK---DMVV----GFANLGILHVTKKKVFETLETRM  114
_XP_009816360.1_   59  NGK----YVHLHAHSLVGKFCE-DGVCTV--NAGPK---DMVV----GFANLGILHVTKKKVFETLETRM  114
_NXO31807.1_       59  NGK----YVHLHAHSLVGKHCE-DGVCTV--SAGPK---DMVV----GFANLGILHVTKKKVFETLETRM  114
```

```
_NWW07363.1_       59  NGK----YVHLHAHSLVGKFCE-DGVCTV--NAGPK---DMVV----GFANLGILHVTKKKVFETLETRM  114
_XP_054681626.1_   59  NGK----YVHLHAHSLVGKFCE-DGVCTV--NAGPK---DMVV----GFANLGILHVTKKKVFETLETRM  114
_XP_010286301.1_   59  NGK----YVHLHAHSLVGKFCE-DGVCTV--NAGPK---DMVV----GFSNLGILHVTKKKVFETLETRM  114
_NXC54686.1_       59  NGK----YVHLHAHSLVGKFCE-DGVCTV--NAGPK---DMVV----GFANLGILHVTKKKVFETLETRM  114
_XP_054059788.1_   59  NGK----YVHLHAHSLVGKFCE-DGVCTV--NAGPK---DMVV----GFANLGILHVTKKKVFETLETRM  114
_OPJ75671.1_       59  NGK----YVHLHAHSLVGKFCE-DGVCTV--NAGPK---DMVV----GFANLGILHVTKKKVFETLEARM  114
_XP_010311473.1_   59  NGK----YVHLHAHSLVGKFCE-DGVCTV--NAGPK---DMVV----GFANLGILHVTKKKVFETLETRM  114
_XP_053921662.1_   59  NGK----YVHLHAHSLVGKYCE-DGVCTV--NAGPK---DMVV----GFANLGILHVTKKKVFETLETRM  114
_NWQ94220.1_       59  NGK----YVHLHAHSLVGKFCE-DGVCTV--NAGPK---DMVV----GFANLGILHVTKKKVFETLETRM  114
_XP_025934196.1_   59  NGK----YVHLHAHSLVGKFCE-DGVCTV--NAGPK---DMVV----GFANLGILHVTKKKVFETLEARM  114
_NXS57318.1_       59  NGK----YVHLHAHSLVGKFCE-DGICTV--NAGPK---DMVV----GFANLGILHVTKKKVFETLETRM  114
_NXF52248.1_       59  NGK----YVHLHAHSLVGKFCE-DGVCTV--NAGPK---DMVV----GFANLGILHVTKKKVFETLETRM  114
_OPJ75670.1_       59  NGK----YVHLHAHSLVGKFCE-DGVCTV--NAGPK---DMVV----GFANLGILHVTKKKVFETLEARM  114
_NXD98176.1_       59  NGK----YVHLHAHSLVGKFCE-DGVCTV--NAGPK---DMVV----GFANLGILHVTKKKVFETLETRM  114
_XP_009949173.1_   59  NGK----YVHLHAHSLVGKFCE-DGVCTV--NAGPK---DMVV----GFANLGILHVTKKKVFETLEARM  114
_NWH32710.1_       59  NGK----YVHLHAHSLVGKFCE-DGVCTV--IAGPK---DMVV----GFANLGILHVTKKKVFETLETRM  114
_XP_010178978.1_   59  NGK----YVHLHAHSLVGKFCE-DGVCTV--NAGPK---DMVV----GFANLGILHVTKKKVFETLETRM  114
_NXI82337.1_       59  NGK----YVHLHAHSLVGKFCE-DGVCTV--NAGPK---DMVV----GFANLGILHVTKKKVFETLETRM  114
_NXJ24754.1_       59  NGK----YVHLHAHSLVGKFCE-DGVCTV--NAGPK---DMVV----GFANLGILHVTKKKVFETLETRM  114
_NXM41500.1_       59  NGK----YVHLHAHSLVGKFCE-DGVCTV--NAGPK---DMVV----GFANLGILHVTKKKVFETLETRM  114
_NWW61888.1_       59  NGK----YVHLHAHSLVGKFCE-DGVCTV--NAGPK---DMVV----GFANLGILHVTKKKVFETLETRM  114
_NXE22655.1_       59  NGK----YVHLHAHSLVGKFCE-DGVCTV--NAGPK---DMVV----GFANLGILHVTKKKVFETLETRM  114
_NXF65753.1_       59  NGK----YVHLHAHSLVGKFCE-DGVCTV--NAGPK---DMVV----GFANLGILHVTKKKVFETLEARM  114
_NWS57053.1_       59  NGK----YVHLHAHSLVGKFCE-DGVCTV--NAGPK---DMVV----GFANLGILHVTKKKVFETLETRM  114
_NWU51843.1_       59  NGK----YVHLHAHSLVGKFCE-DGVCTV--NAGPK---DMVV----GFANLGILHVTKKKVFETLETRM  114
_NXB31773.1_       59  NGK----YVHLHAHSLVGKFCE-DGVCTV--NAGPK---DMVV----GFANLGILHVTKKKVFETLETRM  114
_NXE37412.1_       59  NGK----YVHLHAHSLVGKFCE-DGVCTV--NAGPK---DMVV----GFANLGILHVTKKKVFETLETRM  114
_NXM73977.1_       59  NGK----YVHLHAHSLVGKFCE-DGVCTV--NAGPK---DMVV----GFANLGILHVTKKKVFETLETRM  114
_NXK45099.1_       59  NGK----YVHLHAHSLVGKFRE-DGVCTV--NAGPK---DMVV----GFANLGILHVTKKKVFETLETRM  114
_NXT11309.1_       59  NGK----YVHLHAHSLVGKACE-DGVCTV--LAGPK---DMVV----GFANLGILHVTKKKVFETLETRM  114
_XP_039920334.1_   59  NGK----YVHLHAHSLVGKFCE-DGVCTV--NAGPK---DMVV----GFANLGILHVTKKKVFETLETRM  114
_NXQ27105.1_       59  NGK----YVHLHAHSLVGKFCE-DGVCTV--NAGPK---DMVV----GFANLGILHVTKKKVFETLETRM  114
_NXN43964.1_       59  NGK----YVHLHAHSLVGKFCE-DGVCTV--NAGPK---DMVV----GFANLGILHVTKKKVFETLETRM  114
_XP_025934203.1_   59  NGK----YVHLHAHSLVGKFCE-DGVCTV--NAGPK---DMVV----GFANLGILHVTKKKVFETLEARM  114
_KFM03673.1_       59  NGK----YVHLHAHSLVGKFCE-DGVCTV--NAGPK---DMVV----GFANLGILHVTKKKVFETLETRM  114
_NXN08485.1_       59  NGK----YVHLHAHSLVGKFCE-DGVCTV--NAGPK---DMVV----GFANLGILHVTKKKVFETLEARM  114
_NXR70665.1_       59  NGK----YVHLHAHSLVGKFCE-DGVCTV--NAGPK---DMVV----GFANLGILHVTKKKVFETLETRM  114
_XP_010165409.1_   59  NGK----YVHLHAHSLVGKFCE-DGVCTV--NAGPK---DMVV----GFANLGILHVTKKKVFETLESRM  114
_XP_010584501.1_   59  NGK----YVHLHAHSLVGKFCE-DGVCTV--NAGPK---DMVV----GFANLGILHVTKKKVFETLETRM  114
```

```
_NWS41193.1_        59  NGK----YVHLHAHSLVGKFCE-DGVCTV--NAGPK---DMVV----GFANLGILHVTKKKVFETLETRM  114
_KFV51344.1_        59  NGK----YVHLHAHSLVGKFCE-DGVCTV--NAGPK---DMVV----GFANLGILHVTKKKVFETLETRM  114
_NXE09783.1_        59  NGK----YVHLHAHSLVGKFCE-DGVCTV--NAGPK---DMVV----GFANLGILHVTKKKVFETLETRM  114
_NXI10557.1_        59  NGK----YVHLHAHSLVGKFCE-DGVCTV--IAGPK---DMVV----GFANLGILHVTKKKVFETLETRM  114
_NXJ43845.1_        59  NGK----YVHLHAHSLVGKFCE-DGVCTV--NAGPK---DMVV----GFANLGILHVTKKKVFETLETRM  114
_NWS05278.1_        59  NGK----YVHLHAHSLVGKFCE-DGVCTV--LAGPK---DMVV----GFANLGILHVTKKKVFETLETRM  114
_XP_027573674.2_    59  NGK----YVHLHAHSLVGKFCE-DGVCTV--NAGPK---DMVV----GFANLGILHVTKKKVFETLETRM  114
_NXQ94576.1_        59  NGK----YVHLHAHSLVGKFCE-DGVCTV--NAGPK---DMVV----GFANLGILHVTKKKVFETLETRM  114
_KAI1238512.1_      59  NGK----YVHLHAHSLVGKFCE-DGVCTV--TAGPK---DMVV----GFANLGILHVTKKKVFETLEARM  114
_NXK36380.1_        59  NGK----YVHLHAHSLVGKFCE-DGVCTV--NAGPK---DMVV----GFANLGILHVTKKKVFETLETRM  114
_NWH51107.1_        59  NGK----YVHLHAHSLVGKFCE-DGVCTV--NAGPK---DMVV----GFANLGILHVTKKKVFETLETRM  114
_NXW80580.1_        59  NGK----YVHLHAHSLVGKFCE-DGVCTV--NAGPK---DMVV----GFANLGILHVTKKKVFETLETRM  114
_NXY08181.1_        59  NGK----YVHLHAHSLVGKFCE-DGVCTV--NAGPK---DMVV----GFANLGILHVTKKKVFETLETRM  114
_NWU97338.1_        59  NGK----YVHLHAHSLVGKFCE-DGVCSV--NAGPK---DMVV----GFANLGILHVTKKKVFETLESRM  114
_XP_027491592.1_    59  NGK----YVHLHAHSLVGKFCE-DGVCTV--NAGPK---DMVV----GFANLGILHVTKKKVFETLETRM  114
_NXU08140.1_        59  NGK----YVHLHAHSLVGKFCE-DGVCTV--NAGPK---DMVV----GFSNLGILHVTKKKVFETLETRM  114
_XP_029870423.1_    59  NGK----YVHLHAHSLVGKFCE-DGVCTV--NAGPK---DMVV----GFANLGILHVTKKKVFETLETRM  114
_XP_037992222.1_    59  NGK----YVHLHAHSLVGKFCE-DGVCTV--LAGPK---DMVV----GFANLGILHVTKKKVFETLETRM  114
_XP_019470357.1_    59  NGK----YVHLHAHSLVGKFCE-DGVCTV--NAGPK---DMVV----GFANLGILHVTKKKVFETLETRM  114
_XP_051646449.1_    59  NGK----YVHLHAHSLVGKFCE-DGVCTV--NAGPK---DMVV----GFANLGILHVTKKKVFETLETRM  114
_NXT54739.1_        59  NGK----YVHLHAHSLVGKFCE-DGVCTV--NAGPK---DMVV----GFANLGILHVTKKKVFETLETRM  114
_NXG03656.1_        59  NGK----YVHLHAHSLVGKFCE-DGVCTV--NAGPK---DMVV----GFANLGILHVTKKKVLETLETRM  114
_NWT86202.1_        59  NGK----YVHLHAHSLVGKFCE-DGVCTV--SAGPK---DMVV----GFANLGILHVTKKKVFETLETRM  114
_XP_009687644.1_    59  NGK----YVHLHAHSLVGKFCE-DGVCTV--NAGPK---DMVV----GFANLGILHVTKKKVFETLEARM  114
_NWQ73497.1_        59  NGK----YVHLHAHSLVGKFCE-DGVCTV--NAGPK---DMVV----GFANLGILHVTKKKVFETLEARM  114
_XP_050751358.1_    59  NGK----YVHLHAHSLVGKFCE-DGVCTV--NAGPK---DMVV----GFANLGILHVTKKKVFETLETRM  114
_KFO60593.1_        59  NGK----YVHLHAHSLVGKFCE-DGVCTV--NAGPK---DMVV----GFANLGILHVTKKKVLETLETRM  114
_XP_014133950.1_    59  NGK----YVHLHAHSLVGKFCE-DGVCTV--NAGPK---DMVV----GFANLGILHVTKKKVFETLETRM  114
_NXK26872.1_        59  NGK----YVHLHAHSLVGKFCE-DGVCTV--NAGPK---DMVV----GFANLGILHVTKKKVFETLEARM  114
_NWQ68199.1_        59  NGK----YVHLHAHSLVGKFCE-DGVCTV--NAGPK---DMVV----GFANLGILHVTKKKVFETLETRM  114
_XP_030348000.1_    59  NGK----YVHLHAHSLVGKFCE-DGVCTV--NAGPK---DMVV----GFANLGILHVTKKKVFETLETRM  114
_NWI78033.1_        59  NGK----YVHLHAHSLVGKFCE-DGVCTV--NAGPK---DMVV----GFANLGILHVTKKKVFETLETRM  114
_NXO71082.1_        59  NGK----YVHLHAHSLVGKFCE-DGVCTV--NAGPK---DMVV----GFANLGILHVTKKKVFETLETRM  114
_XP_040454521.1_    59  NGK----YVHLHAHSLVGKFCE-DGVCTV--NAGPK---DMVV----GFANLGILHVTKKKVFETLETRM  114
_NXQ12070.1_        59  NGK----YVHLHAHSLVGKFCE-DGVCTV--IAGPK---DMVV----GFANLGILHVTKKKVLETLETRM  114
_NXB56634.1_        59  NGK----YVHLHAHSLVGKFCE-DGVCTV--NAGPK---DMVV----GFANLGILHVTKKKVFETLETRM  114
_NXO78917.1_        59  NGK----YVHLHAHSLVGKFCE-DGVCTV--SAGPK---DMVV----GFANLGILHVTKKKVFETLETRM  114
_NXR80251.1_        59  NGK----YVHLHAHSLVGKFCE-DGVCTV--NAGPK---DMVV----GFANLGILHVTKKKVFETLETRM  114
_NXP31238.1_        59  NGK----YVHLHAHSLVGKFCE-DGVCTV--NAGPK---DMVV----GFANLGILHVTKKKVFETLETRM  114
```

```
_NXE73484.1_        59  NGK----YVHLHAHSLVGKFCE-DGVCTV--NAGPK---DMVV----GFANLGILHVTKKKVFETLETRM  114
_NXM25057.1_        59  NGK----YVHLHAHSLVGKFCE-DGVCTV--NAGPK---DMVV----GFANLGILHVTKKKVFETLETRM  114
_NXA89423.1_        59  NGK----YVHLHAHSLVGKFCE-DGVCTV--NAGPK---DMVV----GFANLGILHVTKKKVFETLETRM  114
_NXB19967.1_        59  NGK----YVHLHAHSLVGKFCE-DGVCTV--NAGPK---DMVV----GFANLGILHVTKKKVFETLETRM  114
_NXP82485.1_        59  NGK----YVHLHAHSLVGKYCE-DGVCTV--NAGPK---DMVV----GFANLGILHVTKKKVFETLEARM  114
_NWW54706.1_        59  NGK----YVHLHAHSLVGKFCE-DGVCTV--NAGPK---DMVV----GFANLGILHVTKKKVFETLETRM  114
_NXF96805.1_        59  NGK----YVHLHAHSLVGKYCE-DGVCTV--NAGPK---DMVV----GFANLGILHVTKKKVFETLEARM  114
_NXY35925.1_        59  NGK----YVHLHAHSLVGKFCE-DGVCTV--NAGPK---DMVV----GFANLGILHVTKKKVFETLETRM  114
_XP_037236065.1_    59  NGK----YVHLHAHSLVGKFCE-DGVCTV--NAGPK---DMVV----GFANLGILHVTKKKVFETLETRM  114
_NWT99824.1_        59  NGK----YVHLHAHSLVGKFCE-DGVCTV--LAGPK---DMVV----GFANLGILHVTKKKVLETLETRM  114
_XP_052631299.1_    59  NGK----YVHLHAHSLVGKFCE-DGVCTV--NAGPK---DMVV----GFANLGILHVTKKKVFETLETRM  114
_NXO12533.1_        59  NGK----YVHLHAHSLVGKFCE-DGVCTV--NAGPK---DMVV----GFANLGILHVTKKKVFETLETRM  114
_NWZ34533.1_        59  NGK----YVHLHAHSLVGKFCE-DGVCTV--NAGPK---DMVV----GFANLGILHVTKKKVLETLETRM  114
_NXA30360.1_        59  NGK----YVHLHAHSLVGKFCE-DGVCTV--NAGPK---DMVV----GFANLGILHVTKKKVFETLETRM  114
_NWU09820.1_        59  NGK----YVHLHAHSLVGKFCE-DGVCTV--NAGPK---DMVV----GFANLGILHVTKKKVFETLETRM  114
_NXO38268.1_        59  NGK----YVHLHAHSLVGKFCE-DGVCTV--NAGPK---DMVV----GFANLGILHVTKKKVFETLETRM  114
_NXY70755.1_        59  NGK----YVHLHAHSLVGKFCE-DGVCTV--NAGPK---DMVV----GFANLGILHVTKKKVFETLETRM  114
_RLW08972.1_        59  NGK----YVHLHAHSLVGKFCE-DGVCTV--IAGPK---DMVV----GFANLGILHVTKKKVFETLETRM  114
_XP_013155126.2_    59  NGK----YVHLHAHSLVGKFCE-DGVCTV--NAGPK---DMVV----GFANLGILHVTKKKVFETLETRM  114
_XP_053830927.1_    59  NGK----YVHLHAHSLVGKFCE-DGVCTV--IAGPK---DMVV----GFANLGILHVTKKKVFETLETRM  114
_XP_040454531.1_    59  NGK----YVHLHAHSLVGKFCE-DGVCTV--NAGPK---DMVV----GFANLGILHVTKKKVFETLETRM  114
_XP_049671253.1_    59  NGK----YVHLHAHSLVGKFCE-DGVCTV--NAGPK---DMVV----GFANLGILHVTKKKVFETLETRM  114
_XP_050829478.1_    59  NGK----YVHLHAHSLVGKFCE-DGVCTV--LAGPK---DMVV----GFANLGILHVTKKKVFETLETRM  114
_XP_029818864.1_    59  NGK----YVHLHAHSLVGKFCE-DGVCTV--NAGPK---DMVV----GFANLGILHVTKKKVFETLETRM  114
_NWV77110.1_        59  NGK----CIHLHAHSLVGKFCE-DGVCTV--NAGPK---DMVV----GFANLGILHVTKKKVFETLETRM  114
_NXW55588.1_        59  NGK----YVHLHAHSLVGKFCE-DGVCTV--NAGPK---DMVV----GFANLGILHVTKKKVFETLETRM  114
_NXK11347.1_        59  NGK----YVHLHAHSLVGKFCE-DGVCTV--NAGPK---DMVV----GFANLGILHVTKKKVFETLEMRM  114
_XP_037236074.1_    59  NGK----YVHLHAHSLVGKFCE-DGVCTV--NAGPK---DMVV----GFANLGILHVTKKKVFETLETRM  114
_NXM20845.1_        59  NGK----YVHLHAHSLVGKFCE-DGVCTV--IAGPK---DMVV----GFANLGILHVTKKKVFETLEARM  114
_XP_009472960.1_    59  NGK----YVHLHAHSLVGKFCE-DGVCTV--NAGPK---DMVV----GFANLGILHVTKKKVFETLETRM  114
_NXP26913.1_        59  NGK----YVHLHAHSLVGKFCE-DGVCTV--NAGPK---DMVV----GFANLGILHVTKKKVFETLETRM  114
_NXB10880.1_        59  NGK----YVHLHAHSLVGKFCE-DGVCTV--NAGPK---DMVV----GFANLGILHVTKKKVFETLETRM  114
_NXQ83786.1_        59  NGK----YVHLHAHSLVGKFCE-DGVCTV--NAGPK---DMVV----GFANLGILHVTKKKVFETLETRM  114
_XP_041571848.1_    59  NGK----YVHLHAHSLVGKFCE-DGVCTV--IAGPK---DMVV----GFANLGILHVTKKKVLETLETRM  114
_NXS86984.1_        59  NGK----YVHLHAHSLVGKFCE-DGVCTV--NAGPK---DMVV----GFANLGILHVTKKKVFETLETRM  114
_XP_031966433.1_    59  NGK----YVHLHAHSLVGKFCE-DGVCTV--NAGPK---DMVV----GFANLGILHVTKKKVLETLETRM  114
_BAA02872.1_        59  NGK----YVHLHAHSLVGKFCE-DGVCTV--NAGPK---DMVV----GFANLGILHVTKKKVFETLETRM  114
_XP_051473650.1_    59  NGK----YVHLHAHSLVGKFCE-DGVCTV--NAGPK---DMVV----GFANLGILHVTKKKVLETLETRM  114
_XP_046771508.1_    59  NGK----YVHLHAHSLVGKFCE-DGVCTV--NAGPK---DMVV----GFANLGILHVTKKKVFETLETRM  114
```

```
_XP_033920424.1_    59  NGK----YVHLHAHSLVGKFCE-DGVCTV--NAGPK---DMVV----GFANLGILHVTKKKVFETLETRM  114
_NXK78645.1_        59  NGK----YVHLHAHSLVGKFCE-DGVCTV--NAGPK---DMVV----GFANLGILHVTKKKVFETLEARM  114
_XP_014795980.1_    59  NGK----YVHLHAHSLVGKFCE-DGVCTV--NAGPK---DMVV----GFANLGILHVTKKKVFETLETRM  114
_NXN79327.1_        59  NGK----YVHLHAHSLVGKFCE-DGVCTV--NAGPK---DMVV----GFANLGILHVTKKKVFETLETRM  114
_XP_041905595.1_    59  NGK----YVHLHAHSLVGKFCE-DGVCTV--NAGPK---DMVV----GFANLGILHVTKKKVLETLETRM  114
_KQK83110.1_        59  NGK----YVHLHAHSLVGKFCE-DGVCTV--NAGPK---DMVV----GFANLGILHVTKKKVFETLEARM  114
_NP_001383324.1_    59  NGK----YVHLHAHSLVGKFCE-DGVCTV--NAGPK---DMVV----GFANLGILHVTKKKVFETLETRM  114
_NWX31294.1_        59  NGK----YVHLHAHSLVGKFCE-DGVCTV--NAGPK---DMVV----GFANLGILHVTKKKVFETLETRM  114
_XP_050194007.1_    59  NGK----YVHLHAHSLVGKFCE-DGVCTV--NAGPK---DMVV----GFANLGILHVTKKKVFETLETRM  114
_NXP05826.1_        59  NGK----YVHLHAHSLVGKFCE-DGVCTV--NAGPK---DMVV----GFANLGILHVTKKKVFETLETRM  114
_XP_027759894.1_    59  NGK----YVHLHAHSLVGKFCE-DGVCTV--NAGPK---DMVV----GFANLGILHVTKKKVFETLETRM  114
_XP_009873772.1_    59  NGK----YVHLHAHSLVGKFCE-DGVCTV--NAGPK---DMVV----GFANLGILHVTKKKVFETLEMRM  114
_XP_053796910.1_    59  NGK----YVHLHAHSLVGKFCE-DGVCTV--IAGPK---DMVV----GFANLGILHVTKKKVFETLEARM  114
_XP_039406722.1_    59  NGK----YVHLHAHSLVGKFCE-DGVCTV--NAGPK---DMVV----GFANLGILHVTKKKVLETLETRM  114
_NWU26823.1_        59  NGK----YVHLHAHSLVGKFCE-DGVCTV--NAGPK---DMVV----GFANLGILHVTKKKVFETLETRM  114
_XP_051473648.1_    59  NGK----YVHLHAHSLVGKFCE-DGVCTV--NAGPK---DMVV----GFANLGILHVTKKKVLETLETRM  114
_Q04861.2_          59  NGK----YVHLHAHSLVGKFCE-DGVCTV--NAGPK---DMVV----GFANLGILHVTKKKVFETLETRM  114
_NXL17972.1_        59  NGK----YVHLHAHSLVGKFCE-DGVCTV--LAGPK---DMVV----GFANLGILHVTKKKVFETLETRM  114
_XP_054239251.1_    59  NGK----YVHLHAHSLVGKFCE-DGVCTV--NAGPK---DMVV----GFANLGILHVTKKKVFETLEARM  114
_AAB58343.1_        59  NGK----YVHLHAHSLVGKFCE-DGVCTV--NAGPK---DMVV----GFANLGILHVTKKKVFETLETRM  114
_NXO56761.1_        59  NGK----YVHLHAHSLVGKFCE-DGVCTV--NAGPK---DMVV----GFANLGILHVTKKKVFETLETRM  114
_XP_021251730.1_    59  NGK----YVHLHAHSLVGKFCE-DGVCTV--NAGPK---DMVV----GFANLGILHVTKKKVFETLETRM  114
_XP_015717578.1_    59  NGK----YVHLHAHSLVGKFCE-DGVCTV--NAGPK---DMVV----GFANLGILHVTKKKVFETLETRM  114
_NWX21242.1_        59  NGK----YVHLHAHSLVGKFCE-DGVCTV--NAGPK---DMVV----GFANLGILHVTKKKVFETLETRM  114
_NXR39429.1_        59  NGK----YVHLHAHSLVGKFCE-DGVCTV--NAGPK---DMVV----GFANLGILHVTKKKVFETLETRM  114
_XP_027532933.1_    59  NGK----YVHLHAHSLVGKFCE-DGVCTV--NAGPK---DMVV----GFANLGILHVTKKKVFETLETRM  114
_NXT85173.1_        59  NGK----CVHLHAHSLVGKFCE-DGVCTV--NAGPK---DMVV----GFANLGILHVTKKKVFETLETRM  114
_XP_030803683.1_    59  NGK----YVHLHAHSLVGKFCE-DGVCTV--LAGPK---DMVV----GFANLGILHVTKKKVFETLETRM  114
_XP_041340240.1_    59  NGK----YVHLHAHSLVGKFCE-DGVCTV--LAGPK---DMVV----GFANLGILHVTKKKVFETLETRM  114
_NWT29590.1_        59  NGK----YVHLHAHSLVGKFCE-DGVCTV--LAGPK---DMVV----GFANLGILHVTKKKVFETLETRM  114
_NXH28317.1_        59  NGK----YVHLHAHSLVGKFCE-DGVCTV--NAGPK---DMVV----GFANLGILHVTKKKVFETLETRM  114
_NWS12389.1_        59  NGK----YVHLHAHSLVGKFCE-DGVCTV--NAGPK---DMVV----GFANLGILHVTKKKVFETLETRM  114
_NXF08296.1_        59  NGK----YVHLHAHSLVGKFCE-DGVCTV--NAGPK---DMVV----GFANLGILHVTKKKVFETLETRM  114
_NXP45252.1_        59  NGK----CVHLHAHSLVGKFCE-DGVCTV--NAGPK---DMVV----GFANLGILHVTKKKVFETLETRM  114
_XP_048159384.1_    59  NGK----YVHLHAHSLVGKFCE-DGVCTV--NAGPK---DMVV----GFANLGILHVTKKKVLETLETRM  114
_NXG13678.1_        59  NGK----YVHLHAHSLVGKFCE-DGVCTV--NAGPK---DMVV----GFANLGILHVTKKKVFETLETRM  114
_NXG81801.1_        50  NGK----YVHLHAHSLVGKFCE-DGVCTV--NAGPK---DMVV----GFANLGILHVTKKKVFETLETRM  105
_NXM64740.1_        59  NGK----YVHLHAHSLVGKFCE-DGVCTV--NAGPK---DMVV----GFANLGILHVTKKKVLETLETRM  114
_NXJ10506.1_        59  NGK----YVHLHAHSLVGKFCE-DGVCTV--NAGPK---DMVV----GFANLGILHVTKKKVFETLETRM  114
```

_XP_005418238.1_	59	NGK----YVHLHAHSLVGKFCE-DGVCTV--LAGPK---DMVV----GFANLGILHVTKKKVFETLETRM	114
_XP_009078875.1_	59	NGK----YVHLHAHSLVGKFCE-DGVCTV--NAGPK---DMVV----GFANLGILHVTKKKVFETLETRM	114
_XP_010204515.1_	59	NGK----YVHLHAHSLVGKFCE-DGVCTV--NAGPK---DMVV----GFSNLGILHVTKKKVFETLETRM	114
_XP_008939135.1_	59	NGK----YVHLHAHSLVGKFCE-DGVCTV--NAGPK---DMVV----GFSNLGILHVTKKKVFETLETRM	114
NXA77498.1	59	NGK----YVHLHAHSLVGKFCE-DGVCTV--NAGPK---DMVV----GFANLGILHVTKKKVFETLETRM	114
_XP_025906692.1_	59	NGK----YVHLHAHSLVGKFCE-DGVCTV--NAGPK---DMVV----GFANLGILHVTKKKVFETLEARM	114
NWS73106.1	59	NGK----YVHLHAHSLVGKYCE-DGVCTV--NAGPK---DMVV----GFANLGILHVTKKKVFETLETRM	114
_XP_042726775.1_	59	NGK----YVHLHAHSLVGKFCE-DGVCTV--NAGPK---DMVV----GFANLGILHVTKKKVFETLETRM	114
_XP_031464691.1_	59	NGK----YVHLHAHSLVGKFCE-DGVCTV--NAGPK---DMVV----GFANLGILHVTKKKVFETLETRM	114
KFP33485.1	59	NGK----YVHLHAHSLVGKFCE-DGVCTV--NAGPK---DMVV----GFSNLGILHVTKKKVFETLETRM	114
_XP_054146082.1_	59	NGK----YVHLHAHSLVGKFCE-DGVCTV--LAGPK---DMVV----GFANLGILHVTKKKVFETLETRM	114
_XP_041280451.1_	59	NGK----YVHLHAHSLVGKFCE-DGVCTV--LAGPK---DMVV----GFANLGILHVTKKKVFETLETRM	114
_XP_021387126.1_	59	NGK----YVHLHAHSLVGKFCE-DGVCTV--IAGPK---DMVV----GFANLGILHVTKKKVFETLETRM	114
NXH08922.1	59	NGK----YVHLHAHSLVGKFCE-DGVCTV--LAGPK---DMVV----GFANLGILHVTKKKVFETLETRM	114
NWV89860.1	59	NGK----YVHLHAHSLVGKFCE-DGVCTV--NAGPK---DMVV----GFANLGILHVTKKKVFETLETRM	114
NWS31855.1	59	NGK----YVHLHAHSLVGKFCE-DGVCTV--NAGPK---DMVV----GFANLGILHVTKKKVFETLETRM	114
NXC72334.1	59	NGK----YVHLHAHSLVGKFCE-DGVCTV--NAGPK---DMVV----GFANLGILHVTKKKVFETLETRM	114
KAJ7408913.1	59	NGK----YVHLHAHSLVGKFCE-DGVCTV--NAGPK---DMVV----GFANLGILHVTKKKVLETLETRM	114
KFV88798.1	59	NGK----YVHLHAHSLVGKFCE-DGVCTV--NAGPK---DMVV----GFANLGILHVTKKKVFETLEARM	114
NXF34295.1	59	NGK----YVHLHAHSLVGKFCE-DGICTV--NAGPK---DMVV----GFANLGILHVTKKKVFETLETRM	114
NWR93222.1	59	NGK----YVHLHAHSLVGKFCE-DGVCTV--NAGPK---DMVV----GFANLGILHVTKKKVFETLETRM	114
NXR48213.1	59	NGK----YVHLHAHSLVGKFCE-DGVCTV--NAGPK---DMVV----GFANLGILHVTKKKVFETLETRM	114
NXY44121.1	51	NGK----YVHLHAHSLVGKYCE-DGVCTV--NAGPK---DMVV----GFANLGILHVTKKKVFETLETRM	106
_XP_009935286.1_	59	NGK----YVHLHAHSLVGKFCE-DGVCTV--NAGPK---DMVV----GFANLGILHVTKKKVFETLETRM	114
_XP_026704017.1_	59	NGK----YVHLHAHSLVGKFCE-DGVCTV--NAGPK---DMVV----GFANLGILHVTKKKVFETLETRM	114
_XP_009513483.1_	59	NGK----YVHLHAHSLVGKFCE-DGVCTV--NAGPK---DMVV----GFANLGILHVTKKKVFETLETRM	114
OXB77562.1	59	NGK----YVHLHAHSLVGKFCE-DGVCTV--NAGPK---DMVV----GFANLGILHVTKKKVFETLETRM	114
_XP_026647812.1_	59	NGK----YVHLHAHSLVGKFCE-DGVCTV--LAGPK---DMVV----GFANLGILHVTKKKVFETLETRM	114
NXC04840.1	59	NGK----YVHLHAHSLVGKFCE-DGVCTV--NAGPK---DMVV----GFANLGILHVTKKKVFETLETRM	114
_XP_009990600.1_	59	NGK----YVHLHAHSLVGKFCE-DGVCTV--NAGPK---DMVV----GFANLGILHVTKKKVFETLETRM	114
NXW31285.1	51	NGK----YVHLHAHSLVGKFCE-DGVCTV--NAGPK---DMVV----GFANLGILHVTKKKVFETLEARM	106
AAA49000.1	59	NGK----YRHLHAHSLVGKFCE-DGVCTV--NAGPK---DMVV----GFANLGILHVTKKKVFETLETRM	114
_XP_032540773.1_	59	NGK----YVHLHAHSLVGKFCE-DGVCTV--NAGPK---DMVV----GFANLGILHVTKKKVFETLETRM	114
_XP_054489767.1_	59	NGK----YVHLHAHSLVGKFCE-DGVCTV--LAGPK---DMVV----GFANLGILHVTKKKVFETLETRM	114
NXI38931.1	59	NGK----YVHLHAHSLVGKFCE-DGVCTV--NAGPK---DMVV----GFANLGILHVTKKKVFETLEARM	114
NWZ75946.1	51	NGK----YVHLHAHSLVGKFCE-DGVCTV--NAGPK---DMVV----GFANLGILHVTKKKVFETLETRM	106
TRZ15085.1	59	NGK----YVHLHAHSLVGKFCE-DGVCTV--NAGPK---DMVV----GFANLGILHVTKKKVFETLETRM	114
NWT43768.1	51	NGK----YVHLHAHSLVGKFCE-DGVCTV--NAGPK---DMVV----GFANLGILHVTKKKVFETLETRM	106
_XP_042667699.1_	59	NGK----YVHLHAHSLVGKFCE-DGVCTV--NAGPK---DMVV----GFANLGILHVTKKKVFETLETRM	114

```
_KFW75731.1_        59  NGK----YVHLHAHSLVGKFCE-DGVCTV--NAGPK---DMVV----GFANLGILHVTKKKVFETLETRM  114
_XP_048796949.1_    59  NGK----YVHLHAHSLVGKFCE-DGVCTV--NAGPK---DMVV----GFANLGILHVTKKKVFETLETRM  114
_NWR08112.1_        59  NGK----YVHLHAHSLVGKFCE-DGVCTV--NAGPK---DMVV----GFANLGILHVTKKKVFETLETRM  114
_KAJ7414413.1_      59  NGK----YVHLHAHSLVGKFCE-DGVCTV--NAGPK---DMVV----GFANLGILHVTKKKVFETLETRM  114
_NWI28654.1_        59  NGK----YVHLHAHSLVGKFCE-DGVCTV--NAGPK---DMVV----GFANLGILHVTKKKVFETLETRM  114
_NXQ74308.1_        59  NGK----YVHLHAHSLVGKFCE-DGVCTV--LAGPK---DMVV----GFANLGILHVTKKKVFETLETRM  114
_NWR61490.1_        59  NGK----YVHLHAHSLVGKFCE-DGVCTV--NAGPK---DMVV----GFANLGILHVTKKKVFETLETRM  114
_NXW00255.1_        51  NGK----YVHLHAHSLVGKFCE-DGVCTV--NAGPK---DMVV----GFANLGILHVTKKKVFETLETRM  106
_XP_010131965.1_    59  NGK----YVHLHAHSLVGKFCE-DGVCTV--NAGPK---DMVV----GFANLGILHVTKKKVLETLETRM  114
_KAF1499115.1_      51  NGK----YVHLHAHSLVGKFCE-DGVCTV--NAGPK---DMVV----GFANLGILHVTKKKVFETLETRM  106
_XP_054018945.1_    59  NGK----YVHLHAHSLVGKFCE-DGVCTV--NAGPK---DMVV----GFANLGILHVTKKKVFETLEARM  114
_XP_052539264.1_    59  NGK----YVHLHAHSLVGKFCE-DGVCTV--NAGPK---DMVV----GFANLGILHVTKKKVFETLETRM  114
_NXC30330.1_        59  NGK----YVHLHAHSLVGKFCE-DGVCTV--NAGPK---DMVV----GFANLGILHVTKKKVFETLETRM  114
_KFR08581.1_        59  NGK----YVHLHAHSLVGKFCE-DGVCTV--NAGPK---DMVV----GFANLGILHVTKKKVFETLETRM  114
_XP_039557931.1_    59  NGK----YVHLHAHSLVGKFCE-DGVCTV--LAGPK---DMVV----GFANLGILHVTKKKVFETLETRM  114
_KAF1486784.1_      51  NGK----YVHLHAHSLVGKFCE-DGVCTV--NAGPK---DMVV----GFANLGILHVTKKKVFETLETRM  106
_OXB59608.1_        59  NGK----YVHLHAHSLVGKFCE-DGVCTV--NAGPK---DMVV----GFANLGILHVTKKKVFETLETRM  114
_XP_021138721.1_    59  NGK----YVHLHAHSLVGKFCE-DGVCTV--NAGPK---DMVV----GFANLGILHVTKKKVFETLEARM  114
_KAF1514432.1_      51  NGK----YVHLHAHSLVGKFCE-DGVCTV--NAGPK---DMVV----GFANLGILHVTKKKVFETLETRM  106
_NWV14170.1_        59  NGK----YVHLHAHSLVGKFCE-DGVCTV--NAGPK---DMVV----GFANLGILHVTKKKVFETLETRM  114
_KAF1548068.1_      51  NGK----YVHLHAHSLVGKFCE-DGVCTV--NAGPK---DMVV----GFANLGILHVTKKKVFETLETRM  106
_NXI51730.1_        59  NGK----YVHLHAHSLVGKFCE-DGVCTV--NAGPK---DMVV----GFANLGILHVTKKKVFETLETRM  114
_NXE50896.1_        51  NGK----YVHLHAHSLVGKFCE-DGVCTV--NAGPK---DMVV----GFANLGILHVTKKKVFETLEARM  106
_NXT41700.1_        51  NGK----YAHLHAHSLVGKFCE-DGVCTV--NAGPK---DMVV----GFANLGILHVTKKKVFETLETRM  106
_KAF1659387.1_      51  NGK----YVHLHAHSLVGKFCE-DGVCTV--NAGPK---DMVV----GFANLGILHVTKKKVFETLETRM  106
_NWR74867.1_        51  NGK----YVHLHAHSLVGKYCE-DGICTV--NAGPK---DMVV----GFANLGILHVTKKKVFETLETRM  106
_NXV33343.1_        51  NGK----YVHLHAHSLVGKFCE-DGVCTV--NAGPK---DMVV----GFANLGILHVTKKKVFETLETRM  106
_NXW99502.1_        51  NGK----YVHLHAHSLVGKFCE-DGVCTV--NAGPK---DMVV----GFANLGILHVTKKKVFETLETRM  106
_NXV14161.1_        51  NGK----YVHLHAHSLVGKFCE-DGVCTV--NAGPK---DMVV----GFANLGILHVTKKKVFETLETRM  106
_NWX63354.1_        51  NGK----YVHLHAHSLVGKFCE-DGVCTV--SAGPK---DMVV----GFANLGILHVTKKKVFETLETRM  106
_NXL58318.1_        51  NGK----YVHLHAHSLVGKFCE-DGVCTV--NAGPK---DMVV----GFANLGILHVTKKKVFETLESRM  106
_NWV41036.1_        51  NGK----YVHLHAHSLVGKFCE-DGVCTV--NAGPK---DMVV----GFANLGILHVTKKKVFETLETRM  106
_KAF1609011.1_      51  NGK----YVHLHAHSLVGKFCE-DGVCTV--NAGPK---DMVV----GFANLGILHVTKKKVFETLETRM  106
_NWT52190.1_        59  NGK----YVHLHAHSLVGKFCE-DGVCTV--NAGPK---DMVV----GFANLGILHVTKKKVFETLETRM  114
_NXO93770.1_        59  NGK----YVHLHAHSLVGKFCE-DGVCTV--TAGPK---DMVV----GFANLGILHVTKKKVFETLETRM  114
_NXI62681.1_        59  NGK----YVHLHAHSLVGKFCE-DGVCTV--NAGPK---DMVV----GFANLGILHVTKKKVFETLETRM  114
_NXH21058.1_        59  NGK----YVHLHAHSLVGKFCE-DGVCTV--NAGPK---DMVV----GFANLGILHVTKKKVFETLEARM  114
_KFV75676.1_        59  NGK----YVHLHAHSLVGKFCE-DGVCTV--NAGPK---DMVV----GFANLGILHVTKKKVFETLEARM  114
_KAF1435617.1_      51  NGK----YVHLHAHSLVGKFCE-DGVCTV--NAGPK---DMVV----GFANLGILHVTKKKVFETLETRM  106
```

```
_KAF1479350.1_      51  NGK----YVHLHAHSLVGKFCE-DGVCTV--NAGPK---DMVV----GFANLGILHVTKKKVFETLETRM  106
_NXW86014.1_        51  NGK----YVHLHAHSLVGKFCE-DGVCTV--NAGPK---DMVV----GFANLGILHVTKKKVFETLEARM  106
_KAF1483835.1_      51  NGK----YVHLHAHSLVGKFCE-DGVCTV--NAGPK---DMVV----GFANLGILHVTKKKVFETLETRM  106
_NWW87838.1_        59  NGK----YVHLHAHSLVGKFCE-DGVCTV--NAGPK---DMVV----GFANLGILHVTKKKVFETLETRM  114
_NXF79309.1_        59  NGK----YVHLHAHSLVGKFCE-DGVCTV--NAGPK---DMVV----GFANLGILHVTKKKVFETLETRM  114
_XP_030305668.1_    59  NGK----YVHLHAHSLVGKFCE-DGVCTV--NAGPK---DMVV----GFANLGILHVTKKKVFETLESRM  114
_NXS27685.1_        59  NGK----YVHLHAHSLVGKFCE-DGVCTV--NAGPK---DMVV----GFANLGILHVTKKKVFETLETRM  114
_XP_036237629.1_    59  NGK----YVHLHAHSLVGKFCE-DGVCTV--LAGPK---DMVV----GFANLGILHVTKKKVFETLETRM  114
_NXM97765.1_        59  NGK----YVHLHAHSLVGKFCE-DGVCTV--NAGPK---DMVV----GFANLGILHVTKKKVFETLETRM  114
_KAF2988548.1_      59  NGK----YVHLHAHSLVGKFCE-DGVCTV--LAGPK---DMVV----GFANLGILHVTKKKVFETLETRM  114
_NWH87727.1_        59  NGK----YVHLHAHSLVGKFCE-DGVCTV--NAGPK---DMVV----GFANLGILHVTKKKVFETLETRM  114
_NXR25130.1_        51  NGK----YVHLHAHSLVGKFCE-DGVCTV--TAGPK---DMVV----GFANLGILHVTKKKVFETLETRM  106
_NXV50350.1_        51  NGK----YVHLHAHSLVGKFCE-DGVCTV--NAGPK---DMVV----GFANLGILHVTKKKVFETLETRM  106
_NWT72232.1_        51  NGK----YVHLHAHSLVGKSCE-DGVCTV--LAGPK---DMVV----GFANLGILHVTKKKVFETLETRM  106
_KAF1435460.1_      51  NGK----YVHLHAHSLVGKFCE-DGVCTV--NAGPK---DMVV----GFANLGILHVTKKKVFETLETRM  106
_KAF1424376.1_      51  NGK----YVHLHAHSLVGKFCE-DGVCTV--NAGPK---DMVV----GFANLGILHVTKKKVFETLETRM  106
_NWX41614.1_        51  NGK----YVHLHAHSLVGKFCE-DGVCTV--NAGPK---DMVV----GFANLGILHVTKKKVFETLETRM  106
_NXD28887.1_        51  NGK----YVHLHAHSLVGKFCE-DGVCTV--NAGPK---DMVV----GFANLGILHVTKKKVFETLETRM  106
_NXY59218.1_        51  NGK----YVHLHAHSLVGKFCE-DGVCTV--NAGPK---DMVV----GFANLGILHVTKKKVFETLETRM  106
_KAF1399315.1_      51  NGK----YVHLHAHSLVGKFCE-DGVCTV--NAGPK---DMVV----GFANLGILHVTKKKVFETLETRM  106
_NXJ95672.1_        51  NGK----YVHLHAHSLVGKFCE-DGVCTV--NAGPK---DMVV----GFANLGILHVTKKKVFETLETRM  106
_NWW19682.1_        51  NGK----YVHLHAHSLVGKFCE-DGVCTV--NAGPK---DMVV----GFANLGILHVTKKKVFETLETRM  106
_NWU76879.1_        51  NGK----YVHLHAHSLVGKFCE-DGVCTV--NAGPK---DMVV----GFANLGILHVTKKKVFETLETRM  106
_NWY75431.1_        51  NGK----YVHLHAHSLVGKFCE-DGVCTV--TAGPK---DMVV----GFANLGILHVTKKKVFETLETRM  106
_NXX32566.1_        51  NGK----YVHLHAHSLVGKFCE-DGVCTV--NAGPK---DMVV----GFANLGILHVTKKKVFETLETRM  106
_NXL48469.1_        51  NGK----CVHLHAHSLVGKFCE-DGVCTV--NAGPK---DMVV----GFANLGILHVTKKKVFETLETRM  106
_NXN66426.1_        51  NGK----YVHLHAHSLVGKFCE-DGVCTV--NAGPK---DMVV----GFANLGILHVTKKKVFETLETRM  106
_NXV94077.1_        51  NGK----YVHLHAHSLVGKFCE-DGVCTV--NAGPK---DMVV----GFANLGILHVTKKKVFETLETRM  106
_KFO88230.1_        59  NGK----YVHLHAHSLVGKFCE-DGVCTV--NAGPK---DMVV----GFANLGILHVTKKKVLETLETRM  114
_NXD82005.1_        59  NGK----YVHLHAHSLVGKFCE-DGVCTV--NAGPK---DMVV----GFANLGILHVTKKKVFETLETRM  114
_NWY36867.1_        51  NGK----YVHLHAHSLVGKFCE-DGVCTV--NAGPK---DMVV----GFANLGILHVTKKKVFETLETRM  106
_NWZ53691.1_        51  NGK----YVHLHAHSLVGKFCE-DGVCTV--NAGPK---DMVV----GFANLGILHVTKKKVFETLETRM  106
_NXE95961.1_        51  NGK----YVHLHAHSLVGKFCE-DGVCTV--NAGPK---DMVV----GFANLGILHVTKKKVFETLETRM  106
_NXB76170.1_        51  NGK----YVHLHAHSLVGKFCE-DGVCTV--SAGPK---DMVV----GFANLGILHVTKKKVFETLETRM  106
_NWR51229.1_        51  NGK----YVHLHAHSLVGKSCE-DGVCTV--IAGPK---DMVV----GFANLGILHVTKKKVLETLETRM  106
_NXB96832.1_        51  NGK----YVHLHAHSLVGKFCE-DGVCTV--IAGPK---DMVV----GFANLGILHVTKKKVFETLEARM  106
_NXW19741.1_        51  NGK----YVHLHAHSLVGKFCE-DGVCTV--NAGPK---DMVV----GFANLGILHVTKKKVFETLETRM  106
_NWV61316.1_        51  NGK----YVHLHAHSLVGKFCE-DGVCTV--NAGPK---DMVV----GFANLGILHVTKKKVFETLETRM  106
_NXD68485.1_        51  NGK----YVHLHAHSLVGKFCE-DGVCTV--NAGPK---DMVV----GFANLGILHVTKKKVFETLETRM  106
```

```
_XP_038033784.1_    59  NGK----YVHLHAHSLVGKFCE-DGVCTV--NAGPK---DMVV----GFANLGILHVTKKKVFETLETRM  114
_XP_009918158.1_    59  NGK----YVHLHAHSLVGKFCE-DGVCTV--NAGPK---DMVV----GFANLGILHVTKKKVFETLETRM  114
_NWI62302.1_        51  NGK----YVHLHAHSLVGKYCE-DGVCTV--NAGPK---DMVV----GFANLGILHVTKKKVFETLEARM  106
_NXV82871.1_        51  NGK----CVHLHAHSLVGKFCE-DGVCTV--NAGPK---DMVV----GFANLGILHVTKKKVFETLETRM  106
_NXX21988.1_        51  NGK----YVHLHAHSLVGKFCE-DGVCTV--NAGPK---DMVV----GFANLGILHVTKKKVFETLETRM  106
_NWZ28840.1_        59  NGK----YVHLHAHSLVGKFCE-DGVCTV--NAGPK---DMVV----GFANLGILHVTKKKVFETLETRM  114
_NXA68287.1_        51  NGK----YVHLHAHSLVGKFCE-DGVCTV--NAGPK---DMVV----GFANLGILHVTKKKVFETLETRM  106
_NXL73547.1_        50  NGK----YVHLHAHSLVGKFCE-DGVCTV--IAGPK---DMVV----GFANLGILHVTKKKVFETLETRM  105
_NXV08833.1_        51  NGK----YVHLHAHSLVGKFCE-DGVCTV--NAGPK---DMVV----GFANLGILHVTKKKVFETLETRM  106
_NWY20334.1_        51  NGK----YVHLHAHSLVGKFCE-DGVCTV--NAGPK---DMVV----GFANLGILHVTKKKVLETLETRM  106
_NXA13401.1_        51  NGK----YVHLHAHSLVGKFCE-DGVCTV--NAGPK---DMVV----GFANLGILHVTKKKVFETLETRM  106
_NXY15168.1_        51  NGK----YVHLHAHSLVGKFCE-DGVCTV--NAGPK---DMVV----GFANLGILHVTKKKVFETLETRM  106
_NXJ48973.1_        59  NGK----YVHLHAHSLVGKFCE-DGVCTV--NAGPK---DMVV----GFANLGILHVTKKKVFETLETRM  114
_KAI6070014.1_      59  NGK----YVHLHAHSLVGKFCE-DGVCTV--NAGPK---DMVV----GFANLGILHVTKKKVFETLETRM  114
_NXH91756.1_        50  NGK----YVHLHAHSLVGKFCE-DGVCTV--NAGPK---DMVV----GFANLGILHVTKKKVFETLETRM  105
_NXL04686.1_        50  NGK----YVHLHAHSLVGKFCE-DGVCTV--NAGPK---DMVV----GFANLGILHVTKKKVFETLETRM  105
_XP_040411521.1_    59  NGK----YVHLHAHSLVGKFCE-DGVCTV--NAGPK---DMVV----GFANLGILHVTKKKVFETLETRM  114
_XP_035395635.1_    59  NGK----YVHLHAHSLVGKFCE-DGVCTV--NAGPK---DMVV----GFANLGILHVTKKKVFETLETRM  114
_NXU37184.1_        51  NGK----YVHLHAHSLVGKFCE-DGVCTV--NAGPK---DMVV----GFANLGILHVTKKKVFETLETRM  106
_NWV21895.1_        51  NGK----YVHLHAHSLVGKCCE-DGVCTV--NAGPK---DMVV----GFANLGILHVTKKKVFETLETRM  106
_NXY81878.1_        59  NGK----YVHLHAHSLVGKFCE-DGVCTV--NAGPK---DMVV----GFANLGILHVTKKKVFETLETRM  114
_NXH41439.1_        50  NGK----YVHLHAHSLVGKFCE-DGVCTV--IAGPK---DMVV----GFANLGILHVTKKKVFETLETRM  105
_NWW81943.1_        51  NGK----YVHLHAHSLVGKFCE-DGVCTV--NAGPK---DMVV----GFANLGILHVTKKKVFETLETRM  106
_NXJ61295.1_        51  NGK----YVHLHAHSLVGKFCE-DGVCTV--NAGPK---DMVV----GFANLGILHVTKKKVFETLETRM  106
_NXN34085.1_        51  NGK----YVHLHAHSLVGKFCE-DGVCTV--NAGPK---DMVV----GFANLGILHVTKKKVFETLETRM  106
_NXK62748.1_        52  NGK----YVHLHAHSLVGKFCE-DGVCTV--NAGPK---EMVV----GFANLGILHVTKKKVFETLETRM  107
_NWH66460.1_        51  NGK----YVHLHAHSLVGKYCE-DGVCTV--NAGPK---DMVV----GFANLGILHVTKKKVFETLEARM  106
_NWS97842.1_        51  NGK----YVHLHAHSLVGKFCE-DGVCTV--NAGPK---DMVV----GFANLGILHVTKKKVFETLETRM  106
_XP_013051206.2_    59  NGK----YVHLHAHSLVGKFCE-DGVCTV--NAGPK---DMVV----GFANLGILHVTKKKVFETLETRM  114
_NWY58828.1_        51  NGK----YVHLHAHSLVGKFCE-DGVCTV--NAGPK---DMVV----GFANLGILHVTKKKVFETLETRM  106
_NWR27345.1_        51  NGK----YVHLHAHSLVGKFCE-DGVCTV--NAGPK---DMVV----GFANLGILHVTKKKVFETLETRM  106
_NXM04963.1_        50  NGK----YVHLHAHSLVGKFCE-DGVCTV--NAGPK---DMVV----GFANLGILHVTKKKVFETLETRM  105
_NWZ13908.1_        51  NGK----YVHLHAHSLVGKFCE-DGVCTV--LAGPK---DMVV----GFANLGILHVTKKKVFETLETRM  106
_NWI56539.1_        51  NGK----YVHLHAHSLVGKFCE-DGVCTV--NAGPK---DMVV----GFANLGILHVTKKKVFETLETRM  106
_NXL35478.1_        51  NGK----YVHLHAHSLVGKFCE-DGVCTV--NAGPK---DMVV----GFANLGILHVTKKKVFETLETRM  106
_NWY90605.1_        51  NGK----YVHLHAHSLVGKFCE-DGVCTV--LAGPK---DMVV----GFANLGILHVTKKKVFETLETRM  106
_NXF23305.1_        51  NGK----YVHLHAHSLVGKFCE-DGVCTV--LAGPK---DMVV----GFANLGILHVTKKKVFETLETRM  106
_XP_035182229.1_    59  NGK----YVHLHAHSLVGKFCE-DGVCTV--NAGPK---DMVV----GFANLGILHVTKKKVFETLETRM  114
_EOB02688.1_        59  NGK----YVHLHAHSLVGKFCE-DGVCTV--NAGPK---DMVV----GFANLGILHVTKKKVFETLETRM  114
```

```
_NXP86430.1_       51  NGK----YVHLHAHSLVGKFCE-DGVCTV--LAGPK---DMVV----GFANLGILHVTKKKVFETLETRM  106
_NXK90683.1_       59  NGK----YVHLHAHSLVGKFCE-DGVCTV--NAGPK---DMVV----GFSNLGILHVTKKKVFETLESRM  114
_NWU68452.1_       51  NGK----YVHLHAHSLVGKFCE-DGVCTV--NAGPK---DMVV----GFANLGILHVTKKKVFETLEARM  106
_NXT19433.1_       51  NGK----YVHLHAHSLVGKFCE-DGVCMV--NAGPK---DMVV----GFANLGILHVTKKKVFETLEARM  106
_NXV56948.1_       51  NGK----YVHLHAHSLVGKFCE-DGVCTV--LAGPK---DMVV----GFANLGILHVTKKKVFETLETRM  106
_NXX85020.1_       51  NGK----YVHLHAHSLVGKFCE-DGVCTV--NAGPK---DMVV----GFANLGILHVTKKKVFETLETRM  106
_NWY25110.1_       51  NGK----YVHLHAHSLVGKFCE-DGVCTV--LAGPK---DMVV----GFANLGILHVTKKKVFETLETRM  106
_XP_032041859.1_   59  NGK----YVHLHAHSLVGKFCE-DGVCTV--NAGPK---DMVV----GFANLGILHVTKKKVFETLETRM  114
_NWZ68267.1_       51  NGK----YVHLHAHSLVGKFCE-DGVCTV--NAGPK---DMVV----GFANLGILHVTKKKVFETLETRM  106
_NXG60352.1_       51  NGK----YVHLHAHSLVGKFCE-DGVCTV--NAGPK---DMVV----GFANLGILHVTKKKVLETLEMRM  106
_NXT59875.1_       51  NGK----YVHLHAHSLVGKFCE-DGVCTV--NAGPK---DMVV----GFANLGILHVTKKKVFETLETRM  106
_NWY04604.1_       51  NGK----YVHLHAHSLVGKFCE-DGVCTV--NAGPK---DMVV----GFANLGILHVTKKKVFETLEARM  106
_NXC95671.1_       51  NGK----YVHLHAHSLVGKFCE-DGVCTV--TAGPK---DMVV----GFANLGILHVTKKKVFETLETRM  106
_NXD14679.1_       51  NGK----YVHLHAHSLVGKFCE-DGVCTV--NAGPK---DMVV----GFANLGILHVTKKKVFETLEARM  106
_NWX87899.1_       51  NGK----YVHLHAHSLVGKFCE-DGVCTV--NAGPK---DMVV----GFANLGILHVTKKKVFETLEARM  106
_NWZ96914.1_       51  NGK----YVHLHAHSLVGKFCE-DGVCTV--LAGPK---DMVV----GFANLGILHVTKKKVFETLETRM  106
_KFP08036.1_       59  NGK----YVHLHAHSLVGKFCE-DGVCTV--NAGPK---DMVV----GFANLGILHVTKKKVFETLESRM  114
_NXJ81100.1_       51  NGK----YVHLHAHSLVGKFCE-DGVCTV--NAGPK---DMVV----GFANLGILHVTKKKVFETLETRM  106
_KAF1419467.1_     51  NGK----YVHLHAHSLVGKFCE-DGVCTV--NAGPK---DMVV----GFANLGILHVTKKKVFETLETRM  106
_NXA54431.1_       51  NGK----YVHLHAHSLVGKFCE-DGVCTV--NAGPK---DMVV----GFANLGILHVTKKKVFETLEARM  106
_NXI22500.1_       50  NGK----YVHLHAHSLVGKFCE-DGVCTV--NAGPK---DMVV----GFANLGILHVTKKKVFETLETRM  105
_XP_030348001.1_   59  NGK----YVHLHAHSLVGKFCE-DGVCTV--NAGPK---DMVV----GFANLGILHVTKKKVFETLETRM  114
_XP_032041862.1_   59  NGK----YVHLHAHSLVGKFCE-DGVCTV--NAGPK---DMVV----GFANLGILHVTKKKVFETLETRM  114
_XP_054059792.1_   59  NGK----YVHLHAHSLVGKFCE-DGVCTV--NAGPK---DMVV----GFANLGILHVTKKKVFETLETRM  114
_NWI98852.1_       51  NGK----YVHLHAHSLVGKFCE-DGVCTV--NAGPK---DMVV----GFANLGILHVTKKKVFETLEARM  106
_XP_040979578.1_   59  NGK----YVHLHAHSLVGKFCE-DGVCTV--NAGPK---DMVV----GFANLGILHVTKKKVFETLETRM  114
_XP_035182231.1_   59  NGK----YVHLHAHSLVGKFCE-DGVCTV--NAGPK---DMVV----GFANLGILHVTKKKVFETLETRM  114
_NWI17161.1_       51  NGK----YVHLHAHSLVGKFCE-DGVCTV--NAGPK---DMVV----GFANLGILHVTKKKVFETLEARM  106
_XP_017677707.1_   59  NGK----YVHLHAHSLVGKFCE-DGVCTV--NAGPK---DMVV----GFANLGILHVTKKKVFETLETRM  114
_XP_031966434.1_   59  NGK----YVHLHAHSLVGKFCE-DGVCTV--NAGPK---DMVV----GFANLGILHVTKKKVLETLETRM  114
_XP_014795990.1_   59  NGK----YVHLHAHSLVGKFCE-DGVCTV--NAGPK---DMVV----GFANLGILHVTKKKVFETLETRM  114
_XP_041905596.1_   59  NGK----YVHLHAHSLVGKFCE-DGVCTV--NAGPK---DMVV----GFANLGILHVTKKKVLETLETRM  114
_XP_039406723.1_   59  NGK----YVHLHAHSLVGKFCE-DGVCTV--NAGPK---DMVV----GFANLGILHVTKKKVLETLETRM  114
_XP_048159386.1_   59  NGK----YVHLHAHSLVGKFCE-DGVCTV--NAGPK---DMVV----GFANLGILHVTKKKVLETLETRM  114
_NXA36485.1_       51  NGK----YVHLHAHSLVGKFCE-DGVCTV--NAGPK---DMVV----GFANLGILHVTKKKVFETLEARM  106
_XP_010001479.1_   59  NGK----YVHLHAHSLVGKFCE-DGVCTV--NAGPK---DMVV----GFANLGILHVTKKKVLETLETRM  114
_KFU93771.1_       59  NGK----YVHLHAHSLVGKFCE-DGVCTV--NAGPK---DMVV----GFANLGILHVTKKKVLETLETRM  114
_NXE65168.1_       51  NGK----YVHLHAHSLVGKFCE-DGVCTV--LAGPK---DMVV----GFANLGILHVTKKKVFETLETRM  106
_KAF4794888.1_     59  NGK----YVHLHAHSLVGKFCE-DGVCTV--TAGPK---DMVV----GFANLGILHVTKKKVFETLETRM  114
```

```
_XP_027532934.1_   59  NGK----YVHLHAHSLVGKFCE-DGVCTV--NAGPK---DMVV----GFANLGILHVTKKKVFETLETRM  114
_XP_046795876.1_   59  NGK----YVHLHAHSLVGKFCE-DGVCTV--NAGPK---DMVV----GFANLGILHVTKKKVFETLETRM  114
_KFV96277.1_       59  NGK----YVHLHAHSLVGKFCE-DGVCTV--NAGPK---DMVV----GFANLGILHVTKKKVFETLETRM  114
_XP_010157325.1_   59  NGK----YVHLHAHSLVGKFCE-DGVCTV--NAGPK---DMVV----GFANLGILHVTKKKVFETLETRM  114
_NWI02032.1_       59  NGK----YVHLHAHSLVGKFCE-DGVCTV--NAGPK---DMVV----GFANLGILHVTKKKVFETLETRM  114
_XP_035746225.1_   59  NGK----YVHLHAHSLVGKFCE-DGVCTV--NAGPK---DMVV----GFANLGILHVTKKKVFETLETRM  114
_NWH69936.1_       51  NGK----YVHLHAHSLVGKYCE-DGVCTV--NAGPK---DMVV----GFANLGILHVTKKKVFETLETRM  106
_NXH96653.1_       50  NGK----YVHLHAHSLVGKFCE-DGVCTV--NAGPK---DMVV----GFANLGILHVTKKKVFETLETRM  105
_NXN58828.1_       59  NGK----YVHLHAHSLVGKFCE-DGVCTV--NAGPK---DMVV----GFANLGILHVTKKKVFETLETRM  114
_XP_010083670.1_   59  NGK----YVHLHAHSLVGKFCE-DGVCTV--NAGPK---DMVV----GFANLGILHVTKKKVFETLEARM  114
_RMC13788.1_       59  NGK----YVHLHAHSLVGKFCE-DGVCTV--NAGPK---DMVV----GFANLGILHVTKKKVFETLETRM  114
_XP_023801002.1_       ----------------------------------------------------------------------
_KAJ1206281.1_     59  SGK----DVHLHAHSLVGKHCE-DGICTV--TVGPK---DMIV----GFANLGILHVTKKKVFDTLESRM  114
_XP_029452911.1_   59  QGK----DVRLHAHSLVGKHCE-DGICTV--TAGPK---DMVV----GFPNLGILHVTKKRVFETLNSRM  114
_XP_030046614.1_   59  NSK----EVRLHAHSLVGKHCE-DGICTV--TAGPK---DMLM----GFPNLGILHVTKKKVFETLNSRM  114
_XP_033791760.1_   59  SSK----EVRLHAHSLVGKHCE-DGICTV--PAGPK---DMIV----GFPNLGILHVTKKKVFETLNSRM  114
_NXS74679.1_           ----------------------------------------------------------------------
_XP_019782049.1_    1  ------------------CHLT-PVRMAV--IKENT---KNKC----CFANLGILHVTKKKVFETLEARM   42
_XP_030700974.1_    1  ------------------CHLT-PVRMAV--IKENT---KNKC----CFANLGILHVTKKKVFETLEARM   42
_XP_026972817.1_    1  ------------------CHLT-PVRMAV--IKENT---KNKC----CFANLGILHVTKKKVFETLEARM   42
_XP_042107134.1_    1  ----------------------------------SP---TLRS----NFANLGILHVTKKKVFETLEARM   29
_XP_016807420.1_    1  ----------------------------------------MVV----GFANLGILHVTKKKVFETLEARM   26
_XP_031520261.1_    1  -----------------------------------------------CFANLGILHVTKKKVFETLEARM   23
_XP_025146162.3_    1  ---------------------------------------TLRS----NFANLGILHVTKKKVFETLEARM   27
_MXQ81613.1_        1  ---------------------------------------TFLL----SFANLGILHVTKKKVFETLEARM   27
_XP_014391951.1_    1  ---------------------------------------TLRS----NFANLGILHVTKKKVFETLEARM   27
_KAF5926647.1_      1  ---------------------------------------RMIQ----NFANLGILHVTKKKVFETLEARM   27
_XP_014315807.1_    1  ---------------------------------------TLRS----NFANLGILHVTKKKVFETLEARM   27
_XP_044243992.1_    1  ----------------------------------------MVV----GFANLGILHVTKKKVFETLEARM   26
_XP_015425475.1_    1  ---------------------------------------TLRS----NFANLGILHVTKKKVFETLEARM   27
_XP_036196822.1_    1  ---------------------------------------TLRS----NFANLGILHVTKKKVFETLEARM   27
_XP_036266201.1_    1  ---------------------------------------TLRS----NFANLGILHVTKKKVYETLEARM   27
_XP_025729301.1_    1  ----------------------------------------MVV----GFANLGILHVTKKKVFETLEARM   26
_XP_051041401.1_    1  ---------------------------------------TLRS----NFANLGILHVTKKKVFETLEARM   27
_XP_029330831.1_    1  ----------------------------------------MVV----GFANLGILHVTKKKVFETLEARM   26
_XP_021051020.1_    1  ----------------------------------------MVV----GFANLGILHVTKKKVFETLEARM   26
_XP_023493402.1_    1  ----------------------------------------MVV----GFANLGILHVTKKKVFETLEARM   26
_XP_021101132.1_    1  ----------------------------------------MVT----SFANLGILHVTKKKVFETLETRM   26
_BAC35117.1_        1  ----------------------------------------MVV----GFANLGILHVTKKKVFETLEARM   26
```

```
_XP_006732650.2_     1  ------------------------------------------S----SFANLGILHVTKKKVFETLEARM   24
_KAB0368408.1_      24  NGK----NIHLHAHSLVGKHCE-DGICTV--TAGPK---DMVV----GFANLGILHVTKKKVFETLEARM   79
_XP_030675033.1_    21  NGK----NIHLHAHSLVGKHCE-DGICTV--TAGPK---DMVV----GFANLGILHVTKKKVFETLEARM   76
_XP_012291953.1_    18  NGK----HIHLHAHSLVGKHCE-DGICTV--TAGPK---DMVV----GFANLGILHVTKKKVFETLEARM   73
_XP_047422690.1_    18  NGK----NIHLHAHSLVGKHCE-DGICTV--NAGPK---DMVV----GFANLGILHVTKKKVFETLEARM   73
_XP_051675507.1_    21  NGK----NIHLHAHSLVGKHCE-DGICTV--NAGPK---DMVV----SFANLGILHVTKKKVFETLEARM   76
_XP_025146160.1_    21  NGK----NIHLHAHSLVGKHCE-DGVCTV--TAGPK---DMVV----GFANLGILHVTKKKVFETLEARM   76
_KAB0345267.1_      17  NGK----NIHLHAHSLVGKHCE-DGICTV--TAGPK---DMVV----GFANLGILHVTKKKVFETLEARM   72
_XP_020014165.1_    20  NGK----NIHLHAHSLVGKHCE-DGICTV--TAGPK---DMVV----GFANLGILHVTKKKVFETLEARM   75
_XP_026917376.1_    18  NGK----NIHLHAHSLVGKHCE-DGICTV--TAGPK---DMVV----GFANLGILHVTKKKVFETLEARM   73
_XP_019271516.2_    18  NGK----NIHLHAHSLVGKHCE-DGICTV--TAGPK---DMVV----GFANLGILHVTKKKVFETLEARM   73
_XP_045327688.1_    18  NGK----NIHLHAHSLVGKHCE-DGICTV--TAGPK---DMVV----GFANLGILHVTKKKVFETLEARM   73
_XP_023108675.2_    18  NGK----NIHLHAHSLVGKHCE-DGICTV--TAGPK---DMVV----GFANLGILHVTKKKVFETLEARM   73
_XP_014391950.1_    12  NGK----SIHLHAHSLVGKHCE-DGICTV--TAGPK---DMVV----GFANLGILHVTKKKVFETLEARM   67
_XP_023614158.1_    12  NGK----SIHLHAHSLVGKHCE-DGICTV--TAGPK---DMVV----GFANLGILHVTKKKVFETLEARM   67
_XP_032256797.1_    21  NGK----NIHLHAHSLVGKHCE-DGICTV--TAGPK---DMVV----GFANLGILHVTKKKVFETLEARM   76
_XP_015425474.1_    12  NGK----SIHLHAHSLVGKHCE-DGICTV--TAGPK---DMVV----GFANLGILHVTKKKVFETLEARM   67
_XP_027709164.1_    19  NGK----HVHLHAHSLVGKHCE-DGICTV--TAGPK---DMVV----GFANLGILHVTKKKVYETLEARM   74
_KAG8519429.1_      21  NGK----SVHLHAHSLVGKHCE-DGVCTV--TAGPK---DMVV----GFANLGILHVTKKKVFETLEARM   76
_KAB1282111.1_      18  NGK----NIHLHAHSLVGKHCE-DGICTV--TAGPK---DMVV----GFANLGILHVTKKKVFETLEARM   73
_XP_016049280.1_     1  ----------------------------------------MVV----GFANLGILHVTKKKVFETLEARM   26
_OBS59044.1_            ----------------------------------------------------------------------
_KAI2535356.1_          ----------------------------------------------------------------------
_XP_054846407.1_    59  NGK----QPHLHAHSLVGKHCE-NGVCTV--NVGPK---DVVV----GFPNLGILHVTKKRVLDTLEARM  114
_XP_015261255.1_    59  NGK----QPHLHAHSLVGKHCE-NGICTV--NAGPK---DTVV----GFPNLGILHVTKKRVFDTLEARM  114
_XP_028599456.1_    59  NGK----HAHLHAHSLVGKHCE-NGVCTV--NAGPK---DMVV----GFPNLGILHVTKKRVLDTLEARM  114
_QCQ80695.1_        59  NGK----HAHLHAHSLVGKHCE-NGVCTV--NAGPK---DMVV----GFPNLGILHVTKKRVLDTLEARM  114
_XP_048365177.1_    59  NGK----QPHLHAHSLVGKHCE-NGICTV--NAGPK---DTVV----GFPNLGILHVTKKRVFDTLEARM  114
_EGW14115.1_            ----------------------------------------------------------------------
_KAJ6668884.1_       1  ----------------------------------------QKI----SFPNLGILHVTKKRVLDTLESRM   26
_XP_053108974.1_    59  NGK----HAHLHAHSLVGKHCE-NGVCTA--NAGPK---DMVV----GFPNLGILHVTKKRVFDTLEARM  114
_XP_007429861.1_    59  NGK----HVHLHAHSLVGKHCE-QGVCTV--NAGPK---DMVV----GFPNLGILHVTKKRVLDTLETRM  114
_XP_047654926.1_        ----------------------------------------------------------------------
_XP_011884266.1_        ----------------------------------------------------------------------
_KAI2535355.1_          ----------------------------------------------------------------------
_PNJ84388.1_            ----------------------------------------------------------------------
_XP_038167493.1_        ----------------------------------------------------------------------
_ERE89661.1_            ----------------------------------------------------------------------
_XP_038167494.1_        ----------------------------------------------------------------------
```

```
_KAH0615883.1_      59  NGK----QVHLHAHSLVGKHCE-QGVCTV--NAGPK---DMVV----GFPNLGILHVTKKRVLDTLETRM  114
_XP_020661083.1_    59  NGK----HVHLHAHSLVGKHCE-QGVCTV--NAGPK---DMVV----GFPNLGILHVTKKRVLDTLEARM  114
_XP_053259916.1_    59  NGK----HAHLHAHSLVGKHCE-NGVCTV--NAGPK---DMVV----GFPNLGILHVTKKRVLDTLEARM  114
_XP_044284768.1_    59  NGK----HPHLHAHSLVGKHCE-QGVCTV--NAGPK---DMVV----GFPNLGILHVTKKKVLETLETRM  114
_XP_034967560.1_    59  NGK----HAHLHAHSLVGKHCE-NGVCTV--NAGPK---DMVV----GFPNLGILHVTKKRVLDTLEARM  114
_XP_033016273.1_    59  NGK----HAHLHAHSLVGKHCE-NGVCTV--NAGPK---DMVV----GFPNLGILHVTKKRVLDTLEARM  114
_KAJ7327088.1_      59  NGK----HARLHAHSLVGKHCE-QGVCTV--NAGPK---DMVV----GFPNLGILHVTKKRVLDTLEARM  114
_XP_044284772.1_    52  NGK----HPHLHAHSLVGKHCE-QGVCTV--NAGPK---DMVV----GFPNLGILHVTKKKVLETLETRM  107
_XP_044284769.1_    51  NGK----HPHLHAHSLVGKHCE-QGVCTV--NAGPK---DMVV----GFPNLGILHVTKKKVLETLETRM  106
_XP_039206640.1_    59  NGK----HVHLHAHSLVGKHCE-QGVCTV--NAGPK---DMVV----GFPNLGILHVTKKKVLETLETRM  114
_XP_042325082.1_    59  NGK----HVHLHAHSLVGKHCE-HGVCTV--NAGPK---DMAV----GFPNLGILHVTKKRVLDTLETRM  114
_XP_015683855.1_    59  NGK----HVHLHAHSLVGKHCE-QGVCTV--NAGPK---DMVV----GFPNLGILHVTKKKVLETLETRM  114
_KAF7252349.1_      59  NGK----HPHLHAHSLVGKHCE-QGVCTV--NAGPK---DMVV----GFPNLGILHVTKKKVLETLETRM  114
_XP_053259920.1_    18  NGK----HAHLHAHSLVGKHCE-NGVCTV--NAGPK---DMVV----GFPNLGILHVTKKRVLDTLEARM   73
_XP_028599459.1_    18  NGK----HAHLHAHSLVGKHCE-NGVCTV--NAGPK---DMVV----GFPNLGILHVTKKRVLDTLEARM   73
_XP_044284775.1_     1  ---------------------------------------IHKL----KFPNLGILHVTKKKVLETLETRM   27
_XP_039206641.1_     1  ------------------------------------------S----KFPNLGILHVTKKKVLETLETRM   24
_XP_013807108.1_     1  ---------------------------------KNH---TLRS----KFANLGILHVTKKKVFETLEARM   30
_XP_042664115.1_     1  ----------------------------------------MVV----GFANLGILHVTKKKVFETLETRM   26
_XP_042664114.1_     1  ---------------------------------KNH---TLRS----KFANLGILHVTKKKVFETLETRM   30
_NXS19671.1_         1  ------------------------------------------------FANLGILHVTKKKVFETLETRM   22
_XP_030305671.1_     1  ----------------------------------NH---TLRS----RFANLGILHVTKKKVFETLESRM   29
_PKU34609.1_         1  ---------------------------------KNH---TLRS----KFANLGILHVTKKKVFETLETRM   30
_KFP37264.1_        19  NGK----YVHLHAHSLVGKFCE-DGVCTV--NAGPK---DMVV----GFANLGILHVTKKKVFETLETRM   74
_NWX68872.1_        18  NGK----YVHLHAHSLVGKFCE-DGVCTV--NAGPK---DMVV----GFANLGILHVTKKKVFETLETRM   73
_KFP53260.1_        19  NGK----YVHLHAHSLVGKFCE-DGVCTV--NAGPK---DMVV----GFANLGILHVTKKKVFETLETRM   74
_NXP61177.1_        18  NGK----YVHLHAHSLVGKFCE-DGVCTV--IAGPK---DMVV----GFANLGILHVTKKKVFETLETRM   73
_XP_009482766.1_    17  NGK----YVHLHAHSLVGKFCE-DGVCTV--NAGPK---DMVV----GFANLGILHVTKKKVFETLETRM   72
_XP_010215381.1_    17  NGK----YVHLHAHSLVGKFCE-DGVCTV--NAGPK---DMVV----GFANLGILHVTKKKVFETLEARM   72
_NWI90635.1_        19  NGK----YVHLHAHSLVGKFCE-DGVCTV--NAGPK---DMVV----GFANLGILHVTKKKVFETLETRM   74
_NXU77174.1_        18  NGK----YVHLHAHSLVGKFCE-DGVCTV--NAGPK---DMVV----GFANLGILHVTKKKVFETLESRM   73
_NWR18823.1_        19  NGK----YVHLHAHSLVGKFCE-DGVCTV--LAGPK---DMVV----GFANLGILHVTKKKVFETLETRM   74
_POI31681.1_        17  NGK----YVHLHAHSLVGKFCE-DGVCTV--NAGPK---DMVV----GFANLGILHVTKKKVFETLEARM   72
_NXR92200.1_        18  NGK----YVHLHAHSLVGKFCE-NGVCTV--LTGPK---DMVV----GFANLGILHVTKKKVFETLETRM   73
_NXL85091.1_        60  NGK----YVHLHAHSLVGKFCE-DGVCTV--NAGPK---DMIV----GFANLGILHVTKKKVFETLEARM  115
_XP_005044951.1_    59  NGK----YVHLHAHSLVGKFCE-DGVCTV--TAGPK---DMVV----GFANLGILHVTKKKVFETLETRM  114
_NXC19638.1_        50  --------LTLHADVIV-------GICMSLRKYFPP---SKFG----RFANLGILHVTKKKVFETLETRM   97
_ETE71838.1_            ----------------------------------------------------------------------
_KAG8134450.1_      59  NGK----HVHLHAHSLVGKHCE-QGVCTV--NAGPK---DMVV----GFPNLGILHVTKKKVLDTLEMRM  114
```

```
_XP_026528490.1_    59  NGK----HVHLHAHSLVGKHCE-QGVCTV--NAGPK---DMVV----GFPNLGILHVTKKKVLDTLEMRM  114
_XP_044284773.1_    51  NGK----HPHLHAHSLVGKHCE-QGVCTV--NAGPK---DMVV----GFPNLGILHVTKKKVLETLETRM  106
_XP_026554053.1_    59  NGK----HVHLHAHSLVGKHCE-QGVCTV--NAGPK---DMVV----GFPNLGILHVTKKKVLDTLEMRM  114
_XP_032080378.1_    59  NGK----HVHLHAHSLVGKHCE-QGVCTV--NAGPK---DMIT----GFPNLGILHVTKKKVLDTLEARM  114
_XP_034298076.1_    59  NGK----HVHLHAHSLVGKHCE-HGVCTV--NAGPK---DMVV----GFPNLGILHVTKKKVLDTLENRM  114
_ABR18728.1_        14  NGK----NIHLHAHSLVGKHCE-DGICTV--TAGPK---DMVV----GFANLGILHVTKKKVFETLEARM   69
_XP_004459938.1_    59  NGK----NIHLHAHSLVGKHCE-DGICTV--TAGPK---DMVV----GFANLGILHVTKKKVFETLETRM  114
_XP_036619706.1_    59  NGK----HVHLHAHSLVGKHCE-DGICTV--TAGPK---DMVV----GFANLGILHVTKKKVYETLEARM  114
_XP_028932520.1_    59  HGK----NVHLHAHSLVGKHCE-DGICTV--TAGPK---DMVV----GFANLGILHVTKKKVYETLETRM  114
_MBZ3886481.1_      53  NGK----NIHLHAHSLVGKHCE-DGICTV--NAGPK---DMVV----GFANLGILHVTKKKVFETLEARM  108
_XP_047574052.1_    51  NGK----NIHLHAHSLVGKHCE-DGICTV--TAGPK---DMVV----GFANLGILHVTKKKVFETLEARM  106
_XP_032716757.1_    51  NGK----NIHLHAHSLVGKHCE-DGICTV--TAGPK---DMVV----GFANLGILHVTKKKVFETLEARM  106
_EFB13115.1_        54  NGK----NIHLHAHSLVGKHCE-DGICTV--TAGPK---DMVV----GFANLGILHVTKKKVFETLEARM  109
_XP_044923034.1_    51  NGK----NIHLHAHSLVGKHCE-DGICTV--TAGPK---DMVV----GFANLGILHVTKKKVFETLEARM  106
_KFO25880.1_        53  NGK----NIHLHAHSLVGKHCE-DGVCTV--TAGPK---DMVV----SFANLGILHVTKKKVFETLETRM  108
_EPY78492.1_        52  NGK----NIHLHAHSLVGKHCE-DGICTV--TAGPK---DMVV----GFANLGILHVTKKKVFETLEARM  107
_XP_006233422.1_    59  NGK----NIHLHAHSLVGKHCE-DGICTV--TAGPK---DMVV----GFANLGILHVTKKKVFETLEARM  114
_XP_038625183.1_    59  NGK----NVHLHAHSLVGKHCE-DGICTV--TAGPK---DMIV----GFANLGILHVTKKKVYETLETRM  114
_XP_039769612.1_    59  HGK----NVHLHAHSLVGKHCE-DGICTV--TAGPK---DMVV----GFANLGILHVTKKKVYETLETRM  114
_XP_026972816.1_    59  NGK----NIHLHAHSLVGKHCE-DGICTV--TAGPK---DMVV----GFANLGILHVTKKKVFETLEARM  114
_XP_030700972.1_    59  NGK----NIHLHAHSLVGKHCE-DGICTV--TAGPK---DMVV----GFANLGILHVTKKKVFETLEARM  114
_XP_019782048.1_    59  NGK----NIHLHAHSLVGKHCE-DGICTV--TAGPK---DMVV----GFANLGILHVTKKKVFETLEARM  114
_XP_044537564.1_    59  NGK----HVHLHAHSLVGKHCE-DGICTV--TAGPK---DMVV----GFANLGILHVTKKKVYETLEARM  114
_EDL12139.1_        59  NGK----NIHLHAHSLVGKHCE-DGVCTV--TAGPK---DMVV----GFANLGILHVTKKKVFETLEARM  114
_EDL82274.1_        59  NGK----NIHLHAHSLVGKHCE-DGICTV--TAGPK---DMVV----GFANLGILHVTKKKVFETLEARM  114
_XP_014391954.1_    59  NGK----SIHLHAHSLVGKHCE-DGICTV--TAGPK---DMVV----GFANLGILHVTKKKVFETLEARM  114
_XP_014391955.1_    59  NGK----SIHLHAHSLVGKHCE-DGICTV--TAGPK---DMVV----GFANLGILHVTKKKVFETLEARM  114
_XP_033258015.1_    59  NGK----NIHLHAHSLVGKHCE-DGICTV--TAGPK---DMVV----GFANLGILHVTKKKVFETLEARM  114
_XP_030700975.1_    59  NGK----NIHLHAHSLVGKHCE-DGICTV--TAGPK---DMVV----GFANLGILHVTKKKVFETLEARM  114
_XP_014315810.1_    59  NGK----SIHLHAHSLVGKHCE-DGICTV--TAGPK---DMVV----GFANLGILHVTKKKVFETLEARM  114
_XP_014315809.1_    59  NGK----SIHLHAHSLVGKHCE-DGICTV--TAGPK---DMVV----GFANLGILHVTKKKVFETLEARM  114
_XP_019782050.1_    59  NGK----NIHLHAHSLVGKHCE-DGICTV--TAGPK---DMVV----GFANLGILHVTKKKVFETLEARM  114
_XP_026972818.1_    59  NGK----NIHLHAHSLVGKHCE-DGICTV--TAGPK---DMVV----GFANLGILHVTKKKVFETLEARM  114
_XP_015425477.1_    59  NGK----SIHLHAHSLVGKHCE-DGICTV--TAGPK---DMVV----GFANLGILHVTKKKVFETLEARM  114
_XP_036266227.1_    59  NGK----SIHLHAHSLVGKHCE-DGICTV--TAGPK---DMVV----GFANLGILHVTKKKVYETLEARM  114
_XP_036196846.1_    59  NGK----SIHLHAHSLVGKHCE-DGICTV--TAGPK---DMVV----GFANLGILHVTKKKVFETLEARM  114
_XP_027242102.1_    59  NGK----NIHLHAHSLVGKHCE-DGICTV--VAGPK---DMVV----GFANLGILHVTKKKVFETLEARM  114
_EDL82271.1_        59  NGK----NIHLHAHSLVGKHCE-DGICTV--TAGPK---DMVV----GFANLGILHVTKKKVFETLEARM  114
_XP_035951696.1_    59  NGK----NIHLHAHSLVGKHCE-DGICTV--TAGPK---DMVV----GFANLGILHVTKKKVFETLEARM  114
```

```
_XP_024309837.1_    59  NGK----NIHLHAHSLVGKHCE-DGICTV--TAGPK---DMVV----GFANLGILHVTKKKVFETLEARM  114
_CAB94757.1_        59  NGK----NIHLHAHSLVGKHCE-DGICTV--TAGPK---DMVV----GFANLGILHVTKKKVFETLEARM  114
_AAA36361.1_        59  NGK----NIHLHAHSLVGKHCE-DGICTV--TAGPK---DMVV----GFANLGILHVTKKKVFETLEARM  114
_NP_001369554.1_    59  NGK----NIHLHAHSLVGKHCE-DGICTV--TAGPK---DMVV----GFANLGILHVTKKKVFETLEARM  114
_XP_034814839.1_    59  NGK----NIHLHAHSLVGKHCE-DGICTV--TAGPK---DMVV----GFANLGILHVTKKKVFETLEARM  114
_BAF84139.1_        59  NGK----NIHLHAHSLVGKHCE-DGICTV--TAGPK---DMVV----GFANLGILHVTKKKVFETLEARM  114
_CAH18336.1_        59  NGK----NIHLHAHSLVGKHCE-DGICTV--TAGPK---DMVV----GFANLGILHVTKKKVFETLEARM  114
_XP_032033228.1_    59  NGK----NIHLHAHSLVGKHCE-DGICTV--TAGPK---DMVV----GFANLGILHVTKKKVFETLEARM  114
_XP_054340511.1_    59  NGK----NTHLHAHSLVGKHCE-DGICTV--TAGPK---DMVV----GFANLGILHVTKKKVFETLEARM  114
_XP_024101589.1_    59  NGK----NTHLHAHSLVGKHCE-DGICTV--TAGPK---DMVV----GFANLGILHVTKKKVFETLEARM  114
_XP_003257527.1_    59  NGK----NIHLHAHSLVGKHCE-DGICTV--TAGPK---DMVV----GFANLGILHVTKKKVFETLEARM  114
_XP_009205528.3_    59  NGK----NIHLHAHSLVGKHCE-DGICTV--TAGPK---DMVV----GFANLGILHVTKKKVFETLEARM  114
_XP_014994319.1_    59  NGK----NIHLHAHSLVGKHCE-DGICTV--TAGPK---DMVV----GFANLGILHVTKKKVFETLEARM  114
_XP_005555609.1_    59  NGK----NIHLHAHSLVGKHCE-DGICTV--TAGPK---DMVV----GFANLGILHVTKKKVFETLEARM  114
_XP_024650030.1_    59  NGK----NIHLHAHSLVGKHCE-DGICTV--TAGPK---DMVV----GFANLGILHVTKKKVFETLEARM  114
_XP_023075867.2_    59  NGK----NIHLHAHSLVGKHCE-DGICTV--TAGPK---DMVV----GFANLGILHVTKKKVFETLEARM  114
_XP_017732574.1_    59  NGK----NIHLHAHSLVGKHCE-DGICTV--TAGPK---DMVV----GFANLGILHVTKKKVFETLEARM  114
_XP_010361621.1_    59  NGK----NIHLHAHSLVGKHCE-DGICTV--TAGPK---DMVV----GFANLGILHVTKKKVFETLEARM  114
_XP_007997596.2_    59  NGK----NIHLHAHSLVGKHCE-DGICTV--TAGPK---DMVV----GFANLGILHVTKKKVFETLEARM  114
_EHH53872.1_        59  NGK----NIHLHAHSLVGKHCE-DGICTV--TAGPK---DMVV----GFANLGILHVTKKKVFETLEARM  114
_XP_005555612.2_    59  NGK----NIHLHAHSLVGKHCE-DGICTV--TAGPK---DMVV----GFANLGILHVTKKKVFETLEARM  114
_XP_033070771.1_    59  NGK----NIHLHAHSLVGKHCE-DGICTV--NAGPK---DMVV----GFANLGILHVTKKKVFETLEARM  114
_XP_003929561.1_    59  NGK----NIHLHAHSLVGKHCE-DGICTV--TAGPK---DMVV----GFANLGILHVTKKKVFETLEARM  114
_XP_017364149.1_    59  NGK----NTHLHAHSLVGKHCE-DGICTV--TAGPK---DMVV----GFANLGILHVTKKKVFETLEARM  114
_XP_012291952.1_    59  NGK----HIHLHAHSLVGKHCE-DGICTV--TAGPK---DMVV----GFANLGILHVTKKKVFETLEARM  114
_XP_002745591.1_    59  NGK----NTHLHAHSLVGKHCE-DGICTV--TAGPK---DMVV----GFANLGILHVTKKKVFETLEARM  114
_XP_032117501.1_    59  NGK----NTHLHAHSLVGKHCE-DGICTV--TAGPK---DMVV----GFANLGILHVTKKKVFETLEARM  114
_XP_011798604.1_    59  NGK----NIHLHAHSLVGKHCE-DGICTV--TAGPK---DMVV----GFANLGILHVTKKKVFETLEARM  114
_XP_050647861.1_    59  NGK----NIHLHAHSLVGKHCE-DGICTV--TAGPK---DMVV----GFANLGILHVTKKKVFETLEARM  114
_XP_050647862.1_    59  NGK----NIHLHAHSLVGKHCE-DGICTV--TAGPK---DMVV----GFANLGILHVTKKKVFETLEARM  114
_XP_016807418.1_    59  NGK----NIHLHAHSLVGKHCE-DGICTV--TAGPK---DMVV----GFANLGILHVTKKKVFETLEARM  114
_XP_008070272.2_    59  NGK----NIHLHAHSLVGKHCE-DGICTV--TAGPK---DMVV----GFANLGILHVTKKKVFETLEARM  114
_XP_016079084.1_    59  NGK----STHLHAHSLVGKHCE-DGICTV--TAGPK---DMVV----GFANLGILHVTKKKVFETLEARM  114
_XP_054447653.1_    59  NGK----SIHLHAHSLVGKHCE-DGICTV--NAGPK---DMVV----GFANLGILHVTKKKVFETLEARM  114
_XP_014643400.1_    59  NGK----NIHLHAHSLVGKHCE-DGICTV--TAGPK---DMVV----GFANLGILHVTKKKVFETLEARM  114
_ABU63584.1_        59  NGK----NIHLHAHSLVGKHCE-DGICTV--TAGPK---DMVV----GFANLGILHVTKKKVFETLEARM  114
_XP_025146157.3_    59  NGK----NIHLHAHSLVGKHCE-DGVCTV--TAGPK---DMVV----GFANLGILHVTKKKVFETLEARM  114
_XP_047654925.1_    59  NGK----NIHLHAHSLVGKHCE-DGICTV--TAGPK---DMVV----GFANLGILHVTKKKVFETLEARM  114
_DAA28856.1_        59  NGK----NIHLHAHSLVGKHCE-DGVCTV--TAGPK---DMVV----GFANLGILHVTKKKVFETLEARM  114
```

```
_XP_025839601.1_    59  NGK----NIHLHAHSLVGKHCE-DGICTV--TAGPK---DMVV----GFANLGILHVTKKKVFETLEARM  114
_XP_005899878.1_    59  NGK----NIHLHAHSLVGKHCE-DGVCTV--TAGPK---DMVV----GFANLGILHVTKKKVFETLEARM  114
_XP_043726831.1_    59  NGK----NIHLHAHSLVGKHCE-DGICTV--TAGPK---DMVV----GFANLGILHVTKKKVFETLEARM  114
_KAF6372160.1_      59  NGK----NIHLHAHSLVGKHCE-DGICTV--TAGPK---DMVV----GFANLGILHVTKKKVFETLEARM  114
_KAF4019754.1_      59  NGK----NIHLHAHSLVGKHCE-DGICTV--TAGPK---DMVV----GFANLGILHVTKKKVFETLEARM  114
_XP_027399763.1_    59  NGK----NIHLHAHSLVGKHCE-DGVCTV--TAGPK---DMVV----GFANLGILHVTKKKVFETLEARM  114
_XP_008579050.1_    59  NGK----NIHLHAHSLVGKHCE-DGICTV--TAGPK---DMVV----GFANLGILHVTKKKVFETLEARM  114
_NP_001069877.1_    59  NGK----NIHLHAHSLVGKHCE-DGVCTV--TAGPK---DMVV----GFANLGILHVTKKKVFETLEARM  114
_XP_032962399.1_    59  NGK----NIHLHAHSLVGKHCE-DGICTV--TAGPK---DMVV----GFANLGILHVTKKKVFETLEARM  114
_CAD7684753.1_      59  NGK----NIHLHAHSLVGKHCE-DGICTV--TAGPK---DMVV----GFANLGILHVTKKKVFETLEARM  114
_XP_041613896.1_    59  NGK----NIHLHAHSLVGKHCE-DGICTV--TAGPK---DMVV----GFANLGILHVTKKKVFETLEARM  114
_XP_019481431.1_    59  NGK----NIHLHAHSLVGKHCE-DGICTV--TAGPK---DMVV----GFANLGILHVTKKKVFETLEARM  114
_XP_019817941.1_    59  NGK----NIHLHAHSLVGKHCE-DGVCTV--TAGPK---DMVV----GFANLGILHVTKKKVFETLEARM  114
_XP_016014547.2_    59  NGK----NIHLHAHSLVGKHCE-DGICAV--NAGPK---DMVV----GFANLGILHVTKKKVFETLEARM  114
_KAI4543101.1_      59  NGK----NIHLHAHSLVGKHCE-DGVCTV--TAGPK---DMVV----GFANLGILHVTKKKVFETLEARM  114
_XP_043294197.1_    59  NGK----NIHLHAHSLVGKHCE-DGICTV--TAGPK---DMVV----GFANLGILHVTKKKVFETLEARM  114
_XP_010846585.1_    59  NGK----NIHLHAHSLVGKHCE-DGVCTV--TAGPK---DMVV----GFANLGILHVTKKKVFETLEARM  114
_XP_017904753.1_    59  NGK----NIHLHAHSLVGKHCE-DGVCTV--TAGPK---DMVV----GFANLGILHVTKKKVFETLEARM  114
_XP_052497597.1_    59  NGK----NIHLHAHSLVGKHCE-DGVCTV--TAGPK---DMVV----GFANLGILHVTKKKVFETLEARM  114
_KAJ1061279.1_      59  NGK----NIHLHAHSLVGKHCE-DGVCTV--TAGPK---DMVV----GFANLGILHVTKKKVFETLEARM  114
_KAF6372159.1_      59  NGK----NIHLHAHSLVGKHCE-DGICTV--TAGPK---DMVV----GFANLGILHVTKKKVFETLEARM  114
_XP_032962400.1_    59  NGK----NIHLHAHSLVGKHCE-DGICTV--TAGPK---DMVV----GFANLGILHVTKKKVFETLEARM  114
_XP_019481432.1_    59  NGK----NIHLHAHSLVGKHCE-DGICTV--TAGPK---DMVV----GFANLGILHVTKKKVFETLEARM  114
_XP_040093689.1_    59  NGK----NIHLHAHSLVGKHCE-DGVCTV--TAGPK---DMVV----GFANLGILHVTKKKVFETLEARM  114
_XP_012034999.2_    59  NGK----NIHLHAHSLVGKHCE-DGVCTV--TAGPK---DMVV----GFANLGILHVTKKKVFETLEARM  114
_XP_019594487.1_    59  NGK----NIHLHAHSLVGKHCE-DGICTV--TAGPK---DMVV----GFANLGILHVTKKKVFETLEARM  114
_XP_011359814.1_    59  NGK----NIHLHAHSLVGKHCE-DGICTV--TAGPK---DMVV----GFANLGILHVTKKKVFETLEARM  114
_XP_006918936.1_    59  NGK----NIHLHAHSLVGKHCE-DGICTV--TAGPK---DMVV----GFANLGILHVTKKKVFETLEARM  114
_XP_003796373.2_    59  NGK----NTHLHAHSLVGKHCE-DGICTV--TAGPK---DMVV----GFANLGILHVTKKKVFETLEARM  114
_VFV45675.1_        59  NGK----NIHLHAHSLVGKHCE-DGICTV--TAGPK---DMVV----GFANLGILHVTKKKVFETLEARM  114
_XP_014938375.1_    59  NGK----NIHLHAHSLVGKHCE-DGICTV--TAGPK---DMVV----GFANLGILHVTKKKVFETLEARM  114
_XP_038299711.1_    59  NGK----NIHLHAHSLVGKHCE-DGICTV--TAGPK---DMVV----GFANLGILHVTKKKVFETLEARM  114
_XP_030169211.1_    59  NGK----NIHLHAHSLVGKHCE-DGICTV--TAGPK---DMVV----GFANLGILHVTKKKVFETLEARM  114
_XP_019594488.1_    59  NGK----NIHLHAHSLVGKHCE-DGICTV--TAGPK---DMVV----GFANLGILHVTKKKVFETLEARM  114
_XP_046947838.1_    59  NGK----NIHLHAHSLVGKHCE-DGICTV--TAGPK---DMVV----GFANLGILHVTKKKVFETLEARM  114
_XP_043427583.1_    59  NGK----NIHLHAHSLVGKHCE-DGICTV--TAGPK---DMVV----GFANLGILHVTKKKVFETLEARM  114
_XP_026253918.1_    59  NGK----NIHLHAHSLVGKHCE-DGICTV--NAGPK---DMVV----GFANLGILHVTKKKVFETLEARM  114
_XP_007081014.2_    59  NGK----NIHLHAHSLVGKHCE-DGICTV--TAGPK---DMVV----GFANLGILHVTKKKVFETLEARM  114
_XP_005325802.1_    59  NGK----NIHLHAHSLVGKHCE-DGICTV--NAGPK---DMVV----GFANLGILHVTKKKVFETLEARM  114
```

```
_XP_025777165.1_    59  NGK----NIHLHAHSLVGKHCE-DGICTV--TAGPK---DMVV----GFANLGILHVTKKKVFETLEARM  114
_XP_053413718.1_    59  NGK----NTHLHAHSLVGKHCE-DGICTV--TAGPK---DMVV----GFANLGILHVTKKKVFETLEARM  114
_KAF6131715.1_      59  NGK----SIHLHAHSLVGKHCE-DGICTV--NAGPK---DMVV----GFANLGILHVTKKKVFETLEARM  114
_NP_001041697.1_    59  NGK----NIHLHAHSLVGKHGE-DGICTV--TAGPK---DMVV----GFANLGILHVTKKKVFETLEARM  114
_XP_039076751.1_    59  NGK----NIHLHAHSLVGKHCE-DGICTV--TAGPK---DMVV----GFANLGILHVTKKKVFETLEARM  114
_XP_047711530.1_    59  NGK----NIHLHAHSLVGKHCE-DGICTV--TAGPK---DMVV----GFANLGILHVTKKKVFETLEARM  114
_XP_045648536.1_    59  NGK----NTHLHAHSLVGKHCE-DGICTV--TAGPK---DMVV----GFANLGILHVTKKKVFETLEARM  114
_XP_036708419.1_    59  NGK----NIHLHAHSLVGKHCE-DGICTV--TAGPK---DMVV----GFANLGILHVTKKKVFETLEARM  114
_XP_027791168.1_    59  NGK----NIHLHAHSLVGKHCE-DGICTV--NAGPK---DMVV----GFANLGILHVTKKKVFETLEARM  114
_KAF7487162.1_      59  NGK----NIHLHAHSLVGKHCE-DGICTV--NAGPK---DMVV----GFANLGILHVTKKKVFETLEARM  114
_XP_004380306.2_    59  NGK----NIHLHAHSLVGKHCE-DGICTV--TAGPK---DMVV----GFANLGILHVTKKKVFETLEARM  114
_XP_048960883.1_    59  NGK----NIHLHAHSLVGKHCE-DGICTV--TAGPK---DMVV----GFANLGILHVTKKKVFETLEARM  114
_XP_007172981.1_    59  NGK----NIHLHAHSLVGKHCE-DGICTV--TAGPK---DMVV----GFANLGILHVTKKKVFETLEARM  114
_XP_015340088.1_    59  NGK----NIHLHAHSLVGKHCE-DGICTV--NAGPK---DMVV----GFANLGILHVTKKKVFETLEARM  114
_XP_049488091.1_    59  NGK----NIHLHAHSLVGKHCE-DGICTV--TAGPK---DMVV----GFANLGILHVTKKKVFETLEARM  114
_XP_046314717.1_    59  NGK----NIHLHAHSLVGKHCE-DGICTV--NAGPK---DMVV----GFANLGILHVTKKKVFETLEARM  114
_XP_045327678.1_    59  NGK----NIHLHAHSLVGKHCE-DGICTV--TAGPK---DMVV----GFANLGILHVTKKKVFETLEARM  114
_KAI5775569.1_      59  NGK----NIHLHAHSLVGKHCE-DGICTV--TAGPK---DMVV----GFANLGILHVTKKKVFETLEARM  114
_XP_044080328.1_    59  NGK----NIHLHAHSLVGKHCE-DGICTV--TAGPK---DMVV----GFANLGILHVTKKKVFETLEARM  114
_XP_008154865.2_    59  NGK----SIHLHAHSLVGKHCE-DGICTV--TAGPK---DMVV----GFANLGILHVTKKKVYETLEARM  114
_KAI5937862.1_      59  NGK----NIHLHAHSLVGKHCE-DGICTV--TAGPK---DMVV----GFANLGILHVTKKKVFETLEARM  114
_XP_031546207.1_    59  NGK----NIHLHAHSLVGKHCE-DGICTV--TAGPK---DMVV----GFANLGILHVTKKKVFETLEARM  114
_XP_053779051.1_    59  NGK----SIHLHAHSLVGKHCE-DGICTV--NAGPK---DMVV----GFANLGILHVTKKKVFETLEARM  114
_XP_007463874.1_    59  NGK----NIHLHAHSLVGKHCE-DGICTV--TAGPK---DMVV----GFANLGILHVTKKKVFETLEARM  114
_XP_022368452.1_    59  NGK----NIHLHAHSLVGKHCE-DGICTV--TAGPK---DMVV----GFANLGILHVTKKKVFETLEARM  114
_XP_036876060.1_    59  NGK----NIHLHAHSLVGKHCE-DGICTV--TAGPK---DMVV----GFANLGILHVTKKKVFETLEARM  114
_XP_029798483.1_    59  NGK----NIHLHAHSLVGKHCE-DGICTV--TAGPK---DMVV----GFANLGILHVTKKKVFETLEARM  114
_XP_036109130.1_    59  NGK----SIHLHAHSLVGKHCE-DGICTV--MAGPK---DMVV----GFANLGILHVTKKKVFETLEARM  114
_XP_010951892.1_    59  NGK----NIHLHAHSLVGKHCE-DGICTV--TAGPK---DMVV----GFANLGILHVTKKKVFETLEARM  114
_XP_031324505.1_    59  NGK----NIHLHAHSLVGKHCE-DGICTV--TAGPK---DMVV----GFANLGILHVTKKKVFETLEARM  114
_XP_023982362.1_    59  NGK----NIHLHAHSLVGKHCE-DGICTV--TAGPK---DMVV----GFANLGILHVTKKKVFETLEARM  114
_XP_028362571.1_    59  NGK----SIHLHAHSLVGKHCE-DGICTV--NAGPK---DMVV----GFANLGILHVTKKKVFETLEARM  114
_XP_032316661.1_    59  NGK----NIHLHAHSLVGKHCE-DGICTV--TAGPK---DMVV----GFANLGILHVTKKKVFETLEARM  114
_XP_051675506.1_    59  NGK----NIHLHAHSLVGKHCE-DGICTV--NAGPK---DMVV----SFANLGILHVTKKKVFETLEARM  114
_VCW95948.1_        59  NGK----NIHLHAHSLVGKHCE-DGICTV--TAGPK---DMVV----GFANLGILHVTKKKVFETLEARM  114
_XP_045676060.1_    59  NGK----SIHLHAHSLVGKHCE-DGICTV--NAGPK---DMVV----GFANLGILHVTKKKVFETLEARM  114
_XP_040481442.1_    59  NGK----NTHLHAHSLVGKHCE-DGICTV--TAGPK---DMVV----GFANLGILHVTKKKVFETLEARM  114
_XP_047422689.1_    59  NGK----NIHLHAHSLVGKHCE-DGICTV--NAGPK---DMVV----GFANLGILHVTKKKVFETLEARM  114
_XP_040333287.1_    59  NGK----NIHLHAHSLVGKHCE-DGICTV--TAGPK---DMVV----GFANLGILHVTKKKVFETLEARM  114
```

```
_XP_032256795.1_    59  NGK----NIHLHAHSLVGKHCE-DGICTV--TAGPK---DMVV----GFANLGILHVTKKKVFETLEARM  114
_XP_036892131.1_    59  NGK----SVHLHAHSLVGKHCE-DGICTV--NAGPK---DMVV----GFANLGILHVTKKKVFETLEARM  114
_XP_049741557.1_    59  NGK----NIHLHAHSLVGKHCE-DGICTV--TAGPK---DMVV----GFANLGILHVTKKKVFETLEARM  114
_XP_003410513.1_    59  NGK----NIHLHAHSLVGKHCE-DGICTV--TAGPK---DMVV----GFANLGILHVTKKKVFETLEARM  114
_XP_024591836.1_    59  NGK----NIHLHAHSLVGKHCE-DGICSV--AAGPK---DMVV----GFANLGILHVTKKKVFETLEARM  114
_XP_022438147.1_    59  NGK----NIHLHAHSLVGKHCE-DGICTV--TAGPK---DMVV----GFANLGILHVTKKKVFETLEARM  114
_KAF0884117.1_      59  NGK----NIHLHAHSLVGKHCE-DGICTV--TAGPK---DMVV----GFANLGILHVTKKKVFETLEARM  114
_XP_034886066.1_    59  NGK----NIHLHAHSLVGKHCE-DGICTV--TAGPK---DMVV----GFANLGILHVTKKKVFETLEARM  114
_NP_001003344.1_    59  NGK----NIHLHAHSLVGKHCE-DGICTV--TAGPK---DMVV----GFANLGILHVTKKKVFETLEARM  114
_XP_020757604.1_    59  NGK----NIHLHAHSLVGKHCE-DGICTV--TAGPK---DMVV----GFANLGILHVTKKKVFETLEARM  114
_XP_045736627.1_    59  NGK----NIHLHAHSLVGKHCE-DGICTV--TAGPK---DMVV----GFANLGILHVTKKKVFDTLEARM  114
_XP_032488543.1_    59  NGK----NIHLHAHSLVGKHCE-DGICSV--AAGPK---DMVV----GFANLGILHVTKKKVFETLEARM  114
_XP_029090486.1_    59  NGK----NIHLHAHSLVGKHCE-DGICTV--TAGPK---DMVV----GFANLGILHVTKKKVFETLEARM  114
_XP_036735088.1_    59  NGK----NIHLHAHSLVGKHCE-DGICTV--TAGPK---DMVV----GFANLGILHVTKKKVFETLEARM  114
_XP_007951843.2_    59  NGK----NIHLHAHSLVGKHCE-DGICTV--TAGPK---DMVV----GFANLGILHVTKKKVFETLEARM  114
_XP_045392602.1_    59  NGK----SVHLHAHSLVGKHCE-DGICTV--NAGPK---DMVV----GFANLGILHVTKKKVFETLEARM  114
_XP_021536093.1_    59  NGK----NIHLHAHSLVGKHCE-DGICTV--TAGPK---DMVV----GFANLGILHVTKKKVLETLEARM  114
_XP_027453602.1_    59  NGK----NIHLHAHSLVGKHCE-DGICTV--TAGPK---DMVV----GFANLGILHVTKKKVFETLEARM  114
_XP_023108674.2_    59  NGK----NIHLHAHSLVGKHCE-DGICTV--TAGPK---DMVV----GFANLGILHVTKKKVFETLEARM  114
_XP_045853066.1_    59  NGK----NIHLHAHSLVGKHCE-DGICTV--TAGPK---DMVV----GFANLGILHVTKKKVFETLEARM  114
_XP_036266160.1_    59  NGK----SIHLHAHSLVGKHCE-DGICTV--TAGPK---DMVV----GFANLGILHVTKKKVYETLEARM  114
_XP_037012773.2_    59  NGK----SIHLHAHSLVGKHCE-DGICTV--NAGPK---DMVV----GFANLGILHVTKKKVFETLEARM  114
_XP_004401911.1_    59  NGK----NIHLHAHSLVGKHCE-DGICTV--TAGPK---DMVV----GFANLGILHVTKKKVFETLEARM  114
_KAI5226086.1_      59  NGK----NIHLHAHSLVGKHCE-DGICTV--TAGPK---DMVV----GFANLGILHVTKKKVFETLEARM  114
_XP_032716751.1_    59  NGK----NIHLHAHSLVGKHCE-DGICTV--TAGPK---DMVV----GFANLGILHVTKKKVFETLEARM  114
_XP_032188916.1_    59  NGK----NIHLHAHSLVGKHCE-DGICTV--TAGPK---DMVV----GFANLGILHVTKKKVFETLEARM  114
_XP_027453603.1_    59  NGK----NIHLHAHSLVGKHCE-DGICTV--TAGPK---DMVV----GFANLGILHVTKKKVFETLEARM  114
_XP_049564805.1_    59  NGK----NIHLHAHSLVGKHCE-DGICTV--TAGPK---DMVV----GFANLGILHVTKKKVFETLEARM  114
_XP_004401912.1_    59  NGK----NIHLHAHSLVGKHCE-DGICTV--TAGPK---DMVV----GFANLGILHVTKKKVFETLEARM  114
_XP_047574051.1_    59  NGK----NIHLHAHSLVGKHCE-DGICTV--TAGPK---DMVV----GFANLGILHVTKKKVFETLEARM  114
_XP_032488539.1_    59  NGK----NIHLHAHSLVGKHCE-DGICSV--AAGPK---DMVV----GFANLGILHVTKKKVFETLEARM  114
_XP_012510833.1_    59  NGK----NIHLHAHSLVGKHCE-DGICTV--NAGPK---DMVI----GFANLGILHVTKKKVFETLEARM  114
_XP_006893175.1_    59  NGK----NTHLHAHSLVGKHCE-DGICTV--TAGPK---DMVV----GFANLGILHVTKKKVYETLEARM  114
_XP_037686273.1_    59  NGK----NIHLHAHSLVGKHCE-DGICTV--NAGPK---DMVV----GFANLGILHVTKKKVFETLETRM  114
_XP_046512032.1_    59  NGK----HTHLHAHSLVGKHCE-DGICTV--TAGPK---DMVV----GFANLGILHVTKKKVFETLEARM  114
_XP_005608646.3_    59  NGK----HTHLHAHSLVGKHCE-DGICTV--TAGPK---DMVV----GFANLGILHVTKKKVFETLEARM  114
_XP_036196809.1_    59  NGK----SIHLHAHSLVGKHCE-DGICTV--TAGPK---DMVV----GFANLGILHVTKKKVFETLEARM  114
_XP_034527338.1_    59  NGK----NIHLHAHSLVGKHCE-DGICTV--TAGPK---DMVV----GFANLGILHVTKKKVFETLEARM  114
_XP_048960884.1_    59  NGK----NIHLHAHSLVGKHCE-DGICTV--TAGPK---DMVV----GFANLGILHVTKKKVFETLEARM  114
```

```
_XP_036046011.1_    59  NGK----NIHLHAHSLVGKHCE-DGICTV--VAGPK---DMVV----GFANLGILHVTKKKVFETLEARM  114
_XP_006872788.1_    59  NGK----NIHLHAHSLVGKHCE-DGICTV--TAGPK---DMVV----GFANLGILHVTKKKVFETLEARM  114
_XP_044923033.1_    59  NGK----NIHLHAHSLVGKHCE-DGICTV--TAGPK---DMVV----GFANLGILHVTKKKVFETLEARM  114
_KAB0398538.1_      59  NGK----NIHLHAHSLVGKHCE-DGICTV--TAGPK---DMVV----GFANLGILHVTKKKVFETLEARM  114
_TEA30114.1_        59  NGK----NIHLHAHSLVGKHCE-DGICTV--TAGPK---DMVV----GFANLGILHVTKKKVFETLEARM  114
_XP_004703501.1_    59  NGK----SIHLHAHSLVGKHCE-DGICTV--AAGPK---DMVV----GFANLGILHVTKKKVFETLEARM  114
_XP_045371697.1_    59  NGK----NIHLHAHSLVGKHCE-DGICTV--TAGPK---DMVV----GFANLGILHVTKKKVFETLEARM  114
_XP_045371695.1_    59  NGK----NIHLHAHSLVGKHCE-DGICTV--TAGPK---DMVV----GFANLGILHVTKKKVFETLEARM  114
_XP_045371698.1_    59  NGK----NIHLHAHSLVGKHCE-DGICTV--TAGPK---DMVV----GFANLGILHVTKKKVFETLEARM  114
_XP_051021942.1_    59  NGK----NIHLHAHSLVGKHCE-DGICTV--SAGPK---DMVV----GFANLGILHVTKKKVFETLEARM  114
_XP_028616215.1_    59  NGK----NIHLHAHSLVGKHCE-DGICTV--TAGPK---DMVV----GFANLGILHVTKKKVFETLEARM  114
_XP_040822498.1_    59  NGK----NIHLHAHSLVGKHCE-DGICTV--TAGPK---DMVV----SFSNLGILHVTKKKVFETLEARM  114
_XP_051041400.1_    59  NGK----NIHLHAHSLVGKHCE-DGICTV--VAGPK---DMVV----GFANLGILHVTKKKVFETLEARM  114
_XP_034356238.1_    59  NGK----NIHLHAHSLVGKHCE-DGICTV--TAGPK---DMVV----GFANLGILHVTKKKVFETLEARM  114
_XP_028713251.1_    59  NGK----NIHLHAHSLVGKHCE-DGICTV--VAGPK---DMVV----GFANLGILHVTKKKVFETLEARM  114
_ERE89660.1_        59  NGK----NIHLHAHSLVGKHCE-DGICTV--VAGPK---DMVV----GFANLGILHVTKKKVFETLEARM  114
_XP_052035128.1_    59  HGK----NIHLHAHSLVGKHCE-DGICTV--TAGPK---DMVV----GFANLGILHVTKKKVFETLEARM  114
_XP_006970398.1_    59  NGK----NIHLHAHSLVGKHCE-DGICTV--VAGPK---DMVV----GFANLGILHVTKKKVFETLEARM  114
_XP_042557701.1_    59  NGK----NIHLHAHSLVGKHCE-DGICTV--TAGPK---DMVV----GFANLGILHVTKKKVFETLEARM  114
_XP_031231473.1_    59  NGK----NIHLHAHSLVGKHCE-DGICTV--TAGPK---DMVV----GFANLGILHVTKKKVFETLEARM  114
_XP_012879285.1_    59  NGK----NIHLHAHSLVGKHCE-DGICTV--TAGPK---DMVV----GFANLGILHVTKKKVFETLEARM  114
_XP_003515839.4_    59  NGK----NIHLHAHSLVGKHCE-DGICTV--VAGPK---DMVV----GFANLGILHVTKKKVFETLEARM  114
_XP_012626422.1_    59  NGK----NIHLHAHSLVGKHCE-DGICTV--NAGPK---DMVV----GFANLGILHVTKKKVFETLEARM  114
_XP_048189722.1_    59  NGK----NIHLHAHSLVGKHCE-DGICTV--TTGPK---DMVV----GFANLGILHVTKKKVFETLEARM  114
_XP_052586690.1_    59  NGK----NIHLHAHSLVGKHCE-DGICTV--VAGPK---DMVV----GFANLGILHVTKKKVFETLEARM  114
_NP_032715.2_       59  NGK----NIHLHAHSLVGKHCE-DGVCTV--TAGPK---DMVV----GFANLGILHVTKKKVFETLEARM  114
_AAA40415.1_        59  NGK----NIHLHAHSLVGKHCE-DGVCTV--TAGPK---DMVV----GFANLGILHVTKKKVFETLEARM  114
_XP_049488087.1_    59  NGK----NIHLHAHSLVGKHCE-DGICTV--TAGPK---DMVV----GFANLGILHVTKKKVFETLEARM  114
_XP_044998939.1_    59  NGK----NIHLHAHSLVGKHCE-DGICTV--NAGPK---DMVV----GFANLGILHVTKKKVFETLEARM  114
_XP_049999310.1_    59  NGK----NIHLHAHSLVGKHCE-DGICTV--VAGPK---DMVV----GFANLGILHVTKKKVFETLEARM  114
_XP_040607681.1_    59  NGK----NIHLHAHSLVGKHCE-DGICTV--VAGPK---DMVV----GFANLGILHVTKKKVFETLEARM  114
_XP_045676057.1_    59  NGK----SIHLHAHSLVGKHCE-DGICTV--NAGPK---DMVV----GFANLGILHVTKKKVFETLEARM  114
_XP_032753167.1_    59  NGK----NIHLHAHSLVGKHCE-DGICTV--TAGPK---DMVV----GFANLGILHVTKKKVFETLEARM  114
_XP_028347494.1_    59  NGK----NIHLHAHSLVGKHCE-DGICTV--TAGPK---DMVV----GFANLGILHVTKKKVFETLEARM  114
_XP_031546209.1_    59  NGK----NIHLHAHSLVGKHCE-DGICTV--TAGPK---DMVV----GFANLGILHVTKKKVFETLEARM  114
_XP_010951893.1_    59  NGK----NIHLHAHSLVGKHCE-DGICTV--TAGPK---DMVV----GFANLGILHVTKKKVFETLEARM  114
_XP_005357381.1_    59  NGK----NIHLHAHSLVGKHCE-DGICTV--VAGPK---DMVV----GFANLGILHVTKKKVFETLEARM  114
_XP_031546210.1_    59  NGK----NIHLHAHSLVGKHCE-DGICTV--TAGPK---DMVV----GFANLGILHVTKKKVFETLEARM  114
_XP_031324516.1_    59  NGK----NIHLHAHSLVGKHCE-DGICTV--TAGPK---DMVV----GFANLGILHVTKKKVFETLEARM  114
```

```
_XP_031324521.1_    59  NGK----NIHLHAHSLVGKHCE-DGICTV--TAGPK---DMVV----GFANLGILHVTKKKVFETLEARM  114
_XP_041511026.1_    59  NGK----NIHLHAHSLVGKHCE-DGICTV--VAGPK---DMVV----GFANLGILHVTKKKVFETLEARM  114
_XP_032316666.1_    59  NGK----NIHLHAHSLVGKHCE-DGICTV--TAGPK---DMVV----GFANLGILHVTKKKVFETLEARM  114
_XP_032316670.1_    59  NGK----NIHLHAHSLVGKHCE-DGICTV--TAGPK---DMVV----GFANLGILHVTKKKVFETLEARM  114
_XP_021794361.1_    59  NGK----NIHLHAHSLVGKHCE-DGICTV--TAGPK---DMVV----GFANLGILHVTKKKVFETLEARM  114
_XP_021509291.1_    59  NGK----NIHLHAHSLVGKHCE-DGICTV--TAGPK---DMVV----GFANLGILHVTKKKVFETLEARM  114
_XP_021536094.1_    59  NGK----NIHLHAHSLVGKHCE-DGICTV--TAGPK---DMVV----GFANLGILHVTKKKVLETLEARM  114
_XP_013360849.1_    59  NGK----NIHLHAHSLVGKHCE-DGVCTV--TAGPK---DMVV----SFANLGILHVTKKKVFETLEARM  114
_XP_038959153.1_    59  NGK----NIHLHAHSLVGKHCE-DGICTV--TAGPK---DMVV----GFANLGILHVTKKKVFETLEARM  114
_XP_048312756.1_    59  NGK----NTHLHAHSLVGKHCE-DGICTV--VAGPK---DMVV----GFANLGILHVTKKKVFETLEARM  114
_XP_044622324.1_    59  NGK----HTHLHAHSLVGKHCE-DGICTV--TAGPK---DMVV----GFANLGILHVTKKKVFETLEARM  114
_XP_044622325.1_    59  NGK----HTHLHAHSLVGKHCE-DGICTV--TAGPK---DMVV----GFANLGILHVTKKKVFETLEARM  114
_XP_008527175.1_    59  NGK----HTHLHAHSLVGKHCE-DGICTV--TAGPK---DMVV----GFANLGILHVTKKKVFETLEARM  114
_XP_003468075.1_    59  NGK----NVHLHAHSLVGKHCE-DGVCTV--TAGPK---DMVV----SFANLGILHVTKKKVFETLEARM  114
_XP_027628732.1_    59  NGK----NIHLHAHSLVGKHCE-DGICTV--TAGPK---DMVV----GFANLGILHVTKKKVFETLEARM  114
_XP_046512030.1_    59  NGK----HTHLHAHSLVGKHCE-DGICTV--TAGPK---DMVV----GFANLGILHVTKKKVFETLEARM  114
_VFV45676.1_        59  NGK----NIHLHAHSLVGKHCE-DGICTV--TAGPK---DMVV----GFANLGILHVTKKKVFETLEARM  114
_XP_046512031.1_    59  NGK----HTHLHAHSLVGKHCE-DGICTV--TAGPK---DMVV----GFANLGILHVTKKKVFETLEARM  114
_XP_020843959.1_    59  NGK----HVHLHAHSLVGKHCE-DGICTV--TAGPK---DMVV----GFANLGILHVTKKKVYETLEARM  114
_XP_010639891.1_    59  NGK----NIHLHAHSLVGKHCE-DGVCTV--TAGPK---DMVV----SFANLGILHVTKKKVFETLETRM  114
_XP_025146161.3_    59  NGK----NIHLHAHSLVGKHCE-DGVCTV--TAGPK---DMVV----GFANLGILHVTKKKVFETLEARM  114
_XP_029416093.1_    59  NGK----NIHLHAHSLVGKHCE-DGICTV--AAGPK---DMVV----GFANLGILHVTKKKVFETLEARM  114
_XP_007495939.1_    59  NGK----HVHLHAHSLVGKHCE-DGICTV--TAGPK---DMVV----GFANLGILHVTKKKVYETLEARM  114
_XP_007495937.1_    59  NGK----HVHLHAHSLVGKHCE-DGICTV--TAGPK---DMVV----GFANLGILHVTKKKVYETLEARM  114
_XP_049564806.1_    59  NGK----NIHLHAHSLVGKHCE-DGICTV--TAGPK---DMVV----GFANLGILHVTKKKVFETLEARM  114
_XP_027709162.1_    59  NGK----HVHLHAHSLVGKHCE-DGICTV--TAGPK---DMVV----GFANLGILHVTKKKVYETLEARM  114
_XP_037365527.1_    59  NGK----STHLHAHSLVGKHCE-DGICTV--TAGPK---DMVV----GFANLGILHVTKKKVFETLEARM  114
_XP_004594353.1_    57  NGK----NIHLHAHSLVGKHCE-DGICTV--TAGPK---DMVV----SFSNLGILHVTKKKVFETLEARM  112
_XP_036619704.1_    59  NGK----HVHLHAHSLVGKHCE-DGICTV--TAGPK---DMVV----GFANLGILHVTKKKVYETLEARM  114
_XP_043828877.1_    59  NGK----HVHLHAHSLVGKHCE-DGICTV--TAGPK---DMVV----GFANLGILHVTKKKVYETLEARM  114
_XP_012407475.1_    59  NGK----HVHLHAHSLVGKHCE-DGICTV--TAGPK---DMVV----GFANLGILHVTKKKVYETLEARM  114
_XP_051822145.1_    59  NGK----HVHLHAHSLVGKHCE-DGICTV--TAGPK---DMVV----GFANLGILHVTKKKVYETLEARM  114
_XP_031798171.1_    59  NGK----HVHLHAHSLVGKHCE-DGICTV--TAGPK---DMVV----GFANLGILHVTKKKVYETLEARM  114
_XP_004686510.2_    59  NGK----SVHLHAHSLVGKHCE-DGVCTV--TAGPK---DMVV----GFANLGILHVTKKKVFETLEARM  114
_XP_021509292.1_    59  NGK----NIHLHAHSLVGKHCE-DGICTV--TAGPK---DMVV----GFANLGILHVTKKKVFETLEARM  114
_XP_052035129.1_    59  HGK----NIHLHAHSLVGKHCE-DGICTV--TAGPK---DMVV----GFANLGILHVTKKKVFETLEARM  114
_XP_019065790.1_    59  NGK----NIHLHAHSLVGKHCE-DGVCTV--TAGPK---DMVV----SFANLGILHVTKKKVFETLETRM  114
_XP_031231475.1_    59  NGK----NIHLHAHSLVGKHCE-DGICTV--TAGPK---DMVV----GFANLGILHVTKKKVFETLEARM  114
_XP_034356239.1_    59  NGK----NIHLHAHSLVGKHCE-DGICTV--TAGPK---DMVV----GFANLGILHVTKKKVFETLEARM  114
```

```
_XP_027251570.1_    59  NGK----NIHLHAHSLVGKHCE-DGICTV--VAGPK---DMVV----GFANLGILHVTKKKVFETLEARM  114
_XP_029330830.1_    59  NGK----NIHLHAHSLVGKHCE-DGICTV--TAGPK---DMVV----GFANLGILHVTKKKVFETLEARM  114
_XP_045426343.1_    59  NGK----SIHLHAHSLVGKHCE-DGICTV--TAGPK---DMVV----GFANLGILHVTKKKVYETLEARM  114
_NP_001397371.1_    59  NGK----NIHLHAHSLVGKHCE-DGVCTV--TAGPK---DMVV----GFANLGILHVTKKKVFETLEARM  114
_XP_040607683.1_    59  NGK----NIHLHAHSLVGKHCE-DGICTV--VAGPK---DMVV----GFANLGILHVTKKKVFETLEARM  114
_XP_026639883.1_    59  NGK----NIHLHAHSLVGKHCE-DGICTV--VAGPK---DMVV----GFANLGILHVTKKKVFETLEARM  114
_XP_029393278.1_    59  NGK----NIHLHAHSLVGKHCE-DGICTV--TAGPK---DMVV----GFANLGILHVTKKKVFETLEARM  114
_XP_049642488.1_    59  NGK----STHLHAHSLVGKHCE-DGICTV--TVGPK---DMVV----GFANLGILHVTKKKVFETLEARM  114
_XP_004617756.1_    59  NGK----SVHLHAHSLVGKHCE-DGVCTV--AAGPK---DMVV----GFANLGILHVTKKKVFETLEARM  114
_XP_021536097.1_    59  NGK----NIHLHAHSLVGKHCE-DGICTV--TAGPK---DMVV----GFANLGILHVTKKKVLETLEARM  114
_NWW35472.1_        59  NGK----YVHLHAHSLVGKFCE-DGVCTV--NAGPK---DMVV----GFANLGILHVTKKKVFETLETRM  114
_KAG8543434.1_      59  NGK----DVRLHAHSLVGKHCE-DGICSV--NVGPK---DNVV----GFPNLGILHVTKKKVMEVLETRM  114
_KAG8543366.1_      59  NGK----DVRLHAHSLVGKHCE-DGICSV--NVGPK---DNIV----GFPNLGILHVTKKKVMEVLETRM  114
_XP_044130636.1_    59  NGK----DVRLHAHSLVGKHCE-DGVCSI--SVGPK---DNVV----GFPNLGILHVTKKKVIEILEMRM  114
_XP_040274154.1_    59  NGK----DVRLHAHSLVGKHCE-DGVCSI--SVGPK---DNVV----GFPNLGILHVTKKKVIEILEARM  114
_XP_053317513.1_    59  NSK----DVRLHAHSLVGKHCE-DGICSV--KVGPK---DSVV----GFANLGILHVTKKKVNEILETRM  114
_ALA55868.1_        59  NGK----DVRLHAHSLVGRHCE-DGVCSI--NMGPK---DMVV----GFPNLGILHVTKKKVIEILERRM  114
_XP_040185842.1_    59  NGK----DVRLHAHSLVGRHCE-DGVCSI--NMGPK---DMVI----GFPNLGILHVTKKKVIEILERRM  114
_XP_040185853.1_    59  NGK----DVRLHAHSLVGRHCE-DGVCSI--NMGPK---DMVI----GFPNLGILHVTKKKVIEILERRM  114
_XP_018414489.1_    59  NGK----DVRLHAHSLVGRHCE-DGVCSM--SVGPK---DMVV----GFPNLGILHVTKKKVIEILEGRM  114
_XP_053559470.1_    59  NGK----DVRLHAHSLVGKHCE-DGICTI--NVGPK---DMIV----GFANLGILHVTKKKVIEVLEARM  114
_XP_053559471.1_    59  NGK----DVRLHAHSLVGKHCE-DGICTI--NVGPK---DMIV----GFANLGILHVTKKKVIEVLEARM  114
_KAG8454149.1_      59  NGK----DVRLHAHSLVGKNCE-DGISCM--TVGPK---DTII----GFPNLGILHVTKKKVIEILETRM  114
_NP_001087808.1_    59  NSK----DIRLHAHSLVGKNCE-DGICSL--TVGPK---DTIV----GFPNLGILHVTKKKVIEILEARM  114
_XP_018098880.1_    59  NSK----DIRLHAHSLVGKNCE-DGICLL--TVGPK---DTVV----GFPNLGILHVTKKKVIEVLEARM  114
_XP_002934805.3_    59  NSK----DIRLHAHSLVGKNCE-DGICSL--TVGPK---DTIV----GFANLGILHVTKKKVIEILEARM  114
_OCT97186.1_        59  NSK----DIRLHAHSLVGKNCE-DGICLL--TVGPK---DTVV----GFPNLGILHVTKKKVIEVLEARM  114
_CAI5653158.1_      59  ALT---VVPHLHAHSLVGKQCD-KGICIA--DLQSK---DSTI----SFPNLGILHVTKKNVAKTLEERM  115
_XP_032876238.1_    59  GGG---DQAHLHAHSLVGKQCE-RGICVV--QAGPK---DMLS----TFPSLGILHVTKKNVASTLEQRM  115
_XP_038648821.1_    59  FGR---DTVHLHAHSLVGKQCE-KGICLI--PVGPK---EMIT----TFPNLGVLHVTKKNVASTLEQRM  115
_XP_038648817.1_    59  FGR---DTVHLHAHSLVGKQCE-KGICLI--PVGPK---EMIT----TFPNLGVLHVTKKNVASTLEQRM  115
_GCB77082.1_        59  FGR---DTVHLHAHSLVGKQCE-KGICLI--QVGPK---EMIT----TFPNLGILHVTKKNVANTLEQRM  115
_XP_051900898.1_    59  AGR---DVVHLHAHSLVGKQCE-KGICLV--QIGPK---EMTS----TFPNLGILHVTKKNVASTLEQRM  115
_XP_048452319.1_    59  YGK---DNVHLHAHSLVGKQCE-KGICLV--QVGPK---EMIS----TFPNLGILHVTKKNVASTLEQRM  115
_XP_048382772.1_    59  YGK---DNVHLHAHSLVGKQCE-KGICLV--QVGPK---EMIS----TFPNLGILHVTKKNVASTLEQRM  115
_XP_043530455.1_    59  YGK---DSVHLHAHSLVGKQCE-KGICLV--QVGPK---EMIS----TFPNLGILHVTKKNVASTLEQRM  115
_GCC38268.1_        59  YGK---DSVHLHAHSLVGKQCE-KGICLV--QVGPK---EMIS----TFPNLGILHVTKKNVASTLEQRM  115
_XP_041045532.1_    59  FGR---DTVHLHAHSLVGKQCE-KGICLV--QVGPK---EMVS----TFPNLGILHVTKKNVASTLEQRM  115
_XP_007890062.2_    59  CGK---TAVHLHAHSLVGKQCE-KGICLV--QLGPK---EMTS----VFPNLGILHVTKKNVASTLEQRM  115
```

```
_XP_007897340.2_    58  NNK----PHMCHAHSLVGKHCTEEGTCVV--SVGPK---DMTA----QFANLGILHVTKRNVPKVLLRRF  114
_XP_007897342.2_    51  NNK----PHMCHAHSLVGKHCTEEGTCVV--SVGPK---DMTA----QFANLGILHVTKRNVPKVLLRRF  107
_XP_032868306.1_    59  AKE----PPRLHAHSLVGKQCTDEGICIV--HVGPK---DMMA----QFTNLGILHVTKKNVPEVLQDRI  115
_XP_051897529.1_    59  AKD----PPRLHAHSLVGKQCTEDGVCIV--QVGPK---DMTA----QFTNLGILHVTKKNVPEVLLDRI  115
_XP_048419270.1_    59  AKD----PPKLHAHSLVGKQCTDDGVCIV--QVGPK---DMTA----QFTNLGILHVTKKNVPDVLFDRL  115
_XP_048472262.1_    59  AKD----PPKLHAHSLVGKQCTDDGVCIV--QVGPK---DMTA----QFTNLGILHVTKKNVPDVLFDRL  115
_GCC17239.1_        59  AKD----PPKLHAHSLVGKQCTDDGVCIV--QVGPK---DMTA----QFTNLGILHVTKKNVPDVLFDRL  115
_XP_043534576.1_    59  AKD----PPKLHAHSLVGKQCTDDGVCIV--QVGPK---DMTA----QFTNLGILHVTKKNVPDVLFDRL  115
_XP_041032206.1_    59  AKD----PPKLHAHSLVGKQCTDDGVCIV--QVGPK---DMTA----QFTNLGILHVTKKNVPDVLFDRL  115
_XP_038639209.1_    51  AKD----PPKLHAHSLVGKQCSDDGVCIV--PVGPK---DMTA----QFTNLGILHVTKKNVPDVLFDRL  107
_XP_032825135.1_    59  ENA----PYRLHPHSLVGKNAE-NGAVSC--TVGPH---DMTA----MFPSLGVLHVTKKSVASVLEKLW  114
_AUA18026.1_        59  ENE----PYRLHPHSLVGKNAE-NGAVSC--TVGPH---DMTA----MFPSLGVLHVTKKSVASVLEKLW  114
_XP_043559628.1_    20  IDE----PPLLHVHSLVGRQCE-NGICVI--QMDSE---DMTA----SFPNLGILHVPKKDVVTIIEQQL   75
_GCC23558.1_        18  IDE----PPLLHVHSLVGRQCE-NGICVI--QMDSE---DMTA----SFPNLGILHVPKKDVVAIIEQQL   73
_XP_048459926.1_    59  IDE----PPLLHVHSLVGRQCE-NGICVI--QMDSD---DMTA----SFPNLGILHVPKKDVVAIIEQQL  114
_XP_048399043.1_    59  IDE----PPLLHVHSLVGRQCE-NGNCVI--QMDSD---DMTA----SFPNLGILHVPKKDVVAIIEQQL  114
_XP_043559626.1_    59  IDE----PPLLHVHSLVGRQCE-NGICVI--QMDSE---DMTA----SFPNLGILHVPKKDVVTIIEQQL  114
_XP_043559625.1_    59  IDE----PPLLHVHSLVGRQCE-NGICVI--QMDSE---DMTA----SFPNLGILHVPKKDVVTIIEQQL  114
_XP_043559624.1_    59  IDE----PPLLHVHSLVGRQCE-NGICVI--QMDSE---DMTA----SFPNLGILHVPKKDVVTIIEQQL  114
_XP_043559627.1_    59  IDE----PPLLHVHSLVGRQCE-NGICVI--QMDSE---DMTA----SFPNLGILHVPKKDVVTIIEQQL  114
_XP_041050315.1_    59  IDE----PPLLHVHSLVGRQCE-NGICVI--QMDSE---DMTA----SFPNLGILHVPKKDVVAIIEKQL  114
_GCB72915.1_        59  IDE----PPLLHVHSLVGRQCE-NGICVI--QMDSE---DMTA----SFPNLGILHVPKKDVVAIIEEQL  114
_XP_051870516.1_    59  IDE----PPLLHVHSLVGRQCK-NGICVV--QIDSE---DMTA----SFPNLGILHVPKKDVAAIIEEQL  114
_XP_032885971.1_    59  SDE----PPLLHVHSLVGRQCK-NGICFA--EIDSE---DMTA----SFPNLGILHVPKKDVAAIIEEQL  114
_XP_007904454.1_    59  IDE----PALLHVHSLVGRQCG-GGTCVV--RIDSE---DMTA----SFPNLGILHVPKKEVATIIEDQL  114
_CAD5120983.1_      58  TDL----QPILHPHKLVGKHCK-YGFYVQ--DFQPN--DKMTF----TLQGLSIIHVTKKEVPELLAKRI  114
_KAI6649899.1_      59  CAE---DSEEVHMHNLHGKSVF-NGEYLE--ELRPNNRGDVIH----SIQGISILHTKIQDKNEILGCKL  118
_KAI6652168.1_      57  GDE----GRCQHANSLVGRDVH-RGVMVR--YGVVSS-SSPKM----LLGNISIQHCKKKLITQSAEMQL  114
_KAI6653779.1_      58  PK-----SDEPHPFSLTGKGVQ-NGLFVS--VIRDGTTQGVEV----RLKDLSILHVKKQEVMTALQHKY  115
_NP_001267795.1_    64  EHD----NETMHIHSLVYNKTVTNGVHLL--TLNE----VDEV----ELEHVAVQQEKTKETKNFLFKER  119
_XP_047123977.1_    64  EHD----NETMHIHSLVYNKTVTNGVHLL--TLNE----VDEV----ELEHVAVQQEKTKETKNFLFKER  119
_XP_047123978.1_        ----------------------------------------------------------------------
_KAG8178714.1_      63  NEQ----EPRPHVLALVGKNCK-NGECWE--TISK----DGTA----VFRNLEIESKIRSQFIECLMNEK  117
_GFT03926.1_        59  NEI----NPKPHVHELTGKNCR-NGECLV--NLDE----DFSA----SFPSLAIQTRKKEEVLKTLMERK  113
_KAI6646872.1_      58  DVSRDPLTYGMHGHTFFGKNVY-NGQYIA--QLTASD-SKFNH----QLQGVTVIHTKSSDKNSILSGKY  119
_XP_033625504.1_    60  DD-----VRCLHPYILEGDDCV-EGVCTK--TIKAK---GEVI----SFPGLSIRKVSRKEMDKVLSKRV  114
_XP_038072325.1_    60  DD-----VIYPHPYVLEGVDCQ-DGVCTK--TILAK---GQII----SFPGLYVRSVSKEDIPKTLERRL  114
_XP_038072324.1_    60  DD-----VIYPHPYVLEGVDCQ-DGVCTK--TILAK---GQII----SFPGLYVRSVSKEDIPKTLERRL  114
Consensus_aa:           s.p.......HhHpLhG+ptp.sGhhhh..ph.sp...s..h.....h.sltlbHhs++ph.phLb.+h
```

```
Consensus ss:                       eeeeeee     eeee  ee        ee    ee   eeeeee   hhhhhhhhh

Conservation:
_KAH7637713.1_     117  SSS--------HQLNRRR--LLRRTGQQQQ------------------QQH-------------CLNN--  143
_XP_023220155.1_   115  LKE--------MALD-------------------------------------------------------  121
_XP_009016345.1_   115  RES--------LKIEK------------------------------------------------FCYG--  126
_ELU08708.1_       116  LEG--------FQQSFLN--GE-----------------------------------------DVGTL--  132
_UCK81536.1_       114  LES--------TRSFKLQ--RLIQERKASA------------------ARG-----I-D-T-SEISIT--  145
_CAH1795432.1_     117  RED--------IKLQHCV--KNNVPP------------------------------------EQYRIS--  138
_CAE1313443.1_     116  LES-------MLLNKRIC--SGNVND------------------------------------ENIQLN--  138
_XP_048764075.1_   115  LES--------MKLDKQV--KLGNLN------------------------------------AQADLS--  136
_XP_009060044.1_   114  KNK--------LLRDKKI--FLRNLN------------------------------------AIVSLS--  135
_XP_050391129.1_   117  KNS--------IIRDKQT--AMRDLK------------------------------------APMSLT--  138
_RUS85575.1_       115  LDR--------IKLNRRL--SGTNLN------------------------------------PDQTIS--  136
_GFS09172.1_       115  LDT--------LKLNRRL--AAAKLN------------------------------------LESSISDE  138
_XP_005091024.1_   115  LDA--------IKLNKKL--KENNMN------------------------------------FEPPIT--  136
_XP_005091023.1_   115  LDA--------IKLNKKL--KENNMN------------------------------------FEPPIT--  136
_KAH9508414.1_     114  VDT--------IKLNKKL--KANNMN------------------------------------FEPQIT--  135
_XP_013071937.1_   115  IDT--------IKLNKKL--KANNMN------------------------------------FEPHIT--  136
_KAI8766750.1_     115  IDT--------IKLNKKL--KANNMN------------------------------------FEPHIT--  136
_KAI8794647.1_     115  IDT--------IKLNKKL--KANNMN------------------------------------FEPHIT--  136
_NP_001298220.1_   115  IDT--------IKLNKKL--KANNMN------------------------------------FEPHIT--  136
_CAG5129174.1_     115  LDS--------IILNKKL--KANNMN------------------------------------FDPPLT--  136
_KAG5707696.1_       1  -----------------------------------------------------------------ALS--    3
_XP_025114345.1_   115  LEA--------RKLEKRL--KASDMS------------------------------------LDPKIT--  136
_QNL15288.1_       115  LEQ--------IKLNKRM---IGGLT------------------------------------NEPTTT--  135
_XP_041362807.1_   114  MES--------MLLDKRV--KLSNLQ------------------------------------AEVFIS--  135
_XP_046550037.1_   115  IES--------LILERKV--QQGHMS------------------------------------AEISIT--  136
_XP_048248211.1_   115  IES--------LILERKV--EQGHMN------------------------------------VEIPIT--  136
_XP_046550036.1_   115  IES--------LILERKV--QQGHMS------------------------------------AEISIT--  136
_CAC5363017.1_     115  IES--------MKMDKMV--KLGNLN------------------------------------EQAYLS--  136
_VDI06196.1_       115  IES--------MKMDKMV--KLGNLN------------------------------------EQAYLS--  136
_AHI17304.1_       115  IES--------MKMDKMV--KLGNLN------------------------------------EQAYLS--  136
_XP_052068168.1_   115  IES--------MKMDKMV--KLGNLN------------------------------------EQAYLS--  136
_XP_052068167.1_   115  IES--------MKMDKMV--KLGNLN------------------------------------EQAYLS--  136
_UZH84476.1_       115  IES--------IKLDKQV--KCQNLN------------------------------------VDISLT--  136
_XP_033756865.1_   115  LES--------IKLDKLV--KLGDLN------------------------------------AQADLT--  136
_AKC01669.1_       115  LES--------IKLDKLV--KLGDLN------------------------------------AQADLT--  136
```

```
_KAJ8319099.1_     115  VES--------MKLDKLV--KIGNLN------------------------------------AEAELS--  136
_XP_022318640.1_   115  LES--------MKLDKQV--KLGNLN------------------------------------MQTDLS--  136
_XP_011423502.2_   115  LDS--------MKLDKQV--KLGNLN------------------------------------VQTDLS--  136
_XP_052690447.1_   115  LDS--------MKLDKQV--KLGNLN------------------------------------VQTDLS--  136
_AEY99616.1_       114  HES--------LKLDKLV--KMGDLN------------------------------------AEADLT--  135
_XP_052263328.1_   114  RES--------CILHKKI--QHNDFN------------------------------------FNATLT--  135
_XP_052263327.1_   114  RES--------CILHKKI--QHNDFN------------------------------------FNATLT--  135
_XP_052761314.1_   115  MAA--------MILAKQM--EDGDFS------------------------------------STPQLS--  136
_XP_053392566.1_   115  CNS--------IIEARKR--ESKNWN------------------------------------TPILLR--  136
_KAI0238513.1_     118  IEQ--------MKFNNKI--MGDALN------------------------------A-G-D-ISAEVP--  142
_XP_029638652.1_   115  LES--------MILKKRI--DSGNVND-----------------------------------TSINAD--  137
_AUR53646.1_       115  LES--------MILKKRI--DSGNVND-----------------------------------MSINAD--  137
_XP_036360935.1_   115  LES--------MILKKRI--DSGNVND-----------------------------------TSINAD--  137
_XP_036360934.1_   115  LES--------MILKKRI--DSGNVND-----------------------------------TSINAD--  137
_XP_029638651.1_   115  LES--------MILKKRI--DSGNVND-----------------------------------TSINAD--  137
_XP_014776042.1_   114  LES--------MILKKRI--DSGNVND-----------------------------------TSINAD--  136
_XP_014776041.1_   114  LES--------MILKKRI--DSGNVND-----------------------------------TSINAD--  136
_XP_052825578.1_   114  LES--------MILKKRI--DSGNVND-----------------------------------TSINAD--  136
_XP_052825580.1_    25  LES--------MILKKRI--DSGNVND-----------------------------------TSINAD--   47
_AEP33644.1_       115  LEH--------QKVYNAL--IGPN-----------------------------------------SST--  131
_PIK44524.1_       115  LEH--------QQIRSMI--LGS-----------------------------------------NAEP--  131
_KAJ8022831.1_     115  LEN--------QRICNAV--LGP-----------------------------------------EAIQ--  131
_XP_030838637.1_    72  REQ--------QKMYRSV--LSDKD-------------------------------------CRAQGM--   92
_XP_038060093.1_   115  REQ--------QKMYRSV--LSDKD-------------------------------------CRAQGM--  135
_XP_022087493.1_   115  RQQ--------QKLYCSV--LGDK-------------------------------GY-Q-S-STTHGI--  138
_XP_033643738.1_   115  REQ--------QKLYRTF--LGD--------------------------------KG-F-V-PGAQHL--  137
_XP_033100082.1_   115  FDY--------QETSKNL--WGQ------------------------------------------SND--  130
_CCC55418.2_        94  LAQ--------HRLYKDL--INNSG-------------------------------------GEGQWM--  114
_XP_054773081.1_   115  LAQ--------HRLYKDL--INISQ-------------------------------------GENRWM--  135
_XP_030834883.1_   119  LAQ--------HRLYKDL--INN---------------------------------S-T-P-GESHWT--  140
_NP_999819.1_      115  LAQ--------HRLYKDL--INN---------------------------------S-T-P-GESHWT--  136
_XP_030834884.1_   119  LAQ--------HRLYKDL--INN---------------------------------S-T-P-GESHWT--  140
_XP_041485048.1_   115  LAQ--------HRLYKDL--INI---------------------------------S-Q---GENRWM--  135
_QIA61835.1_       118  I----------DGLRVSR--GDFSG-------------------------------------GDIPLG--  136
_NP_001266238.1_   118  LQQTTPRGQMVDQMEVVD--VDMTT---------------------------------------AQLT--  144
_QHX41456.1_       117  IQN-----------KAMQ--ITMQ--------------------------------G-M-D-PAVAIG--  136
_XP_022789210.1_   116  IKM--------QTLHTAT--LNALTSADG-KGFDVT-----VLGDH-VDGDRSS-FD-K-H-LAEAVA--  162
_XP_046859971.1_   115  LKR--------LSLEKTS--ISQSSNLL---------------------LNVHTNVD-S-ERIAEVIT--  149
_XP_028414684.1_   116  LKK--------HRLEKSF--NSGVSSGDQV-----------------MHVDFHDPAS-S-GAIAEAIT--  154
```

```
_XP_028414852.1_  116  LKK--------HRLEKSF--NSGVSSGDQV-----------------MHVDFHDPAS-S-GAIAEAIT--  154
_ADQ57372.1_      117  IKW--------QTLQNAT--FAKLSEGIKDGVDLSL-----FGVNTAINSNKLG-FD-K-S-VALSVA--  165
_XP_048580668.1_  117  IKW--------QTLQNAT--FAKLSEGIKDGVDLSL-----FGVNTAINSNKLG-FD-K-N-VALSVA--  165
_EDO32923.1_      117  IKW--------QTLQNAT--FAKLSEGIKDGVDLSL-----FGVNTAINSNKLG-FD-K-N-VALSVA--  165
_ABU48530.1_      117  IKW--------QTLQNAT--FAKLSEGIKDGVDLSL-----FGVNTAINSNKLG-FD-K-N-VALSVA--  165
_ALG40993.1_      117  TRM--------QELQSAT--MTALGATNADPAALFGISGLGAQNDSVPGVDTKT-FD-K-N-LAMSVA--  170
_ALG40994.1_      117  TKM--------QELQSLT--MSVMSTTHNDPASLFGIS--GLGAQHAAGTDNKP-FD-K-N-LALSVA--  168
_XP_031565156.1_  117  TKM--------QELQSLT--MSVMSTTHNDPASLFGIS--GLGAQHAAGTDNKP-FD-K-N-LALSVA--  168
_ALG40992.1_      117  TKM--------QELQNAT--MTALGATNNDPSTFFGISGLNSHPTGILGTENKP-FD-K-N-FALTVA--  170
_KXJ21435.1_      117  TKM--------QALQNAT--LTALATNNSTPSSFMNF---GSVAREQVMASQGP-FD-R-N-LAAAVA--  167
_XP_020916759.1_  117  TKM--------QALQNAT--LTALATNNSTPSSFMNF---GSVAREQVMASQGP-FD-R-N-LAAAVA--  167
_XP_015766406.1_  116  LKM--------QMLHTAT--LNALTT-DPRVFDIGA-----MVDEATADGDRGE-FD-K-Q-IADTIA--  163
_UBR69922.1_      116  LKM--------QMLHTAT--LNALTT-DPRVFDIGA-----MVDEATADGDRGE-FD-K-Q-IADTIA--  163
_XP_029186950.2_  116  LKM--------QMLHTAT--LNALTT-DPRVFDIGA-----MVDEATADGDRGE-FD-K-Q-IADTIA--  163
_UCR60992.1_      116  LKM--------QMLHTAT--LNALTT-DPRVFDIGA-----MVDEATADGDRGE-FD-K-Q-IADTIA--  163
_XP_027038010.1_  116  IKM--------QTLHTAT--LNALTSGDGKVFGVAG-----LVDQAMVDGDRGS-FD-K-R-LAEAVA--  164
_NWX00991.1_      115  IDA--------CKKGYNP--ELLVHPEL----------------------GYLQ-AD-A-C-GDRQLT--  146
_NXS91232.1_      115  IDA--------CKKGYNP--GLLVHPEL----------------------GYLQ-AE-G-C-GDRQLT--  146
_NXG78778.1_      115  IDA--------CKKGYNP--GLLVHPEL----------------------GYLQ-AD-G-C-GDRQLT--  146
_XP_025964754.1_  115  IDA--------CKKGYNP--GLLVHPEL----------------------GYLQ-AE-G-C-GDRQLT--  146
_XP_014730709.1_  115  IDA--------CKKGYNP--GLLVHPEL----------------------SYLQ-AE-G-C-GDRQLT--  146
_NXW45082.1_      115  IDA--------CKKGYNP--GLLVHPEL----------------------GYLQ-AE-G-C-GDRQLT--  146
_XP_015479466.1_  115  IDA--------CKKGYNP--GLLVHPEL----------------------GYLQ-AE-G-C-GDRQLT--  146
_XP_009577719.1_  115  IDA--------CKKGYNP--GLLVHPEL----------------------GYLQ-AE-G-C-GDRQLT--  146
_NXC45563.1_      115  IDA--------CKKGYNP--GLLVHPEL----------------------SYLQ-AE-G-C-GDRQLN--  146
_XP_009883811.1_  115  IDA--------CKKGYNP--GLLVHPEL----------------------GYLQ-AE-G-S-GDRQLT--  146
_NXH56308.1_      115  IDA--------CKKGYNP--GLLVHPEL----------------------SYLQ-AE-G-C-GDRQLT--  146
_XP_023780422.1_  115  IDA--------CKKGYNP--GLLVHPEL----------------------GYLQ-AE-G-C-GDRQLT--  146
_XP_009271830.1_  115  IDA--------CKKGYNP--GLLVHPEL----------------------GYLQ-AE-G-C-GDRQLT--  146
_XP_009325314.1_  115  IDA--------CKKGYNP--GLLVHPEL----------------------GYLQ-AE-G-C-GDRQLT--  146
_NXM85820.1_      115  IDA--------CKKGYNP--GLLVHPEL----------------------SYLQ-AE-G-C-GDRQLT--  146
_NXI98149.1_      115  IDA--------CKKGYNP--GLLVHPEL----------------------GYLQ-AE-G-C-GDRQLT--  146
_XP_013807107.1_  115  IDA--------CKKGYNP--GLLVHPEL----------------------GYLQ-AE-G-C-GDRQLT--  146
_NWS89770.1_      115  IDA--------CKKGYNP--GLLVHPEL----------------------SYLQ-AE-G-C-GDRQLT--  146
_NXN94237.1_      115  IDA--------CKKGYNP--ELLVHPEF----------------------GYLQ-AE-G-C-GDRQLT--  146
_NXH67539.1_      115  IDA--------CKKGYNP--GLLVHPEL----------------------GYLQ-AE-G-C-GDRQLT--  146
_NXQ49570.1_      115  IDA--------CKKGYNP--GLLVHPEL----------------------SYLQ-AE-G-C-GDRQLT--  146
_NWT07854.1_      115  IDA--------CKKGYNP--GLLVHPEL----------------------GYLQ-AE-G-C-GDRQLT--  146
_XP_032915292.1_  115  IDA--------CKKGYNP--GLLVHPEL----------------------SYLQ-AE-G-C-GDRQLT--  146
```

```
_XP_014117319.1_  115  IDA--------CKKGYNP--GLLVHPEL----------------------GYLQ-AE-G-C-GDRQLT--  146
_XP_009816360.1_  115  IDA--------CKKGYNP--GLLVHPEL----------------------GYLQ-AE-G-C-GDRQLT--  146
_NXO31807.1_      115  IDA--------CKKGYNP--GLLVHPEL----------------------GYLQ-AE-G-C-GDRQLT--  146
_NWW07363.1_      115  IDA--------CKKGYNP--GLLVHPEL----------------------SYLQ-AE-G-C-GDRQLT--  146
_XP_054681626.1_  115  IDA--------CKKGYNP--GLLVHPEL----------------------GYLQ-AE-G-C-GDRQLT--  146
_XP_010286301.1_  115  IDA--------CKKGYNP--GLLVHPEL----------------------GYLQ-AE-G-C-GDRQLT--  146
_NXC54686.1_      115  IDA--------CKKGYNP--ELLVHPEL----------------------GYLQ-AE-G-C-GDRQLT--  146
_XP_054059788.1_  115  IDA--------CKKGYNP--GLLVHPEL----------------------DYLQ-AE-G-C-GDRQLT--  146
_OPJ75671.1_      115  IDA--------CKKGYNP--GLLVHPEL----------------------GYLQ-AD-A-C-GDRQLT--  146
_XP_010311473.1_  115  IDA--------CKKGYNP--GLLVHPEL----------------------GYLQ-AE-G-C-GDRQLT--  146
_XP_053921662.1_  115  IDA--------CKKGYNP--GLLVHPEL----------------------GYLQ-AD-G-C-GDRQLT--  146
_NWQ94220.1_      115  IDA--------CKKGYNP--GLLVHPEL----------------------GYLQ-AE-G-S-GDRQLT--  146
_XP_025934196.1_  115  IDA--------CKKGYNP--GLLVHPEL----------------------GYLQ-AE-G-C-GDRQLT--  146
_NXS57318.1_      115  IDA--------CKKGYNP--GLLVHPEL----------------------AYLQ-AD-G-C-GDRQLT--  146
_NXF52248.1_      115  IDA--------CKKGYNP--GLLVHPEL----------------------GYLQ-AE-G-C-GDRQLT--  146
_OPJ75670.1_      115  IDA--------CKKGYNP--GLLVHPEL----------------------GYLQ-AD-A-C-GDRQLT--  146
_NXD98176.1_      115  IDA--------CKKGYNP--GLLVHPEL----------------------SYLQ-AE-G-C-GDRQLT--  146
_XP_009949173.1_  115  IDA--------CKKGYNP--GLLVHPEL----------------------GYLQ-AE-G-C-GDRQLT--  146
_NWH32710.1_      115  IDA--------CKKGYNP--GLLVHPEL----------------------SYLQ-AE-G-C-EDRQLT--  146
_XP_010178978.1_  115  IDA--------CKKGYNP--GLLVHPEL----------------------GYLQ-AE-G-C-GDRQLT--  146
_NXI82337.1_      115  IDA--------CKKGYNP--GLLVHPEL----------------------SYLQ-AE-G-C-GDRQLT--  146
_NXJ24754.1_      115  IDA--------CKKGYNP--GLLVHPEL----------------------SYLQ-AE-G-C-GDRQLS--  146
_NXM41500.1_      115  IDA--------CKKGYNP--GLLVHPEL----------------------SYLQ-AE-G-C-GDRQLT--  146
_NWW61888.1_      115  IDA--------CKKGYNP--GLLVHPEL----------------------SYLQ-AD-G-C-GDRQLT--  146
_NXE22655.1_      115  IDA--------CKKGYNP--GLLVHPEL----------------------GYLQ-AD-G-C-GDRQLT--  146
_NXF65753.1_      115  IDA--------CKKGYNP--GLLVHPEL----------------------GYLQ-AE-G-C-GDRQLT--  146
_NWS57053.1_      115  IDA--------CKKGYNP--GLLVHPEL----------------------AYLQ-AE-G-C-GDRQLT--  146
_NWU51843.1_      115  IDA--------CKKGYNP--GLLVHPEL----------------------GYLQ-AE-G-C-GDRQLT--  146
_NXB31773.1_      115  IDA--------CKKGYNP--GLLVHPEL----------------------GYLQ-AE-G-C-GDRQLT--  146
_NXE37412.1_      115  IDA--------CKKGYNP--GLLVHPEL----------------------GYLQ-AE-G-C-GDRQLT--  146
_NXM73977.1_      115  IDA--------CKKGYNP--GLLVHPEL----------------------GYLQ-AE-G-C-GDRQLT--  146
_NXK45099.1_      115  IDA--------CKKGYNP--GLLVHPEL----------------------GYLQ-AE-G-C-GDRQLT--  146
_NXT11309.1_      115  IDA--------CKKGYNP--GLLVHPEL----------------------GYLQ-AE-G-C-EDRQLT--  146
_XP_039920334.1_  115  IDA--------CKKGYNP--GLLVHPEL----------------------GYLQ-AE-G-C-GDRQLT--  146
_NXQ27105.1_      115  IDA--------CKKGYNP--GLLVHPEL----------------------GYLQ-AE-G-C-GDRQLT--  146
_NXN43964.1_      115  IDA--------CKKGYNP--GLLVHPEL----------------------GYLE-AE-G-C-GDRQLT--  146
_XP_025934203.1_  115  IDA--------CKKGYNP--GLLVHPEL----------------------GYLQ-AE-G-C-GDRQLT--  146
_KFM03673.1_      115  IDA--------CKKGYNP--GLLVHPEL----------------------GYLQ-AE-G-C-GDRQLT--  146
_NXN08485.1_      115  IDA--------CKKGYNP--GLLVHPEL----------------------AYLQ-AD-G-C-GDRQLT--  146
```

```
_NXR70665.1_       115  IDA--------CKKGYNP--GLLVHPEL----------------------SYLQ-AE-G-C-GDRQLT--  146
_XP_010165409.1_   115  IDA--------CKKGYNP--GLLVHPEL----------------------GYLQ-AE-G-C-GDKQLT--  146
_XP_010584501.1_   115  IDA--------CKKGYNP--GLLVHPEL----------------------GYLQ-AE-G-C-GDRQLT--  146
_NWS41193.1_       115  IDA--------CKKGYNP--GLLVHPEL----------------------GYLQ-AE-G-C-GDRQLT--  146
_KFV51344.1_       115  IDA--------CKKGYNP--GLLVHPEL----------------------GYLQ-AE-G-C-GDRQLT--  146
_NXE09783.1_       115  IDA--------CKKGYNP--GLLVHPEL----------------------GYLQ-AD-G-C-GDRQLT--  146
_NXI10557.1_       115  IDA--------CKKGYNP--GLLVHPEL----------------------SYLQ-AE-G-C-EDRQLT--  146
_NXJ43845.1_       115  IDA--------CKKGYNP--GLLVHPEL----------------------GYLQ-AE-G-C-GDRQLT--  146
_NWS05278.1_       115  IDA--------CKKGYNP--GLLVHPEL----------------------SYLQ-AE-G-C-EDRQLT--  146
_XP_027573674.2_   115  IDA--------CKKGYNP--GLLVHPEL----------------------GYLQ-AE-G-C-GDRQLT--  146
_NXQ94576.1_       115  IDA--------CKKGYNP--GLLVHPEL----------------------GYLQ-AE-G-C-GDRQLT--  146
_KAI1238512.1_     115  IDA--------CKKGYNP--GLLVHPEL----------------------SYLQ-AE-G-C-GDRQLT--  146
_NXK36380.1_       115  IDA--------CKKGYNP--GLLVHPEL----------------------GYLQ-AE-G-C-GDRQLT--  146
_NWH51107.1_       115  IDA--------CKKGYNP--GLLVHPEL----------------------GYLQ-AE-G-C-GDRQLT--  146
_NXW80580.1_       115  IDA--------CKKGYNP--GLLVHPEL----------------------GYLQ-AE-G-C-GDRQLT--  146
_NXY08181.1_       115  IDA--------CKKGYNP--GLLVHPEL----------------------GYLQ-AE-G-C-GDRQLT--  146
_NWU97338.1_       115  IDA--------CKKGYNP--ELLVHPEF----------------------GYLQ-AE-G-C-GDRQLT--  146
_XP_027491592.1_   115  IDA--------CKKGYNP--GLLVHPEL----------------------GYLQ-AE-G-C-GDRQLT--  146
_NXU08140.1_       115  IDA--------CKKGYNA--GLLVHPEL----------------------GYLQ-AE-G-C-GERQLT--  146
_XP_029870423.1_   115  IDA--------CKKGYNP--GLLVHPEL----------------------GYLQ-AE-G-C-GDRQLT--  146
_XP_037992222.1_   115  IDA--------CKKGYNP--GLLVHPEL----------------------SYLQ-AE-G-C-EDRQLT--  146
_XP_019470357.1_   115  IDA--------CKKGYNP--GLLVHPEL----------------------GYLQ-AE-G-C-GDRQLT--  146
_XP_051646449.1_   115  IDA--------CKKGYNP--GLLVHPEL----------------------GYLQ-AE-G-C-GDRQLT--  146
_NXT54739.1_       115  IDA--------CKKGYNP--GLLVHPEL----------------------GYLQ-AE-G-S-GERQLT--  146
_NXG03656.1_       115  IDA--------CKKGYNP--GLLVHPEL----------------------GYLQ-AE-G-C-GDRQLT--  146
_NWT86202.1_       115  IDA--------CKKGYNP--GLLVHPEL----------------------SYLQ-AE-G-C-GDRQLT--  146
_XP_009687644.1_   115  IDA--------CKKGYNP--GLLVHPEL----------------------GYLQ-AE-G-C-GDRQLT--  146
_NWQ73497.1_       115  IDA--------CKKGYNP--GLLVHPEL----------------------GYLQ-AD-A-C-GDRQLT--  146
_XP_050751358.1_   115  IDA--------CKKGYNP--GLLVHPEL----------------------GYLQ-AE-G-C-GDRQLT--  146
_KFO60593.1_       115  IDA--------CKKGYNP--GLLVHPEL----------------------SYLQ-AE-G-C-GDRQLT--  146
_XP_014133950.1_   115  IDA--------CKKGYNP--GLLVHPEL----------------------GYLQ-AE-G-C-GDRQLT--  146
_NXK26872.1_       115  IDA--------CKKGYNP--GLLVHPEL----------------------GYLQ-AE-G-C-GDRQLT--  146
_NWQ68199.1_       115  IDA--------CKKGYNP--GLLVHPEL----------------------GYLQ-AE-G-C-GDRQLT--  146
_XP_030348000.1_   115  IDA--------CKKGYNP--GLLVHPEL----------------------GYLQ-AE-G-C-GDRQLT--  146
_NWI78033.1_       115  IDA--------CKKGYNP--GLLVHPEL----------------------SYLQ-AE-G-C-GDRQLT--  146
_NXO71082.1_       115  IDA--------CKKGYNP--GLLVHPEL----------------------SYLQ-AE-G-C-GDRQLT--  146
_XP_040454521.1_   115  IDA--------CKKGYNP--GLLVHPEL----------------------GYLQ-AE-G-C-GDRQLT--  146
_NXQ12070.1_       115  IDA--------CKKGYNP--GLLVHPEL----------------------SYLQ-AE-G-C-EDRQLT--  146
_NXB56634.1_       115  IDA--------CKKGYNP--GLLVHPEL----------------------SYLQ-AE-G-C-GERQLT--  146
```

```
_NXO78917.1_      115  IDA--------CKKGYNP--GLLVHPEL----------------------GYLQ-AE-G-C-GDRQLT--  146
_NXR80251.1_      115  IDA--------CKKGYNP--GLLVHPEL----------------------GYLQ-AE-G-C-GDRQLT--  146
_NXP31238.1_      115  IDA--------CKKGYNP--GLLVHPEL----------------------SYLQ-AE-G-C-GDRQLT--  146
_NXE73484.1_      115  IDA--------CKKGYNP--GLLVHPEL----------------------GYLQ-AE-G-C-GDRQLT--  146
_NXM25057.1_      115  IDA--------CKKGYNP--GLLVHPEL----------------------GYLQ-AE-G-C-GDRQLT--  146
_NXA89423.1_      115  IDA--------CKKGYNP--GLLVHPEL----------------------GYLQ-AE-G-C-GDRQLT--  146
_NXB19967.1_      115  IDA--------CKKGYNP--GLLVHPEL----------------------SYLQ-AE-G-C-GDRQLT--  146
_NXP82485.1_      115  IDA--------CKKGYNP--GLLVHPEL----------------------AYLQ-AD-G-C-GDRQLT--  146
_NWW54706.1_      115  IDA--------CKKGYNP--GLLVHPEL----------------------GYLQ-AE-G-C-GDRQLT--  146
_NXF96805.1_      115  IDA--------CKKGYNP--GLLVHPEL----------------------AYLQ-AD-G-C-GDRQLT--  146
_NXY35925.1_      115  IDA--------CKKGYNP--GLLVHPEL----------------------GYLQ-AE-G-C-GDRQLT--  146
_XP_037236065.1_  115  IDA--------CKKGYNP--GLLVHPEL----------------------GYLQ-AE-G-C-GDRQLT--  146
_NWT99824.1_      115  IDA--------CKKGYNP--GLLVHPEL----------------------SYLQ-AE-G-C-EDRQLT--  146
_XP_052631299.1_  115  IDA--------CKKGYNP--GLLVHPEL----------------------GYLQ-AE-G-C-GDRQLT--  146
_NXO12533.1_      115  IDA--------CKKGYNP--GLLVHPEL----------------------GYLQ-AE-G-C-GDRQLT--  146
_NWZ34533.1_      115  IDA--------CKKGYNP--GLLVHPEL----------------------GYLQ-AE-G-C-GDRQLT--  146
_NXA30360.1_      115  IDA--------CKKGYNP--GLLVHPEL----------------------GYLQ-AE-G-S-GDRQLT--  146
_NWU09820.1_      115  IDA--------CKKGYNP--GLLVHPEL----------------------GYLQ-AE-G-C-GDRQLT--  146
_NXO38268.1_      115  IDA--------CKKGYNP--GLLVHPEL----------------------GYLQ-AE-G-C-GDRQLT--  146
_NXY70755.1_      115  IDA--------CKKGYNP--GLLVHPEL----------------------GYLQ-AE-G-C-GDRQLT--  146
_RLW08972.1_      115  IDA--------CKKGYNP--GLLVHPEL----------------------GYLQ-AE-G-C-EDRQLT--  146
_XP_013155126.2_  115  IDA--------CKKGYNP--GLLVHPEL----------------------GYLQ-AE-G-C-GDRQLT--  146
_XP_053830927.1_  115  IDA--------CKKGYNP--GLLVHPEL----------------------SYLQ-AE-G-C-EDRQLT--  146
_XP_040454531.1_  115  IDA--------CKKGYNP--GLLVHPEL----------------------GYLQ-AE-G-C-GDRQLT--  146
_XP_049671253.1_  115  IDA--------CKKGYNP--GLLVHPEL----------------------GYLQ-AE-G-C-GDRQLT--  146
_XP_050829478.1_  115  IDA--------CKKGYNP--GLLVHPEL----------------------SYLQ-AE-G-C-EDRQLT--  146
_XP_029818864.1_  115  IDA--------CKKGYNP--GLLVHPEL----------------------GYLQ-AE-G-C-GDRQLT--  146
_NWV77110.1_      115  IDA--------CKKGYNP--GLLVHPEL----------------------GYLQ-AD-G-C-GDRQLT--  146
_NXW55588.1_      115  IDA--------CKKGYNP--GLLVHPEL----------------------GYLQ-AD-G-C-GDRQLT--  146
_NXK11347.1_      115  IDA--------CKKGYNP--GLLVHPEL----------------------GYLQ-AE-G-C-GDRQLT--  146
_XP_037236074.1_  115  IDA--------CKKGYNP--GLLVHPEL----------------------GYLQ-AE-G-C-GDRQLT--  146
_NXM20845.1_      115  IDA--------CKKGYNP--GLLVHPEL----------------------SYLQ-AE-G-C-EDRQLT--  146
_XP_009472960.1_  115  IDA--------CKKGYNP--GLLVHPEL----------------------GYLQ-AE-G-C-GDRQLT--  146
_NXP26913.1_      115  IDA--------CKKGYNP--GLLVHPEL----------------------GYLQ-AE-G-C-GDRQLT--  146
_NXB10880.1_      115  IDA--------CKKGYNP--GLLVHPEL----------------------GYLQ-AE-G-C-GDRQLT--  146
_NXQ83786.1_      115  IDA--------CKKGYNP--GLLVHPEL----------------------GYLQ-AE-G-C-GDRQLT--  146
_XP_041571848.1_  115  IDA--------CKKGYNP--GLLVHPEL----------------------SYLQ-AE-G-C-EDRQLT--  146
_NXS86984.1_      115  IDA--------CKKGYNP--GLLVHPEL----------------------GYLQ-AE-G-C-GDRQLT--  146
_XP_031966433.1_  115  IDA--------CKKGYNP--GLLVHPEL----------------------SYLQ-AE-G-C-GDRQLT--  146
```

```
_BAA02872.1_       115  IDA--------CKKGYNP--GLLVHPEL----------------------GYLQ-AE-G-C-GDRQLT--  146
_XP_051473650.1_   115  IDA--------CKKGYNP--GLLVHPEL----------------------AYLQ-AE-G-C-GDRQLT--  146
_XP_046771508.1_   115  IDA--------CKKGYNP--GLLVHPEL----------------------GYLQ-AE-G-C-GDRQLT--  146
_XP_033920424.1_   115  IDA--------CKKGYNP--GLLVHPEL----------------------GYLQ-AE-G-C-GDRQLT--  146
_NXK78645.1_       115  IDA--------CKKGYNP--GLLVHPEL----------------------GYLQ-AE-G-C-GDRQLT--  146
_XP_014795980.1_   115  IDA--------CKKGYNP--GLLVHPEL----------------------GYLQ-AE-G-C-GDRQLT--  146
_NXN79327.1_       115  IDA--------CKKGYNP--GLLVHPEL----------------------SYLQ-AE-G-C-GDRQLT--  146
_XP_041905595.1_   115  IDA--------CKKGYNP--GLLVHPEL----------------------SYLQ-AE-G-C-GDRQLT--  146
_KQK83110.1_       115  IDA--------CKKGYNP--GLLVHPEL----------------------GYLQ-AE-G-C-GDRQLT--  146
_NP_001383324.1_   115  IDA--------CKKGYNP--GLLVHPEL----------------------GYLQ-AE-G-C-GDRQLT--  146
_NWX31294.1_       115  IDA--------CKKGYNP--GLLVHPEL----------------------GYLQ-AE-G-C-GDRQLT--  146
_XP_050194007.1_   115  IDA--------CKKGYNP--GLLVHPEL----------------------GYLQ-AE-G-C-GDRQLT--  146
_NXP05826.1_       115  IDA--------CKKGYNP--GLLVHPEL----------------------AYLQ-AE-G-C-GERQLT--  146
_XP_027759894.1_   115  IDA--------CKKGYNP--GLLVHPEL----------------------GYLQ-AE-G-C-GDRQLT--  146
_XP_009873772.1_   115  IDA--------CKKGYNP--GLLVHPEL----------------------GYLQ-AE-G-C-GDRQLT--  146
_XP_053796910.1_   115  IDA--------CKKGYNP--GLLVHPEL----------------------SYLQ-AE-G-C-EDRQLT--  146
_XP_039406722.1_   115  IDA--------CKKGYNP--GLLVHPEL----------------------SYLQ-AE-G-C-GDRQLT--  146
_NWU26823.1_       115  IDA--------CKKGYNP--GLLVHPEL----------------------SYLQ-AE-G-C-GDRQLT--  146
_XP_051473648.1_   115  IDA--------CKKGYNP--GLLVHPEL----------------------AYLQ-AE-G-C-GDRQLT--  146
_Q04861.2_         115  IDA--------CKKGYNP--GLLVHPEL----------------------GYLQ-AE-G-C-GDRQLT--  146
_NXL17972.1_       115  IDA--------CKKGYNP--GLLVHPEL----------------------SYLQ-AE-G-C-EDRQLT--  146
_XP_054239251.1_   115  IDA--------CKKGYNP--GLLVHPEL----------------------AYLQ-AD-G-C-GDRQLT--  146
_AAB58343.1_       115  IDA--------CKKGYNP--GLLVHPEL----------------------GYLQ-AE-G-C-GDRQLT--  146
_NXO56761.1_       115  IDA--------CKKGYNP--GLLVHPEL----------------------GYLQ-AE-G-C-GDRQLT--  146
_XP_021251730.1_   115  IDA--------CKKGYNP--GLLVHPEL----------------------GYLQ-AE-G-C-GDRQLT--  146
_XP_015717578.1_   115  IDA--------CKKGYNP--GLLVHPEL----------------------GYLQ-AE-G-C-GDRQLT--  146
_NWX21242.1_       115  IDA--------CKKGYNP--GLLVHPEL----------------------AYLQ-AE-G-S-GDRQLT--  146
_NXR39429.1_       115  IDA--------CKKGYNP--GLLVHPEL----------------------GYLQ-AE-G-C-GDRQLT--  146
_XP_027532933.1_   115  IDA--------CKKGYNP--GLLVHPEL----------------------GYLQ-AE-G-C-GDRQLT--  146
_NXT85173.1_       115  IDA--------CKKGYNP--GLLVHPEL----------------------GYLQ-AE-G-C-GDRQLT--  146
_XP_030803683.1_   115  IDA--------CKKGYNP--GLLVHPEL----------------------SYLQ-AE-G-C-EDRQLT--  146
_XP_041340240.1_   115  IDA--------CKKGYNP--GLLVHPEL----------------------SYLQ-AE-G-C-EDRQLT--  146
_NWT29590.1_       115  IDA--------CKKGYNP--GLLVHPEL----------------------SYLQ-AE-G-C-EDRQLT--  146
_NXH28317.1_       115  IDA--------CKKGYNP--GLLVHPEL----------------------SYLQ-AD-G-C-GDRQLT--  146
_NWS12389.1_       115  IDA--------CKKGYNP--GLLVHPEL----------------------GYLQ-AE-G-C-GDRQLT--  146
_NXF08296.1_       115  IDA--------CKKGYNP--GLLVHPEL----------------------GYLQ-AE-G-C-GDRQLT--  146
_NXP45252.1_       115  IDA--------CKKGYNP--GLLVHPEL----------------------GYLQ-AE-G-C-GDRQLT--  146
_XP_048159384.1_   115  IDA--------CKKGYNP--GLLVHPEL----------------------SYLQ-AE-G-C-GDRQLT--  146
_NXG13678.1_       115  IDA--------CKKGYNP--GLLVHPEL----------------------GYLQ-AE-G-C-GDRQLT--  146
```

```
_NXG81801.1_      106  IDA--------CKKGYNP--GLLVHPEL----------------------GYLQ-AE-G-C-GDRQLT--  137
_NXM64740.1_      115  IDA--------CKKGYNP--GLLVHPEL----------------------SYLQ-AE-G-C-GDRQLT--  146
_NXJ10506.1_      115  IDA--------CKKGYNP--GLLVHPEL----------------------GYMQ-AE-G-C-GDRQLT--  146
_XP_005418238.1_  115  IDA--------CKKGYNP--GLLVHPEL----------------------SYLQ-AE-G-C-EDRQLT--  146
_XP_009078875.1_  115  IDA--------CKKGYNP--GLLVHPEL----------------------GYLQ-AE-G-C-GDRQLT--  146
_XP_010204515.1_  115  TDA--------CKKGYNP--GLLVHPEL----------------------GYLQ-AE-G-C-GERQLT--  146
_XP_008939135.1_  115  IDA--------CKKGYNP--GLLVHPEL----------------------GYLQ-AD-G-C-GDRQLT--  146
_NXA77498.1_      115  IDA--------CKKGYNP--GLLVHPEL----------------------SYLQ-AE-G-C-GDRQLT--  146
_XP_025906692.1_  115  IDA--------CKKGYNP--GLLVHPEL----------------------GYLQ-AE-G-C-GDRQLT--  146
_NWS73106.1_      115  IDA--------CKKGYNP--GLLVHPEL----------------------GYLQ-AD-G-C-GDRQLT--  146
_XP_042726775.1_  115  IDA--------CKKGYNP--GLLVHPEL----------------------GYLQ-AE-G-C-GDRQLT--  146
_XP_031464691.1_  115  IDA--------CKKGYNP--GLLVHPEL----------------------GYLQ-AE-G-C-GDRQLT--  146
_KFP33485.1_      115  TDA--------CKKGYNP--GLLVHPEL----------------------GYLQ-AE-G-C-GERQLT--  146
_XP_054146082.1_  115  IDA--------CKKGYNP--GLLVHPEL----------------------SYLQ-AE-G-C-EDRQLT--  146
_XP_041280451.1_  115  IDA--------CKKGYNP--GLLVHPEL----------------------GYLQ-AE-G-C-EDRQLT--  146
_XP_021387126.1_  115  IDA--------CKKGYNP--GLLVHPEL----------------------SYLQ-AE-G-C-EDRQLT--  146
_NXH08922.1_      115  IDA--------CKKGYNP--GLLVHPEL----------------------SYLQ-AE-G-C-EDRQLT--  146
_NWV89860.1_      115  IDA--------CKKGYNP--GLLVHPEL----------------------SYLQ-AE-G-C-GDRQLT--  146
_NWS31855.1_      115  IDA--------CKKGYNP--GLLVHPEL----------------------SYLQ-AE-G-C-GDRQLT--  146
_NXC72334.1_      115  IDA--------CKKGYNP--GLLVHPEL----------------------GYLQ-AD-G-C-GDRQLT--  146
_KAJ7408913.1_    115  IDA--------CKKGYNP--GLLVHPEL----------------------GYLQ-AE-G-C-GDRQLT--  146
_KFV88798.1_      115  IDA--------CKKGYNP--GLLVHPEL----------------------GYLQ-AE-G-C-GDRQLT--  146
_NXF34295.1_      115  IDA--------CKKGYNP--GLLVHPEL----------------------GYLQ-AE-G-C-GDRQLT--  146
_NWR93222.1_      115  IDA--------CKKGYNP--GLLVHPEL----------------------GYLQ-AE-G-C-GDRQLT--  146
_NXR48213.1_      115  IDA--------CKKGYNP--GLLVHPEL----------------------SYLQ-AE-G-C-GDRQLT--  146
_NXY44121.1_      107  IDA--------CKKGYNP--GLLVHPEL----------------------GHLQ-AD-G-G-GDRQLT--  138
_XP_009935286.1_  115  IDA--------CKKGYNP--GLLVHPEL----------------------GYLQ-AE-G-C-GDRQLT--  146
_XP_026704017.1_  115  IDA--------CKKGYNP--GLLVHPEL----------------------GYLQ-AE-G-C-GDRQLT--  146
_XP_009513483.1_  115  IDA--------CKKGYNP--GLLVHPEL----------------------SYLQ-AD-G-C-GDRQLT--  146
_OXB77562.1_      115  IDA--------CKKGYNP--GLLVHPEL----------------------GYLQ-AE-G-C-GDRQLT--  146
_XP_026647812.1_  115  IDA--------CKKGYNP--GLLVHPEL----------------------SYLQ-AE-G-C-EDRQLT--  146
_NXC04840.1_      115  IDA--------CKKGYNP--GLLVHPEL----------------------SYLQ-AE-G-C-GDRQLT--  146
_XP_009990600.1_  115  IDA--------CKKGYNP--GLLVHPEL----------------------GYLQ-AE-G-C-GDRQLT--  146
_NXW31285.1_      107  IDA--------CKKGYNP--GLLVHPEL----------------------GYLQ-AE-G-C-GDRQLT--  138
_AAA49000.1_      115  IDA--------CKKGYNP--GLLVHPEL----------------------GYLQ-AE-G-C-GDRQLT--  146
_XP_032540773.1_  115  IDA--------CKKGYNP--GLLVHPEL----------------------GYLQ-AE-G-C-GDRQLT--  146
_XP_054489767.1_  115  IDA--------CKKGYNP--GLLVHPEL----------------------SYLQ-AE-G-C-EDRQLT--  146
_NXI38931.1_      115  IDA--------CKKGYNP--GLLVHPEL----------------------DYLQ-AD-G-C-GDRQLT--  146
_NWZ75946.1_      107  IDA--------CKKGYNP--GLLVHPEL----------------------GYLQ-AE-G-C-GDRQLT--  138
```

```
_TRZ15085.1_       115  IDA--------CKKGYNP--GLLVHPEL----------------------NYLQ-AE-G-C-GDRQLT--  146
_NWT43768.1_       107  IDA--------CKKGYNP--GLLVHPEL----------------------DYLQ-AE-G-C-GDRQLT--  138
_XP_042667699.1_   115  IDA--------CKKGYNP--GLLVHPEL----------------------SYLQ-AE-G-C-GDRQLT--  146
_KFW75731.1_       115  IDA--------CKKGYNP--GLLVHPEL----------------------GYLQ-AE-G-C-GDRQLT--  146
_XP_048796949.1_   115  IDA--------CKKGYNP--GLLVHPEL----------------------GYLQ-AE-G-C-GDRQLT--  146
_NWR08112.1_       115  IDA--------CKKGYNP--GLLVHPEL----------------------GYLQ-AE-G-C-GDRQLT--  146
_KAJ7414413.1_     115  IDA--------CKKGYNP--GLLVHPEL----------------------GYLQ-AE-G-C-GDRQLT--  146
_NWI28654.1_       115  IDA--------CKKGYNP--GLLVHPEL----------------------SYLQ-AD-G-C-GDRQLT--  146
_NXQ74308.1_       115  IDA--------CKKGYNP--GLLVHPEL----------------------SYLQ-AE-G-C-EDRQLT--  146
_NWR61490.1_       115  IDA--------CKKGYNP--GLLVHPEL----------------------GYLQ-AE-G-C-GDRQLT--  146
_NXW00255.1_       107  IDA--------CKKGYNP--GLLVHPEL----------------------GYLQ-AE-G-C-GDRQLT--  138
_XP_010131965.1_   115  IDA--------CRKGYNP--GLLVHPEL----------------------GYLQ-AE-G-C-GDRQLT--  146
_KAF1499115.1_     107  IDA--------CKKGYNP--GLLVHPEL----------------------GYLQ-AE-G-C-GDRQLT--  138
_XP_054018945.1_   115  IDA--------CKRGYNP--GLLVHPEL----------------------TYLQ-AD-G-C-GDRQLT--  146
_XP_052539264.1_   115  IDA--------CKKGYNP--GLLVHPEL----------------------SYLQ-AE-G-C-GDRQLT--  146
_NXC30330.1_       115  IDA--------CKKGYNP--GLLVHPEL----------------------GYLQ-AD-G-C-GDRQLT--  146
_KFR08581.1_       115  IDA--------CKKGYNP--GLLVHPEL----------------------GYLQ-AE-G-C-GDRQLT--  146
_XP_039557931.1_   115  IDA--------CKKGYNP--GLLVHPEL----------------------SYLQ-AE-G-C-EDRQLT--  146
_KAF1486784.1_     107  IDA--------CKKGYNP--GLLVHPEL----------------------GYLQ-AE-G-C-GDRQLT--  138
_OXB59608.1_       115  IDA--------CKKGYNP--GLLVHPEL----------------------GYLQ-AE-G-C-GDRQLT--  146
_XP_021138721.1_   115  IDA--------CKKGYNP--GLLVHPEL----------------------GYLQ-AD-A-C-GDRQLT--  146
_KAF1514432.1_     107  IDA--------CKKGYNP--GLLVHPEL----------------------GYLQ-AE-G-C-GDRQLT--  138
_NWV14170.1_       115  IDA--------CKKGYNP--GLLVHPEL----------------------GYLQ-AE-G-C-GDRQLT--  146
_KAF1548068.1_     107  IDA--------CKKGYNP--GLLVHPEL----------------------GYLQ-AE-G-C-GDRQLT--  138
_NXI51730.1_       115  IDA--------CKKGYNP--GLLVHPEL----------------------GYLQ-AD-G-C-GDRQLT--  146
_NXE50896.1_       107  IDA--------CKKGYNP--GLLVHPEL----------------------GYLQ-AE-G-C-GDRQLT--  138
_NXT41700.1_       107  IDA--------CKKGYNP--GLLVHPEL----------------------GYLQ-AE-G-C-GDRQLT--  138
_KAF1659387.1_     107  IDA--------CKKGYNP--GLLVHPEL----------------------GYLQ-AE-G-C-GDRQLT--  138
_NWR74867.1_       107  IDA--------CKKGYNP--GLLVHPEL----------------------GYLQ-AD-G-C-GDRQLT--  138
_NXV33343.1_       107  IDA--------CKKGYNP--GLLVHPEL----------------------DYLQ-AE-G-C-GDRQLT--  138
_NXW99502.1_       107  IDA--------CKKGYNP--GLLVHPEL----------------------DYLQ-AE-G-C-GDRQLT--  138
_NXV14161.1_       107  IDA--------CKKGYNP--GLLVHPEL----------------------GYLQ-AE-G-C-GDRQLT--  138
_NWX63354.1_       107  IDA--------CKKGYNP--GLLVHPEL----------------------SYLQ-AE-G-C-GDRQLT--  138
_NXL58318.1_       107  IDA--------CKKGYNP--GLLVHPEL----------------------GYLQ-AE-G-C-GDRQLT--  138
_NWV41036.1_       107  IDA--------CKKGYNP--GLLVHPEL----------------------SYLQ-AE-V-C-GDRQLT--  138
_KAF1609011.1_     107  IDA--------CKKGYNP--GLLVHPEL----------------------GYLQ-AE-G-C-GDRQLT--  138
_NWT52190.1_       115  IDA--------CKKGYNP--GLLVHPEL----------------------GYLQ-AE-G-C-GDRQLT--  146
_NXO93770.1_       115  IDA--------CKKGYNP--GLLVHPEL----------------------SYLQ-AE-G-C-GDRQLT--  146
_NXI62681.1_       115  IDA--------CKKGYNP--GLLVHPEL----------------------GYLQ-AE-G-C-GDRQLT--  146
```

NXH21058.1	115	IDA--------CKKGYNP--GLLVHPEL----------------------GYLQ-AD-G-C-GDRQLT--	146
KFV75676.1	115	IDA--------CKRGYNP--GLLVHPEL----------------------TYLQ-AD-G-C-GDRQLT--	146
KAF1435617.1	107	IDA--------CKKGYNP--GLLVHPEL----------------------GYLQ-AE-G-C-GDRQLT--	138
KAF1479350.1	107	IDA--------CKKGYNP--GLLVHPEL----------------------GYLQ-AE-G-C-GDRQLT--	138
NXW86014.1	107	IDA--------CKKGYNP--GLLVHPEL----------------------GYLQ-AD-A-C-GDRQLT--	138
KAF1483835.1	107	IDA--------CKKGYNP--GLLVHPEL----------------------GYLQ-AE-G-C-GDRQLT--	138
NWW87838.1	115	IDA--------CKKGYNP--GLLVHPEL----------------------GYLQ-AE-G-C-GERQLT--	146
NXF79309.1	115	IDA--------CKKGYNP--GLLVHPEL----------------------GYLQ-AE-G-C-GDRQLT--	146
_XP_030305668.1_	115	IDA--------CKKGYNP--GLLVHPEL----------------------AYLQ-AD-G-C-GDRQLT--	146
NXS27685.1	115	IDA--------CKKGYNP--GLLVHPEL----------------------GYLQ-AE-G-C-GDRQLT--	146
_XP_036237629.1_	115	IDA--------CKKGYNP--GLLVHPEL----------------------SYLQ-AE-G-C-EDRQLT--	146
NXM97765.1	115	IDA--------CKKGYNP--GLLVHPEL----------------------SYLQ-AE-G-C-GDRQLT--	146
KAF2988548.1	115	IDA--------CKKGYNP--GLLVHPEL----------------------SYLQ-AE-G-C-EDRQLT--	146
NWH87727.1	115	IDA--------CKKGYNP--GLLVHPEL----------------------GYLQ-AE-G-C-GDRQLT--	146
NXR25130.1	107	IDA--------CKKGYNP--GLLVHPEL----------------------SYLQ-AE-G-C-GDRQLT--	138
NXV50350.1	107	IDA--------CKKGYNP--GLLVHPEL----------------------GYLQ-AE-G-C-GDRQLT--	138
NWT72232.1	107	IDA--------CKKGYNP--GLLVHPEL----------------------SYLQ-AE-G-C-EDRQLT--	138
KAF1435460.1	107	IDA--------CKKGYNP--GLLVHPEL----------------------GYLQ-AE-G-C-GDRQLT--	138
KAF1424376.1	107	IDA--------CKKGYNP--GLLVHPEL----------------------GYLQ-AE-G-C-GDRQLT--	138
NWX41614.1	107	IDA--------CKKGYNP--GLLVHPEL----------------------GYLQ-AE-G-C-GDRQLT--	138
NXD28887.1	107	IDA--------CKKGYNP--GLLVHPEL----------------------GYLQ-AE-G-C-GDRQLT--	138
NXY59218.1	107	IDA--------CKKGYNP--GLLVHPEL----------------------GYLQ-AE-G-C-GDRQLT--	138
KAF1399315.1	107	IDA--------CKKGYNP--GLLVHPEL----------------------GYLQ-AE-G-C-GDRQLT--	138
NXJ95672.1	107	IDA--------CKKGYNP--GLLVHPEL----------------------GYLQ-AE-G-C-GDRQLT--	138
NWW19682.1	107	IDA--------CKKGYNP--GLLVHPEL----------------------SYLQ-AE-G-C-GDRQLT--	138
NWU76879.1	107	IDA--------CKKGYNP--GLLVHPEL----------------------GYLQ-AE-G-C-GDRQLT--	138
NWY75431.1	107	IDA--------CKKGYNP--GLLVHPEL----------------------SYLQ-AE-G-C-GDRQLT--	138
NXX32566.1	107	IDA--------CKKGYNP--GLLVHPEL----------------------GYLQ-AE-G-C-GDRQLT--	138
NXL48469.1	107	IDA--------CKKGYNP--GLLVHPEL----------------------GYLQ-AE-G-C-GDRQLT--	138
NXN66426.1	107	IDA--------CKKGYNP--GLLVHPEL----------------------GYLQ-AE-G-S-GDRQLT--	138
NXV94077.1	107	IDA--------CKKGYNP--GLLVHPEL----------------------GYLQ-AE-G-C-GDRQLT--	138
KFO88230.1	115	IDA--------CRKGYNP--GLLVHPEL----------------------GYLQ-AE-G-C-GDRQLT--	146
NXD82005.1	115	IDA--------CKKGYNP--GLLVHPEL----------------------GYLQ-AD-G-C-GDRQLT--	146
NWY36867.1	107	IDA--------CKKGYNP--GLLVHPEL----------------------SYLQ-AE-G-C-GDRQLT--	138
NWZ53691.1	107	IDA--------CKKGYNP--GLLVHPEL----------------------GYLQ-AE-G-C-GDRQLT--	138
NXE95961.1	107	IAA--------CKKGYNP--GLLVHPEL----------------------GYLQ-AE-G-C-GDRQLT--	138
NXB76170.1	107	IDA--------CKKGYNP--GLLVHPEL----------------------GYLQ-AE-G-C-GDRQLT--	138
NWR51229.1	107	IDA--------CKKGYNP--GLLVHPEL----------------------GYLQ-AE-G-C-GDRQLT--	138
NXB96832.1	107	IDA--------CKKGYNP--GLLVHPEL----------------------SYLQ-AE-G-C-EDRQLT--	138

```
_NXW19741.1_      107  IDA--------CKKGYNP--GLLVHPEL----------------------GYLQ-AE-G-C-GDRQLT--  138
_NWV61316.1_      107  IDA--------CKKGYNP--GLLVHPEL----------------------SYLQ-AE-G-C-GDRQLT--  138
_NXD68485.1_      107  IDA--------CKKGYNP--GLLVHPEL----------------------GYLQ-AE-G-C-GDRQLT--  138
_XP_038033784.1_  115  IDA--------CKKGYNP--GLLVHPEL----------------------GHLQ-AE-G-C-GERQLT--  146
_XP_009918158.1_  115  IDA--------CKKGYNP--GLLVHPEL----------------------GYLQ-AE-G-C-GDRQLT--  146
_NWI62302.1_      107  IDA--------CKKGYNP--GLLVHPEL----------------------GYLQ-AD-G-C-GDRQLT--  138
_NXV82871.1_      107  IDA--------CKKGYNP--GLLVHPEL----------------------GYLQ-AE-G-C-GDRQLT--  138
_NXX21988.1_      107  IDA--------CKKGYNP--ELLVHPEL----------------------GCLQ-AE-G-C-GDRQLT--  138
_NWZ28840.1_      115  IDA--------CKKGYNP--GLLVHPEL----------------------GHLQ-AE-G-C-GDRQLT--  146
_NXA68287.1_      107  IDA--------CKKGYNP--GLLVHPEL----------------------GYLQ-AE-G-C-GDRQLT--  138
_NXL73547.1_      106  IDA--------CKKGYNP--GLLVHPEL----------------------SYLQ-AE-G-C-EDRQLT--  137
_NXV08833.1_      107  IDA--------CKKGYNP--GLLVHPEL----------------------GYLQ-AE-G-C-GDRQLT--  138
_NWY20334.1_      107  IDA--------CKKGYNP--GLLVHPEL----------------------SYLQ-AE-G-C-GDRQLT--  138
_NXA13401.1_      107  IDA--------CKKGYNP--GLLVHPEL----------------------GYLQ-AE-G-C-GERQLT--  138
_NXY15168.1_      107  IDA--------CKKGYNP--GLLVHPEL----------------------GYLQ-AE-G-C-GDRQLT--  138
_NXJ48973.1_      115  IDA--------CKKGYNP--GLLVHPEL----------------------GYLQ-AE-G-C-GDRQLT--  146
_KAI6070014.1_    115  IDA--------CKKGYNP--GLLVHPEL----------------------GYLQ-AE-A-C-GDRQLT--  146
_NXH91756.1_      106  IDA--------CKKGYNP--GLLVHPEL----------------------GYLQ-AE-G-C-GDRQLT--  137
_NXL04686.1_      106  IDA--------CKKGYNP--GLLVHPEL----------------------SYLQ-AE-G-C-GDRQLT--  137
_XP_040411521.1_  115  IDA--------CKKGYNP--GLLVHPEL----------------------GYLQ-AE-G-C-GDRQLT--  146
_XP_035395635.1_  115  IDA--------CKKGYNP--GLLVHPEL----------------------GYLQ-AE-G-C-GDRQLT--  146
_NXU37184.1_      107  IDA--------CKKGYNP--GLLVHPEL----------------------GYMQ-AE-G-C-GERQLT--  138
_NWV21895.1_      107  IDA--------CKKGYNP--GLLVHPEL----------------------SYLQ-AE-E-C-GDRQLT--  138
_NXY81878.1_      115  IDA--------CKKGYNP--GLLVHPEL----------------------GYLQ-AD-G-C-GDRQLT--  146
_NXH41439.1_      106  IDA--------CKKGYNP--GLLVHPEL----------------------SYLQ-AE-G-C-EDRQLT--  137
_NWW81943.1_      107  IDA--------CKKGYNP--GLLVHPEL----------------------GYLQ-AE-G-C-GDRQLT--  138
_NXJ61295.1_      107  IDA--------CKKGYNP--GLLVHPEL----------------------GYLQ-AE-G-C-GDRQLT--  138
_NXN34085.1_      107  IDA--------CKKGYNP--GLLVHPEL----------------------GYLQ-AE-G-C-GDRQLT--  138
_NXK62748.1_      108  IDA--------CKKGYNP--GLLVHPEL----------------------GYLQ-AE-G-C-GDRQLT--  139
_NWH66460.1_      107  IDA--------CKKGYNP--GLLVHPEL----------------------GYLQ-AD-G-C-GDRQLT--  138
_NWS97842.1_      107  IDA--------CKKGYNP--GLLVHPEL----------------------GYLQ-AE-G-C-GDRQLT--  138
_XP_013051206.2_  115  IDA--------CKKGYNP--GLLVHPEL----------------------GYLQ-AE-G-C-GDRQLT--  146
_NWY58828.1_      107  IDA--------CKKGYNP--GLLVHPEL----------------------GYLQ-AE-G-S-GDRQLT--  138
_NWR27345.1_      107  IDA--------CKKGYNP--GLLVHPEL----------------------GYLQ-AE-G-C-GDRQLT--  138
_NXM04963.1_      106  IDA--------CKKGYNP--GLLVHPEL----------------------GYLQ-AE-G-C-GDRQLT--  137
_NWZ13908.1_      107  IDA--------CKKGYNP--GLLVHPEL----------------------SYLQ-AE-G-C-EDRQLT--  138
_NWI56539.1_      107  IDA--------CKKGYNP--GLLVHPEL----------------------GYLQ-AE-G-C-GDRQLT--  138
_NXL35478.1_      107  IDA--------CKKGYNP--GLLVHPEL----------------------GYLQ-AE-G-C-GDRQLT--  138
_NWY90605.1_      107  IDA--------CKKGYNP--GLLVHPEL----------------------SYLQ-AE-G-C-EDRQLT--  138
```

```
_NXF23305.1_       107  IDA--------CKKGYNP--GLLVHPEL----------------------SYLQ-AE-G-C-EDRQLT--  138
_XP_035182229.1_   115  IDA--------CKKGYNP--GLLVHPEL----------------------GYLQ-AE-G-R-GDRQLT--  146
_EOB02688.1_       115  IDA--------CKKGYNP--GLLVHPEL----------------------GHLQ-AE-G-C-GERQLT--  146
_NXP86430.1_       107  IDA--------CKKGYNP--GLLVHPEL----------------------SYLQ-AE-G-C-EDRQLT--  138
_NXK90683.1_       115  IDA--------CKKGYNP--GLLVHPEL----------------------GYLQ-AE-G-C-GDRQLT--  146
_NWU68452.1_       107  IDA--------CKKGYNP--GLLVHPEL----------------------GYLQ-AE-G-C-GDRQLT--  138
_NXT19433.1_       107  IDA--------CKKGYNP--GLLVHPEL----------------------GYLQ-AE-G-C-GDRQLT--  138
_NXV56948.1_       107  IDA--------CKKGYNP--GLLVHPEL----------------------SYLQ-AE-G-C-EDRQLT--  138
_NXX85020.1_       107  IDA--------CKKGYNP--GLLVHPEL----------------------GYLQ-AE-G-C-GDRQLT--  138
_NWY25110.1_       107  IDA--------CKKGYNP--GLLVHPEL----------------------SYLQ-AE-G-C-EDRQLT--  138
_XP_032041859.1_   115  IDA--------CKKGYNP--GLLVHPEL----------------------GHLQ-AE-G-C-GDRQLT--  146
_NWZ68267.1_       107  IDA--------CKKGYNP--GLLVHPEL----------------------GYLQ-AE-G-C-GDRQLT--  138
_NXG60352.1_       107  IDA--------CKKGYNP--GLLVHPEL----------------------AYLQ-AE-G-C-GDRQLT--  138
_NXT59875.1_       107  IDA--------CKKGYNP--GLLVHPEL----------------------GYLQ-AE-G-C-GDRQLT--  138
_NWY04604.1_       107  IDA--------CKKGYNP--GLLVHPEL----------------------GYLQ-AE-G-C-GDRQLT--  138
_NXC95671.1_       107  IDA--------CKKGYNP--GLLVHPEL----------------------SYLQ-AE-G-C-GDRQLT--  138
_NXD14679.1_       107  IDA--------CKKGYNP--ELLVHPEL----------------------GYLQ-AE-G-C-GDRQLT--  138
_NWX87899.1_       107  IDA--------CKKGYNP--GLLVHPEL----------------------GYLQ-AE-G-C-GDRQLT--  138
_NWZ96914.1_       107  IDA--------CKKGYNP--GLLVHPEL----------------------SYLQ-AE-G-C-EDRQLT--  138
_KFP08036.1_       115  IDA--------CKKGYNP--GLLVHPEL----------------------AYLQ-AD-G-C-GDRQLT--  146
_NXJ81100.1_       107  IDA--------CKKGYNP--GLLVHPEL----------------------GYLQ-AE-G-C-GDRQLT--  138
_KAF1419467.1_     107  IDA--------CKKGYNP--GLLVHPEL----------------------GYLQ-AE-G-C-GDRQLT--  138
_NXA54431.1_       107  IDA--------CKKGYNP--ELLVHPEL----------------------GYLQ-AE-G-C-GDRQLT--  138
_NXI22500.1_       106  IDA--------CKKGYNP--GLLVHPEL----------------------SYLQ-AE-G-C-GDRQLT--  137
_XP_030348001.1_   115  IDA--------CKKGYNP--GLLVHPEL----------------------GYLQ-AE-G-C-GDRQLT--  146
_XP_032041862.1_   115  IDA--------CKKGYNP--GLLVHPEL----------------------GHLQ-AE-G-C-GDRQLT--  146
_XP_054059792.1_   115  IDA--------CKKGYNP--GLLVHPEL----------------------DYLQ-AE-G-C-GDRQLT--  146
_NWI98852.1_       107  IDA--------CKKGYNP--ELLVHPEL----------------------GYLQ-AE-G-C-GDRQLT--  138
_XP_040979578.1_   115  IDA--------CKKGYNP--GLLVHPEL----------------------GYLQ-AE-G-C-GDRQLT--  146
_XP_035182231.1_   115  IDA--------CKKGYNP--GLLVHPEL----------------------GYLQ-AE-G-R-GDRQLT--  146
_NWI17161.1_       107  IDA--------CKKGYNP--ELLVHPEL----------------------GYLQ-AE-G-C-GDRQLT--  138
_XP_017677707.1_   115  IDA--------CKKGYNP--GLLVHPEL----------------------GYLQ-AE-G-C-GDRQLT--  146
_XP_031966434.1_   115  IDA--------CKKGYNP--GLLVHPEL----------------------SYLQ-AE-G-C-GDRQLT--  146
_XP_014795990.1_   115  IDA--------CKKGYNP--GLLVHPEL----------------------GYLQ-AE-G-C-GDRQLT--  146
_XP_041905596.1_   115  IDA--------CKKGYNP--GLLVHPEL----------------------SYLQ-AE-G-C-GDRQLT--  146
_XP_039406723.1_   115  IDA--------CKKGYNP--GLLVHPEL----------------------SYLQ-AE-G-C-GDRQLT--  146
_XP_048159386.1_   115  IDA--------CKKGYNP--GLLVHPEL----------------------SYLQ-AE-G-C-GDRQLT--  146
_NXA36485.1_       107  IDA--------CKKGYNP--GLLVHPEL----------------------GYLQ-AE-G-C-GERQLT--  138
_XP_010001479.1_   115  IDA--------CKKGYNP--GLLVHPEL----------------------AYLQ-AE-G-C-GDRQLT--  146
```

```
_KFU93771.1_      115  IDA--------CKKGYNP--GLLVHPEL----------------------AYLQ-AE-G-C-GDRQLT--  146
_NXE65168.1_      107  IDA--------CKKGYNP--GLLVHPEL----------------------SYLQ-AE-G-C-EDRQLT--  138
_KAF4794888.1_    115  IDA--------CKKGYNP--GLLVHPEL----------------------SYLQ-AE-G-C-GDRQLT--  146
_XP_027532934.1_  115  IDA--------CKKGYNP--GLLVHPEL----------------------GYLQ-AE-G-C-GDRQLT--  146
_XP_046795876.1_  115  IDA--------CKKGYNP--GLLVHPEL----------------------GYLQ-AE-G-C-GDRQLT--  146
_KFV96277.1_      115  IDA--------CKKGYNP--GLLVHPEL----------------------SYLQ-AE-G-C-GERQLT--  146
_XP_010157325.1_  115  IDA--------CKKGYNP--GLLVHPEL----------------------SYLQ-AE-G-C-GERQLT--  146
_NWI02032.1_      115  IDA--------CKKGYNP--GLLVHPEL----------------------GYLQ-AE-G-C-GDRQLT--  146
_XP_035746225.1_  115  IDA--------CKKGYNP--GLLVHPEL----------------------GYLQ-AE-G-C-GDRQLT--  146
_NWH69936.1_      107  IDA--------CKKGYNP--GLLVHPEL----------------------GYLQ-AD-G-C-GDRQLT--  138
_NXH96653.1_      106  IDA--------CKKGYNP--GLLVHPEL----------------------GYLQ-AE-G-C-GDRQLT--  137
_NXN58828.1_      115  IDA--------CKKGYNP--GLLVHPEL----------------------GYLQ-AE-G-C-GDRQLT--  146
_XP_010083670.1_  115  IDA--------CKKGYNP--GLLVHPEL----------------------GYLQ-AE-G-C-GDRQLT--  146
_RMC13788.1_      115  IDA--------CKKGYNP--GLLVHPEL----------------------GYLQ-AE-G-C-GDRQLT--  146
_XP_023801002.1_       ----------------------------------------------------------------------
_KAJ1206281.1_    115  IEA--------FRRGYNA--GLLIHPDI----------------------DYIP-SE-S-G-GERPLT--  146
_XP_029452911.1_  115  IEA--------FRRGYNA--GLLVHPEL----------------------EYVP-GE-H-G-GERQLT--  146
_XP_030046614.1_  115  IEA--------FRRGYNA--GLLVHRDL----------------------DYVP-GE-H-G-VERQLT--  146
_XP_033791760.1_  115  TEA--------FRKGYNA--GLLVHRDL----------------------DYIP-GE-H-S-GERQLT--  146
_NXS74679.1_        1  -----------------------------------------------------------------QLT--    3
_XP_019782049.1_   43  TDA--------CIRGYNP--GLLVHPDL----------------------AYLQ-AE-G-G-GDRQLT--   74
_XP_030700974.1_   43  TDA--------CIRGYNP--GLLVHPDL----------------------AYLQ-AE-G-G-GDRQLT--   74
_XP_026972817.1_   43  TDA--------CIRGYNP--GLLVHPDL----------------------AYLQ-AE-G-G-GDRQLT--   74
_XP_042107134.1_   30  TDA--------CVRGYNP--GLLVHPDL----------------------AYLQ-AE-G-G-GDRQLT--   61
_XP_016807420.1_   27  TEA--------CIRGYNP--GLLVHPDL----------------------AYLQ-AE-G-G-GDRQLG--   58
_XP_031520261.1_   24  TEA--------CIRGYNP--GLLVHPDL----------------------AYLQ-AE-G-G-GDRQLG--   55
_XP_025146162.3_   28  TDA--------CIRGYNP--GLLVHPDL----------------------AYLQ-AE-G-G-GDRQLT--   59
_MXQ81613.1_       28  TDA--------CIRGYNP--GLLVHPDL----------------------AYLQ-AE-G-G-GDRQLT--   59
_XP_014391951.1_   28  TDA--------CIRGYNP--GLLVHPEL----------------------AYLQ-AE-G-G-GDRQLT--   59
_KAF5926647.1_     28  TEA--------CVRGYNP--GLLVHPDL----------------------AYLQ-AE-G-G-GDRQLT--   59
_XP_014315807.1_   28  TDA--------CIRGYNP--GLLVHPEL----------------------AYLQ-AE-G-G-GDRQLT--   59
_XP_044243992.1_   27  TEA--------CTKGYNP--GLLVHPDL----------------------AYLQ-AE-G-G-GDRQLT--   58
_XP_015425475.1_   28  TDA--------CIRGYNP--GLLVHPEL----------------------AYLQ-AE-G-G-GDRQLT--   59
_XP_036196822.1_   28  TDA--------CIRGYNP--GLLVHPEL----------------------AYLQ-AE-G-G-GDRQLT--   59
_XP_036266201.1_   28  TDA--------CIRGYNP--GLLVHPDL----------------------AYLQ-AE-G-G-GERQLT--   59
_XP_025729301.1_   27  TEA--------CTKGYNP--GLLVHPDL----------------------AYLQ-AE-G-G-GDRQLT--   58
_XP_051041401.1_   28  TEA--------CIRGYNP--GLLVHSDL----------------------AYLQ-AE-G-G-GDRQLT--   59
_XP_029330831.1_   27  TEA--------CIRGYNP--GLLVHSDL----------------------AYLQ-AE-G-G-GDRQLT--   58
_XP_021051020.1_   27  TEA--------CIRGYNP--GLLVHSDL----------------------AYLQ-AE-G-G-GDRQLT--   58
```

```
_XP_023493402.1_   27  TEA--------CVRGYNP--EILVHPDL----------------------RYLQ-AE-G-G-GDRQLT--   58
_XP_021101132.1_   27  TDA--------CIRGYNP--GLLVHPDL----------------------AYLQ-AEGG-G-GDRQLT--   59
_BAC35117.1_       27  TEA--------CIRGYNP--GLLVHSDL----------------------AYLQ-AE-G-G-GDRQLT--   58
_XP_006732650.2_   25  IEA--------CTKGYNP--GLLVHPDL----------------------AYLQ-AE-G-G-GDRQLT--   56
_KAB0368408.1_     80  TDA--------CIRGYNP--GLLVHPDL----------------------AYLQ-AE-G-G-GDRQLT--  111
_XP_030675033.1_   77  TEA--------CIRGYNP--GLLVHPDL----------------------AYLQ-AE-G-G-GDRQLG--  108
_XP_012291953.1_   74  TEA--------CIRGYNP--GLLVHPDL----------------------AYLQ-AE-G-G-GDRQLG--  105
_XP_047422690.1_   74  TDA--------CVRGYNP--GLLVHPDL----------------------AYLQ-AE-G-G-GDRQLT--  105
_XP_051675507.1_   77  TEA--------CIRGYNP--GLLVHPDL----------------------AYLQ-TE-G-G-GDRQLT--  108
_XP_025146160.1_   77  TDA--------CIRGYNP--GLLVHPDL----------------------AYLQ-AE-G-G-GDRQLT--  108
_KAB0345267.1_     73  TDA--------CIRGYNP--GLLVHPDL----------------------AYLQ-AE-G-G-GDRQLT--  104
_XP_020014165.1_   76  TDA--------CVRGYNP--GLLVHPEL----------------------GYLQ-AE-G-G-GDRQLT--  107
_XP_026917376.1_   74  TEA--------CTKGYNP--GLLVHPDL----------------------AYLQ-AE-G-G-GDRQLT--  105
_XP_019271516.2_   74  TEA--------CTKGYNP--GLLVHPDL----------------------AYLQ-AE-G-G-GDRQLT--  105
_XP_045327688.1_   74  TEA--------CTKGYNP--GLLVHPDL----------------------AYLQ-AE-G-G-GDRQLT--  105
_XP_023108675.2_   74  TEA--------CTKGYNP--GLLVHPDL----------------------AYLQ-AE-G-G-GDRQLT--  105
_XP_014391950.1_   68  TDA--------CIRGYNP--GLLVHPEL----------------------AYLQ-AE-G-G-GDRQLT--   99
_XP_023614158.1_   68  TDA--------CIRGYNP--GLLVHPEL----------------------AYLQ-AE-G-G-GDRQLT--   99
_XP_032256797.1_   77  TEA--------CTKGYNP--GLLVHPDL----------------------AYLQ-AE-G-G-GDRQLT--  108
_XP_015425474.1_   68  TDA--------CIRGYNP--GLLVHPEL----------------------AYLQ-AE-G-G-GDRQLT--   99
_XP_027709164.1_   75  TDA--------CIRGYNP--GLLVHPEL----------------------NYLQ-PE-G-G-GERQLT--  106
_KAG8519429.1_     77  TDA--------CMRGYNP--GLLVHPDL----------------------AYLQ-AE-G-G-GDRQLT--  108
_KAB1282111.1_     74  MEA--------CIRGYNP--GLLVHPDL----------------------AYLQ-AE-G-G-GDRQLT--  105
_XP_016049280.1_   27  TDA--------CLRGYNP--GLLVHPEL----------------------SRLQ--A-D-G-GERRLT--   57
_OBS59044.1_        1  -----------------------------------------------------------------QLT--    3
_KAI2535356.1_         ----------------------------------------------------------------------
_XP_054846407.1_  115  MDA--------CKKGYNP--GLLVHPEL----------------------GYIQ-AE-G-G-GERQLT--  146
_XP_015261255.1_  115  IDC--------CKKGYNP--GLLVHPEL----------------------SYIQ-VE-G-G-GDRQLT--  146
_XP_028599456.1_  115  IDS--------CKKGYNP--GLLVHPDL----------------------SYIQ-VE-G-G-GERQLT--  146
_QCQ80695.1_      115  IDS--------CKKGYNP--GLLVHPDL----------------------NYIQ-VE-G-G-GERQLT--  146
_XP_048365177.1_  115  MDC--------CKKGYNP--GLLVHPEL----------------------SYIQ-VE-G-G-GDRQLT--  146
_EGW14115.1_           ----------------------------------------------------------------------
_KAJ6668884.1_     27  VDA--------CRKGYNP--GLLVHPDL----------------------NYIQ-VE-G-G-GERQLT--   58
_XP_053108974.1_  115  MDA--------CKKGYNP--GLLVHPEL----------------------SYIQ-VE-G-G-GERQLT--  146
_XP_007429861.1_  115  IDA--------CKKGYNP--GLLVHPDL----------------------NYIQ-VE-G-G-GERQLT--  146
_XP_047654926.1_    1  ----------------------------------------------------------------TWWS--    4
_XP_011884266.1_    1  ------------------------------------------------------------------LG--    2
_KAI2535355.1_         ----------------------------------------------------------------------
_PNJ84388.1_           ----------------------------------------------------------------------
```

```
_XP_038167493.1_     1  -----------------------------------------------------------------QLT--    3
_ERE89661.1_         1  -----------------------------------------------------------------QLT--    3
_XP_038167494.1_     1  -----------------------------------------------------------------QLT--    3
_KAH0615883.1_     115  IDA--------CKKGYNP--GLLIHPDL----------------------GYIQ-VE-G-G-GERQLSVS  148
_XP_020661083.1_   115  IDA--------CKKGYNP--GLLIHPDL----------------------NYIQ-AE-G-G-GERQLT--  146
_XP_053259916.1_   115  IDS--------CKKGYNP--GLLVHPDL----------------------SYIQ-VE-G-G-GERQLT--  146
_XP_044284768.1_   115  IDA--------CKKGYNP--GLLVHPDL----------------------NYIQ-VE-G-G-GERQLS--  146
_XP_034967560.1_   115  IDS--------CKKGYNP--GLLVHPDL----------------------SYIQ-VE-G-G-GERPLT--  146
_XP_033016273.1_   115  IDS--------CKKGYNP--GLLVHPDL----------------------SYIQ-VE-G-G-GERQLT--  146
_KAJ7327088.1_     115  IDA--------CKKGYNP--GFLIHPDL----------------------NYIQ-AE-G-G-GERHLT--  146
_XP_044284772.1_   108  IDA--------CKKGYNP--GLLVHPDL----------------------NYIQ-VE-G-G-GERQLS--  139
_XP_044284769.1_   107  IDA--------CKKGYNP--GLLVHPDL----------------------NYIQ-VE-G-G-GERQLS--  138
_XP_039206640.1_   115  IDA--------CKKGYNP--GLLVHPDL----------------------NYIQ-VE-G-G-GERQLT--  146
_XP_042325082.1_   115  IDA--------CKKGYNP--GLLVHPDL----------------------GYIQ-VE-G-G-GERQLT--  146
_XP_015683855.1_   115  IDA--------CRKGYNP--GLLVHPDL----------------------NYIQ-VE-G-G-GERQLT--  146
_KAF7252349.1_     115  IDA--------CKKGYNP--GLLVHPDL----------------------NYIQ-VE-G-G-GERQLS--  146
_XP_053259920.1_    74  IDS--------CKKGYNP--GLLVHPDL----------------------SYIQ-VE-G-G-GERQLT--  105
_XP_028599459.1_    74  IDS--------CKKGYNP--GLLVHPDL----------------------SYIQ-VE-G-G-GERQLT--  105
_XP_044284775.1_    28  IDA--------CKKGYNP--GLLVHPDL----------------------NYIQ-VE-G-G-GERQLS--   59
_XP_039206641.1_    25  IDA--------CKKGYNP--GLLVHPDL----------------------NYIQ-VE-G-G-GERQLT--   56
_XP_013807108.1_    31  IDA--------CKKGYNP--GLLVHPEL----------------------GYLQ-AE-G-C-GDRQLT--   62
_XP_042664115.1_    27  IDA--------CKKGYNP--GLLVHPEL----------------------GYLQ-AE-G-C-GDRQLT--   58
_XP_042664114.1_    31  IDA--------CKKGYNP--GLLVHPEL----------------------GYLQ-AE-G-C-GDRQLT--   62
_NXS19671.1_        23  IDA--------CKKGYNP--GLLVHPEL----------------------SYLQ-AD-G-C-GDRQLT--   54
_XP_030305671.1_    30  IDA--------CKKGYNP--GLLVHPEL----------------------AYLQ-AD-G-C-GDRQLT--   61
_PKU34609.1_        31  IDA--------CKKGYNP--GLLVHPEL----------------------GYLQ-AE-G-C-GDRQLT--   62
_KFP37264.1_        75  IDA--------CKKGYNP--GLLVHPEL----------------------GYLQ-AD-G-C-GDRQLT--  106
_NWX68872.1_        74  IDA--------CKKGYNP--GLLVHPEL----------------------GYLQ-AE-G-C-GDRQLT--  105
_KFP53260.1_        75  IDA--------CKKGYNP--GLLVHPEL----------------------GYLQ-AE-G-C-GDRQLT--  106
_NXP61177.1_        74  IDA--------CKKGYNP--GLLVHPEL----------------------SYLQ-AE-G-C-EDRQLT--  105
_XP_009482766.1_    73  IDA--------CKKGYNP--GLLVHPEL----------------------GYLQ-AE-G-C-GDRQLT--  104
_XP_010215381.1_    73  IDA--------CKKGYNP--ELLVHPEL----------------------GYLQ-AE-G-C-GDRQLT--  104
_NWI90635.1_        75  IDA--------CKKGYNP--ELLVHPEL----------------------SYLQ-AE-G-C-GDRQLT--  106
_NXU77174.1_        74  IDA--------CKKGYNP--GLLVHPEL----------------------AYLQ-AD-G-C-GDRQLT--  105
_NWR18823.1_        75  IDA--------CKKGYNP--GLLVHPEL----------------------SYLQ-AE-G-C-EDRQLT--  106
_POI31681.1_        73  IDA--------CKKGYNP--GLLVHPEL----------------------GYLQ-AE-G-C-GDRQLT--  104
_NXR92200.1_        74  IDA--------CKKGYNP--GLLVHPEL----------------------SYLQ-AE-G-C-EDRQLT--  105
_NXL85091.1_       116  IDA--------CKKGYNP--GLLVHPEL----------------------GYLQ-AE-G-R-GDRQLT--  147
_XP_005044951.1_   115  IDA--------CKKGYNP--GLLVHPEL----------------------SYLQ-AE-G-C-GDRQLT--  146
```

```
_NXC19638.1_        98  IDA--------CKKGYNP--GLLVHPEL----------------------GYLQ-AE-G-C-GDRQLT--  129
_ETE71838.1_         1  ----------------------------------------------------------------RQLT--    4
_KAG8134450.1_     115  IDA--------CKKGYNP--GLLVHPDL----------------------NYIQ-VE-G-G-GERQLT--  146
_XP_026528490.1_   115  IDA--------CKKGYNP--GLLVHPDL----------------------NYIQ-VE-G-G-EERQLT--  146
_XP_044284773.1_   107  IDA--------CKKGYNP--GLLVHPDL----------------------NYIQ-VE-G-G-GERQLS--  138
_XP_026554053.1_   115  IDA--------CKKGYNP--GLLVHPDL----------------------NYIQ-VE-G-G-EERQLT--  146
_XP_032080378.1_   115  IDA--------CKKGYNP--GLLVHPDL----------------------NYIQ-VE-G-G-GERQLT--  146
_XP_034298076.1_   115  IDA--------CKKGYNP--GLLVHPDL----------------------NYIQ-VE-G-G-GERQLT--  146
_ABR18728.1_        70  TEA--------CTKGYNP--GLLVHPDL----------------------AYLQ-AE-G-G-GDRQLT--  101
_XP_004459938.1_   115  TEA--------CVRGYNP--GLLVHPDL----------------------AYLQ-AE-G-G-GDRQLT--  146
_XP_036619706.1_   115  TDA--------CIRGYNP--GLLVHPEL----------------------NYLQ-PE-G-G-GERQLT--  146
_XP_028932520.1_   115  TDA--------CIRGYNP--GLLVHPEL----------------------NYLP-AE-G-G-GERQLT--  146
_MBZ3886481.1_     109  TDA--------CVRGYNP--GLLVHPDL----------------------AYLQ-AE-G-G-GDRQLT--  140
_XP_047574052.1_   107  TEA--------CTKGYNP--GLLVHPDL----------------------AYLQ-AE-G-G-GDRQLT--  138
_XP_032716757.1_   107  TEA--------CTKGYNP--GLLVHPDL----------------------AYLQ-AE-G-G-GDRQLT--  138
_EFB13115.1_       110  TEA--------CTKGYNP--GLLVHPDL----------------------AYLQ-AE-G-G-GDRQLT--  141
_XP_044923034.1_   107  TEA--------CTKGYNP--GLLVHPDL----------------------AYLQ-AE-G-G-GDRQLT--  138
_KFO25880.1_       109  TDA--------CIRGYNP--GLLVHPDL----------------------AYLQ-AEGG-G-GDRQLT--  141
_EPY78492.1_       108  MEA--------CIRGYNP--GLLVHPDL----------------------AYLQ-AE-G-G-GDRQLT--  139
_XP_006233422.1_   115  TEA--------CIRGYNP--GLLVHSDL----------------------AYLQ-AE-G-G-GDRQLT--  146
_XP_038625183.1_   115  TDA--------CIRGYNP--GLLVHPEL----------------------NYLP-AE-G-G-GERQLT--  146
_XP_039769612.1_   115  TDA--------CIRGYNP--GLLVHPEL----------------------NYLP-AE-G-G-GERQLT--  146
_XP_026972816.1_   115  TDA--------CIRGYNP--GLLVHPDL----------------------AYLQ-AE-G-G-GDRQLT--  146
_XP_030700972.1_   115  TDA--------CIRGYNP--GLLVHPDL----------------------AYLQ-AE-G-G-GDRQLT--  146
_XP_019782048.1_   115  TDA--------CIRGYNP--GLLVHPDL----------------------AYLQ-AE-G-G-GDRQLT--  146
_XP_044537564.1_   115  TDA--------CIRGYNP--GLLVHPEL----------------------NYLQ-PE-G-G-GERQLT--  146
_EDL12139.1_       115  TEA--------CIRGYNP--GLLVHSDL----------------------AYLQ-AE-G-G-GDRQLT--  146
_EDL82274.1_       115  TEA--------CIRGYNP--GLLVHSDL----------------------AYLQ-AE-G-G-GDRQLT--  146
_XP_014391954.1_   115  TDA--------CIRGYNP--GLLVHPEL----------------------AYLQ-AE-G-G-GDRQLT--  146
_XP_014391955.1_   115  TDA--------CIRGYNP--GLLVHPEL----------------------AYLQ-AE-G-G-GDRQLT--  146
_XP_033258015.1_   115  TDA--------CIRGYNP--GLLVHPDL----------------------AYLQ-AE-G-G-GDRQLT--  146
_XP_030700975.1_   115  TDA--------CIRGYNP--GLLVHPDL----------------------AYLQ-AE-G-G-GDRQLT--  146
_XP_014315810.1_   115  TDA--------CIRGYNP--GLLVHPEL----------------------AYLQ-AE-G-G-GDRQLT--  146
_XP_014315809.1_   115  TDA--------CIRGYNP--GLLVHPEL----------------------AYLQ-AE-G-G-GDRQLT--  146
_XP_019782050.1_   115  TDA--------CIRGYNP--GLLVHPDL----------------------AYLQ-AE-G-G-GDRQLT--  146
_XP_026972818.1_   115  TDA--------CIRGYNP--GLLVHPDL----------------------AYLQ-AE-G-G-GDRQLT--  146
_XP_015425477.1_   115  TDA--------CIRGYNP--GLLVHPEL----------------------AYLQ-AE-G-G-GDRQLT--  146
_XP_036266227.1_   115  TDA--------CIRGYNP--GLLVHPDL----------------------AYLQ-AE-G-G-GERQLT--  146
_XP_036196846.1_   115  TDA--------CIRGYNP--GLLVHPEL----------------------AYLQ-AE-G-G-GDRQLT--  146
```

```
_XP_027242102.1_  115  TEA--------CIRGYNP--GLLVHSDL----------------------AYLQ-AE-G-G-GDRQLT--  146
_EDL82271.1_      115  TEA--------CIRGYNP--GLLVHSDL----------------------AYLQ-AE-G-G-GDRQLT--  146
_XP_035951696.1_  115  TEA--------CTKGYNP--GLLVHPDL----------------------AYLQ-AE-G-G-GDRQLT--  146
_XP_024309837.1_  115  TEA--------CIRGYNP--GLLVHPDL----------------------AYLQ-AE-G-G-GDRQLG--  146
_CAB94757.1_      115  TEA--------CIRGYNP--GLLVHPDL----------------------AYLQ-AE-G-G-GDRQLG--  146
_AAA36361.1_      115  TEA--------CIRGYNP--GLLVHPDL----------------------AYLQ-AE-G-G-GDRQLG--  146
_NP_001369554.1_  115  TEA--------CIRGYNP--GLLVHPDL----------------------AYLQ-AE-G-G-GDRQLG--  146
_XP_034814839.1_  115  TEA--------CIRGYNP--GLLVHPDL----------------------AYLQ-AE-G-G-GDRQLG--  146
_BAF84139.1_      115  TEA--------CIRGYNP--GLLVHPDL----------------------AYLQ-AE-G-G-GDRQLG--  146
_CAH18336.1_      115  TEA--------CIRGYNP--GLLVHPDL----------------------AYLQ-AE-G-G-GDRQLG--  146
_XP_032033228.1_  115  TEA--------CIRGYNP--GLLVHPDL----------------------AYLQ-AE-G-G-GDRQLG--  146
_XP_054340511.1_  115  TEA--------CIRGYNP--GLLVHPDL----------------------AYLQ-AE-G-G-GDRQLG--  146
_XP_024101589.1_  115  TEA--------CIRGYNP--GLLVHPDL----------------------AYLQ-AE-G-G-GDRQLG--  146
_XP_003257527.1_  115  TEA--------CIRGYNP--GLLVHPDL----------------------AYLQ-AE-G-G-GDRQLG--  146
_XP_009205528.3_  115  TEA--------CIRGYNP--GLLVHPDL----------------------AYLQ-AE-G-G-GDRQLG--  146
_XP_014994319.1_  115  TEA--------CIRGYNP--GLLVHPDL----------------------AYLQ-AE-G-G-GDRQLG--  146
_XP_005555609.1_  115  TEA--------CIRGYNP--GLLVHPDL----------------------AYLQ-AE-G-G-GDRQLG--  146
_XP_024650030.1_  115  TEA--------CIRGYNP--GLLVHPDL----------------------AYLQ-AE-G-G-GDRQLG--  146
_XP_023075867.2_  115  TEA--------CIRGYNP--GLLVHPDL----------------------AYLQ-AE-G-G-GDRQLG--  146
_XP_017732574.1_  115  TEA--------CIRGYNP--GLLVHPDL----------------------AYLQ-AE-G-G-GDRQLG--  146
_XP_010361621.1_  115  TEA--------CIRGYNP--GLLVHPDL----------------------AYLQ-AE-G-G-GDRQLG--  146
_XP_007997596.2_  115  TEA--------CIRGYNP--GLLVHPDL----------------------AYLQ-AE-G-G-GDRQLG--  146
_EHH53872.1_      115  TEA--------CIRGYNP--GLLVHPDL----------------------AYLQ-AE-G-G-GDRQLG--  146
_XP_005555612.2_  115  TEA--------CIRGYNP--GLLVHPDL----------------------AYLQ-AE-G-G-GDRQLG--  146
_XP_033070771.1_  115  TEA--------CIRGYNP--GLLVHPDL----------------------AYLQ-AE-G-G-GDRQLG--  146
_XP_003929561.1_  115  TEA--------CIRGYNP--GLLVHPDL----------------------AYLQ-AE-G-G-GDRQLG--  146
_XP_017364149.1_  115  TEA--------CIRGYNP--GLLVHPDL----------------------AYLQ-AE-G-G-GDRQLG--  146
_XP_012291952.1_  115  TEA--------CIRGYNP--GLLVHPDL----------------------AYLQ-AE-G-G-GDRQLG--  146
_XP_002745591.1_  115  TEA--------CIRGYNP--GLLVHPDL----------------------AYLQ-AE-G-G-GDRQLG--  146
_XP_032117501.1_  115  TEA--------CIRGYNP--GLLVHPDL----------------------VYLQ-AE-G-G-GDRQLG--  146
_XP_011798604.1_  115  TEA--------CIRGYNP--GLLVHPDL----------------------AYLQ-AE-G-G-GDRQLG--  146
_XP_050647861.1_  115  TEA--------CIRGYNP--GLLVHPDL----------------------AYLQ-AE-G-G-GDRQLG--  146
_XP_050647862.1_  115  TEA--------CIRGYNP--GLLVHPDL----------------------AYLQ-AE-G-G-GDRQLG--  146
_XP_016807418.1_  115  TEA--------CIRGYNP--GLLVHPDL----------------------AYLQ-AE-G-G-GDRQLG--  146
_XP_008070272.2_  115  TEA--------CIRGYNP--GLLVHPDL----------------------AYLQ-VE-G-G-GDRQLT--  146
_XP_016079084.1_  115  TEA--------CIRGYNP--GLLVHPDL----------------------DYLQ-AE-G-G-GDRQLT--  146
_XP_054447653.1_  115  TEA--------CIRGYNP--GLLVHPDL----------------------AYLQ-AE-G-A-GDRQLT--  146
_XP_014643400.1_  115  TEA--------CVRGYNP--GLLVHPDL----------------------AYLQ-AE-G-G-GDRQLT--  146
_ABU63584.1_      115  TEA--------CIRGYNP--GLLVHPDL----------------------AYLQ-AE-G-G-GDRQLT--  146
```

```
_XP_025146157.3_  115  TDA--------CIRGYNP--GLLVHPDL----------------------AYLQ-AE-G-G-GDRQLT--  146
_XP_047654925.1_  115  TEA--------CIRGYNP--GLLVHPDL----------------------AYLQ-AE-G-G-GDRQLT--  146
_DAA28856.1_      115  TDA--------CIRGYNP--GLLVHPDL----------------------AYLQ-AE-G-G-GDRQLT--  146
_XP_025839601.1_  115  TEA--------CTKGYNP--GLLVHPDL----------------------AYLQ-AE-G-G-GDRQLT--  146
_XP_005899878.1_  115  TDA--------CIRGYNP--GLLVHPDL----------------------AYLQ-AE-G-G-GDRQLT--  146
_XP_043726831.1_  115  TDA--------CIRGYNP--GLLVHPDL----------------------AYLQ-AE-G-G-GDRQLT--  146
_KAF6372160.1_    115  TEA--------CIRGYNP--GLLVHPDL----------------------AYLQ-AE-G-G-GDRQLT--  146
_KAF4019754.1_    115  TDA--------CIRGYNP--GLLVHPDL----------------------AYLQ-AE-G-G-GDRQLT--  146
_XP_027399763.1_  115  TDA--------CIRGYNP--GLLVHPDL----------------------AYLQ-AE-G-G-GDRQLT--  146
_XP_008579050.1_  115  TEA--------CIRGYNP--GLLVHPDL----------------------AYLQ-AE-G-G-GDRQLT--  146
_NP_001069877.1_  115  TDA--------CIRGYNP--GLLVHPDL----------------------AYLQ-AE-G-G-GDRQLT--  146
_XP_032962399.1_  115  TEA--------CIRGYNP--GLLVHPDL----------------------AYLQ-AE-G-G-GDRQLT--  146
_CAD7684753.1_    115  TEA--------CTKGYNP--GLLVHPDL----------------------AYLQ-AE-G-G-GDRQLT--  146
_XP_041613896.1_  115  TEA--------CTKGYNP--GLLVHPDL----------------------AYLQ-AE-G-G-GDRQLT--  146
_XP_019481431.1_  115  TEA--------CIRGYNP--GLLVHPDL----------------------AYLQ-AE-G-G-GDRQLT--  146
_XP_019817941.1_  115  TDA--------CIRGYNP--GLLVHPDL----------------------AYLQ-AE-G-G-GDRQLT--  146
_XP_016014547.2_  115  TEA--------CIRGYNP--GLLVHPDL----------------------AYLQ-AE-G-G-GDRQLT--  146
_KAI4543101.1_    115  TDA--------CVRGYNP--GLLVHPDL----------------------AYLQ-AE-G-G-GDRQLT--  146
_XP_043294197.1_  115  TDA--------CIRGYNP--GLLVHPDL----------------------AYLQ-AE-G-G-GDRQLT--  146
_XP_010846585.1_  115  TDA--------CIRGYNP--GLLVHPDL----------------------AYLQ-AE-G-G-GDRQLT--  146
_XP_017904753.1_  115  TDA--------CVRGYNP--GLLVHPDL----------------------AYLQ-AE-G-G-GDRQLT--  146
_XP_052497597.1_  115  TDA--------CIRGYNP--GLLVHPDL----------------------AYLQ-AE-G-G-GDRQLT--  146
_KAJ1061279.1_    115  TDA--------CVRGYNP--GLLVHPDL----------------------AYLQ-AE-G-G-GDRQLT--  146
_KAF6372159.1_    115  TEA--------CIRGYNP--GLLVHPDL----------------------AYLQ-AE-G-G-GDRQLT--  146
_XP_032962400.1_  115  TEA--------CIRGYNP--GLLVHPDL----------------------AYLQ-AE-G-G-GDRQLT--  146
_XP_019481432.1_  115  TEA--------CIRGYNP--GLLVHPDL----------------------AYLQ-AE-G-G-GDRQLT--  146
_XP_040093689.1_  115  TDA--------CIRGYNP--GLLVHPDL----------------------AYLQ-AE-G-G-GDRQLT--  146
_XP_012034999.2_  115  TDA--------CVRGYNP--GLLVHPDL----------------------AYLQ-AE-G-G-GDRQLT--  146
_XP_019594487.1_  115  TEA--------CIRGYNP--GLLVHPDL----------------------AYLQ-AE-G-G-GDRQLT--  146
_XP_011359814.1_  115  TEA--------CIRGYNP--GLLVHPDL----------------------AYLQ-AE-G-G-GDRQLT--  146
_XP_006918936.1_  115  TEA--------CIRGYNP--GLLVHPDL----------------------AYLQ-AE-G-G-GDRQLT--  146
_XP_003796373.2_  115  TEA--------CIRGYNP--GLLVHPDL----------------------AYLQ-AE-G-G-GDRQLT--  146
_VFV45675.1_      115  TEA--------CTKGYNP--GLLVHPDL----------------------AYLQ-AE-G-G-GDRQLT--  146
_XP_014938375.1_  115  TEA--------CTKGYNP--GLLVHPDL----------------------AYLQ-AE-G-G-GDRQLT--  146
_XP_038299711.1_  115  TEA--------CTKGYNP--GLLVHPDL----------------------AYLQ-AE-G-G-GDRQLT--  146
_XP_030169211.1_  115  TEA--------CTKGYNP--GLLVHPDL----------------------AYLQ-AE-G-G-GDRQLT--  146
_XP_019594488.1_  115  TEA--------CIRGYNP--GLLVHPDL----------------------AYLQ-AE-G-G-GDRQLT--  146
_XP_046947838.1_  115  TEA--------CTKGYNP--GLLVHPDL----------------------AYLQ-AE-G-G-GDRQLT--  146
_XP_043427583.1_  115  TEA--------CTKGYNP--GLLVHPDL----------------------AYLQ-AE-G-G-GDRQLT--  146
```

```
_XP_026253918.1_  115  TDA--------CVRGYNP--GLLVHPDL----------------------AYLQ-AE-G-G-GDRQLT--  146
_XP_007081014.2_  115  TEA--------CTKGYNP--GLLVHPDL----------------------AYLQ-AE-G-G-GDRQLT--  146
_XP_005325802.1_  115  TDA--------CVRGYNP--GLLVHPDL----------------------AYLQ-AE-G-G-GDRQLT--  146
_XP_025777165.1_  115  TEA--------CTKGYNP--GLLVHPDL----------------------AYLQ-AE-G-G-GDRQLT--  146
_XP_053413718.1_  115  TEA--------CIRGYNP--GLLVHPDL----------------------AYLQ-AE-G-G-GDRQLT--  146
_KAF6131715.1_    115  TEA--------CIRGYNP--GLLVHPDL----------------------AYLQ-AE-G-A-GDRQLT--  146
_NP_001041697.1_  115  TEA--------CIRGYNP--GLLVHPDL----------------------AYLQ-AE-G-G-GDRQLT--  146
_XP_039076751.1_  115  TEA--------CTKGYNP--GLLVHPDL----------------------AYLQ-AE-G-G-GDRQLT--  146
_XP_047711530.1_  115  TEA--------CTKGYNP--GLLVHPDL----------------------AYLQ-AE-G-G-GDRQLT--  146
_XP_045648536.1_  115  TEA--------CTKGYNP--GLLVHPDL----------------------AYLQ-AE-G-G-GDRQLT--  146
_XP_036708419.1_  115  TDA--------CIRGYNP--GLLVHPDL----------------------AYLQ-AE-G-G-GDRQLT--  146
_XP_027791168.1_  115  TDA--------CVRGYNP--GLLVHPDL----------------------AYLQ-AE-G-G-GDRQLT--  146
_KAF7487162.1_    115  TDA--------CVRGYNP--GLLVHPDL----------------------AYLQ-AE-G-G-GDRQLT--  146
_XP_004380306.2_  115  TEA--------CIRGYNP--GLLVHPDL----------------------AYLQ-AE-G-G-GDRQLT--  146
_XP_048960883.1_  115  TEA--------CTKGYNP--GLLVHPDL----------------------AYLQ-AE-G-G-GDRQLT--  146
_XP_007172981.1_  115  TDA--------CIRGYNP--GLLVHPDL----------------------AYLQ-AE-G-G-GDRQLT--  146
_XP_015340088.1_  115  TDA--------CVRGYNP--GLLVHPDL----------------------AYLQ-AE-G-G-GDRQLT--  146
_XP_049488091.1_  115  TEA--------CTKGYNP--GLLVHPDL----------------------AYLQ-AE-G-G-GDRQLT--  146
_XP_046314717.1_  115  TDA--------CVRGYNP--GLLVHPDL----------------------AYLQ-AE-G-G-GDRQLT--  146
_XP_045327678.1_  115  TEA--------CTKGYNP--GLLVHPDL----------------------AYLQ-AE-G-G-GDRQLT--  146
_KAI5775569.1_    115  TEA--------CTKGYNP--GLLVHPDL----------------------AYLQ-AE-G-G-GDRQLT--  146
_XP_044080328.1_  115  TEA--------CTKGYNP--GLLVHPDL----------------------AYLQ-AE-G-G-GDRQLT--  146
_XP_008154865.2_  115  TDA--------CIRGYNP--GLLVHPDL----------------------AYLQ-AE-G-G-GDRQLT--  146
_KAI5937862.1_    115  TEA--------CTRGYNP--GLLVHPDL----------------------AYLQ-AE-G-G-GDRQLT--  146
_XP_031546207.1_  115  MEA--------CIRGYNP--GLLVHPDL----------------------AYLQ-AE-G-G-GDRQLT--  146
_XP_053779051.1_  115  TEA--------CIRGYNP--GLLVHPDL----------------------AYLQ-AE-G-A-GDRQLT--  146
_XP_007463874.1_  115  TDA--------CIRGYNP--GLLVHPDL----------------------AYLQ-AE-G-G-GDRQLT--  146
_XP_022368452.1_  115  TEA--------CTKGYNP--GLLVHPDL----------------------AYLQ-AE-G-G-GDRQLT--  146
_XP_036876060.1_  115  TEA--------CTRGYNP--GLLVHPDL----------------------AYLQ-AE-G-G-GDRQLT--  146
_XP_029798483.1_  115  TEA--------CTKGYNP--GLLVHPDL----------------------AYLQ-AE-G-G-GDRQLT--  146
_XP_036109130.1_  115  TEA--------CIRGYNP--GLLVHPDL----------------------AYLQ-AE-G-G-GDRQLT--  146
_XP_010951892.1_  115  MEA--------CIRGYNP--GLLVHPDL----------------------AYLQ-AE-G-G-GDRQLT--  146
_XP_031324505.1_  115  MEA--------CIRGYNP--GLLVHPDL----------------------AYLQ-AE-G-G-GDRQLT--  146
_XP_023982362.1_  115  TDA--------CIRGYNP--GLLVHPDL----------------------AYLQ-AE-G-G-GDRQLT--  146
_XP_028362571.1_  115  TEA--------CIRGYNP--GLLVHPDL----------------------AYLQ-AE-G-A-GDRQLT--  146
_XP_032316661.1_  115  MEA--------CIRGYNP--GLLVHPDL----------------------AYLQ-AE-G-G-GDRQLT--  146
_XP_051675506.1_  115  TEA--------CIRGYNP--GLLVHPDL----------------------AYLQ-TE-G-G-GDRQLT--  146
_VCW95948.1_      115  TEA--------CTKGYNP--GLLVHPDL----------------------AYLQ-AE-G-G-GDRQLT--  146
_XP_045676060.1_  115  TEA--------CIRGYNP--GLLVHPDL----------------------AYLQ-AE-G-A-GDRQLT--  146
```

```
_XP_040481442.1_  115  TEA--------CTKGYNP--GLLVHPDL----------------------AYLQ-AE-G-G-GDRQLT--  146
_XP_047422689.1_  115  TDA--------CVRGYNP--GLLVHPDL----------------------AYLQ-AE-G-G-GDRQLT--  146
_XP_040333287.1_  115  TEA--------CTKGYNP--GLLVHPDL----------------------AYLQ-AE-G-G-GDRQLT--  146
_XP_032256795.1_  115  TEA--------CTKGYNP--GLLVHPDL----------------------AYLQ-AE-G-G-GDRQLT--  146
_XP_036892131.1_  115  TEA--------CIRGYNP--GLLVHPDL----------------------NYLQ-AE-G-A-GDRQLT--  146
_XP_049741557.1_  115  TEA--------CIRGYNP--GLLVHPDL----------------------AYLQ-AE-G-G-GDRQLT--  146
_XP_003410513.1_  115  TEA--------CIRGYNP--GLLVHPDL----------------------AYLQ-AE-G-G-GDRQLT--  146
_XP_024591836.1_  115  TDA--------CIRGYNP--GLLVHPDL----------------------AYLQ-AE-G-G-GDRQLT--  146
_XP_022438147.1_  115  TDA--------CIRGYNP--GLLVHPDL----------------------AYLQ-AE-G-G-GDRQLT--  146
_KAF0884117.1_    115  TEA--------CTKGYNP--GLLVHPDL----------------------AYLQ-AE-G-G-GDRQLT--  146
_XP_034886066.1_  115  TEA--------CTKGYNP--GLLVHPDL----------------------AYLQ-AE-G-G-GDRQLT--  146
_NP_001003344.1_  115  TEA--------CTKGYNP--GLLVHPDL----------------------AYLQ-AE-G-G-GDRQLT--  146
_XP_020757604.1_  115  TDA--------CIRGYNP--GLLVHPDL----------------------AYLQ-AE-G-G-GDRQLT--  146
_XP_045736627.1_  115  TEA--------CTKGYNP--GLLVHPDL----------------------AYLQ-AE-G-G-GDRQLT--  146
_XP_032488543.1_  115  TDA--------CIRGYNP--GLLVHPDL----------------------AYLQ-AE-G-G-GDRQLT--  146
_XP_029090486.1_  115  TDA--------CIRGYNP--GLLVHPDL----------------------AYLQ-AE-G-G-GDRQLT--  146
_XP_036735088.1_  115  TEA--------CTRGYNP--GLLVHPDL----------------------AYLQ-AE-G-G-GDRQLT--  146
_XP_007951843.2_  115  TEA--------CVRGYNP--GLLVHPDL----------------------AYLQ-AE-G-G-GDRQLT--  146
_XP_045392602.1_  115  TDA--------CIRGYNP--GLLVHPDL----------------------AYLQ-AE-G-G-GDRQLT--  146
_XP_021536093.1_  115  IEA--------CTKGYNP--GLLVHPDL----------------------AYLQ-AE-G-G-GDRQLT--  146
_XP_027453602.1_  115  TEA--------CTKGYNP--GLLVHPDL----------------------AYLQ-AE-G-G-GDRQLT--  146
_XP_023108674.2_  115  TEA--------CTKGYNP--GLLVHPDL----------------------AYLQ-AE-G-G-GDRQLT--  146
_XP_045853066.1_  115  TEA--------CTKGYNP--GLLVHPDL----------------------AYLQ-AE-G-G-GDRQLT--  146
_XP_036266160.1_  115  TDA--------CIRGYNP--GLLVHPDL----------------------AYLQ-AE-G-G-GERQLT--  146
_XP_037012773.2_  115  TEA--------CIRGYNP--GLLVHPDL----------------------AYLQ-AE-G-A-GDRQLT--  146
_XP_004401911.1_  115  TEA--------CTKGYNP--GLLVHPDL----------------------AYLQ-AE-G-G-GDRQLT--  146
_KAI5226086.1_    115  TEA--------CTRGYNP--GLLVHPDL----------------------AYLQ-AE-G-G-GDRQLT--  146
_XP_032716751.1_  115  TEA--------CTKGYNP--GLLVHPDL----------------------AYLQ-AE-G-G-GDRQLT--  146
_XP_032188916.1_  115  TEA--------CTKGYNP--GLLVHPDL----------------------AYLQ-AE-G-G-GDRQLT--  146
_XP_027453603.1_  115  TEA--------CTKGYNP--GLLVHPDL----------------------AYLQ-AE-G-G-GDRQLT--  146
_XP_049564805.1_  115  TDA--------CIRGYNP--GLLVHPDL----------------------AYLQ-AE-G-G-GDRQLT--  146
_XP_004401912.1_  115  TEA--------CTKGYNP--GLLVHPDL----------------------AYLQ-AE-G-G-GDRQLT--  146
_XP_047574051.1_  115  TEA--------CTKGYNP--GLLVHPDL----------------------AYLQ-AE-G-G-GDRQLT--  146
_XP_032488539.1_  115  TDA--------CIRGYNP--GLLVHPDL----------------------AYLQ-AE-G-G-GDRQLT--  146
_XP_012510833.1_  115  TDA--------CIRGYNP--GLLVHPDL----------------------AYLQ-AE-G-G-GDRQLT--  146
_XP_006893175.1_  115  KDA--------CIRGYNP--GLLVHPDL----------------------AYLQ-AE-G-G-GDRQLT--  146
_XP_037686273.1_  115  TEA--------CIRGYNP--GLLVHPDL----------------------AYLQ-AE-G-G-GDRQLT--  146
_XP_046512032.1_  115  TEA--------CVRGYNP--EILVHPDL----------------------RYLQ-AE-G-G-GDRQLT--  146
_XP_005608646.3_  115  TEA--------CVRGYNP--EILVHPDL----------------------RYLQ-AE-G-G-GDRQLT--  146
```

```
_XP_036196809.1_  115  TDA--------CIRGYNP--GLLVHPEL----------------------AYLQ-AE-G-G-GDRQLT--  146
_XP_034527338.1_  115  TEA--------CTKGYNP--GLLVHPDL----------------------AYLQ-AE-G-G-GDRQLT--  146
_XP_048960884.1_  115  TEA--------CTKGYNP--GLLVHPDL----------------------AYLQ-AE-G-G-GDRQLT--  146
_XP_036046011.1_  115  TEA--------CIRGYNP--GLLVHSEL----------------------AYLQ-AE-G-G-GDRQLT--  146
_XP_006872788.1_  115  IDA--------CIRGYNP--GLLVHPEL----------------------VYLQ-AE-G-G-GDRQLT--  146
_XP_044923033.1_  115  TEA--------CTKGYNP--GLLVHPDL----------------------AYLQ-AE-G-G-GDRQLT--  146
_KAB0398538.1_    115  TDA--------CIRGYNP--GLLVHPDL----------------------AYLQ-AE-G-G-GDRQLT--  146
_TEA30114.1_      115  TDA--------CIRGYNP--GLLVHPDL----------------------AYLQ-AE-G-G-GDRQLT--  146
_XP_004703501.1_  115  TEA--------CIRGYNP--GLLVHPDL----------------------AYLQ-AE-G-G-GDRQLT--  146
_XP_045371697.1_  115  MEA--------CIRGYNP--GLLVHPDL----------------------AYLQ-AE-G-G-GDRQLT--  146
_XP_045371695.1_  115  MEA--------CIRGYNP--GLLVHPDL----------------------AYLQ-AE-G-G-GDRQLT--  146
_XP_045371698.1_  115  MEA--------CIRGYNP--GLLVHPDL----------------------AYLQ-AE-G-G-GDRQLT--  146
_XP_051021942.1_  115  TEA--------CIRGYNP--GLLVHSDL----------------------AYLQ-AE-G-G-GDRQLT--  146
_XP_028616215.1_  115  TEA--------CIRGYNP--GLLVHSDL----------------------AYLQ-AE-G-G-GDRQLT--  146
_XP_040822498.1_  115  TEA--------CIRGYNP--GLLVHPDL----------------------AYLQ-TE-G-G-GDRQLT--  146
_XP_051041400.1_  115  TEA--------CIRGYNP--GLLVHSDL----------------------AYLQ-AE-G-G-GDRQLT--  146
_XP_034356238.1_  115  TEA--------CIRGYNP--GLLVHSDL----------------------AYLQ-AE-G-G-GDRQLT--  146
_XP_028713251.1_  115  TEA--------CIRGYNP--GLLVHSEL----------------------AYLQ-AE-G-G-GDRQLT--  146
_ERE89660.1_      115  TEA--------CIRGYNP--GLLVHSDL----------------------AYLQ-AE-G-G-GDRQLT--  146
_XP_052035128.1_  115  TEA--------CIRGYNP--GLLVHSDL----------------------AYLQ-AE-G-G-GDRQLT--  146
_XP_006970398.1_  115  TEA--------CIRGYNP--GLLVHSEL----------------------AYLQ-AE-G-G-GDRQLT--  146
_XP_042557701.1_  115  TDA--------CIRGYNP--GLLVHPEL----------------------AYLQ-AE-G-G-GDRQLT--  146
_XP_031231473.1_  115  TEA--------CIRGYNP--GLLVHSDL----------------------AYLQ-AE-G-L-GERQLT--  146
_XP_012879285.1_  115  TDA--------CIRGYNP--GLLVHPEL----------------------AYLQ-AE-G-G-GDRQLT--  146
_XP_003515839.4_  115  TEA--------CIRGYNP--GLLVHSDL----------------------AYLQ-AE-G-G-GDRQLT--  146
_XP_012626422.1_  115  IDA--------CIRGYNP--GLLVHPDL----------------------AYLQ-AE-G-G-GDRQLT--  146
_XP_048189722.1_  115  TDA--------CIRGYNP--GLLVHPDL----------------------AYLQ-AE-G-G-GDRQLT--  146
_XP_052586690.1_  115  TEA--------CIRGYNP--GLLVHSEL----------------------AYLQ-AE-G-G-GDRQLT--  146
_NP_032715.2_     115  TEA--------CIRGYNP--GLLVHSDL----------------------AYLQ-AE-G-G-GDRQLT--  146
_AAA40415.1_      115  TEA--------CIRGYNP--GLLVHSDL----------------------AYLQ-AE-G-G-GDRQLT--  146
_XP_049488087.1_  115  TEA--------CTKGYNP--GLLVHPDL----------------------AYLQ-AE-G-G-GDRQLT--  146
_XP_044998939.1_  115  TEA--------CIRGYNP--GLLVHSDL----------------------AYLQ-AE-G-G-GDRQLT--  146
_XP_049999310.1_  115  TEA--------CIRGYNP--GLLVHSDL----------------------AYLQ-AE-G-G-GDRQLT--  146
_XP_040607681.1_  115  TEA--------CIRGYNP--GLLVHSDL----------------------AYLQ-AE-G-G-GDRQLT--  146
_XP_045676057.1_  115  TEA--------CIRGYNP--GLLVHPDL----------------------AYLQ-AE-G-A-GDRQLT--  146
_XP_032753167.1_  115  TEA--------CIRGYNP--GLLVHSDL----------------------AYLQ-AE-G-G-GDRQLT--  146
_XP_028347494.1_  115  TDA--------CIRGYNP--GLLVHPDL----------------------AYLQ-AE-G-G-GDRQLT--  146
_XP_031546209.1_  115  MEA--------CIRGYNP--GLLVHPDL----------------------AYLQ-AE-G-G-GDRQLT--  146
_XP_010951893.1_  115  MEA--------CIRGYNP--GLLVHPDL----------------------AYLQ-AE-G-G-GDRQLT--  146
```

```
_XP_005357381.1_  115  TEA--------CIRGYNP--GLLVHSDL----------------------AYLQ-AE-G-G-GDRQLT--  146
_XP_031546210.1_  115  MEA--------CIRGYNP--GLLVHPDL----------------------AYLQ-AE-G-G-GDRQLT--  146
_XP_031324516.1_  115  MEA--------CIRGYNP--GLLVHPDL----------------------AYLQ-AE-G-G-GDRQLT--  146
_XP_031324521.1_  115  MEA--------CIRGYNP--GLLVHPDL----------------------AYLQ-AE-G-G-GDRQLT--  146
_XP_041511026.1_  115  TEA--------CIRGYNP--GLLVHSDL----------------------AYLQ-AE-G-G-GDRQLT--  146
_XP_032316666.1_  115  MEA--------CIRGYNP--GLLVHPDL----------------------AYLQ-AE-G-G-GDRQLT--  146
_XP_032316670.1_  115  MEA--------CIRGYNP--GLLVHPDL----------------------AYLQ-AE-G-G-GDRQLT--  146
_XP_021794361.1_  115  TEA--------CIRGYNP--GLLVHPDL----------------------AYLQ-AE-G-G-GDRQLG--  146
_XP_021509291.1_  115  TEA--------CIRGYNP--GLLVHSDL----------------------AYLQ-AE-G-G-GDRQLT--  146
_XP_021536094.1_  115  IEA--------CTKGYNP--GLLVHPDL----------------------AYLQ-AE-G-G-GDRQLT--  146
_XP_013360849.1_  115  TDA--------CIRGYNP--GLLVHPDL----------------------AYLQ-AE-GSG-GDRQLT--  147
_XP_038959153.1_  115  TEA--------CIRGYNP--GLLVHSDL----------------------AYLQ-AE-G-G-GDRQLT--  146
_XP_048312756.1_  115  TEA--------CIRGYNP--GLLVHSDL----------------------AYLQ-AE-G-G-GDRQLT--  146
_XP_044622324.1_  115  TEA--------CVRGYNP--EILVHPDL----------------------RYLQ-AE-G-G-GDRQLT--  146
_XP_044622325.1_  115  TEA--------CVRGYNP--EILVHPDL----------------------RYLQ-AE-G-G-GDRQLT--  146
_XP_008527175.1_  115  TEA--------CVRGYNP--EILVHPDL----------------------RYLQ-AE-G-G-GDRQLT--  146
_XP_003468075.1_  115  TDA--------CVRGYNP--GLLVHPDL----------------------AYLQ-AE-GGG-GDRQLT--  147
_XP_027628732.1_  115  TEA--------CMRGYNP--GLLVHPDL----------------------AYLQ-AE-G-G-GDRQLT--  146
_XP_046512030.1_  115  TEA--------CVRGYNP--EILVHPDL----------------------RYLQ-AE-G-G-GDRQLT--  146
_VFV45676.1_      115  TEA--------CTKGYNP--GLLVHPDL----------------------AYLQ-AE-G-G-GDRQLT--  146
_XP_046512031.1_  115  TEA--------CVRGYNP--EILVHPDL----------------------RYLQ-AE-G-G-GDRQLT--  146
_XP_020843959.1_  115  TDA--------CIRGYNP--GLLVHPEL----------------------NYLQ-PE-G-G-GERQLT--  146
_XP_010639891.1_  115  TDA--------CIRGYNP--GLLVHPDL----------------------AYLQ-AE-GGG-GDRQLT--  147
_XP_025146161.3_  115  TDA--------CIRGYNP--GLLVHPDL----------------------AYLQ-AE-G-G-GDRQLT--  146
_XP_029416093.1_  115  TEA--------CIRGYNP--GLLVHSDL----------------------AYLQ-AE-G-G-GDRQLT--  146
_XP_007495939.1_  115  TDA--------CIRGYNP--GLLVHPEL----------------------NYLQ-PE-G-G-GERQLT--  146
_XP_007495937.1_  115  TDA--------CIRGYNP--GLLVHPEL----------------------NYLQ-PE-G-G-GERQLT--  146
_XP_049564806.1_  115  TDA--------CIRGYNP--GLLVHPDL----------------------AYLQ-AE-G-G-GDRQLT--  146
_XP_027709162.1_  115  TDA--------CIRGYNP--GLLVHPEL----------------------NYLQ-PE-G-G-GERQLT--  146
_XP_037365527.1_  115  TDA--------CVRGYNA--GVLVHPDL----------------------AYLQ-AE-G-G-GDRQLT--  146
_XP_004594353.1_  113  TEA--------CIRGYNP--GLLVHPDL----------------------AYLQ-TE-G-G-GDRQLT--  144
_XP_036619704.1_  115  TDA--------CIRGYNP--GLLVHPEL----------------------NYLQ-PE-G-G-GERQLT--  146
_XP_043828877.1_  115  TDA--------CIRGYNP--GLLVHQEL----------------------NYLQ-PE-S-G-GDRQLT--  146
_XP_012407475.1_  115  TDA--------CIRGYNP--GLLVHPEL----------------------NYLQ-PE-G-G-GERQLT--  146
_XP_051822145.1_  115  TDA--------CIRGYNP--GLLVHPEL----------------------NYLQ-PE-G-G-GERQLT--  146
_XP_031798171.1_  115  TDA--------CIRGYNP--GLLVHPEL----------------------NYLQ-PE-G-G-GERQLT--  146
_XP_004686510.2_  115  TDA--------CVRGYNP--GLLVHPDL----------------------AYLQ-AE-G-G-GDRQLT--  146
_XP_021509292.1_  115  TEA--------CIRGYNP--GLLVHSDL----------------------AYLQ-AE-G-G-GDRQLT--  146
_XP_052035129.1_  115  TEA--------CIRGYNP--GLLVHSDL----------------------AYLQ-AE-G-G-GDRQLT--  146
```

```
_XP_019065790.1_  115  TDA--------CIRGYNP--GLLVHPDL----------------------AYLQ-AE-GGG-GDRQLT--  147
_XP_031231475.1_  115  TEA--------CIRGYNP--GLLVHSDL----------------------AYLQ-AE-G-L-GERQLT--  146
_XP_034356239.1_  115  TEA--------CIRGYNP--GLLVHSDL----------------------AYLQ-AE-G-G-GDRQLT--  146
_XP_027251570.1_  115  TEA--------CIRGYNP--GLLVHSDL----------------------AYLQ-AE-G-G-GDRQLT--  146
_XP_029330830.1_  115  TEA--------CIRGYNP--GLLVHSDL----------------------AYLQ-AE-G-G-GDRQLT--  146
_XP_045426343.1_  115  TDA--------CIRGYNP--GLLVHPDL----------------------AYLQ-AE-G-G-GERQLT--  146
_NP_001397371.1_  115  TEA--------CIRGYNP--GLLVHSDL----------------------AYLQ-AE-G-G-GDRQLT--  146
_XP_040607683.1_  115  TEA--------CIRGYNP--GLLVHSDL----------------------AYLQ-AE-G-G-GDRQLT--  146
_XP_026639883.1_  115  TEA--------CIRGYNP--GLLVHSDL----------------------AYLQ-AE-G-G-GDRQLT--  146
_XP_029393278.1_  115  TEA--------CIRGYNP--GLLVHSDL----------------------AYLQ-AE-G-G-GDRQLT--  146
_XP_049642488.1_  115  TEA--------CIRGYNP--GLLVHPDL----------------------AYLQ-AE-G-G-GDRRLT--  146
_XP_004617756.1_  115  SEA--------CIRGYNP--GLLVHPDL----------------------AYLQ-AE-G-A-GDRRLT--  146
_XP_021536097.1_  115  IEA--------CTKGYNP--GLLVHPDL----------------------AYLQ-AE-G-G-GDRQLT--  146
_NWW35472.1_      115  IDA--------CKKGYNP--GLLVHPEL----------------------GYLQ-AE-G-C-GDRQLT--  146
_KAG8543434.1_    115  IDA--------FKKGYNA--GLLVHPEF----------------------TYSYS-------ADRPLT--  143
_KAG8543366.1_    115  IDA--------FKKGYNA--GLLVHPEF----------------------TYSYS-------ADRPLT--  143
_XP_044130636.1_  115  LDA--------FKKGYNA--GLLVHPEL----------------------NYPCL-------PDRPLT--  143
_XP_040274154.1_  115  LDA--------FKKGYNA--GLLVHPEL----------------------NYPCL-------PDRPLT--  143
_XP_053317513.1_  115  LDA--------FKKGYNG--EVLVHPEL----------------------NYVYS-------GDRPLT--  143
_ALA55868.1_      115  IDA--------FKKGYNA--SLLVHPEL----------------------NYQYL-------GDRTLT--  143
_XP_040185842.1_  115  IDA--------FKKGYNA--GLLVHPEL----------------------NYQYL-------GDRTLT--  143
_XP_040185853.1_  115  IDA--------FKKGYNA--GLLVHPEL----------------------NYQYL-------GDRTLT--  143
_XP_018414489.1_  115  TDA--------FKKGYNA--GLLVHPEL----------------------NYQYS-------GDRLLT--  143
_XP_053559470.1_  115  TDA--------FKKGYNA--GLLVHQEL----------------------SYPYS-------ADRPLS--  143
_XP_053559471.1_  115  TDA--------FKKGYNA--GLLVHQEL----------------------SYPYS-------ADRPLS--  143
_KAG8454149.1_    115  TDS--------FKKGYNA--ALLVHPEL----------------------NYTYS-------GDRPLT--  143
_NP_001087808.1_  115  TDA--------FKKGHNA--ALLVHPEL----------------------NYTNS-------EDRPLN--  143
_XP_018098880.1_  115  TDA--------FKKGHNA--ALLIHPEL----------------------NYTYS-------EDRQLN--  143
_XP_002934805.3_  115  TDA--------FKKGHNA--ALLVHPEL----------------------NYTYS-------EDRQLN--  143
_OCT97186.1_      115  TDA--------FKKGHNA--ALLIHPEL----------------------NYTYS-------EDRQLN--  143
_CAI5653158.1_    116  IEA--------FKLGYNC--GVSIHPDI----------------------DSVQ-GE-V-R-VPREVT--  147
_XP_032876238.1_  116  SQA--------LLQGYNA--GVLIHPDL----------------------DVLH-AD-T-C-PPAELT--  147
_XP_038648821.1_  116  THA--------YVMGYNN--GVLIHQEL----------------------NHQH-GE-G-Y-SQRELT--  147
_XP_038648817.1_  116  THA--------YVMGYNN--GVLIHQEL----------------------NHQH-GE-G-Y-SQRELT--  147
_GCB77082.1_      116  TQA--------YVMGYNN--GVLIHQEL----------------------NHQH-GE-G-Y-SQRELT--  147
_XP_051900898.1_  116  SQA--------YLMGYDN--GVVIHPEL----------------------NHRH-SE-G-Y-GQMELT--  147
_XP_048452319.1_  116  TQA--------YVMGYNN--GVVIHPEL----------------------NHRH-SE-G-Y-GQRELT--  147
_XP_048382772.1_  116  TQA--------YVMGYNN--GVVIHPEL----------------------NHRH-NE-G-Y-GQRELT--  147
_XP_043530455.1_  116  TQA--------YVMGYNN--GVVIHPEL----------------------NHRH-SE-G-Y-GQRELT--  147
```

```
_GCC38268.1_       116  TQA--------YVMGYNN--GVVIHPEL----------------------NHRH-SE-G-Y-GQRELT--  147
_XP_041045532.1_   116  TQA--------YVMGYNN--GVVIHPEL----------------------NHRH-SE-S-Y-GQRELT--  147
_XP_007890062.2_   116  VHA--------CIMGYNN--GIVIHPEI----------------------NHRH-IE-T-C-SMRELT--  147
_XP_007897340.2_   115  LGP--------TESDPAA-------------------------------------TK-T-P-VRKNSQ--  134
_XP_007897342.2_   108  LGP--------TESDPAA-------------------------------------TK-T-P-VRKNSQ--  127
_XP_032868306.1_   116  FNE--------LYQK-----------------------------------NPLSSQD-F-L-SKSLLS--  137
_XP_051897529.1_   116  FNE--------MYQK-----------------------------------NPLNPKN-F-I-SKSLLS--  137
_XP_048419270.1_   116  FNE--------TYQR-----------------------------------NPLDRKN-F-L-NKAILS--  137
_XP_048472262.1_   116  FNE--------TYQR-----------------------------------NPLDRKN-F-L-NKAILS--  137
_GCC17239.1_       116  FNE--------TYQR-----------------------------------NPLDRKN-F-L-NKAILS--  137
_XP_043534576.1_   116  FNE--------TYQR-----------------------------------NPLDRKN-F-L-NKAILS--  137
_XP_041032206.1_   116  FNE--------MYQR-----------------------------------NPLDRKN-F-L-SKGIMS--  137
_XP_038639209.1_   108  FNE--------MYQR-----------------------------------NPMDRKS-F-L-SKSILP--  129
_XP_032825135.1_   115  SDA--------QVSGLASIGNIVLENSF----------------------NNVN---------ARALT--  143
_AUA18026.1_       115  SDA--------QISGLANIGNIVLESGF----------------------HNVH---------ARDLT--  143
_XP_043559628.1_    76  IKS--------WTSDAQN--GNASLLNG----------------------NNEN-EM-E-L-QQKQLK--  107
_GCC23558.1_        74  IKS--------WTSDAQN--GNASLLNG----------------------NNEK-EM-E-L-QQKQLK--  105
_XP_048459926.1_   115  IKS--------WTSDAQN--GNASLPNG----------------------NSEN-EM-E-L-QQKQLK--  146
_XP_048399043.1_   115  IKS--------WTSDAQN--GNASLPNG----------------------NSEN-EM-E-L-QQKQLK--  146
_XP_043559626.1_   115  IKS--------WTSDAQN--GNASLLNG----------------------NNEN-EM-E-L-QQKQLK--  146
_XP_043559625.1_   115  IKS--------WTSDAQN--GNASLLNG----------------------NNEN-EM-E-L-QQKQLK--  146
_XP_043559624.1_   115  IKS--------WTSDAQN--GNASLLNG----------------------NNEN-EM-E-L-QQKQLK--  146
_XP_043559627.1_   115  IKS--------WTSDAQN--GNASLLNG----------------------NNEN-EM-E-L-QQKQLK--  146
_XP_041050315.1_   115  IRS--------WTSDAQN--GSVPLPDG----------------------NIEN-EM-E-L-QRKQLQTG  148
_GCB72915.1_       115  IKS--------WTSDTQN--GNISLPDG----------------------KNEN-EM-E-L-QHKQLQ--  146
_XP_051870516.1_   115  IKS--------WMSDINN--GNISLSDED--------------------------GV-E-L-QEEQLQ--  143
_XP_032885971.1_   115  VKS--------WMSDLND--ENPSLSDED--------------------------GV-E-L-QQNQLQ--  143
_XP_007904454.1_   115  VRA--------WSLESQD--GQESPQDR----------------------NSGNPTA-S-S-SQKQLR--  147
_CAD5120983.1_     115  EDD--------KIFKSEI--------------------------------------------LRASDL--  130
_KAI6649899.1_     119  AQC--------ELLRQQG--ISQYIG-----------------------------MM-T-G-WTKHHT--  144
_KAI6652168.1_     115  ETR--------RLVEKIG--VDALRSALASSN-------------LNGTGSESA-LE-E-L-IKKHLT--  155
_KAI6653779.1_     116  AVE--------WFIKAHG--IHAYIQSSDAHSKRNP-----TPGDDDHDESQKSYGA-V-L-FKRLRP--  165
_NP_001267795.1_   120  VLQ--------SEYLKKY--NSINPPIDVETF-------------VKRLEDRED-KK-L-I-NRLTGV--  160
_XP_047123977.1_   120  VLQ--------SEYLKKY--NSINPPIDVETF-------------VKRLEDRED-KK-L-I-NRLTGV--  160
_XP_047123978.1_     1  ------------------------------------------------------------LINRLTGV--    8
_KAG8178714.1_     118  IRN--------LEDTE------------------------------------------------------  125
_GFT03926.1_       114  ISE--------YGLKSLT----------------------------------------------------  123
_KAI6646872.1_     120  IQD--------IILSRRG--ICDYSRLMKL------------------HNSTCTTMQ-L-N-LQWLQT--  156
_XP_033625504.1_   115  DRL--------KQLVP------------------------------------------------------  122
```

```
_XP_038072325.1_   115  DIL--------KEHLP------------------------------------------------------  122
_XP_038072324.1_   115  DIL--------KEHLP------------------------------------------------------  122
Consensus aa:           .p.......................s........................s..p.........p...s..
Consensus ss:           hh                                                       - -

Conservation:                                                                            5  75
_KAH7637713.1_     144  --------------------------------------QQLRRQLQL--------EAEKEAKDLNLNSVR  167
_XP_023220155.1_   122  ---------------------------------------VEQIREIS--------KEESKTMVSQLHVVR  144
_XP_009016345.1_   127  --------------------------------------NFEEEDIKK--------KAEEQSKSLQMSVVR  150
_ELU08708.1_       133  --------------------------------------EQQRSKAMK--------QAEDQARSIQLNTVK  156
_UCK81536.1_       146  --------------------------------------EAERREAQA--------EAEEQAKTMQLNTVR  169
_CAH1795432.1_     139  --------------------------------------SDEEKMAKK--------EAEKQAKDMQLNVVR  162
_CAE1313443.1_     139  --------------------------------------DTEKKTAKE--------QADTQAKSMHLNVVR  162
_XP_048764075.1_   137  --------------------------------------DDEKSQAST--------QANQLAKDMQLNVVK  160
_XP_009060044.1_   136  --------------------------------------------------------TSEQAKSTQLNNVK  149
_XP_050391129.1_   139  --------------------------------------CEEEEHAEK--------MARDQAKSTQLNVVR  162
_RUS85575.1_       137  ---------------------------------------------------------EDQSKNMQLNVVC  149
_GFS09172.1_       139  ------------------------------------CTAEDRKVARK--------QAEDQSKNMQLSVVC  164
_XP_005091024.1_   137  --------------------------------------DEDRRLARS--------QADEQSKNMQLNVVR  160
_XP_005091023.1_   137  --------------------------------------DEDRRLARS--------QADEQSKNMQLNVVR  160
_KAH9508414.1_     136  --------------------------------------DDDERTAKQ--------QAEEQSKNMQLNVVK  159
_XP_013071937.1_   137  --------------------------------------EEDSRTAKL--------QADEQSKTMQLNVVK  160
_KAI8766750.1_     137  --------------------------------------EEDSRTAKL--------QADEQSKTMQLNVVK  160
_KAI8794647.1_     137  --------------------------------------EEDSRTAKL--------QADEQSKTMQLNVVK  160
_NP_001298220.1_   137  --------------------------------------EEDSRTAKL--------QADEQSKTMQLNVVK  160
_CAG5129174.1_     137  --------------------------------------DEDKRTARQ--------QAEEQSKTMQLNVVR  160
_KAG5707696.1_       4  --------------------------------------AEETNSAKK--------QAQEQARSMSLNVVR   27
_XP_025114345.1_   137  --------------------------------------AEENMNAKK--------QAQEQARSMSLNVVR  160
_QNL15288.1_       136  --------------------------------------AEENLDAKK--------EAQELSRNMSLNVVR  159
_XP_041362807.1_   136  --------------------------------------DQEKENARK--------QAREQAKSMSLNVVR  159
_XP_046550037.1_   137  --------------------------------------KDDKDKARK--------QAQEQAKNMSLNVVR  160
_XP_048248211.1_   137  --------------------------------------KEDREKARK--------QAQDQAKSMSLNVVR  160
_XP_046550036.1_   137  --------------------------------------KDDKDKARK--------QAQEQAKNMSLNVVR  160
_CAC5363017.1_     137  --------------------------------------EDELAQAKQ--------QAEKQAKDMQLNVVK  160
_VDI06196.1_       137  --------------------------------------EDELAQAKQ--------QAEKQAKDMQLNVVK  160
_AHI17304.1_       137  --------------------------------------EDELAQAKQ--------QAEKQAKDMQLNVVK  160
_XP_052068168.1_   137  --------------------------------------EDELAQAKQ--------QAEKQAKDMQLNVVK  160
_XP_052068167.1_   137  --------------------------------------EDELAQAKQ--------QAEKQAKDMQLNVVK  160
```

```
_UZH84476.1_       137  --------------------------------------DEEVKHARS--------QAEKQAKEMQLNVVR  160
_XP_033756865.1_   137  --------------------------------------VEDVNQAKV--------QSEKLAKEMQLNVVK  160
_AKC01669.1_       137  --------------------------------------MEDIKQAKT--------QSEKLAKEMQLNVVK  160
_KAJ8319099.1_     137  --------------------------------------ESERETAKQ--------QAEKLAKDMQLNVVK  160
_XP_022318640.1_   137  --------------------------------------DDEKSQACS--------QAKQLAKDMQLNVVK  160
_XP_011423502.2_   137  --------------------------------------DDEKSQASK--------QSKQLAKDMQLNVVR  160
_XP_052690447.1_   137  --------------------------------------DDEKSQASK--------QSKQLAKDMQLNVVR  160
_AEY99616.1_       136  --------------------------------------DDEKRQAKE--------QAVQAAKDMQLNVVK  159
_XP_052263328.1_   136  --------------------------------------AEECERAKK--------EAVASAKNMQLNMVR  159
_XP_052263327.1_   136  --------------------------------------AEECERAKK--------EAVASAKNMQLNMVR  159
_XP_052761314.1_   137  --------------------------------------GEERDQARA--------EAQNSAKTMQLNVVR  160
_XP_053392566.1_   137  --------------------------------------DEDKKRAKL--------EAAAQAKGMHLNVVR  160
_KAI0238513.1_     143  --------------------------------------PEMRQRVRK--------QAEQQSKDIQLNVIR  166
_XP_029638652.1_   138  --------------------------------------ENEINDAEE--------SARKQGKNMSLNVVR  161
_AUR53646.1_       138  --------------------------------------ENEINDAEE--------SARKQGKNMSLNVVR  161
_XP_036360935.1_   138  --------------------------------------ENEINDAEE--------SARKQGKNMSLNVVR  161
_XP_036360934.1_   138  --------------------------------------ENEINDAEE--------SARKQGKNMSLNVVR  161
_XP_029638651.1_   138  --------------------------------------ENEINDAEE--------SARKQGKNMSLNVVR  161
_XP_014776042.1_   137  --------------------------------------EIEIHAAEE--------SARNQGKNMSLNVVR  160
_XP_014776041.1_   137  --------------------------------------EIEIHAAEE--------SARNQGKNMSLNVVR  160
_XP_052825578.1_   137  --------------------------------------EIEIHAAEE--------SARNQGKNMSLNVVR  160
_XP_052825580.1_    48  --------------------------------------EIEIHAAEE--------SARNQGKNMSLNVVR   71
_AEP33644.1_       132  --------------------------------------QIAEEEAEK--------RAKSMAKDMDLSRVK  155
_PIK44524.1_       132  --------------------------------------QMSVAEAEE--------RAKLMAKDMDLSKVK  155
_KAJ8022831.1_     132  --------------------------------------QITPEDAAA--------RAKAMAKDMDLSKVR  155
_XP_030838637.1_    93  --------------------------------------MDTEDQLPR--------RQRKWPKXVDLSVVR  116
_XP_038060093.1_   136  --------------------------------------MDTEDQLAK--------KAKEVAKEMDLSVVR  159
_XP_022087493.1_   139  --------------------------------------LDSEEDLTK--------KAKEMSKEMDLSVVR  162
_XP_033643738.1_   138  --------------------------------------LETEPALAK--------KAKEMAKEMDLSKVC  161
_XP_033100082.1_   131  --------------------------------------QLTDPIAEQ--------RAKELAKDMDLASVR  154
_CCC55418.2_       115  --------------------------------------EPSDQEIEK--------KAKEMAKDMDLSVVR  138
_XP_054773081.1_   136  --------------------------------------EPSDAEIEK--------KAKEMAKDMDLSVVR  159
_XP_030834883.1_   141  --------------------------------------EPSDAEIEK--------KAKEMAKDMDLSVVR  164
_NP_999819.1_      137  --------------------------------------EPSDAEIEK--------KAKEMAKDMDLSVVR  160
_XP_030834884.1_   141  --------------------------------------EPSDAEIEK--------KAKEMAKDMDLSVVR  164
_XP_041485048.1_   136  --------------------------------------EPSDSEIEK--------KAKEMAKDMDLSVVR  159
_QIA61835.1_       137  --------------------------------------HDELDQIKS--------QATQYAKTMNLSVVR  160
_NP_001266238.1_   145  --------------------------------------SEEQDEIHQ--------QAQTLAKSMNLSVVR  168
_QHX41456.1_       137  --------------------------------------DVEKEAARR--------EADKVAKGMNLSVVR  160
```

```
_XP_022789210.1_   163  --------------------------------------EEESQKVRT--------MVEEQKQSMNLNAVR  186
_XP_046859971.1_   150  --------------------------------------NEEIAKIDR--------IVEQEAKNMNLSSVR  173
_XP_028414684.1_   155  --------------------------------------NEEMKSIEV--------IAKQEADKMNLSTVR  178
_XP_028414852.1_   155  --------------------------------------NEEMKSIEV--------IAKQEADKMNLSTVR  178
_ADQ57372.1_       166  --------------------------------------NEEVAKSRE--------YAEQQAAAMDLSAVR  189
_XP_048580668.1_   166  --------------------------------------NQEAAKSRE--------YAKQQAAAMDLSAVR  189
_EDO32923.1_       166  --------------------------------------NQEAAKSRE--------YAKQQAAAMDLSAVR  189
_ABU48530.1_       166  --------------------------------------NQEAAKSRE--------YAKQQAAAMDLSAVR  189
_ALG40993.1_       171  --------------------------------------DEEAKKIRK--------MVDEQAKSMNLSAVR  194
_ALG40994.1_       169  --------------------------------------DEEAKKIRK--------MVDDQAKQMNLSCVR  192
_XP_031565156.1_   169  --------------------------------------DEEAKKIRK--------MVDDQAKQMNLSCVR  192
_ALG40992.1_       171  --------------------------------------DDEAKKIRK--------MVDDQAKNMNLSAVR  194
_KXJ21435.1_       168  --------------------------------------GEETKKILK--------LVQEQSKTMNLSAVR  191
_XP_020916759.1_   168  --------------------------------------GEETKKILK--------LVQEQSKTMNLSAVR  191
_XP_015766406.1_   164  --------------------------------------EEEASKVRS--------LVEKQKNSMNLSVVR  187
_UBR69922.1_       164  --------------------------------------EEEASKVRS--------LVEKQKNSMNLSVVR  187
_XP_029186950.2_   164  --------------------------------------EEEASKVRS--------LVEKQKNSMNLSVVR  187
_UCR60992.1_       164  --------------------------------------EEEASKVRS--------LVEKQKNSMNLSVVR  187
_XP_027038010.1_   165  --------------------------------------EEESQKVRA--------MVEEQKQSMNLNAVR  188
_NWX00991.1_       147  --------------------------------------ERERETIRQ--------AAVQQTKEMDLSVVR  170
_NXS91232.1_       147  --------------------------------------ERERETIRQ--------AAVQQTKEMDLSVVR  170
_NXG78778.1_       147  --------------------------------------ERERETIRQ--------AAVQQTKEMDLSVVR  170
_XP_025964754.1_   147  --------------------------------------EPEREIIRQ--------AAIQQTKEMDLSVVR  170
_XP_014730709.1_   147  --------------------------------------ERERETIRQ--------AAIQQTKEMDLSVVR  170
_NXW45082.1_       147  --------------------------------------ERERETIRQ--------AAVQQTKEMDLSVVR  170
_XP_015479466.1_   147  --------------------------------------ERERETIRQ--------AAIQQTKEMDLSVVR  170
_XP_009577719.1_   147  --------------------------------------ERERETIRQ--------AAVQQTKEMDLSVVR  170
_NXC45563.1_       147  --------------------------------------ERERETIRQ--------AAVQQTKEMDLSVVR  170
_XP_009883811.1_   147  --------------------------------------ERERETIRQ--------AAIQQTKEMDLSVVR  170
_NXH56308.1_       147  --------------------------------------ERERETIRQ--------AAIQQTKEMDLSVVR  170
_XP_023780422.1_   147  --------------------------------------ERERETIRQ--------AAIQQTKEMDLSVVR  170
_XP_009271830.1_   147  --------------------------------------ERERETIRQ--------AAIQQTKEMDLSVVR  170
_XP_009325314.1_   147  --------------------------------------ERERETIRQ--------AAIQQTKEMDLSVVR  170
_NXM85820.1_       147  --------------------------------------ERERETIRQ--------AAIQQTKEMDLSVVR  170
_NXI98149.1_       147  --------------------------------------ERERETIRQ--------AAIQQTKEMDLSVVR  170
_XP_013807107.1_   147  --------------------------------------ERERETIRQ--------AAVQQTKEMDLSVVR  170
_NWS89770.1_       147  --------------------------------------ERERETIRQ--------AAIQQTKEMDLSVVR  170
_NXN94237.1_       147  --------------------------------------ERERETIRQ--------AALQQTKEMDLSVVR  170
_NXH67539.1_       147  --------------------------------------ERERETIRQ--------AAVQQTKEMDLSVVR  170
```

```
_NXQ49570.1_       147  --------------------------------------ERERIIRQ--------AAIQQTKEMDLSVVR  170
_NWT07854.1_       147  --------------------------------------ERERIIRQ--------AAIQQTKEMDLSVVR  170
_XP_032915292.1_   147  --------------------------------------ERERIIRQ--------AAIQQTKEMDLSVVR  170
_XP_014117319.1_   147  --------------------------------------ERERIIRQ--------AAIQQTKEMDLSVVR  170
_XP_009816360.1_   147  --------------------------------------ERERIIRQ--------AAVQQTKEMDLSVVR  170
_NXO31807.1_       147  --------------------------------------ERERIIRQ--------AAIQQTKEMDLSVVR  170
_NWW07363.1_       147  --------------------------------------ERERIIRQ--------AAVQQTKEMDLSVVR  170
_XP_054681626.1_   147  --------------------------------------ERERIIRQ--------AAVQQTKEMDLSVVR  170
_XP_010286301.1_   147  --------------------------------------ERERIIRQ--------AAVQQTKEMDLSVVR  170
_NXC54686.1_       147  --------------------------------------ERERIIRQ--------AAIQQTKEMDLSVVR  170
_XP_054059788.1_   147  --------------------------------------ERERIIRQ--------AAVQQTKEMDLSVVR  170
_OPJ75671.1_       147  --------------------------------------ERERIIRQ--------AAVQQTKEMDLSVVR  170
_XP_010311473.1_   147  --------------------------------------ERERIIRQ--------AAVQQTKEMDLSVVR  170
_XP_053921662.1_   147  --------------------------------------ERERIIRQ--------AAIQQTKEMDLSVVR  170
_NWQ94220.1_       147  --------------------------------------ERERIIRQ--------AAIQQTKEMDLSVVR  170
_XP_025934196.1_   147  --------------------------------------ERERIIRQ--------AAVQQTKEMDLSVVR  170
_NXS57318.1_       147  --------------------------------------ERERIIRQ--------AAIQQTKEMDLSVVR  170
_NXF52248.1_       147  --------------------------------------ERERIIRQ--------AAVQQTKEMDLSVVR  170
_OPJ75670.1_       147  --------------------------------------ERERIIRQ--------AAVQQTKEMDLSVVR  170
_NXD98176.1_       147  --------------------------------------ERERIIRQ--------AAIQQTKEMDLSVVR  170
```
[truncated: 438,200 more chars]
